# Genome-Wide Identification of Mitogen-activated Protein Kinase Cascade Genes and Transcriptional Profiling Analysis during Organ Development in *Eucommia ulmoides*

**DOI:** 10.1038/s41598-017-17615-4

**Published:** 2017-12-18

**Authors:** Teng Jing, Lin Wang, Huimin Liu, Ta-na Wuyun, Hongyan Du

**Affiliations:** 1 Non-timber Forest Research and Development Center, Chinese Academy of Forestry, Zhengzhou, Henan 450003 China; 2 The Eucommia Engineering Research Center of State Forestry Administration, Zhengzhou, Henan 450003 China

## Abstract

The mitogen-activated protein kinase (MAPK) cascades, which play crucial roles in plant development processes, are universal modules of signal transduction in eukaryotes and consist of a core module of three sequentially phosphorylated kinases: MAPK, MAPK kinase (MAPKK), and MAPKK kinase (MAPKKK). This is the first report on the identification and analysis of MAPK cascades in *Eucommia ulmoides*. We conducted a genome-wide screening and identified 13 EuMAPKs, five EuMAPKKs, and 57 EuMAPKKKs. The construction of phylogenetic trees revealed that EuMAPKs and EuMAPKKs were divided into four groups (A, B, C, and D), and EuMAPKKKs were divided into three subfamilies (MEKK, RAF, and ZIK). These subfamilies were further confirmed by conserved domain/motif analysis and gene structure analysis. Based on the expression profiles of all identified EuMAPK cascades in various organs at different developmental stages, three genes (*EuRAF22-2*, *EuRAF34-1*, and *EuRAF33-2*) with stable expression patterns at all stages of fruit or leaf development, three genes (*EuRAF2-3*, *EuMPK11*, and *EuMEKK21*) with differential expression patterns, and two highly expressed genes (*EuZIK1* and *EuMKK2*) were screened and validated by qRT-PCR. Overall, our results could be used for further research on the precise role of MAPK cascades during organ development in *E. ulmoides*.

## Introduction

*Eucommia ulmoides* is a tree widely cultivated in the temperate zone, and it produces Eucommia rubber (Eu-rubber), a *trans*-polyisoprene (*trans*-1, 4-polyisoprene, TPI), is a special natural material. These specific properties, including high rigidity, low coefficient of thermal expansion/contraction, exceptional insulation, and resistance to acid and alkali conditions, could be exploited as an raw material for pharmaceutical, and industrial instruments^1–4^. However, the relatively low rubber content in *E. ulmoides* organs greatly increases the production cost. Previous studies reported that the accumulation of Eu-rubber is related to its organ development^5^. Hence, the systematic identification of regulatory genes for organ development in *E. ulmoides* might help to elucidate the underlying molecular mechanisms of Eu-rubber accumulation. A concrete step in this direction was the genome sequencing of *E. ulmoides*, which provides a comprehensive overview of various gene families.

To regulate the development of organs, plants have acquired complex mechanisms during their long evolution. Mitogen-activated protein kinase (MAPK) cascades are universal modules of signal transduction in eukaryotes that play crucial roles in plant development processes^6^. MAPK cascades consist of a core module of three kinases, namely MAPK, MAPK kinase (MAPKK), and MAPKK kinase (MAPKKK), which connect upstream sensors/receptors to downstream targets^7^. MAP kinases form a linear cascade of three consecutively acting protein kinases: MAPKKK are activated by interlinking MAPKKK kinases, by receptor phosphorylation, or by physical interaction, then, MAPKKKs activate downstream MAPKKs by phosphorylating the serine/threonine residues in the conserved S/TXXXXXS/T motif, and MAPKKs activate MAPKs by phosphorylating the tyrosine and threonine residues in the conserved TEY or TDY motif^8^. The activated MAPKs phosphorylate multifarious signaling components, transcription factors, or enzymes that modulate the downstream gene expression to achieve signal amplification^9,10^.

Plant MAPK cascade genes were first reported in *Arabidopsis thaliana* ^6^. Based on phylogenetic analyses, MAPKs and MAPKKs were divided into four groups (A–D)^6^, whereas MAPKKKs were classified into three subfamilies, namely MEKK, RAF, and ZIK, based on differences in the conserved domain or signature motif^11^. Previous studies have reported that MAPK cascade genes play various roles in plant innate immunity^12^, biotic^13^ and abiotic defense^14–17^, stress and hormone response^18,19^, organ and tissue development^20,21^, cell division^22^, differentiation^23^, and death^24^, and mRNA regulation^25,26^.

The genome sequencing of various plant species has allowed the identification of MAPK cascades: 20 MAPKs, 10 MAPKKs, and 80 MAPKKKs were reported in *A. thaliana* ^6,8^; 16 MAPKs, eight MAPKKs, and 75 MAPKKKs in rice^27,28^; 38 MAPKs, 11 MAPKKs, and 150 MAPKKKs in soybean^29^; 16 MAPKs, five MAPKKs, and 89 MAPKKKs in tomato^30^; 10 MAPKs, five MAPKKs, and 32 MAPKKKs in mulberry^31^; 14 MAPKs, six MAPKKs, and 59 MAPKKKs in cucumber^32^; 16 MAPKs, 12 MAPKKs, and 73 MAPKKKs in *Brachypodium distachyon* ^33^; and 25 MAPKs, 10 MAPKKs, and 77 MAPKKKs in banana^34,35^. However, little information about MAPK cascades have been reported in *E. ulmoides*.

In this study, we identified 13 MAPKs, five MAPKKs, and 57 MAPKKKs in *E. ulmoides* that named based on the corresponding homology with *A. thaliana* MAPK cascades. All the protein sequences were used to construct phylogenetic trees and study the evolutionary relationships in dicots. The predicted conserved domains, motifs, and gene structures were subsequently analyzed. The transcript profiles of all predicted EuMAPK cascades in various organs at different development stages were analyzed, and several genes with special expression patterns were screened and validated by qRT-PCR. Overall, our study provides a solid foundation for further studies on the precise roles of MAPK cascades in organ development and signaling pathways in *E. ulmoides*.

## Results and Discussion

### Identification of MAPK, MAPKK, and MAPKKK families in *E. ulmoides*

The availability of *E. ulmoides* sequences allowed the genome-wide identification and analysis of MAPK, MAPKK, and MAPKKK families. A BLASTP search was performed in the *E. ulmoides* protein database using *A. thaliana* MAPK cascade protein sequences as queries. After screening and validating the conserved domains of all candidate sequences using the Batch Web CD-Search Tool, we identified 13 EuMAPKs, five EuMAPKKs, and 57 EuMAPKKKs (Supplementary Files S1, S2, and S3). The predicted MAPKs, MAPKKs, and MAPKKKs in *E. ulmoides* were named based on their corresponding homology with MAPK, MAPKK, and MAPKKK proteins from *A. thaliana* ^6,8^, similarly as in soybean^29^, cucumber^32^, and *Brachypodium distachyon* ^33^. If two or more *E. ulmoides* genes had the same homolog in *A. thaliana*, they were distinguished by an additional part such as −1, −2, −3. Furthermore, a BLASTN search was conducted and showed that all the predicted EuMAPKs (Table 1), EuMAPKKs (Table 2), and EuMAPKKKs (Table 3) were supported by the existence of ESTs or unigenes.

**Table 1:** Characteristics of the MAPKs in *E. ulmoides*.

Gene name	Gene ID	Deduced polypeptide				Number of ESTs	Location	Homologous gene name	Homologous gene ID
		Length	Mw (kDa)	PI	Subcellular location				
EuMPK2-1	EUC23670-RA	368	42.3	6.89	Nuclear	10	scaffold198_obj	AtMAPK2	AT1G59580
EuMPK2-2	EUC18639-RA	373	42.9	6.67	Nuclear	10	scaffold1630_obj	AtMAPK2	AT1G59580
EuMPK3	EUC01391-RA	373	43.1	5.63	Cytoplasmic	63	scaffold708_obj	AtMAPK3	AT3G45640
EuMPK4-1	EUC00181-RA	375	43.1	6.54	Nuclear,Mitochondiral	25	scaffold1066_obj	AtMAPK4	AT4G01370
EuMPK4-2	EUC12684-RA	434	49.6	5.98	Nuclear,Mitochondiral	13	Super-Scaffold_139	AtMAPK4	AT4G01370
EuMPK4-3	EUC05265-RA	373	42.8	5.20	Cytoplasmic	11	Super-Scaffold_85	AtMAPK4	AT4G01370
EuMPK6	EUC17437-RA	396	45.4	5.61	Cytoplasmic,Nuclear	43	Super-Scaffold_325	AtMAPK6	AT2G43790
EuMPK9-1	EUC13785-RA	591	67.3	8.66	Nuclear	26	Super-Scaffold_28	AtMAPK9	AT3G18040
EuMPK9-2	EUC07900-RA	570	64.6	8.92	Nuclear,Cytoplasmic	9	scaffold95_obj	AtMAPK9	AT3G18040
EuMPK9-3	EUC01764-RA	682	77.6	9.27	Nuclear	30	Super-Scaffold_143	AtMAPK9	AT3G18040
EuMPK11	EUC21330-RA	343	39.4	7.64	PlasmaMembrane	19	scaffold24872_obj	AtMAPK11	AT1G01560
EuMPK15	EUC25435-RA	599	67.7	9.38	Nuclear	31	Super-Scaffold_183	AtMAPK15	AT1G73670
EuMPK16	EUC24948-RA	515	58.9	6.42	Cytoplasmic,Nuclear	121	scaffold728_obj	AtMAPK16	AT5G19010

**Table 2:** Characteristics of the MAPKKs in *E. ulmoides*.

Gene name	GENE ID	Deduced polypeptide				Number of ESTs	Location	Homologous gene name	Homologous gene ID
		Length	Mw(kDa)	PI	Subcellular location				
EuMKK2	EUC24332-RA	352	39.1	5.94	Cytoplasmic	37	scaffold211_obj	AtMKK2	AT4G29810
EuMKK3	EUC24464-RA	488	54.4	5.67	Cytoplasmic	21	Super-Scaffold_505	AtMKK3	AT5G40440
EuMKK5	EUC14834-RA	353	39.0	9.22	Nuclear	10	scaffold122_obj	AtMKK5	AT3G21220
EuMKK6	EUC01374-RA	360	40.7	5.66	Cytoplasmic	11	scaffold704_obj	AtMKK6	AT5G56580
EuMKK9	EUC01494-RA	349	38.8	6.35	Nuclear	12	Super-Scaffold_896	AtMKK9	AT1G73500

**Table 3:** Characteristics of the MAPKKKs in *E. ulmoides*.

Gene name	GENE ID	Deduced polypeptide				Number of ESTs	Location	Homologous gene name	Homologous gene ID
		Length	Mw (kDa)	PI	Subcellular location				
EuMEKK2	EUC05489-RA	659	72.30	5.62	Nuclear,Cytoplasmic	44	Super-Scaffold_90	AtMAPKKK2	AT1G54960
EuMEKK3-1	EUC17818-RA	884	95.49	9.49	Nuclear	34	Super-Scaffold_255	AtMAPKKK3	AT1G53570
EuMEKK3-2	EUC12664-RA	832	89.80	9.48	Nuclear	59	Super-Scaffold_139	AtMAPKKK3	AT1G53570
EuMEKK3-3	EUC09325-RA	571	63.77	9.92	Nuclear	11	scaffold560_obj	AtMAPKKK3	AT1G53570
EuMEKK4	EUC05370-RA	636	69.50	9.32	Nuclear	37	Super-Scaffold_4	AtMAPKKK4	AT1G63700
EuMEKK5	EUC05776-RA	684	75.20	9.32	Nuclear	16	Super-Scaffold_64	AtMAPKKK5	AT5G66850
EuMEKK10-1	EUC20951-RA	590	65.39	5.47	Nuclear	15	Super-Scaffold_307	AtMAPKKK9	AT4G08470
EuMEKK10-2	EUC13910-RA	608	66.50	5.25	Nuclear	18	Super-Scaffold_12	AtMAPKKK9	AT4G08470
EuMEKK12	EUC24974-RA	701	77.63	7.94	Nuclear	24	scaffold723_obj	AtMAPKKK12	AT3G06030
EuMEKK13	EUC16831-RA	395	43.30	5.16	Nuclear,Chloroplast	19	Super-Scaffold_39	AtMAPKKK13	AT1G07150
EuMEKK16	EUC21870-RA	382	42.40	4.69	Cytoplasmic	14	Super-Scaffold_160	AtMAPKKK16	AT4G26890
EuMEKK21	EUC00773-RA	363	39.53	5.15	Chloroplast	10	Super-Scaffold_233	AtMAPKKK21	AT4G36950
EuRAF2-1	EUC04041-RA	1018	111.49	5.71	Nuclear,Chloroplast	19	Super-Scaffold_6	AtRaf 2	AT1G08720
EuRAF2-2	EUC03132-RA	933	103.54	6.24	Nuclear,Cytoplasmic,Chloroplast	37	Super-Scaffold_150	AtRaf 2	AT1G08720
EuRAF2-3	EUC15935-RA	747	83.92	6.65	Cytoplasmic,Nuclear	24	scaffold792_obj	AtRaf 2	AT1G08720
EuRAF3-1	EUC07090-RA	379	43.32	5.80	Nuclear	18	Super-Scaffold_372	AtRaf 3	AT5G11850
EuRAF3-2	EUC17152-RA	757	84.09	5.36	Cytoplasmic	56	Super-Scaffold_279	AtRaf 3	AT5G11850
EuRAF3-3	EUC17921-RA	853	94.65	6.16	Nuclear	34	Super-Scaffold_144	AtRaf 3	AT5G11850
EuRAF3-4	EUC03449-RA	793	87.69	5.58	Cytoplasmic,Nuclear	18	Super-Scaffold_172	AtRaf 3	AT5G11850
EuRAF5	EUC21207-RA	947	104.83	5.97	Cytoplasmic,Nuclear	108	Super-Scaffold_100	AtRaf 5	AT1G73660
EuRAF8	EUC07535-RA	734	82.15	5.68	Nuclear	37	Super-Scaffold_91	AtRaf 8	AT3G06630
EuRAF10	EUC24537-RA	762	84.39	7.09	Nuclear	14	Super-Scaffold_37	AtRaf 10	AT5G49470
EuRAF15	EUC00315-RA	815	91.90	6.10	Nuclear	21	Super-Scaffold_160	AtRaf 15	AT3G58640
EuRAF16-1	EUC11981-RA	1278	140.71	5.10	Nuclear	19	Super-Scaffold_52	AtRaf 16	AT1G04700
EuRAF16-2	EUC08948-RA	1190	131.55	5.18	Nuclear	21	Super-Scaffold_120	AtRaf 16	AT1G04700
EuRAF19-1	EUC20242-RA	382	43.59	8.95	Nuclear	18	Super-Scaffold_11	AtRaf 19	AT1G62400
EuRAF19-2	EUC21989-RA	354	39.96	8.20	Cytoplasmic	14	Super-Scaffold_6	AtRaf 19	AT1G62400
EuRAF20-1	EUC11169-RA	1259	139.57	5.68	Nuclear	19	Super-Scaffold_101	AtRaf 20	AT1G79570
EuRAF20-2	EUC05347-RA	1046	117.71	5.69	Nuclear	32	scaffold85_obj	AtRaf 20	AT1G79570
EuRAF20-3	EUC16268-RA	1290	140.30	5.30	Nuclear	34	Super-Scaffold_36	AtRaf 20	AT1G79570
EuRAF20-4	EUC10624-RA	1118	124.00	5.21	Nuclear	15	Super-Scaffold_14	AtRaf 20	AT1G79570
EuRAF22-1	EUC20307-RA	363	40.55	7.08	Nuclear,Cytoplasmic	27	Super-Scaffold_16	AtRaf 22	AT2G24360
EuRAF22-2	EUC10582-RA	125	14.29	6.71	Mitochondiral	23	Super-Scaffold_14	AtRaf 22	AT2G24360
EuRAF29	EUC17901-RA	574	65.55	5.92	Cytoplasmic,Nuclear	63	scaffold855_obj	AtRaf 29	AT4G35780
EuRAF30-1	EUC26609-RA	567	64.26	6.06	Cytoplasmic	15	scaffold713_obj	AtRaf 30	AT4G38470
EuRAF30-2	EUC06660-RA	566	64.57	6.33	Cytoplasmic	82	scaffold1037_obj	AtRaf 30	AT4G38470
EuRAF30-3	EUC14489-RA	554	62.45	4.88	Cytoplasmic	30	Super-Scaffold_26	AtRaf 30	AT4G38470
EuRAF30-4	EUC03168-RA	537	60.75	5.22	Cytoplasmic	47	Super-Scaffold_179	AtRaf 30	AT4G38470
EuRAF31	EUC03978-RA	346	38.62	6.27	Cytoplasmic	13	Super-Scaffold_381	AtRaf 31	AT5G01850
EuRAF33-1	EUC10175-RA	377	42.12	6.52	Nuclear	20	Super-Scaffold_113	AtRaf 33	AT5G50000
EuRAF33-2	EUC21992-RA	378	42.06	7.12	Cytoplasmic,Nuclear	33	Super-Scaffold_6	AtRaf 33	AT5G50000
EuRAF34-1	EUC24477-RA	252	28.40	6.26	Cytoplasmic	20	Super-Scaffold_505	AtRaf 34	AT5G50180
EuRAF34-2	EUC03396-RA	565	63.88	6.31	Cytoplasmic	67	Super-Scaffold_177	AtRaf 34	AT5G50180
EuRAF36	EUC20904-RA	488	55.04	9.32	Mitochondiral	20	scaffold1136_obj	AtRaf 36	AT5G58950
EuRAF39-1	EUC09794-RA	402	44.91	8.51	Cytoplasmic,Nuclear	11	scaffold298_obj	AtRaf 39	AT3G22750
EuRAF39-2	EUC16639-RA	402	44.70	8.71	Cytoplasmic	9	Super-Scaffold_34	AtRaf 39	AT3G22750
EuZIK1	EUC20701-RA	535	62.17	5.23	Nuclear	27	scaffold786_obj	AtZIK1	AT3G51630
EuZIK4-1	EUC10801-RA	595	67.61	5.25	Nuclear,Cytoplasmic	17	scaffold700_obj	AtZIK4	AT3G04910
EuZIK4-2	EUC04221-RA	632	72.64	6.01	Nuclear	27	Super-Scaffold_10	AtZIK4	AT3G04910
EuZIK4-3	EUC14352-RA	655	74.24	5.00	Nuclear	21	scaffold906_obj	AtZIK4	AT3G04910
EuZIK8-1	EUC16962-RA	299	34.31	5.31	Nuclear	17	scaffold246489_obj	AtZIK8	AT5G55560
EuZIK8-2	EUC06431-RA	310	35.29	5.26	Nuclear	24	scaffold166_obj	AtZIK8	AT5G55560
EuZIK8-3	EUC04697-RA	393	45.28	8.03	Mitochondrionl,Cytoplasmic	14	Super-Scaffold_3	AtZIK8	AT5G55560
EuZIK8-4	EUC09614-RA	340	38.90	5.16	Cytoplasmic,Nuclear	18	scaffold294_obj	AtZIK8	AT5G55560
EuZIK8-5	EUC15557-RA	433	48.56	5.12	Nuclear,Cytoplasmic	1	scaffold484_obj	AtZIK8	AT5G55560
EuZIK9	EUC10368-RA	693	79.18	5.28	Nuclear	60	Super-Scaffold_46	AtZIK9	AT5G28080
EuZIK11	EUC07070-RA	629	70.97	5.13	Nuclear	20	Super-Scaffold_127	AtZIK11	AT3G48260

The 13 EuMAPK predicted proteins contained 343 (EuMPK11) to 599 (EuMPK15) amino acid residues with a putative pI ranging from 5.20 (EuMPK4-3) to 9.38 (EuMPK15) and a putative Mw ranging from 39.4 (EuMPK11) to 67.7 (EuMPK15). EuMAPKs were predicted to be localized in the nucleus, cytoplasm, mitochondria, or plasma membranes (Table 1). The five EuMAPKK predicted proteins contained 352 (EuMKK2) to 488 (EuMKK3) amino acid residues with a putative pI ranging from 5.67 (EuMKK3) to 9.22 (EuMKK5) and a putative Mw ranging from 39.0 (EuMKK5) to 54.4 (EuMKK3). EuMAPKKs were predicted to be localized in the nucleus or cytoplasm (Table 2). The 57 EuMAPKKK predicted proteins contained 125 (EuRAF22-2) to 1,290 (EuRAF20-3) amino acid residues with a putative pI ranging from 4.69 (EuMEKK16) to 9.92 (EuMEKK3-3) and a putative Mw ranging from 14.29 (EuRAF22-2) to 140.71 (EuRAF16-1). EuMAPKKKs were predicted to be localized in the nucleus, mitochondria, cytoplasm, or chloroplasts (Table 3).

### Phylogenetic relationship and evolution pattern analysis

**Figure 1:**
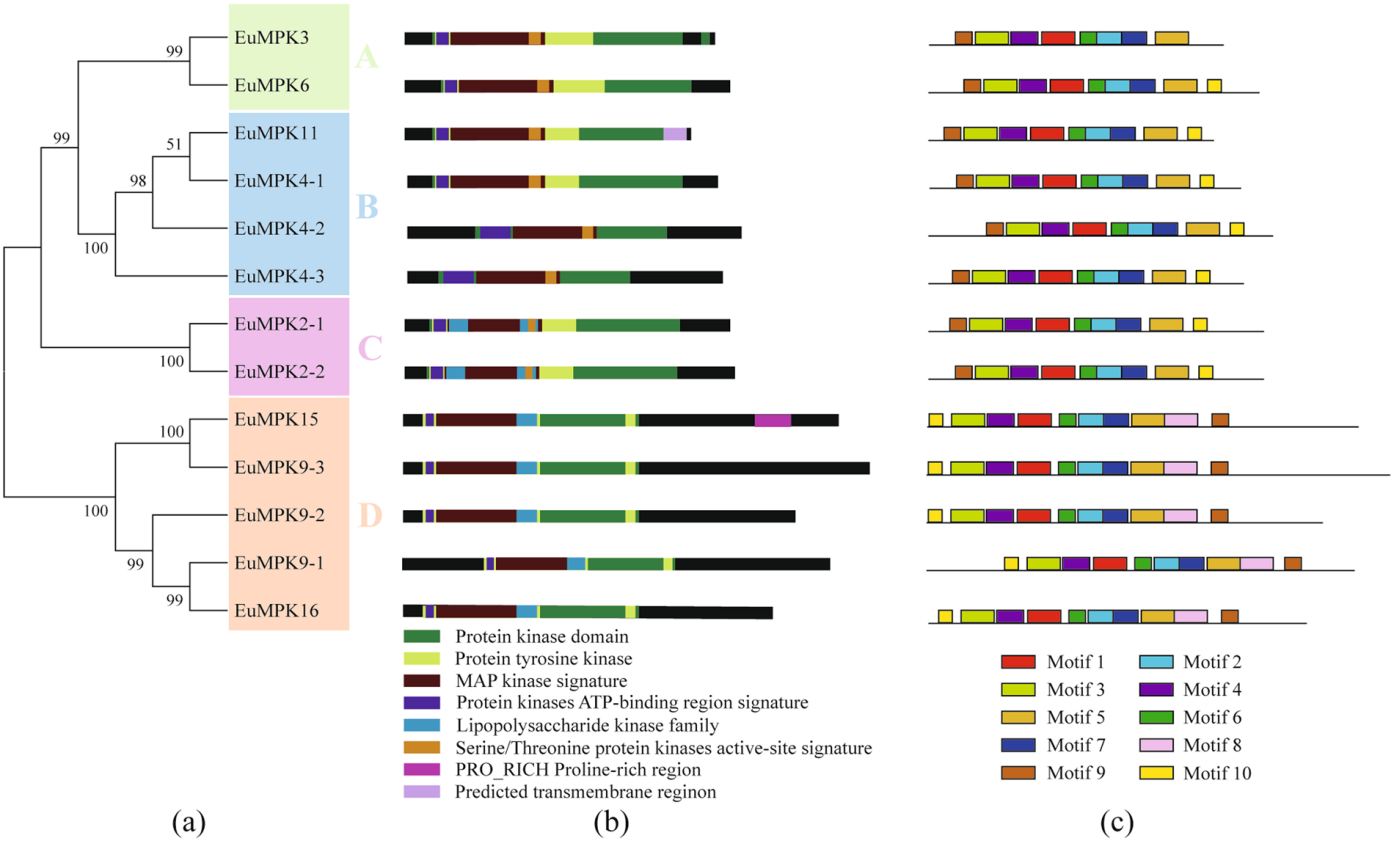
Phylogenetic relationship, conserved domain and motif analysis of MAPKs in *E. ulmoides*. **(a)** The unrooted phylogenetic tree was construceted based on the amino acid sequences by the NJ method using MEGE 7.0. Bootstrap supports from 1000 replicates are indicated at each branch. The members of each subfamily are indicated with the same color. **(b)** Conserved domain was analyzed by searching those known domains with PlantsP. **(c)** Motif was analyzed by MEME program online. Different colors of boxes represent different motifs in the corresponding position.

**Figure 2:**
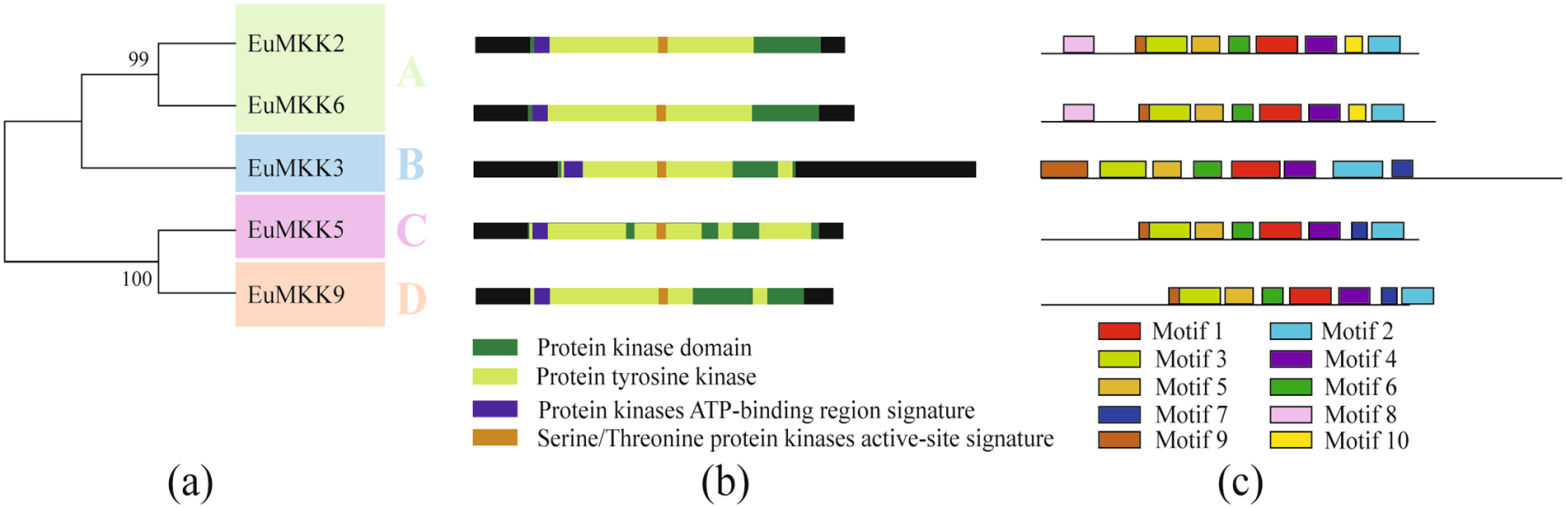
Phylogenetic relationship **(a)**, conserved domain **(b)**,and motif analysis **(c)** of MAPKKs in *E. ulmoides*. Additional details were shown in the Fig. 1.

**Figure 3:**
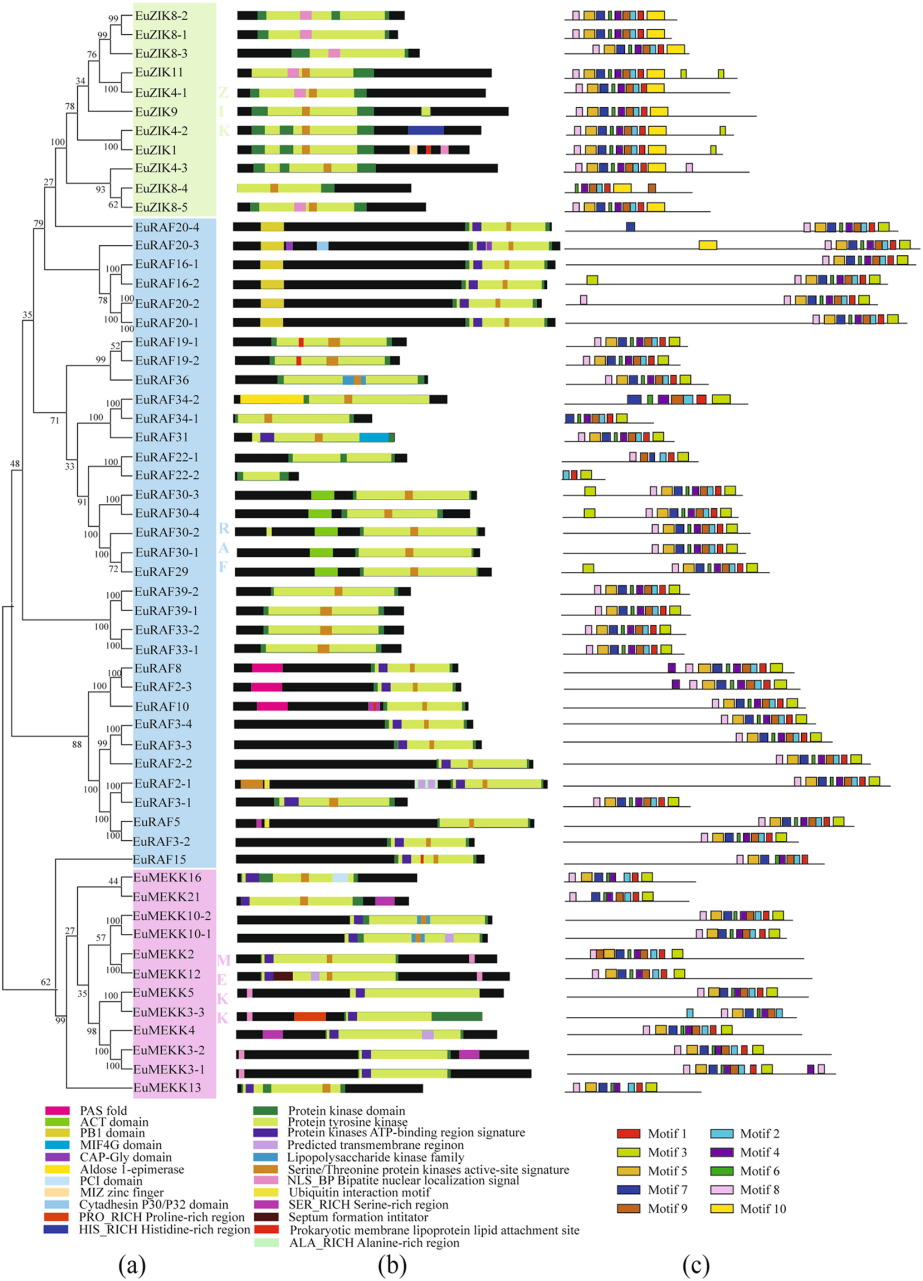
Phylogenetic relationship **(a)**, conserved domain **(b)**, and motif analysis **(c)** of MAPKKKs in *E. ulmoides*. Additional details were shown in the Fig. 1.

Unrooted phylogenetic trees were generated based on the aligned protein sequences of all 13 EuMAPKs, five EuMAPKKs, and 57 EuMAPKKKs and showed similar topologies, except for only minor modifications at deep nodes. Based on the phylogenetic trees and the homology with *A. thaliana*, the 13 EuMAPKs were classified into four groups (A–D; Fig. 1a); the five EuMAPKKs were also classified into four groups (A–D; Fig. 2a); whereas the 57 EuMAPKKKs were classified into three sub-families (12 MEKKs, 34 RAFs, and 11 ZIKs) (Fig. 3a). These results were consistent with those reported in previous studies on rice^28^, tomato^30^, and cucumber^32^.

To study the evolutionary relationships of the MAPKs, MAPKKs, and MAPKKKs in dicots, we compared the member number of each family in *E. ulmoides* with that in other dicotyledons. According to the Angiosperm Phylogeny Group (APG IV) classification^36^, both tomato and *E. ulmoides* were classified as Asterids, and *A. thaliana* and *Populus tremula* were also selected as a model plant and model forest tree, respectively. Τhe MAPK cascades of all the above species were re-confirmed using the most updated genome versions and the same screening criteria. The number of MAPKs, MAPKKs, and MAPKKKs in different species is listed in Table 4. Unrooted phylogenetic trees were constructed based on 71 MAPKK, 31 MAPKK, and 339 MAPKKK sequences (Supplementary Table S1). The results showed that MAPKs and MAPKKs were clearly classified into four distinct groups (Supplementary Figs S1 and S2), and MAPKKKs were classified into three subfamilies, namely, MEKK, RAF, and ZIK (Supplementary Fig. S3). Meanwhile, all groups and subfamilies contained most members of the four species, indicating that MAPK cascades might derive from a common ancestor. The evolutionary relationship of MAPK cascades in *E. ulmoides* and those in tomato was closer than that of the same genes in *A. thaliana* and those in *P. tremula*, results that were in conformity with the APG taxonomic system.

**Table 4:** The number of MAPK cascades in *E. ulmoides, S. lycopersicum, A. thaliana*, and *P. tremula*.

Species	MAPK	MAPKK	MAPKKK				Taxonomy
			Total	MEKK	RAF	ZIK	
*E. ulmoides*	13	5	57	12	34	11	Asterids
*S. lycopersicum*	16	5	89	33	40	16	Asterids
*A. thaliana*	20	10	80	21	48	11	Rosids
*P. tremula*	22	11	113	31	65	17	Rosids

### Analysis of conserved domains/motifs and gene structure

All the members of the three MAPK families harbored a protein kinase domain (Figs 1b, 2b, and 3b), confirming the reliability of all predicted EuMAPK cascades. In the EuMAPK family, the members of group D had an extended C-terminal region, but lacked a serine/threonine protein kinase active-site signature (Fig. 1b), similarly as those in *A. thaliana* ^6^ and cucumber^33^; EuMPK11 was predicted to harbor a transmembrane region (Fig. 1b), which confirmed its predicted subcellular localization in the plasma membrane. All EuMAPKKs harbored a protein kinase domain, a tyrosine kinase, an ATP-binding region, and a serine/threonine protein kinase active site, and EuMAPKK3 was predicted to have a long C-terminal region (Fig. 2b), similarly to MAPKKs in cucumber^33^. All EuMAPKKKs contained a protein tyrosine kinase. The kinase domain of most ZIK subfamily proteins was located at the C-terminal, whereas that of most RAF subfamily proteins was located at the N-terminal. A protein kinase ATP-binding region signature was only found in the MEKK subfamily. All these results were consistent with those previously reported in *A. thaliana* ^8^, rice^28^, and tomato^30^.

The motifs were analyzed by the MEME. In the EuMAPK family, almost all the members in the same subfamily shared a similar quantity of motifs (Fig. 1c). For instance, all the members of group D had ten motifs, whereas all the members of group A, B, and C had nine motifs, except for EuMPK3. Meanwhile, all the members of group D had the 9^th^ motif in the N-terminal region and the 10^th^ motif in the C-terminal region, whereas the opposite trend was observed for all the members of group A, B, and C. The same results were obtained for the EuMAPKK and EuMAPKKK families (Figs 2c and 3c), indicating that the classification was supported by motif analysis.

**Figure 4:**
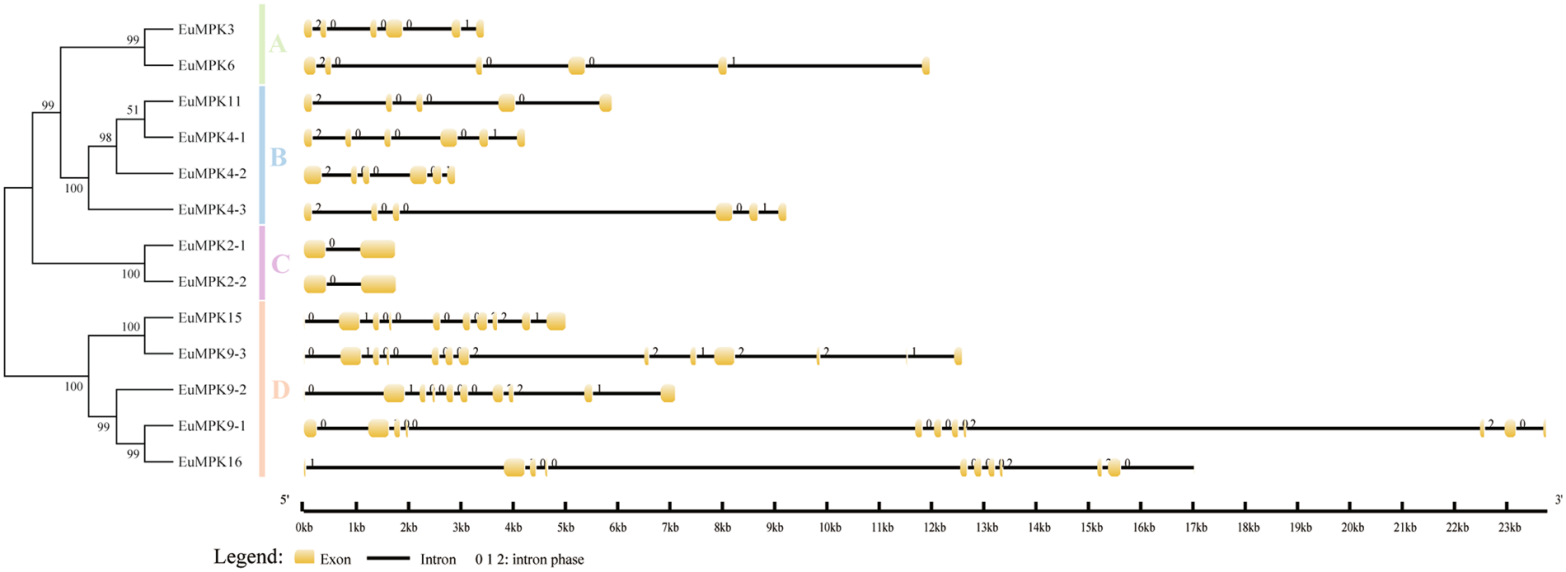
Phylogenetic relationship and gene structure analysis of *MAPKs* in *E. ulmoides*. Right part illustrates the intron/exon configurations of the each *EuMAPK*. The yellow boxes denote the exons, and the lines denote the introns.

**Figure 5:**
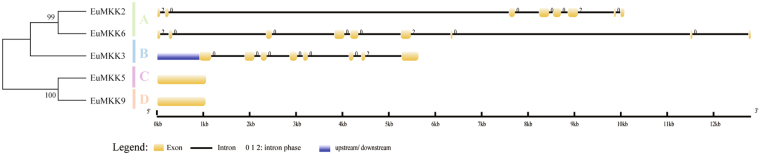
Phylogenetic relationship and gene structure analysis of *MAPKKs* in *E. ulmoides*. Additional details were shown in the Fig. 4.

**Figure 6:**
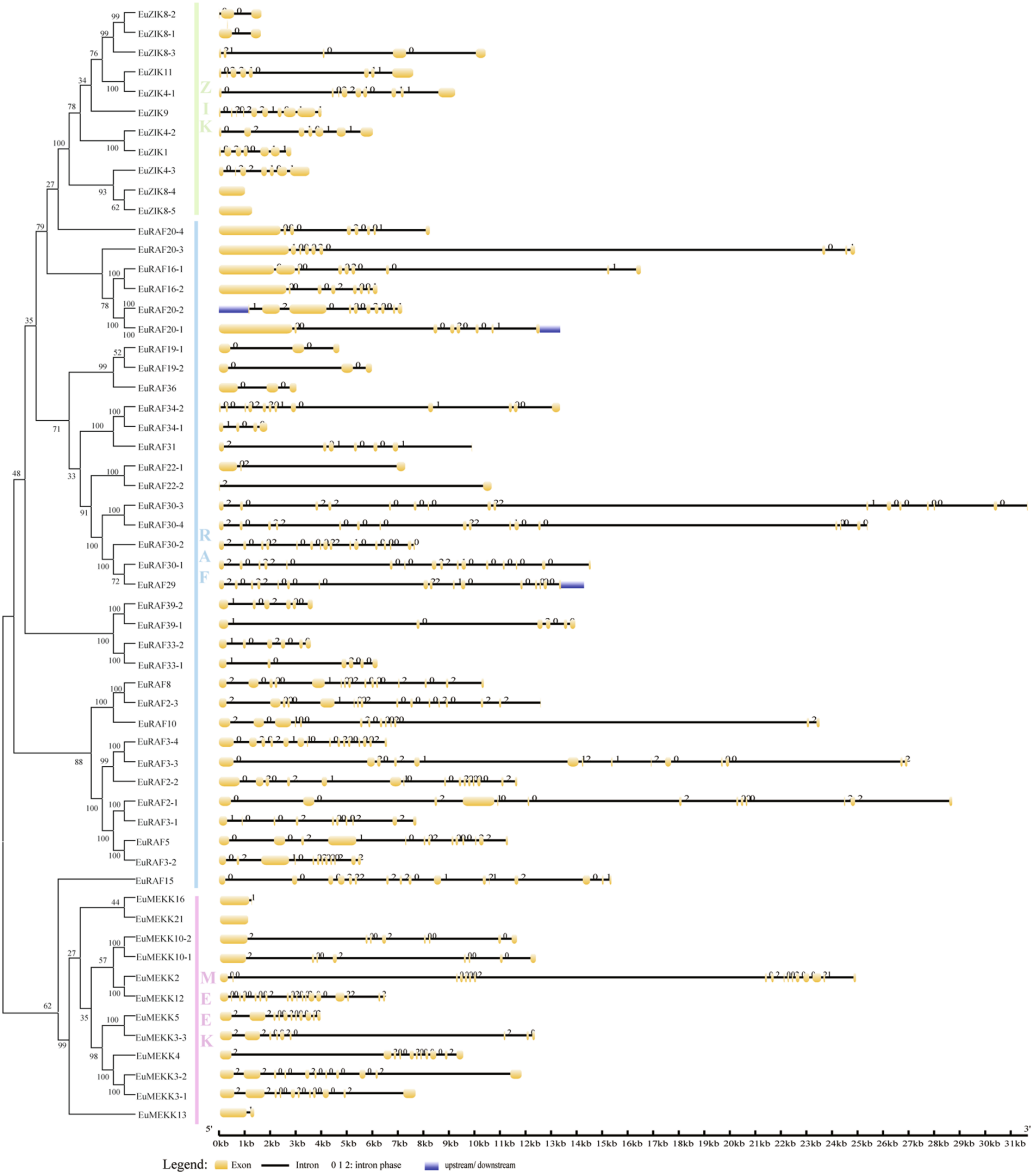
Phylogenetic relationship and gene structure analysis of *MAPKKKs* in *E. ulmoides*. Additional details were shown in the Fig. 4.

To evaluate the phylogenetic relationships based on the gene structure, the exon-intron organization of all EuMAPK cascades was analyzed. The number of introns in the *EuMAPKs* was 1–12 (Fig. 4), and that in the *EuMAPKKs* was 0–8, the intron phase and exon/intron organization in the *EuMAPKs* and *EuMAPKKs* were relatively conserved within the same group (Fig. 5), indicating that the classification of *EuMAPKs* and *EuMAPKKs* was supported by the gene structure analysis. However, the number of introns displayed a higher degree of variability in the *EuMAPKKKs* (Fig. 6), ranging from 0 to 17. In the MEKK subfamily, the number of introns was 0–17; *EuMEKK21* had no introns, *EuMEKK16* and *EuMEKK13* had only one intron, whereas the remaining members had 7–17 introns, results that were consistent with those reported in cucumber^32^. The RAF subfamily members had 1–16 introns, whereas the ZIK subfamily members had 0–9 introns, results that were consistent with those reported in *B. distachyon* ^33^. Collectively, the classification of the *EuMAPKKKs* was supported by the comparison with orthologous families. The size of introns in the three *EuMAPKs* was positively correlated with the genome size in *E. ulmoides*, *A. thaliana* ^6^, *B. distachyon* ^8^, cucumber^32^, and banana^35^, whereas the number of introns was relatively conserved among the species.

### Expression analysis of *EuMAPK*, *EuMAPKK*, and *EuMAPKKK* genes in various organs at different developmental stages

**Figure 7:**
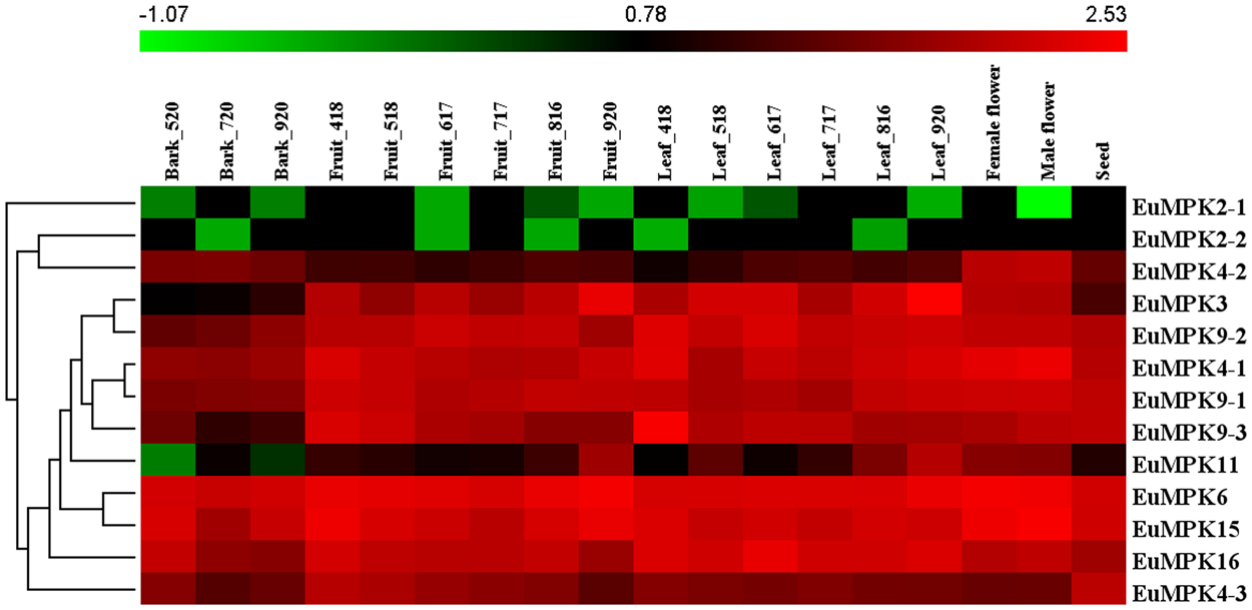
Expression profiles of *EuMAPKs* in various organs at different developmental stages based on RNA-seq data. The expression levels of genes are presented in heatmap using fold-change values transformed to Log2 format by HemI 1.0. The color scale and Log2 values are shown at the top of the heatmap. Genes were clustered according to their expression profiles.

**Figure 8:**
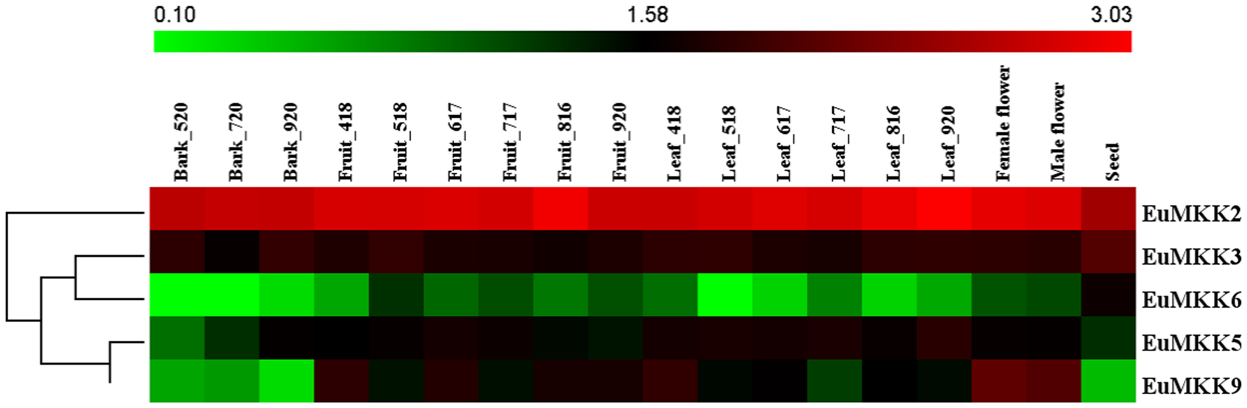
Expression profiles of *EuMAPKKs* in various organs at different developmental stages based on RNA-seq data. Additional details were shown in the Fig. 7.

**Figure 9:**
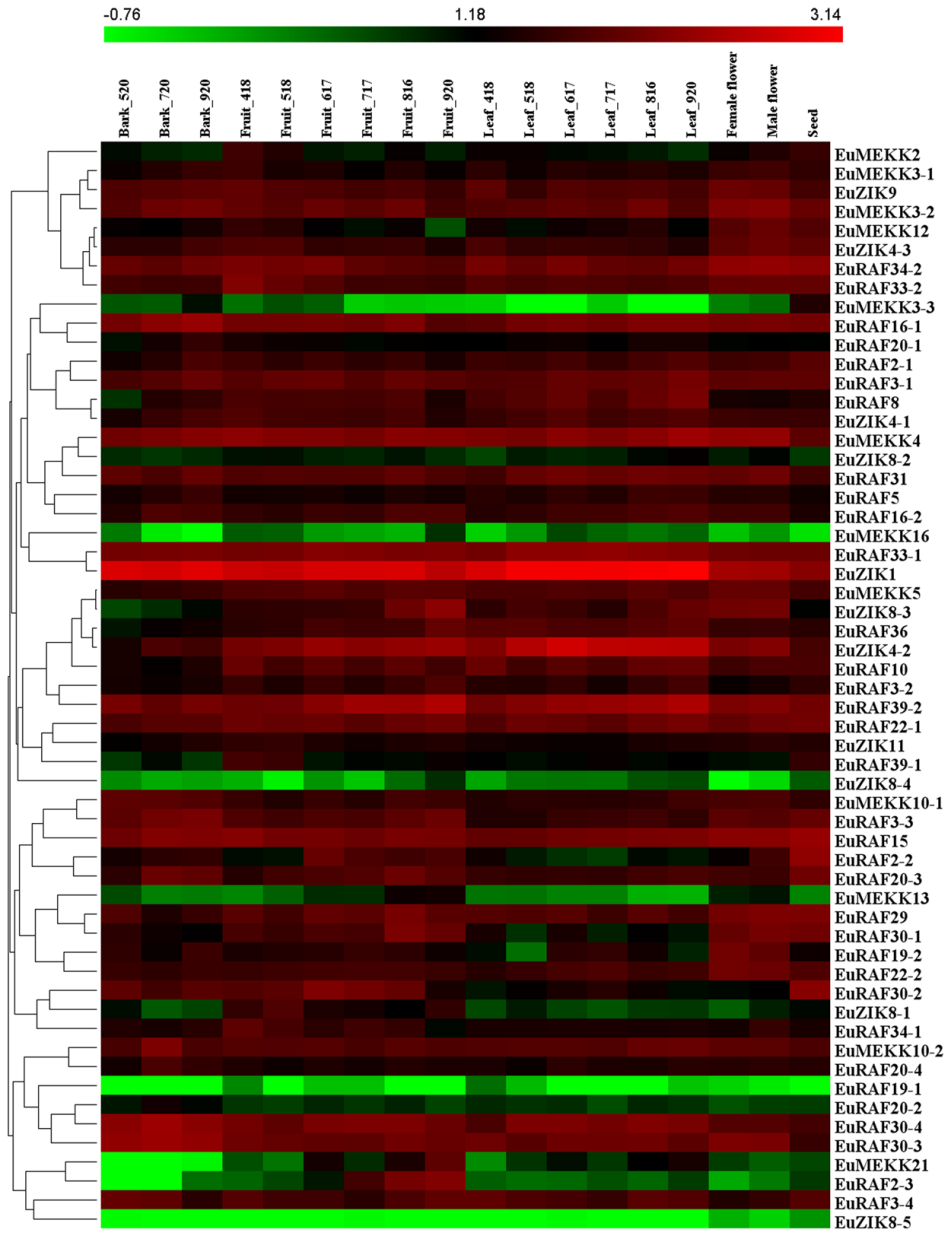
Expression profiles of *EuMAPKKKs* in various organs at different developmental stages based on RNA-seq data. Additional details were shown in the Fig. 7.

To reveal the temporal and spatial expression patterns of EuMAPK cascades, we compared the transcription levels in various organs at different developmental stages, including fruits, leaves, barks, male flowers, female flowers, and seeds. The expression levels of these genes were clustered and presented in heatmaps (Figs 7, 8, and 9). The results revealed all MAPK cascade members were expressed in almost all tested organs.

To find the key members of EuMAPK cascades in the course of *E. ulmoides* organ development, the coefficient of variation (CV) of gene expression levels in all tested organs at various developmental stages (CV_all_) as well as in the fruits and leaves at all developmental stages (CV_F_ and CV_L_, respectively) were calculated (Supplementary Tables S2, S3, and S4). The results showed that no genes had a CV_all_ lower than 10%, and only one had a CV_all_ higher than 200% (*EuRAF2-3*; 262.63%). *EuRAF3-1* and *EuRAF22-2* showed the lowest CV_all_ (23.1%) and CV_F_ (9.58%), respectively, and *EuRAF34-1* and *EuRAF33-2* had the two lowest CV_L_ (1.64% and 8.79%, respectively), indicating that these genes had stable expression levels and might play important roles in the corresponding organs at all developmental stages.

The relative expression is an important indicator of the gene function. Based on the Fragments per kilobase of per million fragments mapped (FPKM) values, we found that the relative expression of *EuZIK1* and *EuMKK2* was significantly (*p* < 0.01) higher than that of the other 73 *EuMAPK*s, suggesting that these two genes might play important roles in the *EuMAPK* cascade. Additionally, Our results showed that some genes expression levels were significantly higher in fruits and seeds at late developmental stage than those in other organs, therefore, we calculated the log_2_-base ratio value between different organs or between different stages of the same organ. The expression levels of *EuRAF2-3* increased more than 5.5-fold (log_2_-base value) and 7.5-fold (log_2_-base value) in fruits and seeds, respectively, at late development stages, suggesting that this gene might participate in fruit and seed ripening. The expression levels of *EuMPK11* and *EuMEKK21* increased more than 2.5-fold (log_2_-base value) in fruits and leaves and more than 4.5-fold (log_2_-base value) in fruits, respectively, at late development staged, suggesting that both genes might participate in fruit ripening, whereas the former might also participate in leaf development.

### Validation of key MAPK cascades by qRT-PCR

**Figure 10:**
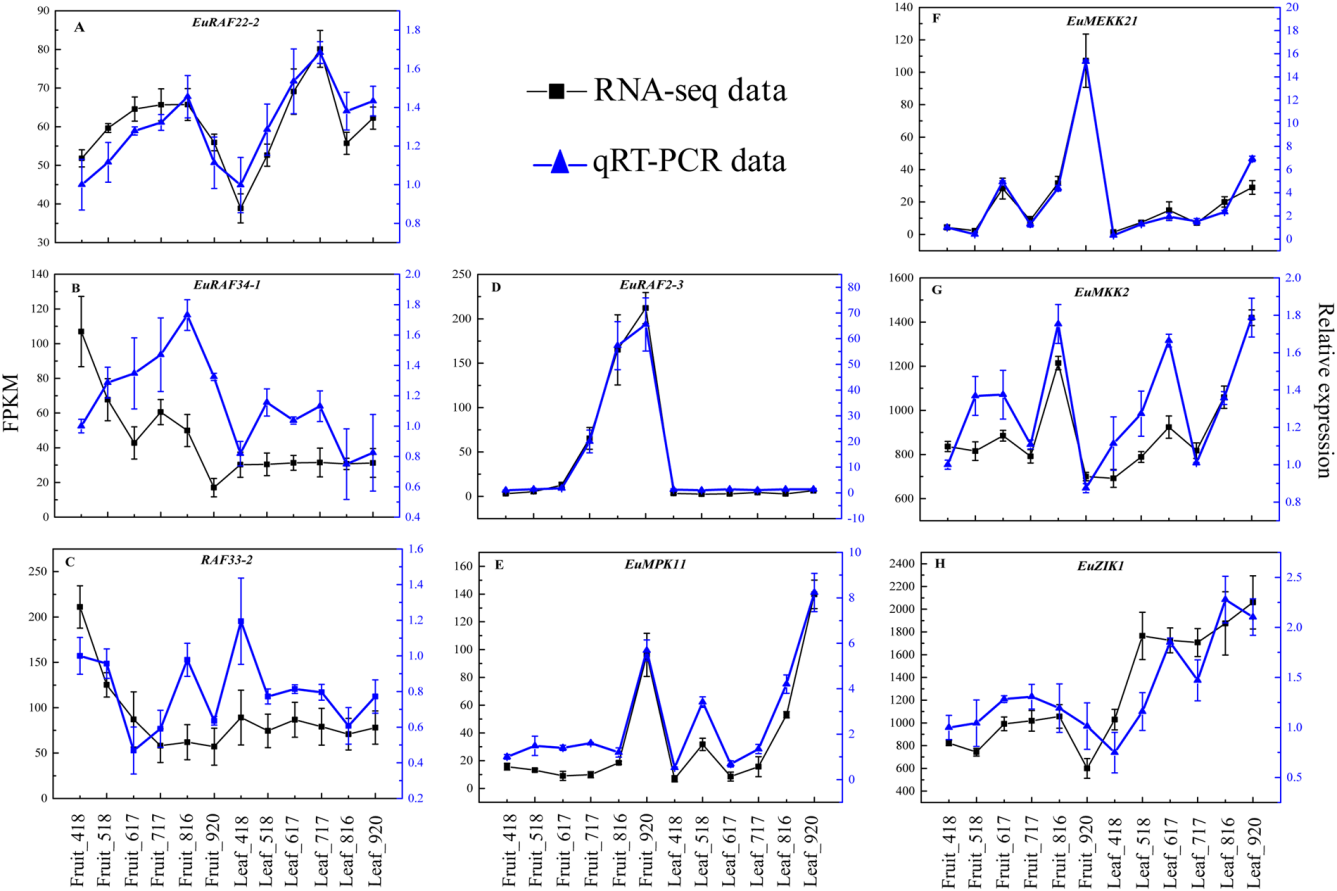
qRT-PCR analysis of relative expression of eight screened genes during *E. ulmoides* fruits and leaves development.

Three genes (*EuRAF22-2*, *EuRAF34-1*, and *EuRAF33-2*) with stable expression patternsat all stages of fruit or leaf development, three genes (*EuRAF2-3*, *EuMPK11*, and *EuMEKK21*) with differential expression patterns, and two highly expressed genes (*EuZIK1* and *EuMKK2*) were selected for qRT-PCR analysis to validate the RNA-seq data. The integral trend of expression patterns of all the selected genes was consistent with that obtained from the RNA-seq data, confirming data reliability (Fig. 10).

## Methods

### Search for MAPK cascades and sequence analysis

The predicted *E. ulmoides* peptide sequences were acquired from the *E. ulmoides* genome database to construct a local protein database. A BLASTP search was performed using 20 MAPK, 10 MAPKK, and 80 MAPKKK protein sequences from *A. thaliana* (Supplementary Table 5) as queries in The *Arabidopsis* Information Resource (TAIR; http://www.arabidopsis.org/), the National Center for Biotechnology Information (NCBI; https://www.ncbi.nlm.nih.gov/), and the Universal Protein Resource (Uniprot; http://www.uniprot.org/) databases with an e-value of 1e-10 and a minimum amino acid identity of 50%. Then, a self-BLAST of all hits was carried out to remove redundancies. All the candidate genes were detected by the NCBI Batch Web CD-Search Tool (http://www.ncbi.nlm.nih.gov/Structure/bwrpsb/bwrpsb.cgi) database to confirm the presence of the kinase domain. MAPKs should contain a T(E/D)YVxTRWYRAPE(L/V) signature motif, MAPKKs should contain a VGTxxYM(S/A)PER motif, whereas MAPKKKs should contain one of the three signature motifs: G(T/S)(P/A)x(W/F/Y)MAPE (MEKK-like), GTxx(W/Y)MAPE (Raf-like), or GTPE(Y/F)MAPExY(ZIK-like)^8^. A local BLASTN search was performed against the *E. ulmoides* expressed sequence tags (ESTs) and unigenes to verify the existence of the predicted genes. The putative isoelectric point (pI) and the molecular weight (Mw) of the obtained protein sequences were predicted using Compute pI/Mw (http://web.expasy.org/compute_pi/). The subcellular localization of each gene was predicted using CELLO 2.5 (http://cello.life.nctu.edu.tw/).

### Multiple sequence alignment and phylogenetic tree construction

The predicted full-length EuMAPK cascade protein sequences were aligned using Clustal W. Phylogenetic trees were constructed in MEGA 7.0^37^ using the Neighbor Joining (NJ) methods with 1,000 bootstrap replications.

### Conserved motif/domain and gene structure analysis

Domains and motifs were discovered by PlantsP (http://plantsp.genomics.purdue.edu/cgi-bin/fscan/feature_scan_rest.cgi?db = PlantsP) and MEME (http://meme-suite.org/tools/meme). The exon-intron organization and intron phase were analyzed by the Gene Structure Display Server (http://gsds.cbi.pku.edu.cn/).

### Gene expression analysis and qRT-PCR

To study the transcriptional expression characteristics of each predicted member of the EuMAPK cascades, the raw reads were downloaded from National Center for Biotechnology Information (NCBI, https://www.ncbi.nlm.nih.gov/) under accession numbers: female/male flower buds (SRR2170964, SRR2170970), seeds (SRR3203241), and fruit, leaf, and bark during the developmental stages (unpublished). Firstly, raw reads were pre-processed to remove low quality regions and adapter sequences. Index of the reference genome was built using Bowtie v2.2.3 and paired-end clean reads were aligned to the *E. ulmoides* genome (unpublished) using TopHat v2.0.12^38^. Then, HTSeq v0.6.1 was used to count the reads numbers mapped to each gene^39^. Finally, FPKM each gene was calculated based on the length of the gene and reads count mapped to this gene^40^.

Based on FPKM values, heatmaps and hierarchical clusters were created by HemI 1.0 (http://hemi.biocuckoo.org/down.php). Coefficients of variation (CV) and *p* values were calculated by Minitab 16 (http://www.minitab.com/zh-cn/). To obtain candidate genes that potentially control *E. ulmoides* organ development, special genes identified by CV and *p* values were selected for qRT-PCR. Total RNA was extracted, and reverse-transcribed into cDNA using the AMV First Strand cDNA Synthesis Kit (Sangon, Shanghai, China). Primers were designed by Primer 5.0 (Supplementary Table S6 **)**, and 18S was used as an internal reference gene. qPCR was performed using an ABI StepOnePlus system (Applied Biosystems, Foster City, CA, USA). The expression levels were calculated by the 2^−ΔΔCt^ method^41^. Each sample was repeated in triplicate.

## Electronic supplementary material

Supplementary material
