# Supplementary material for: Genome-Wide Identification of Mitogen-activated Protein Kinase Cascade Genes and Transcriptional Profiling Analysis during Organ Development in Eucommia ulmoides
Source: Sci Rep. 2017 Dec 18;7:17732. doi: 10.1038/s41598-017-17615-4 (PMC5735150; doi:10.1038/s41598-017-17615-4)
Supplement: Supplementary file 1 — Supplementary material [file 41598_2017_17615_MOESM1_ESM.pdf]

Genome-Wide Identification of Mitogen-activated Protein Kinase Cascade Genes and Transcriptional Profiling Analysis during Organ Development in *Eucommia ulmoides*

Teng Jing[1,2‡], Lin Wang[1,2‡], Huimin Liu[1,2], Hongyan Du[1,2*] and Ta-na Wuyun[1,2*]

[1]Non-timber Forest Research and Development Center, Chinese Academy of Forestry. Zhengzhou, Henan, 450003, China
[2]The Eucommia Engineering Research Center of State Forestry Administration, Zhengzhou, Henan, 450003, China

*Correspondence:
Ta-na Wuyun
tanatanan@163.com
Hongyan Du
dhy515@126.com

‡ These authors have contributed equally to this work.

Supplementary material

Supplementary Table and legends

Supplementary Table S1. Gene name and ID of the MAPK cacadesin *S. lycopesicum* and *P. tremula*

Supplementary Table S2. The CV values of all *EuMAPK* cascades in various organs at different developmental stages

Supplementary Table S3. The CV values of all *EuMAPK* cascades in fruits at various developmental stages

Supplementary Table S4. The CV values of all *EuMAPK* cascades in leaves at various developmental stages

Supplementary Table S5. The AtMAPKs, AtMAPKKs, and AtMAPKKKs used as the queries for the local Blast search in this study

Supplementary Table S6. Primer sequences of eight screened genes for qRT-PCR expression analysis

Supplementary Figures and legends

Supplementary Figure S1. The phytogenetic tree of MAPKs from *E. ulmoides*, *S. lycopesicum*, *A. thaliana* and *P. tremula*.

Supplementary Figure S2. The phytogenetic tree of MAPKKs from *E. ulmoides*, *S. lycopesicum*, *A. thaliana* and *P. tremula*.

Supplementary Figure S3. The phytogenetic tree of MAPKKKs from *E. ulmoides*, *S. lycopesicum*, *A. thaliana* and *P. tremula*.

Supplementary files

Supplementary File S1. The coding sequences of EuMAPK cascades

Supplementary File S2. The protein sequences of EuMAPK cascades

Supplementary File S3. The genomic DNA sequences of EuMAPK cascades

Supplementary Table and legends

Supplementary Table S1. Gene name and ID of the MAPK cacadesin *S. lycopesicum* and *P. tremula*

	S. lycopersicum			*P. tremula*		
	Gene name	Gene ID	Homologous *Arabidopsis* Name	Gene name	Gene ID	Homologous *Arabidopsis* Name
MAPK	SlMAPK6-1	Solyc12g019460.1.1	ATMAPK6	PtMAPK6-2	Potri.002G032100.1	ATMAPK6
	SlMAPK6-2	Solyc08g014420.2.1	ATMAPK6	PtMAPK9-6	Potri.007G070100.1	ATMAPK9
	SlMAPK3	Solyc06g005170.2.1	ATMAPK3	PtMAPK16-1	Potri.015G040300.1	ATMAPK16
	SlMAPK13	Solyc11g072630.1.1	ATMAPK13	PtMAPK16-2	Potri.012G048600.1	ATMAPK16
	SlMAPK4	Solyc01g094960.2.1	ATMAPK4	PtMAPK19	Potri.001G381300.1	ATMAPK19
	SlMAPK11-1	Solyc05g049970.2.1	ATMAPK11	PtMAPK2-1	Potri.005G231100.1	ATMAPK2
	SlMAPK11-2	Solyc08g081490.2.1	ATMAPK11	PtMAPK2-2	Potri.002G032100.1	ATMAPK2
	SlMAPK7	Solyc02g084870.2.1	ATMAPK7	PtMAPK20-1	Potri.010G029700.1	ATMAPK20
	SlMAPK2	Solyc04g080730.2.1	ATMAPK2	PtMAPK3-1	Potri.009G066100.1	ATMAPK3
	SlMAPK8	Solyc10g007500.2.1	ATMAPK8	PtMAPK3-2	Potri.001G271700.1	ATMAPK3
	SlMAPK9-1	JF795446	ATMAPK9	PtMAPK4-1	Potri.014G088500.1	ATMAPK4
	SlMAPK9-2	Solyc07g056350.2.1	ATMAPK9	PtMAPK4-2	Potri.003G131800.1	ATMAPK4
	SlMAPK9-3	JF795448	ATMAPK9	PtMAPK4-3	Potri.002G162500.1	ATMAPK4
	SlMAPK16-1	Solyc04g007710.2.1	ATMAPK16	PtMAPK4-4	Potri.001G099900.1	ATMAPK4
	SlMAPK16-2	JF795451	ATMAPK16	PtMAPK6-1	Potri.007G139800.1	ATMAPK6
	SlMAPK9-4	Solyc12g040680.1.1	ATMAPK9	PtMAPK7-1	Potri.007G020100.1	ATMAPK7
				PtMAPK7-2	Potri.005G119500.1	ATMAPK7
				PtMAPK9-1	Potri.010G112200.1	ATMAPK9
				PtMAPK9-2	Potri.008G200800.1	ATMAPK9
				PtMAPK9-3	Potri.008G130000.1	ATMAPK9
				PtMAPK9-4	Potri.005G201800.1	ATMAPK9
				PtMAPK9-5	Potri.002G059900.1	ATMAPK9
MKK	SlMKK2	Solyc12g009020.1.1	ATMKK2	PtMKK6	Potri.018G068500.1	ATMKK6
	SlMKK5	Solyc03g123800.1.1	ATMKK5	PtMKK3	Potri.001G345500.1	ATMKK3
	SlMKK6	Solyc03g119490.2.1	ATMKK6	PtMKK10	Potri.001G138800.1	ATMKK10
	SlMKK9	Solyc03g097920.1.1	ATMKK9	PtMKK2-2	Potri.006G146500.1	ATMKK2
	SlMKK3	Solyc03g019850.2.1	ATMKK3	PtMKK5	Potri.008G009800.1	ATMKK5
				PtMKK9-2	Potri.008G183700.1	ATMKK9
				PtMKK7	Potri.010G049500.1	ATMKK7
				PtMKK4	Potri.010G249300.1	ATMKK4
				PtMKK9-1	Potri.012G043200.1	ATMKK9
				PtMKK9-3	Potri.015G030700.1	ATMKK9
				PtMKK2-1	Potri.018G050800.1	ATMKK2

	SlZIK10	Solyc10g009350.2.1	ATZIK10	PtZIK1	Potri.019G128600.1	ATZIK1
	SlZIK11-1	Solyc07g047990.1.1	ATZIK11	PtZIK4-1	Potri.004G093300.1	ATZIK4
	SlZIK11-2	Solyc07g065250.2.1	ATZIK11	PtZIK4-2	Potri.012G086700.1	ATZIK4
	SlZIK4-1	Solyc03g112140.2.1	ATZIK4	PtZIK4-3	Potri.013G155100.1	ATZIK4
	SlZIK4-2	Solyc06g071800.2.1	ATZIK4	PtZIK4-4	Potri.015G084600.1	ATZIK4
	SlZIK4-3	Solyc06g082470.2.1	ATZIK4	PtZIK4-5	Potri.016G134600.1	ATZIK4
	SlZIK4-4	Solyc09g018170.2.1	ATZIK4	PtZIK5-1	Potri.001G085500.1	ATZIK5
	SlZIK4-5	Solyc10g079130.1.1	ATZIK4	PtZIK5-2	Potri.003G145300.1	ATZIK5
ZIK	SlZIK5	Solyc01g096170.2.1	ATZIK5	PtZIK5-3	Potri.005G057300.1	ATZIK5
	SlZIK6	Solyc02g087590.1.1	ATZIK6	PtZIK5-4	Potri.010G225000.1	ATZIK5
	SlZIK7	Solyc10g009060.1.1	ATZIK7	PtZIK5-5	Potri.014G101500.1	ATZIK5
	SlZIK8-1	Solyc02g031860.2.1	ATZIK8	PtZIK8-1	Potri.008G037200.1	ATZIK8
	SlZIK8-2	Solyc05g041420.2.1	ATZIK8	PtZIK8-2	Potri.014G164100.1	ATZIK8
	SlZIK8-3	Solyc08g082980.2.1	ATZIK8	PtZIK9-1	Potri.005G049800.1	ATZIK9
	SlZIK8-4	Solyc09g076000.2.1	ATZIK8	PtZIK9-2	Potri.008G152300.1	ATZIK9
	SlZIK9	Solyc01g097840.2.1	ATZIK9	PtZIK9-3	Potri.010G087900.1	ATZIK9
				PtZIK9-4	Potri.013G036300.1	ATZIK9
	SlRaf1-1	Solyc04g076480.2.1	ATRaf1			
	SlRaf1-2	Solyc09g009090.2.1	ATRaf1	PtRaf1-1	Potri.002G111200.1	ATRaf1
	SlRaf10-1	Solyc08g007910.2.1	ATRaf10	PtRaf1-2	Potri.006G115800.1	ATRaf1
	SlRaf10-2	Solyc08g062140.1.1	ATRaf10	PtRaf1-3	Potri.016G095800.1	ATRaf1
	SlRaf11-1	Solyc10g085670.1.1	ATRaf11	PtRaf10-1	Potri.010G146000.1	ATRaf10
	SlRaf11-2	Solyc12g099250.1.1	ATRaf11	PtRaf10-2	Potri.T122400.1	ATRaf10
	SlRaf13	Solyc12g013980.1.1	ATRaf13	PtRaf11	Potri.008G105100.1	ATRaf11
	SlRaf14	Solyc02g076780.2.1	ATRaf14	PtRaf12	Potri.001G108900.1	ATRaf12
	SlRaf15	Solyc07g055130.2.1	ATRaf15	PtRaf15-1	Potri.002G214400.1	ATRaf15
	SlRaf17-1	Solyc01g010950.2.1	ATRaf17	PtRaf15-2	Potri.014G142700.1	ATRaf15
	SlRaf17-2	Solyc10g017490.1.1	ATRaf17	PtRaf16-1	Potri.001G123500.1	ATRaf16
RAF	SlRaf19-1	Solyc03g114310.2.1	ATRaf19	PtRaf16-2	Potri.003G110000.1	ATRaf16
	SlRaf19-2	Solyc04g014690.2.1	ATRaf19	PtRaf16-3	Potri.005G095200.1	ATRaf16
	SlRaf19-3	Solyc11g012050.1.1	ATRaf19	PtRaf16-4	Potri.007G069500.1	ATRaf16
	SlRaf20-1	Solyc07g006760.2.1	ATRaf20	PtRaf17-1	Potri.008G090800.1	ATRaf17
	SlRaf20-2	Solyc07g008400.1.1	ATRaf20	PtRaf17-2	Potri.010G164500.1	ATRaf17
	SlRaf21	Solyc11g033270.1.1	ATRaf21	PtRaf18-1	Potri.003G176100.1	ATRaf18
	SlRaf22-1	Solyc07g042680.2.1	ATRaf22	PtRaf18-2	Potri.006G170700.1	ATRaf18
	SlRaf22-2	Solyc07g042890.2.1	ATRaf22	PtRaf18-3	Potri.018G114300.1	ATRaf18
	SlRaf22-3	Solyc12g009340.1.1	ATRaf22	PtRaf19-1	Potri.004G001900.1	ATRaf19
	SlRaf24	Solyc07g007140.2.1	ATRaf24	PtRaf19-2	Potri.011G022800.1	ATRaf19
	SlRaf27	Solyc02g071740.2.1	ATRaf27	PtRaf19-3	Potri.012G080000.1	ATRaf19
	SlRaf3-1	Solyc06g068980.2.1	ATRaf3	PtRaf19-4	Potri.015G075200.1	ATRaf19

SlRaf3-2	Solyc08g080460.1.1	ATRaf3	PtRaf2	Potri.012G048400.1	ATRaf2
SlRaf3-3	Solyc09g018060.2.1	ATRaf3	PtRaf20-1	Potri.001G052000.1	ATRaf20
SlRaf3-4	Solyc10g085570.1.1	ATRaf3	PtRaf20-2	Potri.018G095800.1	ATRaf20
SlRaf30-1	Solyc01g111880.2.1	ATRaf30	PtRaf22-1	Potri.006G279900.1	ATRaf22
SlRaf30-2	Solyc10g055720.1.1	ATRaf30	PtRaf22-2	Potri.006G280000.1	ATRaf22
SlRaf30-3	Solyc12g094410.1.1	ATRaf30	PtRaf22-3	Potri.018G001800.1	ATRaf22
SlRaf33-1	Solyc03g121780.1.1	ATRaf33	PtRaf22-4	Potri.018G001900.1	ATRaf22
SlRaf33-2	Solyc06g071410.2.1	ATRaf33	PtRaf23-1	Potri.007G142100.1	ATRaf23
SlRaf33-3	Solyc12g062280.1.1	ATRaf33	PtRaf23-2	Potri.017G007800.1	ATRaf23
SlRaf34	Solyc02g093410.2.1	ATRaf34	PtRaf23-3	Potri.017G008300.1	ATRaf23
SlRaf36-1	Solyc02g078140.2.1	ATRaf36	PtRaf24	Potri.006G190200.1	ATRaf24
SlRaf36-2	Solyc03g006400.2.1	ATRaf36	PtRaf27	Potri.004G064400.1	ATRaf27
SlRaf4-1	Solyc03g119140.2.1	ATRaf4	PtRaf29-1	Potri.005G106200.1	ATRaf29
SlRaf4-2	Solyc10g083610.1.1	ATRaf4	PtRaf29-2	Potri.007G060600.1	ATRaf29
SlRaf47	Solyc07g055870.2.1	ATRaf47	PtRaf3-1	Potri.006G229400.1	ATRaf3
SlRaf5	Solyc01g097980.2.1	ATRaf5	PtRaf3-2	Potri.013G034800.1	ATRaf3
SlRaf7	Solyc01g059860.2.1	ATRaf7	PtRaf3-3	Potri.015G040100.1	ATRaf3
			PtRaf3-4	Potri.016G134900.1	ATRaf3
			PtRaf3-5	Potri.018G053800.1	ATRaf3
			PtRaf30-1	Potri.002G015400.1	ATRaf30
			PtRaf30-2	Potri.002G070000.1	ATRaf30
			PtRaf30-3	Potri.004G179100.1	ATRaf30
			PtRaf30-4	Potri.004G179500.1	ATRaf30
			PtRaf30-5	Potri.005G190200.1	ATRaf30
			PtRaf30-6	Potri.005G246500.1	ATRaf30
			PtRaf30-7	Potri.009G139400.1	ATRaf30
			PtRaf33-1	Potri.001G351300.1	ATRaf33
			PtRaf33-2	Potri.012G079000.1	ATRaf33
			PtRaf33-3	Potri.015G074400.1	ATRaf33
			PtRaf33-4	Potri.017G072900.1	ATRaf33
			PtRaf34-1	Potri.001G343900.1	ATRaf34
			PtRaf34-2	Potri.017G065500.1	ATRaf34
			PtRaf36-1	Potri.005G082200.1	ATRaf36
			PtRaf36-2	Potri.007G085300.1	ATRaf36
			PtRaf39-1	Potri.002G049700.1	ATRaf39
			PtRaf39-2	Potri.005G213200.1	ATRaf39
			PtRaf39-3	Potri.008G156000.1	ATRaf39
			PtRaf39-4	Potri.010G083500.1	ATRaf39
			PtRaf5-1	Potri.005G047600.1	ATRaf5
			PtRaf5-2	Potri.005G153100.1	ATRaf5

				PtRaf5-3	Potri.010G234700.1	ATRaf5
				PtRaf6	Potri.008G025800.1	ATRaf6
				PtRaf7	Potri.003G122700.1	ATRaf7
	SlMAPKKK10-1	Solyc01g104530.2.1	ATMAPKKK10	PtMAPKKK1	Potri.013G022700.1	ATMAPKKK1
	SlMAPKKK10-2	Solyc07g053170.2.1	ATMAPKKK10	PtMAPKKK10-1	Potri.002G088900.1	ATMAPKKK10
	SlMAPKKK12	Solyc01g079750.2.1	ATMAPKKK12	PtMAPKKK10-2	Potri.005G172200.1	ATMAPKKK10
	SlMAPKKK15-1	Solyc01g103240.2.1	ATMAPKKK15	PtMAPKKK11	Potri.010G038700.1	ATMAPKKK11
	SlMAPKKK15-2	Solyc07g051870.1.1	ATMAPKKK15	PtMAPKKK12-1	Potri.008G149500.1	ATMAPKKK12
	SlMAPKKK15-3	Solyc07g051930.1.1	ATMAPKKK15	PtMAPKKK12-2	Potri.010G092000.1	ATMAPKKK12
	SlMAPKKK15-4	Solyc08g076490.2.1	ATMAPKKK15	PtMAPKKK13	Potri.001G278600.1	ATMAPKKK13
	SlMAPKKK16	Solyc07g064820.1.1	ATMAPKKK16	PtMAPKKK14	Potri.009G073200.1	ATMAPKKK14
	SlMAPKKK17-1	Solyc07g051860.1.1	ATMAPKKK17	PtMAPKKK17	Potri.002G228200.1	ATMAPKKK17
	SlMAPKKK17-2	Solyc07g051920.1.1	ATMAPKKK17	PtMAPKKK18	Potri.014G155000.1	ATMAPKKK18
	SlMAPKKK18-1	Solyc01g005030.2.1	ATMAPKKK18	PtMAPKKK2	Potri.005G033400.1	ATMAPKKK2
	SlMAPKKK18-2	Solyc07g051880.1.1	ATMAPKKK18	PtMAPKKK21-1	Potri.005G139200.1	ATMAPKKK21
	SlMAPKKK18-3	Solyc07g051890.1.1	ATMAPKKK18	PtMAPKKK21-2	Potri.005G139300.1	ATMAPKKK21
	SlMAPKKK18-4	Solyc12g005360.1.1	ATMAPKKK18	PtMAPKKK21-3	Potri.007G044800.1	ATMAPKKK21
	SlMAPKKK2	Solyc01g098980.2.1	ATMAPKKK2	PtMAPKKK3	Potri.002G073100.1	ATMAPKKK3
	SlMAPKKK20-1	Solyc02g064930.1.1	ATMAPKKK20	PtMAPKKK4-1	Potri.001G102900.1	ATMAPKKK4
MAPKKK	SlMAPKKK20-2	Solyc02g090990.1.1	ATMAPKKK20	PtMAPKKK4-2	Potri.003G129000.1	ATMAPKKK4
	SlMAPKKK20-3	Solyc03g117640.1.1	ATMAPKKK20	PtMAPKKK4-3	Potri.005G062500.1	ATMAPKKK4
	SlMAPKKK20-4	Solyc08g069090.1.1	ATMAPKKK20	PtMAPKKK4-4	Potri.007G106800.1	ATMAPKKK4
	SlMAPKKK21-1	Solyc02g064980.1.1	ATMAPKKK21	PtMAPKKK4-5	Potri.012G143900.1	ATMAPKKK4
	SlMAPKKK21-2	Solyc02g090970.1.1	ATMAPKKK21	PtMAPKKK4-6	Potri.015G146700.1	ATMAPKKK4
	SlMAPKKK21-3	Solyc02g090980.1.1	ATMAPKKK21	PtMAPKKK5-1	Potri.002G129100.1	ATMAPKKK5
	SlMAPKKK21-4	Solyc06g068510.1.1	ATMAPKKK21	PtMAPKKK5-2	Potri.005G135100.1	ATMAPKKK5
	SlMAPKKK21-5	Solyc07g047910.1.1	ATMAPKKK21	PtMAPKKK5-3	Potri.007G039800.1	ATMAPKKK5
	SlMAPKKK3-1	Solyc04g064590.1.1	ATMAPKKK3	PtMAPKKK5-4	Potri.014G035500.1	ATMAPKKK5
	SlMAPKKK3-2	Solyc08g081210.2.1	ATMAPKKK3	PtMAPKKK7-1	Potri.005G094200.1	ATMAPKKK7
	SlMAPKKK4-1	Solyc03g025360.2.1	ATMAPKKK4	PtMAPKKK7-2	Potri.007G070200.1	ATMAPKKK7
	SlMAPKKK4-2	Solyc06g036080.2.1	ATMAPKKK4	PtMAPKKK8	Potri.017G029500.1	ATMAPKKK8
	SlMAPKKK4-3	Solyc11g006000.1.1	ATMAPKKK4	PtMAPKKK9-1	Potri.011G007700.1	ATMAPKKK9
	SlMAPKKK5-1	Solyc02g065110.2.1	ATMAPKKK5	PtMAPKKK9-2	Potri.018G026100.1	ATMAPKKK9
	SlMAPKKK5-2	Solyc02g090430.2.1	ATMAPKKK5	PtMAPKKK9-3	Potri.001G018000.1	ATMAPKKK9
	SlMAPKKK8-1	Solyc04g079400.2.1	ATMAPKKK8			
	SlMAPKKK8-2	Solyc12g088940.1.1	ATMAPKKK8			

Supplementary Table S2. The CV values of all *EuMAPK* cascades in various organs at different developmental stages

Gene name	N	N*	Mean value	SE mean	SD	Variance	CV	Minimum	Maximum
EuZIK9	21	0	84.27	5.24	24.03	577.63	28.52	46.85	139.88
EuZIK8-5	21	0	0.294	0.113	0.518	0.269	176.19	0	1.908
EuZIK8-4	21	0	2.212	0.429	1.966	3.864	88.87	0	8.382
EuZIK8-3	21	0	66	14	64.1	4105.6	97.01	5.3	250.7
EuZIK8-2	21	0	11.078	0.851	3.899	15.204	35.2	5.448	20.863
EuZIK8-1	21	0	18.72	4.4	20.18	407.22	107.78	3.39	83.44
EuZIK4-3	21	0	70.92	6.79	31.1	967.11	43.85	32.51	140.02
EuZIK4-2	21	0	279.1	51	233.6	54587.7	83.73	29.8	887.8
EuZIK4-1	21	0	60.08	3.55	16.29	265.38	27.12	29.78	90.57
EuZIK11	21	0	32.16	1.73	7.91	62.49	24.58	20.81	49.06
EuZIK1	21	0	990	122	561	314797	56.65	235	2060
EuRAF8	21	0	71.21	9.08	41.63	1733.02	58.46	7.62	183.14
EuRAF5	21	0	36.07	2.26	10.36	107.4	28.73	24.16	58.85
EuRAF39-2	21	0	238.7	23.2	106.1	11264.6	44.47	112.7	493.2
EuRAF39-1	21	0	23.75	3.35	15.35	235.72	64.66	7.02	66.03
EuRAF36	21	0	60.95	6.35	29.09	846.43	47.73	13.08	121.03
EuRAF34-2	21	0	150.5	13.5	61.7	3811.6	41.03	72.3	290.1
EuRAF34-1	21	0	39.7	4.28	19.59	383.93	49.36	17.01	106.99
EuRAF3-4	21	0	73.05	6.15	28.19	794.58	38.59	35.67	125.8
EuRAF33-2	21	0	85.13	8.93	40.92	1674.33	48.07	24.22	211.07
EuRAF33-1	21	0	187.55	9.65	44.24	1957.43	23.59	130.45	278.65
EuRAF3-3	21	0	92.82	8.86	40.6	1648.38	43.74	35.2	178.5
EuRAF3-2	21	0	42.2	3.16	14.47	209.4	34.29	22.52	79.16
EuRAF3-1	21	0	102.79	5.18	23.75	563.85	23.1	69.64	164.44
EuRAF31	21	0	106.67	6.26	28.71	824.15	26.91	63.92	148.83
EuRAF30-4	21	0	177.8	17.7	81.2	6588	45.65	65.6	348.8
EuRAF30-3	21	0	166.3	17.3	79.1	6263.2	47.6	53.6	337.9
EuRAF30-2	21	0	141.2	42.6	195.3	38144.6	138.35	13	694
EuRAF30-1	21	0	82.8	14.7	67.3	4528.2	81.24	8	219.6
EuRAF29	21	0	128.4	18.4	84.4	7121	65.71	33.5	377.8
EuRAF2-3	21	0	149.7	85.8	393.1	154524.3	262.63	0	1336.3
EuRAF22-2	21	0	69.51	6.21	28.46	809.79	40.94	38.85	157.73
EuRAF22-1	21	0	120.7	6.51	29.84	890.49	24.72	69.8	165.19
EuRAF2-2	21	0	101	32.8	150.1	22531.9	148.63	6.4	584.5
EuRAF2-1	21	0	55.83	4.05	18.56	344.41	33.24	26.32	96.22
EuRAF20-4	21	0	38.88	2.85	13.07	170.74	33.61	23.86	73.09
EuRAF20-3	21	0	92.5	12	54.9	3019.3	59.4	37.9	233.3

EuRAF20-2	21	0	9.07	1.1	5.04	25.39	55.57	4.3	27.04
EuRAF20-1	21	0	23.36	1.62	7.43	55.24	31.81	14.03	48.62
EuRAF19-2	21	0	44.2	7.4	33.9	1148.87	76.68	2.48	156.37
EuRAF19-1	21	0	0.342	0.137	0.629	0.396	184.18	0	2.516
EuRAF16-2	21	0	57.96	3.7	16.97	287.83	29.27	26.17	85.63
EuRAF16-1	21	0	168.8	10.4	47.7	2275.4	28.25	88.4	315.4
EuRAF15	21	0	182	11.3	51.9	2695.8	28.52	113.7	309.3
EuRAF10	21	0	82.4	7.43	34.06	1160.13	41.34	21.18	134.74
EuMPK9-3	21	0	143	20.4	93.6	8758	65.46	14.6	435.5
EuMPK6	21	0	275.8	16	73.3	5365.9	26.56	163.2	412.2
EuMPK15	21	0	236.3	18.3	84.1	7064.8	35.57	100.1	446.8
EuMPK16	21	0	177.3	15.4	70.7	4997.7	39.88	65.4	333.6
EuMPK4-1	21	0	169.8	18.3	84	7051.3	49.45	69.1	363.9
EuMPK9-2	21	0	145.7	14.8	67.7	4583.7	46.46	34.5	280.9
EuMPK9-1	21	0	136.8	11.6	53.1	2814.7	38.79	51.9	211.7
EuMPK3	21	0	130.6	26.4	121.1	14661.8	92.75	6.8	481
EuMPK4-3	21	0	67.83	8.49	38.9	1513.19	57.35	27.99	155.21
EuMPK4-2	21	0	38.02	9.05	41.46	1718.63	109.05	8.6	165.89
EuMPK11	21	0	27.97	7.81	35.81	1282.17	128.03	0.77	139.83
EuMPK2-1	21	0	0.294	0.105	0.483	0.234	164.54	0	1.584
EuMPK2-2	21	0	0.0967	0.0359	0.1646	0.0271	170.15	0	0.4319
EuMKK9	21	0	43.56	9.48	43.44	1887.35	99.73	1.2	161.59
EuMKK6	21	0	17.99	5.13	23.5	552.09	130.61	0	79.5
EuMKK5	21	0	42.09	3.82	17.53	307.15	41.64	9.05	74.59
EuMKK3	21	0	73.79	3.85	17.63	310.7	23.89	47.8	134.77
EuMKK2	21	0	786	56.6	259.5	67314.3	33.01	376.3	1419.4
EuMEKK5	21	0	83.81	4.68	21.44	459.84	25.59	39.8	123.84
EuMEKK4	21	0	206	13	59.8	3574.8	29.03	102.4	352.5
EuMEKK3-3	21	0	5.12	2.09	9.57	91.61	186.9	0	35.58
EuMEKK3-2	21	0	124.51	9.34	42.8	1831.65	34.37	62.62	227.56
EuMEKK3-1	21	0	38.3	2.67	12.22	149.41	31.91	20.98	63.85
EuMEKK21	21	0	13.73	5.15	23.58	556.01	171.68	0	107.14
EuMEKK2	21	0	25.51	3.86	17.67	312.11	69.26	8.17	66.08
EuMEKK16	21	0	1.769	0.426	1.951	3.806	110.25	0	7.814
EuMEKK13	21	0	7.74	1.89	8.67	75.11	112.02	0.7	27.02
EuMEKK12	21	0	37.45	7.32	33.55	1125.44	89.57	4.47	128.42
EuMEKK10-2	21	0	90.04	6.81	31.23	975.25	34.68	44.15	191.08
EuMEKK10-1	21	0	67.01	7.42	34.01	1156.98	50.76	35.9	154.51

Supplementary Table S3. The CV values of all *EuMAPK* cascades in fruits at various developmental stages

Gene name	N	N*	Mean value	SE mean	SD	Variance	CV	Minimum	Maximum
EuRAF22-2	6	0	60.56	2.37	5.8	33.66	9.58	51.8	65.74
EuMEKK10-2	6	0	85.78	3.77	9.24	85.29	10.77	70.83	96.72
EuRAF5	6	0	28.59	1.27	3.1	9.63	10.85	24.16	33.19
EuMEKK5	6	0	90.8	4.16	10.19	103.9	11.23	79.72	102.74
EuRAF20-4	6	0	31.33	1.67	4.09	16.72	13.05	25.21	36.24
EuRAF33-1	6	0	189.5	10.2	25	623.1	13.17	157.5	226
EuMEKK4	6	0	213	11.9	29	842.6	13.63	167.8	252
EuRAF30-3	6	0	127.85	7.15	17.52	307	13.7	108.76	153.03
EuRAF22-1	6	0	128.8	7.38	18.08	327.02	14.04	98.27	151.49
EuRAF31	6	0	89.73	5.41	13.24	175.33	14.76	80.34	116
EuMKK3	6	0	65.32	4.24	10.39	107.98	15.91	54.86	85.19
EuRAF3-1	6	0	106.03	7.06	17.29	298.8	16.3	86.71	126.43
EuRAF15	6	0	174.1	12.2	29.8	886.8	17.11	138.7	227.8
EuMPK6	6	0	323.2	23.2	56.8	3226.6	17.58	242.3	409.6
EuMPK4-2	6	0	19.81	1.43	3.51	12.31	17.71	14.53	24.65
EuMPK9-1	6	0	166.7	12.6	30.9	952.2	18.51	125.7	211.7
EuMKK5	6	0	44.77	3.58	8.76	76.71	19.56	33.07	56.75
EuZIK8-2	6	0	11.892	0.971	2.379	5.661	20.01	8.672	14.378
EuMKK2	6	0	873.7	72.5	177.5	31514.7	20.32	700.1	1213.9
EuZIK1	6	0	872	73.7	180.6	32622.3	20.71	599.6	1056.7
EuRAF20-1	6	0	22.73	1.93	4.73	22.35	20.8	16.99	30.64
EuRAF2-1	6	0	47.72	4.29	10.51	110.43	22.02	31.18	58.75
EuZIK11	6	0	37.48	3.47	8.5	72.22	22.68	26.39	49.06
EuRAF30-4	6	0	162.8	15.1	37	1368.9	22.73	106.4	197.1
EuMEKK10-1	6	0	51.12	4.76	11.66	135.97	22.81	35.9	66.84
EuRAF16-1	6	0	151.4	14.6	35.8	1284.2	23.66	88.4	197.5
EuMPK9-2	6	0	157.1	15.3	37.4	1398.4	23.8	97.4	204.5
EuRAF20-2	6	0	7.598	0.745	1.824	3.326	24	5.573	10.009
EuRAF19-2	6	0	37.96	3.72	9.11	83.04	24.01	25.15	51.2
EuZIK4-2	6	0	247.9	25.2	61.7	3807.6	24.89	162.6	317.8
EuZIK4-1	6	0	63.46	6.69	16.39	268.74	25.83	36.33	86.22
EuMEKK3-2	6	0	106.3	11.3	27.8	770.6	26.12	62.6	137.1
EuRAF8	6	0	65.26	6.98	17.1	292.28	26.2	31.97	79.37
EuZIK9	6	0	78.88	8.75	21.43	459.27	27.17	53.3	115.45
EuRAF16-2	6	0	59.17	6.62	16.22	263.19	27.42	40.84	79.56
EuMPK4-1	6	0	172.1	20.8	51	2605.8	29.66	124.2	260.6
EuMPK16	6	0	160.3	19.5	47.8	2287.1	29.83	87.8	234.5

EuRAF3-2	6	0	51.34	6.62	16.21	262.64	31.57	33.07	79.16
EuRAF10	6	0	86.7	11.2	27.4	750.7	31.59	61.6	128.4
EuMPK15	6	0	259.4	33.7	82.6	6824.8	31.85	152	372.9
EuRAF3-3	6	0	89.7	11.8	28.9	835.4	32.23	60.5	140.2
EuRAF29	6	0	108	14.4	35.3	1245.5	32.68	63	170.6
EuRAF34-2	6	0	131.8	17.8	43.7	1911.4	33.18	78	179.7
EuZIK4-3	6	0	57.98	7.87	19.28	371.8	33.25	32.51	81.12
EuMKK9	6	0	55.93	7.63	18.68	349.03	33.4	33.22	79.6
EuRAF3-4	6	0	74.4	10.5	25.6	657.5	34.48	41.4	112.9
EuMEKK3-1	6	0	34.03	5.49	13.44	180.64	39.49	20.98	58.19
EuRAF20-3	6	0	81.2	13.6	33.4	1116.5	41.15	37.9	137.6
EuRAF36	6	0	65.5	11.8	29	839	44.22	40.6	121
EuRAF39-2	6	0	281.4	55.8	136.8	18713.3	48.62	137.5	493.2
EuMPK4-3	6	0	83.6	16.8	41.2	1701.5	49.34	29.7	144
EuMKK6	6	0	12.1	2.47	6.04	36.53	49.95	4.22	21.63
EuRAF30-2	6	0	116.1	24.2	59.2	3501.3	50.95	30.9	204.2
EuZIK8-1	6	0	43.33	9.14	22.4	501.54	51.68	19.96	83.44
EuRAF34-1	6	0	57.5	12.2	29.9	895.2	52.04	17	107
EuRAF30-1	6	0	97	20.7	50.6	2563.7	52.21	53.8	187.2
EuMPK9-3	6	0	136.7	32.6	79.9	6383.4	58.46	64.3	260.5
EuRAF33-2	6	0	100.1	24.6	60.3	3634.3	60.21	57.1	211.1
EuMPK3	6	0	155.6	38.8	94.9	9011	61.01	74.1	338.3
EuMEKK12	6	0	25.26	6.75	16.55	273.77	65.51	4.47	49.04
EuRAF2-2	6	0	61.2	17.4	42.6	1812.2	69.51	14.4	128.2
EuRAF39-1	6	0	31.92	9.24	22.64	512.55	70.93	13.36	66.03
EuMEKK2	6	0	25.75	8.18	20.05	401.87	77.84	10.61	61.13
EuZIK8-3	6	0	96.3	34.2	83.8	7016.9	86.97	44.4	250.7
EuMEKK13	6	0	12.24	4.4	10.79	116.33	88.14	1.65	27.02
EuMEKK3-3	6	0	1.961	0.729	1.786	3.188	91.05	0.431	4.554
EuMEKK16	6	0	2.89	1.11	2.71	7.35	93.84	0.71	7.81
EuRAF2-3	6	0	77.2	36.9	90.5	8185.9	117.14	2.9	212.1
EuMPK11	6	0	27.1	13.9	34	1158.6	125.63	9.1	96.2
EuMEKK21	6	0	30.4	16.2	39.6	1565	129.98	2.3	107.1
EuZIK8-4	6	0	2.28	1.27	3.11	9.64	136.34	0.23	8.38
EuRAF19-1	6	0	0.451	0.254	0.621	0.386	137.9	0	1.59
EuMPK2-2	6	0	0.1244	0.0786	0.1926	0.0371	154.92	0	0.3751
EuMPK2-1	6	0	0.389	0.25	0.613	0.376	157.79	0	1.584
EuZIK8-5	6	0	0.0325	0.0325	0.0796	0.0063	244.95	0	0.195

Supplementary Table S4. The CV values of all *EuMAPK* cascades in leaves at various developmental stages

Gene name	N	N*	Mean value	SE mean	SD	Variance	CV	Minimum	Maximum
EuRAF34-1	6	0	30.893	0.207	0.506	0.256	1.64	30.222	31.465
EuRAF33-2	6	0	79.74	2.86	7.01	49.16	8.79	70.87	89.14
EuMEKK5	6	0	83.28	4.28	10.48	109.82	12.58	74.32	103.29
EuMPK4-3	6	0	51.93	2.77	6.78	45.9	13.05	44.73	61.79
EuMKK3	6	0	74.13	4.13	10.1	102.11	13.63	59.22	82.46
EuMPK6	6	0	269.5	15.6	38.1	1453.4	14.14	241.8	345.7
EuRAF39-1	6	0	18.44	1.07	2.61	6.81	14.16	15.05	22.91
EuMKK5	6	0	60.01	3.51	8.6	73.91	14.33	48.57	74.59
EuZIK11	6	0	27.77	1.73	4.23	17.89	15.23	23.72	34.5
EuMPK15	6	0	216.5	13.6	33.3	1108.2	15.38	179.4	269.4
EuRAF36	6	0	90.75	5.73	14.04	197.12	15.47	75.07	113.33
EuMEKK3-1	6	0	35.36	2.58	6.32	39.92	17.87	25.02	42.57
EuMPK16	6	0	252.8	18.6	45.6	2075.8	18.03	216.2	333.6
EuRAF30-3	6	0	130.25	9.62	23.56	554.98	18.09	97.3	151.74
EuRAF20-1	6	0	24.55	1.85	4.53	20.52	18.45	18.81	29.66
EuRAF33-1	6	0	234.7	18.7	45.8	2097.8	19.52	152.7	278.6
EuRAF20-4	6	0	33.77	2.74	6.7	44.91	19.84	23.86	42.39
EuRAF15	6	0	152.2	12.4	30.3	918.6	19.91	115.4	186.1
EuRAF20-3	6	0	55.3	4.51	11.04	121.81	19.96	45.47	74.18
EuZIK4-1	6	0	71.75	6.06	14.84	220.08	20.68	51.29	90.57
EuZIK1	6	0	1694	143	351	122956	20.7	1029	2060
EuRAF34-2	6	0	138.8	11.8	29	841.4	20.9	107	170.6
EuRAF29	6	0	81.87	7.01	17.18	295.26	20.99	57.53	97.25
EuMPK9-2	6	0	213.6	18.6	45.6	2075.5	21.32	170.5	280.9
EuMEKK10-2	6	0	100.72	8.78	21.5	462.34	21.35	83.62	130.81
EuMEKK10-1	6	0	44.45	3.97	9.73	94.64	21.89	36.04	63.5
EuRAF3-3	6	0	47.51	4.26	10.45	109.1	21.98	35.2	61.36
EuRAF20-2	6	0	7.903	0.709	1.738	3.02	21.99	4.723	9.48
EuRAF16-1	6	0	165.8	15.4	37.8	1429.5	22.8	100.2	200.8
EuRAF2-1	6	0	62.06	5.8	14.21	201.8	22.89	47.07	86.6
EuRAF5	6	0	45.4	4.29	10.5	110.35	23.14	33.42	58.85
EuRAF22-1	6	0	126	11.9	29.2	850.7	23.15	85.8	165.2
EuRAF22-2	6	0	59.77	5.81	14.23	202.6	23.81	38.85	80.13
EuZIK4-3	6	0	52.14	5.12	12.55	157.5	24.07	34.81	72.94
EuZIK9	6	0	80.73	8	19.6	384.32	24.28	50.78	107.76
EuMEKK3-2	6	0	101.4	10.3	25.2	634.6	24.84	79.6	147.9
EuMPK9-1	6	0	144.1	14.7	36	1294	24.97	102.8	193.4

EuRAF31	6	0	123.1	12.6	30.8	951	25.05	63.9	148.8
EuRAF3-4	6	0	82.27	8.67	21.23	450.73	25.81	51.09	109.88
EuRAF3-1	6	0	114.1	12.5	30.6	935.6	26.81	79.8	164.4
EuMKK2	6	0	950	107	262	68655	27.58	692	1419
EuMEKK4	6	0	230.5	27	66.1	4369	28.67	170.2	352.5
EuRAF30-4	6	0	167.3	19.6	48.1	2312.9	28.75	75.1	206.8
EuRAF10	6	0	101.7	12	29.4	866.2	28.93	64.3	134.7
EuRAF16-2	6	0	61.89	7.33	17.96	322.41	29.01	38.66	85.63
EuRAF3-2	6	0	44.36	5.46	13.37	178.87	30.15	28.94	67.47
EuMEKK12	6	0	25.79	3.37	8.26	68.22	32.03	15.23	37.51
EuMPK4-1	6	0	200.2	26.6	65.2	4246	32.56	109.3	290.3
EuMPK4-2	6	0	20.21	3.01	7.38	54.45	36.51	8.6	27.49
EuRAF39-2	6	0	291.5	44.1	108.1	11685.7	37.09	147	459.7
EuMEKK2	6	0	16.54	2.53	6.21	38.55	37.54	8.17	23.81
EuZIK4-2	6	0	573.2	89.5	219.3	48074.8	38.25	205.2	887.8
EuRAF8	6	0	110.9	17.4	42.6	1813.1	38.39	68.1	183.1
EuRAF2-3	6	0	3.709	0.59	1.446	2.091	38.98	2.534	6.362
EuRAF30-2	6	0	24.28	4.03	9.87	97.49	40.67	13.04	39.43
EuZIK8-1	6	0	6.66	1.11	2.72	7.38	40.78	4.03	11.78
EuZIK8-2	6	0	12.59	2.24	5.49	30.16	43.63	5.45	20.86
EuMEKK13	6	0	1.593	0.289	0.709	0.502	44.47	0.704	2.33
EuZIK8-3	6	0	68.2	12.6	30.9	954.3	45.29	38.3	123
EuMKK9	6	0	43.98	8.72	21.35	455.73	48.54	18.19	83.03
EuRAF2-2	6	0	13.53	2.87	7.04	49.56	52.03	6.42	26.08
EuRAF30-1	6	0	19.11	4.07	9.98	99.59	52.23	7.99	31
EuZIK8-4	6	0	2.756	0.608	1.489	2.219	54.04	0.899	4.898
EuMPK3	6	0	235.3	54.4	133.2	17749.9	56.62	112.4	481
EuMEKK16	6	0	2.447	0.655	1.604	2.572	65.54	0.415	4.996
EuMPK9-3	6	0	180.4	52	127.5	16243.5	70.66	100	435.5
EuMKK6	6	0	4.4	1.29	3.17	10.04	72.03	1.26	9.28
EuRAF19-2	6	0	24.37	7.49	18.34	336.48	75.27	2.48	47.91
EuMEKK21	6	0	13.31	4.12	10.08	101.69	75.75	1.47	28.99
EuMPK11	6	0	42.6	20.7	50.7	2566.9	118.81	7	139.8
EuMEKK3-3	6	0	0.1438	0.0913	0.2236	0.05	155.54	0	0.4629
EuMPK2-1	6	0	0.378	0.24	0.589	0.347	155.64	0	1.519
EuMPK2-2	6	0	0.1292	0.0825	0.2021	0.0409	156.42	0	0.4319
EuRAF19-1	6	0	0.607	0.398	0.976	0.952	160.73	0	2.516
EuZIK8-5	6	0	0.0327	0.0327	0.08	0.0064	244.95	0	0.196

Supplementary Table S5. The AtMAPKs, AtMAPKKs, and AtMAPKKKs used as the queries for the local Blast search in this study

Gene Short Name	Locus (TAIR)
AtMAPK1	AT1G10210
AtMAPK2	AT1G59580
AtMAPK3	AT3G45640
AtMAPK4	AT4G01370
AtMAPK5	AT4G11330
AtMAPK6	AT2G43790
AtMAPK7	AT2G18170
AtMAPK8	AT1G18150
AtMAPK9	AT3G18040
AtMAPK10	AT3G59790
AtMAPK11	AT1G01560
AtMAPK12	AT2G46070
AtMAPK13	AT1G07880
AtMAPK14	AT4G36450
AtMAPK15	AT1G73670
AtMAPK16	AT5G19010
AtMAPK17	AT2G01450
AtMAPK18	AT1G53510
AtMAPK19	AT3G14720
AtMAPK20	AT2G42880
AtMKK1	AT4G26070
AtMKK2	AT4G29810
AtMKK3	AT5G40440
AtMKK4	AT1G51660
AtMKK5	AT3G21220
AtMKK6	AT5G56580
AtMKK7	AT1G18350
AtMKK8	AT3G06230
AtMKK9	AT1G73500
AtMKK10	AT1G32320
AtZIK1	AT3G51630
AtZIK2	AT5G58350
AtZIK3	AT3G22420
AtZIK4	AT3G04910
AtZIK5	AT3G18750
AtZIK6	AT5G41990

AtZIK7	AT1G49160
AtZIK8	AT5G55560
AtZIK9	AT5G28080
AtZIK10	AT1G64630
AtZIK11	AT3G48260
AtRaf1	AT5G03730
AtRaf2	AT1G08720
AtRaf3	AT5G11850
AtRaf4	AT1G18160
AtRaf5	AT1G73660
AtRaf6	AT4G24480
AtRaf7	AT3G06620
AtRaf8	AT3G06630
AtRaf9	AT3G06640
AtRaf10	AT5G49470
AtRaf11	AT1G67890
AtRaf12	AT4G23050
AtRaf13	AT2G31010
AtRaf14	AT2G42630
AtRaf15	AT3G58640
AtRaf16	AT1G04700
AtRaf17	AT1G14000
AtRaf18	AT1G16270
AtRaf19	AT1G62400
AtRaf20	AT1G79570
AtRaf21	AT2G17700
AtRaf22	AT2G24360
AtRaf23	AT2G31800
AtRaf24	AT2G35050
AtRaf25	AT2G43850
AtRaf26	AT4G14780
AtRaf27	AT4G18950
AtRaf28	AT4G31170
AtRaf29	AT4G35780
AtRaf30	AT4G38470
AtRaf31	AT5G01850
AtRaf32	AT5G40540
AtRaf33	AT5G50000
AtRaf34	AT5G50180
AtRaf35	AT5G57610

AtRaf36	AT5G58950
AtRaf37	AT5G66710
AtRaf38	AT3G01490
AtRaf39	AT3G22750
AtRaf40	AT3G24720
AtRaf41	AT3G27560
AtRaf42	AT3G46920
AtRaf43	AT3G46930
AtRaf44	AT3G50720
AtRaf45	AT3G50730
AtRaf46	AT3G59830
AtRaf47	AT3G58760
AtRaf48	AT3G63260
AtMAPKKK1	AT1G09000
AtMAPKKK2	AT1G54960
AtMAPKKK3	AT1G53570
AtMAPKKK4	AT1G63700
AtMAPKKK5	AT5G66850
AtMAPKKK6	AT3G07980
AtMAPKKK7	AT3G13530
AtMAPKKK8	AT4G08500
AtMAPKKK9	AT4G08480
AtMAPKKK10	AT4G08470
AtMAPKKK11	AT4G12020
AtMAPKKK12	AT3G06030
AtMAPKKK13	AT1G07150
AtMAPKKK14	AT2G30040
AtMAPKKK15	AT5G55090
AtMAPKKK16	AT4G26890
AtMAPKKK17	AT2G32510
AtMAPKKK18	AT1G05100
AtMAPKKK19	AT5G67080
AtMAPKKK20	AT3G50310
AtMAPKKK21	AT4G36950

Supplementary Table S6. Primer sequences of eight screened genes for qRT-PCR expression analysis

Gene Short Name	Primer Sequecnes	Tm	Target Fragments Length
18S-F	5' TTCTTAGTTGGTGGAGCGATTT 3'	58.7	150 bp
18S-R	5' CCTGTTATTGCCTCAAACTTCC 3'	58.5	
EuRAF22-2-F	5' CGCAGAAAGTCGATGTTTATAGC	59.2	159 bp
EuRAF22-2-R	5' CAGTGAGAATGGGAAGGCAGT 3'	59.1	
EuMKK2-F	5' TCTCAAGCTTTCGCTGCCT 3'	58.5	163 bp
EuMKK2-R	5' CAACTGATTGTCTGATGGGTGTAT 3'	58.4	
EuRAF33-2-F	5' ATGTGATGTCTACAGTTTCGGGAT 3'	59.6	149 bp
EuRAF33-2-R	5' CCAATGAACTCGGGCAACA 3'	59.8	
EuMEKK21-F	5' TGCGAGGCGAAGTTATTGG 3'	59.6	134 bp
EuMEKK21-R	5' GTTGACGAGCGAAGCAGAGTT 3'	59.7	
EuRAF34-1-F	5' ATTGTGACATCGTGCTGGAAC 3'	58.0	98 bp
EuRAF34-1-R	5' GGCGGATGGATTGCTGAT 3'	58.6	
EuZIK1-F	5' GTCCGCCTGTCATACATCGA 3'	58.9	123 bp
EuZIK1-R	5' TGTGAGCGTGGTTTGAGTCTT 3'	58.1	
EuRAF2-3-F	5' TATTGCGTCGTTGATGCTGA 3'	58.3	179 bp
EuRAF2-3-R	5' CTTGGAAACTGATTTAGAGGTCG 3'	58.0	
EuMPK11-F	5' GACGATGCTAGTCTTGGCTTTC 3'	58.6	166 bp
EuMPK11-R	5' TGATTCGCTGGGTGGGAT 3'	58.8	

Supplementary Figures and legends

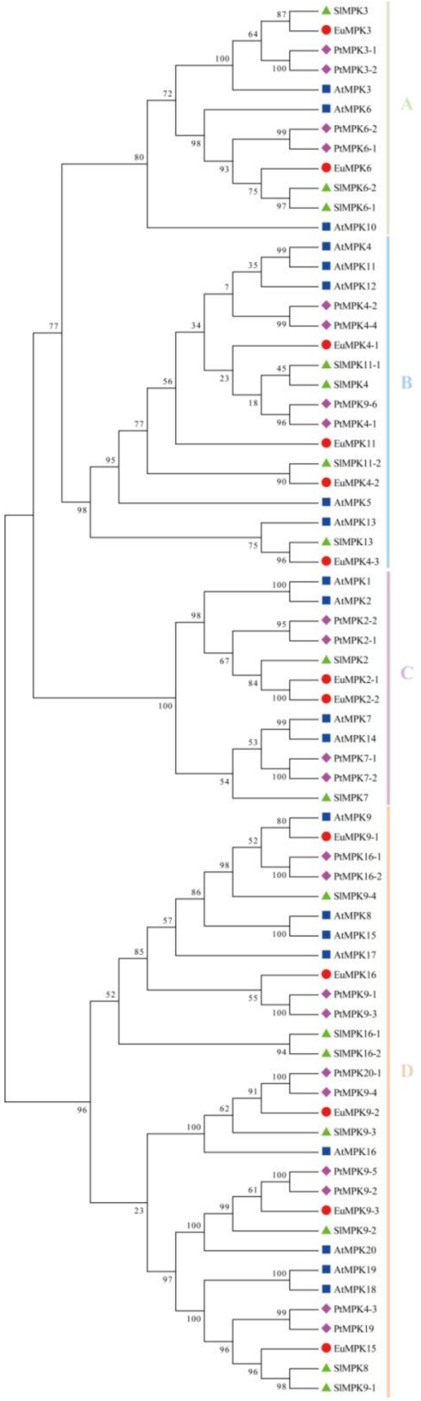

Supplementary Figure S1. The phytogenetic tree of MAPKs from *E. ulmoides*, *S. lycopesicum*, *A. thaliana* and *P. tremula*.

The Neighbor-joing (NJ) tree was constructed using MEGA 7.0 software with 1000 bootstrap replicates. Signs of different shapes represent MAPK proteins from *E. ulmoides* (red round, Eu), *S. lycopersicum* (green tirangel, Sl), *A. thaliana* (blue square, At), *P. tremula* (violet diamond, Pt)

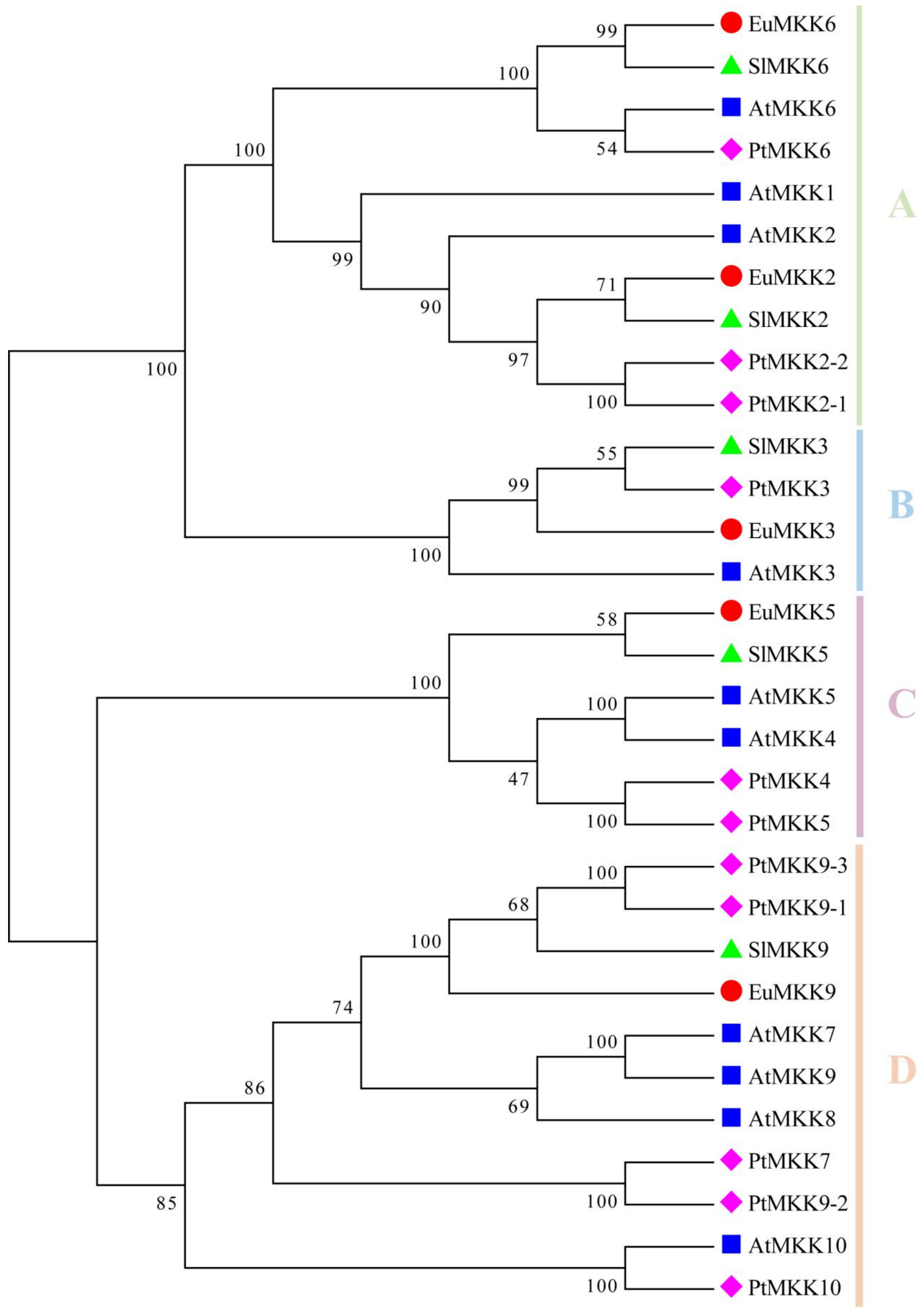

Supplementary Figure S2. The phytogenetic tree of MAPKKs from *E. ulmoides*, *S. lycopesicum*, *A. thaliana* and *P. tremula*. The Neighbor-joing (NJ) tree was constructed using MEGA 7.0 software with 1000 bootstrap replicates. Signs of different shapes represent MAPK proteins from *E. ulmoides* (red round, Eu), *S. lycopersicum* (green tirangel, Sl), *A. thaliana* (blue square, At), *P. tremula* (violet diamond, Pt)

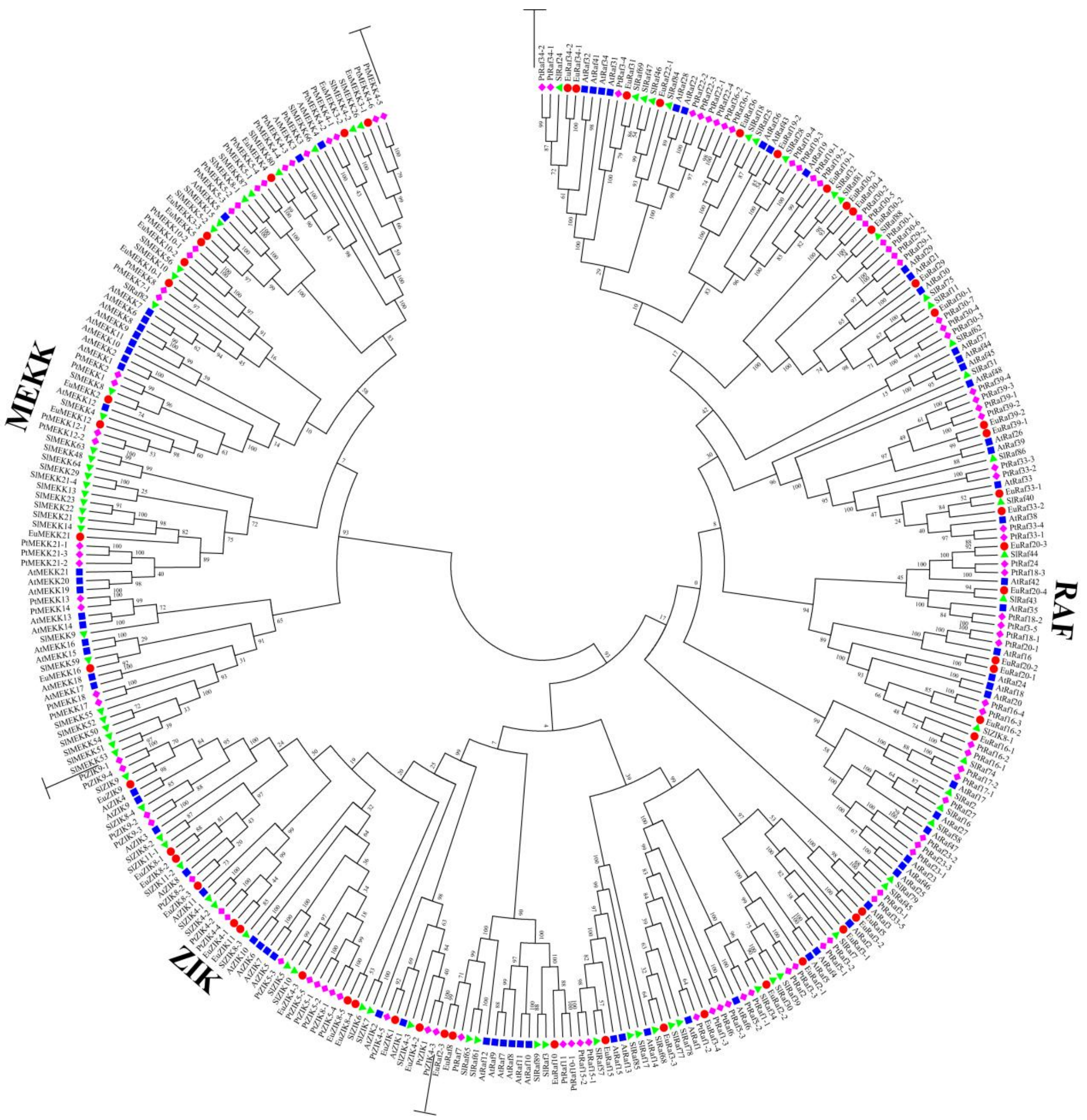

Supplementary Figure S3. The phytogenetic tree of MAPKKKs from *E. ulmoides*, *S. lycopesicum*, *A. thaliana* and *P. tremula*. The Neighbor-joing (NJ) tree was constructed using MEGA 7.0 software with 1000 bootstrap replicates. Signs of different shapes represent MAPK proteins from *E. ulmoides* (red round, Eu), *S. lycopersicum* (green tirangel, Sl), *A. thaliana* (blue square, At), *P. tremula* (violet diamond, Pt)

Supplementary files

Supplementary File S1. The coding sequences of EuMAPK cascades

>EUC04697-RA [mRNA]
ATGCACAATCTTACTCTGTTTTCGAGATTTGAACCCGGAATACAGTGGAA
AAACATTTACTATTTTGCCAAGGCTCAGCCTGTGTTCTTTATTACTTGGA
GTTCACATGGGCATCTGTTTCCAGTCCACTACAAGGGATTGTTTGATTCT
TCCTTCTCAGAGTCCAGCTGTTTCAGATGGATGCTTCTCTTTCAATCTCA
CAGAAGACAATTCAGGAATCGCTTTCCTTTTGCGCTTAAGATAGTACCAT
CCTTCCTTGTTACTGAGGATTTCCGGTTGACCTCTAGAATGCCATCTGAT
AATCTTAATCCACCCGATCAAGACCGGGAACCATTTGTCGAGGTTGATCC
ATCAGGGCGTTTTGGCAGGTACAATGACCTTCTCGGCTCTGGATCGGTTA
AGAAGGTATATAGGGCGTTCGATCAAGAAGAAGGTAGAGATGTGGCTTGG
AACCAGGTTCGGTTGAGGAATTTCAGCAGCGATCCATCTGTAGTGAACAG
GCTTTACTCAGAGATCAAGCTGCTGACTACGTTAAAGAACGATCACATCA
TTGTGCTCTACCATTGCTGGAAGGATAGAGAGCATAACACGTTGAATTTC
ATTACAGAGGCGTGTACCTCGGGCAATTTGAGGGATTATAGGAAGAAGCA
TCGTCATGTTTCGCTCAAGGCATTGAAAAAGTGGGGGAAACAGATTCTGA
AGGGGTTGGAGTATCTTCATACCCATGAGCCTTGCATCATCCATAGGGAT
CTGAATTGCAGCAACATTTTTATCAATGGAAACGTTGGGAAGGTTAAGAT
TGGAGATCTAGGGTTAGCAACGATAGTGGGGAAAAGTCACGCGGCGCATT
CCTTACTAGGCACACCCGAATACATGGCACCCGAACTCTACGAAGAGAAC
TACACAGAGCTAGTGGACATATACTCCTTCGGGATGTGCTTGCTCGAAAT
GGACACCAAAGAAATACCCTACAGCGAGTGCGATAGCATCGCCAAAATCT
ACAAGAAAGTGACTGGCGGGGTGAAGCCTCAGGCATTGGATAAAGTAAGT
GATCCTGAATTGAAGGCTTTTATTGAGAGGTGCATTGGTCAGCCTAGGGC
CAGGCCTTCCGCTTCTGATCTTCTCAACGACCCTTTTCTCTCTGATGTTG
CCGAATTGGACGAGGCAAGTCCTATTTCTTGA

>EUC04041-RA [mRNA]
ATGAAGAATCTTTTAAAGAAACTGCATGCCATGAACAATCAATCTGGAGA
CTCAGAGGGGTCTACTTCTTCCAAGAGCAACCGGTTGAGAGACGGTTCGT
CTCCCGACACCCCTCCACAATGTAGGTCCCCTAACAGTTCTGAGCATAAA
CCCTTTGCAGCTATTTCCGGATGGTTGAGTTCAGTTACCAAAAGGCATAG
TTCAAGTCCCCCATCATCTTCGAATGTGAAAAGAGATCATAGAATGGAAC
GGTTGGATTCCATGAGGAGTACCGGTGCTATTGGGCTCAATTCCGAGTCT
AGCAACTCAGGGGATCCTGAAGTTGAGGAGGAGTATCAAATTCAGTTAGC
TCTGGAATTGAGCACAAGGGAAGATCCTGAAGCAGTTCAGATTGAAGCTG
TTAAGCAGATCAGTTTGGGATCATGTCCGCCTGAGAACTCTCCAGCAGAA
ATTGTTGCATACCGATATTGGACTTACAATGCTCTTAGTTATGATGACAA
GATCCTGGATGGCTTTTATGATCTATATGGCATATGGATGGAGTCCAGTT
CATCAAAGATGCCTTCCCTGATTGATCTTCAAGAAATACCAGTGTCAGAC
AACTTAAGTTGGGAAGCAATTCTTGTTAATAAGGTGGATGATTCTAAGTT
GTTACAACTCGAAAGGAAGACCTTGGAGATGGCTGTAAACTCAAGGTCAG

GATCTATGAATTTTGTTAGTGGCAATTTGGTACGGAAGCTTGCTGCTCTA
GTTTCTGAACACATGGGAGGGCCTGTTACTGATCCAGACAACATGTTGAT
GGCATGGAAAAGTCTAAGTTACCGTTTGAAGGCAAACATTGGGAGCATGG
TTTTGCCACTTGGTTCTTTCTCGGTTGGGCTTGCCCGTCATCGCGCATTG
CTATTCAAGGTTTTGGCTGATAGTGTCGGCATCCCTTGCCAATTGGTGAA
GGGACAACAGTATACCGGTTCAGATGATGTAGCAGTGACCTTAGTAAAGA
TTGATGATGGAAGGGAATACATTGTTGATTTGATGGCGGATCCAGGGACA
CTCATTCCATCAGGCGCAGCAGGATTATATGTAGAATATGAAGTACCATT
ATTTTCCACTAGTCCATGGTCTAAGGATAGAGATTTGTCTCAGGTAGTTC
CTTCTACTAGTAGGGTTAGTGGATCATTTGAAGAACATTCTATGTTTGGG
ACAGTTGACAAAAGATCCATGTTTACAATTCGTAATGCTGTGGGAAACGA
ATCTGATGATAGAGGCAAAAATATGCATTCTGCAAATTTAGTGCAACAGG
GTGGAGCTGAGGAAGGATCCACAAGCTACTCAGATGATTTAAAAAATCCA
TGCAAAGTAAAGGAGAAAATTACTTGGGAAATTCCACGTAGGCCTAGTCT
TTGCATGCATGCAAGATCTCCGTCATGGACTGAAGGTGTAACTTCTCCAG
CTGTACGAAGAATGAAGGTGAAGGATGTTTCTCAATACATGATTGATGCA
AGCAAAGAAAATCCACACTTAGCTCAGAAACTTCATGACGTGTTACTTGA
AAGTGGCGTTGTAGCTCCTCCAAATTTGTTTACTGAAATCTACCCTGAAC
GGTTAAATGTCTTGCCTTCCAAGTTTCCTGCTGGAGTTAAAGGGGAGAAC
AAAGAGGGTGATGATATCCAAAATAAGTGTCGAAATGATCTTAACCGAGC
TAGCTTTTTGCCTCCTCTGCCTTATCATGGTGTGAAATCAAAAGAAAGTT
CTAGCGGACAACCAGAGCATCAGCTTGATTCAAGGGATGGAACTGGACTG
CATGTTTCATCACAATCTGGATCAAATGCAGTAAAATATGAAAAAAATGT
TCCTGTTGCTGCCGCTGCCGCTGCCGCTGCTGCTGTTGTGATGTCTTCAA
TGCTGGTTGCCGCAGCAAAAGTCAGCACTGCCTCCAACCTTGAACTCCCT
GTGGCAGCTGCAGCCACAGCCACCGCTGCAGCTGTGGTAGCAACCAGTGC
AGCTGTCAGCTTCCAGAATGAGAACATGGACTGTGCAAGAAGCGATGGAG
ATGAGTATGCTGCTGTTTGTGAGTCACGTGATAGTGTTGACGGGGGGAAT
GATGCTGTGGCCTCAAGCCAAGAGGGTGATAGAATATCTGATAGGTCAAC
AGGTAATGATAGTGCAAAGTCTGATGCAGCAATTGATGATGTAGCTGAGT
GTGAGATTCAATGGGAGGATGTCACCTTGGGTGAGCGTATCGGACTTGGA
TCATATGGTGAGGTATATCGTGGAGACTGGCATGGAACTGAAGTCGCTGT
GAAGAAGTTCCTTGACCAGGATTTAACCGGTGAATCCCTTGAAGAATTCA
TTAGCGAGGTACGCATCATGAAAAGAGTTAGGCACCCTAATGTTGTTCTT
TTCATGGGAGCTGTTACTCGACCTCCAAATCTTTCAATTGTTACTGAATT
TCTTCCTAGGGGAAGTTTGTATAGGTTAATCCATCGGCCCAACAATCAAT
TAGATGAACGCAGACGTTTGAAGATGGCTCTGGATGCTGCTCGAGGAATG
AATTATTTGCACAACTGTACGCCTGTAATTGTTCATCGTGACTTGAAGTC
ACCTAATCTTCTTGTTGATAAAAATTGGGTTGTCAAGGTTTGTGACTTTG
GATTATCCAGAATGAAGTTCAGCACATTTCTCTCGTCAAGGTCAACTGCA
GGGACGGCAGAGTGGATGGCTCCGGAAGTGCTAAGAAATGAACCATCAAA
TGAAAAGTGTGATGTTTATAGCTTCGGTGTCATACTATGGGAGCTATGTA
CTTTGCGACAACCATGGGGGGGAATGAACCCAATGCAAGTGGTTGGTGCC
GTTGGATTTCAGTATCGCCGTCTTGACATTCCTGACGATATGGATCCTGC

TATTGCAGATATCATTAGGAAATGTTGGCAAACAGATCCCAAATTACGTC
CTTCATTTGCCGAAATTATGGTTGCTCTGAAGCCATTGCAAAAGCCGCCT
GTTACAAGTGCACAAGTGCCCAGACGTAGTTCTTCAGTACGTGGTGGCCA
GGTGTAG
>EUC04221-RA [mRNA]
ATGTTTAAGGGACGATCGCTAAAAGGGTACAGTGATGAATTAGAGGATTA
TCATGGGTATGTGGAAACCGATCCGACTGGTCGATATGGTCGGTTTGATG
AAGTCCTTGGAAAAGGGGCTATGAAAACGGTGTATAAGGCATTAGATGAG
ACACTTGGGATGGAGGTGGCGTGGAGTCAGGTGAAGCTGACTGACTTCCT
CCATTCGCCGGAGGATTTAGAGCGGTTGTACTCCGAGGTACACCTCCTCA
GAACATTGAATCACGAGTCCATCATCCGATTCTACACCTCTTGGATCAAT
GTTGATCAACGGACATTCAATTTCATCACAGAAATGTTTACTTCCGGCAC
CCTCAGAGAATACAGGAAGAAATATAAGCAAGTCGATATTAGAGCAGTTA
AGATTTGGGGTCGCCAAATCCTGGCAGGTCTTGTTTATCTGCATGGCCAT
GATCCACCGGTTATACATAGAGACCTCAAGTGCGATAACATCTTTGTCAA
TGGCCATCTCGGACAAGTTAAACTTGGTGATCTAGGATTGGCAACGATTC
TCCGTGGTTCCCACAGAGCTCACAGTGTCATAGGTACGCCCGAGTTCATG
GCTCCAGAACTCTACGAAGAGAATTACAATGAACTTGTCGATGTCTACTC
ATTTGGCATGTGTATTCTTGAGGTGCTTACTGGTGAATATCCATACAGTG
AATGTGCCAATCCAGCTCAAATATACAAGAAAGTTACTTTGGGTAAGAAG
CCGAGGGCGTTCTACAAGGTACAAGACTTGGAAGCTCAAAGATTCATTGG
AAAATGCCTAGAGACTGCTTCTAAGAGATTATCAGCTAAAGATCTTTTGC
TCGATCCCTTTTTAGCGTTCGATGAAGATGATCCATTGCTTGACTTGAAG
ATCGGTCATCCAAAGCCCTTTTTAAACATCGATATTGGAGTTGAGGAGCT
GAGATTGAACGAGTACAGACCGAAAACCAACATGACTATCACGGGGAAGT
TGAATCCTGAAGATGATACCATCTTCCTTAAAGTGCAGATTGCTGATGAA
GAAGGTACGGTTAGAAATGTATATTTTCCATTTGACATCTTAAGTGATAA
TCCTCTAGATGTCGCCACTGAAATGGTGAAGGAGTTAGAGATAACTGACT
GGGAACCAACCGAAATTGCAAACATGATCGAGGCAGAGATATCGGGTTTG
GTTCCAAATTGGAGGAAATTAGATCGTTCTCAATCGACCCCACAACCATAT
AATGAGTCACGTAGAAGACGACGACGACGATATTGATCACATTCACCACC
ACCACCTTCACTCTCCCTCCACTTCTTCATCATCCCAAGTCTCGTTCTCG
GGTTTAATAGCTTCCCATGAAACAGATGACAGGACCCAAATCCGCGATTG
GCACCAAGAGGACTTGTTTGATGATAGCAGCTCTCATGGCTCGTCGCATT
CTGGAAATTATTCGAACATGTATTACCATTACAGAGACGAACATGAACAG
ATCACAAGTCCCAGGATACGAAATCACCACCACCACAACTGTACGAGGTT
TTGCCCAGGAGAAAATTCAAGAACAACAGGGCATTTAATTGCAAGAAATT
GCTATAATCAGTGCAAGGCATTGGTGCTCGAGTCGCAAGGTGAGAAATTG
TTGAAGGACAGGCGGGTATTAACAAGGAATAGATCATTGGTTGATATGAG
AAGCCAATTGCTCCACCGGTCACTGGTGGAGGAGATAAGTAAGAGGCGGT
TGTTCAAGACTGTTGGGGCGGTGGAGAATATTGGGTTTCAGGCACCTTGT
GAGGTTTCCGACTCTCATCAGCCGGTAGGTGGTGGTGTTTATTCAATGAG
GAGCAACAGAGATGGGAAGAAACAAGGGCATAAAGGAAGAAGAGTTTGA
>EUC20307-RA [mRNA]

ATGTTGGAGGGTCCGAAATTCACTGGAATTATAGACCTGAACAGCAGCCA
TGATGATATTTCGCAGAATTTTTACCATAAGCTCGGCGAGGGATCAAACA
TGTCAATCGACAGTTTCGGGAGCTTGCAGATGAGCAATGGTGGAGGGTCA
GTTGCCATGTCCGTCAGTAGCGTTGGATCGAATGATTCCCACACTCGTAT
GCTAAAACATCAGGGCCTGAAGCCTGGCAACAATTACTCTGTTGCCCATA
GTGTTAACCGGGGAAAAGTCTCCCAGGGACTGAGTAATGATGCATTGGCA
CAAGCTTTAATGGATCCTCGCTTCCCCACAGAGGGGCTTGAGGACTTCGA
CGAGTGGACGATTGATTTGAGGAAGCTTAACATGGGAGCAGCCTTTGCCC
AGGGTGCCTTTGGAAAGCTCTACAGAGGTACTTACAATGGTGAGGATGTT
GCCATTAAGCTTTTGGAAAAGCCAGAAAACGATAGAGAAAGGGCACAGTT
AATGGAGCAACAGTTTCAGCAGGAGGTCATGATGCTTGCGACGCTAAAGC
ACCCAAACATAGTCCGTTTTGTTGGTGGATGTCGTAAACCAATGGTATGG
TGTATTGTTACTGAATATGCCAAAGGAGGTTCAGTTCGGCAGTTTTTGAC
CAAGAGGCAGAACCGATCGGTGCCATTGAAATTGGCTGTGAAGCAAGCCT
TGGATACCGAAGGAATGACGCCAGAGACAGGCACTTACCGTTGGATGGCT
CCTGAAATGATCCAGCACAGGGCTTACACACACAAAGTAGACGTCTACAG
CTTTGGGATCGTTCTGTGGGAACTCATCACCGGAATGCTACCCTTCCAGA
ACATGACTGCTGTTCAGGCGGCTTTTGCGGTGGTCAACAAGGGGGTCCGG
CCAAATATTCCGACTGACTGTCTCCCCATTCTGGCAGAAATCATGTCACG
TTGCTGGGACCCGAACCCTGACGTCAGGCCTCCGTTTACTGATGTTGTCA
GAATGCTGGAGGCGGCAGAGATGGAGATCATGACGACAGTTAGGAAGGCT
CGTTTCAGGTGCTGCATAAGCCAACCGATGACTACTGACTAA
>EUC20242-RA [mRNA]
ATGGAGAGAACAACCTCAAACAAACCGAAATTCCCACTCTGTTTCAATCC
TTTCAGGATCCTGAAATCGAGGAACAAACCGCCGTCGATCCCTTCTTCTT
CAAGAACCCAGTTCAATTCCGACACGGAGAGCATGGAGAGGAAGAGATTT
GACAGCCTGGATTCATGGTCGATGATTCTGGAGTCCGAGAATGTCGAAAC
GTGGGAAACGTCGAAGGATGATCAGGAGGACTGGACGGCGGATCTGTCGC
AGCTGTTCATCGGAAATAAATTCGCCTCCGGCGCACATAGCCGGATTTAC
AGAGGGATATATAAGCAGAGGGCGGTGGCGGTGAAAATGGTGAGGATTCC
GACACACAAGGAGGAGACAAGGGCCATGCTCGAACAGCAGTTTAAGTCTG
AGGTTGCCTTGCTATCTCGCCTCTATCACCCCAATATAGTTCAGTTCATT
GCAGCATGTAAGAAGCCTCCAGTATACTGCATCATCACCGAATACATGTC
CCAAGGAACCCTAAGAATGTACCTGAACAAGAAGGAACCCTACTCTCTCT
CTACAGAAACCATTCTCCGACTTGCCCTCGACATCTCCCGCGGCATGGAG
TACCTTCACTCCCAAGGCGTCATCCACAGGGACCTTAAATCCAACAATCT
TCTCCTCAACGACGACATGCGCGTTAAGGTTGCCGATTTTGGGACCTCCT
GTCTGGAAACGCAGACCCAGGAATCGAAGGGGAACATGGGGACTTACCGG
TGGATGGCGCCGGAGATGGTGAAGGAGAAGCCGTATACCAGGAAAGTCGA
TGTTTATAGCTTTGGGATTGTGCTTTGGGAGTTGACGACGGCGTTGCTTC
CGTTTCAGGGGATGACTCCGGTGCAGGCGGCGTTCGCCGTCGCCGAGAAG
AATGAACGACCGCCACTTCCAGCCAGTTGCCAACCAGCACTGGCTCACCT
CATAAAGCGATGTTGGGCATTAAATCCCTCAAAAAGGCCTGATTTTAGCG
ACATCGTATCAGCTCTGGAGAAGTACGATGAGTGCGTGAAAGAGGGTTTG

CCTCTCACTCTACATTCAGGTCTCGTAAGTAAAAATGCAATCCTCGAACG
GTTAAAAGGATGTGTTTTGATGAATTCTTCAGTACTTGTACATGCTTAA
>EUC13910-RA [mRNA]
ATGCATCGAATACCTCAATTCTTCAATCACAGTAAAAGTTCCACAAGCTC
GAAGCACAGGCGAACCGCCAGTGCCAAACCCAAGCTCAAGCGCTCAAACG
CAGCGCGGCATATCGACTACGAGGCCCCCTCAACTTCGTCCTCTGATCAA
TCTTCATCCGCACTGCGAGCTCGATCACTGGGCATATTGCCTTCCAACGA
TCGCACCAGCTTCAGGGTCGAAGGAAATGAAGGAGAACTCGAAGTTATCT
GCCGGAGATTGGGGCTTTCGGGGCCGGAAGAGTTCGAGATCCCGGCTGCG
GCGTGGGAAGCGATGAAGTCCCGCTCTTCATCGGATGTTCTCCCGTTGTC
TGGGGAGCTTCGATTTGATAGCCTTAACGTAGAAGTACAAGTAGAAAATG
AAGGTCCTAGTGAAAACCTAGATGGGGTTTTAGATAGTTTGAGGGCTTGT
AACGTAAACGATCTTCAAATTGAAGTCGTTCCGTTGAATGACTGCAAACC
TGTTGGCGGAGATGGTGATGATGCTGATGAATTGGTGAATAGCTTAGAGG
ATAGTGTTAGGGTAAGTAGTTTGATTGTGAATAAGAATGAGCTTGTTGAA
ACTGCTGGATCTGGAATTAGGGTTCAGCATTCCAATGGTGGAAGAGGAAT
CAAAGGTGATAGGCCACCAACTCTTGAACCTCCGCCATCAATGTTGCTAC
CTGTAATAGATAATGGGTGTTCAACTTGGGATCTTTTGAGATCCTTTGCT
CCGGGCAGCGATATAATTTCAGGGTTTGAAATTCATGGAGGTATTGCTTC
TGATGATGAACAAGATGAGGGCAATGAAGAGAGTGGTAACAGGGATGGAG
AGCATAATGATGTGAGGGTAGGAGGGCCTTGTGTTCTTTATGAGTCGTGT
TCGTTTACAACTAGTAACGATGATGATTCATCTAGTACTACAACAGATCC
TGCGTCGAATAATATATCACCAAACGTAAGATCTAAGCGTAATATAACAG
ATTGGGAGAAAGGTGAGCTTCTTGGACGTGGGACGTTTGGATCTGTATAT
GAAGGAATTGACGATGGTGGATTCTTTTTTGCCGTCAAAGAAGTTTCTTT
GCTTGATCAAGGGGAGCTAGGGAGGCAATCTATTCATCAGCTTGAACAGG
AAATTGCTCTTCTAAGTCAGTTTGAACACGAGAACATAGTCCAATATTAC
GGCACTGATAAGGCTGAATCAAAACTCTTCATCTTCCTCGAGCTGGTGCC
CAAAGGTTCCCTTTTACGTCTCTATCAGAAATATACTCTTCGAGATTCTC
AAGTCTCTTCCTATACGAGACAAATTTTGCATGGTTTGAAGTATCTGCAT
GACCGCAAAGTGGTACACAGGGATATTAAATGTGCTAATATATTGGTGCA
TACTACCGGTTCAGTAAAACTTGCAGATTTTGGGTTGGCCAAAGCTACCA
AGTTGAATGACATAAAATCTTGCCAGGGAACTGCCTTCTGGATGGCTCCG
GAGGTGGTTAACAGGAAGAACCAAGGGTATGGCCTTGCAGCAGATATATG
GAGCCTCGGATGCACAGTGTTGGAGATGCTGACTCGTCAGCTTCCCTACT
CCCCTTTGGAATGTATGCAGGCGTTGTTTAAGATTGGGGGGGGTATTCCG
CCACCTGTTCCTGATTCCCTCTCCACAGATGCACGCGATTTCATATTACA
GTGCCTTCGAGTGAACCCAGCGGCTCGTCCCACTGCTGATCAACTCTTAG
GACATCCCTTCGTCAACAAACCGCTTTCCTCTTCTTTGGCCTTTGCTTCT
CCTCTTGATCCTCGCAGGAGAGTGTGA
>EUC14489-RA [mRNA]
ATGGATTTGGTGGAAGAAGTAGGAGAGAGCTCGTCGCCGGCTCGGAGCTT
CGGGAGTTTCAGTGGACACGACATAAGGAATGATGTGTACAATCGATTGG
TGGAGAGTGGGCATGAAGAGGCGGTCTCTGATCCCGGGTTTCGTGAACAG

TTGGATGCTCATTTCAATCGTTTGCCCGCTAGTTATGGCTTGGATGTAAA
CATGGATAGAGTGGAAGATGTTTTAGTGCATCAGAAGCTTCTTGCTTTGG
CGAAGGATCCTGATAATCGACCTGCTTTCCATGTCCGCTTTTTGGAGAAT
TTCTGGACTAGAGCAGATGGAGATGAAGACCAACAATGCTTTGATCTACC
TTCTACTTCAAGGCCATCATTTAATGTTGATAATGAGGAAGAGTTTCCAT
CCCCCGACAGGAACAGGAACTGTGAGAATGACTGTGAACCTTGCTCTAAG
CTTGAGGACCTTAATTTGGATGTTAGAAAGAGTTCCAATGGGTTGGAGAT
GGGAACTCTTGATGAAGACTTTCCCAGAAGGCAAGAACTTCCACATGTAC
CAATTCATGAAGTGATATTTTCCACCATTGACAAGCCCAAGCTCTTAAGT
CAGCTTTCTGCATTGCTTTCTGACATTGGACTCAATATCCGTGAAGCACA
TGTGTTCTCAACAACTGATGGCTACTCTTTGGATGTATTTGTGGTGGATG
GATGGCCTATTGAGGATACAAAGGGTTTGAGTGAAGCTGTGGAGAAAGCA
ATTGGTCGAAGCGAGGGATCGTGGTCTGGTTGTTCACATTCTCAATTAGC
CCGAGACAAAAAAATAGAATTGCAAGAACAATCTGGAGATTGGGAAATAG
ATAGAAGATTATTGAAGATGGGGGAAAGAATTGCATCAGGATCTTGTGGA
GACTTGTATCGAGGATTATACCTTGGTCAGGATGTTGCTGTTAAAGTTGT
TAGATCTGAGCATTTGAGTGACACTTTGGAAGTTGAGTTTGCTCAGGAGG
TAGCTATTCTAAGGGAGGTTCAACATAGAAACATTGTTCGGTTCATTGGT
GCATCAACAGTGTCTCCTGATCTCTGTATTGTCACAGAATACATGCCTGG
AGGGAGCTTGTATGACTATATACATAGAAATCATACTGTCTTGCACCTCC
CACAGGTGCTAAAGTTTGCACTTGATGTGTGCAAAGGGATGGAGTACTTG
CATCAGAACAATATAATCCATAGGGACCTGAAGACCGCAAATTTATTGAT
GGATGCTCAATCTGTTGTCAAGGTGGCAGATTTTGGTGTTGCTCGCTTTC
AGAATGAAGGGGGTGTGATGACTGCAGAGACCGGAACATATAGATGGATG
GCTCCAGAGGTTATAAATCATCAGCCCTATGATCATAAAGCCGACGTATT
TAGTTTTGCAATTGTTCTTTGGGAGCTTGTGACTGGCAAGGTTCCATATG
ATACCATGACTCCTCTACAAGCTGCCCTGGGAGTGCGGCAGGGCCTTCGA
CCGGAACCTCCAAAGGAGGCACACCCTAAGCTGGTGGACTTGATGCAGAG
ATGTTGGGAAGCTACTCCATCCGATCGACCATCTTTCTCTGACATAAGAA
TCGAACTTGAGCAACTCCTCATAGAAGTCCAGGATACCTCAAAGGCACAA
AATGGTTGCTGCTGA
>EUC13785-RA [mRNA]
ATGGGGAGTGGATCCACGTTCGTGGATGGGGTTCGTCGCTGGTTCCAACG
CCGCTCTTCATCTTCATCGACGGTCATCGCAAACAATGAAAATGTCCGCG
TAGCTGCGACCACCAACGATTCTGAATCGCAATCATCTAGTACACTAGAA
CACGTAAAAGATCAGCTTAATATTGTTCGAGACTTTGATATCTCTGCTCT
CAAGCTTGTTAAAGTCCCCAAACGCATTGGTTTCCCAATGGATCCTCACA
AAAAGAACACATTGGAGACAGAATTTTTCACAGAGTATGGAGAGGCAAGC
AGATACCAAATTCAAGAGGTTGTTGGGAAAGGTAGTTATGGTGTTGTGGG
TTCTGCAATAGATACCCACACTGGAGAAAGGGTTGCAATCAAGAAGATCC
ATGATGTCTTTGAGCATGTCTCTGATGCCACACGAATTCTTAGAGAAATA
AAGCTACTTCGACTACTTCGACATCCAGATATTGTAGAAATAAAACACAT
TATGCTCCCTCCCTCTCGTAGAGAGTTCAAAGATATTTATGTTGTGTTTG
AACTGATGGAATCTGACCTTCACCAAGTAATTAAGGCAAATGACGATCTT

ACTCCTGAGCATTATCAGTTTTTCCTGTACCAGCTTCTACGTGGCTTAAA
GTTTATACACACTGCTAATGTTTTTCATCGAGATTTGAAGCCTAAAAATA
TTCTTGCTAATGCTGATTGTAAGTTGAAGATATGTGATTTTGGCCTTGCC
CGAGTGTCATTTAATGATGCCCCATCAGCTATTTTCTGGACAGATTACGT
TGCAACTCGATGGTACCGTGCTCCCGAGCTTTGTGGTTCCTTTTTCTCCA
AATATACTCCTGCTATTGATATTTGGAGCATTGGGTGCATATTCGCAGAG
ATGCTTACAGGAAAACCACTGTTTCCTGGGAAGAATGTTGTTCACCAGTT
GGATCTTATGACTGATTTGCTAGGCACTCCTTCCCCTGAATCGACTGCAA
GGATTCGAAATGAAAAGGCAAGAAGATATCTCAGCAGCATGCGTAAGAAA
CATCCAGTTCCTTTTACTCAGAAATTCCCTTCTTCAGATCCTTTGGCTCT
TCGCTTACTTGAGCGCTTGCTTGCATTTGATCCTAAAGATCGACCCTCTG
CTGAAGAGGCATTAGCTGATCCTTATTTCATGGTTTGGCGAATGTAGAC
CGTGAGCCCTCCACTCAACCCATATCAAAACTTGAGTTTGAGTTTGAAAG
GAGGAAGTTGACAAAAGATGATGTTCGAGAGTTGATATATAGAGAGATTC
TAGAGTATCATCCCCAGATGCTTCAGGAGTATCTTCGTGGAGGAGAACAG
ACTAGTTTCATGTACCCAAGTGGTGTGGATCGGTTTAAGCGGCAGTTTGC
CCACCTGGAGGAGCATTTTGGTAAAGGTGAAAGAAGTACTCCACTGCAAA
GGCAGCATGCTTCCTTGCCTAGAGAGCGGGTTCCTGCACTCAAGGATGAA
AAAACTACCCAAAATGATGATTTAGAAAAGCGTTCTGCGGCTTCTGTTGC
TACAACTCTTCAGAGCCCTCCAAGGCAGCCTGCGAGATTGGAAAATGCAG
ATATCAATGCACAAAAGGCGAACAACAGTGCTCGTAGCCTGCTAAAGAGT
GCTAGTATTAGTGCTTCAAAATGTATAGGCGTGAAAGCAAGAAAAGATCA
AGAGGATGAAGCAATTCCAGAGCAACACGACGAGGTCGATGGCTTGTCTC
AAAAAGTTGCAGCGCTCAAAGCTTGA
>EUC16639-RA [mRNA]
ATGGATTTGGGAACAAACGAAGGGATTGAATTTGTTCAAAAATCTGGCGA
TCAAGAGGTGGGATTGGGCTCAAAATTGAAGGGCAGAGGATCTTTGAGTG
AGAAGAACATGTCTGGGAGGAGTTCCACAAGCCTAGGTAGCAAAGACATG
ATTTTTCGGGCGGACAAAATCGATCTGAAAAACTTGGATGTTCAGCTGGA
GAAGCACTTAAGTCGAGTTTGGTCGAAAAATATTGAGACCCAAAGGCCTA
AAGAAGTTTGGGAAATCAACTCTTCTAAATTGGATATTAGGTATTTCATT
GCTCAGGGGACCTATGGAACCTTGTACCGGGGAACCTACGATAATCAAGA
TGTCGCTGTGAAGCTTTTGGATTGGGGAGATGATGGCGTAGCTACAACTG
CTGAAACTGCCGCTCTACGAGCATCATTTCAGCAAGAAGTCGCCGTTTGG
CACAAGCTTGACCATCCAAATGTTACTAAATTCATTGGCGCTTCAATGGG
GACTTCTCATCTTAAGATTCCTCCGAAAAACCCATCTTCGGGTGGTGGGT
TTATCGATCTTCCTTCTCGAGCTTGTTGTGTGGTGGTTGAATATATTCCC
GGTGGAACTTTGAAGGGATTTTTGTACAAAAACCGGAAAAAGAAATTGGC
TTTTAAAATTGTGGTTAAACTCGCTTTGGATCTTGCTAGAGGATTGAGTT
ATTTGCATTCGAAGAAAATCGTACATCGTGATGTGAAAGCGGAGAATATG
TTGCTGGATTCAAATATAACTCTGAAAATTGCTGATTTCGGGGTAGCTCG
TGTTGAAGCTCAGAATCCAAAGGACATGACAGGTGAAACCGGAACACTTG
GATATATGGCACCTGAGGTTCTTGATGGGAAGCCTTATAACAGGAAATGC
GATGTCTACAGCTTCGGGATATGTCTATGGGAGATCTATTGCTGTGATCT

TCCCTACCTTAACCTCAGTTTCGCCGAAGTTTCTTCTGCAGTCGTTCGAC
AGAACTTGAGGCCTGAAATACCGAGATGTTGTCCGAATTCTTTTGCGAAT
ATAATGAAGAAATGTTGGGATTCAAACCCCCAAAAACGACCAGAGATGGA
AGAAGTGGTGAAACTTTTGGAAGTGATCGATACGAGCAAGGGAGGAGGGA
TGCTACCGGAAGATCAAGCTCGGGGTTGCTTCTGTTTTCATCCGTCTCGG
GGTCCTTAA
>EUC16268-RA [mRNA]
ATGGCTTTTGATCAGAACTCAATCCCCAAAGATCTACGCCCGTTGAATAT
TGTTCGATCAACGCCTGAAGAATCGCGCATTGCACCAGTTGCGACGTCAG
GGAGGGCAGTTGAGGGGTTTTATGCAAACCCACCCCGCGATGTCACGAGT
CCCCGGTCGGCACCTCTCTATTACAACCCTGCAATGGTGACTGATGCTGG
ACTTGCAGGTCTAGGTTATAACAATGCCCCTTCGAGTGTAGCTGGTTGGG
TGCCGCATGTGCCACCAGTTATTGTGGGCACTAGTGGTGTAAATCCAGCA
ATTGGGCACTGTTACAATCCAAATGTTGGGACTCGAGTTGGTGTTATTTC
TTCGGATCAGGGCAGTGAGGAGGGCGGTGAAGATTCAGTGTCTGGGAAGA
AGGTTAAGTTCTTGTGTAGTTATGGGGGGAAGATTTTACCTAGGCCCAGT
GATGGGGTTTTGAGATATGTTGGTGGGCAGACTAGGATTATTACCGTTAG
GAGAAATGTTAGCTTTAATGAATTAGTTCAGAAGATGACTGATACTTATG
GGCAAAATGTCATTATCAAGTACCAGTTACCAGAGGAGGAGCTTGATGCC
CTTGTGTCAGTTTCATGCCCAGATGATCTGGATAATATGATGGACGAATA
CGATAAGTTGGTCGAGAGGTCTACGGATGGGTCAGTTAAGTTAAGGGTGT
TTTTATTTTCGGCTTCGGACATTGATTCTTCTTTCGGAGATTTGCAAGCA
AGTGGCAAGAGATATTTCGAAGCAGTTAATGGAATTACGGAGGGTGGTGG
TGGTATTACAAGGAGGGATAGCATAACAAGTGCCGCTTCAACACAGAATT
CGGATTTGAGTGGGATTGAGCCTGTTGATAATGCAGGCTACAGTCATGAT
GATGCTACTGGGTTGCCATCAACTGTCTTGTTGTCACCTAGACGAAATTC
TACTACTTCTCAGGAAAGTGGTCAAAGGTTGGTGGTGGATCCTAGTCAGG
CAAATTATGCTGATGCTTCTGCTCTATTAGCTAATTCGGCGGTTAAGACT
GGTTATCCTCTATCATCACAGCCTGAGCCTGCTCATGTCATACAGCAGCA
GCTGGCCTTCGATTTGCAGCAGCCTGGAGTAAATTTTCCGGCACCTGCAT
CTTACTTGCAGGCTTATGTGGATCCTCGCCAACGCCAAGAAACAGTGAAT
CATTCAGATTATGCACAGCAGCCTCCTGGAGTTGGGTTCCCACCTCACCT
GTTGGGAACTGTTGGGCCTGTATTCACCCATCAACAGTTCTCTCCTGGCA
TGTCCCCTCAGCAGTTTATTCCTGCAGTGCACATGACAATGACTCCTTCT
ACTCATGTTATGATGAGACCGAATATGGTTCAACCATCGGTTCATCCCAA
CCAAGTCCAATTAGAGCGTTATCCTGAAGAAAACACGTTCAGCCAGAGGG
GTGTCCAGGTTCCTGGTAGCCAAAGTTACAGCACATATCAAGCTCAGGTA
CCGGCTCCGGTTATGGGGGGAGGTTATGGATGGAATCAGGTTCAGGTCAT
ACACCCAGAGCAAGTGACCTTCTCAGAGGGTTGGGTACCTCAGCCTCAGC
AACAGGTAATGGCTCCTGAAAAAATTGCTAGACTGGAGGACTGTCATATG
TGTCAGAAAGCATTGCCTCATGCACATTCTGATACATTGGTGCAGGGCCA
AAGAGAAAGCCCTGTCAGCACAATATCTGATTCAAACATTGTTTATCATA
GTCTCCGTTTGGATGACAAAGGGCGGCCCAATAAATGGGATAATACCGAT
CATCCTAATGTGACAGCTGCTCAGGGTGTGGTGGGGTTATCAAGTGGTTT

GCAATCTCCTTATGGTGCGTTTGTAGGTAATAGTCCTCAGGTTGCTAGTG
ATATCCCTTCCGTTGGCGGCATGCCTTTCCAAGCTTCAGACCACCTTGTT
TTTGAATCTCCAAAAGACTTTTCTGGTAAATTTCCCAAAGAAGATACTGT
GCAGTCTGCCGTTACATATGACCATCTGAGACAAATTGATGGGCGAATGG
GAAATCTCGGGATACACTCCCCTGAAGCTTTAGTGAATAACGAGCTGAGC
AAATCACAGGTTGATAATCCAAAATGGGAAAATGTAAACCATGCTGAGCC
AAATGAAATGCTTTATCAAGCAGTGGATTCTAATGAAGTGCCACAACCAC
CTCGCTTAGGTACTCCTGGTTTATATCCTCAGCCAAATCTTAGTGCCAAC
TATTTAGTCCCTGATGAGATTGCCTCCACTGGTCCTGCATTTGTTGCAGC
TGATCCAGCTCATACAGTCGAAAGAATTACACCTATTGGCCAGTGGAAGG
ATAACTGTTCCCGAAATCAGACAAATATCGCCGCCACTAATGTTGAAGTT
GTTTCATCAGATGGGAACACACCAACTTCTCTATCTTCATCTGATAGGGT
TGCAAACGTACAGGATAGCTCAAACTCACTTTTCAGCAACCAGGATCCAT
GGAGTTTGCATCATGATACACATTTCCCTCCCCCTAGACCAAACAAAATT
CTGACAAAGAAGGAAGACACTGTCAGTAGAAATCCTTTTGGTGACAGCCG
ATTCGGTGACAGTGCTGAATCGCTTGGCAACGTTGGGGGGTTGAGAACAG
ATTTGCAGCTTGATGATGGAGCTTATGAGCCATCTAGCTATTTGCACATG
GATATTCCTCTCGAGCATGGACGGTCCAACAAATCTGAAGGATCAGCGGA
GGAACTAATCAAGCAAGAGCTTCAGGCTGTTGCTGAAGGTGTTGCTGCTT
CTGTACTTCGGACTTCAGCACCTTCTAATCCTAACTCAGCTGAACATGGG
AGGAGTGAGTCTCATTTTAAAAGCAATGAATACAGTGAAGTTAAGGCCAG
TGATGGAGAATTGCCAGACAAAGAGAAATCTGAGGATATCAGTATCAAAA
CGCCACAAAAGTCAAATTTTGGGTTCCCCTTGTCAGATGGGATTGGTCGC
TTACAGATTATAAAGAACAGTGACCTTGAAGAGCTTCGAGAGTTAGGTTC
TGGTACCTTTGGTACAGTTTACCATGGGAAGTGGAGGGGTTCTGATGTTG
CAATCAAGCGAATCAACGACAGGTGTTTTGCTGGAAAACCTTCAGAACAG
GAACGCATGAGAGATGACTTCTGGAATGAGGCAATCAAGCTTGCTGATTT
GCATCATCCAAACGTGGTGGCTTTCTATGGCGTTGTACTTGATGGTCCTG
GTGGTTCAGTGGCGACAGTAACTGAATTCATGGTTAATGGTTCTCTGAGA
ACTGCTTTGCAGAAGAATGAGAGGCAACTTGATAAACAGAAGCGTATCTT
GATTGCCATGGATGTGGCATTTGGCATGGAGTACTTGCATGGAAAGAATA
TCGTTCACTTTGACCTGAAAAGTGACAACTTGCTTGTCAACCTTCGTGAT
CCACACCGCCCGATATGCAAGGTTGGTGATCTGGGTCTGTCGAAGGTGAA
ATGCCAGACTCTAATCTCCGGCGGCGTGCGCGGAACTCTTCCTTGGATGG
CACCTGAACTTTTGAATGGTAGCAGTAGCCTTGTCTCAGAGAAGGTTGAT
GTATTCTCATTCGGTATTGTACTGTGGGAACTTCTTACCGGAGAGGAACC
CTATGCAGACTTGCATTATGGGGCTATCATCGGCGGTATCGTGAGCAACA
CATTGCGGCCCGCGGTGCCGGAATCATGCGACCTGGAATGGAGATTGCTG
ATGGAGAAGTGCTGGTCGTCTGAGCCATCTGAAAGGCCAAACTTCACCGA
GATTGCAAACCATCTTAGGTCTATGGCAGCCAAGATTCCAATAAAGGGAC
AAAACCCAACAGCAAGTTCTTAA

>EUC16831-RA [mRNA]

ATGGAGAAATTCAACGCCGAACGGAGCTGGGTCAGGGGAAGCTGTATTGG
AAAAGGCTCCTTTGGGTCCGTCAGTCTCGCCGTCGACCTGTCGGATGGCG

AGGTTTTTGCCGTCAAGTCTGTCGATCGGAACTCGTCTTCGCCGGCGCAG
GTGGAGGCGATTGAGAACGAGATTCGAATTCTGCGGTCGATCTCGTCGCC
GTACGTGGTGGGGTATCTCGGCGACGGCTGGACCAGTGAGTTTCCCTCGG
CGTCGTACAGGAATATGTATTTGGAGTACTTGCCAGGTGGCACCGTTGCT
GACTTGGCGAAGCGGTCCGACGGTGACGTGGATGAGGAGACGGTGCGGCG
CTACACATGGTGCATTGTGTCGGCGCTCAGATACGTTCACTCAAAAGGCA
TTGTCCATTGCGACGTCAAAGGGAAGAACGTTTTGGTGGGACCCGCGAAC
GGCGCCGTCAAGCTCGCCGATTTCGGGTCGGCGACGGAATTCAATGATCG
GAAATATCCACGTGGAAGTCCGTTGTGGATGGCGCCGGAGGTGGTGCAGG
GAGGGTACCAGGGACCGGAGTCCGACGTCTGGTCCTTGGGTTGCACAGTC
ATCGAAATGATCACCGGGAAGCCGGCATGGGAAGACCGTGGCGCCGACAC
CCTGTTCGAAATCGGTTACTCCGGCAAATTGCCGGAGTTCCCAACCCATC
TACCGGAGCATGGTCGCGATTTCCTCGAGAAGTGCTTGCAGAGAGACCCC
TGTAAGAGGTGGAATTGCGATCAGCTACTCCGGCACCCATTTATTTCCTC
CTCTTCGCCGGATGATACGAATACTGATTCAACCCCTCGTTGCGTGATCG
ATTGGTTCAGTTCGGAATCCGGTGAAGACTCCGACGCCGACGCATCATGC
GAAACAGAGGATTTCAATGAAAACGCAAAACAGAGAATTTCGGAATTAGC
CACAAATTCAGGGGCAAATTGGGAATCAGATGGGTGGATGTTAGTGAGGA
ATTTGATCACGGAGAGTGAGCCAGCATCAGCACTTTACAGGCGAAACCGG
AACACAAAAATGGCGTCGTCTTCCGCCGGAGGAGGGACAGTGTCGGAGCC
AGTTGGAGGTGTCAGGATGGGTGGTGGACAGATAGGAAATCGACGGTGGA
GAGCAGGGAGAAAGGGATTTACAGCTGTAGCGAATTAA
>EUC10582-RA [mRNA]
ATGACACCCGAGACAGGCACTTACCGTTGGATGGCTCCGGAAATGATCCA
GCACAGGCCTTATACGCAGAAAGTCGATGTTTATAGCTTCGGAATTGTTC
TGTGGGAACTCATCACGGGAATGCTTCCTTTTCAGAACATGACTGCTGTT
CAGGCAGCATTTGCTGTTGTCAACAAAGGGGTCCGTCCTAACATACCCAA
TGACTGCCTTCCCATTCTCACTGAGATTATGACCCGTTGCTGGGATGCTA
ATCCCGATGTCAGACCACCTTTTGCTCATGTGGTTAGAATGCTTGAGGCT
GCTGAGACTGAGATATTGACTACTGTGAGAAAAGCCCGTTTCAGGTGCTG
CATAAGTCTACCAATGACTACTGATTAA
>EUC10624-RA [mRNA]
ATGGACAGCCCTACTGGAACTTTGACCACAACTGGATCAGGCCCGAGTTC
TAATGAGGAAAATCCACGTGTGAAGTTCTTGTGTAGCTTTTCGGGTAGTA
TATTGCCTCGACCACAAGATGGGAAGCTTAGATATGTTGGTGGTGAGACC
AGGATTCTTAGTGTCCCACGGGATATTAGTTATGAGGAGCTAATGGTGAA
GATGAGGGAGCTCTTTGATGGTGTTACTGTGTTGAAGTACCAGCAACCTG
ATGAGGATCTTGATGCTCTTGTATCTGTTGTTAATGATGATGATGTTACC
AACATGATGGAGGAGTATGAAAAGTTGGGAGCTGGTGACGGGTTCACTAG
ACTAAGGCTTTTTCTGTTTTCACATCCTGATCAAGATGGGTCCATGCATT
TTGTTGATGGGGATGAAAGAGATAATGAGAGGAGGTATGTGGACGCTTTG
AACAGCCTCAATGAATCTCCAAACTTCAGAAAGCAGCCGTTTAGTGATTC
CCTACTAATGGGTCCTATAGATGATATCAATGTGACTGAAGAGTACTTGA
ACCAGATAAGTCTTGAAGGAAGTGTCCATAACCAGAGGAATTTTGACATG

CACATGCCTCATATAAATTTGCGTCACCTCACAATTCCGCAAATGGGTTC
AGGTCAGCATCAGCTATCTGTCAGTCAAAGGTATAATGACATGGAAGCTC
CATGGAGTCCCATATACTATTCTCCAGGGCATCCTGGGCATCATGACCCA
AGACCAGCCGCCGAGTTTCCTGCTTCACCGTCTTCTGCTGGTCACCCCAC
ACCATTTGGGGAGCTTTCTGATAGAACATTTGATAGAATTCCTGAAGATT
ATAGTCTCCATCAAGTGAATAACCAATATGACTATCTTCCACAATTTTCA
GACAAAGTTGCATTCTTCTCAGGTGGACCTGTTTCTGCTGACAAGGCTGG
TTTTCCGGGTAACATACTTCAGGGCCCCAGCATCTTTGAAGGAAACAATG
TATGTGAGAATTGTAGAATGACATTTCAAAGAAATCAAGCTTATTCTGAT
TCCCCATGGAAGCATGGAGAACAGCCGCGTTTGGAGTCGTCTAATGTTGG
GAGTGTGTTTCACCAACCAGCAAATCCTTGTGCTGATTGTCATTCAAATA
GGGAAATTTATATGCTGCATACGGATGCGAGCATGCAACAGCCATTATAC
AGAGAGCAAAACAATTCCCGAACCCTTCATAATGAGGCTCAGGAAAGAGG
ATGGGTATTTCAGCAAAATTCAAATCTTCGGGTCGATAAACCAGTGCCAC
AATTATCTGGAACGGGAAGGTTGATGGATCACTATGTTGTCAATGGTAAT
GGTATCCCTGCTCCGGCAGCACATGGCAATCTATCGAATGGTCATCATGT
GCCCTCACATTATGCACATCATGAAGATGTACGTTACCCCTGTGTTGGAC
ATGAGTTGGGGAATCAAGTCTTTAATGACCAAAATGTGACTTCTGGATCC
CAAATTCACACTCATACACCTCCCCCTGAAGAGTGTGGGGTTCTATATGG
GAATGTGACATATGCTTATGGAGCGGATAATCTTTACCAATTCTCACGTG
CACGCACACCTCCAAATGCCATTTGGAGAAATGTTCATAATCCAGTGCAT
GGAGCTCAGTCTTATGAAACATCTAGCTCTCATTTACTTGCAAATGGTTC
AGTTGGTGCAGGATTCATAAGGTGTGCATTGGAGAGTAGTCCTAGGATAC
GACTAGGTGTGGAGAATCAGAACCCTTGGGTTGATTCTTCACAGTACATG
ATGGGTTCTGAGGGGGCTGCTGTACCGAAATATTCTCATGGAAATGCTCC
ACAATTGGTGCCAAACACATGCTGTCATGAAAATCAGCACTTGCCTACTC
CAGAACCACAACCATCTCCAGCTGAATTGCTAAACCATTTGACTCCTGTC
GAACCTATACTGAATTCTGATGTAGCATCAAAAGTGGTTGATTATAAAGT
TGTTCCTCTCACAACTTCTGGTCCAGAAGCAAGCAACGATACTGACCTTA
CTGAATCAGAAAGGTTGGTGCAGCAAAGTTCTCATGGAGGAGAAGAAACT
AATCATGAAGCTAACATGCCGAGTATATCTGCTCTAGTGAAAAGTGAGAA
TCCAGACGATATAGGCTCTCCCGAGGCTATCAATTCAAACTCTTCGAAGC
CTGTTGAGGAGAGAAATGGAGGTGTGAAGTCAATTGAAAATCATGCTCTT
GCTCCTCTTGACAGTGAGCAGCTCCTGGATCGGATTGTGACCGAAAATAA
AGGAGAACTAGAGGGTCTTGGAGTGAAAATTGACGATGATGAAAATTCTG
CTTCAGTTATCGAGCATAATGCTGCTGCAAAGGAAGCTCAGAATGATAGA
GAGTCAGTGGAGGCACATGAAGATTGTGAGTTCGGTTCTGATAATGACAA
TTCTAACGATATCAAGATTGAGCCGACAAAGGCAGAGGCAGAAGCTATAG
ATAGGGGTCTGCAGACGATAAAAAATGAGGACTTGGAGGAGATCAGAGAA
TTGGGTTCGGGAACCTATGGTGCTGTTTATCATGGGAAGTGGAAGGGATC
TGATGTGGCAATAAAGAGAATTAAAGCAAGCTGCTTTGCTGGTAGGCCAT
CCGAAAGAGAACGTCTGATTGCAGATTTCTGGAAGGAGGCTTTGATATTG
AGTTCGTTACACCATCCAAATGTTGTTTCTTTCTATGGAGTAGTTCGTGA
TGGCCCTGATGGATCTTTAGCAACAGTGACAGAGTTTATGATTAATGGTT

CTCTGAAACAGTTTCTGCAGAAAAAGGACAGAACAATTGATCGTCGGAAG
AGACTCATGATAGCAATGGATGCTGCATTTGGAATGGAGTATTTGCATGG
GAAGAACATCGTGCATTTTGATCTGAAATGTGAAAATCTGCTTGTGAACA
TGAGAGATCCTCACCGACCAGTTTGCAAGATCGGTGATCTTGGCTTATCA
AAGGTGAAGCAGCATACATTAGTGTCAGGAGGTGTACGGGGAACCTTACC
CTGGATGGCACCTGAACTTCTAAGTGGAAAAAGTAACATGGTAACAGAAA
AGATAGATGTATACTCATTTGGGATTGTTATGTGGGAGGTTTTAACAGGC
GATGAGCCTTACTCAGACATGCACTGTGCTTCTATAGTTGGTGGGATCGT
GAATAACACATTACGCCCAAAAATTCCCACGTGGTGTGACCCTGAATGGA
AGGCCCTGATGGAGAGTTGCTGGGCCTCTGATCCTGGGGAGAGACCTTCA
TTCTCAGAGATCTCTCAGAGGTTAAGAAATATGTCTGCATCATTGAATCC
CAAATAA
>EUC10368-RA [mRNA]
ATGAGGAAAGTTATTGTAATTGTAGTGGTGTACTTGTCCATCGTCATCCG
TAATACCAATCCAATAGAGGAAACAACTCCTCTCTGTCTCTCTGGTTTTT
CACGTTTCATTGTGGGATCACATTTCATGGCTTCTGCCTCGTACAATGAA
ATCCTGGGCAAAGGAGCTTCAAAAACAGTTTACAGAGCATTTGATGAGTA
CGAAGGAATTGAGGTTGCTTGGAACCAGGTGAAGCTATATGATTTTTTGC
AAAGCCCTGAAGATCTCGAGAGACTTTACTGTGAAATTCATTTGCTGAAG
ACATTAAAGCACAAGAACATTATGAAGTTCTACACCTCTTGGGTTGATAC
GGCCAATAGGAACATCAATTTCGTCACTGAGATGTTCACTTCCGGGACTC
TAAGACAGTATAGGCTAAAGCACAAAAGGGTTCACATGAGGGCAGTAAAG
CATTGGTGCAGGCAGATCTTAAGAGGGCTTCTCTATCTTCATAGCCATGA
TCCCCCTGTGATCCACAGAGATCTCAAGTGTGATAACATCTTTATCAATG
GGAACCAAGGGGAAGTCAAAATTGGCGATTTGGGCTTAGCTGCTATCCTC
CGGAAATCACATGCTGCTCATTGTGTTGGGACGCCGGAATTTATGGCTCC
AGAAGTTTATGAAGAGGAGTACAACGAATTAGTAGACATCTATTCTTTTG
GGATGTGTATTTTGGAAATGGTCACCTTTGAGTATCCATATAGTGAATGC
ACTCATCCCGCTCAAATCTACAAGAAAGTTATCTCTGGGAAAAGGCCCGA
AGCTTTATACAAAGTGAAAGATCCAGAAGTACGTCAATTTGTGGAGAAAT
GCTTGGCTACTGTGTCTTTGAGGCTTTCTGCTAGGGAGCTTCTTGATGAC
CCTTTTCTCCAACTTGATGAGTCTGAATCTGATTTGAGACTCATAGACAA
CGGGAGAGAACTTGATTCCATGGGCCCTTTATTAGGGCAGCCTTTTTTCG
AACTAGATTATGAAGGGAAGTCGTTTAGTAATAGCTCCTACAATGGATAC
TCTAACGGTTATGCGTTTGATGCTCAAAATGGATGGGGATATCACTCATC
GGAGATTGAGCAAAGTGGAATTGAACTTTTCGACTACAATGATGATGACA
ACGATCAGAACGAACACTTTGAAGGACTTGACATAACTATCAAGGGAAAG
AAAAGGGAAGATGATAGCATCTTTCTAAGACTCAGAATTTCAGATAAAGA
AGGTCGTATCAGGAACATCTACTTCCCATTTGATACTGAAAACGATACAG
CACTGAGCGTGGCAACTGAAATGGTAGCAGAGCTTGACATAATGGATCAA
GACGTTACTAAAATAGCTGATATGATTGATGGGGAAATCGCTTCATTGGT
ACCCGAATGGAAGCAAGGGCCTGGGATCGAAGAAACACCTTGCTTCACAG
ATCACGGTTTCTGTAACAATTGTGCTTCCAATCGCACCTCTAATGGCTCG
TTCTTGAATTTCCTCTCGAAACATCCAAGTGCCCAGAATTTGCAAATTTT

GCATTGTTCTGGAAATTGTTGTGCTGCTATGCATGGCCGGTTTGAAGAGA
TCACGTATCAAGTCGATAGCCCTCGCCAATATGTTCAAAATAAACCATTC
AAATCAAGCCAAACCAATGATTTTCATCAAACGGAGTGTTGGTATCAGCA
TGAAAGCCAAGAATGCAGTTCAACTGGCTCAAAAGAGAGCCCATCTGTTG
GAGATCACAAGAATTTGGATCGAAAATATTCAGCAAAGGATGAATTAGGG
ATAGGAATAGCAAAGGAAATATCAACTCATGAAATGTCGGATGAGAATCA
GCAGAATATGCGATGGCTGAAAGCCAAGTATCAGATGAAACTGAGGGAGC
TTACAGATCAACACTTGGGAGTTGTACCAAATTCTCCAAGGTCAGATTCG
GCAACACAAAGGGCTGAAAAATGCGAGGCCAGCAAAGAGTCCCAACCGGA
TGATGCATTCTGCACCAATTCATTGCTTCCAAACACACTTCACAGAACGT
CGTCCCTACCCGTTGATGCTGTTGATATATGA
>EUC11981-RA [mRNA]
ATGGAGAGAAATTTGAGAAAAGGAGTGACAGGTCAGCAGAGAAACCACGA
ACAAGTTCGATACAATTCTAGGCAAAATGAAAATGAAGGACTTGGTTCTG
CCAATCAAATACTTTTTCAGGATCCGCCTAGTTCTATTAATAGAGATATT
ACACGGCAGGATTTTTTAAAAATGGCTGGAGCTAGACCTGTTCTGAACTA
TTCCATTCAGACAGGCGAGGAATTCGCACTTGAGTTTATGCGAGAAAGAG
TAAACTCTCAGCAGCATTTCATTCCAAATGCTTGTACCGAGCCTGTTGGT
GCGACTTCATATGTAGAACTGAAGGGCATGATGGAAATACCTCATACGGT
TTCAGAGAGCGAATCGGATACCTCTATGATTCAATCTGTAGACAAAAATC
GCATCCAAGATCATGAGAGAAAGCGCACTTCTCTAAATGATGACAAGGCT
TACTATGAATCTGGGCAATCAGTGTCACGGACCTTGTCAAGAAATGAAAA
CAGGCAGGGAGTTCATAGTAAGGCCCCTTCAGGTGCTTCATCTAACTCAT
CAATGAAGTTGAAGTTTCTATGCAGTTTTGGTGGTAAAGTTTTGCCGCGT
CCTAGTGATGGAAAGCTTAGGTATGTTGGAGGTGAAACACGTATTATTCG
GATTAGCAAGGACATTTCCTTACAGGAGTTTAGGCAGAAAACCTTGACAA
TTTATAATCAAGCTCGTATGATCAAATATCAACTTCCTGGTGAGGATCTT
GATGCGTTGGTGTCTGTTTCTTGTGATGAGGACTTGCAAAACATGATGGT
GGAATGTGCAGTACTTGATGATGGTGGATCACATAAACTCAGGATGTTTC
TCTTTTCCGATAGTGATTTGGAGGATTCACAGCTTGCCATGGGGAGTGTT
GATGCTGATTCTGGGATTCAATACATGGTTGCTGTTAATGGAATGGACAT
TGGGGCAAGCAAGAACTCAATTGGTTTGGCTAGTGCATCAGCTTATAATT
TGGAGGAACTACTCGGTTTAAATGATGAGAGAGAGAGAGTGGTCGAGTTGTG
GCAAATTTAGCTGCAGTTGGAACTGAACCTTCATTAGTTGCTATGCCATC
AACAATTCAGTCTTCACGGACAGTGCTTCCAAGTGCATCTGTAGCTTCTG
AATTGAATCCACAGGCTTACCAAAGTCAGATGATGCATAATGGAGAAGCT
GAATGTCATTCATTATCCTCTGGGTACCCTATGGAAAGACTTGACCCTAA
AGAAAGGGGTACTGTTCTTTCAGGTGTCCCATCTCAATATGATGACGTCT
CTCATCAATCCAATTATGTATCAGCTGGAGAAAAAATGATTCTGCCACCT
CTTCATGGGCATATTACTCAGGAGGGAGGGGCAGTAGAAGATCAGTCATA
TAATGCCGTGAATGTTCAGGTTCCAGAAGTGTCACTAATGGAGGCAGAAC
TGAAAGATGATAGCTCATTCCTGAAGAAGAATGATTTTTCTAAAGATTTA
TCGCCAAAAAAGGAAGTCCTAGTAGATGAGGTAAAGTTGGAAAGCGATAG
CTCAGTTCCTGAATCAAATGAAGCTGAAATTGTTCCATTTCTGGAGGATG

AGTACATTGTTTCTTCTCATCTGTATGAGAGTTCTTTGAACTACGTATCT
AAGGATGAAAAGTTGATCGCGAGCACTGCTGCAGACACTGGTACTTCTGC
CTTGCCAATAAAAAATGTTGTAATTTTCCAGGAACCTATGCAGAATTTAT
TTCCATCAGCAGCTGAAAAGGAAGAAAAAGTGAATGAAAGTGATGAAAAT
GACCATTTTTATACATCTGAAAAAACATCTACTCTTGGAGATGATGACTC
TGTTGGTTACTCAATTGAATTTAGCCATAAAGAACCACCAACTCTTTCTC
ATCGCGGTTTCCATTCTGAACGAATTCCCAGGGAGCAGACAGGACTTGGC
CGCCTGTCCAAATCTGACGATTCATATGGTTCCCAGTTTTTAATAAGCCA
CACACACTCTGATGTTTCTAAACAAATAGCTGCCTCATTTCATGATGAAA
ATGTGTCTTCTCAGAGAGAGGGGTTTATCTCTTCTGCAGAACCACTGTGT
GCAAATGTGCAAACTGCTGAAAATGGGCTAGGACAATATCTGAACTCTGA
AGAGGTAGTGAATAATCGCAAAGAGGACTCTGACAACAGTAAGGAAGCCT
CTGGGTTCAATCTTTGTACTACAAGCCAAGGAATTTCTGGCAACTTTAAT
GGAGATTCTACTTCTAAGTCACCTGAGCCTCAATCAGATGAAAATTCTGC
TAGAAAGAGCAATGCAAGCAGTAAGGGGCTGTTGGCCAGGTCAGAGCGCT
CTATTGCACCACTTCCTCCTGGGGTGTCCTCTGTTGGTGCTGGAACTACT
GAACAGGGAGACATCATTATTGATGTCAACGACCGTTTTCCTCGTGATTT
CTTCTCTGATATATTTTCCAAGGCTGGAACTATTGATGGTTCAGCAGGAA
TTGGTCTACCGGACGGTGACAGAACTGGTTTGAGCTTTAAACCTAAGCGT
TGGTCATTTTTCCAGAAGGTGGCCGAAGATGATTTTGGTAGAAAAGATGT
TTCACTCATGGATCAGGACCATCTTGGTTTCTCATCCCTACTTACAAATA
TTGTAGAAGGGGCTCCTCTTGATTATAATTTTCCACCTTTTCAAGCTGGT
GGTGTTGCACAATTGGGCTCCCATATTAATTTTGATACTGCTGTGCAGCA
GCGGTCACATGATATTATGGGATCCAACACTTTGGATCTACATTCAGATT
ACAATCCTTCCGAGGCCACTGACAATCAAATTGTGCAGTCTAGTGGCTTG
TTGAGGAAGGCAGTGGTTGGTGAATCTTATTTTGAGGATGGGAAGCTGGA
ATCCAATACTACTGGTCAACCTCTCTTCGATCCTTCTCTGGGAGTTTTTG
ATCCTAGTGCCCTGCAGATAATCAAGAATGAAGATCTTGAAGAGTTGAGG
GAATTAGGCTCTGGAACATTTGGAACCGTCTATCATGGAAAATGGAGAGG
AACAGATGTTGCCATTAAGCGAATAAAGAAGAGCTGCTTCACGAGTCGTT
CATCAGAACAGGAGAGACTGACAGTCGAGTTCTGGCGTGAAGCTGAAATT
CTCTCAAAGCTTCATCATCCCAATGTGGTGGCATTTTATGGTGTGGTGCA
GGATGGACCGGGGGGAACACTAGCTACAGTGGCAGAATACATGGTCAATG
GTTCTCTTAGACATGTTCTACTTTGCAAGGACAGGCATCTTGATCGTCGT
AAGCGGCTGATAATTGCTATGGATGCGGCATTTGGAATGGAATACTTACA
TTCCAGGAACATTGTGCATTTCGATTTGAAATGCGACAACTTGCTTGTGA
ACCTTAAAGACCCTTTACGACCTATTTGCAAGGTGGGTGATTTTGGGTTG
TCGAAAATCAAAAGAAATACTTTGGTCACTGGTGGTGTTAGGGGAACCCT
TCCATGGATGGCTCCAGAGCTGCTAAATGGTGGCAGCAGTAAGGTTTCTG
AGAAGGTTGATGTGTTTTCGTTTGGGATTGTGTTATGGGAAATTCTTACA
GGCGAAGAGCCTTATGCCAACATGCATTATGGGGCGATAATAGGAGGTAT
AGTGAGTAACACACTGAGGCCACATGTGCCCAACTACTGTGATTCGGATT
GGAGATCACTTATGGAGCAGTGTTGGGCTCCAGATCCTGAAGTACGCCCA
TCATTCACTGAAATTGCTATACGCTTACGTGTGATGTCCACAGCAAAACC

TCAGGGTTATCCTACCCAAAACAAGGCGTCCAAGTGA

>EUC05776-RA [mRNA]

ATGTCTTGGTGGCAGAGCGGCTCCTCTTCCTCTTCTTCCAAATCATCTCC

CGAAGATGGTCTCCGCAACATGTTTAGACTTTTCTCCGGCCGGAGACACA

GCCACAGCTCCCAGCCACGGCAGTTCAGTCGCGGCAGGAAACTTCGGTAC

TTCGCAGAAGACGATGTCCGCCGTCCACCGCCTCCGGTAGACCCTCCCGA

GCTGTGGCGATCAGCGACCGCTCTTGAAATTTCGCCGAATCGCTCGTCGT

CAGGTGCCGCACCGGCTGCTGCTCCGCTGCCCCTCCCTTTGCCGGAATTA

GGGGCGTTGCTCCGCCGGGATTCAAGCTTGGTTTCCAATTCGAACTCGGC

GGATTGTCTTCTGATGTCACTGAAGGAGGCCGGCGGCAGAGAAGGCGTAG

AGGATAGAGGAAAATCGGAACGTTTGAATGGCGACGGAGTCATGATTGGG

AGTAAATTTTCTGGCCACGATGCTCGAAAGAGTACTGACCATTCGGAGCC

TCAATCATCAAAGAAATCGAATGGCAAGAAATCAAATAAAAACAACTGTC

AGATCAACATTCCGGTCAGTGCTCCGACCAGTCCTTACTCTAGTCCTCTA

CGCAGCCCACACGGAAACAACGGTGACCTTTATCTACCTCCTTACATGAT

TCCACCCATATTTCAAGTTTGGTCTGCACCGGAGTTGCCCTCTTCGGACA

TGAACCAAGGACTAGGTTTCCCTCATCAAACGTTTCCTGAAAAAAACTGCG

TTTAGCGTCGACAGCTCGCCGCTCCGTAGTCCAAGAGTAAGCCCTCACCA

GACCGCCAGAAGTCCCAGTGGGCCCATCTCGCCATTGCATAGCAAATCGA

CAGTTGAGATCGCATCATCACGATGGGAGGGTAATCCTCAGGCCGATGTC

CACCCCTTGCCACGTCCTCCCGGAGGTATGATCCCTTCACAGACAACTGT

TCCCCAATTTACGATAAAACCGGAGGTGATGCCATTGAGGAGACAATGGA

AGAAAGGAAAGCTTATTGGACGAGGAACATTTGGGAGTGTTTATGTTGCA

TCAAATCGAGAAACTGGAGCTTTATGTGCGATGAAAGAAGTGGAGCTATT

GCCGGAAGACACTAAATATTCAGAGAACATAAAACAGTTAGAGCAGGAAA

TCAAAGTCCTCAGTCAACTAAAGCATCCAAACATCGTTCAATATTATGGC

AGTGAAATAGTTGGTGATCGATTTTACATATATCTCGAGTACGTTCATCC

GGGCTCAATCAACAAATTTATACATGATCATTGTGGAGCAATTACGGAAT

CTGTTGTACGAAATTTTACTCGCCATATTCTCTCCGGGTTGGCTTACTTG

CATAGTACGAAAACTATACACAGGGATATTAAAGGGGCTAACTTGCTTGT

GGATGCATATGGGGTTGTCAAGCTTGCTGACTTTGGGATGGCTAAGCATC

TTACTGGACAAGCGGCTGATCTTTCTATGAAGGGAAGTCCATACTGGATG

GCTCCCGAGCTGTTGCAGTCAGTAATGCAGAAAGATGCTAGGGCTGATCA

TGCTTTAGCTGTCGATATTTGGAGTTTAGGTTGTACTATAATTGAAATGC

TGAATGGGAAACCTCCTTGGAGTGAATACGAAGGCGCTGCAGCCATGTTT

AAGGTGATGAGAGAGACACCACCTATTCCTGAAACATTGTCTGCGGAAGG

CAAGGATTTCTTACATTGTTGCTTCCTTCGAGACCCGTCTGAACGCCCAA

CAGCCGCCGCCTTGTTAGAACATAGATTCTTGAGAAATTCTCAGCAGCTG

GATGTTCCAATTTGCTCCCAGTTCGTTCATGGCATGAAATTAACGGATAA

AGCACAAGCTCCGGGAGAGGTTGCCAGTCATAAAGTTAATCAGTTGCCGG

AGGGAAAATTGGTAAAAAAAACATTGCGGGAATTAAAAGTGGCGCCACAC

CGTTCGCCGCGTTCGATTCTGGAGGCAATTTCTAGTTTGTCTCCTCCGAG

GTTGGGCCGAACATCGGACCCTGTCCTTACTGCCGGTCCTAAAAATAACC

CTTAA

>EUC05265-RA [mRNA]
ATGGAGAAGGAAGAATCCGGTACAGCGATTCAAATAAAAGGCGTTCCGTC
TCACGGCGGGAAGTATATTCAGTACAATGTCCTGGGGAATCTCTTCGAGG
TATCTTCTAAGTACGCTCCTCCTATTCAGCCCGTCGGCCGCGGGGCTTAT
GGCATCGTCTGCTGTGCTACAAATTCGGAAACAAAGGAAGAAGTTGCAAT
AAAGAAAATTGGAAATGCTTTTGACAACAGAATTGATGCTAAAAGGACCC
TGCGTGAGATCAAACTCCTTTGCCATATGGACCATGATAATATTATCAAA
ATTAAGGACATTATACGGCCACCAGATAGGGAGAATTTCAATGATGTTTA
TATTGTATATGAGCTAATGGATACTGATTTACATCAGATAATACGCTCTT
CCCAAGCACTCACAGAGGATCATTGCCAGTACTTTCTGTATCAGTTACTG
CGGGGACTGAAGTACGTGCACTCTGCTAATGTTTTGCACCGAGATCTAAA
ACCAAGCAATTTGCTTCTCAATGCAAACTGTGACCTCAAGATTTGTGACT
TTGGGCTTGCAAGAACCACCTCAGAGACTGATTTCATGACAGAATATGTG
GTGACCCGTTGGTATCGAGCTCCTGAGTTACTACTCAACTGTTCAGAGTA
CACTGCAGCGATTGATATTTGGTCTGTTGGTTGCATACTAATGGAGATTT
TAAAACGGGAACCCCTGTTCCCCGGTAAAGACTATGTTCAACAGCTTGGA
CTCATAACTGAGCTATTAGGTTCGCCAGAAGATGCAGATCTTGGATTTCT
AAGGAGTGATAATGCTCTACGGTATGTTAAGCAGCTTCCTCATTTTCCGA
AGCAACCTTTTTCCAAGAAGTTCCCGGAGGTGTCCCCAGAAGCCATTGAT
CTTGCAGAGAAAATGCTTGTTTTTGATCCATCCAAACGCATCACCATTGA
GGAAGCACTGAATCATCCATTTTTATCAAGCCTCCATGAGATCAATGAGG
AGCCCGTTTGCCCATCTCCTTTTTTCTTCAATTTCGAGCAGTCTTCTTTG
AGTGAAGACGACATCAAGGAGCTCATATGGAGGGAGTCTATAAACTTCAA
CCCAGACAAAATGTTGGAGTAA
>EUC05347-RA [mRNA]
ATGGCATTTGGAGCTGAAAATCAGAATAATCCACCATTCAGACAAACAGC
TCCACATACTTGTAAGACCATGCCCGGTGAAAGACCTCGTACTTCACGCC
CGCGTTTCAACAACGGTACATCCAACAACCTCAACCGAGCGGACGAATCG
TATCGCGACATCTCAGTTCAGACCGGCGAAGAATTCTCTGCCGATTTCCT
CCGAGAGCGCGTCTCGCACAGAGCGGCATCAGCAGACATGGACGAGCAGA
TGAAGAACGGGGTTGGTTTCATCGTTGGTCAGAATCATGAGCTTGTTTAC
GAAGATCTCAGCCGTATTCTCGGTATCCGAAGGCGAGATTCCGATAACGC
CCCCGAAGTTTCCGAATTCTATCAGGTTCAAAAAAATTATGATTTTGCCC
CTGAAATCCGAAACCACAGAGATTACAGGTTCAATAATGATATCCAACCG
AGAAAAAAATCTTTCGAGATCGTGTTTAGTCCGACTGGTCCGCCCCTTCA
TTCGTCGGATTCTCCGAGTTCGCAACAGCCACATTACGGTTTGGGCGTTT
CAGACGGTTCTTCATCCGGAAAGGTCAAGTGTTTATGCAGTTTTGGAGGC
AAGATAATACCAAGGCCGTCCGACGGGAAGCTTCGATACATAGGTGGAGA
GACGAGGATCATGTCAATCAGGAGAAACGCGACGTTTTCTGAACTCATGA
AGAAGACTTGCGCGATTTGCAACCAACTCCATACGATCAGGTACCAGCTC
CCCGGCGAAGATCTAGATGCGCTTATCACTGTATCGTCAGACAACGATCT
TCACCTCATGATTGAAGAGTTTCGTGACTTGGACAAATTTTCACAGAGGC
TAAGAATATTTCTCATCTCTCTGAACGAATCTGATGGCGGCTCATCTTCT
TCTTTCGAAGGCAAAAATTTGCAGCCAAACGAGGGCGATTATCACTACAT

GAACGCTCCTAATGGTATGAGCTCAAGCAGGGAGAGCCTGCCAAGTCCAA
GCCAATTGAAAATTCACGAATTGGATAGTCCCGATAATCCAAATCTACAA
ATGTACGACGATCACCCGTTTGGAGGTAATTTTATGTACACCAACAATCA
ACGCGAATACAAAAATCCATATTTTGACACCACGGGATATTACTATAGTA
ACCCTGTCAATAATGTCCCCGCGATGAATTATCAAAATCAGAACAATTTT
TTTGCGGAACTGGAATTGGGTCATGATTTGTATCCTCAAAATCATGTCCA
GGGTAGCGAGTTTCCTCCTCTACCTCTTTATGGCGATTTTGCTAATGACA
GGCCCGTGCATAACTGTTTCACGTATTTTTCGACGAAATCGACATTTTCC
GAGAAGAAAAACGATCATTTTTCTGGATCGGACATTCCAATTGAGGATTG
TCATGAACAGTCAAGTGATGCACAGTTGCAGCTTGACACAACAATGTCGG
GTTTGTCGCAAGATCTGGTAATGCAAAAGCTAGAGACGGGTGATGAGAAA
TATCAGAAGGGGGGGGAAAGTGTAAATATGCTCAAGTTCACTATGAACAA
CGACCAAAGCATGGAATACATCGACTGGGGGAAAACTACGATAAATTGGA
TGGGGAAGAAAGAATCTAACGATCGCGAAGGAGATGAAGATATTAAGGCC
AATGCGAATATTCGATCTCGTGAAAATTCGAGATCCGGTGTTCGAATCGT
TGAGCAAGAACGTGAAGCTCGAAAATCTTCTTTAAACAATGCGGCGGATC
AATCTCCTGGAATGGTTGGGTTTGAACCATTCTTCACGAATCAGGGTAAT
GCGATCCCGTTTGCTGAATACGAATCGTGTTTGAGCTTGGATTGGCATGA
AAACGACCGTCACATGATTGATTTGTCTCTTCCTTCTTCGATCACTGCTA
TCTTGGGTAGAAAAATTTCTAACAAGGTTGAAACCGAGGTAAAACCGACC
TCGAAAGGCTCGAATTTTGTCGAAATTCGACAATCGGGTGATCGAAAACA
CGTCAGCTTGCAAAAGTCGACATTTAATGTTGAAGATGTGACTCATATCG
TGCCCCATGATGTCGCATCGTCCTCGAAAATCGTTCCTTACGTACACGAT
GGAACCAGTGACGGAAGTACATCGCCTAAAGGATCGGAAACGGAATCAGA
AACCGGAAGCAGTATATCCAAAGAATTTAACGTGGAGATTGCTGAAGTCG
ATGGAAACGGTAAGGAAGAGGCTGCCACTCCCGAAGTTTGTATAGCTGAA
ATGGAAGCAGATTTTAATAACTTGCAGATCATTAAAAACGTGGATCTTGA
AGAACAACAGGAGTTGGGATCTGGCACGTATGGAACTGTTTATCACGGGA
AGTGGCGAGGAACGGATGTCGCTATCAAGAGAATAAGAAAGAGTTGTTTT
GCAGGGGGGCCGGCGGAGAAGGAGCGTTTGATCAAAGATTTCTGGAGAGA
GGCGAATATCTTGTCGAAACTTCACCACCCAAACGTTTTAGCCTTTTATG
GGGTAGTTCCTGATGGACCTGGCGGAACATTGGCTACTGTAACCGAATAC
ATGGTTAATGGCTCATTACGCCATGTCCTCTTAAGCAAAGAACATACACT
TGACAGGAGAAAGAAGCTTTTGATTGCGTTAGACGCTGCTTTTGGCATGG
AATACTTGCATATGAAAAACATCGTACATTTTGATTTGAAATGCGACAAC
TTGCTCGTAAACCTACGAGATCCACAGAGACCAATATGCAAGGTTGGTGA
TTTAGGGTTGTCGAGGATAAAGCATAACACGCTCATTTCGGGTGGCGTAA
GAGGAACTCTTCCATATATGGCACCGGAGTTGTTGACTGGTAACAGTGGT
CGGGTTTCTGAGAAGGTTGATGTATTCTCCTTCGGCATTGCACTGTGGGA
GATCTTGACCGGCGAGGAGCCATATGCAAATATGCATTGTGGTGCCATTA
TAGGTGGGATTTTGAGTAACAAACTTAGGCCGACTATACCTGAAAATTGC
AATCCAGATTGGAGAAAATTGATGGAAGAATGCTGGTCAGGTAACCCTAC
AGAAAGACCTTCATTCACAGAGATAACAAACCGGCTCAGGGCTATGACAA
CGTCGCTTCAACCTAAACGACATAATTTCTCAAAGAGATGA

>EUC05370-RA [mRNA]
ATGTCTGCTTGGTGGAAGAGAAAATCTACCAAGAACAAAGAACATCAACA
GGTTCAGCAAAAGCCACAAAGCGATTACCAGAATACAAGCAGAAAAGCTT
CAATCAAGGATGATAAGAAGAGAGGCCAAGACAAACCCAAGAGCTTTGAC
GAGGTCTCCACGCTTGTCTTCTCGCATAATTCGCCGCGCAACAGCAAGGA
ATTTGGTGGTTTAGTTGGTGGAAGTAGCGGCGGCTCATCGGGATTTTCGG
GATTCGATTCGTCGGAGAGAGGGCACCCTCTGCCGCGACCGTCGGTGTCT
TCGACGCAGTCGTTTGGGGTTGATCATCTTCAAGGTGTCGGATCGGGATC
TGGGTCGGTCTCGGTTTCCAGTGTGAGCTCATCTGGATCCTCCGATGATC
AAGCTCATCTTGATCAAGTTCATGGCCAATTGGGCTTTGTCTCAAGAGTA
CATGGTGGCGATACAAAGTTTTATACAGTACCAAGAAGCCCAGGTCCAGC
TTTGAGAGGTGCAACAGCTAATACATCGCCTCTTCATCCACGATCTTCTC
TAGACTCCCCAACGACAAGGGTGGAAGATGGGAAGATCGAATGCCATCGG
CTGCCCCTTCCACCGGGTTCCCCTACTAGTCCTTCTGCCTTGCCCATGCC
TAGAGCTTGTGGAGTCACTGACAACTCCTTGTCAAAGTGGAAGAAAGGAA
GGCTTCTGGGAAGGGGCACGTTTGGGCATGTTTACGTTGGATTTAACAGT
GAGACTGGGCAAATGTGTGCAATAAAAGAAGTTAAGGTTGTTGCAGATGA
TCAGACATCAAAAGAAAGTCTCAAGCAATTGAATCAGGAAATCAATTTGC
TTAGTCAGCTTTCTCATCCGAACATTGTTCAGTACTATGGCAGTGAACTG
AGTGAAGAAACACTGTCTGTTTTCTTGGAATATGTTTCTGGGGGTTCAAT
CCACAAACTACTTCAAGAATATGGCTCTTTTAGAGAGCCCGTCATTCAAA
ATTACACTAGACAGATCCTCTCTGGTCTGGCCTACTTGCATGGAAGAAAT
ACATTGCACAGGGATATTAAAGGAGCAAACATATTAGTAGATCCCAATGG
TGAAATCAAGCTTGCGGATTTTGGCATGGCAAAACATATAACGTCTTGTT
CTTCAATGCTTTCTTTCAAAGGAAGTCCTTACTGGATGGCGCCTGAGGTC
ATTATGAATACAAACGGCTCTGGCCTTGCAGTGGATATATGGAGCTTAGG
ATGTACAATTCTTGAAATGGCAACATCGAAACCCCCTTGGAGTCAATTTG
AGGGGGTGGCTGCTATATTTAAAATTGGAAACAGCAAAGATATCCCTGAA
ATTCCCGATCGCCTGTCCGATGAGGCGAAGAGTTTCATAAGGTTATGCTT
GCAGCGGGATCCATCTGCACGGCCAACAGCATTAAAACTACTGGATCATC
CTTTTGTCAGAGAACAATCTACCGCAAGAACTGCTAATGTCAATTTAACC
AGGGAAGCCTTCCCTTCCTCCTTTGATGGGAGCCGCACACCGACGGCTCT
AGAGATGCATTCCAACAGAAATAGTATTACTTCCTTTGATGGAGATTATT
TTGCAAAACCCATTGTGGCAGTCCCCAGAGCTTTGATCAGCCAGAGGGAA
AGTGCAAGAACTATAACATCGTTACCCGTATCTCCATGTTCAAGCCCATT
ACGACAATACGGACCAGCGCACAAGAGCTGTTTTCTGTCACCTCCACATC
CATCATACCGTTTGGTAGCACAAAATGGTTATAATTTAACAGACTACTCG
GTTTTGACAGCGAGGCACAGCAGAAAGACTACTCTCGACCCGTGGCTGGA
AATCCCCCAGTTTAATCCCCAGAATCCTGGCAGATCCTCCAGACCTAGAC
CCATTTTATAG
>EUC05489-RA [mRNA]
ATGCAAGAAATTTTCGGATCAGTCAGGCGATCACTAGTTTTCCGTACACC
GGAAGGTGGTCTTGCCGACGAAGCCACCGCCGGCGGAACCCTAGTCAACA
AGATCAACTCATGCATCCGTAAGTCTAAAGTTTTTTGCAAACCCTCGCCG

CCGTCTCTCCCTCCTTCTCGAACGATCATTAAAGACAACAACGCACCGCC
ACCTACGATTCGGTGGCGCAAAGGGGAACTGATCGGCTGCGGGGCCTTCG
GCCAAGTCTACATGGGCATGAATCTTGACTCTGGAGAGCTTCTTGCGGTC
AAACAGGTTTTGATTGCCGCAAACGGTTCTTCGAAAGAGAAGGCACAGTC
TCACATTCGGGAGCTTGAGGAAGAAGTTAAGCTTCTTCAGAATCTATCTC
ATCCGAATATTGTCAGGTATTTGGGTACTGTGAGAGAGGCGGAAACTTTG
AATATTCTTTTGGAGTTTGTTCCTGGAGGATCAATATCATCTCTTCTAGG
GAAATTTGGATCTTTCCCCGAGGCTGTTATAAGAATGTATACAAAGCAAT
TGCTATTGGGGTTGGATTATCTCCACAAGAATGGAATCATTCACAGGGAC
ATTAAGGGGGCAAACATCCTTGTTGATAATAAAGGTTGCATTAAACTTGC
AGATTTTGGTGCATCCAAACAGGTTGTTGAGCTGGCCACTATTTCAGGTG
CCAAGTCTATGAAGGGTACACCGTACTGGATGGCTCCTGAAGTCATTCTC
CAGACTGGTCATAGTTTCTCTGCTGACATATGGAGTGTTGGATGCACTGT
TATTGAGATGGCTACAGGCAAACCACCATGGAGTGAACAGTATAAGGAGG
TTGCTGCTCTCTTTTATATTGGGAAGACCAAGTCTCATCCACCAATTCCT
GAGCATCTCTCTGCGGAGGCAAAAGAATTTCTGCTGAAATGTTTACAGAA
GGAACCAGAGTCAAGACCCCCAGCATTTGAGTTGCTGCAGCATCCTTTTG
TTACCGGGGAATCCATGCCTTCTCCTCTTGTTCATTCTTCATCCTGGGAA
TGCTCTAAAACCCCCAATTCTTTGTGCGGTAAACATCTGGACAATATGTC
TAGCTCGATTGATGTCTGTAATTTGGGCACTCTGAATTTCTCCAGTGTAA
ATACTGAGAAATTATCAGAAAGTAGATATATTTCTGAAGTAAATAAGAGT
GATGATTACATGTGTCAGATTGATGACAGTGATGACTTTATGAGTTATAA
CCCGATATGTGAGTCCTCTGATGACTGGAAGTGCATTGAAAGTTCTGGAG
TGGAACAAGAAAGAATGGACACGGACAATGATCAACAAGTTGATATACTG
GCCACTACCTCTGGGTTATCCATGAAGGGTGAAAACAGTTTCTCACTTAA
TGGTGCTGCGTCTCTGTCTGAGGATGAAGATGAGGTTACTGAGTCTAAAA
TTATAGCCTTTCTGGATGAGAAGGCTCTAGAACTAAAGAAATTGCAAACA
CCTCTTTATGAAGAGTTCTACAACAGCTTGAATGCTTCATGCTCCCCGAA
TTTGGCTGAGACTCTGCATGATGGAAGTGCTCTGAATTACTTGAGATTAC
CTCCTAAAAGTAGGTCACCTAATCGTGCTGCCGTCGGAAGTCCTTCTAAA
GCAGTTGATGCTACTCATGCAAGCCCTGGAAGGAATAACAAGCACATTTC
AAATGTTGGCAGTGCAAGTAACCACACAATTCAGGACAAAACATCACCTC
AGCCTAATGATTCGGAAGGAGTTCTAGTAGATTCTGAGCAGGAACCAGAT
AGCCCGAGTGCTAGTTTTTCCGATATACAGAGAAAGTGGAAAGAAGAGCT
TGTTCAAGAGCTCGAGAGAAAGCGAGAGATGATGCGCCAGGCTACTACAG
GCGGGAAAACATCATCTCCAAAGGATCCATCTTTGAACAGGAACAGAGAA
CTGTCGAGGTTTGCATCTCCAGGCAAATAA
>EUC07900-RA [mRNA]
ATGCAGCATGATCAAAGGAAAAAGGCATCTGTGGATGTGGACTTCTTCAC
TGAATATGGTGAGGGGAGTCGGTACAAGATAGAGGAAGTAATTGGCAAAG
GAAGCTATGGTGTTGTTTGCTCAGCTTATGATACTCATCTCGGAGAAAAG
GTTGCAATAAAAAAGATAAACGATATTTTTGAGCATGTGTCTGATGCCAC
ACGCATCCTTCGTGAGATCAAGCTTCTTAGGTTGCTTCGACATCCTGATA
TTGTAGAAATCAAGCATATCTTACTACCTCCTTCGAGAAGGGAATTTAAG

GATATATATGTAGTTTTTGAGTTGATGGAATCCGACTTGCACCAAGTTAT
TAAAGCCAATGATGACTTGACTCCAGAGCATTATCAGTTCTTTCTGTATC
AGCTTCTTCGTGGCTTAAAGTACATACACACAGCCAACGTATTTCATCGA
GATCTAAAACCGAAAAACATCCTTGCTAATGCTGATTGCAAACTCAAGAT
ATGTGACTTCGGCCTTGCAAGAGTAGCCTTCAATGATACGCCTACTGCTA
TATTTTGGACTGATTATGTTGCAACGAGGTGGTATAGAGCTCCAGAATTG
TGTGGATCCTTTTTCTCTAAGTACACGCCTGCAATAGATATATGGAGCAT
TGGCTGCATATTTGCAGAACTATTAACAGGAAAACCTCTTTTCCCTGGAA
AAAATGTAGTTCACCAGTTGGACATAATGACTGATCTCTTGGGAACACCA
TCTGCTGAAGCCATTGCTAGGATACGAAATGAAAAGGCTCGACGTTACAT
AAGCAGCATGAGGAAGAAAAGGCCAGTTCCTTTCTCCCATAAGTTCCCAA
ATGCAGATCCCCTTGCACTTCGTTTATTAGAAAGGATGCTAGCATTTGAT
CCCAAGGATCGACCAACTGCTGAAGAGGCACTTGCAGATCCATATTTTAG
GAACTTGGCAAAGGTCGAGAGAGAGCCTTCGGCTCAACCCGTCACAAAAA
TGGAATTTGAATTCGAAAGACGGAGAATTACGAAGGAAGATGTGAGGGAG
TTGATATACCGTGAGACTCTTGAGTATCATCCCAAGATGCTCAAAGAGTT
CTTGGATGGAGCTGAACCAACAGGCTTTATGTATCCGAGTGCGGTTGATA
AGTTTAAGAAACAATTTGCATACCTTGAGGAACGGTATGGGAACGGTGGA
GCTGCTCCTCCTCCCGAGAGACAACATGCTTCTTCATTGCCCAGACCGTG
TGTATTATATTCGGATAATTCAAATTCAGCACAAAATTCATCGGACATCT
CGAATGAACTCTCCAAATGTTCAATTAAAGAAGCTGAGAAGCCACATCAT
ACTAACAGGACTTCTGCTATCCCTATAACAAGGCTTCCGCTCCATGTTCC
TCAAACCATACAAGCAGGAGGCGCGGCAAGGCCGGGAAAAGTCGTCGGTT
CCGTACTTCATTACAACAACTGTGATGCCATTGTCTCAGCTGCGGCGCCG
GCGGCAGAGGCAGCACACCAGCAAAGAATTGTCAGGAATCCGGCAGTCCC
CTCGCAATACACCACCGTCTCTACCTCCTATCCCAAACGAAGCTCCGTAT
GTAAAAACGATAGGGGAGAAGACGGAACTCAGATGCCGCCCAAACCTGAT
CAGTACATTCCGAGGAAAGTTGCTGCCGCTCAAGGTGGCGGTTCCGGAAG
CCACTGGTATTGA
>EUC11169-RA [mRNA]
ATGAACAATACAGCACCTAGGCCTGCTGGTCAATGGATCCAGCAAGCGGT
AGGTGTCATTCCAAAAGATGTGTTGGTGGAAAAGAATGTAAGTAACAACA
TTTCTGTGCAGACAGGAGAGGAATTTTCTTTGGAGTTTCTTCTGGATCGT
GCTACTCCAAAGCAAGTTCCTGTGATGCCTGATATGTCCAAGAAGCAGGA
GAATGGACTGCGATTCATTAACAAGCAGAACCACCATAGGGGTTATGAGG
AAATAACCAGACTTCTGGGGTTGAAGAGGATGGATTCTGAATGTGGTTCT
GATATAACTGAGTTTGCTTCTGCAAGAGGCTCCTCTTTTGAGATTGAGAA
TGTGCCTAATATTACCAAAGAATGTGGCTACCATGTGGAACAAAATATGA
ATGGGCATACACCAAGGAAGGCTTCTGTTGAAAAAACTTACAATTGGAAC
GTAATAGAACCAAATGCTTCACCTGTAAGTGGATCTGATTCGTCTCACAA
ACGCCAACCTTCAGCGGATAGTTCTGGACCTGGCAAGATGAAGTTCCTCT
GCAGCTTTGGTGGTAAAATCTTGCCTCGGCCAAGTGATTCTAAACTTAGA
TATGTCGGGGGAGAGACCCGCATTATTTCTATCAGGAAGGATATTTCCTG
GGAAGAACTTGTGAAGAAAACCACAGGAATCTGCAACCATCATCACATGA

TCAAGTATCAGCTTCCAGGGGAGGAACTTGATGCCCTTATATCTGTGTCT
TCAGATGAAGATATTCAGAATATGATAGAGGAGTACCATGGCCTCGAAAC
TCTTGGTGGCTCTCAACGTCTAAGGATATTTTTGATTCCTTCAAATGAAT
CTGAAAATACATGTAACCTTGATTCTGGGAACTTACAGCCAAGCAGTCCT
GATTTTGATTATGTTGCCGCTGTAAATGGCATAGTTGACCCTAGTCCCCG
GAAAAACTATGATAGGCAGCAGCATTTGGCAGGTGATTTAAGTCAGCTGA
AATCTAATGAGAATTCTCCCTCGACTTTGGTTCCTCTGGACATCAATGAC
ACCTTTGATGTCTCACATACTACTAAGAATGCTTCCCCCAATTTAATCAT
GTCTCCCCGTGGGTCTCCTTTTTCACCAGTGATTGTTCGTCAGGGAGAGT
CAAAGAGTGTTCATGCACAACCATACAAGGACGATGCATCCTGTGGTAGT
TCTGAAAGCACCAGGCTGTTCAGCAATGCTCAATTAGCTCCAGAAGACTC
CAGTAGCAATATTGCTGGGAATAATTGTCCCCATCTTGAGGCAGCAAATG
TGGTAAGTTATCAATCTCCTAACCAAGATAATGATTTGGGCCATTCTGGC
AAATCAGTTGGAGCGCATTTTGACAATCCGAATCACAACAGAGATTTTTC
AGTGCCTCATTTAGTTAATCAGAATGATAAGGTGTCTGATAAATGCCATC
TTGAGAGGCCAGTTGTTACAGGTAGGCCATTTCATTCTGATAAGCCCATC
GCTTGCCTTGATGATCCATTGGGTCTACAGTTGGGGTCTAATGATTCCAT
TGGCTCTCATCGTGGGGTGCCACATGTGTTTTCAGATTCACAACTACAGC
TAAATGCCGGAAGGTCTACTTACTGCTCACAAGAAAGACCAAACCCTTCT
TCTCCCTTAACCTTTGCCGTTCCACAGTTATCTCCGCCGGTGGCATCTGC
TGCTTTGCAAGAAAAACCAGTCCATCTTCGTCAGAATATTGCTCCAGTCA
GTCCTTATCGTGAAACTAAGTTACCGGATGTAGAGCCAACTGTTTCTAAT
GTTAGGATGGATTTGCCGAAGTCTTCTTTAGAGTTGGGGACCAAGGTGGA
ACGTGCACGTTGGGAAGCAGCGACTATAGATGAGAAGTGCAAATCAGCCG
AAGAAGATTTAAATAAATACAGCTTCATGATGCAAAAGCATGATCAGAAA
AATCATTTGATTGGTAAGTTGATGAATGATCCAGATGAAGAGGACCCTCT
TTGGTGTCGGGTTGAAAAGCCTTGTGAAAGTTTAGCTGATAACAGTTTGA
CAGATGTGAGCAATTCCCCAAATATTGGTCGCGACCCATTCTTTATGATT
GGTGATAACACTACAACACAAGATTGGCAAATTCCAGGGGGAATGGTTCC
TGGTGCTTCTGCAATTTTTTTGAATCCTTTTGCAGATCAACCAATGCATC
AGGCCCCAAACAATCAGTTGGTGGAAGCCCCATCTGAGATTAATGTAAAG
GGCCAGAGGCCTACAGAGAATCATCAGATTCTTTCAACTGGGATAACGAA
TGGTGAACAGGAAACCAATTTCTCATGGGCCAAGAAAGTGGAACTATCAA
GAATAATTCAAGGTTCTAAACAGCAGGCCTATTATGAGAGTTCCTTCAGT
AATCCACTCCAAGCATTTCACAGTGGACTAGTTTTTGATGAACAAATTGT
GCACCAACCTTTCGCTCATCTTAGTCCCGAGGAATTGCGCCCATCTCAAG
TTTATGATGCAGCTGCCTTGCACACAGACATGCATACCCCTACTTCAGAT
CAGAATCCGACCAACGATACTGGAATTACGAGAGAAGCTTATATCCTTCA
TGATAGGTTTGCAAATTACTCTGATCAGAAGGTAGAAAATTCAGGGACTG
CTGAATTCTCCTTTGAGAAACCAAAACTACGAAATAACGTACCAATTAGA
TATGATCAGGGAAATCAGCTAGAGGTTGTGAATACTGTAGAAGGCGCGAG
TAACACTATATTACCTGGCATATCTCCTACTTTGAAGATGCCTTATGGAG
TGGATGCAAATGGTTGTAAAGTTCCGTACTCTCTTGCAATGCAAGCTAAC
AGCATCTCTGATTTCAAGAATGGTGAAGCTGATTATGCTGATAAGGATGA

ATCTATCAGCGACATTCGGATGGCTGAAATGGAAGCGGGTATATATGGGT
TACAGATTATAAAGAATGCTGATCTTGAAGAAATACGGGAGTTAGGATCC
GGTACATATGGAACTGTTTTTCATGGGAAATGGAGGGGAACGGATGTTGC
TATAAAGAAAATAAAAAAGAGCTGCTTTTCTGGGCGATCATCAGAGCAAG
AAAGATTGGCAAAAGATTTTTGGAGAGAGGCACAGATCCTTTCAAATCTT
CATCATCCAAATGTGCTTGCGTTTTATGGGGTGGTACCTGATGGTGCTGG
GGGAACGTTGGCAACTGTAACTGAATTCATGGCAAATGGATCACTTAGGC
ATGTTCTACTCAAGAAGGATAGGCTACTCGACCGGCGTAGAAAGCTTATA
ATTGCTATGGATGCAGCTTTTGGAATGGAATACTTGCATTCGAAGAATAT
TGTTCATTTTGATCTTAAATGTGATAATTTGCTCATTAATATGAGGGATC
CATATCGACCCATATGCAAGGTTGGTGACTTTGGATTATCACGTATTAAA
AGGAATACTCTTGTTTCTGGTGGTGTGCGAGGAACCCTACCATGGATGGC
ACCAGAACTGTTGAATGGTAGCACTAGCCATGTCTCGGAGAAGGTTGATG
TGTTTTCGTTTGGCATTGCATTGTGGGAGATCCTGACAGGAGAAGAACCT
TATGCAAATATGCATTGTGGGGCAATCATTGGAGGTATTGTGAAGAATAC
TCTCCGGCCACCAATTCCAGAGCGGTGCGACACTGAGTGGAGGCGGCTGA
TGGAGGAGTGCTGGTCAGCTGACCCTGCTGCCCGCCCATCATTCACTGAT
ATAACGGAGAGAGACTTCGATCCATGGCCTGA

>EUC08948-RA [mRNA]

ATGGAGCAATCAAGAAATAATGTCAATTTCAACTCCACAAGACCTGAAAA
TGAGGAGCTTGATCCGAGATCTCAAGGTTACACGCGAGACTCATCAGGCC
ACGTAATTGCTAATATAGATCCGGCAGAAATCAAATTTCCACAAGCTAAA
CCCGTGCTTAATTACTCTATACAGACGGGTGAAGAGTTTGCTCTTGAATT
TATGCGTGAACGGGTGAATCCCAAGAAACCATTTGTGCCAAATGTTGATC
CCAGTTATACACCCTGCTACATGGAACTAAAAGGGATTTTAGGGATTAGT
CATACTGGGTCAGAAAGTGGGTCAGATGTTTCCATGCTCACCATAGTGGA
AAGAGGTTCCAAAGAGTTAGATCGGAGGAACTCCTCTCTGTATGAGAATA
GAAGTAACTATGGGTCTGTGCAATCTGTGCCACGGACTTCATCAGGCTAC
AATAGTAATCAAACAATCATTCAAGATTATTCCTCTTCGGGGGCATCCGG
AAGCGTATCTACAAAAATGAAGGTACTTTGCAGCTTTGGTGGTAGAATCC
TTCCTCGGCCAGGTGATGGAACGCTCAGGTATGTCGGAGGTGAAAATCGC
ATCATCCGGATTAGCAAGGATATCTCTTGGCAGGATTTATGGATAAAAAT
AAAAACAATCTATGAACTCACTCAAACGATTAAGTATCAACTTCCTGGGG
AGGATCTTGATGCCTTGGTTTCTGTTTCTTGTGATGAAGACTTGCAGAAT
ATGATGGAGGAATGCAAGGTGATTGAAGTAGGAGAACGATCCAACAAGCT
TAGGATGTTCTTATTCTCCATAAGTGATTTGGATGATGCTCATTTTAGTC
TGGCCAACTCAGAGGGTGACTCAGAGATTCAGTTTGTGGTTGCTGTCAAT
GGCATGGACATCGGATCAAGAAAGGATTCGAATTTGCATGGCCTGGCTAG
CTCTTCAGCAAATACTTTGGATGAACTCAATATGCAGAATGTCCAGAGGA
ACAAAAGTGGAGCTGCAGCTGACTTTTTTAGTGATAATACTTTTACCTCC
ACTGGCTTTGTCACCATGCCACCAACATCTACATCTGTTTCACCTGTTGT
ACCAACTTCATCCCATGTTACTGAAACTGCCCCACAGTTGTATCACAGTG
ACATGGTACATCAGGACGCAGCTAAGGAAGATGACTCTCATTCTTCTCAA
ATATCTTTTTTTGGAGAAACTTCTGTTCCACAGCCTATTCAAAGTGGCTC

CGAAAGGCAACAATCCCAGGGTGAACAGGTTGTGGAAACACATGTGGAGG

ATGCTAAACAGAAAAATGTTGGTTCTGTTCAGCAGGGGCATGAACAGGAA

ACTATTCGGCCCTTGGCAAATGAGTATCTTGTTCCACCAAACCCTTATAA

TGGTAGTGTGACAGATTATTTTCCTGCTCGAGAAGTTGTTGGCCCTACAC

CGGAAGGAAATTTTTCTTTAGATAGTATGAAGAATGAGGCAAGGCCCCAT

GAACCTGTAGAGGTCTCTTCGGCCCTTGGTGCAGCAGAAAATCATAAATC

CATTGGTGAGGACTTTCACGCATCTAGTGGTGCATTTGCTCCTGGACCTG

TCAATTCCGAGAATGATCCTACTGATCCAGTTTTTGTTGAGCCTGACATA

CCTACTCAAAGAGTTTTTCATTCTGAGAGATTTCCTCGAGGACATGCAGA

GTCGCTGAACAGGTTATCAAAGTCAGACGACTCACATGGTTCTCAATTTC

TCATGACTCATTCACGTTCTGATATTGCACAGAAAGATTTAATCGTGGAA

TCTGCTGAAAAATTGCAGAACGGAAATTATGGTCGACAGAATGAGCTGGT

CAATACTGGCACTACAATTGATGGAGTTAGGAATCTACAACAGTTGAATC

AGTCAATTCCATCCCCTGGTAATGGTGAAAATGTTGTGCTCGAGAATCAG

ATTACTAAAACTGACCAGGGAGTAACCATAGATAACAGCGGGAATCTCCT

TGTTGACGAATTCGAAAAAACTGGATCAGAGCTCCCTGCTGTGAGCAGAG

TTGATTCGAGGAAGCACCATGATGAACCTGCTAGTAGTCGTCTGGATGTT

CAGTTGGGTGATACAGTGGGAAGCGATTCCACCCCTAGTAACACTCGGGG

GCTTGCTCCGACTTCTGCTCGGGTAGGGCGCCCAGCTGGAGATTTCTCTT

TGGATGAATCTTCTGTACATGCTCCTAGACCAGAGCGTGGTGACATACTT

ATTGATATTAATGACCGCTTCCCTCGTGATTTCCTTTCTGATATATTCTC

AAGAGCTGTACTTTCAGAGGACCCTTATAACATTGGCCCGATGGATAAAG

ATGGAGCTGCTTTGAGCTTGAACATGGAAAACCATGAACCTAAACGTTGG

TCATTTTTTCAGAAATTGGCTCGGGATGACTTTGCTCGAAAAGATGTTTC

TCTAATTGACCAAGATCCAGGATTTTCATCTAGACTTCCAAAAGTCGAAG

AAGAACTATCTGGTTCTGTTATACCTGACACTATAGCTTTGCATTCAAAT

TATGTTTCTTCCATTGCGAAAGTCAGTGAAGGAATACAGTACCACGATTT

GGTGGACAATCTTAGGATAGTGGAGTCAGAGTATGAGGCTGCAATGAGGA

CCATTGGCCTCTCCCCTCTAGATTCATGTTTGGCTGAATTCGATATAAAT

TCTGTGCAGATTATAAAGAATGAAGACCTTGAGGAGCTCCGGGAACTTGG

CGCTGGCACATTTGGGACGGTCTACCATGGAAAATGGAGAGGTACAGATG

TTGCAATTAAGAGGATAAAGAAGAGTTGCTTCTCAGTTCGATCATCAGAG

CAAGAGAGATTGACCATAGAGTTCTGGCGGGAAGCTGAAATTCTCTCAAA

GCTTCACCACCCAAATGTAGTGGCATTTTATGGTGTTGTGCAAGATGGGC

CTGGGGCAACACTAGCGACTGTGACAGAATTCATGGTTGATGGTTCACTA

AGGCATGTTTTACTTCGCAAGGATAGACATCTTGATCGTCGGAAGCGGCT

TATAATTGCCATGGATGCTGCCTTTGGGATGGAATATTTGCATTCCAAGA

ATATTGTTCATTTTGATCTGAAGTGTGACAATTTGTTAGTCAACTTGAAA

GATCCTTCTCGACCCATTTGCAAGGTAGGTGATTTTGGCCTATCAAAAAT

AAAACGGAACACTTTGGTTTCTGGTGGAGTTAGGGGTACTCTACCATGGA

TGGCGCCAGAGCTGCTGAATGGTAGCAGTAATAAGGTCTCTGAAAAGGTT

GATGTGTTTTCCTTTGGTATTGTTTTGTGGGAGATTCTGACCGGGGAGGA

GCCTTATGCCAACATGCATTACGGTGCAATTATAGGGGGAATTGTAAGCA

ACACGTTGAGACCAACGATACCAAGCTACTGTGATGCTGAATGGAGAAAA

TTAATGGAGCAGTGTTGGGCTCCAAATCCTGTGGCAAGGCCTTCTTTCAC
TGAAATTACCAGTCGGTTGCGTGTGATGTCTTCTGCTCCCCAACCCAAAA
CCCACAAGCCATCGAACAACTAA
>EUC14834-RA [mRNA]
ATGAGACCGCTTCAGCCTCCGCCAGCCGCCTCTGGCTCCGCCGCAGCCAA
TCGCAACCGTCCACGGAGGCGTCCTGACCTAACCTTGCCTCTCCCGCAAC
GCGAGACTTCCTTGGCCGTACCGCTCCCTCTGCCGCCGTCCTCAGCTCCA
TCGTCTTCGAATCCGTCGCAGTTTCCGTCCGCCTCGACGCCGATTAATTT
TTCCGCTCTGGAGCGGATCTGTCGAATCGGGAGCGGTAGCGGCGGCACGG
TCTATAAGGTACGCCACCGTCCTACCGGGAAGCTGTACGCGCTCAAGGTG
ATCTACGGTCACCACGACGACTCCGTCCGCCGCCAGATCTGCCGCGAGAT
CGAGATCCTACGCGACGTCGATAATCCCTCCGTCGTCAAGTGTCACGATA
TGTTCGACCACAACGGTGAAATTCAGGTCCTCCTCGAGTACATGGACGGC
GGATCTCTTGAAGGCACTCACATCCACCACGAACCCTCCCTCGCCGATCT
CGCTCGCCAGGTCCTCTCCGGCCTCGCCTACCTCCACCGCCGGAGAATCG
TCCACAGAGACATCAAACCCTCCAATCTACTAATCAACACTCGCAAGCAA
GTGAAAATCGCCGATTTCGGGGTTTCAAGAATCCTAGCTCAAACAATGGA
TAACTGTAACTCCTCCGTCGGTACAATCGCTTACATGAGCCCGGAGCGGA
TCGATACGGATCAGAATCACGGAAAATACGATGGCTACGCCGGCGATATC
TGGAGCTTTGGGGTAAGCATACTTGAATTTTATCTTGGCCGGTATCCATT
TGCGGTGGGTAGACAAGGCGACTGGGCTAGTCTCATGTGCGCAATCTGCA
TGTCCCAGCCGCCGGAAGCGCCACCCACAGCGTCGCCGGAGTTTCGAGAC
TTCATAGCTTGCTGTTTGCAAACAAACCCAGCAAAACGGTCGACCGCCCC
AAAATTGTTAGCCCACCGTTTCATTGCGCAGTACGCCGGCTCTACTGGCC
ATAGCGGCAATCATCAGATGCACCAAGTACTCCCTCCACCTCGACATTTC
TCTTCTGCTTGA
>EUC12664-RA [mRNA]
ATGCCTCCATGGTGGAGCAAGTCTGCTTCAAAAGACGTAAACAAGAAGGC
AAACAAGGAGAGTTTTATTGATACGATACACAGAAAGTTCAAGATTGTCT
CCGAGGGGAAGTGTGGAAGTAGGGCATTTTCGGACAAGGCTTCTCGTAGA
AGAAGATCCAGCGACACATTTTCAGACAAAGGTTTATCGCCATTGCCATC
TACTCATGTTTCAAGGTGTCAGAGTTTTGCCGAGAGGGCTCAAGCTCAGC
CACTTCCTCTTCCCGGTTTACAAAATCCAGCAAATCTCCGACTGGCCGGA
AATAGGGCGTCAAAAAAATCAGGCAGTGAAGAAGGATGTAAAACTCCAAA
TTTGCCCGTCCCTAGATCTGGAAATAGTCCTAATGTGTTGAATGAGGGGG
ATTTAGCGACTGCTTCTGTTTCGAGCGATAGTTTGAGCGATAGTGATGAT
CCGTTTGAGTCACGTCTCTTAAGCCCTCAGGTGTCTGACTATGAGAACGG
GAACAAAACTGCCATCAGCAGCCCTACAAGTGTGAAGCACAAGGATCAAT
CCCCACTCACATCCCAAATGAATGCAAGAATGGCTAACATGTTGTTGAGT
AATCAAACTCAGCCTGTCCGGGCATCAGCTAGAAGGGTGCCGAATTTGCA
GATTCCAAACCCTAATATTTTTCATAGTGCGCCAGATAGTTCAATGTCAA
GTCCTTCAAGAAGTCCCATGAGAATTTTCGACCCTATCACGAGTCCTTAT
TTCTATTCAGGACACTGCTCTAGTCCTGGTTCGGGTCAAAATTCAGGGCA
TAATTCGATTGGAGGAGACATGTCAGGACAACTCTTTTGGCCACACAGCC

GTAGCCCTGAGTGTTCGCCAATACCTAGCCCCAGAATGACTAGCCCTGGA
CCAAGCTCACGAATACATAGTGGTGCAGTCACCCCATTGCATCCACGAGC
CTGTGGGTTAGCCGATGATGGGAAGCAACAGAGCCACAGGCTACCCCTTC
CACCATTAACGATACCTAATTCTTCTCATGTTTCTCATTCGTATTCGACT
GGAACGACTCCTATTGTTCCAAGAAGTCCTGGTAGAACGGAAAATCCGCT
GAGTCCCGGGTCACGTTGGAAGAAAGGACGATTGCTTGGTAGAGGCACAT
TTGGGCATGTTTATCTTGGCTTTAACAGTGGAAGTGGTGAGATGTGTGCA
ATGAAGGAGGTGACACTGTTTTCCGAAGATGCAAAGTCAAAGGAAAGTGC
GCAACAACTAGGGCAAGAAATTGCTCTATTGAGTCGTTTACGGCACCCGA
ACATTGTGCAATATTATGGATCTGAGACAGTAGATGACAAACTATACATT
TACCTGGAATATGTTTCGGGCGGTTCCATTTACAAGCTTCTTCAAGAATA
CGGCCAGTTGGGCGAAGCAGCAATTCGAAGTTATACTCATCAAATTTTGT
CCGGGCTTGCTTATTTGCATGCTAAAAATACTGTGCATAGAGATATTAAA
GGTGCAAATATACTGGTTGACCCCAATGGCCGTGTGAAATTGGCAGACTT
TGGGATGGCAAAGCATATCACTGGGCAGTCTTGTCCTCTGTCATTGAAGG
GAAGCCCTTATTGGATGGCACCTGAGGTTATAAAGAATTCAAACGGTAGC
AATCTTGCTGTGGATATATGGAGCCTTGGATGCACGGTTTTGGAGATGGC
AACAACAAAACCACCTTGGAGTCAATATGAAGGGGTTGCTGCGATGTTTA
AGATTGGGAACAGCAAGGAACTCCCTACAATCCCTGATCATCTATCGGAT
GACGGTAAAGACTTTGTTAGGCGGTGTTTGGATCGCAACCCGCTACACCG
TCCTACAGCTGTAAAGCTGTTGGAACACCCTTTTGTGAAAAATGCTTCTC
CATTGGAAAGACCAATTCTATATCCTGAAGAAGGATCATCTCCAGCGACA
AATTCAATAAGATCACTCGGTATTGGAGGATATGCCACAAGTTCTTCACT
CTTTGAATCATCTGCTTCTCATCTTTCTAGAAGTCCGAGAAATGGTTCAG
CCTTCAGCGATGTGAATACACATAAAAACATATCATGCCCGGTTTCCCCT
ATTGGTAGCCCCCTCTTGCATTCAAGGTCACCACAATGCACGAGCGGAAG
ACTCACACCATCTCCCATATCAAGCCCAAGAGCCGTTCTGTCTGGTTCAT
CAACCCCTCTCACTGGCGGCGGCAATGGCTTCCACCCCCAAACTCATCCG
CCACTTAACCACCAGAATGAAAATATGGGAACAATGTTCCCACTTCCCCA
GAATCATTCCCACCCCAAACCCGATATGTTCCGAGGAATTTCTCAAAATG
GGTTTCTTGGATATCAGTTTGGTGGGCAATCCGTTTTGGCAGATAGGGTT
TCGCAGCAGCTTTTGAAGGGGAATGTGAAAGTCAACCCATTGTTTGACCT
AAATCATGGATCTCCTGTTCTTGGCCATGGCAATGGAACAAGCTATTGA
>EUC12684-RA [mRNA]
ATGCTACAGCTACTCCGTCCGAACTCATTAAATTCCACTCTTTCGGGCGA
ATCGGCCGACGATTTCTCCGCCTATTACAGCCGAAGTTCGTCCACTCCTA
GCGGCGCACGGCACTACACGACTCGCACGCCTCTCTATTCTGAGTTGATC
GATCGTAGCTTGGCCATGCCAATGGAGTCGACTTCAGCTTCGGCTTCCGG
ATCGAGAAATGACGGCGGGATTAAAGGAGTGCTCACTCATGGAGGTCGAT
ACGTCCGGTATAATTTGTACGGAAGCTTGTTCGAGGTCTCCAGTAAGTAC
GTCCCTCCTCTTCGGCCCATCGGCAGAGGTGCTTATGGCCTTGTCTGTGC
TGCTGTAAATTCAGATACACGTGAGGAAGTTGCTATTAAGAAGATTGGCA
ATGCATTCGACAATCGAATAGATGCCAAAAGGACATTGAGAGAAATTAAG
CTTCTCCGTCACATGGATCATGAAAATATTATTGCCATCAAGGACGTCAT

AAGACCGCCAAAGAAGGATATTTTTAATGATGTTTATATCGTTTACGAAT
TGATGGACACCGATCTTCATCAGATAATTCGGTCGGATCAACCCTTGACT
GATGATCACTGCCAGTACTTTTTGTATCAGTTGTTGAGAGGATTAAAATA
CGTACACTCAGCTAACGTCTTGCACCGTGACCTAAAGCCCAGCAATTTGC
TCCTAAATTCCAATTGTGACCTCAAAATTGGAGATTTTGGGTTGGCAAGA
ACAACATCGGAAACAGATTTCATGACTGAATATGTGGTCACCCGCTGGTA
CAGGGCACCCGAATTGCTCCTTAATTGCTCGGAATACACTGCCGCTATTG
ATATCTGGTCTGTTGGTTGCATACTAGGTGAAATAATGACCAGAGAACCG
CTGTTCCCGGGCAAAGATTATGTTCATCAGCTTAGGCTCATCACAGAGCT
ATTAGGTTCACCAGATGACTCCAGCCTTAGATTTCTCAGAAGCACCAACG
CCCAAAGATACGTTAAACAGCTTCCCCATTACCCAAAACAACAATTCTCA
TTGAGATTCCCAAACATGTCCCAACTAGCCGTCGATTTACTCGAAAAAAT
GCTCGTTTTCGACCCCAACAAACGCATTACAGTTGATGAAGCTCTTTGTC
ATCCGTACTTATCGTCTCTTCATGATATCAACGACGAGCCCATTTGCCCG
ATGCCTTTCAGTTTTGATTTTGAGCAGCCATCATTTAATGAGGAGAACAT
TAAGGAGCTTATTTGGAGTGAAACTCTCAAGTTCTATCCAGATTTAAGCA
ATTAG
>EUC01764-RA [mRNA]
ATGCAGTCCGATCACCGAAAGAAAAATTCAGCAGAGATGGACTTCTTCTC
TGAGTATGGTGATGCAAGTAGGTACAAAATCCAGGAGGTAATAGGAAAAG
GCAGCTATGGTGTTGTTTGCTCAGCATTAGATACTCATACTGGTGAAAAA
GTGGCAATAAAGAAGATACATGATATCTTTGAACACATCTCTGATGCTGC
ACGAATCCTCCGTGAGATCAAGCTTCTCAGGCTTTTACGGCATCCTGATA
TAGTTGAAATCAAGCACATCATGCTTCCTCCTTCAAGAAGAGATTTTAAA
GATATTTATGTAGTTTTTGAGCTCATGGAGTCGGATCTTCACCAAGTCAT
CAAAGCTAATGATGATTTGACACGTGAGCACTATCAGTTTTTTCTTTACC
AGCTGCTTCGGGCTCTAAAGTATATTCATACAGCAAACGTCTACCATCGG
GACTTAAAACCAAAAAATATATTAGCAAATGCAAATTGCAAACTCAAAAT
TTGTGATTTTGGTTTGGCGAGAGTTGCGTTCAACGATACACCTACAACAA
TATTTTGGACGGACTATGTTGCAACGAGATGGTATAGAGCTCCAGAACTA
TGTGGCTCATTTTTCTCCAAGTATACACCTGCAATCGACATATGGAGTAT
AGGATGCATCTTTGCGGAGGTTTTAACCGGAAAACCACTTTTCCCTGGAA
AAAATGTCGTTCACCAGTTGGATTTGATGACTGATCTTCTTGGGACGCCA
TCAATGGATACAATTTCTCGGGTTCGAAATGAGAAGGCCAGGAGATACCT
AACTAGCATGAGGAAGAAACAGCCTGTGCCATTTGCCCAGAAATTTCCTA
ATGCTGATCCTTTGGCATTGCGTTTATTGGCAAAGCTACTTGCTTTTGAT
CCAAAAGATCGGCCAACTGCTGAAGAGGCCTTGGCTGATCCTTACTTCAA
GGGATTGGCCAAAGTTGAGAGAGAACCATCTTGCCAGCCAATTACCAAGA
TGGAGTTTGAATTTGAGAGGCGAAGGGTTACAAAGGAGGATATACGTGAA
TTGATTTTCCGAGAAATACTAGAGTACCATCCCCAGCTACTAAAGGACTA
TGTGAATGGAACAGAGAGAGCTAACTTTCTCTACCCCAGTGCTGTCGATC
AGTTCAGGAAACAGTTTTCTCATCTTGAAGATGGTGGTGGTAAAAGTGGG
CAAGTGGTTCCGCTCGAGAGGAAGCATGTTTCACTCCCTAGGTCTACAAT
AGTACATGCAAATCCAATACCTCCTAAAGAACAACCAAATGTTGCTAAAG

ATCGGCAAATCTTGGAAGAGTCTCGCGGTAGAACTTCCCAGCCGCCACAG
AGGACAATGCCAATGGCGAAACCCGGAAAAGTTGTGGGTTCGGTTGGGTC
ATATGAAAACGGAAGTGTGGCCGGAGAAACTCGTGATCCGAGGACTTTTT
CAAGAAGCTCAGGCATTCATAACCAGGTTATTACGCCGGAATATTATTAC
AACAGGGGAAAGCAGGAATTGTCTTCTCAGAATCGACGAGTCCCTCAGTG
TGGAATGGCGGCCAAATTAGCGCCGGATATTGCCATCAACATCGACACCA
ATCCATTTTACATGACCCGTGTAGGGGTAATGAAGATGGACCGTGTTGAA
GATAGGGTTGAGATCGACACGAACTTGTTGCAGGTGCGAGCTCAATACAG
CGGTGGAATTGGAGGTGCAGCAGCGGCAGCTGCAGCGGCTGTTGCTCACA
GAAAAGTTGGGAGTGTTCAGTTAATCAGAATTCGAAGCGAACCTGATCCA
TGGAAGACACGGCAGTTGAAAAGCAGCCAGATCACCGAAAGATGGTCGGT
GGCCAAGATCGATCAAGCATTGAAAGATTTGAGTGACACAAATTTACGTG
AATTGCCATCTTCTGTCGGAAATTTGAGGTCGTTGTGCTATCTCGATGTC
TCTGAGACTCCCTATCAAATGGTTACCAGAGTCGAGTCGATAGATCACCT
TGGAAAATTGCTGACCCTGAGGCTCAGAGGTTGTGCAGACCTCACTTGA
>EUC00315-RA [mRNA]
ATGGAGGACACACGAGATGAGTCAGGGACATCAGAGCAGAGGCCTTCTTG
TGCTACCTGGTGGTCTTCAGATTTCATGGAGAAATTTGCATCTGTTTCTG
TGGACTCTAAAGAAGAAGCTTTGAACCGTAAAGTGTCAAACAGTAACAAT
ATGTATGATAGGTTTTCATCTGAGACAGCATCACAAATCCTCTGGAGAAC
TGGAATGCTTGTTGAACCAATTCCAAATGGTTTCTACTCTATAATTCCCG
AGAAAACGCTCAAGGAACTATTTGATGATATTCCTAGTTTAGAGGAGCTT
CATGCTCTTGAGCTTGAGGGTCTAAGAGCTGATATCATACTTGTAGATGC
AGAGAAAGATAAGAAGCTTTCTATGCTAAAGCAATTGATAGTTGCTCTGG
TGAAAGGTTTAAACTCAAATCCTGCAGCAATGATAAAAAAGATTGCTGGA
CTGGTTTCCGATGTTTCTAAACGGCCAAATGTGGAACTAAGTCCAGCAAG
GGCTGTTCTTGAGGAAGCCTCTTATGCTTTGGAGAATCGAGGTGTCCAGA
TGTTGGGGCAAATAAAACATGGATCATGCCGGCCTCGGGCAATTTTGTTC
AAAGTTCTTGCAGATACTGTAGGTCTTGAAAGTAGGCTAATGGTGGGTTT
GCCTAATGATAGAGATCCTGAATGTCTGGAATCATATAAGCATATGTCTG
TAGTAGTTGTGCTGAATTCTGTGGAGCTACTTGTTGATCTTATGCGGTCT
CCTGGCCAACTGATACCTCTATCTGCCAAGGCAATTTTCATGACGCATAT
ATCTGCAGCTGGGGAAAGTGATTCTGCAGAAAATGACTCCTGCGATTCAC
CATTAGAACCAAACAGTCCTCTATATGGGTTTTCGGAGAGAGTGGATCCA
GAGAGTGCTGAGAAAGATGACAACCTTCAGTACGAGCAGAGATTAGAAGC
ATCTTCAAATGTTCCTGGGCATTCTTTAAGGAATAGGATGTTACGATCTT
CCACCTCCATTGACAGAAGAATGAGTTTGTCACATAGCGAACCCAATATT
GCAACTACTTTTTGGAGGAGGAGCCGGCGAAAGGCTATTGCAGAACAACG
GACTGCAAGTTCAAGTCCAGAGCATCCTTCATTTCGAGCTCGTGGTCGAT
CAATGCTTAGTGGCGATAGGCAAACATTTAGAAATTATTCTGACCACATT
GCTGCTTCAAGCTACAGGTCAGAAGGAGCATCAACCTTGGAAACGCGTAG
ACTACGAAGAAGAAGTATCAGTATAACGCCAGAGATCAGTGATGACATCG
TGAGGGCTGTACGAGCAATGAATGAAACTTTGAAGCAAAATCGTCTCTTG
AGAGAACAAGGGGATAATAGGTCAGATCCCTATTCTTCAAATGATGGAAA

TCATGCGGCAGATCTTCAAAAAAATGTATCTGGTTCTCATGATGGAACAT
CTGGCGAGAGGCTTTCTTCATTTGCCCGCATCAGCAGGGAGCACATGAGC
TCTCAAAAAGCAATGTCTCTACCATCATCTCCTCATGAGCTTAGGAGTCG
GTCTTCTGACTACCTGGTCAATAATGATGAAATGGTCGCAGCATGGAACA
AAATTCTGGAATCCCCCATGTTCGAAAATAGGCCTCTGCTGCCTTATCCG
GAATGGAACATTGATTACTCAGAACTAACTGTTGGTACTCGTGTCGGAAT
AGGGTTCTTTGGAGAAGTTTTTCGTGGAACTTGGAATGGAACAGATGTGG
CTATCAAGGTTTTTCTGGAGCAAGATCTCACTCCTGAAAACATGGAAGAT
TTCTGTAATGAAATATCAATCCTGAGCCGCCTCAGACACCCAAATGTTAT
ATTGTTTCTCGGTGCATGCACAAAACCTCCACACCTGTCCATGGTTACTG
AGTACATGGAGATGGGTTCCTTGTATAATTTGATCCATTTAAGTGGTCAG
AAGAAGAAACTCAGCTGGCGGAGGAAGCTTAAAATGCTGCGTGATATATG
CAGAGGGCTTATGTGCTTACACCGAATGAAGATAGTTCATCGCGATCTAA
AGAGTGCGAATTGCCTTGTGAATAAGCATTGGATGGTCAAGATCTGCGAT
TTTGGCCTCTCGAGAATAATGACAGATGGAGCAATGAGAGATTCTTCGTC
AGCAGGGACTCCGGAGTGGATGGCTCCTGAACTAATCAGAAACGAGCCTT
TTACTGAGAAATGTGATATTTTCAGCCTAGGGGTCATAATGTGGGAGCTA
TGTGCCCTAAATAGACCATGGGAAGGTGTCCCGCCAGAACGGGTTGTCTA
TGCTGTTGCTAATGAGGGATCAAGGTTGGAGATTCCTGAAGGTCCCCTTG
GCAGGCTAATTGCAGATTGTTGGGCTGAACCGCATGAGCGACCAAGCTGC
AAGGACATTCTTACCCGTTTGCTAGACTGCGAGTTTTCCCTCTGCTAA
>EUC06431-RA [mRNA]
ATGGCGGTTTTTTTGTTGAAGATGCCAAGCGCGCAGTCCGATCAGGCTGA
TCGCGATGCGGAACCGTTTGTGGAGGTCGATCCGACGGGCCGATACGGCC
GGTACAGCGTGTTGCTCGGTTCCGGCGCCGTGAAGAAAGTGTATCGAGCG
TTTGATCAGGAGGAAGGGATCGAGGTGGCTTGGAACCAGGTGAAATTGCG
GAAATTCAGCGAAGATCAAGCGATGAAGGATCGGCTGTTCTCGGAGGTCC
GATTGCTGAGGGATCTGAGGAACAAAAACATCATTGCGCTGTACAGCGTG
TGGACGGACGAAGAGCGCGGCATGTTGAATTTCATCACCGAAGTTTGTAC
CTCCGGGAACTTGAGAGAGTACAGGAAGAAGCACAAACAAGTATCGATGA
AGGCATTGAAGAAGTGGTCAAAGCAGATTCTCGAGGGTTTGGACTATCTC
CACACGCATGAACCGTGTGTCATTCACAGAGATCTCAATTGCAGCAACGT
GTTCATCAATGGCAATGTTGGTCAGGTAAAAATTGGTGATTTGGGTTTGG
CAGCAATCGTAGGAAAGAATCACTCAGCACATTCTGTACTTGGGACGCCA
GAATTCATGGCACCAGAGCTATACGAGGAGGATTACAACGAGCTGATCGA
CATCTATTCCTTTGGGATGTGTTTGCTCGAGATGGTGACACTTGAACTTC
CTTACAGCGAGTGCGACAGCATTGCCAAGATATACAAAAAGGTTACATCT
GGGGTGAGGCCTCAGTCCATGAACAAGGTTAAGGACCCCAAGGTGAAAGA
ATTCATAGAGAAGTGCCTCGCTCAGCCAAGGGCAAGGCCTTCTGCTTCTG
ATCTCCTGAAAGATCCCTTTTTTGAGGGGATAGACGACGAAGATGATGAC
GAAGAAAACAACAACAATGGTGATGTTTCATAA
>EUC03449-RA [mRNA]
ATGGGTGTTAATGGTAGACGATCTAATTACACTTTGTTGAGTCAGATACC
GGACGAGCAGCATCACCAGCAACCGCCGGCGAAGGTAGCCGCCGTTGCAG

CTCAGTATTACGAGTCTCAAATCATGGGGAAGAGCGACAGAGGATTCGAC
TGGGAATTAGTCGATCAGAGAGTTGTTCCGCCGCAGAGTCGGATGGGGGC
GACGGCTTTTTCCGGGACGATCGGGTCACAAAGGCATTCGAGTGGGAGTA
GTTTCGGCGAAAGCTCGATTTCCGGCGACTATTATGTGCCGTCGCTGTCT
AATCCGGATGGGTTTGTACAGTTGCATGACGACGGAGGAGTTGAGTTGCT
GTTGAATACGGCCGAGGGCGGTGGCGGTGGAGGATCATCGTCGACAAAGA
GTTGGGCGCAGCAGACGGAGGAAAGTTATCAGTTGCAGCTGGCGTTGGCG
CTGCGATTGTCGTCGGAAGCTACTTGTGCCGATGATCCCAATTTCTTGGA
TCCGGTGACGGATGAATCGATGGCGAGATCATCTTCCTCCTCTGCTTCTG
CAGAGGCTATGTCACATCGATTTTGGGTAAAGGGCTCCTTATCATATTTG
GACAAAGTTCCCGACGGTTTCTACTTAATCCATGGAATAGATCCATGTAT
ATGGTCTGTATGCGTTGATTTTGAAGAGAATGGACGCATCCCATCGATTG
AATCACTGAAGAACCTTGATCCTGGGGCCGAATCGTCAATTGAAGTCATT
TTGATCGATCGATTTAGTGATCCAAGCTTGAAGGAACTTCAAAATAGGAT
TCATACCTTGTCTTGTAGCTGCATCACCTTGAATGAGGTCGTTGATCATC
TTGCGAAGCTCGTATGCAGTCATATGGGGGGTGTATCTCACATTGGGGAA
GATCACTTAATTCCGAACTGGAAGGAGTGCACTGACGAGCTAAAGGATTG
TCTTCGGTCCGTTGTCCTCCTGATTGGTAGCTTGCCCATTGGCCTCTGCA
GGCATCGGGCTTTGCTATTCAAAGTGCTAGCTGACACAATCGGTTTACCT
TGTCGAATTGCCAAAGGGTGCAAATATTGTACAAGAGATGATGCTTCCTC
ATGCGTTGTTCGTTTTGGTTTCGACAGGGAATATATGGTTGATTTGATTG
GGAATCCGGGATGCTTATGTGAGCCTGATTCTTTGCTAAATGGTCCATCT
TCCATCTCAATTTCTTCACCGTTGTGCTTTCCACGACTCAGACAGGTTGA
ACCTCCAATTGATTTCAGGTCATTGGCCAAGCAGTATTTTGCGGACTCTC
TGTCTCTTAATCTTGTATTTGATGATTCTTCTGCAGGCGATGTTCAACAT
CAACATTTGTTGCCTGGTACAAGCGGCAACAATGATGTTTCTCACTCAGC
TCTTCTACCATTGAATGCATGGCAAGAGGGTTTTAATCGAGATTCCCCAA
AGTCTGATTTCTCAAGAAATATACCTCCAATTGGGCATACAGATGATGTT
CAACAATCTTTACCCTCTAATGACTTGAGGTTTGGTATGGAAGATTTAGA
CATTCCGTGGAGTGATCTTGTTTTAAAAGAAGGGATTGGAGTGGGTTCTT
TTGGTAATGTTCATCGTGCAGATTGGAATGGATGTGATGTGGCTGTGAAG
ATTCTCATGGAGCAGGATTTCCATGAAGAGAGAGTTAATGAATTCTTGAG
GGAGGTTGCAATAATGAAACGACTACGGCATCCAAATATTGTGCTCTTCA
TGGGCGCTGTCACGCAGCCCCCAAATTTGTCCATCGTGACTGAATATTTA
TCAAGGGGTAGTTTATTCAAACTTTTGCATAAACCTGGCGGTAGGGAAGT
ATTGGATGAGAGACGCCGGTTGTTAATGGCTTACGATGTGGCGAAAGGAA
TGAATTATTTGCACAAGCATAATCCACCCATTGTTCATCGAGATTTGAAA
TCTCCGAATCTTTTGGTTGACAAGAAATATACGGTGAAGGTCTGTGATTT
TGGCCTTTCCCGTCTAAAAGCAAATACCTATCTTTCATCGAAGTCGTTGG
CGGGAACGCCGGAGTGGATGGCGCCTGAAGTTCTTAGAAACGAACAATCA
AATGAAAAATCAGATGTTTATAGCTTCGGCGTTATCTTGTGGGAGCTTGT
AACTATGCAACAACCCTGGAGAAATTTAAATGCTGCTCAGGTTGTTGCAG
CTGTTGGTTTTCAATATAAGAGGCTCGAGATTCCGAAAGATTTAAATCCG
AAAGTAGCCGCCATAATTGAGGCTTGCTTCGCAAATGAGCCATGGAAACG

CCCTTCGTTTGCCAGTATCATGGAGTCCCTAAGACCGCTGACTAAAGTTC
CGACGCCTCTAAGCATGCCATCGCTCACATGA
>EUC03396-RA [mRNA]
ATGAATGTGATTCAAAAGGCAGATGGATCGCGCAGGATTGTGTTGTCTGA
ACCGACTGGATCAACGGCTGAGGTGCTTCTCCATGGTGGTCAGGTTGTTT
CTTGGAAGAATGAAAGAAGAGAAGAACTGCTTTTTATGAGCAGCAAGGCA
ACAACAAAACCTTCTAAAGCCATGTCAGGTGGCGTATCCGTCTGTTTTCC
ACAGTTTGCAAATTTGGGTTCGGTAGAACAACATGGATTTGGAAGGAACA
GATTGTGGTCGGTGGATAATGATCCTTCACCTCTACTTCCTGCTAATAAC
CAATCAACAGTCGATCTAATACTAAAATATGCAGAGGAAGATCTAAAGAC
TTGGCCACACAGATATGAGTTAAGGCTTCGTATTTCTCTGAGTGCTAACA
AGCTCACTTTGATCCCTCGTATGAGAAATATCGACAACAAGTCCTTCTCG
TTTATGTTTGGTTTGCGTAATTACTTATCAGTATCTGATATCAGCGAAAT
TCGTGTTGAAGGGTTGGAAACACTTGACTACTTCGATAATCTGTTGCGCA
GAGAGAGGTATACCGAGCAGACTGATGCAGTTACCTTTGATGCTGAGATT
GATAGAGTGTACTTAAGCACGCCGACAAAGATAGCCATGATAGACCACGA
GAAGAAGAGGACCTTTGTGCTGCGTAAAGACGGAATGCCTGATGCAGTTG
TTTGGAATCCTTGGGACAAGAAGGCGAAAGCGTTACCGGATTTGGGGGAC
GACGATTACAAGACAATGTTGTGTGTGGATTCAGGTGTTGTTGAGACATC
AGTTGTATTGAAACCTTGTGAAGAGTGGAAGGGGTTTCAAGAGCTGTCTA
CTGTCTCCTCAAGCTATTGCAGTGGCCAGTTGGATCCAAGGAAGTTCATT
GGAGCTTGTAAAGAACCGATCATGGTGATCGTAACTGAGCTTCTTCTTGG
CGGCACATTGCGTAAATACTTGCTGAACATGCGGCCGAGATGCTTGGACA
GGGGGGTCGCTATCGGATTTGCCCTCGACATAGCACGCGCAATGGAATGC
TTACACTCTCACGGCATCATTCATCGTGACCTCAAACCTGAAAACTTGCT
GTTGACGGCAGACCACAAAACAGTAAAGCTTGCGGATTTTGGTTTGGCAA
GAGAAGAATCGTTAACCGAGATGATGACTGCTGAAACAGGAACCTATCGC
TGGATGGCTCCAGAGCTTTACAGCACAGTGACATTAAGGCATGGAGATAA
GAAGCATTACAACCACAAGGTTGATGCTTACAGCTTCGCAATTGTATTGT
GGGAGCTCATACACAATAAACTTCCATTTGAAGGCATGTCAAATCTTCAA
GCAGCTTATGCCGCCGCGTTTAAAAATGTGAGGCCGAACGTTGAAAACTT
ACCGGAGGATTTGGGTTTAATAATTACTTCGTGTTGGAAGGAAGATGCGA
ACGAGCGGCCAAACTTCAGCGAGATAATACAGATGCTGCTTCATTATCTC
TCCACCATTACTCCAATAATGGAACCTGCTGTTCCTCCCCGTATAATATG
TACCTCCAAAAACTCCGTGTTGCCGCCTGAATCTCCAGGCACAAGCTCTT
TAATGTCCAAAAGGGATGATTCCGGGGACACGCCTAAAACCCCCATGGAG
CGTGAACCCGGAGGCGGACTTTTCTTCTGCTTCAGACATTGTTACTAG
>EUC03168-RA [mRNA]
ATGGATTTGGCGGAAGGAGTTGGGGAGAGTTCGTCGCCGCCTCGGAATTT
TGGGAGCTTCGTTGGATACGATATCAGGACGCATGTGTATAGCAGGCTAG
TCGAGAGTGGAAATGAAGAGGCTATGTTTCCTGAATTTCGGGAACAGCTG
GAAGCTCACTTCAATCGACTGCCTCCTAGTTACGGTTTGGATATTAACAT
CGATAGGGTGGAAGATATATTGTTGCATCAAAGGCTTCTTGATTTGGCGA
AGGACCCTGATAAACGACCAATTTTTCATGCTCGTCTTTTGGAGAAGTTC

TGGAGTAGAGCAAAGGCTGATGATGGCCAAACTTTAAGTGTTCTTTCCAC
CCCAAGGCTATCTTGTAATGTTGATAATGATGGATCTGTTCCATCGCTGG
ACAGGCTTGAGGACCTAAATTTGGATGTCAAAAAGAATTCTCTTAAGATG
GAGACGGAAGTTCCTGCAGAAGACTTTCCAAGGAGGCAGGAAGTTCCTCA
AATTCCAATTCATGAAGTAATATTTTCCACCATTGACAGGCCCAAGCTCC
TTAGTCAGCTTTCTGCGTTGCTCTCTGACATTGGACTTAATATCCGGGAA
GCGCATGTGTTTTCGACAACGGATGGATACTCTCTGGATGTATTTGTGGT
GGATGGTTGGCATGCTGAGGATACAGACAGCTTGTGTGAAGCTATGAGAA
ATGCAATTGCCAGAAGTGAGGGATCCTGGTCTGGTTCTTCACATTCTCAG
TCAGCCAAAGAAAAAACTATAGTGCTGCCACCTAAATCTGGAGATTGGGA
AATCGACAGAAGACTATTGAAGATGGGTGAAAGAATAGCATCGGGATCTT
GTGGAGATTTGTATCGTGGATTATACCTTGGTCAGGATGTTGCTATCAAA
GTTCTTAGATCTGAGCAATCAAATGACACTTTGGAAGATGAATTTGCCCA
GGAAGTGGCTATCCTTAGAGAGGTTCAGCATAGAAATATTGTTCGTTTCA
TTGGTGCGTCTACAAGGTCTCCTAATTTCTGCATTGTTACAGAGTACATG
CCTGGAGGGAACTTGTACGATTATTTGCATAAAAATCATATCATCCTGAA
GCTCCCACAATTACTGAAGTTTGCAATAGATGTCTGCCGAGGGATGGAGT
ACTTGCATCAGAATCATATAATTCATAGGGATTTGAAGACGGGAAACTTA
TTAATGGATACTCATAATGTTGTCAAGGTGGCAGATTTTGGTGTTGCTCG
GTTCCAAAACCAAAGAGGTGTAATGACAGCGGAGACCGGTACGTACAGAT
GGATGGCCCCTGAGGTTATAAACCATCAGCCTTATGATCAGAAAGCAGAC
GTTTTCAGCTTTGCAATTGTGCTGTGGGAGCTTGTGACGGCCAAGGTTCC
ATATGACGGCATGACCCCTTTACAAGCTGCACTGGGAGTTAGACAGGGCC
TTCGACCTGATCTTCCCCACGAACACAAACCCTAAATTGTTAGACTTGATG
CAGAGATGCTGGGAAGCCACGCCTGTCAACCGGCCATCTTTTTCTGAGAT
AAGAATCGAACTTGAAGAACTACTCGAAGTTCAGGATTCCTCAGAACAAG
CAAATGGTAGCTGA
>EUC03132-RA [mRNA]
ATGCCGCATAGAACGACTTACTTTTTCCCGAGGCAATTTCCGGACCGTAA
ATTCGATGCATCGTCAAAGTTTCAGTTAGATCACGAGAGCAAAAACGCAA
AAGACAGCGAAAATGACAGGAAAGCATCAAAGCAAAACACAGATGTCACG
GTTGGTGGTAATAGTGCTGCAAGTGCAAAAGATTCAACTGCATCGGATCG
TTTCACGGGTGATAGGATTCACGAGAAGCAATTGGCCGCTTTTGTCAACT
GGTTGGGGAAGAAAAAAGTCGGGGTGTCCGGTCACGTGAAGGTGAGGTTG
GACTCTACAGACGAAGATCGCGAGCACTTGCTTCCACCTGAGGCTGATGA
ACCGCCGGCGACAGAAGTAGTCAGAGATCGTAATTTCGAGCGCGAGATTT
CTTCACAGAAATCGCCGATTGGTAGTGGGAGTAGCTATGGCGGTCCGCCG
GCGGCGACAGAGGTGGTGGTCGGTGCCGGCAGGGATCGAGGGTTCGACCG
GCAGATGTCGTTGCAGAGGCTGTCGAGTGGGAGTAGTTATGCTGGAAGCT
TGTTTTCGGGGACGACTGTTGACGGGAACTGGTCTAGTGGTGTCAAGGAT
TCTCAGATGTCCACTACGAGGGAAGAAGAGGAAGAGAAGACTGATAGTTT
GGCGCAGAGGTCAAAAGAGAGTTACTATTTGCAGCTTACGCTTGCGAAGC
GGCTAACTCAGCAAGCAACGCTTGCTTACGAGCCTATACTTCTGCAGGAG
TGTAAGACGGATGCTCTTGGTGGTTATGATGCCGAAACCGTCTCGTATCG

TCTATGGGTTAGTGGGTCTCTGTCCTATTCTGATAAGATATCAGATGGAT
TCTACAGCATCCTTGGAATGAATCCCTATATGTGGGTGATGTGCAATGAC
TTGGAGGAAGGTACGCGCCTTCCACCTCTAATGGCTCTTAAAGCTGTTGA
ACCCAGTGACACGTCGATGGAGGTAGTTCTTATTGATAGACATGGGGACT
CAAGGCTAAGGGAGCTGGAAGATAAAGCCCAAGAATTGTATTTTGCTGCT
GAAAACACTTTAGTGTTAGCTGAAAAACTTGGCAAACTTGTTGCTGTCTA
CATGGGGGGATCTTTTCCGGTGGAGCGAGGCGATCTCCACATGCGCTGGA
AGTTGGTTAGCAAGAGGCTAAAGGATCTTCAGAAGTGCATTATTCTCCCA
ATCGGCAGCCTCTCCATGGGACTTTGCAGGCATCGTGCCATTCTATTTAA
GAAACTGGCGGACTACATAGGTCTGCCTTGTAGGATTGCTCGAGGTTGCA
GATACTGTGTTGCCGACCATCGATCTTCATGTCTTGTAAAAATAGAGGAT
GACAAAAAGTTCTCCAGGGAATTTGTAGTTGACCTCGTTGGGGAACCAGG
AAATGTTCATGGCCCAGACTCCTCTATCAATGGTGGTCCACTTTCTTCGG
TGCCTTCACCGTTTCAGATATCTCATCTAAAAGAAGTTCAGCAACCTTAC
ATGGATAGTAGTGAGATGTCCTGTGCAATCATAAATTCAAAGCACAAATC
TGCTCTTTCTGAAAGCTCTCCATATTCAGGTGATTGGGAAGAAGGACGTC
AGCGAATGCAGGATATAGGTTGTGTGCATGAAAATCGAGACGAGGCGCTG
TATGGTCTTGTTGGTCAAGCTCATGTACAAAGTGAATCACCTGAAGGTGT
GGCTGTTGTTTCAGCAGCTATATCACAAGGAGAATACTCTGCTGCGAAAG
GAGATAAAATTGTTATTCGACAGGCTTACAGAGAAGAGATAGTTGTATCC
GAAAGTCCTGTTACAAACGCTGCCCTCAATCAATCTCCTGCTGTAATAAT
ATCTGATAAATCAAACCCTATGGAAATCAAGAGTGGAGTTGAGAATCAAG
GGAATTTTAACACTGTAATAATCCCAAGATACTTAAATCTTGAGCCATCT
CTTGCAATGGATTGGTTGGAGATCTCCTGGGAGGAATTGCATATCAAGGA
GCGAATAGGAGCAGGTTCTTTTGGGACTGTTCATCGGGCTGAATGGCATG
GATCGGATGTTGCAGTTAAGGTTTTAACAGTCCAAGATTTTCTCGATGAT
CAGTTGAAAGAGTTCCTTAGAGAGGTTGCGATAATGAAACGTGTCCGGCA
TCCCAATGTGGTTCTCTTCATGGGTGCTGTTACAAAACGTCCGCATCTTT
CAATTGTGACTGAATATTTACCAAGGGGTAGCCTATTCCGCCTCATACAC
AGACCAGCTTCTGGCGAAATTTTGGACCAGAGGAGACGGATACGCATGGC
TCTGGATGTGGCAAAGGGCCTCAATTATCTTCATTGTCTTAGCCCCCCCA
TAGTTCACTGGGATCTTAAATCTCCCAATCTGTTGGTTGATAGAAATTGG
ACTGTGAAGGTATGTGATTTTGGCTTGTCGCGATTTAAAGCAAACACGTT
CATCTCATCAAAATCAGTTGCTGGAACCCCTGAGTGGATGGCGCCTGAAT
TCCTTCGTGGAGAGCCCTCGAATGAGAAGTCTGATGTGTTCAGTTTTGGA
GTGATATTGTGGGAGCTTGTCACCATGCAACAGCCTTGGAGTGGACTTAG
CCCTGCCCAGGTAGTGGGAGCTGTAGCTTTCCAAAATCGGAGGCTTTCTA
TCCCACCCGGCACTTCTCCGGTACTGGCCTCACTCATGGAATCTTGCTGG
GCAGATGATCCCGCCCAGCGTCCCACGTTTGCTAGCATCGTGGATGCACT
GAAGAAGCTGTTGAAGTCGCCAACGCAGCTAATTCAGATGGGAGGCCCAT
AG
>EUC25435-RA [mRNA]
ATGCAGCAAGATCAGCGAAAAAAGAGCTCAAAAGAAATTGAATTTTTCAC
CGAGTATGGTGATGCAAATCGATACAAAATCCTTGAAGTCATAGGGAAGG

GAAGCTATGGAGTTGTTTGCGCAGCCATTGACACTCATACTGGGGAAAAA
GTGGCTATCAAGAAAATAACTGACATATTTGAACATATATCTGATGCTAT
TCGAATCTTGCGCGAGGTCAAGTTGCTAAGACTGCTGCGCCATCCTGATA
TTGTTGAAATTAAACGCATTATGCTGCCACCCTCAAGGCGGGAGTTCAAA
GACATATATGTTGTTTTTGAGCTCATGGAGTCTGACCTTCACCAAGTCAT
CAAAGCCAATGATGACTTGACGCATGAGCATCATCGGTTTTTCCTTTATC
AGATGCTACGTGCATTAAAATATATGCATACTGCAAATGTTTACCATCGA
GATCTTAAACCAAAGAATATATTGGCAAATGCAAATTGTAAACTGAAAAT
ATGCGACTTCGGACTCGCAAGAGTTGCATTCAGTGATACACCAACGACCA
TATTTTGGACGGATTATGTTGCTACGAGGTGGTATAGAGCTCCTGAGCTT
TGTGGATCATTTTTCTCTAAGTATACGCCTGCTATTGATATTTGGGGTAT
TGGCTGTATCTTCGCCGAGGTATTGACGGGGAGGCCATTGTTTCCCGGTA
AAAGTGTTGTTCATCAGTTAGATTTGATAACCGATCTTCTTGGCACACCT
GCAGCAGATACCATTTCGGGAGTTAGAAATGAGAAGGCTAGAAAATACTT
GACAAACATGCGGAAAAAGTACTCTGTGCCGTTCTCTGAGAAATTTCCAA
ATGCAGATCCTCTAGCACTCCCTCTTTTGCGAAGGCTTCTAGCTTTTGAT
CCAAAAGATCGACCAACAGCTGAGGAGGCACTTGCAGATCCGTACTTCAA
GGGCCTGGCCAAAGTTGAGAGGGAACCATCTAGTCAGCCGATCTCAAAGT
TGGAGTTTGAATTTGAGAGGCGTAAGGTGACGAAGGAGGACATTAGGGAA
CTTATTTTCCAGGAAATTCTTGAATACCATCCACAATTGCTCAAGGATTA
CATGGCGGGGAATGAAGGCACTCATTTTCTCTATCCTAGTGCCATTGGTC
AGTTTAGGAGGCAGTTTGCATATCTAGAGGAAAATTTTGGTAAAAGTGGA
CCTGTTATTCCTCCGGAGAGGAAGCATGTTTCACTTCCGAGGTCTACTGT
AAACTCGAGTGTGATCCCTCCTAAAATGCAGCAAAATTCGGGTGCATTTG
ATAACAGGCGAACTTCAGAAGAATCATGTAGTAGTAATGGTGTCCGAGTT
ACGGATGCCATCAACACAAATGCAATGAAGGCCACACGACCACCGCCACC
TCGGGTGCCCACAGCCAAACCTGGAAGAGTGGTGGGGCCGGTTCTACCGT
ACGAAAGTGGCAGGAACAATCCTAGGGTTTTTGTTCGTAATGCGGGCCCA
CCTCCTCCGCCTCAGACAATATCTCCACATTATTGCTTCAGGTCTAATAA
TCAAGAGAAGTCATCATCAGGGCAAGAGGTGAAACATCAGTCAATTTCGG
GCAAACCGGGTCCCGGGATGGGGATGGAAATGAACACCACCCCTTATTAC
CAGACGCAGACGAAGCCATCGCAATTGAACAATCAAATTGCGCTGAATGC
CAAACTGTTTCAGGCACAGTCTCAGTTTGGGGCAGTTGGAGCGGCAGCTG
TTGCTGTTGCCGCGCATAGAGAGGCTGGTGTTGTTCAGTTTGGCCTCTGA
>EUC23670-RA [mRNA]
ATGGCAACTCCAGTTGAGCCTCCGAATGGGGTCAAATCACCGGGAAAGCA
TTACTTCTCCATGTGGCAAACCTTGTTCGAAATTGATACCAAATATGTAC
CCATTAAACCAATTGGGCGGGGGGCCTATGGTATTGTCTGTTCCTCTGTC
AACAGAGAAACGAACGAGAAGGCTGCAATCAAGAAGATAAATAATGCCTT
TGAAAATCGTATCGATGCTCTGAGGACGTTGCGTGAACTGAAGCTTCTGC
GCCATCTGAGGCATGAAAACGTGATTGCTCTAAAAGATGTGATGATGCCC
ATCCACAGAAGAAGCTTCAAGGATGTCTACTTGGTTTATGAACTTATGGA
TACGGATCTGCATCAGATTATCAAGTCGTCTCAAGCACTCACCAACGACC
ATTGCCAATATTTTCTCTTCCAGTTGCTTCGAGGCCTCAAGTATCTTCAC

TCAGCAAACATCCTTCACCGTGACTTGAAGCCTGGGAACCTACTCATCAA
CGCAAACTGTGACCTAAAAATATGCGACTTTGGACTTGCACGGACAAGCA
ACGGTAAGGGCCAGTTCATGACCGAGTACGTTGTCACCCGCTGGTACCGA
GCCCCAGAGCTTCTCCTCTGTTGCGACAACTATGGCACCTCCATTGACGT
ATGGTCTGTTGGTTGCATCTTCGCCGAACTTCTCGGCCGAAAACCAATTT
TCCCCGGAACGGAATGTCTCAACCAGCTTAAACTGATCATCAACATACTG
GGCAGCCAGAGAGAAGAAGACCTTGAGTTCATTGACAACCCAAAGGCCAG
AAAATACATTAAATCACTCCCTTATTCCCTCGGAACTCCTTTTTCCCGTC
TCTACCCCCATGCGCATCCACAGGCAATTGATATTCTTCAGAAGATGCTC
GTCTTTGACCCTTCAAAGAGAATCAGCGTGACAGAAGCACTCCAACACCC
TTACATGTCTCCGCTGTACGATCCGAGTTCCAATCCTCCGGCACAGGTCC
CGATCGACCTCGACATAGATGAGGATTTGAGTGAAGAGATGATAAGGGAG
ATGATGTGGCAGGAAATACTTCACTACCATCCTGAAGTTGCTGCCAGCCA
ATCCTGA
>EUC24332-RA [mRNA]
ATGAAGAAAGGAAACCTAGCTCCTAATCTCAAGCTTTCGCTGCCTCCTCC
CGATGAAGTCTCTAAGTTCCTGACTAAATCGGGAACGTTTATGCATGGTG
ATCTGCTGGTGAACAGAGATGGTGTTCGAATTGCGTCTCAGGCGGAAGTG
GAAATTCCATCCTTAATACACCCATCAGACAATCAGTTGAGCTTAGCTGA
CTTTGATGCTGTTAAAGTCATTGGAAAGGGAAATGGTGGAATTGTGCGAT
TGGTGCAACATAAATGGACGGAGCAGTTTTTTGCTTTGAAGGTTATTCAA
ATGAATATTGAGGAGTCTGCTCGCAAGCAGATTGCGCAAGAACTAAAAAT
TAATCAATCCTCACAATGTCCAAATGTTGTTGTCTGTTACCAGTCTTTCT
ATGATAACGGTGCCATTTCTATAATATTGGAGTACATGGATGGGGGATCT
CTTGCAGATTTCTTGAAAAAGGTCAAAACAATACCTGAACCTTATCTTGC
AGCCATCTCCAAGCAGGTGCTCAAGGGCTTGTGGTATCTCCACCATGAGA
AACATATTATTCACAGGGACTTAAAACCTTCTAATTTGTTAATAAACCAT
AGAGGTGAAGTCAAAATCACCGATTTCGGTGTTAGTACAATCATGGAAAA
CACCTCTGGACAGGCCAATTCTTTTGTTGGCACATACAACTATATGTCTC
CTGAGAGAATTGTTGGCACTAAATATGGCTACAGAAGTGACATATGGAGC
CTGGGGTTAGTTTTGCTCCAGTGCGCAACGGGACATTTCCCATATTCCCC
GCCAGAAGGGGAGGAGGGATGGGTTAATGTCTATGAACTTATGGAAACAA
TAGTTGGCCAACCACAGCCTTGTGCGCCCTCCAATAATTTTTCTCCTGAG
TTCTGCTCTTTTATTAATGCATGTGTGCAGAAAGACCCAAAGGCCAGACA
GTCTGCAAATGAGCTATTGGCGCATCCTTTCATTAGCATGTTTGACGATA
TGGACATTGATCTAGCATCTTACTTCACTAATGTGGGATCTCCACTTGCA
ACTCTATAA
>EUC21870-RA [mRNA]
ATGGAGTGGACTAGGGGTCCTACCATCGGCCATGGTTCTTCCGCCGCCGT
TTATCTTGCCACCGGTGCTTCTGGTGAGCTCTTCGCCGTTAAGTCCGCCC
AGCTCTCTCGCTCTAGCTTCTTGCAGAGGGAGCAGAAGATTCTCTCTCAG
TTGAGGTCTCCTCATGTTGTTGGGTACCTGGGTTTTGATGTTACGTGCGA
GAAAAACGAGCACGTTTACAATCTGTTGATGGAGTATGTCTCCGGCGGTA
CAATTTCCGACGCGGTAAAGAACAGAGGAGGCTCGCTTGATGAAGCGATG

ATCCGGTTATATTCTCATCAAATTCTGCAGGGATTGGATTATCTGCACTC
GAATCGTTTGGTGCATTGCGACATAAAGGGGAAGAATCTTCTGATCGGGA
AAGATTGCGTGAAGATTGCGGATTTGGGATGCGCTAGATTTGTGGAAGAC
GGAGATTCTGCCGCTGAGGCGTCGATGTTTTCCGGTACGCCGGCGTTCAT
GGCTCCGGAGGTTGCGCGCGGCGAGGATCAAGGATACCCAGCAGATGTTT
GGGCTCTCGGGTGCACTTTGATCGAAATGGCAACCGGGTCTAACCCGTGG
CCGGAACTGAACGACCCTGTTTCTGCCCTTTATCGAATCGGGTATTCCGG
CGACTTGCCGGAGTTTCCACGATGGCTGTCGGAAGTTGCTCAAGATTTTC
TGAGCAAGTGTTTGAAGACAAATCCCAAGAACCGGTGGACAGCTGAAGAA
CTACTCAAACACCCGTTTGTTGCCGATTGTGAGCAAAAGCTAGAAACATT
TACAAGAAATTCTCCTACTGCCGTCTTGGATCAATTGTTTTGGGATTCTC
TGGAGGCATCGGAATCATCACCGGATCAAACCCAGATCAGTTCACATTTG
AATTCTCCAGCGGAAAGGATGAGACAATTGATCGGAGATGCTTCTTCATC
AAATTCCGATTTGCCCAAATGGGCAGAGGAAGAAGATTGGATTACTGTTA
GAAACAATCATACAGAGGAAAGCACTCAATTTCCCCAGCTGAACGCCGAT
CTGGAAGATGAGAACAGATTATTTTCTGAAACAGAATCATTATTGTCTTC
AACTGTTAATCAAGAAGAGCTTGTGAGCTCAATGATTGTCTTCAATTAG
>EUC21989-RA [mRNA]
ATGAAGAATTTTCAATGGTTTAAGCAAATCGCGAACAACGGGAAGCTCGA
GAGGCGACTCTCTCTGGGGGAGTACAAACGGGCTGTATCGTGGTCCAAGT
ACCTGGTTTCCTCCGGCGGCGAGATCAAAGGGGAGGGAGAAGAGGAATGG
AGCGCCGACATGTCTCAGCTGTACATTGGGAACAAATTCGCTTCGGGTCG
CCATAGCAGGATCTACAGAGGTGTGTATAAGCAAATGGATGTGGCGATCA
AACTCATCAGTCAGCCAGAAGAAGACGGAGACTTGGCTTCTCTACTAGAG
AAGCAGTTTACGTCGGAGGTGGCTTTGCTTTTCCAGCTGAAGCATCCCAA
TATCATCACTTTTGTGGCGGCATGTAAGAAACCCCCTGTATTTTGCATAA
TCACCGAGTACTTGTCTGGGGGCTCCCTCAGGAAATTCCTCCACCAGCAG
GAGCCGCACTCGCTTCCGCTCAACCTCGTTCTCAAATTAGCTCTCGACAT
TGCACACGGAATGAAATACCTTCATGCAAAGGGCATACTTCACAGAGATC
TCAAATCCGAAAATCTACTCTTGGACGAAGATATGTGTGTGAAGGTGGGA
GATTTCGGTATATCGTGCTTGGAATCTCAGTGTGGAAGCGCTAAAGGATT
CACAGGCACTTACCGCTGGATGGCACCGGAAATGATCAAAGAAAAACACC
ATACAAAGAAAGTCGACGTTTACAGTTTTGGTATCGTGTTGTGGGAGCTT
TTGACCGCCTTGACCCCCTTCGATGACATGACACCGGAACAAGCTGCATT
TGCTGTTTGTCAGAAGAATGCGAGACCACCGTTACCTTGTTCATGCCCAA
AGGCATTACAGCAGTTGATTAACCAATGCTGGTCCAGTAATCCGGTTAAG
AGACCACATTTCAGGGAGATTGTTTCGATTTTGGAAAGTTATGCCGGCTT
GCTGGAGCAGGATCCAGAATTTTTTGCATCTTATGAACCTCCAGATGATC
GTTCTTCTTTACGGTTTGTTCCCAAGTGTATTTCTGCTTGTAGATCTTCT
GCTGCTGCTTTTTAG
>EUC21992-RA [mRNA]
ATGAAGGAGAATAATGATGGGTTCGTGAGGGCGGATCAAATCGATCTGAA
GAGCTTGGATGAGCAGCTCGAGAGGCACCTCAATCGGGCATGGACGATGG
AGAAGAACAAGAAGAGTCACGACTCCGATTCCGGCACCACCGCCGCTGCC

GCTGTCTCAGCTGCTTACTCCACATCCAGAACCTCCAGGCAGAGAGAAGA
GTGGGAGATCGACCCATCCAAGCTCATCATCAAGGGCGCAATCGCTCGTG
GCACCTTCGGCACGGTCCACCGTGGCATCTACGACGGCCACGATGTCGCC
GTAAAACTGTTGGATTGGGGGGAAGAGGGACACAGGACGGATGCGGAAAT
AGCATCATTAAGGGCAGCATTTACACAAGAAGTAGCGGTTTGGCACAAAC
TCGAGCATCCTAATGTAACTAAGTTCATAGGGGCGACAATGGGCTCATCA
GAGCTAAAAATACAAACAGAAAACGGTCTCGTGGGAATGCCGAGTAATGT
CTGTTGTGTTGTGGTCGAATATCTGGCCGGTGGCGCCTTGAAGTCATACC
TCATAAAGAACAGGAGAAGGAAGCTCGCTTTCAAAGTCGTTGTCCAGATG
GCACTTGATCTTGCAAGAGGGTTGAGTTACCTCCACTCTCAGAAGATAGT
ACACCGAGATGTGAAGACAGAGAATATGCTGCTGGACAAAACGCGAACAG
TGAAAATAGCTGATTTTGGGGTTGCTCGTGTGGAGGCCTCTAATCCAAAC
GACATGACCGGAGAGACCGGAACACTTGGTTATATGGCTCCTGAGGTTCT
CAACGGAAACCCTTATAACAGAAAATGTGATGTCTACAGTTTCGGGATAT
GTCTTTGGGAAATATATTGCTGCGACATGCCCTATCCTGACCTTAGTTTC
TCTGAAGTGACTTCTGCCGTTGTTCGTCAGAATTTGAGGCCGGAAATACC
GAGGTGTTGCCCGAGTTCATTGGCGAACGTGATGAAAAGGTGTTGGGACG
CTAATCCAGACAAACGGCCAGAGATGGACGAGGTGGTTTCGATGATTGAG
GCCATTGATACCTCGAAAGGGGGAGGTATGATACCGGGGGATCAACCTCA
AGGTTGTCTCTGTTTTCGCAAGCACAGAGGGCCTTGA
>EUC00773-RA [mRNA]
ATGGAGTGGGTGCGAGGCGAAGTTATTGGTCATGGAAACTTTGGCACAGT
CAATTTGGCGATACCCAGAAGCCAGAATCCTCAAATTCCGCCATTGATGG
CCGTCAAGTCTTGCTCTGTTTCGAACTCTGCTTCGCTCGTCAACGAGAAG
TCGGTGTTGGATGCGCTGAATGGTTGTTCTGAGATTGTTGGTTGCTTCGG
CGATGGTTTGAGCTTCGAAAATGGAGAGAAATTGTATAATGTTTTCTTGG
AGTACGCTTCTGGAGGGGCTTTGGCTGACTTAATCAAGAATTCCGGCAAT
ATCCCGTTGCCGGAAAAAGATGCCCGGAGTTACACGCGGTCAATTTTAAG
GGGAATTCACCATGTTCACAAGAGTGGGTATGTTCACTGCGATATTAAGC
TTCAAAACATTCTGTTATTTTCTTCGGAAGTTGGTTGTGACGATCCGGTG
AAGATCGCCGATTTTGGGTTGGCGAAGAAATCCGGCGAAAAACAGAGTAG
TTCAGGGTGCGAATTGAGAGGCACGCCTCTGTACATGGCGCCGGAGACAG
TAACCGGCGGCGAACAGGAGCCGCCGTCGGATATATGGGCTCTCGGATGC
GTGTTGGCGGAGATGGTCACCGGTTCTCCAGCGTGGCGGTGCCGGGAAAA
ATCCGACGTGGCGGGGCTATTGATGAGGATTGGCGTTGGCGAGGAGTTGC
CAGAGATTCCTGGAAAATTATCGGCGGAAGGAAAAGATTTTCTAGGAAAG
TGCTTTGTGAAAGACCCGAGAAAAAGATGGACGGCTGAGATGCTTCTGAA
TCATCCTTTCGTCGCGAATTCCGATGACGATCGTGAAGCTGTCTCGTCGA
AGGACGCCCAAGAAATCCTTTGGACATCTCCGAGATGTCCCTTCGATTTT
CCCGGTTGGGTCTCCCCCGTGCAGTCGCCGGCCACATCTTTATTTTCTTC
TTCGCCCTCGTCTCTCTCCGACCCATGGTCGGATTCATTTTCCTGGTCGC
CGTCGAATTCTCCGGCAGAAAAGCTACGGCAGCTGACGACGGAGCTGAAA
CCAGATTGGTCCTTTTCGGAAAGCTGGATCACCGTCAGATAA
>EUC17818-RA [mRNA]

ATGCCTCCATGGTGGAAAGCATCAACAAAAGAAACAAAAAAGAAAACAGG
CAAGGAGAGTTTCATCGACACATTACACCGTAAATTTAAAATTCCATCCG
AAAGTAAGTCCACAAGTAGATCAGGAGGGTCTCAAAGACTTGGCAGGGAC
ACAATTTCAGAAAGGGGGTCTCAATCGCGACCACAGTCAAGGTCACCGTC
ACCTTCTAAACACGTATCGAGATGTCAAAGTTTTGCTGAAAGGCCCAAGG
CACAACCACTCCCACTTCCAGGTCTGCGTCCTGCAGGTGTAGTCCGTACG
GATTCTGGTATAAGCGTATCGGCCAAGCAAAGGCAGGAAAGAGGGTCTAG
GCCATCATTATTTCGTCCTCTTCCAAGACCTGCATGCATCCGAAACAAGC
CAGATCCCACTGATTTGGATGGAGATTTGGTCACTGCTTCTTTTTCCAGT
GAAGGCTCGATTGAGATTGATGATCCAGCTGACTCACGTCAACGTAGTCC
CCTGGTGACAGACTCTGCTACTGGGAACAGAACTACCACATGCAGCCCTT
CCAGGATGACGGTTAAGGATCAGACCCCTGTTGTGCAGACGAACTCAGTG
GAGGCATTAAGACCTGCGAATCCTTTGTTCAATAACCACATCTCTCCTTC
GTCTCCTAAAAGGAGACCTCTGAATGGTCACATGACAAATCTGCAGGTTC
CTTGTCATGCGGCTTTCTCAAGTGCTCCAGACAGCTCAATGTCAAGTCCT
TCGAGAAGTCCCATGAGAGCATTTGGCACTGATAAAGTCACAAATTCCAC
TTTCTATGCTGGAAAGCCTTATCCAGATCTTCCTTTACTTGGATCTGGAC
AATGCTCCAGCCCAGGGTCAGGTCAAAATTCTGGGCATAATTCAATGGGA
GGCGATATGTCAGGCCCATTATTTTGGCAGCCCAGTAGGGGTAGCCCAGA
GTACTCTCCAATTCCTAGCCCAAGAATGACTAGCCCTGGACCTAGCTCCA
GGATTCAAAGTGGTGCTGTCACACCACTTCATCCCAGAGCTGGAGGGGCA
GCCTCTGAATCACAGACTAGCTGGCCTGATGATGGAAAACAACAAAGTCA
CAGGTTGCCCCTTCCTCCTGTAACAGTTCCCCATTCTTCACATTTCTCTC
ACCCAAATTCAGCTGCAACATCTCCGTCTCTACCACGGAGTCCAGGAAGG
GCTGAGAACGTTACAAGCCCTGGTTCACGCTGGAAAAAGGGAAAGCTTCT
TGGCAGGGGCACATTTGGACATGTTTATGTTGGTTTTAACAGTGAAAGCG
GTGAAATGTGTGCAATGAAGGAGGTTACATTATTCTCAGATGATGCTAAG
TCTAAGGAAAGTGCAAAGCAGTTGGCACAAGAAATTGCTTTGCTGAGCCG
CTTGCGGCATCCAAATATCGTTCAGTACATTGGGTCTGAAACGGTAGGAG
ATAAACTCTATATATATTTGGAATATGTGTCGGGGGGTTCCATCCATAAG
CTTCTTCAAGAATATGGACAATTTGGTGAATCAGCCATTCGTAGTTATAC
TCAACAAATCTTGTCAGGCCTTGCTTATTTACATGCTAAAAATACCGTCC
ACAGGGATATTAAAGGAGCAAATATCCTTGTGGATCCAAGCGGTCGTGTC
AAATTGGCAGACTTTGGTATGGCGAAGCATATCACAGGGCAGTCCTGTCC
ATTATCATTCAAGGGAAGCCCTTATTGGATGGCACCTGAGGTTATAAAAA
ATTCAAATGGCTCTAACCTTGCTGTTGATGTATGGAGCCTTGGCTGCACC
GTTTTGGAGATGGCTACATCAAAACCACCTTGGAGCCAGTATGAAGGGGT
TGCTGCCATGTTCAAGATTGGCAACAGCAAAGAATTGCCTGCAATTCCTG
ATAACCTCTCAGACGATGGAAAAGATTTTGTGAGGCAGTGTTTGCAGCGC
AATCCACTGCATCGTCCAACGGCTGCTCAGCTTTTGGAGCACCCTTTTGT
TAAAAGTGCTGCACCTTTGGAAAAACCAGTTGTTGGTTCTGAGCCTTCGG
ATCTTCCTGGTGTTTCAAATGCAGTGAAATCTGTGGGTATTGGAAATGCG
AGCAATATTTCGAATGTAGATTTGGAAAGACTTGCTATCCATTCGTCTAG
AGTATCAAGATCTAATTTCCACACCACTGACATCCACATACAGAGAAACA

TATCATGCCCCGTTTCACCAATTGGGAGCCCTCTTCTATATCCAAGGTCA
CCACAACACTTCAACAACGGTAGAATGTCTCCTTCACCTATATCTAGCCC
TCGCACCACCTCGGGTTCATCCACACCTCTAACAGGCGATATCCCATTTC
ATCAACTTAGTCAATCAGCTTACTTGCATGAAGGCTGCTTTGGAAGCTTG
CCAAAGCCCAACAACAATCCCTTTACCAACGGCTTGCCCTATTGCGATCC
AAACCCTAATAATATCTTTCGAGGATCTCATGCTTTTCCATCTTATGAAA
ATGACAGTTTTGGGAAACGATTTGGTAGGACTCCTCACGGGGAGCTTTAT
GATGGACAGTCGATCTTGGCCGATCGTGTTTGTCACCAACTTTTGGGAGA
TCATGTAAAGTTGAATCCGTCCCTGGATTTGAATCCATGCCTGAATGGCT
CCTAG
>EUC21207-RA [mRNA]
ATGAAGAATTTCCTGCGAAAGCTTTACATCAGTGGTGGTGGCTTGCCCGA
TCACCAACACCCACCCGTGATCAGTAATCCTCTTTCAACACCACCTGAAA
CGACGGAAATGCATCCTTCATGTTCAGCTTCTTCTCCTTCGTCTACTTCT
TCGTCTTTATCTTCGTCCGCATTGGTGAGAAGAACTGGAGCGGTTAATTC
GGCGGCAGAAAACTCGAATCCCGCGGACCCCAATTTCTTTGAAGAGGAAT
TTCAAATTCAGCTTGCGCTGACCATCAGTGTCTCTGATCCCGATGCGCGT
GAAGATTCGGAAACGGCACAGATCAATGCTGCCAAGCAAATAAGCTTGGG
GTTTTCGCCCTCAGGATCCGTTGTTGAGTTTCTTTCGCTTCGGTATTGGA
GCCAGAAACTTATAAATTATGATGAGAAAGTGATGGATGGTTTTTATGAT
GTTTATAGAATTACTTCAAATTTAGTCGGACCGGGTAAATTGCCACCATT
GGTCGATCTTGAAGCAATTTCTGTGTCTGAAAATGTTGATTATGAGGTTA
CTTTTGTGTACCGTATGAATGATTCTGAATTACGTCAGCTTGAGGAAAGG
GTGTATCATATGTCCATGGAATGGAAGAATTTGAAAAATGGACTTACTAT
GAGTGGTTTGATTCAAAAACTTGCTGACCTTGTCGTTGATAGAATGGGTG
GCCCAGTTACTGATGTGGAGGAAATGTCAAAAAGGTGGAGATCCAGGAGT
TATAAATTACGGAATTCGCTCCAAAGTATAATCTTTCCTCTTGGCTGTCT
TGGTGTTGGACTTTCACGTCACAGAGCTTTACTTTTTAAGGTACTTGCTG
ATAAGATAGACCTTCCATGTATGCTGGTCAAAGGAAGCTATCATACTGGG
ACTGATGATGGAGCGTTGAACGTTATTAAAGTTGATAACGGAAGTGAATA
TATTATTGACCTAATGGGTGCTCCAGGCACATTAATTCCTGCTGAGATCC
CCAGTAGTCACCGTCAACTTGCTGGGTTAAATATAAGGAGCATTGCTTCC
ATTGCCGATATTGTCCGCGATTCATCTTCAGCTTTTGATAAAGAAAGAGA
AACAGTGACAGTTTCTCCTGACCTAAAGGGTATTGCAAAAAGCTGCAGTT
CAATTTCGGGAAAGGAATCATTAGTTGGAAACCAGATAAAAGTGGACGAC
AGAAGCATTGTGGAGAAAAACTTAACTGATATATTTACCTATGGGAAGCT
TCTTCCTGCACGATATAGATTGAATGAAGGCACACTTTGCAAGAAAGTAT
CAACTGCACAAGAGATGCAAGTTAAAGATGTTTCGGAACATGTAATTAGT
GCAGCAAAACATCCAGAATTCGCTCAAAAATTGCATGCTGTTTTGTTAGA
GCCTCGTGCACCACGACCTCCGGATTTGTTTTCAGAAATAAATCCGAGTG
AACGTGGAGAAGAAATATCCCTCGGAAAGCTTCAATATCATCCGGTACTC
TCCTTGGTAAACAATGAGATGTCTCTTACGTCTACTGGAGTTGATTGGGC
CTTCAGCAGTGTTCATTCTGATAGCAAATGGAAGCCCTCTGTTGATGATT
TGGCAACTGAACAGAAGGGTTTCAAGTGCTCTGCAACTTATCCTGGTAAT

AATTTAATCTCTAATACTACAAGTGATGGATTCATACGGGTAAATAGTGG
ACCTAGTGAAATGATCCAAGTTCATGATGCATCTCTACGTATTGATCAAT
CCGGAATACCCGCAAGAGCTTCAGATTGGGAACAGCCTCATGAAACATTT
GTGCCTGCTGAAGTCAACCCTTTAACAGCGCAGCCTAAAACTGGGCTTTC
CAGTGAGGATAAAATCGTGAAACATCATAATAATACTGGAAAAGACAATA
TATATATTGCAGAACAGTTGGAAATGGATAAAAGTGATCACCTTACTTCT
TGCACTGCTCACAAGAAGATTTACCCAATGCTGCGTGATGTTGCTGAATG
GGAAATTCCATGGGAGGATCTTCAGATTGGAGAGCGGATTGGCATTGGAT
CATTTGGTGAGGTTTATCGGGCTCAATGGAATGGCACTGAAGTTGCGACA
AAAAAGTTCATGAATCAAGATATCACGGGCGATGCACTCGAACAGTTTAA
ATGTGAAGTTGAGATCATGTTGAGGTTGAGACATCCTAATGTTGTTCTTT
TTATGGGAGCAGTTACTCGTCCACCGAATCTCTCTATCTTGACAGAGTTT
CTACCAAGGGGAAGTTTGTTTAGACTGCTTCATCGTCCAAATACTCACAT
TGATGAAAAGAGGCGATTACGGATGGCTCTTGATGTGGCCAAGGGAATGA
ACTACTTACACACAAGCCAACCTATTATTGTGCATCGAGATCTAAAGTCT
CCAAATCTCCTTGTTGATAAGAACTGGGTTGTTAAGGTTTGTGATTTTGG
GATGTCACGTATGAAGTACTGTACTTACCTGTCTTCAAAATCTGCTGCTG
GAACGGCCGAATGGATGGCACCAGAAGTTCTAAGGAATGAACCATCAAAT
GAGAAGTCTGATGTTTACAGCTTTGGCGTAATATTGTGGGAGTTGGCAAC
TTTGCAAATACCGTGGACCGGGATGAATCCAATGCAGGTTGTTGGTGCAG
TTGCATTCCAGAATAGACACCTAATTATTCCAGCTGAGGTTGATCCAATG
GTTGCGGAGATAATCACGGACTGTTGGAGTCCTAACCTACACACTCGACC
CTCTTTTGGACATATCATCGCACGTCTCAGGTGTCTTCATTGTCTGAGAA
GACTGGCTTGTACTGATCAAGAATAA
>EUC17921-RA [mRNA]
ATGGAAATGCCTGGTAGACGATCGAATTACACTTTGCTGAGTCAGATTCC
GGACGATCACCTCCACCAGCAACCGCCGTCGAAGTTCGTTGCCTCAGATT
ACGAGTCTCTCCCCGGAGACCAAAATAAAGGGAAGTGTGATAGAGGTTTC
GTCTGGGACCTGACAGATCATCGAGCAGTTCAGCCGCATAGTCGGATCGG
CACGGTGGCTTTTCCGGGGACCCTAGGCATTCAGAGGCAGTCTAGCGGGA
GTAGCTTCGGCGAGAGCTCCATTTCTGGTGACTTCTATGCGCCGTCTCTG
TCCAATCCGGATGGCTTTGCGTATTTGCATGACGGTGGAGGTGAATTGAG
ATTCAAGGCTGTAGACGGTGGCGGAGGATCGTCTTCATCGAAGAGTTGGG
CGCAGCAAACGGAGGAGACTTACCAGCTCCAGCTGGCCTTGGCGCTACGA
TTGTCGTCCGAAGCTACGCGTGCCGATGATCCCAATTTTTTGGATCCTGT
GCCGGATGAATCGACGTCACGGTTTTCATCGTCGTCTGCTTCAGCAGAAG
CCACGTCACATCGATTTTGGGTAAACAGCTGCTTGTCGTATTCGGACCGA
GTTCCAGATGGTTTCTACTTGATTTCTGGGATGGATCCATACATTTGGAC
TGTATGCACTGATCTTCAAGAGAACGGCCGTATTCCATCAATTGAATCAT
TAAGAGCTGTTGATCCTGGCATTGAATCCTTAGTTGAAGTGATTTTGATA
GATTTTCTTAGTGATCCTAGCTTGAAGGAACTACAGAGCCGTATTCAGAA
TGTGTCTTCAGGTTGTATCACCACAAAAGAGGTTGTTGACCAGCTTGCAA
AGATGGTCTGCAGTTGCATGGGGGGTGCAGCTCTCATTGGAGAAGATGAC
TTGGTTCCTATCTGGAAAAAAATGCAGTGATGATCTAAAGGATTGTTTAGG

ATCTATGGTGCTCCCAATTGGTCGGCTGTCTGTTGGTCTTTGTAGACATC
GTGCTTTGCTATTCAAAGTGCTAGCAGACACAATTGATTTACCATGTCGA
ATTGCCAATGGATGCAAATATTGCACAAGAAATGATGCATCCTCATGTCT
TGTTCGTTTTGGGATTGACAGGGAATATTTTGTTGATTTGATTGCAAAGC
CAGGATGCTTATTTGAGCCTGATTCATTGCTCAACAGTCCATCTTCGATA
TCAATTTCTTCACCGTTGCGCTTCTTCCCACGGGTCAGGCAGGCTGAACC
TACATCTGATTTCAGGTCACATGCCAAACAGTACTTGGCAGACTGCCAAT
CACTTAATCTTTTATTCGATGATTCATCTGCAGGTAATATTATTGATGGA
GATGCTGGAGGTGCCGTATATCCGCAGCTATCAGATAGTAATTATATGGA
TAAAAATAATGTGACTAGTCCAAGCAACAGCAATGAATTTTTACAGTTGC
CTTTGCCTCCAATAAATACCAGGCAAAAGACTCGTGGTAGAGATTCACGA
CCTCATAAGGTATTTAATGCTCAGACCACTATGGATTCAACAAATATAGC
CAAGGATTTAGTCGCCTCGAAACATATGCCACCAATCTGGCATGGAGATG
TCCAACTAAGGGTGTCCCAACCAAAGGTGGACACTAAGGATATGCACTTT
GTTGATAAAAGTCATCTGGTACCAGCTAAACAAAGTAGGCAACTTACCCT
TGATGTCGAAAATTTAGAAATTCCATGGAGTGATCTCGTTCTGAAGGAGA
GAATTGGAGCTGGTTCTTTTGGTACCGTTCACCGTGCTGAATGGAATGGC
TCAGATGTGGCTGTAAAGATTCTCATGGAGCAAGACTTCCACGCAGAGAA
AGTACAAGAATTTTTGAGAGAGGTTGCGATAATGAAGCGATTGCGGCATC
CAAACATTGTGCTTTTTATGGGTGCTGTCACGCAGCCACCAAATTTATCA
ATAGTAACAGAATATCTATCTAGAGGTAGTTTATTTAGACTTTTGCATAA
ACCTGGTGCAAGGGAAGTGTTGGATGAGAGGCGCCGATTGGGTATGGCTT
ATGATGTGGCAAAGGGAATGAATTATCTCCACAAAAGCAATCCTCCCATT
GTTCATCGAGATCTGAAATCTCCGAATCTGTTGGTTGACAAAAAATACAC
TGTGAAGGTCTGTGATTTTGGTCTTTCCCGTCTAAAAGCAAATACATTTC
TTTCGTCTAAGTCTGCTGCAGGAACTCCCGAGTGGATGGCACCAGAAGTT
CTCCGTGATGAACCATCAAATGAGAAGTCAGATGTGTACAGTTTTGGAGT
GATCTTATGGGAGCTTGCAACTTTGCAACAGCCTTGGAGTAATTTAAATC
CAGCTCAGGTTGTTGCTTCTGTTGGTTTCAAGGGTAAGAGGCTTGAGATT
CCACGGAATATAAATACTCAAGTAGCTGCCATAATTGAGGCCTGCTGGGC
GAATGAGCCATGGAAGCGCCCTTCTTTTTTCAATATCATGGAATGCCTGA
GACCGTTGATTAGACCTCCCACGCCACAGCCGGGACATACAGACATGTCA
TTGCTCATGTGA
>EUC17152-RA [mRNA]
ATGATTGATTTGGAATTGCGTCACCTTGAGGAAAGGGTGTACCTCATGTC
CTTGGAGTGGCAGATTTTGGAGAAGGGGCTTACCACAACTGGTTTGATTC
AGAAAATTGCGGACACAGTTGTTGAGAAAATGGGTGGTCCAGTGATTGAT
GCACAAGAAATGATGGAAAGGTGGAGTGCCAGGAGTTATGAATTGAGGAA
TGATTTGAACACTATAATCCTTCCCCTTGGCTGTCTTGATGTTGGACTTT
CACGTCACAGGGCTTTGCTTTTTAAGGTACTTGCTGATAAGATTGATCTC
CCATGTATGCTGGTGAAAGGGAGTTACTATACTGGGACTGACGACGGAGC
TGTGAACTTGATTAAAGTTGATGATGGAAGTGAATATATTATTGATTTAA
TGGGTGCTCCGGGCACACTAATTCCAGCCGAGGTTCCCAGTGGTCACCTT
CAAAATTTGGGGTTAGATATGAGAAGCCATGCATCCATTGCTGACAAAAC

CAAAGATTCATGTCCAATAGCTTTTAAAGGAATTGAATCAGGGATGGTTT
CATCTGCTCTTAGTGGCAGTGCACAATTCAGCCCTTCAAATTCAGATGAA
ACATCTCTCGTAGGCATGCAGCCAAAAAGGGATGACAAAAACACTGGGGG
GAAAAATGAAACTGAGATGTTTAAGGTTGGAAATTTTCTCCCTTTTGAAG
GCTCATCCAGTTTTGGCAAGAATGTGTTGGCTGCAAATGAGTTGAAAGTA
AAAGATGTTTCCAGATATGTCATTAGTGCTGCAAAAAACCCAAATTTTGC
TCAAAAACTTCACGCTGTTTTATTAGAGAGCGGTGCATCTCCTCCTCTGG
ATTTGTTGTTTTCAGAAATAAATCCCAATCATCCGGAAGAACAGAAAGCA
CTTGAGATGGCTCATTGGTTAAATGTAGATGCCATGGTTGACAGGCCTAG
ACTTTTTCAAGAAATCCCTGTGGCAAACAATGATATGTCTCTTATTCCCT
TCACTGGAGGGCATCAGGTCTTCAATAATGTCCAGTATAACATCCCACAA
AATGTTTCTGCCGAGGAAATGGCAGCAAAACAACAGGATTTAGAGTCTGG
TGTGCCTTCTGATACAAGTGAGAGATTTGTGCTTGTAAATAGTGGACCTA
GTGAAATGATCCAGGCAGATGGTGCAGCTGTTCGTAATGATTCTGTTCAG
CCATCTGAAATGGTTGCAAGAGCTTCTGCGTTCTCTCCAACAGGACTGCC
TGAAAATGCCATTGCTGCTGATGATAAAAAATTCTTGAACAATAATGGGG
GAAAGCTTCTCAGCAACATTGAAAAAGACAAAGGACCTTCTGTAAATACT
ACAGGAACAGACAATTCTATTTTCTCCACTCATGAGAGAATTTACCCTGT
GCTGGGTGAGGTTGCTGAGTGGGAAATTCCGTGGGAGGATCTTCAGATTG
GAGAACGGATTGGAATTGGTTCCTATGGTGAGGTTTATCATGCAGAATGG
AATGGGACTGAAGTTGCTGTTAAAAAGTTTATGAATCAAGATATCTCAGG
CGATGCACTGATACAATTTAAATGCGAAGTCGAAATCATGTTGAGGTTGA
GACATCCTAATGTTGTTCTTTTCATGGGAGCAGTTACTCGCCCCCCAAAT
CTCTCTATATTGACTGAGTTCTTACCAAGGGGGAGTTTATTTAAGCTGCT
GCATCGTCCAAATATTCAAATTGAGGAAAAGAGGCGATTGAGAATGGCTC
TTGATGTGGCAAAGGGGATGAATTACTTGCATACAAGTCATCCTGTAATA
GTCCATCGAGATCTGAAGACTCCAAATCTTCTTGTCGATAAGAACTGGGT
CGTTAAGGTTTGTGATTTTGGGATGTCACGCATGCAGCATCATACTTTCC
TGTCCTCAAATTCTGCTGCTGGAACAGCTGAATGGATGGCACCAGAAGTT
CTAAGGAATGAACCCTCCAATGAAAAATCTGATGTTTACAGCTTTGGGGT
GATATTATGGGAGCTTGCAACATTGCAAGTACCCTGGATTGGGATGAACT
CAATGCAGGTTGTTGGAGCCGTTGGTTTTCAGGACAGACACCTCCATATT
CCACTCGACATTGATCCAACCGTAGCACAGATTATAACCGACTGTTGGAA
TTCTAATCCACAGGCTCGGCCCTCTTTTGGACAGATAATAACTCGTCTGA
GGATTCTTCAGCGTTTGAAAAGAGAATCTCAAACAAACCAACAAGAACAA
CAACAACAACAACAACGTTGTTGA
>EUC07535-RA [mRNA]
ATGGACGGCGACGATTCACAGATGAACTCATACAGAATCCTTCTGGATCG
ATTCCAGAGTTTGGAGACGAGTCTGGCAAGGCTCAGGGAGCAGCTCGATG
TGCTGGTGCAGCAAAGAGCTGTTAAAATGAATTTTCCGGGGAAAGGGACG
TCGGATTCCGGTGAAACAACTTCATACGACGGCTGGGAGTACGTTCCGCC
GGCGTTCTTTTCTAGTAGTCCGTACAGGAAGGTGTTGGATCACTTAGGTC
ATGCGGTGCACGTTAGCATACCCGAGTCTGGGGAGATCGTTTATTGGAAT
CATTCTGCTGCAAAACTTTATGGGTACGATTACCATGAAGTGCTGGGGCA

GGGAGACGAAGAACTGCTCACTGACGAAGAACATTACAACTCTGCAAAGA
TGATCTTGCAAAGGTTGAGCTCTGGTCAACCTTGGTCCGGTCAGTTCCCC
TTTAGGAAGAAGTCGGGAGAGATATTTATGGCAATAGCAACCAAAACCCC
ATTATATGAAGATGGTGAGCTTGTCGGGATTGTCACTGTTTCAAGTGATG
CAGATGTATTTAACAGAATAAAGTCGGAAAACGTTGGCCAACCTAGAGCA
CGGAGAATAAATTTCAAAAAGTTTCAGTGCCACCCACAGCAGCAAATTGC
ATCAGTGCCACAGATTGCTGCCTCTGTTTCCAATTTGGCTTCAAAGGTCC
TTTTGCGGAAATGTGGAGATGACACAAGCAACAATTGTACAACTTCTGGG
GAAGAGGCAGAGAGGAATGAAACCAATGTTAAAGACGTAAGATACGATAG
GCCACCAAAAGCACCTGCAACATATTCTGATTACGGTTCGCACGTGGATA
AGACCACAATTGATGCAGATTGCTCTGGAAAGAGAGAGTCTACGCCTGAA
TTTGTTCAACCTTCTAGAATTGCGGCGAAGATCTTATCAAAGCTGAATGT
TAGGAGACTTGCAAACCTTGGAAAAGAGAAGGATGGGGGCATTCAGAAGG
ATGATACTTCAATGCACAAAGAAATAGCAAATGAATCATATTCTGCAGCA
GATTTAAATGCAATGGTTTCAGAACACTGTACTGTTGATGTGGATAGAAG
AACTCAACCTGCTTCTGAAAAGTCAAATGCCACTGGAGAAGGTTTGGCTG
GAACTTTTTGTTGTGATTGTTCTGAGATTCCCAAACCTGCAGGTCAGTCG
CCAAGATCTGAAATCTACTTGCATGCAAACGAGTTCGGGACTGATTTACT
GAATTCAAAACCTTCGGAGATTGAAGATGCAGTGCAGCTTCCAAGCCCTG
GGGAGGGCATTAATAGCAGCGGCAGTTCATCAAGCAAAGGCGATAATGAC
TCTAATGTGATAGTAGATTGTGAAATTTTCTGGGAAGATCTACATTTGCG
CGAAGAGATTGGGCAAGGGTCTTATGCTGTTGTATATCGGGGAATTTGGA
ATGGATCAGACGTGGCTGTTAAGGTTTATTTTGGAAATCAATACGGTGAA
CAGACGTTGCTTGACTACAAAAAGGAGATCGATATAATGAGAAGACTGAG
ACATCCAAATGTGTTGCTGTTTATGGGAGCAGTATATTCACAGGAAAAAC
TAGCTATTGTTACAGAGCTTTTACCCAGGGGAAGCCTTTATAAAACAATT
CACAAGAACAATCAGGCATTAGACCTCAGACGGCGTCTAAGGATGGCTCT
TGATGTTGCTAGAGGTATGAATTATTTGCATCACAGAAATCCACCAATAG
TTCATAGAGACTTGAAATCTTCGAATCTTCTGGTTGATAAAACCTGGAAT
GTCAAGGTTGGGGACTTTGGCTTATCGAAGTTGAAGAATGAAACATTCAT
AACAGCAAAATCAGGAAGAGGAACACCACAGTGGATGGCTCCTGAAGTTC
TTCGGAATGAACCTTCGAATGAGAAGTCAGATGTCTTCAGTTTTGGTGTC
ATCCTCTGGGAACTAATGACTGAATCCGTTCCCTGGATCAATATGAATTC
CTTACAGGTAGTTGGAGTAGTCGGCTTCATGGATCGTCGGTTAGAAGTAC
CAGAGTCTCTTGATCCCCGTGTTTCCTCGATCATCCATGATTGTTGGCAA
AGCAATCCAGATGATCGACCATCATTTGAAGACATTATTGAGCGAATGAC
CGATTTAATCCAATCGGTTACCGGTGTTTTTGCTCGAAAGTGTTCAAAGC
CTTGA
>EUC09614-RA [mRNA]
ATGATCACGGAGCTCTTCTGCTCCGGGAGTTTAAGACAGTACCGGAAAAA
ACACAGGAACGTCGATCTCCGAGCGATCAAGAACTGGGGAAGACAGATCT
TAAAAGGGTTAAACTATCTCCACACCCATGATCCACCGATCATCCACCGC
GATCTGAAGTGCGACAACATTTTTGTGAACGGAAATCAAGCCGAAATCAA
GATCGGCGATCTGGGTTTCGCCACCATCATGAATCAACCCACTTCCCGGA

CCTTGATCGGAACGCCGGAATTCATGGCGCCGGAGATGTACGATGAGGAA
TACGACGAACTCGTCGACGTCTACGCCTTCGGTATGTGCATGTTAGAATT
GATCACCTGCGAGTACCCCTACAGCGAGTGCAGAAACCCTGCTCAAATCT
ACAAGAAGGTGACAACCGGCGTAAAACCGGCGGCTCTGCAAAAACTGAAA
GATCCCCATGTGAAGCTCTTCATCGAGAAATGTCTGGTTCAAGCTTCTCT
CCGGCCATCAGCCATGGAGCTTCTCAAGGACCCATTCTTATCAATGGAGG
AAACCGTGTTTCAGTTTCCGACATTACCCAAATCTTTAAACCCTCCAAAA
ATCGAATCCCATCATTCTGCCATGGAAGCTTGGAGGGTTAAGGACGTGAT
TCAGTTCGGGCTAAAAGGGTTTAAGGTTGATCAGAACTCCATCTCTTTAA
ACCTTAAAATCATCGATTTATCCACCGGTAAGCTCGAGAATTCGACGTTT
GAGTTCTATCTGAGTTCCGACGATGCGCCCTCCATTGCTGTGGAGCTTGT
TAGAGAGAGAATCTTGTCGGTGAATGATTTGCCGGTTGTTTGTGAGATGA
TCGACGGGATGATTCTTGAGTTTGTGCCGGATTGGGAACCTTCATATGGT
TATTACAAGGATTCTGGGTTGAACGAAGCCATGTCTGAAAATTACAGTTC
GGAGATTTCTTTTACAGATTCATCATGCAACGCCATGTCTAAGGAATTTG
GGTATGAGCATTTCTTTATGTAG
>EUC09794-RA [mRNA]
ATGGATTCAAAAACCAATGGAAACGCTCCGAAAACTCAGAGTTTGGGCGA
TCCAGAAGCATTTCGGGAGTTGAAATTGAATGGTGCAGGAAGTGTTAGTG
AGAAAAATATTTCTCAGAGGAGTTCTACTAGCATTAGTAGTCGAGATATG
ATTTTTCGAGCCGATAAAATCGATCTGAAAAGCTTGGATGTTCAATTGGA
GAAGCATTTGAGCAGGGTTTGGTCAAGAAATACGGAGCCCCAAAGGCCTA
AAGAGCTGTGGGAGATTGATCCATCTAAGCTGGATATAAGATATCTTATT
GCTAAGGGTACATATGGTACCGTGTATCGCGCTACCTATGATAACCAAGA
TGTTGCAGTGAAGCTGTTGGACTGGGGGGAGGACGGCATGGCTACAAATG
CCGAAACCGCAGCTTTACGTGCATCGTTTCGGCAAGAAGTCGCTGTTTGG
CACAAGCTTGACCACCCCAATGTTACAAAATTTGTTGGTGCTTCCATGGG
AACATCAAATCTTAAGATACCCTCTAAGAACCCTTCAACCGATGGTTATA
TTGATCTCCCATCAAGGGCGTGTTGTGTGGTTGTTGAATACCTACCGGGT
GGAACATTGAAGAACTTCTTGTATAAGAACCGGAAGAAGAAACTTGCATT
TAGAATTGTGATTCAACTTGCTCTTGATCTCGCTCGAGGGTTGAGCTATC
TACATTCGAAGAAGATTGTACATCGTGATGTGAAAGCTGAAAACATGTTG
CTTGATATTAATAGAACTCTGAAAATTGCGGATTTTGGAGTGGCTCGCGT
CGAGGCTCAAAATCCAAAGGACATGACTGGCGAAACTGGAACTCTTGGCT
ACATGGCTCCAGAGGTTCTGGATGGGAAGCCTTACAACAGAAAATGCGAT
GTCTACAGCTTCGGCATATGCCTATGGGAAATTTACTGCTGTGATCTTCC
ATACCCAAATCTTAGCTTTGCCGATGTCTCATTTGCTGTTGTTCGACAGA
ATTTGAGGCCTGAAATCCCGAGATGTTGCCCTAATTCTTTTGCGAGCGTA
ATGAGGAAATGCTGGGATGTGAATCCAGAAAAACGGCCGGAGATGGAAGA
GGTGGTTGGGTTGTTGGAAGCCATTGATACCAGCAAGGGAGGGGGGATGA
TACCTGATGATCAGGCTGCTCACGGCTGTTTCTGCTTTGCTCCCTCCAGG
GGTCCTTAA
>EUC20951-RA [mRNA]
ATGAATATGAAGCATAAGAGGCTGGAGCGGAAGCTGGATCGGCGGAACGC

GATAAAGAACATCGACTACGACGCTTCGAACTCGTCGACTTCGACTTCGA
CTTCGACTTCGTTCGACGGTCAACCGACTCACCGGACGCGTTCCCTTGAC
ATTTACGCCCACACGGATCGCTCCAGCTTCCGAGTCGACGGAATTGATGG
GTTCGATCAGATTTGCAAATCTCTAGGGCTTTCCGGACCTGAGGACTTCT
CAATTCCTACTGCTGCTTGGGAGGCGCGAAAGGCTCGGTCTCCTTCTGAG
CTTTTTCCGAGCTCTAGGTTTTGCGATTATTACAATTCGGTGAAGGAAAC
GACTGAGGATGGTTTGTCGAATGGTATTCCAGCTAAAGTTAGGACTAGTG
ATGAGTACAAAAAGTGTGAAAACGAGTGTTCTAGGTTAGAGAGCGGGTTG
AATTCAGCTAGGGTTAGGGTGAGTGACGGAGTGAGTCTTGAAGATGATGA
GGCCGTTTACTTAAATTTTCCTACAAATACGGCGGGCGGAAGGTGTGCTA
GGGATGGTGGATGTGGGATTAAGGGTGTCCGACCTCCAGTGCTGGCTCCT
CCCCCGCCCTTGTCACGGGCAGTTGTGGATAATAGGAGATCAACTTGGGA
TTTATTCCGAGATTTCGGTCCTCGAGATGATGAAGATATTGGTTTGCCTT
CAGGTGTGGGGCTAGCAAACTCTTTCAGTGAAACAGATCAGGCAATTGAA
GAAAATAAGGAGGTTGATGGACGGCTAGTTTCTAAACAGGATTTGAATGG
GGAGAGGCTGATAGCCTCTATTGCCTTTACAGAATCTTGTTCAAATTCAT
CGAATGATGATGACGATGATGATTCCTTCAGCATGGTTTTGGAGCCTGAA
TATTCTGTTTCACCTGATAGAATATTCAGGCCTAGTATCAAGTCTTGGCA
GAAGGGTGACTTTTTGGGAAGTGGGTCATTTGGAACAGTTTATGAAGGAT
TCACTGATGATGGGTTCTTTTTTGCTGTAAAGGAGGTTTCCTTGCTTGAT
CAAGGAAGCCAGGGTAAGCAAAGCATTTCTCAGCTTGAACAGGAAATTTC
TCTTCTAAGTCAATTTAAACATCAAAACATCGTGCGATATCTTGGCACGG
AGAAGGATGAGGCCAAGCTCTATATTTTCCTTGAGCTTGTAAACAAGGGT
TCCCTGGCACATCTCTATCAAAAGTATCACTTGAAGGATTCCCAGGTCTC
CGGATACACAAGGCAAATTTTGAATGGATTGAACTATCTTCACAGGCAAA
ATGTTGTTCACAGGGATATAAAATGTGCTAACATATTGGTGGATGTAAGT
GGATCAGTGAAACTTGCAGATTTTGGATTGGCAAAGACAACCACATTCAA
TGATGTAAAATCGTGCAAAGGGACTCCGTTCTGGATGGCCCCAGAGGTTG
TTAAAGGAAGGAACCGTGGCTATGGGCTTATGGCTGACATATGGAGCCTT
GGTTGCACTGTGTTAGAGATGTTAACTGGTCAAATTCCATATTCTCACTT
GGAAGGGCCGATGCAAGCAATGTTTAGAATTGGCAGAGGCGAACCTCCTC
CAATACCTGATTCCTTATCAAATGAGGCCCAAGATTTCATCCTCAAATGC
TTGCAAGTTGACCCAGATCATCGCCCTACTGCTGCTCAACTATTGGATCA
TCCATTTTTAAAGAAGTCATCCTCAGCTTCCCTGAGCCCTGCATCTCCCA
ATTACTATGGTGGGAGACAATAG
>EUC17437-RA [mRNA]
ATGGACGGTTCTGCTCAGCCATCAGACACCGTAATGTCGGAGGCAGCGGC
GGCGCCTCCCCAATCTGACTATCCGCATCCGCATACTCATCCGGCGGCGG
GGATGGAGAACATTCCTGCGACTCTGAGCCACGGTGGCCGCTTCATCCAG
TACAACATCTTCGGCAACATATTTGAAGTCACCGCCAAGTATAAACCTCC
CATCATGCCCATCGGCAAAGGCGCCTACGGCATCGTCTGTTCGGCTTTGA
ATTCGGAGACTAATGAGCATGTGGCTTTAAAGAAGATTGCCAATGCTTTC
GACAACAAAATTGATGCGAAGAGAACTTTGCGTGAAATCAAACTTCTTCG
CCACATGGATCATGAAAATGTTGTTGCAATTAGAGACATAATTCCACCAC

CTCAGAGGGATTCATTTAACGATGTTTATATTGCGTACGAGCTTATGGAC
ACTGATCTCCATCAAATCATTCGTTCAAATCAAGGATTATCAGAGGAGCA
TTGCCAGTATTTCTTATACCAGATCCTTCGTGGGTTGAAGTACATACATT
CTGCAAATGTTTTGCACAGGGACTTGAAGCCTAGCAATCTCCTTCTGAAT
GCAAATTGTGATCTAAAAATATGTGATTTTGGACTAGCTCGTGTCACCTC
AGAAACTGATTTTATGACAGAATATGTTGTTACAAGGTGGTATCGGGCAC
CGGAGCTGTTGTTAAATTCTTCTGATTATACGACAGCAATTGATGTATGG
TCAGTGGGTTGCATTTTCATGGAATTAATGGATCGGAAGCCTTTGTTTCC
TGGTAGAGATCATGTGCACCAGCTACGATTGCTTATGGAGTTGATTGGCA
CCCCATCAGAGGCTGAGTTGGGGTTTCTGAATGAAAATGCAAAGCGATAC
ATCCGGCAGCTTCCTCTCTACCGTCGGCAATCATTCAATGAAAAATTTCC
ACATGTACACCCTGCTGCGATTGATCTCATTGAGAGAATGTTGACTTTTG
ATCCGAGACAGAGAATTACAGTTGAAGATGCACTGGCACATCCCTACCTA
ACGTCCCTCCACGACATAAGTGATGAGCCAGTTTGCACGACTCCATTTAG
CTTCGACTTTGAGCAACATGCCCTTACTGAGGAACAAATGAGGGAATTGA
TATACAGAGAGGCTCTTGCATTCAATCCCGAGTACCAGTAA
>EUC07090-RA [mRNA]
ATGTCAACAGAGTTTCAGTTTGACAAGTTATCCATGGATGATAGAAACGT
TGATAATAACGAGGAACACCTCGTGGATGAAAAGGCCTTGCGCAATTTAG
ACATGGCAAAAGAATGTAAAAACAGTGATATTGTGCAGCATGATTGTAGA
AAGTATACACAAGATAGATTTAAGGGGTCCGACATGAAACTTAAGGAAAC
AGACTGTTCAAGCTCTTCATTTTATCATACTGTAGCTCAGGTTGATCCAG
AAATTGATGATGTTGGTGAATGTGAAATTCCGTGGGAAGATTTGGTCATT
GGTGAGAGGATTGGACTAGGTTCGTATGGAGAGGTCTATCGTGCTGATTG
GAATGGCACTGAGGTCGCTGTGAAGAAATTCCTTGATCAGGATTTTTCTG
GTGCTGCCTTGGCTGAATTCAAGAGAGAAGTACGAATAATGCGTAGATTA
CGCCATCCAAATGTTGTTCTTTTCATGGGTGCTGTAACTCGCCCTCCAAA
TCTTTCAATCATCTCTGAGTTTCTGCCGAGAGGAAGCTTATTTCGGATAA
TTCACCGTCCTAACTGTCAAATGGATGAAAAGCGTAGAATTAAAATGGCT
CTTGATGTGGCTAAGGGCATGAATTGCTTACATACTAGCACACCGACAAT
TGTTCATCGTGATTTGAAGTCACCTAATCTATTGGTGGATAATAATTGGA
ATGTCAAGGTATGTGATTTTGGGTTGTCAAGGTTGAAGCATAATACATTT
TTGTCATCAAAATCAACCGCTGGAACGCCTGAGTGGATGGCACCAGAAGT
TCTCCGCAATGAACCTTCGAATGAAAAGTGTGATGTATATAGCTTTGGTG
TAATTTTGTGGGAACTTGCGACTCTGAGATTGCCATGGACTGGGATGAAC
CCAATGCAGGTTGTGGGTGCAGTGGGCTTTCAAAACCGTCGGCTTGAAAT
TTCCAAGGAAGTTGATCCCTTGGTAGGAAGAATTATTTGGGAATGTTGGC
AGACAGATCCAAACTTGCGTCCTTCGTTTGCAGACCTCACGGTGGCTTTG
AAGTCCTTACAACGGCTTGTCATCCCATTGCATATTGACCAACAAAGCTC
ACCTCTACCCCAAGAAATCTCAGTAAATTTGACTCCTTGA
>EUC07070-RA [mRNA]
ATGCCGCAGGATAGTTCTTCTTCCGAGCAAGAGCCGGACGACTCGGATGC
TGAGTTCGTCGAGCTCGATCCATCTGGTCGTTACGGTCGGTATAAGGATG
TTTTGGGAAAGGGTGCTTTTAAGAAAGTGTACCGAGCATTTGATGAATTG

GAAGGAATAGAAGTAGCTTGGAATCAAGTTAAGGTTGCGGATCTCTTACG
AAATTCGGAAGACATGGAGCGTTTGTATTCAGAAGTTCACTTGCTTAAAA
CCTTGAAGCACAAGAACATTATCAAATTTTACAACTCATGGGTCGACACA
AAAAATGAGAGTATCAATTTCATTACCGAGATTTTCACATCCGGGACGTT
GCGACAATATCGTAAGAAACATAAACATGTCGATTTGAGAGCATTGAAGA
AATGGTCTAGACAAATCCTCGAGGGACTTTCCTACCTTCACAGCCACGAC
CCTCCTGTCATTCATCGGGATCTTAAGTGTGACAATATATTTGTAAATGG
AAACCAAGGTGAGGTCAAAATCGGGGACTTGGGACTTGCTGCCATTCTTC
GCCAGGCTCGTTCAGCTCATAGTGTAATTGGTACTCCTGAGTTTATGGCA
CCAGAATTTTATGAGGAGGAGTACAATGAGCTTGTAGATATTTATGCTTT
CGGAATGTGCTTACTGGAGTTGGTGACATTTGAGTACCCATATATTGAAT
GCACCAATGCTGCTCAAATATATAAGAAAGTCGTATCGGGAATCAAGCCG
GCATCATTGGCTAAAGTGAAGGATCCTGCAGTCAAAGCATTTATAGAAAA
ATGTATTGCACAAGTGTCTGAACGGTTGCCTGCCAAGGAGCTATTGATGG
ATCCTTTTCTGCAGTCAGATGAGAATAACGGAAGCATAGGTCGATTCCTT
CAACCAGATTTATATCATGCAGACAGTATTACTGACCACTTTGAGATCAG
TACAATCCCCAAGGATTCCGTGCCTGATGAAAGTCGAGACTTCACACTGC
AGGGTCAGAGGAAATACCTCAACACGATATTTCTAAAATTACGAATACCA
GAATCTACAGGTTGTTATATTCGGAACATCCACTTCCCATTTGATGTTGA
GGTCGATACGCCAACAGCTGTTGCAAGTGAAATGGTTGAAGAGCTTGACC
TAACGGAGCAAGATGTGCCAACCATTGCTGCAATGATCCAGTCAGAAATT
AGATCATGTATCCCGGAGTGGGTTCCCAGAGAAATTTCTGGTGATCATAC
TTGTAATGAAGTTACAATAGCCGATACATGTGGCTCTGAAGAAGCACAGA
GCAACACTTCACCCTTAACTAATGAGTCTTCTCCCCATTCCGCCGGGTTT
TTTCTAGAACGAATGCCTTCAGGTCGGAAATATTGGTGCAACTCAACAAA
GGGAAGCGGTGGAAGCACCCCACTAAGACAACCGTCTTCAAATTTGTTGC
GTGCTGATTCAGTGATATCTGGAGATAGTTGGGCTGAAGAAAACTATCAT
TCGCCTGAAAGTAGTAAAGAAGGGAGCAGCTTTGTAGAGGAACAGGCTAA
TCTTCCTGCTGATTCACGTTTTAGTGAACCTGATAGTGATTCTGACAAGG
GACATCAACACATTGGAGGAGGAAATAACAGAGCGTTAAACGATACCGTT
TCGGATGATGTCAAAATCATCATGGAAAAACTCGAGAATCTATTGGTTGA
GCAGCGAAGGGAGCTCGATGAACTCAGGCAGAAGCAAGATTTGGCTGTGT
CGGATCTTCTGAAAGGACTTCATCCAGAGGTTCGCAGGAGAGTTTGTAAC
ATTTGTAGTTTGAAGATTTTTGTCCATAAAGTTCCTTGTGCTGGCTGTTC
TATTGAGAATCGCGCCGATCTTTGCTCATTGCGCATGTAA
>EUC03978-RA [mRNA]
ATGAGTTGCAGTGAGAAGAACAGGGCAGGGGAAGATTTGGAGTATGAGAA
ATCTGTGAAAGGGGTTTTGTCGAATGGATCAATCTCAACCGTCCTTCAAC
CCACGATCGACGAGAGAGTATTGGTTGACCCGAAATTGTTGTTTATCGGA
TCGAAAATCGGAGAGGGTGCTCATGGGAAAGTTTATGAAGGAAGGTATGG
AGATCAAATAGTTGCCATAAAAGTACTTAACTGTGGAAGCACTTCAGAAG
AAAGAGCTTCACTCGAGAGCCGTTTTGCCCGCGAAGTTACTATGATGTCT
AGGGTAAAGCATGACAACCTCGTAAAGTTTATTGGAGCTTGTAAAGATCC
TTTAATGGTGATAGTTACGGAGCTATTACCCGGAATGTCGCTACGGAAGC

ATTTAGCAAATATTCGGCCAAAACAATTAGACCTTCATGTTGCTTTGAGT
TTTGCTCTTGACATCGCCCAGGCAATGGAATGCCTGCACGCCAATGGAAT
TATACACAGAGACCTAAAACCCGACAATTTGTTGCTTACTGCCAATCAGA
AGTCGGTGAAACTTGCTGATTTCGGTCTTGCAAGGGAAGAAACAGTGACG
GAGATGATGACTGCTGAAACAGGAACTTACCGTTGGATGGCGCCCGAGTT
GTATAGTACAGTGACATTAAGACAAGGAGAAAAAAAGCATTACAACAACA
AGGTCGATGTTTATAGCTTCGGCATTGTCCTATGGGAAATACTGACGAAC
CGTATGCCGTTTGAAGGGATGTCCAATTTGCAGGCAGCTTATGCTGCTGC
TTTTAAGCAAGAGAGGCCGAGTCTTCCAGAAGATATTCACAACGATCTGG
CATTCATCATACAATCGTGTTGGGTTGAGGATCCCAATATGCGGCCAAGT
TTCAGCCAGATTATCCGAATGCTAAACGCATTTCAATTCACACTTCCTCT
GCCTTCTCCTCCTCCTTCTCCGCCACCGCCCATAAAAGAATCTTCTAATA
ATAATGAGGCGTCGGCGGCTACTAATATTTACAAGAATTAA
>EUC15557-RA [mRNA]
ATGGGTTCGTCGTTAATGGGATCGGAGACCGGAGACGACGACATAGCCGA
GACATCACCGGATGGACGATACATTCGTTACCACGATGTTATCGACCGGT
GTTCTTTCAAGGTCGTTTACAAGGGTTTCGATCAGGACGACGGGAAAGAG
ATTTCTTGGTGTCAAATTTGCATCGACGACTGCGTTATACAGTCGACGGA
GCATCGCCGGGCCTTGTTGTCGGAGGCCAATCTTGTGAAATCTCTGCGGC
ACGGGAACTTGGTCAAGTGTTACCAGTACTGGCTTGACGGAAAGAACAAG
ACGATCAACATGGTTACAGAGTTGTTCAGTTCCGGCAATCTCCGGCACTT
CCGGAAAAAGCATAAGAGCGTCGGCCTGAAGGTGATCAAGAAATGGGGGA
GGCAGATTCTAAGAGGATTACACTATCTCCACACCCAGAATCCGCCGATC
ATCCACTGGGATTTGAAGTGCGACAACATCTTCATCAACGGGAACCACGC
CGAGGTCAAAATCGGCGATCTCGGGCTAGCCACCGTCCTTCACCGGGGAA
CAGAACGGAGCATTATCGGAACGCCGGAATTCATGGCACCGGAGCTCTAC
AACGATGAATACAACGAACTCCTTGACATCTATTCCTTCGGCATGTGTAT
GCTTGAACTGATCACCTGCGAATGCCCTTATAGTGAATGCGAAAATCCGG
CAGAGATATGCGAGAAGGTGACCGCTGGAGTAAAACCTGTTGCGCTGGGA
AAAGTGAAGGATCCGAAATCCAAGGAGTTCATAGAGAAATGTATTATTCC
GTCGTCTCAGAGACCGTCGGCGTTGGAGCTTTTGGAGGACCCGTATCTGT
CTATTGAGAATTTCACCGATTCACCGCAGATTCCGAATGCGATTCCGGCG
AACTTACCGAAATTCGATTCTCAAATCTCCATCGATTTGAGCTCGGAGGA
AGGTAACGATCTCTCCGTCGGAAGCTCTACGGTCGATTCAGTTTCTTCTC
TGGAGATGTGGAGATCGAACGAGACGGTGAAATTGCGGCTGAGTGGCACA
AAGGTCGACCAGAAATCCATCACGTTCAACATGAAGATTGGGAGCTTGTC
GGCTTCGACAACCGAAGTTTGCGAATTCGCGTTTGACGTCTGCGCCGACG
ACGCGCTCACGGTGGCCGTCGAGATGGTGAGGCAGGAGATGCTGTCGTTC
AAGGACGTAGCTCTGGCGGTGGAGCTCATTGACTGCATGCTTCTGGAGCT
TGTACCCAGCTGGAAACCGTCCTATGGGTACTACGTGGGACCAAAGAGCT
GA
>EUC24464-RA [mRNA]
ATGGTATCAGATGGAGGGACTGTGAATTTACTGAGTAGATCTTATGGAGT
GTACAACATTAATGAGCTTGGATTACAAAAGTGCCCCTCATGGCCTGTGG

ATGATGCAGATGAGAGTGAAAAAACATATAGGTGTGCCTCCAGTGAGATG
AGGATATTTGGCGCCATTGGTAGCGGCGCAAGCAGTGTTGTTCAGAGAGC
TATTCATATTCCTACTCATAGGATTATTGCCTTGAAGAAGATTAATATTT
TCGAAAAGGAAAAAAGGCAACAGCTCCTTACTGAGATAAGAACATTGTGT
GAAGCACCTTGTTATCAAGGTCTAGTGGAATTTTACGGGGCTTTTTATAC
TCCTGATTCAGGCCAAATTAGCATAGCTTTGGAGTACATGAACGGAGGGT
CTCTAGCTGATATAATTCGAGTGCGAAAATGCATACCGGAGCCAGTGCTT
TCACATATGGTTCATAAGCTACTGCATGGTTTAAGTTACTTGCATGGCGT
TAGACATTTAGTTCACAGAGATATAAAGCCGGCAAATTTGCTTGTAAATC
TCAAGGGCGAGCCGAAAATAACAGATTTCGGCATAAGTGCAGGCTTGGAG
AATTCAATGGCAATGTGTGCTACTTTTGTTGGAACGGTTACATACATGTC
GCCTGAGCGAATTCGAAATGAGAATTATTCATATCCGGCTGATATTTGGA
GCCTTGGCCTTGCGCTTTTTGAGTGTGGCACCGGAGAATTTCCATATACT
GCTACTGAAGGACCTGTTAATCTTATGTTGCAGATTCTTGATGATCCATC
TCCCTCACCATCAAAGCAACACTTTTCAGCCGAATTTTGCTCATTTGTGG
ATGCTTGTCTGCAGAAAGATGCAGATGCAAGGCCAACAGCAGAGCAGCTT
CTTTCTCACCCATTTATTACAAAGTACAAGGATGCCGGAGTTGATTTAGC
TACATTTGTTTGCGGTGTTTTTGATCCAACACAGAGGATGAAAGAATTGG
CAGATATGCTTACAATACACTATTACTTGCTTTTTGATGGGCCCGATGAC
CTTTGGCAACACACGAAGAACTTATACAATGAACGCTCAATTTTCAGCTT
CTCTGGCAAAGAATCGATTGGCTCGAACGATATATTCACTCGTTTGTCAA
GCATTAGAAGTACATTAGCCGGTGAATGGCCGCCTGAAAAGCTAGTGCAT
GTTGTGGAAAAACTTCAATGTCGTGCCTATGGCCAGAACGGCATTGCGAT
TCGTGTTTCTGGTTCGTTCATCGTTGGGAATCAGTTTCTGATATGTGGAG
AGGGTATCCAAGTGGATGGATTGCCAAATTTTAGGGATCTTTCTGTAAAT
ATCGCTAGCAGGCGAATGGGAACATTTCAGGAGCAGTTTGTTGTAGAACA
GGGGAATGTTATTGGTTGCTATTTCATAGCCAAACAAGAGCTTTATGTTG
CCCAAGTGGAAATGTGA
>EUC24477-RA [mRNA]
ATGGTCATAGTGACTGAACTTCTTCTTGGTGGGACATTGCGCAAGTACTT
GCTGAATATGAGGCCTAGGTGCTTGGATATGCGGGTTGCCATCGGCTTTG
CACTTGACATTGCCCGTGCCATGGAATGCTTACACTCTCACGGGATCATT
CATCGTGATCTAAAACCTGAGAACTTGCTGTTGACAGCTGACCACAAAAC
TGTAAAACTTGCGGATTTTGGCTTGGCAAGAGAAGAATCATTGACAGAGA
TGATGACTGCTGAAACAGGAACCTATCGCTGGATGGCTCCAGAGCTTTAC
AGCACAGTGACATTGAAGCATGGAGAGAAGAAGCACTATAACCACAAGGT
GGATGCCTACAGCTTTGCAATTGTGTTATGGGAGCTCATACATAACAAAT
TGCCATTTGAAGGCATGTCCAATCTCCAGGCTGCATACGCTGCCGCCTTC
AAAAACGCGAGGCCCAGCGCTGAAGATCTACCGCAGGATCTTGCTTTAAT
TGTGACATCGTGCTGGAACGGGGACCCTAATTCTAGGCCAAACTTTGGGG
AAATAATAAAGATGCTGCTGCATTACCTATCAGCAATCCATCCGCCGGAA
CCCATTATTCCTCCAAGGATATTCAAATCGGAGAATGCAGTTTTGCCACC
GGAATCGCCAGGTACAAGCTCCTTGATGGCGATAAGGGACGAGTCCCCCA
AGACGCCTGTAGAAAACGAACCAGGAAGTTTCTTCTTCTGCTTTAACCAC

TGTTACTAA

>EUC24537-RA [mRNA]

ATGGATAACAGCAATACATCAACGCCGCCGGCGGAAGAGCTTCTGAAGAA

GATCCAAGAGCTGGAAGCTGGCCATGCCCACCTCAAGCAAGAGATGTCCA

AGCTCATAATTTCTACAGATCATCGCAAATCGGAACGACAGAGGTCTCAC

TCGATATCCCCGCAGCGGACGGCGGTGCCGTCGCGTAGGAGGGTTGGTGG

GGAAGGAGGATTTGACGGTGGCACGGTGGCGGCGTGGAAGAAGGGTTCCG

CCTCGTTCCGGCATTCGTCGCCGTTGCAGAGGGAGAGCCGTAATCGGGAG

GCCTGTGGTGGCGGTGTTGGTGGCGGCTGTGGTAGTGGCCCGGCGGCCGT

GAAGTTTACTGATAAGCAGTATCTGAATATATTGCAGTCAATGGGTCAAT

CGGTGCATATATTTGATCTCAACCGTCAAATAATTTACTGGAATCGAGCT

GCCGAGCTCCTTTATGGTTACTCTGCAGCAGAAGCTCATGGGAAAGATCC

CATTGAGCTCTTAACAGATAGCCAGGATTATGCTGTTGCTGATGATATAG

TCCGTCGTGTGGCAATGGGGGAGAGCTGGACTGGGCAGTTTCCTGTTAAG

AATAAGCAAGGCGATAAATTTGTAGTAATTGCAACCAACACTCCCTTCTA

TGATGATGATGGAACTCTAGTTGGGATTATTTGTGTTTCGACAGATTCAC

AACCATTCCAAGAAATTAGGGGTCCGTTATCTAGTACAAAGCACGGTGAA

TCAGGCTTCAGCCGGCCAAGAAGCATTGCTTCTGCTAAGCTTGGGCTTGA

CCCTCAACAACCTTTACAAACCGCAATTGCTTCCAAGATTTCAAATTTGG

CTTCTCGGGTGAGCAGCAAAGTTAAGTCAAAGATCAAAACCGGAGAGAAC

AACATGGTTGGTGAAGGTGGGAGTGGAGATAGTCATCATTCGGACCACGG

TTTCTTAGATGATCATAGGGAGGATGCTAACTCAAGTGGAGCTAGCACGC

CGAGAGGAGACATTAACCAATCTCCCTTTGGAGTGTTTTCTCAACATGCG

AGGGATTCTGGTGACGATAGTGAAGGAAAACCGGGGATCTCAAGGATCAT

TAGCTCAAAAGCTGAGGCATGGATGGGTAAGAAAGGCATATCATGGCCTT

GGAAAGGAAATGAAGGAGGTTCAGAAGCAAAAACAACTAGATTTGTTTGG

CCTTGGTTACATAATGAGCAAGAGAATGACCCGGGTCCTCCAAAGAGTTA

TGGTTCTTTTACAAGGCACGAGAATCAGGTGGGTGAAAGTAACCGAAATG

CTAACAATGAGGCTTCAGGGTCGTGGTCCTCATTTAATGTCAATAGTACA

AGCAGTGCAAGTAGCTGCGGAAGTACAAGTAGTAGTGCTCTTAACAAAGT

GGACATGGACTCTGACTGCTTGGATTATGAGATTTTGTGGGAAGACTTGA

CGATAGGTGAACAAATTGGACAAGGATCTTGTGGAACTGTTTATCATGCT

CTGTGGTATGGATCAGATGTTGCTGTTAAGGTGTTTTCCAAGCAAGAATA

TTCAGATGACATGATACTTTCATTCAGACAAGAAGTATCACTAATGAAAA

GACTACGGCATCCAAATGTTCTGCTTTTTATGGGGGCAGTAACCTCACCG

CAGCGTCTCTGTATTGTGACAGAGTTCCTTCCACGTGGTAGTTTGTTCCG

ATTACTACAGAGGAATATATCCAAAATAGATTGGAGACGGCGTGTACAAA

TGGCTTTGGATATAGCTCAAGGCATGAATTATCTTCACCATTACAACCCT

CCAATTATTCATCGTGATTTAAAGTCCTCAAATCTTCTTGTTGACAAGAA

TTGGACTGTGAAGGTCGCTGACTTTGGCCTTTCACGTCTAAAACATGAGA

CATTCCTGACAACAAAAACGGGAAAAGGAACGCCTCAATGGATGGCTCCA

GAGGTTCTTCGCAACGAACCCTCAGATGAGAAGTCGGATATATACAGCTA

CGGAGTGATATTGTGGGAAATTGCAACTGAAAAGATTCCTTGGGAAAATC

TCAACTCAATGCAGGTAATTGGAGCTGTAGGGTTCATGAACCAGCGGCTC

GAGATCCCAAACGATGTCGATCCTCAGTGGGCTTCCTTAATTGAGAGCAC
TTGGCACACCGATCCAAGGTGCAGACCGACATTCCAAGAGATACTGGAAA
AGCTTAAAGATCTGAGGAGACAATTTGCGATTAGAGTTCAGGCAGCCCGT
TCTGCAGCTGGAGAGAGCAGCAGCCGAAAGGAATTGTAG
>EUC09325-RA [mRNA]
ATGCATTGGTGGCAGAGCGCCTTCTCCTCACCCTCACCCTCACCCTCTCC
GTCCTCCTCTCCTCCCTCCAAATCCGACAATAACGTCGTCAACAGGTTCA
ACATCTTCTCTACCCGCGGCCGTCGCTTGAGGTCTCATCATCGCCGTCTC
ACCCGGGCCAAGAAACTCCGGCACCTCTCAGAGAATGAAGCTGAACGTCG
CCTTCCCCCTATTGACCCTTCCGAGCTTTGGAGATTGCCTAGTACTCTCG
AGCATTCTGTTCGATCATCATCCCAAACGACCGCAGCTGCTCCGCAGCCC
CTCCCTTTGCCAGAATTGGGTCAGTTGCTCCGTCGCGACGCTAACTTGGT
GTCATGTTCGAACCCTGGCGATCGTCATCTGCGATCGCCTAAAGACGTTA
CAGGAGGTGGTGGAGACGAGAGAGAAAAATGCAATGTAGTGAACGGCGAT
GGAGTTCCGTCTGGGAGTCGAATAGCTAGCCAAGATGCTCAGAGCAGTAC
AGAGCAACCAAAATCTCGACACCGGAGAAAGTCCCCTCAGCCGGTGAATG
GTGGAGCAAGAAATAAAGGCAACTACAGGATCAGCATTCCAACCAGTGCT
CCGACCAGCCCATTTTCAAGCCCTAGCCCACAAAGAAATACCCCAGATTT
TTACACATCATATTACATGACGCCCCCAATTTTTCAAGTTTGGTCTGCAC
CCGAGTTGCCCCCTTCAGACATGACCGTAGGCCAAGGATTTCCTTATCTA
ATGTCTCCTGAGAAAACTGCATTTAGTGTAGATAGCTCACCCCTTCATAG
TCCAAGGTTAAGTCCTCACCCAACCACCAGGAGTCCTTGTGGACCTCCAT
CACCCCCTACAGCTAGACGTGAAAGTAATAATCAGGTTAACATACACCCC
TTACCCCGTCCTCCGGTAGCTACAATACCTTCACCTCCAGCTCTTGTTCC
CCAAACTACAACTAAAAAGGGGATTAGGCCATTGAAACGTCGATGGAAAA
AAGGAAAGCTTATTGGACGTGGGACATTTGGAAGTGTTTATATTGCCTCC
AATCGGGAAACTGGAGCTTTGTGTGCAATGAAACAAGTAGAGATATTACC
AGATGACCCCAAGTCTGCCGAGTGTATAAAGCAATTAGAGCAGGAAATTA
AAGTTCTCAGCAAGCTAAAGCATCCAAACATAGTCCAATATTATGGTAGT
GAAACGGTTCGTAACCGGTTTTACATATACCTAGAGTATGTTCATCCCGG
TTCAATTACTAAGTATATACATGACCATTTTGGAGCAATTACTGAACCTG
TTGTTCGCAATTTCACTCGACATATTCTTTCGGGGCTGGCTTACTTGCAT
AGCATGAAGACAATTCACAGGGACATTAAAGGGGCTAATTTGCTTGTTGA
TGCATATGGGGTTGTCAAGCTTGCCGACTTCGGAATGGCTAAACATCTGA
CTGGACAAGGAACTAATCTTTCTATGAAGGGAAGTCCTTACTGGATGGCG
CCAGAGGTATTTCTGATAAAACGCAAACTCCAAGAGAATCTTCACCTTCT
GGTTTTAAATATGACTATCCCCAAATATGAATGGCACCAAAGGGAGATCA
CCGAACAACTTGACTGGTGTTTTTTGTTCTCCGGTGAAGCTGGCCGACAA
CCTCCTCATGAGACTTCTGACTTAATGGTGCGTTCTTTTGGATCTTATGC
AAATGCATCCATTTAG
>EUC10175-RA [mRNA]
ATGAAGGAGGGGAGCGATGGGTTCGTGAGGGCAGATCAAATTGATCTGAA
GAGCTTGGATGAGCAGCTCGAGAGACACCTCAGCAGGGCATGGACCTTGG
AGAAGAACAAAAATCAGCACGACTCCGAATCCGCCACCGTCTCTTCCGAT

TCCTCCTCCGCCGTCAACAACAACAACGCCGTCAGGAGGAGACAGGAGTG
GGAGATCGACCCCTCCAAGCTCATAATCAAAAGCGTCATTGCTCGTGGCA
CTTTCGGCACCGTCCACCGCGGCGTATACGACGGCCAAGATGTCGCCGTT
AAACTGCTGGACTGGGGGGAAGAGGGCCACAGGACAGAAGCTGAAATAGC
ATCTCTAAGAGCAGCTTTCACACAGGAAGTTGCCGTTTGGCATAAACTCG
ACCACCCTAACGTAACTAAGTTTATAGGGGCAACAATGGGCTCTTCGACA
CTAAACATACAGACAGAAAACGGTCACATTGGCATGCCAAGTAATATTTG
TTGTGTGGTTGTGGAATATCTTCCTGGGGGTGCCCTTAAATCTTACCTCA
TAAAGAACCGAAGGAAGAAGCTGGCCTTCAAAGTTGTTGTCCAAATGGCT
CTCGACCTTTCACGAGGGTTAAGTTATCTTCACTCTCAGAAGATTGTACA
CAGAGATGTAAAGACGGAGAATATGCTGTTGGACAAATCACGAACTGTAA
AAATCGCAGATTTTGGGGTTGCCCGTGTCGAGGCCTCTAATCCTAATGAC
ATGACTGGAGAAACCGGAACCCTTGGTTATATGGCACCTGAGGTTTTAAA
TGGAAATCCGTACAACAGAAAATGCGATGTGTACAGTTTTGGCATCTGTT
TGTGGGAGATTTACTGCTGTGACATGCCGTATCCTGACCTTAGTTTCTCA
GAAGTCACTTCAGCCGTTGTTCGTCAGAATCTAAGGCCGGAGATACCAAG
GTGCTGCCCGAGCTCCCTGGCGAATGTGATGAAACGCTGTTGGGATGCGA
ATCCAGACAAACGGCCTGAGATGGACGAGGTTGTTTTCATGTTGGAAGCC
ATTGATACATCGAAAGGTGGAGGTATGATTCCGATTGATCAACCTCAGGG
CTGTCTCTGTTTCCAAAAGTACAGAGGCCCGTGA
>EUC10801-RA [mRNA]
ATGGCGAGTCTTTTCAGTAGTGATCATGGACTGGAATCTTCTCGTTACTT
GCTCCGATTTCTTTACCAGTTGGCTTATCAGTCGGTATTTCCGCCTTTTC
CGGAAACTGACCAGGAACCAGATGATTCCGATGTTGCTGAATTCGTTGAG
GTCGATCCTTCTGGTCGTTATGGTCGGTATAAGGAGGTTCTAGGCAAGGG
TGCTTTCAAAAAAGTATACAGAGCATTCGATGAATTAGAAGGAATCGAGG
TTGCTTGGAATCAAGTTAAGGTTGTGGATCTTTTAAAGCATCCAGAAGAC
TTGGAGCGCCTGTATTCAGAAGTTCATTTGCTTAAAACCCTAAAACACAA
GAACATCATCAAATTTTACAATTCGTGGGTCGATTCGAAAAAGGAGAGCA
TCAATTTCATCACCGAGATTTTCACCTCCGGAACACTAAGACAGTATCGA
AAGAAACATAAGCATGTCGATTTGAGGGCATTGAAGAAATGGTCCAGGCA
GATTCTAGAGGGACTCTTTTACCTTCACAGCCATGATCCACCCGTGATTC
ATCGGGATCTGAAGTGTGATAACATCTTTGTTAATGGAAACCAAGGCGAG
GTTAAAATTGGGGATTTAGGACTCGCTGCAATTCTTCGCCAGGCTCGTTC
TGCTCACAGTGTCATAGGTACGCCGGAGTTCATGGCACCGGAGCTCTACG
AGGAGGAATACAATGAACTTGTTGATATCTACGCCTTTGGTATGTGTTTG
CTCGAGTTGGTGACTTTCGAGTATCCATATGTGGAGTGTGCAAATGCTGC
TCAAATCTATAAGAAAGTGACATCAGGAATCAAGCCAGCTTCATTAGCTA
AAGTCAAGGATCCCGCGGTTAGGGCATTCATAGAAAAGTGTATTGTGGAA
GTGTCTGAGCGGTTGCCCGCCAAGGACCTATTGATGCATCCCTTTCTTCA
GTCCAATGATGATAATGGAAGCATAGGTCGATCTTTGCGACCCAATCCCA
ATCACACAGACAATCTGATTGAAACTCACAAGGATTCTTTGCTTGATGGA
AGCCGAGATTTCTCAGTCGAGAGTCAAAGGAAAGACCTCAACACGATATT
CCTTAAACTACGAATAGCCGATTCTTCAGGTCAAATTCGGAACATACACT

TCCCTTTCGACATTGAGGTCGATACCCTGACAGCCGTTGCTAGCGAAATG
GTTGAAGAACTCGACCTAACCGATCAAGACGTTTACGCCGTTGCTTCAAT
GATCGAATTGGAAATTCAGTCATGCATTCCGAATTGGGTTCCCAGAGAAT
TTTCCGGCGATGAAGTCAGCAATGCAGATGGTGGTGGTTCTGCCGCTATC
TCCGCCTTCGAATCTCAGCCCGATGCCTCTCCCCACTCTCCCTCTTCTAA
TGCTTCACCCCGTTCCCCCGGTCTGGTTCTAGAACGACTGCCCTCCGGTC
GGAAATATTGGTGCGACTCGCCCAAGTCTAGACCGGGACCCTCAAACTTA
TCACCCTCTAATTTATCACAGTCCAATTCGATGAACCCTAGAGAAAACAA
TGAATCGCCAGATTCGAATGAGGAGCTTGAGCTTGAGATTGTGGAGAAGC
TTGAGAATCTGTTGGTTGAACAGAGGAGGGAGATGGAAGAACTCAAGATG
AAGCATGAACTAGCTTTATCGGATTTCCTGAGGGAGATTCCTCACGAAAT
TCGTATAGCTGTATGCAAAATGTGCGATCCGAAGGTTTCTGACCATGAAA
AAACCCATTTCGAGACGAGATTTTCGTTGAAAACATGA
>EUC01374-RA [mRNA]
ATGAAGACGATGAAGCCACTGAAGGAGCTGAAGCTCTCTGTTCCAGCTCA
AGAAACACCAATCACCAGCTTTTTAACTGCCAGTGGAACATTTCATGACG
GTGATTTGCTCTTGAACCAGAAAGGTCTGCGATTGATTTCTGAAGAAAAA
GAACCTTGTCCTTCTGAAACTAAAGAAATTGATCTTCAATTCTCATTGGA
AGATCTTGAGACTATCAAAGTCATCGGGAAGGGAAGTGGTGGCGTAGTTC
AACTTGTTCGCCATAAATGGGTTGGAACACTATTTGCCTTAAAGGTAATC
CAGATGAATATACAAGAGGATATTCGAAAACAGATTGTGCAGGAGCTGAA
AATAAATCAAGCATCACAATGCTCTCATGTTGTAGTTTGCTACCACTCTT
TCTATCACAATGGAGCTATCTCTTTGGTGCTTGAATACATGGATCGTGGA
TCGTTGGTAGATGTAATCAGACAACTCAACACAATTCTTGAACCATACCT
TGCCGTTGTTTGCAAACAGGTCTTACAGGGTCTTGTGTACTTACACCATG
AGAGACATGTAATTCACAGGGACATAAAGCCTTCCAACCTGCTTGTAAAT
CACAAAGGTGAGGTAAAAATCACGGATTTTGGTGTAAGTGCAATGCTGGC
GAATTCTATGGGACAGCGAGATACATTTGTTGGAACTTACAATTACATGG
CGCCCGAAAGAATTAGTGGCAGCAGCTATGACTATAAGAGTGATATCTGG
AGTTTGGGCATGGTGATACTTGAGTGTGCTATTGGACGTTTTCCTTACAT
ACAATCTGAAGACCAGCAACGCTGGCCAAGCTTTTATGAGCTGTTAGAGG
CAATTGTGGACAGTCCACCGCCTTCTGCTCCTCCTGATCAATTTTCCCCG
GAGTTCTGTTTGTTCATCTCAGCCTGCATACAGAAGGAACCTACTGATAG
ATCGTCATCTTTGGACCTTTTGAATCACCCATTCATCAAGAAGTTCGAAG
ACAAGGACATCGATCTGGGAATTTTGTTACCGGAAGGGCCTCTACAATGT
CAACATCCCAGAGGACCTTATCTCGAGTCTTAG
>EUC01391-RA [mRNA]
ATGGCTGACGTCAACGCAGCGGCGGGACAGTACCCCGAATTTCCGGCGGT
TCCGGCACACGGAGGTCAGTACGTTCAGTACAACATTTTCGGAAACCTGT
TCGAGATCACCAACAAATACCGCCCTCCGATCATGCCCATCGGCCGCGGT
GCTTATGGAATCGTCTGCTCGGTTTTGAATTCGGAGACGAATGAGATGGT
CGCGATTAAGAAGATAGCCAATGCTTTTGATAATTATATGGACGCCAAGC
GAACGCTTCGCGAGATCAAACTTCTTCGACATTTGGATCACGAAAATGTT
ATAGCTTTACGAGATGTGATTCCCCCACCTCTACGGCGAGAATTTTCAGA

TGTCTACATTGCCACTGAACTCATGGACACTGATCTCCATCAAATCATTC
GGTCTAATCAGGGTCTATCGGAGGAGCACTGCCAGTACTTCTTGTATCAG
CTCCTTCGAGGATTAAAATACATCCATTCAGCAAACGTTATTCATCGTGA
TTTGAAGCCCAGCAACCTTTTGCTCAACGCAAATTGTGATCTTAAAATCT
GTGATTTTGGTCTTGCCCGGCCAAACACAGAGAACGAAGGCATGACTGAG
TACGTAGTAACCAGATGGTATAGAGCACCTGAGCTACTGCTCAACTCTTC
AGATTACACAGCTGCAATTGATGTTTGGTCGGTTGGATGCATCTTCATGG
AGCTCATGAACAGAAAACCTCTGTTTCCTGGTAAAGACCACGTTCATCAG
ATGCGCCTGCTGACTGAGCTTCTTGGAACACCCACCGACGCTGATATCTG
GTTCATGCAAAATGAGGAGGCAAGAAGGTATATCAGACAGCTACCACGTC
ATCCACGGCAGGAGTTGGCAAGAGTCTTCCCACACGTTCATCCTCTGGCA
ATGGATCTTGTTGATAAAATGTTGACATTTGATCCCACTAGAAGAATTAC
AGTTGAAGAAGCATTGGCTCACCCTTATCTAGCGAGATTACACGACATAG
CCGATGAACCAGTCTGCTCTAAGCCATTCTCTTTTGAATTCGAGAAACAA
GGCTTGGAAGAAGAACAAATAAAGGATATGATTTACCAGGAGTCCATAGC
CCTCAATCCAGAGTATGCATAA

>EUC26609-RA [mRNA]

ATGATGATAGAAGATAGCGAGAGCTGTGGGAGCAGAGCGAGCGAATCTTC
GCCGGCGAATACTCGACAACAGAGAAAAAGGCAGGAGGTCTACAATGAGA
TCCTTCGTAGGCTTAGAGAATCTAATAACCAGGAGGCTAAGGAGCCAGGA
TTCGACGATGAGCTTTGGGCTCACTTTAACAGACTTCCTTCTCGGTATGC
ATTGGATGTGAATGTGGAAAGGGCAGAAGATGTTCTCACGCACAAGCGTT
TGCTGAATCTTGCTCATTATACTGCTAATAAAACTGTATTTGACGTTCGC
CTGGTGCAGGTAGCTCCTATATCTGATGGGAATTCACCTGATTCAGTTCA
TTCAAGGTCTCCAAGGAAGGAGGTCGTTCAAAGCATTCATCCACCACCTG
CCTTCGGCTCATCTCCAAATCTTGAAGCCCTTGCACTTGAAGCAAGCAAA
GCTGAACTTCAAGATGGCGACAGTGCTGCAAATACCCATGCAAAGTTTTC
TCGGCCCATGCATGAAATCACATTTTCAGCAGATGACAAACCTAAGCTCC
TCAGTCAGTTGACTTCCTTACTGGCTGAAGTTGGGCTGAACATCCAGGAA
GCGCATGCTTTTTCTACAATTGATGGCTACTCCTTGGATGTTTTTGTTGT
TGATGGTTGGCCGCACGAGGAAACCGTGCTGCTCCGAACCACATTAGAGA
AGGAAATTTTGAAGACTGAGATGCACTCTTGGCCAAATCAACATTCATTT
TCTTCTCTGGGCGAGCAAGAGCAACCTGGGAGGATCAAACAAGAACCTGA
TTTTTTGACAATTCCAAATGATGGTACTGATGTTTGGGAAATTGATCCTC
GATTTCTGAAATTTGAGAACAAAGTTGCTTCTGGATCTTATGGTGATCTA
TACAAGGGTACATACTGTAGTCAAGAGGTAGCTATCAAAGTTCTCAAGGC
GGAGCGTTTAGATTTAGACATGCAGAGGGAGTTTGCCCAAGAAGTATTCA
TCATGCGGAAAGTTCGACACAAGAATGTTGTACAATTCATTGGTGCATGT
ACAAAACCCCCAAGCCTTTGCATCGTGACAGAATTTATGTCTGGTGGGAG
TGTTTACGACTTTCTACACAAACGGAAGGGAATTTTTAAGCTTCCATATT
TACTCAAAGTAGCAATTGATATATCAAGAGGAATGGACTACCTGCACCAG
AACAACATAATCCACAGGGACTTGAAGGCTGCCAATCTTCTAATGGATGA
AAATGAAGTTGTTAAGGTGGCTGATTTCGGAGTTGCCAGAGTGAAAGCCC
AGACCGGAGTTATGACAGCAGAGACTGGGACATATAGATGGATGGCGCCC

GAGGTTATAGAACACAAGCCCTATGATCACAAGGCTGATGTCTTTAGTTT
TGGGGTTGTACTATGGGAATTGCTAACTGGAAAGCTTCCATACCAGTACT
TAACCCCATTACAAGCAGCCGTTGGAGTAGTCCAAAAGGGTCTACGGCCT
ACCATCCCCAAGAACACTCCTCCAACGCTTGCTGAGCTACTTGAGAGGTG
CTGGGAGAAAGATCCGACTTTAAGACCCAATTTTTGTGAGATCATAGAAA
TCCTGCAGCGTTTAGTGAAGGAGGTCGGAGATGAAGCAGAGGAGCGGCGT
AAGGATAAATCATCCGGTGGTTTCTTCTCCGTTCTTAAACGAGCACATCA
TTGA

>EUC24974-RA [mRNA]

ATGCAGGACTTTATCGGTTCGGTTCGTCGATCTCTAGTCTTCAAGCCATC
AGGAGACTTAGACGATGGTGTTGGTGGTGGATTTGGAGGTTTTGTTGAGA
AGATCGGCTCCAGCATTCGGAAATCAAGAATTGGTCTCTTCCAGAAGCCA
CAATTTCAGGCTTTGCCTCCGATTGCAAAGCCCGATAGAGTGAAAGCCAA
AAAGCATGAATCTTCTCCGATCCGGTGGAGGAAAGGTGAATTGATCGGTT
GTGGTGCATTTGGTAGGGTTTATATGGGGATGAATCTCGACTCTGGAGAG
CTACTTGCTGTCAAGGAGGTATCAATTGCAGCGAATAGTGCATCAAATGA
GAAAGCACAGGCTCACATTAGAGAGCTTGAGGAAGAAGTCAATCTGCTAA
AGAATCTCTCACATCCCAACATTGTTAGATACTTGGGAACCGCCAGAGAA
AATGATTCACTGAATATATTGCTGGAATTTGTTCCTGGTGGATCAATCTC
TTCACTTCTTGGGAAGTTTGGATCCTTCCCAGAGTCTGTAATAAGAATGT
ACACAAAACAACTGTTATTGGGACTAGAGTACCTTCACAAGAATGGTATT
ATGCACAGGGACATAAAGGGGGCAAATATTCTTGTTGATAATAAAGGACG
TATTAAACTTGCAGACTTTGGTGCATCCAAGAAAGTTGTTGAACTGGCTA
CCATAAATGGCGCCAAGTCAATGAAGGGTACTCCATATTGGATGGCTCCT
GAAGTCATTCTCCAGACGGGTCACAGCTTCTCTGCTGATATATGGAGCGT
TGGATGTACTATCATCGAAATGGCTACTGGAAAGCCTCCTTGGAGTCAGC
AGTATCAGGAGGTTGCTGCCCTCTTCCATATAGGGACAACTAAATCTCAT
CCACCCATTCCCGATCATCTCTCATTACAGGCAAAGGATTTTTTGTTAAA
ATGTTTACAGAAGGAACCAAACTTAAGGCCCACGGCTTCAGATTTGCTGC
AGCATCCATTTGTCACTGGGTACTACAGAGAGGCTCACCATGTTTTTCGA
TCTTCAGTAACGGAAAGCTTTGGAAACCAGAATGCAGTACTAGGATCTGA
CCTTACAAAATCCATGAATCCCGAGATCAGGACAACTTGTTCTGGTTTGA
AGGATGTTGGTGATATGAGCAGTGTGAGATGCTCCACTATATATCCTGAA
AAATTCTCAGGAATAGGCTCAATGTGGACATCGACCAACAATGATGATGA
CATGTGTTGTATAGATGATAAAGACGATCTCATGATTGGTTCGTCAATGA
AGTTTACTTCAACTTTGCTGTCTCATGATTTAAATAAGAGCTTCAATCCT
ATGAGCGAGCCTAACGATGACGGGCAATGCAAGTTTGATGGAAGTCCAGA
GATTGAGAGGAGTGAAGCAAATTTATATGCCAGTCAGGACAAGGATTTCA
CGTTTATGAACGGGCCATTAGTAGCCGAGGATGAGGATGAATTAATCGAC
TCCAAAATCAAAGCTTTCCTTGATGAGAAGGCTTTAGATCTGAAGAAGCT
GCAAACGCCTCTATATGAAGAATTCTACAATTCGCTAAATGCTATCAGCT
CTCCAAGTCATATCGGAAATGGGGAGAAGGAAAATATTTCGAATAATATG
AATTTGCTGCCACCCAAAAGCAGGTCGCCCAGTCGGGTGCTTAGTAGAAG
ACTCTCTGCTGCTGTTGATTTTGCCTATAATGCCAGTAGTAGCCCGGGAG

GTCGTTCCAAGCGTAATATATCAAATCTTCAAAGTTCTTCCACCAGTTTA
GCTCCTCAAGAAGTTTCTTCACCTCAGATTAATGAAGGGAAAGGTGTTCT
TGGGTCTCAGCAGGAAGCAATTAGTCCAAGTATGAGCTTCTCCGAGAGAC
AAAGGAAATGGAAGGAAGAGCTTGATGAGGAGCTCGCGAGAAAGCGAGAA
ATGCTTCGCCAGGCGGGTAAAGTAAAGACATTGTCTCCAAAGGATCGGAT
TATGAACCGACAGATAGATCGATTAAGAGAAGATCCAACCGGTTTTTGTG
AGGGAAGAAGAACGACGTCATCTCCCAAAGATCAAACCCTAAATTGGCCA
AAGTGA
>EUC24948-RA [mRNA]
TCGCCTCAATCGAACGAAGAGTTTTGCCTATTCATATCCACCGATAAGAA
TATGCTTGACAAAGATTTCTTCACCGAGTATGGTGAAGCATGTCAGTATG
AAATCCTTGAGGTTGTTGGCAAAGGAAGTTATGGCGTAGTTGCAGCTGCG
GTTGATACTCACACTGGAGAGAAGGTAGCTATCAAGAAGATGAAAGATAT
ATTTGAGCATGTTTCAGATGCCACTCGCATTCTTAGAGAAATCAAGCTTC
TCCGGCTACTCCGACACCCAGATATTGTAGAGATAAAGCATATAATGCTT
CCTCCTTCTCCCAGAGAATTTAAAGATATATATGTTGTTTTTGAGTTGAT
GGAATCTGACCTTCACCAAGTAATCAAAGCAAACGATGATCTTACTCCTG
AACATCATCAGTTTTTCCTGTACCAGCTTCTTCGAGGTTTAAAGTATATG
CATACAGCGCATGTGTTCCATCGAGATCTAAAACCGAAAAATATCCTTGC
TAATGCTGATTGCAAGTTGAAGATTTGTGATTTTGGGCTAGCACGCGTAT
CATTTAATGAGTCCCCATCGGCTATTTTTTGGACTGATTATGTGGCAACT
CGGTGGTACCGTGCTCCTGAACTTTGTGGTTCTTTTTGCTCCAAATACAC
CCCTGCCATTGATATTTGGAGCATAGGATGTATATTTGCAGAAATGCTTA
CAGGGAAGCCATTGTTTCCTGGAAAGAATGTGGTGCACCAATTGGATCTC
ATAACCGACTTGCTTGGTTCTCCCGCGGCAGAATCTGTTGCAAGGATTCG
GAATGAAAAGGCAAGGAAATATTTAAGTAGCATGAAGAAGAAAGCACCTG
TTCCTTTATCTCAAAAATTCCCTAATGTTGATCCTTTGGCTCTTCGTTTA
CTTGAGCGTTTGCTTTCATTTGATCCCAAATTTCGTATATCTGCTGCAGA
GGCATTAGCAGATCCTTATTTTCGTAGTGTTTCAAATGTGGAACAAGAAC
CGTCCAGACAACCAATATCGAAATTTGAGTTTGAGTTTGAAAGAAGGAAG
TTGACAGAAGATGATGTTAGAGAGCTAATTTATGGGGAGATCTTGGAGTA
TCACCCGCAGATGCTTCAGGAGTACCTGCGTGGTGTAGATAAGACTAGCT
TCATGTATCCAAGTGGAGTTGATCAATTTAAACAACAATTTGCCCGTCTC
GAGGAGCATTATGGTAAAGGAGAAAAAAGCACTCCACCTCGAAGACAATA
CACATCTTTACCAAGGGAACGGGTCCGTGCACTTGAAGATGATGGTGTGG
ATGAAAGCAGTGATCTTGAAAGGCGCGATGATGTTGCTTTTATGCGTATG
TCCCTTCAAAGCCCCACAACATCACGGGGGAGCAAGGAAGTGGAAATCAA
TGACCCAAGTGCAGTAGCTGCACAAGATGGTCTGGGTGTATCAAAATATA
GTACGCGTGCTATGTCGAGAAGTCCTACCGTTAGCGCTTCCACGTGCGTA
GGCATGCAAAGAAGGCATTTGCAGGGCGCAATCTCGGAGGTTTTCTAA
>EUC20701-RA [mRNA]
ATGTACAAGTCGCGATTGGGTGAACCCGTCGAGTTGGGTTACATAGAAAT
GGACCCTTCTTCTCGATATGGACGATTCAGAGAAATACTGGGTAAGGGTG
CGATGAAGACGGTGTACAGAGCATTTGATGAGGTGTTGGGCATGGAGGTA

GCTTGGAACCAGGTGAAGCTCAACGATGTTTTTCAATCGCCAGATGAACT
GCAGCGTCTCTACTCGGAGGTTCATCTCCTCAACAACCTTGACCATGAAT
CCATCATGAAATTCCACACCTCTTGGATCGACGTTGATCGGAGAACTTTC
AATTTTATAACCGAGATGTTCACCTCCGGCACCCTCAGAGAGTATAGGCA
GAGATACAAGCGAGTGGATATGAGGGCGGTTAAGAATTGGAGTCGACAAA
TCCTGAGAGGACTTGCTTATCTGCACGGACACAGTCCGCCTGTCATACAT
CGAGACCTCAAGTGCGATAACATCTTTGTAAATGGGCATCTTGCTCAAGT
TAAGATTGGTGATCTAGGGTTAGCTGCCATACTTAAAGACTCAAACCACG
CTCACAGCGTTATAGGAACGCCTGAATTCATGGCACCTGAACTGTATGAG
GAGGACTACGACGAGCGGGTGGATGTGTACTCCTTTGGCATGTGTGTTTT
GGAGATGCTCACTTCTGAATACCCTTACAACGAGTGCTCTAACCCGGCTC
AAATTTACAAGAAAGTCACTTCTGGAAAGCGGCCGGAAGCATTTCACAGG
ATTCATGATGGTGAAGCCAGACTGTTTGTGGGTAAATGCTTGGAGAGTGC
TTCAAAGAGGCCATCTGCAGAGGAGCTCTTGATGGACCCTTTTCTTGCTG
CTGATGATGAAGATGAACATGAAGCCATCCCAGATGATCGCAATTCATAT
GTTTTAAATGGAGGAAGAACCAAGGAAGTCCCACAAATATCGATTTTGGG
TGATTCAGGCTCTCCTAAAAGATCAGACATGAGAATCACAGGCACTATAG
ATCCCCAAGATGACACCATATTTCTCAAAGTTCAGATCTCCGATGAGGAT
GGGGAAGGTAGAAACATATACTTCCCATTTGACATATCAAGTGACACAGC
GCTTGATGTAGCCATTGAAATGGTGAAGGAATTAGAGATCAGAGATTGGG
ATTCCTTGGAGATTGCTGAGATGATTGATGAACAGATTTCTACTTTAGTT
CCTACTTGGAAGTGCAACCATTGTCAGCAGCATAGTTTCGGCTACCACTA
CCAAGAAGAAGATGAATATGAAGAAGATAATGATGATGACGACGATAGAA
CCCACCACCCCTTTTACTCTCTCTCCTCCCATTCTTCCTCCCGAGCGTCT
CTCCCGCCTGCCCTTTTCACTTCCTGCCATGATTGGCTTCAAGAGATGAA
GATGGACAGGGATTGTTACAGGCATTGGAATGATGGGAGAGATAAACTGA
CTAGGATACAATCGTTTGTGGATGTTCGTAGGCAGCTGCTGCACCGGACA
CTTGTGGAGGAGATAAACAAGAGGAGGATGTTCAAGACCGTTGGGGCGGT
TGAGAACATCGGTTTTCAAGACCCTTGTCATGGTTTTCACCGCAAGACAA
GGATTTGA
>EUC15935-RA [mRNA]
ATGGCGGGCACCGAAACAGACGCGGGTCGCTACAGGGTGCTGGTAAATCG
GTTTCGGAGTTTGGAAGTGAGTCAGGCAAAGCTCAAGGAGCAGCTCGGTG
TGCTGGCTCAAGAGACAGGCGTTGAAGATTTTTGGGAAGAAGTGGTGCCG
GAGGCCGGTGAGGGGAAGTCGTCGTACCCCGGTTGGAGTCGTATTCCTGG
TGTGTTCTTGTCGGGGATTCCTTACAGGCAGGTGCTGGATTGCATGGGTC
ATGCAGTTCATATTAGAAGAGCCGACTCTGGGGTGATCATTTACTGGAAT
CCTTCTGCGGAAAAGCTGTATGGGTACAAAAAATCTGAAGTACTTGGACG
AACAGTTGAAGAGTTGCTCATTGACGACAACTACTACAGTTCCACACAGG
AAATCATGGAAAGGTTGACAGATGGTCAATCTTGGTCTGGTCAGTTTCCT
TTGAAGAAGAGGTCTGGCAAGGTATTCATGGCGCTTGTAACAAAAACCCC
ATTGTACGAAGACGGTGTGCTTGTTGGTGTTATCACAGTTTCAAGCGACG
CAGCGGTATTTAATAAAATAAACTCAGAAACACTAAGCAAAACTCAGGAT
CAGGATCATGCTAACGTCAACCAATCAGGATTTCAGAGGATGAACCTCAA

AAACATTCAGTGGCCTCCACGAGCGCAAATGGCATCCTCAATTTCTAATC
TGGCTTCAAAAATGCTTTCACAAAAACGTGAAGATTATAGATGGAATGAA
TATACTAATACTGCAACGGAAAGAAAGGAGATTGTAGTCGAGGGCCTGGA
GGCTCAAGACGCAACGAAGGCGACGAACGGCAAAGATGGTGGGGAAAGCT
ATAGGAAGAATGAGTCTACATTTGAGCTTGTTCAACCTTCTAAAATGGCT
GCTAAGGTATTACCTGAGCTGTGTAATGGGAACCTCCAAAAGGACGAGGC
TGGAAGCATTTTACAAAACGGCTCTTCAATTATTGAGCAAATAACAAATG
ATTCATATTTTCCAAGACGTCTACAAGCAACGACTTCAGACTATTGCGTC
GTTGATGCTGATTATAGTAAAAACAACTTCAGTAGAGGAATTTCGCCTGC
TGTGAAAGGAATATGTCCACTTATGAGTGCCAAAATAATTTCCGCCGATA
TTGGACCAGGCAATGCTAAGATCTTCCCCAGATGTCCTAAAGTGCCTCGA
CCTCTAAATCAGTTTCCAAGATCTGGCATCCAGTTATACGCAAACGAGTT
TGAGGCAGATGTAAATGATTCAAAACCCACGGAGATGGAGAATGCATTAC
TATGGCTGCCGGATCCTCAAAAGCAACCCTGCTCAGGGGAGAACAGTGGT
GGCAGCCATGGCATTGCACCAAGCAAGGGTGAAAATGAGTCCAACATGAT
AATAGATTGTGAAATTCACTGGGAAAATTTACAGATCCGTGAAGAGATAG
GGCAAGGTTCATATGCAGTTGTGTATCGTGGAATTTGGAATGGATCGGAA
GTGGCTGTGAAGGTTTATTTTGGGAATCAATACAGTGAAGTGGCCTTACT
TGACTACAGAAAAGAGATAGATATAATGAGGAGACTGAGACATCCAAATG
TGTTGCTGTTTATGGGAGCGGTGTTTTCACAAGATAAGCTCGCCATAGTT
ACGGAGTTCTTGCCCAGGGGAAGTCTTTTTAAAGCACTTCACAAGGGCAA
TCAGAAATTAGACATCAAAAGGCGTTTGAGGATGGCTCTTGATATTGCAA
GAGGTATGAATTACTTGCATTGCATAAATCCACCAATTGTGCACAGAGAC
CTTAAATCTTCGAATCTTCTGGTTGACAGAAACTGGAGTGTGAAGGTCGG
AGACTTTGGCTTATCGAGGTTAAAGATTGCAACGTTCTTGACAGCGAAAT
CTGGGAGAGGAACTCCCCAATGGATGGCTCCTGAAGTCCTACGAAGTGAA
CCTTCAAATGAGAAGTCAGATGTCTTTAGTTTTGGAGTTATTCTGTGGGA
ACTAATGACAGAGTCAATCCCATGGAGCAACCTCAACCACTTGCAGGTTG
TTGGAGTTGTTGGCTTCATGGATCGTAGATTGGACATACCAGAAGGCCTT
GATCCCCGAATTGCTTCCATCATCACCGACTGTTGGCAAAGCAATCCAGA
AGATCGTCCTTCATTTCAAGACATTATACAAGAAATGGGCAACCTTATCT
ACCGTTACGGGACCATTGGATCAAAGAAGTGGCATTGCAGATGA
>EUC17901-RA [mRNA]
ATGGCGATGGAAGACAACGAGAGCTGTGGGAGCAGAGCGGTGGAATCGTC
ACCATCAACGAAGCAGAACCGACAGGAGAGACAGAGGCGCGAGGTTTTTA
ACGAAGTGCTTAGCAGGCTACAAGACTTGAATCACGAAGAAGCTAGGCTT
ACTGGTTTTGAAGACCAGCTCTGGCTTCATTTCAATCGCCTACCTCCTAG
ATATGCACTGGATGTAAATGTGGAAAGAGCGGAAGATGTTCTTACGCATA
AGAGACTGCTATCTTTGGCAGAAGACCCTGCCAATAGACCTGCCTTTGAT
ATTCGCCTTGTGCAGGTTCCTTCTACCTCTGGAAATGCTAGTGATTCTAT
CAATTTGAATTCTCCTGTTGAAGAAGACAATCACAGCTTTCGAAAAGAGG
GAATCCATCCTCCACCTACCTTTGGTTCGTCAGCCAATCTTGAGGCCCTT
GCACATCGAGCTAATGACTCTCATGATGATGATGATGGAGACAGATCAAT
TTCACAGATTCAAAGGCCTATGCACGAGATCACATTTTCAACAGTTGACA

AGCCAAAACTGCTCAGCCAGCTGACTTCGTTACTCTCTGAGATTGGACTT
AACATTCAAGAAGCGCATGCTTTTTCCACCCATGATGGTTTTTCCTTGGA
TGTTTTTGTAGTTGATGGGTGGCCTCATGAGGAAACCATGCAGCTCAGAA
ATGCACTGGAAATGGAAATCTTAAAGACTAAGGGGCAATTTTTTACAAAA
CAACATTCAGTTTCTAGGGTGGGTGACCACTCCAAAGCAGTTTCTGAGTC
TTTTCATAACTTGGTGAAAATCCCAACTGATGGATCTGATGTCTGGGAAA
TTGATGCCAGCATGCTGAAATACGAGAACAGATTTGTGTCTGGGACATTT
TTTGAACTGTATAAAGGAACATATTGTAGCCAGGACGTTGCTATAAAAAA
AAACCTCATGAATGACAGGCTAAATGCAGATATGTTGAAGGAGTTTTCCC
AGGAAGTCTTTATCATGAGGAAAATTCGGCACAAGAATGTTGTTCAATTC
ATCGGTGCATGTACCCAACCTCCAAATCTATGTATTGTCACTGAGTACAT
GTCCAGAGGAAATGTTTACGATCTTCTGCACAAGAAAAAGGGTTTCTTTG
AGCTTCCAACTGTACTCAAGGTAGCTATTGACACTTCAAAGGGGATGAAC
TATTTGCACCAAAATAATATAATCCACAGGGATCTGAAAACTGCTAATCT
TCTGATGGATGAAAACGAAGTTGTAAAGGTTGCTGATTTTGGAATTGCCA
GGGTGCAGGATCATACTGGTGTGATGACTGCCGAAACTGGAACATACCGT
TGGATGGCTCCTGAGGTTATTCAACACAAGCCATATGATGACAAGGCTGA
TGTTTTCAGCTTTGGGATAGTGTTATGGGAGCTTCTAACAGGAGAAATTC
CTTATTCCTACTTATCACCATTACAAGCTGCAATTGGCGTGGTACAACAG
GGTTTAAGACCTACAATTCCTAAGCAAACTCCTCCAAAGCTTGCAGAACT
AATAAAAAAATGCTGGCATCAAGACCCAACTCTAAGACCCAATTTCTCTG
AAATACTCGAGATCTTGCAAAAGATAGCCAGAGAGGTAATATGGCGATGG
CGGTTTCAAATGCCTATTCGTGAATTATGGAGTTGTAAAGTAGAAATTGT
CGGAGTTAAGACAGGCAATAGTTAA
>EUC01494-RA [mRNA]
ATGGCACTAGTCCGAGACCACCGCCGCCACCTTAATCTCAGCCTACCTTT
GCCGGAACACTCCGAGCGCCGCCCTCGCTTCTCCTTCCCCCTCCTCCCTC
CCCCTTCCCTCTCCACCGCCGTTCCCTCCTCCAACAACACCATTACAGCC
GCGGACCTCGAAAAACTCCACGTCCTCGGCCACGGCAACGGAGGGACCGT
CTACAAAGTCCGGCACAGGAAGACCTCCGCCATCTACGCCCTTAAAGTCG
TCCACGGCGACAGCGACCCCGTCGTCCGCCGCCAGATCTTCCGCGAAATG
GAGATCCTCCGCCGGACGGATTCCCCCTGGGTAGTCCAGTGCCACGGCAT
CTTGGAGAAACCCGGCGGCGACATCGCAATCCTGATGGAGTACATGGACG
CCGGGACTCTCGACTCTTCCCTCAAAATCAACGGGACCTTCAGCGAGACA
TGGCTCGCCGGCATAGCGAAACAGGTGCTCAACGGCCTGAGCTACCTCCA
CTCCCTTAAAATCGTCCACAGGGACATCAAACCTTCGAATCTGCTGGTGA
ACCAGAAGGGCGATGTGAAGATTGCCGATTTCGGAGTGGCTAGATTCATG
TACCGTTCGCTGGATCCCTGCAACTCATACGTCGGCACCTGTGCTTATAT
GAGCCCGGAGAGATTCGATCCCGACACTCACGGCAGGAATTACAACGGCT
ATGCAGCTGACATATGGAGCCTGGGATTGACGCTGCTGGAGCTGTACATG
GGCCATTTTCCTTTGCTAACGCCTGGTCAGAGACCCGATTGGGCAACGCT
AATGTGCGCCATATGCTTCGGAGAACCGCCGTGCTTGCCGGATAATAGCG
CGTCGGAGGAGTTTCGGAGTTTCGTGGACTGTTGTCTTCAGAAGGATTCA
AGCAAGAGATGGACGGCCTCTGAGCTGCTGTCGCATCCATTTCTGTTGTT

GCGTCAATCTGATAACTTAACGACAAAATCTGATGACGGCTTATCGGCCG
GAAGTATCTCGTTGGCCGGCGACCTAGAGATCTCCGACGATAATCAATAG
>EUC14352-RA [mRNA]
ATGAAGTCCGGCGGTGGATCTTCACTGCCTGATTCTGAAATTGGAAATGG
AAATGAAAATCGAAATGGGTTTGTGTTTCTCGTGAATAATGAGGTTGGCT
TATCCGTTCGTACTGATCCTCCTCCTGGCTCGGAGGTCGACTACGTGGAG
AAAGACCCCCGAGGGCGATACGTTCGGTACAGTGAGATATTGGGGAAGGG
TGCATTCAAGACTGTCTTCAAGGCATTTGATCAACTTGATGGAATAGAAG
TTGCTTGGAACCGAGTTAAGATTGATGATGTGTTACAATCACCCGAAAAT
TTGGAGAAACTTTATTCAGAAGTGCATCTTCTTAGATCGCTCAAACATGA
AAACATCATCAAAATGTATGATTCTTGGATCGACGAAAAGAAGAAGACCA
TTAACATGATCACTGAGTTGTTCACTTCTGGAAACCTGAGGCAATACCGT
AAAAGGCATAAGAGTGCTGATATGAAAGCTATCAAAAACTGGGCGAGACA
GATTCTCCAGGGGTTGGTCTATCTTCATAGTCAGAACCCGCCCGTTATTC
ACAGGGACTTGAAATGCGACAATGTGTTTGTTAATGGAAATCATGGAGAA
GTTAAAATTGGAGACCTTGGTTTGGCAATTGTTATGCAACAGCCAACTGC
TAAGAGTGTTATTGGAACCCCTGAATTCATGGCTCCGGAGCTTTATGAAG
AGGAATACAATGAACTTGTTGATATATATTCTTTTGGTATGTGCATGCTG
GAAATGGTTACTGCTGAGTATCCCTATAATGAGTGCAAAAATCCTGCTCA
AATTTACAAGAAAGTTACCTCTGGCATTAAGCCTGCTTCTCTTAGCAAGG
TGAATGATCCTGAAGTGAAGGAATTCATTGAGAAATGCATAGTTCCGGCC
TCCCAAAGGCCGCCTGCTAAGGACCTCCTTAAAGATCAATTTCTTCAGCT
ACAAAATCCAATCAACAATCCAATGCAGTTAGCGAATCGCATTCCTAAAT
CGCTAAGTTTATTGGATTGCGGGCCTCTTTCAATGGAAATAGATTCTGAT
TACAACCAGTCGGTTTGTACGGATTCCAACTGTGGAAGTCCACAGTCTCT
GGTCTTGGAATTTCAAAGGATGCATCAAAACAATGAATTCAGATTGAAAG
GCAAGAAACACGATGATAATTCAGTATCACTCACCCTGCGAATTTCATAT
CCATGTGGACGGGTGAAGAATATACACTTTCTGTTCTATCTTGACACGGA
CACTGCAGTCTCAGTGGCGGGTGAGATGGTCGAACAATTGGAGTTGGAAG
ATCACGACGTGGCCTTCATTGCTGAATTCATTGATTTCTTGATTATCAAA
ATTCTACCCGATTGGAAACCTTCATCCGATCACCATCATGATCATGATAG
CCCCCCAACCAACTTAACCACCTCTACTGAAAATCCTCCTCAAGAAGGCC
TATTGGATGGAAATAATTTTGAGTGCACATCTCCTTTATCGGTCTTCTAC
TCCCCTTCCAGTTTGCTAAACTTTGATGATAAAGAATCACAGGCATCAGC
TGGTTCTGAAGACGTTTCGATGAAGAACGAGAAAACATCAGACTGTGTTG
AGTACTTCATCGGGGGGAATATGTCTGAGCTGGAGTTTGGAGATTTGTAC
GAAGAAGAAGACGAGTACAGCAAGATACATGAGGAGGAGGAGGGGAGGAG
TGGTCTTGAGTGCATTGCCTTGAATGGATTCTCACGGTTGGCGTCACTTG
ATTTTGGCAATTTTTCTAAAGTGACAAGCCTGTCGAGCAGCTGTTCTTCA
CCTGATCAAAATCAAAACCATCAGGACGTTGAACTGAAGATGGAGCTTGA
TAAGGTGGAAGCGCAGTATCAGCATTTGTTTCAGGAGTTAACGAGAATGA
AGGAGGAGGAAACCAAGGCTGCAAAGAAGAGGTGGATGGAGAGGAAGAAG
ATATCATCAGTTCTTTGA
>EUC06660-RA [mRNA]

ATGGTGATGGAATATACTGAGAGTTGCAGTAGTAGAGCTTCGGATTCACT
GCCGAAGCAAAGTCGGTTGCGAATACAGAAGGTTGAGGTTTATCGCGAGG
TTCTTCGTAGACTCAAGGAGTTGGACATTGAAGAGGCGAGTCAGCCTGGT
TTTGACGATGAGCTTTGGGCTCATTTCGATCGACTTCCGATTAGGTATGC
ACTTGATGTGAATATTGAAAGGGCACAGGATGTTCTTATGCACAAGAGAT
TGTTGCATATGGCACATGATCCTACAATGAGGCCTGCATTTGAAGTCCGA
TTAGTGCAGGTTTATCCCCAAAGTGATCCGGATTGTGGCGAATCTGTTCA
TTCAAAATTTACTATAGAAAATGATGATCACTACATTGAGTATCCTGGCA
GCCATAGCAAACATCTGCCACCTGCATTTGGTCTGTCACCTGGTTTTGAG
CTTGTACTTGAAGCCAATACACGTCAGCAAAATTGCAGCAGTTCGGGGGG
TGGCAATCTGCAGTTATTGCGGCCTTTGCATGAGATTACAATTTCAGCAA
ATGACAAGCCAAAGCTTCTCAGTCAGTTGACTTCCTTACTTTCTGAAATT
GGGCTGAACATTCAAGAAGCCCATGCCTTTTCCACCACAGATGGTTACTC
CTTGGATGTGTTCCTTGTGGATGGCAGAGCATTCGAGGAAACCGAGCAGC
TTAGGAATGTACTGGTAAAGGAAATTAAAAAAATTGAGAAGCAATCTTGG
TTGAATCCTTACGTCATGGTTTCTGCTGCGGATTTAAGGAAAACTGGGGT
CAATCTTTTCCAGAGCCATGTAAACATACCAACTGATGGGACTGATGTGT
GGGAAATAGATGTCAAGCTTTTGAAAACTGAGAGAAAAATTGCCGTTACA
TCATATGGGGATTTGTATAAAGGTACTTATTGTAGTCAGGATGTGGCTAT
CAAAGTTCTCAAACCTGAATATCTAAATGAAGATGTGCAGAGGGATTTTG
CCCAAGAAGTTTATATATTGAGGAAAGTACGTCACAAGAATGTTGTGCAA
TTTATCGGAGCCTGTACACATCCTCCAAGCCTATGTATTGTGACAGAATA
CATGTGTGGCGGAAGTGTCTACGATTTGTTGCATAAACAAAAGGGCATAC
TCAAGCTCCCAGCTACACTCAAAGTAGCAATTGATGTTTCCAAGGGAATG
AACTACTTGCATCAAAATAATATAATTCACAGAGACCTTAAGGCTGCCAA
CCTTTTGATGAATGAAAATGAAGCTGTTAAGGTTGCGGACTTTGGCATCG
CCAGAGTGAAGCCTCAGACTGGCGTGATGACTGCAGAAACAGGCACATAC
CGTTGGATGGCTCCGGAGGTAATTGAACACAGGCCATATGATCACAAGGT
TGATGTTTTCAGCTTTGGAATCACTCTTTGGGAGCTTTTAACAGGGAAGC
TTCCGTATGAGCACTTAACCCCATTACAGGCAGCACTTGGAGTGGTCCAT
AAGGGTCTAAGGCCTACAATACCAAGGCAAACTCATCCCAAGCTTGTGGA
ATTGCTGGAGAGATGTTGGCAGAGAGACCCAACATTGAGACCGGAGTTTT
CTGAAATTATTGAAATTTTGGAAAACATAGCTAAGGCCATGAAGGTTGTT
GAAGGTGAGAGCAATAGTAAAAAGAGAAGGAATTACCTAGAATTTTGTTG
A
>EUC00181-RA [mRNA]
ATGTCGGTGGAATCGAGCTCTGCTTCAGCTGACCATGGCCACATCAAAGG
AGTACCCACTCACGGCGGTCGTTACGTTCAGTACAATGTCTACGGCAACC
TCTTCGAGGTCTCCCGAAAGTATGTGCCTCCGATTCGGCCCGTCGGTCGT
GGTGCGTACGGAATCGTTTGTGCTGCTATGAACTCGGAAACGCGAGAAGA
AGTTGCAATCAAGAAGATCGGTAATGCGTTTGACAACAGGATAGACGCCA
AAAGGACTCTACGGGAGATTAAGCTTCTTCGCCACATGGATCATGAAAAT
GTAATTGCTATCAAAGACATAATACGCCCTCCGCAGAAGGAAAACTTCAA
TGATGTCTACATAGTTTATGAGCTAATGGATACTGATCTTCATCAGATTA

TCCGATCCAACCAGCAGCTGGCTGATGATCATTGTCGGTACTTCCTTTAC
CAAATTCTAAGAGGACTCAAATATGTTCATTCCGCAAATGTCTTGCATCG
TGACCTAAAACCTAGCAATTTGCTACTTAATGCGAATTGTGATCTGAAAA
TTGGAGACTTTGGACTTGCGAGAACAACATCCGAAACCGATTTCATGACT
GAGTACGTTGTTACTCGCTGGTACCGGGCCCCGGAATTGCTCCTTAATTG
CTCAGAATACACTGCAGCTATTGATATTTGGTCAGTGGGTTGTATTCTCG
GCGAAATCATGACCCGACAACCCCTCTTCCCCGGCAAAGACTATGTTCAT
CAGCTAAGGCTTATCACTGAGCTCATAGGTTCGCCCGATGATGCAAGTCT
TGGGTTTCTCCGTAGCAATAATGCCCGAAGATACGTTAGGCAGCTTCCTC
AGTACCCAAGGCAACGGCTCGTTACTAGATTTCCTAATTCGTCTCCTAGT
GTTGTTGATCTTCTTGAAAGAATGCTAGTCTTTGATCCAACCAAGCGTAT
TACAGTTGATGAGGCTCTCTGCCACCCGTACTTGGCACCACTCCACGATA
TCAACGAGGAGCCGGTTTGCCCGAGGCCTTTCAGCTTCGACTTCGAGCAT
CCTTCTTGCACCGAAGAGAACATCAAGGAGCTCATCTGGAAGGAATCCGT
TAAATTCAATCCCGATCCAAGTCATTGA
>EUC20904-RA [mRNA]
ATGGATGAAGAGGCTAATTCTTGGTTAAGGAGGGCGAAGTTCTCTCATAC
GGTTTGTCACCGTTTCGACGTAGCGAGATTATCCTCCGTTCCTCTATCGA
TCCAGCAAGATCGATTTTTGGATTTGGGTTCGAAATCTAGGACTTTTGGC
CTCGATTTGGGGTCAAAATCGGGCGAAAAAATACTTGAATTTCGTCGAAA
TTCAACTGTAAACAAGCAAAGGGCGGTTTCTCCTCTCCCAGAGACAAAAA
TTCCTACTACTTTTAAGGAAGCCCGATCCGATCGAAAACGATTCTCGACT
CCTCTTCCCCGGAGGAAACAACCAGATAAGGGAATTTCCAAGGGTAAATC
GATCAATTCACATTCGGATTTGAACATAAATACGAGTCCGCTCAAACATT
TTTCTTCATTGAAAATCGGTGAAAAAGGGAAGGGGGGGAATAGGGATTCG
TCGTGGGCTAAGTACTTTGATTATGGCGGAGGGGGAAGGGTCAGGGCGTT
GGATGCGGCGGACGAGCACACGGTGAATCTCTCTAAGCTTTTTCTAGGGC
TTAGATTCGCCCATGGAGCCCACAGTCAGCTATATCACGGGATATATAAC
GATGAGGCGGTGGCCGTGAAGATAATTAGGGTTCCTGATGATGACGAAAA
TAACGGATTAAGCTATCGGTTGGAGAATCAATTCAATAGGGAGGTCACAC
TCTTGTCTCGTCTCCACCACCCGAATGTGATCAAGTTTGTGGCGGCGTGT
CGAAAACCGCCGGTTTTCTGTATAATCACTGAATATCTATCCGAGGGTTC
TTTGAGGGCATATCTACACAAGCTCGAGCATAAACTTCTACCATTAGAGA
AGCTCATAGCGATGGCGTTAGACATTGCTCGGGCAATGGAATATATTCAT
TCGCAAGGCGTTATTCATCGTGATCTTAAGCCCGAAAACATACTCATAAA
TCAAGCTTTCCGATTGAACATTGCTGATTTCGGAATAGCTTGTGAGGAGG
CTTATTGCGATCTCTTGGCCGATGATCCCGGAACTTACCGGTGGATGGCG
CCGGAGATGATCAAGCGGAAATCGTACGGCCGGAAAGTTGATGTTTACGG
ATTTGGGCTTATTTTATGGGAAATGGTTGCCGGAAATATCCCATACGAGG
AAATGACGCCGATTCAAGCCGCTTTTGCCGTCGTGAATAAGAATTTGAGG
CCGGCTATTCCGGTGGGTTGCCCGCCCGCGATGAGCGCTTTGATCCAGCA
ATGTTGGGCCTTGAACCCGGAAAAAAGGCCCGAATTCTGGCAGGTAGTGA
AGGTTCTAGAACAATTCGAATCCTCCGTGGCCCGCGATGGAACCCTTAAT
CTTGTGCCGAATTCAACATGTCAAGATCACAAGAAAGGGCTTCTTCATTG

GATTCATAAGCTCGGGCCCGTTCATCCCGATGCTTCTGGGCCCGTTCCCA
AGCCTAAATTCGCCTGA
>EUC18639-RA [mRNA]
ATGGAATGCAGGGATATGGCAACTCCAGTTGAGCCTCCGAATGGGGTCAA
ATCACCGGGAAAGCATTACTTCTCCATGTGGCAAACCTTGTTCGAAATTG
ATACCAAATATGTACCCATTAAACCAATTGGGCGGGGGGCCTATGGTATT
GTCTGTTCCTCTGTCAACAGAGAAACGAACGAGAAGGCTGCAATCAAGAA
GATAAATAATGCCTTTGAAAATCGTATCGATGCTCTGAGGACGTTGCGTG
AACTGAAGCTTCTGCGCCATCTGAGGCATGAAAACGTGATTGCTCTAAAA
GATGTGATGATGCCCATCCACAGAAGAAGCTTCAAGGATGTCTACTTGGT
TTATGAACTTATGGATACGGATCTGCATCAGATTATCAAGTCGTCTCAAG
CACTCACCAACGACCATTGCCAATATTTTCTCTTCCAGTTGCTTCGAGGC
CTCAAGTATCTTCACTCAGCAAACATCCTTCACCGTGACTTGAAGCCTGG
GAACCTACTCATCAACGCAAACTGTGACCTAAAAATATGCGACTTTGGAC
TTGCACGGACAAGCAACGGTAAGGGCCAGTTCATGACCGAGTACGTTGTC
ACCCGCTGGTACCGAGCCCCAGAGCTTCTCCTCTGTTGCGACAACTATGG
CACCTCCATTGACGTATGGTCTGTTGGTTGCATCTTCGCCGAACTTCTCG
GCCGAAAACCAATTTTCCCCGGAACGGAATGTCTCAACCAGCTTAAACTG
ATCATCAACATACTGGGCAGCCAGAGAGAAGAAGACCTTGAGTTCATTGA
CAACCCAAAGGCCAGAAAATACATTAAATCACTCCCTTATTCCCTCGGAA
CTCCTTTTTCCCGTCTCTACCCCCATGCGCATCCACAGGCAATTGATATT
CTTCAGAAGATGCTCGTCTTTGACCCTTCAAAGAGAATCAGCGTGACAGA
AGCACTCCAACACCCTTACATGTCTCCGCTGTACGATCCGAGTTCCAATC
CTCCGGCACAGGTCCCGATCGACCTCGACATAGATGAGGATTTGAGTGAA
GAGATGATAAGGGAGATGATGTGGCAGGAAATACTTCACTACCATCCTGA
AGTTGCTGCCAGCCAATCCTGA
>EUC21330-RA [mRNA]
ATGGCAGTGGAATCGAGCTCTGCTTCAGCTGAACATGGACATATGAGAGG
CGTCCCGACTCACGGTGGCCGCTACATGCAGTACAATGTGTACGGCAACC
TCTTCGAACTTTCCAGAAAGTACATCCCTCCGATTCGGCCAATTGGTCGT
GGCGCGTATGGCATGGTTTGTACCGCTGTCAACTCAGAGACACGAGAAGA
AGTTGCTATCAAGAAGATTGGCAATGCATTTGACAATATAATTGATGCCA
AAAGGACACTACGGGAAATCAAGATTCTTTGCCACATGGATCACGAAAAT
ATTGTTGCGATCAAAGACATCATACGGCCTCCACAAAAGGAAAACTTTAA
TGATGTATACATCATTTCTGAGCTAATGGACACTGATCTTCATCAGATAA
TTCGATCCAACCAACCATTGACTGATGATCACTGTCGGTATTTCCTCTAC
CAACTACTTCGAGGACTCAAATATGTTCATTCAGCAAAAGTGTTGCACCG
TGATTTAAAACCCAGCAATCTCTTCCTCAATGCCAATTGTGACCTAAAGA
TCGGAGACTTTGGGCTGGCAAGAACAACCTCTGAAACAGATTTCATGACT
GAATACGTTGTTACTCGCTGGTATAGGGCTCCGGAATTACTCCTTAATTG
TTCTGAGTATACTGCTGCCATTGATATTTGGTCAGTTGGTTGCATACTTG
GTGAACTCTTCACCAGACGAACCCTTTTCCCTGGCAAAGATTATGTTCAA
CAGCTGACGCTTATCACTGAGCTAATAGGTTCGCCGGACGATGCTAGTCT
TGGCTTTCTTCGAAGTGAAAATGCCCGGAAATATGTCCGACTGCTTCCCC

AGTACCCACGGCAACAATTTTCTTCTAGATTTCCCAATGCCTCTCCTGGA
GCTGTTGACTTGCTAGAAAAAATGTTGCTCTTTGATCCCACCCAGCGAAT
CACAGGTAGACAAACACACTTACTTCAAATCTTTTATGCGGTGTTTCTTT
TAGTGTTTCTGTTCTCAGATTTTTGTTTCTAA
>EUC16962-RA [mRNA]
ATGCCGAGCATGGAGTCCGATCAGGACTCCGATCGCGATTCGGAGCCGTT
CGTAGAGACCGATCCGACCGGCAGATACGGCCGTTACGGCGAGCTCCTCG
GTTCCGGCGCCGTTAAAAAGGTGTATCGGGCGTTCGATCAAGAAGAAGGA
ATCGAGGTGGCCTGGAACCAAGTCAAATTGCGAAACTTCTGCGAAGATCA
ATCCATGGTCGACCGGCTGTTCTCGGAGGTACGGCTGCTGCGAAAGCTGA
AGAACACGAACATCATAGCTCTGTACAGTGTGTGGAGAGACGAAGATCGT
AACACGTTGAACTTCATCACCGAAGTCTGCACCTCCGGCAACCTGAGGGA
TTACCGGAAAAAGCACAAACAGGTTTCGATCAAAGCTTTGAAGAAGTGGT
CGAAGCAGATCCTCGAGGGTTTGGACTATTTGCATACGCATGATCCTTGC
ATCATCCACAGAGATCTCAATTGTAGCAACGTCTTCATCAATGGCAATGT
TGGTCAGGTAAAGATTGGCGATTTTGGATTAGCAGCAGTAGTCGGAAAGA
ACCACTCAGCCCATTCAGTTCTAGGAACCCCAGAATTCATGGCGCCGGAG
CTATACGATGAAAATTATACTGAGCTAATCGACATATACTCGTTCGGTAT
GTGTTTGCTCGAGATGGTAACTCGAGAATTACCCTATAGTGAATGCGATA
ATGTCGCTAAAATCTACAAAAAGGTCACCTCAGGTGTGAGACCTCGAGCC
ATGGACAAGGTCAAAGATCCAGAGGTCAAAGTGTTTATAGAGAAGTGCCT
CGCTCAACCAAGGGCGAGACCATCAGCCTCTGATCTGCTCAAAGACTCGT
TTTTTTATGGTATCGACGATGACGACGAAAATATATATGATTTTTTATAA

Supplementary File S2. The protein sequences of EuMAPK cascades

>EUC04697-RA [mRNA] [translate_table: standard]

MHNLTLFSRFEPGIQWKNIYYFAKAQPVFFITWSSHGHLFPVHYKGLFDSSFSESSCFRW
MLLFQSHRRQFRNRFPFALKIVPSFLVTEDFRLTSRMPSDNLNPPDQDREPFVEVDPSGR
FGRYNDLLGSGSVKKVYRAFDQEEGRDVAWNQVRLRNFSSDPSVVNRLYSEIKLLTTLKN
DHIIVLYHCWKDREHNTLNFITEACTSGNLRDYRKKHRHVSLKALKKWGKQILKGLEYLH
THEPCIIHRDLNCSNIFINGNVGKVKIGDLGLATIVGKSHAAHSLLGTPEYMAPELYEEN
YTELVDIYSFGMCLLEMDTKEIPYSECDSIAKIYKKVTGGVKPQALDKVSDPELKAFIER
CIGQPRARPSASDLLNDPFLSDVAELDEASPIS

>EUC04041-RA [mRNA] [translate_table: standard]

MKNLLKKLHAMNNQSGDSEGSTSSKSNRLRDGSSPDTPPQCRSPNSSEHKPFAAISGWLS
SVTKRHSSSPPSSSNVKRDHRMERLDSMRSTGAIGLNSESSNSGDPEVEEEYQIQLALEL
STREDPEAVQIEAVKQISLGSCPPENSPAEIVAYRYWTYNALSYDDKILDGFYDLYGIWM
ESSSSKMPSLIDLQEIPVSDNLSWEAILVNKVDDSKLLQLERKTLEMAVNSRSGSMNFVS
GNLVRKLAALVSEHMGGPVTDPDNMLMAWKSLSYRLKANIGSMVLPLGSFSVGLARHRAL
LFKVLADSVGIPCQLVKGQQYTGSDDVAVTLVKIDDGREYIVDLMADPGTLIPSGAAGLY
VEYEVPLFSTSPWSKDRDLSQVVPSTSRVSGSFEEHSMFGTVDKRSMFTIRNAVGNESDD
RGKNMHSANLVQQGGAEEGSTSYSDDLKNPCKVKEKITWEIPRRPSLCMHARSPSWTEGV
TSPAVRRMKVKDVSQYMIDASKENPHLAQKLHDVLLESGVVAPPNLFTEIYPERLNVLPS
KFPAGVKGENKEGDDIQNKCRNDLNRASFLPPLPYHGVKSKESSSGQPEHQLDSRDGTGL
HVSSQSGSNAVKYEKNVPVAAAAAAAAAVVMSSMLVAAAKVSTASNLELPVAAAATATAA
AVVATSAAVSFQNENMDCARSDGDEYAAVCESRDSVDGGNDAVASSQEGDRISDRSTGND
SAKSDAAIDDVAECEIQWEDVTLGERIGLGSYGEVYRGDWHGTEVAVKKFLDQDLTGESL
EEFISEVRIMKRVRHPNVVLFMGAVTRPPNLSIVTEFLPRGSLYRLIHRPNNQLDERRRL
KMALDAARGMNYLHNCTPVIVHRDLKSPNLLVDKNWVVKVCDFGLSRMKFSTFLSSRSTA
GTAEWMAPEVLRNEPSNEKCDVYSFGVILWELCTLRQPWGGMNPMQVVGAVGFQYRRLDI
PDDMDPAIADIIRKCWQTDPKLRPSFAEIMVALKPLQKPPVTSAQVPRRSSSVRGGQV

>EUC04221-RA [mRNA] [translate_table: standard]

MFKGRSLKGYSDELEDYHGYVETDPTGRYGRFDEVLGKGAMKTVYKALDETLGMEVAWSQ
VKLTDFLHSPEDLERLYSEVHLLRTLNHESIIRFYTSWINVDQRTFNFITEMFTSGTLRE
YRKKYKQVDIRAVKIWGRQILAGLVYLHGHDPPVIHRDLKCDNIFVNGHLGQVKLGDLGL
ATILRGSHRAHSVIGTPEFMAPELYEENYNELVDVYSFGMCILEVLTGEYPYSECANPAQ
IYKKVTLGKKPRAFYKVQDLEAQRFIGKCLETASKRLSAKDLLLDPFLAFDEDDPLLDLK
IGHPKPFLNIDIGVEELRLNEYRPKTNMTITGKLNPEDDTIFLKVQIADEEGTVRNVYFP
FDILSDNPLDVATEMVKELEITDWEPTEIANMIEAEISGLVPNWRKLDRSQSTHNHIMSH
VEDDDDDIDHIHHHHLHSPSTSSSSQVSFSGLIASHETDDRTQIRDWHQEDLFDDSSSHG
SSHSGNYSNMYYHYRDEHEQITSPRIRNHHHHNCTRFCPGENSRTTGHLIARNCYNQCKA
LVLESQGEKLLKDRRVLTRNRSLVDMRSQLLHRSLVEEISKRRLFKTVGAVENIGFQAPC
EVSDSHQPVGGGVYSMRSNRDGKKQGHKGRRV

>EUC20307-RA [mRNA] [translate_table: standard]

MLEGPKFTGIIDLNSSHDDISQNFYHKLGEGSNMSIDSFGSLQMSNGGGSVAMSVSSVGS
NDSHTRMLKHQGLKPGNNYSVAHSVNRGKVSQGLSNDALAQALMDPRFPTEGLEDFDEWT
IDLRKLNMGAAFAQGAFGKLYRGTYNGEDVAIKLLEKPENDRERAQLMEQQFQQEVMMLA

TLKHPNIVRFVGGCRKPMVWCIVTEYAKGGSVRQFLTKRQNRSVPLKLAVKQALDTEGMT
PETGTYRWMAPEMIQHRAYTHKVDVYSFGIVLWELITGMLPFQNMTAVQAAFAVVNKGVR
PNIPTDCLPILAEIMSRCWDPNPDVRPPFTDVVRMLEAAEMEIMTTVRKARFRCCISQPM
TTD
>EUC20242-RA [mRNA] [translate_table: standard]
MERTTSNKPKFPLCFNPFRILKSRNKPPSIPSSSRTQFNSDTESMERKRFDSLDSWSMIL
ESENVETWETSKDDQEDWTADLSQLFIGNKFASGAHSRIYRGIYKQRAVAVKMVRIPTHK
EETRAMLEQQFKSEVALLSRLYHPNIVQFIAACKKPPVYCIITEYMSQGTLRMYLNKKEP
YSLSTETILRLALDISRGMEYLHSQGVIHRDLKSNNLLLNDDMRVKVADFGTSCLETQTQ
ESKGNMGTYRWMAPEMVKEKPYTRKVDVYSFGIVLWELTTALLPFQGMTPVQAAFAVAEK
NERPPLPASCQPALAHLIKRCWALNPSKRPDFSDIVSALEKYDECVKEGLPLTLHSGLVS
KNAILERLKGCVLMNSSVLVHA
>EUC13910-RA [mRNA] [translate_table: standard]
MHRIPQFFNHSKSSTSSKHRRTASAKPKLKRSNAARHIDYEAPSTSSSDQSSSALRARSL
GILPSNDRTSFRVEGNEGELEVICRRLGLSGPEEFEIPAAAWEAMKSRSSSDVLPLSGEL
RFDSLNVEVQVENEGPSENLDGVLDSLRACNVNDLQIEVVPLNDCKPVGGDGDDADELVN
SLEDSVRVSSLIVNKNELVETAGSGIRVQHSNGGRGIKGDRPPTLEPPPSMLLPVIDNGC
STWDLLRSFAPGSDIISGFEIHGGIASDDEQDEGNEESGNRDGEHNDVRVGGPCVLYESC
SFTTSNDDDSSSTTTDPASNNISPNVRSKRNITDWEKGELLGRGTFGSVYEGIDDGGFFF
AVKEVSLLDQGELGRQSIHQLEQEIALLSQFEHENIVQYYGTDKAESKLFIFLELVPKGS
LLRLYQKYTLRDSQVSSYTRQILHGLKYLHDRKVVHRDIKCANILVHTTGSVKLADFGLA
KATKLNDIKSCQGTAFWMAPEVVNRKNQGYGLAADIWSLGCTVLEMLTRQLPYSPLECMQ
ALFKIGGGIPPPVPDSLSTDARDFILQCLRVNPAARPTADQLLGHPFVNKPLSSSLAFAS
PLDPRRRV
>EUC14489-RA [mRNA] [translate_table: standard]
MDLVEEVGESSSPARSFGSFSGHDIRNDVYNRLVESGHEEAVSDPGFREQLDAHFNRLPA
SYGLDVNMDRVEDVLVHQKLLALAKDPDNRPAFHVRFLENFWTRADGDEDQQCFDLPSTS
RPSFNVDNEEEFPSPDRNRNCENDCEPCSKLEDLNLDVRKSSNGLEMGTLDEDFPRRQEL
PHVPIHEVIFSTIDKPKLLSQLSALLSDIGLNIREAHVFSTTDGYSLDVFVVDGWPIEDT
KGLSEAVEKAIGRSEGSWSGCSHSQLARDKKIELQEQSGDWEIDRRLLKMGERIASGSCG
DLYRGLYLGQDVAVKVVRSEHLSDTLEVEFAQEVAILREVQHRNIVRFIGASTVSPDLCI
VTEYMPGGSLYDYIHRNHTVLHLPQVLKFALDVCKGMEYLHQNNIIHRDLKTANLLMDAQ
SVVKVADFGVARFQNEGGVMTAETGTYRWMAPEVINHQPYDHKADVFSFAIVLWELVTGK
VPYDTMTPLQAALGVRQGLRPEPPKEAHPKLVDLMQRCWEATPSDRPSFSDIRIELEQLL
IEVQDTSKAQNGCC
>EUC13785-RA [mRNA] [translate_table: standard]
MGSGSTFVDGVRRWFQRRSSSSSTVIANNENVRVAATTNDSESQSSSTLEHVKDQLNIVR
DFDISALKLVKVPKRIGFPMDPHKKNTLETEFFTEYGEASRYQIQEVVGKGSYGVVGSAI
DTHTGERVAIKKIHDVFEHVSDATRILREIKLLRLLRHPDIVEIKHIMLPPSRREFKDIY
VVFELMESDLHQVIKANDDLTPEHYQFFLYQLLRGLKFIHTANVFHRDLKPKNILANADC
KLKICDFGLARVSFNDAPSAIFWTDYVATRWYRAPELCGSFFSKYTPAIDIWSIGCIFAE
MLTGKPLFPGKNVVHQLDLMTDLLGTPSPESTARIRNEKARRYLSSMRKKHPVPFTQKFP
SSDPLALRLLERLLAFDPKDRPSAEEALADPYFHGLANVDREPSTQPISKLEFEFERRKL
TKDDVRELIYREILEYHPQMLQEYLRGGEQTSFMYPSGVDRFKRQFAHLEEHFGKGERST

PLQRQHASLPRERVPALKDEKTTQNDDLEKRSAASVATTLQSPPRQPARLENADINAQKA
NNSARSLLKSASISASKCIGVKARKDQEDEAIPEQHDEVDGLSQKVAALKA
>EUC16639-RA [mRNA] [translate_table: standard]
MDLGTNEGIEFVQKSGDQEVGLGSKLKGRGSLSEKNMSGRSSTSLGSKDMIFRADKIDLK
NLDVQLEKHLSRVWSKNIETQRPKEVWEINSSKLDIRYFIAQGTYGTLYRGTYDNQDVAV
KLLDWGDDGVATTAETAALRASFQQEVAVWHKLDHPNVTKFIGASMGTSHLKIPPKNPSS
GGGFIDLPSRACCVVVEYIPGGTLKGFLYKNRKKKLAFKIVVKLALDLARGLSYLHSKKI
VHRDVKAENMLLDSNITLKIADFGVARVEAQNPKDMTGETGTLGYMAPEVLDGKPYNRKC
DVYSFGICLWEIYCCDLPYLNLSFAEVSSAVVRQNLRPEIPRCCPNSFANIMKKCWDSNP
QKRPEMEEVVKLLEVIDTSKGGGMLPEDQARGCFCFHPSRGP
>EUC16268-RA [mRNA] [translate_table: standard]
MAFDQNSIPKDLRPLNIVRSTPEESRIAPVATSGRAVEGFYANPPRDVTSPRSAPLYYNP
AMVTDAGLAGLGYNNAPSSVAGWVPHVPPVIVGTSGVNPAIGHCYNPNVGTRVGVISSDQ
GSEEGGEDSVSGKKVKFLCSYGGKILPRPSDGVLRYVGGQTRIITVRRNVSFNELVQKMT
DTYGQNVIIKYQLPEEELDALVSVSCPDDLDNMMDEYDKLVERSTDGSVKLRVFLFSASD
IDSSFGDLQASGKRYFEAVNGITEGGGGITRRDSITSAASTQNSDLSGIEPVDNAGYSHD
DATGLPSTVLLSPRRNSTTSQESGQRLVVDPSQANYADASALLANSAVKTGYPLSSQPEP
AHVIQQQLAFDLQQPGVNFPAPASYLQAYVDPRQRQETVNHSDYAQQPPGVGFPPHLLGT
VGPVFTHQQFSPGMSPQQFIPAVHMTMTPSTHVMMRPNMVQPSVHPNQVQLERYPEENTF
SQRGVQVPGSQSYSTYQAQVPAPVMGGGYGWNQVQVIHPEQVTFSEGWVPQPQQQVMAPE
KIARLEDCHMCQKALPHAHSDTLVQGQRESPVSTISDSNIVYHSLRLDDKGRPNKWDNTD
HPNVTAAQGVVGLSSGLQSPYGAFVGNSPQVASDIPSVGGMPFQASDHLVFESPKDFSGK
FPKEDTVQSAVTYDHLRQIDGRMGNLGIHSPEALVNNELSKSQVDNPKWENVNHAEPNEM
LYQAVDSNEVPQPPRLGTPGLYPQPNLSANYLVPDEIASTGPAFVAADPAHTVERITPIG
QWKDNCSRNQTNIAATNVEVVSSDGNTPTSLSSSDRVANVQDSSNSLFSNQDPWSLHHDT
HFPPPRPNKILTKKEDTVSRNPFGDSRFGDSAESLGNVGGLRTDLQLDDGAYEPSSYLHM
DIPLEHGRSNKSEGSAEELIKQELQAVAEGVAASVLRTSAPSNPNSAEHGRSESHFKSNE
YSEVKASDGELPDKEKSEDISIKTPQKSNFGFPLSDGIGRLQIIKNSDLEELRELGSGTF
GTVYHGKWRGSDVAIKRINDRCFAGKPSEQERMRDDFWNEAIKLADLHHPNVVAFYGVVL
DGPGGSVATVTEFMVNGSLRTALQKNERQLDKQKRILIAMDVAFGMEYLHGKNIVHFDLK
SDNLLVNLRDPHRPICKVGDLGLSKVKCQTLISGGVRGTLPWMAPELLNGSSSLVSEKVD
VFSFGIVLWELLTGEEPYADLHYGAIIGGIVSNTLRPAVPESCDLEWRLLMEKCWSSEPS
ERPNFTEIANHLRSMAAKIPIKGQNPTASS
>EUC16831-RA [mRNA] [translate_table: standard]
MEKFNAERSWVRGSCIGKGSFGSVSLAVDLSDGEVFAVKSVDRNSSSPAQVEAIENEIRI
LRSISSPYVVGYLGDGWTSEFPSASYRNMYLEYLPGGTVADLAKRSDGDVDEETVRRYTW
CIVSALRYVHSKGIVHCDVKGKNVLVGPANGAVKLADFGSATEFNDRKYPRGSPLWMAPE
VVQGGYQGPESDVWSLGCTVIEMITGKPAWEDRGADTLFEIGYSGKLPEFPTHLPEHGRD
FLEKCLQRDPCKRWNCDQLLRHPFISSSSPDDTNTDSTPRCVIDWFSSESGEDSDADASC
ETEDFNENAKQRISELATNSGANWESDGWMLVRNLITESEPASALYRRNRNTKMASSSAG
GGTVSEPVGGVRMGGGQIGNRRWRAGRKGFTAVAN
>EUC10582-RA [mRNA] [translate_table: standard]
MTPETGTYRWMAPEMIQHRPYTQKVDVYSFGIVLWELITGMLPFQNMTAVQAAFAVVNKG
VRPNIPNDCLPILTEIMTRCWDANPDVRPPFAHVVRMLEAAETEILTTVRKARFRCCISL

PMTTD
>EUC10624-RA [mRNA] [translate_table: standard]
MDSPTGTLTTTGSGPSSNEENPRVKFLCSFSGSILPRPQDGKLRYVGGETRILSVPRDIS
YEELMVKMRELFDGVTVLKYQQPDEDLDALVSVVNDDDVTNMMEEYEKLGAGDGFTRLRL
FLFSHPDQDGSMHFVDGDERDNERRYVDALNSLNESPNFRKQPFSDSLLMGPIDDINVTE
EYLNQISLEGSVHNQRNFDMHMPHINLRHLTIPQMGSGQHQLSVSQRYNDMEAPWSPIYY
SPGHPGHHDPRPAAEFPASPSSAGHPTPFGELSDRTFDRIPEDYSLHQVNNQYDYLPQFS
DKVAFFSGGPVSADKAGFPGNILQGPSIFEGNNVCENCRMTFQRNQAYSDSPWKHGEQPR
LESSNVGSVFHQPANPCADCHSNREIYMLHTDASMQQPLYREQNNSRTLHNEAQERGWVF
QQNSNLRVDKPVPQLSGTGRLMDHYVVNGNGIPAPAAHGNLSNGHHVPSHYAHHEDVRYP
CVGHELGNQVFNDQNVTSGSQIHTHTPPPEECGVLYGNVTYAYGADNLYQFSRARTPPNA
IWRNVHNPVHGAQSYETSSSHLLANGSVGAGFIRCALESSPRIRLGVENQNPWVDSSQYM
MGSEGAAVPKYSHGNAPQLVPNTCCHENQHLPTPEPQPSPAELLNHLTPVEPILNSDVAS
KVVDYKVVPLTTSGPEASNDTDLTESERLVQQSSHGGEETNHEANMPSISALVKSENPDD
IGSPEAINSNSSKPVEERNGGVKSIENHALAPLDSEQLLDRIVTENKGELEGLGVKIDDD
ENSASVIEHNAAAKEAQNDRESVEAHEDCEFGSDNDNSNDIKIEPTKAEAEAIDRGLQTI
KNEDLEEIRELGSGTYGAVYHGKWKGSDVAIKRIKASCFAGRPSERERLIADFWKEALIL
SSLHHPNVVSFYGVVRDGPDGSLATVTEFMINGSLKQFLQKKDRTIDRRKRLMIAMDAAF
GMEYLHGKNIVHFDLKCENLLVNMRDPHRPVCKIGDLGLSKVKQHTLVSGGVRGTLPWMA
PELLSGKSNMVTEKIDVYSFGIVMWEVLTGDEPYSDMHCASIVGGIVNNTLRPKIPTWCD
PEWKALMESCWASDPGERPSFSEISQRLRNMSASLNPK
>EUC10368-RA [mRNA] [translate_table: standard]
MRKVIVIVVVYLSIVIRNTNPIEETTPLCLSGFSRFIVGSHFMASASYNEILGKGASKTV
YRAFDEYEGIEVAWNQVKLYDFLQSPEDLERLYCEIHLLKTLKHKNIMKFYTSWVDTANR
NINFVTEMFTSGTLRQYRLKHKRVHMRAVKHWCRQILRGLLYLHSHDPPVIHRDLKCDNI
FINGNQGEVKIGDLGLAAILRKSHAAHCVGTPEFMAPEVYEEEYNELVDIYSFGMCILEM
VTFEYPYSECTHPAQIYKKVISGKRPEALYKVKDPEVRQFVEKCLATVSLRLSARELLDD
PFLQLDESESDLRLIDNGRELDSMGPLLGQPFFELDYEGKSFSNSSYNGYSNGYAFDAQN
GWGYHSSEIEQSGIELFDYNDDDNDQNEHFEGLDITIKGKKREDDSIFLRLRISDKEGRI
RNIYFPFDTENDTALSVATEMVAELDIMDQDVTKIADMIDGEIASLVPEWKQGPGIEETP
CFTDHGFCNNCASNRTSNGSFLNFLSKHPSAQNLQILHCSGNCCAAMHGRFEEITYQVDS
PRQYVQNKPFKSSQTNDFHQTECWYQHESQECSSTGSKESPSVGDHKNLDRKYSAKDELG
IGIAKEISTHEMSDENQQNMRWLKAKYQMKLRELTDQHLGVVPNSPRSDSATQRAEKCEA
SKESQPDDAFCTNSLLPNTLHRTSSLPVDAVDI
>EUC11981-RA [mRNA] [translate_table: standard]
MERNLRKGVTGQQRNHEQVRYNSRQNENEGLGSANQILFQDPPSSINRDITRQDFLKMAG
ARPVLNYSIQTGEEFALEFMRERVNSQQHFIPNACTEPVGATSYVELKGMMEIPHTVSES
ESDTSMIQSVDKNRIQDHERKRTSLNDDKAYYESGQSVSRTLSRNENRQGVHSKAPSGAS
SNSSMKLKFLCSFGGKVLPRPSDGKLRYVGGETRIIRISKDISLQEFRQKTLTIYNQARM
IKYQLPGEDLDALVSVSCDEDLQNMMVECAVLDDGGSHKLRMFLFSDSDLEDSQLAMGSV
DADSGIQYMVAVNGMDIGASKNSIGLASASAYNLEELLGLNDERESGRVVANLAAVGTEP
SLVAMPSTIQSSRTVLPSASVASELNPQAYQSQMMHNGEAECHSLSSGYPMERLDPKERG
TVLSGVPSQYDDVSHQSNYVSAGEKMILPPLHGHITQEGGAVEDQSYNAVNVQVPEVSLM
EAELKDDSSFLKKNDFSKDLSPKKEVLVDEVKLESDSSVPESNEAEIVPFLEDEYIVSSH

LYESSLNYVSKDEKLIASTAADTGTSALPIKNVVIFQEPMQNLFPSAAEKEEKVNESDEN
DHFYTSEKTSTLGDDDSVGYSIEFSHKEPPTLSHRGFHSERIPREQTGLGRLSKSDDSYG
SQFLISHTHSDVSKQIAASFHDENVSSQREGFISSAEPLCANVQTAENGLGQYLNSEEVV
NNRKEDSDNSKEASGFNLCTTSQGISGNFNGDSTSKSPEPQSDENSARKSNASSKGLLAR
SERSIAPLPPGVSSVGAGTTEQGDIIIDVNDRFPRDFFSDIFSKAGTIDGSAGIGLPDGD
RTGLSFKPKRWSFFQKVAEDDFGRKDVSLMDQDHLGFSSLLTNIVEGAPLDYNFPPFQAG
GVAQLGSHINFDTAVQQRSHDIMGSNTLDLHSDYNPSEATDNQIVQSSGLLRKAVVGESY
FEDGKLESNTTGQPLFDPSLGVFDPSALQIIKNEDLEELRELGSGTFGTVYHGKWRGTDV
AIKRIKKSCFTSRSSEQERLTVEFWREAEILSKLHHPNVVAFYGVVQDGPGGTLATVAEY
MVNGSLRHVLLCKDRHLDRRKRLIIAMDAAFGMEYLHSRNIVHFDLKCDNLLVNLKDPLR
PICKVGDFGLSKIKRNTLVTGGVRGTLPWMAPELLNGGSSKVSEKVDVFSFGIVLWEILT
GEEPYANMHYGAIIGGIVSNTLRPHVPNYCDSDWRSLMEQCWAPDPEVRPSFTEIAIRLR
VMSTAKPQGYPTQNKASK
>EUC05776-RA [mRNA] [translate_table: standard]
MSWWQSGSSSSSSKSSPEDGLRNMFRLFSGRRHSHSSQPRQFSRGRKLRYFAEDDVRRPP
PPVDPPELWRSATALEISPNRSSSGAAPAAAPLPLPLPELGALLRRDSSLVSNSNSADCL
LMSLKEAGGREGVEDRGKSERLNGDGVMIGSKFSGHDARKSTDHSEPQSSKKSNGKKSNK
NNCQINIPVSAPTSPYSSPLRSPHGNNGDLYLPPYMIPPIFQVWSAPELPSSDMNQGLGF
PHQTFPEKTAFSVDSSPLRSPRVSPHQTARSPSGPISPLHSKSTVEIASSRWEGNPQADV
HPLPRPPGGMIPSQTTVPQFTIKPEVMPLRRQWKKGKLIGRGTFGSVYVASNRETGALCA
MKEVELLPEDTKYSENIKQLEQEIKVLSQLKHPNIVQYYGSEIVGDRFYIYLEYVHPGSI
NKFIHDHCGAITESVVRNFTRHILSGLAYLHSTKTIHRDIKGANLLVDAYGVVKLADFGM
AKHLTGQAADLSMKGSPYWMAPELLQSVMQKDARADHALAVDIWSLGCTIIEMLNGKPPW
SEYEGAAAMFKVMRETPPIPETLSAEGKDFLHCCFLRDPSERPTAAALLEHRFLRNSQQL
DVPICSQFVHGMKLTDKAQAPGEVASHKVNQLPEGKLVKKTLRELKVAPHRSPRSILEAI
SSLSPPRLGRTSDPVLTAGPKNNP
>EUC05265-RA [mRNA] [translate_table: standard]
MEKEESGTAIQIKGVPSHGGKYIQYNVLGNLFEVSSKYAPPIQPVGRGAYGIVCCATNSE
TKEEVAIKKIGNAFDNRIDAKRTLREIKLLCHMDHDNIIKIKDIIRPPDRENFNDVYIVY
ELMDTDLHQIIRSSQALTEDHCQYFLYQLLRGLKYVHSANVLHRDLKPSNLLLNANCDLK
ICDFGLARTTSETDFMTEYVVTRWYRAPELLLNCSEYTAAIDIWSVGCILMEILKREPLF
PGKDYVQQLGLITELLGSPEDADLGFLRSDNALRYVKQLPHFPKQPFSKKFPEVSPEAID
LAEKMLVFDPSKRITIEEALNHPFLSSLHEINEEPVCPSPFFFNFEQSSLSEDDIKELIW
RESINFNPDKMLE
>EUC05347-RA [mRNA] [translate_table: standard]
MAFGAENQNNPPFRQTAPHTCKTMPGERPRTSRPRFNNGTSNNLNRADESYRDISVQTGE
EFSADFLRERVSHRAASADMDEQMKNGVGFIVGQNHELVYEDLSRILGIRRRDSDNAPEV
SEFYQVQKNYDFAPEIRNHRDYRFNNDIQPRKKSFEIVFSPTGPPLHSSDSPSSQQPHYG
LGVSDGSSSGKVKCLCSFGGKIIPRPSDGKLRYIGGETRIMSIRRNATFSELMKKTCAIC
NQLHTIRYQLPGEDLDALITVSSDNDLHLMIEEFRDLDKFSQRLRIFLISLNESDGGSSS
SFEGKNLQPNEGDYHYMNAPNGMSSSRESLPSPSQLKIHELDSPDNPNLQMYDDHPFGGN
FMYTNNQREYKNPYFDTTGYYYSNPVNNVPAMNYQNQNNFFAELELGHDLYPQNHVQGSE
FPPLPLYGDFANDRPVHNCFTYFSTKSTFSEKKNDHFSGSDIPIEDCHEQSSDAQLQLDT
TMSGLSQDLVMQKLETGDEKYQKGGESVNMLKFTMNNDQSMEYIDWGKTTINWMGKKESN

DREGDEDIKANANIRSRENSRSGVRIVEQEREARKSSLNNAADQSPGMVGFEPFFTNQGN
AIPFAEYESCLSLDWHENDRHMIDLSLPSSITAILGRKISNKVETEVKPTSKGSNFVEIR
QSGDRKHVSLQKSTFNVEDVTHIVPHDVASSSKIVPYVHDGTSDGSTSPKGSETESETGS
SISKEFNVEIAEVDGNGKEEAATPEVCIAEMEADFNNLQIIKNVDLEEQQELGSGTYGTV
YHGKWRGTDVAIKRIRKSCFAGGPAEKERLIKDFWREANILSKLHHPNVLAFYGVVPDGP
GGTLATVTEYMVNGSLRHVLLSKEHTLDRRKKLLIALDAAFGMEYLHMKNIVHFDLKCDN
LLVNLRDPQRPICKVGDLGLSRIKHNTLISGGVRGTLPYMAPELLTGNSGRVSEKVDVFS
FGIALWEILTGEEPYANMHCGAIIGGILSNKLRPTIPENCNPDWRKLMEECWSGNPTERP
SFTEITNRLRAMTTSLQPKRHNFSKR
>EUC05370-RA [mRNA] [translate_table: standard]
MSAWWKRKSTKNKEHQQVQQKPQSDYQNTSRKASIKDDKKRGQDKPKSFDEVSTLVFSHN
SPRNSKEFGGLVGGSSGGSSGFSGFDSSERGHPLPRPSVSSTQSFGVDHLQGVGSGSGSV
SVSSVSSSGSSDDQAHLDQVHGQLGFVSRVHGGDTKFYTVPRSPGPALRGATANTSPLHP
RSSLDSPTTRVEDGKIECHRLPLPPGSPTSPSALPMPRACGVTDNSLSKWKKGRLLGRGT
FGHVYVGFNSETGQMCAIKEVKVVADDQTSKESLKQLNQEINLLSQLSHPNIVQYYGSEL
SEETLSVFLEYVSGGSIHKLLQEYGSFREPVIQNYTRQILSGLAYLHGRNTLHRDIKGAN
ILVDPNGEIKLADFGMAKHITSCSSMLSFKGSPYWMAPEVIMNTNGSGLAVDIWSLGCTI
LEMATSKPPWSQFEGVAAIFKIGNSKDIPEIPDRLSDEAKSFIRLCLQRDPSARPTALKL
LDHPFVREQSTARTANVNLTREAFPSSFDGSRTPTALEMHSNRNSITSFDGDYFAKPIVA
VPRALISQRESARTITSLPVSPCSSPLRQYGPAHKSCFLSPPHPSYRLVAQNGYNLTDYS
VLTARHSRKTTLDPWLEIPQFNPQNPGRSSRPRPIL
>EUC05489-RA [mRNA] [translate_table: standard]
MQEIFGSVRRSLVFRTPEGGLADEATAGGTLVNKINSCIRKSKVFCKPSPPSLPPSRTII
KDNNAPPPTIRWRKGELIGCGAFGQVYMGMNLDSGELLAVKQVLIAANGSSKEKAQSHIR
ELEEEVKLLQNLSHPNIVRYLGTVREAETLNILLEFVPGGSISSLLGKFGSFPEAVIRMY
TKQLLLGLDYLHKNGIIHRDIKGANILVDNKGCIKLADFGASKQVVELATISGAKSMKGT
PYWMAPEVILQTGHSFSADIWSVGCTVIEMATGKPPWSEQYKEVAALFYIGKTKSHPPIP
EHLSAEAKEFLLKCLQKEPESRPPAFELLQHPFVTGESMPSPLVHSSSWECSKTPNSLCG
KHLDNMSSSIDVCNLGTLNFSSVNTEKLSESRYISEVNKSDDYMCQIDDSDDFMSYNPIC
ESSDDWKCIESSGVEQERMDTDNDQQVDILATTSGLSMKGENSFSLNGAASLSEDEDEVT
ESKIIAFLDEKALELKKLQTPLYEEFYNSLNASCSPNLAETLHDGSALNYLRLPPKSRSP
NRAAVGSPSKAVDATHASPGRNNKHISNVGSASNHTIQDKTSPQPNDSEGVLVDSEQEPD
SPSASFSDIQRKWKEELVQELERKREMMRQATTGGKTSSPKDPSLNRNRELSRFASPGK
>EUC07900-RA [mRNA] [translate_table: standard]
MQHDQRKKASVDVDFFTEYGEGSRYKIEEVIGKGSYGVVCSAYDTHLGEKVAIKKINDIF
EHVSDATRILREIKLLRLLRHPDIVEIKHILLPPSRREFKDIYVVFELMESDLHQVIKAN
DDLTPEHYQFFLYQLLRGLKYIHTANVFHRDLKPKNILANADCKLKICDFGLARVAFNDT
PTAIFWTDYVATRWYRAPELCGSFFSKYTPAIDIWSIGCIFAELLTGKPLFPGKNVVHQL
DIMTDLLGTPSAEAIARIRNEKARRYISSMRKKRPVPFSHKFPNADPLALRLLERMLAFD
PKDRPTAEEALADPYFRNLAKVEREPSAQPVTKMEFEFERRRITKEDVRELIYRETLEYH
PKMLKEFLDGAEPTGFMYPSAVDKFKKQFAYLEERYGNGGAAPPPERQHASSLPRPCVLY
SDNSNSAQNSSDISNELSKCSIKEAEKPHHTNRTSAIPITRLPLHVPQTIQAGGAARPGK
VVGSVLHYNNCDAIVSAAAPAAEAAHQQRIVRNPAVPSQYTTVSTSYPKRSSVCKNDRGE
DGTQMPPKPDQYIPRKVAAAQGGGSGSHWY

>EUC11169-RA [mRNA] [translate_table: standard]
MNNTAPRPAGQWIQQAVGVIPKDVLVEKNVSNNISVQTGEEFSLEFLLDRATPKQVPVMP
DMSKKQENGLRFINKQNHHRGYEEITRLLGLKRMDSECGSDITEFASARGSSFEIENVPN
ITKECGYHVEQNMNGHTPRKASVEKTYNWNVIEPNASPVSGSDSSHKRQPSADSSGPGKM
KFLCSFGGKILPRPSDSKLRYVGGETRIISIRKDISWEELVKKTTGICNHHHMIKYQLPG
EELDALISVSSDEDIQNMIEEYHGLETLGGSQRLRIFLIPSNESENTCNLDSGNLQPSSP
DFDYVAAVNGIVDPSPRKNYDRQQHLAGDLSQLKSNENSPSTLVPLDINDTFDVSHTTKN
ASPNLIMSPRGSPFSPVIVRQGESKSVHAQPYKDDASCGSSESTRLFSNAQLAPEDSSSN
IAGNNCPHLEAANVVSYQSPNQDNDLGHSGKSVGAHFDNPNHNRDFSVPHLVNQNDKVSD
KCHLERPVVTGRPFHSDKPIACLDDPLGLQLGSNDSIGSHRGVPHVFSDSQLQLNAGRST
YCSQERPNPSSPLTFAVPQLSPPVASAALQEKPVHLRQNIAPVSPYRETKLPDVEPTVSN
VRMDLPKSSLELGTKVERARWEAATIDEKCKSAEEDLNKYSFMMQKHDQKNHLIGKLMND
PDEEDPLWCRVEKPCESLADNSLTDVSNSPNIGRDPFFMIGDNTTTQDWQIPGGMVPGAS
AIFLNPFADQPMHQAPNNQLVEAPSEINVKGQRPTENHQILSTGITNGEQETNFSWAKKV
ELSRIIQGSKQQAYYESSFSNPLQAFHSGLVFDEQIVHQPFAHLSPEELRPSQVYDAAAL
HTDMHTPTSDQNPTNDTGITREAYILHDRFANYSDQKVENSGTAEFSFEKPKLRNNVPIR
YDQGNQLEVVNTVEGASNTILPGISPTLKMPYGVDANGCKVPYSLAMQANSISDFKNGEA
DYADKDESISDIRMAEMEAGIYGLQIIKNADLEEIRELGSGTYGTVFHGKWRGTDVAIKK
IKKSCFSGRSSEQERLAKDFWREAQILSNLHHPNVLAFYGVVPDGAGGTLATVTEFMANG
SLRHVLLKKDRLLDRRRKLIIAMDAAFGMEYLHSKNIVHFDLKCDNLLINMRDPYRPICK
VGDFGLSRIKRNTLVSGGVRGTLPWMAPELLNGSTSHVSEKVDVFSFGIALWEILTGEEP
YANMHCGAIIGGIVKNTLRPPIPERCDTEWRRLMEECWSADPAARPSFTDITERLRSMA
>EUC08948-RA [mRNA] [translate_table: standard]
MEQSRNNVNFNSTRPENEELDPRSQGYTRDSSGHVIANIDPAEIKFPQAKPVLNYSIQTG
EEFALEFMRERVNPKKPFVPNVDPSYTPCYMELKGILGISHTGSESGSDVSMLTIVERGS
KELDRRNSSLYENRSNYGSVQSVPRTSSGYNSNQTIIQDYSSSGASGSVSTKMKVLCSFG
GRILPRPGDGTLRYVGGENRIIRISKDISWQDLWIKIKTIYELTQTIKYQLPGEDLDALV
SVSCDEDLQNMMEECKVIEVGERSNKLRMFLFSISDLDDAHFSLANSEGDSEIQFVVAVN
GMDIGSRKDSNLHGLASSSANTLDELNMQNVQRNKSGAAADFFSDNTFTSTGFVTMPPTS
TSVSPVVPTSSHVTETAPQLYHSDMVHQDAAKEDDSHSSQISFFGETSVPQPIQSGSERQ
QSQGEQVVETHVEDAKQKNVGSVQQGHEQETIRPLANEYLVPPNPYNGSVTDYFPAREVV
GPTPEGNFSLDSMKNEARPHEPVEVSSALGAAENHKSIGEDFHASSGAFAPGPVNSENDP
TDPVFVEPDIPTQRVFHSERFPRGHAESLNRLSKSDDSHGSQFLMTHSRSDIAQKDLIVE
SAEKLQNGNYGRQNELVNTGTTIDGVRNLQQLNQSIPSPGNGENVVLENQITKTDQGVTI
DNSGNLLVDEFEKTGSELPAVSRVDSRKHHDEPASSRLDVQLGDTVGSDSTPSNTRGLAP
TSARVGRPAGDFSLDESSVHAPRPERGDILIDINDRFPRDFLSDIFSRAVLSEDPYNIGP
MDKDGAALSLNMENHEPKRWSFFQKLARDDFARKDVSLIDQDPGFSSRLPKVEEELSGSV
IPDTIALHSNYVSSIAKVSEGIQYHDLVDNLRIVESEYEAAMRTIGLSPLDSCLAEFDIN
SVQIIKNEDLEELRELGAGTFGTVYHGKWRGTDVAIKRIKKSCFSVRSSEQERLTIEFWR
EAEILSKLHHPNVVAFYGVVQDGPGATLATVTEFMVDGSLRHVLLRKDRHLDRRKRLIIA
MDAAFGMEYLHSKNIVHFDLKCDNLLVNLKDPSRPICKVGDFGLSKIKRNTLVSGGVRGT
LPWMAPELLNGSSNKVSEKVDVFSFGIVLWEILTGEEPYANMHYGAIIGGIVSNTLRPTI
PSYCDAEWRKLMEQCWAPNPVARPSFTEITSRLRVMSSAPQPKTHKPSNN
>EUC14834-RA [mRNA] [translate_table: standard]

MRPLQPPPAASGSAAANRNRPRRRPDLTLPLPQRETSLAVPLPLPPSSAPSSSNPSQFPS
ASTPINFSALERICRIGSGSGGTVYKVRHRPTGKLYALKVIYGHHDDSVRRQICREIEIL
RDVDNPSVVKCHDMFDHNGEIQVLLEYMDGGSLEGTHIHHEPSLADLARQVLSGLAYLHR
RRIVHRDIKPSNLLINTRKQVKIADFGVSRILAQTMDNCNSSVGTIAYMSPERIDTDQNH
GKYDGYAGDIWSFGVSILEFYLGRYPFAVGRQGDWASLMCAICMSQPPEAPPTASPEFRD
FIACCLQTNPAKRSTAPKLLAHRFIAQYAGSTGHSGNHQMHQVLPPPRHFSSA
>EUC12664-RA [mRNA] [translate_table: standard]
MPPWWSKSASKDVNKKANKESFIDTIHRKFKIVSEGKCGSRAFSDKASRRRRSSDTFSDK
GLSPLPSTHVSRCQSFAERAQAQPLPLPGLQNPANLRLAGNRASKKSGSEEGCKTPNLPV
PRSGNSPNVLNEGDLATASVSSDSLSDSDDPFESRLLSPQVSDYENGNKTAISSPTSVKH
KDQSPLTSQMNARMANMLLSNQTQPVRASARRVPNLQIPNPNIFHSAPDSSMSSPSRSPM
RIFDPITSPYFYSGHCSSPGSGQNSGHNSIGGDMSGQLFWPHSRSPECSPIPSPRMTSPG
PSSRIHSGAVTPLHPRACGLADDGKQQSHRLPLPPLTIPNSSHVSHSYSTGTTPIVPRSP
GRTENPLSPGSRWKKGRLLGRGTFGHVYLGFNSGSGEMCAMKEVTLFSEDAKSKESAQQL
GQEIALLSRLRHPNIVQYYGSETVDDKLYIYLEYVSGGSIYKLLQEYGQLGEAAIRSYTH
QILSGLAYLHAKNTVHRDIKGANILVDPNGRVKLADFGMAKHITGQSCPLSLKGSPYWMA
PEVIKNSNGSNLAVDIWSLGCTVLEMATTKPPWSQYEGVAAMFKIGNSKELPTIPDHLSD
DGKDFVRRCLDRNPLHRPTAVKLLEHPFVKNASPLERPILYPEEGSSPATNSIRSLGIGG
YATSSSLFESSASHLSRSPRNGSAFSDVNTHKNISCPVSPIGSPLLHSRSPQCTSGRLTP
SPISSPRAVLSGSSTPLTGGGNGFHPQTHPPLNHQNENMGTMFPLPQNHSHPKPDMFRGI
SQNGFLGYQFGGQSVLADRVSQQLLKGNVKVNPLFDLNHGSPVLGHGNGTSY
>EUC12684-RA [mRNA] [translate_table: standard]
MLQLLRPNSLNSTLSGESADDFSAYYSRSSSTPSGARHYTTRTPLYSELIDRSLAMPMES
TSASASGSRNDGGIKGVLTHGGRYVRYNLYGSLFEVSSKYVPPLRPIGRGAYGLVCAAVN
SDTREEVAIKKIGNAFDNRIDAKRTLREIKLLRHMDHENIIAIKDVIRPPKKDIFNDVYI
VYELMDTDLHQIIRSDQPLTDDHCQYFLYQLLRGLKYVHSANVLHRDLKPSNLLLNSNCD
LKIGDFGLARTTSETDFMTEYVVTRWYRAPELLLNCSEYTAAIDIWSVGCILGEIMTREP
LFPGKDYVHQLRLITELLGSPDDSSLRFLRSTNAQRYVKQLPHYPKQQFSLRFPNMSQLA
VDLLEKMLVFDPNKRITVDEALCHPYLSSLHDINDEPICPMPFSFDFEQPSFNEENIKEL
IWSETLKFYPDLSN
>EUC01764-RA [mRNA] [translate_table: standard]
MQSDHRKKNSAEMDFFSEYGDASRYKIQEVIGKGSYGVVCSALDTHTGEKVAIKKIHDIF
EHISDAARILREIKLLRLLRHPDIVEIKHIMLPPSRRDFKDIYVVFELMESDLHQVIKAN
DDLTREHYQFFLYQLLRALKYIHTANVYHRDLKPKNILANANCKLKICDFGLARVAFNDT
PTTIFWTDYVATRWYRAPELCGSFFSKYTPAIDIWSIGCIFAEVLTGKPLFPGKNVVHQL
DLMTDLLGTPSMDTISRVRNEKARRYLTSMRKKQPVPFAQKFPNADPLALRLLAKLLAFD
PKDRPTAEEALADPYFKGLAKVEREPSCQPITKMEFEFERRRVTKEDIRELIFREILEYH
PQLLKDYVNGTERANFLYPSAVDQFRKQFSHLEDGGGKSGQVVPLERKHVSLPRSTIVHA
NPIPPKEQPNVAKDRQILEESRGRTSQPPQRTMPMAKPGKVVGSVGSYENGSVAGETRDP
RTFSRSSGIHNQVITPEYYYNRGKQELSSQNRRVPQCGMAAKLAPDIAINIDTNPFYMTR
VGVMKMDRVEDRVEIDTNLLQVRAQYSGGIGGAAAAAAAAVAHRKVGSVQLIRIRSEPDP
WKTRQLKSSQITERWSVAKIDQALKDLSDTNLRELPSSVGNLRSLCYLDVSETPYQMVTR
VESIDHLGKLLTLRLRGCADLT
>EUC00315-RA [mRNA] [translate_table: standard]

MEDTRDESGTSEQRPSCATWWSSDFMEKFASVSVDSKEEALNRKVSNSNNMYDRFSSETA
SQILWRTGMLVEPIPNGFYSIIPEKTLKELFDDIPSLEELHALELEGLRADIILVDAEKD
KKLSMLKQLIVALVKGLNSNPAAMIKKIAGLVSDVSKRPNVELSPARAVLEEASYALENR
GVQMLGQIKHGSCRPRAILFKVLADTVGLESRLMVGLPNDRDPECLESYKHMSVVVVLNS
VELLVDLMRSPGQLIPLSAKAIFMTHISAAGESDSAENDSCDSPLEPNSPLYGFSERVDP
ESAEKDDNLQYEQRLEASSNVPGHSLRNRMLRSSTSIDRRMSLSHSEPNIATTFWRRSRR
KAIAEQRTASSSPEHPSFRARGRSMLSGDRQTFRNYSDHIAASSYRSEGASTLETRRLRR
RSISITPEISDDIVRAVRAMNETLKQNRLLREQGDNRSDPYSSNDGNHAADLQKNVSGSH
DGTSGERLSSFARISREHMSSQKAMSLPSSPHELRSRSSDYLVNNDEMVAAWNKILESPM
FENRPLLPYPEWNIDYSELTVGTRVGIGFFGEVFRGTWNGTDVAIKVFLEQDLTPENMED
FCNEISILSRLRHPNVILFLGACTKPPHLSMVTEYMEMGSLYNLIHLSGQKKKLSWRRKL
KMLRDICRGLMCLHRMKIVHRDLKSANCLVNKHWMVKICDFGLSRIMTDGAMRDSSSAGT
PEWMAPELIRNEPFTEKCDIFSLGVIMWELCALNRPWEGVPPERVVYAVANEGSRLEIPE
GPLGRLIADCWAEPHERPSCKDILTRLLDCEFSLC
>EUC06431-RA [mRNA] [translate_table: standard]
MAVFLLKMPSAQSDQADRDAEPFVEVDPTGRYGRYSVLLGSGAVKKVYRAFDQEEGIEVA
WNQVKLRKFSEDQAMKDRLFSEVRLLRDLRNKNIIALYSVWTDEERGMLNFITEVCTSGN
LREYRKKHKQVSMKALKKWSKQILEGLDYLHTHEPCVIHRDLNCSNVFINGNVGQVKIGD
LGLAAIVGKNHSAHSVLGTPEFMAPELYEEDYNELIDIYSFGMCLLEMVTLELPYSECDS
IAKIYKKVTSGVRPQSMNKVKDPKVKEFIEKCLAQPRARPSASDLLKDPFFEGIDDEDDD
EENNNNGDVS
>EUC03449-RA [mRNA] [translate_table: standard]
MGVNGRRSNYTLLSQIPDEQHHQQPPAKVAAVAAQYYESQIMGKSDRGFDWELVDQRVVP
PQSRMGATAFSGTIGSQRHSSGSSFGESSISGDYYVPSLSNPDGFVQLHDDGGVELLLNT
AEGGGGGGSSSTKSWAQQTEESYQLQLALALRLSSEATCADDPNFLDPVTDESMARSSSS
SASAEAMSHRFWVKGSLSYLDKVPDGFYLIHGIDPCIWSVCVDFEENGRIPSIESLKNLD
PGAESSIEVILIDRFSDPSLKELQNRIHTLSCSCITLNEVVDHLAKLVCSHMGGVSHIGE
DHLIPNWKECTDELKDCLRSVVLLIGSLPIGLCRHRALLFKVLADTIGLPCRIAKGCKYC
TRDDASSCVVRFGFDREYMVDLIGNPGCLCEPDSLLNGPSSISISSPLCFPRLRQVEPPI
DFRSLAKQYFADSLSLNLVFDDSSAGDVQHQHLLPGTSGNNDVSHSALLPLNAWQEGFNR
DSPKSDFSRNIPPIGHTDDVQQSLPSNDLRFGMEDLDIPWSDLVLKEGIGVGSFGNVHRA
DWNGCDVAVKILMEQDFHEERVNEFLREVAIMKRLRHPNIVLFMGAVTQPPNLSIVTEYL
SRGSLFKLLHKPGGREVLDERRRLLMAYDVAKGMNYLHKHNPPIVHRDLKSPNLLVDKKY
TVKVCDFGLSRLKANTYLSSKSLAGTPEWMAPEVLRNEQSNEKSDVYSFGVILWELVTMQ
QPWRNLNAAQVVAAVGFQYKRLEIPKDLNPKVAAIIEACFANEPWKRPSFASIMESLRPL
TKVPTPLSMPSLT
>EUC03396-RA [mRNA] [translate_table: standard]
MNVIQKADGSRRIVLSEPTGSTAEVLLHGGQVVSWKNERREELLFMSSKATTKPSKAMSG
GVSVCFPQFANLGSVEQHGFGRNRLWSVDNDPSPLLPANNQSTVDLILKYAEEDLKTWPH
RYELRLRISLSANKLTLIPRMRNIDNKSFSFMFGLRNYLSVSDISEIRVEGLETLDYFDN
LLRRERYTEQTDAVTFDAEIDRVYLSTPTKIAMIDHEKKRTFVLRKDGMPDAVVWNPWDK
KAKALPDLGDDDYKTMLCVDSGVVETSVVLKPCEEWKGFQELSTVSSSYCSGQLDPRKFI
GACKEPIMVIVTELLLGGTLRKYLLNMRPRCLDRGVAIGFALDIARAMECLHSHGIIHRD
LKPENLLLTADHKTVKLADFGLAREESLTEMMTAETGTYRWMAPELYSTVTLRHGDKKHY

NHKVDAYSFAIVLWELIHNKLPFEGMSNLQAAYAAAFKNVRPNVENLPEDLGLIITSCWK
EDANERPNFSEIIQMLLHYLSTITPIMEPAVPPRIICTSKNSVLPPESPGTSSLMSKRDD
SGDTPKTPMEREPGGGLFFCFRHCY
>EUC03168-RA [mRNA] [translate_table: standard]
MDLAEGVGESSSPPRNFGSFVGYDIRTHVYSRLVESGNEEAMFPEFREQLEAHFNRLPPS
YGLDINIDRVEDILLHQRLLDLAKDPDKRPIFHARLLEKFWSRAKADDGQTLSVLSTPRL
SCNVDNDGSVPSLDRLEDLNLDVKKNSLKMETEVPAEDFPRRQEVPQIPIHEVIFSTIDR
PKLLSQLSALLSDIGLNIREAHVFSTTDGYSLDVFVVDGWHAEDTDSLCEAMRNAIARSE
GSWSGSSHSQSAKEKTIVLPPKSGDWEIDRRLLKMGERIASGSCGDLYRGLYLGQDVAIK
VLRSEQSNDTLEDEFAQEVAILREVQHRNIVRFIGASTRSPNFCIVTEYMPGGNLYDYLH
KNHIILKLPQLLKFAIDVCRGMEYLHQNHIIHRDLKTGNLLMDTHNVVKVADFGVARFQN
QRGVMTAETGTYRWMAPEVINHQPYDQKADVFSFAIVLWELVTAKVPYDGMTPLQAALGV
RQGLRPDLPTNTNPKLLDLMQRCWEATPVNRPSFSEIRIELEELLEVQDSSEQANGS
>EUC03132-RA [mRNA] [translate_table: standard]
MPHRTTYFFPRQFPDRKFDASSKFQLDHESKNAKDSENDRKASKQNTDVTVGGNSAASAK
DSTASDRFTGDRIHEKQLAAFVNWLGKKKVGVSGHVKVRLDSTDEDREHLLPPEADEPPA
TEVVRDRNFEREISSQKSPIGSGSSYGGPPAATEVVVGAGRDRGFDRQMSLQRLSSGSSY
AGSLFSGTTVDGNWSSGVKDSQMSTTREEEEEKTDSLAQRSKESYYLQLTLAKRLTQQAT
LAYEPILLQECKTDALGGYDAETVSYRLWVSGSLSYSDKISDGFYSILGMNPYMWVMCND
LEEGTRLPPLMALKAVEPSDTSMEVVLIDRHGDSRLRELEDKAQELYFAAENTLVLAEKL
GKLVAVYMGGSFPVERGDLHMRWKLVSKRLKDLQKCIILPIGSLSMGLCRHRAILFKKLA
DYIGLPCRIARGCRYCVADHRSSCLVKIEDDKKFSREFVVDLVGEPGNVHGPDSSINGGP
LSSVPSPFQISHLKEVQQPYMDSSEMSCAIINSKHKSALSESSPYSGDWEEGRQRMQDIG
CVHENRDEALYGLVGQAHVQSESPEGVAVVSAAISQGEYSAAKGDKIVIRQAYREEIVVS
ESPVTNAALNQSPAVIISDKSNPMEIKSGVENQGNFNTVIIPRYLNLEPSLAMDWLEISW
EELHIKERIGAGSFGTVHRAEWHGSDVAVKVLTVQDFLDDQLKEFLREVAIMKRVRHPNV
VLFMGAVTKRPHLSIVTEYLPRGSLFRLIHRPASGEILDQRRRIRMALDVAKGLNYLHCL
SPPIVHWDLKSPNLLVDRNWTVKVCDFGLSRFKANTFISSKSVAGTPEWMAPEFLRGEPS
NEKSDVFSFGVILWELVTMQQPWSGLSPAQVVGAVAFQNRRLSIPPGTSPVLASLMESCW
ADDPAQRPTFASIVDALKKLLKSPTQLIQMGGP
>EUC25435-RA [mRNA] [translate_table: standard]
MQQDQRKKSSKEIEFFTEYGDANRYKILEVIGKGSYGVVCAAIDTHTGEKVAIKKITDIF
EHISDAIRILREVKLLRLLRHPDIVEIKRIMLPPSRREFKDIYVVFELMESDLHQVIKAN
DDLTHEHHRFFLYQMLRALKYMHTANVYHRDLKPKNILANANCKLKICDFGLARVAFSDT
PTTIFWTDYVATRWYRAPELCGSFFSKYTPAIDIWGIGCIFAEVLTGRPLFPGKSVVHQL
DLITDLLGTPAADTISGVRNEKARKYLTNMRKKYSVPFSEKFPNADPLALPLLRRLLAFD
PKDRPTAEEALADPYFKGLAKVEREPSSQPISKLEFEFERRKVTKEDIRELIFQEILEYH
PQLLKDYMAGNEGTHFLYPSAIGQFRRQFAYLEENFGKSGPVIPPERKHVSLPRSTVNSS
VIPPKMQQNSGAFDNRRTSEESCSSNGVRVTDAINTNAMKATRPPPPRVPTAKPGRVVGP
VLPYESGRNNPRVFVRNAGPPPPPQTISPHYCFRSNNQEKSSSGQEVKHQSISGKPGPGM
GMEMNTTPYYQTQTKPSQLNNQIALNAKLFQAQSQFGAVGAAAVAVAAHREAGVVQFGL
>EUC23670-RA [mRNA] [translate_table: standard]
MATPVEPPNGVKSPGKHYFSMWQTLFEIDTKYVPIKPIGRGAYGIVCSSVNRETNEKAAI
KKINNAFENRIDALRTLRELKLLRHLRHENVIALKDVMMPIHRRSFKDVYLVYELMDTDL

HQIIKSSQALTNDHCQYFLFQLLRGLKYLHSANILHRDLKPGNLLINANCDLKICDFGLA
RTSNGKGQFMTEYVVTRWYRAPELLLCCDNYGTSIDVWSVGCIFAELLGRKPIFPGTECL
NQLKLIINILGSQREEDLEFIDNPKARKYIKSLPYSLGTPFSRLYPHAHPQAIDILQKML
VFDPSKRISVTEALQHPYMSPLYDPSSNPPAQVPIDLDIDEDLSEEMIREMMWQEILHYH
PEVAASQS
>EUC24332-RA [mRNA] [translate_table: standard]
MKKGNLAPNLKLSLPPPDEVSKFLTKSGTFMHGDLLVNRDGVRIASQAEVEIPSLIHPSD
NQLSLADFDAVKVIGKGNGGIVRLVQHKWTEQFFALKVIQMNIEESARKQIAQELKINQS
SQCPNVVVCYQSFYDNGAISIILEYMDGGSLADFLKKVKTIPEPYLAAISKQVLKGLWYL
HHEKHIIHRDLKPSNLLINHRGEVKITDFGVSTIMENTSGQANSFVGTYNYMSPERIVGT
KYGYRSDIWSLGLVLLQCATGHFPYSPPEGEEGWVNVYELMETIVGQPQPCAPSNNFSPE
FCSFINACVQKDPKARQSANELLAHPFISMFDDMDIDLASYFTNVGSPLATL
>EUC21870-RA [mRNA] [translate_table: standard]
MEWTRGPTIGHGSSAAVYLATGASGELFAVKSAQLSRSSFLQREQKILSQLRSPHVVGYL
GFDVTCEKNEHVYNLLMEYVSGGTISDAVKNRGGSLDEAMIRLYSHQILQGLDYLHSNRL
VHCDIKGKNLLIGKDCVKIADLGCARFVEDGDSAAEASMFSGTPAFMAPEVARGEDQGYP
ADVWALGCTLIEMATGSNPWPELNDPVSALYRIGYSGDLPEFPRWLSEVAQDFLSKCLKT
NPKNRWTAEELLKHPFVADCEQKLETFTRNSPTAVLDQLFWDSLEASESSPDQTQISSHL
NSPAERMRQLIGDASSSNSDLPKWAEEEDWITVRNNHTEESTQFPQLNADLEDENRLFSE
TESLLSSTVNQEELVSSMIVFN
>EUC21989-RA [mRNA] [translate_table: standard]
MKNFQWFKQIANNGKLERRLSLGEYKRAVSWSKYLVSSGGEIKGEGEEEWSADMSQLYIG
NKFASGRHSRIYRGVYKQMDVAIKLISQPEEDGDLASLLEKQFTSEVALLFQLKHPNIIT
FVAACKKPPVFCIITEYLSGGSLRKFLHQQEPHSLPLNLVLKLALDIAHGMKYLHAKGIL
HRDLKSENLLLDEDMCVKVGDFGISCLESQCGSAKGFTGTYRWMAPEMIKEKHHTKKVDV
YSFGIVLWELLTALTPFDDMTPEQAAFAVCQKNARPPLPCSCPKALQQLINQCWSSNPVK
RPHFREIVSILESYAGLLEQDPEFFASYEPPDDRSSLRFVPKCISACRSSAAAF
>EUC21992-RA [mRNA] [translate_table: standard]
MKENNDGFVRADQIDLKSLDEQLERHLNRAWTMEKNKKSHDSDSGTTAAAAVSAAYSTSR
TSRQREEWEIDPSKLIIKGAIARGTFGTVHRGIYDGHDVAVKLLDWGEEGHRTDAEIASL
RAAFTQEVAVWHKLEHPNVTKFIGATMGSSELKIQTENGLVGMPSNVCCVVVEYLAGGAL
KSYLIKNRRRKLAFKVVVQMALDLARGLSYLHSQKIVHRDVKTENMLLDKTRTVKIADFG
VARVEASNPNDMTGETGTLGYMAPEVLNGNPYNRKCDVYSFGICLWEIYCCDMPYPDLSF
SEVTSAVVRQNLRPEIPRCCPSSLANVMKRCWDANPDKRPEMDEVVSMIEAIDTSKGGGM
IPGDQPQGCLCFRKHRGP
>EUC00773-RA [mRNA] [translate_table: standard]
MEWVRGEVIGHGNFGTVNLAIPRSQNPQIPPLMAVKSCSVSNSASLVNEKSVLDALNGCS
EIVGCFGDGLSFENGEKLYNVFLEYASGGALADLIKNSGNIPLPEKDARSYTRSILRGIH
HVHKSGYVHCDIKLQNILLFSSEVGCDDPVKIADFGLAKKSGEKQSSSGCELRGTPLYMA
PETVTGGEQEPPSDIWALGCVLAEMVTGSPAWRCREKSDVAGLLMRIGVGEELPEIPGKL
SAEGKDFLGKCFVKDPRKRWTAEMLLNHPFVANSDDDREAVSSKDAQEILWTSPRCPFDF
PGWVSPVQSPATSLFSSSPSSLSDPWSDSFSWSPSNSPAEKLRQLTTELKPDWSFSESWI
TVR
>EUC17818-RA [mRNA] [translate_table: standard]

MPPWWKASTKETKKKTGKESFIDTLHRKFKIPSESKSTSRSGGSQRLGRDTISERGSQSR
PQSRSPSPSKHVSRCQSFAERPKAQPLPLPGLRPAGVVRTDSGISVSAKQRQERGSRPSL
FRPLPRPACIRNKPDPTDLDGDLVTASFSSEGSIEIDDPADSRQRSPLVTDSATGNRTTT
CSPSRMTVKDQTPVVQTNSVEALRPANPLFNNHISPSSPKRRPLNGHMTNLQVPCHAAFS
SAPDSSMSSPSRSPMRAFGTDKVTNSTFYAGKPYPDLPLLGSGQCSSPGSGQNSGHNSMG
GDMSGPLFWQPSRGSPEYSPIPSPRMTSPGPSSRIQSGAVTPLHPRAGGAASESQTSWPD
DGKQQSHRLPLPPVTVPHSSHFSHPNSAATSPSLPRSPGRAENVTSPGSRWKKGKLLGRG
TFGHVYVGFNSESGEMCAMKEVTLFSDDAKSKESAKQLAQEIALLSRLRHPNIVQYIGSE
TVGDKLYIYLEYVSGGSIHKLLQEYGQFGESAIRSYTQQILSGLAYLHAKNTVHRDIKGA
NILVDPSGRVKLADFGMAKHITGQSCPLSFKGSPYWMAPEVIKNSNGSNLAVDVWSLGCT
VLEMATSKPPWSQYEGVAAMFKIGNSKELPAIPDNLSDDGKDFVRQCLQRNPLHRPTAAQ
LLEHPFVKSAAPLEKPVVGSEPSDLPGVSNAVKSVGIGNASNISNVDLERLAIHSSRVSR
SNFHTTDIHIQRNISCPVSPIGSPLLYPRSPQHFNNGRMSPSPISSPRTTSGSSTPLTGD
IPFHQLSQSAYLHEGCFGSLPKPNNNPFTNGLPYCDPNPNNIFRGSHAFPSYENDSFGKR
FGRTPHGELYDGQSILADRVCHQLLGDHVKLNPSLDLNPCLNGS
>EUC21207-RA [mRNA] [translate_table: standard]
MKNFLRKLYISGGGLPDHQHPPVISNPLSTPPETTEMHPSCSASSPSSTSSSLSSSALVR
RTGAVNSAAENSNPADPNFFEEEFQIQLALTISVSDPDAREDSETAQINAAKQISLGFSP
SGSVVEFLSLRYWSQKLINYDEKVMDGFYDVYRITSNLVGPGKLPPLVDLEAISVSENVD
YEVTFVVYRMNDSELRQLEERVYHMSMEWKNLKNGLTMSGLIQKLADLVVDRMGGPVTDVE
EMSKRWRSRSYKLRNSLQSIIFPLGCLGVGLSRHRALLFKVLADKIDLPCMLVKGSYHTG
TDDGALNVIKVDNGSEYIIDLMGAPGTLIPAEIPSSHRQLAGLNIRSIASIADIVRDSSS
AFDKERETVTVSPDLKGIAKSCSSISGKESLVGNQIKVDDRSIVEKNLTDIFTYGKLLPA
RYRLNEGTLCKKVSTAQEMQVKDVSEHVISAAKHPEFAQKLHAVLLEPRAPRPPDLFSEI
NPSERGEEISLGKLQYHPVLSLVNNEMSLTSTGVDWAFSSVHSDSKWKPSVDDLATEQKG
FKCSATYPGNNLISNTTSDGFIRVNSGPSEMIQVHDASLRIDQSGIPARASDWEQPHETF
VPAEVNPLTAQPKTGLSSEDKIVKHHNNTGKDNIYIAEQLEMDKSDHLTSCTAHKKIYPM
LRDVAEWEIPWEDLQIGERIGIGSFGEVYRAQWNGTEVATKKFMNQDITGDALEQFKCEV
EIMLRLRHPNVVLFMGAVTRPPNLSILTEFLPRGSLFRLLHRPNTHIDEKRRLRMALDVA
KGMNYLHTSQPIIVHRDLKSPNLLVDKNWVVKVCDFGMSRMKYCTYLSSKSAAGTAEWMA
PEVLRNEPSNEKSDVYSFGVILWELATLQIPWTGMNPMQVVGAVAFQNRHLIIPAEVDPM
VAEIITDCWSPNLHTRPSFGHIIARLRCLHCLRRLACTDQE
>EUC17921-RA [mRNA] [translate_table: standard]
MEMPGRRSNYTLLSQIPDDHLHQQPPSKFVASDYESLPGDQNKGKCDRGFVWDLTDHRAV
QPHSRIGTVAFPGTLGIQRQSSGSSFGESSISGDFYAPSLSNPDGFAYLHDGGGELRFKA
VDGGGGSSSSKSWAQQTEETYQLQLALALRLSSEATRADDPNFLDPVPDESTSRFSSSSA
SAEATSHRFWVNSCLSYSDRVPDGFYLISGMDPYIWTVCTDLQENGRIPSIESLRAVDPG
IESLVEVILIDFLSDPSLKELQSRIQNVSSGCITTKEVVDQLAKMVCSCMGGAALIGEDD
LVPIWKKCSDDLKDCLGSMVLPIGRLSVGLCRHRALLFKVLADTIDLPCRIANGCKYCTR
NDASSCLVRFGIDREYFVDLIAKPGCLFEPDSLLNSPSSISISSPLRFFPRVRQAEPTSD
FRSHAKQYLADCQSLNLLFDDSSAGNIIDGDAGGAVYPQLSDSNYMDKNNVTSPSNSNEF
LQLPLPPINTRQKTRGRDSRPHKVFNAQTTMDSTNIAKDLVASKHMPPIWHGDVQLRVSQ
PKVDTKDMHFVDKSHLVPAKQSRQLTLDVENLEIPWSDLVLKERIGAGSFGTVHRAEWNG
SDVAVKILMEQDFHAEKVQEFLREVAIMKRLRHPNIVLFMGAVTQPPNLSIVTEYLSRGS

LFRLLHKPGAREVLDERRRLGMAYDVAKGMNYLHKSNPPIVHRDLKSPNLLVDKKYTVKV
CDFGLSRLKANTFLSSKSAAGTPEWMAPEVLRDEPSNEKSDVYSFGVILWELATLQQPWS
NLNPAQVVASVGFKGKRLEIPRNINTQVAAIIEACWANEPWKRPSFFNIMECLRPLIRPP
TPQPGHTDMSLLM
>EUC17152-RA [mRNA] [translate_table: standard]
MIDLELRHLEERVYLMSLEWQILEKGLTTTGLIQKIADTVVEKMGGPVIDAQEMMERWSA
RSYELRNDLNTIILPLGCLDVGLSRHRALLFKVLADKIDLPCMLVKGSYYTGTDDGAVNL
IKVDDGSEYIIDLMGAPGTLIPAEVPSGHLQNLGLDMRSHASIADKTKDSCPIAFKGIES
GMVSSALSGSAQFSPSNSDETSLVGMQPKRDDKNTGGKNETEMFKVGNFLPFEGSSSFGK
NVLAANELKVKDVSRYVISAAKNPNFAQKLHAVLLESGASPPLDLLFSEINPNHPEEQKA
LEMAHWLNVDAMVDRPRLFQEIPVANNDMSLIPFTGGHQVFNNVQYNIPQNVSAEEMAAK
QQDLESGVPSDTSERFVLVNSGPSEMIQADGAAVRNDSVQPSEMVARASAFSPTGLPENA
IAADDKKFLNNNGGKLLSNIEKDKGPSVNTTGTDNSIFSTHERIYPVLGEVAEWEIPWED
LQIGERIGIGSYGEVYHAEWNGTEVAVKKFMNQDISGDALIQFKCEVEIMLRLRHPNVVL
FMGAVTRPPNLSILTEFLPRGSLFKLLHRPNIQIEEKRRLRMALDVAKGMNYLHTSHPVI
VHRDLKTPNLLVDKNWVVKVCDFGMSRMQHHTFLSSNSAAGTAEWMAPEVLRNEPSNEKS
DVYSFGVILWELATLQVPWIGMNSMQVVGAVGFQDRHLHIPLDIDPTVAQIITDCWNSNP
QARPSFGQIITRLRILQRLKRESQTNQQEQQQQQRC
>EUC07535-RA [mRNA] [translate_table: standard]
MDGDDSQMNSYRILLDRFQSLETSLARLREQLDVLVQQRAVKMNFPGKGTSDSGETTSYD
GWEYVPPAFFSSSPYRKVLDHLGHAVHVSIPESGEIVYWNHSAAKLYGYDYHEVLGQGDE
ELLTDEEHYNSAKMILQRLSSGQPWSGQFPFRKKSGEIFMAIATKTPLYEDGELVGIVTV
SSDADVFNRIKSENVGQPRARRINFKKFQCHPQQQIASVPQIAASVSNLASKVLLRKCGD
DTSNNCTTSGEEAERNETNVKDVRYDRPPKAPATYSDYGSHVDKTTIDADCSGKRESTPE
FVQPSRIAAKILSKLNVRRLANLGKEKDGGIQKDDTSMHKEIANESYSAADLNAMVSEHC
TVDVDRRTQPASEKSNATGEGLAGTFCCDCSEIPKPAGQSPRSEIYLHANEFGTDLLNSK
PSEIEDAVQLPSPGEGINSSGSSSSKGDNDSNVIVDCEIFWEDLHLREEIGQGSYAVVYR
GIWNGSDVAVKVYFGNQYGEQTLLDYKKEIDIMRRLRHPNVLLFMGAVYSQEKLAIVTEL
LPRGSLYKTIHKNNQALDLRRRLRMALDVARGMNYLHHRNPPIVHRDLKSSNLLVDKTWN
VKVGDFGLSKLKNETFITAKSGRGTPQWMAPEVLRNEPSNEKSDVFSFGVILWELMTESV
PWINMNSLQVVGVVGFMDRRLEVPESLDPRVSSIIHDCWQSNPDDRPSFEDIIERMTDLI
QSVTGVFARKCSKP
>EUC09614-RA [mRNA] [translate_table: standard]
MITELFCSGSLRQYRKKHRNVDLRAIKNWGRQILKGLNYLHTHDPPIIHRDLKCDNIFVN
GNQAEIKIGDLGFATIMNQPTSRTLIGTPEFMAPEMYDEEYDELVDVYAFGMCMLELITC
EYPYSECRNPAQIYKKVTTGVKPAALQKLKDPHVKLFIEKCLVQASLRPSAMELLKDPFL
SMEETVFQFPTLPKSLNPPKIESHHSAMEAWRVKDVIQFGLKGFKVDQNSISLNLKIIDL
STGKLENSTFEFYLSSDDAPSIAVELVRERILSVNDLPVVCEMIDGMILEFVPDWEPSYG
YYKDSGLNEAMSENYSSEISFTDSSCNAMSKEFGYEHFFM
>EUC09794-RA [mRNA] [translate_table: standard]
MDSKTNGNAPKTQSLGDPEAFRELKLNGAGSVSEKNISQRSSTSISSRDMIFRADKIDLK
SLDVQLEKHLSRVWSRNTEPQRPKELWEIDPSKLDIRYLIAKGTYGTVYRATYDNQDVAV
KLLDWGEDGMATNAETAALRASFRQEVAVWHKLDHPNVTKFVGASMGTSNLKIPSKNPST
DGYIDLPSRACCVVVEYLPGGTLKNFLYKNRKKKLAFRIVIQLALDLARGLSYLHSKKIV

HRDVKAENMLLDINRTLKIADFGVARVEAQNPKDMTGETGTLGYMAPEVLDGKPYNRKCD
VYSFGICLWEIYCCDLPYPNLSFADVSFAVVRQNLRPEIPRCCPNSFASVMRKCWDVNPE
KRPEMEEVVGLLEAIDTSKGGGMIPDDQAAHGCFCFAPSRGP
>EUC20951-RA [mRNA] [translate_table: standard]
MNMKHKRLERKLDRRNAIKNIDYDASNSSTSTSTSTSFDGQPTHRTRSLDIYAHTDRSSF
RVDGIDGFDQICKSLGLSGPEDFSIPTAAWEARKARSPSELFPSSRFCDYYNSVKETTED
GLSNGIPAKVRTSDEYKKCENECSRLESGLNSARVRVSDGVSLEDDEAVYLNFPTNTAGG
RCARDGGCGIKGVRPPVLAPPPPLSRAVVDNRRSTWDLFRDFGPRDDEDIGLPSGVGLAN
SFSETDQAIEENKEVDGRLVSKQDLNGERLIASIAFTESCSNSSNDDDDDDSFSMVLEPE
YSVSPDRIFRPSIKSWQKGDFLGSGSFGTVYEGFTDDGFFFAVKEVSLLDQGSQGKQSIS
QLEQEISLLSQFKHQNIVRYLGTEKDEAKLYIFLELVNKGSLAHLYQKYHLKDSQVSGYT
RQILNGLNYLHRQNVVHRDIKCANILVDVSGSVKLADFGLAKTTTFNDVKSCKGTPFWMA
PEVVKGRNRGYGLMADIWSLGCTVLEMLTGQIPYSHLEGPMQAMFRIGRGEPPPIPDSLS
NEAQDFILKCLQVDPDHRPTAAQLLDHPFLKKSSSASLSPASPNYYGGRQ
>EUC17437-RA [mRNA] [translate_table: standard]
MDGSAQPSDTVMSEAAAAPPQSDYPHPHTHPAAGMENIPATLSHGGRFIQYNIFGNIFEV
TAKYKPPIMPIGKGAYGIVCSALNSETNEHVALKKIANAFDNKIDAKRTLREIKLLRHMD
HENVVAIRDIIPPPQRDSFNDVYIAYELMDTDLHQIIRSNQGLSEEHCQYFLYQILRGLK
YIHSANVLHRDLKPSNLLLNANCDLKICDFGLARVTSETDFMTEYVVTRWYRAPELLLNS
SDYTTAIDVWSVGCIFMELMDRKPLFPGRDHVHQLRLLMELIGTPSEAELGFLNENAKRY
IRQLPLYRRQSFNEKFPHVHPAAIDLIERMLTFDPRQRITVEDALAHPYLTSLHDISDEP
VCTTPFSFDFEQHALTEEQMRELIYREALAFNPEYQ
>EUC07090-RA [mRNA] [translate_table: standard]
MSTEFQFDKLSMDDRNVDNNEEHLVDEKALRNLDMAKECKNSDIVQHDCRKYTQDRFKGS
DMKLKETDCSSSSFYHTVAQVDPEIDDVGECEIPWEDLVIGERIGLGSYGEVYRADWNGT
EVAVKKFLDQDFSGAALAEFKREVRIMRRLRHPNVVLFMGAVTRPPNLSIISEFLPRGSL
FRIIHRPNCQMDEKRRIKMALDVAKGMNCLHTSTPTIVHRDLKSPNLLVDNNWNVKVCDF
GLSRLKHNTFLSSKSTAGTPEWMAPEVLRNEPSNEKCDVYSFGVILWELATLRLPWTGMN
PMQVVGAVGFQNRRLEISKEVDPLVGRIIWECWQTDPNLRPSFADLTVALKSLQRLVIPL
HIDQQSSPLPQEISVNLTP
>EUC07070-RA [mRNA] [translate_table: standard]
MPQDSSSSEQEPDDSDAEFVELDPSGRYGRYKDVLGKGAFKKVYRAFDELEGIEVAWNQV
KVADLLRNSEDMERLYSEVHLLKTLKHKNIIKFYNSWVDTKNESINFITEIFTSGTLRQY
RKKHKHVDLRALKKWSRQILEGLSYLHSHDPPVIHRDLKCDNIFVNGNQGEVKIGDLGLA
AILRQARSAHSVIGTPEFMAPEFYEEEYNELVDIYAFGMCLLELVTFEYPYIECTNAAQI
YKKVVSGIKPASLAKVKDPAVKAFIEKCIAQVSERLPAKELLMDPFLQSDENNGSIGRFL
QPDLYHADSITDHFEISTIPKDSVPDESRDFTLQGQRKYLNTIFLKLRIPESTGCYIRNI
HFPFDVEVDTPTAVASEMVEELDLTEQDVPTIAAMIQSEIRSCIPEWVPREISGDHTCNE
VTIADTCGSEEAQSNTSPLTNESSPHSAGFFLERMPSGRKYWCNSTKGSGGSTPLRQPSS
NLLRADSVISGDSWAEENYHSPESSKEGSSFVEEQANLPADSRFSEPDSDSDKGHQHIGG
GNNRALNDTVSDDVKIIMEKLENLLVEQRRELDELRQKQDLAVSDLLKGLHPEVRRRVCN
ICSLKIFVHKVPCAGCSIENRADLCSLRM
>EUC03978-RA [mRNA] [translate_table: standard]
MSCSEKNRAGEDLEYEKSVKGVLSNGSISTVLQPTIDERVLVDPKLLFIGSKIGEGAHGK

VYEGRYGDQIVAIKVLNCGSTSEERASLESRFAREVTMMSRVKHDNLVKFIGACKDPLMV
IVTELLPGMSLRKHLANIRPKQLDLHVALSFALDIAQAMECLHANGIIHRDLKPDNLLLT
ANQKSVKLADFGLAREETVTEMMTAETGTYRWMAPELYSTVTLRQGEKKHYNNKVDVYSF
GIVLWEILTNRMPFEGMSNLQAAYAAAFKQERPSLPEDIHNDLAFIIQSCWVEDPNMRPS
FSQIIRMLNAFQFTLPLPSPPPSPPPPIKESSNNNEASAATNIYKN
>EUC15557-RA [mRNA] [translate_table: standard]
MGSSLMGSETGDDDIAETSPDGRYIRYHDVIDRCSFKVVYKGFDQDDGKEISWCQICIDD
CVIQSTEHRRALLSEANLVKSLRHGNLVKCYQYWLDGKNKTINMVTELFSSGNLRHFRKK
HKSVGLKVIKKWGRQILRGLHYLHTQNPPIIHWDLKCDNIFINGNHAEVKIGDLGLATVL
HRGTERSIIGTPEFMAPELYNDEYNELLDIYSFGMCMLELITCECPYSECENPAEICEKV
TAGVKPVALGKVKDPKSKEFIEKCIIPSSQRPSALELLEDPYLSIENFTDSPQIPNAIPA
NLPKFDSQISIDLSSEEGNDLSVGSSTVDSVSSLEMWRSNETVKLRLSGTKVDQKSITFN
MKIGSLSASTTEVCEFAFDVCADDALTVAVEMVRQEMLSFKDVALAVELIDCMLLELVPS
WKPSYGYYVGPKS
>EUC24464-RA [mRNA] [translate_table: standard]
MVSDGGTVNLLSRSYGVYNINELGLQKCPSWPVDDADESEKTYRCASSEMRIFGAIGSGA
SSVVQRAIHIPTHRIIALKKINIFEKEKRQQLLTEIRTLCEAPCYQGLVEFYGAFYTPDS
GQISIALEYMNGGSLADIIRVRKCIPEPVLSHMVHKLLHGLSYLHGVRHLVHRDIKPANL
LVNLKGEPKITDFGISAGLENSMAMCATFVGTVTYMSPERIRNENYSYPADIWSLGLALF
ECGTGEFPYTATEGPVNLMLQILDDPSPSPSKQHFSAEFCSFVDACLQKDADARPTAEQL
LSHPFITKYKDAGVDLATFVCGVFDPTQRMKELADMLTIHYYLLFDGPDDLWQHTKNLYN
ERSIFSFSGKESIGSNDIFTRLSSIRSTLAGEWPPEKLVHVVEKLQCRAYGQNGIAIRVS
GSFIVGNQFLICGEGIQVDGLPNFRDLSVNIASRRMGTFQEQFVVEQGNVIGCYFIAKQE
LYVAQVEM
>EUC24477-RA [mRNA] [translate_table: standard]
MVIVTELLLGGTLRKYLLNMRPRCLDMRVAIGFALDIARAMECLHSHGIIHRDLKPENLL
LTADHKTVKLADFGLAREESLTEMMTAETGTYRWMAPELYSTVTLKHGEKKHYNHKVDAY
SFAIVLWELIHNKLPFEGMSNLQAAYAAAFKNARPSAEDLPQDLALIVTSCWNGDPNSRP
NFGEIIKMLLHYLSAIHPPEPIIPPRIFKSENAVLPPESPGTSSLMAIRDESPKTPVENE
PGSFFFCFNHCY
>EUC24537-RA [mRNA] [translate_table: standard]
MDNSNTSTPPAEELLKKIQELEAGHAHLKQEMSKLIISTDHRKSERQRSHSISPQRTAVP
SRRRVGGEGGFDGGTVAAWKKGSASFRHSSPLQRESRNREACGGGVGGGCGSGPAAVKFT
DKQYLNILQSMGQSVHIFDLNRQIIYWNRAAELLYGYSAAEAHGKDPIELLTDSQDYAVA
DDIVRRVAMGESWTGQFPVKNKQGDKFVVIATNTPFYDDDGTLVGIICVSTDSQPFQEIR
GPLSSTKHGESGFSRPRSIASAKLGLDPQQPLQTAIASKISNLASRVSSKVKSKIKTGEN
NMVGEGGSGDSHHSDHGFLDDHREDANSSGASTPRGDINQSPFGVFSQHARDSGDDSEGK
PGISRIISSKAEAWMGKKGISWPWKGNEGGSEAKTTRFVWPWLHNEQENDPGPPKSYGSF
TRHENQVGESNRNANNEASGSWSSFNVNSTSSASSCGSTSSSALNKVDMDSDCLDYEILW
EDLTIGEQIGQGSCGTVYHALWYGSDVAVKVFSKQEYSDDMILSFRQEVSLMKRLRHPNV
LLFMGAVTSPQRLCIVTEFLPRGSLFRLLQRNISKIDWRRRVQMALDIAQGMNYLHHYNP
PIIHRDLKSSNLLVDKNWTVKVADFGLSRLKHETFLTTKTGKGTPQWMAPEVLRNEPSDE
KSDIYSYGVILWEIATEKIPWENLNSMQVIGAVGFMNQRLEIPNDVDPQWASLIESTWHT
DPRCRPTFQEILEKLKDLRRQFAIRVQAARSAAGESSSRKEL

>EUC09325-RA [mRNA] [translate_table: standard]
MHWWQSAFSSPSPSPSPSSSPPSKSDNNVVNRFNIFSTRGRRLRSHHRRLTRAKKLRHLS
ENEAERRLPPIDPSELWRLPSTLEHSVRSSSQTTAAAPQPLPLPELGQLLRRDANLVSCS
NPGDRHLRSPKDVTGGGGDEREKCNVVNGDGVPSGSRIASQDAQSSTEQPKSRHRRKSPQ
PVNGGARNKGNYRISIPTSAPTSPFSSPSPQRNTPDFYTSYYMTPPIFQVWSAPELPPSD
MTVGQGFPYLMSPEKTAFSVDSSPLHSPRLSPHPTTRSPCGPPSPPTARRESNNQVNIHP
LPRPPVATIPSPPALVPQTTTKKGIRPLKRRWKKGKLIGRGTFGSVYIASNRETGALCAM
KQVEILPDDPKSAECIKQLEQEIKVLSKLKHPNIVQYYGSETVRNRFYIYLEYVHPGSIT
KYIHDHFGAITEPVVRNFTRHILSGLAYLHSMKTIHRDIKGANLLVDAYGVVKLADFGMA
KHLTGQGTNLSMKGSPYWMAPEVFLIKRKLQENLHLLVLNMTIPKYEWHQREITEQLDWC
FLFSGEAGRQPPHETSDLMVRSFGSYANASI
>EUC10175-RA [mRNA] [translate_table: standard]
MKEGSDGFVRADQIDLKSLDEQLERHLSRAWTLEKNKNQHDSESATVSSDSSSAVNNNNA
VRRRQEWEIDPSKLIIKSVIARGTFGTVHRGVYDGQDVAVKLLDWGEEGHRTEAEIASLR
AAFTQEVAVWHKLDHPNVTKFIGATMGSSTLNIQTENGHIGMPSNICCVVVEYLPGGALK
SYLIKNRRKKLAFKVVVQMALDLSRGLSYLHSQKIVHRDVKTENMLLDKSRTVKIADFGV
ARVEASNPNDMTGETGTLGYMAPEVLNGNPYNRKCDVYSFGICLWEIYCCDMPYPDLSFS
EVTSAVVRQNLRPEIPRCCPSSLANVMKRCWDANPDKRPEMDEVVFMLEAIDTSKGGGMI
PIDQPQGCLCFQKYRGP
>EUC10801-RA [mRNA] [translate_table: standard]
MASLFSSDHGLESSRYLLRFLYQLAYQSVFPPFPETDQEPDDSDVAEFVEVDPSGRYGRY
KEVLGKGAFKKVYRAFDELEGIEVAWNQVKVVDLLKHPEDLERLYSEVHLLKTLKHKNII
KFYNSWVDSKKESINFITEIFTSGTLRQYRKKHKHVDLRALKKWSRQILEGLFYLHSHDP
PVIHRDLKCDNIFVNGNQGEVKIGDLGLAAILRQARSAHSVIGTPEFMAPELYEEEYNEL
VDIYAFGMCLLELVTFEYPYVECANAAQIYKKVTSGIKPASLAKVKDPAVRAFIEKCIVE
VSERLPAKDLLMHPFLQSNDDNGSIGRSLRPNPNHTDNLIETHKDSLLDGSRDFSVESQR
KDLNTIFLKLRIADSSGQIRNIHFPFDIEVDTLTAVASEMVEELDLTDQDVYAVASMIEL
EIQSCIPNWVPREFSGDEVSNADGGGSAAISAFESQPDASPHSPSSNASPRSPGLVLERL
PSGRKYWCDSPKSRPGPSNLSPSNLSQSNSMNPRENNESPDSNEELELEIVEKLENLLVE
QRREMEELKMKHELALSDFLREIPHEIRIAVCKMCDPKVSDHEKTHFETRFSLKT
>EUC01374-RA [mRNA] [translate_table: standard]
MKTMKPLKELKLSVPAQETPITSFLTASGTFHDGDLLLNQKGLRLISEEKEPCPSETKEI
DLQFSLEDLETIKVIGKGSGGVVQLVRHKWVGTLFALKVIQMNIQEDIRKQIVQELKINQ
ASQCSHVVVCYHSFYHNGAISLVLEYMDRGSLVDVIRQLNTILEPYLAVVCKQVLQGLVY
LHHERHVIHRDIKPSNLLVNHKGEVKITDFGVSAMLANSMGQRDTFVGTYNYMAPERISG
SSYDYKSDIWSLGMVILECAIGRFPYIQSEDQQRWPSFYELLEAIVDSPPPSAPPDQFSP
EFCLFISACIQKEPTDRSSSLDLLNHPFIKKFEDKDIDLGILLPEGPLQCQHPRGPYLES
>EUC01391-RA [mRNA] [translate_table: standard]
MADVNAAAGQYPEFPAVPAHGGQYVQYNIFGNLFEITNKYRPPIMPIGRGAYGIVCSVLN
SETNEMVAIKKIANAFDNYMDAKRTLREIKLLRHLDHENVIALRDVIPPPLRREFSDVYI
ATELMDTDLHQIIRSNQGLSEEHCQYFLYQLLRGLKYIHSANVIHRDLKPSNLLLNANCD
LKICDFGLARPNTENEGMTEYVVTRWYRAPELLLNSSDYTAAIDVWSVGCIFMELMNRKP
LFPGKDHVHQMRLLTELLGTPTDADIWFMQNEEARRYIRQLPRHPRQELARVFPHVHPLA
MDLVDKMLTFDPTRRITVEEALAHPYLARLHDIADEPVCSKPFSFEFEKQGLEEEQIKDM

IYQESIALNPEYA

>EUC26609-RA [mRNA] [translate_table: standard]

MMIEDSESCGSRASESSPANTRQQRKRQEVYNEILRRLRESNNQEAKEPGFDDELWAHFN
RLPSRYALDVNVERAEDVLTHKRLLNLAHYTANKTVFDVRLVQVAPISDGNSPDSVHSRS
PRKEVVQSIHPPPAFGSSPNLEALALEASKAELQDGDSAANTHAKFSRPMHEITFSADDK
PKLLSQLTSLLAEVGLNIQEAHAFSTIDGYSLDVFVVDGWPHEETVLLRTTLEKEILKTE
MHSWPNQHSFSSLGEQEQPGRIKQEPDFLTIPNDGTDVWEIDPRFLKFENKVASGSYGDL
YKGTYCSQEVAIKVLKAERLDLDMQREFAQEVFIMRKVRHKNVVQFIGACTKPPSLCIVT
EFMSGGSVYDFLHKRKGIFKLPYLLKVAIDISRGMDYLHQNNIIHRDLKAANLLMDENEV
VKVADFGVARVKAQTGVMTAETGTYRWMAPEVIEHKPYDHKADVFSFGVVLWELLTGKLP
YQYLTPLQAAVGVVQKGLRPTIPKNTPPTLAELLERCWEKDPTLRPNFCEIIEILQRLVK
EVGDEAEERRKDKSSGGFFSVLKRAHH

>EUC24974-RA [mRNA] [translate_table: standard]

MQDFIGSVRRSLVFKPSGDLDDGVGGGFGGFVEKIGSSIRKSRIGLFQKPQFQALPPIAK
PDRVKAKKHESSPIRWRKGELIGCGAFGRVYMGMNLDSGELLAVKEVSIAANSASNEKAQ
AHIRELEEEVNLLKNLSHPNIVRYLGTARENDSLNILLEFVPGGSISSLLGKFGSFPESV
IRMYTKQLLLGLEYLHKNGIMHRDIKGANILVDNKGRIKLADFGASKKVVELATINGAKS
MKGTPYWMAPEVILQTGHSFSADIWSVGCTIIEMATGKPPWSQQYQEVAALFHIGTTKSH
PPIPDHLSLQAKDFLLKCLQKEPNLRPTASDLLQHPFVTGYYREAHHVFRSSVTESFGNQ
NAVLGSDLTKSMNPEIRTTCSGLKDVGDMSSVRCSTIYPEKFSGIGSMWTSTNNDDDMCC
IDDKDDLMIGSSMKFTSTLLSHDLNKSFNPMSEPNDDGQCKFDGSPEIERSEANLYASQD
KDFTFMNGPLVAEDEDELIDSKIKAFLDEKALDLKKLQTPLYEEFYNSLNAISSPSHIGN
GEKENISNNMNLLPPKSRSPSRVLSRRLSAAVDFAYNASSSPGGRSKRNISNLQSSSTSL
APQEVSSPQINEGKGVLGSQQEAISPSMSFSERQRKWKEELDEELARKREMLRQAGKVKT
LSPKDRIMNRQIDRLREDPTGFCEGRRTTSSPKDQTLNWPK

>EUC24948-RA [mRNA] [translate_table: standard]

SPQSNEEFCLFISTDKNMLDKDFFTEYGEACQYEILEVVGKGSYGVVAAAVDTHTGEKVA
IKKMKDIFEHVSDATRILREIKLLRLLRHPDIVEIKHIMLPPSPREFKDIYVVFELMESD
LHQVIKANDDLTPEHHQFFLYQLLRGLKYMHTAHVFHRDLKPKNILANADCKLKICDFGL
ARVSFNESPSAIFWTDYVATRWYRAPELCGSFCSKYTPAIDIWSIGCIFAEMLTGKPLFP
GKNVVHQLDLITDLLGSPAAESVARIRNEKARKYLSSMKKKAPVPLSQKFPNVDPLALRL
LERLLSFDPKFRISAAEALADPYFRSVSNVEQEPSRQPISKFEFEFERRKLTEDDVRELI
YGEILEYHPQMLQEYLRGVDKTSFMYPSGVDQFKQQFARLEEHYGKGEKSTPPRRQYTSL
PRERVRALEDDGVDESSDLERRDDVAFMRMSLQSPTTSRGSKEVEINDPSAVAAQDGLGV
SKYSTRAMSRSPTVSASTCVGMQRRHLQGAISEVF

>EUC20701-RA [mRNA] [translate_table: standard]

MYKSRLGEPVELGYIEMDPSSRYGRFREILGKGAMKTVYRAFDEVLGMEVAWNQVKLNDV
FQSPDELQRLYSEVHLLNNLDHESIMKFHTSWIDVDRRTFNFITEMFTSGTLREYRQRYK
RVDMRAVKNWSRQILRGLAYLHGHSPPVIHRDLKCDNIFVNGHLAQVKIGDLGLAAILKD
SNHAHSVIGTPEFMAPELYEEDYDERVDVYSFGMCVLEMLTSEYPYNECSNPAQIYKKVT
SGKRPEAFHRIHDGEARLFVGKCLESASKRPSAEELLMDPFLAADDEDEHEAIPDDRNSY
VLNGGRTKEVPQISILGDSGSPKRSDMRITGTIDPQDDTIFLKVQISDEDGEGRNIYFPF
DISSDTALDVAIEMVKELEIRDWDSLEIAEMIDEQISTLVPTWKCNHCQQHSFGYHYQEE
DEYEEDNDDDDDRTHHPFYSLSSHSSSRASLPPALFTSCHDWLQEMKMDRDCYRHWNDGR

DKLTRIQSFVDVRRQLLHRTLVEEINKRRMFKTVGAVENIGFQDPCHGFHRKTRI
>EUC15935-RA [mRNA] [translate_table: standard]
MAGTETDAGRYRVLVNRFRSLEVSQAKLKEQLGVLAQETGVEDFWEEVVPEAGEGKSSYP
GWSRIPGVFLSGIPYRQVLDCMGHAVHIRRADSGVIIYWNPSAEKLYGYKKSEVLGRTVE
ELLIDDNYYSSTQEIMERLTDGQSWSGQFPLKKRSGKVFMALVTKTPLYEDGVLVGVITV
SSDAAVFNKINSETLSKTQDQDHANVNQSGFQRMNLKNIQWPPRAQMASSISNLASKMLS
QKREDYRWNEYTNTATERKEIVVEGLEAQDATKATNGKDGGESYRKNESTFELVQPSKMA
AKVLPELCNGNLQKDEAGSILQNGSSIIEQITNDSYFPRRLQATTSDYCVVDADYSKNNF
SRGISPAVKGICPLMSAKIISADIGPGNAKIFPRCPKVPRPLNQFPRSGIQLYANEFEAD
VNDSKPTEMENALLWLPDPQKQPCSGENSGGSHGIAPSKGENESNMIIDCEIHWENLQIR
EEIGQGSYAVVYRGIWNGSEVAVKVYFGNQYSEVALLDYRKEIDIMRRLRHPNVLLFMGA
VFSQDKLAIVTEFLPRGSLFKALHKGNQKLDIKRRLRMALDIARGMNYLHCINPPIVHRD
LKSSNLLVDRNWSVKVGDFGLSRLKIATFLTAKSGRGTPQWMAPEVLRSEPSNEKSDVFS
FGVILWELMTESIPWSNLNHLQVVGVVGFMDRRLDIPEGLDPRIASIITDCWQSNPEDRP
SFQDIIQEMGNLIYRYGTIGSKKWHCR
>EUC17901-RA [mRNA] [translate_table: standard]
MAMEDNESCGSRAVESSPSTKQNRQERQRREVFNEVLSRLQDLNHEEARLTGFEDQLWLH
FNRLPPRYALDVNVERAEDVLTHKRLLSLAEDPANRPAFDIRLVQVPSTSGNASDSINLN
SPVEEDNHSFRKEGIHPPPTFGSSANLEALAHRANDSHDDDDGDRSISQIQRPMHEITFS
TVDKPKLLSQLTSLLSEIGLNIQEAHAFSTHDGFSLDVFVVDGWPHEETMQLRNALEMEI
LKTKGQFFTKQHSVSRVGDHSKAVSESFHNLVKIPTDGSDVWEIDASMLKYENRFVSGTF
FELYKGTYCSQDVAIKKNLMNDRLNADMLKEFSQEVFIMRKIRHKNVVQFIGACTQPPNL
CIVTEYMSRGNVYDLLHKKKGFFELPTVLKVAIDTSKGMNYLHQNNIIHRDLKTANLLMD
ENEVVKVADFGIARVQDHTGVMTAETGTYRWMAPEVIQHKPYDDKADVFSFGIVLWELLT
GEIPYSYLSPLQAAIGVVQQGLRPTIPKQTPPKLAELIKKCWHQDPTLRPNFSEILEILQ
KIAREVIWRWRFQMPIRELWSCKVEIVGVKTGNS
>EUC01494-RA [mRNA] [translate_table: standard]
MALVRDHRRHLNLSLPLPEHSERRPRFSFPLLPPPSLSTAVPSSNNTITAADLEKLHVLG
HGNGGTVYKVRHRKTSAIYALKVVHGDSDPVVRRQIFREMEILRRTDSPWVVQCHGILEK
PGGDIAILMEYMDAGTLDSSLKINGTFSETWLAGIAKQVLNGLSYLHSLKIVHRDIKPSN
LLVNQKGDVKIADFGVARFMYRSLDPCNSYVGTCAYMSPERFDPDTHGRNYNGYAADIWS
LGLTLLELYMGHFPLLTPGQRPDWATLMCAICFGEPPCLPDNSASEEFRSFVDCCLQKDS
SKRWTASELLSHPFLLLRQSDNLTTKSDDGLSAGSISLAGDLEISDDNQ
>EUC14352-RA [mRNA] [translate_table: standard]
MKSGGGSSLPDSEIGNGNENRNGFVFLVNNEVGLSVRTDPPPGSEVDYVEKDPRGRYVRY
SEILGKGAFKTVFKAFDQLDGIEVAWNRVKIDDVLQSPENLEKLYSEVHLLRSLKHENII
KMYDSWIDEKKKTINMITELFTSGNLRQYRKRHKSADMKAIKNWARQILQGLVYLHSQNP
PVIHRDLKCDNVFVNGNHGEVKIGDLGLAIVMQQPTAKSVIGTPEFMAPELYEEEYNELV
DIYSFGMCMLEMVTAEYPYNECKNPAQIYKKVTSGIKPASLSKVNDPEVKEFIEKCIVPA
SQRPPAKDLLKDQFLQLQNPINNPMQLANRIPKSLSLLDCGPLSMEIDSDYNQSVCTDSN
CGSPQSLVLEFQRMHQNNEFRLKGKKHDDNSVSLTLRISYPCGRVKNIHFLFYLDTDTAV
SVAGEMVEQLELEDHDVAFIAEFIDFLIIKILPDWKPSSDHHHDHDSPPTNLTTSTENPP
QEGLLDGNNFECTSPLSVFYSPSSLLNFDDKESQASAGSEDVSMKNEKTSDCVEYFIGGN
MSELEFGDLYEEEDEYSKIHEEEEGRSGLECIALNGFSRLASLDFGNFSKVTSLSSSCSS

PDQNQNHQDVELKMELDKVEAQYQHLFQELTRMKEEETKAAKKRWMERKKISSVL
>EUC06660-RA [mRNA] [translate_table: standard]
MVMEYTESCSSRASDSLPKQSRLRIQKVEVYREVLRRLKELDIEEASQPGFDDELWAHFD
RLPIRYALDVNIERAQDVLMHKRLLHMAHDPTMRPAFEVRLVQVYPQSDPDCGESVHSKF
TIENDDHYIEYPGSHSKHLPPAFGLSPGFELVLEANTRQQNCSSSGGGNLQLLRPLHEIT
ISANDKPKLLSQLTSLLSEIGLNIQEAHAFSTTDGYSLDVFLVDGRAFEETEQLRNVLVK
EIKKIEKQSWLNPYVMVSAADLRKTGVNLFQSHVNIPTDGTDVWEIDVKLLKTERKIAVT
SYGDLYKGTYCSQDVAIKVLKPEYLNEDVQRDFAQEVYILRKVRHKNVVQFIGACTHPPS
LCIVTEYMCGGSVYDLLHKQKGILKLPATLKVAIDVSKGMNYLHQNNIIHRDLKAANLLM
NENEAVKVADFGIARVKPQTGVMTAETGTYRWMAPEVIEHRPYDHKVDVFSFGITLWELL
TGKLPYEHLTPLQAALGVVHKGLRPTIPRQTHPKLVELLERCWQRDPTLRPEFSEIIEIL
ENIAKAMKVVEGESNSKKRRNYLEFC
>EUC00181-RA [mRNA] [translate_table: standard]
MSVESSSASADHGHIKGVPTHGGRYVQYNVYGNLFEVSRKYVPPIRPVGRGAYGIVCAAM
NSETREEVAIKKIGNAFDNRIDAKRTLREIKLLRHMDHENVIAIKDIIRPPQKENFNDVY
IVYELMDTDLHQIIRSNQQLADDHCRYFLYQILRGLKYVHSANVLHRDLKPSNLLLNANC
DLKIGDFGLARTTSETDFMTEYVVTRWYRAPELLLNCSEYTAAIDIWSVGCILGEIMTRQ
PLFPGKDYVHQLRLITELIGSPDDASLGFLRSNNARRYVRQLPQYPRQRLVTRFPNSSPS
VVDLLERMLVFDPTKRITVDEALCHPYLAPLHDINEEPVCPRPFSFDFEHPSCTEENIKE
LIWKESVKFNPDPSH
>EUC20904-RA [mRNA] [translate_table: standard]
MDEEANSWLRRAKFSHTVCHRFDVARLSSVPLSIQQDRFLDLGSKSRTFGLDLGSKSGEK
ILEFRRNSTVNKQRAVSPLPETKIPTTFKEARSDRKRFSTPLPRRKQPDKGISKGKSINS
HSDLNINTSPLKHFSSLKIGEKGKGGNRDSSWAKYFDYGGGGRVRALDAADEHTVNLSKL
FLGLRFAHGAHSQLYHGIYNDEAVAVKIIRVPDDDENNGLSYRLENQFNREVTLLSRLHH
PNVIKFVAACRKPPVFCIITEYLSEGSLRAYLHKLEHKLLPLEKLIAMALDIARAMEYIH
SQGVIHRDLKPENILINQAFRLNIADFGIACEEAYCDLLADDPGTYRWMAPEMIKRKSYG
RKVDVYGFGLILWEMVAGNIPYEEMTPIQAAFAVVNKNLRPAIPVGCPPAMSALIQQCWA
LNPEKRPEFWQVVKVLEQFESSVARDGTLNLVPNSTCQDHKKGLLHWIHKLGPVHPDASG
PVPKPKFA
>EUC18639-RA [mRNA] [translate_table: standard]
MECRDMATPVEPPNGVKSPGKHYFSMWQTLFEIDTKYVPIKPIGRGAYGIVCSSVNRETN
EKAAIKKINNAFENRIDALRTLRELKLLRHLRHENVIALKDVMMPIHRRSFKDVYLVYEL
MDTDLHQIIKSSQALTNDHCQYFLFQLLRGLKYLHSANILHRDLKPGNLLINANCDLKIC
DFGLARTSNGKGQFMTEYVVTRWYRAPELLLCCDNYGTSIDVWSVGCIFAELLGRKPIFP
GTECLNQLKLIINILGSQREEDLEFIDNPKARKYIKSLPYSLGTPFSRLYPHAHPQAIDI
LQKMLVFDPSKRISVTEALQHPYMSPLYDPSSNPPAQVPIDLDIDEDLSEEMIREMMWQE
ILHYHPEVAASQS
>EUC21330-RA [mRNA] [translate_table: standard]
MAVESSSASAEHGHMRGVPTHGGRYMQYNVYGNLFELSRKYIPPIRPIGRGAYGMVCTAV
NSETREEVAIKKIGNAFDNIIDAKRTLREIKILCHMDHENIVAIKDIIRPPQKENFNDVY
IISELMDTDLHQIIRSNQPLTDDHCRYFLYQLLRGLKYVHSAKVLHRDLKPSNLFLNANC
DLKIGDFGLARTTSETDFMTEYVVTRWYRAPELLLNCSEYTAAIDIWSVGCILGELFTRR
TLFPGKDYVQQLTLITELIGSPDDASLGFLRSENARKYVRLLPQYPRQQFSSRFPNASPG

AVDLLEKMLLFDPTQRITGRQTHLLQIFYAVFLLVFLFSDFCF

>EUC16962-RA [mRNA] [translate_table: standard]

MPSMESDQDSDRDSEPFVETDPTGRYGRYGELLGSGAVKKVYRAFDQEEGIEVAWNQVKL

RNFCEDQSMVDRLFSEVRLLRKLKNTNIIALYSVWRDEDRNTLNFITEVCTSGNLRDYRK

KHKQVSIKALKKWSKQILEGLDYLHTHDPCIIHRDLNCSNVFINGNVGQVKIGDFGLAAV

VGKNHSAHSVLGTPEFMAPELYDENYTELIDIYSFGMCLLEMVTRELPYSECDNVAKIYK

KVTSGVRPRAMDKVKDPEVKVFIEKCLAQPRARPSASDLLKDSFFYGIDDDDENIYDFL

Supplementary File S3. The genomic DNA sequences of EuMAPK cascades

>EUC04697-RA [gene]

ATGCACAATCTTACTCTGTTTTCGAGATTTGAACCCGGAATACAGTGGAA
AAACATTTACTATTTTGCCAAGGCTCAGCCTGTGTTGTATGTTGAATAAC
ATATGGTTGAAGGATCCTTTTTGGGTGAATGGTAATGAGGTTATGGTATG
TTTATAGCTTGATCATGAATATAGGGGCACAGCTTTATTACTTGGAGTTC
ACATGGGCATCTGTTTCCAGTCCACTACAAGGGATTGTTTGATTCTTCCT
TCTCAGAGTCCAGCTGTTTCAGATGGATGCTTCTCTTTCAATGTAAGTTA
ATGTGGGGATCTGGAAATATGGTTTGTGGAGCTGGAAAAGTGATTTCTCT
ACTCACATGAATAATGAGCATGCATTTAATGAATTTGGGTGCATTTTCTT
GTAAGTTATGCCTTCAAGTGAAGGTTTGAGAAGAGAAAAAAGAAGGTTGT
TTAATTATACATCAGAATTAATTTTAAAAAAGTTTCATACGCAATTATAA
CTTATGTTTTAGTCCCGTGCATTAAAAAAAGATGGAATGCCTAACACACA
AGCCAGCACTTCGCAGTTACGTGTATTTAATTACATTTATATATAATTAC
GTGTAATTAATATTACACAGGTATATGTAACTAATATTACACAATGCACA
AGCACATGTGAGTTTAATACGGAATCAAAAATCAGTTTAAGATACTAAAT
CAAATCTTATCTTTTTAAGGGGACTAATAATAGATTATACTCACTATTTG
AGACTAATTGTCAATCATCTCTTAATTTTAAGTTTTAAAAATTGACAAGA
TTAAAATGTAAACAAACCGTAAATATAGGAGTGTGCTGCGAATAGTCCTA
AGGTACTCTTTGGCTGTAAAGTCCAGGTCCAACTACTGACTCTTTTCGAA
ACTCCATTATCGGACAAGAAAGACCGTTAGTGGATGCCCACCACTTTTCA
TTGGTTAGAGAGACCCTCTTTAATACATATTGAAAAGATGTTCGCAGGGA
CTAGAAAGACTTGGTCATAGGAACTTAGGCCACTTACCCACTGGTAAAAG
AAAACCACCTTGACTGGATCAGAGTAAACAATAAGGTTGAATCAGGCCCC
TTGATTGGAGCCTCGAAATTAAAATTAAAGATGGAATTTTTCAATTTTTT
TACCTCTTTTTGTAATTTCAATTTGGGAAAACTATATTCTTGGTTCCTTT
GTATTATAAGGTGAGTGTCTATATAGATCTTGAGGTTCATCAAATTACAC
ATTTAATCTCTCAAATTTTATATTTGTTTTGATGCCACAAAATTAAAAAT
ATTTGTTAATAATTTTGAATGAAACATTTCAACACGTGAGCTTTTCGTTA
TTTTATCACTCCACTAAACTTAAGACATTTTATGATAATAATGCTTTAGA
TTCTTTCACTTATTATTTAGACGAATCATTACTAGGAAAAGATTATGAAA
AATGTCATAAATTTAGTGAAATAATGAAATGACAAAAATATATTTTATTA
GAAAGTCGTGTGTTAAACTTAATCTTTTTTTAATCTAAAATCATTAAAAA
AGATTTTTAATTTTGTAATATTGAAATAAATAAAAAATTTAAGAGAGAAT
TTGGTAAACTAGGGTTCACCGGTCGCGTAGACATTCATGACTCGCCCACC
CGTAGCAAAAAGGTGCCAAAATCAAGTTGTGATTTTACCCCTTTTTTTTT
CATTTTCACGGGGCCCAACTATTGAAAATTTTATGCATTCATGCATTTAA
GCGGTCAATTGCTTTCACGTGGCATTTGGTATTTGGTTGCGACTAATATG
CTCCCATTTTTCTTCTCCTTCTCGCTCTCAATTTTATAATTTTCTTTTTC
GGTATATCCATCCACGAGTGATCTCTATCCACACAAGGTAAAAGATTAGC
TCGTTCGGGATGCGTGTCCTCTCAATTTTATAATAAATATATAGCAAGGT
GTTTAGTTTGTTTCTAAACAAATATTAATTTTTATTTACTCACACAAAAT
TATTCTCACCATCATCTGTTGATAATCGGTATCCTACCAATTACTACCAT

CCTGTTGCGACCAAACATGTTTTTTGTTGCGACCAAACATGTTTCTTGTT
TAATTTTCATAGATTAAAAGAAATAAGTTCAAGACAATTTTTGTTATCGA
ACATCTTTCTATTCTCGAAATATTTCGAAACTGAAACAAAATATCCCAAT
TTATTAGTAGAAACAGAAAAGCAGAAACACTAACCAAACACCACCGTAGT
AGTTGTCTCGGAAAGAGAAACGGGTACTTTCGAGAGATTAAGTTAAAAAA
AATAATAATAATAAATTGTAAAAATTGCAAACGTATTAAGATAAAAATAT
TTTTAAAAATTATTAAATTAATTAACCGCTTAACAAATTTAGCAATTATT
AATTTTTTTTAATTTTTAACATGTAATTTTTAATTTTATCAAATGGGTGT
TGAGAGAAGATAAAAAGCTTTGGCGTAATAATTATGCTATTTTATTTTGC
TGAACTTATTTATTTATAAATTTAATTTTATTCTTAAAAAGATATATTTA
TATTCTAAACATCCAATAAATAAATGTAATATTCTATTGTTTCTTTATTA
TTTTTTTATTATTTTTTTTATGAAATATTTTAATTTTTTTATCCTTGTCA
ATATTTGTAGTTAACATATTAAAATACAAATTCAATGTATAGTTAAAATT
AATTCAATAGATTGAATTAAGTATTAACTGTGGAAGTAAATTAAAAAAAT
TGAAGTTGAAGGATGATATAATAATATTTCCTACTAACATTCTAGATAAG
TAAAACAATTGTACTACAATTTAGTGATTTATTAGTTTTTTTTGTTAATT
TATGTATTCTATTAATATATTTAAAAATATTTTGACGGCATTTGGTAACA
TTTTTAAGTATTTTTTTAGAATAATTTTTTTTTTTTTGACAAAAATGGAC
TTTTACTTAAAAGAGAAATTAAAAATGCACTTGATATCTTAATATTTTTT
TATTAAATAATTTTCTAATTTTAAAAAAATCATAAATAATTGAAAAAAAA
TAATAGATAGACATGTCATAATCCATTGATCTCTATTGAATTCTTAAAAT
ATAAAACTTGAAAATATAAAAAATAGGTAATTTTTATTTTTCAAAAAAAA
AAAATCACATTATCAATATTTTACATTCAATTTAAAATTAAAAAACTGTA
TCACTAAAATATCTCATTCTTTATTTTTTATTTTTAATTAAATGATTCAA
ATCAAAATTACTGAAATTCTAAATTTAATATTTTATTGCTTTTTATTTTT
TTTTTTAGATAATTAGCGATTAGTCCTAAATAACGGATATAGTATGTTAT
TAGTCCTCTTAAAAAAATAAGTTTTGATTTAGTCTCTTAAACTGATTTTC
GGTTTCGTATTAGTCATGCATGTGCTTGTGCATTGCGTGTAGAAGGTAGA
GAGAGTTTAATAAATAAATCTGTATTATAATAAATATTAGTTACATATAA
CTAAAATTAGGTACAATATAATAATAATTACAAATCATATATAACTATAT
TTTATATATATATATGTGTAATATTAGTTGTATAATATTACTTACAAGTA
ATTATAAATACATATAACTATAGTGCATATATTTGTGTAATATTTATTAA
ATATAACAAATATTTTACACATAAATTAGATACATACAATTGTGAGAGTG
CAAATGTTTATATTGCCTCTTATCTTATGTATTAATGCGGAAAAATAAAA
CAATGTGACTTTTTAAAATTAAAAAGTAAAATAAAAGATTTGTCACCACC
GAAATAATATGTTTTTTTTTTTTTAATCATAAACAGAATTTAATGTTGGA
AAATATCGGGGCCTTTAGCTATTAAAGCCCACCACGAAGCCTATATATTA
AGACCTTTCAACTCTACAGTCTCTTCCTCCCCCATTCTCACATGTTATTC
ACAGTAACAGTTCACGTCGACTGAGAAATATCCTCTCGTCCTCTCTCTCT
CTCTCTCTCTCTCTAAATCTACATTCATGCATCTATATGTGATGAATCTA
CACGCGTACAGCTCACAGAAGACAATTCAGGAATCGCTTTCCTTTTGCGC
TTAAGATAGTACCATCCTTCCTTGTTACTGAGGTAATCTTGATTCGATCT
TCTGGACTTCTTTTGCATCTTAGTTTTTCATGATTTGAATTTTGATATCT
ATTCTTTGAATTCGAGTAAGAATTTAGCTTCGCATTGGTATATTTTGTAT

CATCCTCAGTTAGATTACGTTTCTCTCTTTCGTTATTTCCTATTGAATTC
GTATATTCTACAACGCCACCTGATCGAACTTACGGTTTTATAAACCCTAG
TCCAGTGTTCTTATCGTTTGAGCTTCAATTGTACCCGTCGCTTTCGATTT
TGCTTCTAAAGCTTGCCTTAGCTGTCTTTCCGTCCGGATTGCGTTTGCTT
TGATTTCACGCTTCGACTTCCAAATTTTCTGAGAAGACTACGATTTTGGG
TCAATGGAAATTTGATTTGTGAACCTAAAATTCTAAGAAGATCAAGAGTT
TGGGTCTTGATTGTCTCGTTAACTAAAGTGATCGTCGCCTTTTTCGCGTG
ATTGACGCACTTAAGCATACTGGTATGTTCAGATTTCCAGACTTAGGAAT
TTCGATTTTGTTTGTTTCATTTCTGAAGTCAACTCATTGCATGTTAGGGA
TTAGGGAATTTAGATGTCGTATTCTATTAAACCATCCCAATAACTTAAGA
TGTTGAATAACATAAAGATTGACTTAAGCGTATGATTGGTTGGAAGGATG
GAAAAGAGAGGATAAAAATTTTATCATTGTGGAAGGAAAATTATTTTTCC
GAATGTAGTTTGATTAGGAAGGAAGAATAAAAAGAACGAAAATAGTCCAC
TTTAATATAATTTATTATTTTACCTTGGTATTACAACAGTAAATTAAAAA
TTCTATTATAAAAAATGTATAATTGTAATATTACTATCTTCTTGAAAAAC
TCTCCTCATTTTTCTTCATTTTCCTCTCAAATTGGTATGGTAAAAAAAGG
GGTAATCTGCAATTAATCCGATAAACTGATTTTGGATTTTGTATTAGTTC
CGTGCGTGCTCACGCATTATATGTGGAGCGTGAAATTAATAATTACATAT
AATTATATTGCATGTACTTGTGCAATATTTGTTACATCTAACAAATATTT
TAAGCACAAGTTAGATACATGTAACAGATGATTAAAACACAGGTTGCAAT
TGGGTAAATTTTTTTTTTTTTAAATTAAGAATAAAATAAAGGACTTGCCAC
TAAAACACCCAAAAAATTGTGTATTTTCCTTCTTATTTCTTTTTTCTCT
CATTTTCTCTCCATAGCAAAAAAATATTTTTTTTTTATCCTATTTTTCTT
TTCATTTTACCCATCTTTTTTTTTTCCCCACCCAAATAACTGGTGCGTAA
GAGAAGAACAAGAGACACGAGATTATTGTCTTCTTTGTTGATACGCCCCG
ATATGTGCATGGAGCATATAAATTGTCAAAAAACTTCATATGAATATACA
AAGCTATGAGTAATATTTTAAGTAATTCTTTTTTTAATCATCTATGTTTT
TCTTCCACCACATGTATACACACTACTAGAGCCCACTCATATAATAAAAT
AATGTATCTTAGGAAAGATTAGCATCATCATTTATAATCATTAATATATT
AATATTATCTCACTTTTATCTGTTATTTGAGATTTTTTTTTTTAAATTGT
ATCACTTAATCTATAATTTTTTTAAGGATGGTTATGTGATAAATCTCTTA
TTTTAACCTTAATTTTTTTTTAAAAAAAATCCCATATATAATTGCAACTT
GTGTTCAAATCCTGCACATTAAAAAAAAGGCGAAAGACCCAATATATGCG
CTGCACTATCGCAGCTATATGTTTGTAACTTGTGTATAAAATATTTGTTA
GATATGATAAATACTATACAATATATATGTTACATAGTTACATGTAATTT
GTAACATGTAATTAATATTACACAGGTACATTAACTAATATTACACAAGT
ATGAGCACATGTAAGACTAATACGCAATAAAAAATCAATTTAAGAGACTA
AATCAAAACTTATGTTGTAAATGGGACTAATAACAGATTACACCCGTTAT
TTGAAACTAATCACCAATTATCCCCTTTTTTAATTATATATTTGTTATTT
TAGTTTACTACGTGCATCTACTACCACTTGTAATGTACATAGGCTAATGA
ATAATGAGAGTTCATAAAGTATGTGATCATATCTTATATACTCCCATAAA
ACCATACAATAACAAGAACCGTGATAAAAATTAATGATAAGAAAACCACA
ACTATGTTACTCTCCCTGGTGATGTTCAGTCATTTTGGCTGTAGATAATG
ATTTTTTCTCGGGAATTACTAGATACCCAATTTCCACAATTATGTATAAG

TAATTAAGGCACCGATGGGTAAAAAAAAAAAGCAAAAGAGCACTTAATTT
TAAAACTAGTGTTTGTTTTTAACCAAAAATAAAATAAAATAGAAAAAGTA
AAAACATGAATTTGGGTAGTTCTTTACCAAAAGATAAAAATAGTTGTCTT
TTTGTTTATTCGATTTTTATATTATAAGAGATCGATGAACTATGACCTAT
TCACCTATAATTTATTTTTTTTAAGTAAGAGATCCTTCGTAAACATAATA
CTAAGATATTATCTCTACACTATCGGACACACTTTGGATTTTTCGTTTTT
GCTTTCAGATAACAAAGTATACTTTTGTCGAATTAAGTTCTTTTAATTTA
ATTGCAGGATTTCCGGTTGACCTCTAGAATGCCATCTGATAATCTTAATC
CACCCGATCAAGACCGGGAACCATTTGTCGAGGTTGATCCATCAGGGCGT
TTTGGCAGGTACAATGACCTTCTCGGCTCTGGATCGGTTAAGAAGGTATA
TAGGGCGTTCGATCAAGAAGAAGGTAGAGATGTGGCTTGGAACCAGGTTC
GGTTGAGGAATTTCAGCAGCGATCCATCTGTAGTGAACAGGCTTTACTCA
GAGATCAAGCTGCTGACTACGTTAAAGAACGATCACATCATTGTGCTCTA
CCATTGCTGGAAGGATAGAGAGCATAACACGTTGAATTTCATTACAGAGG
CGTGTACCTCGGGCAATTTGAGGGATTATAGGAAGAAGCATCGTCATGTT
TCGCTCAAGGCATTGAAAAAGTGGGGGAAACAGATTCTGAAGGGGTTGGA
GTATCTTCATACCCATGAGCCTTGCATCATCCATAGGGATCTGAATTGCA
GCAACATTTTTATCAATGGAAACGTTGGGAAGGTATTTGCTTTTCACCTT
TTGAACTTGGCCCTTAGAAATATTCGGGCTACTGCACCCGGCCGGCCAAC
CCATAAATTACTATTCTTTTTAAAAGAAACGTGTTATTTNATGTTTTTGC
AAAGCCTGGCCCGCCCTGGCCTGGGGAACAGGCCTTAAATCATGAGACAA
ACCTGGCCCTTAGAAACTTCTTGCTTAATGGCCTGGGGAACTTGAGGAAA
CTTCTTGCTTAATGGCATATCTTAGGTAGTGGTGTTCATAGGCCCGTTCA
GGCCGGGCCATATAAGGGTAATTACATGTTTTTGCAAAGCCTGGCCCGCC
CTGGCCTGGGGAACAGGCCTTAAATCATGAGACAAACCTGGCCCTTAGAA
ACTTCTTGCTTAATGGCCTGGGGAACTTGAGGAAACTTCTTGCTTAATGG
CATATCTTAGGTAGTGGTGTTCATAGGCCCGTTCAGGCCGGGCCATATAA
GGGTAATTACATGTTTTTGCAAAGCCTGGCCCGCCCTGGCCTGGGGAACA
GGCCTTAAATCATGAGACAAACCTGGCCCTTAGAAACTTCTTGCTTAATG
GCCTGGGGAACTTGAGGAAACTTCTTGCTTAATGGCATATCTTAGGTAGT
GGTGTTCATAGGCCCGTTCAGGCCGGGCCATATAAGGGTAATTACATGTT
TTTGCAAAGCCTGGCCGCATTGGTGGAACCGCTTACATCCAGACAACTGC
CTTAAAGTATTCGGGCTACGCACCCGCCGGCCACCATAAATTCCTATTCT
TTTAAGAAATTGTTATTTTATATAACAATATAACGTAAATAAATTACTTA
AATTAAAAACCTCAAATTGATAATATGTTACACTTCATTATAATCTTGAA
GTATATAATAAATATATATTTAGTGTTTTATTACAATTAATAATTTGACG
GCCAGCCATACTATAAAAAATTGAAGCCGTAGCCCACTAAATATTTAACG
GGCTAAAATCTAAACTTACCAACCTATAGGTTAACCCTCTAACCCCAAAC
CTAAATTTAAGGGCTGGGCAAGGTCACTGGGCCCACTACGATGGACACCG
CTAAAACAACGGTTGATAATTCTCTAGTATACTAAGGCCCATTTGTAATT
TTATTTTTTTCAAATATAATTTGATGATGTCAAGTTGCATATTTTCTTCA
ATATCCATTCATCCTAATCTAAGATTAATTGTTTATATACGGGTTCAGAA
ATTCTGAGTACGAAATCTTGTCATTACATATCTTAAGAATACAAAAAATT
TTTTTTTTTTCTTGGCGACCTATTTTTTTTCAGAAAATATTTCTTCTGAT

TTATATATATATATATTTTTTTTTAAAGCAAAAAGCCTGTAAAACGGCGCG
TCAATGCTACCTAATCTCTAGGTCAACCGAGATTCACACAGATTAGCTCA
CTTTTTTTAAAGCTTGTCGGCGAATAATATCCTCATATACCGGTTGTCCT
GAGATGAGATAGGATAAAAGTCTTATGTCTGTTCTACAAGTATTCTGGAA
AAGAATATTATTCTCACCCGATATTCTGAATGTTGCTTTTAATTTATGAG
AATCAAAAATAAGAAACACCAGTTTGCTACAACATTTTTTTATTTTTCTC
TTTAAAGTTGGGTGCTGGCGGAGTGGTAATGGCGACGGGTGATGGTGGCC
TGGTGAATCAGTGATCAAACGAGGCCACGCTTGTTACAGATTCCTCTGTT
CTATTCCATACCCTTGCGACATAGCAAAGTCAACATATCTTCGTTCATCA
TTTCTGATCTCTGGGAATAAAAAGATACATCTGAGAACGGAAACATTAGG
GGCGTTGTAACCGTTTCTGTTTCCATCTCCAGCAGTGGGATGGAATGGGA
TGGAATGAGATCAGAATTTTGTTTTCCTTCTTGAGGTAGAATCCGCGTTT
GTACGATAATATTTTTTTGTTTTGGGAAGAAATGCGTTCTTGGTAGCCAA
CACCTCTCTTCTCTCCTCCGCTCTTTTTTTTTCCTTTTTCCGTTTGCGCT
GGGCTGGCAGCCCGCCGCCGGGTGGTGCGGTGCCCCAGCCTCGCCGGTCG
AGCGCGCGAGTTGTCAGTGTGGCCGCACCGGCGGTGACTGGCGGCGATGC
TGGAGCTGACGGTCGACGGTCGCGCGATGCCGCACGTGCCGGCTCCGGCG
CATTGAAATAACTGGAAGCGAGGAGAATTATGCAGACACGACTTTTTTTT
ATTTCTCATTTCTTAAGAAAAAAATGGGCACAATTCTCAAATGGGAATGG
AATGGGAACGAGAACAAAAATTACCAAACGCTATTCCCAAACAGAATTCC
CAAAAATCTGGAAATGGAATGAGAATTTTTGTAATGGAAACAGTTACCAA
ACGGGGCCTAAACAAACGCTACCTTGGTATTATATATTGTGAAGTATAAA
CTACAAATTTTGAGACTTTCCCTTTGGATGCTCCCCGCCCCCCCTCTTTA
TGAACTTCCAAAACCAGAGTTATAAAGTACTTGAGACTTGTAGTAGTGTT
TGGTTTAGGGAGTTAGAGGTTTTGAATTAAAAAAAATAAACACATGTAAT
TATATTGAAATAAACACATAATGGGCCAAGGTCCATATACACACAAAAGC
AACGCGGGTTAAGTGCTAGGAGCGCTAGTGTGCGTCAGTGTAAGCAATCA
ACAGAATGTTGTGCACAGAAAGAAGTAAATCGATACTGATGCATTCTTTT
TACAGGTTAAGATTGGAGATCTAGGGTTAGCAACGATAGTGGGGAAAAGT
CACGCGGCGCATTCCTTACTAGGCACACCCGAATACATGGCACCCGAACT
CTACGAAGAGAACTACACAGAGCTAGTGGACATATACTCCTTCGGGATGT
GCTTGCTCGAAATGGACACCAAAGAAATACCCTACAGCGAGTGCGATAGC
ATCGCCAAAATCTACAAGAAAGTGACTGGCGGGGTGAAGCCTCAGGCATT
GGATAAAGTAAGTGATCCTGAATTGAAGGCTTTTATTGAGAGGTGCATTG
GTCAGCCTAGGGCCAGGCCTTCCGCTTCTGATCTTCTCAACGACCCTTTT
CTCTCTGATGTTGCCGAATTGGACGAGGCAAGTCCTATTTCTTGA
>EUC05370-RA [gene]
ATGTCTGCTTGGTGGAAGAGAAAATCTACCAAGAACAAAGAACATCAACA
GGTTCAGCAAAAGCCACAAAGCGATTACCAGAATACAAGCAGAAAAGCTT
CAATCAAGGATGATAAGAAGAGAGGCCAAGACAAACCCAAGAGCTTTGAC
GAGGTCTCCACGCTTGTCTTCTCGCATAATTCGCCGCGCAACAGCAAGGA
ATTTGGTGGTTTAGTTGGTGGAAGTAGCGGCGGCTCATCGGGATTTTCGG
GATTCGATTCGTCGGAGAGAGGGCACCCTCTGCCGCGACCGTCGGTGTCT
TCGACGCAGTCGTTTGGGGTTGATCATCTTCAAGGTGTCGGATCGGGATC

TGGGTCGGTCTCGGTTTCCAGTGTGAGCTCATCTGGATCCTCCGATGATC
AAGCTCATCTTGATCAAGTTCATGGCCAATTGGGCTTTGTCTCAAGGTTT
GTGTGATGAATACCCAAATTATATTTTTTAGATTTCCAATTCTTTAGGGG
TTTGTTTCAAATTTTGTAGGGATAATACCCGAATTCGATATATACTCATA
TATCTGTCTGTGTGTACTGATTTTGAATTGATTGGAGTTTTCTAAGGGTT
GATTGATTTGGTGATCTAAATGATTTGTTCATCAAACTTAGGACTTCAAA
CTGTCTAATTTTAGCCCCCCAAAAGTAGGGTTTCATTTGCGGGTGAAAAG
TTCTTTGCCGCCGTAAAATTCGACTGCGTTTGCAATTTCTGGAATTTACA
TGAAATGGGCAGGTGTTTTGCTGATTTTCCAGCATTTTTTGTTCAATTTA
ATACTCCAGCAGCCATCGTTTTTCTCGTTGATCTTTTTGGGGTGTTTAAA
ACACATAAAATACGATATAAGTTTCACCTTTGGAGATTTATGATATGGTG
GTGAACTTGTTAAAATTATGGGTCCTATATTTCCCTTGACATAGCATTTG
TTAGCATGGAATTCAACTTCGGAATTGTAATTCGAAATGAACCCATGTAA
TTTTCTTTTTACATCCGATCAATTCTAATTTCAACTCCATGAAGCCAAAC
GCGTTCAAGTGCTCTCGATGATTGGGCTTGTCTCACAGTTGTCGGATTGA
ATTGCTTTCGACAAAATTGGTAGTAAATTCAAGACCTTGGACNGAGATCA
GAGAAAAGAATAGATCGGAGATCGATGATTGAACAAGGCTTATTCTCTCT
TTCTCTCTCGACGAATCAAAGATCAAACGACCTTCCTCCTTCGATGACGA
CGATGAACCAATTTTGGATTTCACGATCGAATTTGGTGTTCTTCGATGAA
CAGATAAACAGGGATTCTCAATTCATAGGTTGAACAGGGATTTATCTCTC
TCTCTCGACGAATTAACGATCGAACTTCCTCCTTCGACAACGACGACAAC
GAACCCAATCAACCGCCGACGACCTTCAATGCCTAAGGTCTCTCTTCATC
TTAATCTCTCTCTCTCTCTCTAACCCAACGAAGATAAACTGAAGAAATTA
ACATTTTTTTTGTCTAAGTGAGAAATGGACTTTCACCAAAACGTTTCTAG
CAAATGAAAAATAAAAGGGTAAATCGATAATTTTACTTAGAATTTTTTTA
ATTGTATAATATCTAACAAAAAACAAGGATAAAATAGTATTTTCACACAA
AAATATTGGTAAATAGAGTTGACTGGCATTTGTCAAATGACAAGGGAGGT
TTCTGGTATAAGTGCAAAAGATAGAGGAGATTTTTTGTATAAACCCTTAT
ATATAGATTGGATGGAATAACCTAAATCCTCGCTAGAAAATCTAGACTCA
TCGTTTGGATCTCGGTTAAACCTACAAAATAAAACTCAAAAAAGAAAAGA
GACGAATACACACACACACACACACACACACATATATATATGGATGGAGAAA
AAAAATAGAAATAAACAATTAGCGATGCAGATCCCCGACAACGACGCCAA
AATTTGATCACTCAAAAATAATACTTTTAATTTGGTACCACAATACTGAT
GTAGTACAGTGTTCAAATAGGGCATCAAACCACATATATTAGTCTTGTAA
TTATCAATTTTCAATTATTTTTCTTCCTTTAACGAAAACGAAATTTGATT
TGAGTTTTGAGTTTTTAATGTAAGAAAAATAAAATACTAAATTTAAAATA
AATGAAAATAGATAACCGGGTTGGGAATTATGGGTTACATATTTTGGGGT
TTATATTGTTTTTGTATTTTTTTTAATTCAGATTTGTGCAAATGTGTGTT
CCTAACTACAAGTCGGAACTACTCAGAAATGTTAGATAGATTATTATTTG
GCATTGTTTATTTTTGGCATTTAATTCCTACGTTACTCTAATCTAGGGTG
AATTAAAATTTAAACATAAACCATCAATTCCTGCTACTCAGAAAGGTTAG
ATAGATTATTCTTTGGTATTGTTTATTTTTATCATTTAATTCCTACGTTA
CTCTAATATAGGGTGAATTAAAATCTTGGCATAAACTATCAAGCCCAATA
ATTAAATCTCAAGAATACTATTATATGATTGTTAGATCTAGACCACATGT

ACTAGATCTAACGTTTTAGAAGTGATTGAATCTAAAATTTAAAATCTCTA
CAAAAACAATTTGAAAATAAATTCAATCACAAATAAACCGAATCAATAAC
GAAATTCCTCATCATAAAATTGTTACACATAATCTAAAATTAAATTAAAA
ATACAATATTACATGAAATCAAAACACATACCACAATCCTAACCCGGTTG
AAGGAATATTAGTCACGCATTGATTTGTTTTCCATTTGAGCTTTCCTTCC
AAATTCCAATATTTCTTTTGAGTTTTAGAAAAAGAAGAATAAAAAAACCT
AAAGTCCTAAAACCCTATTGCTGAAAAACAAAAGAAAAACCAATCAAGAA
GGAATAAAAAATAAAAAGAAGAAGCAAAAAAATGGGAAAAATCTAGTCTC
CTCTCCACTTTTTGAAATCCCCTTTCCTTAATTAATCCTATGGCCTTGAA
TGGCTATTCAAGGCCATTTACAACCCATTATAATAGCCTAAATCTCACCA
AACATGGTCTACTGGCCAAAACGTGCGCGCGATGCCTGCGTGCCACATTT
GGTAAACAGACACGCTCAACTTCAGATGTCTGTAGATCCACGTGCGACTA
GGCGAACACGCCATGGTTGGCTTTAAGAAACTCAATCAGTTGCTTATTTT
TATCTCCCAAGAGAGCCATCTTGATCTGGACAATTCATTTCGGATCGATT
GAGTGTATCCAAACATCTTTCAACTCTTCGTCTGGATGAGGTCGTGGTTG
ACTTGACTGCTTAGTTGGGAAGTCCAGGATGTCCCCTTTCTGGAGATGCT
CTACCATGTCCTTGAGCAAAAAGTCCCCATTGACCTCCTCATCCTTTGAT
TGTTATTTGGGAATATCCATCTTGGTGACGAAGCACTCCCGAGCTACCTT
TTGGCTTTCCCGTACCTCGTCGATCTCGTTCTTGGTTGGGAACTTCAACA
TCATATGGTACGTTAAAGTGATAGCCTTTAAAGCGTCTAGGAACGTCCTT
TCAATGATTGCATTGTACGCCGACGGATTGTCCACTACCATAAACTGTGC
GTCGATTATAGAGTGTTTCAGTTCTATGCCAACTATGATTTGGAGAGTGA
TCCAGCCTAGCGGCCTCACTGTTTTTCTTCTTGAAATTGACCAGGGAGGT
CATCATCGGCTTCAGTTTGTCATAGTCGATTCTCATCCTGTTAAGTACAG
TTCACTCTTCCTTTTCCCTTCGACATTTTTTTCTTTTTAGCTAGAGCCCG
ATATACGTCATCTATAGCGATGAACTCGATGGAGAGAGCAAAGGCCACAA
CTACAATTTTCGGCATTGCGCGGGCCATGTCGTACAAGAATGGACCTGTT
CACACCCTTCCAACAAGGCGGCTACTGTCATCCCGTCATTTCGTTCTTCT
ACTTGATCAGATTTATCATCGTCCCAGATCTTAGAGTAAGCATAAATATT
TGTTAAAATGGTGAGAAAGACAATTAGTTGTTCCTACAAACGGCGTCAAA
CTGGTGATGTAGAAATCTTGTTGGATAGATCACAGTCAATGTGGATGAAT
TCCGCCAAGTTGACTTCATTGATCTGTCCTTGCAAAACAAGCCTTAGACA
ACCGGGGGTCGGGTTCCCGGTGAAACCCTTCCTGTGTGAAAATAAGTCAC
CGTTATTTAGAGGAAAAATATACTTCAGGAAATTTTAAGTAGAATTTATA
CTGAGGTAGAGTTTACTGCCCTCTATAAGAAGTGTAGGTTCGAGAATTCG
GCTTGCCTATTTCGCTCTTAGGCCAAGACTTGTCTTCAAATAGATAGTAG
AATGAAAGACTTGTATTTTGTAGTCAAATTTTGATGCATTCTTTGGTGGA
GAATGAACCCCCTGTTTATAGGAGTTTCTGGTATTAATTTTGGAGTTAAA
TACTTATTTGGTAGTGAAATTAAGTCATTACTAATTAATAGTTAGGCCAT
CTAGAGTTGTAGTAGATTTAGTGGGGTTAAAGTGATAAATTTAATGATAT
GATTAGTAAAATTAGCCATAAGAATTCTGTAAATTTTATAGTTAAAAAAT
AAATTCTCAAATGAATGAAATCGAGAAGATATTTTCTTCCGATAAGGAGG
AAAAAATCTGAACTACTATTTACACAAATTATTCTTCCATATGTACATAT
CACACCCCCAACCACCCCTTCAGGTTCAACCAATTGAATATACTAAATAC

ACGCGGACTTGTATCCTATTCTTATCTATTTGTATTTTCCTCTCCAACAT
AAATTAGAGAAAAGAAAAATAGAAGCCACGTCATCCATGATCCATCCACG
GCTGCTTAACCCAAGTAACCAACCACCGGCTGCCGTCCCCCACTAGCTGA
GCTGTTCCTCAATGTTGCTAGGACCCGCACCCCCCCTTCCCCTTTCACCC
ACTTCTCTCTCCAAGCGCCCCTCTGCATCTTTGTCTCGCTTTCTTCTTCG
ACTTTTCTTCTTCAGCTTCATCACATAGTGCATCCTTCGTCTCAAGACTC
GTAAGCCAACTTTCTATGTTCCTAGTCTCAACAGGCAAAAGGATTGACTG
TAATTCATCACTTCCTTCTCTCTTAGTCGTCAATCGAAGCTCGAAATGTC
TGCTTGGTGGAAGAGAAAATCTACCAAGAACAAAGAACATCAACAGGTTC
AGCAAAAGCCACAAAGCGATTACCAGAATACAAGCAGAAAAGCTTCAATC
AAGGATGATAAGAAGAGAGGCCAAGACAAACCCAAGAGCTTTGACGAGGT
CTCCACGCTTGTCTTCTCGCATAATTCGCCGCGCAACAGCAAGGAATTTG
GTGGTTTAGTTGGTGGAAGTAGCGGCGGCTCATCGGGATTTTCGGGATTC
GATTCGTCGGAGAGAGGGCACCCTCTGCCGCGACCGTCGGTGTCTTCGAC
GCAGTCGTTTGGGGTTGATCATCTTCAAGGTGTCGGATCGGGATCTGGGT
CGGTCTCGGTTTCCAGTGTGAGCTCATCTGGATCCTCCGATGATCAAGCT
CATCTTGATCAAGTTCATGGCCAATTGGGCTTTGTCTCAAGGTTTGTGTG
ATGAATACCCAAATTATATTTTTTAGATTTCCAATTCTTTAGGGGTTTGT
TTCAAATTTTGTAGGGATAATACCCGAATTCGATATATACTCATATATCT
GTCTGTGTGTACTGATTTTGAATTGATTGGAGTTTTCTAAGGGTTGATTG
ATTTGGTGATCTAAATGATTTGTTCATCAAACTTAGGACTTCAAACTGTC
TAATTTTAGCCCCCCAAAAGTAGGGTTTCATTTGCGGGTGAAAAGTTCTT
TGCCGCCGTAAAATTCGACTGCGTTTGCAATTTCTGGAATTTACATGAAA
TGGGCAGGTGTTTTGCTGATTTTCCAGCATTTTTTGTTCAATTTAATACT
CCAGCAGCCATCGTTTTTCTCGTTGATCTTTTTGGGGTGTTTAAAACACA
TAAAATACGATATAAGTTTCACCTTTGGAGATTTATGATATGGTGGTGAA
CTTGTTAAAATTATGGGTCCTATATTTCCCTTGACATAGCATTTGTTAGC
ATGGAATTCAACTTCGGAATTGTAATTCGAAATGAACCCATGTAATTTTC
TTTTTACATCCGATCAATTCTAATTTCAACTCCATGAAGCCAAACGCGTT
CAAGTGCTCTCGATGATTGGGCTTGTCTCACAGTTGTCGGATTGAATTGC
TTTCGACAAAATTGGTAGTAAATTCAAGACCTTGGACTTTTATATGGTAA
ATTCACTAATGAATGACATTTTCTCAATTTTCAGAGTACATGGTGGCGAT
ACAAAGTTTTATACAGTACCAAGAAGCCCAGGTCCAGCTTTGAGAGGTGC
AACAGCTAATACATCGCCTCTTCATCCACGATCTTCTCTAGACTCCCCAA
CGACAAGGGTGGAAGATGGGAAGATCGAATGCCATCGGCTGCCCCTTCCA
CCGGGTTCCCCTACTAGTCCTTCTGCCTTGCCCATGCCTAGAGCTTGTGG
AGTCACTGACAACTCCTTGTCAAAGTGGAAGAAAGGAAGGCTTCTGGGAA
GGGGCACGTTTGGGCATGTTTACGTTGGATTTAACAGGTTAAGTTATATT
TTTAACAGTCATAGATGATCAGACATCATTATCGTCAATCATTACTTATA
ATGCATCATTTGCTATATATTAACAGTGAGACTGGGCAAATGTGTGCAAT
AAAAGAAGTTAAGGTTGTTGCAGATGATCAGACATCAAAAGAAAGTCTCA
AGCAATTGAATCAGGTGCCCCCTCGCCCCTTTTTCCCCCAAATTTTTTTT
CGCTTGTTTCTTTTCCCATTGAACTTTGAAAATGTTCATCCATGTTCTTA
ATACCTTTTTTCTATTGATTCTTATCATATTTTCTTTCATAAATAGGAAA

TCAATTTGCTTAGTCAGCTTTCTCATCCGAACATTGTTCAGTACTATGGC
AGTGAACTGGTATGTCAGCCATTGTCTTGTTTGAAATTTCTCATTTTAGA
AAACCTTCAGGAAGAGGCATTTGTGTTCTGTAATGTTAGGTAGCTTTTGT
TTTGTGTTTCTAATTTTTAACTTGGAAAAGAAACAGAATCTGGGAACCAG
AAAAAAACTGTTTCTGTTCTGTTCCCATGATTCTTTTTCTAAATATGGGA
ACATAACAAAAAAAAAACAAAAAATCTTCCCCAGATTTTTTTTCCTTTTC
CAAGTTTTTTGGTGACAAAAAAGGTAGAAACACTAAACAAATGCCAACTT
ACACTAAATTGATGATGCCTCTGGTTTACTATGTCCTCAGAGTGAAGAAA
CACTGTCTGTTTTCTTGGAATATGTTTCTGGGGGTTCAATCCACAAACTA
CTTCAAGAATATGGCTCTTTTAGAGAGCCCGTCATTCAAAATTACACTAG
ACAGATCCTCTCTGGTCTGGCCTACTTGCATGGAAGAAATACATTGCACA
GGTAAAAAAAAAATTGTCACTCGCCTTGTGATAACATTTTTGAAATTACA
GTATCATCCAAAAGCGTCATATTTTGGCAGGGATATTAAAGGAGCAAACA
TATTAGTAGATCCCAATGGTGAAATCAAGCTTGCGGATTTTGGCATGGCA
AAACATGTATGTCCTAATAATACCGATTGTGTATACACAACCATTCTAGT
TATTCTACTGTGTCTCATTGCATAAAGAACATTATGCTTGTTATCATGAA
ACAGATAACGTCTTGTTCTTCAATGCTTTCTTTCAAAGGAAGTCCTTACT
GGATGGCGCCTGAGGTACTCTACACACGCACACACGAATGTTGCTCGTCA
ATGATTTTTTGTTTCTTTACTTTTTTTGTGTTTTGATCTATTTTCTCTTG
AAAGCTAGAATTTCTTATTTAGCTTTATTTTCAACGGTTGTATCAAATCT
CAGGTCATTATGAATACAAACGGCTCTGGCCTTGCAGTGGATATATGGAG
CTTAGGATGTACAATTCTTGAAATGGCAACATCGAAACCCCCTTGGAGTC
AATTTGAGGGGGTACCTTTTTTTCCCTTTTACTTGCCAAATATATATTTC
CGTTTGGACACCATAAATTTATGTTGTATTTTACAGGTGGCTGCTATATT
TAAAATTGGAAACAGCAAAGATATCCCTGAAATTCCCGATCGCCTGTCCG
ATGAGGCGAAGAGTTTCATAAGGTTATGCTTGCAGCGGGATCCATCTGCA
CGGCCAACAGCATTAAAACTACTGGATCATCCTTTTGTCAGAGAACAATC
TACCGCAAGAACTGCTAATGTCAATTTAACCAGGGAAGCCTTCCCTTCCT
CCTTTGATGGGAGCCGCACACCGGTAAATGCTTAAAACATGCTTCTTGTT
CTCGTTATTATTGGGTTTCCTTTTTTAAAAAATTTCCTTCTCTTATGCGG
TTGTATATTTTCGATCTCTACATGGTTTTGTTTCTGTTCCTAGAAAACCT
GAAATAGAAAAACAGAAACGAAAATAATAATTATGTCTGTTTAGTCTAGT
TTTAGATGGTATAGGGTGCTGGTGGCCTTGATTTCATTTGCCATTTGGAT
TGCTTCCATTCAATTTAAATGCTCTTTCATTGTCTTATCAGCATCTAATA
TGTCATGGTAAAGAAATTGTCAGTAACAGCTTGATTGTTTTCCATTTGAA
ACAGACGGCTCTAGAGATGCATTCCAACAGAAATAGTATTACTTCCTTTG
ATGGAGATTATTTTGCAAAACCCATTGTGGCAGTCCCCAGAGCTTTGATC
AGCCAGAGGTACGCCTCCCCTTGCCCTCCCACACACACACACGCGCGCGC
GCGGAGAGAGGTTGCACTTGACAGAAGGACTGAGGTCGTACTTGTTTATT
GTTTCTGTATTTTTATTTTTTGTTCTCAGAAATATGGGAACGAGAAAGTC
TTTTCTGTTCCATTCCCAGATTTTTAAAACAAAATTCTTGGAACAGAATA
GAATTGTTTCTCCGATTTCTATTTATGTTCCAAGTAGTATGGGAACAGAA
ACACTAAACAAACACCACCAGAGAGACTTTATCAATATTCTCTCGGCTGG
AGATCTGTTATTGCTCGATACTAATGTGGGTGTTTGGCTACTCATACTAC

ACAGGGAAAGTGCAAGAACTATAACATCGTTACCCGTATCTCCATGTTCA
AGCCCATTACGACAATACGGACCAGCGCACAAGAGCTGTTTTCTGTCACC
TCCACATCCATCATACCGTTTGGTAGCACAAAATGGTTATAATTTAACAG
ACTACTCGGTTTTGACAGCGAGGCACAGCAGAAAGACTACTCTCGACCCG
TGGCTGGAAATCCCCCAGTTTAATCCCCAGAATCCTGGCAGATCCTCCAG
ACCTAGACCCATTTTATAG
>EUC04041-RA [gene]
ATGAAGAATCTTTTAAAGAAACTGCATGCCATGAACAATCAATCTGGAGA
CTCAGAGGGGTCTACTTCTTCCAAGAGCAACCGGTTGAGAGACGGTTCGT
CTCCCGACACCCCTCCACAATGTAGGTCCCCTAACAGTTCTGAGCATAAA
CCCTTTGCAGCTATTTCCGGATGGTTGAGTTCAGTTACCAAAAGGCATAG
TTCAAGTCCCCCATCATCTTCGAATGTGAAAAGAGATCATAGAATGGAAC
GGTTGGATTCCATGAGGAGTACCGGTGCTATTGGGCTCAATTCCGAGTCT
AGCAACTCAGGGGATCCTGAAGTTGAGGAGGAGTATCAAATTCAGTTAGC
TCTGGAATTGAGCACAAGGGAAGATCCTGAAGCAGTTCAGATTGAAGCTG
TTAAGCAGATCAGTTTGGGATCATGTCCGCCTGAGAACTCTCCAGCAGAA
ATTGTTGCATACCGATATTGGGTAATTCTCCATCTAATTAATAGCTTATA
AAAATGCAATCATTCTAGCCTTCCAATACATATAATTATTGACATTAAAA
AGTGGTGGCCGATGAACGGATTGTCCTTCGGGAAGTATAGTGCCCATATT
ATTTTCCATATCTTCTTGCTAGGTTTTAAGCCTAATGTGTGCAAATGTAG
CTTTGATGGCGTGGCCCAAATATCTACCTAGGAGGGGGAGGGGGATTGAA
TTGGGTAGGGCAAATATTTTGGATAATTAAAATTAACTTAATTGCACACA
ACAATTTATATAGTGGAAGCAAATTAAATAATAATGACACGAATATTAAC
ACGTGCAATAATTAAAAGAGAATAAGGGATGGAATAATATTGCGATTTGT
AGTATTTTGGCAAGGCATCGTGCCTATGTCTAGTCCTTGGAGCTCTTTCT
GAGATTTCAATTCACTAATCAATTGATTCTCTCATGGGTGAGAACCAAAC
CTTATGTCACCAAAGGTACCTCGCTTGGAAGTCGCTTAATCTCTCTCAAC
AAGAGATTGAAACTTACTTGAGATTCATTCTCTCTAGACTTTCAAACAAG
CGAAGTCCTCTCCTCTCGGTTCTCTTAATTCGAGCAAACAACAGTTGCTT
GATTACAATCAAATAAAAATAAAAAAATAAAAAAATCTCAATAGTTGAAT
TGAACAATAAATCAATTGAGCTTCTATAATAATCTTCAAGATGAAAATTA
ATAATTGACTCACTATTGTTACAAAAGTGTAGAATCAGTCATTCTTTATA
TATGATGAATAATAAAGAAAACAAAGTGATTTCACAAGAAGACAAGTGTT
ATGAATGAATAAATCTTGAAAGAAATAGCTGTAAGTAAAAGAAATCCTGT
ACATATCTTTTTTGAGTAGGCACCAATTTATACAATATAAAAATGAGTGA
TTATGGAAATTGCTCTTAAACTATTGCCTTAATTTCTTCCTAAATTAATT
ATAATTGATTGATTTTACAATTAATGGGATGCTAAAATCATGGATGTCTA
ACAATAGCACCACTTTTGAATATATGAACACAATACTGTAGGCCTGTTTG
CTCCTTGTATAGTTCAATAATGTGTGTAATTGATGTATAAACCCATTTAG
TGGGAAATTTATGGTAGCCGTTTGGGTTAATTATAGAAGGAAACTAACTG
TTAGAAATCATAAAAGGAAGTGTTTCCGTAGCTCTCAAACAAAATTAGGT
GGCCAAATTTCACTTTTAGTTGCCACCAGAACCCTAACCTTTGGTGCCCT
GCACAATTTAGGCACTCAAACGCATGGACTTGGTCGCCCAAATTTGATTG
GAACTCAGACCTCCACTCTCACCATAAGTTAGGCTCCTAAAATTATACTT

TTAGTGGCCTACTTTCACTAAAATTAAGTTTTTCACCTGTTTTATGTTGC
AACATCTAGATCATCATAAAACATGTTTAAGATGATTTTTAAGCACAATT
GTGATCTATAATATTTTTCAAATTTTTTTGCAACTCATTTGTGAAAAGAC
AAAAGTACCCTTAAAAGATTTAAAATGTTGTAAGCATGAGTGAGGGTAAT
TTGACTTTATATCTATGCATGATGAGGTCCAGTGTGTGAATCTTAATGAG
TCAATGAGTAATCAACCAAAAGATTATTACTTAAGTAATAAATAAACACT
TGATCTTTCAGTAGACTGTCTCTTGAACTCTTCAAATATTTAATACGAAG
CTTATTCAAGAATTCTTTTAATTCCACAACTTCTTTAATCAATCAACTCC
ATTTCATGGCTCTTTAATATCCGAAGCTTATTCATGAATTCTTTTAGTTC
CACAACTTTTTTAATCAATCAATTCCATTTCATGGCTCTTTAATATCTTG
AATGCTTTAATTCCTACAATTAAACACTTGACTCAAAGAAAATGTTAGCC
CTCAAAATTTCAAATGAGGCATTATCATCAAAACTAAAAGGAGGGGTCCC
TAATGGTTAAAATCACTTGGGCTAACAATGGAGAATTTGTGGTACATGAC
ACCCCTGCAACAGGGTAAATGTAAAATTGAAAGAGCACTCAAAGGATTGA
TAGAAATGGACGGACAAAAGATTAAGCCTCCTTTGCATTTCCTTACCAAA
TGAATTAATTTTGTGTCGGTGTTAGTATTTTTCAAATCATGCTTGTCAAT
GTTAAAAAAATTTACTTCATCAGTTCATCAAGTGACTCGACGGAATGAGA
GATCGCCTGTAAGCAACAGTTGTGAAAAGTATGTATTTAAAATATTGCAC
CAGGGTGTATTATATTGACCTTTTCTGATCTCTTCGCTTTCTCTGCCCAT
TTAACTCATATTCTGTATTTGGCATTGTACTGATAAGGGCTCTAGGTGTT
CCATTTAGTCTTGGGGTATAATATTATTTTAGGATTGTTACTTGTGCAAT
GGTAAAATCTTGACTACTTTGCCAAAAAGGGAGGGGGCGGGGGTTAAAAT
TTTGAACTCGCAATCTTGACATTCTAACTTGTGTAGCATTACAAGTTTGA
ACTGACAAATGAGATACTGCGATTTCAAATGTGAGGAAACTCATATGTAT
ACTGAGTGCAAAGGTTAGTTGTGCACGCATTTCATCCTCCCAGAATCAGT
AACTGATCACCACCTAATTGGTAAAGACATTTTATGCTTAATTAATGAAG
AAGGAAGAGAGTAGCGTTGGAAAAGTTGAGTTTTACATTATGTAGTAGGA
CAATTTTAGTACTTGGTATTTTCTCAATCGTATATTGCATTCGTTCCATT
CCTTTCTTGCTTTTCTCATGTCGTAGATTTTTACATGTTTTACAGACTTA
CAATGCTCTTAGTTATGATGACAAGATCCTGGATGGCTTTTATGATCTAT
ATGGCATATGGATGGAGTCCAGTTCATCAAAGATGCCTTCCCTGATTGAT
CTTCAAGAAATACCAGTGTCAGACAACTTAAGTTGGGAAGCAATTCTTGT
TAATAAGGTGGATGATTCTAAGTTGTTACAACTCGAAAGGAAGACCTTGG
AGATGGCTGTAAACTCAAGGTCAGGATCTATGAATTTTGTTAGTGGCAAT
TTGGTACGGAAGCTTGCTGCTCTAGTTTCTGAACACATGGGAGGGCCTGT
TACTGATCCAGACAACATGTTGATGGCATGGAAAAGTCTAAGTTACCGTT
TGAAGGCAAACATTGGGAGCATGGTTTTGCCACTTGGTTCTTTCTCGGTT
GGGCTTGCCCGTCATCGCGCATTGCTATTCAAGGTAATTTTCCCTTTCAG
GCAATTAAGTTTTAAAAATAGAATAAACCTGATGCAAAGCATGTATACGA
ATTTCGCTGTTGTAGTGAACCATTTACTGCCTTATTTGGATTTGTACCAG
ACATAAACAAGATTCAGATGCAATTCTTGTGAAGTATGGTAATAAAAGGA
TATAGGAGAAAGATGAAAAATATAATTGATACTTTCAGTACATGCAGAAT
GGGATCTATGCCCCTAATTATATAGCCACAGGAGCAAACTAAATTAAGAA
GAATATCACCAACATAAATGGGCTAATAAAGCCATACAATAATGATACCA

ATAAAAATAAATGCCTAAATATTATATTTCAACAGTATTGATGGGTCAGA
TTATGAACCAATTTAATACAAAGTTGGACCTCATTATGATATAGTTGTTG
TACCATTTATTATTTGTGATTAGGACCATATTGGATTTGACAGGTTCAAA
TATAATTGCATCTTGGCATATATCTACCTTGACCATTGTTCCCTTGACGT
TACTAGCCGCCACAGTCACTGTTCACTGATTTTGCCTAGGTCGGTTTGCC
TAGAGCCCCCCCACAACCCAGACCGGCTTTGCCATTACAGATTAGACCTC
CATTACTGCAGTACCTCGCTTCGCCGGACCCTCACAACTCAGATCCGACT
TCGCCATCGTAGATTAGACCCCAGCCGCAACAGTCCCTCGCTTCAATGCC
CAGATCTGGCTTTGCCATTGCCATTCGGACCTACGCCGACACTGTTTTGA
CCAAATATTGCTTCGCCGCCTCCGTCCAAGAACTTCGCTACTGCTGTTCT
GTCGCCGCCCTACCCTTTTCAAGCCTCGTCTATGTCAAATCTATCCTTCT
ACCTTCTCGACGACTACAACTCCAAAGAACCAGTCTTTACTCTGTAAGTA
ACTACTTAAATTGGTCCAAATCTGTGAAAGTTGTCCTTACTGGACAAGGG
AAACATCCTCATCTCACTGAATCAACCCTTGCACAAGATGATAAGAAGTA
CGAGGAGTGGATTCAATCTGATGCGCAGATCGTATCCGTGTTGTGGAATT
CGATGGAGCCTCAAGTTGCTGATATGTTTACTCATTTGGATACGTGTTAA
TCAATATTCCTATATCTTCGGTGTTGTATTCTAGTAATCTTACTTGGATG
TATGACTTAGGTTGAGTACTTTCAGTTTCAACAAGCCACATAATTGGTTA
CGGATTATTTCGCTGTATTCAGGCTTTATGAGGAGTTAACTATTGTTCTA
CCTATGACTACCGATATGAAAGAAATGCATAAGCAATGTGAGCAGATGAC
CCTCATGAAATTCTAATTTGGATTACGTCTGGAATTTGAACAAATAAGAT
ATCAGATCTTTGCCAGTGCTACCCTTTCCTCTATCACAGAAACCTATTCA
CGGGTTTTGTGAACCATTAACAGGTATAATGTTTCTAGCTCCAGTTCATT
TGCTCCATCTGAGCATTTGACCCTTGTTACACATATTCCTCCACGTGGTC
GTGGTGATCAACCTAGTGGTGGTCGTGGTGGTTCCACTCGTGGTGGCCCT
TGGGGTAGACACAATGGTTGTAGTCGTTGCCCACATTGTCAATGCTCCTT
CTGTTGAGAAGAGAATTATACTGGAGAAACTTGTTGGGACTTAATCAGCC
ATCCTCCCAAATCCGCCAACGTGGTGACATTTGATCCAAATGATCCTTCT
GTAATTCCAATCTCATGCGGCCAAGGATACTCTGTTGAAGAATATGCCTA
ATTCCTTCAGTATAAGGCATCCCAGCAGGTATCTTTCTCCATTACTTCTT
TAGCTCAACAAGGTACTTCTACTGTTTGTCTTTCAATTTGCCCTTCCCCT
CCCATACCCTGGGTTATATATTTTACTGCCACTAATCACATGTTAGACAA
CTTTTTCCTGGTTATACATATTTAGTCACCTCCTTCGTTGTATCCAATTA
TTGTCGCTGATGGTTCCATAGTCCCTATCCGTGGTATCTGTGCCTTGGAA
TCCTCACCCTCTTTATCTCTATCATCTGTTTTGTATATTCTTTCATTTTC
TTTTAATTTGATGTCATTAAGTAAGCTTACTAAACATCTTAATTGCTCTG
TTTTATTTTTCTCTGATTCTTTCATTGTCCAGGATTTGAGGACTCAGAAG
ATGATTGGCACAAGGAGTGAGGAAAATGACCTTGACTATTTGTGATAATG
ACGAGAGCCCTGCTCTCCCATCTATTGCATGTCTGGCTGTTTCATCGGCC
TTCCAGATTGATTGACACCTCGACCATCATTGCCTTGGCAACCTCAAGCT
ATTTGTTCCTGATTTGAGTCATTTGTATTCCTAAAGTACGAGTCTTGTCA
ATTGGGTAAGTATCATCGTAGTTTATTTGTTTCTCCAGCTCATGTTAGAG
CATCAAGTACTTTTAGTTTAGTTCATTCTGATGTTTGGGGTCCTTCTCGT
GTAACTTCTACATTAGGTTTTCATTACTTTGTTCACATTTGTGGATGATT

TTTGTAGAGTTACATAATTTTATTTAATGAAAGATTGTTCTGAATTAATC
TCCATTTTTTCTGTGCGTTTTGTGCTGAAATTAAAACCCAATTTAATGTA
CTAGTGCATATATTTCGAAGTGATCATGCTCGAGAGTATTTTTCTCAACC
ATTTACTTTATGTCAAACTGTGGTATCTTGTATCAGTCTTCTTGTCCTCA
CATACTGCAGCAAAATGAGGTTGCAAAGTATAAGAATCAACATCTCCTAG
AAATAGGTCGTACTCTTCTTTTTTAAATGAATGTTCCCAAATGCTTCTAA
GGTGATTCGGTTCTTACAGATTGCTTCTTGATCAGCAAGATGGCGTCCTC
TACCCTAAATGGCAAGGTTCCCTATTCAATTCTTTTTCCGCGTTCTCCCT
TGTTTGGTTTAGCTCGTTGTATATTTGGTTGTGCCTGTTTTGTTCATCAG
TTGACTCTCGGTAGAGATAAGTTAGATCCCTATGCTTTAAAGTGTGTCTT
CCTTGGCTTTTCTTGCACACGAAAGGGCTATCGTTGTTATAGTTCATCGT
TACGTTGCGCATTTGTGTGTGTAGATGTTACTTTCTTTGAGTCCATTCCT
TACTTTTCTTTTCCATCCTCTAAGGATGATCTGGATGTTACACTCCTTTT
GTCAATCCTTTCAAACCCTACACCATCTCCCTCTGCTCTAAAACCTGTCC
CGCCTAGTCGTTCTTCATCTCCTTTGCAGACATATTTTCTTCGCCAAAAG
GTAGCTTCCTCGACGCGCCCATTTTCTCTGTCCTCAGATTTTTCTCCAGT
TGATCCATCCACGTTTGACTTGGACATGCCCATTGTTGTCTGGAAGGGTA
AGCGTACATGTACAAACCATGTTATTTCTAACTTTCTCTCGTACGTACAC
CTCTCTCTTTCATACTCCTCTTTTGTTGCTTATTTGCCCTCTGTTTCTAT
TCCTAAGTCTGTCTCAGAAGCTATCGCTCACCTTGGTTGGACAACCGCAA
TGGAAGAAGAAATGCTAGCCTTAGATAAGAATGGTACTTGGGATTTCGTC
TTGTTCTGCTTGACAAAACAATAGTTGGATGTAAATGAGTGTACATAGTC
AAAATTCATCCGGATAGTACCATTGATCGACTCAAGGCTCGCTTAGTTGC
CAAAGGTTATACCCAAGTATATTGTCTTGATTACTCTGAGACTTTTTCTC
CTGTTGCGAAGCTCGCTTTTGTTCACTTGTTCGTCTCTCTGGCTGCCATC
TTTCGTTGGCATTACATCAGCTCAAGATCAAGAATGCTTTCTTAAATGGC
GATCTGACTGAGGAAGTATACATAGAGCAACCTCTTGGGTTTGTTGCTCA
AGGAGTGTCCGGTTTGGTATGTCACTTAAAGAAATCCATGTATAGCCTCA
AGTAATTTCCTTGAGCATGGTTTGGGAGATTCAGTGAAGTGGTCATTGAA
TTTAGTCTTCAGCGTTGTAGAGTCGATCATTCCGTGTTCTACAGTCACAA
TAAAGTTGAGAGAATATTGTTGATTGTGTATGTAGATGATATCATGATTA
TCGGGGACGACCACTGTGTTATTCAAGAACTCAAAAGGTTTTTGCATAGT
AAGTTTCAAATACAAAATTTGGGTCAGTTGAATTATTTCTTGGGGATTGA
AGTTGCTTGATCGAATTAAGGCATTTACCTGTCTCAGAGGAAATGTCTTT
GATTTGTTGAGCGAAATATGAATGTTAGTAGCTAAATCGATTGACTCGCC
TATAGATCCAAACACCAAACTATTGGCAGATCAAGGTGTACCATTGATAA
ATTCAACACAATATAGATGATTAGTTGGGAAGTTGAACTATCTGATAGAC
TAGACCATATATCTTCTTTGCTATTAGTGTTATAAGTCAATTCATGGATC
ATTCCACAACTTGTCACTGAGATGCAGTGATTCATATTCTCCGATATTTA
AAAAGTGCTCCTGGTTATAGTTTGTTATATCAGAACTTTGGTCACACTCA
AATAAAGGGGTATACAGATGTAGATTGGGCCGGATCACCATTGGATAGGA
AATCTACTACATGGTATTGTGTGTTTGTTGGATATAATCTTGTCTCTTGG
AAGACTACAAAACAAACGATAGTGGTTTGGTCAAGTGTAGTTTCAAAATA
CAGGGCGATGACCCATACCACTTGTGAGTTAACATAGTTGAAACATTTGT

CGGAAGAACTTAGATTTAAACACTTTTTACCGATGAATCTGGTATGTGAT
AATCAAGCAAAACTTCATGTTACATTCAACCCAGTCTTCCATGAGAAAAA
TACATTGAAGTTGATTGTCACTTTATTAGAAAAAAAATTGTTTGCAGAAG
TTGATTATGACTAAATATGTAAATTCAGTTGATTTATTTACTAAACCGTT
AGGGGGCACAAGGATAAAGTATATTTGTAAGATAGTTGAAGCATACAATA
TATGTGCTCTTGCTTGAGGGGAATATTAAAGGGTATTTATGATATTCCTG
TATTATATAGGGTTTCCTGTATTGTTTAGGAGAGTTTCCTATATTGTTTC
CTAATTTCTATTATAAATAGAGGGAGCGGCTAACACAATTGTTAGGCTGT
CTCATTCTTTTTTACTCATGTTTTATTAACAGTGTATTTTGAGGATGTCT
TTTCGAAAGTTACTTGTATAGTATTAGTTTTCTCTAGCTGTGTGGTTACA
TCACGTCCAGATATGCAACCTGCACCAGGGAACTAAGGTTGGAAGTAGTG
ATGAATCTTATTCCCGTCATAGATGGTGTGAGAACATCTCTATTTTTTTT
TGTACACTTGCTGCTAGTAAGCATTTACTTTATATTTGATATGACATTGA
CAATATAAAATATAACGGCTCATTGTATGAAATTAGATGGGACTGCCAAC
TCTTATCCAAATATTGGATATGCAATGTAGATTAGACTTCCTGGATAAAA
CATCACACGGAAATTTCTTTTACATTGATGAAATACAAGGAAAAAGAAGA
AGATTCTACTTGAGCTACAAGAGACTCCCATGACTCTTTTGTTGATAAGA
ATGTTGTTCTATAACAAAATCAAGTTTAATTTTTTAGGAGGCAGTTGTCT
TAAAAGAGCTGATTCTATAATTTTATTGTTGTTATTCTTGATATTAATTT
TTTTAAGAAAAAATAAATCAAAATTTGTGTATCACGTAATTTGAACTGTT
TCTACATCTCATATTTTTTGACGGATGCATCAATTAATATTATCCTTATT
TTACCCGATGGAATGTGCTAAAGTAAACGAGTGTGAGAAAAGAATGGGAT
AACTTGATCATAGTGGTCAACCTCTCTTATTATTTATAATAAAGTATGGG
TCATATAGCCTGTACACTGAAACCCTAATACAAATATCAATACTAAAGTA
TCATCTAACACTCCCTATCAAGTTGGGGCATACATATGTTCCCATCTTGT
TACAAATATATTTTACCTGAGAACTCCCAAAGAGTTTAGTGAATAAATTA
GTTAACTGATAAAGAGAGTTAACATGTCGAGTGTGGATGAACTTCTATAG
TAGTTTCTTTCAAATGAAGTGACAATCAACCTTTTTCGTTCTCTCATGAA
AAAATAGGTTTCATGCTATATGGAATGCTGCTTGATTATCACACACTAGA
CTTATGAGAGGAGAATGGTCGAAGCCAAGTTCTTTTAGCATGTGGTTCAA
CCAAATCAACTCACATGTGGTATGAGCCATTGTTCGATACTCTGACGCAT
TTGATTGGGCTACCACACTTTTTTTCTTACTCTTTCAAGAGACCAAATTA
ACTTCAACAAATACACAGTAGCCTGTTGTAGATATTCTGTCTGAAGGAGA
TCCTGCCAGTCTTCATCCATGTATCCCTCTATGTGAGTGTCTACATGATT
CTTATACAACAGACCACATCCTGGTGCACTTTTTAGGTAACTAACAATGC
GAACAAAAACTTTGCAATAACTCGTTCTTGGATGATTTAAAGATTGACTC
ATAACACTAATGGGAAAGGATATAATTGAAATTAAATAATCAATTGTGTG
TCTTTCTAATTGTACAATTAGGCTACATATAGGAGTAATTAAGACTAAAT
AAGGAAATTAACTACGACAGATAATACCTTCCTAATCTACATATTTGACT
TTCCCTATATACAAATAATAACAATATTGAATATTCCAACACTCCCCCTC
AAGCTGGTATGAAGATATCTTCCATTGTCAGCTTGCATACTAGATAATCG
AATCTCTTCTTTGGTAATCCTTTTGTTAGAATGTCTGCGATCTGTTCAGT
AGTCGGTACATATGGCATACATATTTGTCCACTCTCGATCTTTTCCTTGA
TGAAATGCTTGTCCACTTCCACATGTTTTGTCCGATCGTGCAACACCGGA

TTGTGAGCAATCGCAATGGCTGATTTGTTATCACAGTAGACCTTCATAGG
TACTGAATTAGATATCTTCAACTCTCCGAGTAATCTGTTAATCCATAACA
CTTCACAGATGCCGTGAGCCACTGATCTGTACTCCGCTTCTGCGCTGCTT
CTAGCTACGACATTCTGTTTCTTGCTCCGCCATGTGACGAGGTTCCCACC
AACAAAAGTACAGTAACCGGAGGTCGATCTCCTGTCCGTGATGCTCCCAG
CCCAGTCTGCATCGGTATAAACCTCTACAAGGTGGTGGCCACGTTTCTTG
TACAAAATTCCCTTTCCAGGAGTACCCTTTAAGTACCTTAGTATTCTATA
GGCAGCATCGAAGTGTTCTTGTCCTGGTGAGTGCATGAATTGGCTGACCA
TGCTTACGGCAAAGGCTATATCAGGACGGGTATGTGATAAGTAGATTAAC
CGCCCCACTAGTCTTTGATATTGTTCTTGGTTTATCACAACTTCAGGTTT
GGCCGGTTGTAGTTTCTCATTCGGTTCACTTGGAGTTTCTACAGCTTTGC
ACCCGAGTAACCCTGTTTCATTCAGTAGGTCGAGTACATACTTTCTTTGA
CTCACAAATATGCCTCCCTTTGATCTAGCAAATTCCATTCCAAGAAAATA
TTTTAATGGACCTAAGTCTTTGATTTCAAAATTATCAGCAAGTTTTCCTT
TAAGTCTTCTCAACTCGTCACTATCACTTCCTGTTAAGATAATATCATCT
ACATAGACAATTAAAATGGCAATCTTACCATCAATTGAGTGTCTATAAAA
CATAGTATGATCTGCTTGGCTTTGTTGATAACCCTGACCTTTTACAGTGG
TTCCAAAGCGTTCAAACCACGCTCTTGGAGACTGTTTTAGGCCGTATAGT
GACTTCTTTAACTTGCACACCTTTCCTTGTCCTATCTTTCTTTCAAAGCC
CGGTGGTAAACTCATGAACACTTCTTCCTCCAAGTCTCCATTTAGAAAGG
CATTTTTCACGTCTAATTGGTGTAGTGGCCAATCAAGATTCACAGCTAAG
GACAATAGAACCCGAATCGAGTTTATCTTAGCAACTGGAGCAAATGTTTC
TTGGTAGTCTATTCCATATGTCTGCGTGAACCCTTTAGCCACGAGCCTTG
CTTTATACCGTTCTATGCTTCCATCTGCCTTACACTTAACTGTGAATACC
CACTTGCACCCGACTATCTTTGTATCCTTTGGCGAATCAACTATTTCCCA
CGTACCACTCCTTTTAAGAGCATTCATCTCTTCCAGAACTGCTAGCTTCC
AACCTGTATCACCTAGTGCTTCCTCAATGTTCCTTGGAATAAACAGATTA
GTAACTTTAGAAGTAAAGACTCTATAAGGTTCGGAAAGTTTTGTGTAGGA
TACATATCTAGATATGGGATGATTGGTGCAGGTTCTAACTCCTTTCCTGA
TGGCTATAGGGATATTAAGATCTGGAGGAATACTGGAATGAGAAGATTCA
TCAGGAGGTGGTGAAGATATAGGATTACCTGGATCATTGCTTGGGGAGTC
GGATTGACCTTGTGCTAGAACAGTAGGCTGATCTGTATTCTTTTTTGTAC
CTCTCCGAGTATAAACCCGCAACTCAGTACATGATTGTGGTATTTCTTCA
TCTGTTTGTGAAGGAATTATGCTCGGCACTTGTGTACTAGTAGGGATCCC
TTTTTGTTTTTCAATATTGTCATCACTTGTTTTTGACTGATTTGGAGGAA
AAATTTCTAAGACTGTGGTAGGGAGAGGATCATCAGTGACCCAAAAATTC
CCTTCCATTACATTCTCCCCCTGAAAGGGATTTTTGTCAAAGAAGGAAAC
ATTTTCCAAAAACGTGACGTCCATACTAACATGAATTTTCTTGGTTTGTG
GATTGTAACACTTATACCCTTTTTGATTTGGTGCATAACCGATGAAAACA
CATTTTTCTGCTCTAGGATCAAGTTTAGACCGAAATTGGCTAGGAATATG
AACATAAGAGGTGCAGCCAAATATTTTAAGAGGCAGGTCAGCTGATATCC
TAGAAGCTGGAAAGTGTGTTTTGAAGTTATCAAATGGTGTGGTGTAATTT
AGAATACGTGTAGGCATGCGATTTATTAAATATGCGGCTGTTAAAAGAGC
ATCACCCCACAAATATTTGGAATATGCATAGAAAACATGAGTGCACGTG

CGACTTCGAGTAAGTGTTTATTTTTTCGTTCAGCAATGCCATTTTGTTGG
GGCGTGTCACGACACGTGGATTGATGTAGAATACCATTTGTTTTCAAAAA
ACTGCCTAAACATTCATTGAAATATTCTGTTCCATTATCTGTGCGAAGAA
TGCTAGGTTTCATTTGAAATTGGTTTTCTAGCATAATGCAAAAATCTTTA
AATAAAGCTGCTACATCAGATTTTGCACGCATCAAATATGTCCAACATAA
ACGTGTATGATCATCAATAAATGTCACAAACCATCTTTTTCCGGATAGTG
TTGTGATTTTTGAGGGACCCCATACATCGCTGTGAATTAAATAAAATGGT
TTAGATGCACGATATGGTTTTGAAATATAAGAAGTACGCTGACTTTTTGC
AAACAAACAACTTTCACAATAAAAGGAAGAACAATCCATATTCGTAAATA
ACGTCGGAAACAAATGTTTAAGATATTGAAAACTTGGATGTCCTAATCTA
AGGTGCCAAAGCATGATTTTATCACGAACAGACGTTGAACTCATACCACT
GGTGCCGTGAGTTGTTTTATTCCTCAAGGAGTTATCATCGAAGTAATACA
GGCCATCCACCAATCTAGCACTGCCAATCATCTTCCCCGAGCTCTGGTCC
TGAAAGAAACATTGAGATTCAGAGAAAATGGCATGACAATTCGAATCTCG
CAATAGTTTACTTACAGATAAGAGGTTACAAGTAAGTTTAGGAACGTGGA
GGACCGTTTTTAGATTAACACTATCTGAGATTGTTATTTGGCCTTTTCCT
GCAATGGGTGAGAAACTACCGTCTGCAATTCTAATTCTTTCATTACCAGA
ACACGGTGAATAGGTATTGAAAAGGTGATAAAAACTTGTCATATGATCAG
AAGCACCTGAATCAATAATCCAAGGTGCTGAGTTTAAGGAACAAGAAAAG
GCTCTAAATTTACTACCTGTTTGAGCTAAGGAAGCATTAGGAGTACCAAA
TGATGGATTGACAGTCAGCAGTTTCAGAAGTTGATCAATCTGTTCTTTGT
TGAATGGAGTGGTGGCAGCTTCATGCGCGGAAGGATAGGATCGGTTAGAT
TTTTCTCCCGTTTTGTTGCTCTTCAAATTTGCTGGTTTCCCATGGATTTT
CCAGCATGTTTCCCGTGTATGACGGGGCTTGTTGCAGTAGTCGCACCATA
TACGAGGACGTTCCTCTGCTCGGCGTGGAAACCTGCTGGAGTGAGCATTA
GCAGCAGCCAAAGCAGACTGGTCAATTGGAGCAACTGTAGGGCTGATAAT
TTGTGGCTTGTTAAGCATAACGTTCCGACGGGTCTCCTCCCTTCGGACCT
CCGAGAAAACCTCATTAATTGAGGGAAGAGGCTGACGACCAATGATTCGT
CCCCTAACTTCGTCAAGTTCCGGTTTCAGACCTGCTAGGAACTTAAAAAC
ACGATCATCATCCACCAACTTCTTATAGTGGCTATAATCCTCGGTCGAAT
GCCATTCATGATCGTCGAACATATCTTGATCTTGCCATATTCTCTTCAAT
GAATTGAAATACTTGGTGATACTGTCTTCGCCTTGACGGATATCACGGAG
TTTTAGAGTCAACTCATACTTTTGAGATTGGTTTCCCAGATCAGAATACA
TCTGATTTACATTGTCCCAAAGTTCTTTCGCCGTAGGGTAGCACATGTAG
TTGGAGCTGATTTCCTCATCCATAGAGTTTACGAGCCATGTCATCACCAT
GGAGTTTTCAGCATCCCAAGTCGTATGCGACGGATCTTTGATGGAGGGCT
CCTTTGCATCACCGGTTAGATATCCCATCCGTCCTCGTCCACGGATATAC
ATTCGCACTGATTGAGACCAACGTAAGAAATTGGTTCCATTCAGACGAAC
AGAGGTGATTTGGATGGAGTGGGATTCGGAAGGGAGATGCTTTGGTTCGG
TAAGTTGGCTGAACCTTGGGATGGATTCCGAGGTAACGTCGGACATGGTG
AAGGCAAGGGAAAATACCGTCAGAAATTAAGAATCGTCGGGTTGGCCGTC
AACGAGTAAATCTCGCGAAACCAAGCTGCTGGATGCTGAGTTGACTCACT
GAGAATCACTGAGTTGACTCGCTGAGAATCGTCTGAGTTGACTCACTGAG
AATCACTGAGTTGACTCGGTTTCGTCGATTTCAAGCAAATCCGGCAGCGA

TGGCGGTGGCTAGGGCCGCGAGTGTTCGTCGGTGGCTGCAATTGAACCTC
ACGAAGTGCAATCGAGTTGTAATCGCAAGATCTGTTTCGTACAGATCTCG
CGAGGGCTCGTTCGTCGGCTGCTAGGGCTCCGGCGAGGCTGTCGGCGGCG
AATAGAGGACTGACGAGGCTTCGTTGTCGGTTGTAGAAGGCCGGAGAGTG
TTCGTCGGCGGCTGTAGAGGACCGGCGAGGCTTCGTCAGTTGTAATGAGG
GCTCGTCGTCGGCTGTAGGGGAGTTTTCGTTCGTCGCTTGGCTGTAAGTG
ACGCCGGCGAACCTTCGTCGGCTGTTGTTGGATGCCGGCGAACGTCGTCG
ACGACTGCAATGGCCGGCGAACTTCGAAAACTTGTAAATGGCCGGCGGCT
GAGCAGAGGTTTAGGGTTTTTTTGGGTTTTGCTCTGATACCAACTTGAAA
TGAATTAATCAAATTGTATATTTTATTAATCATACAATATGAGACTACAT
ATAGGAAAACTTAAGAACTAAAAAAGGTAAATAACTAATTACAATAAACT
ATTTTCCTAATCTGTATATTTGACTTTCCATATATACAAATACTACCAAT
ATTGAATATTCCAACACTCCCCCTCAAGCTGGTATGAAGATATCTTCCAT
TGTCAGCTTGCATACTAGATAGTCGAATCTCTTCTTTGGTAATCCTTTTG
TTAGAATGTCTGCGATCTGTTCAGTAGTCGGTACATATGGCATACATATT
TGTCCACTCTCGATCTTTTTCTTGATGAAATACTTGTCCACTTCCACATG
TTTTGTCCGATCGTGCAACACCGGATTGTGAGCAATTGCAATGGCTGATT
TGTTATCACAGTAGACCTTCATAGGTACTGATTTAAATATCTTCAATTCT
CCGAGTAATCTGTTAATCCATAACACTTCACAGATGCTGTGAGCCACTGA
TCTGTACTCCGCTTCTGCGCTGCTTCTAGCTACGACATTCTGTTTCTTGC
TCCGCCATGTGACGAGGTTCCCACCAACAAAAGTACAGTAACCGGAGGTC
GATCTCCTGTCCTTGATGCTCCTAGCCCAATCTGCATCGATATAAACCTC
TACAAGGTGGTGGCCACGTTTCTTGTACAAAATTCCCTTTCCAGGAGTAC
CCTTTAAGTACCTTAGTATTCTATAGGCAGCATCGAAGTGTTCTTGTCCT
GGTGAGTGCATAAATTGGCTGACAATGCTTACAGCAAAGGCTATATCCGG
ACGGGTGTGTGATAAGTAGATTAACCGCCTCACTAGTCTTTGATATTGTT
CTCGGTTTATCACAGCTTCAGGTTTGGCCGGTTGTAGTTTCTCATTCGGT
TCACTTGGAGTTTCTACAGCTTTGCACCCGAGTGTCTATAAAATATAGTA
TGACCTAAGTCTTTGATTTCAAAATTATCAGCAAGTTTTCCTTTAAGTCT
TCTCAACTCGTCACTATCACTTCCTGTTAAAATGATACCATCTATATAGA
CAATTAAAATGACAATCTTACTATCAATTGAGTGTCTATAAAATATAGTA
TGATCTGTTTGGCTCTGTTGATAACCCTAACCTTTTACAGTGGTTTCAAA
GCGTTCAAACCACGCTCTTGGAGATTGTTTTAGGCTGTATAGTGACTTCT
TTAGCTTGCACACATTTCCTTGTCCTATCTTTCTTTCAAAGCCCGATGGT
AAACTCATGAACACTTCCTCCTCCAAGTCTCCATTTAGAAAGACATTTTT
CACGTCTAATTGGTGTAGTGGCCAATCAAAATTCACAGCTAAGGACAATA
GAACCTGAATTGAGTTTATCTTAGCAACTGGAGCAAATATTTCTTGGTAG
TCTATCCCATATGTCTGTGTGAACCCTTTAGCCACGAGCCTTGTTTTGTA
CCGTTCTATGCTTCCATCTGCCTTACACTTAACTGTGAATACTCACTTGC
ACCCGACTATCTTTGTATCCTTTGGCGAATAAACTATTTTCCACGTACCA
CTCCTTTTAAGAGCATTCATCTCTTCCAGAACTGCTAGCTTCCAACCTGT
ATCACCTAGTGCTTCCTCAATGTTCCTTGGAATAAACAAATTAGTAATTT
TAGAAGTAAAGACTCTATAAGGTTCGAAAAGTTGTGTAGGATACATATCT
AGATATGGGGTGATTGGTGCAGGTTCTAACTCCTTTCCTGATGGCTATAG

GGATATTAAGATCTGAAGGAATACTGGAATGAGAAGATTCATTAGGAGGT

GGTAAAGATATAGGATTACCTGGATCATTGCTTGGGGAGTCGGATTGACC

TTGTGCTACTTCTGGTTGCTAGAACAGTAGGCGCTGACGCTTGTTTTCTT

TTTTGTACCTCTCGAGTATAACCCGCACCTCAGTACACATGTGTGGTATT

TGTTATCAGTTTGACGGAATTACTGCGTCGGCCCTAGTGTACTTCCAGTA

GGGATCCTCTTGTGGTTTGGTTTGAATGTTTGTATCAGCGTATGTGGTTG

GACGGATTTTTGGAGGCGAGTCTGCTCCTCNNNNNNNNNNNNNNNNNNNN

NNNNNNNNNNNNNNNNNNNNNNNNNNNNNNNNNNNNNNNNNNNNNNNNNN

NNNNNNNNNNNNNNNNNNNNNNNNNNNNNNNNNNNNNNNNNNNNNNNNNN

NNNNNNNNNNNNNNNNNNNNNNNNNNNNNNNNNNNNNNNNNNNNNNNNNN

NNNNNNNNNNNNNNNNNNNNNNNNNNNNNNNNNNNNNNNNNNNNNNNNNN

NNNNNNNNNNNNNNNNNNNNNNNNNNNNNNNNNNNNNNNNNNNNNNNNNN

NNNNNNNNNNNNNNNNNNNNNNNNNNNNNNNNNNNNNNNNNNNNNNNNNN

NNNNNNNNNNNNNNNNNNNNNNNNNNNNNNNNNNNNNNNNNNNNNNNNNN

NNNNNNNNNNNNNNNNNNNNNNNGTTTCCCATGGATTTTCCAGCATGTTT

CCCGTGTATGACGGGGCTTGTTGCAGTAGTCGCACCATATACGAGAACGT

TCCTCTGCTCGGCGTGGAAACCTGCTGGAGTGAGCATTAGCAGCAGCCAA

AGCAGACTGGTCAATTGGAGCAACTGTAGGGCTGATAATTTGTGGCTTGT

TAAGCATAACGTTCCGACGGGTCTCCTCCCTTCGGACCTCCGAGAAAACC

TCATTAATTGAGGGAAGAGGCTGACGACCAATGATTCGTCCCCTAACTTC

GTCAAGTTCCGGTTTCAGACCTGCCAGGAACTTAAAAACACGATCATCAT

CCACCAACTTCTTATAGTGGTTATAAACCTTGGTCGAATGCCATTCATGA

TCGTCGAACATATCCTGATCTTGCCATATTTTCTTCAATGAATTGAAATA

CTTGGTGATACTGTCTTCGCCTTGACGGATATCACGGAGTTTTAGAGTCA

GCTCATACTTTTGAGATTGGTTTCCCAGATCAGAATACATCTGATTTACA

TTGTCCCAAAGTTCTTTCGCCGTAGGGTAACACATGTAGTTGGAGCTGAT

TTCCTCATCCATAGAGTTTACAAGTCACGTCATCACCATGGAGTTTTCAG

CATCCCAAGTTGTATGTGACGGATCTTTGATGGAGGACTCCTTTGCATCA

CCGGTTTGATATCCCATTCGTCCTCGTCAACGGATATACATTCGCACCGA

TTGAGACCAACGCAAGAAATTGGTTCCATTCAGACGAACAGAGGTGATTT

GGATGGAGTGAGAGTGGGATTGGGAAGGAAGATGCTTTGGTTCGGTAAGT

TGGCTGGACCTTGGGATGGATTCCGAGGTAACGTCGGACATGGTGAAGGC

AAGGGGAAATATCGTCAGAGATTGAGAATCGTCGGGTTGGCCTTCAACGA

GTAAATCTCGTGAAACCAAGTTGCTTGATGCTGAGTTGACTCATTGAGAA

TCGTCTGAGTTGACTCGGTTTCGTCGATTTCAAGCAAATCCGGCAGCGGT

GGCGGTGGCTAGGGCCGCGAGTGTTCGTCGGTGGCTGCAATTGAACCTCA

CGAAATGCAATCGAGTTGTAATCGCAAGATTTGTTTCGTACAAATCTTGC

GAGGGCAAGTTCGTCGGCTGCTAGGGCTCCAGCGAGGCTGTCGGCGGCGA

ATAGAGGGCCGACGAGGCTTCATTGTCGGTTGTAGAAGGCCGGAGAGTGT

TCGTCGGCGGCTGTAGAGGACTGACGAGGCTTCGTCAGTTGTAATGAGGG

CTCGTCGTCGGCTGTAGGGGAGATTTCGTTCGTCGCCTGGCTGTAAGGGA

CGTTGGCGAACCTTCGTCGGCTGTTGTTGGATGCCGGTGAACGTCGTCAA

CGACTATATTGGCCGGCGGTGAACGTCGAAAGCTTGTAAAGGGCCGGCAG

CGGCTGCAAAGGCTAAGGTTTTTTGTTTTAGTTCTGATGCCAAATTGAAA

TTAAATAATCAATTGTGTGTCTTTCTAATTGTACAATTAGGCTACATATA
GGAGTAATTAAGACTAAATAAGGAATTAACTACGACAGATAATACATTCC
TAATCTACATATTTGACTTTCTCTATATACAAATAATAACAATATTGAAT
ATTCCAACGGATATATCAGGTTTAGTAACAATCAAACAATTGAGCTTCCC
AACAAATCCTCCATACTATCTTGATTTCTTTAATTACTCTCCTCGGTTTA
CCTATAATTTTGTGTTTAGATCCACCGGTCAGTTGTTGGTTTGGACCTTA
ACATCTCTTTCTTTACTCAACAAGTCAATCACATATTTCCATTGAGAAAG
GTAAATACCTCGACTTGACTAAACAAATCTATACCCAAGAAATATTTCAA
CTGTCCCAAGTCCTCACTTTGAAACTTACCATGAAAAAATTGTTTGAATG
ATTCAATGCCGTTGTGGTTGTCTCCTGTAATCACGATATCATCAATATAC
ACAATCAATAATATTCTCCCTATGGTACCAAGATTGTAGAACACTGATTA
TCGACTTTACATTGTTGAAGACCAAACTTAGTAATCACGTCACTAAATCT
TCCAAACCATGCTCGAGGAGACTGTTTAATGCCATAAATTGATTTCTTCA
ACAAACATACCGATCCCGACTCTCCCTGAGCAACAAACCCTAGCGGCTTC
TCCATATACAACTCTTCAAGTATATTGCTGTTCAGGATTGCATTCTTGAT
GCCTAACTGATGCAACGAGCAATGAAATGTGGTTGTAAGAGAGATAAATA
GGCAAACAAATGCAAACTTGGAAATAGGAGAGGACATCTCTGAGTAATCA
ACACCATGGACTTGAGTCTAATCTTTGGCAACTAAGCGTGCCTTGTGTTG
ATCAATGGTGTCATCCGGGTGAACTTTGACGGTGTATATCCACTTACAAC
CTACAACTGTCTTGCCAGGTGGGAGAGAGACGAGATCCCACATGCTATCC
TAGTGTAAGGCAAGGATTTCTTCCCCCATTGCAGCCTTCCAACTAGGGTG
AGTGACTCAAACAGAGTTAGGAAGAGAAACAAAGGACAAAGAAGCAATAA
AAGCATAATAAGAAAGGGAAAGATGAGGATATGAAACAAAGTTAGATATA
GGATGTTTAGTACATGAACATTATCCCTTCCTAACAGCAATGAGTATGTC
CAAATCATGCGGTGATTGGATCGATGAGGAAGTATCTGGGGACACATGGA
CGATAGGCTCGTCATAGAAGATCGAGGGGGCAGATGAGACTTTCGGACGA
TAAGAGTAAACTTGCAAGGTAGGATAGGGAGGTGGGTGGGGAGCCTGGTT
TCCTTAGGAGATACAAATAGAGTTGGGGTAGGTAAGCTAGGCAAAGGAAG
TATAACTTCAAGATCTGAACGAGTGTGAGGCTCAAAAAAGTAAGGAGTGG
ATTCAAAGAAGGTAGCATCAACACACAAAAGAATGACGCAAAGAGGGACT
ATAGCGCTAATCCCTCTTTTGGGTGCTGGAGTAACCAAGGAAGACACAAT
TCTGGGCACGAGGGTCTAACTTGTCTTGACCAGGTACAAGTTGATGAACG
AAGAAGGCACAACCAAAAATACGAAAGTGTAAGCCGAAGGAGTGAGAATG
AGGGAAAATAACGGAGGGTGGACTTTTTCCATTCAACGAGGTGGCCGGAA
TGAGGCTCAATGCCGTGAGAAGGCGATGGTCGGAAGGAGGTTCAGTGAGG
CGGGAAGGCAATGGCGAGGAAGAGTAGTAATGATGATTGGATAGTAGCAG
TGGCGGTGGCTAGACAGGACCAGCGGCGGCAGCTGGACTGGAGCTGCGAC
AACTCCAGTGGCAGTGACTGGAAGGTGGTAAAGTGCCCCTGGGGGGGGCT
AGGATTTTCCTCGATGCCTCCTATTATTTTTCTCTTTTTATGCTCTGATA
CCAAAAAGGATTTTGGAGAGAAAGATAATTATTTTAGTATGATTTAATAA
TTCTTTACAATAAGGCTCCATAGTTCCTATTATATCACCATAACGGTGGG
CTAAATTAAGAAGAATATCACGCTAATAAAGGCTAATTAATGGACTAGAG
GAAGCTACCAAATTGGTCAGCATGACCTAAATAGGAAATAAGACCTAAAT
ACTATGTAAAATATTAAGTATTTCAATAGTCTCGAGTAGTGGCATGCTGA

GCTTGAGAGTGGGTCTCAGCAAACGAAGGGAGAGTAACCCCTTCAAGAAT
CTGTGAACAAACTGGCTCAAACTCTAGACGAAATCTGGCAAGGAACTTTA
GGACCTCTNTTAATTTTTTTTTTTTTTTTAACCATCTCTTCCTCTTGCTTC
TATATAACATTCACATTAGAGGACATTGGGAGTTTGGCACTGATAAAGTT
GTCATTCGTGAGTTTGACATTGATAAGGTTGGTAATAGTGAGAATGGAGA
GAGTTTGGGGTGCAGTAGAGGTGGATTGGAATGAGTAGACATGATTGATA
TAGGGCAGTGGGTGAAAAGGTATAGGGAAGAAACTGTGAATTCCAGAAAG
TTTGGGCTGTTTCTGTCAATTTCAGAAAGATTTGTGGTAGCTTCTATAAA
TTTTTAAAAACGTCGTTTGTAGGCTGAAAAATTGGGGGAAATCAGTGAGT
TTGAGAAAGTATTGGGTAGTTTCTGTGATTTGCGGAAAAAAAGTGGGGAC
TCTTTCTGAAGGATAAAAGAAAGTGCAGGGAAGAAGGAATTGAAAATCCA
ATGTTTGACTGACCTGCGACTAGAAGCTGAGAAGAAGCGATTGATCTACT
GATAATGCCTGGAGTGATTGGCTGGGTGCTTGAGGATTCCAGATTTAGAG
GCCAGAATTCTGTTTCGTGCAGGATTGGAGAGAGAAATTGAAGAGAAGAA
CAGCTGCAGATTTTGTGGAGGCTGGAGATAGGGTGGCTGCGGCAGAGGAG
TTCGCGAATTAGGCTTCGCGGAGGAAGAAGGGAGGATCGTTAGGTTGATG
CCATGTAAGTTGAAGAAATTCTGTATATTTTCTTTTAGAAGGCTCCAATT
TATACAATATACAAATAAATGATTTTGGAAATTGCTCCTTACTATTGGCT
TAATTTATTTCTCAATTAATTATAATTGATTGATTATTCATCGCTCAAAT
TTAATACTCAATTTAAGCGTAAAATATTATAGTGTAGTGTGCTTAAGTAA
GAGTGTCGAATCACAGGGATTGGATTGTAATTATTAATTATTTTCTTATT
TTTCGATAGGATTAAAGAAATTGGGTTTTGGATTTTTATTATAAAAATAG
AAAACTACTTAATTTAAAATACTAAATGATAAATGACCAGGTTGGGGGCA
TGTATTCGTTCTTCGATTATTCTAATGCACTTTATTCTCTATCTATTTTA
ATTCTATGTATAATGAAGTAATTTAACTACAAGAAAAATTACAACGAATG
TTTGATTATTTAATTGGCATGAACTATCTTCATAATTTGGTTCTTAATTT
ATGGTGAATCAATTCCTAAGCATAATTCATTTTATCAAATATAACGATTA
ATCGACCGTTTAATCGGTATGGACTATCTTCGGGATTCGATTCCTACAAA
CCTACGGTGAATCAATCCCTAAGTATAACCAATCCTATTAAACCGTATCA
ATTCCAATAAATACGGATTGTATATCGGTTGGATCTAAACTACAAGTATC
AGATCTAACCCGTAAGCATCAATAATTATTTCTAACATTTAAAAATGATA
AAAACAATAATTAATTTCATAAACAATGTAAGAACATGAATTTCTAGTCA
TACAATCTTATACATGGATATGATAAATCAAGAAATAAAATTGCATTGAG
AAATCTTAAACCAATATAGTCTAACCCGGTTTGCGCCTTAGCCGTTCATG
GCGTTGTCCTTCTTCCCAAATGATTTCCAATATAACCCAATGAAGAACTC
CCAAACAGTGGTCGAAGGAACCAAAATGAAGAAGAAAAAAGACTAAACTA
AAAAGTGCAAAAAATTCGTGAAAAAATAGCCCCCCAACCAAATGAAAGAA
ATGGTTTCTATATGATCTGCCGACCAATGGTCTTAAATGGCCACTTAAGG
ACATTCCATTTCCAGCGTAATGGTCGGAGGAAGTCTGAACCAAAAACTCT
TGAAAAGAAGTGAGAAAAAATGATCAATAACTCTCTCCAAAATAGCTAAT
GTTTTTCGGCTTAGAAAAAAAAATAAAAATGAATGATCATATCAATTACC
TTTTCTTCATATCCCAAATCCTCAATTTGATTCCCCTTAATTGCCTACTT
TTGCATTTTCCCCTTTAATTCTCATTTAATGGCCATTATGGTCATTAAAT
GGCCATACTGACCAATTGACCATTTCTCAGTAATGGACAAACTTGGTTTG

TAACATAGGCGTGCAAGGCGGGTCGTAGGTCGTGGGTCATGGGTTGCCGG
TCGGGACGGTTTTCTTCATTTTTGAACATTTTATGGTTATTTTCTTCATT
TGTCTTTTTAGTGTTTCTTTAATTATTTCCAAGCTTTTACCTACAAAAAC
AATGAATAGTATTATTACTTATTAAAAATTAATAAAAAGACTATCAAAAG
TGTAGATTAAGGGGTGAAAATTTGGTATAAAATTGGGAACAATCAATTGT
ACAATTGGTGATATGTTAAAATCAAGGAGGTCCAACGCCCGACACCCCCA
ACGTCACCAGGATCATGTGTAGAAGCTCTATCCATATTAAGCGCCAAACC
CATACGTGGATGTGTCCAGTATATCAGCATTGGAGTTTTCTTTTTGTCGT
CTTTATGTCTAAGGCCAAAGAAATGAGAGCACCACTGATCATTAGCAGAT
GTAGGAGAATGTATTTCTGTAAAGAACTAGAAATATCATGGAGCAGACAA
AATGAATGGTAAGCTGCATTTTCATAGGTTTATTGTAAAAAATGGAAGCA
CATATGGATTTCCAGTTTTACAAAAATAATCCGAATGGGAATGTAGTGCA
GAAGCTGTTTATGAAAAAGGGTCACAGACTGTTCCACCACTTAGCTGGTC
AGAAATTTAGGGAACATTTGCATTAACCCCCATCCTATTCCCAAAATACT
GTCATGTTTCAATGGCCATCTCCCCTAGCAAAAATAGGTGATCTTTGCAT
TTGGATTTAGGAAACAAATGACAGCAAAAACATTTGGATACAATGGGAAT
TCCTTTGATAATCAGATTCATATTGTTGAATCCCACCTCGCTTATGTTAC
CTGATCCTAAGTTATATATAAAGCCTAGGGCAATCCTCCTCTTGCAAGGC
GTCTTTTGAGGGTGAGTTAGGCCCATTGGGTTCCCACATGATATCTGAGC
CAGGTTTTTGTCTGATGTTGGGCTTGTTGAATCCCACATCAATGATGAGT
CTCGAGATCCGTTCATAGTGGTTTGTCAAAGTCCACCCTGCGCGTGAGGG
GGTGTGTTGAATCTTTTCTTGCTTGTGTTACCTTATCCTGAGCTATATAT
AAAGCTAGGTCAATCCTCCTTTTGCAAGGCGTCTTTTGAGAGTGAGTTAT
GCCCTTGGGTTCCTACACATACTCATAGGAAAGCAATTAAAATGGATTCT
CCCAAAAAAAAAAAAAAAAAAAAAATTGACACTGAAATCTTTTTTGGAAT
AAATGGATGCCAGAATTTTGAAACATACCAATCCTCCTGGTATTATCTGA
TTAGGTTCCAAGCCGTCTTAACAGAAAAATTACCGTCGGAATCTGCAGCC
AGAATCATCTGATCCTCCTCCTCATGGTGCAGACCTGTGTACATAAAAAT
CATATCTGCAATATCAGGAGGCAATAAAACACCGATCAAATTAGCTTTCC
AAGTACCATTTTCCTACATAACTTAATTTTTAAGTGAGATGATGAGGAAT
TGAACAAGGTAGTATCAGGGGAGTGAAAGTAAGGATCCCACCATGCTGAT
ATACTCCACCCTTGCCAATCAGCCAGATGATATGATCTTCAACCACTTCT
CTAATATCCAGCATGTGTACCACATTTCTGACTGCCCATAAGACCACTTG
AAGTTATGAAAAAAATGCCCCTTTCTATATTTGTCTTCAAGAAATTTGGA
CCAAAGGGATGTGCCCCCCCTGGATTTCCACCACAATTTTACACCTAAAG
GCCCTCACCACATTTGCTAGACTTCTCAGCCCAAGACCACCTTCAGAGGT
TGATGGATGAATATCCTTCCAAGCTTTCCAATGCCAATGTTTTTTCTGAT
CAGTGCTAACCTTGAAAAAATCTGCAAAATTTTTTTCGGTCTCTCTCAAA
ACAGCTTTTGGAGGTTGCAAGACAGCAATGCTAACTTTTCATTTACCAAT
GAAACCCTTTTTTCCTTGGTACCAGTGAAACCTTTAAAAGTAAAACTATT
CAAGTTAGAATTTTCGGATTGCCAAATTTAGTGTTTCAAAAACGATATTG
TAGTGTGTTTTGGAACAATAGTACCAATTTTAGGGTCGATATTGTTCTGG
TGAATTGTAGTAACTTTTGCTCTGGTGCACCTCATCTTATGGCATGTGGG
TCTTGCTTATGGGCATGTTTTGTTCCATATGATTTCTCCGTCCNCTTTTT

CTGTTTTTATTGTTCCAATTCTTTATTATTTACGTATTCATTTTCATCAG
ATTTTTATTTCTTTTGGTGCACATCTTGGACCGCATTATTTACCTTGTCA
ACTCCATGTTTTTTTTTTTTTTGGAATGTTTCTGTAGGTTTTGGCTGATA
GTGTCGGCATCCCTTGCCAATTGGTGAAGGGACAACAGTATACCGGTTCA
GATGATGTAGCAGTGACCTTAGTAAAGATTGATGATGGAAGGTTATTCTT
TTCCTGTAAAATTGATGGTTGAAGGATTATTCTTTTCTTACCATACATTA
TGAAAATGAATTATTACTCATCAAGATCTTATGAACTGCTTTGGTATCTT
TGTTACCCAGCTGGTGCACACTACTTTGCAGTAGGCCATTTCCTCACTAA
AGGAGTTCAAACATTGCTAGAATAGGCTAGAGTAGAATTGTTCCTCAAGT
TTTGAAGAAAACAAAAGAAGGAAAGAATGAAAAGAGAAAAAATGAAAAAA
GAAAAATAAATAAAAAGGATACAAAAGGACGGGCCAATCGGTTAATGTGG
TGACTGGTGACTAATATTCCTCAAACAGCAAACCTCTAGCTTCTTTAAGG
GGTAAAATGACATTTTTATTGAGCAGTAACATACATTTAATAAAATCTTG
CTCTTATTTTGCTTTGCTGAACATTTACTTTTAATTCTCGATATTTATCT
CCTTTATTTTTTATCACACCTTATATTTTTCCTGATAAAAACATTCAAGT
GGTACAATAGTAGTGAATAAAATCATTTTATCATTTTCAGGACTGTAAAA
ATAGTAGTTGCACTCTTTCGTTTAGTCCTACAAGCACTGAACCTAAAACC
ATATTGCAAATGGTTGCGTAGACCCTTTTGTGTAATAAGCTAGTTTTGGA
GGTTCCATTGAACGATTTGGGACATAACACTCACTCTCGCGTGCAACTCT
GATCGCACATGAAGAGATTGAATAAACATAAAAAAATTGGTCAGAATAGA
GGTGAGGCTTCCCAAAATCCATTTATGATACTATCTTTACTAGAGTCCAA
CCTAAAACCATATGACAATAATTGTAGTCCCTTCGACTAATGTCATTAGT
TGGGAAGCCACATCAAATATTTATCTTTTAACATCGATGCTTCTTAAATA
GTTTATTTGGCAGAAAATGTTAAATCTATTATATAATTATTTCATTTGGA
TCTGTAGGTTACTATAAGTACAGTAATGTGGTAGTTAAATTTCAGGGAAT
ACATTGTTGATTTGATGGCGGATCCAGGGACACTCATTCCATCAGGCGCA
GCAGGATTATATGTAGAATATGAAGTACCATTATTTTCCACTAGTCCATG
GTCTAAGGATAGAGATTTGTCTCAGGTAGTTCCTTCTACTAGTAGGGTTA
GTGGATCATTTGAAGAACATTCTATGTTTGGGACAGTTGACAAAAGATCC
ATGTTTACAATTCGTAATGCTGTGGGAAACGAATCTGATGATAGAGGCAA
AAATATGCATTCTGCAAATTTAGTGCAACAGGGTGGAGCTGAGGAAGGAT
CCACAAGCTACTCAGATGATTTAAAAAATCCATGCAAAGTAAAGGAGAAA
ATTACTTGGGAAATTCCACGTAGGCCTAGTCTTTGCATGCATGCAAGATC
TCCGTCATGGACTGAAGGTGTAACTTCTCCAGCTGTACGAAGAATGAAGG
TGAAGGATGTTTCTCAATACATGATTGATGCAAGCAAAGAAAATCCACAC
TTAGCTCAGAAACTTCATGACGTGTTACTTGAAAGTGGCGTTGTAGCTCC
TCCAAATTTGTTTACTGAAATCTACCCTGAACGGTTAAATGTCTTGCCTT
CCAAGTTTCCTGCTGGAGTTAAAGGGGAGAACAAAGAGGGTGATGATATC
CAAAATAAGTGTCGAAATGATCTTAACCGAGCTAGCTTTTTGCCTCCTCT
GCCTTATCATGGTGTGAAATCAAAAGAAAGTTCTAGCGGACAACCAGAGC
ATCAGCTTGATTCAAGGGATGGAACTGGACTGCATGTTTCATCACAATCT
GGATCAAATGCAGTAAAATATGAAAAAAATGTTCCTGTTGCTGCCGCTGC
CGCTGCCGCTGCTGCTGTTGTGATGTCTTCAATGCTGGTTGCCGCAGCAA
AAGTCAGCACTGCCTCCAACCTTGAACTCCCTGTGGCAGCTGCAGCCACA

GCCACCGCTGCAGCTGTGGTAGCAACCAGTGCAGCTGTCAGCTTCCAGAA
TGAGAACATGGACTGTGCAAGAAGCGATGGAGATGAGTATGCTGCTGTTT
GTGAGTCACGTGATAGTGTTGACGGGGGGAATGATGCTGTGGCCTCAAGC
CAAGAGGGTGATAGAATATCTGATAGGTCAACAGGTAATGATAGTGCAAA
GTCTGATGCAGCAATTGATGATGTAGCTGAGTGTGAGATTCAATGGGAGG
ATGTCACCTTGGGTGAGCGTATCGGACTTGGTACAAACCCACACATTAAA
GTCTAATTTGTGTGCTTCACATTTAGTATTCTGGTTTTATTTTGCCACAT
ACACATGTTTGTAGAATCATATATTTGGTATATTCTTTCTGTTCCAGGAT
CATATGGTGAGGTATATCGTGGAGACTGGCATGGAACTGTGAGTTTTACC
TATCTCTGATGCTTATTACGGGTCTCTTTCTTCCTTGTTAGTTGCTCCCT
CCCTCCCCTTTCCCCCGCCGGTGGTCCGGATACAAGGGTAACAATTAGTA
CTTCTGCATTGTGGCCGTTACTCTAGTTTTCAGTTATTATGGAGTCTCTG
GACTGAGAATGAGAGAAATGTAAATTGCATTTGCAACTTCAGTGGACTTA
AAAGGTGCAGGATACTGAAAACATATGTTTGAACCATATCTCTTACCCAC
TAATAAATACCTCTTCATGCATTTTTTATTTTAAATTTCTTTGATAAGAG
TATGTGTGATGTGCTCTGTGTAAACTTCAGGCCCATGGTTCCATTAAGTT
TGAAATCTTTTGCTCTGGTCTAACATTCTGTTATCTAAGTCCAAGCTAGA
AAACAAATTTGCGTTTGAATAATACACTGTTCATCTTTTTTAGTCTATCT
CAAGTATGTCTATTTTTGTGTTTTCATTTGTTAGGGAAGCTCCTGTCTCT
ATATTTCATTATACAAGTACAAACTGCTTGCTCAGATTAGAGGGGAGTCA
TTTTATTATTTTATGGTGAATCAGTAGGGATGGGTCTTATTGTCCATCAG
CGAATCTTTCTATTGCTTACCCACATTGCTAGTTTGCTGTTTCTCCTCAA
ACGTCCCTGTCCCTTTATATTTTGCATGGCCTGAGAAAAGATTATGGTGG
AATGTTGTCTGTAGACAGAAAAGAAACATCCTTGTTTCGTGGACAGTAAA
TTTTAGAACCAATCCCTTGGTGACTTATCTGATGTAATTTTTATGGAAGA
TCTATCCATCCCTCAATAATTTGAACCTATTTCTTTTTATGTCATTGTCT
TTTCTGCATGTTTCATTCCCTTAACATGTTTCAGGCCCATACCGTGACAC
ACATTACTTTAAGTTGAACATGCATAATTTACAAGCAGCATCTTTATTGT
TCAGTCACGTGTGTTGTCAAGATTTTGGATTCTGGATCATCTAACTTCAC
ATTGATAATAATATTTGCAATTATGGTTATCATAAGTATTGTTTGTTTGT
TGATCCTTGTTAGTGTTCTAGTTAATTCTATTATCTTCCCTTTCTCGAAA
GGTAAATATTTGAACTGTAAGCACTGATATCTCAGGAAGTCGCTGTGAAG
AAGTTCCTTGACCAGGATTTAACCGGTGAATCCCTTGAAGAATTCATTAG
CGAGGTGCATGTTTCTTCTCTTATATGTTCTTGTCCTTATTAGCCTCTTG
AGCAAAGCCTACCTTTTTGATAAATACTAGCTTCGTGAACTTGTTAAATA
TCCCTTACAGTTCCATAGAGTGGGATTTAGGATTTTTAGGAAATTCTACT
GCTACTGATGCAGTATTAGAATAGTCGCACTTGCTGCTATGTTTGCATTT
TGATTGACCATTGATCAAGCACACTTATGTGAGGCAAAGGAATAAGGAGA
ATAAGGAATAGAAGCTTCTATTTTCTGTTGGGTTGTTAAGTTGTTAAGCT
GTTGCAGCTGTGTTGGTGGAGTTGTTTCAGCTGGGTAGCTGTTAAATTTA
GGAGATACTGTCAAGTATAAATAGCAGAGGGAAAAGAAGAAAAGGCACCG
AAATTGTGAGGTCATAATTGGGAGAAACCACGTTCTCGAAATCCTGGAAG
TTCATTTACTGTTTTCCTATATTTTCATTCTTCAATTACTGTTATCTGTA
TTGGTTCTCATTGAATCAATCACGTTTTCTTTCAATTTCTCTATTATTTC

AATCAATTGGTCAAGATTGTGAGTGTGATTCTATCAGATTTCAACATAGC
ATTGATGTTACAAAGGTGATGCACATAGTATATAAACTGTGTTGGAGCGA
GTAATTCTTCATAGCTGGGGCACTGTGACCAAGTATATGTGGGCTCGGAC
TTGACCGGGGGGGTGAATTGGATCATGAAACTTTTTCGTCTTTTTCTTTA
TCTCCACAAATTCTTATTACTTAAACCATTCCCTTTATTAGATAATGAAT
TATAAAGATAATGTGAAAGGATTCATTTAGTAGATTGTTTGTCTCAATGT
GATATATTTAACAGTTTTGTGTAATGCCGCGGGATAAAGTGATTACAATC
CAAACAACATATAACAATAAATTATAGGAGGATAGAGATAGATACAATCA
ACGAAAAAACACACAAGGATTTTGATAGTAGTTCGACAAAATCAATTTTC
CTACGTCTACTCCTCAGAGTTTCCTCTGAGAGTTCTAGTCCAATAAGTAT
TCAGATCTTCTTCCCTGGTGAAGACCAAACCGTTACAATCTTTCACAGGA
CCAGATTATTTATGTTCTACTACACTCTCACTAGACAAGGTCGTTTATGC
TTAGTTACAATCTCTCACAAGGTAAGGTCGTTTATACCTTACTATTCTCT
CACAAGACAAGCTACATTTATGTTTTTCTACATTTTATCACTAGACAAGA
TACGTTTATGTCTAGCTACAATCTCTCACAAGGCAAGCTACATTTATGCC
TTACTATACTTTTACCAAGGTTCGTACCCCTGGGATTTCTTAATCCCTTT
CCCTAGGCAAAGATCATGCCTTACCAAGGTTCACCACCTTAGATTTCTCA
AGTACAACAATAATCGATACAACTCTCAAACAAGCAAATACAACTACGAC
AACATGTATACGAAACTCAAACTAATCTCTATCTACTCTTGAGATTATGG
CGATTCAATCTCTCGAATTATCTCTTCCCTATAGAGATTTGGCCAATGAC
TATATATTGATTCTTCTCTTAATCTCTTCATCACTCGGCTCAATAATATT
CTACTTGTCATCTTTCAAACATCATCCCAATGAGATGTATATGTGATGTG
CGAACGCTTAACCATCTAAGTATACTGGACATGTGACTTCAGACTTTGTG
ATCTATCCCAAGGTCTGACGAAGCTTCTAAACACTCTCTTTAAGATGTAT
ATCCAGGAATAAGCCTTCTAGTGTTTTTTGCTCACATGCAATGTACACTT
TATTTATTTCATGAATATTCTTGAGGTGCAAAGTGTAAACAAGACTAATG
ATATTCCTATTAAGGGTCAATTCTAGTTTTAAGATTCAACCCATTTATGT
AAGTGTTAGATCACTTTGTTTTCTTAATCACAGAAGATGATATAACATGA
ATACACAATGACAATGATCATGATACACATAAGCACATAAGAGCAAGTAG
GAAGTCATAACATCATGTTCATGATATTTGATTAGCATTTTACTTTAATC
AATAGCTTGTGATATAGTGCACACGTAATCACATATAAGATGTTATCAAT
CAGTAATGTGATATAAAGAATCAGTTTATTAGCTCAGCTCAACATATAAT
ATGTATGATTTTTTTTTTCTTTTTTGTTGTGAAGGTAAGACAATTTTCAA
TAAAAAACTCTTAGAAAGCTTAGTGAAAATGTCCAATTTAGCTCACCTCT
TGTTGGAATGGTACATGGTGGATTTAAGATCTTGATGAGTAATTTAATCT
CGCTGGCAGTGCGAGTTCTTCCTGTGTCCAATATACCAATTCCAAATAAT
TCATGATTAAGCAAAAAATTCTTTATGATATCTTTTACAAAAGCACTTAG
ATTCTCCAAGACGATAGATTTCAAGGAGTAATGAAAATACTCTTAGGTTG
CGTTTGACAATATTAAATAATGGGATTTCGAAATCCCATAATTTAACAAA
TCCCTTGTTTGGATAGAACAAAAACAATAGAACAAAAACCAAACATCACC
TCAATTGGATGAACTATGAACAGTAAAATGAAAATGGAAACAGGAATCGA
AATCAGAAATCGGGATACCCCACGAAATTGGAACCCTAAATTTAAAATCC
TAATCCGAAATTGAGAACCCTAAATTGAGAACCACGAATTGGAATCCTAA
ATTGAGAACCCTAATCAGAAATTGAGAACTCTAATAAAAAATTGGAACCC

TAAATTGAGAACCCTAATCAACAATACCCCATGAAATTGAACCCTAAACC
CTTACCTGAAGGCGAACGATCGATGAAGGAGACGACGAAGACGAAGCTGC
GATGATCGATGAAACTGCGACGACGACCGACGAAGCTGGGACGACCGATG
AAGCTGCGACGACGATGAAGACGAACGACAATGAAGAAGACGAACAAAGA
AGACGGCGAAGCTGCGACGATTGATTAACGAAGATGATCGATGAGCAAGA
AGGATTTGTCAAATTCTGCCTATTTCAAAGGGATTTCAAAATCCTTTCTT
TTGCCCTTTTTTTAAATTTAAAGGATTTTATAATTCCCTCCCTCCTAAAA
CCCAAACAATGAATTTGTCAAAAGAGGATTTTAAAATCCTCTAAACAAAT
GAAATCTTCCCTACAAATCCTTTACCTAAACACAACCTTAGAAAGCTCAT
TGTGAAATCTTCAATCTTTTTATCCCGGAATCAATTTTATATACTTTATC
ATATAGAGATATAGATAAGATCAATATAAGCGATCTCTAGATGTATAAGT
GTGCATATAGGTAAATTAATCATTTTACCAAGCATGGACATGCATAAAGT
GTCTAGACAAATAACATGGAGCTATACGATATGTTCTAGACTTTCTCAAA
TAAGCACATAATGGGCATGTTCTAAGATTTAAACAATTAAGCGCAAAGTA
ATGATTAATGGAACAAACTCACCAGATGTAAAGATAGTGATGTGAGCATG
TTGCATCTTCTGGGATGACCAATTTTCCTCCTTTTCAATTCCCTCATGAC
CTTAGTGCATCACAAGATTCAGCTTGAAAGATCTACTCTAGGTTTTGGCA
CTTATAGGATCTAACTCTTGTTTTTGTTGTGTTTTCTCACATCATATCAC
TCATTTAAGAACTAAGAGGTGTTTCTAATGGATGGTATACATATATCAAT
TAAGTTGAGACCTTCATTCGAAACTCCCCTTAAAAAAAATGTTAAGATCT
TCATCCTTGTGCAATTCATGCAACCAAGTGGGGTTTATATAGGAATGGTC
AAGGTAGTGACTGTTGCACCAAATCTAAGAACAAAGGGTTGTTTATCCAT
TACAATTAATCTGATCACTAATAGTCATGTAATCTGATCACTAATAGTCA
TCTACAATTTTCTAATTTGATTAATTTGGGCAAGAACCCGCTGGGAGACA
TGGTGCATCAAGAAAGGCTGAAATGCCCATTGATTTTGGTCCATAATTCG
TAATTCTTGATTCAAATGATAGGAAATAAATTCAATCAAAGATTGACATC
TTCAAGAATTACTTTGGTATTCAAAACAAGGTCGAATTTGATTTTTCCCT
CAATGTATACAAATAGATCCTGTAAATTGGTCAACCGGTTTGGCTGATTG
GTTGACCGATATTCTTGAAAGGTTATTCAGAACATCCTACTGGTGTGATT
AGTTGACCGATTGTTCAGATTATATTAGATTGGGACCTACTGCCGTAATC
GGTTGACCGACATTCTTTAATCGGTTGACCGATTGAGGTTCTTTCTTAGT
CAATTAGCCTATTGGTGGAATTGTTTTTCGGTCGACTAGTTAAGTCTATT
TGTTTTCATTTTAGGGTATTGGTAGGACCAGTTGACATGTTGAGTGGTTC
ATCAACCGGTTCAAACTCATTTCAATTTTAAGCCTAAATTTCATTTTATT
TTTAATCAACATCAATTATATTTTTGACTCAAATCCAATTTTGATAATCT
TAAGCATTTTAACATACATATTTTCAGCATTAAAGAAGCCACATAATTGT
CTAGTAGAGGCTTATCTTGATATACATCTTGAGAGTATTATTGCCTTAAG
ACTCGATTTTGGTTGTGGTGTAGCTTCTTGATCTTCTTCCGCAACGAGTC
GTTTGACTTTCTTCTTCTTCTTTGATTGCCTCTTTCTTATTTCATGCAAT
GTCTCAGTCTTCTTTCATCCTTGTTGTTTCATTCGTAAGTTTGTACTTTT
GGTTTTCCTGCAAGATCACTTACTCAAGAATGCTACCACGATTAGTACAT
GGATAGTAATAATCAATATATTCAATTATATATATATATTCCTGAATGAT
CACATGTATACATAAACATAGTATATCTAAAGAATCTCAGGTAATCACAT
GTATCACATATGTGTGAATAGTGACATGGATTGGTATAGCGTAATTGCAT

AGTCACATAGGATATAAGCATGTAGAGATATAAGCTTGATGTAAGCATTA
TTCCCCTCAATCCTTTACATTGACATCCATAGTATGACATCATCAATACC
TAATATTTCAACAGTATAGTGGTAGGTACAAAAATATAAAATTATAGATT
TTTTATTGTTTAGTCCAAATTTTGCACTCTCATTCCTTAGTGAAACTTTT
TAACAAAAAGGTTATTATTTAATCCCTCTCTTATTATTTAGTCTTTTGGC
AGTTAAAAATAATAATGAACAGATAATTATACCCTTATATAAATATAAAA
CCCTAGCCCATCTCTTTCTACTCCAAAATAGGGTTTTTCTCTCTCTCTCT
CTCGACCACAGGGGTGTGTATCTCTCTCTAAATTGAGAATTGAAAAAGCT
CTTTTGTGGAATTTATACTTTTATGGTCTTCGATAATGGTGATATAAATC
GGACGTATCACCATTATTTCTCCAAACAAGTTCATTTTTATTCGGCCAGA
AGAAATTAAATTATCCAGCCATAACATTTCCTCTCTCTGTTTCGTGCTCT
CTTTCTCTCTCCGCCGCCAACTGCCTCTCTCAGTCGCGATGGCAACGATG
GTGTTCCTCTCTCCCTCTCATGCTTTCTCTCTCTTCGCCACCCACCGCCT
CTATCTCAGCCGTGATCGCAACGATTCTGATGGCTATGAACCGATAACAC
GGTGGTGTTTAGATCTGGTGGCCATGAACCGATGGCGACGGATGTGGTGG
CGTTCAGATCAGGGTCGGGCCTGGACAACATCAGATCTAAGTGGTAGGTT
GGCGGCGGCGGGGATGTGTGTGCTTGAAAATACGTTGGCTAGGGTTTTCA
AACCTTTTTTTTGGTAATGGTTGGTGATGGTCGGCGAGCTAGGGTTTTCA
TTGTTAACATTCACAGACCAGTATGAACGTTCTTAGATCTGGAACAGTGA
ACAAGGTTTATCGTTGATCGTATACTGATGAACATTCTCGGACCTGGATC
TGAATCTAATGAACACTTGCGGCTCTTTCTCTCTCTCTCTCTTAGATCTG
GAATATTCAACAAGAGATGAACGGACGACGACCTTAATTGTTTTTTGATT
TAAAATGTTCACTGAATGTTCATGTGTTTGGTGAAATATTTACATTAGTT
TGTGTTTTTGGTCAATTTTTTGTGATTCGATTTTGGTGTTTTGTGAATAT
TCACATCAGTTTCTTTTTATTGATCTTAGAGTTTATATTAACATTTTGTA
AATGTTGATAATTTTTTTTTTCGATGTTTGATTTTGTTGTTCTCTTTGAT
TTGTGAACGTTTGGTGATTGTTCTTGTTTGATTGGTTTGTTTTTTGCGAC
TGTTTATATTGTTGTTGTTGTTTGTTTTTTGTTGTTTTTTTTTTTTTTTT
TTTTTTTTTTTTTAATCAAGGGGTTTTCAATGTCTTGATTTATTGTTTCT
TAATTGTTTTGGTGAATATTCATGTGGTTTAGTGAAATGTTCACAAGAGT
TTATTTTTCGTGATCTTAGTTTTTTTCCCCTAAAATATTCATAACCATTT
TAAATAGTAGTGTGATTTATATAATACAGAACATGAATATTCTTTTTTCG
TAGATTTTGTGAACATAACAAAAGGACGTAAAACTAAAGAACACGTGAAC
ATAACAAAAACGTAATTTTGGTCACATAAAAACAATGAACATTCACAGGA
GCACAAATCACAAGATGTGAAAATGTATTAAAATGTTTAGAAAAAAAAAA
AAGAATAAAAATGTGAATGTTCATGAATTTTTTTTTTTGGTTACAGAATG
GCATTGTACGTAAATAATGGGAAAAAAATGTTCAAATAACTGAAAACTATT
TTCTTAAAATTTCTAAGGACTAAAAACTTTATTATGTTAGTTGGGGGTCC
ATTGTCCGATTTATATCTCTCTTAAAGACTATATATTAAATTTCCCTAAA
ATTATATTGTTGAATACTGATCGATTTGGTTACCAATTAAAACCATTCTT
TTTCTCGTGGAAGTGTTGTACAATCGTGTTTTTTTCACCATCACTAATTT
TTTTTATTTTGCATAACAAAATTACCATTTAACCATTGGATTCATATTGG
TTTTGCATAGACAATAATTTACAAATTTCATAGGAAAAATTTATCTTAAC
CATACTCAGGTATTGGTCTTCGTGATAATTTCTAAGGGAGAGAAGAAAAG

TCAAGGATCTTATATACGGAAGGTGATGGGAAGAGTAGAAAAATAAATTA
CGCACTTATTACAGGCGTAAATCAAGGCTAAATTGAAGAATAAAAAAACT
CTTAAATTTGAAAAATCTCTATATACATTTTGATTATTGTAATCTCTGAT
TGTCCCTCTAAACTTGCGCCTCCTAAAGTAATATCTAGGCTTCCTCCATA
TAAAGGAGTCTATGTCATCCCTAGAGGACAGATTCCTACAATTGAATTGA
TTGAATAAAAGAACTGTTCTTGATTTTATATTTTTGTATTCATTTCCATT
GTTTATTTTTTATTCAAATCCGGTCTTACAGGGTAGGGGGAAAATGGACT
TAAATCATTGGTTGTGTCAAGTAAGAAAGGGAAAAGGCGAAAAGTCAAAA
TATGTAAGGGCCAAAACGAAATAATTTATCATAGCTGCAGATAAAAGTTA
AAAGTAGGAGCCAATAAATAAAACTTTTAAGATTGTTCAGAAATCCACCG
CGGGCCTCAATGCTTCTGGCCTGTACAGACCCCCTCTCCTTTATCTAAGT
TTTATTGAATCTCTACCAAAATTTCAAGACCAATTTCTTCGCATGTTATG
TAATACTTCTAAAATGTAATTCTTATTGCCTTTTTACCTTCAACTACTGA
GTTGATTCTATCGAACAAGATTGTCAAAGCTTCTAAAGATGTAATGAAGT
AAAAGTATGATCTCATTTCAGAGATGGATCCTTTATGCTACGGTCCTTGA
TCTTTTGCTCCTTAAATCTTCATGAACTTCATAAGCTGATTTTGGAACCA
ATAGCCATTATGTTATCAGCTATCTTGTCTTCTTTTTTGAGGTTAAACAT
GCTGGTCTGGAGGTTATCTGTACCCTTTTTTTTTGGGGGGGGACTTTGAA
CTATTCAGTATTTTGGTAGAGAAATCGTTTCAGTTTTGCAATTATCGAGG
TTAAGTTTATCAGAGAATCTTTTGAAAGCAAAATGTTTCCTAGGAAGTGA
TAGTAACTCGAATAGTTGTCTGACCATATTTTCCACTCCTTGACTCCCTT
AATGACACTATTACATCCATCTCAATCTCGATTTAGTTATAAGTCTATGT
TATTACTTATAGCATATTTTGAATTAGAAACTAACATCAATAATTTTCCT
TGTTTTACCTGAAAGGTACGCATCATGAAAAGAGTTAGGCACCCTAATGT
TGTTCTTTTCATGGGAGCTGTTACTCGACCTCCAAATCTTTCAATTGTTA
CTGAATTTCTTCCTAGGTATGTCTAAAGTGTATTAATTCATCCATTTTTT
TCACTTTCGCGATAAATTTTTTGAGAGTGTCTCTGTTAAATTGTATTGTC
TTGTTTAAAGAAATCAAGCATTGTTGCATGCCATGTAACATTAAATAATT
CTCACATACAATACGATATTTTGAACTGACCCCAAACCTTTTAGTTTTCT
TGTTGCTGATGCCTTTCCTTTAACTGGTGGTGAGTGAGAACAAGGGAAAA
TACAGCCAATTTTTTTTTTTTAGTACTAATAATATGTTGTTAAGTCCCATA
TTCATTTAGTGAGACTCAACAAACTAACGTAACTGTCCAAGGAGCTCTTT
CATGGATGCTGTTACATGTTATTGGAGCTGTTTCCCATGCTGGGTTCTTC
CGATCAACATGTTTTTGGTGGATGCTCTGTCATGGTTTATTTTACTTTAT
TTTATTAAGGGGAACCCCGGCGGAACAGTAAAAGCTTGGGTTAATTGCCT
TTTATTCCCGTCCTATAACCTTGTTTCACTTTACCCCTGTATACCTTAAA
CTTAATACACATAAACTCCTCCCACCAAAAAAATGAAAAAAAAACTGGGAT
TTGTTAGGTTTAACTGAATGTGTCATAAACACACTTAAAGTGTGTGATTC
TTAAAACAAATAATTTTTTCATGTCAAAATTACCCCTCTGAATTTGTTTG
TACACTTCTCTCCATTATTTTATTTACCTTTTCGTCTTTAAAAATAAAAC
TAATAAGAGAGGAGAGAGACTGAGTATATAGACTTCCTGGGTTCATTGAA
GAGAGTCAGAAAGATCTCAAATGGAACCTAAATTTAAGGCGAATTTTAAG
CTCCATCACCAATTTAATTTCAATGCAAAAATAAAAAAGTTAAAAAACAT
CAATTATATTTAAAAAAGACTTCCTCAATGGATCTCGTCTGCCTGCAGCC

TCATCACTTTGATTGAATTTAATTCATCACAGCGCAATTACTTTTCTGAT
TTTTTCATTTTTATTTCTCTTCTTACTTTGTCGAGTAGATACTAACTGGG
GTTTTCCCTAGATATTTGGAAGGGGTGGTGGAGGTGCCTGAGACGAAGGA
AACATCTAGGTTATTAGGGCCTTTGAATGGGGATGATGGAGAAATTGATG
GGTTTTGAATGGGGTTGATGGAGAATGTGATGGGTTGTTAGGGTTTTTGA
ATGGGTGTGATGGAGAGTGAAGCACTTCTGTTTTGTTTTCAAATGTGTGA
CGCTGGGTTTGCCAAATGAAAGTGCTTCAGTTTCGACTTTGATATTGAAA
AAATTACCAATGTTGAAAGCAAAAACCCTAGTGAAAACCTTCGGACTAAA
AGAGAATTGGTAACGTGGCTTAAAAGTGAATCATGCTAGAAATGCCTTAT
ACAAGTGGTTCTATAACTGAGGGGGTTGATGAGGTTCGGGGTCATCGAGG
AAACACTTCATAGTAAGCTTGATAACCAAAAGTGGCACCTTGAGAGACCA
AAAAACGAAGAGAAGTAAATTTTTCAACCAAAGAAAAAGTTTCGAAGTAA
TCAAGAACATAAATTTGTGTGTATCCTTTAATCACCAAGCGAGATTTCAA
TCTGCTAAATCCCACATCGCCTTTGTGATTATGAAACTAGTGAATATATG
AAAGGAGTTGCATCACCTAATTCCATATGGTTTTAGATTGGGGCCTCCTA
GCATGGTATCAATGGTGGGTTTGGGAAGTCTCGTTTATTTGTCCCAGAGT
TGTTTGGAGGGTGAATATTGAATTTCATATCGCTTTCTGAGGCTTCTCAA
TCAAGTCAATGTAGGAACTACTCCACCTATTACTATATCGTTTTAGGTTG
GAACCTCCTAACACAATCTATGGACAATAGCGTGAAGATGAATTTTTACA
TTATTTACACGTTTGAAATTAAAAATGTTTTTTCCAGTAGAAAAGTGTCA
AGGTCCCAATATTATTTTGTTTCAATGCAAGCATTACATCCTCCTTGCTT
TCTTCCACCCAAAATGAGATGAGATTTGCCAGAATGACATAAGGAACGAG
ATAAGGCCTGTGGAATAGACTTAGGGAGAGAAACAAATGGCAAAGGAGCA
ATAAAGGAAGAATATAAATGGGACAACTATGATACAAAGAAAATCTCTGC
TTGATCTTAAATTAACCAAAGTCTTTGATTTTAGTCGTCAATTAAAATAC
CATGTGATTATTTGAAATTTTTTTTGCGGGTTGTCTCTTACGATTAAGTT
GTCACCAAAAAAAAATAGGCACAATAATAAGCAAGTTGTGATACAAAGAT
TTTTACCTGGAAACTCTTGGGAAAAATTACGGTGTTGCTTCTCGAGACAC
TAAGGTGGCGTTTGATTAGTGTTCCCACTTTTCTCAGCACTACTGGGATG
GGAACAAAATTGTGTTTGGTAACGACAATTTTTTTTGTGCTGGAAACAG
AATCGCCTTTGGTAACACATAATTCTGTTTCTTTCCGAGAACAGAATGGC
ATTTGGTAACGAATTATTCTGGAATTATTTCCATAACAACAGCAACAACA
AGACAAAACAAAGATTCCATAATTCCTACAGCAAGCTCTTGCAAAAACCA
ACACAATCATCTTCCATAATTCATCAAAATAGAATAGGGAGATAGAGAAA
GAAAGGGAGAGAGACTGATGAAATCGAACCTAAAATCAAAGATTCAACAT
TTTAGATCTCAAAGATTCAGGAAATCAAACCCTAATTCTAAGAAATCGAA
CCTAAAATCAAAAAATCGAACCCAAAATCAAGAAATCAAACCCTTATTCC
AATTTTTGAAATAAAACCCTAAAATTATTTCGTGAATGGAATCGATTTCG
TCAATGAAACGAAATCGATTTCGAGAACCCAAATGATGAAGGGGAGAGAG
AGAGACGACGATGCTTCGACGACGAATGAACTAACGATGGTTGTTTCGAC
GACGAACAGTGACGCTGCTTGGAGGAGACGAACGACGACGGTGACGGTGT
GAAGTGAAGACAACGACGACGGAGCATAGAGAGGAGGAGACGAAGACGGC
GATGGCGATGACGCCTGTGGCGAGAAGATGAAGACGGGATCGCACGATGG
GGACGACGACGATGAGAGTTGATAGAGGAGGAGGAGGATGGAGCGATGAA

GGCGATGATGGAGGAGGCCGACGGGGAGCTTCAGATGAGAATTGATAGAT
AGAATTGATTCTCTTATTTTAATTTCATGTAATGCCAAGTGTGTCCCTAT
TTTAGGATGAGTTGGGTTGGGCACAAAAAACAACTATTTTTTTCTCCTT
TCTTTAGCTTTTCAAGAATCAATTCTTATTTTTTAGTTGGGTTGGGTTGA
ATTTTGTATTTGCCAAACGCATTTCCAACCATGAATTCTAAAATTTTTGG
GTTGGGCTGGTTGGAAACACTAACCAAACGGGGCCTAACTAATCCACTAT
AACAAAAGAATAATTGAGTTACAAGACTCAACTCGCTTTTGGGTGGCTCA
GCAGAGCAATGGGAGCTTTATTGATAAATGTAAAGAATTCAAAATTTAGC
GGGAGGCGAGGACTGACCTCTGAAATAAGTCAAACAACAACTTATAAATA
ATTAAAATGCATCACAAAATAAACAAAAATCAAGAATTAAAACATAACAG
ATAGTTAAGAACTTTCTTTTATTTTCCGAATGTTAAGAAAGCGAAGCCTA
TCCAGTTTTAGGTTCCCACGAACAGTTGCCAGACATTCTGATTCAGACCA
GAAAATTTGATTTTCTCCACACTTCCTAGCTTGGCCAACAAGTCTACAAC
TATGTTACCTTCCCTGTAACAGTGCAAAATAGAAGCATGTACCTGAGAAA
CCAGAACCTTTATATTTCTAATCAATAACGTTAATCTCCATAGCACTGTA
GATCTCCCAGCAAGCATCTTGACCATCAGGGCATTGTTGGTGACTATATC
GACATAAACCCACTCGAACTCAACACAATAATGAAGTATAACCCACATTG
TTTTTGTCTCTACAAGCAAACTAGAACCAATACCAAGAAATTTAGAGAAA
CCTTAAAATAATGTCCCATTAGCATCTCGCCATGTACCACCTCCTCCAAT
ATAATGAGGGTTGCCTCTAGAACTTCCATCAAAATTAATCTTCAATCTAG
CACACAAGGGGCTCCAATGCACCAACTGAATATTAGAGATTTCTTTTTCC
TCACAGGAAACGCAATTTGGGCCATGGTTTGGTGAATGTTATATAGAAGT
TTTTGAATATCACACGAATGCCATGGAATCTAATCTTGTTATGCATTTTA
TAAATTTCCCAGCACAACACATAGAAGGAATGAGGATATCATAATATTTT
TATAGAGGAATAGATCTATGTTGCTGGCACCAATCAAGCAATCTTCCTAA
CACTTCTCCAGTCCAAGGAGTTACCACAAAAGTGTTCCACACACTCCTAG
CCAAAACCCCTTGTGAGAGCAGATGATGCGATGACTCACCCTAAGGCCTA
AAGCAATAATTGCATTGGGAGCATATTTTCACACTTTTCTGCTGAATCGC
GTCATCCACTGGAACAATTTCTTGAAGAAGCTTCCACATAAAAATAGATA
TTGTGCCAAATTTTTTCAAAGAAAGGATGAGCATCCTCTTGTTTCTTTAC
ATCTTCCCAAGCAGTTTTAGCCGAGAATATCCCCGAGTTAGAGGAATGCC
AAAGCATAACATCATGTTCAGACGCATTAAGAGCCTGAATACACTCCAGT
TCTGTCACATAATCTGCAGGAATAAGAGTTTGAATAAACTGCATATTCCA
TTATGAACTAGGTAACTAAACATCCTTAACTAGTAGATTAGTAAGACGCA
TATCTACCACCTCCACCTCACCCAGCGGAACAGAGAGAAGTCAGGTGTCC
CACGAACAGGGTACACACCCATTTCCTATCAGGAACTGAATATGGTCCTC
GGCAGAAAATTTTCCATCAAGTAGAACATCCCATACGTGGCTTTGGTGAG
AGAAATATCTGCATTTAGCTTATGGATGGTTTGATTGTACTTATCTTTCA
AAAATCAACCCCAAAGAATCCATTCTCTAAACCTCCACCAAAGCTTCATC
TAAAAAGCCTAGATAACATCCTGAAAGTCTTGTACCCCTAGGGCATTCTC
ATCTGTCGAGAAGTACATATTTCTTCATTGCTTCCAATGCAACTTTTTTT
CAGTCTGGATCCCCAAAAGAACCTACCCACACGAATAACAAATATCATAG
GAGAAATCGCCATGAACAAGTCGAACATCATACTCTAAAGAACATGTTTC
AAAAGAATGAGTCGTTCACCACTAGAAAGAAATTTTCCTTGCCATCCAGC

GACCTTTGGTCTAATCTTCATTAGTAGATCTTCAAAAATAGAATATTATT
TCCTACCTTCACATACCGAACAACCTAGGTAACTAAACGAAAAGCTCTCG
CTTGAGTCCAGTATGCCTTAGTATCTAGTGACAGGAGCTGGATTGTCTGG
TAAGAAAATTTGGCCTTTAGCCACATTCATCATCTAGCTTGAGCATGACT
CAAACTCTTTCAAGAATTTCATAAAATTATGAGTGGACTTTTTCCCTGGC
ATTAAGGAACACCATGACGTTGTTAGCATATGAAAGATGCGAAATTGGAT
TCTCATTCGCTGGCTGCGAATATATTTGAATATCTCATGAATCAATCAGT
TTTTAACCCCCCTTGAGAAAACTTCAGCAGCGAGGATGAATAGGCCGGGG
GATAATGGATGATGTGGTCTCTCCCTCTATATAGATTATCAGAAAAGGCA
CTAAAGTGCAGTTCCCAAGGGATCATTGGTGATAGTTATCATCATTGCTG
CAGCTGTCAGCAATCCTCCCCTCCCTCTTCCGGTAGTAGCTGAGCTCCAT
CATCTTTCTCTTTGTGATCAATGTTACTTGGAATGCAGTGCGCTTAGGGT
TCATGGAACAAATTCGAATTCGCGGTACAACAGATTGCAGTACCAAGGGG
GGCTTAGATGGTTACTTCTTTTGATTCACGATTTGCTTTGGTTCGCTTGA
CACATATAAATGAAAATATAAATAAAGAATACCAAGTATTTACATATAAT
AAACACATAAATTATAGAAAATTATAAAATGTAACCTCAAATGCAAGTTT
CACCAGGTTACTTCACTCCATATTGTCTCTTTCTTTTTGGTTTGCATCGC
TAGATGACCACGATCTGCTACACAGAAAGCAGTTCGCAGTTCCATTGGAC
CTGAGGGTTACCAGGAACTAGGCAACTTTTGTTGACATTTAACTTTGTTC
AGTTTAAATTGAACCACATTTGTTAGGGATGATGATTGGCTTCGAGAGAA
TAAGGACATCCAGGATGAGTTAGTGTGTCATTTTAGTAGCTACCTCGCAG
TTACAACTGGTGGATTGCAAATGATTTTCTTTTGCACATCCCTCAGATCT
TAACTGACGCCTATAACCAAAAGATGACTTCTTGCCTTGATTTGATGAAT
GATTATCAAGGTGATTTTGCTACGGATTGTTCCAGTGCACCAGGTCCAGA
TGGGTTCACTGGTTTGTTTCATATGACTTGCTGGGAGAGTGAAGATGGTT
GTATACTACTTCAATGGTGCAGAGTTACCTCCTTGTATAACTAGTACTCT
TTTGGCTCTAATCCTAAATGTGATCCTCCAAACTTCAGGCCTATAAGCCT
ATGAAACTTCTCCTGTAAGATTCTTACCAAGTTGATGGTTGATGGGCTAG
CCGATGTCCTCCCACAATTTATATGTCCTGAACAAGGTGCTTTTGTTCTT
GGCAAAACATCATGGATAACATTATGCTAGTTGAGAAAATGATGCATATG
ATGACTAAGAAGCTGAAGGGTGAGAATGTGATCATCAAGCGGGATATGGG
AGTGTCTTATAATCGGGTTTCTTGGTTGTTCTTGGCTGCTGTTACGAGGG
AATTTTTATTTTGTGAGCAATCGATTGATTCGTTGTATAGAACCTGTTTA
AGTAAAAGATTATTAGAAAAGAGTAAGACGGCTTGACCTCGGTAGTCGGC
CTCTGCCTCTATTTAAAATAAAATAGCAGCCGTACATGGAAGCCTTAATG
CAAAGATAAATACTAAGGTATTGACTAAAATTCTCTCAAGTTGGGGCATA
TATGTTGTATGCTTTCAACTTGTTACAAATGTATTTTATCCAAGAACCCC
AAGCAGTCTAGTAAATGAATCTGTTATCTAATTAAAAGAGTTAACATGTT
GAGTATGGATCAACTTCTGTAGCATTTTCTCTCAAATGTAGTGGCAATCA
ACCTCATGTGCTTTGTTCGCTCATGAAAAATTGGATTTGATTCTATAGTG
ATCCTAGGCATACTCTGACAGGGAAAATATTATTGATTGTGTATGTGGAC
GATATTGTGATCATGTGAGACGATCAAAGCGGTATTTGAGTACTATAGCT
ATTCCTGCGTGAGAAATTTCAGACAAAAGACTTTAGTCAATTAAAGTATT
TGTTGGGTATTGAATTCACTTGATCTAGCCACGACATTTACCTCTCTTGG

AGGAAATATGTTCTTGATTTATTGAGCGAAATAGGGATGTTAGGACCCAA
ACTAGTTGACTCACTGGTACTTATAACAAGCTAGACTGTAATTTAGATGT
TGTTGGTGTACTTCTGTTGCCAATTTTTTATTTCACGGACTTAGTGACAC
TAGGTCTAACATCTAGCTCCTCTATCTATAGGCGTCGGGGTTTACTTAA
TGCTCATACTTGTAAAAATTGTGTTGTGTCATCTATTCACGCCGGATCAC
TTTTTTCACGTATTCATCCACTTGTTTATATGTGAATTTTTTCTACGAAA
TTGAGGATTTCATATTTATTGCTATTTGGCTCTTTTATGATTGCGATTTT
TATTTTTTTTAATCATTGGACATTCAAGAGATGCATCAATAGTAAACGTA
TCTTTTTTTTTTTTTTTCAATTATTTTTTAACATTTTATGGTTTAATTTG
GTGCATACGCATTTGCCAAAATTTTGAAATTAAGAAATCCTTTATTTAAA
ATGGAGATATTTAATCACGTTAATATGGATTTGTGATGCTTGTTTTATGC
TTGCAGGGGAAGTTTGTATAGGTTAATCCATCGGCCCAACAATCAATTAG
ATGAACGCAGACGTTTGAAGATGGCTCTGGATGCTGTATGCTAGAGTTTT
CTTATCTTTGTTTTTAGTCTTAGTAGCAATCTCCTTCCTATTCAGTGCAT
TTACTAGTACTCTGACTTTCTTTTTCCTTTCAGGCTCGAGGAATGAATTA
TTTGCACAACTGTACGCCTGTAATTGTTCATCGTGACTTGAAGTCACCTA
ATCTTCTTGTTGATAAAAAATTGGGTTGTCAAGGTAGCATTATTATGTCAT
ACTTGAGTGTAAGATATATTATTTTGTACTTTCTTCACAAGTATCATCAT
GTGAATTTCCTCACTTGTTCAGGTTTGTGACTTTGGATTATCCAGAATGA
AGTTCAGCACATTTCTCTCGTCAAGGTCAACTGCAGGGACGGTAAGCTCT
ACTGACCAGTTTGTCACTTTACCTTTTCGTAATGCAGCAGGTGGTCAACT
ATATTTTTTCATTTTCTATTTAATTTGATCCATCTTTCATTACGTTTATT
TTACTCTTCTCACATTTATCTTATATCTTATTCCCTAGTCACGGATTTCA
TGTCTGCGGAAATTTTGCATTGTTTTGTTGGTATGCAACAGCCTGCTAAA
GTGCTTATTTTTAGTATGTTAATTCCAATGTAATGTCACGGATTATATGG
CTTAACTATGGTGATTAGCCTTGTATATATTTATAACGATTTGGATACAC
TAAAATAACATAAATACCCATGACACTTTCCCTCAAGGTGGAGTATATAT
ATCATCTGCACCCAACTTCTTACATATACTTTATTCGTGAACCACCTCAT
AGTTTGGTAAACAAATATTCAAGTTGGTCAGCCAAATCACATTGATCAAC
CAAATCAGCTGCGATATGTGAATCAAATTTTACTATAACTATTCTTGAAT
GAAATGAAAATAAACTTCAATATGCTTGTCTTCTCATGGAATACTATATG
TTGGAATTAATGTGAAGAACTACTTAATTATCACATACAGAGCTCATAAG
GCGAGATTTTTCAAAACCAAGCCTTATAGTATACGATTCATACAAATCAA
CTATGTAGTATGTCATAGTTCTATGCTCAGGTTCAATACTTGATCGAGCA
TCCTGTTTCTTGCTTTTTCGAGACACCAATTTACCACCAACAAATACACA
ATAACCTGCAGTGGAGTTTCTATTTGTTGGAGATTTGAAATACTTGACAT
CTATGTACCATTTATTTGATTGTGACCATGGTTATAACATGGATAACCTT
CAAGCATTCATGAATACTTAGGACACTAGGTCTTAAGGCATCCGGGACTG
ACTGACAATGTTCATTGTAGTCTAGTAACAGCTAGATAATTCATTTTCTC
ATCTAGGGACCTATATTGCCTTAGCATCTGTCATTGATCGGCCAAAAGAA
TTATGTTTGGATACCTAAGTGTGTGATTCATTTGGATCGTAGCATTTCCT
TCTGCTTAACTCAATAAGTACATATTTCTTTTGAGAGAGCTAAGGGATTG
TTTGTGATTAAGGCATCCAGTAATTGAATGGCAATGCTCATTGTACAGGA
GATAGCTTGTATGGTAACAACCGGGTAATTTTCTATGTTAGCATCTTTCA

TTTATATGCCAACAAATTTTTTAGGATACAGGATTGTGATTGGTTTGGAT
TATAGCATTGCTGGTCATGTCACTACATATTTCTTCTAAGAGATCTATGG
GGCTATTTGATTTATTACCCAAAGGGTTTTTGTTGAATCCCACATCGCTT
GTGTTACCTGATCCTGAGCTATATATAAAGCCAAGGGTAATCCTCTTCTT
ACAAGACGTCTTTTGAGTGAGTTGTAGGCCCATTAAAATTTCTACAATTT
TTAGATATTTTAAAAATATTTTCCGAAAATAATTTTGGAAAATTGTGAAA
ACTTGTTTGGTTTAGCCAAAAAAAAATTTTTTCGTCTTTTTTTTTTGTTTT
TTTTGAAAACATAATCTTTATTATTTTAAGAGTTAAAAAACAAAAGTAGA
ATTGATTTCATTGATTGGAAACCTTAAAGAATGGAAGGAGAGAGAAGTGA
TGTGATTGGTTGAAATTGTATGTATGAGAGAGAGAGAGAGAGAGAGAGAG
AGAAACATTGATATTAAAAAGTAGGTGATGCGATGGATTGCGAATCGTGA
AACTTGGTTTGTCAAATAGTTTATAATTTGTGTTGTAAAAGGGAATTATT
TGTCTCTTGTTTTTGAAAACAAAGAGTTAGTATCCAAATTTTTTAACCAA
ACACACACTTAATGTCTTGAATTGATCTAGCCACTTCGATACGAAAATTA
CTTAACCTGTCCCAACTCCCAATTATTTTATGTAAAACTTGCAATGCAAA
AAATAGTATTAGTGCTTATATATCTTTTAAAATCATCACCTATTCATAAT
ATCATCCACAACCCAACAATAAATATTTTTCTACTTGTAGGTTTATAGAA
AACTGAATGATCGAACCAAGACCTTGAAGACCGAACTCAATGACCACATC
ACTAAATGTTTTAAACCACACTTAAAGGTATAGTGTTAGGCGATCTGTTG
ACATCTTCAAATGGCTCACTAACTCTGATTATCCCTGAACAACAAAAATT
AGAAGTTGCTCCATATAGAACTCCCTTGTGTAACTGAAAATGTAAGAACA
CATTCTGTATTTCTCGGGTATTGAGTTGACTCGATCGAGTCAAGGCATTT
TCCTCTCCTAGAGGAAATATGTCAGTGATCTATTGAGCGAAACAGGGATA
CTAGGAGCTAAATTGTTTGACTCGCCTATGGATCTTGTTAGAAGTAAAAG
AAGAATAGGAAAAGAATGAGACGGCTTAACTATTGTGTTAGCCTATTCCT
CTATTTATAATAAGAATTAGAAAACCATACAGTTAACCCTAAACCGTACG
GGAAACCCTAAGTAAAACATAAAAGACATAAATACCCTTTAACACTCCCC
CTCAAGGTGGAGCATATATATTTTATGCTTCTAGTTTGTTACAAATATAT
TTTATCCTTAAACCCCCCAATGGTTTAGTAAATATATCAGCTAACTAATCA
ACTGAATTTACACATTTAGTCTTAATCTGTTTTTGCAACAATTTTTCTCA
AATGAAGTGACAATCAACTTCAATGTGTTTGGTTCTCTCATGGAAGACTG
AGTTGGATGCAATGTGAAATGCTACTTGATTATTACATATCAGGTCTATT
GGTGAAGTGTGTTCAAATCCAAGTTCTTTCAACAATTGTTTGAACTAAGC
TAACTCATAATAAGAATTAGAAAACTATACAGTAAACCCTAAATCGTACG
GTAAACCCTAAGTAAGACAGAAAAGACATAAGTACCCTTTAACAGATCTA
AACATAAAACTATTGGCAGATCATGGAGTACTACTGATAGATTCAACGCA
ATATGGACGGTTAGTTGGGAAGTTGATCCCTACTGTTACGAGATCATATA
TCTCATTTGCTGTTAGTGTCATCAGCTAGTTCATGAATCATCCCAAGACT
TGTCACTGGGTTACAGTGATTCGTATTCTCCAATATCTAAAATGTGCTTC
TAGTTGTAGTTTATTGTATCAGAATTTCGGTCACACTCAAATTCAGTGAT
ATATAGATGCTAATTGGGGCTGATCTCTCTCAAATAGGAAGTTCACTATA
GGGTATCATGTGTGGTTGTTGGAAAAGTTCTCTATTGTTTTTACTTCTCT
TGGTTTTTGCCAAGTAATCATGATTTGGTTATTTTTATAAATTGTACACT
TTTAGGCTGTGTTCTACTCTATTTATATGTTGATGACATGATTATAACTA

AGGATATTGCTGATGGGATTGTAGTATTGAAGTGAGAACTATTTTGTAAG
TTTGAAATGAAAGGTTTGTGGACTTTACGTTACTTTCTAAGTATTGAAGT
AGCATTCTCTCAAAATGGCTATTTTTCTCTCGGTCTAACTATGTTACTGA
CATTCTTGAGAGTGCTTGCCTCACTGATATTATGACAGTTGACACTCTTC
TGGAGGTAAATGTTCACTATTCACCTTTTGATGGTGCCCCTTTGCCTGAT
TCTACCTTATATCGTATTAGAAGTCGTTATATCTTATTATTACTCATCCA
GACATTGCCCATGTTGTTCACATCATCTGTCAGTTTTTCACTTCTCCTAT
TACGGTTTATTGGGCTATTGTTCTTTGTATCGATCGGTATTTTTAGGGCA
CTCTTTCAAGTCTTTTACTTCCATCGACTATGTCTTTGGAGTTACGTGCA
CGCTACAATTATTATCGGGCTAGTGATGTCATAGATCGCAAGTCTGTCAC
TGGTTTCTATGTATTTTTAGGCGATTCTCTTATTTCTTGGAAGTGCAGGA
AGGAGGTAGTTGTTTCCAAATCTTCCATGGAAGCTCAGTATAATGTGATG
GAATCTACTACTACAAAAATAGATTCGATATTTTGGTTATTGCAGATATT
GGCGTTTCTCTTTCTCATCCTACTCCTGTGTAATGTGGTAACAAGAGTGA
TATTCAGATTGTTTACAACTCAGTCTTTCATATACAGGCTAATTACATCG
AGATTGATTGTCATCTCACTGGTTGTCACCTTCAACATGACACTATTAAC
TTGTTTTTTGTTTCATCGTCCATGCTGATTACCAACTTGCTCATTCTATT
TTGCGCTTTCATTTTTTTGTCGGCAAACTCTCAAAGCTTCTTGTAGCCAC
ATCGTGAGTTTGAGAGAAATATTAGATTGATTATATTTATTTAGTATGAA
GTGTAAAATAGACTTTTTTTTTTTTTTTTGTTTTGTAGGTCTTTATATTG
TAAGCCGAAATAAGATTTTTTTTTTTTTTTTTAATGTATTTTCTTGTAC
TTTGCATTCATTATTCACAATAATAAAACCTAGCCTCCTTTTTCCTTTTC
TCATTGGATTCTATGATGGCGTTTGATTAGTGTTTCGACTTTTCCAATCC
CTCTCTAAAAATTGGGATGAGAATTATGTTTCCTTCTGGGAACAAAATCG
CGTTTGGTAATGAAAGAATTTTGTTTCCTTTTGGGAACAAAATCACGTTT
GGTAACGATAGAATTCTGTTTCCATTTGGGAACAGAATCGCGTTTGGCAA
TGATATTAAAATGGAATTGTTTCGTCAACAATTGTTTTTTTTATGATTT
TTGACGACTAAAATACAATTGTATAGCGAAATCCTTTTGTCAAAGGTGAC
AAATAGTATTTCGTAGCCAATAGTTTACCGTTTAATAGCAAAATCCTTTT
GGAGAAAAGTACATGTTCCCTCTATATACTCAAAATTATTACCTGATTTA
TTTTTGTGCATGTTTTCAAAACTAAAAACTAAAAGCATAAACAATCAATA
TATAATAAGCAAATAAAAAACATAGATAGTTTGTATCTCAAATTATACAT
ATGTATTAAAAACAATCAAATAACCCGACATGACATCGTCAAACTCTTAT
TTTAAGGTATCAATATACTTGATTACAACCAACTTTTAAACACTAATTTC
ACATAAACAAAATTCAATTAAATTCGTAGATTAACCCTACACAACATAAT
CAAAAGATAAACCCTCAAAGGAACAATGAATGCCAAATGATAAAAACGTC
ATCTATTCCATAATATGTTGAATATGCAAATATTTAAAAAAAAATTATAA
TAAATTGCCCTAAAAATAAAATAAACTTCCAAAATGGGAATTTTACGATT
TGGTCTTCAGGTCCTTTGGTATTGTTCAACATATCTCTGAATATGCTTAT
GAAAGGAGTTGTCCCAATTCCGAGCCCCACAAAGAGTAACATGTCGTATT
GACAGTAGTCTTGCGCTGGAGCACCGTAGCAATTTGGGTAATCTACTTCA
ATGAAGTTGCTCATATTAAATTTTATACCAGTATTGTCTTTTAAATATTA
TAGAATTATTGAAATTACCTTGTTTGATCAACATTTCCGAGTTGGCCGAA
CTTTGTAGGAAACAATCCTCTGTAAATGCTCGTTTAAGTTCTTGAGTTCA

ATCTCCTACCATTTTGATGTGAACACTTACATAGTTGTCACGCGCTGCTG
AAGTTATCCAAAATGGATGCCCGGAGAGAGATCAAGAATATGCATTCATA
TTTTCTAATGAAAATATGGTAGACTACAGATGACTAATGAATAGAGCACA
GACATACATGAAACAAAATAAACTGCATTGAAATATTTCACTAATCAAGA
AACAAATTCCTCCTATCAAGAAATCAAACCCTAATTCTGAGAAATTGAAC
CCTAAATCAAGAAATCAAACCCTAATGCTAAGAAATCGAACCCAAAATCA
AGAAATGGAACCCCAAATCAAGAAATCGGACCCTTATTCTGATTTCTGAA
ATGTAACCTTAAATTTATTTCGTGAACGGAATCGGTTTTGTCAATTAAAC
GAAATCGATTTTGTGAACCCAGATGATGAAGGCTAGGAGGGAGGGAGAGA
GAGAGAGAGAGAGAGATTACATAGATTGATTTCAGTGAAATCGATGTCCA
AAGCTGAAGAAGAAATCAATGCTCAATTCAACAAGTCCAAATTTGAACAA
CGAAATCGATGTTCAGCGACGGTAATGGTGAGAGAGAAGACTACACTTGC
TAGAGACGACAACGAGACTGGCGACAATGGAGGAAGAAAGCATTGATGGC
GAAGTGAAGAAGAAAAGGAACAAAAGTGAAAAAAAAAAAGGAATGAAGAA
TGATCGGGGTCGTTGGGAATTGAATGGGATCAATTTTCCTTCATTGATAA
TTTGGTAAATGACGAGTGTGTCCTATTTTGGGATGGGAAAGAACAAGAAT
GAAAAGAATCAATAATTTTTTTATTCTTACCTCCTTAAGGAATTCAAGAA
TCGATCCCAAAATTTTGGGATGGAATGGGAACGAGAACAGGTTTACTAAA
CACATTCCCAAGCATCAATCCCAAAAATATGGGAGTGGGATGGGATAGGA
ACACTAACCAAACGGGGCCTATGTTATTCATAAATCTTACCAACGGGTAG
CAGTGGTGGCATGAATTTTCTGAGCAGTCACCAACTGCCCAGAACACTTC
TCACATGGAATTAAAACTTCATGAAGTTAAAAGTGGATTGGCATCTCCCA
TTTTTAAAAACCACGACATCATCAACATATGCTTTAAGTGGCTAATAGGA
TGAGGAGAATGATGGTGGGTAAAAGGTTTAATTTTCTGTTGCTCCACCAG
GAAATAGATTGGGAGATGAAGGGTCTGCCTATCTAAGACCCCTCAGAGAC
TTGAAGAAGCCATGGGTGGGACTATTGACAATCACTGAAAAGAAACACAC
TCACACAACTCTGTAGACTAGGTCAATCCAAATCTCAAAAAAAAAGCTAAA
CTTCCTGAAAACACTAGTGAGGAAGCACCAAGAAACCATATCACAAGCCT
TGGCCATATCTAATTTGAAAATCGCATTACTCCCTTTATACTTATTCATC
CACTGAACCATCTCCAGAAAAATATTGTTCACAATATATTTCCTTTTAC
AAAAGCACTTTGTTCAGAGGAGATAACTTCAAGAGTAAGCTTGTTGAGTT
TACAAGCCAGCTTAGTCACTACTTTTTTGTGAAGTTACATAGACTAATAG
GTCTAAAATCTACAAATCCTTGAGCATTGTCACATTTAGGAATCAGTGCA
ATCAAGGTATTAGTGTTGCTGGCAGGCAAGGAAGCTCCATTGATGAAATG
TCTAGCCACCTGTGTCAACTAATCTTTAACAATATCCCAGCAATCAGTGA
AGAACTTACCAGTGTACCCATCAGGGCCTGAGGCACTATCCCCATTCATA
GCAAACACAATTTCCTTAACCTCGTCCTGAGAAGGAATATTGCAAAGCAT
CACATTGTCATCCTAAGAAACCAATTGAGGGATCACATCCATAAGCTCCT
AATAATTAGGAACCTCACTCGAAGCAAGATAACTCTGGAAATGTTGCCCC
CAAGGTCTGTTGAATTTCTCTCTTTCTCCAACCAATCATCTTCACCTTCA
GTATTAAGTAGTCTCATGATGAACGTTCTCCTTATCCTACTTTCAAGTTT
TGAAAAAAGCATGAAAGAACTTAGTATGTTGGTCTCTTTCTTTAAGCCAC
TTGACATCTGATTTTTTCTTCCAAATGCTCTCTTCATACCTGAGAGCAAC
TTTCAACTTGGTCTCTTCCTTATTTAATTGACCAGCTACCTCCAAGGAAG

GATCATTGATATTCAGAATCTCTAAGGCTTGAATTTGCCCCTCGTCTAAG
TTAACTTTATCAAAATTGTTCCCAAAAGTTTGAAACCTTCGTCTGGTCAA
AGAAGTTTTCACCCTTAACATTGCTCACCACACACGAGCTGGGGATTATG
ATCAGAGCAGGTTGTACTCAGGTGCTGAACATTGCCACCCAATAGATCTA
GTCACCGGTTGTTAACCGAAGACCTATCAATCCTACCCTAGTTGCATCCG
ACACCCTGAGTTTTCTTGCGCCAAGTAAACTTAGCACCTGTGAATGGGAG
TTCCAGAATTCCACAACCTTCCATGCAAGTCAACAAATCTTCAGCGGAGC
CGGAATCAGCAGATCTATACTCCAGTCTCTCCTCTGCTGAGATAGTCACA
TTAAAATCACCATAGACTAACCAAGGCAATTGATGTTGGTTTTAGAGCAG
GAGCAACTGGTCCCAAAGAGATATTCTTTCTGTTTTAGAGCATTTAGCCT
AGACAACCCCCACCAAGCAAGGCTCGAGATCAGCAATGCATATCCTCATT
ATTAGAGTTTAATGAGTTATTATTATTATTATTATTATTATTATTATTAT
TATTATTATTAACGGTAGAATAACCTTTTATTTGCTTTCTAGATCTTTCT
ATTGTATGCTTATATAAGTTCTGGATTTCTAGGTTTTCTACTGTATTCTT
GTATTCACTCATTCAGAGATAGTAAACCCTAGCCACCATTTCGCTTTTCT
CTCTAAACTTTATATTTATTCATTGATCTTACACTCATAACAATAAGCTG
GTTTCTGCTATGAATGACTTCAGCTTTAACAAGATTCCTCCATAACATCT
AGATTTTACTCCCATTTGAAAAAGCATGATCAAAACCACCTTCTCCTTAA
AAGGCAACTTGGCATCCCTATTCACCATTGGTTCCATCAATCCCATCATG
GAAATACGAGATAAATCAATGTATCCCTTAGGCGGTGTCAGATCAGGAGA
ATCAAGATTTGATAAAGTGTCTTGATATAGAAGAGCTGCAGTAGGTCGTC
AAGGATATGAATGGAGAGAGTGCCCCAGGCCTAGATGGATTCACTAAAAG
GTTTTATGTAACATGCTGGGAGATTATCAAGGAGGATCTAATTAATATGG
TGACTGGCTTTTTTTGGTGTAGAGTTACATTCTAGTATAACTAGCGCCTT
CATTGTTTTCATTCCCAAGAAGAATAACTCTGTGGTTTCCACCGACTTCA
AGCCGATTAGTTTATGCAATTTCTCAAATCAGGTGGTAATCAAGATCCTG
GCTAATAGAATTGCTTCGTTGTTGCCTAAAATCATTTTTCCTGAACAAAG
TTCTTTTGTGCAAAGTAGAAATATTTTGGACAACATTCTGTTACCGCAGG
AGATAGTGCAAAAATTTAAGTACAAGGTGCAGGGGGAATTGTTATTGTAA
AACTAGACATGGCTAAGTCGTATGACCGAGTCTCTTGGTGCTTCCTAACT
GTTGTGTTAAGGAAATTTGGCTTTGCGAGGTTTGGATCAATATGGTCTAT
AGGGCGATCTCTAATAACCATTTCTCTGTGCTAGTAAATGGGGGTGCGGG
TGGTTTTTTTAGATCTCAGAGAGGGTTTAGACCAAGTGATCCACTATCCC
CCAGCCTATTCATCATTGTTGCTGAAGTTTTCTGAAGGGGAATTAAAAAA
ATTGATTGATACATGAGATATTCAAACATACTCGCAGCAGGCAAATGAGA
ATCTAATTTCGCATCTTTCATATGCTGACCACGTGGTGGTTTTCCGATAA
TTTTATGTTCTTGAAAGATTTTGAATCATGTTTAGGCTAGAGGTTGAATG
TAGACAAAAGCCAAATTTTGTGTGAGAAGGGGAAATTCTTTTCATTGTTC
TTGATATGTACAACAGTGGGTTAATATAGACAACCCTAGCTACAAAAAAA
GGAAAATAAACAAACTCCCCTAATCAAGGGATTACAATGAATCTCCCTAA
TCTTTACAACTGTTAATTGACTTTTCAAAACAATTAATTTCTTTATTTCT
CAACACTCCCCTTAAGGTGGTGAATAGATGTTTTCCATTTCTAGCTTGGA
TATAATCTCTTGAAACACGGGGCCACTTAATCCTTTGGTGAGCACACTTG
CTAGCTGACAGTTAGTAAAGACATATGGTGTGTAAATCAAGCCACTTTCC

AATTTCCCCTTAATGAAGTGTCTGTTGACCTCGATGTGCTTTGTTCGATC

ATGTTGTACTGGGTTATGCGCGATACTGATTGTTGATTTGTTGTCGTAGT

AAAGTCTTATCGGTCCATCCCACTCAATCTTCAGATCTTCCAAAATAATC

TTCAACCATAGTAATTCACAAACTCCTTGAGCCATTGTTTAGAATTCTGC

TTCTGCACTAGATCTTGCTACCACATTTTGTTTCTTACTTCTCCAAGTTA

CAAGATTTTCTCCAAGAAAGGTGCAATAGCCTGAGGTCGATCTTCTGTCA

ATTATTGATCCAGCATAATCAGCGTCTGTATAAGTTTCAAGTATCATTCC

CTCACCCCTTTTAAACAAAATTCCCTTCCCAAGACTTGTTTTTAGGTACT

GCAGCACTTTATCGACCGCTTGCAAATGAATCTCCTTTGGATTGTGCATA

AACTGACTGATCACACTAACAGCGTAGGCTATATCAGGCCTAGTATGAGA

GAGATGAATGAGTCTTCCTACAAGATGTTGATACATCTCTCTATTCATTG

CAATATCTTCTTCAACCTCTCCAAGTTTATGGTTAGGCTCTATTGGTGTA

CTCATCGGTTTGCACACCAATTTCCCCGTCTCTTTCGGCAAGTCAGTAAT

GTACTTCTGTTGAGAGATGAAGATCCCTTGTTTGGAATGTGCAATCTCAA

TTCCAAGGAAGTACTTCAACTTCCCTAGTTCTTTGATCTCAAATTCACTT

GCTAACCGTAGTCTAAGGGCTTATCTTTCCTTCTCATCATTTCCTGTCAC

AATGATGTCGTCAACATATTCTAGGAGTGCAGTAACTCTCCCTGAACTTG

AGTGTTTGACAAACAAGGTATGATCTCCCTGGCTTTGTTTATATCCCAAT

GACACCATCACACTTCCAAACCTTCCAAACCATGTCCTTAGAGATTGCTT

AAGCCCATACAAAGCTTTATTCAATCTACAAACCCAGTTCTTCCCCAAAC

TCCTACCATATCCAGGTGGGATTTCTATGTATATTTCTTCTTCAAGATCT

CCATATAAGAATGCATTTTTTACATCAAACTGTTGCAAATCCCAACCATA

ATTGTCTACCAAAGACAACAAGACTCTGAATGTATTCATTTTAGCAACTG

GAGCAAATGTTTCTTGATAATCCACACTGTATGTCTGAGTATACCCTTTG

GCCACTAATCTCGCTTTATACCTCTCCAAAGTACCATCTGTTTTATATTT

CACTGTGTAGACCTACTTGCACCCCACAAGTTTCTTTCCTGCAGGTAAAT

CTACTATCTCCCAAATTTTATTCTTTTCCAAAGCTTCCATTTCAACATTC

ATATCTTGTTTCCATTTTTCATTAGATAATGCTTCAGACAAGGTGGTAGG

AATGGTAATACTGTTTAAGCTTTATCAGGAAGCTCCTATGAGATGGTAAA

AATCTTCTAAATGTGACAACATGAGAGAGTAGGTAACGGTGATGTTTGGT

GCATTCTCTAGTTCCCTTCCTAAGGGCAATGGGCAAATCTTGATCCGGGT

CACTACTCAAGTGAGGTTCCGGCTCATGGAATGAAGGAGAATCTTGATCC

AGGTCACTACTCAAGTGAGGTTCAAGCTCATGGAGATCAGATTCATTTAA

TGAGGGAGAATCAAAAGAAGGATCGGGGTTAGAAACTGTTACCTCATTTC

TAGAATTCAGTTCTGACTCTTGGGCCTGCATAAGTTCGGAGACCGGAGGT

GCTATCCGCCTTGATTATACTTGACCAAATAATCTCTTGTTATCTCGGGT

AGGAGTAGACTCAGGATTCGTAGCAGGGCTTGACAAGGAGGGAAGAACAA

TGACATCCTCCTTATCTTCTATTACTAAATTCTCCCCTGAAGATAAGAGG

TGGTGAAGTATGACTGACTCTCATGAAAGGTAACATCTGCTGAGACAAAA

AATTTTTTTGAAGGTGGATGATAACATTTATAACCCTTTTGGGTAGAGGA

GTACCCTACAAAGACACTTGATTGCTCTGAGATCCAACTTTCCCCTATTT

TGACTGTGAACATGAACGAAGGACATACAACCGAATATTCTTGGTGTCAA

GTGGTTGGTAGTTTAGAGATTGGGATATGACTCTGAGAGTACTTGCATAG

GACTTTTGTAACTTAGAACTCTAGAAGGTAGACGGTTTATGAGACGTGTT

GCTGTTAAAACAACCTCCCCCAATAAGACTTAGGCACATTATTTTGGAAC
AAAAATGCTCGGGTTGTTTCAAATAAATGACAATTTTTTCTCTCAGCAAC
TCCATTTTTTGTAGAGTGCTAACACAAGATGATATATGAGTTATCCCTTG
CTGAAGGTATGGGGTTAAAAGCTGATTGAAATAATCTCTAGCATTGTCAG
ACCTAAACCTTTTGATTTTTACTCCAAATTGATTTTGAATCGTGGCATGA
AAATTTGGAAGAACAAAACTCACATCAGACTTATTCTTGAGCAGAAAGAG
TCAACACACTCTACTATAATTATCAATGAATGAGACGAACCATCGTGCTC
CAAAAATATTAGGAATAATAGAGAGACCCCAAATGTCACTATGGATTAAA
TAAAAGGGAACTGGACTTCTTTTATTACTAATTAGAAAAGAGACACGCTT
GTGCTTTGCAAACTCACAGACATCACATCGAAAAGAGTCAATATCTAATC
CTTAAACAGACTAGGAAATAGTATTTGAGAGTCTTAAATGATGGATGTCC
AAGTCTACGATGATAGAGCCAAATTTTTTCTTTATTTGATGAATAACACT
CAGAAAGAAATGACACAATTGACTAGCCCTTAGAGAAACTGAGATTACTT
GGTGTTTCAAGGTAGTATAGTCCGTCTCCTTCCATAGCACGTCCAATCAT
CTTTCCCAAAGTCTGGTCCTAAAATGCACAGTAGGTAGGATAAAAAATCA
CACTGTAAGACATTTCTTGAGTTAGTTTTTTTATAGAGACCAGATTCGTG
GACAGTTTTGATACATGAAGTACATTTTTCAATGTGAGTGATGGGCTAAT
CTTAATATCTCCTATTCCTGCTACAATGATTAATGACCTATCGGCAATGG
CAATCTTCCTTGTACTAGGACAAGGGGTATAGGTGCTGAATTTATGTGAT
GAATGTGTCATGTGATCAGTTGCTCCTGAGTCTACAATCCATGAGTTAAC
AAAGGTTCTGTCCGAAGCATTAAATCCAATGGAAATAGGAAAGTTACTTG
AATGTACCAAAGAGCAAGTACCGAGGGCTTCTCAAGACCATAAGCGATCC
CTCTAAGAAGGTTCTCCTTTAGTCCCATAGCATCAAAGCTATCACAAATC
TCATCATATGATGTGAAGAAATCTTGCCCATCAGACGAGAGCAATTCACT
CATTTTGGCATCACACTGCTTAGCATCAAACTGAGATCCTTCAGGAGTTG
AATTTTCCATGACTGAGTGTCGATTTGCAGGTCAACGAACAACGAAAAAT
AAGATTAAAAGAAAATTGGAAATCCCGAAATTTGCTTGAAGTAGAGCTTT
AATCGAGCTTTAGTCGTTCAAGAAAAAAACCACCACCGATGGTTGCAACTG
GAAGAAAAGATTGGTTCGATTTGGGTTCGCGAGGTTGCGATGAAGGTTAG
GGTTTCGTGATCTGGGTTAGTGAGGTCGCGAGATTGCTAGATGGTTCGAG
CTGGTCACAGCTGATCGCGAAGGTTGCAGCAGATCGCAGTTGGTTACGAA
GGGTTAGGGTTTTACAATCTAGGTCAGGGTGTGCGATTCTGGTTTGCGAT
TTTGCAGCGACAATGGATTAGGGTTTCACGGTTGGATTTTTGCGGTTGTG
GATTAGTGGCGGTTCAAATCCGGCAATAGTGGCGGTGCCAAAGCTCAAAG
ATTTTTCTGGCAATGAAGGCCAATCTTGTACATGACGATTAAGGTTGAAA
AAGAAAGAGAAGAAAAGGGCAATTGAGGCCAGAAAAAAACCAAGGATTGAG
CCTTACAACTCTGATACCATGTGAGAAGAGGAAATTCTTCTCATTGTTCT
TGATATGCACAACAGTGGGTTAAACACTCCCCTAATCGAGGGATTACAAT
GAATCTCCCTAATCCTAGAAATTTACAACTGTTAGTTGACTTTCCAAAAC
AACTAATTTCCTTATTTCTCAACATTTTGTTACTAGACAATCTAGTTCCT
GGATACTCAGGCATAAAAGTTAAAGTGAGGTAGTTTTCTTTTTAGTTATC
GAGGTTTTTATGATGGAAAGAAAAAATACGCTATTTTTGAAGACTTGCTT
AGGAAGATTGAGGGCAAGGAAAATTACTCTCTAGTAGTGGGCATCTCATT
CTTTTGAAACATGTGCTTCAGAGTATGCCTTCTATTTGTTAATGGAGATT

TCTCCTTTGATATCCGTTATTCGAGAAGTTGAAAAGATTTTGGTCAGGTT
CTTCTAGGGATCCCGTCCTGAAAAGAAAAGGTTACATTGGAAGCAATGGA
GAAATATTTGTTTCCCTACAAATGAGGGTGGTCTAGGGATTAGAGACTTT
CAGGATGTTATTCGGGCATTTCAAATGAAACTTTGGTGGAGGTTTAGAGA
AGTGACAAGTCTTTGGGCTCAATTTTTGAGAGAGAAGTACAACCAAACCG
GTCCAGCAGCTAAATGCAGAAATTTCTCTTACCAAAGTTATGTATTTGGT
CTTGAAGGAAAATTTTATGCCGAGTAACATATTCAGTTCCTAATAGGAAG
TGAGTGTGTATCATTCTGGTGGGAAACCTGACTTATCTTTGTTCCGATGG
GTGAAGTGCTGGTGGTAATTACGCGTCTTACAAATATACTAGTTAAGGAT
GTTCAGTTAGTTAGTGGAGAATGAAATATGAAGCTTAGTTAAACTCTTAT
CCCAGAAGATTATGTGAGAGAACTTAAGCGTATTCAAATTCTTAATGTGT
TTGACTAGGATGTTATGATTTGGCAGCCCTCTAACTCAGGGGTCTTCTCG
GTGAAAACTGCCTTGGAAGTCGTAAGGAAACATTGGATGCTCATTCGTTT
TTTGAAAGGATCTAGCAGAAGTCCCATTCAATAAATATATGTATCTTTAT
GTGGATGCTTCTACATCAAATTGTCTTGGTGGATGAGGCTATTCATCAGA
AAGGTGTGAAAATATGCTCTTGATATAATTATTGCTTTAGGCCTCAGGTT
GAATCATTGCATCATCAGTTCTCGCAAGGGGTTTTGGCAAATAGCGTGTG
GACCGCTTTTTTGGGGCAACTCCTTGGACTGGAGAAGTGTTTGGAAGATT
GGTTGGTTGGTGGCAGTAACATAGATCAACACCTCTGCAAAAAATATACTG
ATACACTCATTCCTTCTGTGCTTTGCTTGGAAATCTGGAAAATGCATAAC
AAGGTTAGATTTGATGGGTTTCAATGTGATATTTAGAAAGTGCTATTTAA
CATTCACCAAACTGAGGCCAAGATTCCCTTGCTTGTTAAGAAAACAGAAT
TTAAGATTTCAGTTGGTGTATTGGAAGTTTGGAACCCCCCGTGCAATAGA
CTAGAGATTAATTTGGATGGATGGTCTAGAGGAAACCTTGGTTTTGTGGT
ACGCTGCGGGATGCTAATGTGACACTGTTGTTTGGTTTCGTTCAATTTAT
TGGGTTTTGGTCTAGCTTGCTTAAGGAGACAAAATCACTTTGGATCGCAC
TGCAAAACTATGTTGAATTTGAGTTTGTCATTGGATAATAGTCACTGACA
ATGCCCTGGTAGTCGAGATGGTTGGTGGGAGATCTACAGTTCCTTGGAGA
ATAACATCACTGATTCGAAATATAGAGGTTCTGACTTCTAAGGTTCATAC
TTCTATTTCGCACTGCTACAAGGAAGCTAACACGGTTGCTGACATGGTAG
CTAAGCTCGGAAGTGCTAGAGAAAATCGGACCTTCTGGTCTGAATCAAAA
TGTTCGGCAACTGTTCGTGGATGCTTAAGGCTAGATAAGCTTCGGTGGCG
TTTGGTAACCGTTTCTGTTTCCATTCCAGTAGTGGGATGGGAATGGGATG
AGATGAGAATTTTGTTTTCTTCTTGGGAGTAGAATCGCGTTTGGTAACGA
TAATTTTTTGTTTTGTTTTGGGAATAGAATTGCGTTTGGTAACGATTTTA
TCCTAAAATCAATTTTATTTTTTTTGTGAGTTTTTTTTTTTTTTGTTTTG
GCGTTGGCGTTGGCGACGGTGGCCGGCCAGCGACTGTCGCCAGAGGTTCC
TGGAGGTGGCCAGCGACGGTGGCCGGCGGTTGACGGTGGCCGGCAGCAGC
GGATGGTGGTCGCTGGTGTATAAACAGTGCCCAGTGGTTTTGGGAATGAA
ATGGGAATTATGAGAAATAACTTTTTTTTGTTTCTCATTTCTTAAAAAAA
AAAATGGGCATAATTCTCAAATGGGAATGGAATGGAAACGAAAACAAAAA
TTACCAAACGCTATTCCCAAACAGAATTCTCAAAAATCTGGGAATGGAAT
GGGAAATTCTGGGAAGGAATGGGTTACCAAATGGGGCATTTGTTTCCCTA
ACATTTGGAAAATCAAAGGAATTTCTTAATTCATGTTTTAATGCTTCAAT

TTTATGTATTATTGTTATTTTATTTGTTTGATTCGTTTCAGAGGTCAGGC
CTTGCCCCTTTTTGAATTTTGTATTCTTTGCATTTATCAATAATGATCTC
CTAAAAAACAATATTCATCATTGAAATAAAATGACTTAGCCTTCATTTTC
CTTCATTCTCCTGACTGTGGCAGCATTAGCTGCCCCCTTCGAAGTCCAAA
AGAGGAACGTATACATTAATAAGAGCTGGATTTTTCGGTCTGACCAGCAA
TGTCAGACCTTGTTTTAATTGATGATTTAAACTGGACCTTTTGCTTCTTA
CTCTTCCGAGTGAGAAACAACTGAGGCTCAACCTGGTCCTCACCCTCAAG
ATTAGCAAGCTTGAATCAATTGCTCTAGGGCATAAGCTTAAATTGTCAAT
CATATTGTTTCTAATCTCGGGAACACTTGCTTAGCTTAAATTGTCAATCA
TATCATTTCTGTGAGTCTTGGAAATACTTGCCTCGAATTCCTGGCACCAT
CCACAGTCAATGATGCAACCTCAGCCACCTAAGCCACCTCATTTTCTTCT
GTGCCTTGGACTTGTTGAAGCTAGCATTAGAAGATTCTGCTCCGTTTCCA
CGAGTCTCGTGAACGTGTTCCATCTCATGAGAGTTGCTTGAACATTCTAC
GCCATTATGAGTCTCGCGGACTTGTTCCAGTACCTTCTTCCCATGCACCT
CTTCCTGCTCACTTTCTCGAACCTTATTCTTCCTGATGTAAGTTATTATC
TCCGTAACCCTCTATTCCATAACTTTTTCCTTCCCCTTGGAATTTGCAGC
AACTTTCTCAGCTTCAGGCACAACTGCAACAACCTTGTCCCTAGAGTTAT
TAACAACAGCTGTGTGTCTCGCATCTAATTTCTTCTCTCTCGCCATTGTT
TTCGTAGCCAACTATGGATGCTTAATCATGCAAGTATCTCTGCACGACCC
AGCTTGAAACAGTGTTTGTAATACAAAGGAAGATGCTCATACTTGCCAAA
AACTAGATTGGCCACAACCCAACCATATCCACAGACACCCTGGCAGAATG
GGGTCTGGATGAAGCAGAAGTTGCTGCATCCATCTTCATAGGGTTTCCAA
CATCAGAAGGAACTCAAACTCCAACCATATTGGAACCACAGCGGATTTCT
GACTCTAAAATTTGGTGACCACTTGAAAAAACGCGTTGGATGTTTAACAT
ACCACATTTCATGTAACCACAATCACTGAACGTCTTCCTCCCAGTGCCCT
TAAATCAAAACGTGTTTAGATTCCAAAAACAAGTTCAGAAGAGACTTTCA
ATCCAAACGAAAAATTCTCCTTATATCCTCAAACAATGGTTTCCCAAGGA
GAACTTACCTACCAAGTCAAACTCGAAAGGCACAACAAGTGATTGAATCT
CATCATCCAAAACGAATAAAGCTGGGTCTCCTTTGTGGGCTGGAACAGGT
TTAACTCAAATTTCGGGACTCAGGAGTATTAAGGAGACTAGAAAATAACT
TGGAGGCAAAGCTGATCATGGGCATGGAAGGAGAAGATAAGGAACCCCCA
GGCAGGGAGAGGGAAGCAGGATAACCCCTGAGGCCGCCATTATCGGTGGC
GGTGCAACTCAAAATCCAAGGATAGATCTTGGGTTCCTTTTCACGCCACT
TAACAAGTTAAAAAAAATCATTATATCCCCTTGCTCAAATACTTCCTTCG
ATATGTATGTGGACTTTGTGCAGAAAAAAATGCAAATCTGAATAGCAGGT
TTCCTCGTGATGTGCCTAGATATGTAATGCTAACTAAGGTGTTAAACAGT
TTGATTGAAGGGTTAACAATCTTATGATATCGTGTATGTTTCCTTTTATG
TTTTTTGAGAGGATCAGAACTTTGGAGTAATTTCTCAGGATTTTGCAGTT
TGATGTGTTATTTGTCATGGTAGTAAATGGAGGAACTTTGATTTGGTAGG
ACCTAGAGCAGATTAGCTAGTGATGTTCTCTCTCTGTCTCTCTCTATCTC
TCCCTAATATCTTATTTGAAACCTATTTCACATTATAGTTAAGACAGATC
CGGGCTTGGGTGACTTTTGTATAATTTTTATAACTGTATAAACATTTCTT
GTTTTTGGTGCTTCTATATACAAAATTTATATCAACAACTTTGACAATGG
CTAGGTGTTAAACCCTTTTTCAGGCAGAGTGGATGGCTCCGGAAGTGCTA

AGAAATGAACCATCAAATGAAAAGTAAGCACCTACTTCCGACAGAAGATA
AAACGAAAAACAAATTTGACATACAAACTCTTAGCTGTAAATATGTGGTC
TTGGTTGATAATTTTGGTTGACTGTTCTTCACTTTTCCCTTTTGGTGGAT
TGTTCATTGACTTTCCGCTACTGAAGTGGGGCAAAGGCTTTGTTGTTGTT
GTTCATTGACTTTCGGCTTGGATGTGATCTTAGGTGTGATGTTTATAGCT
TCGGTGTCATACTATGGGAGCTATGTACTTTGCGACAACCATGGGGGGGA
ATGAACCCAATGCAAGTGGTTGGTGCCGTTGGATTTCAGTATCGCCGTCT
TGACATTCCTGACGATATGGATCCTGCTATTGCAGATATCATTAGGAAAT
GTTGGCAAACGTGAGTAGATTTTGAATTTGCAGCTTTCCAGTCACTTTTT
TTCTTTTTCTTTCATGTCAATATTTCGTTTTATTCAGATAACAGTTAAGA
TGTTTGAAGTATTTAACGTCTCTGTTTGTCTTATATGACTAGTTTTATCT
CGGCTAGGATTACCATATAATATTGGTGCTTGTTTTCATGTATCCAGTTA
CAAATTTGCTTGCTTCAGATTCTTTCTGGTTATAGACGCTTGTAGGTTTG
TTATTTCAACTGTATGCCTTGTGTGTGTTCTTAAATAGAATTTTTATTTC
CAGATTAGGAATTGTTCAAAGAGGAATTGAACTGCTCAATGGAACATTTC
CTTTTGGCTGGGTTGATTTCCATAAGATTTGGCTGATCTAAAGCCCTCAT
GTTAAGAAATGCCCTCCCCTTTCGTGTAAACCTCTTGCTTCCCGTCACTG
CCACCCTTGGCCTCCAGCCAGGAATTAAATAACCAATATAGCTATTCTAT
TTTAAACTATGCTAATTTTCAGGGATATACCAATCTTATAAACAAAAAAT
TAAAGCTAGAAAATATCCTAGGATTTGGGTAATATCTCAACCAATTTATT
CAAACTTTCCGACATATAGTATTCTGTAACACTTGAAGACCCTTGCTTCG
TATTGTGGACTTGCCGTTTCATCCGAAAAATCTGAGAAGTAAAGCATATC
TTGGAGTATGTTTTATGAACAGCCAACCAAATGTCCCTAGTAGTAGGATA
CAACAGATATGTCCTACTAATCTCTTGTTGCATAGTGTGTGGTCACCACG
ACATGACAGTGGCATTAGCAACCTCCCATGTCCCATAGGATTTATCATTG
TCTGATGGTGCCGTTGCAACTCCAGTCACATAGTTATACTTTCCATGACT
CGTCTGAAAGATTTTGACAATTGTGATGCCTCCATAAAGTTCCTTCCATT
TAGTTTAACTGTAGAAATTTGAAGGGTGGGGTTCTAAGGTTGAATAGGAA
TTTGCTGGGGAATTGAAGTGTCAGACATGATGGTTGGTCAAGTGAAAAAA
TACAGTTTTTTTTTTTTTTTTTTTCTGGANGGAGTTGTCTTGGTTGACTC
GGCTGGGCTGATTCGAATGTGTTGACTCGATAGGGCTTATTCAAATGGGT
TGAATTGTCTGGTTCGGGATGAGTTGCCTCAGTTGTCTTGGTTGAGTGGA
CTCAGTTAACTCGGATCAGAGGAGCCTCAGTCGAGGTGTACGATGGTTTC
CGGCGACGGAATCAGATGTTTCTCGGTGGGATCTGTCGATCAAAACTGTT
AACAGCGGAACTGCTGGGTGTTTTGTCACGAAATTCGTTTAAGCATGGTG
AAATGGAACCTTCGACAGTGGGTCTTTTTTCGCCGTCGTGTGAGAATCTC
CATGGGCGACCTGTCCGTCACAACGATTGAGTTTTGTTTCACGGCAGTGC
CCACTGCTAGGCCTAGGAGTTCTGGAAATTAAGGCTGGGGAAACAAATCT
AGCACACAAGGTCAGTAAAAGAGAAAAAAAAAAAATTCATCAACAAAGAGC
TGGCATTGAAGGCCAGCAAAAAACATTAAAAAGTAGATCCTGGGCTCTAA
TTCCATAATGAAAATAAGAAGAGAATTTTTAAATCACAACTACAATGACT
ACATACATACAACCTAGGAGGATGAATAAAAGAAAAATTACATAAATTAT
AGAGCTAATTTAGCCTCTTTACATATGTGAGATTTTGGCAATCTAATTTG
GAAATGAATGTGTACATATTTAGAAAATAAATCTGGAAATATTTCAACAA

AATGCATTCTTCACCCTAAGCTGACGCGTTGTTCACCCATAGGATATGGC
AAGACTAAGCACAGTACGAACAATAGTGAGTTTGATTATAGGACTGAATG
TCTCATCAAATGTAACACTCGTCTGTTGATTAAAGTCCTTGGCAACTGGA
CGAGTTTTGTATTACTCAATGGAACCATCTGCTTTGCGCTTGAGTTTGAA
TACCTACTTACAATCCACAACATATAGATCTGGAGAAGGAGAAACAATAA
CCTAAGTGGTATTGTTCAACGAGGCATTAAACTAATTGTTCATGGCCGGA
CAACATTCAAGGACATGAACATGTTTGCTATAGCAAGTCGGCTCTTCAGT
GGGAGATGGAAGTTGAGCTTGGACAATCTATGGGAGAGGATATCAGACTA
TACCATGACTTCGGACAAGAGGGAAACATGTACCATCCTGATGAAGTGTA
ATCGCCGAATATTGCGGAGGAACTGGAGAAGGTGTCTTAGGGTTGGCAAT
AGCAGAAGGTTGAGTCTCTCAAGTTCAAGTATGTCGGCGTTGATAATGAT
AGGGAAATGGTTGTTGATGAAGAACTAGAGAACTCGAAGGAGCCGAAGAG
TTATTTTGAGGGGTAGACTCTAGAGGCCTCAACAAGTGTTGGAGGTTGTA
CATGAACAAATGTCATGATAATGGGACTGGAGTGCTGCTGGGGTTAGAGA
TAAATGGGAGGTGGTGTTCATTGAAACAAACTGGAAAGGAAATGTTATTT
ATGTTGGTTATGCAATCCAGAAGACGGTAGCCCTTATGCTACGAGGTCTA
GTCTAAAAACCACACATTCAACATTTATTGTCAAAAGATAACTAGTGAAG
TAAGCACTTAAATGCGTGTGACGTGCACAATCGAACACATGCAGTTCCGT
GTATGAAGTCATATGACCATAACTACAAGGAAATGGCAAGGACCACTTAA
GTTTAGAGGATGGGATACGATTGATTAGGTGAACATTAGTGGGGACCCCT
TCGGGCCAGGTGACGAGGAATATGACTTACGAGAAGGAGGGGGCATGTCC
ACATTGTGGTTGTATTTACATTCATGAGGCCGTTTTATTTAGGAGAGTGA
GGACAAAAGAGACAGATGAATTTTGAAGAAAGAGCAACATGAAGGGACCA
CTATCATATTCCCCACCACTATGACTTTCATATTGTTGGATGTGGGTGCA
AATTGAGTGTGAAATGGGTTAAGAAATTTCTAAAATGGACTTTTGATTTC
TTTTTCATAGATTAAAGTTGGTAAATCCCACATCGCTTGTGTGATCTGAT
CCTGAGCTAAATATAAAGCCTAGGGGTACTCCTCCCTTTCAAGGCATCTT
TTGAGAGTGAGTGAGGCCCATTGGATTTCTACATGGCAATATCCGTTCGC
AGTGGTTTATCAAAATCCACCCTGCGCACGATGAGACGTGTTAAATCCTA
CATCACTTGTGTGGTCTAATCCTGGGCAAATATAAAGCCTAGGGTAATCC
TTCTCTTCCAAGGTGCCTTTTGAGAGTGAGTTAGGCCCATTGGAAATCTA
GGTGGTATCAGAGCTATGCCTTTTGTATGATATTGGGTGTGTTGAATCCC
ACATCAATGTTGAGCTCTCCAATATCCGTTCATAGTGGTTTGTTAAAAGT
TCACCTTCTGCGTGAGGGGGTGTGTTGAATCCCACATTCATGTTGGGCCT
CCTGATATCTATTTGCAGTGGTTTATCAAAGTCTAGCTTGCGCATGAGGG
GGGGTGTTAAATCCTACATCGCTTGTGTGATCTGATCTCGAGCTAAATAT
AAATCCTAGGGCAATCCTTCTTCCCAAGGTGCCTTTTGAGAGTGAGTTAG
GCCCATTAGATTTCTACGAAAGGCATGTGAAGTAAGTGTAATCAATGATA
ACAAATTTATTTATATCCAAAAACTGAAAGTATTGGAGATTGTCATACAT
CAGAGTGAACTATAAATTAAGCGAAAATGTGGTTTTGATTAGATTAGACA
AGGGAAAGGGGAGGTGTGTGGATTTACCCAAGGCAAATCCTAGAAAAAAA
AGGATTTGAATTTGAAAATAGAAGATAATAATTTATTATTACACAATACA
TAACGTGAGCAGAGATGGATGGCCTAGATGATTATGCCATAGGAGGATGA
ATTGCAGGTGGCAGATAAAGGGTGTAGGTGAGGTAAAAGGGTTGGTAACT

TGTATAGATCATCCTTACATAAGCTCTGATAGAGGAGAAAGCCATTGGCA
AGATCAAAGATATAGTATTGATAAAAAAATACAAAGAAAATGATTGTGGTG
CAAAACTGAGCAATAGAGTTTAAGTTTTACGCATGGAAGGAACAACATAG
ACACCATTAAGAAGCAAGGAAGGAGAAATTCTTATATATTCTTTCTCAAT
GAGACATCTATTTATACAGGTATAACTAGTAAATAAGGGAATTTTGATCC
TAAATTATTGCCTATAATTACAATGGTTATTTACTACTTATTTCTCCTGT
CATTTTATGAAGACCACAAATCTCCCCTATGATTTGAAAAATACCAGTCA
ACCCCTTTGACTATTTTTTTTTTTTTTTTTCTTTTTTTTTGTAAAAAAAC
ATAANNNNNNNNNNNNNNNNNNNNNNNNNNNNNNNNNNNNNNNNNNNNNN
NNNNNNNNNNNNNNNNNNNNNNNNNNNNNNNNNNNNNNNNNNNNNNNNNN
NNNNNNNNNNNNNNNNNNNNNNNNNNNNNNNNNNNNNNNNNNNNNNNNNN
NNNNNNNNNNNNNNNNNNNNNNNNNNNNNNNNNNNNNNNNNNNNNNNNNN
NNNNNNNNNNNNNNNNNNNNNNNNNNNNNNNNNNNNNNNNNNNNNNNNNN
NNNNNNNNNNNNNNNNNNNNNNNNNNNNNNNNNNNNNNNNNNNNNNNNNN
NNNNNNNNNNNNNNNNNNNNNNNNNNNNNNNNNNNNNNNNNNNNNNNNNN
NNNNNNNNNNNNNNNNNNNNNNNNNNNNNNNNNNNNNNNNNNNNNNNNNN
NNNNNNNNNNNNNNNNNNNNNNNNNNNNNNNNNNNNNNNNNNNNNNNNNN
NNNNNNNNNNNNNNNNNNNNNNNNNNNNNNNNNNNNNNNNNNNNNNNNNN
NNNNNNNNNNNNNNNNNNNNNNNNNNNNNNNNNNNNNNNNNNNNNNNNNN
NNNNNNNNNNNNNNNNNNNNNNNNNNNNNNNNNNNNNNNNNNNNNNNNNN
NNNNNNNNNNNNNNNNNNNNNNNNNNNNNNNNNNNNNNNNNNNNNNNNNN
NNNNNNNNNNNNNNNNNNNNNNNNNNNNNNNNNNNNNNNNNNNNNNNNNN
NNNNNNNNNNNNNNNNNNNNNNNNNNNNNNNNNNNNNNNNNNNNNNNNNN
NNNNNNNNNNNNNNNNNNNNNNNNNNNNNNNNNNNNNNNNNNNNNNNNNN
NNNNNNNNNNNNNNNNNNNNNNNNNNNNNNNNNNNNNNNNNNNNNNNNNN
NNNNNNNNNNNNNNNNNNNNNNNNNNNNNNNNNNNNNNNNNNNNNNNNNN
NNNNNNNNNNNNNNNNNNNNNNNNNNNNNNNNNNNNNNNNNNNNNNNNNN
NNNNNNNNNNNNNNNNNNNNNNNNNNNNNNNNNNNNNNNNNNNNNNNNNN
NNNNNNNNNNNNNNNNNNNNNNNNNNNNNNNNNNNNNNNNNNNNNNNNNN
NNNNNNNNNNNNNNNNNNNNNNNNNNNNNNNNNNNNNNNNNNNNNNNNNN
NNNNNNNNNNNNNNNNNNNNNNNNNNNNNNNNNNNNNNNNNNNNNNNNNN
NNNNNNNNNNNNNNNNNNNNNNNNNNNNNNNNNNNNNNNNNNNNNNNNNN
NNNNNNNNNNNNNNNNNNNNNNNNNNNNNNNNNNNNNNNNNNNNNNNNNN
NNNNNNNNNNNNNNNNNNNNNNNNNNNNNNNNNNNNNNNNNNNNNNNNNN
NNNNNNNNNNNNNNNNNNNNNNNNNNNNNNNNNNNNNNNNNNNNNNNNNN
NNNNNNNNNNNNNNNNNNNNNNNNNNNNNNNNNNNNNNNNNNNNNNNNNN
NNNNNNNNNNNNNNNNNNNNNNNNNNNNNNNNNNNNNNNNNNNNNNNNNN
NNNNNNNNNNNNNNNNNNNNNNNNNNNNNNNNNNNNNNNNNNNNNNNNNN
NNNNNNNNNNNNNNNNNNNNNNNNNNNNNNNNNNNNNNNNNNNNNNNNNN
NNNNNNNNNNNNNNNNNNNNNNNNNNNNNNNNNNNNNNNNNNNNNNNNNN
NNNNNNNNNNNNNNNNNNNNNNNNNNNNNNNNNNNNNNNNNNNNNNNNNN
NNNNNNNNNNNNNNNNNNNNNNNNNNNNNNNNNNNNNNNNNNNNNNNNNN
NNNNNNNNNNNNNNNNNNNNNNNNNNNNNNNNNNNNNNNNNNNNNNNNNN
NNNNNNNNNNNNNNNNNNNNNNNNNNNNNNNNNNNNNNNNNNNNNNNNNN

NNNNNNNNNNNNNNNNNNNNNNNNNNNNNNNNNNNNNNNNNNNNNNNNNN
NNNNNNNNNNNNNNNNNNNNNNNNNNNNNNNNNNNNNNNNNNNNNNNNNN
NNNNNNNNNNNNNNNNNNNNNNNNNNNNNNNNNNNNNNNNNNNNNNNNNN
NNNNNNNNNNNNNNNNNNNNNNNNNNNNNNNNNNNNNNNNNNNNNNNNNN
NNNNNNNNNNNNNNNNNNNNNNNNNNNNNNNNNNNNNNNNNNNNNNNNNN
NNNNNNNNNNNNNNNNNNNNNNNNNNNNNNNNNNNNNNNNNNNNNNNNNN
NNNNNNNNNNNNNNNNNNNNNNNNNNNNNNNNNNNNNNNNNNNNNNNNNN
NNNNNNNNNNNNNNNNNNNNNNNNNNNNNNNNNNNNNNNNNNNNNNNNNN
NNNNNNNNNNNNNNNNNNNNNNNNNNNNNNNNNNNNNNNNNNNNNNNNNN
NNNNNNNNNNNNNNNNNNNNNNNNNNNNNNNNNNNNNNNNNNNNNNNNNN
NNNNNNNNNNNNNNNNNNNNNNNNNNNNNNNNNNNNNNNNNNNNNNNNNN
NNNNNNNNNNNNNNNNNNNNNNNNNNNNNNNNNNNNNNNNNNNNNNNNNN
NNNNNNNNNNNNNNNNNNNNNNNNNNNNNNNNNNNNNNNNNNNNNNNNNN
NNNNNNNNNNNNNNNNNNNNNNNNNNNNNNNNNNNNNNNNNNNNNNNNNN
NNNNNNNNNNNNNNNNNNNNNNNNNNNNNNNNNNNNNNNNNNNNNNNNNN
NNNNNNNNNNNNNNNNNNNNNNNNNNNNNNNNNNNNNNNNNNNNNNNNNN
NNNNNNNNNNNNNNNNNNNNNNNNNNNNNNNNNNNNNNNNNNNNNNNNNN
NNNNNNNNNNNNNNNNNNNNNNNNNNNNNNNNNNNNNNNNNNNNNNNNNN
NNNNNNNNNNNNNNNNNNNNNNNNNNNNNNNNNNNNNNNNNNNNNNNNNN
NNNNNNNNNNNNNNNNNNNNNNNNNNNNNNNNNNNNNNNNNNNNNNNNNN
NNNNNNNNNNNNNNNNNNNNNNNNNNNNNNNNNNNNNNNNNNNNNNNNNN
NNNNNNNNNNNNNNNNNNNNNNNNNNNNNNNNNNNNNNNNNNNNNNNNNN
NNNNNNNNNNNNNNNNNNNNNNNNNNNNNNNNNNNNNNNNNNNNNNNNNN
NNNNNNNNNNNNNNNNNNNNNNNNNNNNNNNNNNNNNNNNNNNNNNNNNN
NNNNNNNNNNNNNNNNNNNNNNNNNNNNNNNNNNNNNNNNNNNNNNNNNN
NNNNNNNNNNNNNNNNNNNNNNNNNNNNNNNNNNNNNNNNNNNNNNNNNN
NNNNNNNNNNNNNNNNNNNNNNNNNNNNNNNNNNNNNNNNNNNNNNNNNN
NNNNNNNNNNNNNNNNNNNNNNNNNNNNNNNNNNNNNNNNNNNNNNNNNN
NNNNNNNNNNNNNNNNNNNNNNNNNNNNNNNNNNNNNNNNNNNNNNNNNN
NNNNNNNNNNNNNNNNNNNNNNNNNNNNNNNNNNNNNNNNNNNNNNNNNN
NNNNNNNNNNNNNNNNNNNNNNNNNNNNNNNNNNNNNNNNNNNNNNNNNN
NNNNNNNNNNNNNNNNNNNNNNNNNNNNNNNNNNNNNNNNNNNNNNNNNN
NNNNNNNNNNNNNNNNNNNNNNNNNNNNNNNNNNNNNNNNNNNNNNNNNN
NNNNNNNNNNNNNNNNNNNNNNNNNNNNNNNNNNNNNNNNNNNNNNNNNN
NNNNNNNNNNNNNNNNNNNNNNNNNNNNNNNNNNNNNNNNNNNNNNNNNN
NNNNNNNNNNNNNNNNNNNNNNNNNNNNNNNNNNNNNNNNNNNNNNNNNN
NNNNNNNNNNNNNNNNNNNNNNNNNNNNNNNNNNNNNNNNNNNNNNNNNN
NNNNNNNNNNNNNNNNNNNNNNNNNNNNNNNNNNNNNNNNNNNNNNNNNN
NNNNNNNNNNNNNNNNNNNNNNNNNNNNNNNNNNNNNNNNNNNNNNNNNN
NNNNNNNNNNNNNNNNNNNNNNNNNNNNNNNNNNNNNNNNNNNNNNNNNN
NNNNNNNNNNNNNNNNNNNNNNNNNNNNNNNNNNNNNNNNNNNNNNNNNN
NNNNNNNNNNNNNNNNNNNNNNNNNNNNNNNNNNNNNNNNNNNNNNNNNN
NNNNNNNTTTTTTTTTTTCTTGCTCTTTCTTTCTTCTTTTTATGCTTCTT
CTACTCCTCTATTTCTCTCTCTCTGTGGATCCGACATGGATCTAGATAGA

AAGAGAAGAAGAAGAAAAAAAGCCAGCGCGGTGGGGGGTTGTTCTTTCTGG
GTAAGGATGTTAGTGTAAAAAGATAAAGTATATATATAGATATATATAGA
TATAGATATATGATAAAGTAAAAAAGTGATATGAGAAGAATGAGAAGGTT
TTACATTGAGTTAACCTCTCTATCTATTTATATTAATAATTAGGGAATAA
TACAAGAGACCCTAAATAATGTGGGAAACCCTAAACAATACTGATATGAC
ACGATTACCTTTTTAACACTCCCCCTCAAGCTGGAGCATATATATTGTAT
ACTCCTAGTTTGTTACAAATATACTTTATCCTTGAACCCCCTAACGGTTT
AGTGAACAAATAAGTTAACTGATCAACTGACTTTACATGTTTAGTCTCAA
TCTGCTTGTGCAATACTTTTTCTCGAATGAAGTGACAATCAATTTCAATA
TGTTTTGTCCTCTCATAGAAGATTGAGTTGGATGCAATGTGAAGTGCTGT
TTGATTATCACATACCAGATCCATCGGTGAAGAGTGTTCGAATCCAAGTT
CTTCCAACAAACGCTTGAACCAAGTTAACTCACAAGTAGCATGGGTTATC
ACCATATATTCTGATTCTGCACTTGGTCGAGCCACAACTGTTTGTTTTTT
ACTCTTCCAAGAAACGAGATTATCTCCAACAAACACACAATACCCAGTAG
TGGACTTTCTATCCGAGAGAGATCCAGCCCAATCTGCATCTGTATATCCC
TTAATTTGAGTGTGACCAGAGTTTTGGTACAACAAACCACATCCTGGAGA
ACCTTTCAAATATCGAAGAATACGAATTACTGCATCCCAGTGATAAGTTG
TGGGACGATCTAAGAACTGACTGATAACACTCACAGCAAACGAAATATCT
GGTCTAGTAACCATCAAGTAGTTCAATTTTCCAACTAACCGTCTATATTG
CGCTGTATCTGTCAATGGTACACCTTGATCTGTCAATAGTTTGCTGTTTG
GATCTATAGGTGAATCAATCGGTTTAGTTCTAAGCATTCCTGTTTCAATC
AACAGATCGAGAACAAATTTCCTCTGAGACAAGTAAATACCTTGACTTGA
TCGAGCAACTTCAATCCCCAAAAAAATACTTCAATTGACCCAAATCTTTGG
TTTGAAATTTACTATGCAAAAACGTTTTGAGTTCCTGAATACCCTGGTGG
TCATCTCCGGTAATCACGATGTTATCCACATACACAATCAACAATATTCG
TCTGACTTTACTATGCTTATAGAATACAGAGTGATCAACTCTACAATAGC
GAAGACCAAATTCAGTGACAACTTCACTGAATCTCCCGAACCATACTCGA
GGAGACTGTTTGAGGTCATGGATGAATTTCTTAAAGCGACATACCAAACC
AGACTCCCCCTAAGCAACAAACCCAAGAGGTTGCTCCATGTATACTTCCT
CAGTCAAATCACCATTTAAGAACGCATTCTTGATATCAAGTTGATGCAAC
GACCAACGAAAGGTGGCAGTCAAAGAGATGAACAAATGAACAAAAGCGAG
TTTGACAACAGGAGAAAAGGTCTCAGAATAATCAGGACCATATACCTGAG
TGTAACCTTGGGCAACTAACGAGCCTTGAGTCGATCAATGATGCCATCCA
AATGAAGTTTGACTATGTATACGCATTTACATCCAACGATTGTTTTGTCA
GGCAGAAGCGAGACGAGATCCCAAGTACCATTCTGACGTAAGGTTAACAT
TTCCTCCTCCATTGCAGTCGTCTAACCAGGGTGAGTAAGAGCTTTTGTGA
CAGACTGTGGAATAAAAACAGAGGATAGAGCAGCAACAAAAGAGGAATAG
AAAGGAGATAGGTGTGCATACAAAACAAAGTTAGAAATAGGATGTTGAGT
ACATGTACACTTACCCCTCCGGACAGCAATGGATATGTCCAAATCAGACG
CGAATGGATGAACTGACGAAGAATCTGAAGACACAGAGACAATTGGCTCA
ACGGCGGGGACTACCTTCTGGCGACGTGAGTATGCATGTAAAGGCGGTGA
ACAATGACTAGGCGGGACAAGTGCGGGAGCGGAAGGGGATGGTGTCGGGT
CTGGGAGGCTTGGTAAAGGAAGGGTGACATCTAGATTATCCCTAGAGGAT
AGGCCAGAAAAGTAAGGGGTGGACTCAAAGAAGGTGACATATGTAACCCT

TTTGTGTACGAGAATAACCAAGGAAGACACATTTGATGGCACGAGGATCT
AACTTATCCTTGCCAGGAGTCAACAGGTGGACAAAACAGACACATCCAAA
GATACGGGGAGGTAAAACAAACCACGGTGATCAAGGAAAAAGAATAGAAT
GAGGGACTTTGCCATTCAGGGTAGAAAACGACATTTTATTGATCAAGAAG
CAAGCTGTAAGAACTGCATATGCCCATAAGCGTTTGGGAACATTCATCTC
AAACATCAAAGTTCGAGCCACTTCTAGGAGATGTCGATTCTTACGCTCAG
CAACTCCATTTTGCTGCGGTGTGTGAGGATAAGAAGACTGATGCAGTATA
CCAGACTGTGATATGAATGAAGTAAAGGGTTAAGAAAAATATTTTCGAGC
ATTGTCACTACGAAGTATATGCACGGGCACATGAAATTGAGTTTTAATTT
CAGCACAGAAGGCAGAAAAAATGGAGAACAATTCAGAACGATCTTTCATT
AAATAGAGTCATGTAACCATGGAGAAATCATCGACAAATGTGACAAAATA
ATGAAAGCCTAATGTAAAGGCAATACGAGAAGGACCCCAAATGTCTTAAT
GAACTAAACAAAAAGGACTCGATGCTCGAAACTGAGCCGGGGAAGTAAAT
GAAGTACGATGATGCTTTCCCAACTGACAAGACTCACAATCTAAGGAGGA
CAAATGACGTAAACTAGGAACAAGTACCTTTAGATTGGTAAGACAGGGAT
GACCTAGGCAGCAATGAATTTGGTAGGCAGATGCAACAGCAGAACAAGCA
GTGGGCGGAATAACATGTGTCTTTTGTGTTTTCAAATCCTGGATAATGAC
AGAATCAGGAAAGAAGCAAACAGAACAATTAAATTGTTGACTAAGCTTAC
TCACTAATATCAAATTAAAAGCAAAAGATGGAATATACAAAACAGAGGAT
AAAGAGAAAGATGGTGAAAGTTCCACATCGCCAATACCACGAACAGGGAC
GGTGGAATCATCAGCAACAATAACAGGGGTCAATAAAGGAGATGACTGAA
TATGCGTAAATTAAGATGTACCTGACATGTGATCAGTGGCAGCGGAGTCT
ATGATCCAAGGTTTAGAAAGGGAAGGATGAAAAGAAAGACATGCGGTAGG
ATTACCTCGTTGAGCTAGCGAAGTAATGGGGGTAGATGTCTGCTTGGACG
TCTTAAACTGGAGGAACTGAACATACTCGTTATCAGAAATAGCCTTGGGA
GTTGAAGAGTTGGATAATGAAACAAGAGCAACTCTGAACCAACAAGTATT
TTGGGTATGATTTTTCTTGCCACAAAGTAAACACTGACGATGTTGACCAC
AACAATGGCCACCTCGAAATTCATTACTACTACTTCCTCAGTCACCTCGT
TTCCCTTGATTGGTAGCGACTACTTGTACAATTTCGGACCATTGTAAGCA
ATTGGTCCCATTCAACTTGCGTGTTGTCAACGGTCAGGGGATAGCCGTCG
ACATTAATTCCCGTCAATACAACAACTAACAAACAACGTGTACAAGGGAA
ATAAACCAACAATACAAACAATTGTTGGGCTTAAACGAAGAACATAAGAA
TCAGAACAATGCTCACGAACATATGAGACATTTTTACGAACAATAACTCT
TGTGTTACCGGCAGATCTGGACAGAAGCAGCGGAGCAGCGGCGAAGGTCG
TGAGTCTCAAATCAGAGTAATCGAAGGCCGACGAAGGTTTGGGAAGGCCG
GCGAAGGTCCGGGAAGGTCGGCGTAAGTCCGGCGAAGGTTGGTGATGGTC
GGCGACGTCAGACAGTGGACAACAAGTGGGAGATCTTGGTTCGCGATAGT
GGTACGACTCGATTTTGAGGTTGATTAGGGTTCGCGATGATGACGGCGGC
TGTGCGACGGTCGGTGAGGCTCGAAATAGCCTGGGAGACGGTCAGCGAGG
CTCAAACAAGCCTGGGCGACGTGGATCTGCGATGGCGGCGAGACGAATCT
GATGGAGCAGCACGGTGGTTGTCAGCGGTAAGTTGCTAGGGTTCCAGATT
TTTTTTTTNTAATTGTTAAAATAATAGAACATGATATGAAAGGAACGAGA
AGGCTTAACTATTGTGTTAGCCTCTCCCACTATTTATAATAAGAATTAGG
AAACCATACAGTAAACTCTAAGTAATAGAAAAGACATAAATACCCTTTAA

CACTCTCCCTCAAGCTGGAACATATATATTGTATGCTCCTAGTTTGCTAC
AAATGTATTTTATCCTGGAGCCTCCCAATGGTTTAGTAAATATATCAGCT
AACTGATTAATTGAATTTACATGTTTAGTTTCAATCTACTTCTGCAACAA
TTTTTCTCGAATGAAGTGACAATCAATTTCAATATGTTTTGTTCTCTCAT
GGAAGACTGAGATGGAAACAATGTGAAGTGTTACTTGATTATCACATTCC
AGATCCATCGGTAAAGAGTATTCAAACCCAAGTTCTTCCAACAAGTGTTT
CAACCAAGTTAACTCATAAGTGGTATGTGTCATTGCCCTGTATTCCAAAT
CTGCACTTGACCGTGTCACCATAGTCTGCTTTTTACTCTTCCAAGAGACA
ATGTTACCACCAACGAACACACATTATTCAGTAGTAGACTTCCTATCCGA
GGGCGATCCGGCCCAATCTGCATCTTTGTAGCACTTTATTTCAGTATAAC
CGAAGTTTTGATATAGCAGACCATAACTAGCACCTTTCAAATATCGAAAA
ATACGGATCACTGCATGCCAGTGACAAGTCATTGGACGATCTAAGAACTG
ACTGATAACACTAACAGCAAATGAGATATCTGGTCTAGTAACTGTCAAGT
AGTTCAACTTTCCAACTAACTGTCTATATCTGTCAATGGTACACCTTGAT
CTGCTAATAGTTTGGTGTTTGGAACCATAGGCGAGTCAATCGATTTAGTT
CCTAGCATTCCTATTTCGCTTAATAGGTGAAGGACATATTTTCTCTAAGA
CAGGTAAATATCTTGACTTAATCGAGCAACTTCAATTCCCAAGAAGTACT
TTAAGTGAGCTAAATCTTTTATTTGAAATTTACTATTCAAATTTTTTTTG
AGTTCTTGAATACCATCATGGTCATCCCCGGTAATCATGATGTTATCCAC
ATACACAATCAGTAATATTCTCCCGACTTTACAATGCCTGTAGAAGACGG
GATGATCGACTCTAAATCGTTGAAGACCAAACTTAATGACCACTTCACTA
AATATTTCAAACCATGCTTGAGGAGACTGCTTGAGTCCATAAATATATTT
CTTCAAGCGCCATACCAATCCAGATTCCCCCTGACCAACAAACCCAAGAG
GTTGCTCCATAAATGCTTCCTCAGTCAGATCACCATTTAAGAAAGCATTC
TTAATATCGAGTTGATGTAATGGCCAACGAAAAGTGGCAGCCAAAGAGAT
AAACAAGCAAACAGAAGCGAACTTGGCAACTGGAGAGAAGGTCTCAGAGT
AATCAAGGCCGTATACCTGAGTATAGCCTTTAGCAACTAAGCGAGCCTTG
AGTCGATCAATGGTGCCATTAGGATGAACTTTGACAGTGTACACTCATTT
ACATCCAACTATTGTTTTGTCAGGTGGAAGCGAGACTAGATCCCAAGTGT
CATTCATCAGTAAGGCAAACATTTATTCTTCCATTGCTTTTGTCCAACCA
GGGTGAGTGAGGGCCTCTGAAACAGAATTAGGAATAGAAACAAAGGACAG
ATGAGCAACAAAAGAGGAGTACGAAGGAGATAAGTGTGCATACGAAACAA
AGTTCGAAATAGGATGGGTAGTACATGTGCGTTTACCTTTCCGGATGGCA
ATGGGCATGTCCAGGTCAGAGACGGATAGGTCGGCTAGAGAAGAATCTGA
GGACACAGAGTCGATGGACGCGATAGCGGAGGGGGCCTTCTGACAATGAG
AGTAGACCTGCAAAGGAGCTGAAGAACGACTGGGCGGGATAGGATCCGGA
GTGGAGGAAGATTGTGTACGGTTTGGTATTCGTGGCAAGGGAAGTGTAAC
ATCCAAATCGTCTCGAGAGGAAGAGGCTTAGGTAGACTTAAAGAATGTAA
CATCCCCGCACACAAACGAGCGACGTAATGATGGACTGTAACAACAATAA
CCCTTTTGTGTGTGAGAATAGCCGAGGAAGACACATTTTAAAGCACGGGG
ATCTAACTTGTCCCTACCGGGAGTCAACTGATGAACAAAACAAACACACC
CACATATTCAAGGAGGTAAACTAAACAAGGGTGAACGAGGAAAAATAATG
GAATGGGGAACCTTGCCATTTAGGATAAAGGACGGTATCCGGCTGATTAG
GAAGCAAGCTGTAAGAACCGCATCACCCCAGAAGTGTTTGGAAACGTTCA

TCTCTAAAAGAAGAGTACGGATCACTTCTAGGAGACTGATGCAAGATATC
AGAGTGCGACATAAAGGAAGTAAATGGCTGAGAAAAGTATTCTCGAGCAT
TATCACTACGAAGAATACGCACTGGTACATTAAATTGAGTTTTAATTTCA
ACACAAAACCCACGAAAAATATAGAATAATTTTGAACTATCTTTCATTAA
ATAAAGTCAGTTAACTCTGGAAAAATTATTTACAAATGTCACAAAATAAT
GAAAATCTAATGTAGAAGTAACACGAGAAGCACCCCAAACATCTGAATGA
GCTAAAGTAAATGGACTCGATGCTCAAACATGAGCTGGGAAAGCAAATGA
AATACGACGATGCTTACCTAATTGACAAGACTTATACTCTAACGAAAACA
AATGACTCAAATTAGAAACAAGTAACTTGAGGTTGCCAAGGCAGGGATGA
CCAAGGCGACAATGGATGTAGTGAGTGGAGGCAACAGCAGGGCAGGCAGT
AGGTAGGATAGCAGGACTCTCGTCATGGTCATCAATGTAATATTGGCCAT
TTTCCTCACGCCTGGTGCCAATCGTCTTTTGCGTCCTCAAATCCTGAAGA
ATGACAGAATCAGGAAAAAATAAAACAGAACAATTAAGGTGTTTAGTAAA
CTTACTGACTGACATCAAATTAAAAGAAAACGAAGGAATATACAAGACAA
AGGATAGAGATAAGGAGGGTGAGGGTGCCACTGTGCTAATTCCACGGATA
GAGACAGTGGAATCATCAGCAACTGTAACAGAAGACAACGAAGAAGATGA
CTGAATATGTGTAAAGAGGAAAGAAGTACCTGACATGTGATCAGTGACAA
CAGAATCTATGACCTAGGGTGTGGGAGGGGAAGGGCGAATTGAAAGACAA
TCAGTAGAAGTACCTCGTTGAGCTAAAGAAGTAATGGGAAAATATGCCTG
TTGGGATGCCTTATACTGAAGGAATTGAGCATATACATCATCAGAGATGT
CCTTGGCCTCTTAAGATTCTGGAATAGTAGGAATATTTGGATCAGATGTC
ACTGCATTGGTGAATTTGGGAGGACGACCAACTAAATCCCAACATGTTTC
TCAAGTATGGTTATCCTTTCAGCAAAAAGAGCACTGTCGATGTGAGCCAC
GACCACGACCACTACGTCCATCCCGAGGGCCACCACCACAAGAGAAACCA
CCACCATCACGAAAAAAACCACCCCGACCACCATTGAGTGGATCACCACG
ATCTCGTGTAGGAATCTGAGTAACAAGCATCGAATGTTCAGACGACGTAG
ACGAACTCAAGCCAGACACATTGTCCTTGGCAGTGCTACGCGAGATGAGA
GAATACATTTCTGTGATAGTGGGAAGAGTAGCACTAGAAAGGATCTGAGA
TTGGACTGGTTCAAATTCTGGGCCTAATCCAGCCAAGAACTTCATGACAA
CCATTTGCTCACGTTGCTTTTGCATTTCTTTCAGATCATAGTCATCGGTA
GGATAGTATTTATCTCCTCATAGAGGTTGTTGAATGTGGCAAAATAATCT
GTAACAGATTGCGTGCCTTACTGAAGTTGGAAGTACTCTACCGATAAATC
ATACATTCTGGTGAGATTATTAGAGTATAATACACGAAAATACTCCCATA
TATTTTTGCACGTATCTAGATGAGTACAGAGATCAACAACTTTAGGCTCT
ATCGAATTACACAACGATGCTACAATCTGCGCATCAGCCTGAATCCAGTC
GTCATACTTTTTATCATCTGTTGCCGGGGCGGATTCTGTGAGGTGACGAT
GTTTCCCTTGTCCGGTGAGGGCTACTTTCACAGCCTTAGACCAATTTAAA
TAATTGGTTCCATTCAACTTACGGGTTGTTAATGGTTGAGGTGTAGCCAT
CGACATTAGTACCCTCCAATACAACAATTAATAAACAACTTGCACAAGGG
AAACAAACCAACAATACAAACAACTGTAAGGCTTAAACGAAGAACATGAG
AATCAGAACAATACTCACGAACATCTGTAGGGCTGTCTTTCAAACAATAA
CCAGAAGTCTTAAGTCTTACGAACAATAACCAGAAACATAACTAGAAGTC
TCAAGTCAACGCAAAAGGCCGGCGAAGGTCCGGCGAGGGCCGACGACATC
AGAGGAGGACAACGAGTAGGAGAACTGGTTCGCGATGATGATATAGCTCG

ATTTTGGGGTCGATGATGGTTCGCGATGATGATGGCGGCTCAGAATGGCC
TGGGCGACGGTCGGCGAGGAGCGGATCTGAGTGTGTTGAGCGGCGGCGGC
GACGACGGTGAGGGCTTTTAGGAGCTCTTTTTTTTTTTTTTTTTTTTTC
GTATGACCAGTAGGCAAGGGAAAAGAGAAGAAAAAAAAATTTGAACAGAT
CTATGAAATCAATCTCGCATAACCTGCTCTGGATACCATATTAAAGTAAT
AGAATATGATATGAAAAGAACGAGAAGGCTTAACTATTGTGTTAGCCTCT
CCCATTATTTATAATAAGAATTAGGAAACCATACAGGAAATCCTAAGTAA
TAGAAAAGACATAAATACCCTTTAACAAATAATTAAGGAACAATACAAGA
GACCCTAAACAATGTGAGAAACCCTAAACAGTACTGATAAGACATGATTA
CCCTTTAACAATATATATATATATAAATCTTAAAGGGATATTTATGTATTTG
ATTTTTTAATTATTTTAAATTTTTAAGGGTTCAATTTTTATTACAATAAA
AATTTCTGTAATTTTTAACCTATAGAGGAATTAAAAGTTGTTTGACCAAA
AATGATAAGGGGTTGACTAATATTTTTCAAATGATGGAGGAAGTTTGTGG
TTTTAAGCAAAATGAGAGGGGAGGTAAGTAGTAAATATCTTAATTACTAT
TATTTGATGGCATATTAATTCTCATTTATTTGCAATCATGGAGGGGCTGA
AATCATGATGATATAACATAGCCCTGCTAAAGTGAATTATTAATCAAAAC
AAATCTCAAAACTTGTTACGAGTGGATTGAACATTTCGACCATGTTCTTC
ATCATTAGGGCAAGGAACTAGAATTTGAATAAGATAGGCTTCGTATTTCA
GTGGAGAGAGACTGGGTTTAGAGAGTAATATAGATGTTAGAACTTTGTGA
GGCCCCTTTTTTCTGTTATTGACATGAAATGGTAATATCAATTTAAAGCT
TAGACCACTAGAAAGGTGCTTTAGTTTTTAGGCCACCACCCCCCACCAGC
TGCCAAACCATGGCCTTTTGCTGCAAGCTAACCTCAGCTGGTCGTTCTAC
GTTGAATGTCACAAAGGAAGTGGCAGCCTTCAGGGTTACTCATCAATACG
TCTTGACTTACAGCCAAGTTTCTGGTTTTAACCCTTCAATAATTACCAAC
CGACATTAGGACATCCCGAAAGCCTTTCAATCCAAGATACTATATCCTCT
GTTTCCCACTGTGGAAATTCAATATTCATTTCAAGGAAGCCTTGCTCATG
ATTTGGCCTCTCCTGGTCATCATCTCGTTAATAATTGCCAATTTGTTTTC
AGTTTTCAGGCTATTGTGGTTATGAAGATGGGTCTTGAGGAGAACCAATG
AGATTCAATGATAAACCGACTTTATCAATGGTATCATTTTTTCTCTGATG
TCAGAACATAATATTGGATTATTTGAATATTCTCGCCACTAATTAATCTG
TTCATCGTGCAACACTGTCAAAGTTTTGGCCATGTTGTTGAAATTGTCTA
CTAGTTCACACAACACCTTCCTAAAAATATAAGAACCATAAATCATATCT
GGGGACAACTAGAATTTATGGCAGAACTAACTCGAAGAACTCTCCCAAAT
TACAGTTTACTTGAAAAGATCATCTGAATTTTGAATTAGGGAAACTACAC
ATACGCAACCTTAATCGTCGGATAGTTTTTCCCATTGATCATATTTAGAG
GCCTGAGATCAAATCTCGTTTTCGTGTTTTTTTGGAAAAAGGGAAAAGAC
GCCCGCAGGGGGGGGTGTTCGTAATTTTAGGGTGTAATTTTATCTTTATT
GCGAGAATTTTACACGGTTTGGTGCAAATTTTGTTTCCAGTTAGAGAATA
AATTTTTTGAGGTTTGATGTTTGATTATCGTTTATAATAATAGAGATCCC
AAATTACGTCCTTCATTTGCCGAAATTATGGTTGCTCTGAAGCCATTGCA
AAAGCCGCCTGTTACAAGTGCACAAGTGCCCAGACGTAGTTCTTCAGTAC
GTGGTGGCCAGGTGTAG
>EUC21989-RA [gene]
ATGAAGAATTTTCAATGGTTTAAGCAAATCGCGAACAACGGGAAGCTCGA

GAGGCGACTCTCTCTGGGGGAGTACAAACGGGCTGTATCGTGGTCCAAGT
ACCTGGTTTCCTCCGGCGGCGAGATCAAAGGGGAGGGAGAAGAGGAATGG
AGCGCCGACATGTCTCAGCTGTACATTGGGAACAAATTCGCTTCGGGTCG
CCATAGCAGGATCTACAGAGGTGTGTATAAGCAAATGGATGTGGCGATCA
AACTCATCAGTCAGCCAGAAGAAGACGGAGACTTGGCTTCTCTACTAGAG
AAGCAGTTTACGTCGGAGGTGGCTTTGCTTTTCCAGCTGAAGCATCCCAA
TATCATCACTGTAATCTCTCTCTCTCTCTCTCTTTCATACTATTATTACA
TTGCTTCCTAATTTGATCTATACACTCACGGCTCGTTTTGAACATAATTG
GAGTGGAATGGATCGCCATTCCACGTCTATTCCAATTCTCTTATTTGTTC
AATGGAATAAAAAATTAAATTTGATGATTCCATTGGGAGGAAAGATGATG
TAATTGAATTTGATAGTTTATAGGATGGAATCTACCTAACCCAATAGAAT
AAAAAAATAATTTCCACAATAAACTCATTCCAAAAGAAATAATTTATTTT
GTAGTTATTTTTTTTAAATAATCTATTATTAGATATATATTCAAAATTTT
TAAAAAAAAGAAAAAAAAAATATATTCAAAATTAATATCCAAAAGGGCAC
TATGAAATTTTTTCTTTGTTCACTATTTTATTGTGATTCAATTAATAAAT
TTATTCCATTTTTATTCTCATTCCATTCATTTTATTTTACCTTGATTCCA
TTTCATCCAACCAAATGAGAATCACTGTAATAGGATTTCAATTCTTTTTA
AAATTGGAAGCAAGTATAAGGTGGCGTTTGTTTAGTATTTGTGCTCTCAT
ATTTCTATTTCGGTTCTAGAAAACTCAACAAAATCTGAAATTTGGAAACA
ATTCTGTTCTAAAATTATTGGAATAATTTATGAGAACAGAAACCCTAATT
TGTGACTTGTTGGAAGAGAGAACTTTTCCCCTCCTTTTCTGAGAAGGAAA
CTAATTTATATCTAATGGGATTTAGATTCTAGATTGATATAGAATGGTTT
CCATTCTGTCTGGTGTAAGAATAAATTAGGTAATATACCCAAGAACATGA
CTGCCAAAAACAGGTATTCATAGTCCAAGTACATGACGAAGTTCTTCCAT
TTCTAGTACAAAACTACAAACAAAGATGTAAAAAGTGGTTTAAGCTTTTC
TTTTTGTGTTGACAATATCATTATAATGCATTTGTTAAACATCAAATTTA
TTCGGGTAAGCTTTATTGACAAGCAATATATTGGAATAATGGTCGAGCTG
CCAAGAAAATTGCAAAAAGATGCGGTATCGTATTCATTGTATTTGATTTT
ATGATTTGTTAGGATATTCCAACTTAAAATCCTATGGTGCCAAGTGGAGT
AACTTTTTTGACTTGTGTAATGGTTTTGGAACTCTCCAGTGAACAATGTG
GGAGAAGAATTTAACACATGCGCTTAAAGGTATGTTTGGTTGGAAAAATA
GAAAAGGAAAAGAAAAAGGAAAATAAGAGAATAATATTTTTATCCATTAC
GAAAGAAAAATAAATTTTCCTAGTGTTGTTTAGTTAGGAAAGAAAATGAA
AAGAGGAAAAATAATATACTTTAATGTAATTTACTATTTTACTTTTATAT
ATATAAAGATAAATTAAAAAAAAAAATCTATTATTATGGATATAATTGTA
ATTTTGATATTTTTTTCTCAAATTTCAATCATTTCCCCTCTATTTTCATC
CCAAATTTGAGAAGAAAAAAAGAGAAAGAAATGAGCATTTTCTTCCCCCC
TTTTCTTTTCCATTCCATTTTCCTTCTTTACCAAACACATTTTTTTTTTC
CATTTTCCACCTCTTTTTCTTTTTTGTTTTCCCATCCACTCTATTTTCTA
CCCAATCAAACATATCCTAAGTGTAACCTTCGGTTTGCACGAATAATTTG
ACTACGAGAAAATAATAAGAAATGTGCAACTCTTAGTTTGCACGAATGAT
TTGACTATAAGGGAATGATGAGAAATATGCAACCCTGATTTACATGGATG
ATTTGACTATGAGGGAATGATGAGAAATGTGCAACCCTCGGTTTGCACGG
ATAATTTTATTATGAGGCAATGAGAGATGAATTACTTTAACCTTGGCTAT

GACATCATATTAGAAGAGAATCTAACAACCGCCCTCAAGTGCAAGTCTCG
GTTTGTAGGAATGATTTGACTATAAATGAATGAGAGACGAATTAGTATAA
CATTGCCTCTAATACTTATGTTAGTTTGGAGGGAATCTAACAACCCCTCT
CACGAGGGAATGTTTGGTACAAGTGTACAACAAAAAAACCTAGGGGAAAA
AAAATCATGAAGGAAAATTAGAAACGGACAAAAGGTGTCCATTGTAATTG
GTTCCTTTGGTTTGCACGGAAAATAGAGTGGAAATAAAAGGTGGAATTTA
AAATTCCCCTCTTAGATGGAATTTTTTTGAAAATATTGTCTTCATTTACA
AAAATTTACTTTTATACCCATATATATGCTCTTCTTTCTCTGCATTTCAT
GTTTTATATATTAGTATGTACGAGGGTACAACTGTAAATATACTTAACAA
TCATTTTCTTTTCACTTATACCAAACAAAAAAGAAGGATAATGATTTTCT
TTTCTCTAAAGATGAAAATTGCATTCATCCTATTTTCCGTCGTTTTAACT
TTCCATCCTTGGTACCAAACGGAGCTATAATAGTGAATTGCTTTATGATT
GTGTCGGAAGTATTCCTTTCCTTGTTGTCTATTTTACAATTCAATTTCGT
CAGAACCGTAAAGCTATTTTCTTTCATTTTTGTCATCTTATCCAAACATA
TCGTCTACTTTTTCCATGTAATCTATCTGAACATCTTTTCAAACTTTTTA
CTTTGTTATTTTGGCAGCTCTTTACCCTTTTTTTTCCTTTTGGTTTCTCA
ACAATTGAACACCAAAAGTGATTAAAAACATTGATAATTAAGAAATTACT
TTTATGTGTATATATGGGTCTTGACCCAGTTAAATTATAATCCCACCAGA
AAAACCCTAGTCACTTCTTTGTACATCTTTGTAACATTGTAATTTTCACA
CATATCAATATAGTCATCTCAAATGCGCTCCCTGTCAATTATTCTAGCAG
AAATCACTTATTAATAAAATAAATTTTGGTTTCTAATGGAATGGCTTTGT
TGAAGAAATTTAAAAAACAAAAATAGAGATTAGCATTTGGAATTCAACAA
CCCATGATCTTATAGGTAGCGTTTGGTAACCTGGAATTGAGGTCTTGGAA
TTGGAAATAAATGCATGTAATTTTAAATTTTTACATGCATTCATTTATGT
GCATCCAAACATAGGGATTGTGATTTGATCTTACAAGGCAAGTATATAGA
AGAAGACTGATCGGATTGCCCTTGAAGCTAAAAAAAAAAAAAATGGCTCTT
AATGTTACTTGTGAATTGAATGACTTTCGGCATGTTTGGTTGGACGGAAT
GGAATGAGGGTGGAATGGAATGAGAAGATAAGGAATGGAGCTGAAGGAAT
GTAATCGCGATTGTGAAGGAATCCACTATCGCGATTAGCTGTGCTTCTTA
TGAAGAAAAGAAAGCAGGACATATCGCATGGATATTTGCCTAAAATTTAT
TTTCGCCCTTTAAGTTTGAATTTCTTCAGTTGGCATAAAATTAATACCAT
ATTCTATCCTTCCATATATATTTTAATAAACGTCATTTATTATTTCCTCG
ATCGTCTATTTGCCTTCTCATATCAGTTCTATTCGTTAAGTTTCGTTTTT
TTCCAGGTTTCCTATTTCGTTTGTCCCAATTCTAATTCCTTGTCACATTT
CCGTCGCGTAATCTTCTTTTGTTCCGAAGTTCAGTAGTTCTCATTTTCTT
TTTGTGCTCATTGTCTGTTTAGTTCAATTTCGGTTGTCCTTTCTCCCAAT
TCGATCGCTATTTTATGTTTTGTTTTCAATATTCTTTTTTTTCTTCAATT
TCCTTCGCTATTATTTGTTCTCAATTTCATATGTTCTCAATTTTCGTCTT
AATTTTATGTTTTCCAAATTTATTTATTCTCAATTTTCTTTATCGAGTTC
TGTGATTCTCAATTCGTTCAGTCTTAATTCCGTTTCATCTCATTCAGTCT
GTCCAATTTTGTCTCAATTATTATCTTCTCCAACTTTTGGTCTCAAGTTC
CAATTTTGTCTTCAATCATTTTGAATTCGCAATGTCAAGGGTAATAGAAG
GACGATCATGTAATTTCAGAATTGACCATTGGGTTGAACTCTTCCACCCA
TTACAATGGAAGCCATCCATCAAATTTTCATTCCCGAAGTTATCACAAGT

GATTCCAGTGGAGAACCAACAACGAACATAGGGACATCGAGGCCGAATCC
ATCATTCCTCCTTGATTCCATCCAACCAAACATGACGTTTGTGCTTTAGA
AAAAGTGCTTTTAAAAATCAAGAAAGTAAAAGTATATTTCATATCCTGTT
ATCTGACGCACTTCTGATTTTTTGCTTTTGTTTTTTTGACTAAGAATATT
TTTTTAATTCATTAACTGTTCTAATTATAAAAATTACTTAAAAAAAAAAT
CATTACCACGGTGTTTAAAATTTATTTCACCGGTGTATCTAAAAAACCT
TGACTTATGACATCACTCTCTTTCCTCTTTACTTAAGCAGTTTGTGGCGG
CATGTAAGAAACCCCCTGTATTTTGCATAATCACCGAGTACTTGTCTGGG
GGCTCCCTCAGGAAATTCCTCCACCAGCAGGAGCCGCACTCGCTTCCGCT
CAACCTCGTTCTCAAATTAGCTCTCGACATTGCACACGGAATGAAATACC
TTCATGCAAAGGGCATACTTCACAGAGATCTCAAATCCGAAAATCTACTC
TTGGACGAAGATATGTGTGTGAAGGTGGGAGATTTCGGTATATCGTGCTT
GGAATCTCAGTGTGGAAGCGCTAAAGGATTCACAGGCACTTACCGCTGGA
TGGCACCGGAAATGATCAAAGAAAAACACCATACAAAGAAAGTCGACGTT
TACAGTTTTGGTATCGTGTTGTGGGAGCTTTTGACCGCCTTGACCCCCTT
CGATGACATGACACCGGAACAAGCTGCATTTGCTGTTTGTCAGAAGGTAT
TAATTCTTAGGTAGTGTTAGGCAAGACGGAAGTCAAGCCTTCGAATTGGA
TTTGAAAATGAAGGCATCTAATTTTTTTTTACATTGTCACATGCATTTTC
ATTTGTAATTCCAATTCCGAGGCTCTATTCCACCAAACCAAATGCTATTT
ACCATTGCGTAGTTTTCTTTTTCTTTTTCTTTTTAACTGGTTTGATTATG
AGAGAGCTCATGATCATCATATCGCTCTATTATGCACCTTTGCACTAGCA
TTTGAAATGGTTCCATTTAGGTTCTAGGTTTATCTTGGTATTCATATTTT
GCATGTGGTGCATGAACAGCATCTGCACAAGTTTTAAATTTTTATATTAC
TACCTAGATTTTGAAATTTTTTCAATTATGTATTAAAAAAATTTCAATTTA
GTCCATAAAAAAAATTACACTTTTAACCCAATTATTTCACCAAATTTACAA
CAGAGTACCACAATGTATTATGGCCCCTAAATGTTTTTGTGCAGAATGCG
AGACCACCGTTACCTTGTTCATGCCCAAAGGCATTACAGCAGTTGATTAA
CCAATGCTGGTCCAGTAATCCGGTTAAGAGACCACATTTCAGGGAGATTG
TTTCGATTTTGGAAAGTTATGCCGGCTTGCTGGAGCAGGATCCAGAATTT
TTTGCATCTTATGAACCTCCAGATGATCGTTCTTCTTTACGGTTTGTTCC
CAAGTGTATTTCTGCTTGTAGATCTTCTGCTGCTGCTTTTTAG
>EUC21992-RA [gene]
ATGAAGGAGAATAATGATGGGTTCGTGAGGGCGGATCAAATCGATCTGAA
GAGCTTGGATGAGCAGCTCGAGAGGCACCTCAATCGGGCATGGACGATGG
AGAAGAACAAGAAGAGTCACGACTCCGATTCCGGCACCACCGCCGCTGCC
GCTGTCTCAGCTGCTTACTCCACATCCAGAACCTCCAGGCAGAGAGAAGA
GTGGGAGATCGACCCATCCAAGCTCATCATCAAGGGCGCAATCGCTCGTG
GCACCTTCGGCACGGTCCACCGTGGCATCTACGACGGCCACGATGTCGCC
GGTCAGTCACTCACATCACTACTGTTTAAATTTCCTTTTCCTCGAATTTA
ACTTTACCAAATCCAAATAGTTGGTCAATAATTATATTTTAAATGATAAT
TACACCATATAAAATTGCACCTAATTGATTTGCACGAGAGATGATATTCT
ATTTCATAACAAAATTGCATTTTTTTCTTCTTAAAAAAAAATTAGATCTA
CAAATCCAAAAAATAAATGGCGCAAAAAAGTAGCAAGCAAGTCAAATTAT
AGTTATCCTGTCTAATGAAATTAGTAAGATTTTTAGATTTCCCTTCTTTT

TTTTTTTTCTGTTGTTGTTGTGGCGTTGCTTTGAGCTTGTGGTTGGGAGT
GCTCTTAAGGAATCCGTGAGCAGTGCACGGATTGGGATTGAAACTTGTGT
GGGTCACTCACTTTCCTATAAGTAACTTTGCCTGATTCTTGCAGTGTTTC
ATGTGATCCAAATTAAGACAAAACCCTCAATTTATTTAATGTTGTGAAAG
TGAAAGTCTTGCTCAAATTTACACAACTCTCTCTCACTCTCTGAAGGAAA
AAAAAAAAAAAAAAAAACACTTTCCTAGGTTAATGTTTGTCAAAACTAAAA
TGGGTTGACGTTTGTTTGAGTTGATTCCTCAGTAAAACTGTTGGATTGGG
GGGAAGAGGGACACAGGACGGATGCGGAAATAGCATCATTAAGGGCAGCA
TTTACACAAGAAGTAGCGGTTTGGCACAAACTCGAGCATCCTAATGTAAC
TAAGGTCACTTTCTACTACCTCTGCAACCTTTTTTTTTTCTTTTTCTTTT
TTTCAAAAAAAAAAAAAACGATAAGGCAGCATTTGGAAACACGGAATTAGA
GCTTTGGAATTGGAATTAAAAATAAACGCACGTAAATAACTAAAATTTTA
TATGATTTCATTTCTAATTCGAATTCCAAAGCCCCAATTATGTGCAAACA
AACACAACCTCAATGTAATTGCATTTTTTGAATGTTGCTTTCTCTATGTT
GATGGACTTCCTAGCCTATGATTAACATGATCTAAGAAACGAATCTATGG
AACCCTTGAATTTCGTTGAGAAAGCCAAACCGTTGCTTCAATTGTCTCTT
ATGAAGTTCTTCGTAGTGTGAATGGAGTGCCTTTAAGCACTTGGAGGAGA
GCCTAAATGTGATCTAAGAAACGATCCTACATTATTATTCTCTCTCTCCC
CTCAATGGCTTGGAAATGTCATATTTCCCATCGAGTCCCTCTGTCTTTTT
AACTAATATTTCAAAAAAATAAAGAAAAGAAACTCAAAACTAAACCACAC
AACTTCTCAATATGTTCAAAAACGGTTTCGACGATCTGTTTTTGAAATTT
TTTGGGAACTGTTTTCGAATTCTTAACAAAACATACCATAAGATTTTGAG
TCTTCTTGCAGGATAAGTTCCTCATTATGCCCTGAATAGCCTTCTTTGGA
TTTTTGTGTCGCTCTATATGAATTTGCAAGTCTTTTTCAGATGGAAACGT
TTTCGGTGATTGCATTATTTAGTTGAAGTCCTAGAAAAAAACGTTCAAAC
CACAAATTCTCATAAGATTATCCCCGTTTTTGGCAGTTCATAGGGGCGAC
AATGGGCTCATCAGAGCTAAAAATACAAACAGAAAACGGTCTCGTGGGAA
TGCCGAGTAATGTCTGTTGTGTTGTGGTCGAATATCTGGCCGGTGGCGCC
TTGAAGTCATACCTCATAAAGAACAGGAGAAGGAAGCTCGCTTTCAAAGT
CGTTGTCCAGATGGCACTTGATCTTGCAAGAGGGTATATACCCGGAAAAA
AAAAATCTCTTATTTTTTTTGTCCATTAGGCCATGTTTGGCATATGGAAT
TGGAGTTTAAAAAACTTGTAAATTTTTACACCTCATTCAATTCCAATTCT
GAGGATGAGTCTTGGGTACAATGGTAAGATTGCTCCATTGTGAACTGAAG
GTTCCCAGTTCAAATATTGGAAACAATCTTTGTATACAAGGGTAAAGTTA
TGTACATCTGACGCTCTCCAGAACCCGCAATGGCGGGACGATTCATGCAC
TGGACTTCCCTTTTTATTCAATTCCAGTTCTCTCTCTCCACATCCAAACA
CTGACTTATTTTAATTTGTAGGTTGAGTTACCTCCACTCTCAGAAGATAG
TACACCGAGATGTGAAGACAGAGAATATGCTGCTGGACAAAACGCGAACA
GTGAAAATAGCTGATTTTGGGGTTGCTCGTGTGGAGGCCTCTAATCCAAA
CGACATGACCGGAGAGACCGGAACACTTGGTTATATGGCTCCTGAGGTAT
ATACTCTATCTCTTACTTATGCCTAGGCTGTCATCTTCTAGTAGTATGGC
ACACCATATTTGTCACAACTGTTAGAATACTCACCGAAATCAAGAAGTGC
CATACTACTATTGATATGTCATATTGGGTAATGGCACATCAAATTTGGTC
AAGCTATTGAAGATGCTCTAACAAGAGGAAAAAGATGGATCGAAGGCCCC

AAATGGTTGTTTTTTCCCCTAAGGTCGTGTTTGGTTGCCGGAATTGAAAT
TTTAAAGCTTGGAATTGGAATTTGAAATGACCGCATTTAGATTTTTACTT
GCGTTCATTCTGATTCCAATTCCGAACCTCCAATTCCGGGAAACCAAATG
CTACCTAAGATTTATTCATAGTGACTGAATCGAAAATGGTTACTTTACAG
TTGATGGGTTTCTCTATTTGTTACTTGGCAACAAAAATAGGCATTTTTTG
GCTTATTATGAATTGAGGAAAAAGGTGGATGAAAACCAGAAAGAGTTTTT
TCTTTCAAATATTAATTCATAGTCGAAAATGGGTAATTTGCAGGTTCTCA
ACGGAAACCCTTATAACAGAAAATGTGATGTCTACAGTTTCGGGATATGT
CTTTGGGAAATATATTGCTGCGACATGCCCTATCCTGACCTTAGTTTCTC
TGAAGTGACTTCTGCCGTTGTTCGTCAGGTAAACAAACAACAACATTTGA
TTTGACCATCTAAATTTCGATCCTTCTTTTTTTTTTAATGTTTTAATTCT
TCAATTTATAATTTTTTTTTGGGGGGGGGGTTTGGGGGGAGCAGAATTTG
AGGCCGGAAATACCGAGGTGTTGCCCGAGTTCATTGGCGAACGTGATGAA
AAGGTGTTGGGACGCTAATCCAGACAAACGGCCAGAGATGGACGAGGTGG
TTTCGATGATTGAGGCCATTGATACCTCGAAAGGGGGAGGTATGATACCG
GGGGATCAACCTCAAGGTTGTCTCTGTTTTCGCAAGCACAGAGGGCCTTG
A
>EUC04221-RA [gene]
ATGTTTAAGGGACGATCGCTAAAAGGGTACAGTGATGAATTAGAGGATTA
TCATGGGTATGTGGAAACCGATCCGACTGGTCGATATGGTCGGGTAAGTT
TTAATTTCAATCATTTGTGTGCATATATATATATGTATATGTGTATGTAT
ATATGCCTGTTCGTCTGCTTCCACTTCCACTAACTTGATTATTCATACTG
TTCATAGTCGTCTCCCATGGCCGGCAGACTTAAATAAGCAGTAAGCACCC
GTTTGGCGGATTTTTTTTATATTTCAAAACACAGTTTTCAAATTCATAAA
TTAATTGATTTTTCGCCGCTAAACTGCGGTTTCAGATTACACTTTTGCTA
GAATCCTTCTTATTTTAATGGATCAGAGACATCACCTGATGCATGAGGTT
TTGATTTTTTATAATGTTTTTTTTTTTAATGATTTGGTTCGAAGCGAGAT
ATAATTTCATATTTAAATCTATCTTTCTAATGTTACAATTGATTAAATAT
TGGAAGACTTCGAAAAAGTCATACCATGTGTGTGTATATATATTTTGGTT
ATACGTTAAGGTGTACCCAGGCCAGCTTCACCTTCTAGACGTTCCCTATA
TTTGTCTGATGCATTCATCGGCCTAGTACATTCAATTAAATTCAAGATTT
AATTCCCCGAGAGCTCCCAGAAGAGTCGAACTCTGGGGTATCTCCCCTTA
CCAGCTGGACTAACGTACAGCATATATATAATATGCTATAACTATTGCCT
TTTGCTTTTGAGTATCTACTTTGCTTAGTTTAATTCTCTACTCTTCCATT
ATTCAAGCTTAATTTATGATTAAAGTCATCCAAAATATTAAACAAACATC
ACCTAAGACACTGATTTAATATGACACGTGTACACTCTGTGATTGGTACC
TCAAAAAACATTTTTTTTAAACTTTGTAATATAATTGAATTCAACTATGT
AAACAGTGAAATGCAACTATTGTATGTATTGTGGACAGTTTGATGAAGTC
CTTGGAAAAGGGGCTATGAAAACGGTGTATAAGGCATTAGATGAGACACT
TGGGATGGAGGTGGCGTGGAGTCAGGTGAAGCTGACTGACTTCCTCCATT
CGCCGGAGGATTTAGAGCGGTTGTACTCCGAGGTACACCTCCTCAGAACA
TTGAATCACGAGTCCATCATCCGATTCTACACCTCTTGGATCAATGTTGA
TCAACGGACATTCAATTTCATCACAGAAATGTTTACTTCCGGCACCCTCA
GAGAGTAAGTGATTTTCCCCTATTCCCTTTTTCGTTACCATTGTCATATG

ATACACACACACACACACACTATTACATACACATATATATACACCCCTAT
GCCTAGACTTGTTGATCATGGAAACAGTTTTCGAAGTTTCTGAATAATCA
TAAGTGGTAATAGTTTTATAGTTATCTATATAATAGATAAGGACATATTT
TACATTTGAGGCATTTTTAACACTAATATTGAGTCATGTCTAATAATCAA
GACTACTGAACGGTTGTAGACATTTCCAACCGCAGATTTAGATTTTATTG
AGAAAATAAATAACAAACGCTGAACAAAATGTGTTTTTATGATTGACATA
ATATAGGATATTCAAGATTAATGTATTGAATTAAAATTAGTGCATTTAGG
ATATACTAGCATGCATATATATATATATACTTCAAAATAAACTTTTTACA
TTAGGTGAGGAAGTGATCGTTTATATATAAGCGAAAAAAGGGGTCACCAA
ATGGTGCCTAATCTATTATACAAAAAGAAAAAAAAATGAGATGCAACCCCA
AAAAAAACTTAAAAATAGAAGTCTTAACATTAGGTGAGGTCGAATAAAAT
AGTTTAGCCAACATGACAAATGAGTTAGATACTAACTGGAACAAAAGGTG
TTGTTTGGTTAGGTGGCGTTTGGTAGTGTTTCTGTTCTGTTTCTACTTCC
AATAAATTTGGATGGGATGGGATGGAATAGCATGTTTGGTAACGACCATT
ATTCTGAAATTGTTTCTTTTAATTTATGAGAATAGAAACAAGAAACACGT
TTGGTTGCAACATTTTTTTTTCCACTTTAAATGTATGTTGGTCAGCGTGG
TGGTAGTGGCCGGTGGGATGGTAGTGAGAATAGTTTTGTGGTTGTGAATA
AGAATCAACTTTTTTTGTTTCTCAGATTAAGGAATTTAGGAAACAATCCT
AAATTACTCCTAAAATTTTGGGAACAAAAACATATTTACCAAAGACATTT
CTATACATTAATCCTAATATCTTGGGAGCGAGAACAAAAATTGAGGAACA
AAACAGCAACCAAAAGGGACCATTGTTTTGCTTCTGTTCTCAGATAACTT
GGAATAGAACCAAAAAAAATAATAATAATAAAATCCAATTAAATTTTGTT
CCCAGGAATTTCTCCAAATAATTGGAACAAAAAACTATTTATTTTCTAAG
TTCTTCAATACTTGGAAGAACAGGAACGAAAACACTAAGGTGGCGTTTGG
TAACCATTTTTGTCCTCATCCCAGCAGTGGGATCGGAATGTGATGAGAAT
TATGTTTCTTTCTTAGGAGTAGAATCGCGTTTGATAACGATAATTTTTTT
TTTTTTTNCGACAATTTTTTTTTTTTTTTTTTTTTTGTATTGGGAACAGAA
TCACGTTTGGTAACGATTTTATCTTAAAATAAAATAAAATAAAAAATTAT
TTTGAGAACATTTTTTCTATTGTTTTGGCCGGACGACGGCGGTGGCGTCG
GCCGGCCAGAGGTGACCGGCGACGGTTGCCGAAGATGGCTGGCGGCGGAC
GGTGGGCCGGCTGGCGGCGGATGAACAGTGTCGGTGGTTTTTGGGAATGG
GATAAGAATAAGGAGAAACAACTTTTTTTTGTTTCTCATTTCTTAAGAAA
AAAATGGGCACAATTCTCAAATGGGAATGGAATGGAAACAGGAACAAAAA
TTACCAAACGCTATTTCCGAACAGAAATCCCAAAAATCTGGGAATAGGAT
GAGAATTTCTGGGATGAAAATGGTTATCAAACGGAGCCTAAATAAACGCC
ACCGAAATGTATTCTATACAATAGTTGTTTAACAATTAATTATTAAATAA
AACTGTGATGTGATGCAGATACAGGAAGAAATATAAGCAAGTCGATATTA
GAGCAGTTAAGATTTGGGGTCGCCAAATCCTGGCAGGTCTTGTTTATCTG
CATGGCCATGATCCACCGGTTATACATAGAGACCTCAAGTGCGATAACAT
CTTTGTCAATGGCCATCTCGGACAAGTTAAACTTGGTGATCTAGGATTGG
CAACGATTCTCCGTGGTTCCCACAGAGCTCACAGTGTCATAGGTACACTC
ACAATCTCCCCTCCCAAAATTCTTCACCCAGGCACACCCTTAATTGAATT
TCGNAAAAAAAAAAAAAAAAAAAACCAGAAAGAAAGAAAAAGCAGAATTTG
TTTCATATCTGAATACCGTAAAATCTGATTTTTTTTTATCGATTTTAGGTA

CGCCCGAGTTCATGGCTCCAGAACTCTACGAAGAGAATTACAATGAACTT
GTCGATGTCTACTCATTTGGCATGTGTATTCTTGAGGTGCTTACTGGTGA
ATATCCATACAGTGAATGTGCCAATCCAGCTCAAATATACAAGAAAGTTA
CTTTGGTGAGTATTTATTTATTTATTTTTCGGGTGGTTGGGTGAAGGAGG
GCTTGCTGCAAGTAGATATACACTACAGTTGATCAAATCTGGCATTAAAT
ACCTTTTTTTGGGGGGATTCAGGGTAAGAAGCCGAGGGCGTTCTACAAGG
TACAAGACTTGGAAGCTCAAAGATTCATTGGAAAATGCCTAGAGACTGCT
TCTAAGAGATTATCAGCTAAAGATCTTTTGCTCGATCCCTTTTTAGCGTT
CGATGAAGATGATCCATTGCTTGACTTGAAGATCGGTCATCCAAAGCCCT
TTTTAAACATCGATATTGGAGTTGAGGAGCTGAGATTGAACGAGTACAGA
CCGAAAACCAACATGACTATCACGGGGAAGTTGAATCCTGAAGATGATAC
CATCTTCCTTAAAGTGCAGATTGCTGATGAAGAAGGTATTTTGGGTTTAT
ATAATCCAAAAGATTAATTTGACAAAAAATATACTTTTTTACTCAAAAGC
ACGAGTGCAAAATGAAAAGTATATTTCATATTGTACAGAGGTTGTGTCGG
TTTTATTCGTACTTAATCCAAACAGGCTTAAGTGGAGAGGTTATAGTCCA
TCAGTTTCTTGAGTGTTTTTTTATTTTTTTGTTTAAAAAAATACATATAA
GTATTTAAATTCATGTTTTTTACTTTTTATTTTTTATTTTAGAAGTGTTT
TTGTTAAAAGTGCGAAACAAGCCTACTTTTAAAAATTTAATATTTTTTTG
CTTTTGAACAAAAAAAAATATTTTAAAAAAAGCGTAACCAAACGGTACCTT
ATTGTTTTTATTTTTATTTGTTTTTCTTCAGAAACAAAACCATGGGAACA
AAAAAAATCTGTGTTTTCTGTTCTCCAATTTTTAGAACATTTTTTGGGGA
ACAGAAATGCCAAATGAACGCCACTAAAGACTCAACTTGATTTGGGTTGT
TCTTGCAGGTACGGTTAGAAATGTATATTTTCCATTTGACATCTTAAGTG
ATAATCCTCTAGATGTCGCCACTGAAATGGTGAAGGAGTTAGAGATAACT
GACTGGGAACCAACCGAAATTGCAAACATGATCGAGGCAGAGATATCGGG
TTTGGTTCCAAATTGGAGGAAATTAGATCGTTCTCAATCGACCCCACAACC
ATATAATGAGTCACGTAGAAGACGACGACGACGATATTGATCACATTCAC
CACCACCACCTTCACTCTCCCTCCACTTCTTCATCATCCCAAGTCTCGTT
CTCGGGTTTAATAGCTTCCCATGAAACAGATGACAGGACCCAAATCCGCG
ATTGGCACCAAGGTAAAAAAAAAAAAAAAAAGGAATTCTCATTATATAGCC
TTTTTTTTTTTCGGTTCTCAATTTTTTTTTTTTTAGTAACTTATTTTTTC
AGTTTTTACCAATATTTTAGTTAATTTTAACTATGATATCACAAACAAAA
TTAACATCTATTTGACATCAACTCGATAAATTTAATCTAGTTGATATTAG
CTGAAGAACTTGTTGAATCCCACATCGCTTGTGTTACCTAATCTTGAGCT
ATATATAAAGTCTAGCATCGCTTGTGTTACTTATCTTGAGCTATATACAA
AGTCTAGGGTAGTCCTCATCCTGTATGTCTTTAGAGAGCCCACTGGATTT
TTTACAGAACTAAAAACTTATTTGAGGGACTTAAGGAATAAATAAATAAA
ACGAGGATAATAAATTATAAATAATTTATTTCAAGGACAATTTAATAATT
TTCCTGATGAAAGAATGACGACTGAGCAAATGAAGAAAAAGCCAATTTAC
GAGTTTTGAGCCTTTTTCTTTATTATTATTATTATTTTTTTTTTTAATAT
TACTTACGTTTGATTGTACATAATAATGCGTGGTATTGCAGAGGACTTGT
TTGATGATAGCAGCTCTCATGGCTCGTCGCATTCTGGAAATTATTCGAAC
ATGTATTACCATTACAGAGACGAACATGAACAGATCACAAGTCCCAGGAT
ACGAAATCACCACCACCACAACTGTACGAGGTTTTGCCCAGGAGAAAATT

CAAGAACAACAGGGCATTTAATTGCAAGAAATTGCTATAATCAGTGCAAG
GCATTGGTGCTCGAGTCGCAAGGTGAGAAATTGTTGAAGGACAGGCGGGT
ATTAACAAGGAATAGATCATTGGTTGATATGAGAAGCCAATTGCTCCACC
GGTCACTGGTGGAGGAGATAAGTAAGAGGCGGTTGTTCAAGACTGTTGGG
GCGGTGGAGAATATTGGGTTTCAGGCACCTTGTGAGGTTTCCGACTCTCA
TCAGCCGGTAGGTGGTGGTGTTTATTCAATGAGGAGCAACAGAGATGGGA
AGAAACAAGGGCATAAAGGAAGAAGAGTTTGA
>EUC20242-RA [gene]
ATGGAGAGAACAACCTCAAACAAACCGAAATTCCCACTCTGTTTCAATCC
TTTCAGGATCCTGAAATCGAGGAACAAACCGCCGTCGATCCCTTCTTCTT
CAAGAACCCAGTTCAATTCCGACACGGAGAGCATGGAGAGGAAGAGATTT
GACAGCCTGGATTCATGGTCGATGATTCTGGAGTCCGAGAATGTCGAAAC
GTGGGAAACGTCGAAGGATGATCAGGAGGACTGGACGGCGGATCTGTCGC
AGCTGTTCATCGGAAATAAATTCGCCTCCGGCGCACATAGCCGGATTTAC
AGAGGGATATATAAGCAGAGGGCGGTGGCGGTGAAAATGGTGAGGATTCC
GACACACAAGGAGGAGACAAGGGCCATGCTCGAACAGCAGTTTAAGTCTG
AGGTTGCCTTGCTATCTCGCCTCTATCACCCCAATATAGTTCAGGTTAGC
TCAATTTTGAAGACAACCCAATTCAGTTTCTTGAGTTAATGACACTTTAT
CCCCTGTAGTTTAACCAAATCGCACTTTTATCCTTATAGAGTATTTTCGT
AATCACTTGTCCCGTGATCGGTTATTTAGACTAAAATGCAATGATTTCGT
TTTTCGTTAAAATTAACGGTCAAATCTAAAAAAATATCAAAATTAATAAAT
CATTTAACATTTATATCCCAAATTAGGTCGGGTAATAGTCATCTGTCCTC
ATTTTTTTCTTTCCTTTATTTTTTGGGCCAAGTCAGATGAGGTATAAGGA
TAAATTTACAATCTATTAATAATTAACCATTGCACTTACATCTGAATCAC
GGCAAGTAAGTGCTGAGTAAAATACTGTATGGATTTGAAGTGTAATTCGG
TCAAACTAAAAAGAGGCAAAATGTAATTAACCCTTCAATTTTTTAATTCT
ATATATATATATTGAATTATCCATGTCTATGTTAATGTTTTTTTTTTTTG
TTTTTTGTTATTTAGAGGGTGAAATGTTGATTTTTTAAAAATTGGAGGGG
GGCTTTTTGAATTTTCAAGATTGGCTATGTTGATGCTTAATTTTGTAAAT
TAGAGGCAGATTTAAATTTTGATTTCGGCGGGATTAAAAATAATTGGTGG
TTTTAAGATGATTTTATGTAAATTATCGAGGTTTAAAATGGAAAATTTAT
ATAGGCCCCCGTTTGTTTAGTGTTTCCGCTCCCTTATTTATGCTTTTGCT
CCTAGAATTTTGAAATTAAGGTATAGAAATGTGTTTGGCAAACATGTTTT
TGTTCCTAAATATTTTCAAAATTTTGGGAGTAATTCCAAAATTTCTTGAT
TCCTATTCATTCTCACAAAACTATTTTCAATCTATTTTATAAGGATAAAT
GTCAAGAAGCACCACCACGCCGGCCACATCTTCATCTTCTTCTTTTATCT
CCTCTCTCTATATGACGCACACACATAAATGATCCAAATTTAGTTGTAAA
CAATTTCATTTTTTTTGTTTTCATTATGAGAAATTATTAAAATATTTAAA
GATAATTATTGTTATCAAACGCGATTCTGTTATCAGAATAATCTAGAATG
AAAACAAAATTTTCATCCTATTTTATTGGGAGTAGAAACAGAAAAACAAA
AACGTTAATCAAACATCACTTTAGGAACAATTCTAGAAATGGAGACAATA
ATCTTGGAACATAAACCCCTAGTATTTGTGATTTTGGGACTAAGAACAGG
TATTTGTTTAGTGTTTTTATTCCAAAAAAATAAAAATAAAAAAAAATAAA
AAAATGAGAACAATTCAATTAAAAAATTCATGGGGCCTAAAATGTTTAAA

GTAAAGGTTGTAACGCCTCATGTGACCCACTGGTCCAGTACCTTTTGGAC
TGAGTTCACGTGACCTAAAATGCTTAATTTAATGTACTAATAGTAGCCCA
TATAATATCATCCCCCACTAAAAAATGTGATGTTCTCGTCGTGTTGATCC
AATTTATTCTCTTGGTTGATGGTGTAAATAACAGCATATAAGTGGACACA
TAACAAACCCCATAAGCGGACACGAGGTACCTACCCATCCCAGGGCCTCT
CCCCCCTTCAGAAACTTTACTCCTCTATCAACACCGCCTTGGTGCTGAAT
CTGCACTAGAGTTAGCTCCGACACCATCTGTAACGCCCCATGCAACCCAC
TAGCCCAATATCATTTGAACCGAGTTCACATGATCCAAAATGCTTAAACC
AAGACACTACTAGTAACTCACTTGTACTTTATATATCAAACAATATTTTC
TAATTTTCCGATGTGAAATCTCACAAAGGTGGTAGTAGTAGCCCACATTC
ATTTTTTCTAGTACAAATAATTAGGAGAGAGAATTTTGAACACTGACCTC
TCACCAGAGAGGTACACTAGATAACCGCTAAATTACAAACTTTGATATAG
TAGTAGCCCATATTCATGATATTTTATATATCAGATAATTTTTCTAATAC
TTCCATTGGAATGTCACGGTTCATTAACCCTAACTAAATGTTGCTATACA
TGCATATATACACACAGTTCATATACATATATACATTATACATATTCATA
TATATACACAGTTCATTGATCTTAACTAAGTGTTGCTATACATATATGTA
TGTATATATGTATATACAGTTCATCGCAGCATGTAAGAAGCCTCCAGTAT
AAGTTAGCACAATCTTTTATAACATTTTCGATGTGAGATGTCACAAAGGC
CGGTACTTTATACACACATATATACATACACAGTTCATTAACCTTAACTA
AATGTTGCTAAACATGCATATGTACACACAGTTTATATACATATATATAT
ATACACACACAGTTCATATATTGATCTTAACTAAATGTTGCTATACATAC
ATATGTACATATGTATGTATATACAGTTCATTGCAGCATGTAAGAAGCCT
CCAGTATACTGCATCATCACCGAATACATGTCCCAAGGAACCCTAAGAAT
GTACCTGAACAAGAAGGAACCCTACTCTCTCTCTACAGAAACCATTCTCC
GACTTGCCCTCGACATCTCCCGCGGCATGGAGTACCTTCACTCCCAAGGC
GTCATCCACAGGGACCTTAAATCCAACAATCTTCTCCTCAACGACGACAT
GCGCGTTAAGGTTGCCGATTTTGGGACCTCCTGTCTGGAAACGCAGACCC
AGGAATCGAAGGGGAACATGGGGACTTACCGGTGGATGGCGCCGGAGATG
GTGAAGGAGAAGCCGTATACCAGGAAAGTCGATGTTTATAGCTTTGGGAT
TGTGCTTTGGGAGTTGACGACGGCGTTGCTTCCGTTTCAGGGGATGACTC
CGGTGCAGGCGGCGTTCGCCGTCGCCGAGAAGGTTAGTTTTTACTTTTGC
TCTTTCTTTTTTTAAATATATTATATTTTTATATATTTATAATTTTGGTC
CCTGAGGTTTGTCGAATACAAATTTAGTCCTTCAAATTTTATATTTGTTT
TTATTTAAATGCAAAATTAAAAGAAATGGCTAATAATTTTATATGAAAAA
TAAGTTTTGATGACCACGTGGCTTTCTAATGAGAATATTTTCGTCGGATG
AAATGACAAAATATTCTTATTATAAGGCAAGTGCTGAGCATAACCCTTTT
TACAGTCAAAATTATTAATACCGTTTTAATTTTGCAGGATTGAAATAAAT
ATTAAATTTAAAATCAAATATGTAATTTGACAATCCTCAAAGTCCAGGTA
GACATCCAGGATCAAAAACATAATTTTTTTTCCTTTTTTTAATAATTTAG
ACAACATTTGATTGCATTTTTAAGTATTTTTTGTTTGCCTACTAAAAAAA
TTCTTTAAAAAATTAAAAGAATAAAAATACCACTTTTGAAAAGATAAAAA
CATTTGTTTTTTATTATTTTAATTTTTATATTTTAGAAGATCAAAAAAAG
AATTACACTTAATATTTTTATATTAATAGTTCATTTAACTAGATGAGTAT
TTTTTCTAATTAATTTTATTACTACGAAAACAATTAAAAAAAAATTATTAC

CAAATGATACATTATTTATAATTTTATATCCCTACTTTTTTTTTTAATTT
TTGTTGATATTAATTATGTTGAATTTGTGTTTGATATCCTAGTTAACATT
AACTAAAATATTAATAAAAATTAAAAAATATTTTTGTAAGGACTAAAAAA
TAAATCGATCTAGAAACATGATTACAAAAAATGATTATATAATTATAAAC
CCATTTTTTTTTTTCAAAAACAAAAACAAAGGTAAAAACTACAATACAGC
TACAGCCCCATACAGCCCACCAAAGTTACAAGGTTGAACTAAAGAAGTTT
CAATGTTTTAGAAGAACTTGAAACTTATAACTTGTAACTTTCCAAAAAGA
CTTGTAATTTCCTTCAGTCGACCTTGTAATTTCGCCGGTTGTATGGGGAT
GTAGGTTGTATTGTAGTTATCTACAAAAACAAAAATTGCAACATGATCAG
AAAACCTCTATTCTTGTCAGAATGAACGACCGCCACTTCCAGCCAGTTGC
CAACCAGCACTGGCTCACCTCATAAAGCGATGTTGGGCATTAAATCCCTC
AAAAAGGCCTGATTTTAGCGACATCGTATCAGCTCTGGAGAAGTACGATG
AGTGCGTGAAAGAGGGTTTGCCTCTCACTCTACATTCAGGTCTCGTAAGT
AAAAATGCAATCCTCGAACGGTTAAAAGGATGTGTTTTGATGAATTCTTC
AGTACTTGTACATGCTTAA
>EUC13910-RA [gene]
ATGCATCGAATACCTCAATTCTTCAATCACAGTAAAAGTTCCACAAGCTC
GAAGCACAGGCGAACCGCCAGTGCCAAACCCAAGCTCAAGCGCTCAAACG
CAGCGCGGCATATCGACTACGAGGCCCCCTCAACTTCGTCCTCTGATCAA
TCTTCATCCGCACTGCGAGCTCGATCACTGGGCATATTGCCTTCCAACGA
TCGCACCAGCTTCAGGGTCGAAGGAAATGAAGGAGAACTCGAAGTTATCT
GCCGGAGATTGGGGCTTTCGGGGCCGGAAGAGTTCGAGATCCCGGCTGCG
GCGTGGGAAGCGATGAAGTCCCGCTCTTCATCGGATGTTCTCCCGTTGTC
TGGGGAGCTTCGATTTGATAGCCTTAACGTAGAAGTACAAGTAGAAAATG
AAGGTCCTAGTGAAAACCTAGATGGGGTTTTAGATAGTTTGAGGGCTTGT
AACGTAAACGATCTTCAAATTGAAGTCGTTCCGTTGAATGACTGCAAACC
TGTTGGCGGAGATGGTGATGATGCTGATGAATTGGTGAATAGCTTAGAGG
ATAGTGTTAGGGTAAGTAGTTTGATTGTGAATAAGAATGAGCTTGTTGAA
ACTGCTGGATCTGGAATTAGGGTTCAGCATTCCAATGGTGGAAGAGGAAT
CAAAGGTGATAGGCCACCAACTCTTGAACCTCCGCCATCAATGTTGCTAC
CTGTAATAGATAATGGGTGTTCAACTTGGGATCTTTTGAGATCCTTTGCT
CCGGGCAGCGATATAATTTCAGGGTTTGAAATTCATGGAGGTATTGCTTC
TGATGATGAACAAGATGAGGGCAATGAAGAGAGTGGTAACAGGGATGGAG
AGCATAATGATGTGAGGGTAGGAGGGCCTTGTGTTCTTTATGAGTCGTGT
TCGTTTACAACTAGTAACGATGATGATTCATCTAGTACTACAACAGATCC
TGCGTCGAATAATATATCACCAAACGTAAGATCTAAGCGTAATATAACAG
ATTGGGAGAAAGGTGAGCTTCTTGGACGTGGGACGTTTGGATCTGTATAT
GAAGGAATTGACGAGTAAGTACAGTTCATGGGGTTTATTTTTCAATATTC
ACTGATATGATGCTCAAACTTTTAGTGACATTTTTTATGACTTTCCCGTT
TTTAGTATTTTGACCCGATTTTGTTGCCTTGGAGTCTCTATTTTGATGAA
TTAATCTCCCTCCTCCTAACACCCTCCCCAAAAAAAGATAGAGGAAATAA
ATAAGGAAATGGAGAGAGATTGGCTATGAGTTTATGTGGCCATTCGGAAA
TTGGAAGTAGGTAAAATTATAATTCATCTATTTTTCAAGATTTCTGAAAA
CCGTGCCAAACTAAGGCTGTTTTATTTTGTTATCCTATTTCCCTTTTGAG

GCTTGATTTTTCGTTTTTATTTTTAAATAAAATTCTATAGAATATAGATA
GTAACTAGCTCTATTGAAATAGAAGATGAATGCATTTTTCCGATAGAATT
AATCCAATAGGAAAGCGATATTTTTTACTTCTAAAACCAAGTCTGACTGA
ACTATGTTGATCTTTACTGTATTCATGCCCAAGAGGGCGAGAATACTAAT
TACTGATTTGTTGTCGCAGTAAAGTCTCATCTCTCCATCTCACTTAATCT
TCAGATCTTCCAAGATAATCTTCAACCATAGTAATTCACAAACTCTTTGA
GCCATTGCTTGGAATTCTGCTTCTGCACTAGATCTTGCTATCGTATTTTG
TTTCTTACTTTTCCAAGTTACAAGATTTCCTCCAAGAAAGGTGCAATAGC
CTGAGGTCGAACTTCTGTCAATTACTGATCCAGAATAATCAGCGGCTGTA
TAAGTTTCAAGTATCATTCCATCACCCCTTTTAAATAAAATTCCTTTCCT
AGGACTTCCTTTTAGGTACTGCATGACTCTATCGACCGCTTGCAAATGAA
TCTCCTTTGAATTGTGCATAAACTGACTGATCGCACTAACAACGTAGGCT
ATATTTGGCCTAGTCTAAGAGAGATAAATGAGTCTTCCTACAAGACGTTG
ATACATCTCTCTATTCACTGTAATATCTTCTTCAGCCTCTCCAAGTTTAT
GATTAGGCTCTATTGGTGTACTCACCGATTTGCACGCCAATTTCCCTGTC
TCTCTAAGCAAGTCTGTAATGTACTTCTATGGAGAAATGAAGATCCTTTG
TTTGGAATGTGCAATCTCAATTCCAAGGAAGTACTTCAACTTCCCTAGTT
CTTTGATCTCAAATTCACTTGCTAACCGTAGTCTAAGGGCTTGTCTTTCC
TTCTCATTATTTCCTGTCGCAATGATGTCGTCAACATATACTTGGAGTGC
AGTAACTCCCCCTGAACTTGAGTGTTTGACAAACAAGGTATGATCTCCCT
GGCTTTGTTTATATCCCATCGACACCATTACACTTTCGAACCTTCCAAAC
CATACCCTCGGAGACTGCTTAAGCCCATACAAAGCTTTCTTCAATCTACA
AACCCGGTTCTTCCTCAAACTCCTGCAATATCTTGGTGGGATTTTCATAT
ATATTTCTTCTTCAAGATCTCCATGTAAAAATGCATTTTTTAAAACAATC
TGTTGCAAATCCCAACCATAATTGGCTGCCAAAGACAACAAGACTTTGAA
TGTATTCATTTTAGCAACTGGATCAAATGCTTCTTGGTAATTCACACCGT
ATGTCTGAGTATACCCTTTGGCCACTAATCTTGCTTTATACCTCTCCAAA
GTACCATTTGCTTTATATTTCACTGTGTAGATCCACTTGCACCCCCACCGG
TTTCTTTCCCGCATAGATAAATCTACTATCTCCCAAGTTTTATTCTTTTC
TAAAGCTTCTATCTCTACATTCATAGTTTGTTTCTATTTTTCATTAGATA
GTGCTTCAAGTGGTAGGAATGAGAATACTGTTTAAGCTGATTAGGAAGGT
CCTATGAGATGGTGAAAATCTTCTAAATGTGACAACATGAAAGAGTGGGT
AACGGGGATGTTTGGTGCATTCTCTAGTTCCCTTCCTAAGGACAATGGGC
AAATCTTGATTTAGGTCACTACTCAAGTGAGGTTCAGGCTCATTTAATAA
AGGAGAATCAAAAGAAGGATTGGGGTTAGAAATTGTTACCTCAATTCCAG
AATTCAGTTATGACTCTTGGGCCTACATAAATTCAGGAACCGGAGGTGCT
ATCTACCTTGAATATACTTGACCAAATAATTTCTTGTTATCTCGGGCAGG
AGTAGACTCAAGATGCGTAGCAGGGCTTGACAAGGAAGGAAGAACAATGA
GATCCTCCTTATCTTCTATTACTAGATTCTCCCCCTGAAGATAAAGGGTG
GTGAAGTATGACTGACTCTCAAAAAAATGTAACATCTTCTGAGACAAATAA
TTTTTTTGGAGGTGGATGATAACATTTATAACCTTTTTGGGTAGAGGAGT
ATTCTACAAAGATACACTTGATTGCTTTGTGATCTAACTTTCCATTGTTT
TGACTGGGAACATGAACGAAGGATATACAACCGAATATTCTTGGTGTCGA
GTGGTTGGTAATTTGGAGATTGGGATATGACTCTGAGAGCACTTCCATAG

GACTTTTGTAACCTAGGACTCTAGAAGGCAGAAGGTTTATGAGATGTGTT
GCTGTTAAAACAGCCTCCCTCTAATAACTTAGGCACATTATTTTGGAACA
AAAATGCTCGGGTTGTATCAAGTAAATGACCATTTTTCCTCTCAGCAACT
CCATTTTGTTGTGGAGTGCTAACACAGGATGATTCATGAATTATCCCTTG
CTGTTGAAAGTATGAGGTTAAAATCTGATTGAAATAATCTCTAGCATTGT
CTGACCTAAACCTTTTGGTTTTTACTCCAAATTGATTTTGAACCATGGCA
TGAAAGGTAGTATAGTCCGTTCTCTTCCTTAGCACGTCCAATCATTTTTC
CCAAAGCCTGGTCCTGAAATACAAAATAGGTAGGATGAAAAATCACACTG
CAGGACATGTCTTGAGTTAGTTTTTTTTATAGAAATCAGATTCGTGGACA
AATTTGGTACATGAAGTACATTTTTCAATGTGAGTGATGGGCTAATCTTA
ATATCTCCTATTCCTGCTAAAGTGATTAATGACCCATTGGCAGTGGCAAT
CTTCCTAGTATTAGGACAAGGGGTATAGGTGCTGAATTTATGTGATGAAT
GTGTCACGTGATTAGTTGCCCCTGAGTCTATGATCCATGAATTGACAAAG
GTTCTATCTGAAACATTAAATCTAATGGAAATAGGAAATTTACCTGAATG
TACCAAAGAGCAAGTACCTGAGGGTTTCTCAAGACCATAAGCGATCCCCC
TAAGAAGGTTCTCCTTCAGTCCCATAGCATCAAAGCTATCACAAACCTCA
TCATATGATGTGAGGAAATCTTGCCCATTAGACAAGAGTACTTCACTCAT
TTTGGCATCATGGTGCTTAGCATCAAACAGAGATCCTTCAGGAGTTGAAT
TTGCCATGACTGAATGTCGATTTGCAAGTCAACGAACAACGTAAAATAAG
ATTAAGAGAAAATTGAAAATCCTGAAATTTGCTTGAAGTAGAGCTTCAAT
CGAGCTTTAGTCGTTCAAGCAAAAAACCACCACCGATGGTTGCAGCTGGT
TCGATCTGAGTTTCACGAGGATTTGGGTAGAAAAAATTGGCGATAAAGGC
CAGAGGTATGGTCTGCTATGAAGGCCAAAACAGATGAAGCGATGATGGTC
AAGGTTTCGCGATCTAGTTCGCAATTTGATGGCGATTCTGCTTTGCGATT
CAGGAGGTTATGGTTTCGCGATTTTAGTTCGCGATCTGGTAGCGACAATG
GATCTGGGTTTCATGAGTTGAAGGTGGCGGTGTTGGGTAGGATTTCACGA
TTTGACGGCGGCGGCGAAAGGTTGGAAGTTTCGCGATCTGGTTCACGGCG
GCTGGTTAGGGTTTTGGAGTCACGATCTGGTTCGGCGAAGCTAGGGTTTG
TTTTGGAGTCGTGATCTAGTTTCGAAGGGTTTCACGGAGTCGCGATCTGA
TTCAACGGCTAGGGTTTCGTATGTGGGTGAGTCGCAATCTGGTTCGCGAT
TCTGGTAGCAATAGCGGTGGCCAAAGCTTAGAAATTTTCATTGGTAATGA
AGGCTGATTGAAGAAGAAGAGTTACACGATGATTAAGGCCGAAAAAGAAA
GAGAAGAAGAGGGCGATTAAGGCTAGAGAAAAAACCAAGCATCGAGCCTT
ACAACTCTGGTACCATGTAGAAGAAAAGGGAAGAGGAAATTCTTTTCATT
AATCTTGATATGTATAACAGTGGGTTAATATAGATAACCCTAGCTGCAAA
AAAGGAAAATAAACAGACTCCCCTAATTAGGGGATTATTCTAGAAATACA
ATGAATCTGCCTAATCCTAGAAATTTACAGCTGTAAATTAACTTTCCAAA
AGAACTAATTTCCTTATTTCTCAACATGTCCTTTTTTCTTTTTCTTTTTG
GTTGTTTGAATTGCAATTTTCATATTTTTACTGATTGATTCTATATAAAA
AATGGCTCTGTATTTTATCTCCGTCCTGATTTATTATATAGATTTTTTCA
CCATCTTTTCCGATTTTTGGGGGGGTTTCAGTGGTGGATTCTTTTTTGCC
GTCAAAGAAGTTTCTTTGCTTGATCAAGGGGAGCTAGGGAGGCAATCTAT
TCATCAGCTTGAACAGGTAAGATGGGTTGAAAATGCATGCTTAATGTATC
ACTTCTCTGCCCGCTCTAATAAGTTACCCTTTTGTCATCTCCTGTAACTG

TGTACAGGAAATTGCTCTTCTAAGTCAGTTTGAACACGAGAACATAGTCC
AATATTACGGCACTGATAAGGTATCATCTTATTGCCTTCTCTTACACTTT
TTGACTGATGCTCTAGCATAATCTTCCATATGTTTAAAGTTTGGTAGCAC
TTTTCTTAAGCAAATTGCAAAATAATTTTTTTGGGGACAAAATGTGCTTT
GACTTAAAAGCAAAAGCAAGAAATCAAAAACCTGTTTTATAGTGTGTATA
GTGTAGATTGTGTTATCCCTGTAAAAGATTTGTTAATCCAAAAGAATTTC
ATAGATGATTGCTATATTTTGGGTGAAGAAATTCTAGTCCATTGATCTTT
TAACATATAAAAATCAGTAAAACATAAAGAAACTATTTTTTTGGTTTAGA
AAAAAACGTAAACTCATTTTTTTTTTACTGCGAAACAAATGCTTCTTATT
TCCTAATCGGTGGTTTCTGCAGGCTGAATCAAAACTCTTCATCTTCCTCG
AGCTGGTGCCCAAAGGTTCCCTTTTACGTCTCTATCAGAAATATACTCTT
CGAGATTCTCAAGTCTCTTCCTATACGAGACAAATTTTGCATGGTTTGAA
GTATCTGCATGACCGCAAAGTGGTACACAGGTGATTAAACTAAATAACAT
TCTTTCTTCACTCAACCCCCTCTTATTGTGCAAAAATGAATAGCTGATAG
GTTATTTCCAATTCATGAAAAATCGATTTGTTAATTGTGTGCGTGCGCAC
ACACAATCTCTCTCTCTCTCTCTCTCTATATATATATATATATATATATA
TTAATAAATCATGGTAGATTTAAATGCAAAGATATTTTAACGGTTATGAA
TGGTTGAGATTGAACTTAATTTTGGGTTAGAAAAAATTATTTTTTACACA
TTACACCACCTAAAACTTCTCTTTGTCACTCTCTCTACTGTTCTCTTAAC
TATCCATTTCCCTTCCATCTAAACCAACTATCCCACTAGCCTTAAACTTG
TGATAGAGCCCTCTCCCTTCCATCTAAAATTATAAGTGGCTTTTGGCCCG
GAGCATCACTACTATTCTGTAAAACAGAAAGTACAGAGCATATTTTGAGA
ACACATTTTCAAATGATAATTAAAAGGCATTTTTAAGGTGGATCCTCTCA
TTCTCCTTCTCTCTATCTTCAATGCATAGGTGTCAAAGCCCTCCACTTCT
GTCTTATTTATTCTATAATCTCTCACTTCTTTCTTTTTAATTCAAAAGAG
CCTAAATGCCACTACCCCTATTTCTCTTCTTTCCTATTTTCCTTTATATT
ATAATTTTTTTTTTTTTTTTTTAAAGAATCAAATGAAAACTATTTTAATC
GTTTTTGGGGAGAAAAGAATACCATGTCTACGTTATAATTATTTTTTTTG
GCCGAGCAATTTATGTATAGTGATATAAATCTATTCTATAAAACATAATG
GAGGATATTTTGAAAACACATTTTCAAAAGAATTTTTTAAAGCACATTTT
TAAGGGGGTCTCTCATTCTCCTTCTCTCTATCTTCATTGCACATGTTTCA
AAGACTTCCACTTTTGTGTTATTTATTTATTCTTCATTTCCCTCTTCTTT
GTTTTTAATCCAGAAGAGTCCGAATGCCATTACCCCCACATCTCTCTTTT
TCTTTTTCTTGTTTTTCTATATATATATATATATAAATAAAAATAAAATA
CATATCCATTTTAATATTCTGAGGGAGAAAAATATCGTGTCTGCATTAAT
TTTGGCAGAGCAATTTATGCATACTGATATACATATCTAATATCCATACA
ATAAAATAAGAAGGGGGTACAAATTTAAAATATATTTTCAAAATGTATCA
TTGTATTCAATTTTTTTGCTTTAAATCTTCGAGTTACTAAAAAGTTATATT
TTTGTAAATTAGTGATCCTTTTATGTTATTGTCTAAATATTAAATTTGAT
TTATTTATTTATTCAAATTAGAGTTATCTTTTTCAGTCTGTGCCTTCAAG
CACGATTGCACGAGTTACATGCTAGTGTGTTTATATATATATATCGATAA
AGTTGAGTGTCTGAAATATTATTTCCTCTCTTTCTTGCATTTAGGGATAT
TAAATGTGCTAATATATTGGTGCATACTACCGGTTCAGTAAAACTTGCAG
ATTTTGGGTTGGCCAAAGTAAGGCCTCCTGCCCTTCTTCCTCTTCTGTTG

TTATGATGACAATTTCCTTGTTCTTTTTGGTTTTTATGTGATTTAATGCA
TATCAAAATTTCCCTAAATTTGGATCTCTTGGGCAGGCTACCAAGTTGAA
TGACATAAAATCTTGCCAGGGAACTGCCTTCTGGATGGCTCCGGAGGTAT
GCACTTACCATCGTTTTTATATTTTACCTAGAAATTCTTTTGGATTACAC
CATCTTTTATATTGGATGATACTAGGGCTATAAATGAGCCGAGCCGAGTA
GTGGATGTACATGCTTATTTATTTAAGTTACAAGTTAAACGAGCTGAGCA
CGAGCTCAAACCGAGCTTTGAACTGACTGTTCGAACTTGGCTCAGCTAAA
AAAATGGGAGCTTGAGCTCGAAAAACTCGTTTAAGAAGCTCGAGCTCGAA
TAACTTGTTTAATAACCTGGAGCTCGAGCTCGAATAGCTCGTTTAAAAAG
CTCGAGCTTGAGCTCGAATTGCTCGATATGAGCTAAAAAATCAATTAAAA
CTCATTTTGTCTGCTAAATATAAATCCAAATATATTACAAATGACACAAA
ATAAATACAAACAAATCAATACAAATTAAAAAACCCAAAAATACATCAAA
ACATCAAGTAATGACAACATAAAATACCAGTCCAAACCATTACAATACAA
ATTACAAAACCCAAAAATACATCAAATCATCAACCCGATTATCCTCTTGG
CTATGGACATCTTCTTCTTCATGGTCATGAAGAAAAACTGGAAATGATGT
TGAATCTTTCAATGATATGAATCATAATCAACAATAATAATATTTAAAAA
AAAAAAACTGAAACATTAGTAATTGAAACATGAAGTAAAATTGAATCATA
ATCAACACAAACATTAGTAATTGAGACATAAAGCAATCAACAAAAACATT
AGTAATTGAAACATGAAACAAAATTGAATCAAGTCAGTATACTTGACAAC
TAAAACATTAGTAGTCAATAACAGTTCCAAGTCAGTATGCCTGACAAAAC
TTGAAGCAAAATTGAAGGAGAAGCTCCCTGGATCAAAGCTCAAGGGCAAA
CAAAACATTGAATCTCGAATTGAAACACGAGAACGAAGCATTCAGTTCGA
TTCTTGTAACATATGAGCATGATTCAAATCATAAAGAAGAAATAAGCCAA
GATAGAGAGAGAAAAAGAGAGTGTACCAGATCTGTTTGGCACAGGCAAGG
TCGGTGTCGGAGAAGGCAGAGATGGTGTCGAAGAAGGCAAAGATGGCGTT
GGAGTCGAATGGTGGCATTATGGTGAGCAGATCAGCGTTAGTTTGAGCAG
ATCGGTGATAGGGTAGTAGCTTTTCCAGTGTTGTAGACTTGTAGCTTGGC
CAGGGTTGAGCAGATTGGCGAAGTGTGAGCAGCGGCGAAGCAGGGTTGAT
CGGCGGCGAAGGCATGGTTTAGTGGGAGATCGAGATGGGGAAGAGAGTCG
ATGGAGAGGAGAGAACGAGAGATGGCTGCCTGGGAAAGTAGGAAGTAAAT
GAGAACTGAGAAGTTGAGAACCCTAGTACAATTAATAGGTATATATATAA
TTAATAAAAAAATATTTTGTGTTTGCGAACATGAAAATGAGCTTGCGAAC
AGGCTCGGCTCGTTTTGGTCTTGTTCGCGAACAACAAACGAGTAGCTCGC
GAATAAGCTCGGCTCGGCTCGTTTATTAAATGAACATTTTTAGGAGCTCG
AGCTCGACTCGTTTATTTTAATAAACGATTTCGAACAAGCTTTTTTCGAG
CTTAGTTTACAGCCCTAGATGATACGTTAACACTAGGAGGTATTATCTCT
ACATCATCCAATGCACTTTTGATTTTTACTTTTGCTCTCAAGTTTCGTCT
CTTTTTATTTGTTTATTTATTTGTTATTATAAAAAGTACTTAAAAAGTAT
TACTAAACAATGCCTTACATCTGCACATCTATCTTATACGTTTGCTTGTA
AGCTGATGTGAAAGTAGGGCCGACCGTGAGAAATCAGAGGCCCTAGGTGA
AATATAGAAACGGGGCTCCTCAATGTTAAATAATTTTTAAACATATTCAT
TTTTTAATTAAATTAAAATGTCAAAAGAAAATTGGTTTAATGTGATTGAA
CCCAGATTGATTTTTCTTTTAATTTTTTTTTTTTTTTTTTTTTTTTTAATT
TGACGTGATATTAATACAAATATTTTGAAATACAAAAATATATAGGGGTC

AATTTGAAGGGGCACCCGAGGCGACCATCTTTTTGGCCTTGCCTCCAGGC
CGGGCCTGTGTGAAAGCAGCTTTGAAACATTACAGGAGTGGTCCCAGATG
TTTCCTTATATATTCATGCATTGACTGGAGCAAGTGGCATTTGTTTAGCA
TTTCTGTTCCCAAAAATCTGTAACAGAACAGAAATATGGGAACAGTTCTT
TTCTATGCCCAGAAATTTGTTCTTAAAATCTGGGAGCGGAACAAAAATAG
ATTATTTTTTTTGTTTCTGTTCCCAGTTTGCTTGGAACAGAAAAAATAAA
CACATCGGATTTCTAGAATCATGCTTCCTAATCCATCATTAAAAATCTTC
TTCATGTTGATGCATAAACATGTCTTCAACACCCTCTCACCCACTATATC
ATCACAATTGATATTTCTAGCGATGCTTAACGACCAACCTTCCATTCAAA
CCCTTGTCTGGCTTCTCAAGTGATAGTCTCCTGGAGCTGATTTTATAGAC
CCATTAGTCATATTTGTTGCAGGTAGTTACTACTAAAAAAATAAAATCAA
ACTCAACAATAAATGCAGGTGGTTAACAGGAAGAACCAAGGGTATGGCCT
TGCAGCAGATATATGGAGCCTCGGATGCACAGTGTTGGAGATGCTGACTC
GTCAGCTTCCCTACTCCCCTTTGGAATGTGTGAGTACCCCTTCCTCGTTT
TTTTCTTATCCTCTTTTTCTGATGTAAAACGCTATTGCAAAAAATATACA
TTTTAGAGATGTTTGAGATCTATTTACTTATTTTTGTGACGACCAAATTG
ACCTAGTAATTCCTAGCAGTATTTGGATTGTTTCTGTTCCCAGATTTCTG
ATTCCCAAGAAAACATGGAACAAAAAACTGAAATCTGAGAATAATTCTGT
TCTGTTTCCCAGAAATTTGTTCTAAAATTCAGGGAGCAGAACAAAAAGTA
TATTTTTTTTTTTATTTCTGTTCCCATCTGGCTGGGCACAGAAACAGAAT
TATGGGAACAGATACAAAAGGCAAAACCGGTGAAGATAAGTGTTATTTTA
AGTTAGGAATTTCACGATTCTTCTTGTCATAAAACAGATGCAGGCGTTGT
TTAAGATTGGGGGGGGTATTCCGCCACCTGTTCCTGATTCCCTCTCCACA
GATGCACGCGATTTCATATTACAGTGCCTTCGAGTGAACCCAGCGGCTCG
TCCCACTGCTGATCAACTCTTAGGACATCCCTTCGTCAACAAACCGCTTT
CCTCTTCTTTGGCCTTTGCTTCTCCTCTTGATCCTCGCAGGAGAGTGTGA
>EUC10582-RA [gene]
ATGACACCCGAGACAGGCACTTACCGTTGGATGGCTCCGTAAGACCTCTG
ATCTTTAATACCTTAGTTTCTTGTACATGGGGTTGATTCCCTCTTTTAAT
ACATGATTTCTTGAAAGTAAAAAATAGCGGACTTCTACAGTATGTTTTTG
TACGCAGGATTCAACTTCCCATGTTTAGTACGTGAGATTAGTTTTCGTTC
CCAATCCTAGGTATTCTCTAATCCCACAAATCATGGGACTAGACAATCCC
CAAAACATGTTATTAGGTTGGATGGGGTCATGGGATTCAAAATGGGAAAA
TATTGGTAAAATAGTTGTTTTCGTTATCCGGTCACCATTTCCGTCGAAGT
TGCCAAATGTTTGGCAACATCATTGGAATATCGTTGTAACTCCGACAACT
TAATCGGCATGTAACTTGAGTTTTGGCAACGTCTTTGGAATGTTATTGGA
ACTCTGAGAACAATACTGGAACGTCACCAGTTCTTTTGTAATATTGTTGG
AATATTGTCACAAATCCGGTAATGTTATCAGAATATTGTCGGAACTCCAA
CAATTGTTACGTAATGTTGTCGGAACTCTAGTAATGTCGCCGGAATGTTG
CTGTATTTTATTAACTTATATTTGAAGGGAAATTTTCCTTTTTTTTTTTT
TTTTTTTTTTTTAAAAANAAATATTCATCATCATAATTGATATATTTTAT
TTGACAATCCATTCTATTCCAAACACGGGAATGGTAATGGTCATTCTACT
CTTGTCCTATAAAGATTCTAATTCTATACTCATTACAATCCATTGCTACG
TACCAAACGCAACATTAGTTACCAGGACTCTTCTTGGCTCATTATTATGT

ATACTAAATTATTTTAAAAGCTCAAACTATCGCATTAGCTACATTCGTTT
AATTAATAAGTTGGATGGCACTGCCATTTAGCTAGTTGACTTGGGCTAAT
ATTATGAATGCACGTCACATGAATATAAGTGTTAGAATTCCACATTGAAA
TTGTGAGAGTTCCCTAAGTAATATATATGTGAAAGGAATTACTCCACTCG
ATATCATATGAACTTAAGTTGGAACCTCCTAACATGGTATCGGAGCTAGG
TTTGGGAAGTCTCGCTTGTTTAAATGTCTGTCTTTTCTTTTCTGTTTGTT
TGATCGCATCATGTACAACGCGGGGTTGCACGTGAGGGTGGGTATTAGAA
TCCCACGTTGAAATTGTGAGGGTTCCTGAAGTAGTCTATTTGTGAAAGGA
ATTACTCCACTTGATACCATATGGTTTTAAGTTGGAACCTCCTAACAATA
TGATATATCCATTTTTTAATCTTTTTTGGGGTGTGTGTGGTGGTGGTTGG
GGGGGTGGGGGCTGGGGTGCTTAGAATGCCATGAAGGGCTTGACTCTTGA
CTCTCAAGGTTAACATATTCACTTGACAGATCCCAAATGCACTTTTTGGT
TCGGTGTCTTCTAATGCATTATAAAAGCTAAATAATTTGGTTCAAAGTAG
CACAGTGTGCGCCCTTGAAACTGGCACCCTTGAAATGGGGATGCATCCAT
TCTTTTGGTTACAGTTCAATTAATATGAGTGGAGTCACCTTCCTTCCACA
CTATGTTAATGGTTCCTTATGCTTCCTGATTCCACCAGTATTTATATCTA
TGTCTGTTTTGTAATAGATGTCCAATATAAACTCTTTGCTTTGGAACCTT
ACAAAGTTCTCTATTGTTGTGGGCGATAGGAACTAAGTTCATGATCGTTA
GTTTTCATGGTTCCAATTTTTTGGGCTGTGTTTTTGGAATACAACCTGAA
AAAATGCTAGTGAGAAATGTTACGAGTGTTCCCAGTTTTCTAGAAACACA
GAATATTCTGTAGTGTGGAATAGAAGTGGAAATGCGGTTTGCCACTTATC
TTAATGTGTTGGAGCACTAGGGGCCTTTTATATTCTACATAATAGATGTA
GAACCAGTGTTGTGAAAGGCGTGCGCGAGGTGCAGCCAAAGCGCCTCATT
TTGTTGAGGAGAGGCGACTTCAAAGAGGCATGCTCCTTGGCAGCCAGGCA
TGAGGTGCTGCTGAGGCGTGCCTCTTTGGAGCAACTCAATTTTGTTTTTT
AAATCTTTTTGTTGTTTTTTTCATCTTCTTCTTCCACTCTTCTTTTGGTT
CTTCATCTCTGTTTTTTCACTGGGGCTGTTTTGTGCAAAAAAGGGCCCCG
GTTTTTTGTAAAAAATAGACCCACTTCTTCTTATTAATTGCTATGTTTTT
CTTTCTTTCATCATCTCACTGTTTTTGCACAAAGAAGGGTCTGTTTGTTT
TTCTTTCTTTCACCATCTCACTATTTTCGAAGGGTCTGTTTGTACAAAAA
ACAGCCCTTGTTTTTTGCACCAAAAAACCATTTATCCACTATTTTCATTT
AGTTTTTTTGAATTTATAATGTAAATTATATTTATTGACTTATTTGTAGT
TAACTACTTGATTGTAATGTTTGTTGAGTGATTCTTTTTTTTTTTTTTGA
AATTTTGTTATCAAGTGTTTCCTTTACTTTTGCTCGGGAATCATAGTTCG
TTTTGAAGTCCAATATTGCATGAGACTAGTTCAAAGTGTTTTTCAATTAT
TAAACATGCAATATATCTTTTATTGTAGTGATATTTGATTACTTTATGAG
TCCATGACAATAGAAGAGCACATATTTAAAATGTGCGCCTTGCTTCAAAG
AAGCCTGTGCCTCGCCTCCCCAGGGATCATTGGGCCTTAGTGCGCCTCTT
GCCTTAAATAACAATGTGTAGAACTGATCATTTGCATATGGAAGTTCTTA
AATTTTAAATATTCAAGTGAACTTCTGAATTGCTAAATTAATGGTTCTGC
AATCAAAGTATACCATGTTTTGGAACGATTCAACCAATTCAAAATGGGCT
TGGGTATTAGTGTATTCTGTTCACTATGGTTGTGAGTACACATTATCAAA
AAGGAAAGTACATTTTAAGCATTATGAAGATAATAAAAGTTTATGTTGGC
CTATGAAGATAATAAATTTATTTACTTCGACACAAATAGATATTCCTTTA

ATCTGAAGTAGGTTATATTGATGTTTCTTATAAATGTTGTCACTACATTA
GACTTTTTAGAACCTCATTAGCATGGCCAAAGGGCAAGTTGACAGGCAGA
TTGTGGCATGTGCAAAAATTCGAAGGTGTTGATGAAGGGAGCCAATTCAG
TAGATTAAGTTGGGCAGATGGAGTCATTTCTTATTTGCACTTCTAGGTTT
TCTAAGATGTGCTTCTGCGCAAAGATTTTTTTTTTTTTTAGGATTTGCTA
GTTCTTATTGTTATTTTAGTAGCGGGGAACAGCAGCTTCTGTTATCATGA
ATAGATTCGGTAAATATACAATTGAGGCAACAACGCTATGGTTCCCTTCA
AGTGTGTGGTGGCTTGCTCTCCTATCAACCTTTTGCACCAGAATCTAGCA
TGCAAGAAAAAGCTGAAACAATCGTAATGTTAGATTCTATATAAATCAAA
TTGGGCATGATTTCTGTCGACTTGTCATTCTCGCAAGTTTTGTACTTCTT
ATAGTTCCACTTTGCATGCATTCTTAGCTGCAATAGAAATATTTCCCTTC
CCAACATTTATCTCTTTCCCTTGTTGGCACCACATTGAGATTTCAACCTG
TTCAAATCAATTATCATCCTTTTGACTAATTTTCTCTCTTATATTTGTCA
TGTTTATTGGTATTACTAATTTTATATCGTCCACCTTAGCGAGTATATTA
CTGAAAGCCATGTCAGGTTGTATTAATGGTTATGATCCCGACCAACTCAA
ATTAGCCTCCCCTCCCCACAAGTTTTTTGCATGGGTGTGCGCAATGGCTT
CTGGATTAGGCATGTTAAATCTCCCCTGACAAGCTTCTTGGTATGATTGT
GGTTCACAGGTACAAGTTAAATCAAGATTGAACACCTTCAATATTTTTAA
ATCTAGATTGAACACCTTCAATATTTAGGTAGTATGCATGAGTATCTCCA
ATGACTGGTATTGACGACAATGATATTCAAGAATGGACAGTTGGATTCAT
GTACATATAGAAGTCCATGTCGTGGCCTTGTTTGTTGTTCGTTGGAAGTT
GTCATGAAACATAAGCCGAACAGATAGAATCCAATCTAGGTGGCTTAGAA
ATGATGGCAAGAAGCTTGATTACTCGTAGTCCGCTTGAATGCAACTGATT
CTACTAAAGAGACAGAGAGAGAATATCTTTTCTTTGTTCTTGCTCAGAGA
GTTGTTTGAGTACCAACTTCCGTCTTCATGGCTTTATACAGTTGCTTTGC
CATTTCCGGCTAACCAAGTCGTCATGTTTTATTAGCATATCTGAGGGATT
TACACATGATATTTCATCCTAGTGGTTATCTTTGGTTCGAAGGATTCAAT
TGATGCTTTTTTGTTAGAGTTTGGATATTGTTAATGTCATATTGGTAGCA
CAGTTTACTATGAGTGCTGATATAATTTTGAATTTAAGGGATCGTAAGTA
AACATTTTTGCGTTCAAATTGATTGTCTCATTATTTGGATGGCAAAAAAA
TTCCCATCTAATTTCGATTTCAACCTTTTTTTTTTTTTTTCCTTAGAGAA
AGTACTTCTTGACTAAATGGATAGATAATACTGACATGTCATTATTGACA
AAAACTGTGTACATTCTGTGTTTAACTAGTAAGCATGTGCTTTTTTTTTT
TTCTACATGAGTTGTGCCTTTTTTTTTTTTTGGGTTTTGCTAAGCATGCA
TTGATTTTAGTCAACCACATTACTTTGAAGGATAAGGTGGATCGTTCAGC
AGTACGAATATATTGTGTTGCTTTGCACTAGTTATCCGTGCTGACCGAGT
TTTTGTTTCTTAGGATTCACCATCATATACAATTGTAGCATTTTCTAGGT
TTCTTAGTTTTCCTGGTGTTTTTCTCTGTATATACTCCTCACGTCCTTGA
CTAAATGTTTCCTGTCCCATTAAAATGATCACCAAGATTGATTTCTAGCT
CTAAAAAGATGTTAGTTGATGGTAAGATTTGCTTGCATATCATGCATTTG
TATGACTACCTTCATATTAACATTTTTTCTCCCTAAATTTTGAAATTGAT
TCGCTATTTAACATAAAATTATCCGGATGCTAATGTTGTACAAAAACAGT
ATCGTATGATGTGAAGGTGTGAGACAAGGTGGAAGCCTGATATACTTTTG
CTTTAGTATTTTATCAATATTTCAACTTTTAAAAATAATACAAAATTAAC

GCTCAGGGAATATGACCCACATGTCACTTAGGGGACTTCTGTCTGATGTG
CCCTGAGGCATTTAGTATGCCCTCACCTCGGGCAGCTCTGCAATGACACA
CTTCACAACATTGTTTAAAAAGAAACAAATTAATGAACTTGTGATATATA
GGGGTTGGAAGATGGGAAAAATAGCAGTAACTATGAGCATACTAACTAAC
ATGGATTGAGATTTTGAGACTATGGAAGGAAGGGAGCCTCTCAATTATTT
GAATATTGAGGGTAATTCCTTTTTGGATTACCACCTTTTATGCGTACCCT
TTGGCTTCCAAACAACTAGTGCTGTCTATCCAGCTTGGCAAGTTCACTAC
ACGAGTCTCCGTCACTCGTGCCAACTTATTTACTAGAAAGTTCTCATACC
GAGAAAAAGAAATGTTGGCTTTCACTCGGCCAAAAAAGTAACGCTGAAAT
GTCGGCTTCACAGTTATTCATGGATTGTCTTGTATTTCCACTAGAATGTA
TGTCAAATGCTGACTTGGTTTTTATGGGATAAAACAACCTCAAGAAATGA
TTGTTAAGATTTTTTGGTCCTGTAATTATTTTCCAGCCCTCGAGGTAATC
TGCGAGAGGCCCATCTAGGAAACAAAAATCTTTTTTTTGGAGGAGCCAAA
CATTAATTCATAACCTCTTCTTTACTCCTTGCCCTTGAAACTCTGATGTA
TGAAATAATTTCATTTCTACATAGTCTAAAACTTATGGGAAGCACAAATA
CCCTGGCAAATATACCTTTATATAACACTGTTGCACAAATATTGCCAATA
TTTTGACGTCCAGAAGTAAATGCTGGGTGCTACAGATTATGTTACGGGAT
ATAATAATGTATGTTCTAGCTAATGCATCTTCCATATTTAGAGATTCCAT
AACACAAATTTTAGCGTGTGCCATAGAGTAAACCATTGCCACTTCTTTTA
GCTAACTTTGGGCCCGTTTGACAAAATCTAAAAATTAAGTGATGAAAAAA
TGCTGAATTTTAAGTGTTGAAATTATAAGTGCTAAATTTATTAAGTGCTG
AATGTATCAGGTACTTCGTTTTTTAATAATTTTTGTCTACAAAATTCTCT
CTATACAATTTTTTTTTGTCTCTAATATAATCTTGACAACTTGCTCTTTC
TAAACCATTCTCTCTCTACAACTTTCTCCATCTATTTTTTTCTTTTCAAT
TTAGTACTTTTTTTTTTCTCTACAAATGTTTGAATATACCTTATTTTTTT
CTTTCAGGATTCTACAATAATTGTGTGTAACGGTCATCGTGGTAGATAAA
TCCTTGATGGTTTGAAGTGATAAGTGCTAAAAGAATAAGTAACACATCAC
TTAAAATTTTGAACTATTTTCAAGTGATACTTATATTTTAAGTGACTGTC
TCATCTTTTTATCAAACACACTTAATTTTAGTAAGTGCTTAATGTATTCA
ATTTTTAGTGATTAATTATGTTATCAAACAGGCCCTTGGTAATTGGTATA
TTATTTCCATGCATGAATGTTGCATTAGAATATCCCGCTTTCCATGTAAC
GTTCTTCTCGCCCCAATTGTGGCCAATGAGATGCAATCATGGCAGTTTCT
TACTCTGCTATCTGCATGTATGGACCTCCGTTCTTTATTTGGTGACATAG
TTTTAATGAGAAGTGACTACAGTTTGTCACAAGATTTATCTTTGACATTT
TTCGAATAAGCCTATTGATAGCCTTTGATCTTGATTCCCCTTGCTATGAT
TAACTAATTTGACCAATAGTTCTTCCATTTTCAAATTTCTTAGCTCACAG
AGCAACTCTGCATCCAGTCCATGGCACATTGGATTGTTTAATGAAGCACA
GTTTTGTTTAGAGATAAAGTGGTGGTACAACTCTACTGGATCAAGTCATT
ATTTACTCCAACTAGTATACCACTACCGGGGAACCTAAACTTTAATTAAG
AGGTCGGACTATTAATTTGGATTCTGCAAGGAAGTAGCAGTTAGATGCTT
TCCATTTGATAAGGTCTTGTTATGTATTTTTTTGGTTAAAAGCAAAAAAG
AACTTAAAAATCAAAAGTAGCATTTGTTTTGTGTTTTATATTATTTTTAA
AAATCAATAAAGTAATATTGCTATTTAATATTAAAGCGAAAAATAGTTGT
TTTCCATTTCTTCCATGTTTACATTTTATGAGATTGATGGATTAGGACCT

CTCGACCTAAGTTTATTCAATACTCTACAAAATTCTTTTGTATTAATAGA
TCATTTATTTGGATGATGCTAAGATATGATCTTTACGTTACCAAACACAA
TTTTTTGCTTGTAAATTTTTTGTTCAAGTAACTTCTTTTTAATACAAAAA
TACTTAAAAATAGTGTTACCAAATACCTAATTGTTTTAGATGTGGATCGT
GTGATATAGGAAGCACAAACAATTATGAAGTTTACATTGGATGTACATTG
AGCATGACACTCCATGGGAGTGTGTCTGACATTCATTTACTGTGTCTTAG
CCATCTCATATGAAGCCATTTTTGTGTAGAATACTGTATTTCTCCTTTAA
CACAAGACAAAGTAGATTTAGATTATGTAGTTTATTCTTATCAAAAAATC
GTAATCACAATTCTAGGAAAGGCTTGTCAACAACAACAACAACTAAGCCT
TTGTCTCACTTAAGTAGGGTCGGCTACATGAATCTATTTACGCCATAATG
AACGATCAAGGGCCATCCCTTCAGTCAGGTTCAATGTCATAAAATCAATT
CTCATAATCTCGTCCCAAGTCTTAGGAAAGGCTTGTCAATTAAGATAAAA
TATGAAATGTGACAACGAACGTAATGGAAAGGAAAGTATAGAGATCTTGT
TCTTATCATCATTCATTATTTAAAAGGTACGCTCTTAGTTATGGCCTATT
TAGGATATTTTTCCAGTATCAGTGTGACACATTTTTGTTATTATGATAAA
TAAAGTTATTGGTTAATTTATGCATACAAGTCAACAATGACTTTTAGAAC
TAATGAGAGTATAGATTATGAATGATGTGGTGTTTATTTCTTCGTGCATT
GTTCGTAAATTAAGATGATAATTTTTGCTTTGTTCTTGTCTAACATCATT
ATATCATTCCATGCTCATTTGAAATTTGACTGAGTGTTTGGTAGTTTCCA
ACTAGGAATTTTATTATATATAGTCTGTTTATCGTATTGAATTCTACCTT
GTTATTGGAATTACTGGTGGTATGTCCTTGATTTCTAGGTTGTATCGCTT
TATGGAGAACAATGTTTATTGCTTGTCATACTTGTTTATTGAAAAAGAAT
ATTTGTCAACAAATAGTTAACAAGAGTTGGTGGATTATCAAGTAATTCTT
TTTTATCTTTCCCATTGTCACATTTAAGTATCAAATATTTTTTCAAGAAA
ACAGACCAAATTTTGATGAGAGAAAGTTTTAATTGGTAGAAAAAATGGAAC
CAAATGTTGAGCATAATTAGCAATGGAACTCCAAAACCAAAAGGAGCAAG
TGCAACAAAGCATCCGCATGGACTTTTGATAGTCTTATCCTGCATTCTCA
GAAGCAACACCCCCCAGTTTAGTAACAATATCCGCCATTTTTTTTTTGGA
CATCTTATCAATGCTATCAATTTCAGGGTTTCCACCAACTTAGATGTTTA
GGACGGGTGTTTTAAAATGTATTAGGATGAGATTCACTGGTCATTGTAGC
CCTAAAGTATATGGATAGTTTTGGGTTTTGCTTCATGCTTCAAGTGACAA
TGTGACACTGTTTTTTGCGCTTCTATGGCTAATATTTTTCAGATTTGAAC
TTTCTTCCTCTTATATTCTGAGTAACTAAAGAGATGAACTTTCTTCCTCT
TATATTCTGAGTAACTAATGAAGATTTTGTGACCATACATAACCATGTAT
CCTTGTTATAAAAGCCTAGTTTTTAAGTGCTCTATTTTTAAGTTTTCCAA
GTAATTAGCTGCTTCTCTACTGTTTCTATAAACATATACATATTTTAGAA
CCAAATGTATTAATCCACATTAATATTTCTTATGTGCTTTTGTGATGAAA
GCATTGAACTCTAGATTCCATTAAGCTGTTTGCTTCACTGAATGGAAGTC
CATATGCTTTCTGATTTTACAGGAAACTCCCCTATATGGAAATACTAGTA
ATGAATTATGATGTTTAGAATAAAAAGTTGGTCGCGAGTTTGATTCTTAC
ATCGGTATAGCAGTTTGAAGAAGAATTAGCACCATTCGACTAATTTTGAT
GTTGAGGATAAGTTGAGAGAAGATCCTCATAGATGGGTGCACATATTTCT
AGTTAGAGGAATCTTGTATGTTTGTTGACCTACTGGGGTTTCGTCTTCTT
TCTAACTTGAAGGGTTCCTCATCCTTTTATGTCTTTGTTTCTTTGTCTGC

TTCATTACATTTATTGTCCTTCTTAGGTAAAAGATAGGTATTAATTTATT
TAGTTTAAGGATTGAGTATTAGGAACCTTTCTATCTTGACATGGCTGACT
GACCCTAGTTGAAATCTATATTTCAGTTGTCTATTAAGCTAGACACTGTA
GAGTGGGACATGGTCAGTGTTCCACTGTCATTTTTCTGCCCGTTCGGGAC
ATTGTAGACTACACAGTGAGGAGAAGATCCATTACATTGTCCTAGTGCCT
GTCATCTGAAATTCCATTGAAGAATCTTATGCACTTGTTTACTTTAGTAA
TTTGTAGAGTTCGTTTCAATTATCACGGCACCCACAAGTCTACCGAATCC
TGAACTTAAAAGTCATTTTGTTTATCATGAGCAAGAAAGTTGAATCAATA
CCACCGAAAATGAATGCACAACATTAATTGTGTCCAGTTCATATGACAAA
TGGATCAGCTAGTTTTTTTTCTATCAGTTAATAAAAGTTTTCAATTTAAT
GAGCCTTCTCAAAAAAAAATTTTAGTGTTATATTTTCGGTAGTCTGTGAAC
TTGTGTACTGACCGAAGCTTACTGTTATGCAAATCTTGCCTGTGCCTCGT
CATTCTTTGCTTCTTGCTAATTAGTCTTAATTGGCATATTTATTTGTGTT
GTGCGCTAATTGAGTGCATTTCTTCTCTGAATTTGCAGGGAAATGATCCA
GCACAGGCCTTATACGCAGAAAGTCGATGTTTATAGCTTCGGAATTGTTC
TGTGGGAACTCATCACGGGAATGCTTCCTTTTCAGAACATGACTGCTGTT
CAGGCAGCATTTGCTGTTGTCAACAAAGGGGTCCGTCCTAACATACCCAA
TGACTGCCTTCCCATTCTCACTGAGATTATGACCCGTTGCTGGGATGCTA
ATCCCGATGTCAGACCACCTTTTGCTCATGTGGTTAGAATGCTTGAGGCT
GCTGAGACTGAGATATTGACTACTGTGAGAAAAGCCCGTTTCAGGTGCTG
CATAAGTCTACCAATGACTACTGATTAA
>EUC10624-RA [gene]
ATGGACAGCCCTACTGGAACTTTGACCACAACTGGATCAGGCCCGAGTTC
TAATGAGGAAAATCCACGTGTGAAGTTCTTGTGTAGCTTTTCGGGTAGTA
TATTGCCTCGACCACAAGATGGGAAGCTTAGATATGTTGGTGGTGAGACC
AGGATTCTTAGTGTCCCACGGGATATTAGTTATGAGGAGCTAATGGTGAA
GATGAGGGAGCTCTTTGATGGTGTTACTGTGTTGAAGTACCAGCAACCTG
ATGAGGATCTTGATGCTCTTGTATCTGTTGTTAATGATGATGATGTTACC
AACATGATGGAGGAGTATGAAAAGTTGGGAGCTGGTGACGGGTTCACTAG
ACTAAGGCTTTTTCTGTTTTCACATCCTGATCAAGATGGGTCCATGCATT
TTGTTGATGGGGATGAAAGAGATAATGAGAGGAGGTATGTGGACGCTTTG
AACAGCCTCAATGAATCTCCAAACTTCAGAAAGCAGCCGTTTAGTGATTC
CCTACTAATGGGTCCTATAGATGATATCAATGTGACTGAAGAGTACTTGA
ACCAGATAAGTCTTGAAGGAAGTGTCCATAACCAGAGGAATTTTGACATG
CACATGCCTCATATAAATTTGCGTCACCTCACAATTCCGCAAATGGGTTC
AGGTCAGCATCAGCTATCTGTCAGTCAAAGGTATAATGACATGGAAGCTC
CATGGAGTCCCATATACTATTCTCCAGGGCATCCTGGGCATCATGACCCA
AGACCAGCCGCCGAGTTTCCTGCTTCACCGTCTTCTGCTGGTCACCCCAC
ACCATTTGGGGAGCTTTCTGATAGAACATTTGATAGAATTCCTGAAGATT
ATAGTCTCCATCAAGTGAATAACCAATATGACTATCTTCCACAATTTTCA
GACAAAGTTGCATTCTTCTCAGGTGGACCTGTTTCTGCTGACAAGGCTGG
TTTTCCGGGTAACATACTTCAGGGCCCCAGCATCTTTGAAGGAAACAATG
TATGTGAGAATTGTAGAATGACATTTCAAAGAAATCAAGCTTATTCTGAT
TCCCCATGGAAGCATGGAGAACAGCCGCGTTTGGAGTCGTCTAATGTTGG

GAGTGTGTTTCACCAACCAGCAAATCCTTGTGCTGATTGTCATTCAAATA
GGGAAATTTATATGCTGCATACGGATGCGAGCATGCAACAGCCATTATAC
AGAGAGCAAAACAATTCCCGAACCCTTCATAATGAGGCTCAGGAAAGAGG
ATGGGTATTTCAGCAAAATTCAAATCTTCGGGTCGATAAACCAGTGCCAC
AATTATCTGGAACGGGAAGGTTGATGGATCACTATGTTGTCAATGGTAAT
GGTATCCCTGCTCCGGCAGCACATGGCAATCTATCGAATGGTCATCATGT
GCCCTCACATTATGCACATCATGAAGATGTACGTTACCCCTGTGTTGGAC
ATGAGTTGGGGAATCAAGTCTTTAATGACCAAAATGTGACTTCTGGATCC
CAAATTCACACTCATACACCTCCCCCTGAAGAGTGTGGGGTTCTATATGG
GAATGTGACATATGCTTATGGAGCGGATAATCTTTACCAATTCTCACGTG
CACGCACACCTCCAAATGCCATTTGGAGAAATGTTCATAATCCAGTGCAT
GGAGCTCAGTCTTATGAAACATCTAGCTCTCATTTACTTGCAAATGGTTC
AGTTGGTGCAGGATTCATAAGGTGTGCATTGGAGAGTAGTCCTAGGATAC
GACTAGGTGTGGAGAATCAGAACCCTTGGGTTGATTCTTCACAGTACATG
ATGGGTTCTGAGGGGGCTGCTGTACCGAAATATTCTCATGGAAATGCTCC
ACAATTGGTGCCAAACACATGCTGTCATGAAAATCAGCACTTGCCTACTC
CAGAACCACAACCATCTCCAGCTGAATTGCTAAACCATTTGACTCCTGTC
GAACCTATACTGAATTCTGATGTAGCATCAAAAGTGGTTGATTATAAAGT
TGTTCCTCTCACAACTTCTGGTCCAGAAGCAAGCAACGATACTGACCTTA
CTGAATCAGAAAGGTTGGTGCAGCAAAGTTCTCATGGAGGAGAAGAAACT
AATCATGAAGCTAACATGCCGAGTATATCTGCTCTAGTGAAAAGTGAGAA
TCCAGACGATATAGGCTCTCCCGAGGCTATCAATTCAAACTCTTCGAAGC
CTGTTGAGGAGAGAAATGGAGGTGTGAAGTCAATTGAAAATCATGCTCTT
GCTCCTCTTGACAGTGAGCAGCTCCTGGATCGGATTGTGACCGAAAATAA
AGGAGAACTAGAGGGTCTTGGAGTGAAAATTGACGATGATGAAAATTCTG
CTTCAGTTATCGAGCATAATGCTGCTGCAAAGGAAGCTCAGAATGATAGA
GAGTCAGTGGTGAGAGTTAATTGTAATTGTTTTCCTCAAGTATGTTTAAG
TATATTAATTATGGATGAACACTGTTTTCTATTTTTGGATAATCCTTTTC
CTTTTTGCTCCTTCTCCCCTTGAAATGTAGGAGGCACATGAAGATTGTGA
GTTCGGTTCTGATAATGACAATTCTAACGATATCAAGATTGAGCCGACAA
AGGCAGAGGCAGAAGCTATAGATAGGGGTCTGCAGGTTACTATATAGCAT
CAATCTTGCCACTATTATATGTATAATGATTATGTTGCAATCAAAAATGT
GATTACAATCTTCATCCTGTTGAGATTTTGTTCTACCATCGTCTCATTAT
GTTGTAGTAATTATGGTAATTTTGTTTTATTGCAGACGATAAAAAATGAG
GACTTGGAGGAGATCAGAGAATTGGGTTCGGGAACCTATGGTGCTGTTTA
TCATGGGAAGTGGAAGGGATCTGATGTGGCAATAAAGAGAATTAAAGCAA
GCTGCTTTGCTGGTAGGCCATCCGAAAGAGAACGTCTGGTATGTTTTATG
TTATTTCTGATAATCCTTTTGCGAAAAGTATTCCCTCAAACATATCTGGG
ATCTCTTTCTTTCTTTAAGCAAACTGTTAGTAACTACTGTGCTCCGTTTT
GTTGTTAGCTTATCAACATTCTACAATTTTTTATGTGATTAAAGGAATCT
TGTAAGTAATATCTCCAAATTTGCGTGATTCAATGAGCCAAAAATTGCAA
GGACATTTGTATGATTCTGTAAAATGGATAAAAGTAATCTCATGAGTAGT
TTTGGTGAAAAGAACTATAATATCTTTGTTCCGGTCGAGCCCGGAATCAT
GCATTTTCTTGAGATTTCAGTTTTTTAATTGGCTAAGTCTAAAGGATTCT

TCTTCTGTTCCCACTTTTTTGGAGGGAGAGAGAAAATCCATTCAAGCGCA
AGTGATATTAATGCTTCTCTTACTAAGCCAAGGAGATTTGCTATGCAAGT
TTACAAAAAAATAGAATTGAAAATTTCTCGTGTTATGTCCTCATCTGGAG
TCTGCTGAAGGTATCTGTACTAGAAGGGACATACAGGCAATGCGTGAGGT
ACGATAATATTTTATGAACCCCCCCCCAAAAAAAAAAAAAAAAGAGGAAAT
ATTCCAGTAGTTCAAACAGAGAATAGTAAGCCACTATACCTTTAGATTGC
ATGGAGTGTTGACAGTAGAAAATAAGTCTTCTGATTGTGGGTGATGCAAC
CTGTATGACTAAGAACCCATGACTGTAAATAATGGGAACATGAACTTAAA
AAAAAAAAAATGGAGTTTATGGATGGGATATTAGTTGAATCTTTGCTAGA
CTGTAAATAATGGGAACATGAACTTAAAAAAATGGAGTTTATGGATGAGA
TATTAGTTGAATCTTTGCTAGTAAAACTGGATTTAATATAAAGTATAAAC
ATAAGACAACCCTAACACTATTGTCCCACTTAAGTAGGGTTGGCTTTGGC
CTTCGCGTCAGAGAAAATAATTCTAGAGTTATTCCCTCCATTAAATTAAA
TGGTATTAAGTTTTTCCTTATTGTTTATTCCCATGTTTTTAGGTCTACAT
CTCTCACTTGTGACACCTAACTTTTCTCATGATTTAAGTTACACTTTCTA
ACTGGTGTGTCCACCTTTCTTCTATGTACAAAACCATTTTAAACAATTTT
CTCTTATCTTATTCTTAATGGCTGTTCACATTCTTTAATAAGGTAATGTT
AAATACCGTCCATTCTTGTATTACCATTCGCCCACTTTAACATTCTCGTT
TCAGCTAAATTTATTTTATGGACGTACTTTTTGTTTATCGACCAACATGA
GGAACCTACAAGATCTATAGTTCTACAGCCGTTTTATAGAATTTCTCTTT
CATTTCATTGCTATCCTCAATCCTCTGACTATAGGATATTGCTCGTATTG
TGTTGATCTTACTAATTCTTAAACTTTTGGCTCTATAATTTCTCCCCAAT
GTTCTTATTTTTTAGGTTTCATCTAGTTTCATCAACCAGTATTATGTCAT
GGGAACATCATTCTCCATGCCCATCTTGAATATTTTTGTGATTCATTATT
ACTAGAACAAAGAGATATGGACTTAATGATGATCCTTGATGTAAGACTGC
GGATTCAATGTCTGATTAAAATCATGACGTTGATAAATGCAGTCAAACTG
TTTTGGAGGATGATTTCTTGATAAGGTTTGATCTCTTGGCTTGCTTACTT
GTAGATCATTAGCAACGAGGTAGAAGCAAAGTAAATAAAAAATATTGTAC
TTGCTATTTATGCCAGGTTGGTATGCATGTGCAGCGACAATCCTTGCGTA
GTTATTGTTAATTTTGTTCTAGTTTCTTCATAGAAACCAAACCAGAGAAA
AGAATTGTTCTAACTTGACAAACTACCGACCCTTTTCTCTTTTTGCTGAA
TGTGTTCCAATTTTTTTTTCTTTTTTTCTTTGTTTTTTGGTCTGGGAAGT
GGGAAGTACCTTGTACTCCGGTTTCTCTGAATGTGGTCCTTGTGCTGTAT
TAATGTCATATTTTCTTGTTTGATTTTATTTCCCTGATTTTTCTTGATTC
TATAGATTGCAGATTTCTGGAAGGAGGCTTTGATATTGAGTTCGTTACAC
CATCCAAATGTTGTTTCTTTCTATGGAGTAGTTCGTGATGGCCCTGATGG
ATCTTTAGCAACAGTGACAGAGTTTATGATTAATGGTTCTCTGAAACAGT
TTCTGCAGAAAAAGGACAGGTATGTTTGATGATTTATTTCAACTTTTTTA
GTTATTTCTATTCCACGCAACCTGCTGGGTATGCAAATACAAATGTTTTC
CTCAATTATATTGAAATGTTTATTTACTTTATGGCATGCGAAACAATAAT
CTTGTGAACAGAACAATTGATCGTCGGAAGAGACTCATGATAGCAATGGA
TGCTGCATTTGGAATGGAGTATTTGCATGGGAAGAACATCGTGCATTTTG
ATCTGAAATGTGAAAATCTGCTTGTGAACATGAGAGATCCTCACCGACCA
GTTTGCAAGGTATGTTTAAATAATAATTGGGTTGATTATATTTGATACTT

CATCCATAATGTCTTGTCACACCTTTCTGGATCAAGGATAATCTTCTGGG

CTATCTTGTGTAAGTAGTTCTTTTTCAACTCGTTTACACGATGGAATGCA

TTCGTATTCCCCTCTTGATTACATAGGAGTTGCCAGTGATAAATTTTGAA

TTCAAGCTGATCGTTGATTTAGCGATACTAAGCATTTTAGATGCTGATTG

TCTCTCTCAGCAGTGCTTCTTTTGTATTTAAATTTCTCCCACTTGCATTG

TATAAAATATCTAAGCTAATGGAATGCATACGTTTCGTTTTAGATCGGTG

ATCTTGGCTTATCAAAGGTGAAGCAGCATACATTAGTGTCAGGAGGTGTA

CGGGGAACCTTACCCTGGATGGCACCTGAACTTCTAAGTGGAAAAAGTAA

CATGGTAACAGAAAAGGTAAGGCGAATGTCCACATAGTATTTCAAAGTGT

TTTTCTAGTGTATCTCATCGTTCTCCTTTTTGGAGGCTAATGGAGATTTA

TTTTCAGAGGTTTTCTTTGCAGATATTCATTTCTTATTTTTCCTTGCATC

GCAGATAGATGTATACTCATTTGGGATTGTTATGTGGGAGGTTTTAACAG

GCGATGAGCCTTACTCAGACATGCACTGTGCTTCTATAGTTGGTATGTCC

TAGATTATTAACATTTTTTCATGTTTTTTTTTTCTTCCCCTCAAAATCCT

CGTCTGCTTTGTTTGTAATATCCCGCATTTTTATTTATTTATTTTTTTTA

TTTTTTTTATCTCTAGTAATCTGTAGCTTAAAAAGAAAAACTTCAGATTT

GTATTTTATGGTAACTTTCATCTTTTCAAATTTGTTTATTGTATTCACCC

AGTTATTATGGAGTGGTTTTCCCTTTGGCATAGTTGTCAAAGGCACGCCT

AGGCGCAAGGCTCAGCGAGGTTCACCTGGTGAGCCTCGCCTAGATAAAGG

CGCGCAAGCCTTCTGAAAGGCATGCGCCTTCTGAGCCTGGGTGCAAGGCA

CGCCGTACTCGAGATGCTGGGTCTTACTTAAAAAATGAAAGATTTTGCCC

TAGGTCTGTTACATTCACTTGCTTCGCAGCAGTTCTTCCTTTTCGCGGCA

ATTCTTCCTCTTCGCAGCGTTGTTTGGACTTCGATTCTTGAGCAGACATC

TTCGTAGCGTTGGATCTTCCTCTTCACAGCACTATTTGGACTTCGCATAT

AAGTCTTCTTCTCCTTCTTCTTCAAATCTTCTTCTCCTTCTCCTTCGAAT

CTTCTTCTTGTTCTTTCCATCTTCGTTTTGCCCTTGTTCTTCTTCTTCTT

TCTCTACTTCTTCCCTTGTTCAGCTTCTTCTTTCCCTGCTTTGTCTTCCT

CGTCTTCGTTCTTCGGTCTTCTCTTCCTCTTCGAGTACGTTTATTTTTTT

TTCCTTTTTTTTACTGTTATTGTTTATTTGTTTAATTAATTATTTATTTA

AGCAACTTCAATTTAGCTTCTTTTTTTATTGTTATTGTTCACTTCAGCAG

CTTCAATTCCCCGTTTTCATTTTTTTTAAGATTCTTGATTAATTGAATTT

ATATGAAAATTTTATTAGTTAATTCTTCTTATTTATTAGTTCACATGCAA

CTTGTGAGTGTGTTTTATTTTTTATAATTGAAATTTAGTTCATAATTCTA

TTTCTTGATTTTTGATTTGCAATATATTAATGTTATATATATATATATAT

ATATATATATATTTATTTATTTATTTATTTTTTAATGTCGTGCGCTTGGC

TTCGCTGAGGCTAGCGCTTTGCCTTGCTCCTTGTGCCTAGGCTCGGGACC

CCTTGTGCGCCTCGGTGCGCCTAGAGCCTTTAATAACTATGCACCCCATT

GATTTGTATATTGTGGGTTCGAATGTTTGAGACCAGCTACTTTTGCATGT

ATGGCTGAGGTTAGGTCCATTGTCAATATATCCCCTCACAGGCCTCACTT

AGGGCTCACTTGATCAACTGGATTTTTTTGTTAATGACATTTGAATATAT

GTGGATGAAAGGCGGAGGTCCCTCCACCAACCCCTTAAAGGGGGTTCAAT

AATATTTTATTTATTTATCTTAAACTGGCCATGACTTAGGGTTTGCAAAA

TGGCACATTGTAAGTTTCCTTAGGGATCATAGCAACTAATTCATTATGGC

TATTATCCACATGGAAACTACACTAGACAGGGAACTGTACCAGCACCAGC

GAAGCGTTCCGTTCATTGTCTTAGTTATAGTAGCATTCCTAGCGCATAAC
TTGTGGCTAGTTAAAGTTGTCCGCTTGCTGAATGGAGTTTGAGATTTTGG
TAGATTATTTTTGTATGAAGACTCTTTAGGTGTTGTCATCATTAGTGTCA
CAGGATTGCTTCAACCCAATTGATTGTTGATTTTTTTTTTTTTTGCATCT
TTCAGTCCATTAAAAATTGCTTTGTTTGTTACTAATATTTTTCGTGTGCG
CTTCATTAGTCATACCTTATTGACAAATATTTGAGGTTATTCCCTGCCCC
TAGCACCACCGCCCTGATATATGCCCGTTAATCCAACTGGTGCATTCACT
TGTTTTTATCACATTCATCATTTTGCCTCCATCAATATGCAGGTGGGATC
GTGAATAACACATTACGCCCAAAAATTCCCACGTGGTGTGACCCTGAATG
GAAGGCCCTGATGGAGAGTTGCTGGGCCTCTGATCCTGGGGAGAGACCTT
CATTCTCAGAGATCTCTCAGAGGTTAAGAAATATGTCTGCATCATTGAAT
CCCAAATAA
>EUC20307-RA [gene]
ATGTTGGAGGGTCCGAAATTCACTGGAATTATAGACCTGAACAGCAGCCA
TGATGATATTTCGCAGAATTTTTACCATAAGCTCGGCGAGGGATCAAACA
TGTCAATCGACAGTTTCGGGAGCTTGCAGATGAGCAATGGTGGAGGGTCA
GTTGCCATGTCCGTCAGTAGCGTTGGATCGAATGATTCCCACACTCGTAT
GCTAAAACATCAGGGCCTGAAGCCTGGCAACAATTACTCTGTTGCCCATA
GTGTTAACCGGGGAAAAGTCTCCCAGGGACTGAGTAATGATGCATTGGCA
CAAGCTTTAATGGATCCTCGCTTCCCCACAGAGGGGCTTGAGGACTTCGA
CGAGTGGACGATTGATTTGAGGAAGCTTAACATGGGAGCAGCCTTTGCCC
AGGGTGCCTTTGGAAAGCTCTACAGAGGTACTTACAATGGTGAGGATGTT
GCCATTAAGCTTTTGGAAAAGCCAGAAAACGATAGAGAAAGGGCACAGTT
AATGGAGCAACAGTTTCAGCAGGAGGTCATGATGCTTGCGACGCTAAAGC
ACCCAAACATAGTCCGTTTTGTTGGTGGATGTCGTAAACCAATGGTATGG
TGTATTGTTACTGAATATGCCAAAGGAGGTTCAGTTCGGCAGTTTTTGAC
CAAGAGGCAGAACCGATCGGTGCCATTGAAATTGGCTGTGAAGCAAGCCT
TGGATGTAGCTAGGGGGATGGAGTACGTGCATGGGCTTGGATTGATTCAT
AGAGATCTGAAATCCGATAATCTTCTGATTGCAGCGGATAAATCAATCAA
GATTGCTGATTTTGGCGTCGCTCGAATCGAGGTGCAGACCGAAGGAATGA
CGCCAGAGACAGGCACTTACCGTTGGATGGCTCCGTAAGCTTCCATACCC
TTTTAGCTGCCAGGATTTTTCCTCTCTCTGGTCGTTACTGTCACTAGATT
GTTTTTAAAGCTAATACTATGGTTTGACTAGATCTAAATATGATATATCT
ATATTAGTCTACAAGGCATGAACTTTTGGTAATTTCTTGATAGTTTTTTT
AGGATCTATGTTGCATGAATTGTTAAATACTTGAATGATTATCTTCTTTT
TTTTTCCCGTATCTATTAATTTATTCATCCTCTGTCTATTCATCCATATG
CTACTTTGCTCCAAAAAAAGAGATGGAGTGTGAACTTTTTAAATGTTTTA
GGAGGAGTTCAAAGCTTGAAAGTTTGGATGTTTCCTTTATATATTCAAAT
GAATGGTGTAAGGCTTGAAAGATGGAAACATCTCTCAGTTATTCAACATC
ATGAAGGTACTGAATACTAAAAATTTTCTTCGATATTAGGTTTTCTGCGT
GAGTTTATCTATTCCATACTTTCTAACTGTGTCTTCCAATAGAAATTGCA
AATTGTCTCTCTTGATGGGTTTGCTCATCAAGTCTCACCTTCTTGAGTCC
GCAGGATGCATAAGAGGTTTTATCCTCCTAAGTGTTGATCTCAGGGTTGC
TTTTGTCATGAGATGAAGAAATTGGGCTTCGTGTTCTCTTATTCAACTCG

TTTGGTCACTGTGAAATGTCACAACTTAGTGCACAAAAGGCTCTCGCCAT
TGTGGGTTCTAGGGAGGAGGGTCAGTTACACGTAGCCTTTCCCTTGCATG
CGAAGAGATTGTTTTGGAAATTTGAAGCCATGACTTCCAGGTCATAACGG
AGCAACATTTGCCATTATGTCAAGGCTCACCCTCAAATGTCAATAACAGA
TGTTGAATGTTAACCTGAACTGGATGTTTGAGGACCAAATTATCTAAGAT
GGGATATATATGATTATGTCAACCTGGGATATTGTTTTCCAGTCCTTGTG
GAAAGATTAAAAAAGGCATATCTAAAATGTCTTTTCTGTGTTCAATCAGC
ATAATCTTAAATGTGCTTATAAGAGCTTTTTGTAGTTTCCTAAAAAAATT
ATTATATTTTTCCTTAGTCTCATGGTCATAGGAAACAATGATGCCCTATC
AAATGGTTCAATACCATTCCAAAACTCGACATTTTCATGAATAAATTTAT
CATAACTGCATGTGACTATCTCTATTTTGAAGTTTTTGACGAGGATAGTT
AATGTATTGTGATTATACTCCTTACATAAATTTTTGAGTTTTTGATCCTG
CAGTTTCAATCCCCTTCATTTTTTTAATATTACTGCCGAATATTCTTCCT
GTCATAAAAAAATTCAGTTATTGGGCATATTGTCTCCTGCTCCCATTCAAT
GCATTTCAAATATTCCACTTTCCAAGTAAATGTCTTGCTCCATATTTATG
AAACGAGTTCTTATTATATTGCCCTCTGTCATTAGTTTTATTTATGTCCT
ATTCTTTAGCAACTTATTTTTTAATCCTTTTCAACGCATTTTTTAATCCT
TATATACATTTTAGTCAGCGTTAATTATGATATCAGATACAAATTCAACA
TTTAGTTGATACTTGATACTAAGCAAAGGGCTAAAAAATTATTCAAGATA
AAAAATCATAAGAATTGTTTTAAGAATAACATAATAATTTTCTCTTTATA
AATGGATTTAGCCTTTTTTAATCCTCACATCACAGTTCTAGAGCTAAAAA
AATCCTATAAAAACTAGTGTCTGAGACACAGAAGGGAGTGGAGAGGTTGG
GACACTAAAGATCATCTTCCTTAATATATTGTAATAGATAATGATTGAGC
AATGATGGGAAGATGACCAGTTGATAGGCTCGACTCGCAGAAGTTGAGAA
GGAATGGTCAAAAGAAGTGATTTTCTAGTCCCGTTATTATCATAATAGTA
CGAGTGAAAGTCAAGAGGGATGAGACGTGTTTAGCAGCAACGAAATAGAC
TGATGGACAACGATGAGATTGTAAAAGGCCTTGTTTGTACCAGCTACACA
GTCTATTGATGGATCTGCATCAGTCTCTTTTCAAGGGAGTAATAGTTATG
AATATATCCTCAGCATTTGAGCGTTCTATCCCATATTGGTTGTAGTGAAA
GTTTGGAGGCACTGAGTGTGGCTTGAATAAGGGTGCACATAATGCACTTT
GGGTTTGATGGTGAATCCACAATTATGACTTCGTAAATTTGAAAATACGT
CGATAATTTTCCCTCAATTCATCACATCGGCTATTTAGAGCGAGAATATA
GAACCTTTTTTAATTGTGTAAATTATTGGATAGTGTTACGCCAAAAAAAT
GCCAAATGCGTAAGAATTTTTGGCTTTGATGGTGAATCCTCAATTATGAC
TTCCTAAATCTAAAAATTCATTGCGAATTAGCCCCCAATTCGTCCAACTG
ATTATTTAGAGCGAGAGCATAGAGCATTTTTAAGAATGTGGGCATTAGCA
GATTGAGTTGCCCGAAATGATAATTTTTTATCTTAGTTTAAATTGGATGA
CTTTATCAAAATGAGAGATACCTATAATGTTTTCCTTTTTTTAAATACTT
TCTATAATATTTTCTTATTAGGCCTTTTGGTTTTGATGGTGAATCCTCAA
TTATAACTTCCTAAATCTAAAATTTCATTGTGAAGTTGCCCTCAATTCAT
CAATCGATTATTTGGAGTGAGATCATAGAGCATTTTTAAGAATGTGGGCA
TTAGCGGATAGAGTTGCCCCAAATGATATTTTTTTATCTTAGTTTAAATT
GGACGACTTTATCAAAATGAGAGATACTTATAATACTTGTTTGTTTTCCT
TTTTTAAATATTAAATTTCATTTCTAAAAAGAGAACTTGTAAAACTTGTA

AAATTACATTTTCCCTCAATTCATCAAATCGGCTATTTAGAGCGAGAGTA
CAGGACCTTTTTAAATGTGTGAATTTTTGGTTAGTGTTACCCCAAATAAA
TGCCAAATAGGTAAGGATTTTTGGCTTTGATGGTGAATCCTTAATTATAT
CTTCCTATTCTAAAAATTTATGAATTTTCCCTCAATACATCAAATCGACT
ATTTAGAGCGAGAGCATAGGGCATTTTTGAGAATGTGGGCATTAGAAGAT
AGAGTTGCCCCAAATGATACGTTTTGATCTCAGTTTAAATTGGATGACTT
TATCAAAATGAGAGATACCTATAATGCTTGTTTGTTTTCCTTTTTTAAAT
GCTTTCTAAAATAATTTCTTATTAATATTGATAAGTTAAAATTACATTTT
CTAAAAAGAAACTTGCAAAATGACAAAATGAAGTGGTAAACGTTCCTCAA
TACACTTGGGCTTTACACTTTATTAGGATAGGTCGGTGGGCCTATGTTGA
TTGCGGTGTCTATTTGGATGGTGTTTTGCGCCATTTCAATATATTTTGGT
ATAATTCATTATGTATCTAGTGTTTGTCAAGCTTTAAATAATGAAATTTC
CTCTGCGGAAGTAAATGGCGTTTTGTTTGTTTTCCTTTTTAAATACTTTC
TATAAGAATTTCTTATTAATGTTGAGAAATTAAATTACTTTTTCTAAAAG
AAAACTTGGAAAATGGTAAAAATGAATTGCTAAGGTTCCTCAATATACTT
GGGCTTTACGCTTTATTAGGATAGGCCGGTGGGCCTATGCTGATTGGGTG
TCTATTTTGAAGGTGTTTTGCCCCATTTCAATATATTTTTGGTAATAATT
CATTATGCATCTAGTATTTGTCAAACCTTAAGTAATGAAAATTCCTATGT
GGAAGCAAATGGCATTTGGAGGAAAGAAACCACCAAGTGGGATTCCTTGA
GGGGATCTTAATAGGCTCATAATCACTGAAAACTTCAAAGCTAGAGGATG
ACTTTAGACAATATACTAAAAACAGTACAGATGTTGGGGTTTCACCATTA
AAAATTATCTATAGTTCAAACCTAGCTCATTATCTATATTACAGAGAAGC
TCTTTTAGACATTTTAATGGTTGGTATTACAACTAAGAAAATAAAGAGTG
CAGATTGCTTGAAGGGAGATTCCTTTTTTCCCCAACTTTTGCATAGATTG
GTTCTGGCTTAAGTGGTTGAAGGAAAGTCGTTAGGACTGTTGGATGCTGG
AGGTGGCAGATTCAAAAATGGGAACAGCTACTTTTTTGTTGGTTTTTTTT
GTTGTGCATGTGTCTTTTTTTTTTTTTGTTTTTTTTTTTTTGGGGGGGGT
ATTAAATATAAACTAGTACCTTTAGGGGTATTCCCTTGTAAAAGCAATTA
ATAGAGCATCGCTAAAGTGAGCTTTTGTCTTCTTATGTAACTCTGACATG
CATGGTTCATAGCCTGATTGTGGTAGTGTTTGCTATTTTCCCATACTTCA
TAAAAGCAATCTTTTAATATTTCTATGCTTATATTTCGAGGTGATTGACC
ATTTTGTTGTTCTTCACATCTAGAGGTTATCTATAATAGGCATATATCAC
CAGCTAATGTTCGCGTTTCTTGTGTGCTTTTATTTGGAACCTGTCTTTGC
TCGTTTAGGTAATAATGACCTGTCTAAATATTTAGACTTAATAGTGATTA
TCTAGTGTTCTCCTTGCTTGCGTTGTTATCATCATCCTACAATGACAATG
AAAACAAAAATCTTTATCTTAGATTCCTAGTGCCAACAGGTTCTATAAAG
GGCAATCTCCCCTATTAAACCTGATATATGTTTGATATATTGAGTTATTT
TATTTTATTTTATTTTATTCTTTTACGATACTTTCCCCTTGAACTCAAGA
CCTTCTACAAGGAAAGTTAGATACATATTTACTAGGTTACAAGCCTTGGG
ATATCCCAACATTCTATCAATGTAGCATTTGATTTCATGGAATTTGAGTC
TCAGAATTGGAATTGGAAATGAATGCATAACAAAATTTGAAAATTTCAAT
GCGTTCATTTCTAATTTCATTTCCAAGGCTCCGATTCCTTACAACCAAAT
GCTACCTAAGGGTCTTTTGATTTGTACCTTTCGCTAGGTTAAGGTCTTTT
GCTTTGTAATATATCTAATATCTTTCATCCTCTGGAAAGATTGGCAATTA

ACTAATTCGGGTTTGAGGGTTAAATGTTTGATTTTTCACCTGACCCTATT
TCAAGTGTCTCATACATTTTATGAACACCAAACAGGTCAAAGGTCAACAG
TCTTGAACTACACACACACATACATACATATATATATATATATATATATA
TATATATATATACATATATACATACATCTTTTTTTTTTTATTTTTTTTTT
TTTATGTATTTGATTTGAGTATCTTGCCCTTACATTCTTAAGAGACATTA
TATTGCCTGTTGCCATGTATCGTGAAATTACACGATAGGAAACTTCACTT
GTTTAATGTTGTTACTTAGTACCAGTTTGGCGTACGGTTTAATAAACATC
AATCTGTTATTTGGGAAAAAAAAATCAACCCGATTATTGTATTGTTTGGC
TGTACGAAATTTAAGCCTTGGAATTATGACCTATTATGATTATAATTTGA
GAAGTCTCACAACAGAATCCTTAGGTAGCATGGAAAATTCCAAATCCAGG
CTTCCAACCTCTTACCTAAGGGTTTTCCGACCTGATTTAGGCTGTGTTTG
GTTGTTTGAAATTTGAGACCTGGAATCAGAATGAGAACCCTTGGGTAGCA
TTTGGAAGCGTGGAAAGCTGATTAAGGTTGTGTTTAGTTGTACGAATTTG
AGCCCTGAATCAAAATTAGTACCCCTGTGGAAAGCTGATTAAGGTTGTGT
TTCGTTGTATGGAATTTGAGCTCTGGAATCAGAATTAGAACCCTTGATTA
ACATTTGGAAGTGTGGAAAGCTGATGAAGGTTGTGTTTGGTTGTATGAAA
TTTTGAGCCTTTGAATTGGAATTAGAACCCTTGGGTAGCGTTTGGAATCG
TTGAAAAATTCCAATTCCATGCTTCCAAACCGTTCCCAAGGTTTTTCAAA
CCATGATTTTTTACTCTTCAGATACTATTCTGTTCCGCCATATTATTCCT
ATTTTGAATGCCCTAAGGCTTCTTCAATTTCATTTTCCTTTTTATGTTTT
GGCAGTGAAATGATCCAGCACAGGGCTTACACACACAAAGTAGACGTCTA
CAGCTTTGGGATCGTTCTGTGGGAACTCATCACCGGAATGCTACCCTTCC
AGAACATGACTGCTGTTCAGGCGGCTTTTGCGGTGGTCAACAAGGGGGTC
CGGCCAAATATTCCGACTGACTGTCTCCCCATTCTGGCAGAAATCATGTC
ACGTTGCTGGGACCCGAACCCTGACGTCAGGCCTCCGTTTACTGATGTTG
TCAGAATGCTGGAGGCGGCAGAGATGGAGATCATGACGACAGTTAGGAAG
GCTCGTTTCAGGTGCTGCATAAGCCAACCGATGACTACTGACTAA
>EUC14489-RA [gene]
ATGGATTTGGTGGAAGAAGTAGGAGAGAGCTCGTCGCCGGCTCGGAGCTT
CGGGAGTTTCAGTGGACACGACATAAGGAATGATGTGTACAATCGATTGG
TGGAGAGTGGGCATGAAGAGGCGGTCTCTGATCCCGGGTTTCGTGAACAG
TTGGATGCTCATTTCAATCGTTTGCCCGCTAGGTATAACGCTTTTTAGTT
GCATTGCTTTGTATTCGTCTTCGATTTTTATGCACCTAACTGTGTGCGTT
TATGCGCACAAGTTTGATCCATTGGAGTACAGAGTTGCTTATACTTCAAG
TTTTTTTTTTTTTTTTTCGTTCTTTTGTTTTTTTCTTTCTTTTGCAGAA
TAGATGCATTTGATTTTATTGGTTTGGGTACATTTTAATTCGTATAATGG
CCTGATATGAAGTTGCTTTTGATGATATGGAATAAAAACTTACCAATAAA
TTACCTATATATCTCTGTATGCATTTATTTTTCTAAAATATACACTTGAT
CGTATAATTGTATATATGTATATAAGAACTGGTTTGAAAATCAATGAGTT
CGTAGTCCAGCTGCAAATCCAACTATAATGATTTAAGATGGTGAGTATAA
AATTAATTTAAAGCTTGCATATCATTTTCTGGTTCAGTTTATTTGTGTTT
GTTTTGGATTCCAAGGTTGTTGCTTTTAATCAAGTAATGCTAGTCTTGCA
CGTTGAGATTGGAGAAGTGAAGTTTCAAGATTTGGAAAATCATTTAATAT
TCCTATGGAAGAAGCCTATTAATATATATATATATATATATATATATATA

TATTTGTGTTTTTTTTTTTCCAGTTATGGCTTGGATGTAAACATGGATAG
AGTGGAAGATGTTTTAGTGCATCAGAAGCTTCTTGCTTTGGCGAAGGATC
CTGATAATCGACCTGCTTTCCATGTCCGCTTTTTGGAGGTAGTGCTTCTC
TGTTAACCAATGAACATATCGTAAATGGTTGTACTTGCATTGTCAACCAA
TTTAGGATTGAGCTCAAGAGTCTATGTAACTCTCTCAGATTTTTGTTTGA
ATCTTGTCTATCATATCATATCTTATTTTTTCTTGTTTGGTGGGAGTTGT
GATGCAAGGTTTCTTGATAAAATGTTCAGTAAATTGAATGAAACCCTAGT
GTAGCATATTGCTATGCAGTTTGTTCATTGCTGGTTTTAAATATATGCTT
GAATTTTCATTCATTCCCTTGCCTCGGACATAATAAGGTGTTCCAATTTT
TGTGCTTGTCATATTTAATTCTCGGGGATCATCTTTTTAATTGGATTTTC
TTTTTTGACATTCTTGGCCTTTTTAGTGAGTTGATGGTATGTCTTCTTGT
TGTCCTTATTTTTATCATCTTTTTAATTAGGTTTTTTTTTTCCCATTATT
ATCCCTTTTAGTGAGTTGATGGCAGATCTTTTTGTTGTCGTCATGTTTTT
TTATTTTTTGATATATATACATATGTGTTTATTTGTCGTTTACACATGCC
GGAGCTCATTGTGACGTATATGTAAGTGAAGAGGATGCGTGGCCAATGAA
TTGATTGACCATCTCTGATCAAAGGGAAACCTCATGATAGGTTAAAAATG
TTCTGTGTTGGAAATGAATGCTTCTCCTCAAATCAATCTTTTCTTCTTTT
TTTTTTGGCAAGCTGCGTAGGGTCCATGTTTTTTTTTTTTTAAATCATAT
ATTTCATATCATATTTCTGTAGCCCAACGCATCCACCCTAAAGTTAATCC
TTAAAGGTTGAAGATATCATTGACTTCTCATTGCCTTGTCTAATGGTAAT
ACACTTCAATAAGTAATAGTTCTTCAAGAAAAGTGGGGTCTTATTTTGAT
GGCATAGCTAATCTGACTTCTTGAGACCCCAAAAATAACATTGTGTCATG
AACCCATAAAGTTTATTGGAAATCTTCACAGAGGGTTGTAACTTGATGGC
TTGGTGAAAAAAAGTTGTGATATGATGTGCACGTTGTTATTTTAGAACTT
AGTGTTTATGTTGGAAATAATTGAAAGCAATTTAGTAAGAGTTGAGCAAT
AACTTTTTATTTTCTGCATTACCGAAAATTAGGGCTTAAGATATGCTTTC
CACAAGTAATTCATTTAAAGCTCTCAAGGAAATGATCTTGTACTTGTGAA
GGATGTTGTTAGGTGGAGTAGCAAGGGGAAGTGAGCGGAGTGCTCTTACA
GGGGATATGGATAAAATGGCTCATGGTTGCAATAGCTCAATTCTATCCAT
GAAACATCTAGGGACCTTGCTACAGCATGGTTTCATGTATTAAAATAGTA
AAATTCAAGTAATTTTTGTATTTGCTACAACTGAATACAGTCTATCAAAA
TGATGGCAATGATTTAATTATATACTGTAATGGTTAAATTAAATCTGTAT
GACGTGGGCTTGGATCTACAAGTGTTAGGAATTGTTTGTATCTGAATCTT
TGGTTCCTCAAATTTTTTAATCACGAGACATATATTTTTCCTTTATTTGA
CATGGGCTCATGATGTAATTTATATCGTATTACTATATTTTCCTTTATTT
TTTAGAAATAACTACTCCATCTATTCTAGGGTGTTCAATTTGGATGTTCA
CTCATCTTAAAATAATTATCAATCAGTAAAAGGAAAAAACATCAATTTCA
TTTTATCCATGGGAAGCATCGAGCATTAGATTATGAATTTTGGAAGGCCC
CATGTCATGCTGGAAAGGATTATACATTGCTTATTGTTGGGTTTAAAAGG
AGAAAGTCCACAGAAGGGAAGTGTGCAAGATATTGAAAGGGACATTAAGA
AATCAATGTGAAATGAGTCTTGTAATAATGGTAATAGCTGCTCATGCAAA
CTGATAGAATGGAAATAAATGAATTTGGCGGGACATGGAAATATCTTCGA
AGTTTTGTTTTGGTTAAGATAGTAATGTGGACAGGATGGTATGTGAAGAA
ACACGGTACGGTGTATTATGTGATGAGGAAAAGCAACAAATTGTAAAACT

ACTGCAGCGGTAAAAATTGCATGCCTAACAACAAAAGCAAAGGAAAAAAA
AAATAAAAAAATAAAAAAAAAACTAAAATAGCAGATGGTAAAATTGATGT
TGGTAAGCGTCACTCAATTCTTTTAATAAAGGGTTTTGTGGAGGTTTGTT
CATTTGTGTTCTATGTTGCTCATAAAGAAGTAGCGAGGCAAGACACATCA
CTTGCAGAGCAATTACTGAATGAATATAAAAGAAAAGATGAAAACAAAAA
AGGGGAAAAAAAAAAAGAAATGTAACAAGTAGTAACATAGATGTTGGTGA
CCATTACCTATTAGTTGTGATGAAGGATTTTGCTTTGGTTTGTTAGATGT
GGCGCATAAAAAGAAGGCCTGCTTATTTTTCTCCTCTATTTTTCTTCTTT
CCTTTAGTTTTTGTCTGTGTTTTTTTTGTGTGTTTGGGCAGGGTAGGGGG
TGGGTTTCATATATATTGTATGTCCCTTTACTGGGGTCAGGTTTGACTCT
TTTTTTTTTTTAACTTCACATCAAAGTTTGCTTGACTATGCGTCCAGCTT
AATTGTGCCCATCATTTTTTGACAATGTGCCATTCAGTCCATTTATTATC
TATATTGTTAGACTATTTCTTGTCTCTTATTCCTTTTTTTTCATTATGAT
AGTTCCTTGGTAAAACAATTTTTAAGTTTCTTACCACTTTCTTTCTTTTA
GTGATTCATATCTTACTTCCTACTCCCCTTTTTATGTTTTATTGTCCTTC
CTGCATTGTATTCTGCTGGTAGAATTTCTGGACTAGAGCAGATGGAGATG
AAGACCAACAATGCTTTGATCTACCTTCTACTTCAAGGCCATCATTTAAT
GTTGATAATGAGGAAGAGTTTCCATCCCCCGACAGGTACTTAGTAATGAT
TTTTTTTTATGGATTTGTCATCTCTCAGCCTTCTCCCTTGTACTTTAATT
ATGTATGCATGTTTTAGTGGTATTGGATTGACTTGAATTATTTGTTCATA
ACAGCAGTTTCAGTTTCTTCTATTTGGTTGTTCGTGTATGGCACTCTCCC
TTTCTCTCTGGGTTATTTTTTCCCACTTACTGTAATTAGGAACAGGAACT
GTGAGAATGACTGTGAACCTTTCTCTATCCTTGCTTTAATTATGTATGCA
TGTTTTAGTGGTATTGGATTGACTTGCATTATTTGTTCATAACAGCAGTT
TCAGTTTCTTCTATTTGGTTGTTCGTGTATGGCACTCTCCCTTTCTCTTT
GGGTTATTTTTTCCCACTTACTGTAATTAGGAACAGGAACTGTGAGAATG
ACTGTGAACCTTGCTCTAAGCTTGAGGACCTTAATTTGGATGTTAGAAAG
AGTTCCAATGGGTTGGAGATGGGAACTCTTGATGAAGACTTTCCCAGAAG
GTAGTTTCTTAGTAAACTTTCAAATTATGTTTCTCTTTTTTGGAACAGTG
GATATTTGGGGGACAAAATATGATTTTATGATGCTTTGGATCAGAAGATT
GATCCAGCCTAGTTTGGTGAATTTACCTTGGAATAATCGAGGGATAAAGC
TAAGACCAAAAAAAATATTTTATCTGGAAAGACTCTCCATGTGTTCATAA
ATTCATTTGTTTACTTCTCCTGGACTAAGATGGGTAACAGTGGTCTTTAG
CTGCTGTGGAGTTTTGAATTCATCCCAACAGGGTCAAAAGTAAAGATGAA
AAATTTAAGACAAAAAAGTAATTACACTACACTAATAAATTTATGACATT
CAATGTTGTGCTCAGCGATTCTTTATGTTTTGAAATCGTTATATAAAAAC
ACTTTCTCAATTTACACAAGCAAACTATAAGCAACTGATCTCTCATTCGG
TTGCCCCAATGATTCTTAGGGGCTAGGGATTTTAAGTTTATGCATAATGG
GGCTATTTTAAGTGATATTTATAAAATAAAAACATTGGTAAAAACCATAA
ATATTTAAGGGCGAAACTGAAAAAAATAAACCTTAACCTGGGGTTTGAAC
TTTGAATATAAATTAAAAGGGTGCTAAATAACATTTTAAAATGTAGGGTA
GTAAGAAAGAATTTACAAAATTTTTGGGTAGTCAGAGGATAAAGATCCTC
CAAAAGTCAAGAGAAGATGCCGTAACTCCCTGGAGATGCCTCAACCAGAT
GAAGAGCATCTAGCCATTTAAGTATAGTATCTCTACTGTCCCATATGCAC

CAAAATCGTAGAAGCAGGATCACTCTAAAATGGTCAAGCTCTTATGAATT
TTGGGCTTTTTACCGTGAACCTCTCTAGCTCTGCCATATTATAACCAATC
TTTCTCCCACGAAGCTTGCTTTTATATTTTTTTTTAGATTTAGATGAAC
AAGGAATCAATAATAAATTAGGACTTTTTTTAATTGTCTTTTTGTTAAGT
GTAGGATTTATTTGAGGGGAACGGATGGATGAGATTGGATTATACAAGTT
TTTCGGGGGAAAATTCCTGCCGCCTACTTCTAAGATAGTTAAATTAAATT
ATATTGTGTACCCTTTAAGTTTTACATGTGCATTTAATTAATTGGCCATT
TTTTATTATTTATTATCATTTTACCCTTATAAATTAGAAAATCAATATAG
TGCCTTTAGGCACGGAAATCCCTAGTACTTTCATACGGAAAAACTGGTAT
ATGACTTTTCAAAATAAGAAAATTTACAGTTTCAATACCATTTTTAAACC
AAAATCATTTTAACTAATAAATACATTCTATATTTGTCAAAAAACAAAAG
TTACTCGTTTCCAAGCCATTGAACCTATTAGACTTTAATGAGTCCTCCCT
CCGGCACTTGCCTCATGCTTCTAAGGAAAAAAGTAGCTATAAGTGTCAAC
ATTTTGTATAGAATCAAAATCCAGTATAGGAAACATCCATCTTGGCGTTT
ATAATAATGTAGTTTATATAGAGGATCAAGTTGTTTAGGGGTCATTTGAA
TATATCCTCTGATTTCATAAAATGATACCATATTACGATGGATCTAGGGG
TTGTTCGGTGTACTTTTACTTATTGAGCAAGTGATTGTGGACAATTGTGT
ATAATAACAATTGTTCCTTTACATGAATTTTGGATGAACATTATGTGAAT
ACTTTTGATAGAATGAGTTTGTTTGGATTGAATATGAAATAATATGAACA
TTTTGACTATATTTTTCTTGCTATGTTACCAATTTATCGTTTGTTTGAAA
AATCGATTATTTTACCTATTTCGCATTTTCAAAAAATTATCTATGTCATC
ATTTTTAACCCAAAAAACTAAAAACTAATCAATTTTAGAACATACCATCT
TTGGAAAATTGTTAAAATATATTGTTCAAAGAAGGAAAACATAACACGGA
CATATAAGTATCATAATCATGGCCTTAGCATTTTAATTTGTTCGAATGAA
CATAAAAGGCCAAATAAGTAACATAATCTTGGTGTTTATTTTAACTTGTT
CACAAGTAGTTGTAGTGCTCTTAAATAGAAGTAAAGAGATATGATTTCTG
TTTATTAATTTACGGGAATGCTAAACTTGTACAAATAATAGAGATCTAAT
TCCTCTTTGTGGTTATTTTTCCAAATAGTCAAATGAACTTGTTCATATTC
ATTGATATCACATTCTCATCAGTTGTCTTTTTGAGAAATGAGTTGATTGC
TATAGTGCAGGCAAGAACTTCCACATGTACCAATTCATGAAGTGATATTT
TCCACCATTGACAAGCCCAAGCTCTTAAGTCAGGTAAATGTATCTCAATT
TCATTATTTGAAATAGTTAAATCCTTTTTTTTTGCGTCTCAAATTTTCCA
CATGTACCATTTCACGGAAAAGCTGCGAGTCATTATTTAAAATAATTTTA
TTATTTTAAAATAATTCATAGATAAATTTTCCACACGTACCAATTTCATT
ATTTAAAATAATTAAATCCTTTTTTTTTGCGTCTCAAAGTTACCTTTGC
TATTTCTTTATATTTACCTTCATGGAATTAATTTCTAGAATTAGTATGAT
TTCAGTGTGGCTGATCGAAAAAATCATGTCTACATTCTAGTAATGAGAAG
TCGAGAACTCAGGTTGAGTTTCTGAATTTCAGCGAGTGCTATGGTCTCTT
CCACATTAAAGCAGCAATTTTAGTAAACAGTGGCTTACAAGTTGAGGTTG
GTGCAGGAGGTTTGTCAGGATATTTTTCTTGTTACGCTAGGGCTGTCTTT
CTTCTAAAATGGTTTTACCTGTAATTCACATCTTGTGGTTTGACTTTGAT
TTGGTGCAGGACTTTATTCCTCTACTTTCTATGAAAATTGATAAAAGAAA
GGAGACATAAGGCTACACATAGCTCTTTGAATCACACTTTCTTGCAAATA
GTCTTATGATTTTAACTTTGATGCGTGATTAGAAACTCCAGAGTTACCCT

GTGCCACATGTACGGAAGTTTCTAGTGAAAAAATAATCATTCCAATTTCA
CATGATGGGCCTTGTTTTGTGTGTGTTTAAGTACCGTATCTACTTTGCTT
CAATTTGAAATTATGTTTGTATCATAGCTTTCGAGAGATTAATATCAGTA
TATAGCATGTAGATTATTTTACCTCTTCATATATAAATAAATTCAATTCT
TACTGTGATAGTGTGATCCTTGTAGCTTTCTGCATTGCTTTCTGACATTG
GACTCAATATCCGTGAAGCACATGTGTTCTCAACAACTGATGGCTACTCT
TTGGATGTATTTGTGGTGGATGGATGGCCTATTGAGGTAGTTCTTGTTTT
TTCTTTTTTCTTCTGAGTCTAGGTACATTGCTACTTTCTGATTTTTCATA
AACAAAATGTATGATGTTTTCCCCTCTGGTGCTGCATCATAATTTCTGAG
AGATATACATACCTTATAGTTTCCCATTACTTTTTGTAGAAGCGCATGTT
ATGCAAATTTTCTTGTACGATCATGCACATACCTCTAATGAACACACTCT
TCAGGGATTGTTTCTTGTGCGAGATGCGGAAAACCAAAACTAATTGTCTG
ACTTACTGATGGGGAAAACAAAATGATCAGAGATATCTCATTACTTCGAC
TTGATTAATATAAAGAAATTGGTTTCCCTTAGTCTGGATTTTAATCAGGA
GCATTGTGGGTATATCTTCTTAAGATGGGCCCGTGCTAGTCTAAATAATG
CTGACTTTGTAATGTTGTGGTCAGGATACAAAGGGTTTGAGTGAAGCTGT
GGAGAAAGCAATTGGTCGAAGCGAGGTAATTATCTTTTGTCTCTATTTCA
TTGTTTTAGTGCTTGGCTTGCTGATGACATAGGCTTTCTTGAAATCTATT
GAACGAAAATGGTTTTTTATGATGATTATCTTTCATCTTTCTCTAGTTTT
TATATTTCAAAAGGTAAACTGGATTTGATTGTCTATGTTTGTACCTCTGC
GGAAGGAAATAACAATTAATTCCTTGATCCCATTCCAACAAGCATGGTTT
CTTGGGTGGTTTTTGGTTACTGTTTCTGTATTTGGTTTCGGTTCTGAGAT
TTGGAATAGAAGCAAATATTAATATATGTACATATATAGTTAAATACATA
TATATTTTTGGAATCCAGATTTTCAGAGGGATCAGAACAAAAACAGATAT
TTTTGGTTTCTATTTTCTGTTTCTATTCCAATTTCCAAGTTTTTGGGAAC
AAAAATGTAAAATCAAAAAGAGAAACAATAACCAAACGCCACCTTAGTCT
CTAGACTGAAGTACTGTAATGGTGGTGGTGGTTTCTTAGCCCTGTTTGTT
CTAAAACTATAAAGGTTTGGCTTATACAACTTTTGCTTCTGATTTGTGAA
GTAGATCGATTTCTGAATTCTGTTGATGGATTTCAGCAAAACCTGGAAGA
AAGACAGAGATAAATACACCCTACTCCATAGGGATCAGATTGAGATTTGA
AAGTTATAGGATGGGTATTATATTCCTTCTGTAACAAAAGAAAGCAATCC
CATTCACTATAATTTTGTATTATAGAAAGGATTTCATTTGTAGCCAGAAA
ACACAAATAGCAACCTTGTAGAACCTATTCTGCCATTATAACGAGAAGAC
TTCAGTTCATGTAGGAAGTTTCTTGGCCAAGTATTAGTTCCTTTTTGGTC
CCTAAAATATCCTCATATTATCTAGGAATGTAAAACCCAGTGCATAAGGA
CCCTCCCAGTTTGGTTCTTGGGAGGGAAAGATATGCATTGCTTTATGTTA
TGCATGTGGAAAGGTGGTTTCCACCACTGTCACTTACGACCTTTGTTAGG
AGGTTCAAACTTAAAAGCATATCGTGCCATATTGTGTCAAGTGGAGTAGC
TTCATTGGCTTATATAATGGTTTCGGAATTCTCAAAGAATCAATGTGGGA
TAGGAATCTCACAACCTCCCTTAAGTGCAACCTCTAGTTTGCATGAAAGA
TTAATAGAGGGAGAAATGAGAAGAATGTGCCAACCCTGACTTGCACAAAA
GATTTAACTGCGATGGAAAGGAAAAGACTTCCTTTAGCTTTGATACCATT
TAGGAGGTTCCAACTTAAAAGCATATGGTGTCTAGTGGAGTAACTCCTTT
GACTTATACAATGGTTTGGGAAGTCTCAAAGAATCGATGTGGGACAAGAA

TCTAACAGCCTTGAAGATGTTTGAAATCAGATTTACCATGCGCCTATGAA
AACTCTTAACTAGAAGAAGAGAAATAGTGAAACTATCACAAAGAGCCCGT
ATATATGCTACAATTTTAATAGAAAGAAATTAAGTCCAAGAAGATTTTGT
TATATTTCTTCCCTGTTATTTTTCTTTTATAAGCTATGTAACGAAGGCCA
TAAATGAAACTGGGTTTTCTGGGGGTTGTAAGGAGGTAGGAGTGTTTTAC
TGCATTAAGCTTGAAAGTGATATAAATTAAATTTAGGAGTCTTGTATTTT
CTTAGTTAAGTATTCCATCATTATCCTTTAAGGACATATGGTCATTGCAC
TAGTATTGTTGATATTATTATCTGTAGGATTGCCTAGAAAAAGATATAGG
CCTATGTTAATAACGGTGGTTATAAACTGTTAAGGGTCAAACTTAAGGAA
GAGAGATGGGATTATGGATATTAAGGTTTTCCTTGCATGTCAAAGTTCAT
ATGGACAAAACTTGATAGAGAATGTGGTTTTAACTTGTAAAGCAGAAGAT
AGTGACGAGCTTGGAGTCGCTGCTGTGTGTAGTGATTAGTTGCTTGGGCT
AATACCAATGGAGCCTTCGGTTTAAATATGAAGAGTTTTGCTTTCAGGTG
TTATTATTATCTGGCTTCAAATTTTTATGAATTTTTTTTCCTGTTTGAAA
CTCCTCTGCCTTCTTTATATATTGGTTCTCACATCGATCCAGCTCTTTTT
CATTCGTCTTATGGTTTCTGTGGCTGACTAAAATTTTATGGTTGTGCTTG
GATTTATTTGATCTATCAAAAAAACTTGAGATCATGGTGTAACTTATGCA
ATGTTGCTCAAGTAATTTGACAAACAAATGTGTAATGAATCTACTGTTAT
GAACAATATAGGGATCGTGGTCTGGTTGTTCACATTCTCAATTAGCCCGA
GACAAAAAAATAGAATTGCAAGAACAATCTGGAGATTGGGAAATAGATAG
AAGATTATTGAAGATGGGGGAAAGAATTGCATCAGGATCTTGTGGAGACT
TGTGAGTTGGGAATTGTTTTGGATAATTGCCACAACGTAAAATATGTTCT
GTTCCAGCCGCATCTCTCCGTTCCTTACCTTTTTTTGTCCTGTATAGGTA
TCGAGGATTATACCTTGGTCAGGATGTTGCTGTTAAAGTTGTTAGATCTG
AGCATTTGAGTGACACTTTGGAAGTTGAGTTTGCTCAGGAGGTAGCTATT
CTAAGGTAAATTGTCCTTGTGTGTGCGCTATTCTTTTCCATAAATCAAGC
ATAAAGTACTCATTGCTAGTTCTCTAAAAATGTTTCCCGTTAATAGCCTG
AAGTATATTTTGTAAAGGTACATCAAATACAGTAAAAGACTTTTTGGCTT
TCATTGCTAGCTTCTCAAAAAATAAAAATAATCAGGATTCTGTATTTACA
TGCATGAAATTTTTTCTTCGGATATTGTTCTCCCAATGGTTTTTGTTGAT
GTCTTTCCTCTTCCTCCAATGGGGTTAGCCCTTCTGTTCTTTTCCTGATT
GACTTGAATTTCAGGACTCATTAATGTTGTATTTGATTTAATGTTTTACT
TTGTTGAAATTGTTTGTACTATGACTTAATTCCTATTCGAATGTATCCTA
CTCCTGAATCTTGTTATCATGATTTTGGAACTACATTGTTTTATTCTTAA
TAATATAACAAGGTGTCCAGATTCACAAATTATAAGCATTATCTGATGAG
GTGGTATTTTATGTGATGCCTATTGGCTTTGATTTTTGATGTATGAACTT
AGCCAGACGGCGGATGGACTGACTTAATTTTTTAATGTTGAATACTTTGA
TGAACATCATCTAAGTTCTGTGTTCATTCATTTTAAGTATCAGATACCTT
TAAATTTATTTCACTTCACCTCAAGCTAAGTTTGCTCAACGGGAGTAACT
TGCGACTCACCATATGATATGCACACATTACAAAAAAATGTGAACTGGTA
TGGGGTTTTCAATCCCTGTCACCTGACTACCTTGCAGTTTAAAATCTTAT
TTACATCAGAAAGAGAAGCAAGAACCTGATAGGTTGTGCACAGGCTAGCT
TTCTCAGTGTTGGATTTATACACTTGATCTCCTTGCGCTTCTCATGAATA
TTCAAAACTAAATCCATGTAGTTTGATAGTATGTAGGAACAACACACCTA

GAAGCGTGTGATCCCTGGTTAGGATGAATTTAGTTTTTAAACTATGTAAA
TGCCCTTCTACTTCTTGAAAAATGGGGGAGAGATTTTTGGTAGGGGTGTG
GGGAATGGGTTTGTGGGGTTTCCATTCACCACCGGGATAGGTGGGGGAGG
CCGTCATCAGGGAAGGTTATGTTATGGGCAGCTTACTATGCTATTGCAGA
CACATGTACCAAAGTAGTTTATTTATGAAAGGAGTTGTTCCACTTGATAC
TATATGGTTTTAGGTTGGAACCTCTTAACACGGTATTAAGAACCTTATCT
CATGGAAGGAAGTTTGATCTCATTCACAAAACTTTCGGTGTCTGTACAGT
TTCGGTGTCCGTACAATCCCGATGTCCTCACAATTTTGGCTCCTGTGCAA
CTATGACTTTTGGCGTCCGTACAATTTTGGTGTCTGATTTTGTTTTTCTG
CAGTGAATAATCGTATCATCTTTTGGTATTCAATTTTATTTCTCCTTCGT
ATTCGATTTGTGATTTTCTTTTGTTGGTATTCGTATGGATTCTGAAAGGG
TTGATTTGCTTAAGTCCATTAATGTGTGATTGAATGGGATATTTTTTTTT
TTTTTTCCATATTGAAGTTATTTGATGAAATTTTTTCTCAAAGGCGAAAA
GATGGGTTATGTTAGTGGTATATTGGTTAATTGGTAAAAAAGCTGAAAAT
TATGCAGTTTTATTAGAAGTTTGGGATGCAAACAATTAAAAAATCATTAC
TTGTATAAACAACTCTGTTGATCATTCGATTGGCATGTAATTGGTCAAGT
ATGAATATGCCAAAGAGGTGTGGGAATATCGGTCAAGGCTGTACACGTAA
TCAAACTTTGCAAAACATTACCAATTAGAATCTAATATTTGAGCACTGAA
GCAGAATGATATGAGTATTCAAGAATTCTACTCCGTTATGATGAATCTTT
GAGATCAATGTGTTTTCTGTAGGCTCTTGACTACTTGTTGGTAGAAGACA
ACCAATATGATATTGAGCTTGAGCATCTGGGCTAGTGGATTTTCTTTTTT
GTTGAAATGCTTAGTTAGCAAACCTAATTGGACCGTATGAAACTAATATG
TCAGGCTTATGTACACGTACCTTCAGAGAAGGTCGATGGTAAGAGCATCT
CTTGCTCATGAGATTTTCCTTAGAGTTTTGAATAGGTTTGAGCTGTGCAT
TAGATAGTTTGACTAGCTCTTTGGCTTGGGATCTGTCTGAAAAGAAAGAA
ATGACCTGGTCTCGATCTTACGTGCAGGCCCATGGCCGCCTATCAAATGA
ATGGGACGATCGACTGAACAACTTCTCAATACGACTATCTAAGTTAGAAG
TCTCGTTAATAAGGCTCAATAAGCCAATCCGGGCTAATATGTCAAAGCAG
GCCTATGTCTTTCTATGTAGCCCGTGATATCGACCTCCTCTTTCCATAGA
AGGGGAAAGAAAGATCCACACAGAAGGAAGGATCATGGGGTTCCCCCAAC
ATACATATACGGGAGTGCAACAATTTAGTAAGCAATGATCAGAACAGGCG
AATCTTGGGTCGAATTCCTGATCTATTCGTTTCAGCGTTAGAGGATTGAG
CTTTTTGAGAGCATTGACGCGCGACCGAAAGAATCTGACGCCACCTCTAT
GCGCCTTTGTTTTTCAATTGGAAGTGGTGCATATTGTGGTTGTTTCGGTA
CTTCTGTAAGCCTTGCTCCTTTATTGAGTAATGCCTGAGTCGCAACATCA
AGGTCTTACCCAAATTGAGCAAGGGTGGCCACTTCGCAAGGATGGTCAAC
CAAGAACTCTACTAAGGCTTGGCCTTTGACAGCCTTTTGACTAAAGTGTT
GCAAGGAGAATTCCATTAGTGCTAAAGCCCAACAGCCTATCCGGCCTCGC
ATGACAGGTCTAGCTAGTAGGTCCTTCATTCAATCTATTTTATCCATAAA
TCCATATCACAAGCTACTTGTTCAAAGTATCATCTTTCAAGGTCTACTCT
TAAAAAGAAAAAAAAAAAAAAAATGAAGGTAAGGGCTCTCCAGAGAATAT
CCCCGGGATTCCGGAGTCTTAACTATTTACTAGATGAATATTCAGATAGA
TCGAAGTCTTATTTCAAGAGTGGCCTATCTCTTGCCTATGAGCTAGTAGG
GAAGGGAGTGTGACCTTCCTATTTGATTCTTTGTTGGGAGGCTTTGGCTT

GAGAGAAAAATAGAAGGAAGTTTGTAAAGAAAGAAAGGAGGGAATAATTT
TATTATTCATTGATACAATTACAAGGGAGCAAACAACTTTAAATAGGAAA
AGAATGACCCACGGCTACTAACCCTAGGATCCCATATAATCAATGAATTT
CCTAATTTATTCAAATTACTACTTTCCTAATTTATCCAAATCTCTAATCA
ATCAAACCCTGACAAAAAATAACCCTAATTTTCCGACACTCCTCCTCAAG
TTGGTGCAAAGATATCAATCATGCCCAACTTGCTTACTTGAAGCTCGAAA
GGCTGTCTCAACAGTCCCTTATTCAAAACATCTGCAACGTGCTATGTGGT
GGGTACAAATGGAGTACAAATTATTCATTCTTCAAGTTTTTCTTTTATAA
AGTGTCTATTAATCTCCATATGTTCGTCTTGTTATGTAGAACTGGATTAT
GAGCAATGCTGATAGTCACCTTGTTGTCACAATACAACTTCATAGGATGA
CTTATTGGTCTTTTAAGCTCTTCTAGAACCCGTTTTAGTTAAAGAATCTT
GCACACTCCCTGTGACATTAATTTTAATTTCGCCTCAGCACTATTTCTGG
CAACAACACTCTGCTTCTTGCTCCTACAAGTGACTGAATTGCCCCCTAGA
AACGTACAATATCTCGATGCTGACCTTCTATCAGTGACCGAACCTACCCA
ATCAACACCTGTATACACCTCAATCCTTCTCTTCTCATTTTTCTTGAAGA
AGAGTCATTTTCCTGGTGTGCTTTTTAGATAACGAAGAATTCGATACACA
GCTTCCAAATGTTCTTCATACAGGGAATGCATAAATTTACTTACCATACT
TACAGCGAAAACAATGTCAGGACGTGTAGGTGACAAGTAGATCAGTCTTC
CAACCAGTCTTTGGTATCTTCCTGTGTCTACTGGAATGCCATCTTTTACA
TCTCGAAGTTTAGCATTAGTTTTTATTGGAGTGTCAGAAGGCGTGTAACC
ACTCATTCCGGTTTCTTTTAAGAGATCTAGAATATACTTCCGCTGTGAAA
TTACAATGCCCTTTTTTGACCTTGCCACTTCTATTCCAAGAAAATACTTT
AGTTGGGCCAAATCTTTAATATCAAATTCCTTGGTTAGGCTCTGCTTCAA
TGTGCTCATCTCTTCTAAATCATCTCCAATCAGAATTATATCATCCACAT
AGATAATCAAGACCGAGACCTTGTCTTTGATAGATTTCTTTGTGAAGAGA
GAATGATCAGATTGTCCTTGTATATATTCTTGGCTCTTAACAAACTTTGT
AAATCTCTCGAACCAAGCTCTGAGAGACTGTTTAAGACCATATAGAGACT
TCTTTAGTTTACATACCTTGGTTCCAAACTTCTCAGTAAAGCCAGGTGGA
GGCTTCATATAGACTTCCTCCTCTAAGTTTCCATTCAAAAAAGCATTCTT
CATATTCAGCTGATTCAAAGGCCAATCAAGATTAGCAATAACAGAGAAGA
GTACCCGAACTGTATTTAGCTTCGTCACTGGAGAAAAAGTCTTAGAGTAA
TCCACTCTACATGTCTGAGTGGAGCCTTTAGCTACAAGACGAGCTTTGTA
TCTTTCCAAAGATCCATCTGAGTTGCACTTCACGGTAAATACCCATTTGC
AGCCAACAATTGTCTTCCTATTTGGCAGATCCACTTTTTCCCAAGTAGCA
TTATTCTCCAGAGTTCTCATCTCCTAAAAAATCGCTTCTTTCTTCTCCTA
AGCTCTCATCTTCTCAAAAATCACTTCTTTCCACTCAGGAACATTTAGAG
CCTTTTGGACACTATTAGGAATGACCACACTAGACACTTGTGAAATAAAG
ACACAATATGAAGATGAAATATTTTTGTAAGATACAAAATCTGACAAAGG
ATGCTTAGTACAAGACCTAACACCTTTCCTAAGAGTAATGGGAAGATCAA
GCTGACTAGAAGAATTTGACTCAAAATTACTTGGAGGAATCTTTGATGAG
TCATTGTGAATTTCATGATTCAACCTTTGATTAGATTCTTGGTAGTGCTG
TGGGATAGTGACTCTTTTTTGTGCTAGATATTTCTTTGAATATACAAGAC
CTTTATACTATTTCGCACACATCGCTATTGTTTTAGTATTTTAATTCTGA
GATGGCATTACCAAAGGTTGTTCATCGGTGGGTTAAATATTTTCAACATT

TAATTCATTTGTTTCCCCAGACTTTGTATTTTCCATATCATTCTCTTTCG
GTAATTGTTTGTGAGAAACATCATCATGAACAAACTCAAAAATTAAACCA
TCTAAAAAGTCATCTTCACTAGTATTCTCCCCTTGAAGATGGGAATTGCT
ATAGTAAGATTGTCCTCGAAAAAATGTGACATCCATAGATACAAATATTT
TTTTGGAAACATGATCAAAACATTTATAACCTCTTTGATTTCTAGGATAG
CCTACAAAAATAAATTTATGTGCCTTTGTCCTTCTAAAAATGTGACATCC
ATAGACACGAACATTTTTTTGAAACATGATCAAAACATTTATAACCTCTC
TGATTTGTAGGATAATCTACAAAAGTTACATTTATGTGCCTTTGGATCAA
GCTTAGAACGTCTAGGATTATGATGATTATGAACAAAGGCCGCACTTCCA
CAAATTTTTAAGTGAAAATCTGTTGTTAGCCGAGTGGCGGGAAAATATTT
GGTGAAAAGCTTCATTGGTGTTTTGAAGTTCAGAATTCTCATAGGCAGCC
TATTAATCAAATAAGTGGCTGTTAAAACAGTTTCTCCCCAAAGATATTTA
GGAACTTTAGCTTGAAAACTTATAGCTTTAGCAACTTCAAGGAGATGTCT
GTTTTTTCTTTCAGCCACCCTGTTTTGTTGGGGTGTATTATTATTACAAA
AATTTTGGTGAACAATCCTTTTTTCGGCAAAAACATTCCTTAACACAGTG
TTAAAAAATCCCTTTCCATTATCACTTCGAAATTTTTTAATCTGAGTCTG
AAATTGTGTTTGAACCATATTATAAATTTTTTTGAACATAATCTTAGCAT
CATATTTTTCTTTTGAAAGGAACACCCAAGTGACTCTAGTATGATCATCA
ATGAAAGTAATAAACAAGTGTTTTCCTAAAAAAGTTGGCATTCTAGAAAG
ACCCCAAACATCACTATGAATTCATATGGTTGAGATGGAAAAACAATCCG
ATGATGTTTTGCTAATTCACAAGATTCACACCGAAAAACAGAAGGATTTT
TATTCATGAATAACTTGGGAAACAAATGTTTTAAATATTATAAATTTGGA
TGTCTTATTCGATAATGCCATAACATAATCTTACTGTCACTAGAAACAGA
AATAGAATTGAAACAAGTCTGCTGATCTTGCTATCCAAAGGTTGATCCGT
TATTAAAGAAGTGGAGGTCACCATCTTGTTTAGCACCCCCAATCATCTTC
TCTGAGGTTAAATCCTGATATTCATAACGAGAAGACCAAAAATTAACTTG
ACAAATATAATCAAGAGTTAATTTGCTGATAGATATGAGATTATATGACA
AATGAGACACGTGCAAAACATTTTTAAGAATTAGAGAAGGGGAAATGGTA
ACTGTTCATTTCCCAGCAATAGTAGCAAAAGAGCCATCAATAGTTTTAAC
TTTTTGATTCCCTACACAAGGTGTATATGAAGAGAAAAATTGTGAAGTGC
CGGTCATTTGATCGGTAGCATCAGAATCTAATATCCAGGAGTCATTAGAC
TTGGTATAAGCACTGGCGGTAGTGAAAGAGTTACCTGAATGTGTTAGAGA
ATAAGATGGGTTAGAATTTCCTAAGGATTGGGACTGAAACATTTTATATA
GGTACTCTATCTGTTCCTTTGTCAACGGAAATGACTCTAAGGAAGATTGT
TGCCTTGGATTTGTGCTGCTAACTTGAAATGCGCGTACATCTTTCCCTAA
CTCGGTGTCACTGCCTGGTTTTTTCTTCTAATTTGCCAGTTTTCCATGTA
GCTTCCAGCAACTTTTCCGAGTATGCCACGGTCTTCTACAATGGCCACAC
CAAGGCCTCTGCTTTCCTCCTCGCCTATCTCCTTTTGAATCTATACCTTG
GGCCACAAGACTAGATCCCTCATCATCTATTTTTTGGTTCATTGATGTTC
TTCAACATGACTTGCTGCTGTGCTTCTTCTCTTCGCACTTATGAAAAAAC
CTCTCTTATCGTCGACGAGGGTTTCTTTCTTAAAATACGTCCTCGAACCT
CATCGAGTTCTTTATTCAAACGAGCCAAGAACACAAATACCCGGCCTCTC
TCAATCTTCTTTTTGTACCGAGCACTATCATTTGAATTTTCCCATTGTTC
ATCTTTAAACATATCCAATTCTTGCCAAACCGCCATTGTATCATTATAAA

AGGCTATTACCTCACGATCGCCTTGCTGCATCTTCCACATCCGAGTGTTT
AATTTGAACAATTGTGAATGATTGTCCAAATCTAAATAGGTTTTTTGAAC
TATTTCCCAGACATCGCGAGTCGTCAGCAAGAACAGGAAGGGCTTTCCAA
CAACTGGTTCCACAGAGTTGATCAGCCAGGTAGTCACAATTGAGTTTTCC
GACCGCCACTGCCTGTACTTTGGATCATCGGTAGCTGGCGGTTTCCCTTC
GCCATTCAAGTGCTTGAGTTTCCCTTTTCCGTCGATCACCAAACGCACTG
ATTGAGACCACTCTAGGTAATTCTTTCCATTCAATTTGTGAATGGTAATT
TGAAGCGAGTTTTCAAAAAACGAGGCATCTGGAAGCTGCGAAGCTCCCTT
TGATGATGTCGCACTACTGCTGGCGTCTGACGAGGCTGACGCACGACGAT
TGGCGTCTGACGAGGCGGAACCGCCGGAGCCAACCATGGTGGCTTTGAAA
TTCATAATCAAACCCTAGGGCTCTGATGCGATATAGAAAGAAAGGAGAGA
ATAATTCTATTATTCATTGATAAAATTACAAGGGAGAAAATAACTTTAAA
TAGAAAAAGAATGACCTACGACTGCCGACCCTAGAGTCCCATACAATCAA
CGAATTTCCTAATTTATTCAAATCTCTAATCAATCAAACCTAACAAAAAA
TAACCCTAATTTACCGACACTGTCCTTCAAGTTGGTGCAAAGATATTCAT
CATGCCCAACTTGCTCATTTGAAGCTCGAAAGGCTGTCTCAACAATTTCT
TATTCAAAACATTTGCAACTTGCTATGTGGTGGGTAGAAATGGAGTACAA
TTTATTCCTTCTTCAAGCTTTTCTTTTATAAAGTGTATTTTTGATTAATG
TAATTTTTGTATACAGAAGTGTCATTAGAAGGCTTACTGCACTAAGTTGG
GTGGAATGCAGTAACAACAACAACAACATCAACAGGTACTCCCATGAAAA
TCTGACAATCAATCTAAAAAATGACCATACAAGCCTCCACTGCCTAATAC
TGATGCAACTGTACCTTCATCTAATTTATTTGGTTTTGGAGACCCAACAT
CTTCTTATCCCACAATGTCCGCCTTTACTGAGTAGTTTCATTGGTTCCTT
GCCACTTAGTCATACGCTATGTCTGCTGCATCTTCAGTAGGTTCGCTATC
TTCTAGTTCTTCATGTGTGTCTTTCTCTGTATAGGTCTTAGATTCTGGTG
CCTCTAATCATATGTCACTTGATTCTTTTTTCCCTTCTCCAATAAGCATT
GTCATCTTTTACTTCTTTAAATCACACGTCATCTCTATCGGTTATGACTG
TTGATGGTACTCTAATGCCGTTAGTAGGCATTGATTCTGTTGTCATACTT
TGGATATCTCTTTTAAGTATTTACTCTATTCCCAATCTTATATTGAATCT
TGTTTCTATTTGCCAATTATGTGATTTTGGTTACTCAGTCTTTTACTCTT
CTACTTGTCATGGATAGGATCCGCATTTCCAGAAGTTGATTAGGACAAGT
TGTGGACAGGGGGGATTATATATTTTGGACGAGCTAAGAGTACTAGTGTT
GTAACTTCCAGTGTTGACTTGTCATCTTTTCATTTAACTTCCTCTCTTGT
TTTTATTTATGACATTCTCGTCTTGGTCATGTTTTAGCATCTCGTTTAAA
GTACTTAGTGTTTATAGGTGCATTAAGAAATTTGTGAGCTTGTGATATTT
TTTATTTGATTGGTTGTAAACTGGCAAAATTCTCTGTTTTATTTTTTAAT
CGAAGTATTTTTTCTTTTCTTGCACCATTTGATTTGGTTCCTGATCTGTG
GGACCTTCACCTGTTGCTACAAAAGGGGGCCTAAATATTATTTATTTGAT
TGATGATTAGACTCGTTATTTGTTGGATTTATCTTATAAAACGTTATGAA
TTCTTTGGTATTTTTCAAACTTTTTGAGCTCTTGTTAAGTCCCATTATTC
TACTATTATCAGTGTTTTAGATTTGGGTTGGGAGTACAATTCTAATCCTT
TTTTTGGATTGCTTGCTTTAGATGAAATATCATCAAATGTCGTGTACTGA
CACTGCGAAACAAAATAGTGTTGCTAATAGGAAACATAAGCACATTGTTG
AAACTTCTCGTTCCATCTTACTGTCTGCCTTTGCTCCTAGTGCATTTTGG

GGAAAAACAGTCCTCATTGCCGTGAACTTAATTAATAAAATCCCATCTTC

TCACTCTTTAGGTGTGTCTCCATTTGAAAAGTTGTATGGCCATGCTTCAA

ATTATTCTTCCTTAACAGTATTTGAGTATACTTGTTTTGTTCTTCGATCG

AGTGTAAAGTGTAGTAAGTTATCTTCTCGTTCTGCTATTTGTGTCTTTCT

AGGTTATGGTAAGGGATAAAAGGGATATTGTTGTTTTGATCCAATTGCAC

AAAAACTGTGTCCCGTCATGTTTTGTTTCTTGAGCATATACATTTCTTCT

CTATTCGTGCAACTACATATGACTTAACTCAATATGATCTCATTTCTATT

GATCATTTCTATGATGATACAGTTATCTCCTCAAAACCCATGCACTACAA

ATATTGACCTAACTCCTGGTTCTCGACCTCGTGTTCCTATTGCACCTTTT

CCTCTATATTATTCTTGCTAGGTTTGCATTGTTAGTTCTACAGATACTGA

TGCTTCTAATGTTCCTTCCCCTCCAATGACCGACGAACCACCTTCTGAGA

TTGTGGATTCTCCTCCTTGCTAACCTCAGGACATTCGTAAGTCCACTCAC

ATATCAGATTTTTTTACTCTTATTATTCAAATTCTTTTGCTTCATTTTTG

CCTCTATTCACTGTTTCTTTGAGCCTTTGTCTTATAAAGAGGCAACTCTT

GATCCCTTTTGGGAACTGGCTATGAGTGAGGAATTCTTTGCTTTGCACAA

GATAAATACTTGGGATTTGGTATTTTTACCTCTTGGTAAAAGTACAATTG

GTTCTTGTTGGGTCTATAAAATTAAAACCATGTTTGATGGTTTAGTTCAG

CGGTACAAAACTCGATTTGTTGCTAAAGGATTTTTTCAGTAGTATGATAT

GGATTATGAGGAGACCTTTGCTCCTGTTACAAAATTGACAACTGTTCGTA

CTCTTATTGCCATTGTATCTATTTGTCAGTGGCACATTTTTCAGATGGAT

GTTAACAGTGTTTTAAATATCGGCCGTTACGTACCGTAACGGCCGATTCG

TACCGGAAAATGGGTAGGCCGATACCGATACACGGCCGATTCCGGCATCT

AGGGCTGAATCGCCCCGTATCGGGCCGATACGGGCAGTTAACGGCCGTTA

CGGCCGTTACGGAGACCGTAACGGCCGTTACAGTCTGTAAAGACCGACCG

ACCGACCGACCGACTCCTGATTTCGCGCACACTTTTCAGATTTCTTCCAA

GATTCTTCATATAATTGAGGAAGGAAGAGAAAAAAGTAGTAGAAGATAAC

CAAACACTTACCGAACGTTGAGGATTTCGTCGAACGTTGAGGATTTCGTC

TGGTTCGTCTAACAGAAACAGCTCATCGCCGCCGAACGTAGCTCTCGCGT

CGCCGGCGACGTCTCCTCTCCTTCGCGTTGCAGCTCGTCGCCGTCGCCGT

CGCCGTCTCGCCTCCGCTCCGGTCTCGNNNNNNNNNNNNNNNNNNNNNNN

NNNNNNNNNNNNNNNNNNNNNNNNNNNNNNNNNNNNNNNNNNNNNNNNNN

NNNNNNNNNNNNNNNNNNNNNNNNNNNNNNNNNNNNNNNNNNNNNNNNNN

NNNNNNNNNNNNNNNNNNNNNNNNNNNNNNNNNNNNNNNNNNNNNNNNNN

NNNNNNNNNNNNNNNNNNNNNNNNNNNNNNNNNNNNNNNNNNNNNNNNNN

NNNNNNNNNNNNNNNNNNNNNNNNNNNNNNNNNNNNNNNNNNNNNNNNNN

NNNNNNNNNNNNNNNNNNNNNNNNNNNNNNNNNNNNNNNNNNNNNNNNNN

NNNNNNNNNNNNNNNNNNNNNNNNNNNNNNNNNNNNNNNNNNNNNNNNNN

NNNNNNNNNNNNNNNNNNNNNNNNNNNNNNNNNNNNNNNNNNNNNNNNNN

NNNNNNNNNNNNNNNNNNNNNNNNNNNNNNNNNNNNNNNNNNNNNNNNNN

NNNNNNNNNNNNNNNNNNNNNNNNNNNNNNNNNNNNNNNNNNNNNNNNNN

NNNNNNNNNNNNNNNNNNNNNNNNNNNNNNNNNNNNNNNNNNNNNNNNNN

NNNNNNNNNNNNNNNNNNNNNNNNNNNNNNNNNNNNNNNNNNNNNNNNNN

NNNNNNNNNNNNNNNNNNNNNNNNNNNNNNNNNNNNNNNNNNNNNNNNNN

NGTTTTACCCTTTTATTTATTTATTTTTTAATATTTTTTGAATGTTGAAT

GGGCTTGACCCGTAGCAGCAACGACCGTGAGAGGAAGCATGGACTAGCAG
AGATGAGGGAAGCGTGGACAACGACGAGGAGCTTGGTTGTTGACAGTGAT
GTCAATAAGTGTCGCTGACGATGGAAATGATGGGCAAGCTTGATCGATGA
TGGTGACGACAGCGAGCGTGGACGACAATTTCGGCAACAACGAGAGCTTC
CCCTTGGACGATGATGACGGCGACAAAGCAACGAGGGCTGAATGAAATTG
CAAATTCCAATTCTACTAAATAAGGGCTAGTGGAGTGCAACTACTACAGC
TGTACTCCTGCTGTAGTGGAGTACGACAAAGCAACTATATTAATATTATA
GTTATTTTTTTTTTCTAATTTTTCTGCTTTATTTATCTTTTATTTATAAA
TAATAAGTTATAACATATTTTATTTATAAATATAAATTATAAATTATAAC
TTATTAAATTTAAATTATAGCTTAATAAAAGTTTACTATAATATTAATAT
ATTATATTCATATATCATAATTTATTGATGAATTTATATCTATCTTCATA
ATTCATATATATCTTTTTAAATTATTTTTTTGATATTTTTGACTGTATAT
AATATTTTTAAAATATTAAAAATTAATGTATGGCCAAAATGCCCCGCCTA
ATGCCCCTGCCTTTTACAACACTGCTTATACCATACTATTGTTGGCAATT
TGGTATATCTTACTATTACTCGCCCTGACATTGCCCATATTGTTTACATC
GTCAATCAGTTTGTCACTTCTACTACGGTTCATTGGGTGGTTGTTCTTCT
TATCCTTCGATATCTTTGGGGCACCCTCTTTCATAGTCTTTTACTTCCAT
TGACATCATCTTTGGAGTTACGTGTATACTATGATGCTGATTAGGCTAGT
GATCCTACAGATCGCAAGGTTGTCACTGGTTTCTGTTTATTTTAGGACGA
TTCTCTTATTTCATGGAAGAGAAAGAAACACATTGTCTCTAGATCATTCA
CGCAGTTGAGTATCGTGCGATGGCATCCACTACCGTGGAGATAGTTTGGT
TACATTGGTTAGTTGTAGATATGGGTGTTTTTCTTGGTTACTTGCAGATA
TGGGTGTTTTTCTTTCATATCATACTCCTTTGTATTGTGATGACAAGAGT
GTTATTCAGATTACTCACAACTCAGTCTGTAATGAACGAACTAAGCTAAT
CGAGATTGATTGTCATCTCACTTGTCATCACCTTCAACATGGCATTATTA
TTTTGCCTTTCGTTTCATCTTCCAGGCTGATTGCCGACTTGTTTACGAAG
TTGCATTCTATTTTGTGTTTCATTTTTTGATTGGCAACCACTTGATGCTT
TTGCAGCCGCATCGTGAGTTCGAGGAGGAATATTAGAGTTATTATATTTA
TTTAGTATTAAGGGTAGAGTATACTTTAGATTGTTTTCTAGATTTTTCTA
TTAGAAGCCTACATAAGTTATCATTCCTAGGTATTTTCTTGTATTCTCTA
CATTCATTATTAACGATAATAAATCTTATACTCTGTTTCTCTCTCTCCTT
GAATTCTATGTTATTCAGTTGTCTTCACATAGTATCAGAGTAAAGTTTGG
GAAGCCTCGTTTGTTTGTCCCCGATTGTTTGTATGTTTGTCTTGTTTCTT
CTGTTCGTTTGATTGTATCACGTGCAAGGCGAGGTTGCACGTGAGGATGG
TTGTTAGAATTGCACATTGAATTTGTGACGTTTCTCAAAGTAGTCTATAT
ATGAAAGTAGTTACTTCACTTGATATCATATGGTTTTAGCTTAGAATGTC
CTAACACCATTTACTATAGGACATGGTGTGATTTTAGATTTTTGGTGGTA
ACCCAAGAGATGGATAATTATGTTACACATTTTCTATAAGATAAATGTTA
ATTTGCATATTGGATTTTGTGTAAGAAGGGGTTATGTCTATTGTTGCGAA
GTTGAGGGGGCAAAGTGATATTGCCTCATGTAGGGGTTTAATTTAAAACC
GCATCCTGTTTTGGGGNTGAATGTTTTATTGGGGGGCACACAAGGAATTT
CTCTACATTCTACGAGTAACCATATTTGCAGAACTATATGGTCTTGCTGT
CACTAATGGACATCTTGCATGCAACTATATTGGAGTTAATTTAAATGTTT
AATTGGATCTTGATTTGAAACTCTGTGAAATAGCACCGCACCGGGTCATG

GAACAATTATAGAAGTCAATGAAGCTTGCCTTTTTGGGGACTACAATTAT
AGTACAGTGCTGAGTCGGTGATTTTATCCATCAGCAGCAAATATATTGTG
TTTCGTTTTTCCAATTATTTAGGACTGGAAGATGCCCTTTTCCCTTCCCC
ATGCATGGCTCTATATTTTTCCTTCGTTGTGGATCGCCAAATTTTTTGAA
TTATGTTGATAGAGTGTTATTACGTATGTATGGCACTAAATTTTCTGTGG
AAGTGTGTATCATTATTCTAAAATCACTTCATTTCCAAGTGGACATCGGT
TTCTTTCTCTTGATCCCATCGTGTATTATATTTAGATTCTCTTGATGCAG
GGAGGTTCAACATAGAAACATTGTTCGGTTCATTGGTGCATCAACAGTGT
CTCCTGATCTCTGTATTGTCACAGGTACATAAATCCAGCATTGTTTTTAT
GTATATAGGTTCTTTAACTTCTGTTTCCTACCTTTCTTTCACGATGTTGA
ATCGCACATCGCGCTTGTGTTACCTGATCTTGAGCTATATATAGAGCTTA
GGGCAATCCTCGTCTTATTACTTTATCCTGAGCTATATATAGAGCTTAGG
GCAATCCTTATCTTACAAGGCGTCTTTTGAGAGTGAGTTAGGCCCTTTGG
TTTCTTACGTGGTATTAGAGTTAGGACTCTCATCTAATGTTGGGCGTATT
GAATCCCATATCGATATTGAGTCCCTTGATATCTGTTTGCAGTGGCTTGT
CAAAAAGTCCACCCTTCACGTGAGGGGTGTGTTGAATCCCACATCGATGT
TGGGCCCTTCCCCAGATATCCATTTGCAGTATTGCGAACGGATATCGTGG
GAAGGGCCCAACATCGATGTGGGATTCAACACACCCCTCACGTGTAGGGT
GGACTTTTTGACAAGCCACTGCAAACAGATATCGAGGGACCCAACATCGA
TGTGGGATTCAACACATGACGAATCAAATGTAGGAGAATAGTCACATCAC
CCAACTTTTGTTTAATAACTTATACGAAAACAATGTAATGTTTGACTAGC
TCTTAGAACGTTTTTATTGTCTTCGTCTTTATTTTCCTTCCAAGATTGAG
CTGCACCTCTGCCTCCATGTTTTCACGGTTATTCATGTTGTTTGTAGAAT
ACATGCCTGGAGGGAGCTTGTATGACTATATACATAGAAATCATACTGTC
TTGCACCTCCCACAGGTGCTAAAGTTTGCACTTGATGTGTGCAAAGGGAT
GGAGTACTTGCATCAGAACAATATAATCCATAGGGACCTGAAGACCGCAA
ATTTATTGATGGATGCTCAATCTGTAAGTATCTGTAATTGAAATACTATC
CCACTTCCCTACTGAAATTGACTAACTCCATGTTGAAATGGAAATGAAAA
AGAAACCTTGCTCAGATTTTGCTGGTTCATTGAGCTAGATAAGAATCGTT
ATTTGCTTTTCTGATGTGTTTTTCCAATTCCGTGCCAAGTTAAAACTTTT
GAATGGTGTTTGTGGTTTTTTCGGACATTGGAGATGGAATTTTGATGAAG
TTCTTATTTCATGAAATTCCCTGTTATACTGATCCCTTGTTACTATATTT
TAAGAATTTTTTTTTTTTTTTTTGTGTCTTAAGGTTGTCAAGGTGGCAGA
TTTTGGTGTTGCTCGCTTTCAGAATGAAGGGGGTGTGATGACTGCAGAGA
CCGGAACATATAGATGGATGGCTCCAGAGGTATGATACACTTTGATTTGT
CAATTTCTTCTGCTGCTCGTCATTAATTTAATATTGCATTAGATAGGTTT
TACGGTATTTTAATCTAATTTTTCCTGATTGCGGATGCATTTATGATTTT
CTCGCACCAGCCTATCCCATTTATCAGCATTTTATCCATACATCTATAGN
TTTATATATTGGTATGTAAAATATATACATATTTGCCCTCTTCTGCTTAG
AAAAAATCTAAAACTACCCTAACACCCAAATTATGTGTCCATGGCCCGAA
TTTTGGACAGGCTGTAATTGCTTCAGTAATGATGACCATTTTACTCCAGA
AAATTGTTAAATAGCTTCCATATTTTCTCTCTCCGTCACTTTCCTTTTCT
TTGGACTGAACGGGGGGTCAATTATTTAGCTTTACCTTCTGAAAAGGGTG
GAGGGGGGATCGAATGAAAATTCCCCTTTATCAATGGAAAATTTGAAAAT

GTGAGTTAAAAATTGGGGGTCCCCAAATTTAGGGTTCTGAAATGAAAGCA
TGACACGTTTTGAGATAATTGTATGGATTTATTGTTTGTGTTTCTGTGCA
TTCTTACTTCGGACGAGATGCATGCTACTTACCTATTAAATTCAGCATCG
AGGAGGGGTGAATTTGAGGTTATTAAATTTAGGGTCTACTTTTTATGCTC
AATATGAAGATTGATGTTCATTGATGTGTTCCATTATTCCTTAAGCCAGG
CAGGCTAGTTAATTTCCTTCTCAGCCTAAGCTACTGATGCCCATTATCCT
AGTGATTATAGAAGCAAATAAACATGATATCTATACTAGAATCAAACTGG
TTACCTAACCCTTTTTTTAAAAAAATAAAATAAATCTTACAACCAATCAC
TTAATTACAACAATTCAATAATTGATTAATTGAAACGTAGTACAATTTGC
GAATTGAAGGACAACCAAACAGGACCTAACCATTAATGCTGATATTTGCA
CTCTACCTCCACTGCAGGTTATAAATCATCAGCCCTATGATCATAAAGCC
GACGTATTTAGTTTTGCAATTGTTCTTTGGGAGCTTGTGACTGGCAAGGT
TTATTCTTAATCTTTCTAAATAGTAATATAAAATGCTCTTTAGGTAGTGT
TTGGTAGCACAAAATTTAAGACTTAAAATTGGAATTCGAAATGACTGCAT
TCCAATTCCATGAAACCAAACGCTACCTTGGAGTCTTCAAGTTTGAGGAA
CAAGGAAGATTATATTTACTCATTTTGAGATTATAGGTTCCATATGATAC
CATGACTCCTCTACAAGCTGCCCTGGGAGTGCGGCAGGTTTGTTCCTTAT
CATTTCATGAAATAATAAGCCTTTGCCTTTATATCTTCTCTCTTTTACAC
ACAATAATGTCTTTTTAATTTTAATTTTGGACTAGTTATTAGCGTAAATT
GACTACTTTCATATTGGATATAACTTCAGTACTTGAGGCCAATCGTGATT
TGACGTGTGTACAAGATGACAAAATTAAGGAACGTAAGCATTGGTCTTTT
TTTTGGTACACTGTTCGCGGATCACGATTGATTCCAGTACCATAAGTACT
TGAGGATTAGTACAAAAGTTGTATATACTTTGGTATTGGTTTCCCTTATT
TGTTCAAGTAAGCACTTTTGCAACCTTAATTTTAAAAAGTGGGATATTGA
TAAAGACATTTTTTATTTTATATATATATATTAAATGGGTTAATGGCATT
TGTGGTGTTTCATAGATGCCAATTAGATCTTCGTTTTTTGAAATAAATAC
ATTGTGTTCTTCACATTTTAATTAGCCCAAATTATAATAGTATTAGTCAA
TCATAACGGGCAACCGTTCAATATTTTTAAATGACATTTATATTCTGAAA
AAAATTTAATTAGGACTAATTTTATATATTAAATAACTTAAGGGGCTATC
GCCTTTTTTTTTTTGTTGCTTCCTCTTTATCAAGCTCTCTCTCTCTCTGC
AACTTTACCTCTTCGTCCATTGTTGAAACCCACAATCACCACACCTACGT
ACAATAAACACCAACCAACCAACCGGCACCACCATCACCGCCACCTCCAA
CCCACCACCATCACCGTCGAGGACGATGCCGAGGACGGATGACGTCGCGT
AGTGGCCGTTGTTGCGGAGTTTGGGCCGATGACTGCTCAAGGGGCCATTA
CCTAGAGAGAGGACCAAAACATCTTCGACGCCGGTGAAGAAAGGAAAGTC
ACGTTTGTTGTGCAAAATGTGAGTGACGGCGGCGACAGGATTGTTCATGA
CGAGACCGCCGTCGATGGCTAAGCATGAGGTTTTCCCGTCGACGGAAGTT
AGGTTCATCGGCTTGAACATGCACGGGATGGATGACATGGCGCGCTAGAC
CATCCAGAGCTCGAAGTTGTAACTCGGTGACTCGGACGTGTCGGCCGCGT
GAGAAAACGAACGGTGCAGAGCTATTGAGCTCAAAACAAGAGACAAGAAA
TCAGCGATTCGAGAGTTAGACTCGCCGGCTTTAGATCGGATCTGGTCGTC
TAGGTGGACCAAAGCTGATCCAGAAACAATGCCGGTGGTTCCATCTCCGT
CTATGCTTTGGATACGTATCTTCTTGCTCTCGTAGTGGAAAAGCCACTTT
CTGCTCAAGCTTGGAGAAGATCTCCGATGTCACCTTACTCAGTTCCATCG

CCGTCGCTGCACTTTAGAGAGAGAAAGAGAGAGAGAGAGTGTATAAGAGA
GAGAAAGAGAATAGAGAGAGAAATAGAGTATATATCTTCGCCGTCGTCAC
GATGGATCTTCAGCTATCAGTTGCATCGCCGTCGCTGCACTTTAGAGAGA
GAGAGAGAGAGTATAAGAGAGAGAAAGAGAATAGAGAGAAATAGAGTATA
TATCTTCGCCGTCGTCACAATGGATCTTCAGCGACGACGAAGGATCGGAA
GATGATATTCGCCTTCGGAGTTGTTCGCGGTGGGGTGGTTCGCCGTTGGT
GGTGGGTTGAAACCTTTGGGTTGCAGAGAAGAGGAAGAGTAAGGAAGGGG
GTTAAATGAAATATGACAATTACATTTAGACCCCAAAATTTTGGATTTTT
TTTAAATAAACCCTAATGAATTAGTTAGAGTAAAAAGACGAAATTACCCT
TATTTTCGGACATAAAATGACCAAAATGCCCTTAATTTAAATGGTTGACC
GTTAAATTTGACTAACATCATTGCTATTTAGGCCAATTGGAAACGAAATT
GAAATGTGAAGGATCCAATGTGTCTATTTCCAAATATGAGGGTCTAATTG
AACACCCGCTGCATTTGTTCGGGATAAAAAATGTCATTAACCCATATTAA
ATAGAACTTTGAGTGAAATACTACATCAACTGTCCAATTTATGTAATTTT
TATATTGTTTTAGTTTCTGTATTTTTTTTTTCTTCAGCCGATCCAAGTCC
AATCCATTTAGTCTCGTAGAGGTTCTAACTAGGGGAAACGCAGATCCCAA
ATTTGTACTCAAATTTCTCTCTCTGACACATGCACACATGCGCTCACTCA
CCAAAAGAAAAAAAAAAATCATAAATAATAAGAGGCCAACCAATCTACTTG
GGTTGATGATGATGTTGAATTTCTTTATGCTTAGGGCCTTCGACCGGAAC
CTCCAAAGGAGGCACACCCTAAGCTGGTGGACTTGATGCAGAGATGTTGG
GAAGCTACTCCATCCGATCGACCATCTTTCTCTGACATAAGAATCGAACT
TGAGCAACTCCTCATAGAAGTCCAGGTAAAATATGGGACATAGGTAGTGT
TTGGTTGCCCAGAATTGGAGCGGAGCCTTGGAATTGAAACTCAAAATGGG
GACCAAAAAAATCTTCAAATTTATTTTGAATTTCAATTCTATGACTTCAA
TTTGTGCTTCCAAATGCTTTGTATGTTCTTTTTATATTTTTATTTTCATT
TTTTTAGATGGCATTTGAGTAGTACTCTTTAAAAATAAAATAAAAAAAACG
AGTAACATAGGAAAAAAAAAAAAAGACACAATATTATTTTTAAATTGGTAT
TATTTTGATTTTCATTTTTTAAGTGCAATATTGTGATTTTCAAGTCCATG
CTTTGGATTCCTTGTTTCTAAACGTTTTGTTTTTATGTTTCCTGTTCATT
AACTTGGTTGTTTGAATAGTTTTTTTTTTCTTATGCTTCTGAAAACCTGA
AACAAAAATTGTAAGTGTGAGAAAAAAAAATGTTCCTATTTTGTTCCTATT
TGTATGTTTTTATTCTATGTTCTCTAGGCACGGAAACACTAAGGCATTGT
TCTTTTCGATGTTTTTCAGAGTGTAGAGATAATGCCTTAGTATTATCTTC
CATAGAGTTTGGTAATGTTTTTCTTTTTAAGTATTTTTTTTTTTTTTTTT
TAATTCTAAGTGAAAATGTAATTATTTTTTAGAAGTAGTGTTTGTTTTGC
ACTTAAAAAAAATATTTTTAAAAATAGAAAATGTAAAAACATATTTTAAGT
ACTTTTTCAGAAAGTAAAAAATAATTATTTTTAACTATTTTTTCATTATA
TTATTATAAGAGGATTACTAGAGAGCAATGGACTAGGACTTCATCGATAT
GTTTCCATGCATCCATGGACTAGATCCTCTCCATCGACATTTTTAAAGCA
TATACAATTATATTTATTTATTTAATTTTTTTGGATTAAGAGGCCAGTTG
TAGGAATAATAGTAAGATGTAATCACTCCAATATGAAACAAGCTTTTTAT
TTTTTGCTCTTTCTTTGAATAGAAGTATTTTTTCATTAAATTAACTTTTT
TGCTTTAAAAAATACTTAAAAAAGCGTGTTAATATTTTATGTTCAAAAGC
AAAAATTACTTCGAAAACACACTTTTTTATTTTTATTTTTCGTAGTAAAT

CTTTTCCTTTATTTTGTGTTATTTTTACTTTTGCAGGATACCTCAAAGGC
ACAAAATGGTTGCTGCTGA
>EUC13785-RA [gene]
ATGGGGAGTGGATCCACGTTCGTGGATGGGGTTCGTCGCTGGTTCCAACG
CCGCTCTTCATCTTCATCGACGGTCATCGCAAACAATGAAAATGTCCGCG
TAGCTGCGACCACCAACGATTCTGAATCGCAATCATCTAGTACACTAGAA
CACGTAAAAGATCAGCTTAATATTGTTCGAGACTTTGATATCTCTGCTCT
CAAGCTTGTTAAAGTCCCCAAACGCATTGGTTTCCCAATGGATCCTCACA
AAAAGGTCTTTCTTTCATATTCTCCTCCTTTCACAAGCAGAAGACGCGAT
CAGATCTATATGTGTTGATAGTTATACATGGCATGTTGATCATTGATATA
TTAAAACCGATTTTCTTCGATGTTTCGATTATTCTATATTTTAATAGATC
GACCTAATCCAATCCCAATGTCAACTATACGAGAAAGTGCTAAACTTATA
CTGATTGTCGCGTAATATGTTGAGGAATATTATATTTGTGAATCTGAAAA
ATATAAATGTGTTTTTTGTTTGCATTTGCCTACGCGTTTGGTTGGTTCAC
TGCATTTATCATATTGAAATATAGTACGGCACACTTTATCTACTTTGCTC
GCATCTACCATTCTGGAAGATAATAGGATGCAAATTTTGATTTCATTTGC
TGTATCATTTTTGGTTGGGCCTGGAAATTTGTGGAGAAGTTGTACACGCA
TATCTGTCATATGAATTCGACATTCGCGGTGTGATTGTTGCTTTGGGCAT
AGTTTTTATAAAGTGTTTCACAAGGTTATGAGATGAACAAAACTGGACAA
TTTTCTTACATTTTTTAATTAGAAAAAAAGCACATTTATCCTCTTCGGTG
ATGATACTATGAGTTAGTTCTTAGAGAACCAATGCTACTGTAGTTTGAGC
TTTTCAGAATCACTCGGACATTCTACTTTTCAGAGTATAGAGAATGGCCT
CTGAGGATGATAGTTTTAAGCACGTTACATTCCTTAGATGCATATTTTAA
ATGTAAGCAATAACTATTTGATGCAAAGTTGTTTATTTAATTTTCTCGTG
GCATAGAGTATTAGAGTACAAAGAAATTCTGGCTGTTACCTTTGCCTTTT
CTTTGATGTATTTATCCTCCACTCTTCTTTAGACATCTTTTCACTGGATG
TTAATGTTTATTGTTTCTAATGGGTCTTCTTGTTTCTTCTTTTGTCCTCT
TTTTCATTTCCACTTGTTGGTACTCAGAACACATTGGAGACAGAATTTTT
CACAGAGTATGGAGAGGCAAGCAGATACCAAATTCAAGAGGTTGTTGGGA
AAGGTAGTTATGGTGTTGTGGGTTCTGCAATAGATACCCACACTGGAGAA
AGGGTTGCAATCAAGAAGATCCATGATGTCTTTGAGCATGTCTCTGATGC
CACACGAATTCTTAGAGAAATAAAGCTACTTCGACTACTTCGACATCCAG
ATATTGTAGAAATAAAACACATTATGCTCCCTCCCTCTCGTAGAGAGTTC
AAAGATATTTATGTTGTGTTTGAACTGATGGAATCTGACCTTCACCAAGT
AATTAAGGCAAATGACGATCTTACTCCTGAGCATTATCAGTTTTTCCTGT
ACCAGCTTCTACGTGGCTTAAAGTTTATACACACTGGTAGGTTCTCTGGA
GCTAAATCATCACCTCTGTTATTTTTTTCTACTTCTTGTTCATCATCAAC
TTGAGTTCTTATTTTTTTTACAGCTAATGTTTTCATCGAGATTTGAAGC
CTAAAAATATTCTTGCTAATGCTGATTGTAAGTTGAAGATATGTGATTTT
GGCCTTGCCCGAGTGTCATTTAATGATGCCCCATCAGCTATTTTCTGGAC
AGTATGTTTGAATTTGTTTCTCAACTCTTCATACACGTGAGCTGCTTAAT
ACCAAGTATTTTATTGTTTATAATCATCTCTATTGCTTCCTTTGAAGGAT
TACGTTGCAACTCGATGGTACCGTGCTCCCGAGCTTTGTGGTTCCTTTTT
CTCCAAAGTAAGATGAAAATTTAATGTATTAATACCTACTTTTTTTTTT

TTTCCATTTTTTTGTATTTGCGGTTCTTGAAATAACTTTAACCTTGTCTA
GAATGGATGTACTTGGACTTTTCACATGGTTCATAATGCCTACGACCATT
GATATATTTGGCACTTGCCACTCCTTCAATATATTTTGGGCCTTTTCACC
CCAACTCCTAATAGAAGTCAATAATTGATGAATAAACCTATTTTTGTTGA
CTTGAAGCAAAATTTGAAGTGTTGAAGTTGAAGGGTAGCTTAGCCATTTA
CATGTATTGAGGGTTCTCTATGTGGCCCTCTGTACAACTATAAATAAAAG
GGAGAGGGTGACAATAGGGAAAAGCCATTTCGTTCTTCTTTTACTTTTTT
ATTTTAACACGGTATCAGAGCAGGTTAGATTGGGAAATAAATTGCATAGC
TCTGTTTTTTTTTTTCCTTCTTTCCCTTGTCCATTAAAAAAAATTGCAACCG
AAAAATCTAGATTTGTTCAGTACCCTAGTTGCCATTCGATCCAGCTCTAC
CACTGTTCATATCCGTTCTTCACAACCACTTGGTTGCCACTCGTCGACTG
TCACCGACCATTTTAGGAACTCGCCACTTCTCATTGGCGTCTATTGCCTG
TGTAAAAGCCTCCATCGCCAGATCACATCTTGTTGCACTCTTGCCAGATC
GAGCCCTAACACTTGGTTTGTTCTCCTCTACGATAAACGGTCATCGACAC
AATAGTACAAGCCTTCATGACTTCTTCATTGTTCGATTGCCCTGTGACTT
TTAGAAGTTCGTTTCTCAAAAAAAAAAAAAGAACCAAAAAATGTTTATTTT
TGTACTTTCAATTTTTTTTTTTTCAATTTTACTTGTTAGTTTTCCGTTTTA
ATTCACTTATGTTGTAGTTATTGTTATCCTGTTGAACTTTGGCGGATGAC
CCATTTGTTATTTCCAACCACCCGCCTTATTAATGAGGTTAGCCAACCTC
CTTCCTTAATAATGGGGGAGTCCACCCACCTTATTAATGAGGTTAGCCTA
CCACCTTATTAGTGAAGGAGTCCACCCACCAACCAACCATGTCATTAATG
AGGGACCCTAATATAAACACTATAGAAACTAAATTATCCACTAACACTTC
CCATCAAGTTGGATGATATATACCGTATGCTCCCAACTTGTTACAGATAT
ATTTTATCTGAGAACTGCTAAGCGGTTTAGTGAATAAACCAAGTAACTGA
TCATGAGAGTTAATATGTCGAGTGTGGATCAACTTATGTAGTAGTTTCTC
TCGAATGAAGTGACAATCAACCTGAACGTGTTTCGTTCAATCATGAAAAA
CTGGTTGTGATGCTATATGGACTGTTGCTTGATCATCACACACTAGACTC
ATGAGAGGAGAATGGTCGAATCCAAGTTGTTTTGGCATTTTCTTCAACCA
AATCAACTCACACGTCGTATGAGCGATTACTCGATACTTTTATTTGTCAC
TTGATCGGACTACCACACATTTCTAACTCTTCCAAGAGAGCAGATTACCT
CCGACAATTACACTGTTGCCTGTTGTAGAATTTCTATCTAAAGAAGATCT
TGCCTAGTCAGCATTTGTGTATTCTTGTATGTGTGTCCATGATTGTTATA
CAATATACCGCATCCTGGTGCACCTATCGAAGAATGCCGACAACTTCCCA
AGGAGTCGTTCTTGGATGATCTAAAAACTGACTGATAACAATAACTGCAA
AGGATATATCTGGTCTTGTAACAATCAAATAATTGAGCTTCCCAACCTAA
CATCTTTGTTTTACTTTACAAGTCAAGAACATATTTCTTCTGAGAAAAAG
GTAAATTCCTTGACTTGATCCGTTGATTGGCAACTTCTACACCCAAGAAA
TGTTTCAATTGTCCCAAGTCTTTAGTCTGAAATTTATCATGCGAAAATTG
TTTGAGTGCTTCAATGCCATTGTGGTCGTCTCCTGTAATCACGATGTCAT
CAACATACACAATCAATAATACTCTCTCTATGATACCATATCCGTAGAAC
GTTGAATGATTGACTCCACATCGTTGAAGACCAAACTCAGTAATCACGTC
ACTAAATCTCCCAAACCATGCTCGAGGAGACTATTGAGGCCATAAATCGA
TTTCATCAATTGACATATCATTCCTGACTCCCATTGAGCAACAAACCTTG
GAGGTTGCTCACATATACCTCTTCAAGAAAATCATATACCTCTTCAAGAA

AATCATCGTTCAAGAATACATTGTTGATGTCTAACTGATTAACGACCAAT
GAAATGTGGCTGCAAGAGAGATGAATAGGCAAATAGTTGCAAGCTTGGCA
ATATGAGAGAACGTCTCCAAGTAATCAAGGCCATTGGCAATATGAGAGAA
TGTCTCCAAGTAATCAAGGCCATAACCATGGATGTAACCTTTGGTAACTA
AGCGTGCCTTGAGTCGATCAATGGTGCCATCCGGGTAAACTTTGAGGCAT
TCTCCCAATTACAATCGACAACGGTCCTGCCAGGTGGGGAAGAGATGAGA
TCCAATGTACCATTTTGGTGTAAGGCAAGTAATTGTTCCCCCATTGTAAT
CTTCCAGCCAGCGTAATAGAGAGCCTCTAAAACAGATTCAGGAAGAGAAC
CAAAGGACAAAGAAGTTACAAAAGCAGAATAGGAAGGGAAAAATGTGCAT
ATGAAACAAAGTTAGATATAGGATGTTCAATACATAAACGCTTACCCTTC
CTGACAACAATTGGTATGTCCAAATCACACGGTGGTTGGGCAGATAAGGG
AACATCCGAGGATGATGCATGTACAATAGGCACCGTGATGGAGGTCGAGG
GGGAAATGAGACTTTTGGACGATGAGAGTAAACTTGCAAGGGAGGACAAG
GGGTGTTTAGGGAGATTGGTCTCCGTTGGGGGTGCAAATAAATTAGGTGT
GGGTAAGCTAGGTAAAGGAAGTGTAACATCAAGGTCGGTTCTAGTGTGAG
GCTTAAAATAGTAAGGAGTGGACTTAAAGAAGGTAACATCAGCGCACACA
AATGAACGGCGCAAAGACAGACAATAGCAATGATACCCCTTTTGGGCGCG
GGAGTATCCAAGGAAGACACGTTTCTGGACACGAGGGTCTAACTTGTCTT
AACAGGGGCAAGTTGATGTACAAAGTAAACACAACCGAAAATACGAGGGG
GTAGGCCAAAAGAGTGAGAGCGAGGGAAAAGAACGGAATGTGGAATTTTT
CCATATAGGGTAGAGGAAGGCATTCAATTAAATAAAATAACAAGCAGTAA
GAACCGCATCACCTCAGAAGTGCTTTGGAACATTCATTTCAAATGGCAAA
GTATGATCGACCTCAAGGAGATGTTGATTTTTGCGCTCAACGACGCCATT
TTGTTGGAGTGTGTGGGCACAAGATGATTGGTGAACGATGCCCAAATGTC
TCATAAAGAGTGAAATGGATGAGAAATTAAGGGTGAAATGAAAGACGTGC
AGTTGAGGTACCTTGTTGAGCTAGAGATGTAATGGAGGTGGAGAACTACT
AGGTGGCCTCAAACTTAAAGAACTGACATACTCGTCATCGGATATCTCCC
TAGCAGTTGGGGATCAGACAGTGGAATAAGATCGACTTGATCAGATGTCA
CCACTGCATGAGCATACCTTGGAAGACGACCAACTAAATCCCAACAAGTT
TCGCTGGTATGATTCTTCTTGTCAGAGAAGGAATACTGACAATGGTGGCC
ACAACCACGACCACCACGAGAGCCTCTACTACGGCCACCTAGAGAGTGAC
TATGGTGACCACCTCTAGACTGGTCGCTATTCAATTTTCGAGTGGTTAAT
ATTGGAGGTACAGTTGAGGACACACCTAAACAAAAAAATTAATATGACTA
ACAAACAAACCACGAAATTGTAATGGAAGATATTCTGAACTAAATTAAAA
CATGTGACAAGGCAAACTAAAATTTAAGTTAGTAAAGACATTTCGTTTCT
TTTTTTAGGGCATTTTTTTTAGATAGGAAAAAAAATTAAAAGGAAGGAAG
AAAAATGTATATAGAACAAATTAATCAGGGCTAATTACTCATTCAAACCT
GCTTTTGATACCATGTTAAAGTAAAAGATTCATAAGGAAGGCTATAAACA
AGTCTCATTTGCCACTACCACTCTACCATTTATTTATAGAGAATGAGTTC
ACCAACCAACTATCTTATTAATGAGGGAGTCCACCCGCCTCATTAATGAG
GTTAGTCAACCATCTCATTAATAAGGAACCCTAATACAAACACTATAAAA
ACTAAAGTATCATCTTACAGTTTGTTTGTACCGTTTAGTCTGCAGCCAAT
TATGGTTTCTACAGTGCCGCCTTTGCCGTTCACTACTTGGAAGTTGACTG
ACACCTTGTCACATTTGCCAATGAAGGGCCATCTAATTGTCATTTAACCT

CAGTTTCTGGGTTGAAGATGAATGTAGTCCTATCTAAGAGCATTCCGAGT
TTCTACAGTATAAGGAATCACATCATGATGTCTCTGCATTGCATCTTTCC
CCCATTGAGCTAATCCTAAAGTGTTTTTTTTTCTCAATAGTCCTTCTAGT
TCTAAACCTTGGGTCATAGATTCAATGCCACAAAACATATGTTAGGTTCT
CATCACATGTTTACTCATTATCAATCATTTTCCTCTTTACCTCATGCCAC
CTTAGCAAATGGTCCAACTACTCCTATAAAGGGAATTAGGAGTGTGAAAC
CTATTTTATCTCTATCTTTGTCAGCTGTCTTGTGCATTCCTTCGTTTCCT
TTTAACTTATTGCCTGTCAGTAAACTTACCAAAGATCATAATTCTTCAGT
TTCTTTCTTTCTTGACTTTATGGTTATTTAGAATTTTAGGACAATGCAGA
CAATTGGTAAAGGGCGTGACTCCAATGACATGTACTATTTTGCCTGATTA
GTTAATCCTTACTGCTCCTTTTGCATTTCCTCTATAGCTTTTCCCCACCA
AATTCATTGCTGACTTGGCCATCCGTTCTTACCCAATTTGAAGCTTCTTG
TCCTTAGTTTGAGTTGTTTATTTGTCTTCTTTAGATTGTGCCTTGACAAT
TGGGAAAATGTCATCATTTGTCGTTTACTTCATGAGTTAGTAAGGGAAAC
AATTCCTTTATGTTAGTCCATTTTAATGTTTGAGGTCTGTCTCATGTTTC
TTCTACACTCTATTGTCGATACTTGTCACTTTTGTGGATGATTTTTTCAT
AAGTCACTTGGCTTTACCTAATAAAAGATCGTTCCGAGTTCTCTATTTTT
TGTGCCTTTTTATGCTAAAGTCAAAACCAAATATAATGTCCTTTTGCCTA
TATTTCAGAGTGGTATTGCCAAAGAGTATTTTCAAATCCAATTAGTTCTT
TTATGGCCCAATCCGGTATAGTTCAGTAGTCATCATATGCTGACACTCCA
CAACACAATGGAGATACTGAAAGAAAGAACAAACATCTTCTTGAGGCTAC
TCGTGCTTTCCGTTTTGGTTTCAAATGAATCTTCCTAAAAGCTTTTGGGG
TCACGCAGTTTTCAAGGCGTGAAATTTAATTAATTGCCTACCATCTTCTA
CCTTGCATGGGAAGGTCCCACAATCTCTTTTATTCCCCTCGTTCTCCTCC
TTTTCTCTTAACTTATTTGGTTGTGTGTTTTGTAAAACTTGCATTGGTAA
AGACAAGTTAGATCCTTGTTCTATCGAATGTGAATTCTTGGGTTATTCTC
GGACTTGAAAGGGACGTTGTTATACTCCATTATTTTAGGGTTTTTTTGTT
TGGTTTGATGTTACTTTCTCTAAGCTACACCTTGTTATACAAATGAACAT
ACTTTCCCTTATGCTGCCCTTGACACTATGCTTCCATTATACCTAGTGTT
ATTCCATATGTTCCCTCGCCTCCTCTTGCCAACCCTATCCCATAAACTTG
TTTGCCCTGACTTGTAGGTTTATACTTGTCAAAATGTGGTGGCCCCCCCA
ACTAATCTTCAATTAAATACTTCACCAGTTACTCTATTGCCCTTTGATCC
TACTCCACTGTCCTTGGTCTATCTGATTTGGTCATTCCTATTGCTATCTA
GATGGGTAAGCTTTCTTGCACTTCACATCCAATACCTAACTTTGTATCAT
AAAAACATTTCTCCCCTTCTGTTCGTATGTTTCTCTCCCTAAGTCCGTTT
CAGAGGCTCTTTCTCACCTAGGTGGAAGAATGCAATGGAGGAGGAGACAA
GTGCAAAGTGCCTTACACTAGAATGGAACTTGGGACATTGTATCTCTTCT
ACGTGACATGATTGTTGTTGACTGCAAATGGGTATATGGAATTAAAGTTC
ATCTAGATGACACCATTTATCAATTCAAGGTCCAATTAGTTGCCAAATGT
TACACTCAGTTATATGCACTCGATTATTTTGAGGTTTTCTTATCAGTTGT
GATTCTCACTTCTATTCGCTTGTTTATCTCCCTTGTTTCCATATATAAGT
GGTCGTTGCATCAATTGGATATCAAGAATGGATTCATCAACGACAACTTA
CTTGAGGAAGTTTATATGGAGCAACATCTGAGGTTTGTTACTCAAGGGGA
GTCAGGGTTGGTATATCACCTAAACTCCATCTACAACCTCATGCAATCCC

CTTGTTATAGTTTGGAAGATTTAGTAATGTAGTCAACGAGTTTGGTCTTC
AGAGTTGTGGTGTTGATCACTCAATATTTACAAGCAATGCTACGAAAGG
GAAAATAATCTCTATTGCATATGTGGATGGTTTGTTATCGCATGAGATA
ACCACGATGATATTTGAGCACCCGTGTTTTTGCATGGCAAGTTTAGCTAA
AGATTTGGGCTAGTTGAAGACTTGAAGTACTTATTGGGGGATTGAAGTTG
CTCGGTTGAGTTAAGGCATTTATCTATATATATTCTTGACTTTTGGGTCA
GTTGAAGTGCTTATTGGGGATCAAAGTTGCTCGGTTGAGTTAAGGCATTT
ACTTAAATATGTTCTTGACTTGGTGAACGAAATATGAATCGTAAGACTCA
AACCACTAGACTCACCTATGGATCCAAGCACAAAATTATTGGTAGATCAA
AGAGCACTGCGTCAGCTTTTTGAAAGTACAAACAATTCGTTGGAAAGTTA
AAATACTTGACTGTTATTAGATTGCTGCAGTAAGTGATATCAACAAGTTT
CTAGACCATTTGAGAACTACTCACTGGGATGTTGTCATTCACATTTTCAT
ATATTTGAAAGGTGTACCTGCATGGGTCTCTTCACCAGAATCACAATGCA
CTCATCTGTGTAGAGGGATATACAGATGGGATGTTGATTGGGCTAGATCA
TCTTCCAATGGGAAATCAACTACACACCATTTTGTATTTGTTGGAGGCAA
TCTGGTATCTTGGAAAGGTTAGAAACAGGTTGTGGTGGCTTGATTAAGTG
GCGAATTTGGACATAGACTCATGACACATGCTACTTGTGAATTGATTTGG
CTAAAACATATATTGGAAGAACTCGATTTCTATCATTCGCCACCTGTGAA
CTTGGTGTGCCATAATCAAACTGCATTCTGCATTGCATCCAATTCAAATT
TTCGTGATAGAACAAACATATTGAAGTTGATTCTCTTCATTCAAGAGAAG
ATATTGCAGAAGTTGATTCTTTTTTTTCTAGAGAAGAAATTTCAGAAGTT
GATTGAGACTCAGCACGTGAATCAGTTTGCGCATTTGTTTACTAAACCAT
TAGGGGGTTAACGGATAAAGTGTATTCGTAACAAGCTATGAGTATACAAT
GTATATGTTCTACCTTGAGGGAGAGTGTTGAAGGGTTGGTTAGTCATTTC
CTATTTCCTTCTCATTATAAATAAAGGGAGAGACTAACAATAGGGAAAAT
TAGAAGTCATGCAGCGAGGTGATTTTCGGGAGGTGGGTTTAATCATATTG
CTTGGTTTCAATTGAAGCACAAAATCGAGGTTCGACTTCTTCTACGATGT
CTTCTAGGGAAGCACTTTGTTTCTACGCTGATTGGTTAGAATTAGTGTTG
GATAAGTTGAGGGCTATGGGGTTGTTACTGGTGGTGGTGAAGGTGGGTTG
ATGCTGGTCGTTACAGGTTTGCTGGAAGACATGGGGAGGTGCATGGAGGG
GTTTACTAATGGTATCTGTTTCATCTTTGCCCCCACTCCCAAACCCCAAA
AACACTCCCTGCCTTTCCCAAACTCAGCGATTTAGAGTCAGAAAGGAAAA
GAGCAAGACGAAGCTGCCTTGCTGCATAAGTTCGATCTCAAGATTTACAG
GAACAATAAACAAATCTTGACGAAAATCAGGTTGGTGCTTGAGAAGGACA
AACATTGCTTGAGGAGGCTGCACAAGTTGTTCTAATCTATATATGGGTCT
AACGGTCATGAAGCTCAATAATCACTTGCATAACAAACCTTTTTGTTCTA
TGTTTTTGTGTTGGTGGTGGCTGGTAGTTTTTTTTTCCATTTAGATTGAA
AGAACGACGTACTTGGGTTTTTCTAACTTTGTGTTCGAGTTTTGTTTTGG
TCAAAGAATGTTTGACCAAACATGTTTGTTGTACTTGAAAGAAAAAATCT
GTTTGTGAGTTGGTAACTAACAATGCATAAGAGTTTTAAAGTTTTTAGAG
CATGTAAGAAGCATCCAAACATGTTATAATAAATGTATTTGGTTGTTAAA
GAGGAATTTGGATTGGAATAGGATATTTATAATGTGGATTTAGCTCCTTA
AAGTCTTGCACTGTATTGTCCTGCTGGAAAGTACATTTTTGATAAAGATA
ACCTAAGTTTATTAGAGTTACTTAGGTTTGTTTGTGATAGGATCGTGGTT

AAAGGTTTGTTTGTGATAGGATCGTGGTTAAAGAAAAAGGGAAAAAAAAA
AAGGACGAGCAAGGTTCCCATACGCTTCTATGTAAGGCAGAATGATAAGT
CTTAATTGGAGCCTTATTTAGAAGCTTCCTACAATTACGCTAGAAATATG
TGAGCATTTCTGGCAAGGGCTTGCGCTAGCCACTTAAACCATATATTTAT
TTGGAAGAAGGCATGTTGACACCTTGAAAACCAGCTTAGATTAGAAGGGG
TAGGTTCCCAAGAATATCAACTTAGTCAGTGATATACATTAGGCATGAAA
ATCTTCCACGTTTTGCTACCAATGTCCATGGCGTATCAGGCAGGTGATTT
GGTGGATCTGCATCAGGTAGTACCCCTCCCCAAGTTGCCAGCTCATCTGC
TAGTAACCACATCTCATACTGCTTACATGGTGCCTGCTTTGATACTAAAT
GATAAAACTTGGATAGTTGGATAGAACTTACCGTTGAAAACCTGCTTGCA
AGGCGAGGGTGACATGAATCCGGAGTTAGTTGATGTGGGACATTAGGCAT
GTTACAACACATTGTTTCATTTCTTTCCTTTTTAATATGGAAGAAATCAC
TTTAAGGAGAACAAGCAACACAAAATTCTTATATACCCTTTTACCGTTTG
CATTAAATTGCCCAAACTAAGAAAAAAGCTAAAAACAGAGTAGCCAAATA
GGTGATTCCATTTGTGATCTTCTGCGAAGTGCATGGTGCTCGAAAAAACA
TGCGACTATAGTTGATGTGGTTTTCGATTTTAATTGTCTGAGACTTTATG
TTCAATTCTTAAGTTGTTCTTTGTTTGCCACTTGCAGTATACTCCTGCTA
TTGATATTTGGAGCATTGGGTGCATATTCGCAGAGATGCTTACAGGAAAA
CCACTGTTTCCTGGGAAGAATGTTGTTCACCAGTTGGATCTTATGACTGA
TTTGCTAGGCACTCCTTCCCCTGAATCGACTGCAAGGGTTAGTTATCTCA
TTCCATTTCCCCATATTTGAGGGTTCATAATTTGAGGCAGTTATTTTGCA
ACAGTTATTATTTTTTGCAATAGCTTTTTTAGTTGAATTTATTATAATT
AAAATTACTGCTGCGACAGTGTGACTGCACCAAACTTCACGATAGCTCTG
ACAGTTCTATGTTCCTTCGTTGTAATATTGTTGTTATGAGAAATGCAGAT
TCGAAATGAAAAGGCAAGAAGATATCTCAGCAGCATGCGTAAGAAACATC
CAGTTCCTTTTACTCAGAAATTCCCTTCTTCAGATCCTTTGGCTCTTCGC
TTACTTGAGCGCTTGCTTGCATTTGATCCTAAAGATCGACCCTCTGCTGA
AGAGGTAGTGTTTGGTGGCTGAATTGTAAAAGCCATTGGATGTTAACACA
CACACACACACACACACACACACAAAGCTGTATACAGACGTGGGTTTGTG
TATATTTTATGTTAATGCACATTATACTGCAATCTGATTGGCCAGAGCCA
CCTTGTTCCTGATATCTTTTTATGATGAAATATTTGTAGGCATTAGCTGA
TCCTTATTTTCATGGTTTGGCGAATGTAGACCGTGAGCCCTCCACTCAAC
CCATATCAAAACTTGAGTTTGAGTTTGAAAGGAGGAAGTTGACAAAAGAT
GATGTTCGAGAGTTGATATATAGAGAGGTATGGGGTGAAAAAATCACTAG
TGAGGATCTAATAATAAGTACACCTGGATCGATTAGCTAATTATCAATTT
GGTCTGAAGTAAACAGATTCTAGAGTATCATCCCCAGATGCTTCAGGAGT
ATCTTCGTGGAGGAGAACAGACTAGTTTCATGTACCCAAGGCAAGACACT
TGTTTATTTATTTTTTATTTTTTTACCAAATTTAATTATCTTTTCATTGG
GTAGGAGCTATTTTTGTCTTGTTAATTTTTAACTTGCTGTAGTTGTTTGC
TGGTGATCTTATCTCCTTCACGGCATGTGATAGGTCTTAAGTCTTAGTTA
TCTGATGTTTCGAAGTGATGGCGTTCAACGATATGGGCCAGGAGTGGAGA
AAACTTCTTCCTTTTAAGAATTAAGACACATGTGCATGTTATGCTTTATT
ATTTGTGATTTTGATTTGATTTGGTATCCCTTTATATTTTTGTTTGCCTG
GCTTCACTCCATAATTATTGAATTAAAAGCTTATTTTGCTTGTGTCCGAA

GTGATTGCATTCAAACAATAATTATTAGTTCGAGAAACAAAGTCTTCTCT
CTTTTCGAAGTTGAGACACGTGTAGATGTTATGCTTTACTATTTGTGTCT
TTGATTTGATTCGTTTCCCCTTTATACTTTTGTTGGCTTTCTCTCCAAAA
CTATCGAATTGAGGTTCATTGGAGTCGAGTTTTTCTTTTTCTTCTACAAT
CATCCCGTAATCCTAACATGTGCAAAGTCCCCGTATTTTGGCTCGACTAT
AGTGATATTTTCTTTCGAGAAATGGATGACCTACACGTCATCAATTGTAG
CCTTGATTTTTGTGCACCAATCTTGTAGTCAAAGTGAATTGATTTTTCTC
ACGTCACACTTTTTCCCTATTGATCCATCAGTGCCAAGCCGCCAATGACA
TGATGTGGGTAGCCTTCTCAATACACATGAAGTATTGATACACATGTACC
TTTGCAAACACTTTTATGAGTACCATCTAAGAAGCACTGACACGGTGTAT
GGGAAGTGGATAAGGTCTTGATTTTTGTGCACCTAATCTTGTAGTCAAAG
TGAATGATTTTTTTTCGTATCACACTTTTTCCCTAGTGATCCATCAACAC
GGATGACATGTTGTGGGGAAGCCTTCTGAATATACATAAACGACTGACAT
GTTGTCAGAAATGTCTCCGGCTGTGTCAATGGCATTTAAAATAACATGAC
ACAGATTTTGGCGTGTAATAAACATCTCCAGCTGTGTCAACGGCATATCT
GTGTCGGAAATGTGGCTGACACGGATTCTTTGCCTACCTAGGTGTTTTAG
TGCTTCCTAGTATAACACTTGAGCTAGATAGGTGGTGGTGAGCATTTGCT
TTCTATGTCAAATGGTATCTCTTGGTACATTGACAAGGAGGGTGCAGTTT
CCATGTTTTAGTTGGAACATGTATTTTTATGGAGAATGGAATTGTTGTTG
TCAAAGTTGAGCGTCAAGTTGCCTAGGTAATGAGGTGAAGCGAGAGAAGC
CCTTCTCTCCTCCACTTCTGAGGTGAAGCTGTTTTATTTAGGTGCCGACT
GTTGACTATTCTAGCATCTGGATTGAGGCGAGAGCCAAAAGGCAATTCCC
CCCCTCTTATATGTGATTTTACTCGTATGAACCCATGTAATGAAATTGTT
TAATAAACAATATTAAGTCATTTTACCAATTTTGAGAAGGAAAGAACAAT
GTTTATCATTGGAAATAAAGGATTTGAAAAACTATATACCTTTACTGACT
CCTTATCTTGGGTTTAATGCCCATGACAGTTGACATGTACACGTATTTGC
TTTTGTTACTTGGTTCACTAGTGGTGGAACAAGTCCTACAAGTTGGGAGG
GGCCAATTGCAATTTGAGGGGATCATTTTTATATTTTGAAGGTATTTTTG
TGTAATTTTTAAGGACTACATTACACATAAATCCTTGATGTTTCCAAAAT
TTTGTGGGGACTAGCACCCCCACCCCCTCCTCAGCACCCATCCGCCCCCG
CACGCTACTACATAACATATGCAATGGTTTGGAGAGTTAAGATGGAGGTC
TTGTGGTGATATTACACATTCTTCCATAGGAGACATCAGTGCTGGGGAGC
ACACATAATAATGACGCATAAATCCCTGATATTGGTTCTGTTAACTTCTC
CAATAATGAAATTAGGTGGGATCCATGACATTTGTCCATAACTTTGATTC
TTCTTTGCATGATACCTCTAGGAGGGATGCGCTCTCGCTTTTTCACACTG
TGGATGCATGGATGGACACGCGATTCTAACTTCAAGGAGGGTGTCTTTTA
AATATTTTAAGTAATACTAAATTGGAATTAACTAATCAATTGTGTATATT
TCTAATTGTACAATTAGGCTACATATAAGAGTAATTAAGAATAAATAAGG
AAACTAACTATGACAAATAATAACTTTCCTAATCTACATATTTGACTTTC
CCTATATACAAATAATAATAATATCGAATATTTCAACACTCCCTCAAGCT
GGTATGAAGATATCTTCCATTGTCAGCTTGCATACTAGATAGTCGAATCT
CTTATTTGGTAATCTTTTTGTTAGAATGTCTGCGATCTGTTCAGTAGTCG
GTACATATGGCATACATATTTGTCCACTCTCGATCTTTTCCTTGATGAAA
TACTTGTCCACTTCCATATGTTTTGTCCGATTGTGCAACACTGGATTGTG

AGCAATCGCAATGGCTGATTTGTTATCACAATAGACCTTCATAGGTGCTG
AATTAGATATCTTCAACTCTTTGAGTAATCTGTTAACCCATAACACTTCA
CAGATGCCGTGAGCCACTGATATGTACTCTGCTTTTGCGCTACTTCTAGC
TACGACATTTTGTTTCTTACTTCGCCATGTGACGAGGTTCCCACCAACAA
AGGTACAGTAATCAGAGGTCGATCTCCTGTCTGTGATGTTCCCAGCCCAG
TCTGCATCGGTGTAAACCTCTACAAGTCGGTGGCCACGTTTCTTATACAA
AATTTCCTTTCCAGGAGTACCCTTTAAGTACCTTAGGATTCTATAGGCAA
CCTCGAAGTGTTCTTGTCCTGGTGAGTGCATAAATTGGCTGACCATGCTT
ACGACAAAGGCTATATCCGGACGGGTATGTGATAAGTAGATTAAGCGCCT
CACTAGTCTTTGATATTATTCTCGGTTTATCACAGCTTCAGGTTTGGCTG
GTTGTAATTTTTCATTCGGTTCACTTGGAGTTTCTACAGCTTTGCACCCG
AGCAACCCTGTTTCATTCAGTAGGTCAAGTACATACTTTCTTTGACTCAC
AAATATGCCTCCCTTTGATCTAGCAAATTCCATTCCAAGAAAATACTTCA
ACGTACCTAAGGCTTTGATTTCAAAATTATCAGCAAATTTTTCTTTAAGT
CTTCTCAACTCGTCATTATCACTTCTTGTTAAGATGATATCATCTACAAA
GACAATTAAAATGACAAACTTACCATCAATTGAGTGTCTATTGAGTATAG
TATGATCGGCTTGGCTTTGTTGATAGCCCTGACCTTTTACAGTGGTTCCA
AAGCGTTCAAATCACGCTCTTGGAGACTGTTTTAGGCCGTATAGTGACTT
CTTTAACTTGCACATCTTTCCTTGTCCTATCTTTCTTTCAAAACCTGGTG
GTAAACTTATGAACACTTCCTCCTCCAAGTCTCCATTTAGAAAGGGATTT
TTCACGTCTAATTGGTGTAGTGGCCAATCAAGATTCACAGCTAGGGACAA
TAGAACCTGAATCGAGTTTATCTTAGCAACTGGAGCAAATGTTTCTTGGT
AGTCTATCCCATATGTCTGTGTGAACCCTTTAGCCACGAGCCTTGCTTTA
TACCGTTCTATGCTTCCATCTGCCTTACACTTAACTGTGAATACCCACTT
GCACCCGACTATCTTTGTATCCTTTGGCGAATCAACTATTTCCCACGTAC
CACTCCTTTTAAGAGCATTCATCTCTTCCAGAACTGCTAGCTTCCAACCT
GTATCACCTAGTGCTTCCTCAATGTTCCTTGGAATAAACAGATTAGTAAC
TTTAGAAGTAAAGACTCTATAAGGTTCGGAAAGTTTTGTGTAGGATACAT
ATCTAGATATGGGGTGATTGGTGCAGGTTCTAACTCCTTTCCTGATGGCT
ATAGGGATATTAAGATCTGGAGGAATACTGGAATGAGAAGATTCATTAGG
AGGTGGTAAAGATATAGGATTACCTGGATCATTGCTTGGGGAGTCGGATT
GACCTTGTGCTAGAACAGTAGGCTGATCTGTATTCTTTTTTGTACCTCTC
CGAGTATAAACCCGCAACTCAGTACATGATTGTGGTATTTCTTCATCTGT
TTGTGAAGGAATTATGCTCGGCACTTGTGTACTAGTAGGGATCCCTTTTT
GTTTTTCAATATTGTCATCACTTGTTTTTGACTGATTTGGAGGAAAAATT
TCTAAGACTGTGGTAGGGAGAGGATCATCAGTGACCCAAAAATTCCCTTC
CATTACATTCTCCCCCTGAAAGGGATTTTTGTCAAAGAAAGAAACATTTT
CCAAAAATGTGACGTCCATACTAACATGATTTTTTTGGTTTGTGGATTGT
AACACTTATACCCTTTTTGATTTGGTGCATAACCGATGAAAACACATTTT
TCTGCTCTAGGATCAAGTTTAGACCGAAATTGGCTAGGAATATGAATATA
GGATGTGCAGCTAAATACTTTAAGAGGCAGATCAGCTGATATCCTAGAAG
CTGAAAAATGTGTTTTAAAATTATCAAATGGTGTGGTGTAATTTAGAATA
CGTGTAGGCGTGTGATTTATTAAATATGTGGCTATTAAAAGAGTATCACC
CCACAAATATTTTGGAATATGCATAGAAAACATGAGTGTACGTGCAATTT

CGAGTAAGTGTTTAGTTTTTCGTTCAGCGATGCTATTTTGTTGGGGCGTG
TCACGACATATGGATTGATGTAAAATACCATTTGTTTTCAAAAAACTGCC
AAAATATTCGTTGAAATATTCTGTTCCATTATTTGTGCGAATAATGCTAG
ATTTCATTTGAAATTGGTTTTCTAGCATAATGCAAAAATCTTTAAATAAA
GTTTCTACATAAGATTTTGCACGCATCAAATATGTTCAGCATAAACGTGT
GTGATCATCAATAAATATCACAAACCATCTTTTTCCGGATAGTATTGTGA
TCTTTGAGGGACCCCATACATCACTATGAATTACATAGAATGGTTTGGAT
GCACGATATGGTTTTGAAATATAAGAAGTACGCTGACTTTTTGCAAACAA
ATAACTTTCACAATGAAAAGAAGAATAATCCACATTTTTAAATAAAGTCG
GAAACAAATGTTTAAGATATTGAAAATTTGGATGTCCTAGTCTAAGGTAC
CAAAGCATGATTTTATCACGAATAGACGTTGAACTCATACCACTGGGGCT
GTGAGTTGTTTTATTCCTCAAGGAGTTATCATCAAAGTAATAAAGGGCAT
CCACCAATCTAGCACTGTCAATCATCTTCCCCGAGCTCTGATCCTGAAAG
AAACATCGAGATTCAGAGAAAATGGCATGACAGTTCGAATCTCGTAATAG
TTTACTTACAGATAAGAGGTTACAAGTAAGTTTAGGAACATGAAGGACCG
TTTTTAGATTGATACTATCTGAGATTGTTATTTGGCCTTTTCCTGCAATG
GGTGAGAAACTACCGTTTGCAATTCTAATTCTTTCGTTACCAGAACACGG
TGAATAGGTATTGAAAAGGTGATAAAAACTTGTCATATGGTCAGAAGCAC
CTGAATCGATAATCCAAGGTGTTGAGTTTAAGGAACAAGAAAAGACTCTA
GAGTTACTACCTGTTTGAGCTAAGGAAGCATTAGGAGTACCGATGATGGA
TTGACAGTTAGCAGTTTCAGAAGTTGATCAATTTGCTCTTTGTTGAATGG
AGTGGTGGCAGTTTCATGTGCGGAAGGATAGGATCGGTTAGATTTTTCTC
CTGACTTGTTGCTCTTCAAATTTGCCGGTTGCCCATGGATTTTCCAGCAT
GTTTCCCGTGTATGACGGGGCTTGTTGCAGTAATCGCACCATATACGAGG
GCGTTCCTCTGCTCGGCGTGGAAACCTGCTGGAGTTAGCATTAGCAGTAG
CCAAAGCAGACTGGTCACTTGGAGCAACTGTAGGACCGATAATTTGTGGC
TTGTTGAGCATAACGTTCCGACGGGTCTCCTCCCTTTGGACCTCCGAGAA
AACCTCATTAATTGAGGGAAGAGGCTGACGACCAATGATTCGCCCCCTAA
CTTCGTCAAGTTCTGGTTTCAGACCTGCCAGGAACTTAAAAACACGATCA
TCATCCACCAACTTCATATAGTGGCTATAATCTTCGGTCGAATGCCATTC
ATGATCGTCGAACATATCCTAATCCTACCATATTCTCTTCAATGAATTGA
AATACTTGGTGATACTGTATTCGCCTTGACGGATATCACAAAGTTTTAGA
GTCAGCTCATACTTTTGAGATTGGTTTCCCATATCAGAATACATTTGATT
TACATTGTCCCAAAGTTCTTTCGCTGTAGGGTAGCACATGTAGTTGGAGC
TGATTTCCTCATCCATAGAGTTTACGAGCCACGTCATAACCATGTAGTTT
TCAGCATCCCAAGTCGTATGCAACGGATCTATGATGGCGGGTTCTTTTGC
ATCACTGGTTAGATATCCCATCCGTCCTCGTCTACGGATATACATTCGCA
CCGATTGAGACCAACGCAAGAAATTGGTTCTATTCAGACGAACAGAGGTG
ATTTGGATGGAGTGGGATTCGGAAGGGAGATACTTTGGTTCGGTAAGTTG
GTTGAACTTTGGGATGGATTCCGAGGTAACGTCGGACATGGTGAAAGCAA
GGGGAAATATTGTCAGAGGTTGAGAATCGTCGGATTAACCTTCAATGAGC
AAATCTCGCGAAACTAGGCTGCTTGATGCTGAGTTGAGTCGGCTCACTGA
GTTGACTCGGTTCGTCGGCTTCAAGCAAATCTGGCAGCGGCAGCGGTGAC
TAGGGTCGCGAGTGTTCATCGATGGTTGCAATTGAACCTCACGAACTGCA

ATCGAGTTGTAATTACGAGATCTATTTCGTACAAATCTCGCGAGGGCAAG
TTCGTCGGTTGTTAGGGCTCTGGCGAGGCTGTCGGCGGCGAATAGAGGAT
CGACTAGGCTTCGTCGTCGGTTGTAGAGGGCTGGCGAGTGTTTGTCGGCA
GCTGTAGGGAACCAGCGAGGCTTCGTCAGTTGTAAAGAGAGCTTGTCATC
AACTGTAGGGGAGCTTTCGTTTGTCGCCTGGCTGTAAGGAACGCCGACGA
ACCTTCATCGGCTATTGTTGGATGCCGGCGAAAGTCGTCGACGACTATAA
TGGCCGGCGGCGGACGTCGGAAGCTTGTAGAGGGCCAGCGGTGGCTGCAA
AGGCTAGCTAGGGTTTTTGTTTTAGCTCTGATGCCAAATTGGAATTAAAT
AATCAATTGTATATATTTCTAATTGTACAATTAGGCTACATATAGGAGTA
ATTAAGAATAAATAAGGAAACAAACTACGAGAAGTAATAACTTTCCTAAT
CTACATATTTGATTTTCCCTATATACAAATAATAATAATATCGAATATTC
CAACATTCTAGTTATGGCGGACAGACCTAACCCAAATACAAATATAGGCC
TGCCCAGCCTCGACCCCATGGTAGCCTCTCCCTCGACCATGAAACCCCAA
ATCTACCAACTCCACCACCCAATTTTAATTCCGCCTCACACGCTCACATC
TTTACCTATGGTAAAAAGCCTTTACAACACCTGAGCACAGACCGAGAACA
AGAAACCACACGTGATCACAGAATCAGAGCCTATCTGCTGCACTTGTCAA
GTTGTATCAGCTCTACCATCACCGTATGCGCCTAATATATTGTCACAAAT
CAAATCACAAACAGGACTTGGGTTCCCAAAAGCCAACCCAAACTAAAGCT
ACTTATCAGCAAGATGAGCAAAAAAATTGAGTAAATCCCAAGAAATATGA
ATAGAAAAAATTAATTAATTGAAAATTCAATGATTCCGAGATTGAGTAAT
AAATAACAAATTTCAGTTCTTATGTCCTTTCGATATCTTCTGTCCTGGCT
TCTCACGAACACCTCTTGGGTACTCTATATTTACATATGTATCTATCATA
ATTATATATACTAGTAAGTGCCTAACTAAGTACTACCGGTGAATGGAATC
CAGTGGCCGGCAATGGAGGTCTGCTTGTCTGGCAACCGGCAGCCGGAGTG
CGGTAACAAGGGGTGGTGGTGGGTGTCATAGTTAATGTTAAGTGGGAGTT
ACAGTTAATGTTAATAATTGGGTGTTATAATTAATGTTAACTGTGAGTTA
AAAAAATAAATTGCTTTATAAATAAGATAAAAAAATTACAAACCTAAAAT
TTTGAGGACTACATATTAAGGACTACATATATATATATATATATTTTTTT
TTATTAAAGCACTATTAGAACATAACTTTAAGCATGGGGACTTAGTTAGG
CACTTACTAGTATATATAATTATGATAGATACAAATAGATTCCTCCTAAA
TACTAGATGCCCTCGTGTTTCAACACAAGGAGACCACAAAAGTTGCTATA
TCATATGCTGGTTGTTTATATCCCTGGCCTTTCCATCTGATTGCCAAAAC
TTTCAAATTAGTCTTCGAGCCAATGCTGACTCTATTGTGAAGGATTGATG
GCAGTGACATGTGTAATAGAGGACGTGGGTCTTGCTGTGGCGGGCCAATG
AGTGACAGCTTATGGCTGTTGTGTGTGCAGTGGGCCGACGTGGATGGAGC
TGGTGGTGGCTGATCTTAGATCTTTTCCTATGAGCACAAACAGATATCGG
TGGTGTGGTGTTGGGGTGGTGGTGTCACAAAAATCGATTGTGTGGTGAAG
GTAAACGAAGTGAGAGAGGGAAGGAGGGATTTGCTTCAATTTTCATTTAA
TATGTAATGGTTTTGCATGTTTTTTTATTGGTTGAAGTTTACCTGATTTG
AATAATATTTGTGCAAATTGTAGGCATTTTATCAAAGAGTTATCTTGTTC
ATTTGCTCCCTTGTGTTTTATTTGGGCAAATCTTGGTGCTTCTTATGGAG
ACTTTCTGGAGGGAAAGACAATAATTTTTCATATTGATAATTTTAAAAGA
ATACGCGTGAACTTAGCCAAAAAGAGAAGAATATTATGTTCTAGCAAATA
TTCCGGTGCCTAACATTTCTAATGTTTAGATATTAATAAATTTATTAAAA

AGTAAAGAGATTAGAGGGGCATGAGCAATAAATAAGGGCCTTTGGTGGTA
TTTGGTGGTTGGGGTGGGCAATAAATTTCGAGATGGTGGTGGGGTCTGTG
TGTGTGGATGGTGGAGGGGAAGGGGGTGAGGAGGTTTAAGGGGGAGAGAG
AGAGAGAGAGAGCTAGAAAAGAGAAGTGCAATAATTTCTTCTTCTAACCT
TTCTGTTTGTTGTTTTCTTTTGTTCTTCCCATTTTCCGAGTGGTTTGTGT
GCCCCTTTTCTTAACTTGGAGATAGTTAGTGGGTGAGTTTCATCTTCCCA
GTAAAAGGAACATATTTTATTGCTCCACATTTTATTAACATGAAATAGCA
CCCTTAATTATTAAAGAATAGCATCTTGTTCATTTTCCTGATGTTAAAAG
AAATTTCTAGAATTTGAGTTTGTCTTTTATTTATCACTTTTGCTTCTTTT
CGAGCTGACTTTATAAATTCTGAAAATCATTGGGTACTAATACTATTGAA
GCCCTACCAAGTCAACCATTATTTGTCTTATGATGTCATTGATTCATGTC
ATTGTATAAGTTATTTTCTTTCTTTCAAAATACTTTAGTTTAAAATTTGC
TAAAGTTTGTTCATTATTATTGTTCATATCAATTGTAGTGGTGTGGATCG
GTTTAAGCGGCAGTTTGCCCACCTGGAGGAGCATTTTGGTAAAGGTGAAA
GAAGTACTCCACTGCAAAGGCAGCATGCTTCCTTGCCTAGGTATGCTGGT
TGATAAAAACATATATGCTTTTTACTGTTGATATTTAATGGGTCCTTGGG
AATCTGTACACAGCAAGACTGACTTCTAGCAACCCAGATTCCAGATTGCA
TAAACCAGTTTAAGCATGTGCTGACTTGCCCTTATTATCCTCATGGACCA
GCCTTGTTTGCTGAACAGGTTATTTGATACTACGAAGGATATAAAAATAA
ACTATTACATGTAAATAATTATACAAATAAATTTGACTTGAGTCATAATT
ATATTATTTAATACCAATCATATCTAATATTAGACCAAAAGGAAATCTTT
TGACAGCACTATAGCTCCTGCATTATACGTTATTGAATTCCCTAATCTTT
CCCTCAGAGAGCGGGTTCCTGCACTCAAGGATGAAAAAACTACCCAAAAT
GATGATTTAGAAAAGCGTTCTGCGGCTTCTGTTGCTACAACTCTTCAGAG
CCCTCCAAGGCAGCCTGCGAGATTGGAAAATGCAGATATCAATGCACAAA
AGGCGAACAACAGTGCTCGTAGCCTGCTAAAGAGTGCTAGTATTAGTGCT
TCAAAATGTATAGGCGTGAAAGCAAGAAAAGATCAAGAGGTAACTAATAC
TTCTACTTTTCTGTTAGTTCTTTGTTCAGTCTTCAATATTTTATTAAAAT
CAATGCTGTAGTCAACTCGCATGGTACTACCTTTTTTTTATTTGAACAAC
ACAGTACATAAAGTCACCTCATTTTCACAAAGTTGATGTCATAAAAAATTG
AGAAAAAGGATATCTGAAGTTGTCTTTTCTAAAATAGTTAACCTATCATA
GCTTAGTTAGCTTTTGAAACATTTAGAGTGCATTTAGGTGAGGTATTTGA
AGATGTAATTTGATTCAGATAAATGTTCATAAATTGAAGCCAACGATTCA
TGAATCATGCTATTGGTGGCAAAAACAGGAAATAATTAAGAAAAAAGGGG
GACTCCTACAAAGATTGTAACAATTAATATGTACCAAGAACCAAAAAATG
AAAAAAAAATTTAAGGTGAGATTCATATGTGAACAAACGACTTTTGAAATG
CATGGGTCTCTTTTACTGGGTTTTTGTAAAATCTTGTGTTTATAATATAG
GATGAAGCAATTCCAGAGCAACACGACGAGGTCGATGGCTTGTCTCAAAA
AGTTGCAGCGCTCAAAGCTTGA
>EUC16639-RA [gene]
ATGGATTTGGGAACAAACGAAGGGATTGAATTTGTTCAAAAATCTGGCGA
TCAAGAGGTGGGATTGGGCTCAAAATTGAAGGGCAGAGGATCTTTGAGTG
AGAAGAACATGTCTGGGAGGAGTTCCACAAGCCTAGGTAGCAAAGACATG
ATTTTTCGGGCGGACAAAATCGATCTGAAAAACTTGGATGTTCAGCTGGA

GAAGCACTTAAGTCGAGTTTGGTCGAAAAATATTGAGACCCAAAGGCCTA
AAGAAGTTTGGGAAATCAACTCTTCTAAATTGGATATTAGGTATTTCATT
GCTCAGGGGACCTATGGAACCTTGTACCGGGGAACCTACGATAATCAAGA
TGTCGCTGGTATGAATATGCATATCGTTATTATATCTGTTTTAGCGTAAT
TATTTGGTTTAATTTTGTGATTATTTGCTTGAATTTAGGACTTTACGTTG
TCATCCTCTGTTTTTTCAGAGCAAAGATTTGATTCACCTTTTAGGTAGTG
TTTGGGTGAATGAAATTGAAATTACAACCCCATGTTCTGGTGAAATATAT
TGTTTTGATCTGTTTTAGGATGATTTTTTGGCTTAATTTTATGATTATTT
GCTTAAACCTGGGATTTTTATGTGGTTTGTGGAGTGATTAGGTCATCCTC
TGTTTTCGTTAAAAGGCCTTAATTTTGATATGTTTTAGCATGATTGTTTG
GTTTAATTTTATGATTAATTGCTTAAATTTAGTTGTTTGATTCTCTGGGT
TTATCTTGTCATTCTCTGTTTGTTCTCCTCTAGGTAGTGTTTGGTGGTAT
CTAATTAAAGCTTTTAAATTGCAATTACAATCCAGGATTGAAGCTACATG
GGTTCATTTCCAATTCCAGATTCAAGACAACCAAACACTGCCTTAGCATT
ATTTATTTATATTAACTATGCCAAACAACCTATAGTTTTCGACCGCATTG
TTCTTCTTGTTGTTTAACCTTCATTTTGACCTGGAGGTTCATTGTTCTTG
GAAATAGCCTCTCCATATATAGGGGTAAGGCTGGAACCTTCGTGTACTGG
GCTGCCCTCGTAANGGGCTGCCCTTTTTTTTTTTTTTTTGCCTTACATAG
TCAAATTCCTTTATCCTATTTGGTTCTGGAATTGTGAATCAGAAATGAAT
GCATGTAAAATTATAAAAAAAAAAATCCTTTAATTTTTTGTTTTAAGGTTT
CAATTCCATGCTGTCAAACAACACCTTATTTCCACTCTTTTTTTTTTTTT
TCTTTCAGAAAAACGTCGTGAAATTTGCCTATTCCTATTTTTTTTTTCTT
TGTTTCTTTTATGATTCAGTGAAGCTTTTGGATTGGGGAGATGATGGCGT
AGCTACAACTGCTGAAACTGCCGCTCTACGAGCATCATTTCAGCAAGAAG
TCGCCGTTTGGCACAAGCTTGACCATCCAAATGTTACTAAAGTATAAATT
CTATTCCATAGGCACCATTTGGTAACTTTTTTTTAAGTATATTTTTTTTG
TTTGTAATCACAAAGAGCAAAAAAATAAAAATAAAAATAGAGTAGCGTTT
GTTTTGCCCTGAAAAAAAGAAAAAAAAAGTAAAAATATGATTTTATTACT
CTTTACTTTTAAGTAATAGTACATTTTTGACATATTAAATTTTATGATTA
TAAGAGCACGTGAAAAAAAGTTTGACCAAATAGTGCCTTAGTATAAATTT
AAATTGATTTTATCAATATATCGAAAAAAGGAAAACGACATGATTTGGAT
ATGTTTTGTTTGTTTCAGTTCATTGGCGCTTCAATGGGGACTTCTCATCT
TAAGATTCCTCCGAAAAACCCATCTTCGGGTGGTGGGTTTATCGATCTTC
CTTCTCGAGCTTGTTGTGTGGTGGTTGAATATATTCCCGGTGGAACTTTG
AAGGGATTTTTGTACAAAAACCGGAAAAAGAAATTGGCTTTTAAAATTGT
GGTTAAACTCGCTTTGGATCTTGCTAGAGGGTGAGCTTTACTTTCTACTT
CATGTGTTGATAAATGGAGGAGGTTGTGTATGTTTTAGTGTTTCAGTTCC
CGGATTTTTGTTTTATATGGGGAACAAAATACTGAAACAAAATTGTGGGA
ACAAATTTTGATTAAAATTTTGTTCCTAGGAGTTCTTTAGGAATACAAAT
TAAAAAATGTGGAAATATTTATGCTAAATAAAAGGCACCTTAGTGTTTTT
TATTCCAAAAAACTGGGAACAAAAACAGATATATGGGAACAAAAAAATTC
CATTTCCTATTTGCTCTCAGATTTTAAGAATCAAAATAGAATTTTCCCAA
TTTTTTGTATTTGTCGCATGTTCTTTAGGAACAGAAACAGAAACACTAAA
CAAACGCCACCTTATTTGTTTTGTCCCCAAAAAGCTGGAAACAGGAACAT

AAAAATTAAGCGAAAAAATGTTTCTGTTCTGTTCTCAGATTTTCGTTACA
AAATTCTTAGAATTGTTCCCTAATTTCTGTTTGCTGTGAACAGAAACAGA
AAATTGGGAAAAGTTCGGTTCTAATCTTAGGAATGTGTGAGAAATTGAAA
ATTTGGGAGCAGAAACACTACTATAACTTGAGTAAAAAAAATGGTAAAAC
AATTTTTTTTTTTTTTTTAAATTTTGCAGATTGAGTTATTTGCATTCGA
AGAAAATCGTACATCGTGATGTGAAAGCGGAGAATATGTTGCTGGATTCA
AATATAACTCTGAAAATTGCTGATTTCGGGGTAGCTCGTGTTGAAGCTCA
GAATCCAAAGGACATGACAGGTGAAACCGGAACACTTGGATATATGGCAC
CTGAGGTATTTCTCTTTCCGTTCCCAAATTCTCTCTCTCTCTCTCTCTCT
CTCAATATGGTGCTACTTTGTGTTGTGTGTGTAGGTTCTTGATGGGAAGC
CTTATAACAGGAAATGCGATGTCTACAGCTTCGGGATATGTCTATGGGAG
ATCTATTGCTGTGATCTTCCCTACCTTAACCTCAGTTTCGCCGAAGTTTC
TTCTGCAGTCGTTCGACAGGTCAGCATTTATACTGATTCAACAACTTTTC
TTGAAATCTAGGTTTCATTTGGATAATATTTTTTAAAATTATTTACACTC
TCCTAAGTGCACTCCAGAAATTAAATTTTGAATTTAATTCTGAATGCACC
AGAAATTAAATTAAATTTGGGTTTTTTGAGAACAAAAATAGAAATACTGA
CCCAAACGAAACCTTAGTCCTTGATCTTATATATTCAACAATGATTTATT
TTATTTTTTAGTTGAATTTAAGGCATAAAAATTTATTTGAAAGACTAAAA
CATAAAGATCTGAGAATAAAAAAAAATTATAAGTGATTTATATAGCGGAA
AAACTAATAACTTTTTCTTTTTGTTTCGAGTTATTGATAACAGAAACATA
AACATAAATACTAACCCAAACGCGACCTTACTCCTTGTTCTTATGTTTAA
TAATAATTTTTTATTATAGAACTTGAGGCCTGAAATACCGAGATGTTGTC
CGAATTCTTTTGCGAATATAATGAAGAAATGTTGGGATTCAAACCCCCAA
AAACGACCAGAGATGGAAGAAGTGGTGAAACTTTTGGAAGTGATCGATAC
GAGCAAGGGAGGAGGGATGCTACCGGAAGATCAAGCTCGGGGTTGCTTCT
GTTTTCATCCGTCTCGGGGTCCTTAA
>EUC16268-RA [gene]
ATGGCTTTTGATCAGAACTCAATCCCCAAAGATCTACGCCCGTTGAATAT
TGTTCGATCAACGCCTGAAGAATCGCGCATTGCACCAGTTGCGACGTCAG
GGAGGGCAGTTGAGGGGTTTTATGCAAACCCACCCCGCGATGTCACGAGT
CCCCGGTCGGCACCTCTCTATTACAACCCTGCAATGGTGACTGATGCTGG
ACTTGCAGGTCTAGGTTATAACAATGCCCCTTCGAGTGTAGCTGGTTGGG
TGCCGCATGTGCCACCAGTTATTGTGGGCACTAGTGGTGTAAATCCAGCA
ATTGGGCACTGTTACAATCCAAATGTTGGGACTCGAGTTGGTGTTATTTC
TTCGGATCAGGGCAGTGAGGAGGGCGGTGAAGATTCAGTGTCTGGGAAGA
AGGTTAAGTTCTTGTGTAGTTATGGGGGGAAGATTTTACCTAGGCCCAGT
GATGGGGTTTTGAGATATGTTGGTGGGCAGACTAGGATTATTACCGTTAG
GAGAAATGTTAGCTTTAATGAATTAGTTCAGAAGATGACTGATACTTATG
GGCAAAATGTCATTATCAAGTACCAGTTACCAGAGGAGGAGCTTGATGCC
CTTGTGTCAGTTTCATGCCCAGATGATCTGGATAATATGATGGACGAATA
CGATAAGTTGGTCGAGAGGTCTACGGATGGGTCAGTTAAGTTAAGGGTGT
TTTTATTTTCGGCTTCGGACATTGATTCTTCTTTCGGAGATTTGCAAGCA
AGTGGCAAGAGATATTTCGAAGCAGTTAATGGAATTACGGAGGGTGGTGG
TGGTATTACAAGGAGGGATAGCATAACAAGTGCCGCTTCAACACAGAATT

CGGATTTGAGTGGGATTGAGCCTGTTGATAATGCAGGCTACAGTCATGAT
GATGCTACTGGGTTGCCATCAACTGTCTTGTTGTCACCTAGACGAAATTC
TACTACTTCTCAGGAAAGTGGTCAAAGGTTGGTGGTGGATCCTAGTCAGG
CAAATTATGCTGATGCTTCTGCTCTATTAGCTAATTCGGCGGTTAAGACT
GGTTATCCTCTATCATCACAGCCTGAGCCTGCTCATGTCATACAGCAGCA
GCTGGCCTTCGATTTGCAGCAGCCTGGAGTAAATTTTCCGGCACCTGCAT
CTTACTTGCAGGCTTATGTGGATCCTCGCCAACGCCAAGAAACAGTGAAT
CATTCAGATTATGCACAGCAGCCTCCTGGAGTTGGGTTCCCACCTCACCT
GTTGGGAACTGTTGGGCCTGTATTCACCCATCAACAGTTCTCTCCTGGCA
TGTCCCCTCAGCAGTTTATTCCTGCAGTGCACATGACAATGACTCCTTCT
ACTCATGTTATGATGAGACCGAATATGGTTCAACCATCGGTTCATCCCAA
CCAAGTCCAATTAGAGCGTTATCCTGAAGAAAACACGTTCAGCCAGAGGG
GTGTCCAGGTTCCTGGTAGCCAAAGTTACAGCACATATCAAGCTCAGGTA
CCGGCTCCGGTTATGGGGGGAGGTTATGGATGGAATCAGGTTCAGGTCAT
ACACCCAGAGCAAGTGACCTTCTCAGAGGGTTGGGTACCTCAGCCTCAGC
AACAGGTAATGGCTCCTGAAAAAATTGCTAGACTGGAGGACTGTCATATG
TGTCAGAAAGCATTGCCTCATGCACATTCTGATACATTGGTGCAGGGCCA
AAGAGAAAGCCCTGTCAGCACAATATCTGATTCAAACATTGTTTATCATA
GTCTCCGTTTGGATGACAAAGGGCGGCCCAATAAATGGGATAATACCGAT
CATCCTAATGTGACAGCTGCTCAGGGTGTGGTGGGGTTATCAAGTGGTTT
GCAATCTCCTTATGGTGCGTTTGTAGGTAATAGTCCTCAGGTTGCTAGTG
ATATCCCTTCCGTTGGCGGCATGCCTTTCCAAGCTTCAGACCACCTTGTT
TTTGAATCTCCAAAAGACTTTTCTGGTAAATTTCCCAAAGAAGATACTGT
GCAGTCTGCCGTTACATATGACCATCTGAGACAAATTGATGGGCGAATGG
GAAATCTCGGGATACACTCCCCTGAAGCTTTAGTGAATAACGAGCTGAGC
AAATCACAGGTTGATAATCCAAAATGGGAAAATGTAAACCATGCTGAGCC
AAATGAAATGCTTTATCAAGCAGTGGATTCTAATGAAGTGCCACAACCAC
CTCGCTTAGGTACTCCTGGTTTATATCCTCAGCCAAATCTTAGTGCCAAC
TATTTAGTCCCTGATGAGATTGCCTCCACTGGTCCTGCATTTGTTGCAGC
TGATCCAGCTCATACAGTCGAAAGAATTACACCTATTGGCCAGTGGAAGG
ATAACTGTTCCCGAAATCAGACAAATATCGCCGCCACTAATGTTGAAGTT
GTTTCATCAGATGGGAACACACCAACTTCTCTATCTTCATCTGATAGGGT
TGCAAACGTACAGGATAGCTCAAACTCACTTTTCAGCAACCAGGATCCAT
GGAGTTTGCATCATGATACACATTTCCCTCCCCCTAGACCAAACAAAATT
CTGACAAAGAAGGAAGACACTGTCAGTAGAAATCCTTTTGGTGACAGCCG
ATTCGGTGACAGTGCTGAATCGCTTGGCAACGTTGGGGGGTTGAGAACAG
ATTTGCAGCTTGATGATGGAGCTTATGAGCCATCTAGCTATTTGCACATG
GATATTCCTCTCGAGCATGGACGGTCCAACAAATGTGAGTACTTCTTTTG
TTGCTTTTGATTATTGCTGATGTTTACTTAATGTTTTGGAGTCTCATTCG
TTGTGTTCTATGCAGCTGAAGGATCAGCGGAGGAACTAATCAAGCAAGAG
CTTCAGGCTGTTGCTGAAGGTGTTGCTGCTTCTGTACTTCGGACTTCAGC
ACCTTCTAATCCTAACTCAGCTGAACATGGGAGGAGTGAGTCTCATTTTA
AAAGCAATGAATACAGTGAAGTTAAGGCCAGTGATGGAGAATTGCCAGAC
AAAGAGAAATCTGAGGTCCTCAATTTCTTCTTTTGCCCTGCACTGTGTTT

ACGCTATTTATTACCATTAATTTGCATCTTATGGTCTTTTTATACAATTA
CTTTTGCATCATGTGGTCTTTTTAATTTAAGCAATATCATTGCTATTCAG
GATATCAGTATCAAAACGCCACAAAAGTCAAATTTTGGGTTCCCCTTGTC
AGATGGGATTGGTCGCTTACAGGTTGAAAATTGGAGTTTTAACATTTTTG
TTTATCTCTTACCTTCCCTGATTGCCTACCAATATACTCTTATTCTCTTC
CTACTCTTTTCTACCTTTCCTAAATCTCTCCCTCGGTGGCCCATTTTCCA
ATTTATGTTTGCACAGATTATAAAGAACAGTGACCTTGAAGAGCTTCGAG
AGTTAGGTTCTGGTACCTTTGGTACAGTTTACCATGGGAAGTGGAGGGGT
TCTGATGTTGCAATCAAGCGAATCAACGACAGGTGTTTTGCTGGAAAACC
TTCAGAACAGGAACGCATGGTCTGTCTCCTTCGATTGATGTGCTCCAATT
GCTTAGTTTTGTGTACACTGTTTTGCTGATTTTCTCCTCTTACCAATGGT
GTGATATGTTATCTAGAGAGATGACTTCTGGAATGAGGCAATCAAGCTTG
CTGATTTGCATCATCCAAACGTGGTGGCTTTCTATGGCGTTGTACTTGAT
GGTCCTGGTGGTTCAGTGGCGACAGTAACTGAATTCATGGTTAATGGTTC
TCTGAGAACTGCTTTGCAGAAGAATGAGAGGTGATGTTTCAATACTTGGC
ATTATTTTCTTTATGCACTCATGTATATATGCATATAAACTCTATTTTTT
ATGCAATTAATAATTACATTTGGAATAATCATGGGTGGGGGCATTAAGAA
GAGAAGATATTGACTGACAAGCGCATCATGATCAGGCAACTTGATAAACA
GAAGCGTATCTTGATTGCCATGGATGTGGCATTTGGCATGGAGTACTTGC
ATGGAAAGAATATCGTTCACTTTGACCTGAAAAGTGACAACTTGCTTGTC
AACCTTCGTGATCCACACCGCCCGATATGCAAGGTTGGTAACTGCATCCT
TAGATTTGTGCATGGAATTTATACATGTGCATTGTTTATGACGAGAATGC
TTCCTAAGCCTACTCATGTGATATCTACCCTTTGTCAAACAAAAATGTCA
TGATTGGTGGATCACTTGTTTCTTTGATGATAACTATGAAGGCAAAGTGA
GTCTACGGCAAGGTTTTAAATCGCGGTATCGGGTTACGTAACGGTAACGG
TCACGGTGTAACGGTATCGGGAGTATCGGCCTGTCCGTAACGGGAATCGG
CCGTAACGGTCGTGAATTTTTTAAAATCACTAGAACATCCCCAAAAACTC
AATAAGAATAATTGTTTCACTCATTTAACCTTTAATAAACACATTATAAT
AGATCTTATGACATTTTAAGGTTATATACAACACAATAAAGACATAAAAA
ATACGAAAAATCATCGTACTTAACAATTTGAAAAAAATAAGTGGATTGCA
CACATACTTAATAAAAATATATTAACAAAATATAAGTAAAAGCGTTAATG
TACTTGCTACATATTATTACAAAAACAATTTACACTCGTATGAAAATTAT
TATACCAAAAAATTGCGCGGCAGGCTGCTTCATAGTGCATCGGCCATGAT
CACCACTCTGTCTGTTTGTGCCACCTTCCTTATACCACCAAGTATTAGGT
GCCACAATGATCTAAATTGTTGCTAGGCACTCCCATCCCGCCGTACCGCT
ACATTCTGCTTCTTTCTTCGCAGTGACGAAAGGTGTAGAACTCGGAACTC
TTTGATTGTGGGGAGGGAACACGTATGGTAGAGTGGTGGTGGATTGAGTA
TCGGTCTTAGTAGGGGTGTGTGCGGGCATCCTAAAAGGACAATCCAGCAT
ACTGTGGCGCCGAAACGTATTCCGAAGAGGGATAAAAGGGGGTAGGATAT
GGTTGTGGGTTGACGCCGACTGATGAATAATTTCCCCTTGATATCCAGTA
TCCGTTATGCCTCCAAACTCCAGCATACCAAAGCCGGTTTTCATTCATTC
ATATTCTTCGGAATGAGATTGTGGTTTTGGTCTTCTTCTTCGATGATCCG
AATGCACTGGAAACAGAGATTGCTCTTCGTGGGCTCCCTAATATCCCCAC
GTTCGACATTGTCTGGCAAATTCCAGTCATTGTCCTAACTCTCCCATGGA

TCGGGCGAATTGGTCTAGTGTCTCCTCCACCTCCACCTCCTCAGTCCATA
GGGATCTCCACCTAGGTGGGCTCAACGATGGACCACTTTCTATCCCACTT
CCATCTCCAGATGGACTATCACTTTGCCACGTCGAGGTGCTGGCACTGGA
AGTGCGACCTCACACTCGCTTCGTCTAACCATCTGATTGTCTGATCATCT
ATCAAGGTTCTTCTCTCTTCTTGAAGCCATGGAAGATAGTGGTCATTTTT
CCAAAGATATAATCCAAGTTTATAGGGTTGAAGCTGTCTTCATTCACTTT
TGGGTTTATCTCGTAGCCACTGTCTACTTTTCCAACCTCATATTATAAGC
CACGTAAACAAGTTTATTCGTTTTTGATACTTTGAGGATTTCTCGTCCTT
TTTTATGGTATCAAGCTGAATGTGCTCCATTACCGCTCGAATTGAGAGGA
CGATGTTGTTGGCTCAACACGTTTAACCTGCAATCTCTTGGAAGTCAGGG
GCGATGGCACCAAGTGGGAATCCACCATTCAGTGTAAAACAATATGAATT
AACGGTACGAAGATTTGGAGATCTTATATTTAATGTTAATTGTTCATTTA
TAACCTGGGTTTGTCTGTTACTGCTCTTTGTGCCAAATTACCTGTCCCGG
AAAGACCTATGTTCTTTCACGGAAACATCATATCTTATTGTAGGGGAACG
TAACAAATAAAACCCACAATAAAACAATTAGAAACATTAGTACTTGTAGT
GCGTAGTTGGTTACCTTAAAACAAAAAAAAAAAAAAAAGAACTAACTTGA
TTAATGCTTGACTTGTTGTCATCAAATATTCTCCATCTTCTGAATCACTT
TGAACCTTCTATACTCTCTATGGTCAGAAAGTTACCACCTATTGGAAATT
GAGGATTTAAAATTACCTACATGAATTCAAAACAAAACAGCAAATTTTGT
TAAATTGAGTTTTCAACTTTCTGAATAAAGTCAAGAGTTTTAATTGAATT
TACACTTAGTATGTACTTACCATGCTGCAACCTATCATCGGTGTAATTGG
AATTGCCATCTGTTGTCGTCATTTTCAATTTTTTTGTAGTAAGCGGGACA
ATTTTTCTTGCGAAGTGGCCTTTCGCTTTGTCCATGTTCGTGAGACAATC
CCGCATTGTTAGCTTATCCTCCATTTCAAGCGGAAGCACACCCTCACCAA
TGGTACTTGCACTTTTAAAATTTCTGAAGCTTATCCAAAACCATACTTGT
TTCCACAATCTTTTAAGCTTTTTTTCCACATCCTCCTTCGGCCATTCTTT
TGCTTTTCACTCATCACCAATTAACATATTTGTAGGCATTTATAACTACA
ATGCTTCCAGCAATACAATTAGTGGAAAAACGCTGGATCCCGGGCGTCAA
TTTAACTCTTTCCTTCATGTAAATCTCTTCATAAAATCCAATAACCCACC
TAGAGTTATAAATGAAGCGTGATATTTCTTCTTGTCATCATCCGAAACTG
TCCAGCACATTTGTTTTGCTCTCGCAATATCTTTCAATATCAAATAACGG
CACATGTGGGCGAGCGACAACCTGTCCAATGATAAATTTGGTCTTTTTAA
CATAAGTTTTTGCCCCGCCGCTTTTATTGCAGCTCTTATCATGACGTTTG
GAAAACTATATTTTCAGTACCTTTCATCTCCACTTGTCCATAAGGCCAAG
TAACTAATCGGTAGTTTACTAACAACATCGTGGCATACTGATTATGAAAA
GTCCCGGATGCTATAATCAAAATTGATTTAATGTGCCTTCCGGGTAGGAT
CGGCTCATCCATCACACATGATCGTAACGCCCCTTTCCTCCGAATTGTTG
TTTATTTGAGATTATACTTTCTCATTTCTACATTTTAAGTAAGAAAACTT
CAGACACTCGTAAGGAGTAAGGGATTTCACTCTTTTCCAACTCGCTTGCA
ACTCAACATGGCGCAAGCCATGGCGACATTCAAACGTTTGTTTGGCAAAT
GTGGTGTCATCACACAAAATTGACTAATGCGACCCTAATTTTTTCCCCCT
TTTATTTACCTGTTGTTGAGATCTTTGTGTTGGCTTTGTGGCGTGTCAAC
TCATCTCGGCCAAAACCGCGTGTTCAAACGAACATCCCACTTTTTCGAAC
TCGATTCAGAAGCTAGAAATCTTCGCAAAAATTTAGAAATGGGCGAGTAT

GTGTTCCGTCTGGTGCTTTCCAACCCCCGCTATATACTCGTTCTGCCATA
TCGGTTGCCTGCATCCTCCTATATCTTCCCTATCTCTTCTTCTCTCTGCA
ACCTCATTACTTTTTGGTCGTAGCATAAGTCATATCCTGTCATGCCGATT
ATATCAGCGACTCATACGTCGTAAGATTATCTCGATCACGTTGGAAGAGG
CTCGAATTTGGTTGCACTTCAGCTTCTTCTCTTTTTCTTCTTGTTATTCC
ATTTTTTTCTCCAAGTTACCTTCAGATGTCATCAAGCGTTTCTATCGGCA
CTAAACACTTTGGGCATGGAGCTACTTGACCCTTTTTGGCCGCCAAGTGT
TGCTTCAAGTGTGAGTTATTCCCACCATTTATCCTCTCCACAGAAATCTT
ACATTTTAATTTGTTTGAGAACCATACATAGATCCCGGATTTCCATCAAT
TATCCATCACTAGCGAGGACGTGATCTTCTCTCAGCCGACATTGTTAATT
TTAATGTTTCTGGCACATTTCATTACCAAATTTATTCATACTTTTATTCA
GATTAGTAATTGAAGCTAATTAAACTACATTAACCATGTACTCTATAATA
TATTTATATTATCTGTATTTCATTAATATTACTATTATATGGTATTAATG
TGATAAATTAATATGACAAAAAAAAAAATTGCTACAAAAATATAAGTTAAA
AAATTTTAGGACTAAATTGTAATTGTAAAAAAGTTAATTAACAATTTTAG
GACTAAATTGTAATTGTAAACAAGTTAATTAACAATTTTAGGACTAAATT
GTAAAAAAAAAAAATTTTTAAACCCCTTAATTATTTCTAAATTACAATTT
CTAAATAATTTTAATGCCTAAAATCTAATTTCAGCCCCCACCCAAATCAA
ACACCCCCGATTGCCGTGAAATCCCTAACACCCGAATGGAGAGAGAGAGA
GAGAGAGAGAGAGAAACCGAGCGGAGCCGAGACCGGAGCGGAGGCGAGAC
GGCGACGGCGACGGCGACGAGCTGCAACGCGAAGGAGAGGAGACGTCGCC
GGCGACGCGAGAGCTACGTTCGGCGGCGATGAGCTGTTTCTGTTAGACGA
ACCAGACGAAATTCTCAACGTTCGACGAAATCCTCAACGTTCGGTAAGTG
TTTGGTTATCTTCTACTACTTTTTTCTCTTTCTTCCTCAATTATATGAAG
AATCTTGGAAGAAATCTGAAAAGTGTGCGCGAAATCAGGAGTCGGTCGGT
CTTTACAGACTGTAACGGCCGTTACGGTCTCCGTAACGGCCGTAACGGCC
GTTAACTGCCCGTATCGGCCCGATACGGGGCGATTCAGCCATAGATGCCG
GAATCGGCCGTGTATCGGTATCGGCCTACCCATTTCCGGTACGAATCGG
CCGTTACGGTACGTAACGGCCGATATTTAAAACACTGGTCTACGGTTTTT
CTCTTCTAGTATTGTCTCCGCCTGGGGTTGGGGAAAAGATTTTTTTGCTG
CTAGGAACAACCATCGTGTTCTATATTTTCATTCTCATTGTGCTTTCTCA
ATGAACATCATAGAGGCTTTTTGGTTGGTGTTAGCCTTCAAAGTTTCATT
ATTGTTTCTTTGTCCGCATAGAATTTGTTTTTGATTAGGCTTAGGTTTCT
GCATTGACTGGGACCACTGGGTTTGCTAGTCATTTGCCTACACATAAGCC
TTTAAATTACTAGTGTTTACTACTCATTTGCCTGTTAGTAAAGCTTCAAT
TTACTGGGGTTTACAAATTTGAAGAAGTTGAATTATTGTTTTTGTGAGAT
CATGGATCTCCCTATCCTCCCCTATTCCTTCCTCTTTCTTATCCATTTAT
GCAGTTCCTTTTTTGTTGGCTTTTTGCTTGACTGGTCTAGCCCAATTTGT
CCTGAATGAAATTTTCATGGAGTCCAAGCTTGATTAACCCGTTCTCTGTT
TTCTTATTTGTATTTCTATCTGCTGTTCGCTTTCTTTTTTTCTCTATAAC
ACTGTTGCTATGACCACCCAGAATTCTTCAACTTTGGTACCGTTTGATAA
CTCTTTTTTCAGTACTTTTTTGCTTTTCAGCAAAAAATTACTTATTTTTA
GAAGTAGTATTTGTTTTGTACTTATAAAAAAGTGCTTTATTTTTTTAAAG
TAAAAATATGATTTTAAATACTCTATTTAATCTAAAAAGTAGTTCCTATC

TGTTTCATCTATTTTTATATTTTAAAAGGTCGATGAACCAAGACCTCTCC
ACCCAAATGTTCTTATCTATTGGAATTCATTTGAGTTAAGAAATATTCTA
TTTGGCAATAATACTGGATAATACGGGAAGTCTGGAATCCTCAGCTGACA
TTCTTCATCCACTTATCTTGCGACAAAGCAGAATCATGAAGAAAAATGGA
ACTTCATAGTTGCAGTTGTCTTGAAGCGCTGACATAACTATGTTGTACTT
TATAAACAACAATTTAAATATCAGTGGATGTTCTTATGTGGAATCTTCTG
GAAAAATGGCTCTTGGGAAATTTTGTTCAAGAGAAGGAAAGAAAAGAAGA
ACTGAAAGAATCAATTCTTTTATGTGTTCTCCAATAAATTGTGTGTGAAG
GCTGCCCTCAGAATCTTCCCATAATGCTGGAAGAAATTCAAGGGAAATGT
CTCAGTGAAGAATAGTTATGTTGGTCGGAATTGCTATTATGTACACATGT
TGTAATGGAGAACTTTTTCCCCACCCTTTATTTCATTGTACTTGCTATAA
GTCATGTGATGGAGAGAGGGTGTACTGCGGTTGATGAGAACTAGGATGCA
AGGTTGATCAAAGGTGCAATTTTTGTATTTGTAAGACATACTAAAAGGCC
GCAGAGGAGCTGGAACTTGTAAATACCTGAACCAAAATTGACGTGCATAA
AGATCCTCAAAGAACTCTCGAAGGACCTGAACATGAGAATTAGGATGCAA
AGTTGATCAAAGGTGCAATCTTTTTATTTGTAAGATTATATATTAAAAGG
CCCCAGAGGAGTTAAATACCTGAACAAAAACTGACATGCATAAAGATCCT
CAAAGAACTCTTGAAGGACCTGAAATCTGTGGAAAAACTCACCATCCTGC
AACAGAACATGTAAAACAATTGCTGTTGGAGTTGGTTTTATGTGATTTAC
CAAGTCCCTGTCTGTCACTTGTTGTCGTAATTCCCCTTGCCTCTTTCTTC
TGTTCCTTCTATGGCCTTTTGCATGGTTTGGACTTTGGTGGCTGCATTCA
TGATCTTGATGATCTCCTTGATACTTATGCTGAGACTTGAGAGAGATTGG
CCACAATGACAGATTCCCATTGTATATCAGGAAGTTTCGTTTGCAATTTG
AAAACAATTTTTGAAAGCAGACTTTTTGGATATATTTGTGAAAAGAGATC
TTGTCGGAGTGGTTCTAAAAAGAGTTTTTCATAGGATGAAAAACAAAAAA
CATTTGTGTGGTTGCAAACCAAACATACTTGTCGACATAAAAGAAAAGGC
AATAGAAGAAGATGGCTTAACCAAGGTGGTCAGTTGCTTCCTCTATTTAT
AATAGAATATGGGTTGTGCAATGAAGTCTTAATATAAAGGCAACAACTAA
AGTACTCTTCTAACACACCTCTTCAATCTGAAGCATATATATTGTATTCT
CTCAACTTGTCCCAAATATACTTCATCCTAGAGTTCAAGTGGTATAGTGG
ATAAATATACTGACTGATTAAGAGAATTGACATGTCTGATGTGAATCAAT
TTTTATAGTAACTTCTTAAATGAAATGGCAATCATATTTAGAGTTCTTTT
TTGACTCCTCGAAAATTGGATTTGATAGGAATCATAGATTTTAAAATTGG
TCGTTATGTAATGGTTTTTGGGCCTTCCATTACCATTGTTGGCTGCCTTA
GTGGTGTGGCACTGATTTGCTCCCATAATAGGCCTTATTGGCCTGTAACG
GCCCGTAATAACCTTGATGGTTGTTAAGCTATGAAGCACGGACACGCGAC
ACAGACATGACATAGACACAGCGACACCAATAAATAAAGATATATATTTT
TATATACGTTAGCCTAATTAACGAAAGACATGTGCTATTAAGCAAATTCA
TTTAACAAAAACCATTAAACCAATAATCGTAAACATTCAAAGTTGAAAGC
AACATTCACATTAAAAAATTCCCATAAATAAAAGCAAGAAAGTCACAAAC
TCAAGATCGATTGGATGGTTATCATTTTCACACCCTTCATCTGTGAATAA
GACAGTTGGCTTTCTGTCTTTCTCGTTTCTCATTTCTCCAATTGTTAAAG
ACCCATTTAATAGCCTTATTTAATATCAAATTACAAGATACACCCCTTAA
ATAATAATGTATTGACAATAAATTAATTAACAAAAATTACAAAACTGACC

CCCTAAAAAGTCACGACACATGCATATGAAGTGTCCGACACGTTAATTTC
GCGTCAGTGCCGTGTCGAAATGTAAAATAAAAAAAAAAAAAAATTCGACAC
GCCTTGACACATGTTGGACACGTGTTGGAGGTGTGTTGGACACCGACACT
CAGCTTCCGGCAGAATGTCGATGCTTCCTAGCTGTTACTTTAGTCTTAAC
AGTCGTTACATAGCATAAAACGGCCGTTGCGGGTGTTTATGCCAAAAAAC
CCTTATTTCGCAACCAGCCTGCTTGTTTCGGCCTCCTGCCTTATTCCAGC
CTCCAGCCTTTAGCAAACGCCACCTTCGAGCCCTCCTTATGGGTGGTTCC
TTCAGCAAACAAAGAGAGAAGGGCGTAAGTTGGGATTGCAAGCCATTTTT
CCTTTCAATTTCTGCGATTTATGGTACAGATAAGCATTTCACACCCTAGA
TCTGTAGTTTCCTTCTGCAATTTCTGCCCAGATTTCATGTCTCTTTTGAT
GCACAATCATGATTCGTGTTTGCTGCCCAGATTTTGCGTATTCTGCCTGG
ATTTTTGTTCATGTGTAAATTTCCTTTGTTTCTATATTTTTCTGTGTTTT
TTTTTTTGAATTTCGAATCTGATTTATAATGTTGTAAGCTTGTAATGTTG
TATAAAATTAGGCATTGTCGTAAATGTGGTTTTAATGGAAAATCAGATTT
TGATTTTTTTTATAAATCTGATTTTCCTGAAAGTGATCTGATTTTGTTGT
GAACTCTGATTGTAAATCATTTTTTTCAGTTTATAGTTGTATTTGAATTA
TACTTTATAAACTTATAATTCAAAATGCTAAATTTTTATGTGTAAATTGC
ACACCATTATTATTAAAAAAAATGTCGGGGGAAGAAGGTGTGGTTCCTCGT
AGCAATGATATTCAACGGAATTTTGCAATTCCTATTTCAAATAGCTTCGT
GTTTGAACATTAGTGTTTAGGGAGTTCATGATGAAACATCTTCAAGGCTT
CATGGGGAAAAAATGGATGAAAGGAAACAAAGAAGTTACTGAATAAATTC
GTCCTTCTCAACTTAATGAAGATGATCCTTACGAAATTTTAAATGATGAT
GATTTAGATCCGAAAATCAGTTTTGCACGGAAAGAAAGCGAGTGCGAATT
GAGAAGAAGAGGATAAGGAGAGATTCAAGAGGAATAAAAGCGATTGATAT
TGGACGGGGGAATATTGGGATTGGTGGAAGCAACGAACGGAGAACTAATC
GTATTGCTAGTATTTTTCGAAGATCTACTATTCGGGAAACTAGTTCTAAA
AATTCTTGGATTGATCCAACGTTGCTCGAATCACAATTGGTCGATTTTGG
TGTTGGTCTACGTCATAGTAAAGCCCCAAGCAGGCTAAGCTCTTGACAAG
AGTAATGAATGGGTTGAAAAATTAGGCATCACAGTTAGTCAGTTTGTATT
GTAAACACGTTTGTTGGAAAATGTCGTTGCTTTGCCATGGTTTGAGATAA
TTTTGGATGTTGCTAGGGAGGTTGGGAAAGGAGTGAAAGTTTGTAATCTT
TATGAAGTAAAAGTATCTTTTTTTTTAATCACTCTAAACTTACAAGTGCA
TCTAAATGTATTTATAATGAACTTATGAGCTCATGGATTTAAGAGAGAGA
AGAAGCATTGTTTGATGATCAAACAATTCAGATTGGTTACATGTAAGTGA
TGATGATCAATATTATGCCACTAGCAGCTGATAAAGCTGGAATTTTGTCA
ATGGTCCCAACTTCTAAATCCCTGATCAACACTTGCAAAACTTAGGATAA
GGATAAACACTTGTGATACTAGTGTGATGTCGGTCAAATACCCTTCGGCG
TGAGAATAAGTTAATAAAGTATTGAGTGATATCATAATAGAATGTAAATC
GTAAATGTTTGTAAATTACCATTTTTAAATAGTGGAATCTCCTAGATTTG
GGGAGAATAATATCTCCTGGATTTGGAGAGAATAATATCTCTTCGATTGA
GGGAGAATAATCTCTCTCAAATTTAGGGAGAATAAATCAAATTGAACCTG
ATTGACCGGTTCAAATGTTACGCCATTATGGACCTTTGTTTCTCGGCCCG
AATTGATAGTTACTTGTTTCTCATGTTTTATAAGATATGGATTATTCCCA
TTGAAGCCCATTTCAACATCGACTCACTAGGCCTTAATGTACGGGTACTG

ATAACGCCAGAATTTTGTCAATGGTCCTAGCTCCAAAATCCCTGATCAAC

ACACCTGCAAAACTTAGGATAAGCACTTAGGATACCGATTTGATATCGGC

CAAATACCCTCCGGTATGAGAATAAGTTAATCATGAGTTAAGTGATATCA

TAATAGAATGTAAATCGTAAACGTAATCGGTAAATACATTGAAATCTCTT

GGATTTGGGGAGAATAATATCTCCTCGATTTAGGGAGAATAATATTTCCC

AGATTTAGGGAGAATAAATCAAACTGAACTTGGTCGGCCGGTTCAAATGT

TACACCATTATGGATTGTAGTTTCTCTACCCGAATTGATAATTACTTATT

TTTCACATTTTATAAGATATGGATTATCCCGTGAGAGAAATGAAATTGAA

CCGGAATCAACGAAAAAGGCCAAGTTGAATAACGAACTCAAACGCCTGAG

GAAACTGCTTAAGGAAATTACTTTAAGGAAACTGCTTAAGGAAACTACAA

AAGTTAGGTTCAGCAATGAACCCAAGAGCCTCAGATCTAATCTTAATCCA

GGTACCCCAAAACTTAGTTCAATTATAAATTTAAACTAGGGGATAAAATA

TGACCATGGAAGCACAACTCAATAATACTGACCCTATTCAAAAAAAAGGG

GGACAAGAGGGCAGGTAAGCTTAAAAAAACATGTCAGCACAACTCAATAC

CCTATTTAAAAAAGGGGAAAGGGGACAGATAAGCTGAAAAATCAGGTCAG

CGCAACTCAAAACTAAGAGCTCAAAAATATAAGGTCAGCCTAATGAGGTT

AGCTTTAACGAACTCAAGAAACAACTCATTATTCAACAAAGAATGAGGTT

AGCTTAACGAACTCAAGAAACAACTCATTATTCAACAAAGGAAGGTGAGC

CTGTTCACAAACTGACCTCACAACAAAGAAAGGTCAGCACGGTTCACAAA

CTGACCTTAAAACAAAGGAAGGTCTGCTTGTTCACAAACTGACCTCACAA

CAAAGGAAGATCAGTCTGTTCACAAACTGATCTCAAAGCTAAGGAAGGTC

ATCCTGTCGACAAAAGGTTTGCATGTTCATAGATTGACCTTATCACGAGG

AAAGATCAACTTACCTTGCAAAGAGAGGCAAGCCTAAAAGATTCACAGGT

AATCTAAAAGGAGGTTGGCCTATTGACCTTGCGAAGAATATAAGCTTCCT

AGAAAAGGAGTCAGGTCAGCCTGATTGGTTAAATTTCTGAAGGAGATAAA

ATCCAAGTAGAGGCTAGCCTAAGGGCTGATCTTATGGAAGTTCAACCTGT

TAAACACAAACAGATCTGAAAGCAAGCGAACCAAGAAAGCTGAAAGCAAG

CAACCAAGAAAACTGCTGAAGAAAATGCAAAGGTCAGGTTCATCCTATAA

AACCAAGTGCCTTAAGGCCAAGTTCGAAGACAAACTCAAACGCCTAAGGA

AACTGCCGAGGAATCAACATGAGTAGAGGTCAGGTCAACTCACAGATTGG

CCCTAAAAATAAAAAGTCAAGTTCAGCCTCTGAACCCAAGCGCCTAAAGG

TAAAATTTGGTCGTGAAACTCTCTTGATTTAAGAATACTCTAAGAATATA

TTCTTTTATTTGAGGAACCTCCGACCAGGAGGCAATAAGAGAAGACATTG

GTTAAACTCTTGAAAATTGGCTCATAAATTATCTTTAAAGTTAAAAAAAA

ACTAATGAACCACGGGGGCAAATGATGAAGGTAAAAATCACTAGACAAAC

CCGTATATTGAGGCCTAGTGAGTTAATGTCAAAATGGACTTAAATGAGAA

TTTAACCCATATATTATAAAGTGTGAGAAATAAGTAATTATCAATTCGGG

TCGAGAAACTACACTCTATAATAGTGTACCATTTGAACCGGTTAACCAGA

TTCAGTTTGATTTATTCTCCCTAAATCTGGGAGATATTATTCTCCCTAAA

TCGAGGAGATATTATTCTCTCCAAATCCTGGGGATATTTTTCTTCTCAAA

TCCAGGAAATTTTACTCTATAAATACAATAATTTACAGGTTACATTTACG

ATTTATATTCTATTGTGATATCACTCAAAACCTGATTAACTTATTTTTCC

CGCCGAAGGGTATTTGGCCGTCACTACATCTGTGTCCCAAATGCTTATCC

TAAGTTTTACAGGTGTTAATCAGGGATTTGGGAGTTGGTATCATTTACGA

AATTCCAGCGTTACCAATAGCCAAGCACCTTGACTTTGTTAGAGTAATAG
TTCATTGGAATATGGTAGTGGCTTTGATAGTGTTCATGGTTTGAGCCCGC
CTAGTGGACCGTCTAGAGATAATGACAATGATGATCATGGAGGAGACTAG
TAGATCTCCATATTGTAAAGAGGTAGGATGCCTGGATTTGCAGGCAATGT
GGAAGTTGATGATATCTATGAACCACAAAACATTTTATCTCTGGTGGTTG
GAGTGGATTTCCAACCTACAATCCTGATGAATATCATGACATCACAAACC
GCCTTGGTCAACTAGGAGTTAGAGGATATCAAGGAGATAGTTATCATTAG
CCTCAACCACCATATCCTTATCCCGCATATTAATTTTCACGATCATATTA
CATTTGTCCACCGCCACATTATATGGTTATGGGTTCTTTAGTATACTCCC
AACCTCTTGAATACTTGTATGCACCACCTACTTACCAATGTCCCATCACA
TCGAGAGAGAGCTTTGGTAATTACGAGCAACATCAAAGAGAGAACCTAGT
GGTAATGGCAACACTATTTAGATCATTGTACCTTTTCGGCATGTTTGGTA
AACGAAATTCGACAAGGGATTGAAATAAGCATGGGAATGACCATTCCATT
CCCAAGTTTGGTACATGGGAGTAAATTTTATTCCTATTATTAAGGATTTT
CTAATCCCACAATATATAAGAATAACCAGTCTCAAGATAAACTTGATATT
AGTTAATCCTAATTACATGGGATTAGGTTTTATTCCTTCATCGGTCAAAA
ACACGACCAAACACTAACGAAACATTGGAATGGAAAGAAATTTAATTAAA
TTACTATTTTATCCTTTGAAAATTTAATTAAATTGCTATTTTACCCTTTG
AATTTCTAAAACATATATCTGTTAATTTTTTATCTTTTACACTTCTCTCA
TCTCTGAAGGGGGGAGCCTTGGCACAACGGTAAAGGTTGTTGCCATGTAAC
CTAGAGGTCACGAGTCTGAGTCGTGAAAACAGTCTCTTGCAAAAAGTAAG
ATAAGGCTGCGTACAATTGACCTAAGTGGTCCGACCCTTTCCCGGACCCA
GCGCATAACGGGAGCTTTGTAGCACCGAGCTACCCTTTTACACTTCTTTC
TTTCCTACAGCATCTTCTCCGGCGAGAATCCGACGCCGGCTGTCTTTTCT
GGATTTCTCTCTCTCATGCACACACATACACACACACACCTTCTAATTAA
TCCCCCAACCCTGCAGGTACCACCAACCCCACCCGGCCGGCCGATAACAC
CACAACCCATATCTCACCCGCGCCCCACCACCTGTCCACCGCCTCCATCT
TCTTTTTCTTCTTCTTTTCAATCTCTCTCTCTCTCTTATATATATATATA
TATATATATATATATATATATATATATATATATATTTTATATTAGGCNGTAG
AGATTTTATTGTATATATGTATTTCATTCCTGATTTATTCCCAACACTAA
CTAAACACTGGTATAAGAATGGTCATTCTAATACCACATTTAATTATTTT
TGTCACCGAACATAGAAAAAAAAGGGTCCATTCCATTACCATAGTAATTT
AGATCCTAGTGTCATTTCATTACATTCCAGCATACTAGATGCAACATTAA
TTACTACAGTGGATGTGGCCAAGAGCTACAAAATCAGTGGTGATTATGCC
AATGTGGAATATGAAGAGCTTTCACATAAATCCTTTAGGTATCGAGTACT
ATGATGACATGATGTGTGGTTTACTCTTTTATTATCTTTGTCTAATATAT
TTGCATTACATGTTGTGCATTGTCTTATTCTCTTAAGATTGTTAAGTACT
TAATTTCTATTTTTGGCATTTTTTTTTTATATCTTTAGTGTTGCATATAT
GTAGTTTGGTGTCAAAAACATATTTTCATTAGATGTCCCGTTACGGACAT
ATCAATATTCACAATATTGTTACACTGCGATCTTTACCATTATGTAACAC
GATATCGCTACTTAAAACCTTGGATACGATATGGATGCTGCTTGATAATT
ATATACTAAAACTCATATGAGGAAAGTGATTGAACCCAATTTCTTCTAGT
ATATATTTCAGCCAAATCAACTCACAAGTTGTATGTTTCATAAATGGAGA
TTTTGGTTTAGTACGTGATCAGGCCTTCACAATATATTGTTTACTCTTCT

AAGAAATCAAATTACCTCCAACAAATACCCAATAGTCTAGAGGATTTTTT
ATCTGAAGGTGATTCGACCCAGGTAGCTTTTGTATGTCTTTTTATCCGAG
TATGACCAAGTAGACTGTAGCCTAGTGCACCCTTCAAGCAATGTTTTAAA
ATCGGACTATTTGTGGAACTAAAAAAGAGACAGGGTAATGGTCATTGGTT
TAACCGTCGGGTCAACCACGGTTGAACAGTTACATCATAATTATGTTACT
AAATATTAAAAATATATTAATCTACATTTTATAATGGAATGAAAACATAT
AGGTAAAATCATTAAGAAATAGTGAAACTTAAACTCATACTATACTAACG
AATATTGCCAATACCTCCAATAACATATATATTGCAATCCCACTCCGTAG
AGCTAAAGTAGAAATTGGTAACAACCTTTGTAAAATCTTTAAGTGGCTTG
CACAGTTAAGCCATGCATGTGATTAATGTGTGTATGCTCTACGCTCTCTA
CTACATGAAATCAGGAAATTATTTGGACAACTTTGGTTCGTATAACCGCC
TGGGTTTTTCCGTTTTTATTAAAAGGAACGGTTTTAAGGCACTCTATTTT
TGGGAGTTTGATTAGCTCGTTTTATGAAACAAGTTGCGGGTTTCTGGTCA
GATCTATCAGTTTGGTTGGGTTTTTAAAATACTATTTCCAAATACCTAAG
AATGCATATGTCTTCCCAATGATTGGTCCCATCTCGTAACAACCAAGTAA
TTCAACTTCCTAACTAACGGTACTTGTTTGCATATGTCAACAATTCGCCT
TATTTTGCCTATAGTTTTTTGCAAGTATGTGTAGGTTAATTTACTAGTGG
ATCCAAGCATTCTTATTTGGCTCAACAAATTAAGAACATATTTCATCTAA
GAGAGGTAGATGCGTTAACTAGATCGAACAACATCAATACCCAAAAAGTA
TTTCAATTTCCCCAAGTCTTTGGTCTCAAATGTTACATTGCAAAATAGTT
TTCAGTGTTCGAATCCCGTTTTGATCATCTCTTGCAAGCACAATATCATC
CATATGCATAATCATTAATACTATCTGTTGTGGTATGTTTGTAAAACACT
GAATAATCCTTCCCATATCTATTAAATGTCGTGATCGCATCACTTGATTT
CCTAAACCATGCTCAAGGAGATTGGTTGATGGCATAGATAGATTTTTTCA
AACGACACACTAGTCTAGACTCTTCACACTTCCCCTGAGCAACAATTCTT
CAAGGGAGCTCCATATCCTCCTCGTTAAGTAAATCACCACTCAAGAAGTC
GTGATTGATATCGAACTGATGCGAGCTCAGCAATGTGTGAAAGTCCCATG
AGTAATCCAGGCCATATGTTTGTGTATAATCTTTGGCAACCAATCGAGCC
TTGAGTCAAACAAGTGTTATTTGGATGAACTTTGACAGCATACCATTTGC
AATCAATAATTGTCTTGCCAAGTGGAACATATACAAGATCCTAAGTACTA
TTTTGGTGTAAGACAATCATTTCTTCCTCAATTGTAGTATTCAAACTAGG
TTGTGGGAGCGTATTTTAAACAAAGTTGGGAAGAGAAACATAGGACAAAA
TGGTAACAAAAGCATAATAAGGGGATAACTGCGCATATGAAAGATAATTA
GAGATATGATGTTTAGTGCGAAAGAGCTAAACTTTCCAAATAACAATGGG
CAATTTCCAAATCACACAATGGTTGGATAGTCGCAGACAGATAAGGTACA
AAAGGAGAGGAGATGCAATGACCTCGGTAAAGAGGGTGGACATAGACTGT
TTGACAATTGGTGTAGACTTGCAAAGGAGGACAAGAAGGACAACTTGGGG
GATACGTTCGAGTGGATGAGGACAAACTAGAGGTAGGTAATGAGCTAGGT
AGAGGGGGGTAGGTAAGATCAAGATCAAGATCAAGACGAGAGTGAAGTTG
AGAGATTCAAAGAAGGTAATATCAACATGCACAACCTATAGACGTAAAGA
AGGACTGTAACAATACCTCTTTTGAGTGTGAGAATAACTAAGGAAGACAC
ATTTCAGGGTACAAGGGTTTAGGTAACTTCAAAATTCATTCACTAATGTG
ATAAAATAACAAAATTCTAAGGTGATAGTGACACGAGACTAAGAACTCTT
TGGTTAACTCAGGAAGCAAATGAAGTACGATGGTTTTTTTTCCTACCGAC

AAGACACACAATTTAGGGCAGATATGCGCAGACTAGGAACAACTAGCTTG
AGATTTGATAGGGATGGGTGATCAAGGAGACAATGAATTTACGGGCGATA
TTTACGCAGACTATGACCAGCTAGCTTGAGATTTGATAGAGATGGGTGAT
CAAGGCTACAATGAATTTGATGATCAGAATGGTTGGGCATACAATGGGTG
TTCTCTTCAAAATAATGAATATCTTTTTTCTCATTGTCGATTTTGTGTCT
TCAACTCCTAAATAAGAACAAAACTCGTGAAAGAACAAAGCAATTGATGT
GCTTAGTAAGTTTGTTGATTGATATCAAATTAAAAGAAAATAAGGAAATA
TGGAGTACGACAATAAAGATAAAGAGATTTGAACACCTCCACACCACACT
ACTAATAAAGACCATGGAACCATCGACAATGGTAACGACATGAAATAAGT
AAGGTCATTTAATGTGTGCAAAAAGTGAAGGGGGTGCATTATATGTGTGG
TCACTGGCAACAAATTTATGACCCATGGAGTGGGAGAAGAGGGATGAAAG
GAAAGACATGCAATAGATGTACCTTGCTAAGCTAGTGAATTAAGGATGAG
AGAGACTTGTCATGTCAAACTGCGCATGCACATCATCAAAAATCATCTCC
TTGGCAGCCGGAGACTAAGGCTTAGTTCAGTCCCCCTGTTACTTTTAAAA
ATGAGCTGTCAAAATTGAGCTTTTAAAAATGAGCTATTGAAAAGGAGTTG
TGAGTTGTTTGGAAAATTTGTCAAAGAGCAATTGTTGAAAAGCACTCGAT
GAGCGTTCTGTAAATATTTATTAGAGGCATTGAAAAACTATTAAATGACC
GAACCAACTATCCCTTACATTAAAATATTTTTATCCAATACTAAGTATTT
ATAGCAAAATGCTAACAAGTGAAACAAAATGTTAAAATCTAATTGTAAAA
AAGGAATGAACCAGTGAAATTTAATTAAAGAGTGAATAACATTTTCAGTG
AAATATTTTTATCCAATACTAAGTTTATAACAAAATGCTAACAAGTGACA
CAAAATGTTAAAATCTAATTGTTAAATAAGGAATGAACCATGGAAATTTA
ATTAAAGTGTAAATAACATTTTCAGTTATATCACATCGATGGTTAAAGAA
ATTTGCACAATTCTATTGTTTTATAATATTTAATGCTTTAAATAAATTGT
ATTTTAAAGTTATACTGAATCCAATTCAATCTACCTTTTAGGGCATTAAA
CGAGATATACTAAATGGCCAATTTCTAATGGTAGCATATAAATTTCATGC
ATCGAAAGAGAATTTTTTTTTGGGGTTTCAGAAGCACCTGTTGTGAGGCT
TTTAACGGAGCAAACAAGGAGAGCAAACTCTGCCTAAAAAAAGTGTAGGAT
GATCTGATATGAGGTCACAAACATAGGACTTCGGAGGGTGGCCAACTTAG
GGCCTGTTTGATTAATATAATTTTGAATGCATTAAGCACTTACCAAATTA
AGTGATGTTTGATACAAAATGGAGCAACCAATTAAATATAAGTATCAGTT
AAAAATTGTTCAAAATTTTAAGTGATTTGGAACTCACTTATTTTTTTAGC
ACTTCAACCATCAAATATCTATCTACGACAACCACCATTCAACATCACTA
CTATAAAATCTTGCCAAAAAAAAAAGATTATATATAAACAGTTATAGATA
GAGAGAGTACTAAATTGAATAAAATAGAGAGAGAAAATTGTAGAGAGAAT
ATTTTAGAAAAAGAGAGTTGTAAAAATTATGTTGGGATATAAATTTTTAC
AAAGATAAATTTTTTGAAGTTCTATGTTTTTAAAAAGTATATACTTAATA
TGTTCAACACTTAATAATTTCCACACTTATAAATTCAACACGTAAACTAA
AGCAATTAAAAGTTTTCAGCTCTTAAATTTCAGTGTTATCAAACAGGCCC
TCAATCCCAACAAAGCCTCTCTAGTATGGTCTCCTTGCAACAGAAGAAAC
TGGGGGTGAGTGGGCTGAGGTTAACAAGGTTCAGCGATGGTAGTGGTGAG
GTGATCTGTTTCAACGAGGGGCCGAGGGCTGATGAACTATGTACGATCCT
TTCTGACTAAAGTATTCTTTTAATGTACAGACGGTTGATTTTGCAACATC
TCTATCTATTCTTTGTGAAAGGAAAACCTGATATACACACATACAAAGAT

CCATGAGCAACCAATACAAGCCACCCGTGATACTTTGAAGCCATGTGTAT
TGATTGCAGGAGCTAGAATTCCAAAATTAATTCTCTGTTACTGCCATTCA
TGCTGGATTTAGATAGTCTTGTTCATTTTTTGAAGTATTTGTAGTTCTGT
CAAATGTTCTTTGTCACCTCTGTTTGTCTTTGTTTCTGCTTTTGTTTTTC
CCCGTGACTGAAACTTTTTGCAACATAGATATAAAACTTTTGCTGCAGAT
TATTGTGCTTTCTTTCTTTCTTCTTCTTCTACTTCCTTTTCTTTTCTCCA
TTTTTTTTTTTTTTTTTTTTTTTTTTCTGGTCACGAAATCNTTTTTCTTT
CTGGTCACGAAATCATTATGCATTCAGATGGAATTTTTTTGTTCTCATGC
AATTTAGTGATCCACTCCACTGAATTCTGATGTGGTGCAACTATGTTGGC
ATTTGTTTAGTGCTTGTTTTCCAAGATTCTATTTCTGTTTCATTCTGGGA
ACAGAAACATAAATCTGTATGTTCCCGTTCTGTTCTGATTAATTTGTTCT
TGAAACTTGAGAACGAAACAGAAGCATATTTTTGTTCCCATATTTTTGTA
TCTGTTTCATGTTTTCTGAGAGCGGAAACAGAAATCTAGGGACAGAAATC
CTTAACAAATGCCACAGACACTTCTGTTTGTGGAATATTTCTCTGTTATA
ATGGTATTGTTTCTAGTTGCTTTCTGATGTGAACAGGCAATCAACAAATT
TTTGAATTTGCTTTTTCCAGGTTGGTGATCTGGGTCTGTCGAAGGTGAAA
TGCCAGACTCTAATCTCCGGCGGCGTGCGCGGAACTCTTCCTTGGATGGC
ACCTGAACTTTTGAATGGTAGCAGTAGCCTTGTCTCAGAGAAGGTTTGTT
CGCCTAACCCATCTCTCTTCATGAAAATACCATGTAGATGAATAATCTGC
CTTTAGCTAGTGTTCGGGAACACAAAATTGGACATTAAGAACTTGACTAT
AAAATGAACCTATGTACATATCTCGGCCCCATTTAGTCTGGTAACATTTT
TCAAGTTCCGTTTTTCCTTTTTCTTTTTTGACTACAAGGAAAAACCAGGG
AAAAATGGAATTTTGCACGAAAAAAGTTCCTTTGCATTTTTAGAAAACAA
GACTACAAGTAGAAATTAGCTATGATCTCCATTAGTTATTCTCTCATCTT
TCACGAAGATGAGATAATTATTTCATACTTATTAATATATTTTCATTATT
CTTCATTTGTGTTCTTAAGAACTATTGCTTTTGCGTAGAGTGCTCCCCAT
TTTATAGTTGGAGTGTTAAATAGTTCTCTATTGACTTTGATAATTAATTT
TAAGTCCTACTTTGACTATTAAGTTACTGATTAGTTATAATTTGCCGTAA
GATTTTAATAATATAGTTCTGGTGCTATGAATTAAATCTTGAATTGATGT
GACTAGAACTTACAGAAATCGTAAAAGTTGGATTGCTCTACAAAATTTTC
AACTTTTTATTAAATAAATTTTACCAAACAGTGCATGAATTTTGAACTCC
ATTTACAGATTGTGCTGTCAAATACTATATTAATAACATTACTGAACTCC
ATTTACAGATAGTGCTCTCAAATACTCTATTAATAACATTACGCGATCAT
TTGTATTCTGCTTTTTCAGGTTGATGTATTCTCATTCGGTATTGTACTGT
GGGAACTTCTTACCGGAGAGGAACCCTATGCAGACTTGCATTATGGGGCT
ATCATCGGTAATCTTTCACATTTGCCTTTGTTGCCATCTTTCTTCACCCA
ACTGTACTCAAAAGCCATTAATCATTTTTTATTTTTTTTTCGCAATTTTT
TTTGATACTATAAAAACAGGCGGTATCGTGAGCAACACATTGCGGCCCGC
GGTGCCGGAATCATGCGACCTGGAATGGAGATTGCTGATGGAGAAGTGCT
GGTCGTCTGAGCCATCTGAAAGGCCAAACTTCACCGAGATTGCAAACCAT
CTTAGGTCTATGGCAGCCAAGATTCCAATAAAGGGACAAAACCCAACAGC
AAGTTCTTAA
>EUC24537-RA [gene]
ATGGATAACAGCAATACATCAACGCCGCCGGCGGAAGAGCTTCTGAAGAA

GATCCAAGAGCTGGAAGCTGGCCATGCCCACCTCAAGCAAGAGATGTCCA
AGCTCATAATTTCTACAGATCATCGCAAATCGGAACGACAGAGGTCTCAC
TCGATATCCCCGCAGCGGACGGCGGTGCCGTCGCGTAGGAGGGTTGGTGG
GGAAGGAGGATTTGACGGTGGCACGGTGGCGGCGTGGAAGAAGGGTTCCG
CCTCGTTCCGGCATTCGTCGCCGTTGCAGAGGGAGAGCCGTAATCGGGAG
GCCTGTGGTGGCGGTGTTGGTGGCGGCTGTGGTAGTGGCCCGGCGGCCGT
GAAGTTTACTGATAAGCAGTATCTGAATATATTGCAGTCAATGGGTCAAT
CGGTGCATATATTTGATCTCAACCGTCAAATAATTTACTGGTGAGTGAAT
TGAATGAGTTTCCTAATTTAGCTAATCCTGTTGCTCATTTCTTGATCTTT
TTCACATGGGTTTATCTGTTTCTTGATATTATCAATTATTCATTCCTTGA
CTTCCATTAATGGCTGCTTTGATTGGCGCTTATTGATGTTGCCAAAAAAT
CTCTGGTACGTGTTTCAGCCTCTCACGCGTCGAAACCTCCGCAACCCGCA
ATCCGACCTGCAAAACCAGTTAGTTTTATGGGGAGACACTGGTGTGGTGT
CGGCTAAAGAACTTCTGGCGTGAGAATAAGATCAGACTGCTTTAAGAGAT
AATGAAAGCTTTAGAGAGTATATTGAGTATAGTATTAAGAGTTGTGTGTT
ATACCTCATTTCCTTCTGCATATATAGGCCGTACCCTTGGTAACCGTCCT
CGCCATGATCTCCTCCACATATCATACTTAACTGCCGTGAATGTTGCCTG
AGCTCCTATTACGGGATGTCGCGTTAATCATGTTCATGCATGGGGCGGGT
GGTAGGTAAGGTAACGGCTACCGTTGACCTACCATACTTGGATCTGATGA
TCAGGCCTCGCCTAGGAGGCCTTGCCTTGTGCCGGATAAGCCCATGTGTG
GCTGATCTTCATTAGCAGGATATGTGCCTCATTTTTTTGGGTTGTTAGAG
CAGCTGGCCCATGAATAATTACTGGGCTACTACTTTCGGGTTGGGTCTCA
CCAGAAGCCCTGAACCAGCGTTCCACCATTGTCTGCTCCAACTCATGGGT
GGGAATTTTTCCCACATCAATTATAGTTCTCAAAATTGCAGATAAGGGTA
TCTTTATCGGTTGAAACCGACAATTGATTTTCACATTACTAAGATTAACT
AGGAAATATTTACTGAATAATTTCTTGAATGCTTAGATCTTGAGATTTTG
CAGGAATCGAGCTGCCGAGCTCCTTTATGGTTACTCTGCAGCAGAAGCTC
ATGGGAAAGATCCCATTGAGCTCTTAACAGATAGCCAGGATTATGCTGTT
GCTGATGATATAGTCCGTCGTGTGGCAATGGGGGAGAGCTGGACTGGGCA
GTTTCCTGTTAAGAATAAGCAAGGCGATAAATTTGTAGTAATTGCAACCA
ACACTCCCTTCTATGATGATGATGGAACTCTAGTTGGGATTATTTGTGTT
TCGACAGATTCACAACCATTCCAAGAAATTAGGGGTCCGTTATCTAGTAC
AAAGCACGGTGAATCAGGCTTCAGCCGGCCAAGAAGCATTGCTTCTGCTA
AGCTTGGGCTTGACCCTCAACAACCTTTACAAACCGCAATTGCTTCCAAG
ATTTCAAATTTGGTATAAAATGCCTTTTTTTTTCTTCCCAAATATTATTG
TGTGTTTGGATGAAGGGTTTTGGAAGAGGATTTGATTTTCATAAGAGATT
TCTGAAATTCATTAGAATTTCTTGTTAGGCTATGTCATAAAGGAACTTTA
CAAATTCCTTGTAATTTCGAAATGGGATTTAAGGGGGGACAAAATGACAT
CTCCAAATACCTCCTAAATTAAATCCTTTTGAATGAGGTATACCTCTCCA
TCTTGAGGTAAAATTGCAACAATAAAATTTCAGTTGCAAACAAATATACC
CAAACAATGAATTTGCAAATGATGTGATTTTAAAATTCAAAATTTAAAAG
TTCCATTGTTTTTTTAATCAATGAATTTATCAAATTCATTATCCAAACAC
ACTCCTAATGTTGTAGCATACATTAACCTCTAAAACCCAAAGCAGGCTTC
TCGGGTGAGCAGCAAAGTTAAGTCAAAGATCAAAACCGGAGAGAACAACA

TGGTTGGTGAAGGTGGGAGTGGAGATAGTCATCATTCGGACCACGGTTTC
TTAGATGATCATAGGGAGGATGCTAACTCAAGTGGAGCTAGCACGCCGAG
AGGAGACATTAACCAATCTCCCTTTGGAGTGTTTTCTCAACATGCGAGGG
ATTCTGGTGACGATAGTGAAGGAAAACCGGGGATCTCAAGGATCATTAGC
TCAAAAGCTGAGGCATGGATGGGTAAGAAAGGCATATCATGGCCTTGGAA
AGGAAATGAAGGAGGTTCAGAAGCAAAAACAACTAGATTTGTTTGGCCTT
GGTTACATAATGAGCAAGAGAATGACCCGGGTCCTCCAAAGAGTTATGGT
TCTTTTACAAGGCACGAGAATCAGGTGGGTGAAAGTAACCGAAATGCTAA
CAATGAGGCTTCAGGGTCGTGGTCCTCATTTAATGTCAATAGTACAAGCA
GTGCAAGTAGCTGCGGAAGTACAAGTAGTAGTGCTCTTAACAAAGTGGAC
ATGGACTCTGACTGCTTGGATTATGAGATTTTGTGGGAAGACTTGACGAT
AGGTGAACAAATTGGACAAGGTAAAAACACTTCTGGCTATAGGTTAGCAG
TACCACTAATTTGTGTTTATTTTGCATAAAGAAAAGAATATTCTTTGATT
GGGTTTGTGCTATCGCAATTTGTGGAACCAATTATATGTTCCGATTTAAA
TGGTCATCTACTAATCTGTGCAGGATCTTGTGGAACTGTTTATCATGCTC
TGTGGTATGGATCAGTATGTTCCTTTGCTCTTCCTTTCTGATACTGTTAC
TTCAAATAGATAGCCTGAAAGCAGCTGCAGAAAGTTAATTTAACTGTGAA
GATGAAGTCTTGCTGTCAATTGTGGTGGGTGTCCTTGTCGTCATGTATGA
TATATGAATATGTTATTGTTGTGCAGGATGTTGCTGTTAAGGTGTTTTCC
AAGCAAGAATATTCAGATGACATGATACTTTCATTCAGACAAGAAGTGAG
TGTTTTTTCCTCACTATCTCACATTTACTTAATTTATCCTATAAACTCTA
CTCTCTAGTGGTTAATACAATGACTTATGAGCTCCATGACTAGCATGCTT
CGTCTGCTACATTTTATTCAGACTTGAGACATCCACATTAAACACATTTT
AAAGCAATCATACTTCTTCTACAATTTCCTAATGCTTTTTGTGGTCAATA
TTCATAAAGATGTGCGGTAAAGTATTGTAACAATTGATTATAAGGGTCCA
TATCTTAGTGTTATCTCTATAAATGATCTCTTAATCNCCCCCCGAAAAAA
AAAAGAAAAGAAAGAAATAAAATATGGTGGTGAGTTGAGTAAATGTGGGT
GTAGAGGTCTTAGGCTAGGAAACATCGACACTTCTAGTAGGCCAATGTAT
TTGAGTCTGAAACATTTTGGACACGCCTAGATATGTATTCGGCATTGCCT
AAATCTATGTCCTAATATTTTGTGATTAATTTATTATTTGGGCACTTCTT
CGATAAGCGAGGACACGTGTGGGACACAACAAGTGGTACTTCTAGTAATA
TTTAAGAATTAAGAGGGTTGAATTTTAAAACATATTACACATCTATAACG
GTGAGCTAAATTTGGAAGAATATCGCCCTAATAAATGGGCTAATTAATCG
AATAAAGAAAACTAGCAAATTAGTCACATGACCTAAATAGGAAATATAAC
CTAAATACTAAATAGTAAAAATGCATTAATGAAAATATTAAATATTTCAA
CACACCCCAAGATATGTATCCAACATACCTAAATATGTGTCCTAATATTT
TGTAATTAATTAATTAATTATTTGGGCACTTCTTCGATAAGCAAGGACAA
GTGTGGGACACAATGCGTGGTACTTCTAGTAATATTTAGGAAATAAGAGG
GTTGAATTTTAAAACATATTATACATCCATAGTGGTGAGCTAAATTAGGT
AGAATATCGTCCTAACTCCTAATAAATAGACTAATTAATCAAATAAAGGA
AGCTATCAAATTGGTCACATGACCTAAATAGGAAATATAACCTAAATATT
AAATAATAAAAATGCATTAATGAAATATTAAATATTTCACACTTCCCTAA
AGTTGGGGTAAAGATGTTATGGAGTTCCAGCTTGACAAGAGTAAAAATGG
AAGAATTGTTGTTGCCGGAAACAACGGCAGAGACAGGACGAAGTTAAGTG

TCTGATACCAACAATAAAAATTGGAACGCCGGATGCCCAACGGTGACAAT
TGGATTGCAGGATAACTAGCGGTGAACGTTGGGCGACCTACCTCGACAAT
TGGAACGCCGGGTGACCAACGGCCACAGCTGAAACACTAGGCGACACAAG
CTACAAGTGAAACGCCGGGCAACCAATGGCACAACTGGAATGCTGGACGA
CCAACGGCGACAACTAGAACGCTGAGCAACAATACGATGACAACTGCGAC
TAACGGTGACAACTGGAAGACCGAGGGTGCAGCAACGGCGGGACATATGG
TGTTGCAACGGTGGTGGTTGGAGGGTGGTTCGACGCTGCCAGAAGGCAGT
GGCCGGAAGGTGGTGAGGCGTGGGGGCTAGGGTTTCCTCCTCTCCCGCGC
CTCCCATTATTTTTCTTTTCAGGCTTTGATACCAAAAAAGATTTTGCAGA
GGGAGAGAATATTTTATTATTATGTAATAATTCTATACAATAAGGCTTTG
TACTCCTTATTATATAGCCATAACGGTGAGCTAAATTAGGAAGAATATTG
CCGTAATAAATGGTTAATTAATGGAATAAAAGAAGCTACCAAATTGGTCA
CATGACCTAAATAAGAAATATAACCTAAATATTAAATAATAAATAATGCA
TTAATGAAAATATTAAATATTTTAACATAAGTAAAAAAGTATTTAAAAAA
GCGTTATCAAATGACGTCTTAGTTTGTTGCTCTAATCAAGTTCAAGAGGC
TAATCAAGTTGCAGTAATGTAATTACCGCTACTGAAGTTCAAACTGAAGT
TTCCTATGCCACTTGGTAAAAATAAATGAAGTTTCCCATGTCTCAGGGTG
GTTAAGGAGACGACACTCTTCATTTACATTTCTGCAGGGATTGATCTGTA
ATGAATTTTGTTTTCTTTGTTCATTATCTTGAGCAAAGATTGAGGATTTT
ACCTGAATGCCTGATGTATCTTATGCTTTTTTTGACTGGAAATTCATTAG
TTCCTTTGAACGACTTCCCCCCTAATGAAGTGCATAGACATGTGGTAATA
TATATTGTGTTCTTGTGTAGGTATCACTAATGAAAAGACTACGGCATCCA
AATGTTCTGCTTTTTATGGGGGCAGTAACCTCACCGCAGCGTCTCTGTAT
TGTGACAGAGTTCCTTCCACGGTTCGTTCTCATTTCCCTTCTGGAAATTC
TATCTTTTCAAACAATTTTTATGTTCTGGAAATTCTATCTTTTCAAGCAA
TTTTTACGTTCCTCTATATGAATGTTGAAGATTTTGCCACATTCTTTCCA
TGGTGGATTTTCAACTCTTCAGGAATCCTACTGAAAGCAAGCTGGTCCAT
ATTTTGACCATTCCTTATGTGTTTTGTCTTATTTTTGCTCAATGCAGTGG
TAGTTTGTTCCGATTACTACAGAGGAATATATCCAAAATAGATTGGAGAC
GGCGTGTACAAATGGCTTTGGATATAGTGAGTATGATGAGTTCATTTTTA
ACATTAATTTGATAATTTCATGGCCAAATTACCATGTAAGCCCTTATACT
TTTGAACTTGTTTCAATTAAGCTCCTACACTTTAAAACGTCCCAACAAAT
AGGCCACATTTAAAAAAGTAGAGGGGCTTCATTAAGATGTTTTAACTATA
GGAGCTTAGTTATAGTAAGTTCAAAATACAAGCGCTCGTATAGTAGTTTT
GTCTTTCATTTTTCCCATGTTACTCTTTTTGTGGAAACAAAGCATTTTTT
TTTCGGATATTCATTTTTTGGACGTTAGTTACTCTTTTATGTAAACTATC
TCGTTGTTCCTCATGATGCATCATCCTTTTGGTGGCTTTGAAACAGGCTC
AAGGCATGAATTATCTTCACCATTACAACCCTCCAATTATTCATCGTGAT
TTAAAGTCCTCAAATCTTCTTGTTGACAAGAATTGGACTGTGAAGGTCTC
TGTCTTTTCATGATCACCATTGTTCCCCCTCCCCCACCTGTTTGCTTTTG
TTATAAAAAATGAGGATGCTTATTTTCTCGTTATTTATAATTTGACAGGT
CGCTGACTTTGGCCTTTCACGTCTAAAACATGAGACATTCCTGACAACAA
AAACGGGAAAAGGAACGGTACTCATTCAACACTTATTACCTTTTTATTTC
TTTCTAACAATCCATTTTAATTTGTTTAAATATTTTCTTTTTTGTGTGAT

GAATGCAACTTCCTAGATGAATCACAAATTTATTTATTTATTTCTTCTTG
TTTTTATTGTAGCCTCAATGGATGGCTCCAGAGGTTCTTCGCAACGAACC
CTCAGATGAGAAGTATGTCTCTTAGTCAGTGTCGGCTCTCTTTTGGATTC
CAGTGTCATGCACACTGCACAGATCTTAAAGTTTTTGACATTCCGTTTTT
GAAAATGTTTAGGTCGGATATATACAGCTACGGAGTGATATTGTGGGAAA
TTGCAACTGAAAAGATTCCTTGGGAAAATCTCAACTCAATGCAGGTAACG
TCCACTTTTATAAATTAATTGACATTTTTATGCACATGCACATGTAAATC
CTCAAGGTTTCATTCGAATTGAGGGAAATAAGGAGAAGAGGAGAAAAAGA
TATGCTTTCCCCCCACAACTGCCTCCACCATTGTAACCTGGAATTGCCAC
CACCATTGATTGAAACGTCTCTTGCCAAGTGTTATCATCTCAGAATCGAA
GCAAAAAAATCGAACTGATCCTTTGTTTCAAATTGGCAATTGAAATAAAA
TCTTTTCAAAAATGTCTTAGAAATGAAATCACCGCGAGACCAATTCATAT
GAAACTAAATTAGAGAGGTCGAAGAGCAAGAAACCAGTTAAAACATTAAT
TTTTCCGCTTCGTTAGTGTTTATTATTGTACAGGTCATGTTGACCTCCTT
TTTTGGTAGTTTTTCTATTTTGTTTATTGGCCTATTAATTAGGACAAAAT
TCCTCCTTATCTAGTCTATCATGTCGGGAGAGTGTAGAGCTCTATTGTAA
TTGTTAAAGGGTATTTGTCTTTTCTATTACTTAGGGTTTCATGTATGGTT
TCCTAATTCTTATTATAAATAGTGGGAGAGGCTAACACAATAGTTAAGCT
TTCTCGTTTTTTTCACATCATGTTCTATTACTTTAACATGGTATTCAGAG
CAGGTTATGGGAGATTGATTCCATAGATCTGTTTAAACCAATTTTTCTTC
TTCTTTTCTTGCTACTGTTCATACGAAGAAAAAAAAAAAAAGAAAAAGAAA
AAGAAAGAAAAGCTGAAACCCTAGCAGCCCTCGCCCTTTCCGCCACTGCC
GCCGCCACCACTCAACACACTCAGATCCGCGCCCTGCTGCCGTCGCCCAG
GCTATTCCGAGCTTCGCCGACTGTCGCCTAGGCCATTCCGAGCCGCCGTC
ATCATTGCGAACCGTTATCGACCCCAAAATCGCACTGTACCATCATTGCG
AACTAGTTCTCCTACCCGCTGTCCTCCCCTGACGTCGTTGACCTTCGCCG
GACCTTTGCCGGACCTTCGCCGGCCTTTTGCGCTGACTTGAGACTTTTTG
TTTGCATTCTGATAGAACCAACTGCAAACCCTTGTTAAACGATCTCAGCA
GCGCATTTGCATTCTGGTTATGCTTCTGGTTATTGTTCAGTAGACTCAAG
ACTTCGGGTTATGCTTCTGGTTATTGTTCGAAAGACAGCCCTACAGATGT
TCATGAGTATTGTTCTAATTCTCATGTTCTTCGTTTAAGCCCTATAGTTT
ATATTGTTGGTTTGTTTCCCTTGTGCAAGTTGTTTTTTAGTTGTATTGGA
GGGTACTAATGTCAATAGCTACACCTTAACTATTAACAACCCGTAAGTTG
AATGGAACCAATTATTTAAATTGGTCTAAGACTGTGAAAATAGCCCTCAC
CAGATAAGGGAAACATCGTCACCTCACGGAATCCGCCTCGGTAACAGATG
ATAAAAAGTATGACGTCTGGATTCAGGCTGATGCGCAAATTGTAGTTTCG
TTGTGGAATTCGATGGAGCCTCAAGTGGCTGATATCTGTACTCATCTCGA
TACGTGCAAAGATATATGGGAGTATCTTCGTGTATTATATTCCAGTAATT
TCACCAGGATGTATGATTTATCGGTCGAGTACTTCCAACTTCAGAAAGGT
ACACAATCCGTTACAGATTATTTTGCCACATTCAACAGCCTCTATGAGGT
GATGAATACTATCCCACCGATGACTATTGATCTGAAAGAAATGCAAAAGC
AACGTGAGCAGATGGCTGTCATGAAGTTCTTGGCTGGATTACGCCCAGAA
TTTGAACCAGTCCGATCTCATATCCTTTCCAGTGCTACTCTTCCCACTAT
CACAGAAATGTATTCTCGCGTCTCGCGTAGCACTACCAAGGATAATGTGT

TTGGCTTGAGTTCGTCTGCATCGTCTGAGCATTCGACGCTTCTTACTCAA
ATTCCGACATGAGATCGTGGTGTTCCACTCAATGGCGGTGGTGTTTTTTC
TCGTGGTGGTGGCGGTTTCTCTTGTGGTGATGGCCCTCGGGGTGGACGTA
GTGGTCGTGGCTTACATCGATAATGCTCTTTTTGCGGAAAGGATAATCAT
ACTTGAGAAATATGTTGGGATTTATTTGGTCGTCTTCCCAAATTCGCTAA
TGCAGTGACATCTGATTCAAATACTCCTATTATTCCAGAATCTCAGGCGG
CCAAGAACATCTCTGATGACGAATATGCTCAATTCCTTCAGTATAAGGCA
TCCCAACAGGCATCTCTTCCCATTGCTTCTTTAGCTCAACGAGGTACTTC
TACTGCCTGTTTTTCAATTCGCCCTTCCCCTCCCACACCCTGGGTCATAG
ATTCTGCAGCCACTGATCACATGTCAGGTACTTCTTCTCTCTTTACACAT
ATTCAGTCCTCTTCTTCATTGTCTCATGTTACAGTTGCTGATGGTTCCAC
TGTCCCTATCCGTGGCATTGGCACAGTTGCACCCTTACCCTCCTTATCTC
TCTCTTATGTCTTGTATATTCCTTCGTTTTCTTTTAATTTGATGTCAGTC
AGTAAGCTCACCAAACACCTTAATTGTTCTGTTTCATTTTTTGCTGATAA
TGTCATTCTCTAGGATTTGAGGACGCGGAAGATGATTGACACCGGGCGTG
AGGAAAATGGTCTATATTACTTTGATGACCATGACGACAGTCCTGCTATC
TTACCTACTGCCTGTCCTGCTGTTGCCTCCGCTCACCAGATCCATTGTCG
CCTTGGTCATCCATGCCTTGGCAACCTCAAGTTACTTGTTCCTGATTTGA
GTCATTTGACTTCATTAGAGTGTGAGTCTTGTCAACTGGGTAAGTATCAT
CGTATTTTATTTGCTTCCCCAGCTCATGTTCGAGCATCGAGTCCGTTTAG
TTTAGTTCATTCAGATGTTTGGAGTCCTTCTCGTGTTACTTCTACATTAG
GTTTTTATTATTTTGTGACATTTGTGGATAATTTTTCCAGAGTTACTTGG
CTTTATTTAATGAAAGATCGTTCAAAATTATTCTCTATTTTTCGTGCGTT
TTGTGCTGAAATTAAAACTCAATTTAATGTACCAGTTCGTATTCTTCGAA
ATGATAATGCTCGAGAATACTTTTCTCAGCCATTTACTTCCTTTATGTCA
CACTCTGGTATCTTGCATCAGTCTTCTTGTCCTCACACACCGCAGCAAAA
TGGAGTTGCTGAGCGTAAAAATCGACATCTTCTAGAAGTGGCTCGTACTC
TTCTTTTTGAGATGAACGTTCCCAAACATTTTTGGGGCGATGCGATTCTT
ACCGCTTGCTTCTTAATTAACAGGATGCCGTCCTCTACCCTTAACAACAA
GGTTCCCCATTCCATTCTTTTTCCTCGTTCTCCCTTGTTTAGTTTACCTC
CTCGAATATTTGAGTGTGTTTGTTTTGTTCATCAATCGACTCCTGGCAGG
GACAAGTTAGATCCCCGTGTTTTAAAATGTGTCTTCCTCGGCTATTCTCG
CACATAAAAGGGTTATCGTTGTTATAGTCCATCATTACGTCACTCGTTTG
TGTGTGCGGATGTTACATTCTTTGAGTCTACCCCTTACTTTTCTGCTCCT
TCCTCTCGAGACGATCTGGATGTTACGCTTCCCTTGCCAAGTATACCAAA
CCCTACACTATCTTCCTCTACTTTGGATCCTATCCCGCCTAGTCGTTCTT
CAGCTACTTTGCAGGTCTACCCTCGTCGCCAAAAGGCCCCCTCCGCTGTC
GCGCCCATCGACTCTGTGTCCTCAGATTCTTCTCCAGCCGACCTATCCGT
CTCTGACCTGGANNNNNNNNNNNNNNNNNNNNNNNNNNNNNNNNNNNNNNNNNNNNNNNN
NNNNNNNNNNNNNNNNNNNNNNNNNNNNNNNNNNNNNNNNNNNNNNNNNNNNNNNNNNNN
NNNNNNNNNNNNNNNNNNNNNNNNNNNNNNNNNNNNNNNNNNNNNNNNNNNNNNNNNNNN
NNNNNNNNNNNNNNNNNNNNNNNNNNNNNNNNNNNNNNNNNNNNNNNNNNNNNNNNNNNN
NNNNNNNNNNNNNNNNNNNNNNNNNNNNNNNNNNNNNNNNNNNNNNNNNNNNNNNNNNNN
NNNNNNNNNNNNNNNNNNNNNNNNNNNNNNNNNNNNNNNNNNNNNNNNNNNNNNNNNNNN

NNNNNNNNNNNNNNNNNNNNNNNNNNNNNNNNNNNNNNNNNNNNNNNNNN
NNNNNNNNNNNNNNNNNNNNNNNNNNNNNNNNNNNNNNNNNNNNNNNNNN
NNNNNNNNNNNNNNNNNNNNNNNNNNNNNNNNNNNNNNNNNNNNNNNNNN
NNNNNNNNNNNNNNNNNNNNNNNNNNNNNNNNNNNNNNNNNNNNNNNNNN
NNNNNNNNNNNNNNNNNNNNNNNNNNNNNNNNNNNNNNNNNNNNNNNNNN
NNNNNNNNNNNNNNNNNNNNNNNNNNNNNNNNNNNNNNNNNNNNNNNNNN
NNNNNNNNNNNNNNNNNNNNNNNNNNNNNNNNNNNNNNNNNNNNNNNNNN
NNNNNNNNNNNNNNNNNNNNNNNNNNNNNNNNNNNNNNNNNNNNNNNNNN
NNNNNNNNNNNNNNNNNNNNNNNNNNNNNNNNNNNNNNNNNNNNNNNNNN
NNNNNNNNNNNNNNNNNNNNNNNNNNNNNNNNNNNNNNNNNNNNNNNNNN
NNNNNNNNNNNNNNNNNNNNNNNNNNNNNNNNNNNNNNNNNNNNNNNNNN
NNNNNNNNNNNNNNNNNNNNNNNNNNNNNNNNNNNNNNNNNNNNNNNNNN
NNNNNNNNNNNNNNNNNNNNNNNNNNNNNNNNNNNNNNNNNNNNNNNNNN
NNNNNNNNNNNNNNNNNNNNNNNNNNNNNNNNNNNNNNNNNNNNNNNNNN
NNNNNNNNNNNNNNNNNNNNNNNNNNNNNNNNNNNNNNNNNNNNNNNNNN
NNNNNNNNNNNNNNNNNNNNNNNNNNNNNNNNNNNNNNNNNNNNNNNNNN
NNNNNNNNNNNNNNNNNNNNNNNNNNNNNNNNNNNNNNNNNNNNNNNNNN
NNNNNNNNNNNNNNNNNNNNNNNNNNNNNNNNNNNNNNNNNNNNNNNNNN
NNNNNNNNNNNNNNNNNNNNNNNNNNNNNNNNNNNNNNNNNNNNNNNNNN
NNNNNNNNNNNNNNNNNNNNNNNNNNNNNNNNNNNNNNNNNNNNNNNNNN
NNNNNNNNNNNNNNNNNNNNNNNNNNNNNNNNNNNNNNNNNNNNNNNNNN
NNNNNNNNNNNNNNNNNNNNNNNNNNNNNNNNNNNNNNNNNNNNNNNNNN
NNNNNNNNNNNNNNNNNNNNNNNNNNNNNNNNNNNNNNNNNNNNNNNNNN
NNNNNNNNNNNNNNNNNNNNNNNNNNNNNNNNNNNNNNNNNNNNNNNNNN
NNNNNNNNNNNNNNNNNNNNNNNNNNNNNNNNNNNNNNNNNNNNNNNNNN
NNNNNNNNNNNNNNNNNNNNNNNNNNNNNNNNNNNNNNNNNNNNNNNNNN
NNNNNNNNNNNNNNNNNNNNNNNNNNNNNNNNNNNNNNNNNNNNNNNNNN
NNNNNNNNNNNNNNNNNNNNNNNNNNNNNNNNNNNNNNNNNNNNNNNNNN
NNNNNNNNNNNNNNNNNNNNNNNNNNNNNNNNNNNNNNNNNNNNNNNNNN
NNNNNNNNNNNNNNNNNNNNNNNNNNNNNNNNNNNNNNNNNNNNNNNNNN
NNNNNNNNNNNNNNNNNNNNNNNNNNNNNNNNNNNNNNNNNNNNNNNNNN
NNNNNNNNNNNNNNNNNNNNNNNNNNNNNNNNNNNNNNNNNNNNNNNNNN
NNNNNNNNNNNNNNNNNNNNNNNNNNNNNNNNNNNNNNNNNNNNNNNNNN
NNNNNNNNNNNNNNNNNNNNNNNNNNNNNNNNNNNNNNNNNNNNNNNNNN
NNNNNNNNNNNNNNNNNNNNNNNNNNNNNNNNNNNNNNNNNNNNNNNNNN
NNNNNNNNNNNNNNNNNNNNNNNNNNNNNNNNNNNNNNNNNNNNNNNNNN
NNNNNNNNNNNNNNNNNNNNNNNNNNNNNNNNNNNNNNNNNNNNNNNNNN
NNNNNNNNNNNNNNNNNNNNNNNNNNNNNNNNNNNNNNNNNNNNNNNNNN
NNNNNNNNNNNNNNNNNNNNNNNNNNNNNNNNNNNNNNNNNNNNNNNNNN
NNNNNNNNNNNNNNNNNNNNNNNNNNNNNNNNNNNNNNNNNNNNNNNNNN
NNNNNNNNNNNNNNNNNNNNNNNNNNNNNNNNNNNNNNNNNNNNNNNNNN
NNNNNNNNNNNNNNNNNNNNNNNNNNNNNNNNNNNNNNNNNNNNNNNNNN
NNNNNNNNNNNNNNNNNNNNNNNNNNNNNNNNNNNNNNNNNNNNNNNNNN
NNNNNNNNNNNNNNNNNNNNNNNNNNNNNNNNNNNNNNNNNNNNNNNNNN

NNNNNNNNNNNNNNNNNNNNNNNNNNNNNNNNNNNNNNNNNNNNNNNNNN
NNNNNNNNNNNNNNNNNNNNNNNNNNNNNNNNNNNNNNNNNNNNNNNNNN
NNNNNNNNNNNNNNNNNNNNNNNNNNNNNNNNNNNNNNNNNNNNNNNNNN
NNNNNNNNNNNNNNNNNNNNNNNNNNNNNNNNNNNNNNNNNNNNNNNNNN
NNNNNNNNNNNNNNNNNNNNNNNNNNNNNNNNNNNNNNNNNNNNNNNNNN
NNNNNNNNNNNNNNNNNNNNNNNNNNNNNNNNNNNNNNNNNNNNNNNNNN
NNNNNNNNNNNNNNNNNNNNNNNNNNNNNNNNNNNNNNNNNNNNNNNNNN
NNNNNNNNNNNNNNNNNNNNNNNNNNNNNNNNNNNNNNNNNNNNNNNNNN
NNNNNNNNNNNNNNNNNNNNNNNNNNNNNNNNNNNNNNNNNNNNNNNNNN
NNNNNNNNNNNNNNNNNNNNNNNNNNNNNNNNNNNNNNNNNNNNNNNNNN
NNNNNNNNNNNNNNNNNNNNNNNNNNNNNNNNNNNNNNNNNNNNNNNNNN
NNNNNNNNNNNNNNNNNNNNNNNNNNNNNNNNNNNNNNNNNNNNNNNNNN
NNNNNNNNNNNNNNNNNNNNNNNNNNNNNNNNNNNNNNNNNNNNNNNNNN
NNNNNNNNNNNNNNNNNNNNNNNNNNNNNNNNNNNNNNNNNNNNNNNNNN
NNNNNNNNNNNNNNNNNNNNNNNNNNNNNNNNNNNNNNNNNNNNNNNNNN
NNNNNNNNNNNNNNNNNNNNNNNNNNNNNNNNNNNNNNNNNNNNNNNNNN
NNNNNNNNNNNNNNNNNNNNNNNNNNNNNNNNNNNNNNNNNNNNNNNNNN
NNNNNNNNNNNNNNNNNNNNNNNNNNNNNNNNNNNNNNNNNNNNNNNNNN
NNNNNNNNNNNNNNNNNNNNNNNNNNNNNNNNNNNNNNNNNNNNNNNNNN
NNNNNNNNNNNNNNNNNNNNNNNNNNNNNNNNNNNNNNNNNNNNNNNNNN
NNNNNNNNNNNNNNNNNNNNNAATGGGAATAATGAGAAACAATTTTTTTT
TGTTTCTCATTTCTTAAGAAAAAAGTGGGCACAATTCTCAAATGGGAATG
AAATGGAAACGGAAACAAAAATTACCAAACGCTATTCCAAACAGAATTCC
CAAAAATCTGGGAATGAGATGGGAATTTCTGGAATGGAAACGGTTACCAA
ACGGGGCCTTAGTTGCTAAAGGCTATACTTAGGTATACGGCCTTGATTAC
TCTGAGACCTTATTTCTAGTTGCCAAGTTCGCTTCTGTTCCCTTGTTTAT
CTCTTTGGCTGCCACTTTTCGTTGGCCATTACATCAGTTCGATATTAAGA
ATGCTTTCTTAAATGACGATCTAACTGAAGAAGTATTTATGGAGCAACCT
CCTGGGTTTGTTGCTATGGGGGAGTCTGGATTGGTATGTCGCTTGAAGAA
ATCTATTTATGACCTCAAGCAGTCTCGTCGAGTATGGTTTGGAAGATTTA
GTGAAGTGGTCACTGACTTTGGTTTTCGACGATGTGGAGTCGATCATTCC
ATCTTCTACAGGCATAATAAAGTCGGGAGAATATTGTTGATTGTGTTTGT
GGATGACATCGTGATTACCGAGGATGACCACGGTGGTATTCAGGAACTCA
AAAAGTTTTTGAATAGTAAATTTCAAATAAAAGATTTGGGTCACTTAAAG
TGCTTCTTGGGAATTGAAGTCGCTCAATCAAGTCAAGGCATTTACTTGTC
TCAGAGAAAATATGTCCTTGACCTATTGAGCGAAACATGAATGCTAGGAG
ATAAAATGATTGACTTGCTTATGGATCTAAACACCAAACTGTTAGTAGAT
CAATGTGTACCATTGACAGATTCAGCACAATATAGAAGGTTAGTTGGAAA
GTTGAACTACCTGACAGTTATCAGACCAGATATCTCCTTTGCTGTTAGTG
TTATCAGTCAGTTCTTAGATTGTCCAATGACTTGTCACTGGGATGCAGTG
ATCCGTATTCTCCGATATTTGAAAGGTGCTCTTGGTTGTGATCTGCTATA
TCAAAACTTCGGTCATACTCAAATAAAGGACTACACAAATGCAGATTGGG
CTGGATCGCCCTCGGATCGGAAGTCTACTACTGGATATTGTGTGTTCGTT
GGTGGTAACCTTGTCTCTTGGAAAAGTAAAAAGCAAACTGTGGTGGCACG

GTCAAGTGCAGAATCGGAATACAGGGCAATGACCAATACCACTTGTGAGT
TAACTTGGTTGAAACACTTGTTGGAAGAACTTGGGTTTGAACACTCTTTA
CCGATGGATCTGGTATGTGATAATCAAGCAGCACTTCACATTACTTCCAA
CTCAGTCTTCCATGTGAGAAAAAAATATATTGAAGTTGATTGTCACTTCA
TTCGAGAAAAATTGTTACAGAAGCATATTGAAACTAAACATGTAAATTCA
GTTGATCAGTTAGCTGATATATTTACTAAACCATTGGGGGCCTCAAGGAT
AAAATACATTTGTAACAAGTTAGGAGCATACAATATATATGCTCCAGCTT
GAGGAGGACTGTTAAAGGGTATCTATGCCTTTTCTATTACTTAGGGTTTC
ATGTATGGTTTCCTACTTATTATAAATAGTGGGAGAGGATAACACAATAG
TTAAGTCTTTTCGTTCTTTTCATATCATGTTCTATTACTTTAACAGTAAT
ATACCAATGCGACATTGTTATTTAAGGCGCGAGGCGCACTGAGTCACAAT
GACCCTCGAGGTAGCGAGGCGCGGGCTTCTTTGAAGTGCGGCACACATTT
TAAATATGTGCTTTTCTATTATCATGGACGTGGAAAATATTCAAATTTCA
CCAAATTAAAATATATATGCTTGTTTATAAAATTTTAAACCCTAATATAA
TTAAATTTTGTTAAATAAAACTAAACAATAACAGAAAAAGAAACAAAAAT
ACCCAGAAAATAGAGAACCTAAGTTGCTGAACAAGGGAGTTTTTAAATTG
TAGAAGAAGAAGATTGAATGAGAAAAGCTAATTAAAATCTAAAATCCTAA
TAAAATTAAATTTAGTTAAATAAATAAACATTTAGTTAAACAATTTTTTT
TTGGTTAAAGTTAAACAAATAAATAAATAAAACAATAACAGAAAAGCAAA
AGAAAAAAGAAGAAGATACTTACAGGGAGAAAGAAGTCGAAGTCGAAGTC
GACGAACAATGAACTGGAGGAGACGTAGTTGGGCGATGGATTTGAAGGAG
AAGTCGAAGTCGAAGGAGAAGGAGATTCGACGGAGATAACTAACTTGCTC
GAAGAAACTGGGCGAACAGAAGCAGCACCCTTGATTCGCTGAAGGAGAGA
AAAAGATTCGAAGAAGGATAATGCTATCGAAGGCTTGTCCAAAGCTGTCT
TCAAAGAGGCGTGCCTTAGTAGCGCCTCGCGCCTCTTTGAAGGCACCTCA
CCTTACCATCGAGAGGTGATTTCTCAGCGACTCGCCTCGAGGCGCGCGCT
TTTCACAACAATGCAATGCGATAAAATATCTTCTCTTCCTTACTATATCC
GTTTTGGTATTCAAAGCATAAAAGGAAAAATAGAAAAAGATAAAAGGGAG
AAACCCTAGCCTAGTTGCTGCAAACCCTAGCCTAGCCACTACAGATTTGC
TCTGCCACTACCGCCTCCAATCCTAGCCACCACTATCGCGAACTTTGCGT
AGAACCACCATACCCACCACTGCGACTGCGCATAGAATGGCCACACCACC
ATCGTGAACTTCATGTAGACCAGCCATACACTGCAGCGAACTTCGTGCAG
ACCGGCCACACCACTGCTGCTAAATCTTCTGCGCACACCAACCACTAGCA
TCACCGCATCATTCGAGCTGAACAACCTCCTGCATAGCCACACAAATCTG
CCTCTCTTCTGCGTGGATCACTTACCTCTTTTCTACACGAATCACTACTC
TTCCTCTTGCGCAAATCGTTGGCCCTAGCCACCAACATGCTTGCCTTCTC
GCAGTTCTTCTAGCGGCATTGCTTAGTCGCAGCTGAAACCTTCATTTGTA
CTAGACAACGGTATCCCTATCTTATCTCTTGTCGAGGTAACCATGTGTCT
GGTTACCTCTATTATCTTGTTCTTGGCCTCTTGGTAGTTCTTTGGTTGTC
TCCGTCTGGGGTTTTTTTTCCCCAACGTTCTGTGTTTTTGAGACTTATGG
CTTCTACAAAAGAAGCATATTCAATCGGTTACTTGCCCTGTTCTCTCTCG
TGAACCTAATAAGTCTGCTCATCGTGTTACTAGTGTACTGTTGAACAGGA
CAACCTTTCATGCCTGGTCTCGCTCGTTTCGTATCTACGTTGGTGGGAAA
TGCAAGACCAGATGGATCTTGGGAATTGAGCCAAAACTCGGAATAATTGA

TCCCAAATTCGCCCAATGGGATGCAGATAACTGTGTAATCTTGGGCTGAA
TGTTTAATTCTGTGAAGGAACATATCTACAATTTCATATACTATGTGACG
GTTTATGACTTCTGGTTTGCTTTGAAGAAGATGTATGCCCATGCTCACAA
CAAATCTTGATCTTCGAGCTGTATGATGAGATCTCACTTGCCTCTCAAGC
ATCTCTGAGTCTTTCAGTTGCAGATTCCTTGGGGTACCTTAAGACTCGAT
GGGAGGAGCTGGCTCAATATGAATCTCCGAGTGATTTCCCTAATAATTAA
GACCAAGCGCCAGGATCGTCGTTAAACTTCTCACTTCTTAATGGGTTTAA
ACCCTGAGTTTGATAATCTCCGGACTCAGATTTTTAACACATCTTTGATG
CCTAATTTGTACGTCGCCTTTGCGATGGTCAATGGGGATGCACCTCTTGC
CACTTGAGCCCCCTCCCATACAGTCGCGGATCGTATGGCCTTTGCCGCTC
CTTCGGGCATTAGTTCAATAGGACCAAAAACTGTTACTTGCCAGCATTGT
TGTAGGTTAGGTCACCTTTACTGGTGCTTTGAGCTCCACCCCGAATTACG
GCCACAACACTCTCGGAATAGTCGAGGTAAAAGTGCTCCTTAAATTGGCG
CAATAGCTGACATGGCGACTGTCCAACCAGAATCCACCATTTTCACCCCG
ATTACACTCGGCTTTAGGCCTAGATCGCTTAGCTCCAGTTATACTTAGGC
TGGGCGTCAACTTTCGATCCCTCGGCTGCCCTTGCCATAGGTACCCTACC
CCTCTTCATGGTAAGTTTACCAAACTAATTTGGATTCTAGATTTTGGGGC
TAACAATCACATGACCGGTGAGCTTTCTACATTTACCTCTCATGTCACTT
TTGTTAATCAATCTGTTTATATTGTCGATGGGATTTCTATTCCAATCCAT
TGTCATGGAGATGCCTACTTATCTTCATATATTACCTTTCATTTTGTCCA
ATATGTTCCTAACTTTTCCTATAATTTCGTCTGTTAGTCGTCTTGCAAAG
GGTGTTAACTTTGCTGTCATTTTCTTACCTGTCCATTGTTTCTTGCAAGA
CCTGGCATCGAACTCTACTATTTCGACAATCCACCTACCCCCACTGTGCC
TTCATCTAGTATGCAAGTATCAGTTTTATCTACTTTTGACCCATCCATGT
GTTCTTTTCAAAATTTAAACTTATGGCAATGCTTGTTTAGGACATGCGAA
TTTTCAATAATTATGTTGCTTATTTCCAAGTTTTAATAAAGCTTGCAAGG
ATGAAGGATTTTAAGTTTCATTGTGTGACTTGTGAATTTTTTAAATATAC
TCTTACTTCCTGTATTCTTCGCATGCAAAGAGCACCATAGGCCTTTGATC
TAATTTATTCGGATGTATGGGAGCCTTCCCCTGTCACCGGCCATTTCATA
GCATCGTTACTAGGTCAACTTCATAGACGATTATAGTCGGTGCACGTGGG
TTCACTTAGTGAAGAAAAAAATCTAAGGTCGTTTCTCACTTTATCCGTTT
TCTTCAAATGATTAAAACCTAATATTAACCTATTGTTCACTACGTTTGGT
CTGCTAATGGTAGGGAATTATATCTAATGAATTTAGGGCAGAATTAAGCA
AACAAGGCATTCTTCAACAACTTAGTTGTCCTTACACTCCTGAACAAAAT
GGTGTGGCAGACCGTAAGAACCTTTACTTTATGGCTTTTGTTCAATGCCT
CCTACATGGTAGGCATGTCCCCAAATCTTTTTCGCACATGACAGTGCTTA
CAGTCGCCTACCTTACTAATCGTACCTCTAGTCGGGTCCTTAACAACAAG
GCACCCCTACACATACTCTATACCATCTCTTTCCCATCCTACCTCGGGCT
TTTGGGTGCACCTGCTTTTTTCAAAATCGTAGCCCCTCTCGAACTAAGCT
AGATGATAAGGCTATCCGATGTGTCTTTCTCGGGTATTCCTCCATGTGTA
AAGGATATAGGTGCTATGATCATATTACCCGTTACCTTCACCACTCTGTG
GATGTCACATTCCTTAAGAGTGTCCTCTATTCCAGTCTTTGCCTTCCTAG
CTATACCCATGTATTCCTAAGAGGATGTTTCTGTGCTTCCCCCTGTTCCT
ATTCTTCACACATCTTCCCTCTCCACGAGATCATCCTCTAGTGAGTTTAT

GCCACCTAACTCTTCCTCTGTCCCTCTATAGATATAATCCTATCGTCCAC
GTGCTCTTAATCCTCTGCCGACGTCTTTTCTGGATTTAGGTTACCTTCAT
CTCCCATACCCTATGATCCTGTACCTCCTTGTTTCCCCATCAGTGCCTAT
CGCCTTCCTTCCCGTTACCTTCTTTCCACTTGTACTAATAATTCGATTGC
CAAGTATATGTCTTTTTTGGGTCTCTCAGATTAATACAAATCTTTCCTTG
GGTAGGTTGATTCAGTCTCACTTCCTTGATTAGTTCATGACGCCCTCCAG
GATCCAAATTGGGTAGATGCTGTGAAAAGAAATGGGTGTCCTTCAATAGA
ATCATGCTTGGGAACTTGTTGATTTGTCACCTGGTGAGTGGGCGTTGGCT
ATAAGTGGGTGTTCAGTGTGAAGTATCTAGCGGGTGGTTCCATTGGTCGG
TATAAGGCACACCTCGTCACCAAAGGATTCGTGTAGATATCGAGTAAAGA
CTTTGGTGCTACATTTGCCCCAATTAAGTTGACGACAATTCACCTTCTCG
TCTCCTTAGTTACTTTTCTCTTGGCATTTTCATTAGTTGGATATGAAGAA
TGCGTTCTTTAACGGCGATCTGTCTGAAACCATCTACATGGATCCTTTAC
TAGGTTTTCGGGATTAGGGAGAGTGTTCGAGAAAAGCTCGTCGCCTTCGA
AAATCACTTTCTGGACTTAAGCAGTCCCCACGAGAGTGGTTCAGTGATGT
CATCCTTACTATGGGATTTACCTGGTGCCATTTTGATCACACATGCTTCA
TTCGTCATCGTCTTAGGGTCAGTGCATTATCATTTCTGTCTATGTGGATG
GTATTATCATTACAGGAGATGACAAACAAGACATTGTCCGTGTAAAAGTG
ACCTTGGAAAGTCCTTTGACATTAAGGATTTGGGTCTTCTTTGATACTTT
CTTTGTATTGAGGTTGCTCGATAATGCTAGGGTATTATCCCTATGCCAAC
GAAAATATGCTCTTGACCTCCTTCAGGATATGGGCATGCTTGGATGCAAA
TTTGCCTCTACTCCTATGGACCCAAACCTTAAGCTGTCAACAAAATCGGG
AGAACTATTACTGGAGCCATCTGTATATCAGCGACTTGTGGGCCTTCTCA
TCTACCTAACACAAAGCCGGATCTTACCTATGTCCTGAGCATTGTGAGCC
AGTTTATGCGTACTCCACGTACATCTCACTTGGATACAGTTTATCACATC
CTCCGGTATCTTAAGACATGCCCTGGTCTTGGTCTTTTCTATGTGGTCGG
GTCGCAAGAAGGCTGTTCTGCTTTACATATACTGATTATGCAGGATCTAT
TGATGATAGACGATAGACTTTTGGTTAGTGCACCTTTTGAAGCTGCTATC
CTATCTCTTGGAAAAACAAAAAACAAGCTGTTGTCTCCCGAAGTTCCGTG
GAAGCTGAGTGCCGTGCTACGGCTCACGGGATGTGCGAACTTCTTTGGCT
ACGTTCTTTCTTGTTTGAATTAAGATTCCTTTGGACAAACTCGTCTACGC
TATTCTGTGATAACTAGTCTGCCCTAATTTTATCTTCTGATTCAGACATT
CAGTCCTTCATGAGAGAACCAAACATATTGAAATTGATATCCACTTCATC
TGAGTGGAAGTTTGGTTAGAGGTCAGTACTCCTACCTTTGACTCATCGTT
CGAGCAGGCAACTACATGTTCACAAAATCGATTGGACCCTCTCTTCTTCA
TTTAGTCATTGTCAAGTTACAACTCATCAATATCTTTGCCTCAATTTGAG
GGCTAGTGTTGAAATATTTATTTTCCCGCTTCATTTATGTTTAATATCTG
ACAGGTCATGTTGACCTCCTTTTTTCGCTAGTTTTCCTATTTCGTTTATT
GGCCTATTAATTAGGGAAATATTTTTCCTTATTTAGCCTATCGTTTCGCC
TGTATAATGGAGTGTAAACCTCTATTGTAATAATATCAATACAATAAAAT
ATTTTCTCTTCGAAATCCGAAAAATTGAAACCAGAACTGTAAATCGAAAT
AAGAAAAGGTTGATCTATCTCTCAAACCGTAATCTATAACCCTTAACCAA
ATTCCAAGAAATGAGAAATTTCATCTTTGGACTCATCATGAACTCAAACA
CAAATATCGCCGAAATCGTGCTTATGCTTGATTTTCAGCACAAATAGCAA

ACAAACCAGAGAAGATAGGAAGAAGCCAACAATCCATCTTGAACCAAAAT
TAGTAAAAAATGGAGAAAAAAATTGTCCCAAGTCGAAAATGATTCAAGAC
AAACTGGCTCCAAATCAATTGCAAATCATTCAACTTCAAAGAAATCAAAT
GCAATCATGAAGCAAAAAGGGGAGCTGAAATCGAAAATCGAAGAAACAAA
TCTCGGAAAAGAAAATTTGGATTGAAATTGGAAATTCATCTGATCTCAGA
ACCCTAAAGCACAAACCTTCAAATTGCGAGAAGAGGGTCCTGTAATCGAA
GTATACTCATCTTCATCATCAATGTTCCAAATTGAATTGAAATATGGTGG
CAAAGAGACGATGTGGTAGAGATGGAGGAGATCGAACGATGGGATTGTGG
GGAGGAGACCTTAGCCCGTTTGTGTTGAGGTACGATCTTGAAGGAGATTT
TTGTCGTGGGTAATTATGTATTTTTCAAATCGATTAGAAGGGTTTGTTAG
TACAAAGAGCAAAATCTAGTCAATTGAAGTCTAAATTTGTCTATTAAAAA
TTATGTAAGCAATTTGTTACTTGATGGTCATTGACCGTAAATGTTAAGGA
TTTGACTGGTAAATGCATAATGACAGGGGATTCCCTTGGTAAATGCATAA
AGACGGGATTCCCGTGGTAAATGTGTTAAATGAATGGGGGAGTAAATGTA
AAATTACAATAGGTGAGTGGCTTAAACCCATAGTGATGCTTCTTGTTTAC
TCTTAATGAGATTCCCAAACAAGAAGGAAAGAAAGTTAAAGTTCTATTCT
CTTCATTTCTCTTAATGCAATTCTGTTCTATTTTCTCCATTTCTCTTTAT
CCAAACATGGTATTGTGTGAGAACTGTTAGACGGGCTTCCCTTGTATATG
TAGGTCTGTTTGTTTAGCACACTTGCAGGTGATTAGAACCTATTTGTTTT
AGTATATTTGCAGGTAATTGGAGCTGTAGGGTTCATGAACCAGCGGCTCG
AGATCCCAAACGATGTCGATCCTCAGTGGGCTTCCTTAATTGAGAGCACT
TGGCACACGTTTGTAACTCTTTCATGCTATCGTACTGATGTGTAGAGTCT
TAGGTGGCGTTCATTTAGTGTTTCTGTTATATTCCTAGAATTTTGTTCTA
AAAATCTAGGAACATAACAAAAACAGTTGGTCTTTATTTTTTTGTTTCCA
TACTTATGTTTCTGTTCCAAGTTTTCTGGGGAAGAAAACAAAGAACTGAA
ACACTAAACAAACACCACTTAAATTTACAAGTATTTGTATAAAATCCAGC
TTCCACTTCAGCCATAATTCAGAATGAACATTTTTTTTTGGCTCAGCGAT
CCAAGGTGCAGACCGACATTCCAAGAGATACTGGAAAAGCTTAAAGATCT
GAGGAGACAATTTGCGATTAGAGTTCAGGCAGCCCGTTCTGCAGCTGGAG
AGAGCAGCAGCCGAAAGGAATTGTAG
>EUC16831-RA [gene]
ATGGAGAAATTCAACGCCGAACGGAGCTGGGTCAGGGGAAGCTGTATTGG
AAAAGGCTCCTTTGGGTCCGTCAGTCTCGCCGTCGACCTGTCGGATGGCG
AGGTTTTTGCCGTCAAGTCTGTCGATCGGAACTCGTCTTCGCCGGCGCAG
GTGGAGGCGATTGAGAACGAGATTCGAATTCTGCGGTCGATCTCGTCGCC
GTACGTGGTGGGGTATCTCGGCGACGGCTGGACCAGTGAGTTTCCCTCGG
CGTCGTACAGGAATATGTATTTGGAGTACTTGCCAGGTGGCACCGTTGCT
GACTTGGCGAAGCGGTCCGACGGTGACGTGGATGAGGAGACGGTGCGGCG
CTACACATGGTGCATTGTGTCGGCGCTCAGATACGTTCACTCAAAAGGCA
TTGTCCATTGCGACGTCAAAGGGAAGAACGTTTTGGTGGGACCCGCGAAC
GGCGCCGTCAAGCTCGCCGATTTCGGGTCGGCGACGGAATTCAATGATCG
GAAATATCCACGTGGAAGTCCGTTGTGGATGGCGCCGGAGGTGGTGCAGG
GAGGGTACCAGGGACCGGAGTCCGACGTCTGGTCCTTGGGTTGCACAGTC
ATCGAAATGATCACCGGGAAGCCGGCATGGGAAGACCGTGGCGCCGACAC

CCTGTTCGAAATCGGTTACTCCGGCAAATTGCCGGAGTTCCCAACCCATC
TACCGGAGCATGGTCGCGATTTCCTCGAGAAGTGCTTGCAGAGAGACCCC
TGTAAGAGGTGGAATTGCGATCAGCTACTCCGGCACCCATTTATTTCCTC
CTCTTCGCCGGATGATACGAATACTGATTCAACCCCTCGTTGCGTGATCG
ATTGGTTCAGTTCGGAATCCGGTGAAGACTCCGACGCCGACGCATCATGC
GAAACAGAGGATTTCAATGAAAACGCAAAACAGAGAATTTCGGAATTAGC
CACAAATTCAGGGGCAAATTGGGAATCAGATGGGTGGATGTTAGTGAGGA
ATTTGATCACGGAGAGTGAGCCAGCATCAGGTGCGTGCTGTAGTGCCGAC
GACGAAGAAGGGATAAATTCGGAATATCCCCAATTGATGGGGACAGAGGA
GGAAAGTATAGGGGTTATTTCTGTAAATTCGGATTCCACAGCAAGGATAA
AAACGGAATATTACGGCAGCACTTTACAGGCGAAACCGGAACACAAAAAT
GGCGTCGTCTTCCGCCGGAGGAGGGACAGTGTCGGAGCCAGTTGGAGGTG
TCAGGATGGGTGGTGGACAGATAGGAAATCGACGGTGGAGAGCAGGGAGA
AAGGGATTTACAGCTGTAGCGAATTAA
>EUC10368-RA [gene]
ATGAGGAAAGTTATTGTAATTGTAGTGGTGTACTTGTCCATCGTCATCCG
TAATACCAATCCAGTCAGTCCACCATGCATATACAAATCAGTGGTCCGGT
CCACAATCTTATTATTGCGGCCCGTCCTTGTTTTTTTTTTTTTTTTNTCA
TATCAATCTTATATATTTATGATTAAATTTAAAATTTAATTCATCGTCTT
CGACGAAGTTGAATAAGAAAGTGGAATATCTCCTCTTACTAAGTCCACAC
TCTTAGGTGGGCCGACCTTGTTAGGTAGATCATGATACACATTTTCTCAT
ATCAATCTTATGCATTTATGATTAAATTTAACATATAATTCATCGTCTTC
GACGGAGTTGAATAATAAAGTGAATATCTCCCTTTACTAAGCCCACACTT
TTAGGTGGGCCGACCTTGTTAGGCAGACCGTGATGCAGTTTTGATTCTCA
CAGCTATTCTATTCTTGTTGAAGATAGAGGAAACAACTCCTCTCTGTCTC
TCTGGTTTTTCACGTTTCATTGTGGGGTGGGTGGATTTGTATTGCGCTCG
GAAATGTGGTGGAGTTGGGAAGTTTTGTCCTAATATCTTTCGATTAAATT
CTCTCTCTCTCTCTAAAGGTTTGTAATAGAGGTGTGAGCCTTATAATTTT
GTCCTTTTTCCCCTGTGATGCGCACAGATCACATTTCATGGCTTCTGCCT
CGGTGTCAAACCCAAAAGAAGGGAGAAAGCTATAACGGGAAAGAAGAAGA
TTCATACGAGTTCAGAGGATGAATTTTATTTCACATCTTCAACCAGATGA
TTACTCTGAGTTCGTGGAAGTTGATGCAACTGGAAGATATGGCAGAGTAC
TCTCTCTCCTCTCTCTTTCTCTCTCTCTCTCTCGGTGGGATTTAGCTCCG
GGAATAGTGTTAATTATTTCTCTTTCATTTTTTCTTTTGCTGCAGTACAA
TGAAATCCTGGGCAAAGGAGCTTCAAAAACAGTGTATGATTTGGAATTCA
TCCTTCCTTACTTTTTGCTCCATACCCATTTCATCAAATCTATGAATCTT
TCTGGGAATCTTTCTTGGGTAATTCATACATTTGTTAAATGTCCATGTCA
GATTTCTTCGCAATTCGAAGTTCCTAGGGTCTTCCATGGAATTATGATGA
ATGGGCTTCGTGATAAATCTATGAATAGCCACATTTGAAATCTATGAATT
GCCCATTTGGGTGTGGTGGATTTGTGAGTAAATTTATGCTGTTTTTTGTG
GGCTTTTGCAGTTACAGAGCATTTGATGAGTACGAAGGAATTGAGGTTGC
TTGGAACCAGGTGAAGCTATATGATTTTTTGCAAAGCCCTGAAGATCTCG
AGAGACTTTACTGTGAAATTCATTTGCTGAAGACATTAAAGCACAAGAAC
ATTATGAAGTTCTACACCTCTTGGGTTGATACGGCCAATAGGAACATCAA

TTTCGTCACTGAGATGTTCACTTCCGGGACTCTAAGACAGTAAGCACCCA
ACAATTCTTGGTGATTTTTTCATCTCATATGTTGGTTTCTGACAAACCTG
ACTGATGGCCCTTGATCGATCACTATAGCTTAAACGGATTCACGTAGCCG
ACCCTACATAAGTGGGGACAAAGGCATTGTTGTTGTTGTGGTTGTATGTT
GGTTTTTGAGTTTCTAAATCTGGGTTTTCCTCTCTTTTGTGGATTAGGTA
TAGGCTAAAGCACAAAAGGGTTCACATGAGGGCAGTAAAGCATTGGTGCA
GGCAGATCTTAAGAGGGCTTCTCTATCTTCATAGCCATGATCCCCCTGTG
ATCCACAGAGATCTCAAGTGTGATAACATCTTTATCAATGGGAACCAAGG
GGAAGTCAAAATTGGCGATTTGGGCTTAGCTGCTATCCTCCGGAAATCAC
ATGCTGCTCATTGTGTTGGTATACTTCTCATCTTTCAAAACCCTTTCTTT
CATTTGCAAATTCACTTTTAGAACTGCAAAACGATTGAGCTAGTCCTTTT
CTGTTGAATCCCACATCGATGTTAGGCACTTCTCTTGATTTCTGTTCGCA
GCGGTTTGTCAAAAGTCCACCCTGCGTGTGAGGGGACGGGTTGAATCCCG
CATCGCTTGTGTTACTTGATCCTGAGCTATAAATAAAGCCTAGGGCAATC
CTCCTCTTACGAGGCATCTTTTGAGAGTGAGTTAGGCCCATTGGATTTCC
TACGTTTTCCAAAACACAAGATTGCAATAGTGTCATCTGTTCAAAAAGGT
CTTTTCTCATATGGATTTGATTTTATTTGAATTCATTCTATTAGGGACGC
CGGAATTTATGGCTCCAGAAGTTTATGAAGAGGAGTACAACGAATTAGTA
GACATCTATTCTTTTGGGATGTGTATTTTGGAAATGGTCACCTTTGAGTA
TCCATATAGTGAATGCACTCATCCCGCTCAAATCTACAAGAAAGTTATCT
CTGTAAGCACTCTCTTCCTGTATAGCTAAGCGCCCCGCCCCCTAAGTTCT
CTCTCTTTCTCCATGACATGCTCTAGTATTTTTAGGGGAAAAGGCCCGAA
GCTTTATACAAAGTGAAAGATCCAGAAGTACGTCAATTTGTGGAGAAATG
CTTGGCTACTGTGTCTTTGAGGCTTTCTGCTAGGGAGCTTCTTGATGACC
CTTTTCTCCAACTTGATGAGTCTGAATCTGATTTGAGACTCATAGACAAC
GGGAGAGAACTTGATTCCATGGGCCCTTTATTAGGGCAGCCTTTTTTCGA
ACTAGATTATGAAGGGAAGTCGTTTAGTAATAGCTCCTACAATGGATACT
CTAACGGTTATGCGTTTGATGCTCAAAATGGATGGGGATATCACTCATCG
GAGATTGAGCAAAGTGGAATTGAACTTTTCGACTACAATGATGATGACAA
CGATCAGAACGAACACTTTGAAGGACTTGACATAACTATCAAGGGAAAGA
AAAGGGAAGATGATAGCATCTTTCTAAGACTCAGAATTTCAGATAAAGAA
GGTTAGAAACACTAAACAATCGTTTGATGATCTTTGTTTATTTGTGTATC
GTTTTGATCCAATCTCTTTGCAGGTCGTATCAGGAACATCTACTTCCCAT
TTGATACTGAAAACGATACAGCACTGAGCGTGGCAACTGAAATGGTAGCA
GAGCTTGACATAATGGATCAAGACGTTACTAAAATAGCTGATATGATTGA
TGGGGAAATCGCTTCATTGGTACCCGAATGGAAGCAAGGGCCTGGGATCG
AAGAAACACCTTGCTTCACAGATCACGGTTTCTGTAACAATTGTGCTTCC
AATCGCACCTCTAATGGCTCGTTCTTGAATTTCCTCTCGAAACATCCAAG
TGCCCAGAATTTGCAAATTTTGCATTGTTCTGGAAATTGTTGTGCTGCTA
TGCATGGCCGGTTTGAAGAGATCACGTATCAAGTCGATAGCCCTCGCCAA
TATGTTCAAAATAAACCATTCAAATCAAGCCAAACCAATGATTTTCATCA
AACGGAGTGTTGGTATCAGCATGAAAGCCAAGAATGCAGTTCAACTGGCT
CAAAAGAGAGCCCATCTGTTGGAGATCACAAGAATTTGGATCGAAAATAT
TCAGCAAAGGATGAATTAGGGATAGGAATAGCAAAGGAAATATCAACTCA

TGAAATGTCGGATGAGAATCAGCAGAATATGCGATGGCTGAAAGCCAAGT
ATCAGATGAAACTGAGGGAGCTTACAGATCAACACTTGGGAGTTGTACCA
AATTCTCCAAGGTCAGGTAATAGCCGAAACAAAACTGTGAAAAGGGACCA
TATCTACTAGTTATTTATTGTTTATAGTTAACAATCACAGAATACTGAAA
TGTGGTCCTTATGATAAGAATGTTGCTTCAGATTCGGCAACACAAAGGGC
TGAAAAATGCGAGGCCAGCAAAGAGTCCCAACCGGATGATGCATTCTGCA
CCAATTCATTGCTTCCAAACACACTTCACAGAACGTCGTCCCTACCCGTT
GATGCTGTTGATATATGA
>EUC11981-RA [gene]
ATGGAGAGAAATTTGAGAAAAGGAGTGACAGGTCAGCAGAGAAACCACGA
ACAAGTTCGATACAATTCTAGGCAAAATGAAAATGAAGGACTTGGTTCTG
CCAATCAAATACTTTTTCAGGATCCGCCTAGTTCTATTAATAGAGATATT
ACACGGCAGGATTTTTTAAAAATGGCTGGAGCTAGACCTGTTCTGAACTA
TTCCATTCAGACAGGCGAGGAATTCGCACTTGAGTTTATGCGAGAAAGAG
TAAACTCTCAGCAGCATTTCATTCCAAATGCTTGTACCGAGCCTGTTGGT
GCGACTTCATATGTAGAACTGAAGGGCATGATGGAAATACCTCATACGGT
TTCAGAGAGCGAATCGGATACCTCTATGATTCAATCTGTAGACAAAAATC
GCATCCAAGATCATGAGAGAAAGCGCACTTCTCTAAATGATGACAAGGCT
TACTATGAATCTGGGCAATCAGTGTCACGGACCTTGTCAAGAAATGAAAA
CAGGCAGGGAGTTCATAGTAAGGCCCCTTCAGGTGCTTCATCTAACTCAT
CAATGAAGTTGAAGTTTCTATGCAGTTTTGGTGGTAAAGTTTTGCCGCGT
CCTAGTGATGGAAAGCTTAGGTATGTTGGAGGTGAAACACGTATTATTCG
GATTAGCAAGGACATTTCCTTACAGGAGTTTAGGCAGAAAACCTTGACAA
TTTATAATCAAGCTCGTATGATCAAATATCAACTTCCTGGTGAGGATCTT
GATGCGTTGGTGTCTGTTTCTTGTGATGAGGACTTGCAAAACATGATGGT
GGAATGTGCAGTACTTGATGATGGTGGATCACATAAACTCAGGATGTTTC
TCTTTTCCGATAGTGATTTGGAGGATTCACAGCTTGCCATGGGGAGTGTT
GATGCTGATTCTGGGATTCAATACATGGTTGCTGTTAATGGAATGGACAT
TGGGGCAAGCAAGAACTCAATTGGTTTGGCTAGTGCATCAGCTTATAATT
TGGAGGAACTACTCGGTTTAAATGATGAGAGAGAGAGAGTGGTCGAGTTGTG
GCAAATTTAGCTGCAGTTGGAACTGAACCTTCATTAGTTGCTATGCCATC
AACAATTCAGTCTTCACGGACAGTGCTTCCAAGTGCATCTGTAGCTTCTG
AATTGAATCCACAGGCTTACCAAAGTCAGATGATGCATAATGGAGAAGCT
GAATGTCATTCATTATCCTCTGGGTACCCTATGGAAAGACTTGACCCTAA
AGAAAGGGGTACTGTTCTTTCAGGTGTCCCATCTCAATATGATGACGTCT
CTCATCAATCCAATTATGTATCAGCTGGAGAAAAAATGATTCTGCCACCT
CTTCATGGGCATATTACTCAGGAGGGAGGGGCAGTAGAAGATCAGTCATA
TAATGCCGTGAATGTTCAGGTTCCAGAAGTGTCACTAATGGAGGCAGAAC
TGAAAGATGATAGCTCATTCCTGAAGAAGAATGATTTTTCTAAAGATTTA
TCGCCAAAAAAGGAAGTCCTAGTAGATGAGGTAAAGTTGGAAAGCGATAG
CTCAGTTCCTGAATCAAATGAAGCTGAAATTGTTCCATTTCTGGAGGATG
AGTACATTGTTTCTTCTCATCTGTATGAGAGTTCTTTGAACTACGTATCT
AAGGATGAAAAGTTGATCGCGAGCACTGCTGCAGACACTGGTACTTCTGC
CTTGCCAATAAAAAAATGTTGTAATTTTCCAGGAACCTATGCAGAATTTAT

TTCCATCAGCAGCTGAAAAGGAAGAAAAAGTGAATGAAAGTGATGAAAAT
GACCATTTTTATACATCTGAAAAAACATCTACTCTTGGAGATGATGACTC
TGTTGGTTACTCAATTGAATTTAGCCATAAAGAACCACCAACTCTTTCTC
ATCGCGGTTTCCATTCTGAACGAATTCCCAGGGAGCAGACAGGACTTGGC
CGCCTGTCCAAATCTGACGATTCATATGGTTCCCAGTTTTTAATAAGCCA
CACACACTCTGATGTTTCTAAACAAATAGCTGCCTCATTTCATGATGAAA
ATGTGTCTTCTCAGAGAGAGGGGTTTATCTCTTCTGCAGAACCACTGTGT
GCAAATGTGCAAACTGCTGAAAATGGGCTAGGACAATATCTGAACTCTGA
AGAGGTAAATAATAGAAATTTAATGAATTTCATTTTTTGAGCTGAAGTCT
TTAGTACTTAATGTTTTTGATAACCGTAATATGTTGCAGGTAGTGAATAA
TCGCAAAGAGGACTCTGACAACAGTAAGGAAGCCTCTGGGTTCAATCTTT
GTACTACAAGCCAAGGAATTTCTGGCAACTTTAATGGAGATTCTACTTCT
AAGTCACCTGAGCCTCAATCAGATGAAAATTCTGCTAGAAAGAGCAATGC
AAGCAGTAAGGGGCTGTTGGCCAGGTCAGAGCGCTCTATTGCACCACTTC
CTCCTGGGGTGTCCTCTGTTGGTGCTGGAACTACTGAACAGGGAGACATC
ATTATTGATGTCAACGACCGTTTTCCTCGTGATTTCTTCTCTGATATATT
TTCCAAGGCTGGAACTATTGATGGTTCAGCAGGAATTGGTCTACCGGACG
GTGACAGAACTGGTTTGAGCTTTAAACCTAAGCGTTGGTCATTTTTCCAG
AAGGTGGCCGAAGATGATTTTGGTAGAAAAGATGTTTCACTCATGGATCA
GGACCATCTTGGTTTCTCATCCCTACTTACAAATATTGTAGAAGGGGCTC
CTCTTGATTATAATTTTCCACCTTTTCAAGCTGGTGGTGTTGCACAATTG
GGCTCCCATATTAATTTTGATACTGCTGTGCAGCAGCGGTCACATGATAT
TATGGGATCCAACACTTTGGATCTACATTCAGATTACAATCCTTCCGAGG
CCACTGACAATCAAATTGTGCAGTCTAGTGGCTTGTTGAGGAAGGCAGTG
GTTGGTGAATCTTATTTTGAGGTATTTGTTTAATGCTGCAGGCTATTAAT
TCTGTTTATTTCTTTCAGTCATATTTTTTGTAATTGTTTTCTTTCTTTGT
TCTTCCATTTTTCGGCTGCCTCGTGACCACTTAAAGGATGGGAAGCTGGA
ATCCAATACTACTGGTCAACCTCTCTTCGATCCTTCTCTGGGAGTTTTTG
ATCCTAGTGCCCTGCAGGTAGAGTAAGGTTAAAACAAAGCAATGATTTTT
TTTTTCCTGGATGACATGCACATATATTATTTAGGATTTTCAGTAAATCC
TTGTACTCCATGAAAAGTTTAAGGTGCCAACGGCATATTATCTTGAAGCA
TTTCTTATAATGTCAAGCACAAAATGCAAGTGTATATATAAAAATCATCA
AGTGCTCTACCCTAAGAGCATCTCCAACAACTCTTCTGTAAGCTATGTCA
AAAAAATTTAACCACCAATTTGCCATTTTTCAACCCCCCAAAAAAAAGAA
AGAGAGAGAGAGAGGCTCCAACTGGTGTGGGGCACACCTAATCCATATTC
GCCACAAGTCTAGCCTAGTGGAACATCTGGCAAGATACCGTTGGAGGCGA
GAGTCATGAGGATGGGGTGGGTTGGTCCCAATTGAGCAAAGCAAGCGACC
TGCAACAAAGGAATGAGACAACTGTTTTTGATGATAATTTTGAGTTTTGG
TTGTTTATGAACAGGCCAGTTGTGATTGTTGCCCATAAAAACTAGTTTTA
TTTGTTTGTTTATTTTTGTTTTTAAACCTGGTTTGTTTTAATTAGGGTTG
GGTTTGGATTTGGATAAGTTAGTTTGATAATTTTATTTTGTTTGGCTTTG
TTGGAGGATATTTCTGTCATTCTTTGTGTTTAGGGTTTTTGTATGGTTCT
CTTAGGGTTTTTGGATTGTTCTCTTATTTCTTTTATAAATAGAGGGAGAG
GCTCACACATTGGTTAAGCTTTCTCATTCTTTTCGTATTCTCTTCTTACT

TTATCATGGTATGAAGAGCAGTTTATGGGGGCACAAATCACATAGTTCTG

TTCAACCATTATTTAAATTTCTTTCCATATCACTTGTGAGTTAACTTGGT

TGAAACACTTACTACGAAGGGGTTGTCATTGTATTAGTGGCCGCATGACG

TGGAGGTCTATCTATTTATCTTTTAGTCTTGAAGGAGGGTGAACCTTTGT

AACAATGGTAGTATTGCTCCATTGTGGCCTGAAGGTCTTGGGTTCAAATC

TGGAATACCGACTCTCCAGATGCGGGAGTAAGGTTACATACATCTGTCCT

TCCCCAGACCCCACAATAGGAGGAGCCTTCGTGCACTGGACTACCTTTTT

TTTCTTGAAGGATGATGACCTGGACATGTGTGCTGTGGTTATTTTTGATA

ATGTCATACTAATCAGTTGATAGCATGAATGGTTGCTTTGTTCATAATGC

TTCAGTTGTTCATTTGTAGTGGTCAACCATGAGTCAGCAGCTTCTTTTAT

ATTTATCTGTTTAAATTTGTTTTTATTTTGGCTTGTGAGCTTGATATATT

ATGTCTTGATTATGCCCCCAAGCAGCGTTTAATGATGCTCATGGCTTCAT

GTCCATAATGGTTAGAAATGAGAATTTACCTTAGCTCCGGCACATGTATT

TGATGGAACATCATTCGAATCTTTCACCTATTCTTGTTGATTATTTGCTG

ATCTCTTTATTTCTATAGATAATCAAGAATGAAGATCTTGAAGAGTTGAG

GGAATTAGGCTCTGGAACATTTGGAACCGTCTATCATGGAAAATGGAGAG

GAACAGATGTTGCCATTAAGCGAATAAAGAAGAGCTGCTTCACGAGTCGT

TCATCAGAACAGGAGAGACTGGTGTGCTTTATTCAAATGAAGTTATCCTG

TTGAATTGTTATATATACTGAAGTTTCTTTGATTTCATCGTGAATAACAA

GCAAGGTTATTGTGCAGACAGTCGAGTTCTGGCGTGAAGCTGAAATTCTC

TCAAAGCTTCATCATCCCAATGTGGTGGCATTTTATGGTGTGGTGCAGGA

TGGACCGGGGGGAACACTAGCTACAGTGGCAGAATACATGGTCAATGGTT

CTCTTAGACATGTTCTACTTTGCAAGGACAGGTGATTTTTTAAACTCTTT

ATTGACAATAAATAGTGACTTTTTAACTGTTGTAAGCAGTCCTGCTCTTT

TACTGTTCCTGTTCTTATATATTAAGTGTATGATCAGGCATCTTGATCGT

CGTAAGCGGCTGATAATTGCTATGGATGCGGCATTTGGAATGGAATACTT

ACATTCCAGGAACATTGTGCATTTCGATTTGAAATGCGACAACTTGCTTG

TGAACCTTAAAGACCCTTTACGACCTATTTGCAAGGTGACTCTCTTCCCA

CGTGTCAAGTTGAATAAATGAATGAAGCCTCTTGATTTTTCTTGACTTTA

TCAGCACCTGATCTACGACCAACCTCCGGTCTTTGGTCCTTCCAAAGGCA

GCTGGGACATCCTGGACTTGGAACTAGAGGCCTCGCGCGTGAAAAGTCAA

TCATTGAATCTACGGTCCAACCAATTGGGAGACAATTAATAGATTATTTT

TCGGGAGCGATTCCCCCCCATCATTTCCATGATTTTATGGATAAGGGAGC

TTAGTAGTCGCTTCTTATTTTTTTTTTCTTCCCCTTGCCCCACACAGCTG

GACCATGCTTTAATGCATATTGTATTTGTTAAGAACATTTTCACCAAAAT

CCTTGTATTAGCATTTGAGCTAATACAAAAGCAGAAAAAGAAGCTCTAAA

CAGATTCGCCAGTTGGCTTTCTAGTTTAAGAAGGTCACATTTGATTCTCC

AAATGTAGGGTACCACATTTGATTCTCATAATAATAATAATAATAATAAT

AATAATAATAATAATAAGAGAATTTTGGATTTTTTAGGGATTGGATTTTT

TAGGTAATCCCATTTTTAATTATTAAATATACATGAATCCCATTGGCCTA

GCCTTTGTGAGAAAACACATTTTCCATTTTTAACAATAATCTTTTTTTAT

TTTTGTACCCCTTTAAAAAAAGACGAAAAGCTTACCATTTATTTTCATCA

GTCGTCCCCCTCGCCTCACGGCAGTGGGTTGGCGGTGGTGGACCAATGCG

GTGATGCGGTGGTTGGGGAGGTTCACCATAGTGGTCGGCAGTGGTCAGGT

GCAGTGGTCGGACATTTTAATGATATATAAAATGATGGAATGAATTCTAA
GGGCATATTAGTAAATTAGTGTACTTTTTTTTAATTGCTTGGGTTTTGGT
ATAGAAAGAAATTGTCAGAGGTAAACAATTGGGATTTTGGGGTCTAGATT
GAAATACCATAGAATTTATTGTTGGATTAAAGTACTTTACGACTCCCTAT
TTTGTGTATTAAAGGAGTGTTGTTGGAGTTGCTCAACATTTGTCTTGTTT
TAGGACCCATGTTTCTAATCAACACTGTCTCTCAAATGATAAAATTCAGG
ACATATCTTTCTTCATGTTTTTTAAGTGAATGATTTCTAACAATCGAGAA
CAAGTTCTTGAAGAATCAATGTTATCTTTGTGCAGGTGGGTGATTTTGGG
TTGTCGAAAATCAAAAGAAATACTTTGGTCACTGGTGGTGTTAGGGGAAC
CCTTCCATGGATGGCTCCAGAGCTGCTAAATGGTGGCAGCAGTAAGGTTT
CTGAGAAGGTAGGTTTTACTAGTATTAACATGTTTATATGTTCTATAAGT
GGAGCCTGAACTTATTAATGAAGTTAAAGTTAAACTTGAATATTTTTTTG
TATTAGTTTATGTTATTTGCTGGTTGAAGGTTGCATGCAGAATCTATGGA
ATTAGATCCAATTAACTTCAATGGTCTTCGTTTTCTTTTTTTAATCTATC
TCAAATGAAGTCTATTTCTTGCCCCCATTATGATGTCCAACACTATGTAT
GGATTGGGGAAAAGAGAGGGAAGTGGATTGGAGTGGGCCTTCTTTGTTGT
TTGGATGGGAGGGAGGGAAAGACGGGGATGGGGTGGGGGAATAGAAGGAA
GTTGAATCCTTCCAATTTGGAAGGAAAGCCTATATAAATAATTTAAAATT
ATTATTTTACTGTTGTTAGTTTATTTTATTGCAAACATACTGTTTAGGGT
TAGAAAATACAAAAGGGTATTTTAGTAAATATATATATGGAGGGTGCTAT
GTATCTTAGAGAGTCTGTGAAGGGAAAGGGAACAACTGTCAACTCGTCTA
AGGGGGGAGAGAGAGCTACATCACGCAGTTGTTGAACTGACTGTGTGACT
TCAAATTTTTTTAGACTAAAGCTTAGGATGTAAAGATTGTTTTATTTGTT
ATATTTTGTAAGAATTGTATCTCTTCTCGAGTCATGCTGAATGAATCTCA
AATTCCACTATTTATAATACTATTTTTTATTGATTATTAAATGCTGTCAT
TAAATATCATTAATAATGTAATTAGTGGTTTCCATAATTAGCTTATGTCT
GACGTCGAGTCAACCCAACCAATTGGCAACTCTCCCTTATGTGCTAGCAA
ATGAGAGCATGTAATGTTCCCGTGGGTACCACGTTCGATCCTCTATCCCA
CAATTAATCTATAATCTTGAAGCCCGAAAGTTTTTACCGCAACACCGCTC
TCTAAGGGGGCTCACGTGTGTAGCGTATTGCATACGAATTGAACTTTGTA
CAGCTTTCGGGAGCACATGCGGTGATTTTTCTCTCACATGGCTCCTAAGT
TTATGGTGCACCCGATTTGATGTATGTATTGGCTTAAAGATCATTTATTT
AGGGTAAGAAAACATAGTATAAAGAAAAAGTTATATTTTCAGGTAACAAA
TATATATAAATATATAGTGAAAGGAGGAGCTCTTGTGCCGTTGTGGGATA
CTTCGTTCGTCTCAAATTAATCGACTAAATTAATCGACTTACTTTCTTTT
TTTAGTAATTTAAATATCACTTTTTTTTAGCAAACGTACTTTATTGCTTT
TCCCAAAATAACCCTATTCTCAATCACACATTTAAATTTCAAAAACTTTT
ATCACTTTAACACCATTCCCATTAAGGGTAATATGGTTAAGTAAAAAATA
TTCAAACACTATTAAAGTCAAACGTGACAATAATTTTGGACGGTGGGAGT
ATTTGATACATTTTGGTTTGAGAATTCCTACACCATGTTTTCCTATAGAA
GTACTATTGATTTCTCGTGCTCGAAGGCATTTCATGTTATAGAAATAAAG
AAGGGAATAATTCTCTTATTTATTGATATAATTATAAGGAAGCAAACACC
TTTAAATAGGAAGAGAATAACACATGGTTGCTAACCTTAGAATCCATATG
ATGAATGAATTTCCTAATTTATTCAAATCACTACTTTTCTAGATTATTCA

AATCTCTAATTAATCAAATCCATATCAAAACTAACCCTAATTTACTGACA
CTTCCCCTCAAGTTGGTGCAAAGATATCCATCATGCCCCAACTTGCTCAC
TTGAAGCTTGAAAGGCTGTCTCAACAGTCCCTTTGTCAAAACATCTACAA
CTTGCTGTGTGGTGGGTACAAATTATTCCTTTTTCAAGCTTTTCTTTTAT
AAAGTGTATGCCAATCTAAACATGTTTTGTCCTTTCATGTAGAACCGGAT
TATGAGCAATGCTGATAGCTGTCTTGTTGTCACAATACAACTTCATAGGA
AGACTTACTGGTTTTTTAAGTTCTTCCAGAACCCGTTTTAGCCAAAAAAT
CTCGCATACTCCCTGCGCCATTGATCTGAAGTCCGCCTCAGCACTACTTC
TAACAACAATACTCGGTTTTTTGCTCCTCCAAGTGACTAGATTGCCCCAT
AGAAATGTACAGTATCCAAATGTTGACCTTCTATCAGCGACTGAACCTGC
CCAATCACCATCTGTATACACCTCAATCCCTCTCTTCTCATTTTTTTTGA
AGAAGAGTCCTTTTCCTGGTGTGCTTTTCAGATAACGAAGAATTCGATAC
ACAACTTCCATATGTTTTTCGTACGGGGAATGCATAAATTGACCTACTAT
GCTCACAGCGAAAGCAATGTCAGGACGTTTATGTGACAGGTAGATCAGTC
TTCCAACCAGTCTTTGGTATCTTCCCGTGTCTACTGGAACACCATCTTTT
ACATCTTCAAGTTTAGCATTAGCTTCTATTGGAGTTTCAGAAGGTGTACA
ACCACTTATTCCATTTTCTCTCAAGAGATCTAAAATGTACTTCTGCTACG
AAACTACAATGTACTTTTTTGACTTTGCCTCTTCCATTCCAAGAAAATAC
TTTAATTGGCCCAAATCTGTAATCTCGAATTCCTTGGCTAGGCTCTGCTT
CAATTTGCTCATCTCTTCTAAATCATCTCCAGTCAGAATTATATCATCCA
CATAGACAATCAAGATAGAGATCTTGTCTTTGGTAGATTTATTTGTGAAG
AGAGTATGATCAGATTGTCCTTGTGTATATCCTTGGCTCTTAACGAACTT
TGTAAATCTCTCGAACCAAGCTTTGGGAGACTGTTTAAGACCAATTAGAG
ACTTCTTTAGTTCACATACCTTGGTTCCAAACTTGTCAATAAAGCCAGGT
GGAGGCTCTATATAGATTTCCTCCTCTAAATCTCCATTCAAAAATGCATT
CTTCACATCCAATTGATTTAGAGGTTAATCAAGATTAGCGGCAATAGACA
AGAGTACCCGAACTGTATTTAGCTTTGCCACTGGAGAAAAAATCTCAGAA
TAGTCCACTCCATATGTATGAGTGAAGTCTTTAGCTACAAGATGAGCCTT
GTATCTTTCCGAAGATTCATCTGAGTTGTACTTCATGGTAAATGCCCATT
TGCAGCCAACAACAATCTTCCCATCTGGCAGATCCACTTTCTCCCAAGTA
ACATTCTTCTCCAGAGCTCTTATCTTCTCAAAAATCGATTCTTTCCACTC
AGGAACATTTAGAGCCTCTTGGACATTGTTAGGAATGACCACACTAGACA
CTTGTGAAATAAAGGCGCAACATGAAGATGAAATATTTTTGTAAGATACA
AAATTTGATAAATGGTGTTTATTACAAGACCTAACATCTTTCCTGAGAGC
AATGGGAAGATCAAGCTGACTAGAAGAATTTGACTCAGAATTACTTGGAA
GAATCTTTGACGAGTCATCATGAATTTCATGATCCAACCTCTGATTAGAT
TCTTGGTAGTGTTGTAGGATAGTACCTCTTTTTTGTGCTAGATACATCTT
TGAATATACAAGACCTTTATACCGTTTCGCACACATCACTGTAGTTTCAG
TATTTTGATTCTGAGATGGCATTACCAAAGGTCGTTCATTGGGGGGTTCA
ATATTTTTAACATTTAATTCATTTGTTTCCCTATACTTTGTGTTTTCCAT
ATCATTCTTTTTCGGTAACTGGTTATGAGAAATATCATCATGAACAAACT
CGGAAAATAAACTATCTAAAAAATCATCTTCGTTAGTGCTCTCCCCCTGA
AGAATGAAAATTGCTATAGTAGGATTGCCCTTAAAAAAATGTGACGTCCA
TAGACACGAACATTGTTTTGGAAACAGGATCAAAACATTTATAACCTCTT

TGATTTGTAAGATAGCCTACAAAAATACATTTATGTGCCTTTGGATCAAG
CTTAGAACGTCTATCATGATGATGAACAAATGCGACACTTCCAAATATTT
TTAATGGCTCGCTTGGTTGGACGGAATGAAATGAAGGTGAAATAGAATGG
AAGAAGTAATGGAATGAAAGAATGAATGAAATAAAATTGTCAATAGAATC
ACTATTCAATTCTCTTGTTTGGTTGGTATGGAATGAGGGAAGGAATGGAA
CAGTGAAATATAGTAAAATTCCCATTATGCCCTTATAAAATTATTATTCA
ATTCTCTTGTTTAGTTGGTATGAAATGAGTGAATGAATGGAATGAAATAT
AATAAAATTCTCATTATGCCCTTATTACAAAATTTTATTATATTTCACTA
TTCCATTCTTTTCTAATTTTTATTATATTTCACTATTCCATTCAATTTT
AAGTTTTTGTTCTCTAATTTCTATTTGTGTTCTTAATTTTCAGTTTCTAT
GCTCTAATTTCTGTTTCTGTTCGCAATTTTCAGTTTTCGTTCTCTAATTT
TCTGCTTTTATTCTCAATTTTCATTTTATGTTCTCAATTTTTTGTTTCTG
TTTTCAATTTTCAGTTTCTGTTCTCAATTTTCTGTTCTCTAAATTTTTAT
TTTTGTTCTCAATTTTTAGTTTATATTCTCAATTTTCTGTTTTTGTTCTT
AATTTTCATTTTTTATTCTCAATTTTCGGTTTTCGTTCTCAATTTTATGT
TCTCTAATTTTATGTTTTTGTTCTCAATTTTCAGTTTATGTTCTCAATTT
TCTGTTTCTGTTCTCAATTATTTGTTCTCAATTTTCTGTTTCTATTCCCA
ATTTTCTGTTATCTAATTTCTATTTATGTTCTCTAATTTTTGTTTCTGTT
CTTAATTTTTAATTTTTGTTCTTAGTTCTCAAGAGCTTAAATATAAGGGC
TATTATGTAATTTAGGATGTTGATTCCATTGGGTTGGAATTTTATTCCAC
CCCATCTCAAATGGAATCTCATTTCACCCTATTTCATTCCAATGGAATCA
CAAAAGTGATTCCATTGGAGAACCAAACAACGGAATAGGAATCGAGGGCA
GAATGCGGTTCCATTCCGCCTGCAATTCCGTCAAACCAAACATGCCGTGA
GGGAATATCTGTTGTTATCCGAGTGGTGGGAAAACATTCGGTGAAAAGCT
TTATAGGTGTTTTAAAGCTCAAAATTCTTGTAGGCAGCCTATTAATCATA
TAAGTGGTTGTTAAAACAGCTTCTCCCCAAAGATACTTAGGAACCTTAGT
TTGAAAACTTATAGCTCTAGCAACTTCAAGAAGATGTCTGTTTTTTCTTT
CAGCCACCCCGTTTTGTTGAGGTGTATTATTATAAGAATTTTGCTGAACA
ATCCCTTTTTCGGCAAAAACATTCCCTAATATAGTGTTAAAAAATTCATT
TCCATTATCACTTCAAATTTTTTTAATTTGAGTCTGAAATTGTGTTTGGA
CCATATTATGAATTTTTTTAAACATAATCTCAACATCAGATTTTTCTTTT
AAAAGGAACACTCAAGTGACTCTAGTATGATCATCAATGAAAGTAACAAA
TCAATGTTTCCCAGAAAAAGTTGGCATTCTCGAAGGACCCCAAACATCAC
TATGAATCATGGTAAATGGTTTTGATTTTTTATTTGGTTGAGGTGGAAAA
ACAGCCCGATGATGTTTTGCTAACTCACAAGATTCACACCGAAAAACAAA
AGAATTTTTATTCATGAATAATTTGGGAAACAAATGTTTTAAATATTGAA
AATTTGGAAGTCCTAATCGATAATGCCATAACATAATCTTATTGTCACTA
GAAACATAAATAGAATTGAAACAAGTCTGTTGATTTTGCCTTCCAAAGGT
TGATCCGTTATTAAAAAGGTGGAGGCCACAATCTTATTTAGCACCCCCAA
TCATTTTCCCTGAGATTAAATCCTAAAATTCACAACCAGAAGACCAAAAA
TTAACTTGAAAATTATGATCAAGAGTTAATTTGCTGACAGACATGAGATT
ATATGACAAATGGTAAGAATTAGAGAAGGGGAAATGGTAACTGTTCTTTT
CCCAGCAATAGTAGCAAAAGAGCCATCAACAATTTTAATTTTTTGATTCC
CTGCACAAGGTGTATATGAAGAAAAAAATTGTGAAGTGTCGTTCATATGA

TCGGTAGCACCTGAATCTAGTATCCAAGCGCTATTAGACTTGGTACAAGC
GATGTCGGCAGTGAAAGAGTGACCTGATTGTGCTAGAGAACAAGATATGT
TAGAATTTCCTAAGGATTGAGACTGAAATATTCTATATAGGTGCTCTATC
TGTTCCTTTGTAAACGGAAGTGACTCTAAGGAAGATTGTTGCCCTGGATT
TGAGCTGCTAGCTTGAAATGCGCAGGCATCTCCCCATGACCTGGTGTCAC
TGTCTGGTTTTTTCTTCAATTTGCCGGTTTCCCATGTAGCTTTCAACAAC
TTTCCCGGGTATGCCATGGCCTTCTACAATGGTCACACCAAGACCTCTGC
TTTCCTCCTCGCCTATCTCTTTCTGAATCTGCACCTTGGGCCACAAGAGC
AGATCCTTCAACATCTATTTTTGGTTCATCAATATTCTTCAACATCACTT
GTCGTCGTGTTTCTTCTACGTACTTCGGAAACCGCCCTTATCGTCGGCAA
GGGTTGCTTTCCTAAAATATGTCCTCGAACCTCATCGAGTTTTTTGTTCA
AACCAAACAAGAACATAAATACCCGGTCTCTCTCAACCTTCTTTTTGTAC
CGAGCACTACCATTTGGATTTTCCCATTGTTCAACTTCAAATATATCCAA
TTCCTGCCAAGTCATCATCATATCATTATAGTAGGTTGTTACCTCACGAT
TGCCTTGCTGCATCTTCCACATTTGAATGTTTAATTCGAACAATTGTGAG
TGATTGTCCAAATCTGAATAGGTTTCCCGAGTTGCTTCCAATACATTGCA
AGCCGTCGGTAGGTACAAGAAGGGTTTTCCAACAATTGGTTCCATGGAAT
TGATCAGTCAGGCAGTCACCATTGAGTTTTCCGAATGCCATTGCTTGTAT
CTCGGATCGTCGGTGGCTGGCGGTGTTACTTTGCCATTCAAGTGTCCGAG
TTTCCCTTTGCCGTCGATCATTAACTGCACGGACTGAGACCACTCAAGGT
AATTTTTTCCATTTTCAATTTGTGAATGATAATTTGAAGAGAATTTTCAT
AAAAGGAGGTAGCTAAAAGCTACTNCCCCGACGACATCGTCGGTCGCTGC
GAACCCCCCGATGAAATCGCCCCTCTGCTTGTGTCCGACGATGCTGAGTT
GGAGCCAACCATGGAGACTTTGAATCGAACCCTAGAGCTCTAATGCCATA
TAGAAAGAAAGGAGGGAATAATTCTCGTATTCATTGATACAATTACAAAG
GAGCAAACACCTTTAAATAGGAAGAGAATAATACACGGTTGCTAACCCTA
GAATCCATACGATGAATGAATTTCCTAATTTGTTCAAATCACTACTTTCC
CAAATTATTCAAATTTCTAATCAATCAAATTCATACCAAAACTAACCCTA
ATTTACCGACACATGTTCTTTTGGTTTTTTATTATATTAAATAATTTATA
GTTTGTTTAAAAATATATTAAATTTATACTTGTTGGACATTGGAAAATCC
AAAGCTATGTAAATATGTAGTTGTGAGAGAATATATAATTGGTAAAAAAA
TGAAAATAATTAAAAGTGGTAATATATATATATATATATATTTTTATGTT
AGCATGGGTAAAAATAAATATATGAGATTTTAAAAGGGTAAAAAAGGTGG
AAACCAGTACTGAATTAAATGAAAAATTGATTCTAAACCTTTCGTTTTCC
CCGCAGCAATCCTGGCTATCAAAATGTATGTATTTTCAAAAACTGTAAAT
TTTTATTAACTAGATGTTTCTCAAGCTAAAATATTTGATCCCAAGTTATT
TTGAGGCTTCATTATGATGGACGTGAGGTGGTACATTTGAGTAAGTTTGG
TGGGAATTTCTTAGTAAGAAGCATGGAATCTAGTATTTGACTTGGAATTT
GACATTTTAGAAGATTTCGGCAACTAAATGTACCATGTTTTCCTATATAG
AATTATGTTTATTTTGCTAGAATATTTGAGCACAAGTTAGTTTAAGGCTT
GAATATAATGGAAATGTGGTGGTATGCATGCAAGTTTTGTAGGAAGTTCT
TAGTGAGAAGCATAATATCACAAGTCTGATTGTGAGAAGGAATCTGACAT
TTTTAGAAGATTCTGTCAACTGCATATTCGAGGTGTGGTGGGGTGGGTGG
GGTGGGGGGGGACAGAAAATGGAGAACAGATGAAGACTTGTTTAATATTA

CATCATGCATTGTTTTCAATTCTTGTTCAGTTTGTTTTGGAAACCAGTT
TTGAGAGCTCCTAAATTTCAGGTTATTTTCTTTCATTTGCTGAATCTTCT
TGATTTTCCTTATATATGATAAGAAACCATGTTTCACTAAAATGATTGAC
AAATGACTGGAGATCCTGCGGGGAAGCAAAGCAAAGAAGACTCTTTTATA
TGTCAGCCCATGCAGTACTTATGATGACTATTCGTTTTGTGGCAGGTTGA
TGTGTTTTCGTTTGGGATTGTGTTATGGGAAATTCTTACAGGCGAAGAGC
CTTATGCCAACATGCATTATGGGGCGATAATAGGTGTGCTTTCTCTCTCT
CATGTCGCATTTTTGTTCAGGAGATAACCAAACAGGCTGAGTGCATGATG
CTACTATAATAATTGGAAAGTTAGATTTGCTTCAAGTACTCAGAGAACAT
GAAATTACCTTTTGATTTTCAAAAGTATTTTAAGAAAATACCAAACAGTG
CCTAAATTGATCTCATTTTGCATGAATCTCATTTGGCAAGAGTTTGGTAA
AATTTGATTAATTACTTGTTTTACTAAATTAAGAACAATATTGCTTAAAA
CTCGATTTTTTTTTTTGCAAAATACCCAATTGTGCAAACAAAATCGATAT
GCATACATGCTTGTCTTGTACTTTTTCTACTAGAGATACTTATCCCACTT
TTTTGGTACTTTTTTCTTTCGTGATTAATTTGAGTGCAAAATAACTTTTA
TTTCCAAATTAATACATGACTTGTTAAACAATATAATAATTGACTTTTGA
GTTTATAAATTGTTTTTCCTCGTGGATTACAAAATCGTATGTGCAAATTG
AAAACAATATCCAACTCCATCTATTCCTGCATCATCAGAAGAATGTTTAT
ATTTTTATTTGTCAGTTGTCCTGTGATTCGTAACACTTCTTTTGGTCCTC
AATGAGGATAGTGGATTATGCTCAAATGTCATTTTCATGATAAAATAATC
ACTTTCTTGTTTCTTTTTTGGATGAAAAGTGTGGATCTTTTGTAGATTGT
GAAAGTTTTAGTGCAAATGAGGATTTAAGTTCTTACATTTCGTATAAGAA
CGCTTCTGCATGCTTGCATCAAAGTAGAATGTGCAATGGGTTTTCTTTTA
AGTTGAATTCACCTGAATGTATATTGTTGTATGCATACATACATATATAT
ATATATGTGTGTGTGTGTGTGTCTATTTAAATAGAGATATTAATTACCCA
AATCCATGCTAAAATTATATCTTAAATATTCTAAAATTTCCCTGTCGGCA
AATACTGCATTCCTTAATTCAGCATTTATGTGTTGGTGCGTTTTAGTAAT
ATGCTATTCGATCGTCTTTGAAACATGATGCAGGAGGTATAGTGAGTAAC
ACACTGAGGCCACATGTGCCCAACTACTGTGATTCGGATTGGAGATCACT
TATGGAGCAGTGTTGGGCTCCAGATCCTGAAGTACGCCCATCATTCACTG
AAATTGCTATACGCTTACGTGTGATGTCCACAGCAAAACCTCAGGGTTAT
CCTACCCAAAACAAGGCGTCCAAGTGA
>EUC05776-RA [gene]
ATGTCTTGGTGGCAGAGCGGCTCCTCTTCCTCTTCTTCCAAATCATCTCC
CGAAGATGGTCTCCGCAACATGTTTAGACTTTTCTCCGGCCGGAGACACA
GCCACAGCTCCCAGCCACGGCAGTTCAGTCGCGGCAGGAAACTTCGGTAC
TTCGCAGAAGACGATGTCCGCCGTCCACCGCCTCCGGTAGACCCTCCCGA
GCTGTGGCGATCAGCGACCGCTCTTGAAATTTCGCCGAATCGCTCGTCGT
CAGGTGCCGCACCGGCTGCTGCTCCGCTGCCCCTCCCTTTGCCGGAATTA
GGGGCGTTGCTCCGCCGGGATTCAAGCTTGGTTTCCAATTCGAACTCGGC
GGATTGTCTTCTGATGTCACTGAAGGAGGCCGGCGGCAGAGAAGGCGTAG
AGGATAGAGGAAAATCGGAACGTTTGAATGGCGACGGAGTCATGATTGGG
AGGTAGAATTTCTTTGAAAAATCACAAAATTTATTACATAATTATCGTCT
TTATTACAATTTGAATAAATTAAGGTTGAGATTGAACAAATTGAAAACGA

TGATGTTAGAAATTTTCAAACCGATTGCATCGATTATAAGTGCTTATATC
AGAAAAAATGACGTTTCATGTCCTGAATTGTCTGAAAAAATTAGGTTTGA
TGATCCTTGATAAGGATCCACATTAATGCTGTGTGCTAAGACTAAGTTGT
TGTTTGGTTTAGTTTTTGAAAACTGTTTTTGAAAATTTTGAAAATTGTTC
CCAAATGAAAATTGTAAGAAACTCTTTGCTTTAGTTTTGAAAAAGAGAAT
ATTTGATGAAAATATGTTAAAAAATTGAAAATTAAAAGTGATGTATAGAT
TATAATAACGTTTGTTGCGTGAATGTGTGGTGGAGAAAATTTGTAAAAAT
ATAAAAAATATATAAAAAAACCAAAAGAATAAAAGTATAATATCAAAATA
AGTGATTTGATAGATTGATTACTGTGAAAATTGTTTTTTACTTCTGTTTT
ATAAAAGTGAAATAGTTGTATTTTGCGTCTTTGAAAGTAAACTGAACAAC
CCCTAAATATTTAAAGCACTTATTTTAAGGAATCTATATGGTAATGTAAA
TTATTTTACTATATAGATATCTCATAAAAAAAAACTTTCTATTTACAGTAA
ATTTTCTGGCCACGATGCTCGAAAGAGTACTGACCATTCGGAGCCTCAAT
CATCAAAGAAATCGAATGGCAAGAAATCAAATAAAAACAACTGTCAGATC
AACATTCCGGTCAGTGCTCCGACCAGTCCTTACTCTAGTCCTCTACGCAG
CCCACACGGAAACAACGGTGACCTTTATCTACCTCCTTACATGATTCCAC
CCATATTTCAAGTTTGGTCTGCACCGGAGTTGCCCTCTTCGGACATGAAC
CAAGGACTAGGTTTCCCTCATCAAACGTTTCCTGAAAAAACTGCGTTTAG
CGTCGACAGCTCGCCGCTCCGTAGTCCAAGAGTAAGCCCTCACCAGACCG
CCAGAAGTCCCAGTGGGCCCATCTCGCCATTGCATAGCAAATCGACAGTT
GAGATCGCATCATCACGATGGGAGGGTAATCCTCAGGCCGATGTCCACCC
CTTGCCACGTCCTCCCGGAGGTATGATCCCTTCACAGACAACTGTTCCCC
AATTTACGATAAAACCGGAGGTGATGCCATTGAGGAGACAATGGAAGAAA
GGAAAGCTTATTGGACGAGGAACATTTGGGAGTGTTTATGTTGCATCAAA
TCGGTAAGTTAATCTTTTGTATTTTTTTGTTCCAAGATTTCTAAGTATGT
TCCCAATTCAAGAAGCCGAAACAGAATAATGAAAAAAAAAAAAAAAACATG
TGTTTTGCTCCCAAATTTTTAAAACAAATTTCTAGAAAGAAAACAGAATG
GTTCCCAAATCGCAATTCTGTTCCAGATTTTTTAAGAACAGAAACTTTAA
CGGGGAACAAAAACACTAAATCAACGCCACCTAAGTCTTCTGTTTTCTCG
TTCTGTTCTTTAATTTTCCTCGTCGAAACATGTTTTTTAACATGACAAGT
TCTTTCATCATCAGAGAAACTGGAGCTTTATGTGCGATGAAAGAAGTGGA
GCTATTGCCGGAAGACACTAAATATTCAGAGAACATAAAACAGTTAGAGC
AGGTTTTCATTTCATTTTTTTATTTTTTCTTAATATGAAACGTATTTCAT
AATTTTATTTTTTAATTTCGATTTCATACGTTCTTATTCATCTTTCCAAT
TCTTACAGGAAATCAAAGTCCTCAGTCAACTAAAGCATCCAAACATCGTT
CAATATTATGGCAGTGAAATAGTAAGTAATGTCGTGTTTGGATTTGCCGC
TTTGGAGCCTCAGAAGTGGCAAAAAACATAAAATTTAAATGCTGGCCTCC
AAATGCTGCCTTAAGTTTTATTTACAATAATTAGAAATTCTATTCACTTA
CAGGTTGGTGATCGATTTTACATATATCTCGAGTACGTTCATCCGGGCTC
AATCAACAAATTTATACATGATCATTGTGGAGCAATTACGGAATCTGTTG
TACGAAATTTTACTCGCCATATTCTCTCCGGGTTGGCTTACTTGCATAGT
ACGAAAACTATACACAGGTACGAGTCACATTCTTTTTGAGAAATTTCATG
TTGGGTTCTGTCTGAAAACTATGTTACTTGCGACTGTGGAATATCCATTC
TGACGAGCGAAAAAAAAACACTTTTCTCTATAGGGATATTAAAGGGGCTAA

CTTGCTTGTGGATGCATATGGGGTTGTCAAGCTTGCTGACTTTGGGATGG
CTAAGCATGTGAGTTTTGAACTTTCTAAGGGACTGTTTGTTTCACTTTTT
CTCCGTTTTTAAATACTTAAAATACTATTATTTCTTACTGATTCGATTTT
CTTTTTAACCACAGCTTACTGGACAAGCGGCTGATCTTTCTATGAAGGGA
AGTCCATACTGGATGGCTCCCGAGGTATATGCTTATTTTTTTATTTATTT
ATTATTTTTTTTTTAGGTGTTAAGATTTTGAGAACATGTTCTTATTTTTC
TGTATTATTAATTTGATTACAGCTGTTGCAGTCAGTAATGCAGAAAGATG
CTAGGGCTGATCATGCTTTAGCTGTCGATATTTGGAGTTTAGGTTGTACT
ATAATTGAAATGCTGAATGGGAAACCTCCTTGGAGTGAATACGAAGGCGT
AAGTTTATTTACTATTTCTGTTGCCATAAAACTTAGAACAGAAACAGAAA
TAAAGTTTTGATGTTTGTTCTGAATAAATTGGTTATATATATATATCTGT
TTCTGTTTTGACTACAGGCTGCAGCCATGTTTAAGGTGATGAGAGAGACA
CCACCTATTCCTGAAACATTGTCTGCGGAAGGCAAGGATTTCTTACATTG
TTGCTTCCTTCGAGACCCGTCTGAACGCCCAACAGCCGCCGCCTTGTTAG
AACATAGATTCTTGAGAAATTCTCAGCAGCTGGATGTTCCAATTTGCTCC
CAGTTCGTTCATGGCATGAAATTAACGGTATAATTACTTTCCATTTTTCT
TTAAAAAAAATAAAATAAAATAAATTTAAACGTGTAACTTCAACAGGATA
AAGCACAAGCTCCGGGAGAGGTTGCCAGTCATAAAGTTAATCAGTTGCCG
GAGGGAAAATTGGTAAAAAAGTAAGATTTTATTTATTTATTTTTATCGTA
TTTTTTTGGGCCATGATTTTTCTAGTTTTTGGCTTCATCAATGATTTTTT
TTTTTTTTTAACAGAACATTGCGGGAATTAAAAGTGGCGCCACACCGTTC
GCCGCGTTCGATTCTGGAGGCAATTTCTAGTTTGTCTCCTCCGAGGTTGG
GCCGAACATCGGACCCTGTCCTTACTGCCGGTCCTAAAAATAACCCTTAA
>EUC05265-RA [gene]
ATGGAGAAGGAAGAATCCGGTACAGCGATTCAAATAAAAGGCGTTCCGTC
TCACGGCGGGAAGTATATTCAGTACAATGTCCTGGGGAATCTCTTCGAGG
TATCTTCTAAGTACGCTCCTCCTATTCAGCCCGTCGGCCGCGGGGCTTAT
GGCATCGTCTGGTACTCTCTTCTCTCTCTCTCGCTCGCTCTCTGTCTGTG
CATAATCTGTATACGAGTTATATGTGCATGCGTATGTGTTTTCCTGTGTG
CATGTGAAAATTGGGGGCGTTCTGTGCTTCATTCAATTGAGAAAATGGAC
AAAATTAGATGGATTAGAAGACGGGGTATGCGAAGCTACCGGCTCTATCC
TGTCCTAAGAAACGCAGGACTGGTTTTAGTTCTTGGATTTTTTATCATTC
TTTTAATTACTTATTTAGGAGTTGACAATAAAGTATACTTCCAAGTATTT
TATTTGATCATCTGAGGTTTCTTAGCGACACTGTTGAGTGTGATTTCCTT
TGAATTTGTCTTGCTTTTGTATACATAATGTTTTTAAGTCATTTCCACTT
CTTCTTCTTTTTTGTGTGTGTGGTTGGATGGGAGGTGGGATGGGTGGGTG
GATTAATTTTCAGATTCTTATTTCCTTTACTTTTATTAGGAATATTGAAA
ATATGTTATCTTAAGATTCTTAACCATTTTCTCAATTCCCTGAGTTCTGT
CAAAACATGGCGATATTCAATTGTCCTGTTTGTAAAGCATGTGTAAATAC
TGCTAAAATTTAATCAAATTGTAAAATAGAAGTTCATAAATCCAATATCC
GAGTGATGATTATCCATGTCATTCAAAGTGAAACTCTAATGAGAAATTGA
AAATGGTCTATGTAATTGGCGATTAACCCGAGAAGGCCCCTGTCCCAAGG
GGTCATGCATCCATCTCCCCTTCCCAAAGGCTTTTGGGAGGAGGGGATTC
TTTATCGCGGGAGGTTTTACTATGATAAAAATATAGATCTATATAATTGT

TCAGCAGCTCCTATCACAATCCTAATGAGTAGGCTTAATTAATTTAGAAC
TTGAATAAAAATGACATTGAAGTGTGCACCGTAATACTTAAATGTACTTA
TTGCATCTTCTGGTTGACCCAGTCAATTGGGAAGCATAATTATTGAACAT
AGTGAAGATATAATACACAGAAATTTGGCTTGTATTGCTCAAAAAGTGGT
TTTTAGACTTAAATTTCGCTACTGATTGTAAATTGTATTTCATTCTTGGT
GCAAATCTAACTGGGTAGTATTGATTTTCTCATTGCAGCTGTGCTACAAA
TTCGGAAACAAAGGAAGAAGTTGCAATAAAGAAAATTGGAAATGCTTTTG
ACAACAGAATTGATGCTAAAAGGACCCTGCGTGAGATCAAACTCCTTTGC
CATATGGACCATGATAATGTACTCACAATTCCACCGTTCTTATAGTAACT
TCTTTGCAACTGCAAGTTAACTTTGACCATTACCTTTTCTAGCTGTTCCT
AATGTCAGCGCATGAGAACACTAGTTCTACGGGCTTTGTTTGCCATATAT
ACATTTTATGTTTAATAATTTGTTCGTGCTCACCTTGAAAACTAGAAGAT
TCTTTTGATAAAGGAATCTGAAGGCTATTCATGTTTTCCTTTTGTTTTTG
TTGTTCATAGTTTGATATTGTAATCCAAAATCTTTACTATCTTTGCAGAT
TATCAAAATTAAGGACATTATACGGCCACCAGATAGGGAGAATTTCAATG
ATGTTTATATTGTATATGAGCTAATGGATACTGATTTACATCAGATAATA
CGCTCTTCCCAAGCACTCACAGAGGATCATTGCCAGGTACTTCTAAAATT
AAATGTGATATTAATAGCTAAGTATTTTAACTGGTTGCCCAATTTGTAAG
ATTTAACAAAGATAATGAGTCTCTCTCATGAGTAGTTAAATACAAATACA
AGCGCGCATTCATATTGTAACAGGCAGGGATGCAAGTTTGCATAGTGCTT
CATTGGATGCAGAAACATGTTTATAAAGCAAGTATTTGGTCCTGCCTGTT
TCCTTATTTCCAGAGGAGGTGAATTAATATACTTCTTGTGCACATCTAAA
ATTTCAGTTAAAATAAGATAGAAGAAATCAAGATGCACTTCATTTGCTTA
TTGCTTCCTTACGAAAATGTCAATTGTATTTTGATCCTCGTAATTCTTTT
TACATGAATATCTATAAATTTCTTAAGGGTTGTAAATTTTACCAAAAAAA
AGAAGAAAAAACAAATTATTGGGTATCTCGTCCTTACTAATTTATTTTCC
TAGAAATGGGACCTTGTTATTCTTGTCTCAGATAGTTGATTTTCTGTGAC
AATTGTGTGTTCATTGTTTTAGATTTTACAACGCCTTCCCTTATTCCGAT
GCTACTACCAGCCTTTCTGTGTTTCCTATAGAAAATAGCCATCTGAACCT
TTAACTAATAGGTGGAGGCGTGGAATTATACATCTCTATTGACTTTCTAC
TGGAGATCATTCAATCCTAGGATTTCCTGCCACCGTTTTTTTTTTGTTTT
TTTTGTTTTTTAAAGAAAGAAGAAATATATTAATCACAACAAGGAGGAAT
TAAAGACCTCCTAGATTACAAAATTTGATTTTTCTAAAGTTAGAAAAAAC
AAGAGAATAAAGTTTTAAACACCCTCTAACAACATGAGGTAAACGAGCAA
AATTAGAAGAAACAAGAGAATTCCCATCACTAGCAAACCTGAACATCCCA
TCAGCAACAAAATTAACTTCTCTGTAAACATGATAAAGAGGAAAATTACC
CTTCTTAAAGAAATGATTTCAGTAATGTAGGGAAAAACTTTCCAAGGAAT
TGCAGCCTTTTGCAAAGCCATATCAACCAGCACCTTGCTGTCAACTTCCA
AAACTGTCATAAGCCAATTATGAGCAATACAGATTTTAAGTCCTTGCCAA
AGAACAATAACCTCAACAAACAAATTGGTTTGAAAGCCCAATGGAGCAGC
AAAGGCATAACACAATAGACCATGATGATTTCTGATCACTCCACCTACAC
CAGAAGGGCCTGGATTACTTATGGAGCTCCCATCAGAATTTAGCTTATAA
TAACCAAAGGCAAGCTTGGTCCACTTAAGCAACACAAACTTAAAAGAAGT
AGTCTTTTTAGCAAGCTTGGCATGAAGTATGAACTCAATTCTTCTAATCA

CAGAAGGAAAGAAAGGAGCAACTTGATCAAATTCAGCTTGACATCTGTAT
TTCCAAATCTCCCAACAAATCACCACAGGAAGGCCAACATTAAGATGCTT
GTTTTGTCACCAATTTAACAATCTAGACAACAATGTTCCATCATGATAAG
CACTTCCAAAATAAGCCCACACCTTTTGAGCAAAAACGCATGCAGAGAGC
ACATGATCCTATGAATCAGCACCATTACCACAAGAAGCACAGACAGAAGG
TAATTGAAATCCCGAAGTTTGAATTTTGGAATCAACAGGCACAATGCCCT
TGAGCACCTTCCACATGAAGATTGAAGACCTAAGAGGAATGGTATGATAC
CAAATGAGCTTGAAATAGGGAGGAGCCTCATAACTACTGAGAACAAACCA
GAATTAGAAAGCAACCGACACATTCTATCCTTAACATCCGAAGAAAGCGT
TTGCAGTTGCAAAATATTGGATTTGAAATTAGGGAGAAGTTGATCCACCC
TATTCGAATGCCATAAACCATCCACATTGAACAAGTCCTTAACTTGCAAA
TTAGCATACTGCTCCTGCACCAGCATAATTTCACCAAGGGGAAAATTGAT
GAGCCATTGATCCCACCACAAAGAAACATCACCCTTGCCAACTTGAAAGA
GGATGAAATCTTCAGCTAAAACCTAAAGCCTTTCCTTCAACCACATGCCT
CCATCCTATACTTTGATAAGAAGTGATCCTACAAAAATAAGTCTACTTTC
TACTACCATACTTCAACCTCATAAAATTAGCCCACAAAGAGTTGTCATCT
CTGAAATTCCACCATAATTTTGCTGTGAAAGCTAAAACCATATCCGTAAG
CTGCCTTATCCCCAAACCACCTTCTTCAGTTGGTAAGCACATGGCCTTCC
AGGATTTCCAATGGTGTTTTCTATTCCAAAAGAAATTTGAGAAAATACTT
TCCAAAGTTTTAACAACTTTTACAGGAGGAAAGAGAATCATGAACAAGAA
AATTGTCTGGGAAATCAGAACATGTTTGATCAGCACTAATCTACCTCCAC
TGGAAAGTATCTTAGAATGCCAACCTTGCAACTTATTTCTGACTTTAGCA
ATCAGAGAATCAAAAAGGGCCACCTTCTTCTTACCTGCAAAAATAAGACA
TCCCAAATATTTCATTGGGAGAAAGCTCCTGAGAAAGCCTGTTTTAGAGG
CTAATCAAGATAGCTTGTTAGAATGAATATAAGGAGATGGGATGAAACTG
CTTTTTGAGTAATTAATGAGTTGCCCAAAATTGATTTGATAAGAGTTCAG
AAAATCAAGCAATTTAGTGATGGAGGCTTTAGAACCATTTACAAAAATGA
TCATATCATCAGCAAAGGCAAGATGAGAAATAGACAAAGCAAATTTAGGT
TGGGAAAAAGGAAGAATGGAATTATTTTGGACAAGGGCTTTGAGACCTTT
GGAAAAAGTTTCTGCCATGATAATAAAGAGAAGAGGAGATAAGGGATCAC
CTTGGCTTAGACCCCTTTTGGATTGGAAAAAGTCACATGAGCTGCCATTA
ATGATCACAGAAAAGAAACAATTAGATATTAGCCTCCACACCATGTCAAT
GATCCTCTCATGAAATCCAAACTTTCTCAAAGTTGAAACAAGATAGAACT
AAGACACCCTGTCAAAAGACTTAGCCATATCAAGCTTAATAATGAGATTG
CCCCCATTAACTTTTTTAGGAAGAAAATGAACCATTTCTTAAGCAAGCAA
GATATTATCAAAAATATTCCTTCTTTCCACATAGGCACTCTGTTCAGGGG
AGATTAATTTGGGAAGAACCACCTTTAATCTGTTTGCCAGAAACTTGAAA
ATAATCTTATTGAAGAAATTACATAGGCTTGTAGGCCTAAAATCAGGGCT
GTCATTTTTGGGAATCAAAGCCAACAAGGTACTTGTACATGACTTGGAAA
GTTCAGCTCCACAAAAGAACTCCATAACAGCTAGAAACAAATCATCTTTG
ATGATATCCCAACAGTGAGTAAAAAACTTTCCAGAGAAACCATCAGGACC
AGCTGCACTATCCCCTTTAAGTCTAAAAACCACTGCTTTAATTTCATCCA
AAGTGGGAATGGTTAAAAGGCTTTCATTATCAAGATCACTAACCAAATGA
GGAATCGTATCCAAGAAATCCAAATTGGGCAAAGAGGACTGGTCACCTAA

AGCATCCTAAAAATGTTGCAAAAAACAATCTTGAATCTCCTTAGAATCTG
TCAGAGTGACTCGTTGCAAATTCTTAATAGAACTAATTCTGCTCTTTGTT
CTTTGACCTTTAACAGAAGCATGAGAGAACTTTGAATTGGCATCACCATC
TTTGAGCCATTTATAAACAGATTTTTGTTTCCAAAACTGCTCCTCAACTA
AAATAGCCTGCTTGTGCTTAGCCAAGATATGATTATAATTAGCTCAATTT
TCAGCATCATCATTGATGAGACAAACTTCTTCAAGTTTAGAAACCTCTAA
CCTCTATTTCAGCATTTTTGACTGCATTAAGAATGTTACCGTAAAAATTG
GTACTCCACTGCTTGAGCACAGGTTTTAAGGGTTTAAGCTTCTAGGCAAT
GGCTGATAAGGCAGATCCAGTATATTTCAGGATTTCCTGCTGCTTAATAT
TTCTTAGTGTTTTTTTTTTAAGTCTAGTCAGAAAGAATGAAAAGACACAC
AGACAAAGTCAAGACGTGGAGAGGGAGGGGGAGGGGTAGGGTGGGGGGAG
AGATTTTGATACTTGATCCATGTTTCCTAAATACAAAGTTCTGCGATGTG
GTGTATTCCTGGGAAGCTGCATATTGAGTTTATATGTCTCATATTCTATG
TCCACCCCCAAATTGATATTATGAATGATCTGCTCATAAGAAATGGTAAA
GATTTGTCTAAACCAGTGTTGTGAAAGGCTCGCCTCAGGCGAGCGAGGCG
AGGTGCAGCTACTGCGCCTCGGCACTGTGAGGCAAGGCGACTTCAAAGAG
GCGCGCGCCTTGGAGCTGAGGCGAGCAGGCGTAAGGCGCTGCTGAGGCGC
GCCTCTTTGAAGCATGGCTTTTTCAAGTTTTTTTCCATGTGAGTTACCTT
CGTTTGTTGAAAGGTTGAAACCAAAAAAAAAACCTCACCAGTGAAGAATT
AAAAAAAAAAAAAAACTCGCCATTGAGGAAGAAACATTCGAGAGCTTCTT
CTCTTTCTCCTTCAATTTCTGCTTCGAAGTTCGCATAGGTGGCCTTCGTT
GCTCTCCTTCCGAAGCCAAGATCATATTCGTTGATTACCAATTTCTCGAC
TCGCCAAACACGCCCTAGAGATGATTTTGAAAACAAGTACGTGTTTGCCT
CTTCTTGTCGTAATTCGAGAAACATGTATTAACGATTCGAATTCAGCCGC
CATGGAAATGGAGTACGAAGCTCTGATCAAAAGGGGTAGTTCCGATTTCG
AGATCAGGTGGCCAAATGACGAATTTGACCCTATCTCCCTCAATTATACA
TCCGGCACAACGTCGAGCCCCAAAGGGGTGGTCTACAGCCACCGGGGAGC
TTCTAATTTTGATCGGAGGACTCTAAGATTTGCTCCTTTCTAATTTTGAT
CAAGAGAGAACAAAATATCTCAAACAAAATACATAAACCACCCTATAATA
ATATCTAATATGTGATTATGTATAAAAATTTTAAAAATATTTTAAAATAA
CAATTAACCCCCCAATTTTTTTAAAATCAAATCAAAATTAACACATGAAT
CCTCCGTTCAAGTATTTCGATTTGAAATGCAATATTACATGAAACTAGTT
CAAAGTGTTTTTTATTATTAAACATGCAATATATATTTTATTTTGGTGAT
ATTTGAATACTTTTCATGTCCATAATAATAGAAAAACACATATTTAAAAT
GTGCACCTCGCTTCAAAGAAGCCCGCGCCTCGCCTCGCGCCTCGCCGTCT
CGGGGGTCATTGCGCCTCAATGCGCCTTAAATAACAATGGTCTAAACTTT
AATGTTATTGCCTTATTGGATATGCTCCCACTAATTTGACTGTCAGCTTC
TGGTTGGGTTATTTAACTTTTCTATGTTTCCCAAAATATATCTTAACCCT
AATGCTATTATTGTATCCTTCTATTGCCCTATTAAGAGAATGAATGATAA
CCCAATTGACTGTTTTTTCCATTTACAGCAAGATATAGTTATGTTTGCTG
TAGTTTGTTTTTAGCTGCATCAATACATCCTTTGCTGTTTACGAGATATC
AGCAGCGGTTTAAGGCATTTTGATATAAAATTTTTGACAGCAGCAAAGCG
CTTATATCATCTAGGAGTGATTGCAATTTGGAAATTGCCATAGAAGTATT
TGTGCCATCTTAGACATGTTTCTGACATAATTATATCAAGATGGAAGTAT

CTTAGGATGCCATTCCACTGTGTGTTTCAAATGGTCTTGATCTTTTCAAA
GTTGCATTTCATAATTAGCTGTTGTAATTCTAAGTGTGAACAGATTGTGA
TGCATAGTTATCCAAATCATGATGCACGGAAATAATTGATGAATCCGTAG
TGCTGTTTTAAGGCAGAGACATCTTTTGAAAGCAGATAACCCAGTGTCTT
GTTATCCTGACAGTCTAGTTGAATACTGTTTTATTTGTTTTTCTTATTTG
CTAATTATTGAATTTTCTTTGGCAGTACTTTCTGTATCAGTTACTGCGGG
GACTGAAGTACGTGCACTCTGCTAATGTTTTGCACCGAGATCTAAAACCA
AGCAATTTGCTTCTCAATGCAAACTGTGACCTCAAGATTTGTGACTTTGG
GCTTGCAAGAACCACCTCAGAGACTGATTTCATGACAGAATATGTGGTGA
CCCGTTGGTATCGAGCTCCTGAGTTACTACTCAACTGTTCAGAGTACACT
GCAGCGATTGATATTTGGTCTGTTGGTTGCATACTAATGGAGATTTTAAA
ACGGGAACCCCTGTTCCCCGGTAAAGACTATGTTCAACAGCTTGGACTCA
TAACTGAGGTGCTTGTGCTCCCTCTTTCAATTCCCTTTGCTTGTGGATGA
ACATAAATAAGAACAAATTACACGTCTTTAGTTGATTTACCATTCAAATC
TTATGTTTTTAGGTTTAAAAAAATGAGCAATTCGTAAAGCTCTCCCATTT
TTGCAATTTGTGTAAGCTTTAGTTCCATCATTTTTAAGACTTAATTTCTT
GACCCATAAATTTGTAGTTTATTAAGGTATGACAACTAATCATAACTAGT
AACGTAGCAGTTAAAGTAGGACCTAAAGTTAAATATTAACTCATTATTTT
TTTCCTGTGGCAGCTATTAGGTTCGCCAGAAGATGCAGATCTTGGATTTC
TAAGGAGTGATAATGCTCTACGGTATGTTAAGCAGCTTCCTCATTTTCCG
AAGCAACCTTTTTCCAAGAAGTTCCCGGAGGTGTCCCCAGAAGCCATTGA
TCTTGCAGAGAAAATGCTTGTTTTTGATCCATCCAAACGCATCACCAGTA
AATCTCTCTGTGTGACTAATACTATTTCACGCGCATCATCATCAGTAAAA
TAAAACAAAATAAAGGGTGTGTTTTGGATAAGGAATTCGTTAAATTCATG
GAATTTCGACATGATGGGATCTTGAAATTTTGAATTTTAGTCCCATCATT
TGCGAATTTCGTTGTTTGGGTATGTTTTTCAAAATTGAAATTTTACTAAT
GTAATTTTGCCTCAAGAGGGAGTGATATTCCTTAAATCCCATTTTGATAT
TCCAAGGAATTTTGATTTGTGAATTTCAGATATACTTTCAAAATCAAATC
CTTCACCCAAGCACACCTTTCTTTTTTCTTTTCCTCTAACGTCTCTTTCT
TTTGATATTCTTCGTGCAGTTGAGGAAGCACTGAATCATCCATTTTTATC
AAGCCTCCATGAGATCAATGAGGAGCCCGTTTGCCCATCTCCTTTTTTCT
TCAATTTCGAGCAGTCTTCTTTGAGTGAAGACGACATCAAGGAGCTCATA
TGGAGGGAGTCTATAAACTTCAACCCAGACAAAATGTTGGAGTAA
>EUC05489-RA [gene]
ATGCAAGAAATTTTCGGATCAGTCAGGCGATCACTAGTTTTCCGTACACC
GGAAGGTGGTCTTGCCGACGAAGCCACCGCCGGCGGAACCCTAGTCAACA
AGATCAACTCATGCATCCGTAAGTCTAAAGTTTTTTGCAAACCCTCGCCG
CCGTCTCTCCCTCCTTCTCGAACGATCATTAAAGACAACAACGCACCGCC
ACCTACGATTCGGTGGCGCAAAGGGGAACTGATCGGCTGCGGGGCCTTCG
GCCAAGTCTACATGGGCATGAATCTTGACTCTGGAGAGCTTCTTGCGGTC
AAACAGGTCCATTTTGAACTCTTTTAGACGTTTCTTTTTAGTTATGGATT
TTGAGTTGAGTCAAAAATCGAAATACCAATAATTAGTTATTGCTCTCTCT
ATAAATATTTATTGATTTTAAGTTGGTATGTAAGCATGTTTATGTTGGTA
ATTTGATCAGTTTTTTTGTTGATCTGTTATTCAGGTTTTGATTGCCGCAA

ACGGTTCTTCGAAAGAGAAGGCACAGGTTGGGGAAAATCTGCTAGTTTGA
TTTAAAGGTCATTTTGTTATTTTAATTTACATGTCCCTGCAACTGTGTAA
TATCAACAATTTGCAGCATTGTAACTCTAATTTGATGTCAAAAAGCCTAG
GAGAAGATTCAAACTTATATATTAGGGACATTTGGGTGGGAGGTCCCTTA
TTTCACCTCTAATTTTAAAAAAGTCCCAAAGGCAGAAACAACTTGTATTT
TAGTTTCACCTATTTAAAAAAGGCAGAAATCCACTACACGCGCCTTTACA
CTCGCGGTTACATGTAACAATTTTGTGTGTAATTTTTTTTTAGATGTAAC
AAATGTTACTGTATTTTGTAACATGTAACTAATATGTGTGTAAAAATTTA
TTAGATGTACAAAAATCACACAAAATACATATAATTAGTTACATGTAACT
AAATATATACAAATACATAGCTAATACAACAACAATAATAACTAAGCTTT
TATTCCACTTATGTAGGGTTGGTTACATGAACCCATTTACGCCATAATGA
TCGATGAAGGACTATTCCCTCCGTCAGGTTTAATGTCATCAGATAATTTC
TAATAGTCTTATCTAACGTCTTCTTAGGTCTACCTTTCCCTCTAGTAGTT
CCCTGCACTTAAAGTAAATTATATTTTTTCACTGGTATTTCTATGGGTCT
TCTATACATATGTCCAAACCATTGTAAACGATTTTCTTTAATTTTCTCTT
TGATGGGTGTAATTCTTAAATTCTCTCTAATAGTTTCATTTTTTATTTTA
TCCATTCTCGTCTTCCCACTCATCCACTGCAATATCCTCATTTCTGTCAT
CCCAATTTTCTAGATATGATTCGTTTTTACCGCCCAATATTCTGAATCAT
AGAGAAGCGCTGGTCTTATAGTAATTTTGTAATATTTTTCTTTAAGTTTC
AACAGGATCCTACGATCACAAATCACTCCAACTGCACTTCTCCACTTTAA
CCAACTTGTTTTGACTTTATGCGTAATATCCTCCTCGATTTCGCCATTAC
TCTGAATTATTGAATCTAAATATCTAAAATGATCAACTTTTGGCACCTCC
TCATCTTCAATTTTGATCGCATCAACGTTTCTCCGGGCATTACTAAAATT
GCAACGCATGTATTCCGTCTTGCTTCTGTTAATCTTCAAACTTTTAGACT
TTAAAGCTTCTCTCCATCTTTCTAACTTAGTATTAAGACCTATTCTACTT
TCGTCAACTAATACTATGTCATCCGCAAACAACATATTCCAAGGTACTCC
ATCTTGAATATGTTTTGTGAGTTCATCTGTCACTAGAACGAAGAGGTAAT
GATTTAAATATGATCCTTGATGTAAGCTTATATTAATAGGAAATGCACAA
GTATCGTTTCCTCTTGTTCGAACACTAGTGATTATACTTTTATACATATC
CTTAATTACCTCGATGTACACATTGGATACCTGTTTCTTTTCTAGCACCC
ACCACACAACTTCTCTGAGTACCCGATCATAAGCTTTTTCCAGGTCCACA
AATACCATATGTTAATCTTTATTTTTTTCTCTATATTTTTCCATTAATCT
CCTGAGTAAATAGATAGCTTCAGTGGTTGATCGTCTAGGCATAAATCCAA
ATTGGTTCTCTTCTATGCTTGTTTCATGTCTTATTTTATATTTTATCACT
TTCTCCCAAAGTTTCATTGTATAATTCATAAATTTTATGCCCCGATAATT
CGTGCAACTTTGAGCATCCCTTTTATTTTTATAGATGGGCACTACGATAC
TCCTCCTTCAGTCGTCAGACATTCTTTTAGTCTTCAAAATGTCATTGAAA
AGTCTAATCAACCACTCTATCCCAATATCTCCCACACTTTTCCACACCTC
TATAGGGATTTCATCTAGCCCTACTGCTTTTCCAATTCTCATTTTCCTTA
GTGCTATTCTAACTTCCAAAACCCTAATTCTTTTATGAAATCTATAAATC
TTATCTGAAGATAAACTAGGCAACTCTAGTCTTAATTCCCTCATTAAATA
AATTATAAAAATAACTTCTTTATCGTTCCATAATCTCCCCATCCTTCATT
AGTACCTTTTCATCAGTGTCTTTGATGCATCTGACACTTCCAATATCTTT
AGTCTTTCGTTCACATCGCTTAGCAAGTTTAAATATATCATTCTCTCCTT

CTTTTGTATCTAACTTTTGATACAAATTATTATACATCCTAAATTTTGAT
TCCTCAATTACTTTTCTCGTCTCCGTTTTTGCATGTTTATACTTCTCTAA
ATTCTCTCTACTTCGATATTTTTGCCATTCTTTATAAACTATTTTCTTAT
TCTGAACAACTTTTTGGAGCTCCTCATTCCACCACCAACTTTCTTTATAT
TGTGGTCCTCCTCCTTTACAAACTCCTAAAACTTTTCTTCCTATCTCTAT
AATATGACTCGCCATATCAATCCACATTTTGTTGACGTCTTCATGTCTCT
TCTATAACTCATCATTGCTAATTTTATCCTTAAACAACTCCATCTTTTCA
CCTCTTAGATCTCTCAAACTTATCTTTGCCTGCTTATTTATCTTAGACTC
CTGTTTCCACCCCTGAAACTTTAGATCGGTTACCATTAGTCTATGTTGGG
TAGCAATACTCTTTCTTGGTATGACTTTATAGTCCTTATATATTAAACGA
TCATTCATTCTAGCAAGGACAAAATCTATTTGGCTATTCCTCTAACCACT
TTTAAATGTGATAAGGTGTTTATCCTTCTTCCTGAAACATGTATTTATAA
AAACTAAATCATACGCTATAGCAAAATTTAAAATCATCTCACCCATTGCA
TTCTTCTTCCCAAATCCATAACCACCATGTACTTTCTCGAAAATCCTCAT
TTCCTTCCCGACATGTCCATTCAAGTCTCCCCCACTAAATATTTTTTCTT
GGTTAGATATATTTTGAATGACTTTGTCTATTTTCTCCCAAAACTCTTGT
GTAACATTTTCTTGCAGTCTAACTTGGGGAGCGTACACACTAATAACGTT
ACTTACCTCCCCTCTAATCACCAACTTCATAACCATCATCCTATCGGCAA
TTCTCTTAACATCAACAACGCTATCCTTTAAATCCCTATCTACCATGATG
CCCACCCCATTCCTATGTTTGTACTTCACCGTATAACATAATTTATACCT
CGTACTCTCAATTTTTCTGGACTTCTCTCAAACTCGCTTAGTTTTTTGCA
AGTATAGAATATTAATCCTCCTTCTCATCACGTCCACTAATTCTCGAGTC
TTACCTGTCAGCGTTCCTATATTCCAAGTTGTCAACTTAAGCCTATTCTT
ATGGACTAACTTCTTTATTCGCACCCGTCTACCATAATGCGGCAACCCTC
TCTCATTTGACATCGTATCCGAGCGAAAACACGGCGCGATACCCTAGCCC
ACCCTCTCCCATTATTCACTATATCCGGGCGGTGGAAATGTAGCGCGTCG
CTAGTAGGGAACGCCTTAACGTTTAATGTATTTGGATTTATGTTATAAAA
GTTTTGACAAATTTTTTAGTTGGCTGTCAGCTACCTAACGCAACCCTCCT
CCTTTACCCGGGCGTGGGACTGGCTATGTGTAAAGAATAAAGTATCGAAC
CCTACTGGCGGAGTTACAAATACATGTAATTAATATTAAATTAATTTCTG
TGTGTAATATTTGTTTAATGTCACAAATATTACATAGATATATGTAACTA
ATATTACATAAGTAAATGTAATTAAAAATATACAGATATATGTAAAAATA
TAACAATTATTAAATTGTAACTAATATTCACTATAATACGAATTTACTCA
CCAAGTTCTCTCTACAATCCCTGCAATGCGGGATTAAAATTCAAGCAAAA
TCCAACTTAAGGGACTAAATCAAAACTTATATCTTAAGTGGGACTTTTCT
AAAATTAGATCTATTATTTGGGACTTCCGACCGATTATCTCTATATATTA
AGCATTTAAGTTCAAGTTGAAAATTTTATACTTGGGGTAGGTGAGCACTA
TTGTAACATGCGTACTTATATGTGGTATTTTCTGGAAAGTGGAAGTTGTA
GAGACAAACACGGAAACAACAAAGAAACTAGCCACAACAAATAGAATATG
GAAGAACAAGATCATGCTCATCAAATTATAGTCCCCATTTAATGGACAAT
CCATTTGACGGTTGCTGAGAAAAATTCCTAGATATAGTTCTTGATCTCAC
AGCCTGATATATTTCCTGTTCCACAAGCAGAACATCTCTGTGCTTTTGTC
TGAAAATTCTGTTATTTCTTTCTTTTCAAATAGCATAGACAATGGCAGCA
GACAGAAGCTTTCGATATAGAATCTTCATTTTTTCTTCCCTTCAAGTGGA

TTAAGCCCTGATCAACCCAACCATCCTTTGAACTGAAGTTGAGATTTGAG
TAACATTTCCTGTTGAGTGCTGAGAGCACTTGCAAAATAGATGATCACAG
CACTCAGTTTCCTCATTTCACATAATACCCTCAGTATGGGTTGTGAATTC
CCCAAATCTTCCAAGAGTAGCCAGTTTGCCCAGTTTTACTTTACCAAGGG
CATTAAAAAGTGGAACCATTGGTGTACCTCCTTTGCTCCACACCAGTTTC
TCCCATCTCATTTTCACTCCTTGAGGTCTCATTTTTCCCAACTATCGGCA
CATAACTGGTTGCAGACCATTTCCATCCATCGATTTGCATAATCTCCTTT
ACCTTTGAATATCTAATTAGTGCCATGCCATGTATAGGCTGTCTTCCCTT
TATGTCCAGTGGAGGTCCATCAGGGTACCACTGATCAAAAACAAAAATAT
AGTCTCTCCATTTGCTGTTTTATGCTCCAACCATGGTCTAAACACTCTCT
CAATTTCAAAATTTTCCTCCAATTCCAAGAGCATTTCTGTCGCAGTTTCA
AGTTCAAGAAGCTCGCCTTTCAGATCCTGATCCAAGATGTTCAGCTGCAT
TAACTTGAGTTATTAAAAAGTTTCCTTATATGTCTCATCATCAAGATAGC
ATTTCCACCCACTCCACTTAATTCACAAACCTTCCTCCTTGACAAGTTTA
CAAATATTACAAATATTCTCTCAAGCAACTTTGCTTCCTGAGGTTGTAAG
ATCTGTTCTAGACCATCGAAAAGCTTTATATAATTGGTCAATCTTCTTCA
GATTTTCAGTTATTTAAATTAGCATGAACTGATTTAATAAGCTGCAGCTT
TCCAGCGAGGGACAAACTCTTGCTGATCCTAGAATTAATTCTTTTCCCGA
TCTTCTCAATCAATCTTCTAAATCATGATTGGTATGCTTTATCGAAAGCC
AAAATTCTCAAACACCTTATGGTTGGGTGTTCTTCAGGTATACTTCAATA
TTGAAATAAGTGCTCCTTGAGATTCCCCTTCAATTTCGGCCATGAACACA
CTACCCTTTTGGTTATTTATAGCCAGACCAGAAAATGAACAAATTTATGC
AGACCATCCTTCCTAATTTTAGCTGATTCATTATCCCCATTGCAAAGTAA
AAGCAAATCGTAAACAAAACAAAGTTGGACATTTTGATCCTTGCACCTCG
AATTGAATTTAAATTGCACCATATCCTTGTTCTCCTTCTCAATAGTAAGA
GCCAGAACCTGTCTAACCCCTCTTTCACAGACAAATAGGCGGAAATTGGG
TCACCATGTCTTAACTCCTCTTTCTCACTAGCAAAATATCCTGTTAACTC
ACTTTTGACAGAGAGAAAATTTAGGAGTTGTAATATAAAGCTTGATCTAA
TGTATAAATTTCTCAGAAAACCCAAAATCAACATGGCTATTAATAGGAAA
TTCCAATCCAATGAGCCATAAGCTTTTCTTATATCTACCTTCGCAGCCAA
GTAGGATCACCAGGGTTGTGTAGTTGTGAATAACTTTTGATCCAACAAGT
TATTATCCCATATTCTCCATTGGACAAAAGCAGATTGAACTGGACTGATA
ATCTGCCCAAGCACTCATTTGCGTCTATTAGCCAGCAAACTAGATATACC
TTCATATAGAATGTTTCAGCAAGCAGTGGTCTAAGATTTGTTATTGTTGA
GGGGTTTTGAAATTTTGGTACAAGAGGCAACTAGGTGCTTTACTTCTCTT
AACAGTTTACGTTTATTAAAGAACTCCTTAACAACTTCACAAGTTTCATT
TCCAATGATCTCTCTTACCTTCTTGAAAAAATAGGATCCATATCTGTCAG
AACCTGGGGAGTTGCCATAATCAAAGTTGAAATATACATCCTTACTTTCC
TCAGTATTCACAGTCCTCAATAAGTCAGCTTTCTGATTCTGATTTATGCT
TTTTTGAACGATTTCATGTATAGCACTCAGATCATTACCCACCGTATTCC
TTGTCCCAAGAGATTTTGGAGAAAAATGACCCTGGGGAAAAAGGACCGCT
TCCTGTTTAAGTGCATCTGGCCCTTCCAGTTTAGTAACATCCTGAGTGTA
AATGCTCAAATTTTTTCTTCATTGATATAGTTGTTGCATGTCCTTTAGCA
AAAAAGAATATGTAATTGTATCCCTCCACTCCAGCCATCTGATTCTATAT

TTTAATTTGTAGGTTTTCTCCTCCAGGTTTCAAAGCTTAATCATAGGTCC
GTAACAATTAATTCTCCATTCTCAAGAAATCAACACACACTCCATCTTGT
TGCAATTTCCCAAAACCAGTCTAGATGTTTTAACTCATTCAGGGACACCA
CCCACCTTATCTTTGTATATCTTCTTTGGTTTCTCTTTTAACATTCAGTT
TATTTGCAACCTGAAACATTTTGACTCTTTGTACCTCAGTTTGCAAAACC
TTTACCACTGTAGGATGAAAGTTAATCAAATTCACCCATAATGCTAAAAA
CCTTGGAAGGGGGTTTCCTTCTACTACCACCACCACCAATACTAATCAGT
GCAGGGGAATGAGCAGAGACATTAGGAAGTATATAATCGGCAAAGAAATT
ACGGAAAGACAAGTTTTACTTCCAGTTGCAAGAAATTACGGAAAAAATATG
CCCCTTTAGCCTTAGATCCACCAACTCAGCTTTTCGCAAATATTCTTTAA
ACTCTTCATTCGAATAATTTCAATTTTCCCCCCTGCCGTAATACTAGTAA
TTGCATCAAAGGCAGTCTGATCAATCAGCGGTCGCCAAAGAGATTTTCTC
ACCCTCAGCACCAGAATTATAAACTACAATCACCCCAAATTCAGTCTTAT
TCTGAATAGATAAGACAAGCTTGAACAATTTGGCCAGTTTGGATGAAATT
GTCCATTCGAAACTTTTGGGGATTCCATGTAAGCAGGATCCTCCCAAAGC
GTTGGAATTATCTCGTCTATCACTCCCAATTAGGGGCTTTACCCAAATTA
GGAAGTTCAACTCTAGTTTTAATAATACCACAACTATCCACTTTATGTTC
AAAGAGAAATTCCTTAATAACTAATTATTTACGGGGGGTCGTTTGGACCC
GTAATGTTCCAAGAAGGCAAGAAGCTGTGTAACTCCTTTAGAACCTTTAG
AAGTAGGAGTGTCCTCGGGCCTTCTCTCTTTCCCATTAACATTCTCATCT
CCGTCATACTTTTGGAACATGTTTCTTGTTTTCCGCCCAACATTATGAAC
CATAAATAATCACAGATCATGTATGAATTTGAAATTATCTACAACTTCAG
TTTGTGCTCAGGTACCGAGTTAGAAGATAATCAAGATTAAATTGATTAGG
GAGAAGACGAGAAGCAAGGAGGAGAGAAGAATTAGATGAAATGGTTGTAG
AATGAATCTCCAAAATACATATTTACTTTAATTCATAAAACCCTAAAATA
CACATTGTCATGAATACCTTTATAAGTAGTAAAACGCCTAACAAAACATG
GGGAAGCTGGAATTGGTCAAGGAACCATGTTACTACAGTTTCCTTGTTCA
GTTAGTGTGAATTTCAATTAGTATGAATGTTAGAATGGATTTCAAACAAC
TCATTTAACCATGCCGTTTCATACCCTTCTATATGAGTCCTGTGTCTCCT
GTCTAGTAAATTACTCTTCTAGAACTATATATCTAATGCAACTTTTAAAT
TCAAGCTATTTGTAGAATGCCACATCCATTCATTTCAAATCTTTCAGTCC
ATATGAAATTATAACATTCCACATTCTGCAATAAAAAGGATGGTTCATCA
CCACTACTGGATCCTAATCTATCATCTACCTTTTTCTTTTTCCTATGTTA
CTTTCTTATTTGTTCTCTTAATAACAAATGTGATTTTCCAAGTTTGCCTA
TTCATTGCTGAAGGATTCAATTTTGGCCTTGGTATTTGTAATCTCTCCTG
AAAGCCAAAAGTCACCTATATTAAGGCATGCTTTTTTTTTTCTTTAGGAC
TTGAATGTTGAATGATCTAATGCGCTGATCCTCCAAATATTCTTCCTCTT
TCTTTTTGTATATCTCTAAGTTAGTCATAATGAGTTTAGGAGTATGGACT
TGGTTTCAGCATCATTACTCTGAGATATTTGTATTTGTTGTTTCATCACA
ATTTCTTAAGCTCTTCGGCCTCACCTTAGTATTGACTGAGTTTTTTTTTT
TGCTTTTTTTATTTGAACGGTTACTTGGCATCACTAGTCTCACATTCGGG
AGCTTGAGGAAGAAGTTAAGCTTCTTCAGAATCTATCTCATCCGAATATT
GTCGTAAGTAACTATGTTCATTTGAGTGAATTTGTGGCATCTATTACTTT
TTTTTTTTTAATAACCATAGCTATCATCTTCTGGTTTCATAGAGGTATTT

GGGTACTGTGAGAGAGGCGGAAACTTTGAATATTCTTTTGGAGTTTGTTC
CTGGAGGATCAATATCATCTCTTCTAGGGAAATTTGGATCTTTCCCCGAG
GCTGTAAGTTGTTCTACTCTTCGTATTGCATTTTGGGGTTCTGATACTCG
TCTTATGTAAGATAAAATTTTGTTAAGCAGGTTATAAGAATGTATACAAA
GCAATTGCTATTGGGGTTGGATTATCTCCACAAGAATGGAATCATTCACA
GGGACATTAAGGTATATTTAATGTCAGTCATTAACTCATTTGATGTTAAA
ATAGTGAGTCAGCAATAATGCTTGTATTTCATTTTTGTTCAGGGGGCAAA
CATCCTTGTTGATAATAAAGGTTGCATTAAACTTGCAGATTTTGGTGCAT
CCAAACAGGTTGTTGAGCTGGTACGTGCATTGTCTATTCATTGCTTCCAA
ATTTATTTGTTGGGTTTGTGTTGGGAATTAATAGAAATATTTTCTTATAT
TATTCGTCTAACTGAGATGTGGCTACCTTAGGCCACTATTTCAGGTGCCA
AGTCTATGAAGGGTACACCGTACTGGATGGCTCCTGAAGTCATTCTCCAG
ACTGGTCATAGTTTGTAAGTACAAGAACCCTCTCCCTTCTTTGTGACTGC
ATTTCACTAATGTTTTTGTAGAACTTCAGAATTTAACATGCTACCTTTTC
GTAGACAATTGGACTCTTAGACTACACATACTTTAGAAGGGTCTCGAAGA
CCACAGGTTTTCAGCCCCTTAGCTTACCTCGTTTGTTAGATTTCAAGATC
CAAGCCTTTTGAGATATATTATGCTTACCATCTTTAATAAAAATGGTTTG
CTGCTCAATTCCGATTGATTTTGGTTTAATTTCCTTTTACCCCTATGTAC
TTTGAAGTTGTATCACTTAATTGGTTAATCCCCAAATTTTTTATTATACA
TTTTATCCCCTTCCACTCTTGATTGAAGTGTGAAAATAACAATTTTTGTC
AAAATTAATAGAAAAACTATGACGTGCTAAAATAATAATGATATGAAGTT
ACCCTCATCCTTTTATTATTTTTTTAATCATTTTACATAGTTTAATGGAC
GATAAAATAATAAAAAAAAATTTATAGATTGATCTGTCATGCCCCCAAACC
CAATATAGAGACGAGTAATGATAATCCCCGCATAATCATTACTTGCTCTA
TGATTATGCAAGGCTAATTCTGTAGAATAATAGTAACTCACTAATAAAAA
CAATCTTCGAAATTACTGACATTAATCAGGCCAACATTCTTCGCTTATAA
TATTCCAACTAAATCAATAGAAGAGTTGATAGTTATTTTTTATTTTAAAA
TCATGTCCACGAAATAAAATACAACATATTGTCTAAAATTGCAATAAGCA
AATAATTGTTATAAAAGAAAAATAGAATTCCTCGAACTAAATAATTTCAT
TGAATTTGGTTGTCTCCACGAATTTAATACTCCAAATCTGAAAAGATATA
ATTGTCTTTGGGTCGGCTCCCAAATGGTAAGTAATCAATTGACCTAACTA
GGTGGAACAGCCGAAGAAGCATGCTAAAAAATAATAGTTATCAATAATTT
GAACAATATAAATTTTTTTCTAATCAAGCTGATGATTCAAATGATCAAAT
ATAAATAATTTAGTCAATTAAGGTTCTAATAAAAAGATATTGTAGAAAAA
TAGAATTCAAAATTTAGATAGACTATGGCGACAATAATCCTTGTGAGAGA
GTGGATAACTCAGAAATCACAATACAACTGAGTCAATAATTCAGAACATG
TGTTAATCCCTACCGGTAGGTGCAAAATGTCAGAATGTCCATGTTTAACC
TTCAAGGACAGGGTTCAGATCATAGTCAGGCTAAAAGTTAATATTATAAA
GTTTTGTTTAGTCAACATTTCTATGTAACCAAAAGTCATAATACAGTAGA
TTTCAGACAAGTTCATGCTATTCCGATTTTCAAAACATAATTATTTCAAG
TTGTTAAAATGGTATTTCTGTCTTTCCTATATTATTTAGGGTTTCCTATA
CAATTTAGGGTTTGTTGTATTGTTTTTTCGTTTTCTATATAAATAGAGAG
AAAGGCTACCACAATGGTTTAAGCCTTCTCATTCTTTTCATATGACCTTT
TCCACTTCAATATGGTATCCAGAGCAGGTTATGAGAGATTGATTCCATAG

ATCTATTCAAACCAATTTTTTTTTTCTTTTCTTTCCCCTGCCTACTGTTC
ATCAAAAAAAAAAAACCCAAAACAAACTGAAACCCTAGCAGCCCTTGCCCC
CGCCCATCAAACACTAAGACTCGCCGCCGTCGTCCAGGTCGTTTCGACCC
TCCGCCACCTATCGTCGTCCCCAAAAATCGAGCCGTACTTTCCTTGCGAC
CATCATAACGAACCAGATCCCATTCGCGGTCTACCATCGCCATTGTCCAC
GTTGCCGCCGTCTCCTATGCCTTCGTCGACCATCACCGACGACTCGTCGG
CCGTTGTTTGCTCGAAATCGACCCTCACGGCCTTCTGTTTGCCAGCAAAA
TCGTGTTGTTCGCCTTCTGTTCTGTTCGTCGGCCTTCTGGGTCCTGTTCG
CATGACCTTATGGGTCTTGTTCGTAAGTAATGGCTCAGCTTGTCCCGATT
TGAGAGTATTCTGGCCAGATCTGATCACGTATTGTTCGTAAGTAGTGATT
GTTCGATTGTCCAGCTTTGAGACATTATTCTGGCTCTGATCTAATGTCCC
GAATGTTCGTGAGTACTGTTCTGCTTATAATGTTCTTCATTTAAGCCCTA
CAGTTGTTTGTTGTTGATTTGTTTCCCTTGTGCAAGTTGTTTGTTAGTTG
TTGTATTGGAGGGTACTAATGTCAATGACTACACCTCAACCATTAACAAC
CTGTAAGTTGAACAAAACCAATTATTTAAATTGGTCTAAGGCTGTGAAAA
TAGCCCTCACTGGACAAGGGAAACATCGTCACCTCACGGAATCCGCCCCG
GCAACAGATGATGAAAAATATGACGATTGGATTTAAGCTAATGCACAAAT
TGTAGCATCGTTGTGGAATTCGATGGAGCCTCAAGTTGTTGATCTCTGTA
CTCATCTCGATACATGCAAAGATATATGGGAGTATCTTCATGTATTATAT
TCCAGTAATCTTACCAGGATGAATGATTTATCGGTAGAGTATTTCCAACT
TCAGCAAGGCACGCAATTCGTTACAGATTATTTTGCCACATTCAACAGCC
TCTATGAGGAGATAAATACTATCCTACCGATGACTATTGATCTGAAAGAA
ATGCAAAAGCAACGTGAGTAGATGGCTGTCATGAAGTTCTTGGCTGGATT
ACGCCCAGAATTTGAACCAATCCGATTTCAAATCCTTTCCAGTGCTACTA
TTCTTACTATCACAGAAATGTATTCTCGTGTCTTGCGTAGCATTACCAAG
GACAATGTGTCTAGCTTGAGTTCGTTTGCGTCGTCTGAGCATTCAACGCT
TGTTACTCAGATTTCTATACGAGATCGTGGTGTTCCACTTAGTGGTGGTG
GTGGTGGTTTTTCTTGTGGTAGTGGTAGTTTCTCTCGTGGTGGGGGCCCT
CGGGGTAGACGTAGTGGTTGTGGTCGTGACTCACATCGACAGTGCTCTTT
TTGCGGAAAGGATAATCATACTTGAGAAACATGTTGGGATTTAGTTGGCC
GTCCTCCCAAATTCGCTAATGCAGTGACATCTGATCCAAATATTCCTACT
ATTCCATAATCTCAGGCGGGAAAGGACATCTCTGATGATGAATATGCTCA
ATTCCTTCAGTATAAGGTATCCCAGCAGACATCTCTTCCCATTGCTTCTT
TAGCTTAATGAGGTACTTCTACTGCCTGCCTTTCAATTCGCCCTTCCCCT
CCCACACCTTGGGTCATAGATTCTGCTGCTACTGATCACATGTTAGGTAC
TTCTTCCTTCTTTACACGTATTTAGTCATCTTCTTCGTTGTCTCCCGTTA
CAGTTGCTGATGGTTCCACTGTCCCTATCCATGGCATTGGCACAGTGGCA
CCCTCACCCTTCTTAGCTCTATCATCTGTCTTGTATATTCTTTCGTTTTC
TTTTAATTTAATGTCAGTTAGTAAGCTTACCAAACACCTTAATTGTTTTG
TCTCATTTTTTCCTGATTCTGTCATTCTCTAGGATTTGAGGACACGGAAG
ACGATTGGCACCAGGTGTGAGGAAAATGGCCTATATTACTTTGATGACCA
TGACGAGAGTCCTGCTATCCTACCTACTACCTGCCCTGTTGTTGCCTCTG
CTCACTAGATCCATTGTCACCTTGGTCATCCCTGCCTTGGCAACCTCAAG
TTACTTGTTCCTAATTTGCGTCATTTGTCTTCGTTAGAGTGTGAGTCTTG

TCAATTGGGTAAGCATCATCGTATTTCATTTGTTTCCCTAGCTCTTGTTC
GAGCATCGAGTTCGTTTAGTTTAGTTCATTAAGATGTTTGAGGTCCTTCT
CGTGTTACTTCTACATTAGGTTTTCATTATTTTGTGACATTTGTGGATGA
TTTTTCCAGAGTTACTTGATTTTATTTAATGAAAGATTGTTCAAAATTAT
TCTCTATTTTTCGTGCGTTTTGTGCTGAAATTAAAACTCAATTTAATGTA
TCAGTTCGTATTCTTCGAAGTGATAATGCTCGAGAATACTTTTTTCAGCC
ATTTACTTCATTTATGTCACACTCTGGTATCTTGCATCAGTCTTCTTGTT
CTTATACACCGCAGCAAAATGAAGTTGTTGAGCGTAAGAATCGACATCTC
CTAGAAGTGGCTCGTATTCTTCTTTTTGAGATGAATGTTCCCAAACATTT
ATGGGGCAATGCAGTTCTTACCGCCTGCTTCTTAATCAACAGGATGCCGT
CCTCTACCCTCAAAGGCAAGGTTTCCCATTCCATTCTTTTTCCTCGTTCT
TCCTTGTTTAGTTTACCTCCTCAAATATTTGGGTGTGTTTGTTTTGTTCA
TCAAATGACTCCTGGCGAGGACAAGTTAGATCCCCGTGCTTTAAAATGTG
TCTTCCTTGGGTATTCTTGCACAGAAAAGGGTTTATGTTGTTATAGTCCA
TCATTACGTCGCTCGTTTGTGTGTGCGGATGTTACATTCTTGGAGTCTAC
CCCTTATTTTTCTGCCCCTTTCTCTTGAGACGATCTGGATGTTACGCTTC
CCTTGCCAAGTGTACCAAACCCTACACTATCTTCCTCTACTCCGGATCCT
ATACCGCCTAGTCGTTTTTCAGCTCCTTTGCAGGTCTACTCTCGTTGTCA
GAAGGCCCCCTTTGCTGTCGCGCCCATCGACTCTGTGTCTTCAGATTCTT
CTCCAACCGACCTATCTGTCTCTGACCTGGACATGTCCATTGCCATCCGG
AAAGGTAAGCGCACATATACTACCCATCCTATTTCTAACTTTGTTTTGTA
TGCACACCTATCTCTTTCGTACTCCTCTTTTGTTTCTTCTCTGTCCTCTG
TTTCTATTCCTAATTCTGTTTCAGAGACACTCACTCACCCTGGTTGGGCA
GAAGTAATGGAAGATTTAGTGAAGTGGTCACTGAGTTTGGTCTTCAACGA
TGTGGAGTCGATTATTCCGTCTTCTACAGGCATAGTAAAGTCGGGAGAAT
ATTGTTGATTGTCTATTTGGATGATATCGTGGTTACTGGGGATGACCACG
GTAGTATTCAGGAACTCAAAATTTTTTTGAATAGTAAGTTTCAAACGAAA
GATTTGGGTTACTTAAAGTACTTCTTGGGAATTGAAGTCCCTTGACCTAT
TGAGCGAAACAAAAATGCTAGGAGTTAAACTGATTGACTCGCCTATGGAT
CCAAACACCAAACTGTTAACAGATCAAGGAGTACCACTGACAGATTTAGC
ACAATATAGACGGTTAGTTGAGAAATTGGACTAACTGACAGTTACCAGAC
CAAATATCTCATTTGCTGTTAGTGTTATCAGTCAGTTCTTAGATCGTCTA
ATGACTTGTCACTGGGATGCAGTGATCCGTATTCTCCGATATTTGAAAGG
TGCTATTGGTTGTAGTTTGCTATATCAAAACTTCGGTCATACTCAAATAA
AGGGCTACACAGATGTAGATTGGGCCGGATCGCCCTCGGATAGGAAGTCT
ACTACTGGATATTGTGTGTTCGTTGTTGGTAACCTTGTCTCTTGGAAGAG
TAAAAAGCAGATTGTGGTGGCACGATCAAGTGCAGAATCGAAATACAGGG
CAATGACCCATAAACCTTGTGAGTTAACTTAGTTGAAACACTTGTTGGAA
GAACTTGAGTTTGAATACTCTTCACCGATGGATCTGGTATGTGATAATCA
AACAACACTTTACATTGCTTTCAACCCAGTCTTCCATGAGAGAACGAAAC
ATGTTGAAATTGATTGTGATTTCATTCGAGAAAAAATGTTACAGAAGCTG
ATTGAAACTAAACATGTAAATTCAGTTGATCAGTTAATTGATATATTTAC
TAAACCGTTGGGGGGTTCAAGGATAAAATACATTTGTAATAAGTTAGGAG
CATACAATATATATGCTCCAACTTGAAGGGGAGTGTTAAAAGGGTATTTC

TGTCTTTCCTGTATTATTTAGGGTTTTCTATACGGATTAGGGTTTTTTGT
ATTGTTTATTCGTTTTCTATATAAATAGAGGGAGTGGCTACCACGATGGT
TTAAGCCTTCTCATTCTTTTCATATCAACTTTTCAACTTCAACACAAGTA
TATCAGAAATGAATATAATCGATGATTTCAATAAATATGTGATTTTAATG
GAACAAATCAATTTTTTTTACATAATATTTTAATATTTTTATTTTTAAAT
AGAAAGTTAGAAGTAAATTTCAAATGATACATTTCAAAATTATCAATTAC
GAAAGAAAGGGGTAAAACACTTGCATTTAATGTTTTCATAATTTTTCAAA
TAACTAGAAAGGTAATCAATTACTTACCTCGATTCCACAACAAATTTTGT
CAATTGATCAATCAACAAATCTTTTCATGATCCTATTAATCGATAAAATT
AACTATTAGAATGTATCCCTCAGGATCCTAACTTAATTATTTTGGGCCCA
AAGAAACAAAAAACCCAAATCTAATTCTAATCTATCTATCTATCAATAAA
TAGTGATGGAAAGACGTTTTAAAGGTTCACATTTAAATTTAAAAGATGAA
TGGTTGAGATTGAACTTGTACAACCTCTCAACTCACTAACTTCAAATTTC
TCTACACCACTCACCCCACTCAACCCACTTAATGGCTTACTTATATTTTG
TACCATTCAGATTTCATATATGTACTTTATTAACTAATTTTTTATTATTT
ATTATTTTTTTACATTTATAAATTTATGGATTAATTACATTTTACACCCC
TAAAGTTGGACCAAATTGCACTTTGTAACCCTGTGGTATATATGATAGCA
CTTACACCCCATACTTCAAACTTAAGTGCAACAGTCAATTATTCCATTAA
CTTTTAACGGTCAAAGGGAAAAAGTCAACATTATTTTGTTAATTACTATT
AAATGACATTTATACCCTTCAAATTAATAAATCACATTCAATTAATTTTA
TGGGGATGATTTAGAAATACTAAAATTTTAAGGGTGCAAAATGCAATTTG
ACACAATGTACATCATCCCTTTCCTCTTCCTCTAAAAATGAACCAACCAC
CACTACTCTATTTTCTTTCACCATGGAACCAACTAACCATAACCGTTGTT
CCAACGACCTCTTTCTAGCTATTTTTCTCTATCTCTTCTCTCATTTGCTC
TCTCTGCATATATATACATAGAAAAAAAGATCTAAGCTTTAGATCTTTAG
ACCTAAGTGCGGATCTAACAGATCATTGCAAGGATCTGTGTTGGTGGACT
TGCTTAGATCCTTTCAAAGGAGAGCGAGAAAGGAGAGTGAGAGAGATATG
TACCTGAGATTGAAGAAATTGTTTGATTTCATCGTGTGGCTGTTTGATTT
GGTATGCCAAATCTTTGGATAACAGAGGCAATAGAACTGTAGGAAATCAA
TGGGGCTTAACTTACTCTCAAAAGACGCCTTGTAAGAGAAGGATTTCCTT
AGGCTTTATACATAGCTAAAGATCAGGTAACACAAGCGATGTGGGATTCA
ACACGCCCTTTCATGCGCAGGGTGGACTTTGACAAACCATTACGAATGGA
TATCAGGGGGCCTAACATCAGTGTGGGATTTAACAGCCCCCTCATGCGCA
GGGTGAACTTCGAGTGAATTTCGACAAATCATTGCGAACGGATATCGGGC
GACCCAACATTGATGTGGGATTCAACACGCCCAATATTAGACAAAAGGTC
TTGCTCTGATATGTAGGAAACCAATGGGGTCTCACTCTCAAAAGATGCCT
TGTAAGTGAGGGATTGCCTTAAACTTTATATATAGCTAAGGATCATGTAA
GACAAGCGATGTGGGATTCAACAAGAACGGAGGATTGAGAAGAGAGGCGC
TATTGATGTTGGTCGCCAAAAGCTAACCATGGACCGGTAGAGACGAGATT
TTTGGGTGGTGGAATAAAGGAGCTGAGCCAAGTCTATGAGCCGAGACTGA
ATCGAGACAAATGGATTCGTGATCATTGTTCCTCCTCCAGTTGTAAGCTT
TCTTGATCTCTTTCATGGATTATTCGCAATTGCTTTTTCAATAATTCAAT
TGGTTCATGAATCAAAAAATTTATGTAGATAGAGAAAAATCAATACGAAA
GTCATTTTCAAACCTCCATTGTTGAATATCATATGGGGTTGGTCAAGACA

TCCTGCACCTCCTCCTCCAGTTGGTTGTATCTATGTTGACTAATCGTTGG
CGAGTGGGTTAGCCAGAAAAATGCAGACGAAGGAGATCAAATGAAGGAGA
AAAAAATTATTTTTGACCTTTAATATTTAGATGACTTTTTAAATAGATCC
CATTTAATTAGTTAGAGAAAAATGACAAAGTTATCTTAATTTTGGAGTGA
AATGACACAAGTTCTGTTAAATTTTTAGCTGTTGTAATTAAGTCGTAAGC
ACAACGAGTGTAAGTGTTGACAAAAATAACACAGGGGTGCAAAGGGACGA
TGAAGAGGTGGTGAAATGAAGTTGTTTCCCTTATCCATTGAGGGTCGCTT
TGATGACATTGGGCCACTAGATATAATTACTCCTATTCAACTTATTAACG
GTTAACAATAGAAGTACTATCGAAAACATAACTTAGAATCAAACAAAGAA
CAACTAATAAACCGTGAGATTATAATGGGAGATAGACTAACTACAATAAA
ACATGTGACATGGGAAACAAACGAACATGAACACTAATAGAACTTAAAGG
AAAGACATAATTTTTTTTAAAAAAATCGGTGTTTTTTTTATTATGTATTA
CTTATGTTTTTTTTTTTTGAGTAAAAAACAAGTAAAAAAAAAAAAAAGAC
TTAGCTTGTGGGGTTCAAATAACCCTTCAGGATCGGTAAACGGCGGAAGG
GGTAGTGAATGGTGAGAGTGGCAACAAACGTTATGGTCTTTGGTGTTTGG
AGGTGGCCAGAGTAAGGTGGATGCTTGTTGTCGGCGTTCGAAGGTGGCTA
GAGCAAGGCGGGCTCGATCTGGGTCTTTTCTAGCTAGTGAAGTTCACTCG
GAGGTGACAAAGAGGTGCTTCGGCGACGACGAGGGTTGGTGGAGGGGTAC
AAAGCTTCAAAAGGGTTGGTGGCTAGCTACGAAGCTTCGGTCAGGCATGA
TCAGAGCAGAGGTGATGGCTAGGGTTCGGTGGTAGGGAATTGTGGACAAA
GGTGGTGTCTTCTTCGATTTTTTTTCTATACGTTTGTTAAAAAAAATTGA
AGAAAAAAAAGAAAGAAAGAAAGAAAGAAAAATTAGTAGAATAAGATATT
GAATTAGTATCCCATTAGAACCTGTTCTTGATACCACGTTAAAGTAGAAA
AGAATGAGATAACTTGAACTAAGCAATCAATCTCTCCCTAATATAAAGAT
AGAGACTGTGGTGCTCTCTAATAGTTCTTTTTGTTAGCAGGTTTCAACCT
AAAACTATATGGTGTCAAGTAAAGTAACTCTTTTTATATATAGACTACTT
TGGGAAGACTCATAATTTCGATGTGGAATTTTAACACCCACCCTCACGTG
CAACCTTGCCTTGCACGTGATGCAAACAAACAAACAAAAGAAAAAAAGCA
AACATTCAAACAATCGGGTACAAACAAACGATACTTCCCAAACCTAGCTT
TGATACCATGTTAGGACGTTTTAACTTAAAACTCTATGGTACCAAGTGGA
GTTAACTCCTTTCATATATAGAGTACTTTGGGAACCCACACAAATTTGAT
GAGGAATTCTAACACCCACCCTCACGTGTAACCCGGCCTTGCACGTGATG
CAATCAAACAAACACAAAAAAAAAAAAAAAAAAAAANAAAAAAAAAAAAAA
AAAAAAAAAAAAAAAAGAAAGAAAGAAAGAGAAAAGACATATTCAAACAA
TTGGGGACAAACAAATGAGACTTTCCAAACCTAACTTTGATACCATGTTA
GGAGGTTCCAACTTAAAAACCATAATGTACCAAGTGGAGTTAACTCCTTC
CATATATAGAGTACTTTGGGAAGCTTCACAAATTCGATGTGAGATTTTAA
AATCTTACAAATTCGATGTGGGATTCTAACACCCACCCTCATATGCAACT
CCGCCTTGCACGTGATGCAATCAAACAAACGGAAGAGAAATGACAGATAT
TCAAACAATTGGGGACAAACACAAGAGGCTTCCCAAACCTAGTTTTGATA
CCATATGAGGAGGTTCTAACCTAAAACCATATGGTCTTAAGCAGAGTAAC
TCCTTTCATTTATAGATACTTTGAGAAACCTCACAAATTCGATGTGGTAT
TGATTCTAACACCTTTGTTGTCGGATTTATGCTTGACTGACACTTGCATG
GCTGCTTTCTTCACATTGAATCTGATCTTTGTTGACCAAAAAGGAAACAA

AAGGGGGAAGGGTATATGAAGAGATTTCCTAGTAATGAAAGGAGAGACGT
GAATATGCCTTAAAGGAGAAGCTATCATTTTCATTTATCCTTTTGGTCTA
CTGTAGGTTCGCAGCTTCATTCATCAACAGGGACATAGCTAAAAATTCTT
TGTAGAGAGGACACAATATTCTTTTTTTTTTTTTTTTTTAAAAACAAAA
ACAAATGTTGTATAATAGTATCAGTATTCAAATCATTTAATATTTTATAC
AATTAAGTTCAATTGGTTTTTGTAGGCAACTATTCATAATTTCCAAAATG
AATTTTTTACTTTAACTTGGTAGTGGCTCTGGTCACAAGTCTGAACTCAC
AAAAAGAAAAATGAGTGTAAATATTTGGGTTTCCAAAATGAATTGGCCGA
TGGAAGGGAGAGGAAAAATGAGTTTAAATATTTGGGTTATTTAAGTAAAT
TTGGAAAAAGGTAAAAAAGTATGCAATTTTAAGGGTCAAATAAGAAATTT
TAATATTAACTAAAGTGAAATTAATTTTTTAGTAGTGATGTTATAACAAT
GAACATATTTGTAGACGCAGGTTATTAATAACATATTTGAAAAATCCAGT
AGGGTTCCAGCCCTGAGCCTTATGGTTCCATAACCCTGTTTATGAGGTCC
TTAAATTTCTTTACTGGTTAACCTATGTAGTTTCACTCACGAACCAATGA
AATTTGTTGCTTTCTCTGGGTGATATATAATTTTTTGTTCATTTTTTTAA
TCTTCCACTATATGATGTCTTTGTTATTTTTTTTTCCTCTTAGCTCTGCT
GACATATGGAGTGTTGGATGCACTGTTATTGAGATGGCTACAGGCAAACC
ACCATGGAGTGAACAGTATAAGGAGGTACTTAGATAGTTCTGCCATGATT
ATCCAAACAGAGAGAGAGAGAGGTGTCATTTTCCACTTATTTTTTGGCCAGG
CGACATTGCTTTAGTTAAGAATTGAGCCTTCTTGAGTTCATAGGTATTTT
TTTCCTTTGCATCACGCTAACGACAGGCTGCTGCTAGGTTGCTGCTCTCT
TTTATATTGGGAAGACCAAGTCTCATCCACCAATTCCTGAGCATCTCTCT
GCGGAGGCAAAAGAATTTCTGCTGAAATGTTTACAGAAGTAAACATACTG
AACCACTTCTATTATTCCTTTTGCCTCATGAGATTATATAAGATGGAATC
TGTAAATGGCTTTAAAAATATCGTATTTAAACGCATTCCTCTCTAGAAAG
TTTCATGATGCAGTGACTCTGATGTTCAAACTATAAGCATGAAATGAGCT
TTGTTACTATAGTATGTATAACTCTTGGAATTTTTGGTGTTAGTGATTCG
GTAGTCATAGCTTGTTAATAAATTGCAAGTGAAACTTTCTTTTTAACTAT
GTGACCTTTGGACTCTAAGTTTTATGTGGGTTTTATTTCATATTTGCTTT
CACTGAGTACACAAAAATTTGTACTGTTTTTGTACTATTTTATATGAACC
TACAGTCGTGATATGCAGGGAACCAGAGTCAAGACCCCCAGCATTTGAGT
TGCTGCAGGTGCTTCTGATTTCAATCAATACGCTGTAAATAATTTTTTTA
TTTTATTTTTTATTTTTTTGAATTCACTCTTGCATGACATTTGGTGTGTT
CTAATCAATCATATGCAGCATCCTTTTGTTACCGGGGAATCCATGCCTTC
TCCTCTTGTTCATTCTTCATCCTGGGTTAGTCTTCTTACTCTTGGTAGAG
CAGCTGCAGGCTAGTCAAAATGCTTTTCAACATCCAATTACTTTTGCATC
ATTGTCAACATTAGCTTCGTTTGATTCTGCTGCAAGCAGGAATGCTCTAA
AACCCCCAATTCTTTGTGCGGTAAACATCTGGACAATATGTAAGTATTTA
AAAGCTGGTGCCTTCTTGAATAATCATAAAATTCTCGTGATGCTATGCTG
TTAAATTTTGTTTCCTTTTCCAATTAATGATGACCAGGTCTAGCTCGATT
GATGTCTGTAATTTGGGCACTCTGAATTTCTCCAGTGTAAATACTGAGAA
ATTATCAGAAAGTAGATATATTTCTGAAGTAAATAAGAGTGATGATTACA
TGTGTCAGATTGATGACAGTGATGACTTTATGGTGAGTGGAGAAACAAAG
CTCAGTTCTGTTTTAATAGCAAATAGCCTTAATAAGGCATGGAACTCCTT

GATTTCCTCTATGCTTTTGCACTTTTCAAGCATATTAGGCCTTTAACGAC
TAATTGTTTATGTATCATTTGATTTAATTAGAGTTATAACCCGATATGTG
AGTCCTCTGATGACTGGAAGTGCATTGAAAGTTCTGGAGTGGAACAAGAA
AGAATGGACACGGACAATGATCAACAAGTTGATATACTGGCCACTACCTC
TGGGTTATCCATGAAGGGTGAAAACAGTTTCTCACTTAATGGTGCTGCGT
CTCTGTCTGAGGATGAAGATGAGGTTACTGAGTCTAAAATTATAGCCTTT
CTGGATGAGAAGGTCTTCTCTTTCTTTCTCATTATTTTTCCCACATTTCA
ATAGCAAATTAGCCAACACCAATTACTGATTCTAAGTTTCCTTTTGCATG
GACTATTGTAAATTACTTCTTTCTTATTCTACCTTAGGCTCTAGAACTAA
AGAAATTGCAAACACCTCTTTATGAAGAGTTCTACAACAGCTTGAATGCT
TCATGCTCCCCGAATTTGGCTGAGACTCTGCATGATGGAAGTGCTCTGAA
TTACTTGAGATTACCTCCTAAAAGTAGGTCACCTAATCGTGCTGCCGTCG
GAAGTCCTTCTAAAGCAGTTGATGCTACTCATGCAAGCCCTGGAAGGAAT
AACAAGCACATTTCAAATGTTGGCAGTGCAAGTAACCACACAATTCAGGA
CAAAACATCACCTCAGCCTAATGATTCGGAAGGAGTTCTAGTAGATTCTG
AGCAGGAACCAGATAGCCCGAGGTTTGTCCGTGAATACTGCTTCTTTAAG
TTAGTTCCTATATGTGTAATCTACTAGATTTAGTGCCTAAATATAAGTGT
GCTACGTACAGTGCTAGTTTTTCCGATATACAGAGAAAGTGGAAAGAAGA
GCTTGTTCAAGAGCTCGAGAGAAAGCGAGGTGTGGTTTTTTTACTCTTGT
AATTTGAATAAAGTCACCCTCAAATTTTATATTGTCTCTTTTTTTCGCT
CTTTATTTTTTGGGCTCTAACTTGTAGTTGTTCCTTATCTAGTCCTGATT
CCCTGACAGTTCGTTTGGATGGAGGGGAGGAAAGTGGAGGGGAGAGAAGG
GTGAAGAGGGTTGGGTAGATGGAAGAAGATTGTCTTTCCTTCATTGTGGT
GGATTTGTAGGGAGAGATTCACTTTCCATTGTGTAATATGACTATTATTC
CTTTTATCTATATTTATGTATGTAGAAGATTAGTAAAAGGTAATTAGTAG
TAAAATAAATTAAATAAAGGTGATATAATAATTAATAAGATTAGAGTAAA
AGATTGGAAGGAAAGTGCATCCTTACAAAATATTTCCTCCTAAACTACAC
TGGACGTCTGAAACAACGTTAGATGCATTAAATTTTTCCAAACAAATTCG
TGGAACCTCTGACCTTAAGTAGATATTCCGTGATTTGATTATATTTGTGT
TTTTTGTAGCCTTCTCTCTATCTCTAACTTGTATGTGTTGGGAAATTGTC
ATGCAATTGTTTCATGGCTTTCTTCTCAAGTTTACTTTGGTTCTGAGGAT
TATGCAAATGGTTATGCTATATTATTGAGTTGTCGGGTAATAGAATTAAG
CAACGTGTTCGACTATGTTCTAGACTGCCATAGTTTTTACTATTTCCTTG
CTTTTAATTTTTAGAAAAATCCTTTTCGTACTAGATTAGTTCAATTTTTT
TTTATCTGAAGTTATCCTGAAACTTGAGATCCAAATATGTTGTGGAATAG
ATATTTTGAATGCATAAAAGATGTAGCACTAAAAACTTGAGTATGGGACT
TGCATTTTTTCTTTTGGCTGGTTGGCGCCTTTCTAGGTTCATGAGTTCTA
ACACAGTTTTGTAGGTTCTTTGTTTCTTCTCTCTCCCTCTCTCTCTCTCT
GCCTTCTTTTTCCTTTTTTGGCTTTCCCAATTTGCATGGTGACATGTTTT
TTAACTTATAAAAAGGTTTTTTGGAACTGTATTAAGTATTTAAATTCATA
ATGCAAAATGGTGGGTGTAGAGATGATGCGCCAGGCTACTACAGGCGGGA
AAACATCATCTCCAAAGGATCCATCTTTGAACAGGAACAGAGAACTGTCG
AGGTTTGCATCTCCAGGCAAATAA
>EUC07535-RA [gene]

ATGGACGGCGACGATTCACAGATGAACTCATACAGAATCCTTCTGGATCG
ATTCCAGAGTTTGGAGACGAGTCTGGCAAGGCTCAGGGAGCAGCTCGATG
TGCTGGTGCAGCAAAGAGCTGTTAAAATGAATTTTCCGGGGAAAGGGACG
TCGGATTCCGGTGAAACAACTTCATACGACGGCTGGGAGTACGTTCCGCC
GGCGTTCTTTTCTAGTAGTCCGTACAGGAAGGTGTTGGATCACTTAGGTC
ATGCGGTGCACGTTAGCATACCCGAGTCTGGGGAGATCGTTTATTGGTAA
TTTGGTCCCCTTTCAGAGTTTGAATTAGACATAAGCTCTTTTATTGGTCT
TGTTAATCAGTAATGATTTTGTCGATTGCGTGATTAGTTCGTTATTTTGA
ATTGTTTTTAGTAGAATATTTACATGCAAATTTCGTCGAACACCTTTCGT
GCACTGTTGAATAGCGTTTCAATAGAATAGTTGTTTGTAAATTGTTATAG
AAACACCATCTTTGGACTTGTTGGATGAAGGGATTTATAATTCCTGTTTT
ACATGAACTTGTAATTGAAGATAGCTATAAAGGTAACTTACGATATAAAA
ACTTGCATGCTTCTCAAGATCAATGTAAATTCGTATCTCATGGGCGGGCA
TGTTAGATAAGATATTCAACGTGAGTTCTTATCATAATCTTGTTAAAGGT
CCTTCAATTTGAAACCTAAGGCTTGCGATAATATGAGTAATTAATGTAGT
CTGTGCTCTTGGTTTAAGGATTTAAGAATAATCCACTGCATTAATGATTA
GATGTTAATTTGGGTTTCCTAATCTAAAATGAAGTTTAAGTTTGTTGAAA
ATATGTCAATATTTCAAGTTCAGCCACGTAATGCCGTGAATCATAATCTA
TAATCTTAGATATGACTTGGGCATGAGTTAAAGCTTGAATTACTCAATCT
AAGGTGAGAGCACTTAAGTACTCTTTGGTAGCACAAAATTGAAGACTTCG
AATTCAAATTTGAAACAAACTAATGCAAAATTGTAGATTGTTACTCGTGT
TCATTTCTAATTGCAATTCCTAGGTTTCATCCTCAATCAAACCAAAGGTA
CACTAATCAGAAATGGTACATTTCACATTTACATATTGATCCTATGTCCT
TTATAGGAATCATTCTGCTGCAAAACTTTATGGGTACGATTACCATGAAG
TGCTGGGGCAGGGAGACGAAGAACTGCTCACTGACGAAGAACATTACAAC
TCTGCAAAGATGATCTTGCAAAGGTTGAGCTCTGGTCAACCTTGGTCCGG
TCAGTTCCCCTTTAGGAAGAAGTCGGGAGAGATATTTATGGCAATAGCAA
CCAAAACCCCATTATATGAAGATGGTGAGCTTGTCGGGATTGTCACTGTT
TCAAGTGATGCAGATGTATTTAACAGAATAAAGTCGGAAAACGTTGGCCA
ACCTAGAGCACGGAGAATAAATTTCAAAAAGTTTCAGTGCCACCCACAGC
AGCAAATTGCATCAGTGCCACAGATTGCTGCCTCTGTTTCCAATTTGGTA
CATATTTCTCTAGCTCCTTAGCGTGTTTGGATACGGAATTCAATAAGTTC
ATGGAGTTTAGAAATGATGGATTTCAAAATTTCGAATTTTAAAATCCCAT
TGTTTGGGTGTACTTTTTGAAATAAAAAATTTATTGATTTAATTTCACCT
CGAGATGAACAGATATACCTTATTCGAATGTATTTACAAAAATGCATCTT
CAGAAGGTATTTGGAAATTTCATTTTACCCCCCTTAATTCTCATTTCAAA
ATTCCAAGGAATTTTAAAATTTTCATTTTGAAGCAACCAAACGAGGAACT
TTAACTTAAGAATTTCAAAAATCCCTTATCGGAATCAAACCCTCTTCCAA
AATTCTTCACCCAAACACATCCTCATTTCAGTTTTCTGAGCAAGTCTTGC
ATACTTGCATTTCTAAATTATTCAGGCTTCAAAGGTCCTTTTGCGGAAAT
GTGGAGATGACACAAGCAACAATTGTACAACTTCTGGGGAAGAGGCAGAG
AGGAATGAAACCAATGTTAAAGACGTAAGATACGATAGGCCACCAAAAGC
ACCTGTAAGTTATAATTCCTTAGAGTAGCGTCAGTTTTACTTAAAAAGAA
AAGGGATTCATGAGGAAATTTATTGTCCCAGGCAACATATTCTGATTACG

GTTCGCACGTGGATAAGACCACAATTGATGCAGATTGCTCTGGAAAGAGA
GAGTCTACGCCTGAATTTGTTCAACCTTCTAGAATTGTATTTATCCTTGT
CTGATTTCACTTACTTTTGCGCTTTCCTATATTATATAGTCTTCAATTGA
GGCAACACTCAGTAACCCTTTTTTTAAAAAAAAAGAAAAAAGAAGAAGAA
ATACTTTTTATGATACAAAAAAGTTAATTGAACAAAAATGTGCTTCTACG
TAAAAGAAAAGTAAATAAAAAATGTATTCAATAACATATGGCACTATGGA
TGATATCTTAGCATTATCCATATAAAAGATCTCTTAATCTAAAATAATTC
TAGTAGAAAATTTCTAGTCCGGCAATCTCTTAATCTATATAATTAGAACA
TATAAAAAACAAGTATTTTTGCCTTTATAAAAAGTACTTCTACTTTCTTG
ATTTTTATTATCAATTTTTTTAAAGACACAAAACAAACACTATTTCTATT
TTTAAGTGTTTTTTTGCTTAAAGGCAAAAAAGTAAACCGTTGTCAAACGG
TGCCCTAGGTTTCAAGCTGTGTTGTAAAAGGCGGGGACGTTGGGCGGAGC
CTTTTGACCTGTCCACGGCCAGGCGTAAGCCCTGAGGCGTGGGGCTTAAG
CCCCACAATACAATTTTTTTTTAATATTTTATATAATTAAAAAAAAATGGA
AAATAAATACAAAAGATATATATGAAAATAAATATAGATAAAATTATAAG
ATGTCAAAATTTATGAATTATAAATAGTAATTTAATTGAAATTCTATTTT
TTCGGTGAGGCCGACCTATTTAGGGATGCTATTAAATATAATTCTTTATT
TTGTTATCTATTCTCTAACAAGTAACAATCTTCCTTGAATTCTGGTTAAA
GACTCAAAATTTTCTTTTATTAATTTTAAAAAATATAAATAAGAGGAGAC
AATTGCTTTATTTTCAAATAAAACATGAAAAAAAAAAATGCACAACTTAAC
CCTAGTTCTCATATTTCCGCCGCCATCGACCTTTGCTTCCTCATATGAAT
GATAACCTTTACTACCCCAATCCTTGGTTGCACCTTTAGCCTTCAAGGTC
TAGCACTGCCGAAGCGATGAAGACTAAAGTGAAGACAAATACATCAAACA
CGTGCAAAATCAACTTTGTCTCTCTTGATTTTCTGTTGATGGAAACACAG
GGGGTAAAGCCCTGTCAAACTTTGTCGGCGGCGAAGTTGGGCTTCGCCCA
CGGCATCTGGGGGCAAGCCATGCTGGAAAATCACCCTCCTCATCACCCCG
GGGCTTTTTAGGACTGGCCCGCCTCTAGGGGAGCCTCACGAATCCTCACG
AACGCCTTTTACAACGTTGGTTTCAAGAATTAGGTAGATAATAATTCAGA
ATGCAGAAATCAGTTAAAAAAAAACTGATTATTGAACATGTATTTAGGCGG
CGAAGATCTTATCAAAGCTGAATGTTAGGAGACTTGCAAACCTTGGAAAA
GAGAAGGATGGGGGCATTCAGAAGGATGATACTTCAATGCACAAAGAAAT
AGCAAATGAATCATATTCTGCAGCAGATTTAAATGCAATGGTTTCAGAAC
ACTGTACTGTTGATGTGGATAGAAGAACTCAACCTGCTTCTGAAAAGTCA
AATGCCACTGGAGAAGGTTTGGCTGGAACTTTTTGTTGTGATTGTTCTGA
GATTCCCAAACCTGCAGGTCAGTCGCCAAGATCTGAAATCTACTTGCATG
CAAACGAGTTCGGGACTGATTTACTGAATTCAAAACCTTCGGAGATTGAA
GATGCAGTGCAGCTTCCAAGCCCTGGGGAGGGCATTAATAGCAGCGGCAG
TTCATCAAGCAAAGGCGATAATGACTCTAATGTGATAGTAGATTGTGAAA
TTTTCTGGGAAGATCTACATTTGCGCGAAGAGATTGGGCAAGGTGCGATT
TGAGTAACACAGTATGATATTGACCACTGGGAAGTATATCAAGCTTACTT
TTTTTTTTTTTTTTTGGGGGTGGGGGGGGGGTTTGTTTGAAGTTTTATAA
GAAAAATTCTAAAAAAAAACTGTGGCACAGTTTGGTAACAATTTTTACCAT
ATTTTTTTATAATAAAAATGTTAATTAAACAAAAATGTAGGTTTACTAAA
AAAAAAATAAAAAAAAATATCTGATAACTTAGAGCTTGGCGTTATCTATAT

AAGTGATCTCTTAATTCAAAAAAAAAAATTGTAGAAGGTTGAGAAAATTTA
GGTAGAGAGGTCCTAGTGCGTCGATCTCTTAAAAAGTTAAAATATAACTA
TCGAATGATAAAAAACAATTATTTTTTATTTTTGGAATAAATACTGTTAC
TTTCTTGATTTTTTAAAGTACTTTTTTAAAAACGCAAAACAAATGATACT
TTTAATTTTAAGTACTTGTTGTTTAAAAGTGAAAAAAAATAACTTCAAAAA
GCGTTATCGAATGGTGCCTTTATCTTATATAGAATCTGGTTCAGCTTTAA
CTGTATGTTTTTGGATCTATGGAACTCGTTATCTGGCGTAATATATTTAT
TTTCACCTTTCAGGGTCTTATGCTGTTGTATATCGGGGAATTTGGAATGG
ATCAGTATGTTCTTGCACAACTAGTCATGTTTTTTATTCCTGATTAGGAA
TACAAATGAAATCTCTCATCTGAAACCCTGTTTTCTTTCCCTATCAGGAC
GTGGCTGTTAAGGTTTATTTTGGAAATCAATACGGTGAACAGACGTTGCT
TGACTACAAAAAGGAGGTGAATTTTTCTCTCTTTTAAATAAGAATTCTTG
GAAGTTATGCATAGATAAAAGGCAAGACCATGTCCATTCTCATTATGCAT
AAATAATATAGATCGATATAATGAGAAGACTGAGACATCCAAATGTGTTG
CTGTTTATGGGAGCAGTATATTCACAGGAAAAACTAGCTATTGTTACAGA
GCTTTTACCCAGGTAATGGGAAAGTCATCTCTTGTTATGTTGATTATGGA
TTAAAATAAATCACAACTATTATTTCAACTTGAATTTTCTTATGTAGGAT
CAGTGTTTTAGAGAGCACTGTCCAGACTCACCGGGGGTTGTCCCGAGGTG
GGTCAGTCCAAAAAATGGCTCACGCCTCATTTTAGGTTTTAGACGAAAGTT
ATCTTAGTTTATTGTCAATACATTTCACATATTTTTAGGATTTTTTTTTT
TCTTTTTTTATGGTTAGATTAACTCGACTATATAGTTTTGTTCATTTTGG
TTAATATTGGAGCAACAAATACGTATCAAGTTAACACTATTCGTTTTTTC
TTTAGTTTTTTTTTTTTTTCTATTTTTTGTAATTATGTTTATATATTTTAG
AATATTATAAAAAAATCACCAAGCTTACGCCCCACGCCTCGCCTCCCCTAA
CCCCCTTGCCTATTTAAAACTGTGTAATATAGAAAAATTATTACCCTTCG
ATTGTTATCTCATATGTTTTAGATATTGTTTGATATTGTCAGGGGAAGCC
TTTATAAAACAATTCACAAGAACAATCAGGCATTAGACCTCAGACGGCGT
CTAAGGATGGCTCTTGATGTTGTGTGTATTTCATTCTCAAATCAATCAGT
TCATTCAATCATTTTCTCGATGATTGCTTTTCAGACCCTAACCAAAAAAA
AAAAAAAAAGGANCAAAAAAAAAAAAAAAAAGGAGAAGAAGAAAGAAATAA
ATAATAAAAAATTGAGACTTGTTTTCTATCAAGAATATTCTCTTATTCTT
GCATTTTGTCTTTGGCAGGCTAGAGGTATGAATTATTTGCATCACAGAAA
TCCACCAATAGTTCATAGAGACTTGAAATCTTCGAATCTTCTGGTTGATA
AAACCTGGAATGTCAAGGTGTTGTTTGCTTAATACAGTTGCTTCAAATGA
ATATAGAGGTTGAATTCCAGCTTAATCAGATAAATCTTTCCAATTATTCT
CTATCAGGTTGGGGACTTTGGCTTATCGAAGTTGAAGAATGAAACATTCA
TAACAGCAAAATCAGGAAGAGGAACAGTAATGATCTTTACTTTTCTCTTC
CAAGTAGTATTGATTATTATATTTTCTATTGAAGGACAAGTGAGAGTAAT
ATCAGTTGGGTAGTGTTTGGTAGCAAGGAATTCGAGCCTTGGAATTGGAA
TTGGAATTGGAATTGGAACTGGAACTGAAACGAACGCATATAATTTTTTT
AAAACTTCATACGCGTTCATTTCTCATTGGGTTAGTTGCCTTTTACTTTG
GTCCAGCTTCATTTTAGTCCCTCTGCTTTACAAACCTATATATCCCTCCT
CCTACTCTTGACTAAAACAAGAAACTCACATTTTATTGAAAAAATATACA
AAAGCTTAATTACCCTTTATTTTTTTAATTTAAATTTAATATTTAAAAAG

AAAAAAGAGAAAACTAACATTTTCTCTCTCTAGTTCTATTTCTCTCTCTA
CTTCTATTTTTCTTTTTCTTTTTTTAACACAGACAAAATGAAATAAAGGA
TAGTTTTATCCATTTTATGTAAAATTTAGATGAAAAATTTAATTCTACCC
AATTTTTAGCAAATTTTGCTAAAATATAAAACATAGTTTTCCACTCTGTT
ATAAGTACAAGGGAGTAAAGTGTAGATTTTTAAAGTAGAGATGACAAAAT
GAAACAATATCAAATTACATGGGGTAAAACCGTAAAAGGCAATTTACCCT
TTCTAATTCCAATTTTGAGGTTCCGATTCCATGAAACCAAACGCTGCCTT
ACTGATTTTACTATACTATTGTTGCATTTTCTCGACTAATGATATCTTCT
TGTTATTTTAACTGCAGCCACAGTGGATGGCTCCTGAAGTTCTTCGGAAT
GAACCTTCGAATGAGAAGTAACTACTGGAAATTAAATTTAAAAATTATCG
AGCAGGATTGTTTTCACCATTTTTTTCTTTTATCCTTTTTTTCCTAATAT
TTGTTTTTAATAGAATAAATAAAAAAAAAGGGACAATGGATGAGAGGTCCT
ACATTTAAACTCTAATTGGTCAAAAGTCCTACATGTGGTAAAAAATTTTG
ATTTAGTCCCATCTCTTTTAACAAGGTGGAAGTCTTACTCACGTGATAGC
CACGCGTTAATTACATGTAACTACTTTCTGTGTAATATTACTATATTAGT
TACAAGTAACTATTTTTATTTTTTCAGTTACATATTTCAATAATATATAA
TTTTTTAGTTACATGTCTCTGATATGTAACTAAAAAGTTTGATATTTTTC
AGTTACATGTTTCAATAATATGTAACTTTTTAGTTACTTGTCTCTGATAT
GTAACTAAAAAATTTGATATTTTTCACTTACATGTTTCAATAATATGTAA
CTTTAGTTACTTATCTCTGATATGTAACTAAAAAGTTTGTTATTTTTTCT
GTAACGTCACAAAATATTATAATTTTATATATATATATGTAATTGTAACA
TGTAACTAATATTATATAAGTACATATAATTAAAAATACTCAAATATATG
TGAAAAGTAACAATTAGGAAATTGTAATTAATATTAATTACATGTAACAG
AATTCACTATAAAAATCGAATGTACTCACTAAAAGGCTCCCTACAATCTC
TACAATATCACTACAATTCCTGCAATACGGGACTAAATACAAGTGGAATC
TAATTTAAAGGACTAAATTAAAACCTAATAACACAAAACAACTTTTCACC
AATTAGACTTAAAAAAATAGATTTTTCACCGATTTTTCCTAAATAAAATC
CCCACCGAGTATTCCCTCAGTGTTTGAATCAACAAATCAATATTTTTTTC
TGATTAGAAGAGAAATTGGTATCTCTTGTTATTTAGATTTTGCTGCCACA
CAGGTCAGATGTCTTCAGTTTTGGTGTCATCCTCTGGGAACTAATGACTG
AATCCGTTCCCTGGATCAATATGAATTCCTTACAGGTACACTCTCTCTCT
CTCTATGTGTATCTCATATCGATTTTTTTCTTATGTTTCCAGATTTCTTG
AATAAATTAATGGGATCAGAATAAAAACAGTTCTTTCTTTTTGTTTTCAT
TTTTCTGTCTCTGTTTCAAATATGTCAAGAAAAAAGAAAGAGAAATATAA
TGGCGGCTTTTGCTTAGTGTCTCTATTTTCAGACTTAAAATTTGTATAGA
AACAACAATCTGGGAAAACTTCTGTTCCGGTCCCAAAAATTTGTTTTCAA
ATTCTGGGAACAGAAACCACCCAAAAGGGAAAAAAAACTGAACAAGAACA
CTATGCAAATGTTTGGTAACTGTATTTTTAAAGTACTTTTAAGCAAATAA
GAACTTAATTTTTTTTTTAAAAAAAAAAAAAAACATTTGTTATGTACTTAA
AATATATTTTTAGAAATTAAAAGAAAGTACACACATGATTTTAAGTAATT
TCTTCAAAAAACAAAAATAATTATTTTTTATTTTTTCGAGTTTTATATTA
TAAGAGATCAATGGACTAGGACCTCTCCACTCAATCCAATATTATATCTA
TACTATTAAATACTTTCGATTTTTCATGTCTCTTTTAAAGAGGATTTTTT
ATCAAATTATTTTTCTTTTGATTTTGAAAAGTACTTAAATAAAACGTTTG

CAAACTGTGCCTCTAAGTGAATGTGAGCTAGGATTTACACAATTTTGTA
TGCACTAAAAGCCTATTCATTTCAATGTGTAAAAGGTAGTTGGAGTAGTC
GGCTTCATGGATCGTCGGTTAGAAGTACCAGAGTCTCTTGATCCCCGTGT
TTCCTCGATCATCCATGATTGTTGGCAAAGGTGATTCTTATCTTCTATAT
GTATATATTCTAAATTATGATGGTTTTTTAAAATATTTTGATTGGGACTG
ATTTAAGGAGAATAGATGGATGATATTGGATTATAGAAGTTTTTTGGGAT
AAAATTCCTGCTAAAGAAATTCAAACCTCCACTTCTAAAATGGTTAAATT
AAATTATATTTTGTGCCCTTCAAGTTTTACATTTGTATTTAATTGGTCAT
TTTTTATTATTATTTACTATTATTTTATAGTTATAAATTAGAAAATCAAT
ACAGTGACACAGAATCCCTAGTAATAAAATAAAGTTGCCTCTTTCTTTTT
TTGGCCATTTTTTTAATTTATTAGGGAATACTCATTATTATCATCAAGTT
AAAATAAAAAAGTCCCATTATCATATTATATAATTTATAATCCCTCATTT
GAATTTCTTTTTATTTCAAAATTGTATTACTTTTGAGGTTCTTTTCATAT
AGTCTTCTAATTTTTTGTTTTTAAAGTTTTTTATTCTATTTATAGAGATT
TTTTTTCCCTCCTTATCACTTAATTTTTAGTCCTTATCCATACTTTGGTT
AACGTTAACTATGATGTCCAATACAAATTCAACATTAGTTAACATCAAAG
CAATATACTCAATTTAGTTGATGTTAACTTACGAAAAAAAAAAAAATAAAG
AGAAAAAAAACGTGGACAAAATATTACAAAGTGACTTATTTGAAGGACAA
TTTAATAGTGTTTCCATTACTTTATATTCTATTTAAACACGTTATAATTC
AATTCAATTTAATTAATTTCCATGTTAAGTCACGGGCTACCATGTCTTTG
CCACCAAATCTATTTTTGGTTGCACGGCATGTGAGTGTTGGAATTGAAAT
TTGAATTGAATTCATGACTTTAATTACGTGCACTGGTCATTCTCAGTTTT
CAATTTCAAGACTTCAGTTATGTGCTATCAAACACTACCTAAGTGTAGTG
GTTTATCAAGAAAATTGGTTGATTGCGGGCTAAATTTTAGGGAGTCCAGT
AAATATTTTCATCATTTAGGGGGATCTATGTTAAATTTTAAGGGATTTTA
ATTTTTTTTTAAAACTATAGTGAAAATTTTCAAAAGTTGAGGGGAGGCT
CGAGTCTCTCTTAATCCTTAACAGTTCTGCCTCAATGGGCCTTACCTAAG
TGAAGTTTTATTTCATCTCTGTGCATGTAAATGTCTTAAAAATATAAATG
TGTTCATTTTTAGTTCCAATTCCAATACTTCAACCATGTTTTTATTTTGT
TGCTAAAACTTGAAACTCGCAGCAATCCAGATGATCGACCATCATTTGAA
GACATTATTGAGCGAATGACCGATTTAATCCAATCGGTTACCGGTGTTTT
TGCTCGAAAGTGTTCAAAGCCTTGA
>EUC21207-RA [gene]
ATGAAGAATTTCCTGCGAAAGCTTTACATCAGTGGTGGTGGCTTGCCCGA
TCACCAACACCCACCCGTGATCAGTAATCCTCTTTCAACACCACCTGAAA
CGACGGAAATGCATCCTTCATGTTCAGCTTCTTCTCCTTCGTCTACTTCT
TCGTCTTTATCTTCGTCCGCATTGGTGAGAAGAACTGGAGCGGTTAATTC
GGCGGCAGAAAACTCGAATCCCGCGGACCCCAATTTCTTTGAAGAGGAAT
TTCAAATTCAGCTTGCGCTGACCATCAGTGTCTCTGATCCCGATGCGCGT
GAAGATTCGGAAACGGCACAGATCAATGCTGCCAAGCAAATAAGCTTGGG
GTTTTCGCCCTCAGGATCCGTTGTTGAGTTTCTTTCGCTTCGGTATTGGG
TATGTCGACGTTTTCGCCTTAACTATGTCCTTCAATTTTGTGGAAAATTG
TCTTTCTAGAAGTTTTAATTTAGATCTCATTAGGGCTATGCTTGTCTATT
GCTTGCATTTTGATTGTTATTTAATTCACAATTGCAATAATTAGAATCTA

CTGTCGATGGTGATTGCCTGTGCGCACACGAGCACGCATGCACACGGACT
AATGCGTTAAGTTCAATTCAAATTGCAATTTTCCAATTAGGTACATTAGG
ATTGCTGTTGTTCATAAGTAACGGAAATAAATACTTGGCTGGGTTGGGAG
AAAGGGAGAAAGAGAAAAGAAACACCTCTCTCCATTTCTCTTGTTTGACA
ATTTGGGTAAAGTGAGGTGGAAGACGCAAGGACTGGCGAGAAAATGGAGA
GATAATTTTGTTGTGAGCTGGTGAGATTTGGAGAGAAAGAGATGGAGAGG
GGTAGTATTAGTAACGGCCTATATATCCTTAATGAGCTTAATTTCCTTTT
ATGGAACATGTTAAAATACTAAATTGGCTAAATATTACATTTTCTCTTAT
CCTCCTTTTGGATGGAAGGTAGGGGAGGAAAGTTGGGTGGGGAGGAGGAT
TGTATTACTGCAATTTTTTAACATTTTCTCTAAATTACTCTAAATACAAA
AAATCTACTAATTTTCCCTTTATTAAAAGAAAAAGAAAAAGAAAAATCTA
CTAATTTTCTCTGTATTAATGCCAAAAGAATACTAATTTCCTCTATATTA
TTGCTAGAGAAAAAAAAATATATTAACATACACAAAAATATTATTGCATT
TATCCACCTATCATATTGCAAATCACATAAATTCAGTTACCTATTATAAT
ACAAATGCACATAAAATACATTCAAGCAACAACAACAAAAAAAGGAATAA
AAACTAGGAANAGGGAGCTTTCCTTCAAAATCCGGCCAAGTTGGAAGGAT
TGCAAATCCTCCTAAATTATCCCTCCAAACCCCCTCTCTCATCTCCCCTC
CTCCCATCCAAGCACACCAAGAGCTCCCCTCCCCTTCCTTCCCCTCCCTT
CTCCTCGATCCAACATAGTGTTAGTGTTTTTGCTTGTTTGGTGCCAACAC
TCTTTGTACGCACATCTAACACTCGTTATTCGTGACTTGTCTGGGTACAT
GCCGCCGTTATTAGCGAAATACAACTACTGCTCCCATCATTCCTCTATAA
TTGTCCTACTTTCCTTTTTTGGATGTTCCAAAATAAGTCTCCACTTTCTC
AATTAACAAATAAAATTTGTCACATTTCCCAAACTAACCCTCTTTTCAAC
CACTTCTTCAAAAGTCAAAATGTGTTAAAATACTTTTCCACCTTTCACTT
CAAGGGCAATATAGAAAAGTTAAAAATGTTGAAACACTGCATAAAACTTT
GTGCAACGTCAAAGAGGACAATAATAGTGGAAACACTGGAAAGGAGAGTA
TTTTTCCCCGCCCAACAATTAGGAAGTTGTAAATAGAGATCATCTAAATA
ATAATCAATATCACACGAGTCATGACCTTATAAGGATTTGACAGAGACAT
ATGAAGGCATAGTCATTTGCCTCAAAACTCTTATTCGTGGCAACAGATGG
CTTGAATATGTAAGTATGGTGTTATCAGAAACTCTATAGATATCGGCAAT
CGCTTTTTAAATCAACTTGTTTATGCATGCATGTGTTAAATTATGAGCAT
AGTTATATGGGTATTAACTAATGAGAAAAGTATTCTGTTTTTCACTTGCA
GAGCCAGAAACTTATAAATTATGATGAGAAAGTGATGGATGGTTTTTATG
ATGTTTATAGAATTACTTCAAATTTAGTCGGACCGGGTAAATTGCCACCA
TTGGTCGATCTTGAAGCAATTTCTGTGTCTGAAAATGTTGATTATGAGGT
TACTTTTGTGTACCGTATGAATGATTCTGAATTACGTCAGCTTGAGGAAA
GGGTGTATCATATGTCCATGGAATGGAAGAATTTGAAAAATGGACTTACT
ATGAGTGGTTTGATTCAAAAACTTGCTGACCTTGTCGTTGATAGAATGGG
TGGCCCAGTTACTGATGTGGAGGAAATGTCAAAAAGGTGGAGATCCAGGA
GTTATAAATTACGGAATTCGCTCCAAAGTATAATCTTTCCTCTTGGCTGT
CTTGGTGTTGGACTTTCACGTCACAGAGCTTTACTTTTTAAGGTTGGTAG
ATTATTGATATCTCTTTTGAATTTAATTATAAATTGATTTAAGGAGCACA
CTCCTCTAGTCGGATCTAAATTATGTTTCTCATATGATACAACTTGCAAA
GATATTCTGGGTCAATTAGATGTCGATAATTATTCTTGTAGTCTTGTCAG

ATTATTTCCTTTTTTTTTGGGGGGGGGGGCGTTGTCGTCATGATTTCTTC
TTATTTTTTTGCATTGTTGTCAGGATATTGACATTGGTTATTTTTTCCTG
TTTAAAGTTAATTTCTTTCAAATTCATAGTTTTCTTCATGGTCAGTTGTT
TTGTATCTGCTAAGGCATAGCATTACACTTTCCTTGAAGTTTGAAGGCAT
ATGTGGGTTTGCCAATGCACCGTAAATCTTAAAGTTATATCTAGGAAGTT
GGAACCTAAAACCTAAGTTTACACACTTGATAATTATATATACAGGTCTA
ATATCCAATACATGCAACCTTCTTTGAAGTGAAACATCCAAAAGATTTCT
ATGTGCATGTGTGTGTATTTGTTGTAACAAATTTCATGGATCACCATTTT
CATTATTTTGCAATCCGTCCTCTTAACAAATTTTAACTTCAATATTTAAT
GATTTTATGCTTTGATTGCTAGGTACTTGCTGATAAGATAGACCTTCCAT
GTATGCTGGTCAAAGGAAGCTATCATACTGGGACTGATGATGGAGCGTTG
AACGTTATTAAAGTTGATAACGGAAGGTAGTTAAATCAGTTTGTGTTTAT
CAACTCAGTTGAATGTCTAATTTTATGAAGGTCTCCATGGTTCCAATTCT
TTCTTTCTTATTTTAAACTTTCCTTGTTTCTCTCCAAACTTATATATTAC
CCTTACTTTTTCTTTGTGATCAATTAAAAGTCAGTTGTCTACAATTAGCA
CTCACCATGGCTCAAGGTCAATGAGTGTCAAGTCTAAGAGGAGGTCTTAT
ACTGGATGTCTATCGAACACTTGAAATTTTATTTACCGAGGGTTTGGGGG
TTGACAGGATTATGCAAGACTAAGGTGAAATTCATGCTTTAATGATGCTT
AGAAGATTTTCTAGAAACTCTGTTGTGGAAGTGAATTTTGTCATGTGAGC
AGTGTGGTTATCTTCTTATCTCATTACGCATCTCGACCTTCAATTTTCCT
CTTATTTTTGCACCTCTATCTTTCTCCTACCTGTCCATTCGGCACTGAGT
TACTTTATTATGCTTACTCCGAATAAGGATATTGACAACCTTGTTCTGTT
ATTTTAGGCTGCAATTTATATTCAGCTTCGACTATTCTCCTATGTTATCA
TGTCTTCTATGAGAAATGTTAGCTGTCTTGTTTCTCCTTTTTGTTGTAGT
CTTTTTTCTTCTTTTTTATTTGTTCGAAGATAAAGCACAACAGATATTTC
CGTTGCAAGCAAATAATCTCTGTTATTTGTGTATGTTCTCTGCTATAAGT
GCTTATTGACTTTCATTTTTTTATTTTTTATTTTTCATCTTGAAGTCTCT
TGGCAGTTTAGTGGAATATTATGTGTTTTTATCTTCTGTCTTTCCTTAAA
AGCATTGTCAATATTCTAGTTCTTACTCAGAAGAATCCTAGTTCTTACTC
AGAAGAATAGTTATGGCCTTAAACAGTATTGAGAAAAGAGTTAATGGTGA
ATCTAATTGTCGAATTGATGCAGTGAATATATTATTGACCTAATGGGTGC
TCCAGGCACATTAATTCCTGCTGAGATCCCCAGTAGTCACCGTCAACTTG
CTGGGTTAAATATAAGGAGCATTGCTTCCATTGCCGATATTGTCCGCGAT
TCATCTTCAGCTTTTGATAAAGAAAGAGAAACAGTGACAGTTTCTCCTGA
CCTAAAGGGTATTGCAAAAAGCTGCAGTTCAATTTCGGGAAAGGAATCAT
TAGTTGGAAACCAGATAAAAGTGGACGACAGAAGCATTGTGGAGAAAAAC
TTAACTGATATATTTACCTATGGGAAGCTTCTTCCTGCACGATATAGATT
GAATGAAGGCACACTTTGCAAGAAAGTATCAACTGCACAAGAGATGCAAG
TTAAAGATGTTTCGGAACATGTAATTAGTGCAGCAAAACATCCAGAATTC
GCTCAAAAATTGCATGCTGTTTTGTTAGAGCCTCGTGCACCACGACCTCC
GGATTTGTTTTCAGAAATAAATCCGAGTGAACGTGGAGAAGAAATATCCC
TCGGAAAGCTTCAATATCATCCGGTACTCTCCTTGGTAAACAATGAGATG
TCTCTTACGTCTACTGGAGTTGATTGGGCCTTCAGCAGTGTTCATTCTGA
TAGCAAATGGAAGCCCTCTGTTGATGATTTGGCAACTGAACAGAAGGGTT

TCAAGTGCTCTGCAACTTATCCTGGTAATAATTTAATCTCTAATACTACA
AGTGATGGATTCATACGGGTAAATAGTGGACCTAGTGAAATGATCCAAGT
TCATGATGCATCTCTACGTATTGATCAATCCGGAATACCCGCAAGAGCTT
CAGATTGGGAACAGCCTCATGAAACATTTGTGCCTGCTGAAGTCAACCCT
TTAACAGCGCAGCCTAAAACTGGGCTTTCCAGTGAGGATAAAATCGTGAA
ACATCATAATAATACTGGAAAAGACAATATATATATTGCAGAACAGTTGG
AAATGGATAAAAGTGATCACCTTACTTCTTGCACTGCTCACAAGAAGATT
TACCCAATGCTGCGTGATGTTGCTGAATGGGAAATTCCATGGGAGGATCT
TCAGATTGGAGAGCGGATTGGCATTGGTGAGATTTATACCCAGTTTAAGC
TTTATTTCATGATGGCGAATGAGGTACATATAACGTATGAATTTTTGTCT
CTGTCTCATATTTAAGGGTTTAGAAGGTTGAAGTGGAAATAATTTTATCA
TTTTGCTAGTGGTTGTGAATGCTTAATGTCGTCTTTTCTGCTCACCTCTA
CTTTTTTTTTCAACTAAGTGCACATTTCTTACATGTCTTGATCTCCTTGT
GGTATTCTTTTTGAGAAGTTATAGTAACCTTTATAATAATCTCTGGAACT
CCACAGAGGATAGAAGTGTGAAACATAGGAGGAAAATTTGAAAATTGTGC
AATGGGTATGCAGAGCCTTTATGACCAGGCACGTGCAATAAGCCTAGGCT
CATGTCCAGGGCATAGGATCAAGTTTCTAAATTCAATATTCATCATTAAA
TTTAGTAGGGCTAAGCTATTAGTATTTCCTGTTGTGATTGATTTCCAATT
TTCTTTAGCTGGAGATTGCCTATCAATCGGATATGGACTTTTGAAATACT
CATCCAAATATGTGCTAGGATATAAGGGACGATTCCTTATTAAACACGCT
ATATTTCATTGAATACTATTTTAAGAGTGCATATTTTCCTCTATCTCTCA
TTGGATTCGAAATTAGATAGACTGACATTTAGATACTTCTCGTACCCGAT
AACCTTATGGATTGTTGCTTTTAAAGACTAGGATTGTGTACTGGAGCATG
TTTGGACAATTTAAAAAGATATTATAGACTTCATATCCTCTCTTAATCTT
CTTCTTCTTCTCTCTCTCTCTCTCTCTCTCTCTCTCTCTCTCTCTCTCTC
TCACACACACACACACACACATATATTATTAAGCTTCTTTCTTTTTTTCCCT
TGTGCAAGGCACACTTTTTGGTGGCTCGTCATCCTGGAACAATTCCTTTA
ATATTTATGGTCTGTCGTACCATGCCCAAGTGTTGGAGGGATTTGCCTGA
CGCGACCTATAGGCACGACACATATGGGCTGGATGGGTTTTGCTCAGCTC
ATGGTCTATGTGGGCTGGAATGATTCCCTTGGGACTGCATTAAATGCCAA
GAGTGACTAAGGCACAGATATACACAAGAAATAGATTGGATTAATTTCTT
TTTAGTGTAAGCAAATCTTCATAATAAAAAGTATTGGTAGGTGAATGCTT
CGAATGCACTTGTTTTGGAACTTGGAACACTACCCTGGCACTTCAAAGAA
TGTGTATAAGTTTGTAGGGACAAATAATAGAGATTGTACGTGAGTCGTGT
AGAGAGAAAGCGAAGTTTGGAAGGATAATCTTGTTGAATATCTCTTAACA
TGACCGTTTTGAGGGGCAAAAGTATCATTCGGATGCCTCTGTGAAGATAG
GGGGATGGCTGTGTATATCTTCCCTGACCTGCGATTTGAAAAATTAGTAA
AATTAGCCGCTCTGTACTTTCCTCACCCCCTCAAACATAACTCTTCAACC
ATTGCGTGGTCACTTGCCCAGTTTAACCTATTTCATGTTGCCTTTTTGTA
CCGCTCTCTATCTCTTAAATGTCAACACAGTGCTCTGTACCATGCTTGAC
AGGTTGAGAACTTGAATTTTTTACAAAATTTTTGGTCTAGTCAAGCAGCT
TCCAATGTTTTATAGGACAATAATATTTTGGCCTCATTGGTCTCACAATT
CAATAAACTAATTCTTGTGTATGCCTATTTTCTAGGTTGTTTATAGTTCT
ACGCCTATTGGAAGTCCAAATATTTACTTTGGCTACATGGTCAAAGTGGC

ATATTGTATGACCATGTTCCTGTAAAGTTCCTTCTTATGTGCTCAATATA
TTAGAACCTTGTATTGCAGGATTGTGGTCTTCACTTGATTACTGACTGGT
TCTTCATTGGTAACCTTATGAAGTTTCACTTCTTATCTTGCAGGATCATT
TGGTGAGGTTTATCGGGCTCAATGGAATGGCACTGTAAGTTTACATGGGT
ACTTATGTGCAGAATTATGAATATTTATCTTCCTATCGTCATTGGTTTCT
TTTAGATATTGAAGACTGATGTGTTTTTCATATCTTCTTGCAATTCCTTC
TGGTAATGCATTGAGGAAGTAATGAGTGTCTAATGAGGGAGTCAAGATAA
TGGATGTTCCACCATTTACAGTGTCGAAGGTGATTGATTATGTCTGTTTG
CAGAATGTAACATTATGGTATAAATCCATTTTAGGCCAGTAATTTTGTAA
ATGCTAATTTGATTTGAATATTGATTTTTTTTTTTCTTTTAACTAAATTA
TTTTGCTCGGATGGTGGGGAGTGCCGCATATTTCTGTTTTGTACTTTCTA
CCTGATTAGGGCTAGCTGTTCTGTCACTGTTGAAGAGATGCATCCATGCA
TTCTCTACCCGCAGGAAATATGTATTCTAAGGAAGTTAGTAAAACCCGCA
TCTTTGTGTTATAATACAAGATTCTTGTAATCTGCTGAGTGCTGACAGTA
GTTCTTTGACTATAGTTGCATTTTTCCAAAGTAAATGTCGCCATAGTGGT
TCTTATAGTATGGAATGTATTAAATATTCTAAAATGAGATCCCTATTGAT
TTGTCTATTATCTTAGGACCTTGTTTCATTATTTTAATGTGAAAGAACTG
ATGCCGCATGACAGGAAGTTGCGACAAAAAAGTTCATGAATCAAGATATC
ACGGGCGATGCACTCGAACAGTTTAAATGTGAAGTAAGGACGAGATATCT
TGCATCGCACAATGTCATTAACGGTTTTGTTGGTAGTGTTTGTAAGATAT
TCTACTTACTATCTGGGATGTGCTTTACGCAGGTTGAGATCATGTTGAGG
TTGAGACATCCTAATGTTGTTCTTTTTATGGGAGCAGTTACTCGTCCACC
GAATCTCTCTATCTTGACAGAGTTTCTACCAAGGTTGATAACTAACTCAA
CAAGATAATTTTTTTTTTTATTGTTTCTTTTATTAATAATTATAGCTCCT
ATTTGGTACTTGGTCAGTATTAATCTAGATAAGCAGAAAAAATTTCTTCT
CGGCTATCCTGTTATATCAATCATTGTTCATTCAATATCGGGTAAGAACT
AACGTGTATCACCTCTCTCTTCTAAAAAAATACATTTCTGTAGTCTATAT
ACACAAAAATAGTCTAGTTAAATACTAATATTTTTCATACTACGGATTTC
CCGTGCCTAAAGGCACTGTAACATTTTCTATCGTAACACAACAACAACAA
CAAGAAGTTAACAACGAAGCCTTTGTCCCACTTATTTAGGGTCAGTTACA
TGAATCCATTTATGCCATAGTGAAACGTTTTCTAGTGTAAAAAGATATTA
AATAATTAAAAAAATTGGCCAATTTAACACGTGAAATTTAATGGGTAAAA
AGTATAATTTAAATCAACTGCCTAGGAAGTGGGGTGGTGAAATATTGGGG
TTGAAATTTTCCTGCCCAAAATTTTGTTAAAAGAACTTGCCTGTTTATTT
TTATCAAAATCAATCTCAACCCTTAATTAAAAGACATTTTCAAATGCTGT
CCTGATTTATAACATAAATGAAAACCTCTCAGCTTTTATCATCATCTCTG
TGTAGATTTTTTTTTTTTTTTTTATTTTGGGGGTGGGNTTTTTTTTTTTTG
GGGGTGGGGGTGGGGGGAATAGAGTTTCCCAGTATGATTTAAATATTCTG
TTTGTTTCTTTTTTCTTCTGGTTCTTCACTAGTCTTATTGTTGTTGCATG
CAGGGGAAGTTTGTTTAGACTGCTTCATCGTCCAAATACTCACATTGATG
AAAAGAGGCGATTACGGATGGCTCTTGATGTGGTTTGAGTTTGAGATTTA
CTCCTACTTTCTTTACATTATAGGGTACTGTTCCTTTGTTCTAAATCTTG
CTTGGCTTGCAGGCCAAGGGAATGAACTACTTACACACAAGCCAACCTAT
TATTGTGCATCGAGATCTAAAGTCTCCAAATCTCCTTGTTGATAAGAACT

GGGTTGTTAAGGTCTGGTCGAAACCTCAATCCTTAATGATGCTATTATTT
GTCTAATGTAAGAGACACATGTTAAGTATTAACCATCTTAGGTCTTAATC
ACTTGCAATAAGAACTGCACAACCTCTTCGTGTATATTTTCTGGGCATTT
TGCTAAAATATATATTTTCTGTATACTTTGAAGGTTTGTGATTTTGGGAT
GTCACGTATGAAGTACTGTACTTACCTGTCTTCAAAATCTGCTGCTGGAA
CGGTAATTTCACTTAATATACATGTACCAAGAAAATGTAAGCTATATATT
AGAAATATAAGATTTTTCATCATACGCCCACTTTCAAGTGTTTAATATCA
TGCAAACACCACGACCCAACTCCATCAATAAGAGCTCCACTAGTATTTTT
TTTATTTTTTTTACAAAATTAATTTTATATTTTGTTCTCTCTTATTTTTA
GCATAATTGAAAACTTAGCTTCTTTTTTTAAAGAATACAGGAGAGAAAAT
CATATAAATGATACATGGGTAATTTGTTAAAATAGACTCTTTTTAAATTG
ATGGGGTTTTCTTGGTAAAGAAATTCGAAATGGTAACCTAGTGAGCTTAT
AGGGTCCATACAAAAATTACCCTAGAATATATAGTTGATTAATTCCCTGT
ATTCATTTTGTGCCATTATTCTGCAGGCCGAATGGATGGCACCAGAAGTT
CTAAGGAATGAACCATCAAATGAGAAGTTAGTGGATTCCAATATACTAAT
CCTATAATTCAACGGAAGAAAGTGAACAGAAATTAAATATTTCAAATCTA
ATGCTGCCAATAGAATAAAGTGGGCTGTATTGCAGGTCTGATGTTTACAG
CTTTGGCGTAATATTGTGGGAGTTGGCAACTTTGCAAATACCGTGGACCG
GGATGAATCCAATGCAGGTTGTTGGTGCAGTTGCATTCCAGAATAGACAC
CTAATTATTCCAGCTGAGGTTGATCCAATGGTTGCGGAGATAATCACGGA
CTGTTGGAGTCCGTAAGTAATAAATCATGCATTTCTTTTCATTGTTCAAA
TATTATGTTTTTTAGAAAACCAATTTATGGATATAGTTTATGTTAGAGCA
GAGGTGGGTCTTGAGGGCAGCTGAGTGGCAAAAACTTCAGAAACCCCCTT
TTTATATTGTAAAATTACATATTTTTCTAAAGTAACATATCCAGCCNCCC
CCAAATCCCTCAAACAAATTTAGACTAATCCCCTAAATTTGAAATCCCGT
ATCTGTCCCCATGTGAGAGGATATTTGTTTATATGGGTATTGAATTTGTT
TTCATTCTGGGAATTCTTTATTGATCCAAAATTAATTTTCTGAATATATA
TTTAGTGAGAGCGGAGCTGGATCTAGAGAGCAGCTGGGGGCAAAATTTTT
AGAAACATCCTGTTATGTTATAGAAATACGTATATTTGTAAAATATTATA
TCCTGAACCCCCAAACATTGTACACTGGCTGCCAAGTCCCTTAAAAAATT
ATTCTAGAAGGACCCCTCTCTCTCTCTCTCTCTCTCTCTCTCCCGCCCCC
CCCAAAAAAAAAAAAAACTCGTGTAATATTTCTTCATTGTCTGAGAAGACT
GGCTTGTACTGGGAAGTTATATGTTTATTTGGGTGTTGAATTTGTTAGCA
TTTTGAGAACAGCTCCGTCAATCCAAAAATAATTTTTCTTAACAGCATCT
AGAAATATAGTTTATGGTTGGGTGCTGAAAACAGGTTTTGATTTTTTTTT
TCTTCTTTCCAGTTAAAGAGTGTATTTCCTTAGCTTTCCCAATTTTGTAC
TATAAATAGCAGTCAAATTAAATTTTTTTTCCGATATATCATTCACAAGT
CACTCTCTATATTTCTCTTCTGCAGTAACCTACACACTCGACCCTCTTTT
GGACATATCATCGCACGTCTCAGGTGTCTTCATTGTCTGAGAAGACTGGC
TTGTACTGATCAAGAATAA
>EUC11169-RA [gene]
ATGAACAATACAGCACCTAGGCCTGCTGGTCAATGGATCCAGCAAGCGGT
AGGTGTCATTCCAAAAGATGTGTTGGTGGAAAAGAATGTAAGTAACAACA
TTTCTGTGCAGACAGGAGAGGAATTTTCTTTGGAGTTTCTTCTGGATCGT

GCTACTCCAAAGCAAGTTCCTGTGATGCCTGATATGTCCAAGAAGCAGGA
GAATGGACTGCGATTCATTAACAAGCAGAACCACCATAGGGGTTATGAGG
AAATAACCAGACTTCTGGGGTTGAAGAGGATGGATTCTGAATGTGGTTCT
GATATAACTGAGTTTGCTTCTGCAAGAGGCTCCTCTTTTGAGATTGAGAA
TGTGCCTAATATTACCAAAGAATGTGGCTACCATGTGGAACAAAATATGA
ATGGGCATACACCAAGGAAGGCTTCTGTTGAAAAAACTTACAATTGGAAC
GTAATAGAACCAAATGCTTCACCTGTAAGTGGATCTGATTCGTCTCACAA
ACGCCAACCTTCAGCGGATAGTTCTGGACCTGGCAAGATGAAGTTCCTCT
GCAGCTTTGGTGGTAAAATCTTGCCTCGGCCAAGTGATTCTAAACTTAGA
TATGTCGGGGGAGAGACCCGCATTATTTCTATCAGGAAGGATATTTCCTG
GGAAGAACTTGTGAAGAAAACCACAGGAATCTGCAACCATCATCACATGA
TCAAGTATCAGCTTCCAGGGGAGGAACTTGATGCCCTTATATCTGTGTCT
TCAGATGAAGATATTCAGAATATGATAGAGGAGTACCATGGCCTCGAAAC
TCTTGGTGGCTCTCAACGTCTAAGGATATTTTTGATTCCTTCAAATGAAT
CTGAAAATACATGTAACCTTGATTCTGGGAACTTACAGCCAAGCAGTCCT
GATTTTGATTATGTTGCCGCTGTAAATGGCATAGTTGACCCTAGTCCCCG
GAAAAACTATGATAGGCAGCAGCATTTGGCAGGTGATTTAAGTCAGCTGA
AATCTAATGAGAATTCTCCCTCGACTTTGGTTCCTCTGGACATCAATGAC
ACCTTTGATGTCTCACATACTACTAAGAATGCTTCCCCCAATTTAATCAT
GTCTCCCCGTGGGTCTCCTTTTTCACCAGTGATTGTTCGTCAGGGAGAGT
CAAAGAGTGTTCATGCACAACCATACAAGGACGATGCATCCTGTGGTAGT
TCTGAAAGCACCAGGCTGTTCAGCAATGCTCAATTAGCTCCAGAAGACTC
CAGTAGCAATATTGCTGGGAATAATTGTCCCCATCTTGAGGCAGCAAATG
TGGTAAGTTATCAATCTCCTAACCAAGATAATGATTTGGGCCATTCTGGC
AAATCAGTTGGAGCGCATTTTGACAATCCGAATCACAACAGAGATTTTTC
AGTGCCTCATTTAGTTAATCAGAATGATAAGGTGTCTGATAAATGCCATC
TTGAGAGGCCAGTTGTTACAGGTAGGCCATTTCATTCTGATAAGCCCATC
GCTTGCCTTGATGATCCATTGGGTCTACAGTTGGGGTCTAATGATTCCAT
TGGCTCTCATCGTGGGGTGCCACATGTGTTTTCAGATTCACAACTACAGC
TAAATGCCGGAAGGTCTACTTACTGCTCACAAGAAAGACCAAACCCTTCT
TCTCCCTTAACCTTTGCCGTTCCACAGTTATCTCCGCCGGTGGCATCTGC
TGCTTTGCAAGAAAAACCAGTCCATCTTCGTCAGAATATTGCTCCAGTCA
GTCCTTATCGTGAAACTAAGTTACCGGATGTAGAGCCAACTGTTTCTAAT
GTTAGGATGGATTTGCCGAAGTCTTCTTTAGAGTTGGGGACCAAGGTGGA
ACGTGCACGTTGGGAAGCAGCGACTATAGATGAGAAGTGCAAATCAGCCG
AAGAAGATTTAAATAAATACAGCTTCATGATGCAAAAGCATGATCAGAAA
AATCATTTGATTGGTAAGTTGATGAATGATCCAGATGAAGAGGACCCTCT
TTGGTGTCGGGTTGAAAAGCCTTGTGAAAGTTTAGCTGATAACAGTTTGA
CAGATGTGAGCAATTCCCCAAATATTGGTCGCGACCCATTCTTTATGATT
GGTGATAACACTACAACACAAGATTGGCAAATTCCAGGGGGAATGGTTCC
TGGTGCTTCTGCAATTTTTTTGAATCCTTTTGCAGATCAACCAATGCATC
AGGCCCCAAACAATCAGTTGGTGGAAGCCCCATCTGAGATTAATGTAAAG
GGCCAGAGGCCTACAGAGAATCATCAGATTCTTTCAACTGGGATAACGAA
TGGTGAACAGGAAACCAATTTCTCATGGGCCAAGAAAGTGGAACTATCAA

GAATAATTCAAGGTTCTAAACAGCAGGCCTATTATGAGAGTTCCTTCAGT
AATCCACTCCAAGCATTTCACAGTGGACTAGTTTTTGATGAACAAATTGT
GCACCAACCTTTCGCTCATCTTAGTCCCGAGGAATTGCGCCCATCTCAAG
TTTATGATGCAGCTGCCTTGCACACAGACATGCATACCCCTACTTCAGAT
CAGAATCCGACCAACGATACTGGAATTACGAGAGAAGCTTATATCCTTCA
TGATAGGTTTGCAAATTACTCTGATCAGAAGGTAGAAAATTCAGGGACTG
CTGAATTCTCCTTTGAGAAACCAAAACTACGAAATAACGTACCAATTAGA
TATGATCAGGGAAATCAGCTAGAGGTTGTGAATACTGTAGAAGGCGCGAG
TAACACTATATTACCTGGCATATCTCCTACTTTGAAGATGCCTTATGGAG
TGGATGCAAATGGTTGTAAAGTTCCGTACTCTCTTGCAATGCAAGCTAAC
AGCATCTCTGATTTCAAGGTGAGATCAATTTCAGTGTGATTCTGGATTTC
TTTCACAGTTTCTCTACCTGCCCCAGTTTTACGTTGCTACTCTTGTTTTA
TGTAGAATGGTGAAGCTGATTATGCTGATAAGGATGAATCTATCAGCGAC
ATTCGGATGGCTGAAATGGAAGCGGGTATATATGGGTTACAGGTAAATGG
TTTTCAATTACTTCATGCCTTGTTTTCATATGTCGAGGTTCACATGTCCT
GTTTATGTTTTATTTTTACTCGTCGAACAAGGAAATCTTCTGTTTATGTT
CCCATTTTCCGGGAAGAAAGAGGGAAATTTTGGAAGGAATTGGAGGAAAG
GAAAAAAAATTGTCAATTGACAATTAACAAGGACAGTTCATTAACTAATT
TAGTTCGTTACAAATGCCTTTTGTTTCATAAACTGGCAATTAGATTATAA
ATGTGCTCTATTCCGATTCCAACAGGAATGTTTAAGTATGTAAGCTAAGG
CAGTAAAGTTATCTACTCTGAACATCTTTTTCTCAAACTTATCTTGTTGG
TTTAAATGTTTGAGCTGGATAAAAATAAATTATAAGAATATCTTTACTTT
TACTCATGAGTTGAGTATTTTTTCATGCCTGATTTGATGCACATCATGGT
TTTATCCTTGCCTTGCCATCAACAAAGGAGTTCGTCACTAATGTATCCTA
AATTCCTAAATGGGTGGGTAGATGTTGGGTGTACATAGGAACATTGTTCA
ATGTATTTTTGAAACTTTGAAATGATTGTTGTGATTCGTACCTTTTCTTT
GATTTCCTTCTCTCTCCTCTGCTAACTAAGGTGGCGTTTGGTAACCATTT
TTGTTCTCATCTCAACAGTGGGATGGGATGAGAATTTTGTTTCCTTTTTG
GGAGTAGAATTGCGTTTAGTAACGATAATTTTTTTTTTTGGAACAAAAT
TACATTTGGTAACGATTTTATCCTAAAATGAAAAAAAAAATTGTTTTGAG
AACATTTTTTTTTTGTTTTCGCCGGCCGGCCGGCGGCGGTGGCCAGACGG
TGACCGGCGGGTGGATGACGGTGGCCAGCGATGCCGGAGGTGACCGGCGA
CGGTTGCCGGAGATGGCTGCCGGCGGCGACGACCGATGGTGGCCGGTGGC
GGACCGCGGATGAACAGTGGCGGTGGTTTTTGAGAATGGGATGGGAATAA
TGAGAAACATTTTTTTTTTAATGGAATGGGAACGGGAACAAAAATTACCA
AACGCTATTCCCAAACAGAAATTCCAAAAATCTGGGAATGGAATGAGAAT
TTCTGGGATGGAAACCGTTACCAAACGGGGCCTAAGTTTCCCAAAAATCC
CTAAGTGGGTGTAGCATTAATATGACCATAATTTACTTGTCTCTCTCAAT
AACTATTGAACATTTTAACCTTGATTTTATCTCTATGAAACGCAATACCG
CTCATGTCTTCCTCTTATACGCCTATGCATATAGGAATGCCTAGAATCCT
ACTCTTTCACAGTTCAATGAATTTCAGTGCTGTGTCAAATAGAGTAGCAT
GTTCATTCTTTAGTTTTCTCTACTTTTCTTTTCTTAAATAAATTTCTAAA
AATAGTGTAGGAAAATGGGTTTCAATCATATAGGGGTCATTGCCCTTTCA
TGTAAAAGACAATAATGGTACAGTCGGCAATGATAGAATAATCGAATGGT

AAGGCAACAATTGTAAGTTGGATTACGATGTTTTATATTGTGGTATAAT
TTGCACTTTTTCTGATGAGAAATAAAAATGTATTGAGGATCAGGGTATGT
TGGATTTTTTTGTTTCTTCAATTTAAAAATTGAAAAGAACATATGACAAA
AAATATTTTTCACAACGTTTTTGTTACTGTTGATAATGTAATTTTTTTAG
CATTACTGAAATGTGAATGAGGAAAATCATAGAGTTCTATAGAAGTCAAC
CATGCATGTATACATTAAAATTGGAAAATATGGAAAGTTTAGCTGAAAAT
TGTTCAAGGCAAGAGAATTGCATAAAGTGAAATTGGTGAAGAATGAAGGC
ATGAGAATTGGGTTTTCAAAATTGTTACAACTTCTTGTTTAAGTCATGTG
AATTGGGTAATCAAAACGCTAAGTTTTAGCATAAAATTGGTGAAAAATGA
AGACATGAGAGTTTGTTATCCAAAATAGTTTTAAGTTCTGCTTGCCCATG
TTTTGTATTGCTTGATAATTAAATTAGTTACCGGTATATTTTGGAAGAGA
TTTTCTTAGCTATTTAAATAGGATTTATTTGACAATGTGAATTCAAGCAC
AAAAGTGGTTGTAGTGAACATCTCACAAGAGACTAAGTGGCCACTTCAAT
AATAGTATCATGCAAATTATACATTACATCTTCATCTTTAGGGGTTATAT
TGAAAAATAATCTTATTAGCTGAAATATTTAGACAATCCTTCAATAACGA
ACTTCCAAAATTAGCTTCCTATTAGAATTTTAAGATAGTTTTCCATTTAA
TACTTGAGTGTTAAATGGTATCCGTTGGGTATTTAATTTTGGGTCATGTT
ATTAGCTAAGTATTAACTTATGATTAACTATTATATTTTAATGATGGACT
TCTTTATTTGGACCCCATAAGCCTAATGTTTCAGTATAATAATACAAATA
CAAATACTACAACAACAACAACCACCACCACCACCACTAAGCTTTTATCC
CACTTAAGTAGAGTTGGTTACATGAATCCGTTTACGCCATAATGATCGAT
CAAAGGACGTCCCCTCAGTCAGTTTTAACGTCATCAAATCAATTGTAATA
GTCTCGTCCTAAGTTTTCTTAGGCCTACCTCTCCCTCTAGTAGTCTTCCC
GCACTTAAAGTAAATTGCATTTTCTCACTGGTGCTTCTATGGGTATTCTA
TGCATATGTCCAAATCAACATAAACAATTTTCTCTCATCTTCTCTTCTAT
AGGTGATATTTCTAAATTCTCTCTAATAGTTTCATTCTTAATTTTGTCCA
TTCTCATCTTCCTACTCATCTACCGTAACATTCTCATTTTTGTCACACCT
ATTTTCTGAACACGGTTTATTTTACTGCTCAACATTTTGAACCATACAAG
AGCATCGGTCTTATTGCAGTTTTGTAGAATTTTCCTTTTCAACAGTATTC
TACGATCGCATATCACCCCAACTGCATTTCTCCACTCCAACCATCCTGTC
TTGACTCTATGAGTAATATCTTCCTTGGTTTCGCCATCCCCTGAATCATT
GAACTTAAATATCTAAAATGATCAATCTTTGAAACCTCTTCATCTCTAAT
TTTGACCGCACCATCATCTTTCTAGGCATTACTAAAATTGCAATGCATGT
ATTCTGTCTTGTTCCTGCTAACCTTCAGACGTTTAAACTCTAAGAGTTCC
CTTCATCTTTCTAAATTAGTATGACGGCTTATTCTATTTTCATCAACTAG
TACTATATCATCCGCAAAATAATAGTACAAATACAAACTTGTAATTTGTA
GAAATTCCACTCAATTAAAACAATACTTAAACCGTAAAAAGTACTTGTAT
AAACTATTTCAATCCACTAAATTCTATGTCCCTCTCCCGTCCTCCACCTC
CATGATACTCTCTCTCCCATCCTCCAGTTCAACACTGCAATGCTCTGACA
CCATGCTTCGCGATCACCGCCATGGTCATTGATCTTTCCCCCGCTGGTGA
TCCGTGATCCAGACGTCCACCACATCTGTTGTCACCATGGCCATCTAATT
TTGCCAACACTTCTACGGTTAATGCACTAGTCTCAACAGCTCTCATCCTT
TTTCATCCACTACCATCACTGTCTTCGAGGAAGATTATGTTGTAATTCTT
ACTGTGATCAACAATCACAACATGAATTCATCACAAACAAATGCAAACCA

TTCACACTGTGACTCAACAATCACAATAAGAATTCATATGAATCACTCAA
CAACAATTTTGTGATCCTTATTTTGTCTCTAACAAGCTCAGTAAAATTTA
CAGCTAACGAACCTGGAAACATGACTAGGAATTGTAAAAAAGAAGAGAGA
AGAAGCGAAAGAGAAGGAGAAAGTATTTAAGAAACCACCACCCTCCACGG
TATCGTCCTCAAAGTTCTTTTCATCGACCTGGAGAACTCTGATGATGAAA
TAGAACCAGATCTAGGCTCCGCGGCGCCATCCTCAAGGTTCCGGTCACCG
ATTTGGGCAACTTTGACGAGAAACTAGAACCGAATCCGGTCAATACTCTT
CCAAATCTGGAAGTTGATCGGAAGTTCTTCCCCCCCCCCCCGGCACCTCT
TCATCCACGTAACCAGAAGGGGGATTGCGGGCATAGAAAGAGAGATAGAG
TATCGAACATTCGAGGTGCTTGCCTTCTCTAGTTGTCCGCCCTGAACAGC
TTGAAGCATCTAGAAGGGCATTATTGGCAGAAAAAAATGGGGTGAGGTTTT
TTATAAAAGACAATTTAAAAATTGTATTTTTGTCAAATGGGCTCGGGCTT
ATGTGATAGTGTTAATTAAACACTTGGAAATGGGGTTTGTGTGAAAAATT
CCTTTAGTAATATAGTTTTGGTATCATAAAGTGATAGGACTAGAAATTAA
GCCTTAAATTTGATGGAACTAAAACTTATTCAAATTCAAAGTTGGATGGC
TCTATGAGTTTCCCTTTCCAATTTTATGTGGAAGATACTGAGAAAGACTC
TTAAGGTCAACTTAGTTAAGCGTCTGCTGGTCGCATTTTAAGTCTTCAAA
CTAGTGTTTCAGCAGTCATACTTTTTCCTCATCCATTATAAGTTCTGTTT
GAAGATATGGATGGATGCCAACTGATGAAACTTGCTGCCTTTTCTTTCAG
ACAAAAAAATGGCACTGTTATTAACAATGAAAAGAGGTTCTCTATGCAAT
CATATGGCAGCTTTAGAAATAATAAATCACTGAAAGGGAGTTGGGGAGAA
CTCCTTGAAATTGCAATAATTACTGGTCGTAAAGCATAACACATGAATTG
CAGCAAAATGTTGTATATAATCCATTGCTTCCAAAACACTGCTGCTTCTA
CTCTCCCCTACTTCCACACTTTGATGAGCATTTTGATCCATTGTCACAAA
TTAATGGCCTTATGTTATTGATAATTGTTTAGGTTCCAGGATTTGTCATA
TTGTTTCTATTAGATTGTAGTAGACTTAAAATGATCATATACAATGCATG
TGTGCATGCATTAATTTATGGTTTTCTGAGTCCATTCTGCTATATTAGAT
TTTTGTTATGCTCTTTATGATCAAGTGTTTTTTTTTTTTTATAATTCCGT
AGTAATTTCTTATGTAGCATCAGTTACTGGGGCAAATAAACTGCGCCTGC
TATTGAAACAATGAAGATTGTTGGTCTGGTTGCATGAAATTCTGTAATTG
TCTAGGTGTTAAATTTATTAAATACTGACATGAAAAGTTGAATTGATAAT
GCTGCTCATTTTTAATTGTCAATTTGGACTGCATGCCTCTTTGGGACCTT
ACTTTGAGTTTTTATGTTCATCTGAACATTTCTGATGCTGACACAGATTA
TAAAGAATGCTGATCTTGAAGAAATACGGGAGTTAGGATCCGGTACATAT
GGAACTGTTTTTCATGGGAAATGGAGGGGAACGGATGTTGCTATAAAGAA
AATAAAAAAGAGCTGCTTTTCTGGGCGATCATCAGAGCAAGAAAGATTGG
TGAGTCCTTTGCATAATCTTACTTTTCCCGTTGTGTCTTTTCTTGTTTTC
ATCTCCTGCTAATTTTTTAGTGTGTGTTTGGGTGAAAGATTTGGGAGATG
ATTTTTTTTTTATAATGGATTTCTGAAATTCAAAGTTAAAATTCTTTGTT
TGGCTACTTCAAGGAATTTATAAAATTTCTTAAAATTTCTAAATAGGATT
TAAAGGAAGTAAAATGGCATGCCAAATACCTCATAAGGAGGGTTTTTTTT
TTTTTTTTTTCTGTAAATCCTTCTGAGTGAAGGAGATATCTCCACCTCAA
GTTGAAATTACATCAATAAAATTTAAATTTCAAAAGAAAAAAAGAAAAGA
AAAAAAAGGAACAACAATAAATTGCAAATAATGAGATTTTAAAATTCAAG

ATTTCAAAATCCCATTGTTTTTAAATTCCTTGAATTTATCAAATTTCTTA
TCCAAATGCATTCTAATTGTCACTTATTAATGATTGTTTTTCCTGTTACT
AGGCAAAAGATTTTTGGAGAGAGGCACAGATCCTTTCAAATCTTCATCAT
CCAAATGTGCTTGCGTTTTATGGGGTGGTACCTGATGGTGCTGGGGGAAC
GTTGGCAACTGTAACTGAATTCATGGCAAATGGATCACTTAGGCATGTTC
TACTCAAGAAGGATAGGTGAATAAGCATCTTATTGCTTGCTACATGTTTT
GGCTGCTTACTTATTGACAAAGTCAATGATTTATCGGGATTCTTAGTGCA
GGCTACTCGACCGGCGTAGAAAGCTTATAATTGCTATGGATGCAGCTTTT
GGAATGGAATACTTGCATTCGAAGAATATTGTTCATTTTGATCTTAAATG
TGATAATTTGCTCATTAATATGAGGGATCCATATCGACCCATATGCAAGG
TAATTGACAATCATGCACTTCTTAATGCACCAAGGTTGCGTTTGGAGATG
GATTTGAAATGAATGGATTTACAATTAAAATTTATAAATTTAATTTAGAA
TTTGTACTAAGAGATGAATTTTCACAAATTAAAAACACTCATTCATGAGT
TCAAATCGAAGCATTTGAAAGTATTTGGTGAAAGGGGTTATTTGAAATGC
ATCCTACTTGTTTTTTTTTTCTTCCATGTTTCTCTTTTGTGAAAACCACT
AGCATTTTCTTTGCAAATGAAAACACTTAAATTCTTTGATTTAAGTCCAT
TCATCCAAATAGAGCCGAAGGTTAAATCCAGATGTACGCTTTGATATTTC
CTGCATGTTTGCTGTTATTTGATTTTATCACTTGCATGCCGCACTTACTG
GTGTCCATTTTTTGGCTCATAAAAAAATGATAATTTGAGCTAACTGTTTC
CTCATTAACATGGTTTATCTCATAGTTTTATTGTACTTGGGGATCAGATG
TAGTGTAATAGAATTATGTTTTTTTTCCTATTTTTCTTTTAATATTAAAG
ATCATATTTTCTTCAATATGATTGATGAATATGAAGCCTTCCAGGTTGGT
GACTTTGGATTATCACGTATTAAAAGGAATACTCTTGTTTCTGGTGGTGT
GCGAGGAACCCTACCATGGATGGCACCAGAACTGTTGAATGGTAGCACTA
GCCATGTCTCGGAGAAGGTTAGATTAACTAAGGCACCGTTTGATAATGCC
TTTTTTTAGGTCTCTTCTTACCTTAAAGCAAAAAAATTAGAAAATTTAAA
GTGCCATTTGTTTTTCACTTAAAAAATCTAAAGTAAATTATGATTTTAAT
TACTTTTATTTTATAAAAGCAAATTATAATTTTTTTCTTTACTTTTGCAT
ATTTTGAGAAATTGATTGATTATTGTCCTTTCTACTCAAAGTCTCTCTAC
TAGCTAAGAAATTTATTTGGATGAGGAGACCTATAGTAATAACACTAAGA
TATCTTATACCATCAGACACAATTTCAATTTTTCGCTTAATTCAAGCAAT
TTTTTTTTTTTTCTTTGGGACAAATTAAATTGTTTAATTATTAAGATTAC
TTAAAAAAACGTCTGATAACTGTGCCTGTGTTAATTTCTATGAAATATGT
TAGCAGTTGGAGGTAAAAGGAAATAGTTTCTCAATACAAAAATCACTAAT
ATGCTGTTTCCCCCTGACAGGTTGATGTGTTTTCGTTTGGCATTGCATTG
TGGGAGATCCTGACAGGAGAAGAACCTTATGCAAATATGCATTGTGGGGC
AATCATTGGTAAGTCAGTAGTTTAGCTGATAACAATAAATTCTAATTATG
AAGTGAAAAAATAAAAATCAATTCATAAGCTGTTAAAGTAGTTTTTGAAA
CAAATATTATTAGATTTTTGTTCCATTATTGTTGAATCTCACATCGCTTG
TGTTCCTTGATCCCGAGATATATATAAAGACCAGAAGAATTCTCATCTTA
TAAGACATCTTTTGAGAGTGAGTTAGGCCCATTGAGGTTCTACATGGTAT
CAGAGTCAGAATTCTTGTCTGATGTTGGGTGTGTTGAATCCCACATCGAT
ATTGGGCTCCCCAATATTCGTTCGCAGTGGTTTGTCAAAAAGTTTGCCCT
GTACGTGAGGGGACGTGTTGAATCCTACATCGATGTTGGGCCCTTCCTCC

GATATCCGTTCGTAGTGGTTTGTCAAAATCCACTCTACGTGTGAGGGGTG
TGTGTTGAATCTCACATTGCATGCGTTATCTAATCCTGAGCTATATATAA
AGCCTAGGACAATCCTCATCTTACAAGACGTCTTTTGAGAGTGATTTAGG
TCCATTGAGTTTCTACAATTATATCTCAATCCGAGTGCATTGTGCATTTT
TTTTTTATTGAATTTTAAGAAAAAGGAATATGGTATGTTAATTTCAAGAA
GACAATATTTGCTTACTTGGAACTTTTGCTGAAACATATGATATTTCATT
ATCAGTGCATGTCATTTAAACAGCCTACATTTATGAAGCCACATTTGCTA
CTGCAATGTTTAGTAAACATTAGTTAAAAATTAAGAAAATCGGGAAACTT
TAGCTAAACTCGTGAAGATAGCGAATTACAACAGAAACCTGGGAACCTTT
TTTTTTACTGTTTTTGGAAATTTTATTCTCAAAAGTCTTGGAGCAGAAAA
AAATAGTTTTTTTTTTGGTTCCCATATATCTATTTCTGGGAACGTAAAC
ACTTAATTCCCTAATTAAATCAACCATGGCTTTTCTGAATCATTCTCTCT
TGTGAGGTTGCTTTTTTCTTTAGAAGGAAAAAATATTTAATTTTAAAGTA
GTGTTTGTTTTACACTAAAAGATGCTTAAAAAAAAAACATGATTGTTGTT
ACTTTTTTCGGAAAAGAAAAAAGGTTACTCTTTGTTTCTTTCATTTTTAT
ATTATAAAAGATTGACGGATTAGGACCTCTTGATCCAAATTTAAAGTTGC
TTTAAAAGTAAAATAAAATAAAATAAAACGGTAAAAACATGATTTTTGTT
ACTTTTTTTGGAAAAGAAAACAGGTTACTTTTTGTTTCTTTCATTTTTAT
ATTATAAAAGATTGACGGATTAGGATCTCTTGATCCAATTTTTTTTTTCA
TCTATAAATTTTTTATTTCGATTAACAAATTAGTTACAAAGATAATACTA
AGATATTATTTCTATACTAAAAAACACACTTGTCATTTTTTTCTTTTGAG
TGATTTTTTGCTTTTGATTTTATGTACTTTTTGTCCACTTAACTTTTTAA
ATTTAAAAAAGGATTAATAAACATTACCAAACGGTGCCTTAATTTGAATG
AGCTCTAATTTTCCTTATCTCCGTATATCTGACTCTTCCCAAACCCTGCA
ATGGGAGAGTCTTCGTACACTGGGTTGCCCTTTTTTATATCTAATTTTTC
TCATCTCCATATTCTAGGAGGTATTGTGAAGAATACTCTCCGGCCACCAA
TTCCAGAGCGGTGCGACACTGAGTGGAGGCGGCTGATGGAGGAGTGCTGG
TCAGCTGACCCTGCTGCCCGCCCATCATTCACTGATATAACGGAGAGACT
TCGATCCATGTCTTCAGCCCTTCAGGCCAAAGCCACAAAATAGTATGAAC
ATATGTCAAAGTTCACATATATACATATATGTGTATATATGCCTACTTTG
TTCTTGAGTGTCTTGGATGCTTTAGGTAGTGCTTGGTTGCAAGGAATTGG
AGTCTTGAAATTGGAAGTAGAAATGAACGCATAGACATTTTCCAAGTTTT
TGCACGTGTTTATTTCTAATTCCAATTCTGAGACTCCAATTCCTTATAGC
CAAACGCTTTGTCCTAAAAGAATCCCAGGTTGAGGAATTTGGAGGGGGCA
AGAGAAGATCATTTTGTTTTGTTCTTGTCTTTTGATGGTCTAATGCACAT
TGGTGTCTCATCTGATTTTGTTCTGTCAGTTCATTTGTTTATTCTTTTTT
ACTGTAAAAATCATGAAAAAGCTTGTGTGAATTAACTATGAGATAGATTG
CTGTTAATATGATTGTGATCAGAATACATGTATATGTTGATATAACGCAG
AAGAAAAATAGAATTTATGTTTGTTGTTTTATTTATTTATGTATTTATTT
ATTATTATTATCTTTGGCAACCTCCATTGCATAAAAAATACCCATTCCAA
CAAGTAATGATCGGCCGAAACAATTGAGTCATAAAAACTAATGTTTGATC
TTATCGTCTTATTACTATTATTTTTTTCGCTTCACATTTGAGCCATAGCC
TGCATCATATAAAACTTCTCTTAGATCATTATTTTACTTTGTTCTGAATT
TGTGGTGACTGAAGTGTATATTTGTAGAACTGCGTAGTTTTTGTATTTTT

TCATGTGCAGGGCCTGA

>EUC10175-RA [gene]

ATGAAGGAGGGGAGCGATGGGTTCGTGAGGGCAGATCAAATTGATCTGAA
GAGCTTGGATGAGCAGCTCGAGAGACACCTCAGCAGGGCATGGACCTTGG
AGAAGAACAAAAATCAGCACGACTCCGAATCCGCCACCGTCTCTTCCGAT
TCCTCCTCCGCCGTCAACAACAACAACGCCGTCAGGAGGAGACAGGAGTG
GGAGATCGACCCCTCCAAGCTCATAATCAAAAGCGTCATTGCTCGTGGCA
CTTTCGGCACCGTCCACCGCGGCGTATACGACGGCCAAGATGTCGCCGGT
CAGTTACCTCCCCAGTCCCCACCCTTGATTCCCAAAATTTATTAATTTTT
CCATACCCATTTTCGATTTTCTGTGCTTCCCAATTCTAGCTTTTCGTTAT
GCTTTTTTCTGATCAATATCCATACTTGTATATGCTTTAAATCTTTAATC
AGATCATATAATTGATTCCCATATGCATAGTTTTATTGATTTCTTATTGA
TTTTCTACTCTTCAAGTGAATAATTCAACACATTTAGGTCCAACGCTGCA
TCATTTCGACCTTTTTTCTCAAGGGTTGTTTGGATCAATTCAATTGGCCA
CAAAAAAAAAAAAATCATTAAAAAATTCCAATCAAAGGCACAGTCTTGTT
ATTCCTATCAATTTCCAACAATTTTTTTTTTTTTTTTCTTTTTCATTTTT
TTTATTTTTTTGTTTTTTTTTGGATGGTGAGGTGCTTTGATAATGGTGGA
AAGAGTTGTTATGTAATCCGTGATTCATACACGGGTTTAGTTGCGATATG
CAGGTGACCTTTTAAATATCATAGCTCAGGCTGGTGGTGTGTTTGTATTT
ATTTATGAACTTTTTCATGTGATATCCATACTGTTATTTTTGATAAAGTA
AGACACACCAATGAATTAATGATAATATCACATCGAGTCGTGGAAATAGT
ATCTTGCAAAGCAAGGTAACATTGCGTACAATAGACCCAAGTGGTCCGAT
CCTTCCCCGGACCCTGCGCATAGTGGGAGATTTGTGGCACAGAGTTGTCT
TTTTCTTTAATGACTTCAACAACAACAAAGCCGTTTTTTATCAAGCATTT
TCGATTTTTTTTCTCGAATATCAACGAGGTTTCTTAAACTAGCATATTAA
TCAAATGAGTTACTCCAGCTATGCCCATATGATTTTTAACATTGAGTCTT
TAGGCCATATCTAAACTCTCATGATAGTCGTTAATCACTCCACATGTAAG
CAGAGGTTACACGTGAGGGTCAGTGTTGAATCTGAGACTTCTCTAATTAG
CTTTGCATATGAGTCAAATGAGTTTCTTTTGAAGTAGTAAATAGTAATAC
TAATTTATTGTTAAATACAATGCTCTAGTTTTTGTTTTTCATATTTTTTG
GGTCCTGTTTTTAGTGCAACTTATTTTTTATTCCTGTACGACTGAATTTT
TTGTTGTTCTTACCAAGATTTTAGTTGACTTTAACTATGATATCATATGC
AAATTCAACGTCTAGTTGGTTTATTAAATAGATGTATTCAATCTACTTGA
TATTAATTGCAAGAGTAAAAACTTATTCAAAGGACTAAAAAAATAAAGAAA
AAAGAAAACAGGAACAAAAAATTTTTTAAGTGACTTATTTCAAGGATAAC
ATAATGATGTTTGAAATTTATTCACTTTAGTATCTATCTATGTATAAATT
AAAAATTAAATAAATGTACGTGTGTATTAAATTTTTAATAAAATTTGAAT
CAATCGTTTGTTTTATTATTTGATTAAGTGCTTTTCGCAGGATTAAGATT
TGGGTTCCTCTTTATTATTATTATTATTTTATTTTGTTGTTGTTGTGTGA
AAGTAATAATTGATATTTTTTTTTTTCTCATCCTGATCTCTAATCCTCAG
TTAAACTGCTGGACTGGGGGGAAGAGGGCCACAGGACAGAAGCTGAAATA
GCATCTCTAAGAGCAGCTTTCACACAGGAAGTTGCCGTTTGGCATAAACT
CGACCACCCTAACGTAACTAAGGTATATAACATTCTACATCTTTAACTTA
AGGAGCGTTTGGTAGCACAAAATTGAACGAATTGGAATTGTAAATAAAAA

GCCTATGACTTTTAGCCAGCATTTGGAGCTCGGAATTCAAGCCTTAGAAT

TAGAATTGGAACTGAATGCATGTAACTTTTTCAAATTTTTACATAGGTTC

ATTTCTAATTCCAATTCTGAGGCTCCAATTGCTTACAACCAAATACTACC

TTAATTTATTTAAAAAAATGCAAATTGAATTCAGCAACACATACTCTATG

GGTAAAAGTTTCATGCTTCAATCCATGACATTCATCCAATGAAATAATTT

GAACAGTGAGAAACGAGACAAGTAAGGTGCATAATCTCCTTGGGTCACAT

TCGTTTAGTATTTCTACTTGCACTGACCAGTGCTATCCACATGAAAAGTT

AAAATACAACAACAACTACAACTAAGTCTTTGTCCTACTTTAGTACGGTC

AGCTACATGACTCCGTTTACACTAAAGTGATCGATCAAGGGCTATCCTCT

CAATCAGGGTTAATGCCATCAATTCAATTTTAATAGTCTCGTTCCAAGTC

TTCTTAGGCCTACCTCTCTTTTTAATAGCCCCCCACATTTGAACTAAATC

GCATTTTCTCACTGGTGCTTCTATATGGCTTCTATGCACATGTTTAAATT

ATCGTAAACAATTTTCTCTCATCTTATTTGATGGGTGCTATTCTTAAATT

CTCTCTAGTAATCATTCTTAATTTATCCTCGTCTTCCCAATCATCCATCA

TAGCATCTTCATTTCTACCACACCAATTTTTTGGACAAGTTTCGTGTTTA

CTGCTCAACATCTGAATCATACAGAATCGCCAGTATTATTTCAGTTTTGT

AGAATTTTCCTTTGAATTTCAACGGTATCTTACGATCACATATCCCCCCA

ACTGTATTTTTTTCACTTTAACCACCTTGCCTTGACTCGATGAGTAATGG

CCTCGTCGGTTTCGCCATTGCCCTGAATCATTAAACCGAAATATCTAAAA

TGATAAACTTTTGGAACCTCTTAATCTCCGATGTTGATCGCAATATCATC

TCTCCGGGCATTACTAAAATTGTGATGCATATGTTCCGTCTTGTTTCTAC

TAACTTTCAAAACTTTAGATTCTAATGTTTCTCTTCATTTTTCTAATTTA

GTTTTAAGACTTATTTTATTTTCATCAACTAATACTATGTCATCCGTAAA

TAACATACTCCAAGGTACCCCATCTTGAATAAGTTTTGTGAGCTCATCCA

TCACTAGAACGGAGAGGTAAAGATTTAAAGATAATAGGAAATGCACAAAT

ATCGCCTCTTCCTGTTCGAACACTAATGACTATACCTTCATACATATCTT

TAATTACCTCAATATACACATTGGTACCTATTTCTTTTCTAATACCCACC

ACAAAACTTCTTTGGGTACCTGATCATAAGCCTTTTCCAAGTCAACAGAT

ACGATATGTAAATATTTCTTCTTTTCTCTAAATTTATCCATCAATCTTCT

AATTAAATAAATAGTTTCAATGATTGATCGGTTGGATATAAATCCAAATT

GGTTCTCTTATATGTTCGTTTTATGTCTTATTCTATATTCTATCACGTTC

TCCCAAAATTTCATAGTATTATGACTCACAAGTTTTATGTCCCGATAGTT

CGTATAAATTTGAACATCTTTTTTATTTTTATAAATGGATGCTAAGATAC

TCTTCTCTATTCATCAGGCATTCTCTTTGTCAACTAAATGTCATTTTAAA

CTCTAGACAACCAATCTATCCCAAGATCTCTCAAACTTTACCACACCTCT

ATAGGTATCTAATTTGGCCCTATTGTTTTTTCAATCCTCATCTTCCTTAG

TGCTATTCTAACTTCCAAATCCCTAATTCTTCTATGAAACCTATATATCC

TATTTGAAGATAAATTAGGCTACTTTAATCTAGATTCCCCTGCATTCTTC

TCATTAAATAAATTATAAAAAAACTTCTCCATTGTTTCATAATCTCCTCA

TCTTTTACCAGGATCCTTTCATCAACGTCTTTGATGCATCTGACACTTCC

TATATCGTTAGTCTTTCGTTCTGCTTTTCTAACGAGTTTAAATATATTCC

TCTCTCCTGCTTTTGTATTATTAAAATAAGTACTATTAAAAAGTAGTTTT

TGAATTTTTGAAAGTAATATAATATGATAAAGTAAAAAAATTAAATTACA

AAAGATAGTTTTACCTAAAGGTGTAAAGTGTGAATAAAGTTTTTCGTGGG

CTGGAAAAAGTAGAGAGAAGATAGATGGAGAGAAAAAATAGGAAGTTGGT
ATATAAAGTAGGAAATTATCTAGATATTATATTTTAAAAAATAAATAAA
TAAAAATATGATCCAATAAAAAATTGACACATCATTAAAAACATTTACAA
ATTAAAAACTCTACAATTAGGATTCACTGAGGGAAATCTTCTCATGCCTC
CTACATTCCATTACTAATATAGATTTATGTTCTTTTGTTTCCTTTCTCCG
AAAACTTGGAACGGATACAAAATATAGAAATTAGAAAAAAAACTGTTTTT
GTTCCGTTCGTTGATATATGTTCTAAATATGAGGGAACAGAACAAAAACA
TGTTTGTTATGTTTGTTTTGTTCCCATATTTTGCTCCCGTTCCAATTTT
CCAGGAACAGAAACATAAACGCTAAACAAATGGGGCCTTAGATACTAGAT
TGTGTGCCATACATGATATTAGTGGAATTTCAATTTTTTTGCAGTTTATA
GGGGCAACAATGGGCTCTTCGACACTAAACATACAGACAGAAAACGGTCA
CATTGGCATGCCAAGTAATATTTGTTGTGTGGTTGTGGAATATCTTCCTG
GGGGTGCCCTTAAATCTTACCTCATAAAGAACCGAAGGAAGAAGCTGGCC
TTCAAAGTTGTTGTCCAAATGGCTCTCGACCTTTCACGAGGGTAACCTTA
ATTTTTTTATGACGTTAATGCCCTTCCATAATATTGAAATGACTCTTATG
ACCTCTGATTTTTTATTTTTATTTTGTTGTTGTAGGTTAAGTTATCTTCA
CTCTCAGAAGATTGTACACAGAGATGTAAAGACGGAGAATATGCTGTTGG
ACAAATCACGAACTGTAAAAATCGCAGATTTTGGGGTTGCCCGTGTCGAG
GCCTCTAATCCTAATGACATGACTGGAGAAACCGGAACCCCTTGGTTATAT
GGCACCTGAGGTATGTGGTTGCCACTTTATTCGATTAGGAGTTAGGACTA
AAAACCTTAAAAAAATGTCTTAAAATTATCGATCATCATGGCTTAGCAAT
TAAACCATGGATTATCTTGCTTTTTTCCCTTTCGTTTTGGGGTTAAATGG
ATCATTTTTCTTAACAGAAATTAGTTAACTTTTATTTCTTTCTGAACTAA
AATTTCGAGAGGGTAATTATATATGATAAACACGGTTATTTTTGTAAAGA
TTGTTGGTGAATTATAAAGAATTGGTGTAACTACAGGTTTTAAATGGAAA
TCCGTACAACAGAAAATGCGATGTGTACAGTTTTGGCATCTGTTTGTGGG
AGATTTACTGCTGTGACATGCCGTATCCTGACCTTAGTTTCTCAGAAGTC
ACTTCAGCCGTTGTTCGTCAGGTAAAAAAAGAAAAAAGGGGAAATTATTA
TATTGTTCTTGAAATAATTCATTTATGATTTTGTATTCCCATATTTGTCT
TTCTATTTTCCAGTCATTCGAATAAATATTTAGTTCTCCGGTTAATATCA
ACTAAATTGAGTCCATCTGATATACGTGTTGAATTTGTATTCGATATAAT
AGTTAAATTCAACTCAAATATTGACAAAAAAAAAAAATCATTGAAAACAT
GAAAAATTTAAAATATTTAACCATACCTAAATATTTAATCGCAAAAATAA
CCATTCACTGTCAAAAAAGTTTTCTTTTTAAATTTTGGGTAATTTTTTGT
GTGTAGAATCTAAGGCCGGAGATACCAAGGTGCTGCCCGAGCTCCCTGGC
GAATGTGATGAAACGCTGTTGGGATGCGAATCCAGACAAACGGCCTGAGA
TGGACGAGGTTGTTTTCATGTTGGAAGCCATTGATACATCGAAAGGTGGA
GGTATGATTCCGATTGATCAACCTCAGGGCTGTCTCTGTTTCCAAAAGTA
CAGAGGCCCGTGA
>EUC08948-RA [gene]
ATGGAGCAATCAAGAAATAATGTCAATTTCAACTCCACAAGACCTGAAAA
TGAGGAGCTTGATCCGAGATCTCAAGGTTACACGCGAGACTCATCAGGCC
ACGTAATTGCTAATATAGATCCGGCAGAAATCAAATTTCCACAAGCTAAA
CCCGTGCTTAATTACTCTATACAGACGGGTGAAGAGTTTGCTCTTGAATT

TATGCGTGAACGGGTGAATCCCAAGAAACCATTTGTGCCAAATGTTGATC
CCAGTTATACACCCTGCTACATGGAACTAAAAGGGATTTTAGGGATTAGT
CATACTGGGTCAGAAAGTGGGTCAGATGTTTCCATGCTCACCATAGTGGA
AAGAGGTTCCAAAGAGTTAGATCGGAGGAACTCCTCTCTGTATGAGAATA
GAAGTAACTATGGGTCTGTGCAATCTGTGCCACGGACTTCATCAGGCTAC
AATAGTAATCAAACAATCATTCAAGATTATTCCTCTTCGGGGGCATCCGG
AAGCGTATCTACAAAAATGAAGGTACTTTGCAGCTTTGGTGGTAGAATCC
TTCCTCGGCCAGGTGATGGAACGCTCAGGTATGTCGGAGGTGAAAATCGC
ATCATCCGGATTAGCAAGGATATCTCTTGGCAGGATTTATGGATAAAAAT
AAAAACAATCTATGAACTCACTCAAACGATTAAGTATCAACTTCCTGGGG
AGGATCTTGATGCCTTGGTTTCTGTTTCTTGTGATGAAGACTTGCAGAAT
ATGATGGAGGAATGCAAGGTGATTGAAGTAGGAGAACGATCCAACAAGCT
TAGGATGTTCTTATTCTCCATAAGTGATTTGGATGATGCTCATTTTAGTC
TGGCCAACTCAGAGGGTGACTCAGAGATTCAGTTTGTGGTTGCTGTCAAT
GGCATGGACATCGGATCAAGAAAGGATTCGAATTTGCATGGCCTGGCTAG
CTCTTCAGCAAATACTTTGGATGAACTCAATATGCAGAATGTCCAGAGGA
ACAAAAGTGGAGCTGCAGCTGACTTTTTTAGTGATAATACTTTTACCTCC
ACTGGCTTTGTCACCATGCCACCAACATCTACATCTGTTTCACCTGTTGT
ACCAACTTCATCCCATGTTACTGAAACTGCCCCACAGTTGTATCACAGTG
ACATGGTACATCAGGACGCAGCTAAGGAAGATGACTCTCATTCTTCTCAA
ATATCTTTTTTTGGAGAAACTTCTGTTCCACAGCCTATTCAAAGTGGCTC
CGAAAGGCAACAATCCCAGGGTGAACAGGTTGTGGAAACACATGTGGAGG
ATGCTAAACAGAAAAATGTTGGTTCTGTTCAGCAGGGGCATGAACAGGAA
ACTATTCGGCCCTTGGCAAATGAGTATCTTGTTCCACCAAACCCTTATAA
TGGTAGTGTGACAGATTATTTTCCTGCTCGAGAAGTTGTTGGCCCTACAC
CGGAAGGAAATTTTTCTTTAGATAGTATGAAGAATGAGGCAAGGCCCCAT
GAACCTGTAGAGGTCTCTTCGGCCCTTGGTGCAGCAGAAAATCATAAATC
CATTGGTGAGGACTTTCACGCATCTAGTGGTGCATTTGCTCCTGGACCTG
TCAATTCCGAGAATGATCCTACTGATCCAGTTTTTGTTGAGCCTGACATA
CCTACTCAAAGAGTTTTTCATTCTGAGAGATTTCCTCGAGGACATGCAGA
GTCGCTGAACAGGTTATCAAAGTCAGACGACTCACATGGTTCTCAATTTC
TCATGACTCATTCACGTTCTGATATTGCACAGAAAGATTTAATCGTGGAA
TCTGCTGAAAAATTGCAGAACGGAAATTATGGTCGACAGAATGAGCTGGT
CAATACTGGCACTACAATTGATGGAGTTAGGAATCTACAACAGTTGAATC
AGTCAATTCCATCCCCTGGTAATGGTGAAAATGTTGTGCTCGAGAATCAG
ATTACTAAAACTGACCAGGGAGTAACCATAGATAACAGCGGGAATCTCCT
TGTTGACGAATTCGAAAAAACTGGATCAGAGCTCCCTGCTGTGAGCAGAG
TTGATTCGAGGAAGCACCATGATGAACCTGCTAGTAGTCGTCTGGATGTT
CAGTTGGGTGATACAGTGGGAAGCGATTCCACCCCTAGTAACACTCGGGG
GCTTGCTCCGACTTCTGCTCGGGTAGGGCGCCCAGCTGGAGATTTCTCTT
TGGATGAATCTTCTGTACATGCTCCTAGACCAGAGCGTGGTGACATACTT
ATTGATATTAATGACCGCTTCCCTCGTGATTTCCTTTCTGATATATTCTC
AAGAGCTGTACTTTCAGAGGACCCTTATAACATTGGCCCGATGGATAAAG
ATGGAGCTGCTTTGAGCTTGAACATGGAAAACCATGAACCTAAACGTTGG

TCATTTTTTCAGAAATTGGCTCGGGATGACTTTGCTCGAAAAGATGTTTC
TCTAATTGACCAAGATCCAGGATTTTCATCTAGACTTCCAAAAGTCGAAG
AAGAACTATCTGGTTCTGTTATACCTGACACTATAGCTTTGCATTCAAAT
TATGTTTCTTCCATTGCGAAAGTCAGTGAAGGAATACAGTACCACGATTT
GGTGGACAATCTTAGGATAGTGGAGTCAGAGTATGAGGTACTTTTCTTTT
GATGGTGTTTCAAGCTAAAATTCATTTTCTGAAGGTATGCGTTTTCCGCA
GCAATGTCCCCTAATCTCTTTGATGCTTTCAGGCTGCAATGAGGACCATT
GGCCTCTCCCCTCTAGATTCATGTTTGGCTGAATTCGATATAAATTCTGT
GCAGGTACGATGTGAGTCATTGAACTATTGAACCATTGCTATTTTCAAGT
GCTATATGGATATCATCTGGAAGTAGGGTTGAATCTTTAAACTTAACGTT
TTAACATAAATTCATTTTCTAACTCGTCCGATATTTGATTGTGGGACTTC
GTACTTGCAGTGTGTTAACTAGATATTATCATACTTGCAGTCGCTTAGTA
TGTACAGCTGTAGACAATTCTTTCTAGAAATAAGTAGTTCCTAAACATGT
GGTTTATACTGATAAATAGTTCAATTTGTGATGGTTGTTGGCTTAAGCTA
ATCAAGACTAAGTGAGGGAGTTTGCACAAATCATTTCTAGCAGTTCAGCT
CGATCAGGGGCATAGAGTTCTGGCATTCTTGTTGAAATCTAGGAAAAGAA
GTGTATCTGTCTTTGGATATAGAATTTTGACATATGGTGGAAGTTATTGA
GAAAAACTGTGCTTGTATTGATTGATGAAGTCATCCCTTGTCTTCATCTT
AAATATCAACTGCTTCCTATTCAGAGATCAGCTTATTTTTGCAGCATAAT
GATTTGTCATTTTATTTGATTTGTCTTGTGGAGAGGATCGGCCAAAGGTC
AACCTAAGGGTCTGTGTGATGAAACTAAGTGGTAAAAACTAAAAAGTAAT
GAATTTTAAGTGTTGAATTGATTAAGTAAATTTATAATTTAAGTGTTGAA
TTGATTAAGTAAATTTATTTTAAATATTTTATATCTCAATTAATTTGTAT
CTCTACAACTTTTTATTTTTAAATTTCTTTACAACTTGGTCTCTCAATAA
TATGCCCTCAAAGTTCTCTCTAATTCTTTTTCTCAGTAGTACTTTCTCTC
ATAAATTCTTCTCTCTAGATTTTCTAAAGGTTATAGTTCAAAGCTAGTTG
CATTAACAAAAAAATTATAATTAGTAAGTGATAAGTGGATGCACATCGCT
TAAACTTTTAAGTGATTGTTTACTTTTTATCAACACACTTCATTAATAAG
TGCTTAGGATTGAATTGTGTTATCAAATGCGGTTTAAGTTATTTTTCTGG
TTGCATTTAATAGATTATAAAGAATGAAGACCTTGAGGAGCTCCGGGAAC
TTGGCGCTGGCACATTTGGGACGGTCTACCATGGAAAATGGAGAGGTACA
GATGTTGCAATTAAGAGGATAAAGAAGAGTTGCTTCTCAGTTCGATCATC
AGAGCAAGAGAGATTGGTAAGTGGTTCTTTTCTTTTGTAATAATGGACTT
TTGTGTAGACTTACATGTAAACATGATGCCTTGTGGACACCTTTTATTCA
CTATAAGATGAGCAGCGAGTAGCCTTTGATGACAATTATAGCAAATGACA
TTTGATAACAAAACAAGGGTTTTGTTAGTCCTCTTGCCTTCTAATTTATA
CCTTGTTTCGTTCTATTTTTGACATTTGATAACAAAACAAGGGTTTTGTT
ACTCTGCTTGCCATCTAAGTTATATCTTCTCTCATTCAATTTTCTCATTG
ATGTCCCGTGGGCATAATATGTGTTGCATGTGTCTTCTTGCTTGTTCATG
ATATCATGCTTGTCTTACAATATTGATATTATTTGATCCATAGACCATAG
AGTTCTGGCGGGAAGCTGAAATTCTCTCAAAGCTTCACCACCCAAATGTA
GTGGCATTTTATGGTGTTGTGCAAGATGGGCCTGGGGCAACACTAGCGAC
TGTGACAGAATTCATGGTTGATGGTTCACTAAGGCATGTTTTACTTCGCA
AGGATAGGTACAATTTCCACCTTATGGTTCTTGAAATATATGTTTTTTAT

TTTGTTTTCGTAGTTCTAGGCACCATTCATATCACTAAGGCATGTTTTAG
TTCGCAAGGATAGGTTCAATTTCCACCTTATGGTTCTTGAAATATATGTT
TTTTATTTTGTTTTTGTAGTTCTAGGCACTATTCATATTTTTCCCTCAGT
TATTGAGTTTGTCTTTTCAACATTTTGACCAAATCCCAAACTAATAGGCA
ACTAGTTTTCTAATTGTCTTCCTGTTAGATAAATCAATACCTAAAATTTA
CTTTTAAGTTGTCATAGGCATACCAATGTCTTAACATTGTCCCATGGCAC
AAAGTTAACTTTGCTCCTATTGGCTGGGTAATTGTGGCTGAGAATTCCAG
AAACTGCTTGTGTGAAAACAGAGGTAATGAATGCTGCATTATACCTAACC
TTATTTGACCCCAAACAGGGGTGATGGTGTTGGCCACCTCTTTCTGTAAT
AATACTGTCATTTTGGTGAGGGAAATAAGGTTTTAAAAAATTGTGCTGCA
ATTACTGGAAAGAAAGATGATCTCTATTTTGTTTCCTTTGCTTGCTAAAT
GTGAACCATAACTTCTGTCATGAATGCCAGCACTTGATTACCCGCTCTTT
ATTTTTTCTTTTCTTTTTTGCCCTTTTCCCTTGTTCCTTCTTTGCAGACA
TCTTGATCGTCGGAAGCGGCTTATAATTGCCATGGATGCTGCCTTTGGGA
TGGAATATTTGCATTCCAAGAATATTGTTCATTTTGATCTGAAGTGTGAC
AATTTGTTAGTCAACTTGAAAGATCCTTCTCGACCCATTTGCAAGGTTAT
TCTGATGAAATTACAGTTACATGCCTGTGAACAATCTATCCTGTGCCACT
GTTACGAAGAAATGTTGCTATTATTTTCAGCACAGACAGTGTCTAATACT
ACTATTTTGCAGGTAGGTGATTTTGGCCTATCAAAAAATAAAACGGAACAC
TTTGGTTTCTGGTGGAGTTAGGGGTACTCTACCATGGATGGCGCCAGAGC
TGCTGAATGGTAGCAGTAATAAGGTCTCTGAAAAGGTGAGCCACCAGCAT
CTACATGTGTGTTTGTGTGTGTGTGTGTGTGAAGAGACTCATCCAGTCCA
TCCATCACAAAAAGATTGGGGGGGACAAAGGAGAGAAACCTATGTTACAA
ATAATGGACAGAAAGAATAAAGGGCATGGAATTTGTTTAAAGAACTGATT
TTGGTTGTGCATCCAACAGGTTGATGTGTTTTCCTTTGGTATTGTTTTGT
GGGAGATTCTGACCGGGGAGGAGCCTTATGCCAACATGCATTACGGTGCA
ATTATAGGTGTGTGTGCATATCTTTCTTCTTTGTTTCATTTATTTCCTTT
TAGCGCATGAGCATCAGAGCCTGGGATGGGGGAGTTCTGTAATTTGATTA
TTGTTGTGATTATAAATGGCAGGGGGAATTGTAAGCAACACGTTGAGACC
AACGATACCAAGCTACTGTGATGCTGAATGGAGAAAATTAATGGAGCAGT
GTTGGGCTCCAAATCCTGTGGCAAGGCCTTCTTTCACTGAAATTACCAGT
CGGTTGCGTGTGATGTCTTCTGCTCCCCAACCCAAAACCCACAAGCCATC
GAACAACTAA
>EUC07070-RA [gene]
ATGCCGCAGGATAGTTCTTCTTCCGAGCAAGAGCCGGACGACTCGGATGC
TGAGTTCGTCGAGCTCGATCCATCTGGTCGTTACGGTCGGGTTAGTTCAT
TCATAATCGCGTATGTATGATATCAGCATGCATTGAATTTCCTTTGTTTT
TTTGGTTTGCTTGAAATGTGATATGGTTCACTTATTGATGATTCGTGATA
CTCATTCTTTTCGCTGTGGTAGCATTGTTAGTTTTTGTTGAATTGATGAT
TTGACTGTTTTTGCATTTTAATTTCTAATTCATTGTGGTGCAATGCGCAG
TATAAGGATGTTTTGGGAAAGGGTGCTTTTAAGAAAGTGTATCCTTTTCA
CAGTCTCTGTTATGTACTTCTGTTTTACGGTAGCTTGTGTAATGAATTTA
GAAAGAACATAGCGTAGAACTATTGGAAATCCTTGACGGAATAATGGGAT
AAAGGTACCGAGCATTTGATGAATTGGAAGGAATAGAAGTAGCTTGGAAT

CAAGTTAAGGTTGCGGATCTCTTACGAAATTCGGAAGACATGGAGCGTTT
GTATTCAGAAGTTCACTTGCTTAAAACCTTGAAGCACAAGAACATTATCA
AATTTTACAACTCATGGGTCGACACAAAAAATGAGAGTATCAATTTCATT
ACCGAGATTTTCACATCCGGGACGTTGCGACAGTATGTTTTGTCATTTTA
GTGTTTTTTTTTTTTTTTTTTTTTNTGTCATTTTAGTTTTTTTTTTTTTT
TTTTTTTTTAGCTTGTTGTTTGTTTTTGTAGAGATTATATTTCATTGTG
GTATACTGATTGATTAATCTTCAAGCATTGTGCAGATATCGTAAGAAACA
TAAACATGTCGATTTGAGAGCATTGAAGAAATGGTCTAGACAAATCCTCG
AGGGACTTTCCTACCTTCACAGCCACGACCCTCCTGTCATTCATCGGGAT
CTTAAGTGTGACAATATATTTGTAAATGGAAACCAAGGTGAGGTCAAAAT
CGGGGACTTGGGACTTGCTGCCATTCTTCGCCAGGCTCGTTCAGCTCATA
GTGTAATTGGTGAGAAAATCACTTTTTCCTTCCCCTCCAGTGTTCCTCAT
TTTCTTCTCGTCTATCATTTTTCTTGTTGTATGACCATGAGACAATGTTG
TTGAATTTTGGTATTAGGTACTCCTGAGTTTATGGCACCAGAATTTTATG
AGGAGGAGTACAATGAGCTTGTAGATATTTATGCTTTCGGAATGTGCTTA
CTGGAGTTGGTGACATTTGAGTACCCATATATTGAATGCACCAATGCTGC
TCAAATATATAAGAAAGTCGTATCGGTAAGGCAGTGTTGACTATCTGGTC
ATTGACTTCCATTATATTTGTATTACTCTTAATATTAACGTATCGGAATA
ATTGTTTACGAAGTCTAGACGTGGAATACACCTCTTTCACTCTCCCCTTT
TCTAGTTGGGAATTTTACATGCTTTTTTTGCAGACCTCCGGAAATCGAGA
ACACTCCAAAAAAACTTAGTTAAGAAACCTTAATAATGTTGTTTGGGTCC
ATATTTGAGAGAACCTTTTTTGGATTAAAAGTATGTTTCCTGTGAAATAC
TATAAACACTTCCTCTTACTAGATTAGTAAATACTATATACAGCATTGAC
CTTTTTTCATGGAGTATAGTCTAGCATAATTGAATATTAAAAGTATGTTT
CTTGTAAAATACTTTAAACACTTCCTCTTACTAGATTAGTAAATACTATA
TACAGCATTGACCTTTTTTCATGGAGTATAGTCTAGCATAATTGAAAATA
AAGTTTTCTGCAAGATGGTGCTAGTTTATATACATCTCTATTGTCCTTGT
AAATGAGTAAATTGCACTTCAAGATAAGTACACTATCGCTCAAATTATAG
AGTAAAGCGCTATTAACAATCTGTTACACAAAAATATTATATAACATATT
TACGGTGATTTTATATTTGACCAAGTTCTTTAAAAAAAAAAAAATACTTAC
GCTTGTGCAATGATTCGTTGTTCTTTTATTTGATATATAAATTTTAAATG
AGAACAAATAATTAATAATAAAAGTACAACTGGCTGGAGAGCGTGAGAGA
TAGGATTGCACCATCCTCTTCCCTATCCCCAGTGCACCAATCCACCTCTA
AAACTCTGGCAGCTATTCCCTGGTGACGACCACCCCTTCCTAATTTTCCT
TGTTGACCACCCAAAGCCAACTACTCTCTTTCACCATATGTGCCATGCCA
ACTGGAAACTCCGATAACTTTGCCTAATATTTGTAAGTAAAGGGATACAT
TAGATGGTCTTATGAGACCCCAGAAAAACACCGCACTTCCACAAAAAAAC
AAAAGCATTCTTGATATAAATGGATCCGTCCAAAAAAAGCACAAGGTTAT
CAATATATTGTATATTTGCTTAAGTCCAATTTTTTAGTTTTTTATTTTTG
CATTTCTCAAGCTAAATGATCGAAAACAAATGTGTAATTACTATCTAGCA
AACCCTTTACCCACTCTGCTTAACACCTTAGTTATCAAGTCCTAACCTCC
CATGGCCCTTTCACCATAACGAAACACCTTCCCTACTTCCCACAGTTGTC
TTACCTCTTTCTCCATTTAGATTTGCCCCTCCTCCACTCATCCCGACTCT
TCCCCTTAATCATTCTACCATAACGTACCTAAACTCTCCTGCCCACCCAT

GCACCCCTTCATATTATCCCCATTGAACTCAATTTTCTTCCGTCTATATT
GCCCCCTTCCACCGCAACATCTCCATCCCCATGTACAAAACATCTCTCTG
TCATTTTCTCCTTCTACCTTTTTCCCTCTTTTTTTTTTTTTGTCAAAACT
ATAACTAGATACTAAGGAAGAGGGCCTTCGTTGTCACGGACTTTGTTCTT
CCTTATAACTATTTTGCACATGTTTTGCGTGTGCCTCCGTCTCTAGTTTG
TAAATAATTTAAAAGTTGTATAAGAGGCACTTTTGTCTATTAATTTTTTC
CTAATTTTTTTGGAACAAACAACATGTTTTCAACATTTTTCGTCAAAATT
GACAGTCTTTAAGCATTGCAATTTACTTTGAAGATAAAAGGGCACTATTC
TCAATAGAAGGGAGTAAATTGCAGAAGTCTTGTAGCACTTTAATTTAAGA
AAAATAATTCATTTACAATCACCGAAATTTTACATAACCAATAAAATATA
TTAAACAAATATTGGGAAGCTACATTTGGAGTGGCAAGAAACAAACAATC
ATTATGTGGACTATCATTACAAGTTGTATCGTAATAATTTTGAGTGGTTA
ATTTTATAATTTCTTTTTCATTTTTTCTTTCTTTATTTATTTTCTCTATA
TTTGAGTGCATTACTACGTGCACAAATTTGGTCAAGGGTATGGATGTGGG
TGTGCAAGTGAGAGAAAAGTGTTGTGGAAAAAGTTCGATTTCCAGAAAAA
ATTGTATATTAAGCAAGGTTGTCAAAATCGCGATCTGAATCATAGGATCA
CATGGATGTAAGGCCCGAGTTCAGCATAATGACCAAATCGCAATGGGATA
ATTCATTTAGCAGAATTGTTAGGTAGAATCTCCAGATTTTGAATCAAGCC
CTGTTTCTGAAATTTTAAGTTGGACTTTGGACCAAAAATAGTAATTAAAC
CCTTTAAAATTAGCGCTTTACCGAAAATTAACAAACTACAGAAATAAACC
TATACAAAATACCCTTACCTTTCAATTTTAAATTATTTCTGCAATTGATG
TGAAGACGAATAGATGATCGTGCTTCACGAGAGGCAGAACAAAACGTAAT
AGGTTTTTCAATTTTCCTTTCTTTTTCATTTTCAGTTCGTTTTTCGTCTC
CTCCTTTCAATTTTCTTCTTCTGTCATTTTGTCAACTTTTCTTTTACGTA
ATGTGTTTATCATTTTGTTTTAGGACAACTTCTTCAAGAACCCACACCCC
AAAGACATTATATAATTGTATTTTTTTTTAACACAAGCACACGGTGGGAC
TAGAACCCCACACCACTTGTGTGAGGATGAGGTAAGCCTACCACTAGGCT
ACCCCATGAATCCCCATTATATAATTGTATTACTGTTTTGTTTAAAAGTT
AAAATTGAAAGCAAAATAATGTTATGCTTAAAAGTAAAATTTTAAGTAAT
ATAATATTTTGTTTAAAAAGTTCATAGACAGCATATTTTTGTCTTCCCAA
AACTTAGGCGTTTCTGCCTATCTTTATAGAACTTTGGAAAGGTAAATACT
GTGAAATGAGATCAGTAATATATTTAGTTACAGGAAAAAAATAGTGTTATT
ATTCTTTATCCACCACTTCATATTTTTGTTTAGATATGCTTAACCCCCTT
CAAGAAATAAGAGAAAAAAACATTAGGTTGCAGAGTCTTACGATCCTCGA
TCCGATTCTCTAGTACCCCAAAATGATTCGATCTAGGATCCCGATTCGAT
TTTGACAACACATATCCATATTCCACAAGTGGTCTTTATGAACCTACTAC
TTTGACTCACTTTCAGGTGCTTAGGATATATCATATAGCCAATACATGAA
TCTTTTAGTCGGCGTCTGCCTTACCTCTTCTCTTGTTGGAAACATTGTAT
GTGAGGCCTTTGGCAAACTCTATGTTGATTCGTGTAAACTTTAATTTTTA
TTTTTTTTAATATATTTTCAACTAGAAAATAATTTATTTCAAATGTTAAT
TTCAATAGTTGCTATAAACCTCAAGTATGTTGAATACTATATTTGGAGCT
ACGAGCAATAGGTCGAGTTGTGGTGGCACTTGCCTTAATAAATCTTACCT
CTTAAATTAGCCCCTTGAATTAGATAGAAGTAACTAATTTACCTTGTCTA
TTCTTGCTTGCTCATTAGCGATTCCAGCTGCCGTGCCTGACCTGGCCAAG

GCACCATGTAAGTACTACTAGTACTCAGAAGAAACAAAGGTTTGTACTGA
AAAAGGAAAAGAAGAAACAAAGGATCTGAAAAAACTACTAGAGTGGTATA
TTTGTTCCTTAACTGCACAATTCAAGAAGAAACTACTAGTCTGTTGGATA
CAATTCAAGATATTTGACTTGTTGCCGTCTTTTTTTTTCTTCATGTGT
GAGAATGGGGGGCAAAGTTCTTTCGCTATCATACAGAGATACGAGGCATT
TCCAATGAATTTATTACTGTAAGAACTTTCAGGAAAATTGAGAAATAAAT
ATTGCGAAGCTTCATAAAATAGGAAAGAAGAAATGAGTTGATGATTTGGC
GGGGCTTAGTGTTGATTATAATAATATTTGTGGTCGAATCCTTGGGTAAG
ACGTCTTCCATCACTGTTGGACATAAACTTATATCTTCAGCAAAAGGGGA
GGCTTTTTGTGGTTCTGTGGTACTTTCTCCGGAGGGCTGCTCTTGCTTTA
ACATTGGAGAAACAGTCACTGTCAGCACAGCCCTCTCCAAATGTTAGTTG
ATATGATAAATTAAGAACTAGTCAACTAGCTAAGGGATGATTGCTACTGT
ATAATCCTGTACTTACTATGTTGTTTTCCTTCAGTAGTTTCTTATTGTAC
AGAAAAAGCTGGAATTGTGGAAGAATTTTATGTAGTTGCATTTTGTTGAA
ACGGTATCTGTTCAATATCAATGACGAGATGTGCTCTTCAATATCAATAA
ATCTTCATGCTTCGCTTTTATTCATTTGTAGGGAATCAAGCCGGCATCAT
TGGCTAAAGTGAAGGATCCTGCAGTCAAAGCATTTATAGAAAAAATGTATT
GCACAAGTGTCTGAACGGTTGCCTGCCAAGGAGCTATTGATGGATCCTTT
TCTGCAGTCAGATGAGAATAACGGAAGCATAGGTCGATTCCTTCAACCAG
ATTTATATCATGCAGGTTAAAAACAAAATTGCGGAATTTTTTTTTGTAAT
ATTCCATCGCACTTGTTTTAATTTCTTTAGTTTCTGACAATAAAATTTAC
AGACAGTATTACTGACCACTTTGAGATCAGTACAATCCCCAAGGATTCCG
TGCCTGATGAAAGTCGAGACTTCACACTGCAGGGTCAGAGGAAATACCTC
AACACGATATTTCTAAAATTACGAATACCAGAATCTACAGGTTGTTTAGT
GTTCTTTGTTTCTTTTAAGTCTCCATCTGGATGGATGGATGGATTTGAAA
TCAAGGGATTTAAGCATTTTCATTGCAATAAAATATTGTACGTCAGAGTA
AATAGTATTAAATGGGAGTATAGAACTCTTAAAGCAAGTTTGGACTATAA
GATCTAAGTTAGTAGGATGAATTTCGAATACTTCTTTTAGCAATTCATTA
CAAATTCCACCATTTGGCATGTTTGTAGTTTGTTGATATTCACCTCTTAG
TATAAACTTCAGAGCTTTTCTCACTTTGCACTCCTAAAGTTTCAGAGAAA
TCTCACTTTAGCCTCTGTCCTTTCAATTTGAACACATTAAACCCTGAAAT
ATTTAAATCGTGTCAAGTCAGTCCTTTTGTCATTTTTTATGACGAAATTC
ACCCACATGGGGCGATTCCGTGTCTGACACGTGACTAATTTCCGTCATAA
AAAATTACAGAGTTACCGATTTGGGCTAAAGTGGGATTTTTCCAAAACTT
CAGGGGTGCGAAGTACAAAAACCTCTAAATTTTAAATTCAAATTGTAATT
TTCAACTGTAAATCCATTCATTTCAAATCATTCTAACTGGAGCCTTACAG
ATTTCTTTTTCTTTTGGTTGCGAAATTTGTTCTCATTGCCATTATAAAAA
CATACATCTTACAAATTGATTTATGCACTTAGGTTATATTCGGAACATCC
ACTTCCCATTTGATGTTGAGGTCGATACGCCAACAGCTGTTGCAAGTGAA
ATGGTTGAAGAGCTTGACCTAACGGAGCAAGATGTGCCAACCATTGCTGC
AATGATCCAGTCAGAAATTAGATCATGTATCCCGGAGTGGGTTCCCAGAG
AAATTTCTGGTGATCATACTTGTAATGAAGTTACAATAGCCGATACATGT
GGCTCTGAAGAAGCACAGAGCAACACTTCACCCTTAACTAATGAGTCTTC
TCCCCATTCCGCCGGGTTTTTTCTAGAACGAATGCCTTCAGGTCGGAAAT

ATTGGTGCAACTCAACAAAGGGAAGCGGTGGAAGCACCCCACTAAGACAA
CCGTCTTCAAATTTGTTGCGTGCTGATTCAGTGATATCTGGAGATAGTTG
GGCTGAAGAAAACTATCATTCGCCTGAAAGTAGTAAAGAAGGGAGCAGCT
TTGTAGAGGAACAGGCTAATCTTCCTGCTGATTCACGTTTAGTGAACCT
GATAGTGATTCTGACAAGGGACATCAACACATTGGAGGAGGAAATAACAG
AGCGTTAAACGATACCGTTTCGGATGATGTCAAAATCATCATGGAAAAAC
TCGAGAATCTATTGGTTGAGCAGCGAAGGGAGCTCGATGAACTCAGGCAG
AAGCAAGATTTGGCTGTGTCGGATCTTCTGAAAGGACTTCATCCAGAGGT
TCGCAGGAGAGTTTGTAACATTTGTAGTTTGAAGATTTTTGTCCATAAAG
TTCCTTGTGCTGGCTGTTCTATTGAGAATCGCGCCGATCTTTGCTCATTG
CGCATGTAA
>EUC12664-RA [gene]
ATGCCTCCATGGTGGAGCAAGTCTGCTTCAAAAGACGTAAACAAGAAGGC
AAACAAGGAGAGTTTTATTGATACGATACACAGAAAGTTCAAGATTGTCT
CCGAGGGGAAGTGTGGAAGTAGGGCATTTTCGGACAAGGCTTCTCGTAGA
AGAAGATCCAGCGACACATTTTCAGACAAAGGTTTATCGCCATTGCCATC
TACTCATGTTTCAAGGTGTCAGAGTTTTGCCGAGAGGGCTCAAGCTCAGC
CACTTCCTCTTCCCGGTTTACAAAATCCAGCAAATCTCCGACTGGCCGGA
AATAGGGCGTCAAAAAAATCAGGCAGTGAAGAAGGATGTAAAACTCCAAA
TTTGCCCGTCCCTAGATCTGGAAATAGTCCTAATGTGTTGAATGAGGGGG
ATTTAGCGACTGCTTCTGTTTCGAGCGATAGTTTGAGCGATAGTGATGAT
CCGTTTGAGTCACGTCTCTTAAGCCCTCAGGTGTCTGACTATGAGAACGG
GAACAAAACTGCCATCAGCAGCCCTACAAGGTAAATTTTCGAAAGGAAAA
AAAGAAACACAAAGAATAATGGGGAAATTATTAGATTATCCTTAAGTCAT
AATTTTTTATCCTTATTTTTTCTCTTTATTCTTTAGTCCTTTACTCTTCC
GATCAAGTTTTTAGTTTTTCAGTTAATATGAACTAGATTGAATCTATCAA
GTTGATATCAACGAGATGTTGAATCTGTATATCATAGTTGAAGTTAACTA
CAATATTAATAAGAACTAAAAAATAATTTTCTTTGTAAAAATTGAAAACC
GAAAATAGATTACTATGTAATAATCCCAAGAATAATTCATAGTTATTTTT
GTTATGTCAATTCTAGTTTTATTCTATACCTGACTATATATATTTTTTTT
TCTTTTTTTGCTGAAGTGTGAAGCACAAGGATCAATCCCCACTCACATCC
CAAATGAATGCAAGAATGGCTAACATGTTGTTGAGTAATCAAACTCAGCC
TGTCCGGGCATCAGCTAGAAGGGTGCCGAATTTGCAGATTCCAAACCCTA
ATATTTTTCATAGTGCGCCAGATAGTTCAATGTCAAGTCCTTCAAGAAGT
CCCATGAGAATTTTCGACCCTATCACGAGTCCTTATTTCTATTCAGGACA
CTGCTCTAGTCCTGGTTCGGGTCAAAATTCAGGGCATAATTCGATTGGAG
GAGACATGTCAGGACAACTCTTTTGGCCACACAGCCGTAGCCCTGAGTGT
TCGCCAATACCTAGCCCCAGAATGACTAGCCCTGGACCAAGCTCACGAAT
ACATAGTGGTGCAGTCACCCCATTGCATCCACGAGCCTGTGGGTTAGCCG
ATGATGGGAAGCAACAGAGCCACAGGCTACCCCTTCCACCATTAACGATA
CCTAATTCTTCTCATGTTTCTCATTCGTATTCGACTGGAACGACTCCTAT
TGTTCCAAGAAGTCCTGGTAGAACGGAAAATCCGCTGAGTCCCGGGTCAC
GTTGGAAGAAAGGACGATTGCTTGGTAGAGGCACATTTGGGCATGTTTAT
CTTGGCTTTAACAGGTTTAGTTGCATTTTTTTTTTTCTTTATTATATTAA

GTTGTTTGCAATAAGATGTTAGTAGTGCTTAATTTCAACCAATTAAAATG
GGTTTAATTTAATTTTCTTGATTCCTTCCCACAGTGTACTTTGTTGTGTT
TTACTAGATTTATGCTCGTGCTATCCACGAGCTACCATAGTTTCTTTAGA
AGGCCAAATGTAATGCACTACAATGAAAATCACTGCAAATTTTCCATGTA
AAACATTACAATAATTTTCATGTAGTGTAGTGACTTTGGACTTACGAGAA
TTAGTGGATTTCTCAGAAAATTGTTGTAGCTAGTGGGCTGCAAAATTTGG
GCCCATGGCAAAACCCCCATTTTTTTTATTAATTTGTTTTTTTGGGGGGT
GGGGGTTTAGTCCCTTTTATTGAAAAATTAAGGGGTAAAAGTGCTATATT
TTTAAATATGCCACTATTTGCTGCAAAGATGTTTTTCTCCTTTTTACATT
CTCTTTTTAATAATCTGATTTTTATTTGTTGGGTGTAGTGGAAGTGGTGA
GATGTGTGCAATGAAGGAGGTGACACTGTTTTCCGAAGATGCAAAGTCAA
AGGAAAGTGCGCAACAACTAGGGCAAGTGAGTTTCTGTTCTGCGTAGTCT
TCTGAAATCATTACTTAAGACAGATGTTTAGGTGTTGTTTGGTTTAGTTT
TTCGAAGAAAAAAAGTTGGAAACGGTTTTTGAAAATCTCGAAAAGTGTTC
TCGTTTAGTTTTGGAAAAATTATGAAGAGATATTGATTTTTAATGTGTTA
AAAATAGAAAAGTAAAGGTGATGTGATAGATTTGAGTTTTGCAGAAATTG
TTTTTTAAAATTGTTTTTGAGTTGTGTTGTTAAAAGAATAGCAGTTGAAT
TAACGAAACCAAACGGACCCTTAATTCTTGCAGCCAATTTTTTGCAGGAA
ATTGCTCTATTGAGTCGTTTACGGCACCCGAACATTGTGCAATATTATGG
ATCTGAGACAGTAAGTACTGAAACTTTTCCCGGAAAACTTAGAACAGAAA
CAGAAATTTGGGAACAATTCTTCTCTTTTTTCAAAATTTTGTTCTGAAAT
CCCGGGAGCAGAACACAAACTGAATATTGTTTTCATATCCATATTGTGGT
TTCTGTTCCCACCTTTCTTGGAACAAAAATGGAAATATGGGAACAGAAAC
ACTAAGGCCCCATTTGGTTATTGTTTCTCTTTCCTGATTCCTGTCCCGCT
CCCAAAATTTTGGGAGTAATTTTGGACTATTTCCTGAATTTCTTAAGAAT
TTGAGAAGCAAAAAAATATTGATTCTTATTCACTCCCACAAAACTATTCT
CACTACTATCCCGCAAGCAGCCACCACCCCTCCGCCGCCACCATCTGGCC
GGGCCACCATTCATTTAAAGAAAAAAAAAAGAGAACAAACGTTGCAATCA
AACGTGTTTCTTATTTTTGTTCTCATAAATTGAAGAAATAATTTCAGAAA
CAATTTCAGGAGTCAAGACAATGGTCCTTACCCAACAAATTTCTATTTCC
GGAATACTCCAAAACGGAAATAGAATTCTCATCCCATCCCAGTTTATTGG
AAGTAGAAACAAAAAAAACAGAAACACTAACCACACGCCATCTAAACAAA
TAGTGTTTTTGTCTACACAGAGTCACTAAGCTGGAACCGAGACCTTACAC
CATATTAAAAACATCATCTTTGGCTATTCTTTTTTTCCTAATATACAGGT
AGATGACAAACTATACATTTACCTGGAATATGTTTCGGGCGGTTCCATTT
ACAAGCTTCTTCAAGAATACGGCCAGTTGGGCGAAGCAGCAATTCGAAGT
TATACTCATCAAATTTTGTCCGGGCTTGCTTATTTGCATGCTAAAAATAC
TGTGCATAGGTGAGTTCGCTTAGGTAGCGTTCAAACGCGTGGAATTATTG
GAATTAAAAATGAACGCACGCAAAAATTAGAGATGTACAAGCGTTTATTT
CTAACTTCAATTTCAAGGCCTCAATTCCATACAACCAAACAGTGCCTAAT
GTTTGTTGGTGTTTATATTATTGGCTCTTCTTATTGGTTATTTTATTTTG
TCTAATTCAGAGATATTAAAGGTGCAAATATACTGGTTGACCCCAATGGC
CGTGTGAAATTGGCAGACTTTGGGATGGCAAAGCATGTAAGGCAAATCCT
CGAGCTTTTTTAGATATTATTATGTTGGCTGTGAAACATATCCGAAAAGT

CTATAATCGGTGAGAATATATATATATATATATATATATAATAAAAAAAAAT
GTGACGGTATTCTTAGGAGACATGGTGTTGAAAAAATTATTTAAGCTGC
TATCGATGTATTGAATAAAGATAACTATTAAAAAACTTGTACGCTTGAAA
ATGTTTATGTCGACAAAAAATACCAATTTTGCCCTTACGTTATACGAACA
ATGCAGAGTAGACTTTCTATTGTGAATTTATTGATGAGTCTTTCTGTGGT
TTGTCTGATTCTAAGTGCTATAATTTTCAGATCACTGGGCAGTCTTGTCC
TCTGTCATTGAAGGGAAGCCCTTATTGGATGGCACCTGAGGTTTGCATAA
TTATATAGTGATTCTCTCCTAAGTGTTCGTTAGGATCCATTTGGATTGAG
AGAAATGGAGGGAAGAGAAGAGAAAAGTATTGTTTTTTCTTAGTTTCACT
TGTTTGGTAATTTTAAGGAGGAAGAAAGAGAATTGGAGAGAAAGTTCCTC
TGTAAAGCGAGAAAGATGTTTTCTTCCCAATCCAAATGGGCCCCTAAGAG
TGCGTTCTAGAAAATGGGTTTGTATTATTTTCTTTGAAACAATCTTTGGG
AAATTTTACATATCCATTTTCTAAATTGAATACGTTACCCAAACGCACTT
ATACAATGCTCGCTGAATAATATTTTGCATTTCTAATGCCCTTTCAGGTT
ATAAAGAATTCAAACGGTAGCAATCTTGCTGTGGATATATGGAGCCTTGG
ATGCACGGTTTTGGAGATGGCAACAACAAAACCACCTTGGAGTCAATATG
AAGGGGTTAGTACCTATTAGATTGGGATATAAAGCGCCTGTTTGGTTTTG
TGCTCGGAAACAATTTTTTTTTAAAAACCAAGAACAATTTTTGATAATTG
TGTAAAGTTGTTTGGTTTCTTTTTGAAAAAGTTTGAAAAAAGAGAACATT
TATTAACAATGAGTTAAAAAATTGAAAATTAAAGTGTGATTGATATAAAT
ATGATGTTGAAAAAGACTGATAATAATGATGTTTGTTGAGTGAATGTGTG
GTACAAAAAGCCTATGGAGAAATAAAAATAAAAAATATTGAGACAAAAGT
TAGTTTATAAGATAATAAAGGGCAGCCCAGTGCAAGAAGGCTCCTGCCGT
TGCGGTGTCTGGGGAGGATCAGATGTATGCATCCTTACCAATATAAGATA
ATAAACGTATAATAGTAAAAGTAAGCGATGTACCTAAGAGGTGTTAGTCT
GATTGGCAAGGGGCGGTGTCTTCTTTTCTTGTCAACCAGGGTTCAACTCT
CCTAGAGAGTATCCCGGAAATTAAATCTTGAATTTAATCCTGACTGCACC
AGGTCAATGTGGGGGAGTACATCCTTACGATCATTCAGGGAGGTGATAAG
TCGAGGTGCGCGTAAGCTGGTCTGAACAACATCTTACCATAAAACTAAAA
AAATAATAATAATAAATAAATAAAATAGGCAATGTGACCAATTGAATATT
GTGAAAATTATTTTTTAAAATTGTTTTTGAGTTGTGTTTTTTAAAAGAGA
AAATTGAAACAAAGAGGGCCGTAATGATTGTGTTTTTGTTCATTGAAAGG
TTGCTGCGATGTTTAAGATTGGGAACAGCAAGGAACTCCCTACAATCCCT
GATCATCTATCGGATGACGGTAAAGACTTTGTTAGGCGGTGTTTGGATCG
CAACCCGCTACACCGTCCTACAGCTGTAAAGCTGTTGGAACACCCTTTTG
TGAAAAATGCTTCTCCATTGGAAAGACCAATTCTATATCCTGAAGAAGGA
TCATCTCCAGCGACAAATTCAATAAGATCACTCGTATGTGCTCTTCTTTT
TTTGTTATGATGTCTCTTAGGCATCGTTTGGTAATGTTTTTTTCAAGTAA
TTTTAAAAATCAAGAATGTATTTTTTTTTTTGAAAAGCAAGAAAGTAGTA
GTTTTTTATTTCTTTGACTTCTATATTTTAAGAGATTGATGGACTTGGAT
CTCACTACCCAAATTTTTTCAACCCCCTACAAATATCAAACACGCTTTGA
TTTTTGTTCTTGTTTTTGTCTTCAAGTAAAAGTACATTTTGTTCATTAAC
TTTTTGTTTTTTTACCAAACGGTGCCCAAGCTTTTAGAATTATTACTAAG
CGTTAACCTGCCAATTTTCTCAACTCACTAATTGATAAAGTGGTCATCAT

GCTTGATGTTTTTATTAGGGTATTGGAGGATATGCCACAAGTTCTTCACT
CTTTGAATCATCTGCTTCTCATCTTTCTAGAAGTCCGAGAAATGGTTCAG
CCTTCAGGTCTCTCTCTCTCTCTCTCTCTCTCTGTGCGCGCGAGCGCGCG
CGTGTGATGGATATATTTTAGATCTCCGTATGCTAAAAAGGAAAAAAGAC
AGTTTTACATTCATTCTGTGTTTTTTGCTTTCATGCTCTCTTAGGGGGTT
TTAAAAACGCAATTTCGGTAACTTGGACAGTTACTAATTCGTTTCTTGTA
ATCAAGAAACGAATTAGAAATTAGAACCAAATTCCGTTGGCTGCCGTTTG
TTTAGGCCCCGTTTGGTAACCATTTCCATCCCATTCCATTCCCAGATTTT
TGGGAATTCTGTTTGGAAATAGCGTTTGGTAATTTTTGTTTCCGTTTCCA
TTTCATTCCCATTTGAGAATTGTGCCAACTTTTTTCTTAAGAAATGAGAA
ACAAAAAAAAGTTGTTTCTCATTATTCCCATTCCATTCCCAAAACCCATG
ATCACTATTCACCTACCGGCCGACCACCGACCGCCGCCGCCGGCCACCGT
CCGCCGGCCGCCGGTTTATCTCCGGCGACCGTCGCCGGTCACCGTCCGGC
CGGAATTTTNNNNNNNNNNNNNNNNNNNNNNNNNNNNNNNNNNNNNNNNN
NNNNNNNNNNNNNNNNNNNNNNNNNNNNNNNNNNNNNNNNNNNNNNNNNN
NNNNNNNNNNNNNNNNNNNNNNNNNNNNNNNNNNNNNNNNNNNNNNNNNN
NNNNNNNNNNNNNNNNNNNNNNNNNNNNNNNNNNNNNNNNNNNNNNNNNN
NNNNNNNNNNNNNNNNNNNNNNNNNNNNNNNNNNNNNNNNNNNNNNNNNN
NNNNNNNNNNNNNNNNNNNNNNNNNNNNNNNNNNNNNNNNNNNNNNNNNN
NNNNNNNNNNNNNNNNNNNNNNNNNNNNNNNNNNNNNNNNNNNNNNNNNN
NNNNNNNNNNNNNNNNNNNNNNNNNNNNNNNNNNNNNNNNNNNNNNNNNN
NNNNNNNNNNNNNNNNNNNNNNNNNNNNNNNNNNNNNNNNNNNNNNNNNN
NNNNNNNNNNNNNNNNNNNNNNNNNNNNNNNNNNNNNNNNNNNNNNNNNN
NNNNNNNNNNNNNNNNNNNNNNNNNNNNNNNNNNNNNNNNNNNNNNNNNN
NNNNNNNNNNNNNNNNNNNNNNNNNNNNNNNNNNNNNNNNNNNNNNNNNN
NNNNNNNNNNNNNNNNNNNNNNNNNNNNNNNNNNNNNNNNNNNNNNNNNN
NNNNNNNNNNNNNNNNNNNNNNNNNNNNNNNNNNNNNNNNNNNNNNNNNN
NNNNNNNNNNNNNNNNNNNNNNNNNNNNNNNNNNNNNNNNNNNNNNNNNN
NNNNNNNNNNNNNNNNNNNNNNNNNNNNNNNNNNNNNNNNNNNNNNNNNN
NNNNNNNNNNNNNNNNNNNNNNNNNNNNNNNNNNNNNNNNNNNNNNNNNN
NNNNNNNNNNNNNNNNNNNNNNNNNNNNNNNNNNNNNNNNNNNNNNNNNN
NNNNNNNNNNNNNNNNNNNNNNNNNNNNNNNNNNNNNNNNNNNNNNNNNN
NNNNNNNNNNNNNNNNNNNNNNNNNNNNNNNNNNNNNNNNNNNNNNNNNN
NNNNNNNNNNNNNNNNNNNNNNNNNNNNNNNNNNNNNNNNNNNNNNNNNN
NNNNNNNNNNNNNNNNNNNNNNNNNNNNNNNNNNNNNNNNNNNNNNNNNN
NNNNNNNNNNNNNNNNNNNNNNNNNNNNNNNNNNNNNNNNNNNNNNNNNN
NNNNNNNNNNNNNNNNNNNNNNNNNNNNNNNNNNNNNNNNNNNNNNNNNN
NNNNNNNNNNNNNNNNNNNNNNNNNNNNNNNNNNNNNNNNNNNNNNNNNN
NNNNNNNNNNNNNNNNNNNNNNNNNNNNNNNNNNNNNNNNNNNNNNNNNN
NNNNNNNNNNNNNNNNNNNNNNNNNNNNNNNNNNNNNNNNNNNNNNNNNN
NNNNNNNNNNNNNNNNNNNNNNNNNNNNNNNNNNNNNNNNNNNNNNNNNN
NNNNNNNNNNNNNNNNNNNNNNNNNNNNNNNNNNNNNNNNNNNNNNNNNN
NNNNNNNNNNNNNNNNNNNNNNNNNNNNNNNNNNNNNNNNNNNNNNNNNN
NNNNNNNNNNNNNNNNNNNNNNNNNNNNNNNNNNNNNNNNNNNNNNNNNN

NNNNNNNNNNNNNNNNNNNNNNNNNNNNNNNNNNNNNNNNNNNNNNNNNN
NNNNNNNNNNNNNNNNNNNNNNNNNNNNNNNNNNNNNNNNNNNNNNNNNN
NNNNNNNNNNNNNNNNNNNNNNNNNNNNNNNNNNNNNNNNNNNNNNNNNN
NNNNNNNNNNNNNNNNNNNNNNNNNNNNNNNNNNNNNNNNNNNNNNNNNN
NNNNNNNNNNNNNNNNNNNNNNNNNNNNNNNNNNNNNNNNNNNNNNNNNN
NNNNNNNNNNNNNNNNNNNNNNNNNNNNNNNNNNNNNNNNNNNNNNNNNN
NNNNNNNNNNNNNNNNNNNNNNNNNNNNNNNNNNNNNNNNNNNNNNNNNN
NNNNNNNNNNNNNNNNNNNNNNNNNNNNNNNNNNNNNNNNNNNNNNNNNN
NNNNNNNNNNNNNNNNNNNNNNNNNNNNNNNNNNNNNNNNNNNNNNNNNN
NNNNNNNNNNNNNNNNNNNNNNNNNNNNNNNNNNNNNNNNNNNNNNNNNN
NNNNNNNNNNNNNNNNNNNNNNNNNNNNNNNNNNNNNNNNNNNNNNNNNN
NNNNNNNNNNNNNNNNNNNNNNNNNNNNNNNNNNNNNNNNNNNNNNNNNN
NNNNNNNNNNNNNNNNNNNNNNNNNNNNNNNNNNNNNNNNNNNNNNNNNN
NNNNNNNNNNNNNNNNNNNNNNNNNNNNNNNNNNNNNNNNNNNNNNNNNN
NNNNNNNNNNNNNNNNNNNNNNNNNNNNNNNNNNNNNNNNNNNNNNNNNN
NNNNNNNNNNNNNNNNNNNNNNNNNNNNNNNNNNNNNNNNNNNNNNNNNN
NNNNNNNNNNNNNNNNNNNNNNNNNNNNNNNNNNNNNNNNNNNNNNNNNN
NNNNNNNNNNNNNNNNNNNNNNNNNNNNNNNNNNNNNNNNNNNNNNNNNN
NNNNNNNNNNNNNNNNNNNNNNNNNNNNNNNNNNNNNNNNNNNNNNNNNN
NNNNNNNNNNNNNNNNNNNNNNNNNNNNNNNNNNNNNNNNNNNNNNNNNN
NNNNNNNNNNNNNNNNNNNNNNNNNNNNNNNNNNNNNNNNNNNNNNNNNN
NNNNNNNNNNNNNNNNNNNNNNNNNNNNNNNNNNNNNNNNNNNNNNNNNN
NNNNNNNNNNNNNNNNNNNNNNNNNNNNNNNNNNNNNNNNNNNNNNNNNN
NNNNNNNNNNNNNNNNNNNNNNNNNNNNNNNNNNNNNNNNNNNNNNNNNN
NNNNNNNNNNNNNNNNNNNNNNNNNNNNNNNNNNNNNNNNNNNNNNNNNN
NNNNNNNNNNNNNNNNNNNNNNNNNNNNNNNNNNNNNNNNNNNNNNNNNN
NNNNNNNNNNNNNNNNNNNNNNNNNNNNNNNNNNNNNNNNNNNNNNNNNN
NNNNNNNNNNNNNNNNNNNNNNNNNNNNNNNNNNNNNNNNNNNNNNNNNN
NNNNNNNNNNNNNNNNNNNNNNNNNNNNNNNNNNNNNNNNNNNNNNNNNN
NNNNNNNNNNNNNNNNNNNNNNNNNNNNNNNNNNNNNNNNNNNNNNNNNN
NNNNNNNNNNNNNNNNNNNNNNNNNNNNNNNNNNNNNNNNNNNNNNNNNN
NNNNNNNNNNNNNNNNNNNNNNNNNNNNNNNNNNNNNNNNNNNNNNNNNN
NNNNNNNNNNNNNNNNNNNNNNNNNNNNNNNNNNNNNNNNNNNNNNNNNN
NNNNNNNNNNNNNNNNNNNNNNNNNNNNNNNNNNNNNNNNNNNNNNNNNN
NNNNNNNNNNNNNNNNNNNNNNNNNNNNNNNNNNNNNNNNNNNNNNNNNN
NNNNNNNNNNNNNNNNNNNNNNNNNNNNNNNNNNNNNNNNNNNNNNNNNN
NNNNNNNNNNNNNNNNNNNNNNNNNNNNNNNNNNNNNNNNNNNNNNNNNN
NNNNNNNNNNNNNNNNNNNNNNNNNNNNNNNNNNNNNNNNNNNNNNNNNN
NNNNNNNNNNNNNNNNNNNNNNNNNNNNNNNNNNNNNNNNNNNNNNNNNN
NNNNNNNNNNNNNNNNNNNNNNNNNNNNNNNNNNNNNNNNNNNNNNNNNN
NNNNNNNNNNNNNNNNNNNATTTTAGGATAAAATCGTTACCAAACACAAT
TCCCTTCCCAAAAAAAAAAAAAAATTATCGTTACCAAACGCAATTCTACT
CCCAAAAAGAAAACAAAATTCCCATCCCATTCCCATCCCACTGCTGGAAT
TGAAACAGAAACGGTTACCAAACGCCACCTTAATGTTTCTGTTTCTAAAT

TTCCGTTGGGTGGTGTTTGTTTGTTTGTTTGTTTGTTTTTCCCAACCAAA
AACCGAAATTTGGGAATAATTCTGTTCTGGTCCCAGATATTTGTACTTAA
AACTATGGAAGCAGAACAGACTCAGATTTGGTATTTGTATAGTGTTTCTG
TTCCCAGATTTCTGATAGGTAGCATTTGTGTAGGTGGCATTTGGTTGTGT
TTTTGTTTTCTGTTTCTATTTCAAATAAACTGGGATGAGAATTTTGTTTT
CTTTCTGGAGTATTCTGGAAATAGAAACATTTTTGGTAACGACCATTGTC
CCGAAACCTTTTTTTTTTTTTTTTTTTTAATTTATGAGAATAAAAACAAG
AAACGCGTTTAGTTGCAACATTTGTTCGTTTTTTTCTTCTTCTTTAAATG
TACGGTGGCCGTCGGAGTGGTAGTGAGAATAGTTTTGTGGGAGTGAATAT
GAATCAACATTTTTTTCTTTCTTAAATTCTTAAGAAATTCAGGAATTATT
CCCAAAATTTTGAGAACATAAACGAATTTACCGAACGTGTTTTTGTACAT
AAATCCCAAAATTTTGGAAGTGGGAACAAAATTCAGGGAACGGAAACAGT
AACCAAACGGGGCTGTAGTTTTTTTTCCAAACAGAAACAAAAATCTGGGA
ACAATTCTGTTCTATTCTTGGAAATTTGTTCTTAAAATCTGGAAGCGGAA
CAGAAACAGATTTTTATTTTGTTCCCATTTTTCTGTTTATGTTCCAAAAA
ATTGGGAACAGAGACAAAAAACACACTCCAAAAAGGGGACCCCTTAAATC
TTCTTATTTCTTGTTTTTTTGTTCTCCCTTGCAGCGATGTGAATACACAT
AAAAACATATCATGCCCGGTTTCCCCTATTGGTAGCCCCCTCTTGCATTC
AAGGTCACCACAATGCACGAGCGGAAGACTCACACCATCTCCCATATCAA
GCCCAAGAGCCGTTCTGTCTGGTTCATCAACCCCTCTCACTGGCGGCGGC
AATGGCTTCCACCCCCAAACTCATCCGCCACTTAACCACCAGAATGAAAA
TATGGGAACAATGTTCCCACTTCCCCAGAATCATTCCCACCCCAAACCCG
ATATGTTCCGAGGAATTTCTCAAAATGGGTTTCTTGGATATCAGTTTGGT
GGGCAATCCGTTTTGGCAGATAGGGTTTCGCAGCAGCTTTTGAAGGGGAA
TGTGAAAGTCAACCCATTGTTTGACCTAAATCATGGATCTCCTGTTCTTG
GCCATGGCAATGGAACAAGCTATTGA
>EUC12684-RA [gene]
ATGCTACAGCTACTCCGTCCGAACTCATTAAATTCCACTCTTTCGGGCGA
ATCGGCCGACGATTTCTCCGCCTATTACAGCCGAAGTTCGTCCACTCCTA
GCGGCGCACGGCACTACACGACTCGCACGCCTCTCTATTCTGAGTTGATC
GATCGTAGCTTGGCCATGCCAATGGAGTCGACTTCAGCTTCGGCTTCCGG
ATCGAGAAATGACGGCGGGATTAAAGGAGTGCTCACTCATGGAGGTCGAT
ACGTCCGGTATAATTTGTACGGAAGCTTGTTCGAGGTCTCCAGTAAGTAC
GTCCCTCCTCTTCGGCCCATCGGCAGAGGTGCTTATGGCCTTGTCTGGTA
AATTGCTCATCCATTTGGTTTCATTTACATGTATGATTTGGATTTAACAC
TATTGTTTTGTTCAAATTTTCGTTCGTTCTTCGAATGCACTTAGAAGAAC
TGAAATACGTGTAGCTAAGTAGTTTGATTTTGTACGGACACACAGAATAG
AACTCAAATTCGTTTTAGAAAAAATTGGATTCTCCTTTTGGGAATATGTT
TATGCAGTTTGCTTGCACTTTCAGATTATGATCAAGAAAATAAATTTGCA
TTAGCATGGAGATTTTTTAGTTTTTTTTTTTAACAAAAAATGTAAGAGGG
ATTTTGATCATTTCTTTGATGCTCTGGACATTTTGCGCATTTTTCATTGA
TTTTTTGTTAGGTGTGAATATATTATCTTGTTCATTCATTATTTGGTCTG
AATTACAGATTATTTTAAGGGAGTTTAGGCAGATCTTTGATGATTCTATG
AAAGTAAATAAACTGATAAATGTTCATATGAGTTACATAAAAGCTTTCGA

TGTTAGATATAATGCAGTAAAAGATGACGCCATTTGTGTTCTTTCCAGTG
CTGCTGTAAATTCAGATACACGTGAGGAAGTTGCTATTAAGAAGATTGGC
AATGCATTCGACAATCGAATAGATGCCAAAAGGACATTGAGAGAAATTAA
GCTTCTCCGTCACATGGATCATGAAAATGTAAGATTTTCGTCTAAAACAA
CAAGTTTCATCTTGGTTTTAGATTGTTTCTTGGTTATACCATGGATTAAT
AACACCTTTGTTCTTTGTTTTCCATAGATTATTGCCATCAAGGACGTCAT
AAGACCGCCAAAGAAGGATATTTTTAATGATGTTTATATCGTTTACGAAT
TGATGGACACCGATCTTCATCAGATAATTCGGTCGGATCAACCCTTGACT
GATGATCACTGCCAGGTTACCTCTCTCACTCAATGGTTTGGATACTTTTA
TGCTGCTTTTGGATTGAGAGAAATATGATGAAAGAGAAGAAAATCTCGTT
AACAGAAGAGAAGAGAAGAGAGGAGAGAATAATTTCCTCACCATCGTGCC
TGGATAGTTCATTAAAAGACAAGAAAGAGTGTTTTCATATACCACAAAAA
AGTTTCATATGACTACATTGGTTTTTTCATGGCATTTCCCTTCATTTCTC
TGCCCCATTTGGGGAGAGAAAGAACACTTCCAATTTGTAAAGAAACTTTC
CCTCCATTTCTCCTTATTTTCTTCTCTTCCCTCATTAAATTTGCAAATCA
ATAGAAATGAGGAGAACCCATATTCTTTTCTTCTCTTTCCCATTCCTCTT
AATCCAAACAAGTCTTAGTTTTCATGCATCATTTTGAACTGTATTTTCTC
AATTTTTTCCATCCATTGCACCAGAACATCTCTTATGTGTTCAAACATTA
AAAAGGATTTCTTGGTTCTTTCCAAACATTCGGGAGAATTCCTGGTTCGC
TAGAGTAACTTACAGGATTTTAAGATTTCAAATAAAGCAAATCAATACAT
TTACACTAATCCATGGGCCACAATTCACTGAATGCATTTCGGATTGCTCA
GGAGTGTGAAAAAAATGCCTTTTCAGTTTCAGGGAGGTTATTTTCCATTTT
TTTCTAAAGATATACGCCAAACAAGCCATAAACCCCTTACTCAAAGTTGC
ATTTATATTAGACTTCTTTTCCTGCAGTACTTTTTGTATCAGTTGTTGAG
AGGATTAAAATACGTACACTCAGCTAACGTCTTGCACCGTGACCTAAAGC
CCAGCAATTTGCTCCTAAATTCCAATTGTGACCTCAAAATTGGAGATTTT
GGGTTGGCAAGAACAACATCGGAAACAGATTTCATGACTGAATATGTGGT
CACCCGCTGGTACAGGGCACCCGAATTGCTCCTTAATTGCTCGGAATACA
CTGCCGCTATTGATATCTGGTCTGTTGGTTGCATACTAGGTGAAATAATG
ACCAGAGAACCGCTGTTCCCGGGCAAAGATTATGTTCATCAGCTTAGGCT
CATCACAGAGGTAATAATTCTATTTTGTTCTAAAAATCTGGGAACAAAAC
AAAAATCTCTCTCGCTCTCTCTCAAGATACTAATACTGTTCTGTTCTTTC
TTTCCGAAAAGCTATTAGGTTCACCAGATGACTCCAGCCTTAGATTTCTC
AGAAGCACCAACGCCCAAAGATACGTTAAACAGCTTCCCCATTACCCAAA
ACAACAATTCTCATTGAGATTCCCAAACATGTCCCAACTAGCCGTCGATT
TACTCGAAAAAATGCTCGTTTTCGACCCCAACAAACGCATTACAGGTAGA
CCAAAAAAATTGATTATTTCACTTAATATTTTCTCTCCTTTTAAAAAAAA
AAAAAATATTTGAATTTTTTATTCAATATATTATGCAGTTGATGAAGCTC
TTTGTCATCCGTACTTATCGTCTCTTCATGATATCAACGACGAGCCCATT
TGCCCGATGCCTTTCAGTTTTGATTTTGAGCAGCCATCATTTAATGAGGA
GAACATTAAGGAGCTTATTTGGAGTGAAACTCTCAAGTTCTATCCAGATT
TAAGCAATTAG
>EUC01764-RA [gene]
ATGCAGTCCGATCACCGAAAGAAAGTGAGCTTTAATATCTTCCAAAAAAT

GCATAAATAATTCGATTGATTTGTATACATACATATAATTGATAAAAGTA
TTTATGATTGCGTAATTGAGCTGTTGATTATTTTGGCTGTTTCTTGAATT
GAACGGAATATTCTGAATTGATTTATTTCTGTTGATATTTCTTTTATGGT
TCCAGTTGGTTTTCTTTGTTATTTTTTCTGGGAAATAGTTGATGTGAAAT
TAGTAATGATAGAATTATTTGGTTTATTGCATTTTTTGCTGATCCTTTTC
TGGACCCTTTATCTCGGGTTGTCAAATTTAACATAATCCTTTTACTTAGT
AAAAGGTGCATAGTGAAGGATCAAGCATGAATGAAATGGATTCATGTGTT
TTGGTTGTGACCGTTCTTTAATCATTTATAAATTTCATTCTCCTACGGCG
ATAAAAATCGTATTGGGTTTGTTTCCATAAGCATTGTTTACAGAAGAGAT
TCGTGTTAAATGAATGAAGGTCAAAATATATCTTCAATGCCTAACTTATG
TGAACTGAAGAAATTGACAATCTACAAATTTGTTTATACGTGTTTCTTCT
GTTGCTGTTGTACCTTCTTTGATCTGTTGTTTGTTGCATCGGCCATTTTT
CTCTCCGTGCACTCTCATGATTAATTGTCTTGGTGTTATGTTCCTCAGAA
TTCAGCAGAGATGGACTTCTTCTCTGAGTATGGTGATGCAAGTAGGTACA
AAATCCAGGAGGTAATAGGAAAAGGCAGCTATGGTGTTGTTTGCTCAGCA
TTAGATACTCATACTGGTGAAAAAGTGGCAATAAAGAAGATACATGATAT
CTTTGAACACATCTCTGATGCTGCACGAATCCTCCGTGAGATCAAGCTTC
TCAGGCTTTTACGGCATCCTGATATAGTTGAAATCAAGCACATCATGCTT
CCTCCTTCAAGAAGAGATTTTAAAGATATTTATGTAGTTTTTGAGCTCAT
GGAGTCGGATCTTCACCAAGTCATCAAAGCTAATGATGATTTGACACGTG
AGCACTATCAGTTTTTTCTTTACCAGCTGCTTCGGGCTCTAAAGTATATT
CATACAGGTAATTCTTGGTGTAAAATTTAATATTTAGTTAACTAATTGTT
ATTATTTTTGCTAGTTAACCTAACCTTTTTTTTAAGTGAACTTCATATTG
GGTTGATATTTTTATTTTATTTTGCTTTTTGTTGTAAAATTTATCTGATC
AGGTGTTGCCGGGGGTTGGATTCTTTTACATTAAGTGTTATATGCTTCTG
AGACTTTAATGTTTTGATGCAGCAAACGTCTACCATCGGGACTTAAAACC
AAAAAATATATTAGCAAATGCAAATTGCAAACTCAAAATTTGTGATTTTG
GTTTGGCGAGAGTTGCGTTCAACGATACACCTACAACAATATTTTGGACG
GTATGAAATTACGAGGTGTAGATACATGATTTTTTTTCTTTTTTTAAATC
TTTCACGCATTTGATATCTATATTTTATTTCATAGTTTGCTTCTATAGCA
TGTGAAGCTAAAATAATTTTTATGATGCTTTACAGGACTATGTTGCAACG
AGATGGTATAGAGCTCCAGAACTATGTGGCTCATTTTTCTCCAAGGTATT
GTCTTACATGCTATATTCTGTACATCTTTAATTTTTTATATTGCATAGGC
ATAGCATGGTTTGTTGACAAAGTTTTTATATTCCCATTCGCTTCCTCGTT
ACTATTCTATGATATTGGGTATTATATATCTTGAGCCTAGCAAAAGAAAA
CATATATTGATCTAAATAGATGCCATTGATGGGTTTTGAAATCATTCTGA
GCTTTTCTTTACTTGTTGGATAATTTAGATTATAGTTTTTTTTTTTTTTT
TTTCACATGCGCACCCATTGAGATTCGAATCTGCACCTCCTGGGTGGAAT
TCGGGTATGGCCTGGTTAGCCTCATGTAACAAATGGGAATACCAGTAAGA
ATTGTAGCTAAGAAACATATGGAAGAAGCAGTATGTGGAAAAGGGTAACA
ACATTCATAATAATGAGGGTTTAGAAATTGTAGTTTCTTTTACTTTTTTT
AAAAACTTTCTTTGAACAAAGAAATAGAAATTCATTGTTTCTTACCAAGT
ATAATCTGAGAAACTGAGTTTTTCCTGTGAATTTGAACTGGGACGCAAGG
TATACACATTATGGTGCTTGCCTTTTGCGTTAGACTTAGTGGCAGAATGA

TGACTGAACTTGAACTTGAATACATATCATGATATTCTTTTGGCTGTGGG
ATACGGTTTCTTGGCCATGATTTGATCTTTCTTGATGGGACTTGGATTTG
ATTTGATTGTTGTTGCCTGCCAGGAACTGTCGAGCAGAATTTACTCACTT
TAAAATGCTTTACACTTTATTTTGTGATGGTTTTCTGATGCAGTATACAC
CTGCAATCGACATATGGAGTATAGGATGCATCTTTGCGGAGGTTTTAACC
GGAAAACCACTTTTCCCTGGAAAAAAATGTCGTTCACCAGTTGGATTTGAT
GACTGATCTTCTTGGGACGCCATCAATGGATACAATTTCTCGGGTATAAA
TATAATCACAGTCACCTAATGAAGCACTGTATGATACTGCTCAATTTCAT
GATTTCATCTGGAAATTCACTCTCAGTATCAAGTTCCTCGTGTACTTTTT
CAGGTTCGAAATGAGAAGGCCAGGAGATACCTAACTAGCATGAGGAAGAA
ACAGCCTGTGCCATTTGCCCAGAAATTTCCTAATGCTGATCCTTTGGCAT
TGCGTTTATTGGCAAAGCTACTTGCTTTTGATCCAAAAGATCGGCCAACT
GCTGAAGAGGTTTGTTATGGATCATTTACTTCCTTATTTAGATATTTTTG
GGTTTCTAAATCTCCACTGCTTTTTTCCATTATTTAAAATTATTTTGTTT
CCCCAAACAGGCCTTGGCTGATCCTTACTTCAAGGGATTGGCCAAAGTTG
AGAGAGAACCATCTTGCCAGCCAATTACCAAGATGGAGTTTGAATTTGAG
AGGCGAAGGGTTACAAAGGAGGATATACGTGAATTGATTTTCCGAGAAAT
ACTAGAGTACCATCCCCAGCTACTAAAGGACTATGTGAATGGAACAGAGA
GAGCTAACTTTCTCTACCCCAGGTATGAACCTAGTGCAATTTAAACCGCT
CACCCTCCCANNNNNNNNNNNNNNNNNNNNNNNNNNNNNNNNNNNNNNNNNNNNNNNNNN
NNNNNNNNNNNNNNNNNNNNNNNNNNNNNNNNNNNNNNNNNNNNNNNNNNNNNNNNNNNN
NNNNNNNNNNNNNNNNNNNNNNNNNNNNNNNNNNNNNNNNNNNNNNNNNNNNNNNNNNNN
NNNNNNNNNNNNNNNNNNNNNNNNNNNNNNNNNNNNNNNNNNNNNNNNNNNNNNNNNNNN
NNNNNNNNNNNNNNNNNNNNNNNNNNNNNNNNNNNNNNNNNNNNNNNNNNNNNNNNNNNN
NNNNNNNNNNNNNNNNNNNNNNNNNNNNNNNNNNNNNNNNNNNNNNNNNNNNNNNNNNNN
NNNNNNNNNNNNNNNNNNNNNNNNNNNNNNNNNNNNNNNNNNNNNNNNNNNNNNNNNNNN
NNNNNNNNNNNNNNNNNNNNNNNNNNNNNNNNNNNNNNNNNNNNNNNNNNNNNNNNNNNN
NNNNNNNNNNNNNNNNNNNNNNNNNNNNNNNNNNNNNNNNNNNNNNNNNNNNNNNNNNNN
NNNNNNNNNNNNNNNNNNNNNNNNNNNNNNNNNNNNNNNNNNNNNNNNNNNNNNNNNNNN
NNNNNNNNNNNNNNNNNNNNNNNNNNNNNNNNNNNNNNNNNNNNNNNNNNNNNNNNNNNN
NNNNNNNNNNNNNNNNNNNNNNNNNNNNNNNNNNNNNNNNNNNNNNNNNNNNNNNNNNNN
NNNNNNNNNNNNNNNNNNNNNNNNNNNNNNNNNNNNNNNNNNNNNNNNNNNNNNNNNNNN
NNNNNNNNNNNNNNNNNNNNNNNNNNNNNNNNNNNNNNNNNNNNNNNNNNNNNNNNNNNN
NNNNNNNNNNNNNNNNNNNNNNNNNNNNNNNNNNNNNNNNNNNNNNNNNNNNNNNNNNNN
NNNNNNNNNNNNNNNNNNNNNNNNNNNNNNNNNNNNNNNNNNNNNNNNNNNNNNNNNNNN
NNNNNNNNNNNNNNNNNNNNNNNNNNNNNNNNNNNNNNNNNNNNNNNNNNNNNNNNNNNN
NNNNNNNNNNNNNNNNNNNNNNNNNNNNNNNNNNNNNNNNNNNNNNNNNNNNNNNNNNNN
NNNNNNNNNNNNNNNNNNNNNNNNNNNNNNNNNNNNNNNNNNNNNNNNNNNNNNNNNNNN
NNNNNNNNNNNNNNNNNNNNNNNNNNNNNNNNNNNNNNNNNNNNNNNNNNNNNNNNNNNN
NNNNNNNNNNNNNNNNNNNNNNNNNNNNNNNNNNNNNNNNNNNNNNNNNNNNNNNNNNNN
NNNNNNNNNNNNNNNNNNNNNNNNNNNNNNNNNNNNNNNNNNNNNNNNNNNNNNNNNNNN
NNNNNNNNNNNNNNNNNNNNNNNNNNNNNNNNNNNNNNNNNNNNNNNNNNNNNNNNNNNN
NNNNNNNNNNNNNNNNNNNNNNNNNNNNNNNNNNNNNNNNNNNNNNNNNNNNNNNNNNNN
NNNNNNNNNNNNNNNNNNNNNNNNNNNNNNNNNNNNNNNNNNNNNNNNNNNNNNNNNNNN

NNNNNNNNNNNNNNNNNNNNNNNNNNNNNNNNNNNNNNNNNNNNNNNNNN
NNNNNNNNNNNNNNNNNNNNNNNNNNNNNNNNNNNNNNNNNNNNNNNNNN
NNNNNNNNNNNNNNNNNNNNNNNNNNNNNNNNNNNNNNNNNNNNNNNNNN
NNNNNNNNNNNNNNNNNNNNNNNNNNNNNNNNNNNNNNNNNNNNNNNNNN
NNNNNNNNNNNNNNNNNNNNNNNNNNNNNNNNNNNNNNNNNNNNNNNNNN
NNNNNNNNNNNNNNNNNNNNNNNNNNNNNNNNNNNNNNNNNNNNNNNNNN
NNNNNNNNNNNNNNNNNNNNNNNNNNNNNNNNNNNNNNNNNNNNNNNNNN
NNNNNNNNNNNNNNNNNNNNNNNNNNNNNNNNNNNNNNNNNNNNNNNNNN
NNNNNNNNNNNNNNNNNNNNNNNNNNNNNNNNNNNNNNNNNNNNNNNNNN
NNNNNNNNNNNNNNNNNNNNNNNNNNNNNNNNNNNNNNNNNNNNNNNNNN
NNNNNNNNNNNNNNNNNNNNNNNNNNNNNNNNNNNNNNNNNNNNNNNNNN
NNNNNNNNNNNNNNNNNNNNNNNNNNNNNNNNNNNNNNNNNNNNNNNNNN
NNNNNNNNNNNNNNNNNNNNNNNNNNNNNNNNNNNNNNNNNNNNNNNNNN
NNNNNNNNNNNNNNNNNNNNNNNNNNNNNNNNNNNNNNNNNNNNNNNNNN
NNNNNNNNNNNNNNNNNNNNNNNNNNNNNNNNNNNNNNNNNNNNNNNNNN
NNNNNNNNNNNNNNNNNNNNNNNNNNNNNNNNNNNNNNNNNNNNNNNNNN
NNNNNNNNNNNNNNNNNNNNNNNNNNNNNNNNNNNNNNNNNNNNNNNNNN
NNNNNNNNNNNNNNNNNNNNNNNNNNNNNNNNNNNNNNNNNNNNNNNNNN
NNNNNNNNNNNNNNNNNNNNNNNNNNNNNNNNNNNNNNNNNNNNNNNNNN
NNNNNNNNNNNNNNNNNNNNNNNNNNNNNNNNNNNNNNNNNNNNNNNNNN
NNNNNNNNNNNNNNNNNNNNNNNNNNNNNNNNNNNNNNNNNNNNNNNNNN
NNNNNNNNNNNNNNNNNNNNNNNNNNNNNNNNNNNNNNNNNNNNNNNNNN
NNNNNNNNNNNNNNNNNNNNNNNNNNNNNNNNNNNNNNNNNNNNNNNNNN
NNNNNNNNNNNNNNNNNNNNNNNNNNNNNNNNNNNNNNNNNNNNNNNNNN
NNNNNNNNNNNNNNNNNNNNNNNNNNNNNNNNNNNNNNNNNNNNNNNNNN
NNNNNNNNNNNNNNNNNNNNNNNNNNNNNNNNNNNNNNNNNNNNNNNNNN
NNNNNNNNNNNNNNNNNNNNNNNNNNNNNNNNNNNNNNNNNNNNNNNNNN
NNNNNNNNNNNNNNNNNNNNNNNNNNNNNNNNNNNNNNNNNNNNNNNNNN
NNNNNNNNNNNNNNNNNNNNNNNNNNNNNNNNNNNNNNNNNNNNNNNNNN
NNNNNNNNNNNNNNNNNNNNNNNNNNNNNNNNNNNNNNNNNNNNNNNNNN
NNNNNNNNNNNNNNNNNNNNNNNNNNNNNNNNNNNNNNNNNNNNNNNNNN
NNNNNNNNNNNNNNNNNNNNNNNNNNNNNNNNNNNNNNNNNNNNNNNNNN
NNNNNNNNNNNNNNNNNNNNNNNNNNNNNNNNNNNNNNNNNNNNNNNNNN
NNNNNNNNNNNNNNNNNNNNNNNNNNNNNNNNNNNNNNNNNNNNNNNNNN
NNNNNNNNNNNNNNNNNNNNNNNNNNNNNNNNNNNNNNNNNNNNNNNNNN
NNNNNNNNNNNNNNNNNNNNNNNNNNNNNNNNNNNNNNNNNNNNNNNNNN
NNNNNNNNNNNNNNNNNNNNNNNNNNNNNNNNNNNNNNNNNNNNNNNNNN
NNNNNNNNNNNNNNNNNNNNNNNNNNNNNNNNNNNNNNNNNNNNNNNNNN
NNNNNNNNNNNNNNNNNNNNNNNNNNNNNNNNNNNNNNNNNNNNNNNNNN
NNNNNNNNNNNNNNNNNNNNNNNNNNNNNNNNNNNNNNNNNNNNNNNTCT
GATAATTAAACCCACCTTCCCGTACACGTGCTAATGTGAATTGGGCTTTT
TTATGCAGTGCTGTCGATCAGTTCAGGAAACAGTTTTCTCATCTTGAAGA
TGGTGGTGGTAAAAGTGGGCAAGTGGTTCCGCTCGAGAGGAAGCATGTTT
CACTCCCTAGGTAATATACCAATCTATGTTTTTGTTCTTTGTTCAGTAAT

TTTACGTTTGTAGTTTAGCAAAAAATATGTAGATTTACGTGGTTATTCTC
TACAAAAAATCTGCATATTTCTATGGGAAATATAATAGCTTGCCTATAAT
TTGAGAGAGAATACCACCCATTGTGATGCATGATTTTGTGTTTTTGGTTT
TTTTGCCATTGGCCTTTCGAGACCATTGTTTATCCAATCCACTCGCTACA
ATATTGTTATTTTTTAGATTTTTGCTCTTTGCACCCTAGAAGTTTCGGAG
AAATCCCACTTTGGCCTTTGTACTTTCAATTTAAACACATGAAATTCTAA
GGAATCCAAATCATGTCACGTCAGTCAAAATCGGTAATTTCGTTTTTTTT
TTATGGAAATTAGCCATGTGTCAGATATGGAATCACCAGGTGGGTGAATT
CTGTCATAAAAATGACGGAATGATTGACTTTACATAGTTTGGATACTTCA
AGATTTAATGTGTTCAAATTGAAAGGATGGTAGCTAAAGTGGGATTTCTC
CAAAACTTCTGGGGTGCACAGAAAAATGCTCCGTAATATATTTTATGAGG
TCTAATAATTCTGGTAGAAATCCGAAGTCATTGCAAATTTTTCATATGAA
AAAGTTGTGATTTTCATCGAGTATGCTGACTTTGAACTTTGAAAAATTAA
ATTATTGTAGCGCATGTACTGTATATGTATAAATGTTAAGTTTGTGGACT
GTTTACTAAATTTTTGATTTTTGATTTGATTTTTGAAGGTCTACAATAGT
ACATGCAAATCCAATACCTCCTAAAGAACAACCAAATGTTGCTAAAGATC
GGCAAATCTTGGAAGAGTCTCGCGGTAGAACTTCCCAGCCGCCACAGAGG
ACAATGCCAATGGGTACTTCTTCCCCATGTTTCATTCAACGGAAAAAGGG
TTAATTACACTTTATCCCCTGTGGTTTGGTCAATTTGCATTTTATCACCC
TGTAATTTGGAAATTGCCACTTATTCTCCATGTGGTCAGATTTTCAAACC
ACTTACCCCCCCTGATGTAGGGGGATAAGTGCACCAAATATTTGACCACA
GGGGGTAAGTGCCGATTTTCAAATCACATGGAATAAGTGCAAATTGACCA
AACCACAGAGGAGTAAAGTGTAATCAACTCAAAGAAAAATGTCAATGCGA
GATTATTTATGCTTAATATTTTGGAAATTAAATATGTGTTTCAGCGAAAC
CCGGAAAAGTTGTGGGTTCGGTTGGGTCATATGAAAACGGAAGTGTGGCC
GGAGAAACTCGTGATCCGAGGACTTTTTCAAGAAGCTCAGGCATTCATAA
CCAGGTTATTACGCCGGAATATTATTACAACAGGGGAAAGCAGGAATTGT
CTTCTCAGAATCGACGAGTCCCTCAGTGTGGAATGGCGGCCAAATTAGCG
CCGGATATTGCCATCAACATCGACACCAATCCATTTTACATGACCCGTGT
AGGGGTAATGAAGATGGACCGTGTTGAAGATAGGGTTGAGATCGACACGA
ACTTGTTGCAGGTGCGAGCTCAATACAGCGGTGGAATTGGAGGTGCAGCA
GCGGCAGCTGCAGCGGCTGTTGCTCACAGAAAAGTTGGGAGTGTTCAGTA
TGGGATGTCCAGAATGTATTAGAGAGAGAGAGAGAGAGAGAGAGAGAGAG
AGCTTTAATAACTTTGTAGCTTCTCTCTGCGGTGGTGTACATACTGATTG
GGGAAATTTGTATGAGGCCAGTTTCTACTTAATCCTGGTCGTGAGAAAAT
GTAGATGAAGGGGAGTTATATACATTTTAGCACGCTCTCTTTTGTTGTTT
TTTCTTTTTTATTCCAAATTTTCGGATAATAAATCAATGAAATTCAACTC
TTATTTTGGCAGAATTTGTTTTCGTCATGATTAGTTGCTCTAAATTCAAA
CAAAATGTACAACCTTCCTCCCATGGTTTAATTTCATTTTACTTTTTTTT
TTTTCATCTTTTTTAAAAATATACAAACCTCCCCCTACGAAGAAATTCAC
ATAAATAAAAGAACGCAATCATTTTTTTGTTTTTTTCTTTTTTTGGGTAA
ATTTAGTATTTAAAGGTTGTAAAAGAGACAATATTTTCTCATTCAAGTTC
TTTCTCCTTCTCTCTTCTATTTTTTTTTACAAAAGTAAATTTAAAAATAA
AATAAATAATTATTTTGTCCGCTTCATATAAAATTTAGATGAAAATTTTG

ATTTCACTAATTTAAAAAAAAAAAGTTTAGGTCCTGTTTGGTTATTGTTTC

AGTTCCCTTATTTTTGTTTCCGCACTCAAAATTAGATTAATGTATAGAAA

CGCGTTTGGTGAATACGTTTCTATTTTCAAATTTTTGAAAGTATTTTCTA

AATTCCTCAGATATATGAGAAATAAAAATATATTGTTTTTCTATTCACTC

CCACAAAATTATTCCCACCACAAAAATTTGATTTCACTAATTTAGAAAAA

AAAAAAAAAAAAAAAAGGTCTTGTTTGGTTATTGTTTCAGTTCCTTGATTT

TTGTTCCCGCACTCAAAATTAGATTAATGTATAGAAACGCGTTTGGTGAA

TACGTTTCTATTTCCAAAAGTTTGAAAGTGTTTTCTGAATTCTTCAGAGA

TCTGAGAAATAAAAATATATTGTTTTTCTATTCACTCCTATAAAATTATT

CCCACCACCACCTGTCGGCCAATATCATCATATCGGCCACCATACATTTA

AGAAGAAAAAAAAAAAGAATAATGTTGCATATCATAAGTGTTTCTTATTTT

TATTCTCATAATTTGAAATAGTTTCAAAAAAAATGTTCGTTACCAAACAAG

TTTTTATTCCCGAAATACTCGTTAACGGAATAGAATTCATATTCTAGTTT

ATTGAAAGTAGAAACAGTCTAGTTTATTGAGAATCGAAACAGAAAACTAG

AAACACTAACCAAATGCCATTTTAGTTTTAAATTTATTATAAGTAGAGGG

GAATAAGTGTAAATTTTTAAAAGTATAAAATATAAGATAAAATAAGATGA

AAGGATGGGGGGTAAAAGACAAGAAAACCTCATTTCATCTATGTTTCCGG

CAGGGTCCCAAGTGGTATCTTTCGAATATAAGAGGGTGTTTTTTGGAATT

AGGCAAAATGACACGTGGTGTGATGCTATAAACAAGTATTTACGCTTGAA

TTAGGTTAATCAGAATTCGAAGCGAACCTGATCCATGGAAGACACGGCAG

TTGAAAAGCAGCCAGATCACCGAAAGATGGTGAGTTCTAATTTCCATTCG

ACGTTTCCAATTTATTCAACTGATTTGTGTTGTTATATGTATCCGTTGAT

GAAATTATTTATGGTTGAGAAATTGAGCTATTGGTAATTTTGATTGTTTG

TTTCTTAAATTGAAACGAAGGAATATTTGGATTATTTTTCTGGTTTCGTC

GATCAATCCTTTTATATTTGTATGAGATTAATTTGTGATTTGATTTATAG

GTTGATTTTTGGGAAAAAAAAAATTATATTGGCTTTCGAACTCGGGATGAA

GTTATGTGTTTTACGTTTGATCATTGATGATATACTAATACTTTGGGGAC

TTACGAGTTAATGTTTGGATCTTTTGGAGATGCAAAGTAACCTGTGTCGA

CTTTAGTGCTATGATTGTTGGTGGTGGTGAGGACGTCAGCGAGACACGTC

TTCTTGACTGTAGAATAACCTGATCTTGGTTGTAGGGTTTGTGAGGGATT

TGGATGCTTTTTTTTTTTCTTTCTCTACTTTGTGTAGAATTTGAAGCGGC

GATACTTCAGTGGAGGCCAAGGCCATTGGGGAGGGGGGGGGATGTGATTG

AAGTTCCTTCACGATTTTTCTTTTCTTGACAAAATCCTTTCGATTGATGG

ACCTTAAGCTAGACGCCTGATGATTTTTGCAGGTCTGTGGAAAAATGATA

AAAAATACAGCGAGATCACCTAAAGAAACAAGCTTTCATTTACTTCTATG

TTTTATTAGAGCTGATTTGTGTAATTGTCTTGTGATAGGCGTTAAATATA

GTAATAATTGGAAATTGAGCTGTTGATGATTTTTTTTTCATAGACTGAAC

ATAATGAAGTTTGTTTTTCCATTATTTACTTGTTTAGTGAGCCATATTTT

TCTTTTTATAAGATCAATTTATGTTTTTATTTACTGGTTTAGGAAAGAAG

ATCGATGATGTTTTTGCAATAGGTAAAGCTATGGTTTTGTTGTTTGCTCA

GTGATACTTGCGGTCTGTCGGTTTTATATTCTGTATAACTACTACCCCAA

GGAGATAAATGTTTGTTCTTTTTCTTCTCATCTTTCATCTTGGATGACAG

ATTCCTTTGTTGATAGTTATTTCGTTGATATTTTGTTAAATGGGAAGTAA

GATTATCAAAGTGGGGGTGGAAGATGTAATATGGGACTTATTGATTACTT

AGAATGATCACTGCTGACTAGAAGCACAAACAGCTTCTATAGACTTGAGT
CGTTGCTACTACGGTTGTATTGTCATGCAACATTTAGAGTAGAATTATTT
CAAAGGAAAAACACAGTGAATGCAAGCATAGAAGAAAGACCACGTTTTAA
AGTATTTGTCAATTGATTTTAAGACATTATGTTGTTTGTACATAGTACTT
TTTTATTTAAAAGCGTCTTTCAAATTACTTACCTACCATTTCCGAGACAC
TTCAACATGTTAGAACATATATACTATTTCTATTTCACTAACATCTAATT
ATTCTACCTTGGGGGTGGGGTGGGGGAAATAGGAAACCTCATAGGAGAAA
ACCTAAAAGTCTTTGAATTGTTTAGTCTCAGCAGGTGAAATAGTTTTCCA
GTATAATATGGTTGTTGTTGGTTCGTGTCGAATATGATGCCTAATGCTCT
ATTCCTATTGTTTATGAAGGTCGGTGGCCAAGATCGATCAAGCATTGAAA
GGTATTGCAGTAGGAAATTCAAACATCTGGATGTAATTCCTGAGTACGGA
ATCATAAAGGCCCTGCAGGAGATGTCTCTGGATATTTCTATCTTTGATAC
AAGTATGTGCAGTACCAGTGGAAAAGCTGTTTTTCCCAGTAGGAAACAAT
CTGTCAAAGAACTCATGTTCTCTAGTATTTTCTCAAGTCTAGGAAACGTA
CAACCGACAGAGTTAGCAAGTACCATGCATCATTCTTCACTAGATTACTA
CATTTATGGACAAGAGTTCACTGGTGAGTGTAGTACTGGCGGGGATTTTT
TTTACAGGAATTCGCTAGGCGTTTGTGGATTACCAATGTAGAAGCAGAAC
CACTACATACTAGGGGAAAAAACAATGCAGAAATTTCTCGTATCATAGAC
ATGTTGTCGATGTGCATTTTGTGTTGCTCCCTGTACTCTAAGGATTTCAT
ATTTACAAAGCGATCTGCAGTTTGGTTAGGGATAGCTGAAGGGTCAGTTA
ATGAGGTGGGCCAAGATGAACGCCTGGAAGATGTTGGAAGTATGTCCTTT
GATTTTCTGCTGAAGTGGGAATATATAGTGCCCTTTGGGTTTGATTGTTT
TGGTGATGAGGTGGCCTACAAATTAGGTGATGCGATGGCTTTATACTTAG
AGAAACAATCTCTAGAACCAAACTTCGCAGCGTCGAGGATGGCAAGTTGG
GTGATGGCACACCAATGCTAACATTGTATTTGTCCTTGTTGACGCAAGAG
GGAATTGATGTTGATTTGACATTTGAGAGACTGAATAAATTCATGAATTT
GCGCTGTCTTATGTTTTGTGGGTCTTTCTTTAAACGGATACCTCATGATA
TATTCTTGAAGTTGATGCATATACGGGTGTTAGATTTGAGTGACACAAAT
TTACGTGAATTGCCATCTTCTGTCGGAAATTTGAGGTCGTTGTGCTATCT
CGATGTCTCTGAGACTCCCTATCAAATGGTTACCAGAGTCGAGTCGATAG
ATCACCTTGGAAAATTGCTGACCCTGAGGCTCAGAGGTTGTGCAGACCTC
ACTTGA
>EUC17921-RA [gene]
ATGGAAATGCCTGGTAGACGATCGAATTACACTTTGCTGAGTCAGATTCC
GGACGATCACCTCCACCAGCAACCGCCGTCGAAGTTCGTTGCCTCAGATT
ACGAGTCTCTCCCCGGAGACCAAAATAAAGGGAAGTGTGATAGAGGTTTC
GTCTGGGACCTGACAGATCATCGAGCAGTTCAGCCGCATAGTCGGATCGG
CACGGTGGCTTTTCCGGGGACCCTAGGCATTCAGAGGCAGTCTAGCGGGA
GTAGCTTCGGCGAGAGCTCCATTTCTGGTGACTTCTATGCGCCGTCTCTG
TCCAATCCGGATGGCTTTGCGTATTTGCATGACGGTGGAGGTGAATTGAG
ATTCAAGGCTGTAGACGGTGGCGGAGGATCGTCTTCATCGAAGAGTTGGG
CGCAGCAAACGGAGGAGACTTACCAGCTCCAGCTGGCCTTGGCGCTACGA
TTGTCGTCCGAAGCTACGCGTGCCGATGATCCCAATTTTTTGGATCCTGT
GCCGGATGAATCGACGTCACGGTTTTCATCGTCGTCTGCTTCAGCAGAAG

CCACGTCACATCGATTTTGGGTATGCATATAGGGGGTCTACACCCATTGC
TTCCTCTTGTTTATTCATATGAAGCCATGGTTTTCAACATGTGGGATTTA
ATTTAAGAGCGAATTCTCTTATAATTATGCCAAACAATGTATTTTTTCTT
CTGGGGCTAAGAGCGGTGTCGGATTTTGGACGAATAATAATGACAAGGAA
TTCAGTAAATGAATATACGATGCTGTCATTTGGCAGTGTGGATTAGGTCA
AGAATTGGACTGAGGTGTTGTTTAATAAAACTGAAAATATGTTTTGAAAA
TTTGATGCTGATAAATGATGAACTGATTAATCACTTAAGTAAAGGAGTTT
GATTGTGCACTGAAATTTAAGTGATGTTTGATCGATGCAAATTATTAAAT
TTTGAGCCTTTTTAATTACGCTGATTTTACCCTTCACTACTTAAGTTCAA
ATTATATTTTGACAAAATTATTTAACTGAGTATTTTGATATATGTTAATA
AAGATATTTTAATATGTATTTTAGAAATAAGGTGTTCATAATTAAAAACC
TCAAACATAAAATCTCATTTGTGGATTTGGTATGAACTGAGTCTTGTAAA
ATTTATAGAATGTATAAGAAGACATAACAATTTTTGTTATAACGGAAGAA
TATATAAATTGGTGTAGATACAAAATAAGATTACAAGTGGAATCATTATA
ATGTTTATTTTTATTATTATTATTATTATTATTATTATTATTTTGTTAGCAAC
AATTGTATTATCAAACCCTCATAATTTGTTACGCAATTAAACTAATTTAA
AGGTGCTTAATTGAGTAATCACATAGTCTTAATTAAACTAATTGAGGGTG
AGTCTTGGGTACAATGGTAAGGTTGTTCCATTGTGACATGGAGGTCTCAG
GTTTAAATCTTAAAAACAGTCTCTTTGTAAACAAGGGGTAAGGTTGTGTA
TATCTGACTCTCTTCAGACCCCTCAATGGCGGGATTGTGCACTCGGCTGG
CCTTTTTAATAACCTGTCTTTTTCTTTTTTTTTTTTTTGGTAGACAAGGA
TAGCAGAGAACTAGAAGATGCTGAAAATCGATGTAGGTAGTTGTTACGAG
CTGGCCAAAGAGTCTTACAGTCTTAGCCTAAATTGGGATTCTTTGTGAGA
GGCCAGGTTAATGCCACTAAAGGGAGTAATAATTATTTGGATTTTCCAAC
CTTATTGGTTAGTAGCAACCACCAATGAGGTTGAACTAATTATATGTAGT
CCTTTCTAATCTCACAGTTTTCTGGTGTGATCTCTTGGACTTAAAATCAA
AGAGATGAGGGGTCTTGCTCATTGTACTTTTCTCTTGGATAAATTTAACT
ATTTCTATTTTTCTTCTATATAGTTTCCTTTTTTGTTTTTGTTTTTACA
GATGTAATATACATAATGAATTGAGTGCAATGGTTTGAATATGGGTTTAT
CTAGAACATAGTCCACAGTCCACACAATCCACGTGTTGGACTTGGCCGTC
ATCACCTGTCGCTTTGAGTACGGACTTCGCTCTAGACCAGGTTAACCATG
AGTATGCAGATTTGCTCTCTGAAATTCATGAATTCATGTTCCAATACCAC
ATATTCGCTGTATTTTAACCCTACCCTAAATACGTGAGGAGAAAGTCCTG
ACAGTCAATGATCATTTATGATATTAATCCTTAAATTTGTTGATGATCAT
CTATCATATTAAACCCTAAAATTGATTCCTTGATTGAAAAGTAGGTTATT
TATGNGGGGAGAAGAGAGGGGAATAGAGGATGAAGATATGGGTGTGACGA
ATAAGAAGAGTGAGATTGAAAAGAAGGGATGGACTTATTGTTTTTGTATG
ACCAAGGGTTGTGTGTGGGATCACCTATTCATCACCAATTGCAGCCTTGG
ACTTGGGCACAAGTTTGCCTCCTGTTGTCATGAAATAATGCCAATATAGT
TATTTAGACACTTGGGTTGAAATAATCGGCGACAATGTAGAAGCTAATCC
CCATTTTGATGGATGGCATTCTATGTTTATTTTATGTCTAATAGCACGCC
ATTTTAATTTTTTTTTGTTATTTATACAAATATTCACCATTTTAAGATTT
GAAACATATTGTTCTTAGGTCTTAATTATGGAATTCAAAATATCTATAGG
TTTACACTCAGTGTCTCAAGGCGTATTGATGCAGGATCACAACAGATTTC

TAAAGTTTGAATTTCGTATCATCTATAATTTAGATACCTTAACTGTAGAA
TTTCTTTTCTTTAAAGAATCATTTTTTATTAGAATTTTTGGAGTTGTTTG
CCCATAAATAGGGCATGTGTTACATTTCTGCTGACTTGAATATTGATTTA
ATTAGGTGTTGGTCTGTTGAGGTCTCTCTTTTCTTAGTCTCTCTCTTCTT
CCCTATCTTCTATGCGGGCTGTCTTCCACTACATATGCTCAGTATCTCAA
GGCCTATGCCTCACATTGGGAAGTCGAGACTCCTTACTAGATGGTTTGGC
ATTTTACTTTGCACAATACACTTTGCTTTCACTATATGTGTGGTACATTT
ATATTATCTCTTAGTCTTGCATGTATAGCGTAAAACTAATGCAATGTGTT
ATTTGAAAAATTTGACAAATTATATATGTATAGTCTGTAGATATTGATAA
AAGACACCCTTTTTTTTTTTTTCACTAAAAGGTGTGGGCGTAAGGCGGGG
CATTTTAACCTATCCAGGGTTGGGTGTAAGCCCACGAGGGGTGGGGCATA
GGTCCCATGAAATATGAATTTTTAATATTTCAAAAGTATACAAGAATTAA
AATTTACATACAAATAATAGGAAAGAATAGTAGAAGGCAAACAAAAAAAG
GGACTATATATGTAAAAGTTTATTCAAGTTACTAACAAAGTAAAAATGAT
AATTTATATAGCATAATGGAATGTAAATAAGTAAATTTGCAGTTCAATGC
TCACTAGAAATATCTATTTAATCACTCAATGAGATTAAAACAGATGATGA
GGACTTCTAACTATATTTTAGATTTTCTAATAGTAATTTATGTTAAATGC
TAACTGTAGAAAATCTAATGGGCCTAACTCACTCTCAAAATATGCCTTGT
AAGAGGAGGATTGCCCTTGGCTTTATATATAGCTCATGATCAGTTAACAC
AAACAATGTGGGATTCAACACGCCCTCTCACGCACAGGGTAGACTTTGAC
AAATCACTGCGAACGGATATCGGGAGGCCTAACATCAATGTGGGATTCAA
CACGCCCCCTCACGCGCAAGGTGAACTTTGACAAACCATTGCGAATGGAT
ATTGGGGGCCCATTATTGATGTGGGATTTAACACGTCCAATATCAGATAA
AAGTCTGGTTTTGATACCATGTAGAAAATCTAATGGGCCTAACTCACTCT
CAAAAAACGCCTTGTAAGAGGAGGATTGCTCTTGGCTTTATAGATAGCTC
ATGTTCAGTTAACACAAGCGATGTGGGATTCAACACGCCCCCACTTGTAG
AGTGGACTTTGACAAACCACTCCGAATGGATATCGGGAGGCCTAACATCA
ATGTGGGATTCAACACTAACCTTAATTAAAATCTTAAAATAACATAATTT
TAATTAACATGTAATTATTTGAAATAAAAAAATGGAAAAAAAATTAAACA
GCAAATTGGGCTTAAGCCCCAAGCCCTGGGCTTCGTTCAGTTTGCCTAGA
AGTCTGGGGTGTATGCCCAAGGAGCATTTTGTCCAGCCCATAGTTCAGGG
ACATCTCTACTACGCCCTGCCTCTGAGCTCGCTTAAGAGACGCCTTTTGC
AACACTTCTCCATTTTTTTTATTAATAATTATTTGCTAAGAAGTCCCTTA
TTGGTTTAATGGTGCTCCCAAATAAGCATATTACCAAAGGGTCTTCTTCT
CCACCTTTTTCGAAATGGTTTAGGTAAGATGCTCCTTAGTTGTATTAGGG
TTGGGTCTTGGGAAGCATTTGTTGGATCTCATGGGTTTTGACCATCTCTT
TCATCTTTATTCATCTTTCCGTGTGCACACGTGGAGTGCATGTTCAGTCC
CACATCAGTTTAATAGGTCTTTCTAAGCTGACATATTAGTTGTCATATAG
TTTTAGGGTGGATTCTCGTAAGAGATTACTGGTGTAACTAATTATACGGA
AGCTTTTGTGGCATCAACCTTACTAATGCTTAAGTTTTGATGCATTGCAC
TTGCTCTTGTTTTAACTTCTTCACCCATTCCTAAATATACAAGATTTAAT
CTAGCACTATGAAATCTTATTTTTGGTAGCAGAGATGTTCTTGATGATTG
TGATATTTTGTGCAATTAGTGTATTATCTTACTGTTAGCCCCTGGCACTG
CAGATTTAGGAAATGGCACAAACTCGGTGCATTTACTCACCAGGTTTGGT

GCTAGCGACACGCACCAAGGCATCGGTAGCAAAACGTGCCATGGTGCATT
TTCTAACTGGATGTGGTGCTTGCGGCGCGCTACTATGTCGTCTGTACAAA
ATGACTTGGAGGTTGACCTGAGTAGGCACAAGTGTTGGACGAGGACAAGA
GTTGGTTAACAAAAAGTCGGTGTGATGGTGCCAAGATAGGGTAACTTGGT
GCAGACGCTGGCGCTTTCGGCTAATAAGCTAAAGGTGTTGGCCTTGATAG
CTGTACCATTATGGCTTCTCAAGAATTTTGATGTCTACTGCTGCACCTTC
AATTATTTTTCTTAAAGTATTATAAGTGGACCACTCTCCTCGAAGACCCT
CGGTATGGCTGTGAACGTTGTGTGCTGGAGATGTTGGCACAACAAGGTAA
GGTAAGGTGTAAGGCATGACTTACACACGATGGATGGGAAGTTTAGGGCG
TGGCTTACCCGAGAATTTTGGTTTATTGCGGAAATCAGGGTGCAGTGCGT
CGGCGGACAAGGGCGTCCTGGGACAATGACGGTTGGCATTATGCGTCGTT
GAAGCTTAAACTTGAGGTGATCCTAGGCTTGATGCTAGGATTTGTGCTCA
TCGGTGTGGTATGACATCGAGCAAGACTTTGGCACGAGGTCAAGGCAACA
AGCGTCAAAATACGTCGTCAACTCGTTATATGTCGGCAACATGGGGTGGT
GTTGGGCAATGCTGGTGGGCATTGTGCGATGTTGGAACAAGGTGCTTGGA
AAGCGTGGGCAAAATAGGATGTTGTTAGACAATACTTGTGGATATTGGGC
GGTGTTAGCATGAGGTGCAAGGCAATCCTAGGTGCAGTGCTAGGATTGGA
TTTTATGGGGCACGACATGAAACCACATTTTGGTTAGAGTCATATGTGGG
TGATATGGCAAACCGTTGGGGCACAAGAGTGTGTTTGGGTCTTAGTCATG
CATGCGACATGGCAAGCCGTTGGGGCGTAGTGACAAAATGATGAAACAGG
ATGAGCGTAAGGTCAGCTGGACAACTTGGGTGTCGCTGTTGCTTAGTTAA
TGTGGGCAAATTTGGCTAAGGTTTAGTTGGAACATGTGCCTGGCATGTGG
GGGAAGTTACATGAACCTGGCCATAGATTCAGTAGGGTAATTGTCATACA
TTCCTGGCCAGACACTTGTCCGGTAGTTCCTTGCCCATGATCCTAGTAGT
TTGGGGTGTCAAAAGCCTTCTTCCCTCGTTAAATTCTGATTTGAACATTA
GGGTTCAACAGAGAGAGCTGTTCCTGCAACCCGTGACCAGATTTTGTTGT
ATGTTTCCTTGTTTTTGTGATTTATAAAAACAGGGTTGGCTTCAGCCGCA
CGGGTGGTGCGTTTTATACAATTTCTGTTGCGTGTGCATGCGTTGGGTTG
ACTTGACATTGTTGTTGTCTCGAAATAGTGTGGGTGTGACTAATTGATTG
GCGAACAATCAGGCCATCGCATTTGCAAAAATATCATATTTGAAAGTAGA
CCTGTGAGCTGTGACACTTACTGTGGTTACCAGAATACTCTACTGCCTTT
AGCGATAGGTTAATGCATACGGAAGTTCAGATACATGGTATTCTGAGGTT
CCAAAACTCGAAATTTGGCGATCCAAAACTTCTAACTTGAATATTTTAAG
CTTCAATTTTTTTGGGCTAAATTATATTACAAGCTTTTGTACTTTGTACT
TTGTTTAAGTTAAGCTTATCGGCTTTGTCGTAATTGAGTCTTTAACTTTC
AACAATGTGTCACTTAAAGCCAATAGTCTGATAGACCATTAATCAAATGC
ACCTATGTGAGGAATAACAAACAGAGCGTCTAGAAGAAATGCTTGTTGAA
GTTAGTTGAGAAGCTTCTCTACAAGTGGGGTCAAGTTGATGAAGTTGTTT
GACAAGTTGGTTGTTAGGGGAGTTAGTTAGGAGAAGGATCTCAGCCTAAA
TAAATAGAAGGTCATTGGAAGGGAAAAGGGACTGGAAATTTGAAAGTAGA
AAGGGAGAGTCCAGACTCTCGAACACCTGGTTATTTGTTCTATCATTTTG
CTTGTATTTTTTTTCATTCAACTTTGATAATTCAAGTCCAATTCAGCCTA
TATTGCATTTATCTGTTTCTGTTCACTACAAAATTCTCTATTTGGTGAAG
GGTGATATAACAAATTGGTATCAAGAGCAGTAACAATTCTGGGATAAAAA

TGGCACAAAGGAAGGTTGAGGCTTTGGAGGAGAGGATGGACTTTGAAGCT
CAGAAAATCAGTGGTTTGGAGAAGACAATCGGAGGGTTGGAAAAGACAGT
CGGTGGGTTGGAGAAGACAGTTGGGAGCATAGAGAAGACAATGACTCTTA
TGATGGAGAAGTTAGATAAATTGATGCAAAAGGAGGATTCGTGTTCTTTT
TCACGAATGGAGGAGAAGCACAATCAGCCTCAGATGATGGAGTCGCAGAC
TATGGGAGGTGGTGTGATGACTGAAATGTTTGGGAATAGATCCTATCGAA
GATTGGAACTACCAATTTTCACAGGAACGGATCCTGATGGGTGGATTTTT
CGAGTAGAAAGGTATTTCATGATGAATAAGATGAACGATGGAGAGAAATT
AGATGCGGTCGCTTTGAGCTTGGAAGGAAAAGCTCTTGCATGGTATCAAT
ATGTAGAAGATAGGTCTCGATTTCGTACCTGGAATGAATTTAAGTTGGCT
TTCCAGAATCGGTTTTGTTCGAGTATGGAGGGGTCTCTTTGTGAGAAATT
TTTGGCCGTACGCCAAGAATCCACGGTCGAAGAATATCAGGAGTGCTTTG
AAGTCTTGGCATCACCATTGAAGGGCCTATTAGAGGAGGTTATGGAGAGC
ACCTACCTTAATGGATTGAAGTCGACGACCAGAGACCTAGCAATTTGTCT
GAGATGATGGAGGCTGCACGCTTAGTCGAAGATAAGAACAAGGTGATCCA
ATCCACGCGGGTTAGATATGGATCCAATGGGTCGTGGAGCAAGGCATCGG
GTTCTAGGTCGGGTTCAACTTATTCGAACCCTAGGTATACGATGAACTAT
GCAACGACGACGTTTCGACCTTCGAACTCAATCAGATCATCATTGACAAC
GACCACCGGTCACGTCGGACCAGACCAAAGGAAGACACCACCGGAGGAGA
TGACGGCGACACGAAACTCATCGGCTTATAGGCAATTCACAGACACTGAG
TTTTTGAGTCGTCGCGCAAAGGGATTGTGCTTCCGTTGTGAGGGGAAGTA
TTCTCTTGGCCACATATGCAAGAATGGGCAACTGCAGGTGTTAGTTGTAG
CCGATGCTGGTGCAATTGAAGAAGCAGGAGAGGAGGAGTTGGTAGAAGAA
GGTCTTGAGCTTGTGGAAGGCGAAATCGAACTGTCAATGAACTCTGTAAT
GGGGTTTACTGATCCACGTACAATGAAGTTGCGAGGGAAAATCGGATCAC
GAACAGTGACGGTATTGGTAGATTGCGGCACATCGCATAACTTCATCTCT
AACGATGTAGTACAGCGGCTGAATCTGCCAGTAACTGCAACTACGGCGTA
CTAAGTGTTCATGGGAACAAGGGAAGCAATGTGTGCTGATGGAATTTGTA
AAGGAGTATTACTGTCTCTTCCTGCAGTTGAGGTGGTGGAAGATTTTCTA
CCATTGGAGTTAGGCAAAGAAGACGTGATCTTGGGAATGCAATGGTTGAT
AAAATTGGGAGCGATGTAAGTCAATTGGAAAGCATTAACTATGAAGTTCG
CCTTGGGACGAACCATGGTAACATTGAAGGGAGAGCCAGGACTATGCAAA
AAGCCGATATCGCTCAAAGCGATGATGCGTACTATACAACGCGAAGGACA
TGGATATTTGTTAGAATTACAGTCACTAACGTCACAGAACAGTGAAGATG
ATGTGGCTATGGAAGTTTCCCATGCCTTCGGTAAGTTGTTACAAGAGTAT
CCAGAAGTGGTGAAGGAGCCGAGGGGATTACCCCCTCAACGGGAGTTGGA
TCATTGCATCATTTTGAAGGAAGGAGTAGAACCGGTGAGCGTGAGGCCAT
ATCGATACCCACAAGTGCAAAACGACGAGATCGAACGATTGGTAGGTGAC
ATGTTGGCAACTGGTATCATACAACCGAGCCATAACCCTTTTTCGAGCCC
TGTTATTTTGGTAAAAAAGAAAGATGGCAGTTGGAGATTTTGTGTTGACT
ATAGGACTTTGAATCGGGTAACTGTTCCAGACAAATTTCCGATTCCGGTG
ATAGACGAGCTCTTGGATTAACTATTTGGGGCACAATTTCTTTTTCTAAA
TTGGATCTTAAGTCAGGATATCATCAAATCCAAGTGAGCCCTTGGGATGT
GCCTAAGACTGCCTTCAGGACCCATGAAGGTCAATATGATTTTCTTGTGA

TGCCGTTTGGACTGATGAATGCACCAGCGACCTTTCAAGCTTTGATGAAT

GAGGTATTTAAATAGTATCTTCGGAAATTTGTGTTGGTATTTTTTGATGA

TATTCTGGTCTATAGTAAATCTATGGAAGAACACCTGGAACATTTGAGGA

CGATTTTGGAGACTTTGAAGAGACATTCATTGGTAGTTAACCTCAAAAAG

TGCGTTTTCGAGCAAAGTGAGTTGGAATACTTGGGTCATATCGTGTCGGC

TAAAGGGGTGGCGGCTGACCCTAAGAAAGTACAAGCAAAATGATAGAACA

AATAATTAAGTGTTCGAGAGTCTGGACTCTTCATTTCTACTTTCAAATTT

CCGGTCACTTTTCCCTCCCAATGACCTTTTATTTATTTAGGTTAAGATCC

TCCTCCTAACTAACTTCCCTAACAACCAACTTGCCAAACAACTTCAACAA

CTTGGCACTACTTGTGGAGAAGCTTCTCAACTAATTTCAACAAACATTTC

ATCTAGACGCTCTGTTTGTTATTCCTCTCACATAGGTGCATTTGATTAAT

GGTCTATCAATAATCAATGGTGGAATGGCCAACTCCGACTAACTTGAAAG

CCCTACGAGGGTTCTTGGGTTTAACAGGGTACTATTGGAAATTTGTGAAG

AATTATGGGAACATGGCGGCACCACTCACCAATCAACTGAAGAAGGATCG

TTTTGGGTGGAATAATGAAGTACAAAGGGCTTTTGAAGCATTGAAAGGTG

GCAATGACAACGGTCCCTGTATTAGCTCTCCCTAATTTCCATCAACCGTT

TGTGGTGGAAATGGATGCTTCTGGTTTTGGCATTGGTGCGGTGTTGATGC

AAAATCAGTGGCCAATCGCATTCTTTAGCCAGGTCCTTAACCAAAAGGAT

CGATCTAAGTCGGTATATGAGCGCGAGCTAATGGCCATTGTTTTAGCTGT

CCAAAAGTGGCGACCTTATTTTTTGGGACGCAAATTCATTGTGCGTACTG

ATCAGCGGAGTTTGCGATTTTTTCTTGATCAACTGTTGGTGTCTGATGAG

CACCAGAAATGGGTTTCGAAGTTGTGGGGCTACAACTTCGACATAGAATA

CCGACCTGGAAATGAGAACAAAGCGGCCGATGCTTTATCAAGGAGAGAAG

AAGGTTGTGAGCTGGTTGTCCTCACAATTTCATGTTATGCACAGTTTGCA

CACGCCGAAAAACTGCAAAACGAGGTTGATTCCGACCTCATTTATGGACC

CATCAAACAATAGATATTTCAAGGCTCCGATTCATTTCCTGACTATGGCT

TGCAGCGTGGCATTTTGTTGTACAAAGGACGGATGGTGATTCCGTAAGAA

TCCACTCTCATTCCGCAATTGTTAGGAGAGTATCATGGGGGGAAGATTGG

AGGACATTCTGAGGTCTTAAAGACATTTCAACGGCTGTCTCGAGATGTTT

ATTGGCAGGGTATGAGGGAACAAGTGCAGCGTTTTGTAGCTGAATGCTCA

ATTTGTCAACAGAATAAACATGTGGCTCTAAGTCCAGCAGGGCTACTGCA

ACCGCTACCTATACGGGATCAAGTTTTTGAAGATTTGTCCATGGATTTTG

TGGAAGGGTTGCCTAAATCCGATGGGTTTAATTGTTTATTGGTGGTGGTT

GACTGTCTTTCGAAATTCGCTCACTTCATTCCACTAAAACACCCTTTCAA

TGCCCAAGATGTTGCTGCAATATTCGTGAAGGAAGTTATCCGTTTGCATG

GGGTTCCACGCTCCATCGTTTCAGATCGTGATAATGTGTTCCTCAGCAAG

TTTTGGACAGAGTTGTTCCGTTTACAAGGGGGTTCCACGCTCCATCGTTT

CAGATCGTGATAATGTGTTCCTCAACAGTTTTGGACAGAGTTGTTCCGTT

TACAAGGCACCTTGTTGAAGCATAGTACCGCCTATCATCTGCAAAAGGAC

AGCTAGACCGAGGTGGTTAATCGGGGAATCGGGACTTATTTACTGTGTTT

TGCGGGAGATAAACCTAAGCAATGGGTTAAATGGTTACCTTGGGCGGAGT

ATTGGTACACTACTTCATTCCATTCATCTATTTACACCATAGTTGTCAAA

GGCGCGCTTGGGCGCGTGCCCAGGCGTAAGGCGCAGCTAGTTGAGGGTTC

TGCGCCTAGACAAACCCTAGCCGATGAACATCACGAAGGTGAGCGCTTTG

TGAGCCTAGTCGACAGTCGCGCCTCTATGGTCTTTTAATTTTTTTTCTGT

GTTAACCCTAGCCCATAAAATACCCAGATCCCTTACCTAGCCTCCCACGA

AGAGCTCCTTTGATTTGATGTTCGATGTTCGATTTAGCCTCGATTTGAT

CTTCGATGTTCGATTTCAGCTTCGAATTGATCATCGTTGTTCAATTTCAG

CTTCGCATACACTTCAATTCCTTTTCTCCTTTGAATTCGTCTTCTCCATC

GATTTCACCTTCGAATTCAGGTTCGAATTCATCTTCTCCATTTCGTTGCT

GCTCAACGTCATTGCTTTGCTACGATTAGGCTGCTTCTCGTTGCTGCTCC

ACGTCAAGATATGCATTCTGTTATTTAATAGTTATTTAATAATTTTATTT

ATTTATTTATTTATTTATTTTTTTTTTTTTTTTTGTGTGTTAAGTATTTG

TTAACATAATTTAGTTAACAAAATTTAATTAAGTAATTTTTGTACTGCTC

TTTTTGTTCCTTGTTTGTTTGTTTGAGTGATTTAGTTTAGTTAACAAAAT

TTAGTTAAGTAATTTTTTTTTTGGGTACATATTTATTTATCTATATTTGT

AAACATAGTCTAGTTGGGTACATATTTATTTAAGTATTTGTATTTGCTCT

TTGTTCGTGGATGTTTAAAGAATTCTATTTGGAATTTCTGAATGGAATAT

TTAGAATCTCGATGAATTGAATTTATTTGAATTTTTTTTTAGTTAATTAT

TCTTATTTATTGTCTCACATACTACTCTAACTTGAGTGTGTGTTTTATTT

TTTATAATTGAAATTTAGTCATAGTTTTAATTTTAGATTTGCAATATATT

AGTGCTATATATTATTTTTTATTTATTTTTAAATGATGTGTGCCTAGCGT

CGCTCAGGCTAGCCCCTCAACTTGTGCCTAGCGCCTAGGCTCCAGGGGAC

CTTGTGCTCCTCGGTGCGCCTAGAGCCTTTAATAACTATGATTCACACTA

CTCCATTCTGAGTTCTCTATCGTCGTGATCCGCCTCCCTCGTTGCGTTTT

GAAGCTGGCTCCACTCCCATGTCCGAGGTGGATTTTCCACTGCAGGCGGG

GGACCACATCTTGGAGGAACTCAAATACCACTTGAACTGTTCTCAGAAAC

GCATGAAGGATTATGCGGATAAGAAGAGACGTGTAGTCGAGTTTGAGGTC

GGGGAGTGGGTTTATGTGAAGCTACGACCTTATCGTCATATGTCTTTAGC

AAAGAAAGCAAATGAGAAGTTATCCCTGTGGTTCTTCGGTCCCTATCAAG

TGGTGTCTCGGATTGGCGTAGTCGCATACAAGTTGGACCTACCGTTCGCC

ACCTCCATTCATCCCGTGTTCCATGTTTCGGTGTTGAAGAAATCGGTGGG

CAATGTTACTCGGGTACAACCTTTACCGGAGATTCTTACACACGATATGG

AGTGGTTTGTGGAACCTGAAGCCATGCTAGGAATGCGTTACAACGTGCAA

GGGAAATTGGAAGTGTTGATCAAATGGACAAATCTTCCTGATTTTGAAGC

TTCGTGGGAAGAATTCGAGCTTTTGAATGCCCAATTCCCTACTTTCCACC

TTGAGGACAAGGTGAAAGTCCAAGGAGGAGGTATTGATAGACCATTAATC

AAATGCACCTATGTGAGGAATAACAAACAGAGCGTCTAGAAGAAATGCTT

GTTGAAATTAGTTGAGAAGCTTCTCCACAAATGGGGTCAAGTTGTTGAAG

TTGTTTGACAAGTTGGTTGTTAGGGGAGTTAGTTAGGAGGAGGATCTTAG

CCTAAATAAATAGAAGGTCATTGGGAGGGAAAAGGGACCGGAAATTTGAA

AGTAGAAAGGGAGAGTCCAGGCTCTCGAACACCTGGTTATTTGTTCTATC

ATTTTGCTTGTATTTTCTTTCATTTAGCTTTGATAATTCAAGTCCAATTC

AGCCTATATTGCATTTATCTGTTTTTGTTCACTACAAAATTCTCTATTTG

GTGAGGGTGATCTAACATAGTCAACGCATTAATTAGTGCACTTTAAAAAG

CATCCACTGTATGACTTAATTACAAACTTTAGAAAGTATAAATACTCAAT

CAAGAGTATTGAAAGTAGAGGGGCTTAAACAGAATAACTGACGTATTACA

TGCACTTATGTTGCAATTTGGCCAAAAGTTTCATTGTTAAGGGAATATTT

TTTTGTAGGTATTAAATAATGGGAATATATATCTTACATTGTAATTATTA
CTAATAGCATCGATCATTTACTTCTCAATTTTTTTTTCTTGACATTTACA
AGAGGAAATGATTTTCAAGTATTGGCTGCTACATCGCACATTGGTGCATA
ACGGATTTGGAGGAGGCGATAGTGTCATGGGCTGATATGTCAGTATGACT
GAAATTTACAGTACAAACAGATGTTGAGGAACCTTGAACTGATATGGTGC
TTTTAACACCTACCCTCACATGCAAACTTAATTTGCATGTGGAGCGATTT
GACAAAGTCATAGTGGCCAAAACAGAGATCAAAGATGGACTCACGTCTCG
TAGAGAAACGAGAGAGACTTTGCACGTGATACCATCTTAAAAGATTCTAA
ACTAAAACCATACAAGTATAGGTGTAGTAGTTCATTTGACTAATATGTTG
AGAAGTCCTGTCAAACCGACATGGGACTTCACAAAAAGCTTGAACTAGGG
GAGTATCTTCTCACTGTTTTCATATACTTAATGAAGATACTACACGAAGT
CTACCCCTAGTTTACCATGTTTTAGGTCAAGGGTCCAATTATGTTCCCTT
TTCCATGTTTAAGGTAGCATTGTTCTTGACATGAACCACATGGGAATCTT
ATCATACTAGTAAGCACCTACACTAGGGTAGCATTAGGTTATCCGGTGAC
TGAGGGGATAGAATCTTATGCATTAGTTTTGTTTGAAGGAGGCATAACTT
CTGTTGCCAACTGATAGCATGTGTTCAAACTGCTTACATTATCATAGGCT
GTTGTTTTCGAGGTTATATGACAAACAAATACTCGCTGCGAAGGGGAAAA
TACGATTTGTAGTCCTAAGATTCGATTTATGTCCATTGGTTCTGGTCTTA
AATTGTTGATTCTTTCCCCATCCCCTTTTTGTTCGTTGCTTGCTTCTCTA
AATAATCATTTTTTTTGGCTGGTTCTTATAAAACTCATTTATGTATTAGG
TAAACAGCTGCTTGTCGTATTCGGACCGAGTTCCAGATGGTTTCTACTTG
ATTTCTGGGATGGATCCATACATTTGGACTGTATGCACTGATCTTCAAGA
GAACGGCCGTATTCCATCAATTGAATCATTAAGAGCTGTTGATCCTGGCA
TTGAATCCTTAGTTGAAGTGATTTTGATAGATTTTCTTAGTGATCCTAGC
TTGAAGGAACTACAGAGCCGTATTCAGAATGTGTCTTCAGGTTGTATCAC
CACAAAAGAGGTTGTTGACCAGCTTGCAAAGATGGTCTGCAGTTGCATGG
GGTGAGTTGTTGCACAGCATAGTAAGAAACTCCAACAATATGTCTCTCTT
GTATTCTTTTTGAACTAAGTTTTGTTGCCTTCACAAGGGGTGCAGCTCTC
ATTGGAGAAGATGACTTGGTTCCTATCTGGAAAAAATGCAGTGATGATCT
AAAGGATTGTTTAGGATCTATGGTGCTCCCAATTGGTCGGCTGTCTGTTG
GTCTTTGTAGACATCGTGCTTTGCTATTCAAAGTATGTCTTGGTTACATT
TATTGATCAGTGCGCATTATTTCAAGATTCCAATCTCATTCCACTTGAAG
GCATTTTAATAGCTTTGTTTCACCTGATTTTTTATCCTATTTCAATTGTA
TTCTTCTGTCAGTTAAAGCTCAAAGTGTTCATGTATATATATGATCTGAG
ATCGTATTTGGTACTTCCCCTCCTTTGTCTTGTTATTGTAAACCAGAACT
CGTGGCACATGCGATGCATCTGCACTTATATTATTATTCTTAATCAAAAC
ATGTGTATTCTATTTTTTGTCAGTTATATAGTTTTACAATGATGTATGCA
TTCATATTATAGAAAGTTAGTTATTACTTTACAATGGGTATTAATATGGG
AAGGATAGCATTGACGATTAACGTGCTAAAGACACCAAAATAACTGCCAA
CACTGACTCATGCCTAACATATTGTCTAAATAATTAAGGGGAGAAACATA
TTTGGTGGATGACGTGTGAGTGGGGTTTTCATAAGCACTTCAAATATGAT
TTCTGCAGGTGCTAGCAGACACAATTGATTTACCATGTCGAATTGCCAAT
GGATGCAAATATTGCACAAGAAATGATGCATCCTCATGTCTTGTTCGTTT
TGGGATTGACAGGTATACTTCAATAATCAACAAGATATCCAAACCTTTTT

ATTTTGGTCAGATTTTGATTTTTGGAATCCATTCTGCACAAACTTTGGTG
ATCTCAAGCTTCCTAAGATGGTGGTATGTTACTGCCTTGAGCATTTTTTT
AGTCAAGAAGTGGTCTTGTAGCGAATAGTGTGATGCTAATGCCTAATATA
TTGGTAGAAGGTGCCGCTATTATACTTACTAGGTTTTCTAATCATATTGT
TGTTGGTTAGAGCAGTGCCATGCATTATACTCATGCACCTCGGAACCATC
TCATGTCTTCTTTTCCTCCTGTTCATATTTTTTTTCCACAATAACTTGTG
AATACTTTTTGGTTAGGTTTGTGTATTTTGTGTCTCATGCTCATATTTCT
GTTATTTTAGTTTGAACTTTGAAGCGAGAATGCTTTTGAACCGGGTTTTT
GTTGGTTCAGTCTAGAGAATGGTTTGTGAATTTATACATTGCTAATGCAA
TGGTCAATCTTTTGTTTGTGAATAACTTGACAATATTCTTTTCCTGCAGA
ATATTTAATTTTCTCTACTTCAGGCTTTATTGTTGTCCTTTCTGTGTGGT
TTATTGGCATTTGGTGGCAACTGCATCTTTTAGATATATGCAATCACTAG
GAGAACTGTCTTGTCCAGTAAGTAAAATTGTCTGTATCCTTACAGGGAAT
ATTTTGTTGATTTGATTGCAAAGCCAGGATGCTTATTTGAGCCTGATTCA
TTGCTCAACAGTCCATCTTCGATATCAATTTCTTCACCGTTGCGCTTCTT
CCCACGGGTCAGGCAGGCTGAACCTACATCTGATTTCAGGTCACATGCCA
AACAGTACTTGGCAGACTGCCAATCACTTAATCTTTTATTCGATGATTCA
TCTGCAGGTGATTATTTAGTTCTTTCATTCTTTGAATGTTGTTTGGATAC
TTGCTCTCCACCCCCCTGTCCGTTGCTTACCTTGATGACCTTGATGTTGA
AGAGCCATGGAAGATGTAAAGCTTTTTGAGGATCATGACTTCAATGAAAT
TTCATAAATTCATGCCGATCTTTGTACAAAGAAAATATATAAATCGAAAA
CTTCAATTGTTTTGGTATTTAAGTAAGGAGAAAATTATATTTGTTAGGAG
GTTCCAACTTAAAACCATATGGTATTAAGGGGAGTAACTCCTTTCATATA
TAGACACCTTTGAGAAGCCTCACAAACTCAATGTGGGATTCTAACACTTA
CTCTCACGTGCAGCCTTGCCTTGCACGTGATGCAATCGACAAAGAGAATA
GAAAAGATAAGCATTCAAACAATCGGGGGCAAACAAATGAGGCTTCCCAA
ACCTATCTGATACCATGTTAGGAGGTTCCAAATTAAAATCATATGGTATT
AAGTGGAGTAACTCATTTCATATATAAACTACTTTGGGAAGTCTCGCTAA
TTCAATGTGGGATTCTAACAATATTTTTGGTCCTTAAAGTGTGGGGTGGG
TGTCTATGTGGACCCTGAGGTTTTACCAAATTGCACATTTGGTTCCTCAA
GTTTCACATTTTTTTCAATCTTGCAAAATTAAAAATGCCGTTAATGATTT
TGGATGGAAAATGAGTTGTTGTTCGGCATGTGATCTTCTAATGAGGAGAT
ATTTTTGTTATTTCATCACTCTACTAACTCTAAGACGTTTTCCTATATCT
AACTTTTCGTCTGAAGAACCATATAAGGAAGCCTAAATCTTTGTTAGGGG
TGAATGTCTTAGGTTTAATGAAGTGATGAAAATATTACTATTAGAAGGTC
ACGTGCTGAGCACAACCCCTTTTCCGTTCAATCATTAGTAGTATTTTCAA
TTTTGCAGGATTGAAACAGATGTGGAGAACTTGAGAGATTAAATATGCAA
TTTGGCAAATCTCATGGTTCACATAGACATCCACTCCATACATTAGGGAC
CGAAAATGTAGTTTTCCACTAAGGAGTTTGAGCTTTTCATTAGTCTACCA
CATGTTTAAAATTTGATATGGAAAGTAAACAGTTGAGCTTCTAAACTGTA
GATCTTCTGTTATAAATGAATACTGCTTCAAATGGCTACCTTTGTTCTTT
TAGATCGGATGTTTATCATGAGAAAAATACAAGGAACGCCACTTTGAGAC
TTGGTGGGATTAGTTATGATGGTTTTGTTATGCATTATAAAATAGTAAGT
CTGAGGAGGCATGTGGTTTATAGGAAGCTCAAGAAATAGAAAATTACGGA

GAAATCTGGAAATTGTTTCATTAGTTTCAAGTCTTACATTTGACTGTAAT
ATGCTGACAAGCCGATTGTCTTTGATGCATATTTCTATTTTTAGTTGCAG
GATCCTTCTAAAATGGTCATGCTCACGTGCCAGACCTGCAATTATACACC
ACTTCTTATTAGGAACCTTGCACCAAACCCGCTTTTATCATGTTAGAACT
TGCTCCCAACACACTTCCGTCATAACTAAGCCTCAAACCAACTTTTGTTC
AAATATTTTAGCTGAATGGATTACCATTATATAGTAAAGAATGAAATAGG
ACTTCTTAAAAGCAAACTGGATTGTTGAATGTATTATTTCGAACCAGAAT
TCATTTCATCATTCAAATAATGTAAATATTTCTCTGAAAATAGTTGCACG
CGTAGTTGTTAAAGTCGCACTTGGGCGCAAGGTGCACGCAGGCACTACTT
GTGCGCCTCGCCTAGGTCAAGGTTCAGGCCTTGCATAAGGCGCTCTTCTT
AGGGGCTGAGGCTCTTCTGCTTCTCTGCTTCTGTTTCTGCTGCTGCTTCT
GCTTCTTCTTCTTCCTTTCTGTTTTTCTCTCTTTTTTTTTTCTTTTTCAT
TTCTCGTTTTCATTGCGGTTTTTATTTTTTATTTTTATTTTATGTTATTG
TTGTTAGTAAATATTTCATTTTTATACTATAATTGTTGATCAATTATTTA
ATTTTATGCTAAAATCTCTATTTATTTGGAATCAAATTAGTTTATTTTTA
TTTATTGGCTCATATGAGAGTGATTTTTTTTTTTAAATAATTGAATTTTA
GTTCACAATTTCACTTTTTGAGTCTAGTTTTACAATATAACAATACTATA
AATTATTCTCTATTTTTTTTTGTTGTGCTCCTTACTTCGCCTCGCCTTGT
GCCTTGCGCGTAGGCTCCAGGGAACCTTTGCGCCTCGGTGTGCTTAGAGA
ATAACTATGGTTGCACGGTCTTAAGGGCTTCAAACCTTTAAACTCCTAGA
CTCAAACAACCACACTCACGCTCCCCGCCCCCCCGCTTTGCGCGTCTAAG
TTTCGACGTGGATGCTTGGCAAACCACACTTTTATATGTGTTTCTATCCT
ATGCTGCTGCCACAGTTTGGAGGGTGAGTGGCAGATTTTCTTTTAGGTTT
CTTCAAAATCCCCAAAACTACGGGAAGAAGGCTTGAGTGGCTTTAGACAA
GTGGTGAGGGAAATAAACTTGAGAGGGAAGCTTTAGTTCAATCGTGAGAC
CTTGTTATGTCAAACCACTGAGGATGGTTGTACTGTTGCAAAGGGAAGAA
CAGGAGGGAGTCAGTGGCTATCTGGAGGATGAGGCTTGACCTTTTGGTGG
CTTTGATTCATGGGGTTGTTTCCAAGGTGAAAGCAAGGTCATACAACCCT
TGTTTCTTTGTAGAACTCCCTGAAATTGAGTTACAATGCATGGATTGAGG
TGGTGGTGAGGTTCCATTAGATTGGAGGTGGTAGAAAAAGCAATTTCGCT
GCTTCTGTTGGAGTTGCTGCTTCTTCAAGGCCATTTCAGGCAAACGTGGT
CTAGAGGAGTGGAACTTCTTGTGTGAGTGTGACATTGCCGGACCCACTAG
CCACGGAGGTGGCATCGGTCTTGTGAGAGGGGGATTAGATTCAGTTGGGC
AGAGATTCTTGCCTCTTTCACATCCATTTTTTCAAGAAGATTCTGGTCAG
GATTATAGCGCTAGTTTTTGAGCGGTGTCAGAATTGCGGAAATGGGCGAA
TACAAAATGGAGAGTTCCGTCAGGCATCCAGGTGAAGGAATTGAATTCGC
ACCTTTAAATTTTACTTTTCTGAAGCTGCAGCACCGTGGAGTGCTGGAAG
GTGGGAGATGGGTGATGGATGGGTTGGAAGCGTGGTTGGACTTGTGGTGT
TGTGTTTGGGATGTCTCATGTATTGGGCAATTGGAGAAGACGATATGGAT
ACAAGTGGTGGCTGGGGTACCGGTTTTCTTGTGGGGTGAAGAGTTTTTTA
GAGCAATTGGCGAGCAATGCAGAGCTTTTGTTAGGTTGGATGGGGATACG
AGGCGTCAGAGATGGAATTCCGGCGGAGATATAGGTGGGGATGGGGGGAC
ATGGAGGGTTCGAGTCCCTCTGTGTGAGGATTGTTGGGCCACTGGTTTAG
AATCTGGTCAGATGGTCTTTGTCGGTGACAAGATCGAGGCCAAGGTGGGA

AGGGGGAAGGGAGTATACATGTGTGCACGGGAGTTCAATTGGGTGGAGGG
GCCGCCATTGGCAGTGATTTAGAAGGTAAGGATGCAGAACAACTGTTATT
TTCAAAATAATTGCAGGACAACTTGCTTGGGTCGGGTCGGTTGTCGTAGG
TGGGCAGGCGGTTTTGGGCGCTCCAAATAGGGGTGAGGGGATTGTGAGGA
CTGGAAGAGGGATTGAATGTGTTGGTACTTCTGTGTTATCCTGGGAACAA
GGCAAGCCCCAATTAAACCCTACTCCTTTAGTGGCTAGTGGGCCTACTGA
AGCGTTTGGGGGCATGGGCCAGGTTGGTTTGAGAGGAAAGCTTTTGAACT
TGTATTCAAATGGGGTTGGGCTCTTAGGGGCTATTTTTGTTCAGTTCAGG
ATACCATGATGGGCCATAGAATAATTGCCTCTTCTAGGATGAAATAGTCG
CCAACTCAGGAGGTGAGGGGCACTGTTTCTGTGAACAGGGAGGATGGGCC
CAAAATATTGATGGCGGAAGAGGAGTTCGGCAAACCTTCTGAGGCAGTGG
TTTGTCATTGTTCTCAAACCTCTTTTAAGCCCCAAATTTCCTTCCCTCAA
ACTTCAGGTTGAAAAGCAGCGGCAACATGGGGTCTTACAGATTAAATTCT
GAAGTGAGGGATTGAAGATTTAGGGCTGATTGTTCAGGAAACGGCTTCAT
GCGGGGACCCCATTGAGACAACATGTATTTGGGAAATTTAAACTATAATT
TTACAGGTCATCTGGTTGCTTTTGAAGAACAACACTACGCGTCGAGAGTT
AATGGCTCGAATTTGGTTACAGTGCTCAGGAAGGATTAAGTGGCTGATTC
TATTGAGAGGAAACCTTCAGAATGGCTTCTAGCAAAAATAAAGCTGTCAA
AGTTGATAGTTTCAGTGGATTTTTGAAGGCAATTTGGTGGGATTCTTTTT
GGAGGTGGAGAAGAAGAGAGTAAGATTTGGCAGTGGCAAGCCCAAGAAGA
GGAGAGGCTATTTAGAGAGGTGAAGAATGTAATATCTGGCTTTATGATGG
TAGGTGCGAAGTGGGGGAGAGTAGGGAAGAGGGAGGGGGGAGATGAGGTT
GTTTTACTTGACTCTTGTGTTTGAGATGAAGTTGAATTTTTTTTCGTGGA
ATTTGTGGGGGTGAATGATATGACAACAAGGGGTAGTTAGGAGGGAGGTG
TTGACAACATGAGCGGCGGATGTAGCGTGCCTCCAAGAAACAAAGATGGA
AGAGTTGAATTAAGATTGCTTGAAAAATTTGGAATTATAAATGGGTGGAT
GGGGTTTTGATCATTGCAGTTGGGACAACCGATGTTTTTTTTTTTGACAT
GGGACACTAGGGTGGTGCAAAAATCGATATGGAATTCATACGATGGCTTG
CTACTTCTAATCGGTGGTCTATGGGTTTGAGTGATAGTTTGTTGGTTTAG
ATGATCACCTGTGTCCTATTATGTGGGGATGGGCTCAAGCTTTTGGGCTT
GTAGTCGAGTAGGTTGGTATGCTATGGTGGGATTATAATGTTATTCACTT
TCCGCATGAAATTAGTACCTTGATGGCCATGGAGGTGGTTTCATTGATGG
AGAAGGGTTGATTGATTTGCTCCTAGTTGAAGGGGCATTTACTTGGAGTG
ATGCAAGAAATCTCTCTTGTATGTTGCTCATTTCCCTGCTATGGTTGAGT
GTTGCTTGTCTAGGCCAACTTCCGATCCTGGTCCCATGTTGCTTCATTGT
GGGGAGCTTAGTTGAGGGAGGTTACCATTTAGATTTAAGAATATGTGGCT
CATAGTTGATAGATTCTTTGTTAGTGGGTGGTGTTTTAACTATGAGGGGT
TTCAGAACTCGAGTTTTGCTTTTGCACCTTTTGACCTTGTTGAAGCTGGA
TCACGAGAATTGGAATAAAGAGGCTTTTGGAAGGTTGTAGGTGACTAAGG
CATGCTTACTTGATGAGATGCATAGATCAGTTGGGTCCAGTAGTAAAAGA
GGAATAATCGGCGATTAGAGGTAGTTTCTATTAGAATCCCATCTCACCTT
GTTTTGTGGGACTTCATTTAAGGATCTTTTTCACCTAGTTCCATATGGTT
TTAGGTTGGAACATTCTAACATGGTCTCTGAACTTTAGAAAGTCTTTTTG
GAAGTCTACACTTTCTCTTTCTCCCTTGTAGTCAAATTACTTGTGCAAGT

TTGACGTTGCACACTGCTTCCCATAATCAATCTTTCATGCAAATCGGATT
CCAAGTAAGGGTGAGTTTTAGCATTCCATCCCATGTTGGTTTCATGAGAC
CGTCCAAACCATCACATATATTAAAGGAGCTACTCCACTTAGTGGCATAT
GTCTTTAGGTTAAAACTCTCTAACAATGTCAAAGGTGAGTTGATTGAAGT
CTTCTCTGTGAAGTGATGCGTTGGAGGCAAAAGTCGAGGGAATTGTGGCT
TTGAGAAGGTGACAGGAATACTATCTACTCTTGTCTTGTAAGAGAAGGAA
TTCTGTGGCTTCTCTTCTAGTTGATTGAAATCAGGTGCATGCAAGGAGAA
TGTGACTATCAGGGGCTTGTCTTTGAATCAATAACTCTTAGACTTTCTTT
GGATGACCTTGTCTTTTAGGGCATTTGGATGAAAGATGAGGGTTTTTGGA
GGAGGATTTATTGAAAATTGTTGGTGCTTTGAAGTCTTTTCCAAAATCAA
GTTCTAGGGGGTTGGGACAATGGGATTCGCTTTCTCCGTCCTCCTTTTTC
ATTCTTATGAAAGGATAGAGTATCCAAGAGGCGAGAGCAACGAAGATGCC
AATGTTTGGTGCCTTTTTTGGTTGGTAACAAAGAGGGATCTGTGGTGAGA
GTTTTCTATTTATTTGTGGATGCCATGCTTCAATTTTGTCAGCTACGGGT
GCTTCATGTTGTTGTACTTCGAGACAAATTCTAGTTTGTAGGTTAACTTT
GGCAAATTCGATATTTTACTGTTTGGTCCATGTGAGTGGTGTTGATGCTT
TAGCATTGACCTTGTGTTATGGTGTGGGGTCCTTGCCTTGTATTGGGCTA
TCTTTTGGAGCTAAATACGAGTTTAGTGTTATTTGGGAACTGGTGTTGAG
AGGTTTAAGAAGATTTTGGCAGCTTGGATGGTCAGAAAGGTTCAATCTTA
TCGAGAGAACATAAACAAAATACATGCATTGCAAGCTTGGTAATACTCGG
AGAGGTGTATGGTAAAATTTGGAGATTAGGAGGCCCATAGGGTTGATCAT
TTTGTTTGGGTACTTGTATTCTATAATTTAGGAGAATAGAGAAATCAAGG
ACATTACACGTAGAATCAACGAGGACGGATAAGTGGATGGATAAGTGGAG
AAGTGCAGTTGGGGTGATTTGTGACCGTAGGATACCATTGAATTTAAACA
AAAATTCCACAAAACTGTAATAAGAACCATCAATGTCCTTTTATGGATCC
TCTACAATAAGATTGACTATTCTCTATGGTTATAAATGTTGGGCGGTAAA
CAAGAAGCTTGTTCAAAAAGTAGGTATAATGGAGATGTGAATGTTAAGGT
GGATGAGTGGTAAGATGAGAAGGGATAAAATTAGCAATGAGATTATTAGG
GAGATTGGGGGAATAACATCCGTTGAAGATAAGATAAAAAAAAAGTATTC
AATATGGTTTGGACATTTGCATAGAAGACTTGCGGATGTACAAATGAAAA
AAAAAAAAAGGTGTAATGGAGATGTGAATATTAAGGTGTATGAGTGGTAA
GACAAGAATGAATAAAATTAGCAATGAGATTATGAGGGAGAGTTTGGGAA
TAACACCCTTGAAGATAAGATGAGAGACAATTGCTTATGATGGTTTGAAC
ATGTGCATAGAAGACTTGCGGACATACAAATGCGAAATGTAATTTAATTC
AAGTTGAACGTACTCCAAGGGGGAGAGATAGACCTAAGAAAACTTGGAAT
GAGACAATTAGGAATGATCTGATGACGTTGAACTTAACTGAGGAGATGAC
CGTTGATTGTCAATCTGTATGGTGCAAACAGATTCATGTAGACGACCCTA
TTTAGATTGGATAAAGGTTTTTTTTGTTGGATGATATTTGATGTCTATGT
CAGGATTTTGGTCCTTAACGTGGGTTTATCTTGAAGGGGCAAAATTTTGT
TTGTGAAGCTCGCCAATCTTCTAATTTCTTGGAATGTCATTTTTTTTTGT
TTTTAGTAACACAACTTTTTTTAATATATATATATATATATATATTCAGGTA
TTTGAATTTATAGATTCCCCTTTGGGGGACTCAACTTACTGAAAGCTTAA
GTATTTGTTACAATTTGCATGGGGTTGGTATGCCTCTGAATTTCAAATAT
GAGTTTATTTTGCTTCCACAGTTTTCTTTATGTACTCTTTTTACATCAAA

TTTCAAACATTGCTAAAGATTATGGTTTATACTTTACCTCGCTTAAAAGC
CACCTCATCGGGCATCAACTTTTGAGGGCAGTGCTTGCCCTTTTTCTGTG
CTCGAGATAAAAAATCACTTTCTAAATGTGATGACTAGAAGTGCTACGGA
TAATAATGCTCCACATAAAAGAACTGCATAGCCCAATATCATCATGCTTA
CATGCATTATTAACCACTTGGATTGAAGAGCATGTACTCATAGTGTGGGT
TGATGTATTTCAGTTAAAAGATCCAATGTAGCAAAGCCGTGGGTAAAAAG
AACACTTGGAGCAGTTATTGTGCTTAAATCATTTGGATTTATATATATAT
ATCAATATGGGACAAACTCCATGAAAAATAGGCTTTTAGAGATGAGGCCT
TGTGAAGAAGCCAAGTCCATTTTGGGCCACCGAACCCTGCAACTGATGAG
AAGGCCCTATGGAGTCCAAGGAAGCGTATACGTTGTCACACTCCCTGCCT
TCCACAGGTGCCTAGAGAACTGGACAGACGCAACAAAGTGACGACCCGGG
AGCGGAATCCCCACCGATAGGGGGATAGGAGATGGCCATCTCAAGGCATT
TCATGACCTATAGAGGCTTTCAAGCCAACAAGAAAACTACAATTGAGATA
GTAGACTGCTTTGCGCGTGGGGAATCATTTCTATGGAGGGTTGTTTGTCT
GCTGCCTTTGCCCCTGCTGGATAGCTTTCAATAGATGCTAAAAGATAGAA
GGAGTTCACTTCAGCCAGCTTGCTATGGATGTGAAATTCCCTACTTCTCT
TTCTAATCATATGTGGCTCTAGCTTCACTAACTCGATTCTAGGCGACTGT
CAGACAGAGAAGAAGAATGAGTCTTTCTTCCCGATCTCCGCTCCATATAA
TCCGCAATTAGGGGAGATCTCCTTCCTCAAATTCCTCGCCAGCTATTTCC
TAGTAGGGTCTTTTGTCTAGAATATGACTGACTTAAGTAAGGTAATTGAT
AGGAGTTAGCAGGCGAAGGCAATTGTGAAGACTTTCAGACTTCAATCGAT
TTAAAAAGTTAGTTCAAAGCCTTAGTTCAATGAACGCATTCCAAATGCCT
CGTGCAAGAAAGAAATATTAGTAATGGCTTGAATGAAAGAGAGAGTTATG
CAATGTTGGCTACAGAAAGCAAAACAACCTTCGAAAACACTCTAATCCAA
ATCTTAGATCACCAATAATGGGAAAGGTGCATGATGTTTCTTATCGTTTG
TATACCATGGAACCGTCTACTAAACTTCAAATTTATTTGTTTATTTATAT
CAACTTTATTTATTTATTTATTTTATTTTTTATTTCTCTTGCTAATCTTC
CTCATAACGTGTACTAATGGTTATCGAGTAACTGGCTATTTTAAGTGTTT
GCAAGATTTTTAGATCTAGTTCTTTAAATATTATTCTTCTTGGCCATAGA
ACAGCCTTTTGGTCATCTTTTTGCATTTTCTGCTCCTTCATTTTATTATA
TCTTTTACCACTTTCGATATCCAGTGCTGTTATTTAGATATGTCACTGTA
TGATTTTTCCCTTTGACCTTTGCTTTGTCTTGTTTGATTTATAGATTTTT
TTTAGACCTTGGTGCGGGGGTGGTTAACCTTGGCAGTGCGGGGAAGGTTT
TATTGATCATTTGAAATAGAATTCAAAAATAAGAGGGGGGCGGGGGCTTT
TACCTGACCTCTATAAGAGGTTTGGAGTATTGATTATTGTTTCTTTTCTT
TTTTATTTGGCATCCTTTATCACAAGGGCTTCAATTGTGCACCAGCAGTG
GGGTTTGAACCAGAGAATAGGCTTGCGAACGATTTGGCTTTGAATGGGAC
GGAGGTTGTATTACTTTTCATATTTTTTTTTTTCTTTTTTATATAATTTG
GAGGGCAAGGGCTGCGCTAGGCTGTGCCACCAGAGAATGTGTCTTCCGGC
CATTGCCGGCTGTAACCATAGCCATGCCACCAAAACAATCTGCTGGTATC
TGTGGGTTTTCCGGAGATAGGGAGATTTGTGGGAGAGATGGAGATGGCTA
ATAAAGGCCCTGGAATGTTGCAGTTGTTGGGGAGAGGGAGGGCTGGTGAG
GGGAGGGCTGGTGAGGGGAGGGCTGGAGATTGCTGCAGGTGTTTACTGGT
GGGTTGCAGTTGTAGGGAGAATCAGAGGGGTGAGGTCGCGGGAAGGTTGC

GACGGCGAACAGGGGTCATGGTGTGGCAGAAATAGCAGAACTGGGGTTTT
GCTTACAAGGGATTTTCGGCTTTTCCCCAGCAACCAGGGAATACATTAGT
TGAGTCGAGGTCCTTCCTTGATTGATAAATTAATCATCTTTATGCCTGAA
TTTAATGGAACAAGCAAGAATTCGTGAACCACTGTTACTGAATTTTGACA
ATCTGGCAATAGCTCATCAATAAATTCAATCACATGATTCACATCCATGT
AGCGAGACATCGCTTATTTTGGACCTTCTAATTTCTTGAGTAATGGAGAC
ATGAGTGACTTTATTTTACAGTAGCTTTACAATGTAAATTTTCCTCTATT
CTTTTACCTTTCCCTTTTCTTTTCTTCTTCCTTTTCTTTTGAGGTGGTGG
GGTGGTGGTGTTGGTGGTTCTTTTTGTTCTATTCTGCACTGGATGTTATT
GCATTTATGGCCATTTTTTCAGGCACATTCATCTCTAGAGTTTTTGATGT
ATTTCCTCCTGGTGCCTCAAGTGTCCTTTAAGTGTACGTTCATTTATTAC
TGTAGATTTGAGCTTGAGGTTAAAGTTTATTATTATCATTATTACAGGTA
ATATTATTGATGGAGATGCTGGAGGTGCCGTATATCCGCAGCTATCAGAT
AGTAATTATATGGATAAAAATAATGTGACTAGTCCAAGCAACAGCAATGA
ATTTTTACAGTTGCCTTTGCCTCCAATAAATACCAGGCAAAAGACTCGTG
GTAGAGATTCACGACCTCATAAGGTATTTAATGCTCAGACCACTATGGAT
TCAACAAATATAGCCAAGGATTTAGTCGCCTCGAAACATATGCCACCAAT
CTGGCATGGAGATGTCCAACTAAGGGTGTCCCAACCAAAGGTGGACACTA
AGGATATGCACTTTGTTGATAAAAGTCATCTGGTACCAGCTAAACAAAGT
AGGCAACTTACCCTTGATGTCGAAAATTTAGAAATTCCATGGAGTGATCT
CGTTCTGAAGGAGAGAATTGGAGCTGGTAAATTTCCTCCCTGTTTTTTAT
TATGTTGACTGTGATTTTTTTTTTTTTTTTTTGTGGTGTTGAAGGGAATT
TTTTCTTCCCAATCATCTTTAAGAATCTGTACTTGAAAACACCGGTTTCC
ATTTTCCAGGTTCTTTTGGTACCGTTCACCGTGCTGAATGGAATGGCTCA
GTAAGTAATATATGCTTTGTTTCTTTGAGAGAATAGGGTTCTTCTACCGT
ACTGGATTCTAAATTTTTTTGCTATTAACTTACCCGTATAGATTTGTTGA
TGCAACATATGATACATAGTTGGACTTCTGACTGGTCAGAGTAACGGTGG
AGGAAAAGGAAGAACTAGATTTAAAACTTGGCGTCTCAGTCTTCCCCGGG
ATTTAACTCTTTTATCATGCATTACATACTCAATACTTGAGGAAGACACA
AACCGTTGTAAATGAAAATTCATCGTAACTCTTTCTGATAATGTAGTTAT
TTCAACATATATTTGGTGGAACATAACTTGGCAAATGATGATCTAGTGAA
CTTTATCTAGATGACCCGGTTGCGCAAGTTTGACCGTATAAGGCCCATAA
AAAAAATGATCTCCTACGATTTTAAATTTGTGGAACTACCATTGACTATA
GACACGCAATACAATATTGTTGAATCCCACATTACATTTGTGGGGCCTTT
CAAAGTAATCTTTATATGAAAGGAGTTACTTTACCTAATGCCATATGGTT
TGAGGTTGGAATATGATAGACCATTAATCAAGCACACTTATGTGAGACAA
AGGAAAAAGGAGAAAGGTACAACTTAGTTGGCCTAGAAGCTTAGTTGGCA
TAGAAGCTTCCATTTTCGGTTAGGAGGTTGAGTTGTTAGGCTAACAGAAT
GTATTCTGTTATTCAGCTGCATAAGTTGCTACTATTGGGGTTATAAATAG
TAGAGGGGAAGAGGAGAGGGGCACCGAAAATCTGAAGTAGAAATTGGGAG
AAACCAGGTTCTCGAATTCCTGGATATTTGTTCATTTCCTTTATTGTTTT
TCTTTCAATCAAAGTTGTTTTGTATTTCATTATATCGGTGAATGCAATTA
CAGTCCATTTACTTAAATCAATTCTACTATATTTCCTATTTGGTTACATT
TGGTCTATCAAATTGGTAATCAGAGCAGTTTCAATCCTGGAAAACATGGC

TCAAAAGAAGGTTGAAGCATTGGAGGAGAAGATGGACCTTGTTCATTTAG
AATGGCAGAGGGAATTTGACTCTGTGCGGACTGAAGTTCAGAAGATTGGA
AGCTTGGAGAAGGCAATCACGCTGATGATAGGGAAATTCGAGCAGTCCGT
GAGAAGAGATGATACGTGTTCTTCATCACTAGTGGAAGAAAAGCAGAAAC
CGTCTGAGATGATGGAATCGCAGACCTTGGGAGGAGGAATTACGACTGAA
TTTATGGGGAATAGATCGTTCCGGAGATTGGAACTACCAATCTTCACAGG
AACTGATCCTGATGGATGGATTTTTAGAGCTAAGAGATATTATGCGATGA
ATAAGATTGGCGATGGAGAGAAATTGGAAACAGTGGCTTTGAGTATGGAA
GGGAAGGCTCTTGCATGGTATCTATATGTAGAAGATAGACTCAAATTTCA
TACTTGGAACGATTTCAAAATGGCTTTTCTGCATCGTTTTCGATCGAGTA
TGGAGGGATCCCTTTGCGAGAAATTTCTGGCAATACGCCAGGAGTCTTCT
GTTGAAGATTATCAGGAGTGATTCGAAATCTTGGCATCTCCATTGAAGGG
GCTATCGGAGGAGGTTTTGCAAAGCACATACCTCAATGGATTGAAACCAT
CGATCCGAGCAGAGGTAAGGCTTCTCAAACCTAGAAATTTATTGGAAATG
ATGGATGCAACTCATTTGATCGAAGATAAGAACAGGATACTTCAATCCAC
TCGGGTCGGGTCTGGATCCACTGGGTCATGGAGCAAACCGTTAGGCGTTG
GGTCGGGTTCAGTTTATTCTAGCCCGAAGTATTCGTCGAACTTAGCAGCG
ACGACGATTCGACCCTCAACTTCGTTCCGATCATCGTTGACGACAAATAC
TGGTTATGGTGGAGCAAATCAGAGGAAAACACCGCCTGAAGAAACGTCGG
CGACACGGTTTTTGTTGAATTACAGGCAGTACACCGACTCTGAGTTGCTG
AACCGGCGTGCGAAAGGATTATGTTTCAAATATGAAGACAAATATTCTCC
AGGTCATGTTTGCAAGAATCGCAAGCTGCAGGTCTTGATCGTTGCTGATG
TTGGTAAAATAGAAGAAGCAGGTGATGATTATGAAGTAGAAGAGAGTCTC
GAGCTGGTGGAAGGTGCAATAGAATTATCAATGAATTCTGTAATGGGATT
TACTGACCCACATACCATGAAATTGCGAGGGAAAATTGGACCACAAACAG
TGACGGTATTGATAAATTGCGGCGCATCGCATAACTTCATCTCTAAAGAT
GTTGTGCAGCGGCTGGATCTACCCGTAACAGCAACCACGGCTAGGGATGG
TCGTGGGCCGGGCCGGGCTTAGACTAGTGTATTACAAAAAAACGTTGGGC
CCTAGCCCGGCCCGGCCCAGTGATCGGGCCTTAATTCTATGCCCAAGCCC
GGCCCTATAAGAAAAATCTAAGCCTAAAGCCCGGCCCTATTTTTTTTTT
AAATTTCAAAAATAAATACGAAAAATTCAATATCAAACTAATAAAAAATA
TTAAAATTACAATTTCTAAAGGCAAACCAATATTCCAAACAAACCAAACC
AACAAAAAATATTAAAATTACAATTTCTAAAGGAAACCAATATTCCAAAT
ATTAGGGTTGTATTGTAATGTATGTATGTATATATATATTTTATTTTATT
TTTTATTTTTTTAAATATCCTTCGGGCCGGGCCGGCCCGGCTCTAAAGGC
CTTGTCCAAGCCCGACCCTAATTTTGATCGGGCCCTAATACTTAGGCCCA
AGCTCTGCCCATGGGCTCAAACCTTAAGGCCAAGCCCTAAAAAAATAGGG
CCAGGCCATTCGGGCCGAGGGGGCCCCCAGGCCCATGACCATCCCTAACC
ACGGCGTACAAAGTGATTATTGGAACGGGAGAAACAGTGGGTGCAGACGG
AATTTGCAAAGGTGTGGTGCTACTGTCCCTTCCTGAAGTAGAGGTGGTGG
AAGATTTTTTACCACTGAAGTTTGGCAAAGAAGATGTTATTTTGGGAATG
CAGTGGTTGACGAAATTGGGAGCAATGCAAGTTAATTGGAAAGCCTTAAC
TATGAAATTTACTTTGGGACAGACTCTGGTGACATTGAAGGGAGAGTCGG
GATTATGCAAAACACAGATATTGCTCAAAGCGATGATGCGTACTATACAA

CATGAGGGACATGGCTATTTGTTAGAACTGGAGTCTCTAGCGGCACAGAA
CGACAAAGACAAAGCGGCTATGGAAGTTCCCCACATTTTCGATGAGCTGT
TACAAGGATATCCGGAAGTGGTGAACGAGCCGAAGGGATTACCCCCTCAA
CGGGCGCTAGATCACGGCATCATTTTGAAGGAAGGAGTGGAACCAGTGAG
CGTGAGACCATATCAGTATCCACAGGTGCAAAAAGACGAGATCGAACGAT
TGGTCGATGATATGCTGGTAGCAGGTATCATTCAACCAAGTCATAGTCCA
TTTTCCAGCTCTGTTATTTTGGTAAAAAAGAAAGATGGCAGTTGGAGATT
TTGCGTGGATTATAGGGCTTTAAATCGGGTAACTATACCCGACAAATTTC
CTATTCCGGTAATAGATGAACTCTTGGATGAATTATATGGGGCACATTTT
TTCTCAAAACTAGATCTCAAGTCGGGATACTATTAAATTCGAGTGAGCCC
ACGTAATGTGCCTAAAATGGCCTTCATGACACATGAGGGTCACTATGAGT
TCCTTGTGATGCCGTTTGGTTTAATGAATGCCCTAGCCACTTTCCAAGCA
TTGATGAATGAGGTATTTAAAAAGTACCTTCGGAAATTTGTGCTCGTTTT
TTTTGACGATATTCTGGTGTATAGTAAGTCCATGGAAGATCATCTGGAGC
ATTTACGGACGGTATTGGAGATCTTGAAGGAACATTCATTGGTAGTAAAC
CTCAAAAAATGTGTTTTTAGGCAACGGGAGCTAGAGTACTTGGGTCATAT
AGTCTCGGCAGCAGGAGTGGCTGATTCCAAGAAGATCCAGGCGATGGTAG
AATGGCCAACGCTGACTAACTTGAAAGCCCTCCGAGGGTTCTTGGGTTTA
ACGGGGTACTATCGAAAGTTTGTGCGGAATTATGGGCACATGGCGGCATC
ACTCACAAATCAATTAAAAAAAGACCGTTTTGGGTGAAATGATGACGCGC
AATAGGCATTTGAAGTTTTAAAAGTGGTGATGACAACGGTACCTGTATTA
GTCTTTCCTAATTTTCATCAACCGTTTGTGGTGGAAACTGATGCCTCTGG
TGTTGGTCTTGGTGCTGTGTTAATGCAAAATCAGCAACTGATTGCATTTT
TTAGCCAGGTCCTTAGCCAAAGGGATCGGACTAAGTCGGTATACGAACGC
AAGCTAATGGCTATTGTCCTCACTGTCCAAAAGTGGCGACCTTATCCTTT
AGGACGCAAATTCATTGTGCGTACTGATCAGAGGAGTTAGCGATTTTTGC
TTGATCAACGCATGGTGTCTGAGGAGTATTAGAAATGGGTTTCAAAATTG
TGGGGTTACAACTTCGACATTGAATATCGCCCAGGAATTGAGAACAAAGT
GGTCGATGCTTTGTCGCGAAGAGAGGAAGGTTGCGAGCTTACCGTTTTCA
CGTTTTCATGCTGTGCACAAGTTGATACGCTGCAAAACGAGATTGATTCC
GATTGTATTTATGGACCCATCAAGCAACAGCTACTACAAGGGTCGGATTT
GTTTCCTGGCTATAGTATCCGACAGGGCATTCTGTTGTACAAAGGATGAA
TGGTGTTTCTGTCAGAATCTAATCTCATTCCACAACTATTAGACGAATAT
CATGGGGGCAAGATTGGAGGCCACTCTGGGGTCTTAAAAACATTTCAACG
TTTATCCCGAGATGTTTATTGGCGAGGGATGACGAAAAAAATACAGTGTT
TTGTGGCTGAATGTTCCATTTGTCAGCAAAATAAAATGTGTCTTAGTCCA
GCCGGACTACTACAACCGCTACCTATTCCAGATCAAGTTTTCGAAGATTT
GTCCATGGATTTTGTGGAAGGTTTGCCTATGGCCGAGGGGTTCAATTGTT
TGTTTGTGGTGGTAGACCGTCTTTCGAAATACATTCATTTTATTCCACTC
AAACATCCTTTCACTGCCCAAGATGTTGCGGCCATATTCGTGAAGGAGGT
AATCCGTTTGCATGGGGTTCTCGGCTCCATCGTCTTTGATCGTGGTAAAG
TCTTCCTCAGCAAGTTTTGGACGGAGTTGTTTTGTCTGTAAGGCACAACG
TTGAAGCACAATACCGCTTATCATCTGCAAACAGACGGGCAAACCGAGGT
GGTCAACCGAGGCATTGAGACCTATTTACGATGTTTTGCGGGTGACAATC

CCAAACAGTGGGTTAAGTGGTTACCATGGACAGAGTATTGGTACAATACC
TCATTCCACACATCCACTCAGACCACTCCATTTCGAGTCCTCTATGGTCG
TGATCCACCACCACTGCTGCGTTTTGAAATCGGATCCACTCCGATATCTG
AGGTGGATTTTCAATTACAAACACGAGATCAAATCTTGAGGGAACTCAAA
AACCACCTGCATCGTTCTTAGAAACGCATGAAGGATTATGCGAATAAGAA
GCGCCGCAAAGTCGAGTTTGAAATAGGAGATTGGGTTTATGTGAAGCTCT
GGCCTTATCGTCACAAATCCTTGGCAAGGAAAGCGAATGAGAAGTTGTCT
CCTAAGTTTTTTGGCCCTTATCCGGTGCTGTCTAAAGTGGGCATAGTGGC
ATATCGATTGGCCCTGCCACCTACTACTTCCATTCATCCAGTATTCCATG
TTTCGGTGTTGAAGAAAGCAGTGGGCTCTAATATCAGTGTGCAACCCATC
CCGGAGATCCTTACGGATAAGTTGGAGTGGCATGTGCAACCAGCATCGGT
GGCAGGATTGCGCTACAATGTGCACGAGAAGTTGGAGGTATTGATTCAGT
GGCAAGATCTTCCAGATTTTGAAGCATCGTGGGAAGAGTTCGAGTTTTTG
AACACGCAATTTCCCGCCTTTCACTTTGAGGACAAGGTGAGATCTCGAGG
GGGAGGTATTGATAGACCATTAATCAAGCACATTTATGTGAGACAAAGGA
ATAAGGAGAAAAATGCAGCTTAGTTGGCATAGAAACTTCCATTTTCGGTT
AGAAGGTTGAGTTGTTAGGCTAACAGAATATATTTTGTTATTCAGCTACA
TAAGTTGCTACTATTGGGGTTATAAATAGTAGAGGGGAAGAGGAGAGGTG
CACCGAAAATCTGAAGTAGAAATTGGGAGAAATCAGGTTCTCGAATTCCT
GGATATTTGTTCATTTCCTTTATTGTTTTTCTTTCAATGAAAGTTGTTTT
TCATTATACCGGTGAATGCAATTACAGTCCATTTACTTAAATCAATTATG
CTATATTTCCTATTTGGTTTCATTTGGTCTATCAGAATATCTTGACATGG
TATCAGGTATGGGTTTGGGAAGGCTTGTTTGTTTGTCCTCTAGTTGTTTG
GTTTCTCTATTCTGTCTTTTGATGTTCATTAATTGCTACCACGTGCCAGT
AGGGGTTGCAGTTGACTGTGGGTGTTGGGAAGCTTCGTCCTCTTCCTCTT
TGAATTGTTGGGCTTCTTTCTTCTGTCTTCATATGTCTATTAATTGCCAC
CATGTGCAAGTCCGGGGTGCACTTTAGGGTGGATATTTGGAAGTCTCATT
TTTTGTCTTCCGATGTTGGTTAATCGCCGCCATGTGCAAGTCGGGGTTGC
ACTTGAGGGTGGTTGTTGAATCCCATATTTCATTTGTGAGGTGTCTCAAT
TTTTTTTGTATATGAAAGGAGTAGCTCGACCTAATGGCATATGATTTAAG
ATTGGAACCTCGTAACAAATATAATTAATGTTTTTTTAGTTACCCCATTT
TTAAGTATTAAATATAAATGGACTCACTTGGGCAAACCTAACTTATTAAA
GCACAAGTTTTAAAAATTTCTTTTATAAAAAGCCCTACTCCCCCCCCCCT
TTTAATGCCAACTGTACCCTTAAGGATAAAATACATTTTCATAAACCAAA
CCTATCCTTTCTCTCTATCTTTCCTTTTACGCTGCCTTTTTTTTCTATCT
CTCCCTCCTTTCGCCACCGACGGCATGCACAGGATTTTCAACGAACTTGA
TGGGAACGGGCGAGGACCTCATATGCCTTGACTAACTCTGATGGCATCTC
AGGATGATTGGAATTTGTGTATGCATCGGCGATTTTGAGCTTTGGAAGAA
TATATAAGGTTCTGATATGAACCAAATCAGTAACTATGAATTTTATTGAA
CAACCAACAGATAACAAGATAAATCAAACATTCGAGAGGCTTGGGAACTC
TCCAAATGAGTGAAGCTCAATGCAAAACAATTCTAGATAATTCCCTGCCC
ATTCTACCATAGCTACTCCTTTTATGCTTACCATTTTCTTTTCCTAACAG
AATAGTAAAACAGATCCCTAACATAAAATATGCCAAACCAACTAACAACC
ACCATTAATCTAATCTACCCAAACTACCCCCTTCTAGAACCTAGGGCCTA

ACATTACCCACCACCCAAACATTTACCTTGTCCTCAAGGTGAAATTCAGG
AAACTATATATTATTTTTCATCGTTGGCAGTTTCCCGGGATCCTACACCA
TTCGTTTAGTTCGACCATGGTGATCTTTGTGAGCTTTGAGCAGTGCCTTG
CGTGAATCAAATTAGGAACCATGGCCCGGCCCAAATTGGAGACCAAAGTT
TTGCCTAGAACAAAAGGTCAGATTACCTCCTGTTGTCTTGAAAGAATTGG
CAACCATGGCCCGGCCCAAATTAGCGACCTTAATCTTTTCTTCTATTTTC
TGGGCCTCTTTTAATGTTTCTTCTATCTTATGGGCCAAATCCCTGGCTCG
GCCCAAGTTGCAAGGCTCCAAATGAAATGGCAAAATAGAATGGTGGCCCA
AGTTGGTGACCTTATCCTTTTTTTCTATCTTATGGGCCTCATCCAAGGCT
CGGCCCACAAAGTTTCCAGGCTCCAAACAGATTGGCAAAGGAGAATGTTG
AGGTTTCATTCGTTCAGCCCGTGTACTATGCTCCTCTTCTGGCTTCAACC
CATCGATAAGAATCTCTAGGGTTACTTCCTCCAGATTTCCGTCTGACGGC
GCTGCAGACTCGAAGAACCTCCGGCGATTCCCTCATACGCTGCCTTTCTG
CTCCAGACTTAGTTCAGTACCAATTAGTTCAGCCCTAATTTCAGATTTCA
GTCCCTGAACAGAAGCACCGAAGGCCACTTCCTCCGATACCTTATCCAAC
GGCGTCGAGACGAAGATGGCGAAATCGTCGACTCTCGTTGCGATCAGATC
TTCGTCGAAATCGTCGACTCTCGTCGCGATTAGAGACGGTGCTCTTTCCG
GGGGAGACGAGAGTGTGTTGGACCTCCGACGATAATCTCGCACACTGCTC
TCCTGCTCCAACACAAGCCTCTGTTCATGGAGTGACCCTGTCTGAGCAGA
TCGAAAGTGCTTCCAAAGCAATCTCTCCAACTCCTCCCACAGAACGAAGG
ATCGGTTCTTTTCGTCCCACTGGTACCACAATAAGGCGTCTCCCTCGAAG
CTGATGTCCACCGCCTCGAGTTTTTCTTCGTTCGTGAACCGGTGGAGAGA
AAAATAGCTTTCTGCCTTCAAGATCCATCCATCGGTATTCGTACTGTCGA
ACGTCGGCAGCTCCAATTTACGAAATCGGAAATCCCGCCGTTGACCGGAA
ACAATGGCAGACCCATTGTGGTTCGGCGTATCTCCGGCAAAAGGGTTCAC
CTCTGTTTCCTTGGCCTTTCTCGTTGATGAAGCGATACGAATCGATTCCT
CTTGCAACTTAGTAAACTGGTCAACTTTTTGTAGCATCATCTGCATTTCT
CCTCGCAGCGAATCCATCTCACGGCGAAGCATCTTCCCAGGCTTCCATGG
TAACACCTTCACAACGTTGAACCTGTATAATTTCTTCTCCTTTATCTTCT
TGTATCAAAGACGGATTATCTTAGTCCGGCCGCCGTACAGAGCTGCGTAT
GCCTCGATGTCGAGTTGCTCGCCGCTCACGATCGGTGTGCGATATTGCGA
CTCCTCGCCGCCGTTGGCATAGATCTCGTCAATCATTTGGGCTGATTACA
AATCAGATTCTAGAAGGAATTGGTTTATCAAATTGCTCTGGTACCAATTT
GATAAGAACCAACACAGTAGCTAAGTATTTCATTCAACAATCTAACGATT
ATAAGGATAAACCAAGTATTCGAGATGCTTGGGAACTCTCCAAATGAGTT
GAGTTCAATACAAAACAATTTACAATTCCTCCATTGCATCAATCCGTTGA
GCAACATTTCTGGCTGCCATCTAGCCAGGTATCAGTTGTCAATGATGAAG
ATGATGATGAACAAATCAATGATCGCACAACAACACCTAGGTACATGCTA
TGATACCAATTTGATATGAACCAAATCAGTAACTATGAATTTTATTGAAC
AACCAACAGATAACAAGATAAACCAAGCATTCGGGAGGCTTGGGAACTCT
CCAAATGAGTTAAGCTCAATGCAAAACAATTCAGAATAATTCCATGCTCA
TTCTACCACAGCTACCCCTTTTATGCTCACCATTTTCTTTCCCTAACAGA
ATAGTAAAATAGATCCCTAACAGAAAATATGCCTAACCAACTAACAACCA
CCATTAATCTAATCTACCCAAACTAACCTTCTTTCTAGAACCTGGGGCCT

AACAGGTTCCTTCTGCGACGACTGAGGAATAGATTGAGTTTGTTGAAGGT
TGATTTTGGCTATGGTGGAGGAATAGATTTTGAGAGAGGAGGGTGATTTT
TGGCGATTGCGGAGGACGAGATTGACACAGATCAACAGATCTCGAGTGGG
ACGATTTTAGCAATGGCGGAGGATTATCCCCTAGTTGTGCTACCACCACG
CCGCAAAATGGGAGCTTGTGTAGAGCCTCTTTCTTTCTGGCGTAACAATG
GTGGAACGTAACAAAAGATTAGGACCCTTGACTTTATGCCACTCTTACTG
AGTCCGTTCTTGCATATGTTATTGGTTGTTGCCTTATCTCGAGAACTTGA
ATACAACAAGAGCTCTTTTTGAACCAGACAAGGAACAAGACGTTTCGCGT
CAGTGTGGCTTTCATGACAACATATGAAATAGTTCAAGCAATTTGATTGT
TTGCTTTCTTTTGAAGATTTTTTTTTATTGTTGTCAATTTTGTAGAGTTT
TGCTGTTGATTTGATTTTTTTGAAACGTTTTCTGGTTATTTATTGTTTTA
TTTGCGGAATTTTGCCAAAATTTATGGTCGTTTGATTTCTTTAAGTAGTT
TTTTGCTTGTTTGCTGTTGATTTTGCAGAAGTTTGTAAATAAATTGTCGT
CATTGGATTTATTTGAAATATATTTTGACTGTTTGTGGTTGATTTTGTTA
TTTTACAATTTTTTTGCTGTCTATTGATTTTTTTTAGTAGTTTTTCGATT
ATTTGCTGTCGATTTGTAGATTTTTTGGCGGGAGTAAAGTTTGGGGGCAT
ACTACTAAAATTATAGTACTTCTTTTTGATTGCTGGAGTTCTTGTGTAGA
AAAAAATAGCAGGGGTTAGTGTCGTAAACAACTAGGCCTTTGGGGCCTAG
CCCGAATTAACCCTATAATTAAAAACTATTCATATAGCTTAGTGATCTTC
CAGCGAAGTACGACTTGAATTCTATAATATTTCCCTTTTTTTAATTTATA
TTTTGAAATATCTTGCTGTCCTATGCTTTCCTTTCATATTCTAGTTATGC
ATTAAGCTCTACCCTTAAGACGCAGCCTGTTTTTCGCCAAAAAGCTGTTG
CTTTATAATAGGTTCATAGGTACACATTTTGGAATAATTATTGGTTACGT
GCCGTTGTTTTTATGTTTAGTCTCATAAGAGCTGTAACTTTCAGGATGTG
GCTGTAAAGATTCTCATGGAGCAAGACTTCCACGCAGAGAAAGTACAAGA
ATTTTTGAGAGAGGTAAGTGTAGGGTAATTTCTGAATGAGAACAACAACC
GAATGTACCAGTTTATATCCTATTTTATTTCTGTTTGTCCCACCATAAAT
TTGTTGTTCCTCTGCGTTTCTTTGTTAGGTTGCGATAATGAAGCGATTGC
GGCATCCAAACATTGTGCTTTTTATGGGTGCTGTCACGCAGCCACCAAAT
TTATCAATAGTAACAGAATATCTATCTAGGTTTTAGTATCTCCTTTTTTA
TATCTTCCACATGAGGATTCTATTACGCAGTCATTTGTAAAAACTATTGA
AGAAGTTTTGCCTGGCTGATTATCTACGATATTACCGAATGCAGAGGTAG
TTTATTTAGACTTTTGCATAAACCTGGTGCAAGGGAAGTGTTGGATGAGA
GGCGCCGATTGGGTATGGCTTATGATGTGGTATGTGTATGTGATTTTTTT
TTTCCATGGCGTTGATGCTTGTATGTATGTATGATATTCTTTTAATTTTA
GACATCTTTTCGTTCCATTCCCTTTTCAACTTGGTATTTAATTTGTTAAA
GTGCGGCTTAATGGCTTGCTACTTTTTTCATAGGCAAAGGGAATGAATTA
TCTCCACAAAAGCAATCCTCCCATTGTTCATCGAGATCTGAAATCTCCGA
ATCTGTTGGTTGACAAAAAATACACTGTGAAGGTAACGCAACAGGTTTCT
TAAGCATAGCATTTGGCATGGAAGTTTTTGCATAGAGAACCTGGTATTTT
ACCACACTTTCATAGTCAACACGTCAAACTGAAATGAACCTATGTAGATT
GCAGTGTTATTCTTGTCAAACCTACCTATTTTCTATCCTTTTAGTACTCT
CGATGGTCGCTGTGCATTGTGGGCCACACATTAAGCTTTTTGGACCACAG
AAATTGAGTTGCCAATTTCAAATCTAAGTTTTATGATAACATGATACAAT

CTGAGGTTGATAGCTTCTTTACCTACTGGTCATCTCTCTTCAATAGATGC
CAACTAAGGACTTCAGGTGTCACATTTTCTGCAGTGGTGGAGAGCAGAAA
AAAACATGTTTATTTGTCTGCATATTGCTTTTTAGTCTTTTTTGAAAAAC
CCTTCAAAGCATGTTGTAGTACACATTTTAATTGATCCAATTGCTATGCA
GCGCATACTCTCAAGTCACTTGTGCAAAAATAAAAATAAAAAATTATTTT
AAAATCTTGTTTATGCTTGGTGTATGCATATGGCTATTGGCATGTGAAAG
TTGCTATGAGAGCATGATGTCTTTGGCAATTGTTTGAGAAAGGGATTTAC
CCTTCTTGGCTGTGAATACATGTTCATAAACCATACCTTCTATTTGTTGC
TGAAGATTGTCCAACATATTTCATGCATTTTATCGTAAGGAAGATTCTTG
GGAAGTGTGATTTTGGAGAGTAATGAGTAAACCTATTTTAGAGTTTGTGG
CAAAGGATATTGGTTTTGTAAGAGTAAGAAAGAGTGCGTTCTTTGAGAAA
AAGCATATAAGAAGAGGAAAGCCCTGGTGTAAATACGCAAGAAATTCTCT
TGTTTTACTTAATTGTACTGATTCCCACTAGGGCATTTTCCTAAATTAAA
TTTTAGGGTTGGATTTTATTGGATATATGATCTATGACTTAACAGCGTGT
TTCCAGGATGGATAGTTATTTGGGACTCACCAAGAAGTACATGACTATAT
AACTATTAGTAAACATTATAGCTGGTAGATCTGACATTTGACTGTTTCCA
CCTTGTTCTGAAATAATTTTCCTTACTACCTGTGAGTGGTGTTTATATCT
AGAAAACCTTAATGCTGCATTTAGCATGCAGGAATGGAATGGGATAAGTA
TGAGATTAGAATGTTTGTGGGATTGGAATAAAACGACCATTGCCATTCCC
ATGTTTAGTTGACTTGATTAAATATAGGAATAGGAATGGAATGAATTCTC
AAATAAAATATGTCATAGATTCATAATAATATTAAAAAAAGGACAAAGTT
TAGTTAAAAAATGAGCGATCGGAGCTCGAACGACGTAAAAAAAAAACCGAA
CTCTGATTTTTTTGTTGAAAAAATTACCAAACAATAAAAATGAAAATATT
TATGAAATTACTGAAGACATATTCGGACTTCCGGCCACATTCGGATTACA
TTTCCAGAGTTTCGACAACATTCTGGTGACATTAGCGGAGTTCTGACAAC
GATCTGGTAACGTTTTTGGAGTTCCGACAACATTTCAACAATGTTGCCAG
AGTTCCAACGACATCCCGTGGAAGTTGCCAGACATTCAGAAACATTCTGG
CGGGGTGGCAGTAGCTCTGGCGACATCATTGGAAAAGGTGACTGGATGAT
AGAAATAATTGCTTTACCAATATTTCTCCATTTTGAATCGCATGACTCAT
CCCACTTAATACTATGTATGATTTTGGATTGCGTAATCCCATGAATTGTG
GGATCAATGAATGAAATGAAAATCATTTGGATTGCCTAATCCCATGAATT
GTAGGATTAACGAATGGAATGAAAATTATCTCGCGTACCAAATATAGAAA
TGGAAGGGTCAATCTTATAATCATTTCATTCCTTAGTTGAATCCCATGTA
CTAAACATGGTGTAAGGCCTCATTTTGAGTAAATAAATGTACTGTTAAGT
AATCTCAGATGTTCGTACATGTAGAGATACGCAGTGAGGAAATGTGTTGC
GAGGAAGACTTGATTAAGATGTAGATTGCCATAGGGTAGACCTAAGAAAG
TGCAGGAGAAACAATGAGGAAAGTCTTGATTATATGTAATCTAACGCAGT
AGATTATTTACAACAAATGAATAAATGCATTGGCGTAAAATAATTCATTT
GGTTGACTCCTAAGTGGGTGAATGACTTTGTTGTTGTCGTAGTTGTAGTT
GTTATTAAGACCATAGAAATGGTGCACTGGAGTTTACTATCACACTTAGG
GGAGGTCAGGCTAGAGACTTCTGCAATTTTAAAGGTCGATAGGGTGGGCT
TTAGTTTGCTATACGGTATAGAATAGGAAAGTTCCACCGACTATTTAAGA
AAATTTCCAAGAGAGTGAGCAATATATTTTCCTCATTTTTTTTTTATTTC
TCAAGGGTGTTAGAAGGTTCCAACCCAAAATCATATGACATCAAGTGGAG

TAACCCCTTTCATTTTTGAGAAGCCTCACAAATGCGATGTGCAATTCAAC
ACCCACCGCCACATGCAACTCCAACTTGCACGTGGTGTAATCAGACAAAT
ATCAGAAGACATAAAAGAGAAACCAAACAACTCGGGGACAAACTAACGAG
ACTTCTCAAACCCAACTTTAATATCATGTTAGGAGATTCCAACTTAAAAC
CATATGGCAATAGTCTGGGTAGCTCATTTCATATGAAGACTAGTTTGAGT
AGCCTCAAAAATATAATGTGGGATTCAACATCCACTCTCACGTGTAACTC
TGATTTGCATGTGCAGCAATTGACAGGCATCAAAAGATAGAAGAGAGGGG
CCAAAAAACATCATAAGACAGAAGAGAGAAGCCAAACAACTCGGGGTTGG
GGACAAACAAACGAGCCTTCACCAACCTATTTCTGATACCATGTTAGGAG
GTTCCAACCTAAAGCATATGACATCAAGTGGGGTATCCTCTTTCATATAT
AAACTAGTTTGGGAAGCCTCACACATGCAACGAGGTATTTAACAAAGGGA
GAGCGGGGGAAACTTTATTGATCACCAAAAATATAAAACTATTAGGGGGC
ATAACCTAACTGATAGAAGAATACAAATGAGTGAGGAGGATATTAACCCA
ACCCTACAATATAAGAATAGAATCAAAGGGAACCAAGAAAAGAATGAAAC
TAACGAAAAGGAATTCAACCCTAAATTTCTCAAAAATCACTACGCTATAA
TGTAACCTTTATTAGCGGTGTCCAACGGCAGAATGCCTTTGAGCTTGTCA
GGAAACCTCTACCAATTAGTGAAAGTGAATCACCCTTTAAAAAACTCAAA
TTAGCGGCTTTGGTCATTTGTGATCATTTCTTGATGTTAGTAAATCTGCT
ATATATTTGACACGGCTTTGCCGTAATGCTGAAACTATAGCGAATGCATC
TATCGTCATTGATGCATAAATAAAGAGACCAGTGAGTAGTGATTGAATCC
CTTCTATGGTTCCACATGAGAAACTAGTATGAATATAACCTACATGTCCA
ATTGATCTATGAGCTAGAAGTCTTTTGACTTTCGCTTGGGCCATGTCGGC
TAGTGCTCCTAAGATCACAGAAGCCATGCTATCTTCCAAGAAACGGAGAA
GACCCTTTCTCTCTTGGACAGGTCCTTCCAAACCGGTAAGGTTTTAGAAA
CAAAATCCCTAGGAACCTTGAGCATTTTTGAGGGTTCAATACTTCAATGT
GACTAGCCAACACACAGGATGAGACTTTACCATTGTGGAGCGGCTTTTTG
CAAAAAGATGAGGGGGAAAGTCAGTAAGATGTCGGAGAAGCGAAATCACA
AACGCAGATTGGTCGCGGCTGAATATGAACTGAGACTATCTATTATTTCC
CGTTGCTAAATGTGTGTGGCTTTCTTTACATTTGCTCAGTTTTCAACTAG
TTTCTGATGGGGGTTTAGAAAATAAAAATGCCTTGCCTTCTATATGCTAC
TTGCTGATCCAGTTAAAAATCAGTCATACAAATGCTAGCGCATACCATTT
TGAAGTGTATTTTTTTTTGCTGGATTTAACATATTAACTGTCTTCTTAAT
CTTTTATTTCTTCACACCTACCATCACTTCTTCCTGACATACCGATTCTC
ATTTATCAGGTCTGTGATTTTGGTCTTTCCCGTCTAAAAGCAAATACATT
TCTTTCGTCTAAGTCTGCTGCAGGAACTGTAAGTGTTTTTTTTTTTCCGG
GTTTTAATTGTGTCAGCATTTATTCTTGTTATCTGTAACTCTTTTTCAGT
GGTTTCCATCACAAATCAATTTTGCATATCTGCTTTCTATTAATAGCCCG
AGTGGATGGCACCAGAAGTTCTCCGTGATGAACCATCAAATGAGAAGTCA
GATGTGTACAGTTTTGGAGTGATCTTATGGGAGCTTGCAACTTTGCAACA
GCCTTGGAGTAATTTAAATCCAGCTCAGGTTTTTTACAGTTTATGTTCTA
TTGCTACTTTATGCATGGTGCAAGCAGTGGTAAATTATTCCTCACCCTAG
TCCATTAGAGCATCACAAATATGTTGAATTTGGCACATGAATAGTAGCAT
AGCACATGTGGTTTAGGGTGGGTAGTCCAACAGTTGTTGCAAAAACAATG
TGCTATATTTTAATGTATTTTGTATTAAAATAAAAAATAGATATTGATTT

GAATGAAATTTTTATTCATCAAATTTCAGTATAATTTTTTTCATGTACTT
TGAATTTGAATTTTGAGTTAGTATTATGTATCTGCATGTATTAAATTATT
GATTAGATTGTGAATCCAGATGTATCAAATTAAACTAAATAAATCAGGCC
AAGTTGAATCTAAATTAAACTAAAAAAATTAGCTCAAATTAAATTACAAC
CTAACTTAGTTAATAAATTAAAATTACTCTGTGTAAGGCAAGTATTTCCT
TCCCAATTGCAATCTTCCACCCGGAGTGAGAAGAGGCCTCTGAAACAGTG
TGTTTGGAATAGAAGCAAAAGATATAGAAGCAACATAAGAGGAATAGAAA
GGGGATGAATGCGCATATGAGACAAAATTAGAGATAAGATGTGCAATAAA
AAAGCGCTTAACCTGCAGAACAACAATAGGCATGTCCAAATTGGGTGGTG
GTGGGACAAATGGCGAAGGATCTTAGGAAACAAGGACAACTGGTGTAGCA
GCTGAGGTAGATGGGGAAGATGTGGCCATTTGACAACGGGCGTATACCCG
TAGAGGAGGACAAAAAGCTTGGGGATTGGGTTCAAGTGTGGAGGAGGATG
AGACAAGGGAAGACAAACTAGGCAAGGGAAAATGATGAGATCTAAACCAA
CTAGAGATATATATTCAAAGAAATAAGGAATGGGCTCAAAGAAAGTAACA
TCACCACACAAAAATCACGACCTAAAAATGACTATAACAACGATAACCCC
TGTTTAAGTATGAGAATAGCCTAGGAAAAGACATTTTGAGGCTCAAGGGT
CTAACTTGTCTCGACTAGAATTAATCTGATGAATAAAACACATTCAAAAA
TGCGAGAAGGTAGGCCAAAGAAGGGAGCATGAGGGAAAAGACGGGACTGT
GGATCTTGCCATTCAGGGTGGGAGAGGGCATTCGGTTAATCAGGTAGCAA
GCATTAAGGACCGCATAACCCCAAAATTGCTTTGGAACACACATTTTTAA
ATAATAGGGCACGAGTGACCTCAAGATGGTGTCGACTTACGCCCAACGAC
TCCATTTTGTTATGGGGTATGAGTGCAAGATGACCGATATCACCACAATA
TTCGCGAGCATTATCATCAAAAGTCCACGAAACTCCGAACGATCTTTTAT
TAAATAAAGACAAGTAATTTGTGAAAAATCATTTGACAAAAGTCACAAAG
TACCGAAATTTCAAAGTTGACAAGACACAATTTGGACCCCAAACACCCAT
ATTAACTAACATAAATGGACTCAACTTTGTTTAATTGACTAGGAAGCGTA
TCAGACACGATGGTGCTTTCCCAATTGACACAACTCACAATATACGGAGG
ACAAATGACTAAGACTAGGAACTAAAAGTTTGAGCAGGACAAGGGATGGA
TGATTAAGGCGGCATTGAATTTACTGAGTAGATGCAATGACAATACAAGC
AATAGAGGCCACAAAAGTATCATCTCCACTGTCAAAGTTATATAGGTCAA
ATTCACTCCCGACGCCATTCATCTTTTGATTCCCCAAATCCTGAACAAGA
ATAGAATCGAAAAAAAAAAAAAAAGAAACAAAACAATTAAGTTGTTTTGTC
AACTTACTAACATACATGAGGTTAAACGAAAAAGATGGAATATATAAATG
ATAAAGACATGGAGGAAGACCGGTGCACAATGGCCACCACTAATAGGAAC
CATAGAACCATCAATAATAGTGACAGGATGTATCAAGTAAGATGAGAAGA
CTAAATGTGTATAAAAAGAAAAGAATTATCTTACATATGGTCAATGGCAG
CAGAATGTATAACCCATTTTGTGGGAGGGAAAGGGTGATTGAAAGGCAAG
CAGTAGAAGTACCTCGCTAGGGGTAAAGAAGCAATGAGAGAGGCTTGCTA
GGATGCCTTATGTTGAATGAACTGGATAGGTTTCTCATTAGAGATGTCCT
TGGCTGCTTGAGACTCAGAATTTAAAATGGGAACTGAATCTGATGTCATT
GCATTAGCAAAGTTAGGTGGGCAGCCAAGTAAGTCCCAACTAGTTTCTCT
GGTATGATTCTCTATACCACAGAAAGAGCATTGATGATGTTGTTCACGAC
CATGACCATTGCACCCACCTCAAAGGCCCCTCCACAGCCACCATGAGAGA
ACCACCACGACAACCACTTGACTGATCACCCTGATCTCGTGGAGGAATCT

GCGTCACAATGGTTGAATTTTGTGATGATGTAGATAAACTAGAGACGGAA
GCACTTTCTCTGGTGGTGCTATGCAAAACTCGTGAATAGATATCAGTAAT
AGAGGAAGAGTAGCATTAGTAAGCACTTGAGGTTGATTGGCTCAAATTAT
AAGTATACCGCTAAAAATTTCATGACTAACATTTGCTTATGTTGCGTCTT
TTCTTTCACATCAGTAGTTATTAGAAGAACTCTATTTAACTCCTCATGAA
GTATTGAACGTAGCAGAGTAATTTGTAATAAATTGCTTGGCTTGATTGAG
TTGAAAATACCCAACTAATAAATCATACATCAGGGTGAGATTACTGGAAT
ATAGCAAACAAAGATATTCTTGGATCGCTTTATATGTATCCAAATGAATA
CACATATCAACAATTTGATGATCCATCAAGTTCCACAACAATGATACTGT
TAAAGTTACGAGAATGAAAAAAAAAAAGAATGAGACTGCTTAACAATTGT
GTCAACCTCCCGAAAAAATACAGAAACCCTGAACAATACAAAAATGACAT
ACATACCCTTTAACACTCCCTTCAAGCTGGAGCATATATATTGTATGCTT
CTAGCTTGTTACACATGTACTTTATCCTTGAGTCTCCCAATGTTTTAGAC
TTTCAGTAAATAAATCGGCTAACTGATCGAATTAATTTACATGTATAGTA
TCAATCAACTTCTACAACAATTTTCCTCGGAATGAAGTGACTATCAACGT
CAATGTATTTTGTTCTCTCATGGAGGATTGGATTGGATGCATTGTGAAGT
GTTGGTTGATTATCACATTCGAGGTTCATTGGTGAAGAATGTTCAATCCA
AGTTCTTCCAACAAATATTTCAAAAACAGTTAGCAAACAAGTGGTATGGG
TCATCGCGTTGTATTCCTATTCTGCACTTAATCGGCCACTACCGTTTGCT
TTTTACTTTTTCAATATACAAGATTACCTCCAACAAACACACAATACCTT
ATAGTAGATTTTCTATTCGAGGGTGATCTGACCCACTCTGTATCCGTATA
TCCCTTTATTCAAGTGGGACCAAAATTCTAATATAAGAGCAAATGAGATA
CTCCGTCTCGTAACAGTCAAGTAGGTTAACTTCCCAACTAATTGTCTGTA
TTGCACTGAATCTGTCAGTGGTACTCTTTGAGCTTCCAACAGTTTTGTAT
TTGCATCTATAGTTGAGTCAATCGGTTTAGCTCCTAGTATTCATGTTTCG
CTCAACAGATCAAGGACATATTTTCTCTGAGGAAGGTAAATACCTTCTCG
ATATAGCGACTTCAATATCCAAGTAATACTTTATCTGACCCAAACCTATA
ATTTGAAACTTACCGTGCATAAACTTTTTGAGTTCTTGAATACCACTGTA
GTCGTCTCCAGTAATCACTTTGTCAATAACATATACAATCAACAATATTC
TCTCTGTTGTACTATGCGTTTAGAATACTGACTGATCGACTTCACAACGT
AGAAGACCAAACCCAGTGACCAAGTCACTGAATCTCTCGAACCATGCTCA
AGGAGATTGCTTGAGAGCATAAATGGATTTGTTTAAGCGACATACCAAAC
CAGATCCTCTCAACCACAAACCTAGGAGGTTGCTCTATGTATTCTTATTT
AGTTAAATCGCCATTTAAGAAGGCATTTTTGATGTCGAGCTGATGTAACG
GCAACAAAAGGTGGCAGTAAGAGCAGGGGTGTGCAATGGGTTGGGTCAAC
CCACCAAACCTGCCCAAATTGCAACATGTGCATTAAAACGCAAACCGCCC
ATAGTAATCTGTGCATTTTTGGCGGTCCACGGTTTAAATTACCAACCCGC
ATACCCAACCCACATAGTACTGGTTTGGTTCGTTGTTTTGGCCCAACCTT
TCCTAACTCGCCCGTCTCAACCCGCGATTTTCCTCACGTCCTATGTCGAT
TGCCTTACGCGTACTCAAATCCTTGATAATGACAGAATCAGGAATAAAAG
AAACAAATAAATTAAGATGTTTAGCAAGTTTCCTAAGTGACATCAAATTA
AAAGATAAAGAAGCAATATACAAAGCACAGGATAAAAAAGAGGAGAGAGA
GGTTCCATGGCGCCGATGCTACGAATAGGGACTGGATGTATGTGTGTGTA
AATAGGGAAGAGGTACCTAACACGTGGTCAGTGGAAATAGAGCTAATAAC

CCATGGCGAAGATGGGGAAGGATAAAAGGAAAGACAGTTGAGTTAGAGAA
GCAATGGAGGTAGATGCATGCTGATATGCCTTAAAATGAAGGAATTGAGC
ATACTCGTCATTGGAAATTCTCTTGGCTGTTGAAGAGTTAGACAATAGAA
AAGGATCGACCTAATCAGATGCCACATCATGAGATTACTTAGGATGTTGA
CCAACTAAATACCAACAAGTCTCTTGGTTATAATTTTCCTTGCAGTAGGA
GCATTGACGATGTTAGCTGCTACTACAATCCCCTCGGCGCCACCACTACA
ATCACCTTAGGAATGACCATGGCGACCACCACTAGACTGGTCAGTCTACT
GAGATATCTATGTAACCACAACTGAACGATCTGCCGAAGGGAGACTAGAG
AGCGAGTAGTGTCCCGATAGGTTGAGGCGTTGGATTTGAACGGAGACGAA
GGTAGTGACATCAGATTTGGACGATGACGGCGGTGAAGGCTTCGGATTTG
GACGATGTTGGCTTTGTTGATGTCTAATGTGGCCGGCAACGGTGTTGGAG
GCGTTAGGGCTGGATGGTGACAACGGCGGTGGCATTGGATCTGGAATCAG
AGATGGCGGCAATGATAGCAACGTCACGGTACAGATCTTATGAGATTAGG
GTCTCATTGGAACCTACACTTGATATCATGTTAAAGTTACGGGAATAGCA
AAGGAAGAACGAGACTGCTTAACAAATGTATCAGTCTCTTTCTCTATTTA
TGATAGAAATTCGGAAACAATATGAATCCCTAAACAATACAGGGATGACA
TAAATGCCCTTTAATAGATACAATATGGGTATCAACTTAAATCCATTCCT
CTTATCAATTTGTGTTGGGGTGGATTCGGTGAGGTGACGGTGTTCCCTGT
GTTCGGTAAGTGCAACTTGCATAGTTTTGGACCACTGTAAAATAATTGGT
ATCATTCAACTTTCGCATTGTGAATGATTGATGTGACATAGAGTGCCTGC
AAAGTGAATTATACTATACATAGGGGATTGGGAGGACTCCACACCAATCC
AACGGATTATAAGACAATGGACAAAGGAAATGAACAAACAAGAACGAATA
ATAGAGCTTAAATGGAAGATCATTTTTTTTTTTCTTTTTGAAACATACAG
ATGTTTGTAAGGGTTTCATAGGAGGTCACCGCTGGTGGCTGGAGCATGGC
TGTCGGAAGTGGAGTAGGACAGAGGAGGGTGGATGAAGGTCTGAACAACT
ACGGTGAGGCGAGATCCAGTCTAGACGAGTTCAGGCGAAGGTCTAAACGC
AGTGGTGAAGTGAGATTTGGTTTGGATGGGGCCGGACCAGAGTTTTAGGT
TGGCGCTGGCTAGGGTTTTGGGCTGCGAGGGTCTGATGAACACATCTTGG
CCGAACCTGTGCCACAGATGTCTGTGCGGGTTTGCCCGAGGTGGAAGGAT
GACCGAAGAGTTCCGTTTTAGGATCACCAGAGGTCGAATAGGTAAAGCGG
CGATGGTGGCGGCAAGTGAAGGACGACGATCTATGGCTTCATTTTTATTT
TTATTTTTTTGGTGCGGGAGGTGGGAAAAAAAGAGAAAAACATTTGTAGA
GCAACTAGGGGGAGTCATAACTTGCTCTTGATACGATGTTAATGTAAAAG
AGAGTACGAAAAGAATAAGACAACTGACCACTTTGTTAGCCTCCCGCTAT
TTATAATAAAGTAGAGAAACAATCCATAAACCGTAAATATAAATGAATTG
ACAGAATTACCCTATAACAGTATCACATGCGCATGTTTGAGATGGATTTT
AGTGGATGTTGGAAGACATGAAAGTTCTTTAGTTCATACATATAAGACCA
ACTACGAAGCAGTGATTTATTATTGCCATGGTTGGATAACTACCTTTGAT
GAGGTTTCCACACCTTTTCATAGTATAGCTATTCTTGATTAGCCTTTATG
GATTCCAATTTCTTCCGTTCAATTTAATATATCTTTTACTGTCCTGTGTT
TCATCATTGAATACCGAATATGCATAAGGTAGTGTTTTGAGCTCTTTTAT
ATGATTAATTTGTATTAATCTGTGCCAGGTTGTTGCTTCTGTTGGTTTCA
AGGGTAAGAGGCTTGAGATTCCACGGAATATAAATACTCAAGTAGCTGCC
ATAATTGAGGCCTGCTGGGCGAAGTGAGTCAATTCCTTGTTTAGTTTATT

AAATTCTAGCTCGGTTTGCAGTTACTTTTACTCTTACTTTGGTATTACT
GTAGTGAGCCATGGAAGCGCCCTTCTTTTTCAATATCATGGAATGCCTG
AGACCGTTGATTAGACCTCCCACGCCACAGCCGGGACATACAGACATGTC
ATTGCTCATGTGA
>EUC03132-RA [gene]
ATGCCGCATAGAACGACTTACTTTTCCCGAGGCAATTTCCGGACCGTAA
ATTCGATGCATCGTCAAAGTTTCAGTTAGATCACGAGAGCAAAAACGCAA
AAGACAGCGAAAATGACAGGAAAGCATCAAAGCAAAACACAGATGTCACG
GTTGGTGGTAATAGTGCTGCAAGTGCAAAAGATTCAACTGCATCGGATCG
TTTCACGGGTGATAGGATTCACGAGAAGCAATTGGCCGCTTTTGTCAACT
GGTTGGGGAAGAAAAAAGTCGGGGTGTCCGGTCACGTGAAGGTGAGGTTG
GACTCTACAGACGAAGATCGCGAGCACTTGCTTCCACCTGAGGCTGATGA
ACCGCCGGCGACAGAAGTAGTCAGAGATCGTAATTTCGAGCGCGAGATTT
CTTCACAGAAATCGCCGATTGGTAGTGGGAGTAGCTATGGCGGTCCGCCG
GCGGCGACAGAGGTGGTGGTCGGTGCCGGCAGGGATCGAGGGTTCGACCG
GCAGATGTCGTTGCAGAGGCTGTCGAGTGGGAGTAGTTATGCTGGAAGCT
TGTTTTCGGGGACGACTGTTGACGGGAACTGGTCTAGTGGTGTCAAGGAT
TCTCAGATGTCCACTACGAGGGAAGAAGAGGAAGAGAAGACTGATAGTTT
GGCGCAGAGGTCAAAAGAGAGTTACTATTTGCAGCTTACGCTTGCGAAGC
GGCTAACTCAGCAAGCAACGCTTGCTTACGAGCCTATACTTCTGCAGGAG
TGTAAGACGGATGCTCTTGGTGGTTATGATGCCGAAACCGTCTCGTATCG
TCTATGGGTAATGTCTCCTCTTTCTCTCGAACACACACATGTACTTGTCC
ATGTGAAGAGTCGTGCATTAAACACGAGCTCGAATAATTTACGCTGCCGT
TGTTTACATTTCTAACCAATAGATGTTGTACGTGAGAGAGGCCAACTAGT
TGGTCGGAATAATCATACAAAGGGTTAGTGTATCTACTAATTTGCGAATA
TGATTGATTTCCTTCATGACCTAATAGTTGGTGGCCTCGCCTATCCGTCT
TGAGCTCACCGTGGTATGTCTTTGGTCTTTCCGATTCTAAATTGTCACCG
CACCAGGATAACGGCGGTCTTTACGATTTAAAGGTGCTTATAAAGAATCC
GGCTACATTGGACACTATAATGGCTTTTCCACTTTCTTGCTTTTCCTCTT
TTATTTATTTATTTTTGGTTGTGGGTCAGGTTACTGAAATATACATCCAG
GGAGATGGTTCAGTTGAAAAAGAAATTTCACATACACCATTATAATCTTA
GTTGGCTGATGTGCGAAAATGAGTAATGTCTAGTCTAGTGCTGATGTAAC
ATAGTTGTTTCAGTTGAAGTTTTGAGAAAAGTTTACCACCATATTTCCTT
GTAGGCAAGTGTTGTCTAATATGCATGTCACTTGCTCTCAGGTTAGTGGG
TCTCTGTCCTATTCTGATAAGATATCAGATGGATTCTACAGCATCCTTGG
AATGAATCCCTATATGTGGGTGATGTGCAATGACTTGGAGGAAGGTACGC
GCCTTCCACCTCTAATGGCTCTTAAAGCTGTTGAACCCAGTGACACGTCG
ATGGAGGTAGTTCTTATTGATAGACATGGGGACTCAAGGCTAAGGGAGCT
GGAAGATAAAGCCCAAGAATTGTATTTTGCTGCTGAAAACACTTTAGTGT
TAGCTGAAAAACTTGGCAAACTTGTTGCTGTCTACATGGGGTAGGTTTTA
GCATTCTTATTATCCTTCATATTTTTGCCTTCATCTGCTTCTTTACGAAT
GTCAAGTTTTGGATTTTAAGGGGATCTTTTCCGGTGGAGCGAGGCGATCT
CCACATGCGCTGGAAGTTGGTTAGCAAGAGGCTAAAGGATCTTCAGAAGT
GCATTATTCTCCCAATCGGCAGCCTCTCCATGGGACTTTGCAGGCATCGT

GCCATTCTATTTAAGGTGATGATGTACGATGCAATTTGCTTATAAACTTA
TTTACCATGAAAAGAGAAATGAATGGAACTCTAATAATAAGTTTTTTTTT
TTTTTTAAATTACATTTGATTGCATGAACTGTGTGTGCACTGTTAAGTGA
GAATCAACTCAACGAGAATCATCAAGTAACACCTGCAATGATTTATAACA
GTTATTAAGACTTGAAGAGTGCTTCTTGTCCTAATCATTTACTGGGATAT
ACCGATTATGACTGATACTTTCTAAAAAATAATTCGATTTTTCTCGGGCC
ATTGTTTTTTAATAACGCAAGTCCATTTTTTTTTTTAATTTTTTTTTTGT
CTTCAAGTTAATTTTGTGTATTTGGCTTGTCATCAAACGATGTGTTGATT
GGCCACTGCTGAGTGCAGGCTGATTACTAAGGTAGATTATGAGATATGTT
GCATAAAATGGTTTTTTGATCCTTATACCTGTTACATGCTTGTTCCGATA
AATATTCAAAACATATAATTGACAAACTCAAAATATTTGATAGAAGAGGA
TTTAGTTATTACCATTTTTATAACATCATGATTTAGTGTGTGGGGTGGAT
CAATGAATGCCGACCTACTTTCCCTTGACCTCAGTGTGGCTTTGAATCTT
TATTTTTCATAATTGTGTATGAAAGGGATCATAATTTTTAACAGGTGGTT
TGATTTTGTATGTAGAAACTGGCGGACTACATAGGTCTGCCTTGTAGGAT
TGCTCGAGGTTGCAGATACTGTGTTGCCGACCATCGATCTTCATGTCTTG
TAAAAATAGAGGATGACAAAAAGTTCTCCAGGTATTATTCTGTCCATCCC
TATTTGAATGTATTATCTTAATTGTAATTGCCAAAAACAATATGTTCAGC
TTCCAGTTGAAGTAGTATAGTGCTTGCTGAACATAAAGTTCCATTCAATT
CCAAATTGTCTTCTGATCTTGGAGGACTGTCTGTGGGGGCAGGTGATGGG
ATAAAACAACTTACTCGAAAAATTAATTGAATAAGAACACGCAACGGAAA
TTAATTTTATCCCGTTCTTGTGTGAACCTAGTAGGAATTAAATTAAAGGG
ATATTTAAATAACACAACACAAACACAATCACAGGAGACAACTAATTTTT
AATGTGGAAAACCTCTCCAAGGCGAGGAGTAAAAACCACGGGACCGAAGT
TCACCCAAAATCTCTACTATCATAAAATAATGGGAATACAATCCTCTCTA
GTTGCAACTAGAGGTACACAACCACAATTACTTTAAGATAAAAAACAATA
TTGGCAATGGAACAAGATACACGAGCAAAATGTAGAGATCTCACCAAAAC
GCCGGTTTAAACACAACCCGAAACAGTCCCTCCGAAACAGTATCTGCCGA
CCCCGGAATTCGATCTCCACCGTTCAGATTATAGAGCACTGAGTCGCGAA
TCTGATATCCAAATTTAAGCCCAATCGGACGGTGGATCGTCGTCCAATCG
ACGTTTGATCTGAGCAGTGCACAAGGAGTTTTTGGTGGCTTTCTCTTCTC
TTTCTCCCTCACTGTTGCGGCTGTTTTTTTTTTGTTCCCACTTTTCACTG
CTGAAAATTCTGAACACTAGGGATTAAAAAATTAAAAACTCAAAGTGTGA
AATTACAACTTTGCCCTTTTGCATTTCCATGAGCCTAAACAAGTGTTGAG
CCACTCTACATGTTGGAAATTAAAACCAAGCCCAAGAGCGGGAAATGCAG
CTTTATTCTTAATTCTGATTATTAGGAGCCAATAGAGAGAAATGATTTGT
TATATCATTTGGGAAGATTCTATATATCTTTTCGAATGGTGCTACTTGGT
GAAAAGTTTGTCTGTGTAGTGTACATTATATTTGACTTTTGTGTTGATAT
TTTAGAGTAGAATGAAAATTTGAATTGTGTTCTACCAAGATTTTCTTCTC
GTGTCTGATCACTGCCACATATTCATTTTCCATATATTCTTCGGTTTGAA
AATGCACAATTTCTGTTTGTCTCTCAATATAATGTCATTTGTTTATGCAT
GTCATACATTTCTTAATAGGGAATTTGTAGTTGACCTCGTTGGGGAACCA
GGAAATGTTCATGGCCCAGACTCCTCTATCAATGGTGGTCCACTTTCTTC
GGTGCCTTCACCGTTTCAGATATCTCATCTAAAAGAAGTTCAGCAACCTT

ACATGGATAGTAGTGAGATGTCCTGTGCAATCATAAATTCAAAGCACAAA
TCTGCTCTTTCTGAAAGCTCTCCATATTCAGGTACCATATATTTTGCATG
GTTAAAAGCTTGTGTTAAAGTTTTGTATGCAGCTTGGATCAGTGGTATGT
AGAGGATATTAACCGTTCCTTGAGATGTTTAGGTTGAGATTGCTGAATTG
TCCTTGTTCTCGCCATTTTAATTGGCTGACTGTTTGTTCTATTTCTCCTT
GACTTCTATTTTTCTAATAGATCTTCTACTTGGTTCATCGGCATTTGCCA
ATTCTTTATGCACTAAAGATTTTTACCACATATTTTGCTGAAGAATGACT
ACTAGTTTCAAAAACTCTATGTTCTTCTGCTTCAACTTCTCACAAGGGTG
TGTAAAATAAATAATGGAAAAGTTACCAAGAAGTCCTCGTATGAAAACGA
AACTACCAAATACGTATTTTTTAAATACATGCTATAAAATAGTCATTTTG
TCTTTTTGTGACGATTTTATCCCTCATGTTATATTCAATACAAGATCGTA
TTGAATATAACACGAGATTATATTATATTCCACTTTCACTGCCATTCAAA
TTCTACCAAGCCGTCGTACTCATTTCATCTCACCAAAACAACCATACAAA
TATACCCAATATAAGTTCCACTTCACCAGGGACATAGACTAAGTTCATTC
TCTCTCTCACATTTGTTTAGGGCGACGAGTTCGGGGCCGGCGAGAATCGT
GTTATATTTCAATTTTTTCATTCAAATTGATAATTATTTTCTCTACATTT
ATATGTTTGATTTTTTCTAATTCAATTTCTTCTAATCCCTAAATCATACG
ATATAACGACATCTTTAGTAGATCATAACACCACATGGATAAAAAAAAGA
AAAAGAAAAAAAAAGGCTCACTAAAAAAAATGACGATGATGAAAAAGATA
CCGGACCCAAGGAACACCTTTTGGAAACCTGGATTTGGATAAAAAATGAA
TTAAATGAAGAAATTTAAGTCCACGAATGGCAGCATGAGAGCAAAGTTAA
GTAGGATAATAACAGAAATCAACAAGAGTAGTTTAGTAATATTGAAAGAC
TGTTAATCTAATCCTAACGATAGCGAAAACTAACTAATACTAACTAAAGG
GCATCCCGGTGCTACAAAGTTTCTGTTATGCACTGGGTCTGGGGAAAGGT
CGGACCACTTAGATCTATTGTACACAACCTTTCTGCAAGAGGCTGTTTCC
ACGACTCGGACCTCAAACCTCCGGGTCACATGAACAGCTAATAGTGTGAA
ATAAACTATGAACTGCTAATAGTGTGAAATAAAATTGGGGGAAATTTACA
CGGAATCGAAATCCTCAGAAGCTGGAACTTGTTGACGAGAGGAAGCGCTT
CAGTTTGCATAGCTGCCGCCAATCAATTTCAACAAACAACGATTTCAATC
AACTCAATATCGACTAATCAACTAACAATTTACAAATACATATTTACCTA
ACTATACATGTGGAGAGAGATAGAGTAGTACTGATGATGACGACGATGGT
GGAGATGGGGCGTTTTTCGACATTGGAAGCCATCGATTCCTCTGAGACTA
CATATTTATCGTCGTGAGGAGGAGGATGAGCCATAGCCAACCGTCAAGAG
TATATGGCAGATACCTTCAGAGTATATGGAGATGGGGCGTTTTTCGACAT
CGGAAGCCATCGATTCCTCTGAGACTAATGATTTATCGTCGCGAGGAGGA
GGATAAGCCATAGCCAACCTTCAGAGTATATGGCAGATGTGAACAGAGAT
GTCGAGGATGGTAGTGCGGGACAAGCATGGCAGCGGCGAGGCACTGGATG
AGCACGATGGCTGATGGAAGCAGAGACACTGTCTGATAAAGGGATTGCAA
CGCAGAGAATGTGTGGATGAAGAGGTAGGGAAATAAGGGTAAAATTGTTA
ATTTAAAAAATTCAGGATTTTTTGGTACAAAGTAACGCTTTTTGTAGGAC
TATTGGGTGCAAAAAAAAAGTTGTGTAGATTTAGACTTTTAAATAGATTT
TTTAGTATTTTTCTCGTAATAATTCCATATGCTTGAAAGGGACCTCTTTG
ACCTGTTTGACTCTTACTGTGCAAGTTGTCTTGCTTCTGTACTGAAGACT
TGAGTTATGTTTAGTTGGTGATTGAGTGAATTTAATCTCTTTCAATTTGT

TGCATATTTTTGACGTTGTCTTCCATATGTTGTTTTTGTCCTTATTTCAT
TACTTCCTCTAAAGATTTGCTAGCATCACATCTTGGGTCAATGCTAAATT
TCTCATATCAAGGTATATGAAACGGAAATATTTCATTTCTCCAACCAAAC
ACGACCTTATGGTTCCATTACATTAATAACTGGTAAATATTTTTATTGAA
TTAGTTCTTTTGAGTTTTGACCATTGCATTCAAGAACTTTGCTGATCAAT
CTCTGCTACGTTACTCAGAAACAAGCAAACCAATTCAGAAAAATAACTCA
GCCTTGAAATGGCATTCTTTTATCAGTCAAACTTACTGTTTTGTTTCAGG
TGATTGGGAAGAAGGACGTCAGCGAATGCAGGATATAGGTTGTGTGCATG
AAAATCGAGACGAGGCGCTGTATGGTCTTGTTGGTCAAGCTCATGTACAA
AGTGAATCACCTGAAGGTGTGGCTGTTGTTTCAGCAGCTATATCACAAGG
AGAATACTCTGCTGCGAAAGGAGATAAAATTGTTATTCGACAGGCTTACA
GAGAAGAGATAGTTGTATCCGAAAGTCCTGTTACAAACGCTGCCCTCAAT
CAATCTCCTGCTGTAATAATATCTGATAAATCAAACCCTATGGAAATCAA
GAGTGGAGTTGAGAATCAAGGGAATTTTAACACTGTAATAATCCCAAGAT
ACTTAAATCTTGAGCCATCTCTTGCAATGGATTGGTTGGAGATCTCCTGG
GAGGAATTGCATATCAAGGAGCGAATAGGAGCAGGTACATCTTTTAACAG
TTGCAGCCTTATTAAATATTCAATGTTATATGTCTTCCTGTTAAAAATAC
TCTGGTAATCTCCAATTACAGGTTCTTTTGGGACTGTTCATCGGGCTGAA
TGGCATGGATCGGTTAGCAGTCCTAACCTTCTTTCTTTATTACTGAATTG
TTTAAGTGCAAATGTGAAATGATATTTTCAATTTTTCTTTGAATTGAGTT
ATGTCAGTGATATACTTAACGTGTTCGATTGGCGGCCTCATTGAGGCGAT
GCAAGAGAACCCTGGCTTGCACATGGTGGGATGACATAGCTCTAAGAATA
TCTTAGGAGCATCCGGTGCAAAACTCTATAACTATAGGAGTAGCCCCTTC
TATGTGTATAAAATTGTGGAAAGCCTCATTAAGGCGATGTGAGATAACTT
TTTTAATCTTGACATATCTCAAGTGAATTGCAAAAAAAAAATCGGGATAAT
TGCTTGAAAATCCTAATTAGAAGCTCTAATCGATTTTAAATTTTAAAGCC
TATTTTGAATTTTGCTTGAGACATAATCCCGTCAATGTTTTTTGTTGTAT
CCTACATTGATGCTGGGCATCCCGATATCCGTTCGCAGTGGTTTGTCAAA
GTCCACCTTACGCGTGAGGGGATGTATTGAATCCCATATCGCTTGTGTTA
CCTGATCTTGAGCTATATATAAGTTTAGGGGTAATCTTTCTCTTTCAAGG
CGTCTTTTGAGACTGAGTTAGGCTCATTGGGTTACAACAAGGTATCAGAA
GTGTTTGTGTGTGTGTTTTAGTCTTGTGCTTTGTGTTGGTTGACCTTTCT
ACCCTTTCATATTACATTTTGTTGCATTCAACTACACTTGCAACTAAAGA
ACTTAAAAAAAGAAAAACATAATGCAAATAAGTTACATTTAGTCCATAAA
TTAACAAAAATAATTTGTAGTAAAAAAGTGTAACTAAGGATGCGAAAAAA
TAGTATTAATGTGTGTGCATAATGCTTTGGTCGTGTTCAATATTAGTTAT
ATACTTATATTCAATTTTAAACATACAAAATAACACGTCAGGTCCATACA
ACTAAAAACATTAAAAAACGTCACAGTGCCCTCGTTGTGTTCGATATTAG
TTACACTTTTTCCATAAGAGTGAAAAAATTAATGTAAAACTAGAAGTGTA
ACTAAAAACACGAAGAAAAAAAATAGCATCAATTTGTGTCTATAATGCTT
TGATTGTGTTAGTTTTTATTCATTATGCGCATATTATTGGAGCTCTGAAC
AAGACGCCGAAAAAAATATGGATTAATCAATTATACATCCCCATTTCATA
TTTGAGTTGTAATTAGTGTTTTATTGGCTCTAATAGCTCTTTATGAGCCT
TATTCTGACCATAAAGTTAAAAATGAGTTACAATTAGTTTGCATTGGTAA

TATCATTTTTATTGATGAGTTTATCCCACATCAGAGCTGGTGGCCAGGTT

ATAAGTGTGAGACACAACCTCACCTCATGAGTTAGCTTTTGAGGATGAGA

GAGGCTCAAGTCTACCACAACAATTATAAGATTGTGAGACTGCAAATTAA

TTTTAAAGTTAGTAGGACTAAAACTTACCCAAATTATAAAAGTCTACGAA

TTTCCCAAAAATTCTGTCTTGCAGTGTTACCTTCTTTGTCTGACCAGATT

ATATTGCAATCATTGCAGGATGTTGCAGTTAAGGTTTTAACAGTCCAAGA

TTTTCTCGATGATCAGTTGAAAGAGTTCCTTAGAGAGGTGTGTAAATATA

GGTGGCGTTTGGTAATCATTTCTGTTCCCATCCCAATAGTAGGATGGGAA

TGGGATGAGAATTCTGTTTCCTTCTTGGGAGTAGAATCGCGTTTGGTAAT

GATATTTTTTTGTTTTGTTCTANAAAAAAAAAAAAAAAAAAAAAAAAAATT

ATTTTGAGAACATTTTTTTTTTGTTTTGGCCGGCTGGCGGCAGATGGCGG

ATGAATAGTGGCAGTGATTTTTGGGAATGAGATGGGAATAATGAGAAACA

ATTTTTTTTTTTTGTTTCTCATTTCTTAAAAAAAAAAATGGGAACGGAATG

GAAACAGGAACAAAAATTACCAAATGCCATTCCCAAACAGAAATTCCAAA

AATCTAGGAATGGGATGGGAATTTTGGAGATGAAAATGGTTACCAAACGG

GGCCTACTTTTCGTTGTTTTTGTCATGGTTACGGCCTATTATTTTGGTCC

ATAATACATATATATATTTTTTAATTTCTTTTGAAAGGTTGCGATAATGA

AACGTGTCCGGCATCCCAATGTGGTTCTCTTCATGGGTGCTGTTACAAAA

CGTCCGCATCTTTCAATTGTGACTGAATATTTACCAAGGTAATTTATATT

TCCGGTAATGGTATGTACCTATATATATAAAATATTATTTAATGATGCTG

ATGTTGACAAATTTTATTTTCCTAAAACAGGGGTAGCCTATTCCGCCTCA

TACACAGACCAGCTTCTGGCGAAATTTTGGACCAGAGGAGACGGATACGC

ATGGCTCTGGATGTGGTCTGTGCTGATCTTTGTTTCGTGCGTTAATTTCA

CTATTTTTTTTTTCTCTTTAGTGGGATTCCTGAAACAAGACTGTGTTTGT

AGGCAAAGGGCCTCAATTATCTTCATTGTCTTAGCCCCCCCATAGTTCAC

TGGGATCTTAAATCTCCCAATCTGTTGGTTGATAGAAATTGGACTGTGAA

GGTACAACAAACTTGTAATTGCCAGTGATTTTTTTTTAATACTTCAAACC

CATCCTCAAAATTATAACTCATCTTACATATGCCCAGGTATGTGATTTTG

GCTTGTCGCGATTTAAAGCAAACACGTTCATCTCATCAAAATCAGTTGCT

GGAACCGTAAGTCCTCTCTTGCTGATGTTGGATGTGTTAGAGAACAAATT

TTATTTGCTATTTTTTGTATCGGATGTTAATTATTCTATTTATTTATTTT

TACAAAATTTAGCCTGAGTGGATGGCGCCTGAATTCCTTCGTGGAGAGCC

CTCGAATGAGAAGTCTGATGTGTTCAGTTTTGGAGTGATATTGTGGGAGC

TTGTCACCATGCAACAGCCTTGGAGTGGACTTAGCCCTGCCCAGGTAACT

TTCTAATACTAATACTCGACCTTCTTCAAGTCTTAAATTACTTTTACTCA

AAGAGGTGTTATTGTTAGTCTAGTTAGCAAGATGGGGTGCTCTTTTTTCT

TGTCAACTAGAGTTCGACTCTATTGGAGAGCACTTTAGAAATTAAATCTT

GAATTTAATCTTGAATACACTTGGCCGATATGAGAGGGTGTGGCCCGGAT

ACACCTTACCATTAAAAACTCATAAATTACTTTTCAAAATTTGTCAAATT

CTCATTGCAATTAATTTTTCAACCATCTCCAAAAAATTTACATTTGTATG

GCTGCTTTTTAATTTTTGGGGGGTGAATGCACTGATAATTATGCTTATTA

TTTAACCTTTCCTCTTGCTTAATAGTATCGGGCCTAGCCCAAAGTAACCG

CTTTGGGTATCAAATTTTAAGGGACACCATCGAAAAAAAAAAAATGGGTCC

GATTATATTTTCAAATTTTTTAAAACAAAATCGGGTGTGTTTTGAAAATT

AATTTGGCCCTCTTTCGACTTGGAGAAAGAGGCCCATGTTGAAATGAGTT
TGATTGTGGTCTAAGTTCTTAAATTTTGTATTATTTTATTATTCAATATT
TATATTGTATTGCAATGTAAAATTGTAAACTTAATAGTTAAATTAATATA
AAATTATTATATATTGCTATTATTTAAGAAAAGTCAAAGTATTGTGTAGA
GGGCCACCATTTTAGTAGTTTGATTTGAGCACCTAAATACCTAGGGCCGG
CCCTCGTGCTTAGTCTATGATTATAAGTAATTGGTGTCTTAAATTTCAGG
TAGTGGGAGCTGTAGCTTTCCAAAATCGGAGGCTTTCTATCCCACCCGGC
ACTTCTCCGGTACTGGCCTCACTCATGGAATCTTGCTGGGCAGAGTAAGA
ATTTTAACATTTTTTTTAATTATGTATTGGCCTTAGTCCTGAAGTATCCA
AATTGTATTAAGTTGTCATTTTTATTCCGGAAATCAGTTACGTGTCAAAT
ATAAAATCATCCATGTGAGAGAATTCCGTCATAGAAAATGACGGAAGGAC
TGTGACTTGACACAATTTGGTTGGTAAATGTCTTCAGATTGAAAGATCCG
GGGCTAAAGTGGGATTTCTCTAAAACTTCAGGGGTGCAAAGTACAAAAAG
CTCAAATATTTATATATCAAAGTTGAAGTGGAAATAGCATATTCTAAATT
TTTTTTTTTTTGGTACTAATGTTGGGAAAGGGGGTTATGAACTGTAGACC
TCCCATAAAGGTGGCTAGCTAGGCTACGAGTGTTTGAGTTTTCTTCAATG
TATTATGATTTAATCTTCTTAATGCTGTGTTGACAGTGATCCCGCCCAGC
GTCCCACGTTTGCTAGCATCGTGGATGCACTGAAGAAGCTGTTGAAGTCG
CCAACGCAGCTAATTCAGATGGGAGGCCCATAG
>EUC00315-RA [gene]
ATGGAGGACACACGAGATGAGTCAGGGACATCAGAGCAGAGGCCTTCTTG
TGCTACCTGGTGGTCTTCAGATTTCATGGAGAAATTTGCATCTGTTTCTG
TGGACTCTAAAGAAGAAGCTTTGAACCGTAAAGTGTCAAACAGTAACAAT
ATGTATGATAGGTTTTCATCTGAGACAGCATCACAAATCCTCTGGAGAAC
TGGAATGCTTGTTGAACCAATTCCAAATGGTTTCTACTCTATAATTCCCG
TGAGCACCAACAATTGCTTATTAATACTATTAATATATTGCTGTTATTGC
CATTGATGCTTCTGCTTTAGTACTAATCTTTCTGAAGTCTGAGCAATTTG
AGATTTATCAGTGTTTTTTGCCACTAATCAGTCTTTGTTTTTTGTTCTGT
TATTTGTTTATTTTCTCATAGGATACTTTGATTTGTTAATTTCGAATGTA
GGACCATTTGAATATCCGCGAAATTTAAATGTCAACCATCTTGATCAAAG
TTCTCAAGTTTCTTCTGTAAATGTGATAATTTTGATTTATGGCAGTATCA
TGTTTTCAATACCAGGTTATAACTGAACCAATTTCCAAGATCTGTTTCTT
CTTTACTTTCATTTTAGCTTCTTTTGGATTGCATCAAATCCAGAGCCTTA
CACTCATACAGAGAACATAACTCATTTCTCAGCCTATAGACCACTCCCTC
TCTCCGTCGTATTTGCATCCTTACACAGCATGCCATGTTTTATGACCTCG
CTTGAGGTGGAGTTCGGATCAATCATCTTGTTATTTGAATGAACAATATT
GAATTGTGTGGAGCTCACCACATATGATTTTACGAATTATTTATCAAGAA
ATATCAATATTAATATTTCCTGTATTAAGTATTTGTTGGTTTTTTACTGA
ACTCAAGTTGCATGTCTATCATTCTGTTTGGCCTTTTTCTCTTGTTATGT
TTACTTTCTCTTTCTATTACCTTTTAAATGTTCTATCTTGCCTGCCGTTA
CTATATGTTCTGCTTCTACCAGTTATCTGCTTCATTTTTTTATTTTTCTG
GGGCTTCAATGCCCTCCAAGTCTTCCAATATATTGAAAATACGTACTGTT
TTTATTCATAATTCATGCCAACTAGGATTTGAGAGATTGAGAGGTTGGAA
GTGGTGTCGGGGAGGAGGTAAACACCCTGCCCCTGTAGCATGGGGTGGGT

TAAGTGATTACCTAATTTTGGGAGAATATGGGGGAATTTCAAATTGGACT
ACTAGGGAAGATTTTTGGTCACACTTGCCCTCCTCTGATTCTGGACCAGC
AACAGCCTATCGTAGCTCATTGAAGGCTAGAAGTGGTGGCGGTGATTCAG
GTATAATGGTCAAATCAGATGGAATAGGGAAGGCTATCAGAAGTGAATTG
TAGTTATTTTAAAATTAGTCGAGGATACTTACAGTTTTACAAATTTCAGG
CATTGGCCAGACTCACAAAGGATGGCGTAGAAGTTAGAGGTGTTGTGATG
GTTAAGAAGGTAAAAGGTGTTGGTTCTTGAGTTTGTCTAGTTTGCATTCT
AGTCATATCCTCTTAAGTTAGTGAGGCCTCCTCTTCTATGATGGAGGAGG
GCAAAACTAGCCTGAGACGGAGGGAGAGCCCATTACCTCAGATTTTGATG
ATCATGAATGTTGGACTCCCCAAAAACCTACAGTGGATTTTACCCATTAG
TTGAAACGGTGCAAAGGGTAGTGGTTGTATTTCATCACAGAAGAAGGCCA
GGAGATGATAATGACAACAACTGTTTCCCAAATGTGCTTCTTGGTTTAGT
TTTGGTTGTTCTGTTGTTTGGCAGGCTTGCTTTTGCTGGGTTGTTCTTGT
TGTTAGCTTAGGAATCAATTTACTTTGCGTTTATTTGTTGTTTAGATCCT
TAGGGTATACTCCTTGATTGTTTCACATTACAATATACAATGCTTTTTGT
TAGCCTGTCCAAAAGATCTAAGTTTGGATCTTTAATATTTTAAGACGGTG
TAGATCTAGGGCTTTGAGAGCTATAGGAAGCTGATAATTATGCTTAGGGG
GATGAACAGTGTGACACACACACACACGTACTTGTTTATATAAAGTTTCT
GTGACAGCCTGTAACAGACCATAACTGTCGCTACAACAATACAATAATCG
ACCATAATGATTGTGACTTTTGGTAGCGCTGCAGTAGGGGGCCGTGATGG
TTGCAGACGCAACTAAATTTCTTATATAATGGCTGTTATGTGCCATTAGT
GGTATATGTTTAAACCTTGATAGCTGATTTGAAGCATCTGACTCCATTGC
CAACAATAAAGGTCTTAATTCTTTCATTGTGGTTGTCTGCTTGCCAATAA
ATCTGGTGGAAGCTGAGAGAGGAATAGCTATTTTTTATATGTAGGGTAGT
AGCTTTATTTTTCCCTTTTTTTCATGAACATCCTTTTCTCTGCACACATT
GTTTTTGGTTTAAGGGCTATTCCTTCTAGGAGCTTTTCACTCCTCGAATT
CTGGAGGATTTGGAGGCTTCTTAGCATTTACGACTTTGTAGGGTTTGTCG
CCCCTTTTTTCATTTGATGATGTATTTAATTAGCGAACATTAAATCTGGT
GGAAATTTTGGACTACATGCTTACATCATTGTAAAAATTCATTATGCCTC
TTGAAGCAATAATGTTCGTAATTTGATTGATATACTGTTTAGTTGTGTTT
TTCTTTTCCAATTTATTTCCTTAGAATATTATTTGTTGTTACTCAAATAT
GGTTTGTGTTGGTTTTTTTATTTTTTGTTGTTGCAATTATTTCAACTACT
ATCATTGGTTTGCAATTTGACCTCTGGTTTTAAGTCAAGGATATGCAATT
TGATGCAGGAGAAAACGCTCAAGGAACTATTTGATGATATTCCTAGTTTA
GAGGAGCTTCATGCTCTTGAGCTTGAGGGTCTAAGAGCTGATATCATACT
TGTAGATGCAGAGAAAGATAAGAAGCTTTCTATGCTAAAGCAATTGATAG
TTGCTCTGGTGAAAGGTTTAAACTCAAATCCTGCAGCAATGATAAAAAAG
ATTGCTGGACTGGTGAGACTTTACCTATAGTCCGCCTTTTGAATCTGGAA
GAACTTCTAATTTTAATGTGGACTCATTTTTTTTCTTTCTTGTAATTTGT
TTTCTTTTGTTCTTTATTATTATTATTATTATTATTTTTGTTTAATGATC
TTTCTCTTATAATTTCTTACCTAAAAGGCTAAAATGGAATGAGAACTTAC
TTTCTGCAAACCTTGTTGCTGGTAATGAAAGCAGCTTCTAACCATAAAAT
GCATAGTAAGAAATATTTTGTGTGATTCTATTCATCCATTTTGAACTTTT
CTATATTGTACTCTGGAGTTTATGCATTTCGTTTTGCCAACTGTTTGACT

GGGATAAATTTTTCTTAGTTTTCCTTGACCTTAAAAATGTGTTTTTCCGT
CGGAAAATCTATTTCTATTGGTTTTTTCTTTTTGTGTGATAAAGACAAAC
TAATAAGTAAAGAAACGCGTGACTAATATGAATCAAGTTGACTGAAAATT
GCACATTTCATTTAGAGATAAACTATAATATAAAGCTAATTATCTTTCTA
TTTGCCTAATTCTGAAAAATCAAATACTTTTTTGACAACATACTCAAAGC
CACAATACCAAGTTTTACCTCTTTTTATTTTATTATATTCTCATTTATGG
GGTGAATAATCTCCTTTTCCCTCATTCACAATTATTATATTTTTCTTGTG
TTTTCATGTGAGTGGAGTTTGTAAGGTTGCGACGATTTTTTTGATCAAAA
CATGTTTCTACTATCTAGGAATATTTTCAGTGCCCGTGAACCAAAAATTT
CATTGCTTTTATCGTGGAACATCTTGACTTTCAATGGTCTTTTATGAACT
TAAATGAACTTTGTAATATTAGACCTAGTTTGCAAATGTCTAAAACATAA
AATTTACGGTTGGGCCCTAGCTTGTGATCTTACTAGATGTCTGTGTGCCG
TTTACTATGTGAAGTAGGAATTTTTACTTTTGAGTGCGTTATTCTATATT
CTAACTCAGTTTTATGGGAAGAATCTTTTCTTATAAAATATTGAATCTTT
TGCTTCCACACGCCTCTTGGGGGCCGCTCCGCTGTAAATGAGTTTTGATT
TCCTTTGTATCTCTTAATGTATGTCAAAGAATGTTGCCAAAGATATAAAT
TATTGCATCTTGGTCAGTTGATCTACGAATTGCTTCTGTCTGTGTTGTTC
CTTTTCTGATCCTATTGTTCACTCAATAGGTTTCCGATGTTTCTAAACGG
CCAAATGTGGAACTAAGTCCAGCAAGGGCTGTTCTTGAGGAAGCCTCTTA
TGCTTTGGAGAATCGAGGTGTCCAGATGTTGGGGCAAATAAAACATGGAT
CATGCCGGCCTCGGGCAATTTTGTTCAAAGTTCTTGCAGATACTGTAGGT
CTTGAAAGTAGGCTAATGGTGGTAGGTTTTACTTGCTTGCAGTGGCATAC
TAGTACTTTTCATTTTTAGTACCTTAATGTTTCATTCGGGTCCTTTTAGT
TTAATGAAGAGGTTTCTGTTTGTTGATTGCCAAAAATAAAGGTCAATTTG
CGAGCTATGCCATCATAAAGCCTTTAATTCTACATGTTTGTTTGGTTTTC
TTGTCAGGGTTTGCCTAATGATAGAGATCCTGAATGTCTGGAATCATATA
AGCATATGTCTGTAGTAGTTGTGCTGAATTCTGTGGAGCTACTTGTTGAT
CTTATGCGGTCTCCTGGCCAACTGATACCTCTATCTGCCAAGGCAATTTT
CATGACGCATATATCTGCAGCTGGGGAAAGTGATTCTGCAGAAAATGACT
CCTGCGATTCACCATTAGAACCAAACAGTCCTCTATATGGGTTTTCGGAG
AGAGTGGATCCAGAGAGGTTATATAATAATACTTTGGTTGTTTCAGTCAT
ATATTTCGAACTTGGTTTGTAGTATTACTTTGCAACTAAGATTTGATTTT
TTTTTCCTTATATCTGAAATGATACATCCTGTGTTATTCATTCACTATAA
AGTTGATAAGGTAGGTATAACTGCTTTTTGTGTGCTGCAGTGCTGAGAAA
GATGACAACCTTCAGTACGAGCAGAGATTAGAAGCATCTTCAAATGTTCC
TGGGCATTCTTTAAGGAATAGGATGTTACGATCTTCCACCTCCATTGACA
GAAGAATGAGGTGATGATAGATCCTTAATTTTCAGGTCCAATGATCTTCA
ATATTAATTCATCCTTTGAAGTTATATATTTTAAGTTTTATTTTTGGGTC
CTTTCATGCTTCAGTTTGTCACATAGCGAACCCAATATTGCAACTACTTT
TTGGAGGAGGAGCCGGCGAAAGGCTATTGCAGAACAACGGACTGCAAGTT
CAAGGTTCTTAAACTTCATGCTTTATTAATTTGATATCGATGGACTTTAG
ATTGTTTTGACTATTCTTAAATAATTCTACCGTTAGTTTACACTTGGTTA
TGTTCTGTAACTTTTGACAATATTTTGAATACTTAAGGGGCAGCTTTTAA
GGATTTGAAATTCCACTTGATTTCGGGTTGATGAATATGTGTCCTAGCTA

TATCTTTGCATGATCATACTTAAAAAAATGATATATAATGTTTCCATATA
ATTTGGTGACCCTTCCTAATTAGCAACCCTTAATTATTGAGGGTGAGAAG
TTAAGGTTCCATTTGGTTCAAAACTCGAAAATATTTATATAAAATTTTTA
AAACAATTTTTTGAAAATTGTGAAAACTTGTTTTCTTAGATTAAATGAAG
TTTTTGATAACAAAATATTTATTAATAATGTGCTAAAAAGTAAAAAGTAG
AAGTGAAATAATGAGATAGGAGGGAGAGAGTAATGAAATTGATTGAGAAT
TTTTGTGAGTGAGATGTGAGGAGAGAGAGTAAAATGTTGAAAAGTGATTTTT
TCAGTAAATCAATATAAGTATATTTACAATATTAAAAAGATGTGATGATT
GATTGAGAATTGTAAAATTGTTTTCGAGCCGTGTTATAAAAAGTAAAGTA
GGTGACTTTTTGTTTTTCGTTTTTGATGACAAAGAATACATAAGGAAATG
TTAGTCAAGCAGGGCCTAGAAGTTGTATTGTGACTGTGAATTAAATATGA
ATCATTATAAAGTACTACTATTAAGTGGATATAAATTGTCACAGCTAATT
TGTTTCAAATATAGTATTTCTTTCCATCATATGTTTTTCCTTCAGGCCAT
TATGTAAAGTTTGAAAGGATTAACATTAAGATGCATCGTACTGTAGCTGT
CAAAAAGTTGCTAGCTGAGGTTTATCTACGTGCTAGATGGACTTCCGCTT
TTTGGGTCTTCACTAAATTAGTGATTGATCATTCTCCTCAAACAATTTCC
ATGAATATTATGGATAGTGAATGTGCTGGAATTAGTTTCTATATATGACG
AAGCTTTATGTCTAGAAAATTCATGATTCAGTTTATGATATTTTTATTTT
TAAAACAAGGTTCTCATTTTTGTATTTAAATCGTTTTTGATTTTCCAGTC
CAGAGCATCCTTCATTTCGAGCTCGTGGTCGATCAATGCTTAGTGGCGAT
AGGCAAACATTTAGAAATTATTCTGACCACATTGCTGCTTCAAGGTGCTT
ACTCTGTATGCTATCTTTTGCTTTGTTTTGCTTCCTCTCCTCTCTTTTCT
TCTTCTATTTGTTTTTGTTTGGCTTGGTATTCCATGATGGGCCAGAGATT
AAACAGGCAAAATATATAACCAAACTCATCCTATTTATCAAACTCAGAAA
CGTTGTAGCCTTGTTTTGGCCAGCATAATATCTGACTGAGAAGTGTATGC
AAAAAGCGTACGGCATGTTTTGGAACAGGGTTCGTACTGCTGAATTGGGC
TTTGAAAGTCCAGTACGCGTAACACCCCGGTTCCATGTCTTCTGCTTTTA
AATTGTTCAAAAATGGGTGCAATGGGTACAATTTTTAGTAGTTTTGTTTT
GTTCAGTTTGTCAGTTCGTTATTTTACAAATAATTTCAATAATGAGCAGA
ATGCTGATATTTTTTTGTGTGTAGCTACAGGTCAGAAGGAGCATCAACCT
TGGAAACGCGTAGACTACGAAGAAGAAGTATCAGTATAACGCCAGAGATC
AGTGATGACATCGTGAGGTTGACATTTTCTTTCTTTTTGTTTCTTATGTA
CTCATAATTATTATTGTTTGATCTTGCTCATATTGGGGGTTCAACTGCCA
TAGCATCATTTACCTTTCTCTTTCCATGAACATTATTGATTGGATATCAT
TGATGTGTCTGTCTGTTAATTGGATTTTTGTTTTCAAAATTTTATTTACT
CCGGAGTCAGTTCATTCTATTAACGTAACTATTTTGATTGTGATGCCGTA
TTTTTCTCACCTCTCTCCTAGGGCTGTACGAGCAATGAATGAAACTTTGA
AGCAAAATCGTCTCTTGAGAGAACAAGGGGATAATAGGTCAGATCCCTAT
TCTTCAAATGATGGAAATCATGCGGCAGATCTTCAAAAAAATGTATGTTT
TATTTGATCCGTGCAACTTGGGCCTTCTTTAATACGTTACAGTAGATTAG
TTTTTTGCATTTACTTGACATATGGTTTCTGGTAGGATACAATTAAAATT
TTCATCATCCCAAAACTGTCTATGAAACAAATAATGTATAGTCACTTTTG
GTAGAAAATAGATGGTGATGCAAGTGGAGACTCGAGACATATTTGAAATG
ATCTGCAGAACTGGAAAAACAAAGCCACTTCAAATAGTAGACATGCTTTC

CCTTCTATGTTATTATCCCTTGCATCAACCAAAACTCTTGTTTAGTGGCA
CTCGGAAGTGTCACGTCAAAACTTTTTAGTGACATTTTTTTGGTCTACTT
GTGTCTTCTCTGAATATCATGGATAGTGGATACTGACGTTAATAAAGCTC
ATTGGAATTTCCATTGACACCACAAATACAAGATAGCAATGACACCGACA
AATGAATTTTGTGACACTCATAAATGGCATGGGATGCAAACTTTAAATTT
TGACATGTATTTCTCTAACGGTGAAAGAAGTATGTCTCCTAAACAATCCT
AAATGATCCTTTTTGTTTTAATTGCACTGGTCTTCGGAAAGGGAGATCAA
ATTTTGTGCTGATGAATCTAATAAATAAGCTATTTGGTTGAATATGCATA
TCATGATGCTCTCGAGCTTCGCTCACTTAATCTGATTATAGTGCTTTGCT
GATAACATATCAATTAAATACATAATGCTTATGTTCACAAATATTTTGTT
ATCATTTATCATTTCAGTCATGTTTACGTTGTAGCATCTTCCTTTGGATT
GAGGATCTTACTGTTGAAGTGTATTTACTTGAATTGTAACTTTAACTGAC
ACAGCACACTGATTCAGGTATCTGGTTCTCATGATGGAACATCTGGCGAG
AGGCTTTCTTCATTTGCCCGCATCAGCAGGGAGCACATGAGCTCTCAAAA
AGCAATGTCTCTACCATCATCTCCTCATGAGCTTAGGAGTCGGTCTTCTG
ACTACCTGGTCAATAATGATGAAATGGTCGCAGCATGGAACAAAATTCTG
GAATCCCCCATGTTCGAAAATAGGCCTCTGCTGCCTTATCCGGAATGGAA
CATTGATTACTCAGAACTAACTGTTGGTACTCGTGTCGGAATAGGTAAGC
GTTATTGTTTTAGTATTTACAGGTCTATTTCTATCTTCTACACGTGTCCA
TTTTAGTGCTTTACAACGCACAAAAATGGTAAGTCTGCTATAGTTTTCCA
ATGCAGCTGTCTCTGTGGCTTTTTCTGAAGAAGTAATTTTTTTTTCTGCT
TATTTTTTTTATTTTGGAAATTCCGTTTTTTACTGTTGGGTTACGACCTG
TATGGTGGAGCATTTATATTGATTCTTTCCTATGATAATCCGTGTAAGTT
GTTTGACCTCCATGTCCTAAGCTGTTGCAATCGTATGATGTTTCAAATTG
ATCAGTTTACTGTCATCTGATTCCTGGTGAGGCCTTCTTCTCCTTCTCTT
CTTATGCTGTTCAACATCCGGCCTTTTCCTTCTTATGGCTCTAATTTCCA
CGTCTTGACATTTCATCTTTTCCTGATATCTTTAAAATGATTATCACAGT
TTCTTACCTTAAAGTTATTTGTAAGGACATATGTTTGTCATATCTTTCCT
GATAGAAGATATCAGCTCCTTGGGGTAACAATTCTCTTAGTTCTGAAGCC
TCCTATTTTCTCCAGTTCGTCTTGACTACGATCCCTCTAATAGTGGACAT
GGATTAATTTAGGAGGGTGCATGCCTCGTTAAGGTGGAGGTTTCTTAGAA
CTCATATCTTCATTTCTTAGATTCTGTAGAGACATGGCTCATTTTCCATT
TCTGACTTGCGACTTGTAGACCCGTAAAGAGATCTCAGATCTTTCTTGAT
TCCTTTAAACTTTGCATTTGGCTATTGTTGGCTGATGGCATCTGATTAAT
CTTTTGGAAACTTGCTTCACATTGGGATGCCATAAGTTGAAGAACGTAAT
GCTAAATATGCATTGTTGATGGCAAGTATGGAAGCTGTTCTTGGACAAGC
AATGTGGTTGATTAAGGACCAAGAATTACCGCATTAGTCTCTTGGTTCCC
TGTAGAGAGAGGTCACATATCATTTTGGAAGTTTGCATGGTTTTTGGTTG
CTTCAGTACAACCAACATATCAGTACCATGTAAGGTTGCCTTAATCTCCA
ATATTTGCAAACTTGGTAAACGGAGGTTGCCTTATGATTAATTGAAGTTG
TTCTTGTGGAGGTAGATCTTTATGAGCATACGATTAAGAACGGACCCTCC
ATTCTTAAAATAATATATCATTTGACTTTACATAAAATTTTAAGTAGTGG
TTGAAAATATTTGATTTTATTATATTGCGCTTATTGGAAAATGTGTGGCT
CAAAAAGGGAACAGCCGCCTTAGGTCTCATAAAGACCAATTGTTCAACCG

CCTTGGCTCTTACAAAGACCAAGAGAAGTGTTGAAACTTTTTGAAATTTG
AATGAATGGTTGAGGAGAGGGTTAATTGGTAGAAGTGATAAAATATGTTT
GTTAAAAGAAGGAACATGGTCAATTATTTTAGAACATCATAAAAGGAAAG
TAGCACTATTATTTTGGAATGGAGGGAGTACTTCCCTGTTTTTGTTTTTG
TTTTTGTTTTGTTTTGTTTTGTTGATTGACATTTGCTCTGTTCAATTTAG
TCTTATGAATAATTGCTTTGCATATTGTAATATACTGATTATCTTTCTTA
TGTCTTTTTTTTTTTATTTTTTATTTTAGGGTTCTTTGGAGAAGTTTTTCG
TGGAACTTGGAATGGAACAGATGTGGCTATCAAGGTTTTTCTGGAGCAAG
ATCTCACTCCTGAAAACATGGAAGATTTCTGTAATGAAATATCAATCCTG
AGGTATCTTCTCTTTAAATAGCGATGGTACCAGTTTTCTTCATTGTTGGC
TTATCTAACCATTTTTCTTCATTGTTGGCTTCTCTAACCATGTTTTTTTA
ACCCTTCTCTGATATTTTGCATGTTCTCTCCAGCCGCCTCAGACACCCAA
ATGGTACTTTCTTTCACTTAACTCTTTCAAATTGGCCAATGTCATGTCTT
GTTTGTGCTTTCAGAAATATGAAGGAAAAAGAATGGAACAAAAAAATATGC
TGCATGGAATTGGCATATAAGTTAGGCAGTTTCATTAATGGGAAAGCTCT
AATGAAACCCTCTACTCAGTCAAACTGAACCATGGGACCAGAAAAACCCG
TGCTCCAAATAAAGTATTTTGAGCATTTGGGCAGTTTTCGTCTTCTGCTG
GCGGAGGGTCGAAAGAATAGAAACAAGCATATTTTTTTCTGACTGTCGGT
TATTTAGAGCAGTTTATTTTTCCACTCTAAAATGACATGAGAAGTAGGTT
TAGGAAGACTCTTTGGTTAATTATCTAAGTAATCTCAGTTTCGATCAAAC
TTCTCTTTGCTTCACCTGTACGTTGGGGTTACATGAGAGAGTGGTTTTCT
AGGCTCCATTACTTTTCAAACTTCCATGAAAGTTGAAGGGGCTATTCCGC
CTTTTCTTGTAGGGCTTTGGTTTGAGCGCTACTTGCATTTCATATGTTTT
GCTGTGACAGAGCTCATGTAAAAACGTTTATATTCAGTATATGTACGCTT
TTGTACAGAAGTTTAAGTGCCTGCCACATCTTTATATGGGATATGGGTTA
GGGGTCATTGTTTAGTACAACAGTAAAACACTAAGCGAACAATCATGAGG
TTGTGGGTTTGAAATTGACTGGCCAATCTTGCAATTAAGGCCTACTTTAG
GTGTATAGCTTCTCTTCCCTTTGGGCACTCTGTTTACGGGCACCAACCTG
GTTCATGCCATTTCTCATTTGTGCTTGTTAATCTTATTATATATGCAATG
ACATACTATGTGCATGTGCACTGGCACATTGATTTGCATGATATAGTTCC
ATTATTTGCATGTATGTGCTCGTTGCTGTTTCATTTTTGTTGATCCAACT
GATGCTCTTTATTGCAGTTATATTGTTTCTCGGTGCATGCACAAAACCTC
CACACCTGTCCATGGTTACTGAGTACATGGAGATGGGTTCCTTGTATAAT
TTGATCCATTTAAGTGGTCAGAAGAAGAAACTCAGCTGGCGGAGGAAGCT
TAAAATGCTGCGTGATATATGCAGGTTGGTTGACTTTCCCCTCGTATGAA
GATTAAATACAAACCAGTTAACTCATCAGGGTTAATGATATGCAGCATTA
TGTTCATCTTTGTTTATCATTTTATTAATATTCTTGCAAAAGTTTCTTTA
GCAAACGAAAAAAATTCTTGCAGTTTGGTGGAATGTTTTTGAATATCTTC
ATGACCTGTGAAGATTCAATTGTTTTTTTTTGTTGCCCATTGTTTTTAGC
CATGTTTGGGGATAGAAAATGTGTACTTTGAATGCTCATTGTTTGTGTGT
TTTTGTCACTTGCTAATTTCAAGGGTCACTATTTGTGTACACTATTTAAT
GTTGCCTGCATTGATCTGAAAATGATTTTAATATCTAGATGCATTATTAG
GAAGACATTTTTAATTATTTTGGTGTCCACAAGGTAGACAAAGTAGACAC
GATCTTAGATGTATTGATTAATATAGGTGGCATTTGGTTACGTTTTTGTT

TTTCTGTTTCTTCTTCCAAAAAAATAAGATGATAGTTATGTTTTGGTCTC
AATAGTTCTGAAAATAGAAATGCGTTTGGTAACGACAATTGTTCTTAAAT
GTTTTCATAGTTTATGAGATCATAATCAAAGAAATATGGAAAAAATGTGA
AATGAACTTCAGATTGTCTGAAATAAATGAAATAACATTAAGAGAATTCA
GTATGTTGGTAAATTTAAATATATATTTTGATGGTGAACTCCAGGTATAT
GTAAGGTTCTGTTGGTTTCAAACATACCGTAACAGTATGTTTGGTTGGAG
GTAATAGAATGGAATCAAATACTATTCCATTACCTCTGGCCAAACATGCT
GTTGAAGTTATTGCAAAGAGAACAACAAATTCATGGCTGAAAGTGGTCCA
TTTTTGCAGTTGTGCGGTGTTGCTTCTTCGGTGATAGTTCTTCTCTAAAT
ATTCTCCTGTCTACCCTCGCCAAAAACAAAAACTGAAAAGAGAGAGGGAG
AGAGAGAGAGAGAGAGAAAGAGAAAAGAAAGAACTAACTTGATAACATAT
TCATCATTCTCATCCCGAATTTGTTCTTGAATGGATTTGCTCCTCAACCC
TAAAAAAGGCTGAGTTTGAGGGTTATAAATATTTGTTGTGTGTTGATTTT
ACTGTTTCCTAAACTGTGTTGTCTTTGAATCTTTGGCAAGTGCCTGATCA
GTTGGGGGGCGATTGATGATTATACAACAATAACAACTAAGCCTTTGTTC
CACTTAAGTAGGGTTGGTTACATGAATCCGTTTATGCCATAAAGATCGAT
CGAGGGCCATTCCCTCAGTCAGGTTTATGTCATCAAATCAATTCTAATAG
TCTTATCCTAAGTCTTCTTAGGCCTACCTCTCTCTCTAGTAGTCCCCCCG
CACTTGAAGTAAATCACATTTTCTCATTGGTACTCTATGGGTCTTCTGCG
GACATTGTCCAAATCACCGTAAATAATTGATGATTATATTAATAAATATT
TCTTATGATTAGTAATCTGCAGAGCTAGCTTCGAAAGAGTTTTTTGAACC
ACCAAATGGTTCCAAATTCCTGGGGGATTAGGGTTTCTGAGCTAGTTCTT
TTAGTTTAGATAGCACCAGTTAATATCAAGGGAAGTTGGAATTTCCACCG
CCACCACCACCACCATCTAATTGCTAGGTCAAAATGAACTTGCGTTCCCC
ATCGCGCTTCAGTGCACTGTAGTCTATTTACTTTGACAATAGTGTATTAA
TTTTCATGTCAACTTCGACCATTAAGTTTTCCCAATTGCTTTTGAAGTGT
TGGCAGCCGTAAGCCCTTTGATGGGGATAAAAGCTTCCAAGCTGAATAGC
CATACCTCTGCAAGTTCTATTTAGTATTTAAGAAATTTTCATAAAGGGTG
CAACAGATATTAAATTTTTTTTTTTAAAAAATTTATACATGCCATGATTG
ATTGATTGATTGTTTTTCAGCTCACCATATCTTGAAACATGAAACATCGA
TTCGGTCTTTGAAACTGAATCATGCTAATTAGTGGATTTTTGTTTTTCAT
TACATGTTCATGTTTGCAGTGGAATTATGCATTTGCTTTTATATTTATAT
TTGAGACAGATGCTGGGGCAATAATAAGACTGTAATCAACATGCGACGTG
CCAAGAAGATATTTGCCAAATTGAAGTGAAAAACTAAGGCACCGTTTATA
TACTAGAAGAGATAGATGACGTGTGATGGACTTGGACTTCTCTACCCACT
TTCTTTTTCAATTATTTATGAACATTTTTAGAATTAAGAGATCATTTATA
GGTAAGATACTAAGATATTGTCTCTACACTATCATGCACACTTTTGATTT
TTTGCCTTTGCTTTTAAGTATACTTTCTATGTCAAAATAACTTTTTAAAT
TCTAAAAAGGACTTAAAAAAAGCTTTAACCAGCGTTGACAAAATATGACC
CACTTTTTTTTATTAAAAAAAATAATAAGGTAATATTGCAATAGTTGTGT
CATCCATTACCTAACTACTCTTTCTTTTGTCTGGGGCATTAGAGGGCTTA
TGTGCTTACACCGAATGAAGATAGTTCATCGCGATCTAAAGAGTGCGAAT
TGCCTTGTGAATAAGCATTGGATGGTCAAGATCTGCGATTTTGGCCTCTC
GAGAATAATGACAGATGGAGCAATGAGAGATTCTTCGTCAGCAGGGACTC

CGGAGTGGATGGCTCCTGAACTAATCAGAAACGAGCCTTTTACTGAGAAA
TGTGATATTTTCAGCCTAGGGGTCATAATGTGGGAGCTATGTGCCCTAAA
TAGACCATGGGAAGGTGTCCCGCCAGAACGGGTATGCAAAACATGCTTCA
TTCTTGATTATAATACCTAATCTCTTGCATCTCTTTGTTTGCACTTCTCT
GGAAAATTGCACTTTTGATCCTTACAGTTTGGGGTGAGTGTTTTAGTGGT
TCCCGTGATTTGGAAAATAAAAACCTTAAAGTTTCATTCGTTTCAATTTG
GTTCATTCAAAAATGGGATTAGCATGAAAAATGGGATGTGAAATCGTTAT
TTCGTAGATCCTAATGTCAATCCACATGCTCTGCACATGTCTTTTTCCGT
TTCAATATGCTAATTCCATTTTTGATTGGACCAAATTTAAACAAAATTGA
AACTCTTTGGTCAAATGTATTACTTTCCAAACCACAGGGACCACTTAAAC
AACTACCCAAAACTTGAAGCATCAAAAAAGAAACCATCCCGCACTTCTGT
TCGAGTAAGAATTGATTTTTTTTTTTTTCCCGCTTTCATAGGTTGTCTAT
GCTGTTGCTAATGAGGGATCAAGGTTGGAGATTCCTGAAGGTCCCCTTGG
CAGGCTAATTGCAGGTACTCCCGGATCACGTTTTTGTTCGTATTTCGACA
AAAATTATCAATACCACGTTTTGATTTCACTTTTCCTGGGCGATAAGTTA
CCCGGGCAAGCAGAACAAACACATCCACATTTTGTTTTTGTTCTCGTGCT
ATCTATTTCTATTCCTAATTTTCTGGGAACAGAAACACTAAACAAATGCC
ACTAAGCTGATGCCGATTTATCATATTCCTTGCCGCAGATTGTTGGGCTG
AACCGCATGAGCGACCAAGCTGCAAGGACATTCTTACCCGTTTGCTAGAC
TGCGAGTTTTCCCTCTGCTAA
>EUC21870-RA [gene]
ATGGAGTGGACTAGGGGTCCTACCATCGGCCATGGTTCTTCCGCCGCCGT
TTATCTTGCCACCGGTGCTTCTGGTGAGCTCTTCGCCGTTAAGTCCGCCC
AGCTCTCTCGCTCTAGCTTCTTGCAGAGGGAGCAGAAGATTCTCTCTCAG
TTGAGGTCTCCTCATGTTGTTGGGTACCTGGGTTTTGATGTTACGTGCGA
GAAAAACGAGCACGTTTACAATCTGTTGATGGAGTATGTCTCCGGCGGTA
CAATTTCCGACGCGGTAAAGAACAGAGGAGGCTCGCTTGATGAAGCGATG
ATCCGGTTATATTCTCATCAAATTCTGCAGGGATTGGATTATCTGCACTC
GAATCGTTTGGTGCATTGCGACATAAAGGGGAAGAATCTTCTGATCGGGA
AAGATTGCGTGAAGATTGCGGATTTGGGATGCGCTAGATTTGTGGAAGAC
GGAGATTCTGCCGCTGAGGCGTCGATGTTTTCCGGTACGCCGGCGTTCAT
GGCTCCGGAGGTTGCGCGCGGCGAGGATCAAGGATACCCAGCAGATGTTT
GGGCTCTCGGGTGCACTTTGATCGAAATGGCAACCGGGTCTAACCCGTGG
CCGGAACTGAACGACCCTGTTTCTGCCCTTTATCGAATCGGGTATTCCGG
CGACTTGCCGGAGTTTCCACGATGGCTGTCGGAAGTTGCTCAAGATTTTC
TGAGCAAGTGTTTGAAGACAAATCCCAAGAACCGGTGGACAGCTGAAGAA
CTACTCAAACACCCGTTTGTTGCCGATTGTGAGCAAAAGCTAGAAACATT
TACAAGAAATTCTCCTACTGCCGTCTTGGATCAATTGTTTTGGGATTCTC
TGGAGGCATCGGAATCATCACCGGATCAAACCCAGATCAGTTCACATTTG
AATTCTCCAGCGGAAAGGATGAGACAATTGATCGGAGATGCTTCTTCATC
AAATTCCGATTTGCCCAAATGGGCAGAGGAAGAAGATTGGATTACTGTTA
GAAACAATCATACAGAGGAAAGCACTCAATTTCCCCAGCTGAACGCCGAT
CTGGAAGATGAGAACAGATTATTTTCTGAAACAGAATCATTATTGTCTTC
AACTGTTAATCAAGAAGAGCTTGTGAGCTCAAGTGATTATCAGATTTTGT

TATTAGATAGCTCTGTTGAGGAGGAGATTAGTAATAGGATTGATCCTCTT
TTCTGTTTTGATGTTCTTAGTGATTGTCTTCAATTAG
>EUC03449-RA [gene]
ATGGGTGTTAATGGTAGACGATCTAATTACACTTTGTTGAGTCAGATACC
GGACGAGCAGCATCACCAGCAACCGCCGGCGAAGGTAGCCGCCGTTGCAG
CTCAGTATTACGAGTCTCAAATCATGGGGAAGAGCGACAGAGGATTCGAC
TGGGAATTAGTCGATCAGAGAGTTGTTCCGCCGCAGAGTCGGATGGGGGC
GACGGCTTTTTCCGGGACGATCGGGTCACAAAGGCATTCGAGTGGGAGTA
GTTTCGGCGAAAGCTCGATTTCCGGCGACTATTATGTGCCGTCGCTGTCT
AATCCGGATGGGTTTGTACAGTTGCATGACGACGGAGGAGTTGAGTTGCT
GTTGAATACGGCCGAGGGCGGTGGCGGTGGAGGATCATCGTCGACAAAGA
GTTGGGCGCAGCAGACGGAGGAAAGTTATCAGTTGCAGCTGGCGTTGGCG
CTGCGATTGTCGTCGGAAGCTACTTGTGCCGATGATCCCAATTTCTTGGA
TCCGGTGACGGATGAATCGATGGCGAGATCATCTTCCTCCTCTGCTTCTG
CAGAGGCTATGTCACATCGATTTTGGGTATGTTTTTACCATCCTATATTC
CCAATTTCCATTGCCACTAGCTAATTTCTTCAAAATCATAACTATATACA
TGTGTTCAAATTAATCAGTAGATGCCATTTTGAGCAGAAATTTTGTCTGA
AAATCATCCATATTATGTATTTAAAAGTGTCTTTCGATTGATGGGTTGAG
ATTGATATTGATACAAATCAATGGATAAAATTGTTTTGGGAGTTTTTGGT
GTGGGGGTGGGGGGGACAGATTGTTTGTCTGAAAATCATCTATATTATAA
ATCGGAAAAAATGTTCATCCATAGTTGAGATTGATTTTGATACAAATGAA
TGGATAAGATTGCTTTGAGAAATTTGGGAAGTGAAAATTCCCGTCCACAT
ATGAGAAATTTTGTCTTTCATCACATTGCTTTTTAAAGGTTTATTCTAAT
TTGTATAATTATCCTTTTCGATCAAAATTTAAATCAATAAAAAAGGGCTG
CCCAGTACACGAAGAGTCCCGCCATTGCAGGGTTTGGGGGAGGGTCAGAT
GTGTGCAATGTGCATGTATTTTTTAATTGCATTCAAGATTGATACCGTAT
TTTGGTTTACAAATTCATTTTTGCATCAGGTAAAGGGCTCCTTATCATAT
TTGGACAAAGTTCCCGACGGTTTCTACTTAATCCATGGAATAGATCCATG
TATATGGTCTGTATGCGTTGATTTTGAAGAGAATGGACGCATCCCATCGA
TTGAATCACTGAAGAACCTTGATCCTGGGGCCGAATCGTCAATTGAAGTC
ATTTTGATCGATCGATTTAGTGATCCAAGCTTGAAGGAACTTCAAAATAG
GATTCATACCTTGTCTTGTAGCTGCATCACCTTGAATGAGGTCGTTGATC
ATCTTGCGAAGCTCGTATGCAGTCATATGGGGTGAGTTTCGATAAATCAA
TTAAGCACTCAACAAATTCAACTGAACTTAAAATTTTTGAACTTATCAGT
TTTCAACACTTAAGAAATTCAACACACGAACTTCAGAACATATCAGTTTT
CAGCACTTAATTTTCAGTTTTACCAAACGAGTCCTTAGTAACTTGTTTGT
TTCTATGACAAAGGGGTGTATCTCACATTGGGGAAGATCACTTAATTCCG
AACTGGAAGGAGTGCACTGACGAGCTAAAGGATTGTCTTCGGTCCGTTGT
CCTCCTGATTGGTAGCTTGCCCATTGGCCTCTGCAGGCATCGGGCTTTGC
TATTCAAAGTACGTATTGTTGTTGTATTTTTAGAATAGCTTGGTTTGACA
TGAAAATTTAGGTTTCTTATCATCAAATTAGTGTTATTATTGTGTCTTTT
GGTTAGATTTTGCATTTCTATTGAGATAATCATTTGATTATCCCCCTACC
TATTCAATTTTTCGGTGCATAATCAAAATATTGGATTTTTTTTTTTTCCC
GTATCATCTTGCAGGTGCTAGCTGACACAATCGGTTTACCTTGTCGAATT

GCCAAAGGGTGCAAATATTGTACAAGAGATGATGCTTCCTCATGCGTTGT
TCGTTTTGGTTTCGACAGGTAGGCCAGGGGTGGAGCTACTAATGGTCTAG
GGGGGACTCAAGCGCCCCCCCCCCCAAAAAAAAAACTTTTGAAAAAAAAT
ATTATTACCCCTGAAAAAAAATATTATTACCCCTATGCTCTTTGAAAAAA
AAAATGAATGAAAATCCCTCAAAATTTTACATAAGCCCCCTTGAATTAGA
AAAATATCTAATAAACTCCCTAAAAAAAATTAGCCTCCCCTCAACTAATT
TTTTGAACCTGCAGCTGTAGGTAGGCCTCAACAACTTCCACAATATGCAT
CACCTTTAGTTTCATTCTATTTCTTCTGGTTAATTGTTATAATTTTTCTA
GAGTTTGTTGTATGTGTTCATGTCAGGTTGTTCTTCATTTAAGGTTATGG
TATGTGTTTGCATTTACAGGGAATATATGGTTGATTTGATTGGGAATCCG
GGATGCTTATGTGAGCCTGATTCTTTGCTAAATGGTCCATCTTCCATCTC
AATTTCTTCACCGTTGTGCTTTCCACGACTCAGACAGGTTGAACCTCCAA
TTGATTTCAGGTCATTGGCCAAGCAGTATTTTGCGGACTCTCTGTCTCTT
AATCTTGTATTTGATGATTCTTCTGCAGGTGATTCTTTCANCCCCCCCCC
CCCCTTCTCTCTCTCTCTATATATATATGTGCGCGTGCGTGCGTGTGTTT
GATTTGATGTTTATTTATGAGAAGATGCGTACTTGTCAATGATTTCAGCC
ATTCAGTCTGTATATTTGAAACTCAATTATGGTTATTTTGCTTCTTCATT
GATCTTTCTTATTCTTCTTCCCATATTCTGTAGTGGAAGTGATTCCTTGC
GTAGGTAAATTTGATCCTATACTGGTGATTTTGCTATTTTCATGCAACGA
TGAACTCTTAAAGATTTATATTTTATGGATTCGAGCATTTGGGTTTTGAA
AATTAATGGTCTTTATGATGACAGGCGATGTTCAACATCAACATTTGTTG
CCTGGTACAAGCGGCAACAATGATGTTTCTCACTCAGCTCTTCTACCATT
GAATGCATGGCAAGAGGGTTTTAATCGAGATTCCCCAAAGTCTGATTTCT
CAAGAAATATACCTCCAATTGGGCATACAGATGATGTTCAACAATCTTTA
CCCTCTAATGACTTGAGGTTTGGTATGGAAGATTTAGACATTCCGTGGAG
TGATCTTGTTTTAAAAGAAGGGATTGGAGTGGGTAATATTTTTTTTCTTC
ATAATACTTCAGTGATATATATATATATATATATATATATATGGATGTTCTT
GTTGTCTGGAAGCTTTACTTGAACAAGATGACTTGGATTTTCAGGTTCTT
TTGGTAATGTTCATCGTGCAGATTGGAATGGATGTGTATGTAATTTACCT
TTTTTTTTTTTCCCTTTCGTTCTTGTTTCGAAGTTTAATTGGTTATATAT
CTATAAATTTGACATGAATTGCATCTCCTCACCTTCTACTTTGCATTTAA
ACATTGAAAATTATGTGTTGAAAATTTGTAAGTTCTGAATTGATTAAGTG
CTGAATATATTAAGTATTTACTTTTTGAATCCTTTTAAATTTTTGTCCGT
GAAATAATCTTTACGACTTGCTTTTTATTAAAAATTCTCACTCTATATTT
TCTTAATTTTAAGTGGGACAAAGGCTTAGTTATTATTGTTGTGTTGTAGT
ATTTTCTCAATTTACCCTTCCCCAGACTCCGTGCATAGCGGGGGCTTTGT
AGCACCGAGCTGTCATTTTTTAGTATTTTCTCTTTCTATAATTGTTTGTA
TATAACTGTTTTTTTGTAAGATTTTATGGTATTGATGTTGAATAGTGGTT
GTGGTAGATGGATGTTTGACGGTTTTAAGTGAGGAGTGCTGGAAAATTGA
GTGAGTCACAAATTGCTTGAGACTTTGAACAATTTTTTAGTAATACTTAT
CAAACGTAATCCAAAAAATAAAAAATAAAAATTCATGGATTATTGGAAAA
AAAACAATAGAGAAGTTCTTATATAAAAAAAAAAGAAAAAAAAAGAAGTA
ATTATATTTTACTTTTTGAAAAAAAATCTTAAAATCATTTTATTTTTAGA
AGTAGTTTTTTAAATGCGAAACAAATGCTATTTTTAAAAAAAAAAAAAATG

CAAAAAAGTACAAAAAAATTGTTACCAAACGGTGCCTTATTTGTATATTG
TTTTTTTTATAGGATGTGGCTGTGAAGATTCTCATGGAGCAGGATTTCCA
TGAAGAGAGAGTTAATGAATTCTTGAGGGAGGTAAGTTTTAGCAAATTTC
CATAATTCATAATACTTAAAAGTATGAATATATTTAAAAAAAAAATTATGG
TTGATGATGTTCATCTTAATGCAAATTTCCATCCACTTGTGCTTCCGTTT
CGCGCGCTTTTTTTTTTGTAATCATTAATTTGTAAAAGTTTTGACTATTA
ATGAAAATTTTAAAATGTTTTCGTGCGCTCGTGCGCACACGTGTATAAAT
TATTCTTGTCGATTTGTAATTTAATTTACCGTCTTTTTTAGGTTGCAATA
ATGAAACGACTACGGCATCCAAATATTGTGCTCTTCATGGGCGCTGTCAC
GCAGCCCCCAAATTTGTCCATCGTGACTGAATATTTATCAAGGTTTGTCA
TTAATTTTTAAGTGAAATATATCTATATCTATATATCTATATATATATAT
ATATATATTTTATTACTAAGTAGGAGGTCTGCTTGACTGTGATCTATAAT
TTACGAATGCAGGGGTAGTTTATTCAAACTTTTGCATAAACCTGGCGGTA
GGGAAGTATTGGATGAGAGACGCCGGTTGTTAATGGCTTACGATGTGGTA
TATATATATATAATTTTTTTGTGCGCCGACCGTTTTCTATGATTTTTCTT
TATCTTGATTTTGGGTCAAGTTTTTTATTTTTTATTTATTTTTTGTATAG
GCGAAAGGAATGAATTATTTGCACAAGCATAATCCACCCATTGTTCATCG
AGATTTGAAATCTCCGAATCTTTTGGTTGACAAGAAATATACGGTGAAGG
TAAGAGTATTAAGGATTTATTTCGGTTGAGCAATTATTGAACTATTAGTC
CTTTGTATTAGTTTCTCGATTCTCTTGGGCACTGTTTGGTATTAGTTTTT
TTTTTTCCCCCATAAAAAAGTACTCAATTTTAAAAGTAGGGTTTGTTTTA
CGCTTGAAAAATATTTTTAAAAATAGAAAACATAAAAAACATGATTTTAA
GTACTTCTTTTAAAAATAAAATGTTTTTTGTTTTTTCCATTTTATTTTTT
ATTTTGCTTTTAAGTAAAAGTACTTCGTTTTAAATTCACTTTTTATTTAT
TTATTTTTTCAGGTCTGTGATTTTGGCCTTTCCCGTCTAAAAGCAAATAC
CTATCTTTCATCGAAGTCGTTGGCGGGAACGGTAAGTTTTTATTTTTTTT
TAATTTTTTTCCCATTGCAGCATAGAATTAATAAATCAATTTTTCTTAAT
TTTTAATTTTATTTACTTATTTATTATTATTATGATAGCCGGAGTGGATG
GCGCCTGAAGTTCTTAGAAACGAACAATCAAATGAAAAATCAGATGTTTA
TAGCTTCGGCGTTATCTTGTGGGAGCTTGTAACTATGCAACAACCCTGGA
GAAATTTAAATGCTGCTCAGGTACATACATGTTGTTGATTAATTAATGGC
AAATACACATTTCTTTATTGTTGAATAAAAAAAATATTAATTTTTGAGAC
TCGATTATTTACCGTCGAATGTACTGTTTTTTTTTTTTAGGTTGTTGCAGC
TGTTGGTTTTCAATATAAGAGGCTCGAGATTCCGAAAGATTTAAATCCGA
AAGTAGCCGCCATAATTGAGGCTTGCTTCGCAAAGTAAGTCGTCTCTCTC
TCTCTCTCACACACACAGATGCACACACGCTCGTGCGTGCGTGCACACAT
TAAAAAAATAAAAAAAATCCAGCGCCCGATTGCTTAATTTGAGCCCCCTC
TCAAAAAAACAGAAAAATAGATATTTTCATATTTCATAATTCTTGGGGAT
TTATGTCAAATTTTAATGGATTTTTTTTTTTGGGTAAAAGTATCGGGATA
ATAGTGAAAATTTAAAAAAAATCGGGGGCTTGAGTCCCCCCGGGGCACCA
AGCAAATCCGCCCCTCTCTCCCTTTCTCTCTCACATACACAAAAAAAATC
CAGTGGCCGGGTGCTTGGTTTGAGCCCTCTCTCTCTCTAAAAAAACGCAA
CATAAAAAACCCCGGTGGCCGACTGCTTAATTTGAACCCGTGCTCTCAAA
AAATAAAAAGTAAAATCCAGCGGTTCACTGCTTATAGTTTGAGCCCCAGG

GTTTTGATTTGTAATTCATATCGGTTTTCATTGCAGTGAGCCATGGAAAC
GCCCTTCGTTTGCCAGTATCATGGAGTCCCTAAGACCGCTGACTAAAGTT
CCGACGCCTCTAAGCATGCCATCGCTCACATGA
>EUC03396-RA [gene]
ATGAATGTGATTCAAAAGGCAGATGGATCGCGCAGGATTGTGTTGTCTGA
ACCGACTGGATCAACGGCTGAGGTTCGTCTCTTGTTTCTTAATTGTTCTT
GCATGTAATTGATGTGGTATTTTGAAATGTATAAAGAAGATAATGTTGAA
AAAATTAGTTCAAAAATCCCACCATTTTTGAGACCTTTAAGGTTGTCAGT
TCAGTCTGGAGAAATAATTCTTTTAAGGTTGTAAGAACCTTGTAGTTGAA
TTTTGAAAATTGGTGCAGGTGCTTCTCCATGGTGGTCAGGTTGTTTCTTG
GAAGAATGAAAGAAGAGAAGAACTGCTTTTTATGAGCAGCAAGGTCAACC
CTTCTTCTATATTGTTTCTGTTTCCGCTTCCAATAAACTGGGACGAGAAC
TCTGTTTCTGTTCTGGATATTCCATGACTAAAAACATGTTTGTTACCTAA
AATTTTTTCTTTTAATTTAAGAGAACAGAAACAAAGATACACGTTTGATT
GCAACTTTTGTTCCCTTTTTTGTCTTGAACTGTATGACAACAAATATACA
TATTTCTTAGTCGGCAGCTTGGTGGTGGCCGGCGGACTGGTGATGGGAAT
AGTTTTGTGGGAGTGAATAAGGAATTCATGAAATACTCTCAAAATTTTGG
GAGCAGAATTGTGTTTACTAAACGCGTTTTTGTATATTAATCCTAGAATT
TTGGGAGCAGGAACAGAAATCAGAGAACGGAAACAGGGCCTTGAATCCAG
AATTTCGGGAGTAGTAACCAAATGGGGCCTTAATTTCTCATAAAATGCCA
ATTAACATGATTAGAATTCCGAAACTGATTTCTTAGATGGTGTTTGTTTA
TTGTTTCCATTCCTTTTTTTTTTTTTTAAGGAACAGAAGTCAGAAACAGA
GAAAGAAAAAAGGAACAAAAACACTAATGAAAACACCTTCTTACTTGCAA
ATCCATCAAATCTTTATGTATGAAAAATATGAGGATTTTTTCGTTTTACA
GGCAACAACAAAACCTTCTAAAGCCATGTCAGGTGGCGTATCCGTCTGTT
TTCCACAGGTTTGATGCTAAAAAAAAAAAACCTGGTTTTCTACATCTCCA
TCTTTGTCGAAATTTTGAATTTTAACTATTAATTTCAAACCGTAGTTTGC
AAATTTGGGTTCGGTAGAACAACATGGATTTGGAAGGAACAGATTGTGGT
CGGTGGATAATGATCCTTCACCTCTACTTCCTGCTAATAACCAATCAACA
GTCGATCTAATACTAAAATATGCAGAGGAAGATCTAAAGACTTGGCCACA
CAGGTAAACAAACTACATGCAAAAAGCATTATACTTTTAATCCCTCAAAA
TAAAAAACGTTGTTAATGATTTTGGATGTATAAATGGCATGTGCTCAGCA
CGTGACTTTCGAATGAGGATATTTTCGTCATTTCATCACTCCACTAACCT
TAAGACGTCTTCCATTAACCGTTTTCCAACAATGATTCGAGAGAGATAAG
TCTGAAATGTCATCAGGGGAAAACTTCTAGTTTTAATTGAGTGATAAAAT
GACAAAAATATCCCCATTAAAAGTTACGTTCTGACGTTCTGTGCACAACC
CATTTTTCACAGACACCCACCCCATACTTTAGGGACACAAAATATGAATT
TCCCATTCTCTATAGTTGTATAAGGTTTGACAATTTAAAGTAATTTTTTT
TTTTTTGGCAGATATGAGTTAAGGCTTCGTATTTCTCTGAGTGCTAACAA
GCTCACTTTGATCCCTCGTATGAGAAATATCGACAACAAGTCCTTCTCGT
TTATGTTTGGTTTGCGTAATTACTTATCAGTATCTGATATCAGGTTCGAC
TTCATATATATATATATATATATATGCATATATAATCATGGTTTTCCATTTT
AGCCGGTGGGTTGAAAATGAAAATAGGTGTAATTTTTAATTTTTCGCAGC
GAAATTCGTGTTGAAGGGTTGGAAACACTTGACTACTTCGATAATCTGTT

GCGCAGAGAGAGGTATACCGAGCAGACTGATGCAGTTACCTTTGATGCTG
AGGTATTCAGTCTTATGTTATCCATTTTGCAGTGTCTGAATACTAGGTAA
AACAGACAAGTCATTTTTAAACTACTTTTTCGGCTATCAATAAGTAGTAT
TTTTTTTTTTTTTAATTATTTTAAGATTGATAGAGTGTACTTAAGCACGC
CGACAAAGATAGCCATGATAGACCACGAGAAGAAGAGGACCTTTGTGCTG
CGTAAAGACGGAATGCCTGATGCAGGTTTTGATTGTAAACTTCCTTTTTT
TCGGCCGGGGGATTGGCACTTTTTACCCCAAAAGTTTGACCATTATTTCA
CTTTGATCCCTATAGTTGCAATTTGAACACGTAAAACCCTGAAGTTCCGT
GATCATCCCACTTTTGTCCAAATCAATAACTTCATTGCAATTTTGGATGA
CAAATAGTCAATTGTCGTGCATCCAACTTTTTTTGGGGGGGTTTTATGGG
TAGTTCTGCAAAGAAACGCTTATATATATGTATATATATTAGGTTGTATG
TATATTTTTGAGTATTTGGGAAGGAGGCGGCTGAAATTGCCCATAAAATC
TCTTAAAAACAGTTAGATACATGGGATGATGCTGGTTTTCATCTGAAAAT
TGTATTAACTTTGACTATAGTGGGATGATTAAGAAACTTTACGGTGTTCT
ATGTTCAAATTGAAACCACGAGCACCAAAGTGAGATTTTAATCAAACTTC
GGGGATATATAATGTGAAAAACCCTTTCTAAATATATATATTTTTTTTGT
TACGATTTTTTTTTAGTTGTTTGGAATCCTTGGGACAAGAAGGCGAAAGC
GTTACCGGATTTGGGGGACGACGATTACAAGACAATGTTGTGTGTGGATT
CAGGTGTTGTTGAGACATCAGTTGTATTGAAACCTTGTGAAGAGTGGAAG
GGGTTTCAAGAGCTGTCTACTGTCTCCTCAAGCTATTGCAGTGGCCAGTT
GGATCCAAGGAAGGTTCTTCATGGACTTACCGGCTAGTTTCACCTGTCTC
TTTTTCGGGTAAATTGCCCCCGCCCCCCGTCCCGTAATAACTCTTGTTAA
TACAATAAGACGACACTACTACTTTAATCTCTCTACTCTTTTTAACACTA
ATTCTCACTTGCACTCCAATTTCACTATTTATATTTGTCACAAGTCACAA
AAACACGACGGTGGATTCAAAAAATTGGTTGAACGGGGGACTAAATTTTT
TTTTAGGGAGTTCGTTGGGCGTTTTTTCAAAAATACCGTGATAATGGTGA
AATTTTTCAAAAATCCAGGGGAGGGGCTCGAGTCCTCCCGGGTATTTAGT
AACCCGCCGATGGATTCACCTTAGCCCTTACCTCTAAATTTTTTAAAAAT
TCACATTTTTGCATGTTTTATTTTTTGATTCTTGTTTTCTATTTCGACAG
GCAAATTGTGGTATTAAAAAATAAAAATCAAATTATTTATATTCCTCGTT
GAAGGCATGAACCAAGAAAGAGAGTGTTTATACCCGACACTACCTGTTAT
TCTAACAAATATCTCAATATTTGGTATTTCAGATATTATAAAAATAAATT
GAGGTATGTTATTTATTGAAATAATAAGATATCTTTAAATTTGTTTTTCA
AACCAACAAGGTGTAGTTGAAGTAGCAAGGCATACTTGAAAGGTATCTGT
AAGGTCAGGAGTTTGAGCTTGTGAAAAAATATTTGTTAGGAGGGACAACA
ACGCATCCTGAGCGAACTGTTTTTCACTCTGTATTGGCAGCGAATTATTT
TTTACTCTGTGTCGGTAAGGGGACACTCGTATGGTTATAAAAATAGTAAA
AATTATTTTTCAAGGGGATTGATGTAAAATTTATCATTTTAATTAAAAGT
CTTTGAGTTTGACCATGTCCAATTAAGAACATTTTTGTCATGTCATTTGC
AAATGTTGTGATGTCACATCCGATTTCATTTTTTTTTTTCCTTTTTATTAT
ATAATCTAAATATACTGTGTAATTCAACAATATGTAACTCAAGTAATAAG
ATAAATGACAAGACACACTTAAGTTAAAGTAGTTAAGTGGTTTTTGCAAA
GTTTAAAATTTGAACTTGCGAAAAAACACTTATTAGGAGCAGCAGTCACA
CACCCCGAACGAGCTAGTTTTTTACACTGTGTGGGTAGGAGGACACTCGT

GCGTATAAAAAAAATAAAATTATCTTTATTTTAATTATTAGTAAATTCTA
AATGAACTTTTATTATAGAAGTTTCATGGCTTTGATGATGCAAATGAGTG
ATATTATTTCTTTCTTTTTTTATTTTTTAAGAAAAGATAAATGCGTTTGA
GTAAACTTACCAAAGAAGAAATTTGAGTAGAATTATATGATAGATAAAGT
AATAATAATATTATGAGTTTATACTCCAGATATTCTGACGCTCTTATTAT
TTATCATTTTTATAATACGTGAGACAATTTTTTTATTTTTTATTTGATAT
AAAAGTGCCGTTGTTTTTTATTTTTGCACCTCTCACAAAAAAAAAATTCAC
AAATAATCGATTCAATTTTATAAACAAAAATCGATCAATAATATCGAGCT
GTTAAATGTACTCTCGCAATTAACTACTATTAAGAATCAATAAAAGAGTA
TATGCGGCATTTGAACTTAACAATTTGTACCTACTGCCAAAAAAAAAAAA
AAAATCACGAAACAAATTTTTAAAATAATGCATGATTAGCATAACAAACA
ATTTTAACTGAATAATTTGAACCTACTATAAAAAAATATAAAAAATAAAA
TTAGAAAAAAAATACGATCACTTGTTGGATTGAACACGTCTTACGCCCCA
AAATTTGGGACCATAACAAAAAAAACAATATCTTTAAAATAGTGTATGAT
TACGTATAATAAATAGGGGTGGTCAAGGGCCCGGGGGCCCGCCCAGCCCG
AACGGTCCTATCCTATTTTTTGAGGACTTAGGCTTAGGCTTTGAGCCCAT
GAGTAGGGCCTGGGCGTAGGTGTTAGGGCCCGATCAATTTTGAGGCCGGG
TTTGGGCAAGACCAAAAAATTTTAGGGCTGGCCCGACCTGAAGAATAATT
AAATATATATATACACACACACACACATATTATACACACAAGACAACAGT
AATTTTAATATTTCTTGTGGATATGATATTGTAATTTTTGTATTTATTTT
TTTAATTAAAAAAAAATTATAGAGCCGGGGGGCCCTAGGTTTTTTTATAG
TACCGGACTTTGGGCTGAGATTTTTTTTATATGGCAGGGCTGGGGCTTGG
AATTAAGGCCCGATTATAGGGCCGGGTAAGGCTCGGGCCTAGCAATTTAT
TATAATACCCTAATCTAAACCCGGCCCGGCCCATGACCACCCCTGATTAA
TAAACGTATCTTTTCCAAAGGAATCTCATATTATTCCATTTATGTCCCTG
ATTGTCTCCACTCTATTCAGGTCATATTATTATTATTTATTTGATTTAAA
GAGATGTATACGTGTTGTAAATGGAAAATTATATCTTCGGTCCCCAAATT
ACAGGTCGGTATTTATATGGACCATGAGATTTACGAAATTACATATTTGG
TCTTTCAAGTTTCACGTTTGTTTCAATTGTACAAAATTTAAAACTTTGTT
AATGATTTTAGATGAAAGATTGCTCAGCACAGTAACCTTCTAATACGATA
TTTTCGTCATTTTAGACGATTTTTTAAAAAATAAAAGAGAAAAATATGAA
TTTTTATTAGGAAAAAATTATTTTAGGTATAATAGAGTGATGAAATAATG
AAAATACAACTCATTTTTTGTTCAAAATAATTAAAGGGCGCTTACCATGA
AAATAAATATTTTTGTTCAAAATAAATCACGATCATCAAAATGAGAAATT
AATTTTTTTTTTTGGAGTTTTGAATTAGAATGTATATGAATATTATATAT
AACATTAACTGTGATGTTAATGTTATATATAAAACCACTACAATCTGTGT
AACCTGTGAATATTAAATATAACATTCATTGTAATAAAAATAATATCAAT
TTATGTTAATAATAACACAAATTATATGAACGTATTGAATGTAACTCTAT
TTTGATAACGCATAAAATCGTATTAAATATAATACGAATGTGAAAACTGA
AAATTCTGAAAATACCATGTGAATGTAACATGAATATACAAATTTGAAAT
TAATGAAAAAAATAGTAAATGTAATATTATTCAGATAAATCTAAATATCT
AATTAGATCGTAAAAAAAATGTGTACGACAATAGTTAAATTGATTTTAAT
AAATTGATTTTATTGTGAAACTGTTAAATATGAAAAATTCCAATCTCATG
AATCTCACAGATGTAATTTCATGGTTTTTAAATTTGGGATTGGAAAATTT

GAAAATTTTGTATACTGAGATTGTGTGGTTTATTTTTAAATATTAACGAC
GTTTTTTAATTTTGTAAGATTGGAACAAATGTAAAATTTTGACGGAATGT
GTAAGTCTAAAAACTTCGAGGTCCACATATTTAAGGGGAAAAAACGACAG
GAGAAAAGTGGTAGACCATATTGTTTCCTGACGCAGACATCAATTACTTC
CGGTACTCCAAGATCAAGAAACTGAACCGGAGTAATCGTTGATAAGCTTA
AAATCAGCTCACCCAACTACTCAGAATCACTCCCTCAAAAAACCCTTCTG
ATTTGTTCTCTGTTAATCGGAAGTGTTCTGTTCTTCTCCTGTTACCTTCT
TCGTTTTCCAGCCATTGATTTTCGCCGTCGCCGACCGGAGTTTTTTTGTG
GGTTTTTCGAATTTTAGTTTTTTAAACACTAGGCTGTTGCCTGGTGGCCA
GTGGGTTGTCACCGGAAACACGTGGGTTTCTTCCTTTTGTTTTTACCTCC
CAATCGACGGCATCTACTTTGAAAATTCTTCGATCTCAAAGCGTGATTTA
TGGGGGTCGTCTTCAATGAAAGCAGAGCAACAGGGCATTTACTTCTTCTC
TGCAGAGCTACTTAAATGCGTGAAGAACAACTTCAGGGGTTTTCGATCTT
GTTCTGAATTTTGCTGAAACAGAAACAGAAATGGGATCTGGAAATGGGTT
TTATTCAGTGGAACAGTTCGATTTAGAGTCCAAGTGGTTGATTGATCCAA
AGCTTCTCTTCGTTGGACCCAAGATTGGAGAAGGTGCTCATGCCAAAGTG
TACGAGGGAAAGTAAGTCAACTCTTATTGCATCAGTAATCCGCCATTTTT
GTTGCAGCAAGCTATCTTCAGATCTCAAGCTATAAGTTTTGAAAATTGAC
TGTTTTGGACTAATGATACTGGATTGATTTACTACAAGAATTTTTAGTAC
AATGTTGTTCGTATGCCATGGATTGTGGAGTACTTATTTATGCCTTGCTC
TTTTGTTTTTATGGATTTTTTTGGGACCAATTCGTGGGAGTTTGGATGTC
TTGATTCCAAATCGATAAAAAGTTTGGAACACTAATTTTCTGATTAGGGT
TGCATCATTAACCGCCGCCGGCCACCTTACTCTAGCAACCCCTGTAGCTG
AAACTTTGGTCATCGGTTGCCGGAATTGTTTCTAGTTACATTCCCCTACT
TTTTAGTAACGTTTTTTTTAATATACATTTTGGTTACAATTGTTTAGCCA
TATTTAATTAATTTTTCGGTTGCCGGTCGTCAGAAAATTACGTGAATGTA
ACTAAAAAATATTCTAAAATATAGCTAAAAATACACTTAAAAAATGTAACT
AAAAAGTATATTAAAAAAATAGTTACATGTAACTGAAAATACACTAAAAA
AATAGTTAAATGTAACAAAAAAAATCGGCTTGGCGGGGCAACATCGGGTG
GCGCTGACAGTAGATGATGCAAATATCAATTGGAAGCATTTGATTAGAGA
TAGAGCATGGGATCTAAATTTTGGACTATTAACGAATTATCCCTCCTTTT
TTTTCAGATATAAAAACCAGAATGTTGCTATCAAAATTGTTAACAAGCGG
GAGACACAAGACGAGATAGCAAAGAGAGAAGCCTGTTTTGCACGGGAGGT
TGCCATGTTGTCAAGAGTTCAACACAAGAACTTAGTGAAGGTTGCCCTAA
ACTACTTTTTTTTTCTTCCCTATGTCGGAAATATCAATGAATTTCATGCA
TTACTATTACTAATGTTGTATGTATCTATGCATGCAGTTCATTGGAGCTT
GTAAAGAACCGATCATGGTGATCGTAACTGAGCTTCTTCTTGGCGGCACA
TTGCGTAAATACTTGCTGAACATGCGGCCGAGATGCTTGGACAGGGGGGT
CGCTATCGGATTTGCCCTCGACATAGCACGCGCAATGGAATGCTTACACT
CTCACGGCATCATTCATCGTGACCTCAAACCTGGTATTTGTCTATTTTCA
ATATTCTTTTGAATGGATCATAAAAGAATACAGGCTATTCTCTTCTGTTT
ATACAGCGCAACATTAGACTAAATATGGAAGGAAATTGCCCATAATAAAT
GGGCTATGACTAATGAAAACAAGGAAACTATCAAATCGGTCAACATGACC
TAAAAATACAAAATATGACCTAGATACTAGATATTCCAACACTCCCTCTC

AAGTTGGGGAGAAGATGTTTCGTGGTCCTAGTTTAACAAGAGTTGACTAG
AGAAGAGATGGGCCGATCGATTTGGTAAACATATTGCAGCCTGTTCGAGG
ATAAAGCAAAGTGAGGTGTGATAATTCCCGCGACGAACTTTCTCTTGGTC
GAAGTGAATATTGACCTCAATATGCTTCATTTCTCTCATGAATGACTGAG
TCATAGATAGCATGATGGCAGATTAATTATCGCAAAAGAACAAAGATGAG
TCCATCATGGGTAATCCTAACTCAGCCATAATGAGCGAAGCCAAAGGTGT
TCACACATTCCCTAGGCCGTAGCGCGATACTCGACTTCAACAGAGGGTCG
AGAGACGAATGCTTGTTTCTTGTTTTTCCACAAGATAAGATGACTACCCC
GGAAGGTGCATTGTCTTGAGGTGGAATGCCTTTCACTCTTTGACCCTGCA
TAATCAACGTCAGTAAAGTAGGAGAGTCTATCCTGTGCTCTCGCCATATA
GAAGAGACTAAGGCCGGGACAAGTTTTGAGGTACTAAAGGATGTGAGGTG
GCTGCATAAACTACCTCACAACACTCAAGGCATAAGTAAGATCTGGCCTC
GTGTTGGTCAAGTACATAAGACGGCCCACAAGTCTCCAATAATGCTGAAG
GATCGGTTGATAATTCTCCTGATTCAGTTGTCAACTTAAGGTTCGTGTCC
ATTGGGGTCAAGAAAGGTCTACATCCGAGCCAATGTTGCAAAAGGCTTTC
GTGGGACTCGCCCCGGGGCTTGCCCAGAGGTGGGGCTGTCATAAATTGCC
CGGGGCTATGAGGCTGGGCTATTTTCCAATTGGGCTTGCACCCAGATGCT
GTGGGCAACGCCCAACATCGCCCAGGGCTAAGTTTATCAGGACTTACGCC
CTGTGTTTCTATCATTAGAAAAATCAAGAGAGAAGATTTGATTTTGCACT
TGTTTGGTATGTTCGTCTTCGTCTTCGTCTTCATCACTTCGTTGGTTCCA
GACAACGAAGGCTGGAGGTGCGGCCAAGGTTTGGGGTAGCAGATGTTATT
ATTCAAATAGGAAGCAGATGTCGATAGCGGTGGAAATAGGAGAACTATGA
TTAAGTTATGCTGTATTTTTATGTTTTATTTGGAAATAAAGTAGGCATTC
GATAGATTAAAGAAGAACAAAAAAAGAACAATATTTAATAGCATCCCTAA
ATAGGTTGGTCCCATGTGGAAAAATAAATTAAATTACTATTTATGATTCA
TAACTTTTGTTATTTTATGATTTTATCTATATTTATTTTTCATATATATT
CTTTTTATTTATTTTTTATATCTTTTAATTATATATTATATTTTTAAATA
TTAAAAATTTAGGTATCATGGGGCTTACAACACTGATCCGAGCATACCTG
AATCCTGAAGTAAATTGAGAGCATACTTTCGTTGAGATAGAGATATACCT
TGGCGAGAACGAGCTACCTCAACCCCGAGGAAGTATCAAAGAGGACCAAA
ATCCTTAATGTTGAAGATTTTTTCAAGGTTACTCTTTACCCGAACAATAC
CTAATGCGTCATCTTCAGTAATGTGGATGTCATCCACATAGATGGAAATA
ATGATATACTGACCCTAAGGATGATGATAGATGAAGCATGTATGATCTGA
GTAAGTTGGGTAAATCCCATGGTAAAGGAACTACTGGACAAATCCTGTGG
GAACTGCTCAAGTCCATAGAAGGACTTTCAGGTGTGACATATTTTCCCAG
AATACTACCCTTGAGTTCCAAAATTTGGTAGAGGATCCTCGTCGATGATT
TCAGACAGGTTGCCATGAGAATGCATTCTTGTCATCTAAGTTAATGAAAA
GGTCAATAATGGGCAGCAGTCAAAAAGATAAGGAAGCGAACTGTAATCAA
TTTTGCAATAGGGCAAAGTTGCAGTGAAGTTCGTGCCCAGTATCTGAGTG
AATCCTTTGGCAACTAGTTGAGCCTTATATTCGTCAATAGAACTATATGC
CAGGTATTTCACATTGAACATTCACTTACAGCCAATCGGTCACTCACCAG
TAGGACAAGTCCCTAGTCTGATTATGCGGAAGAGCTTCCATTTCCGCTTT
CATAGAGGCCACCCATTTTGATTTTGGAGAGCCTCGTGAACTGATCGAGG
GAGTGGAATTGAATCAACCTTGTCAAGGAAAGATTTGTACTGATCATAGA

GACGCTGAAAGTACATATACTTAGCAATAGGATGATTAGTACTGCTTGAG
AGAAGATAGAGGGAAGGGGTGGCATTCATGGATGGGATAATGGGGTGGAA
TAGAATCAGAGGTTATGGGAGATGGTGGTATACTTCCCAAGAAGATACTG
GCAGGGGAGCTGAAGCACGATAACGATAGGAGTAAACCAGTAACAGGACA
GAAGGAGAGGCCGGAGAGGAATCACTAGAGGAAGGTGTAGTGGTTGGCGT
ATAGGGATTGGGCATGGAAACAAAGAAGGGTCCTCCGTGGAAGGGTCTGT
TGTGGAGTATTCCTTTGCAGGCCTGGCCAAGGGACGATAAGTGCCTGAAA
GAAGAGTTACAGGATCATAGCACTTAACCTTTGAACATGGCGAGTACTCT
TAGAAGACACATAAAAATAACTGAGCCTCATGCTATTGTACTAAAATCAT
GCCGTATTCACAATGGTGTTGTTTTTATCCTATAAACCACATGGCATGCT
TCGAGAACGTTTCTGAATTACTTTGCATTTGTTCCCTCTTCTGTAGAAAA
CTTGCTGTTGACGGCAGACCACAAAACAGTAAAGCTTGCGGATTTTGGTT
TGGCAAGAGAAGAATCGTTAACCGAGATGATGACTGCTGAAACAGGAACC
TATCGCTGGATGGCTCCAGAGGTATATATTACATTTTTTTACAATTTTCT
TTTTATTTCATTTTTTTATTTATGTTTTTTTCCCTTCAGCTTTACAGCAC
AGTGACATTAAGGCATGGAGATAAGAAGCATTACAACCACAAGGTTGATG
CTTACAGCTTCGCAATTGTATTGTGGGAGCTCATACACAATAAACTTCCA
TTTGAAGGCATGTCAAATCTTCAAGCAGCTTATGCCGCCGCGTTTAAAGT
AAAAATTCCCGTCCTTTTCTTTATTTTATTCTCTATTATCGGTTGTTTTT
CATAGAAACAGCCTCTCTGTATACAGGGAAATGGTTGCGTACGTCTGACC
CTCCCCAGACCCCGCAATGACGGGAGCCTTCGTGCACTTGGCTGCCTTTT
TTATTTTCTGTTGCTTTTAGCATATAATTTTCATTAAATATCAAAGTAAG
GAAATGATTTATTTATTTCTCGGGTTTTGATACTTGCACACCTGAAGTTT
GACTGAAATCTCACTTTGGTCCCATACTTCGAATTTAAACATATGAACAC
TGTAAAGTTTCTTAATCATCCCACGACTGTCAGTCGTTATCCCCATTACA
ATTTTGAACAGAAAACAATCATATGCCATGCTACTGATTGTTTTCAAGAG
GCTTTATGGGCAATTCCAACCGCTTCCTTCCCTCCTCTCTTTCCTACCCA
AGTACTTAGCAATATACATTTCACACATACACACTAGGGATTCCCTATGC
CTAAAGGCACTGTATTGATTTTTCTAATTTTTAAATGTAAAAATATAGTA
AATAATAAATTAAATTTAAATTTAAATATAAACTTAAATGGTACAAAATG
TAATTTTAGTTAACTGTTTTAGAAATCGGGGTATAAAATTTTTTGTGAGA
TATTTTAGGATAGATTGTTAGTGTGTTAGAGATATTATATGATATAAGAT
TATAAGAATTTAAAATGATGCCTAATCTCAACCGTCAGATATAAAGTTGA
TCCAACGGTAAAAAAATGTCTATAATATCTCAATCCTTGGATCTAATCCAA
GGGCTGGAAATGTCTCTTGGAATTATTTACTACTGTTCCTTAAATATGTT
CGCTTAATAATAGTACGTGCACAACAATTGACTGTTTGTCGTTCAAAATT
GTAATGGAGTAAAAATTTGGTTTGAAATGAGAGGATTACGGAAATTTAGG
GCGTGATGTGTTCAAATTGGAACTATTGGTGACCAAAGTGAAATAACGGT
CAAACTTCAGGGTTGCAAGTGTCAAAACCCCCTTATTTATTTTCTAATTC
TTGGTTAAGCCTTTTCCTTTGGAGGTGCCTTTTGATTAGCGTTCTTGTTC
CCAGAAACCTTGGAATAGAAACTTAAAAACAGCATAAAAAAATTAATAAT
TTGTTTATGTTCCTATCTTAATTATTTGTTCTAGAAATTTGGGGTCAGAA
CAGAAACAACTTTTCTTGTTCCTCTTTTTTATTTTTATGTTTCTGTTCCA
AGTTTTCTGAGAACAATAAGATATAGAAACAGAAACACGAAACAAACACG

CCTAATTTGAGATTCTAACTCTTTTTTTCCGCAGAATGTGAGGCCGAACG
TTGAAAACTTACCGGAGGATTTGGGTTTAATAATTACTTCGTGTTGGAAG
GAAGATGCGAACGAGCGGCCAAACTTCAGCGAGATAATACAGATGCTGCT
TCATTATCTCTCCACCATTACTCCAATAATGGAACCTGCTGTTCCTCCCC
GTATAATATGTACCTCCAAAAACTCCGTGTTGCCGCCTGAATCTCCAGGC
ACAAGCTCTTTAATGTCCAAAAGGGATGATTCCGGGGACACGCCTAAAAC
CCCCATGGAGCGTGAACCCGGAGGCGGACTTTTCTTCTGCTTCAGACATT
GTTACTAG
>EUC03168-RA [gene]
ATGGATTTGGCGGAAGGAGTTGGGGAGAGTTCGTCGCCGCCTCGGAATTT
TGGGAGCTTCGTTGGATACGATATCAGGACGCATGTGTATAGCAGGCTAG
TCGAGAGTGGAAATGAAGAGGCTATGTTTCCTGAATTTCGGGAACAGCTG
GAAGCTCACTTCAATCGACTGCCTCCTAGGTGAAACTCTGTTCAACATCT
TCACTTCTTCCGATTGTTCGATTTCACAATCTGCACAAACGTATCCTGTA
AAAATATACATCTCTATGCATGATGCTTGTGTAATCCTTGAATACATACA
TGGATTCTTGGTGTTTCATTTTTTTTGTAGATATCTGTTGAAGTTTTCTC
ATGTGCCTTAGTTATATAACATTTTTTTCGAATTCATTTGCAATATTGGC
CTTGAAGTTTTTATTCAAGATTAGGTGTCATATGAAATCGGTTTAATTGG
GAGCTAGCCTACAATTTGTGTATATTTGTTTCTGTATTTTCCTCCTTATT
TTTTGCATGCAAGTGAAGTGGGTCCAAGTTGACGATTATATTCATTGTTA
AAATCTGAATCCAATTATATTGATTTGAGTTATGAATATGGTATTAGATT
ATGCTATATTTAATCTTGTTTTCCAAGTAGTTTACTCTGTGGACATGTTC
AGGATTCGATTTTTTGATTTTTAATCTGGAAATCTGTTTAAACCTATAGT
TCTTTCTCTTCTATACACATTATGGATACCTGTGAATTGAATTTAGTTTC
ATATTTTAAAGTATTTATTGATGTAATGATAATGTTTCGTTTACATGCTT
TTATGTCTCAGTTACGGTTTGGATATTAACATCGATAGGGTGGAAGATAT
ATTGTTGCATCAAAGGCTTCTTGATTTGGCGAAGGACCCTGATAAACGAC
CAATTTTTCATGCTCGTCTTTTGGAGGTAGTGCTTTTAGTTGACATGTTA
TGCTTTTTATTATCAACTAATTGTTGCTTGCTTCACGTCATGTCGTAATC
CTGAATTTTTGTTTGTTTCATGATAGTGTTATGATTTCCTCATCCCCCC
ATTCCCTAAAATGGAATTGGTGAAGCACAATGTTAGAAATAATGTGTGAT
ACCTCAAACTAGGATATACTTTTGGCTGAGATTTACGTTGTTTTTATTTT
GATCTCTCAAGGAAGAGATGACAATAATGAGCGATTTTGGTTAACGATTT
TGTGGATTCCAAGTCAGTTAAGGAGTTTGAGGCTATTATCTACTTAAGGA
ATAAGGAGGAAAGGTCGCTTGTTTTTCTACTTTTCTGGTCAGATGAGTTC
CATTATATTTCATGTCCGTCTTTAAGGGAATAATATCTACTCGAAGAAAG
TGAATTAGGAAAGAAGTCGAGGATTAATCTGACACGAGGTGACTGATGAT
GCTTGCCCTCCAGCTACGTGAAAACTACATGCGTTTGAATGGCGATGCAT
CTGAGAAAAGATTGAATATCCAAGGAGATCTTGATTTGGTTATTGATCAA
CCAGGGATGATGATTGAAAAGAGTATGAATGAATTAAGCTGAGATGGAGA
TGAAAAGGAAAACAACAGACAATTTGATTATGCAGCCAGCTTGTACTAGT
TGGGGTCTTAAGACGAAGTTTTCTGTTCCTATCTGTTGCTTCTGGACAAG
AGTCAGGAATAAACTAACTTATGGTTGCTAACAAATGGTCCATTTACCAT
CTATTTTAATTATGGATACCCTAATAATATTTATCTTGTTTCACTTGTTT

ACTGTAATCAGGTCTTTGTTGATAATTGCATGGCCACCGATGTCATACAT
ATTGTTTTACCACCGAGATGACTGTTTGTTTTCTCCTGCCATTTTTTAGT
GATTCATGTCTTATTTCCTACTTTCTTGTTCATGTTTTTCCTATCTGCCC
CTGGATCTGTGGTGTTGAGTAGAAGTTCTGGAGTAGAGCAAAGGCTGATG
ATGGCCAAACTTTAAGTGTTCTTTCCACCCCAAGGCTATCTTGTAATGTT
GATAATGATGGATCTGTTCCATCGCTGGACAGGTAATTAACTATCTTGGT
CTGAGTTACATGTAATCAAACTGCTCTGCCATTTTTACTGATATTAGACT
TCCATGCGCTATATATCACTGACAGGGTTTGTGTTGTTTAATCATCCATT
GATGGGGCTTCCTCTTTTATTTTATTTACTTCACTTAACGGATAGAGAAG
GGTAATGTGAATTGACTCTGAACCTTACTTTAGGCTTGAGGACCTAAATT
TGGATGTCAAAAAGAATTCTCTTAAGATGGAGACGGAAGTTCCTGCAGAA
GACTTTCCAAGGAGGTAGTTTCTCAGTGAACTTTATAGTAACGCTATCTT
TCTTATATTGAACTTCTACAATGTTTGTATTCATTGGTACTTCAAAACAA
GTTCTATTTGGATGTGGATTAGAAAGACTGATCTAGTATAATTAGTAGCT
CGGTAACTGTACTATTGTATTTTTATTTTGATGTGGATGTGAAAGTCCTA
TCAAGTTCCATTGCCAGCTTTGTAACTTCGGCATGTGATGTAATGCGATG
AAATAGTAATGTCGAAAAAGGACTGTCACATCGAGTGTTTGATATAATTT
TCCAGGTAGGACAATATAATTGTTGGAAAGGTTCTGCGATTTTTTATATC
ATTTACTTGTTTGCTTTTCCTTTTCTAGGCTAACTGGGAGGTATAATGCC
TCTTAGAGCATCAAATCATCATCTGTGACACACTCATTCCCCTTGCATTT
ACATGATCTCAGAACAAGTTTTTAAAATGTGAGGTAAAAAGTTCTCTAGG
AATTTCTGTTGCCCTTGAGTTGGATGCTTTCACCCTCCCAGGTTCTAGAC
CCAGTAGACAAATTTCCAATATGAGCATTCACTGCGTTTATCAGTCCTTG
GAGAGTGAAGGTCGCTAATGGGGTACAATACTTGTTGGAACCATGGGCTG
TTGTCAATGATGAAAATTAGAGAAATAGATGATAGAAGAAGAATCAAATA
GCCACGAAGTTGATCGAAGGTCTTAATACAAGCTTACCATCATAACCCAA
CACGAAGTTTCATAAAATCAGCGAAAAAACCAGGTGGCATATCACTTTTT
ACCCCCGTCCCTGACCAAATGGAGCTCTTGAAGGGAACCGATCTCCTTAG
TTGCACGTATACGCATTGTATCCTTTAGTCTCCATTAATGAGGGTCAGGA
AGGGTGTAGACTGTGAACATACTCGAGCTTTGGCATTATTTCGAGGTTGT
TGCACTTGTCAAGGTTAGCACAAAAAGGAAGCAAACCATAGAATAAAACT
CAATTGAATCTACTTGAAAATTGGGATAACAAAATACCTCATTGAGTTTT
CTTAAAATGTTTCAACATTTCTAATGGAAAAGCATACTCAAATGACCCTG
AAAAATTCGGGAAAAAAAACATCAAAACCAACATGAGGATCCAATTGTGC
AAGACTTGATCATAACTTCCTCACGTTGACGCTAGTTTGATGTCGATGCC
AATTTCGTCTAGACGATATTGATGTCAGCATATTTTTTTGATGATCTTCG
GGTTCGACAATGTGAAGTATTTCATTGACTCAAAAAAGTTCATCCAAAGT
TTGGAGAGAGTAGAATGGAAAGGTTAGAGTGTAAGAGCTGCTGTCAGTTA
CATCTTAGTTCTAGGAATTGTTAATAAAATAAACAATACTGAAATATCCT
CAGTTGCCACTCCACTTGGTTTTCCTCTCTCTCTATAATTATAGATGGGT
GCTCCTTTTATTTCCTTTTCTCTTTCCTTCTCCTGTGTACCTCATCAATG
ATTCATACCCCCTGGAAAGTGAAACTTTATCATGTGAAATAAGGGAAAGG
GAAAGGGAAAGGGAAAGGAAAAGGAAATCAGAACTATAGAGGGTGTGAGT
GATGAACTTTACCAGGAAAAGGAAATCAGAACTATAGAGGGCGTGAGTGA

TGAAACTTCACCATTCATCACAATACTAAGGTTTCTCCTGGCCTGCTGTA
TGCAACTGTTGCGCGTATACCTCATCAATGATTCACACCCCCCTGGAATG
TGAAACTTTATCATGTGAAAGAAGTGAAAGGAAAAGGATATCTGAGCTAT
ACCGGGTGTGAGTGATGAACTTCACCATTCATCACAATATTGAGGTTTCT
CCTGGCCTGCTGTATACTGGTCAAGCGTATGCAACTGTTGCAAATCTGCA
GCACCTATTTAGCATATTTTTCACTTTGATGAGTATCATCGATGACTCAC
ACGCCTGTGTAGCCCATCAACAATTCACACCCTCTGGAAAGTGAACTTTA
TTATGTGAAAGAAGGGAAAGGAAAAGGAAATCAGAACTATAGAGGTTGTG
AGTGATGAGCTTCACCATTCATCACAATGTTAAGGTTTCTCCTGGCCTGC
TGTATGCTGGTCAAGCATATGCAACTGTTGAAAATCTGCAGCACCTATTT
AACATTTTTTTCGCTTTGATGAGTATCATCATCTTATTCTTCTCTAAAAA
TTCAATGTTTGCTGGGTCATGTATGTTTTAGAAATGTGGCATATATGTAT
GCATGCATTGCATGTCTGGAAGAAGTTATATTTGCTGTTTATTTTTTAAT
TTATCTACGATGCAGCCAATCAATGTTTGCACTTATTTATGCTTGTCACC
TTTTCCATTCGTAGTAGTTATAAGAGAATAGTTGACTAGTGCAAAGTCTA
GTGCAGGCAGGAAGTTCCTCAAATTCCAATTCATGAAGTAATATTTTCCA
CCATTGACAGGCCCAAGCTCCTTAGTCAGGTAAATGCTGGGGGTTATAAT
TTCATGATTGCATTAGTCGACTTGAACCCTCCTTTTTCAAATTTGCGTCT
ATTTCATTTTTTCAAATATCATGCTATAAGTTCTCTATAGTTTGTGGGAG
ATTGATGTCTCTGACAAATGTTAAATGTTTGATGTTGTAACCAAGATTGA
TTCAGAGCTATAAGAGGGGCCCATAAGCTGAGGTCAGCTGCGGGATTTTG
TTTTGGATTGTGATTGTTGATGGATTGCATATGAAGTTGACATATTGATG
CCAATCTTTAATGCTATACATACCCATCCTTGCATTGAAAAAGAAAAACA
CGAGGAAATAAATTTTTCTTCATTGCATTGCCCTTGTGTGTTAAGTTGAT
TTCTAAGACTTACCCTTGATATATGATTTTGAAACTCGAAAGGGGTATGC
TACACCAAGAGTTGACAAACTATTTAATGAAAGGCTCATGCATCTAATTA
AAGTTATACCTTATTTCAGGGTTCTTAACAACTTCACATAAAATAACATC
CATTTGGTAAAACTTTTGTGCACGCCCGCACGCATTTTCATGTGTATGCT
TTTGTACATGTTCTATGTAAAAAATTATCATCGAAATGTAATAATTTCTG
TGTAGCTTTCTGCGTTGCTCTCTGACATTGGACTTAATATCCGGGAAGCG
CATGTGTTTTCGACAACGGATGGATACTCTCTGGATGTATTTGTGGTGGA
TGGTTGGCATGCTGAGGTACTTCTCTTATGGTTTCTTTATGCTGCTACTG
ACTCCTCCTATGTATATAATTTTTCATTTACAAACTGACTGTTTACTGAA
CTGCTTGCTTTATAGTTTTTCAATGCTTCCTGTACTATGGGTTGCTTCTA
GAAATACTTCTGCACTGCCAACTCTGTTTACATTTGTCTCTGAACTCAAA
AGAACATGCTTTGCTGGGTTATTACATCCCTGTGGTTGGTGGCGATGTGC
ACAACTTGTTTTCCCGTCGTGTAAATTTTGAATCACTCTCTTTTTGCTGT
TGAAAGACAGTGTTCTTGTTTTTTAGGCTGCAAGTTTCATCTTTTTCTTT
CTGGAAAATTTGTTTCGGAATACTCATTTAGGTGTTCCTCTGTTTCTTTT
ACCTACCCCTCATCCCCCCAAAATGAAAAAATAAAAATAAATAAATAAAT
AAATAAATGTTTAGATTTTAATGTTTCACTATTAAGAACCATTTCCAAAA
ACTGCTACTTCTGCCTTTTTTCCAAGTCCAAGAACAACTGGAGCTATTTC
TGAAATAAAATTCAAAGCACGTATAATAGTACAATTTCTGAGTTACAATC
CAGGCGGGACCGCATGTTGAAAAGACACGGGTGGGGGTGGGATCTTATTT

TATCTTGGTGGTTAGAATAAACTAATGTAGTTGTCCTAAGATAGCATGTC
ATTTATAAGGAGCATTGTGATTATATTTTTCTATGACAGGGATCTGTGCT
GAGTCCAACTGTTGCTGACTTTGCGATGTTGTGGTCAGGATACAGACAGC
TTGTGTGAAGCTATGAGAAATGCAATTGCCAGAAGTGAGGTAATTAATCT
TTGACTAATAGTTTACAATTTTTGCTTTGCTTTTGTGTTGTTGATTCAGG
AATTTCTGTTTTTGGCCATTAGATTTCTGTATTTAATGTTGCTTAAGAGG
CTGAAATCGTTTTATTTTGTCATCTGCTATAATGGCTTTCACTGGCAATG
TTTCAGAAAAGTGCTGGCGCTCTCTGCTTAGGCAGAAGCGAAGGCCCTGT
TTATTTAGTGTTTCTGTCCCCCGATTTTTGTTCGTATTCCTAAAATTTTG
AAATTCAAACATGTTTCTATTCCTAGATATTCCCAAAATTTCATAAGTAA
TCGCAAAATTCTTAGGAATTTGAGAAACAAAAAAAAAAAAAAAAAAAACC
TTGATTCCATTTCATTCCCAAAAAACTAATCCCAAACTATTTCCCAGCAT
AGGAAGCGCTAGAAATTAGTTGATTCATTTATTAAGGTTCACCTTCAAAT
TCTCTTAGCCTCGAAGACAATTTTTCCCTCCCCTTTTTCCTTCTCTGTAT
CACGATCTTCTCTCTCTACCCTCTCTCTAATCTCTTCTTCCGTCTGGGCT
CCTCAGTGACCTTATCATTGGCAATGGTGTTATTGGCCGCTGGCTCTTCT
AAGCCGATCTTAAGAAAAAGCTCGAGGACCTTCTCCTTTTTCTCGTCCTG
CACTAACATTTTTCCTGTAAAACTGCGAGTCCAGCTCAGAAAATGAGAGA
CCCCCATAAAACTCTATTCCGGCCGCAAAGTCCTCCGAAACCATGGTCGC
CGTCTAATTAATGGCCTCGAAGTTGAATCCATGGTCGCCGTCGAATCCAC
CTGGAGAACAACCTGCGATTCAAGAATTCCTCAGTGACCCAAAGCTTCTT
TGCCGCTGCCTCCACTCTGCCTGTCAACACGCCGTCGACATGTGCTCCAA
AGTCGTCCTCCCAGTGTTGATCCGGTATGACGGGAGAAGAATCTACAAAT
CTTGAATTGTGGATTTTTTTCTTAATTTCAGATCTTGAATTCATTTTTTT
TTATATTTGAAATTATTCTGCTCTAGGACTGCAGATCTTGAATTCAATTT
TTTTTGTTCTTAATTTCAGATCTTGAATTCTTTTTTTTTATTTGAAATTA
TTCTGCTTTAGGGGCTGCAGATCTTGAATTCAAGATTTGTTTTTCTTAAT
CTTAGATCTTGATTTTTTTTTTTAATTTGAAATTATTCTGCTTTAGGGGC
TGTAGATCTTGAATTCAAGGAGATTTTTTTCTTCTTCTTAATTTCAGTAC
AGAATCATTTGTATTCATTTTTTTTTTTCAAATTCATTTTGCTTCTTTGT
ATCTGCAACTCCCAGGGAGAATTCAAAAATTAAGCATCATTTTTTTTAAT
GATTTGTGATTGTTGTTTGGATTTTTTTTAATATTTTCATTGATTTATTC
TGAAATTTTCTTTTTTAAAAAAAAGAGTAACAATTTGCTACGAGATTGGT
GAAAAATAGATTGGTTAGTAGCTGGTAGTCGAATATGGCAACGGTGTCTC
ACATGGAGAGAGAGAGAGAGAGAGAGAGAGAGAGAGAGAGAGAAACACAAGG
GAATTTAATGTTAACCAAACTTGTTTTTTTTTTATTTTTATTTTTATTTT
TGTTCTAAGAAATTCCGAAATAATTTTAAAAACAATTGTCATTACCAAAT
GTATTTCCTAGAATTATCGAGAACGGAAACATAATTCTCATCCCATTTTC
TTGGGATTAGAAATAGAAAATCAGAAACGTTAATCAAACACCACCTAGGG
TTTCCATCCTAGAGGATGAAGAGAGTAGTGATGCCAAGTTACATACTTGT
CAGTGCATTTTGTGTCTGCCTTTGATATTTATCACTCTTTACAGGTTACA
TCTGCTATTTTTTTTATTTTTGGCTTCTCTGAATTCTCCAACTGAAAGTT
TGAAATCGTTCAAAAGAAAAATGTCTGTGTTTCACTCTGTCATGCTATTT
CTGCATATTTGTGGACAAATAGTGAAGAAAGGTTTAACGCAATACATGTA

TTTAGATGCCATAACATGGCATGTGCTATTGGGTTTGGCAGTTCCACGTT
TGCATTTTCTTCCTCTAGTGTTCTTATTTGCCCTTTTTCTTCTTTGTGCT
GCACGTCTTGCTCAAACTGGTAAAGGATGACACAGCATGAAAGCATCGTT
GTGTGAGAAAGAGTTTCAAATTCCATATTCATTTCCATCATAGTGATAGT
TAAGTAATTCAGCCCTTGAACTTATGTTGAATTCTAGTCTTCATTAAAAG
GCTATCCTATTGCTTTGCCAGTCCAGATCAATTTACTGATTAAGTCTGTA
CAAACATGAATGGTCAGGAGTGTTCAAAACCCCACATAACCTGATCCGAC
TGCCTGAGCAAGACGAGTCAGCCTGTTTTCTCTCTTGTTTTGGTCAGACT
CGAGTTTGAAAAGAAACAGTAAATTGGTTAAATTTTCAGTGTAAAGGTAT
TGGTTTGTGCTTGTAGATCTATTATCCGTGACACTAAATTTACAGTTGAT
GATTATATATCTTGGGTTCAGAAATCAGTACAATGGTGGCGGCAATGATG
TTTGCCTTAATCAGATGGACCGATTGTTTCAGCGATCTTGAACCCAATTG
AAACTGATGACATTCTAGGTGGGCTATGGGTTCAGATTTCTAAATCCAAA
TATGGTCGGTTCTGTTGGCGTTTTTCCCAAGTGCAACCTGTGGACACCCC
CATGAATGGGTGTAATACCCAATAACCATGAGAATTGTTCAAGTAGCCTC
AAGTTAATCTTAACTATCTTCTGATTTCATTTCGTGTTCCATACAGTGGC
TGTCAGATTGTAGATTTTAGTTCCCCTCACCCCTTAAAAGAGAAGGAACG
AAAAAGAGAGGTGTGGTAGATTCTTCTGTTAGTTCTAGTAATGTATCTGT
TTATTTACCAAGCTATCCCAATTAGCTACTTCGGGCATGGGAAGGCATGT
CTTTGAAGATTCAGGAAGATAATGATCAGCATAGATAATATTAGAACACA
TTGATATATTCTCTAATTAATATTTTCATTTCCTAGCCGATGCCTTTCTT
TAATTTAATGACTTTACTTTGGTAATGCTAGATCTTCTCATGGAAAACAT
ATTTTAATGCTCCTGCCTGTTGTGGTGTGATTGTGATAGGTTGTTTGCTT
AAGTTTATGACAGCAACAAATGTGAGCTGATTAGACTCTTTTGATTATTA
TAGGGATCCTGGTCTGGTTCTTCACATTCTCAGTCAGCCAAAGAAAAAAC
TATAGTGCTGCCACCTAAATCTGGAGATTGGGAAATCGACAGAAGACTAT
TGAAGATGGGTGAAAGAATAGCATCGGGATCTTGTGGAGATTTGTGAGTT
GGGAGATAAGTGTTAAAATTAATTGACTTGTTCTATGCTTACATCTTTCT
GTTGCTCATTTTTCTCTTGGTATGAAATGAAAAGGTATCGTGGATTATAC
CTTGGTCAGGATGTTGCTATCAAAGTTCTTAGATCTGAGCAATCAAATGA
CACTTTGGAAGATGAATTTGCCCAGGAAGTGGCTATCCTTAGGTAACAGG
AGTTACAAGTTTATAAAAAAGTCCACTTGCATTTTATTTCATGTACTGGG
GAGGTCATTATGTTAGTGTTACTGATTTATCTTGAACATGATAGTTCATA
GATCATTATTTAACTGATTTATCTTGAAGACTTGAACATGATAGTTCATA
ACTTATTTGTTTGGACTAATAGTAACTTATTTGGATTAATCTTGCAACTA
ATACATCTTTCAACTTTGCTTGCTAAACTAAGAAAGTGCCTGTTATGTTT
TCCTGGAAGAACCATAAAATTAAATCTACTTTTATGGAGTTATTATTCCA
AGCAACTTGCTTTAATTATCATTTAGGGTTGCTAAAGAACAATGATGTCC
TAATTGTACCATTTTCACCATTATAGAATGGAATTGGAGATTTCACGAAT
ATAGAGCATGTTGACATCAAATACTTAATTTTTCAGCACACGTTGAGATA
ACTGAAGATGTATGTTGGATAGGAATGCCTCAATCAGTAAATACTTAAGT
ACAATATTTTGCTCACCGAATTACATGATTTTTATTATGATTAATGTATG
GCGTGAAGAATATTTTAATTGAATTGAATTCTTAACTTTTGTTAAAACCT
GCAAGAAAGTACACCTTTTTGTTGATGTAGTTATATATGATCATATTAGT

ATATTGGAAAATGTTTGAAAATTTCATTTTTCATTATCTAAATGTCGTAT
ACTTGAATATGCTTGTTGATCAGCTGATTATGTACCTTATGATACACTAG
AGATGCAGGGATTCATGGGGGTAGCCTAGTGGTAGGCTTACCCCTCCCCA
CACAGGTGGTGTGGGGTTCGAATCCCACCGTGTGGATATGTGGGTCCAAG
TCTTTAAAAATATTGCACAAAAAAAAATACAGAGAAGACTTAAGATGTCG
TGGGCCGACTAGACATCACTCACAAAGTGTTTCTTTACTAGTAGTTTGCA
AGTATACTGGAAAATTTATCTATGCAAATAGTTCATGTCGTGTTTGCCTC
CATCTTCAGTGGTCTACGTGGGATCAACTCTATCTGTTAAATTATGATCT
TAGTTAACTAGATCTGAAAAACATGACTCTGGCCAGCTGTTACGTTATGA
GAAGCTTTATATTAAGCTGATCCAAACTATGTAATATAACTTCTTTTAAT
TTTCCATTCACTGAGAACTTAATTTGAGCAGACAACAATACCAAGTAGCG
TAATTGGGTTTTTAGAAGGTCATCAAACGAATACATTTTTTTAACTGGTA
AGGATGCATTCTGTAAGAAACAATTCCATATTGTGTTGCCATTGTGTTTG
TTGATCTTTCTACTCATTCAAGTGTCAGAATTCTTGCCATATTCAGTTCG
AGTGTTGTGTTCATGTATTATTTTTTTTGAAATGACTCGTCATTTCAGTA
GTTTTTTTTAAATAATAAAAAAAAAAAATCTCCAGTTTTATACTTTTAATT
CATTTGAATGCAGAGAGGTTCAGCATAGAAATATTGTTCGTTTCATTGGT
GCGTCTACAAGGTCTCCTAATTTCTGCATTGTTACAGGTAAGGTAGTTTC
TTTGGGGGCAAATTGAAATGGTGACTCATCTATTAGTCAAGATTGTTCAA
AAAAGATATATTAACAATGATATTTTATTTTGCAATGAAAGAACATCTTG
TTTGCTGTGCTCTGCAGAGTACATGCCTGGAGGGAACTTGTACGATTATT
TGCATAAAAATCATATCATCCTGAAGCTCCCACAATTACTGAAGTTTGCA
ATAGATGTCTGCCGAGGGATGGAGTACTTGCATCAGAATCATATAATTCA
TAGGGATTTGAAGACGGGAAACTTATTAATGGATACTCATAATGTAAGTA
TACTAGTAATTACCCGTGTCTAAGGACACATTATTATTCCACTGTTCTTT
TTTTTTTTTTTTGTATTTTAAAATTATAAATTTAAAATGTAATTAATTAC
TTTAAAGTTATCTAATGTAATTATAAATGGTGTGGGTTTCTCAAAGTAAT
TAAGATATAAATGCAAAAGTTAAGATCTCCAAATATATAATCTAAGGAAT
GAAAATATCTTTCTTTTAAATTAAATGGTTGAGATTTCTTTCATGTCTCC
TTTAATAGATCTCAATCATTCTATTGACAAAAAATAACGCATGGAGCCCA
TACAGTCCGTGATTTATTATATAGAGTAGTAATTACCCGTGCCTAAAGGC
ACACTTTAATATTGTTTTAGTAAAAAATAATTTTATTTAATTTAAATATA
ACAAATTTACCTTAAATTATCTAATATATACTATAAATGGCGTGGGTTCC
ACAAAATAATTATGGTTATAAATACAGAGGGTACGATCTCTCAAAATATA
TAAATCCAAGGGATGCAAATCTCTTTAATTAAACGGTTGAGATGTATCTA
TATCGCTTCTAATAGATCTTAGCCGTCTGCTTCACAAAAAATAATGTATG
GAGCCCACACTGTCCTGATTCATAGTATAGATAATTCATAGGGATCCTTA
TCTTGCTTTCTTTCTTGTAACAACTCTTTGTTACATTGATCTCCTACAAC
TGTACTTGTATGATTGATCTCTATTTAATCATTTATTTTTATCTGTCTCA
AGGTTGTCAAGGTGGCAGATTTTGGTGTTGCTCGGTTCCAAAACCAAAGA
GGTGTAATGACAGCGGAGACCGGTACGTACAGATGGATGGCCCCTGAGGT
GTGACTCTCTCATTTGCCCTATTTCTTTGGCTTTTGTTGGATTTACTTCC
TTATTGTGTTATCTGAACTGGAATTTCAATTCATTAGTTTTTTGTTTTTT
TCTTGATTGGCAATATATTCCATGAGACATGTATTGAGTGAGTTGACGTA

TGTGCTAGCTTTAAATATCATGTACAACAACAACAACCACCAAGCCTTTG
TCTCATTTATGTAGGGTCGGTTATATGAATCCATTTACATCATAGAGATC
GATCAAGGGCCATCCCCTCCGTTAGGTTTAATGTCACCAAATTATTTCTA
ATAGTCTCGTCCAACGTCTTCTTAGATCTCTCTCTCTCTCTAGTAGTCCC
CTGAATTTGAACTAAATCACATTTTTTCACTGGTGCTTCAATGGGTCTTC
TATACACATGCCTAAACCATCGTAAACGATTTTATCTAATTTTCTATTTG
ATGGGTATTATTCCTAAATTTTGTCTAATAGTTTCATTCTTAATTTTATC
TATTCTTGTCTTCCTACTCATCCACCATAATATCCTTATTTATGTCACCC
CAATTTTCTGAACATGATTCGTTTTTACCGCCCAACATTCTGAATCATAA
AGAAGCGTTGGTCTTATAGCAGTTTTGTAGAATTTTCCTTTAAGTTTTAA
CAGAATCCTACGATTACAAATCACCCTAACTGCATTTCTCCACTTTATCC
AACTTGCCTTGATTCTATGCGTAATATCCTTATCGATTTCGCCATTTCTC
TGAATCATTGAACCTAAATATCTAAAACGATCAACTTTCGGCACCTCCTC
GTCTCCAATTTTGACCGTACCATCGTCTCTCCGGGCATTACTAAAATTGC
AATATATGTATTCCGTCTTGCTTCTGCTAACCTTCAAATATTTAGACTCT
AATGCTTCTTTCCATCTTTCTAACTTAATATTAAGACCTATTCTACTTTT
TTCCACTAATATGATGTCATTCGTAAATAACATATTCCAAGGTACTCCAT
CTTGAATATGTTTTATGAGTTCATCCATCACTAGAACGAAGAGGTTCGAA
GAGGTAAGGACTTAAAGATGATCCTTGATATAAGCCTATATTAATAGGAA
ATCCACAAGTATCGCCTCCTCTCGTTCGAACACTAGTGGCCATAGTTGTT
AAGGGCGCGCTTAGGCGCAGCGCAGCGCACCCTCTGTGCTTCGCCTAGGG
TTAGGCGATTGACTTGCCTAAAGTGTTTGCCTTCTTCTCTTGGGTGCTGG
GCGCACCTCACACCTTGGGCGCGCCTTGCATGTGAGTCTTGAGCATCGAA
GACATCGCTGATCTTCAATTCTTGAGCAGAAATCACACATCGTAGGTGAG
TCTTCTCTTTCGAATCTTCGATTCTTGAGTAGAAACACACATCACAAGTG
AGTCATCTTCTCTTTCGGCTTCAAGGCTTCAATTCTTGAGCAGAAATCGC
AGGTGAGTCTCCTTCGACTTCGAATTTCTCACCTTTGGTTCTTTAGCAGA
CGATTTCGCAGGTGAGTTTTCTTCGTGAGCATACCATTGCGAACCTTCTT
CTCCTTCAAATTTCTTCTCTGAATCTTTTCTTCCTCTACCCTGTTTTTCT
CCTTCGAATTTTCTCCATTTCGTCTCAGACAAGCAGGTTAGTGCTTTGTT
TCTATTTCATGTTATTTCTTTACTTATTATTTATTTATTTGTTTAACAGG
AAAGTGCTTCCTTGTTTAACTTATTGTATATGCTGAAAAAAAAAAAACTA
TTTATTTATTTAACAAAATTTACTTTTATTAGGGTAAAGATTTTAATCTA
ATTTTCTATTTTTTTCTACTGAGTTGATGTTGTTAGGGTTCCCAGTTTGT
TTGCTCTTTGTTCATGGATGTTTAAAAAATTTTATTTTGAGTTTTTTGGA
ATGGTTAATTTAGATTATTATTTATTTGTTCAATTGTTTATTTAGTTTAT
TAGGGTTTTAAAAAATTTTATTTTGAGTTTTTTGGAATGGTTAATTTAGA
TTATTATTTATTTGTTCAATTGTTTATTTAGTTTATTAGGGTTTTAAATT
TTAATATAATTTCATTAGTTAATTCTTTTTATTTATTGGTTCACGTGCGA
CTACGTAGTGTGTTTTATTTTTTATAATTAAAATTTAGTTGATAATTTTA
CTTCTTGATTTTAGATTTGCAATACATTAGTGCTATAAACTGTTTTTTAT
TTTTTATTTTAGTATCGTGCGCCTGACTTCGCTCAGGCTAGCGCCGCGCC
TTGCGTCTTACGCCCAGGCTCTAGGACACCTTGTGCGTTTCGATGCGCCT
AGAGCCTTTAACAGCTATGCTAGTGACTATATTTTCATACATATTCTTAA

TTACCTCGATATACACATTGGATACCTGTTTCTTTTCTAGCACCCATCAC
AAAACTTCTCTGAGTACCCTATCATAAACCTTTTCTAGGTCCATAAATAC
CATATGTAAATTTGTCTTCTTCTCTCTATATTTTTCCATTAATCTCCTGT
GTAAATAGATAGTTTCAGTGGTTGATCGGTTGGGTATAAATCCAAATTGG
TTCTCGTCTATACTCGTTTCATGTCTTATTCTATATTCAATCACTATCTT
CCAAAATTTCATAGTATGACTCATAAGTTTATGCCCCGATAGTTCGTGC
AACTTTGAACATCCTATTTATTTTTATAGATGGGCACTAAGATACTTCTT
CTCCAGTCGTCAGGTATTTTTTTAGTTCTCAAAATGTCATTGAAAAGTCT
AGTTAACCATTCTATCCCAATATCGCCCAAAATTATCCACACTTCTATAG
GGATTTCATCTGACTTTACTGTTTTTCCAATTCGCATCTTCCTTAGTGAT
ATTCTAATTTCCGAAGTCCTAATTCTTCTATGAAACCTATAAATCTTATT
TGAATATAAATTAGGCAACTCTAGTTTAGATTTCCCGACGTTATTCTCAT
TAAATAAATTATAAAAATAACTTCTCTACCGTTCCATAATCTCCTCATCT
TTCACTAGTATCTTTTCATCAGTGTCTTTGATACATCTGACACTTTCAAT
ATCTCTAGTCTTTCGTTCACGTCGCCTAGCAAGTTTAAATATATCACTTT
TTTTTTTTATCTAACTTTTGATATAAATTATCATACGCCCTAAATTTTGA
TTCCTTAATTATTTTTTTCGCCTCCATTTTTGTATGTTTATGTTTTTCCA
AACTTTCCCGACTTCGATATTTTTGCCATTCTTTATAAGTTATTTTCTTG
TCCCGAACGGCTTTTTGGACCTCCTTATTCCACCATCAACTTTCTTTATA
TCGTGATCCTCCTCCTCTACAAACTCCTAAAACTTCTCTTCATATATCTC
TAATACGACTCACCATATCAATCCACATCTTGTTCACATCCTCATGTCTC
TTCCATAACTCATCATTGCTAATTTTATCTTTAAACAACTCCATCTTTTC
ACCTATTAGATATCTCTAACGTATCCTTGCATGCATGCCTACTTATGTTT
GACTCCCGTTTCCACCCTTGAAACTTGAGATCATTTACCATTAGTTTATG
TTGGGTAGTAATACTCTCTCCGGGTATGACTTTACAGTTCTTACATATTA
AACAATCACTCGCCCTAGTAAGGACAAAATCTATCTGGCTATTCCTCTGA
CCACTCTTATATGTGATAAAGTGTTCATCCATTTTCCTGAAGCATGTATT
TATTAGAAGAACTAAATCATACACTATAGCAAAATCTAAAATCATCTCTC
CTATTGCATTCCTCTTTCCAAATCCATAACCATCATGTACTTTCTCGAAA
GTTCTCATTTCTTTCCCGACATGTCCATTCAAGTCTCCCCCAATAAATAT
TTTTTCTCGGTTAGGTATACTTTGAACAATTTCGTCCATCTTCTCCCAAA
ATTCTCGTCTAACATTTTCTTACAGTCCAACTTGGGGAGCGTACACACTA
ATAACGTTGATCACCTCCCCTCCAATCACCAGCTTCATAACGATCATCCT
ATCGCCAATTCTCTTAATATCAACAACGTTATCTTTTAAATCCCTATCTG
CCATGATATCCACCCCATTTCTATGTTTGTCCTTCCCCGTAGATCATAAT
TTATATCCCGTACTCTCAATTTCTCTAGACCTCTCTCCAACCCACTTAGT
TTCCTGCAAGCATAAAATATTAATCCTCCTCCTACACATCACATCCACCA
ATTCTCGAGTCTTACCCTATATTCCAAGTGGCCAACCTAAGTCTATTCTT
ATGGACTAACTTCTTTATTCGCACTCGTCCATCGTAATGCGACAACCCTC
GTTCATTTGATGTTTTGTATCCAGGCGAAAATACGGCGTGTCGCTTCCGG
GCGATGCCCTAGCCCACCCTAACCCATTATTCACTACATCCAGGCGGTGG
AAATGTAGCGCGTTGCTAGTAGGGAATGCCCCAACATTTAATATATTTAG
ATTCATGTCATAAAAGTTCTGACAATTTTTCTCTGGTTATCAGCTACCTA
ACGCAACACTCCTCTTTTAACCGGGCTTGGGACCGACTGTGTGTAAGGAA

TAAAGTATCAAACCCCACAGGCGGAGTTGCTTTAAATATCATGTATCACT
TAGAAATGGGTCAAATTTTTAAGTGGTTTCTTCTAGTAGCATGTGTTTCG
CCTTTTTAAGTGGCACCACCTTTTTTGAGGAGTTCTCATATCACGTATCA
CTTGGAAATGGGTCAAATTTTAAGTGATTTCTTTGTTTTTTATTTGGGAC
GTTATATTTGAGTCAAATTTCAAATCTCTAAGATGACATCCATCGCCCTA
ATTAACCCAGCTCTAGCGCAATTAAAGCACCTTACAATATTGGCAGTGCT
TTGGGAGGCATTCATGGGTGATAAGTTAGGATTAAGGCGGGCAATATGGG
TATTGACACAAAAACACTACACGAAATCGGCACAAAATTATCGTGTATGG
GTTTAGCCTTAATATGTTTCGAGTCGTTATTGTGTTAGACAAGATAAACA
CAAAAATAAACAAGTCGTGTGTCAGGTTTACCTGTTGATCTAATATTTGA
CATGAAATGGCAGGATACTTTTGAATCGTATTTTGGGTTACCCATTAATA
GATGACGTAATAAATCACATGAAATTAATCAATTCTTTAGATATTTTAAT
GAATTATCTTATAAATATGTATATTTAATAATTAAATTAATGGGTAATGG
GTTTGTTAAAGTAAGAAGATGATACAAAAAGAATGAGAAAATTTAACAAT
TGTATTAGCCTGTCCCTTTATTTATAATATAAAGTAGGAAATAATACAGG
AATGACATAAATATTTTATAACATTCCCCCTCAAGCTGGAGTATATATAT
TGTATACTCCTATCTTGTTACAAATGTACTTTATCTTTGAATTCCTTTAC
GGTTTAGTAAATAATTAGCTAATTGATCAACTAAATTTACATGTATGGTC
TCAATCAACTTTTGCAATAATTTTTCTCGAATGAAGTGACAATTAATTTC
AATGTGTTTTGTTCTCTTATGGAAGACTGAGTTGGATGCAATGTGAAATG
TTTGATTATCACATAGCAGGACCGTTAGTGAAGAGTGATCAAATTCATGT
TCTTTCAATAAATGTTTCAACCATGTTACCTCACAAGTTGTATGGGTCAT
CGCCCTGTATTTCGATTCTACACTTGACCGGGCCACTACCATTTGTTTTT
TACTCTTCCAAGAGACAAGATTATCTCCAACAAATACTCAATACCCTGTA
GTAGACTTCCTATCAGATGGTGATTTATCCTAGTCTGCATTTGTATAGCC
TTTTATTTGAGTGTTAGCAAACTTATGATATAGCAACTCAAACCAGACAC
ACCTTTTAGATATCGAAGAATACAAATCATTACATCCCAGTGACAAGTCG
TGGGACGATCCATGAACTAACTGATAACCCTAACAACAAATGAGATATCA
TGTCTAGTAACCGTCAAGTAATTAAACTTCCGTACTAACTGTTTGTATTG
CACTGAATCTGCTAGTGGTGTACCTTTATCTGGCAACAATTTGGTGTTTG
GATCCATAGGTGGGTCAATCGTTTTAGCTCCTAGCATTTTTGTTTCACTC
AACAAATCAAAGACATATTCCCTCTAAGACAGGTAAATGCCTTGACTCGA
TCGAGCAATTTCAATCCCCAATATATACTTCAACTAACCCAAATCTTTTG
TTTGAAATGGTATGCAAAAACTTTTTGAGTTCCTGAATGCCACGGTAATT
GTCTCTTGTAATCGTGATGTCATCCAAATACACAATCAATAATATTCTCC
CAACTTTAGTGTGCTTACAGAACATGGAATGATCGACTCCACAAAGCCAA
AAGCCAAATTCAGTGACCACTTCACTGAATCTCCCAAACCATGCTCGAGG
AGACTGCTTGACGTTATAAATAGATTTCTTCAAGCAATATAATAAACTAG
ATTGCCCCCGAGTAACAAACCCTGGAGGTTGTTCTATGTATACTTCCTTA
GTCAGATCACCATTTAAGAAGGCATTCTTGATGTTGAGTTGATGTAACGG
GCAACGAAAGGTGGTAGCTAGAGAGATGAACAAGCAAACATAAGCGAGCT
TGACAATGGGAGAAAAACTCTTAAGAGTAATCAAGACCGTATACATGAGT
ATAACCTTTGACAAATAAGTGAGCCTTGAGTTGATCAGTGGTGCCATTCG
GATGAACTTCGACAATATATACGCATTTACACACAACTATTGTTTTGTCA

GGCGAAAGCGAGACAAGATCCCAAGTATCGTTCTAATATAAGGCTAGTAT
TTCCTTTTCCATTGCGGTTGTCTAACAAGGGTGAGTGAGAGCCTTTGAGA
CAAACTTAGGAATAGAAACAGAGGACATAGAAGCAACAAAAAAAGATTAT
GAAGGAGATAGGTGTGTATACGAGACAAAGTTAGAAATAGGATGGATGGT
CAGTACACATGTGTTTACCTCCGGACAACAATGGGTATGTCTAAGCTAGA
AGTGGATGGATGAACTGGAGAAGAATCTGAGGACAGGGGGACGATGGGTG
CGACGACAGAAGCTACATTCTGACGACGAGAATATACCTACAAAAGGGGT
GAAAACGACTCGGTGGTACAAGTTCTAGAGCGGAGAGAGATAGTGTAGGG
TTTGGGAGGCTTGGAAAGGAAGTGTAACATCAAAATCATTATGAGAGGAT
GGAGCAGAAAAATAAGGGGTGAACTTAAAGAAGGTAACATCAACACACGT
AAATGAGTGACGTAGAGATAGGTTATAACAACGATAGCCCTTTTGTGTGT
GAAAATAACCAAGGGAGAAACATTTTAAGGCATAACGATCTAACTTATCC
TTACCTGGAGTTAACTAATGAACAAAATCGACATTGACAAAGATACGTGG
AGGGGGGGGGGGAACCAAAAAGGGGTGAAAACGACTAGGCGGGACAAGTT
CTGGAGTGGAGGGAGATGGTGTAGGGTTTAGGAGGCTTGGCAAAGGGAGT
GTAACATCAAGATCACTCTGAGAGGATGGAGCAAAAAAGTAAAGGGTGGA
TTTAAAGAAGGTAACATCAGCACACATAAATGAGTGACGTAAAGATAGGT
TATAACAATGATAACCCTTTTGTGTGCGAGAATAACTAAGGGAGAAACAT
TTTAAGGCACAGATATCTAACTTATCCTTACATGGAGTCAACTAATGAAC
AAGACATTGGCAAAGATACGTGGAGGAGGGGGGGGGAACCAAACAAGAGG
GAACGTGNNNNNNNNNNNNNNNNNNNNNNNNNNNNNNNNNNNNNAAACCAA
ACAAGAGGGAACATGAAAAAAGAATGAATGGGCAACTTTGCTATGTAAGG
TAGAGAAGACGACACCCTGTTGATCAAGAAGTAACCGATAAGAACTGCAT
TACCCCCAAGTATTTAGGAACATTCATCTAAAAAAGTAGAGTACGAGCCA
CTTCCAAGAGATGTCGATTCCTATGCTCAGCTACTCTATTTTACTGTGGT
GTGTGAGGACAAGAAGGTGGATGCAAGATGTCTGATTGTGACATAAAAGA
AGTAAAGGGTTGATAAAAATATTCCTGGGCTTTATCACTTCGAAGTATAC
ATACCAATACATTAAATTGAGTTCTAATTTCAATACAAAAGTCACAAAAA
ATGAAGAATAGTTCAGAATGATCTTTCATTAAATAAAACCATGTAACTCG
TGAAAAATCATCCACAAAAGTGACAAAGTAATGAAGACCCTATGTAAAGC
AACATGAGAAAGACCCCAAACATCTGTATGAACTAAAGTAAAAGGACTCG
ATACTCGAACATAAAGCCTGGGAAGCAAATGAACTACGATGATACTCTCC
CAACTGACAAGACTCACAGTCTAAGGAAGATAAACAACGTAAATCATAAA
CCAATAACTTGAGGTTGGCAAGACAGAGATGACCTAGGCGACAATGAATC
TGGTGGGCCGATGCAAAAGTAGGACATGCAGTAGGTGGGACAGTAGGGCT
CTTGTCGGGATTATCAAAGTTGTAAAGACCATGTGCCTTACGCTCTGTGC
CAATCAACCGCATTCGCAAATCCTGGATAATGATAAAATCAGGGAAAAAA
GAAAAAGAACAATTAATATGTTTAGTAAGCTTACTAACAGACATCAAATT
TAAAGAAAATGAAAGAATATGCAAGATAGTGGATAGAGAGAAGGAGGGTG
AGGGTTCCACAGTCCGATGCCACTGATAGGGACAGTGTAACCATTGGCAA
CAGTAATAGGGACAATAAAGGAGATTGTTGAAATATTATATTTATGCGTT
AGTTTTTATTTTTGGTATCTTTATTGGCCCATTTATTTCGGTGATATTCT
TCCTAATTTAGCTTACCCCTGTGGTTATATAATTAGGGGAATAAAACTCA
TTCTGTATGTACAAAAATATTAATTATAATTTTTCTCTTTTTCTTATATC

TTTTTTGGTATTTAGAGTCAAGTTTTTTTATCCCACCCAAACCCTAAAAG
CCGGCACCACCTCTACCAATCCGCCAGCCGCTGCCACTGCCGCCACCAAC
CGCGAATCCCTAGCCCAGCTCGCCATCGCCAGACCATCATCAAGTCGCCT
GCCGCGAACCAGATCAACCCTGCTTTACAAATGCCGATTATGTAGGACCG
AAGGATGATAGATGTTTGACCTCTAGTCTGTGCGCCTTTCATGGAAAACA
CCTAATCTTGTGGAAGAGTAAGAAGCAATCCGTAGTTTCTCGATCATTTG
CTGAAGCTGAGTATCACACCATGACTTAGGGAATGTGTTAACTCCTTTGG
CTTTGTTCCCTTTTGACCGAGCTGGGACTTCCCATGAAAGACTCATGTAA
GTTGTTCTGTGACAATAAGTCCACCATAATGTTATCCTCAGACTCAATCC
TCCATGAAAGGATGAAACAAATTGAGGTAGATATCTATTTCATTCATGAG
AAAGTCTGATCAGGAATCATCACACCTACTTTTGTCCCATATTCCGAACA
GTCTGCTGATATGTTTACAAAGTTCATATGACCATCTCTTCTTCAGTCAG
CTATTGACAAAGTGAGGGCTCATCAACATTTTCGCCCCAACTTGAGGGGA
ATTGTTGAAATATTATATTTATACGCTAAGTTTTATTTTTAGTATCTTTA
TTGTAGGGTTTTATTAACCCATTTATTTAGGAGAATAGAACTCATTCTGT
ATGTACAAAAATATCAATTATATTTTTCTTGTATCCTTTTGAGATGACTG
AATATATGTAAATAGGGAAGAGGTACTTGACATGTGATCAGTAGCAGCAA
AATCTATAACCCATGGTATGGGAGGGGAAGCGAGGATCGAAAGACAAACA
ATAGAAGTACCTTGTTGAGCTAAAAGAAGCAATAGGGAGAGATGCCTTGT
ATTGAAAGAATTGGGCATATTTTTCATCAAATATGTCCTTGGCCGCCTAA
GACTTTGGAATTAAAGCAGGATCATTTGGATAAGATGTCACTGCGTTTGG
GTGGATGACCAAGTCCCAACAGGTTTCTCTGGTATGAATCTCCTTGCCAC
AAGAGTACTGACAATGTGGGCCACGACCATGACCACTGCGCCCACGAGAG
GAACCACCACCACGATCTCGTGGAGGGATCTGTGTAACAAGAGCCAAATG
CTTAGACGGCATAGATGGACTGGAGCTAGAAACACTTTCTCGGTTAGTGC
TACGCAAAACTTGTGAATGAGTTTCAGTGATAGAGGGAAGAGTAGCACTA
ACAAGAATTTGAGATTGGACTGGCTCTAATTTTGGGCGGAATGTGGCTAC
CAATTTCATGACGGCTACCTATTCACGTTGCTTGTGTATTTCTTTAATAT
TGGTGGTCATAGGTAGAACAATGATTAGCTACTCATGAATTCTGTTAAAT
GTGGCAAAATGATCCGTAACAAATTACGTGGCTTTTTGAAGTTAGAAGTA
CTCGACCAATAAGTCATATATCTGGGTAAGATTACTTGAATACAATACAC
GAAGATGTTCTCATTTGCACTTATCCAAATGAGGACACTTTAGCAAATTG
AGGCTCCATCAAATTCCACAACATCGATGCAATCTGCGCATCAACTTGAA
TTCATTCCTCATACTTATTATCATCTTTTGCTGGGGCAGAAGATGGTGTT
TTCTTTGTCCAGTAAGGGTTACTTTCACAACTTTGGACCAATTTTGATGG
TCTACATTAACTTGCGAGTTATTAACGATTGGGGTGAAACTGCCAGCATT
AGTGCTTGCCACTACGACACCTTACTCTATTTATTAAAAAAAAAATGGAC
AGGGGAAACGACCAAACAAACAAGAACAAGAGTAAGTTTTAAACAAGTGG
CATTTTTTTTTTTTTTTCATTGAAATACTAAATCAGTGTGTTAAAAAAAT
AGAATATTTGAAAAAAGAGAGTCTCACAAGAGAGCTATCTGGACAATGAC
GACGATGCGAAGGCCATGTCGGCGACGCAATGAATCTAGATCTGGCCTGG
ATCTGGTTCTAGGGTTGCGGTTACAAACCACATATTTTTTTTTCCTTTTT
TACGGAAGGAAGGAAAAAAAATGAGAAGAGAAGAATAAAACTAAAATAAT
GGTTTGAACAAATCTTGATACCATGTTAAAAGTAAAAAGAAAATACAAAA

AGAATGAGAAGGTTTAACAATTCTGTTAGTCTCTCTCTCTATTTATAATA
GAAAGTAGGAAATAATATTGAAACCCCAAATAATATAGGAAACCATAAAT
AATATAAGAATGACATAAATACCCTTTAACAGGGTTAAATAGGTTTTGGG
TCACACAATTAATTATTGTGTTGTGTTATTATACCGCATTATTATTGGGC
ACGATTAATTATCCTGTTGTGTCGACATGATAAGAATTCATGTGTCGTGT
TGTGTTTCAATATTTTGACACAATATGTTATCGGGTCGTGTCACTAATAT
GGTTTGATTTCTGCTACTCTATCCATATGTTCTATGACATAACATCTGTT
ATTTTATATTGGTTTCTTCATACGTTTTTCACAGGTTATAAACCATCAGC
CTTATGATCAGAAAGCAGACGTTTTCAGCTTTGCAATTGTGCTGTGGGAG
CTTGTGACGGCCAAGGTACTTTTTCAAACTTCGATTGATATGGCTATTTA
AAGTAGTTATATTTTCAGTTTCTGGGTGGGAAAGATCTTTATTCATTTCT
CTCCTTGCGGCTCTCAGGTTCCATATGACGGCATGACCCCTTTACAAGCT
GCACTGGGAGTTAGACAGGTTTGTACTTCTCTATAAAACGTGAAATAATA
TCAGGCACTCCTAGCGAGTCTCAGTGCCTAGATACACTAAATTGATTTAT
TTAATTATAAGTATAAAAAAATAATAAATCATAAAAAAAATCGGCAAATTA
GATAAAAATGTGAAACTTAAAACAATACAAAATATAATTTAATTTAACCG
TCTTATATGAAATTTTTGGTCGGGAATTTTCCCACTAAAAAATTATATAG
TCCAATCTCATCCATCCATTCTTCACAAATCAATCTCAACTCTTGACAGA
AAGACATTAAAAAAAAAAAAAAAGTCCTAATTTATAATATAGATTTTTGAA
GTGATTTAATCATTAATACCAAAAGCTTGACCATTACATCAAGGCTAATC
AAGGTTTTCACAATTTAATTATCTATTGGCATATGGATTGGGTAGGCTAA
TCATATCAAGGCTTTTTGAGAGCTTTGAGTTTTCCTTTACTTTCCTATTC
TCCTTTTTATTTGACCAATGAAAAATAATAATAAATTTAAGCGAAAAAGT
TTTTTTTTTTTTTGAATTTAACATATTGTCCACAATGCAGAAGGCAAATA
CAGCTTTTAAATTTTCTTTTGTGTTCTACATGGTAGGGCCTTCGACCTGA
TCTTCCCACGAACACAAACCCTAAATTGTTAGACTTGATGCAGAGATGCT
GGGAAGCCACGCCTGTCAACCGGCCATCTTTTTCTGAGATAAGAATCGAA
CTTGAAGAACTACTCGAAGTTCAGGTAAAATCCATGCACATTGTTATTAA
ATTGGGAAAATTATTATATATGATCTTTAAAATAATTCACTCATCATTTT
TTGTTTTTTTCCCCTTTATTTTTTAGTTTCGCACATGATTATCTTATCAT
CTAGCCTCCTTTTTTTTTAAAAAAAAAAAAATTATATTTTTTAAAGATAATA
CAATAATTTTTCCTATAAATTTCCTCTTTAATTTGATATATGTTGGCACT
GAGATATATTCCACTTCCCACAGGATTCCTCAGAACAAGCAAATGGTAGC
TGA
>EUC25435-RA [gene]
ATGCAGCAAGATCAGCGAAAAAAGGTTTCTCCCTCTCTCTTTTGCTCACC
TGTAGATTTCACATATTTGAAATTAAAACGAGATCTTGACTATATATAGA
CATACACATGTATTCTGGTATTATCTCTTCCAAAATCATTTGATTTTTTT
TTGTTTTTAACTTAACTGTTTTGTTTTTACAAAGGACGAGATCTTGCTTG
CGTATTGAACTTCATATCTATTCGTAAACAAACTAGTAAATGTTTGAGCA
TTTCAATTTCGCTGCTGGTAAATTCCTTCTATTTGAAATTATGATTCTCT
CTCTCTTTTGATTGACATGAATGAAAGCCTGATTTGATGACTTGATTTTA
TGCATCTATCTGTTTTTTCTGTTGAATCCTGGAACTAGGAATTATAGTCT
CATAAAAAAATGCTTTGATCATCTTTGATGTCAAACAGAAGGGGTGTATGT

TTAGCAGGAGCACAAGGTTTTGTTTGATTTTTGCTCCTGTAGGTTGTTGA
GCATTTGTTGTGTTCTTCTCCAAGGAAACTTGCTAGTTTTACATTTTGTG
TTCTATGGATTAGTTTTATTTTTCTTAGTTATTTTCTCCACCTCTCTCTT
TTTCAGTGTCATGATAGTTAGTAAATTGTTGTTGTTCCTGCACTGACCAT
TTTGTGATCGTTTTCAGAGCTCAAAAGAAATTGAATTTTTCACCGAGTAT
GGTGATGCAAATCGATACAAAATCCTTGAAGTCATAGGGAAGGGAAGCTA
TGGAGTTGTTTGCGCAGCCATTGACACTCATACTGGGGAAAAAGTGGCTA
TCAAGAAAATAACTGACATATTTGAACATATATCTGATGCTATTCGAATC
TTGCGCGAGGTCAAGTTGCTAAGACTGCTGCGCCATCCTGATATTGTTGA
AATTAAACGCATTATGCTGCCACCCTCAAGGCGGGAGTTCAAAGACATAT
ATGTTGTTTTTGAGCTCATGGAGTCTGACCTTCACCAAGTCATCAAAGCC
AATGATGACTTGACGCATGAGCATCATCGGTTTTTCCTTTATCAGATGCT
ACGTGCATTAAAATATATGCATACTGGTAAGACTTTAGGAATCCTTCTGG
TAATAATTGTTGATCATTTTAGCTGGTAGGACCTTAGCATTTGTTATTCA
TTTGGGGAGATTTTAAACGAACTCACTTGTCATAATTTTTGGAAACTTCA
TATTTAACCTTTGTTATCTTTGGCCTTTGTCTTTGACTTTTCTTTGTTCT
TTTCATTTGTTCAATATCATTTGTGATGTGTGATTTGGATATCTACTAAC
TTAAAGCTCTTTGGTTCCAGCAAATGTTTACCATCGAGATCTTAAACCAA
AGAATATATTGGCAAATGCAAATTGTAAACTGAAAATATGCGACTTCGGA
CTCGCAAGAGTTGCATTCAGTGATACACCAACGACCATATTTTGGACGGT
ACGTTTTTCTTTTTTGAGTTACTGTGAACTCCGTGTAATGGCATGTACTG
TAATTGATTTTAGTGCGATGGGCATTGCTTTCATATAGCATATCATTCTA
TTATCACGGTCTTCATCACTGTATATGAATTCTAAACTAACATTGTTCAT
ATGTTACTTTTGATAAACGCCATACAGGATTATGTTGCTACGAGGTGGTA
TAGAGCTCCTGAGCTTTGTGGATCATTTTTCTCTAAGGTATAGTTTGTCT
CAACAAGTGCTGCCTTTTTATATATTTTTAAAGTAACTAGAATGTGCTTG
GGGGATGATAATTGATCCCTAAGTTGAGTTGCATTTTCTTCATTGTTAGT
TCAAGAAATGTATAGTTTTTTGTAATAAAGATGGAACTGTTTTCAAATTG
AAGTTCAGTTTTGAATTTTCATCTTCATTGTCGGTTCATGAAATGTATAG
CTGATGGTTACAACCCCCCTTTTTTTGGGTGACGAACTCTAATTTTTTGTC
TGGGTAAATTGGAAGGCATATTTTATTAATGCTGCTTAGAGAAAAGGGAA
AAAAATATACACATTAGCAACCTGAATATTTGTATGAAATCAACTCATTG
TAGGGCAATTTCCACCTTCGGATGGGATGGGATGGGATTGGGGTATTCTC
TTAGGTTTACCTATTTCTCTAGCGGGATTCGTACAAGAGAATTTTTCATT
TGTATGTTTGTCAGTTTACGGACTAAAAAACTGCATCCGAGAAACAATTG
ACAAAAATAAATATTGGTTTATGAGATTTGACTTGGAAGCAGACGGTGAA
TTTCTTTCTATATAGAATGTATTCCAAGTGTCTTAATGTAAGTGGTAATG
CCTAGATTAAAATAATTCTTGTTCAGGGAGGACAATTGAACTCTCCTGGA
AGTTGAAATTTCTGTTTTTCAGGTAACTGTGTGGGGTCTTTTGTCATCTT
TTCTTGGTGCTTAATTATTTTTTTTATGTTTCTCATTCTCGTAAATTGAT
TTTGACATGTTTTTGCAGTATACGCCTGCTATTGATATTTGGGGTATTGG
CTGTATCTTCGCCGAGGTATTGACGGGGAGGCCATTGTTTCCCGGTAAAA
GTGTTGTTCATCAGTTAGATTTGATAACCGATCTTCTTGGCACACCTGCA
GCAGATACCATTTCGGGAGTATGTAACATTTACTTTTTTTCAGTAGCATT

TGCTAAACATTAAGGGCCCGTTTGGTTGTCTAATATCCTTATTTATGAAT
TATCTTACTTTTGATGAATTGATTTGTATATTTTTATATTTACCAGTGAT
TTAGTCAAATTTACATATTATTTGTTTGAACAAAAAACTTAGTTTTTACT
CCAAGAACAGGAGAAAATAAACTTGGAACTAGAACTAAACTGATTAGCTT
GCCATTTTTAGGTGTTTTAGATTCTTACGCCAAACAGTATTACTATAACA
CTTAATAAATAAACGAAATAAAAAATAGTACAATTTGCAAATTGAAGGAC
TACAAAACAGGCCCTAATTCCATACTTTTGTCCCCTTGCTGTCCCTTCTA
TTTTCTTTTAACAGTTTAGTCTGATAATCCCTGCAGGTTAGAAATGAGAA
GGCTAGAAAATACTTGACAAACATGCGGAAAAAGTACTCTGTGCCGTTCT
CTGAGAAATTTCCAAATGCAGATCCTCTAGCACTCCCTCTTTTGCGAAGG
CTTCTAGCTTTTGATCCAAAAGATCGACCAACAGCTGAGGAGGTATCTTT
TTGTCTAGTTTCTTATAGTCTTAACATTATTATGCTTCTTCTTATTGGGT
AAATTGTCTTTTACAATGATTTGACTTCAACCTTTTTTGTATATATGCTT
TTATTAGGCACTTGCAGATCCGTACTTCAAGGGCCTGGCCAAAGTTGAGA
GGGAACCATCTAGTCAGCCGATCTCAAAGTTGGAGTTTGAATTTGAGAGG
CGTAAGGTGACGAAGGAGGACATTAGGGAACTTATTTTCCAGGAAATTCT
TGAATACCATCCACAATTGCTCAAGGATTACATGGCGGGGAATGAAGGCA
CTCATTTTCTCTATCCTAGGTTTATGGAGCACCATGCACTTCATTTGTTT
GTCGTTTTATTTTTTATAATTTGTATTTGATGATTAAAGTTTAAATCAAT
TGTTTGCAGTGCCATTGGTCAGTTTAGGAGGCAGTTTGCATATCTAGAGG
AAAATTTTGGTAAAAGTGGACCTGTTATTCCTCCGGAGAGGAAGCATGTT
TCACTTCCGAGGTAAGGCTCCTTGAGTGTTCAACCAAATGCCAATAAAGC
AAATCATAAATTGACTGCTTGAAAATTTGCCCAATACTCAAATTGAAATA
CAGAAGGAGAGGCTTAGCGCAACGGTAGAAGTGGTCGCCATGTGACCTGG
AGGTCACGGGTTCGAGTCGTGAAAACAGCCTTACTTTGCAAGATAAGGTT
GCGTACAATAGACCCAAGTGGTTAGACCCTTCCCCGGACTCCACGCATAG
TAGGAGCTCCGTAGCACCGGGCTGCCCTACTCAAATTGAAAGACAAATAA
ACAAGCCCTCAAGATTTACCAAGTATTCCTGTATTTTAAGGCACCAAATG
CCTAGCAGTGTGTGATTTGTCATTGCATTCTGTGTGTAATAAATGGCTTC
AATTTTTTTGAACTCAGTGCAGTACTTTCTTTGTTCTCATAATTTTAATT
CTTGGTTTTTGCTTTCAGGTCTACTGTAAACTCGAGTGTGATCCCTCCTA
AAATGCAGCAAAATTCGGGTGCATTTGATAACAGGCGAACTTCAGAAGAA
TCATGTAGTAGTAATGGTGTCCGAGTTACGGATGCCATCAACACAAATGC
AATGAAGGCCACACGACCACCGCCACCTCGGGTGCCCACAGGTATCAGGT
TCTGTTAGGGTGTGTTAGGGTAAGGTATTTGAGAAATGCATTTGATTTCA
AGAAGGGATTAGTAATTAACGAAAATTCTTAAAGGTGTGTTTGGATGAAA
GATTTTGGGGGAGGATTTGATTTTAATTCCACAAATTTATGAAATTATTT
ATCTAAATGAAACCTGATGAAATTTTACAAATCGCCTCTCGAAATCAAAT
ACATTCTTTTTAATATCTCTTTCAGATTACTACCTTTCTGTTTGTTTTTA
GTCATTTTTATGACCCTTTTCTGTTTGTTGCTATTCAGCCAAACCTGGAA
GAGTGGTGGGGCCGGTTCTACCGTACGAAAGTGGCAGGAACAATCCTAGG
GTTTTTGTTCGTAATGCGGGCCCACCTCCTCCGCCTCAGACAATATCTCC
ACATTATTGCTTCAGGTCTAATAATCAAGAGAAGTCATCATCAGGGCAAG
AGGTGAAACATCAGTCAATTTCGGGCAAACCGGGTCCCGGGATGGGGATG

GAAATGAACACCACCCCTTATTACCAGACGCAGACGAAGCCATCGCAATT
GAACAATCAAATTGCGCTGAATGCCAAACTGTTTCAGGCACAGTCTCAGT
TTGGGGCAGTTGGAGCGGCAGCTGTTGCTGTTGCCGCGCATAGAGAGGCT
GGTGTTGTTCAGTTTGGCCTCTGA
>EUC00773-RA [gene]
ATGGAGTGGGTGCGAGGCGAAGTTATTGGTCATGGAAACTTTGGCACAGT
CAATTTGGCGATACCCAGAAGCCAGAATCCTCAAATTCCGCCATTGATGG
CCGTCAAGTCTTGCTCTGTTTCGAACTCTGCTTCGCTCGTCAACGAGAAG
TCGGTGTTGGATGCGCTGAATGGTTGTTCTGAGATTGTTGGTTGCTTCGG
CGATGGTTTGAGCTTCGAAAATGGAGAGAAATTGTATAATGTTTTCTTGG
AGTACGCTTCTGGAGGGGCTTTGGCTGACTTAATCAAGAATTCCGGCAAT
ATCCCGTTGCCGGAAAAAGATGCCCGGAGTTACACGCGGTCAATTTTAAG
GGGAATTCACCATGTTCACAAGAGTGGGTATGTTCACTGCGATATTAAGC
TTCAAAACATTCTGTTATTTTCTTCGGAAGTTGGTTGTGACGATCCGGTG
AAGATCGCCGATTTTGGGTTGGCGAAGAAATCCGGCGAAAAACAGAGTAG
TTCAGGGTGCGAATTGAGAGGCACGCCTCTGTACATGGCGCCGGAGACAG
TAACCGGCGGCGAACAGGAGCCGCCGTCGGATATATGGGCTCTCGGATGC
GTGTTGGCGGAGATGGTCACCGGTTCTCCAGCGTGGCGGTGCCGGGAAAA
ATCCGACGTGGCGGGGCTATTGATGAGGATTGGCGTTGGCGAGGAGTTGC
CAGAGATTCCTGGAAAATTATCGGCGGAAGGAAAAGATTTTCTAGGAAAG
TGCTTTGTGAAAGACCCGAGAAAAAGATGGACGGCTGAGATGCTTCTGAA
TCATCCTTTCGTCGCGAATTCCGATGACGATCGTGAAGCTGTCTCGTCGA
AGGACGCCCAAGAAATCCTTTGGACATCTCCGAGATGTCCCTTCGATTTT
CCCGGTTGGGTCTCCCCCGTGCAGTCGCCGGCCACATCTTTATTTTCTTC
TTCGCCCTCGTCTCTCTCCGACCCATGGTCGGATTCATTTTCCTGGTCGC
CGTCGAATTCTCCGGCAGAAAAGCTACGGCAGCTGACGACGGAGCTGAAA
CCAGATTGGTCCTTTTCGGAAAGCTGGATCACCGTCAGATAA
>EUC17818-RA [gene]
ATGCCTCCATGGTGGAAAGCATCAACAAAAGAAACAAAAAAGAAAACAGG
CAAGGAGAGTTTCATCGACACATTACACCGTAAATTTAAAATTCCATCCG
AAAGTAAGTCCACAAGTAGATCAGGAGGGTCTCAAAGACTTGGCAGGGAC
ACAATTTCAGAAAGGGGGTCTCAATCGCGACCACAGTCAAGGTCACCGTC
ACCTTCTAAACACGTATCGAGATGTCAAAGTTTTGCTGAAAGGCCCAAGG
CACAACCACTCCCACTTCCAGGTCTGCGTCCTGCAGGTGTAGTCCGTACG
GATTCTGGTATAAGCGTATCGGCCAAGCAAAGGCAGGAAAGAGGGTCTAG
GCCATCATTATTTCGTCCTCTTCCAAGACCTGCATGCATCCGAAACAAGC
CAGATCCCACTGATTTGGATGGAGATTTGGTCACTGCTTCTTTTTCCAGT
GAAGGCTCGATTGAGATTGATGATCCAGCTGACTCACGTCAACGTAGTCC
CCTGGTGACAGACTCTGCTACTGGGAACAGAACTACCACATGCAGCCCTT
CCAGGTGAGCTTGCTTACTCTTATCCATACTTTCCTCGTCTATGAACAAC
CCCCCCCCCAACCCAACCCAAAAATAAAAATAAAAAATAATAAATGAAAA
GAAAAAAAGAAAGAATAATAAGCGAGACTTTGACGGCACATTTGGCGGGG
CACGCTTGGGAGGAAGTAATCGAAGCAGCTTCGTGTTGGATGGACACAAA
ATTTGAATGAGCTCCTATTCTAAATTTTATTGCATTCCATAATAATCTTA

TTCCATTATAATGGTATTCCATTATTGCCTTCCGGACATGCCCTTAAGGG
TTCCCCTTTCCCTAACAAATGATAATTGTTGAATTCAATTACATGTGACT
CATTCAAGTCATTTGTCATGTTGCATGTTTATTTTATTTTTCTAGCAATT
TTCTTCATTCCTAATTTATTTACCTCATCTTTTATGAAGGATGACGGTTA
AGGATCAGACCCCTGTTGTGCAGACGAACTCAGTGGAGGCATTAAGACCT
GCGAATCCTTTGTTCAATAACCACATCTCTCCTTCGTCTCCTAAAAGGAG
ACCTCTGAATGGTCACATGACAAATCTGCAGGTTCCTTGTCATGCGGCTT
TCTCAAGTGCTCCAGACAGCTCAATGTCAAGTCCTTCGAGAAGTCCCATG
AGAGCATTTGGCACTGATAAAGTCACAAATTCCACTTTCTATGCTGGAAA
GCCTTATCCAGATCTTCCTTTACTTGGATCTGGACAATGCTCCAGCCCAG
GGTCAGGTCAAAATTCTGGGCATAATTCAATGGGAGGCGATATGTCAGGC
CCATTATTTTGGCAGCCCAGTAGGGGTAGCCCAGAGTACTCTCCAATTCC
TAGCCCAAGAATGACTAGCCCTGGACCTAGCTCCAGGATTCAAAGTGGTG
CTGTCACACCACTTCATCCCAGAGCTGGAGGGGCAGCCTCTGAATCACAG
ACTAGCTGGCCTGATGATGGAAAACAACAAAGTCACAGGTTGCCCCTTCC
TCCTGTAACAGTTCCCCATTCTTCACATTTCTCTCACCCAAATTCAGCTG
CAACATCTCCGTCTCTACCACGGAGTCCAGGAAGGGCTGAGAACGTTACA
AGCCCTGGTTCACGCTGGAAAAAGGGAAAGCTTCTTGGCAGGGGCACATT
TGGACATGTTTATGTTGGTTTTAACAGGTGCATCCAGATGATAAATCTCA
GATTTATGTAGTTCTTTCTTTCATTCCTTTCCCCCTTTTTTCTTTATATA
TTTCTCATTCATTAGCTTCACCATTTCCCATTGGGTTTTAACTCTTGTAC
TTGCATATGATACCAAACAATTTTTCTCAATCATTAGCTAAAGGTTTCTT
TTAGGTTTTTAGGATGCTAGCGTAACTGATTAGAAGTGCTTAAATTTTTA
GGGTTTGTTGTTTCTTAAAATGTTGGTCATTGGTTTATATGTTATAATCT
TAAGTGTTGGTCATTGATTTTAGGTGTTGGTTAATGATTTTTTATCGTTT
ACAGCATTAAATGTTTATAACTGAGTGAGTTTTTGTGTGTTCCTTATTTC
CTCGATACAGTGAAAGCGGTGAAATGTGTGCAATGAAGGAGGTTACATTA
TTCTCAGATGATGCTAAGTCTAAGGAAAGTGCAAAGCAGTTGGCACAAGT
AAGATTATTAGCTAGAGAGGGTGTAGTTTATTATTTCTTTTTATTGCCTT
CCATTAATATCACTTAATATGTAGCTCCAGGTGGAGGTTCTTATCCTTAA
AATTTGAACAGGAAATTGCTTTGCTGAGCCGCTTGCGGCATCCAAATATC
GTTCAGTACATTGGGTCTGAAACGGTATGTTTCATGCCTGCTTGTTTCTT
TTCTTTTCCTTTTTCTTCCCCCCCCTGTTTTTCCATGTTGACTAATTAAG
ATGTTTCCGGACATATTTCTCATTTCTATGTGTTTTTTAATGTTCTGAAA
TAGCTACTTCATCCAGTGAGGCTTAACGCTTAAAATTGTCTTTGCATTTG
ATTCCTAATGTCATAGTTCACAAAAGTTTATCCAATCTAGTACCTTGTTT
GCACATAGATACAGTGTCATATTATTGTCATGCTGTAATGTTCCATCTTA
GATTTTGTTTTTTCCCAATCTCTTCTACCCCATTTTGCAACTTACAGTGA
TCTTTTCTCTAAACTGTATCTGAACTTGTATGTTTGAGTAGGTAGGAGAT
AAACTCTATATATATTTGGAATATGTGTCGGGGGGTTCCATCCATAAGCT
TCTTCAAGAATATGGACAATTTGGTGAATCAGCCATTCGTAGTTATACTC
AACAAATCTTGTCAGGCCTTGCTTATTTACATGCTAAAAATACCGTCCAC
AGGTAAATCGACTTACTTGTATTTGGATTGACTCCCGGGTTTATTCTTCT
CCCAGCCACTTTGTTCTGGATACATATGTTTCTTTTATATAAAACTGCAT

TTTGTTGTAGGGATATTAAAGGAGCAAATATCCTTGTGGATCCAAGCGGT
CGTGTCAAATTGGCAGACTTTGGTATGGCGAAGCATGTAAGTCTTTGGCA
TATTTGAAATGTAACAAGTTAAAAGAAAAANAAAAAAAAAAAAAAAAAAA
AAAAAAGGACCGAAACAGTAGTCAGTAGCCAGACTTGTTGAGAAGTTGTA
GAAATGTAGGAATTGCACATAGAGCAAAAAGCAACCAACAGAATTTCTAT
TGCAAAAAGTGACAACCCAAACCCTGTAAGTTTTGGTAATATCCAATTAG
AAAAGTTGATTGCCTTGAGATTAATGTTCTTAAGCATATTCTCTTAATCT
GGGATTAGAAAAACAGAGAAAATTAGTACATATGATGCAAATTTTCTTGA
GAATCTGTGACATCTTCTTATTGGGTGTCTTGATATCATTTTAATTTTGC
AGATCACAGGGCAGTCCTGTCCATTATCATTCAAGGGAAGCCCTTATTGG
ATGGCACCTGAGGTAACTGTATCATCTTATTGCTTTAATCTTTTGCTTGC
ACTCTCACGACTAGTACATTTCTTATTTCTCTCTTCCACTGTTTATTCAG
GTTATAAAAAATTCAAATGGCTCTAACCTTGCTGTTGATGTATGGAGCCT
TGGCTGCACCGTTTTGGAGATGGCTACATCAAAACCACCTTGGAGCCAGT
ATGAAGGGGTCAGTAATTTTGGCATTGATACTTTCACATGTTTTTATCTT
TTCATTTGATTTAAATTCCTTGCATGTTGAGACCTTTGCATTTGGGTGAG
TTATTTGAAGAGGGAATTTGATTTTGAAAAGGAATTTTTTAACAAAATTT
TACAATTCCATTTTTCCCAAATCAAATTCCTTATTCAAATGCCTTACCCC
AAACATACCCTTAATGTTAGAAAGCTATTTTCTTTGTCGTCTTATGATGA
TATATTTGCTTGTTCTGGTTTACAGGTTGCTGCCATGTTCAAGATTGGCA
ACAGCAAAGAATTGCCTGCAATTCCTGATAACCTCTCAGACGATGGAAAA
GATTTTGTGAGGCAGTGTTTGCAGCGCAATCCACTGCATCGTCCAACGGC
TGCTCAGCTTTTGGAGCACCCTTTTGTTAAAAGTGCTGCACCTTTGGAAA
AACCAGTTGTTGGTTCTGAGCCTTCGGATCTTCCTGGTGTTTCAAATGCA
GTGAAATCTGTGGTATGTTTTTTTATCAGTCTGTTTTTTTACCTCTATTG
AATTGAAATTAAAGCCATTTATACTTCTTGATTCTCTGTTTCCGTCTCTC
TCCAATTTAGGGGAGATATTTGATTGTCCTTAAAATAAGTCACTTATAAT
TTTTTGTCCTCATTTTTTCTCTTTAGTTTTTAGTTCCTCAAATAAATTTG
TAATTCTTTAATTGATATCAACTAGATTGAATCTATTGAGTTGATATCAA
CTAGATATTGAATTTGTATTTGATATCTTAGTTAAAGTCAACTAAAATAT
TGATAAGGATTAAAAAATAAGTAGGTAAGAACTGAAAAATATATTGCTCT
GTAAATTGGGTAAAAGATTTTTTTTCTTTAAAAAAAAATGGAGGACTATAT
AATAAGAACCCCTTCCAATTATTTATTTTGCAAAGAAGAATGAAAATGCT
GATAAATATGTACAAGATTATCTTTACATCCTTGTTTAGATTAATGAAGT
GCCATGCTATGCTACTTTCTTGTCATCCTGGGTATGCGTCGCAATATTTT
TACTGATGTTCTGATTTGTGAAATGCTTCAGGGTATTGGAAATGCGAGCA
ATATTTCGAATGTAGATTTGGAAAGACTTGCTATCCATTCGTCTAGAGTA
TCAAGATCTAATTTCCACACCACGTCAGTCTTCTCCCTCTCTAATGAGAT
GTTTTTTAATACTAGTGTTTATCCTGTGGCTAAGACGGCTAATTGTAATT
TCAAGTCATAATTTGAACCCCACTTGGATATCCTAATTTGAAGGCAGGGC
AAATGGAACTCATATCGTAATTTGAAGGCAGGGCAAATGGAACTCTTTAT
CTTTTAATAAAATCAATGCAAAATTAGAAAAGAATGTAATCAAGCATTTT
AGATTTTGCATTTGCCCAGTAGAAGGCCACAGTTTTTTTTTTTTTTGAAT
CTCACACTACCGCTATATCCATTAAAAAGAAAGATACTGCATTTGACCCA

TTCAAAATAAAATAACAGAAACTTACTTTATTTCGCAATTCTATATTATT
TGTTTATATATATATAAAAGAAAATCTTAAAATAGGTAAAATAAATAAAT
TAATAAAAATTGAACTGCGACCTTGGGATAGAGAAAACTAGAAAGAAAGA
ACAGAGGAAAGTAAGGCCAAGCGTTAGCTGAGGGAATTGAATCGCTTGGG
GCTATAAACAATAAGGGCACGTTTGGGTAGGGTATTTGAAGAATGAATTT
CATTTCAAGAAGGGGGTTTGTAATTTTTCTTAACATAAGTTTAAAAAAAA
ATTACAAAACCCTTCTCGAAATGAAATGCCGCCCCTAAATGGAACATAAA
TAAGTAAGGCCCGTTTGGTAACCGTTTCGTGCCCATCCCATGCATTGGGA
TGGAAATGAGATGAGATGGGATGAAAATTTTGTTTCCTCATTGGGAACAG
AATCGCGTTTGGTAACGATAATTTTTTATTTTTTTTATCTGAGAACAGAA
TTGTGTTTGGTAACGATATCCAAGAAATCAAGAACGTACTTTAAACCACA
ATAAAAAAATCATCAAACAATTAAACCCTAAATTACAATCAAGAAGCAAT
CAAACCCTAAATCACAATCGAAATTGAAATCGAAATCGAACCCTAAAATC
GATTTCGATTTCAAAATCAAAACCTGATTTCGATTTTGAAATCAAAACAC
TATAAGCAGATGAAATCAAAACAGGGTTGAAGAGGCGAAGAACACGGTTG
AAATCGATTTCGATTTTGAAATCGAAACATGGTTTCGATTTGGATTTTGA
AATCAATTTCGAAATGGAAATAGAAACACTATAAGCAGACTAAATCGAAA
CAACGTTGAAGAGGCGAAGAATACGGTTGAAATCGATTTCGAAATCGAAA
CCCTATAAGCAGACGAAATCGATGACGATGGCAAAGAGATGAAGTAGACA
ACGGTGGGTTGGAGGAGGTCAGCGGCAGTGGAGAGCAGAAGGAAGAAGTT
CAACGTTGAAGAAGAAGAAAAAAGAAAAAAGAAAAAAAAAAAAAAGGAGAG
AGAAAAGAGACTACCAAAGTTTGAAGGGGGGTCTCACTAACAGTTGAAAA
AATATCCTCCGTGTCCTTAAGAAATGGGAATGGGATGGGAAAAAATGGGA
TCAATTTTTTTTTGGTTTCTCACCTATTAAGAAAAATGGGAATGGAATCT
AGTTTTGGGAAGGGAATGGGAACGGAAATGGGACCAAACGCTATTTCCAA
ATAGAAATCCTAAAAATCTGGGATGGGATGGGAACGGTTACCAAACGCCA
CCTACAATTCTCCCCTTTACCAGACCAATATAGCTAAGGTCTATTTATTT
CGAATCTTGGGAAAATCAATCCAAACCCTTCATTTTTTCAAAACCAACAT
GTCTAGAGTGTTAAGGGTTGGTTTAATTTACATCTGGGATATGAATTCTG
TGTCAAAAACAAAAAATTTAACTACTTGTACTTTTAAAATATAAATCAAG
AAACTATTTTACGAAACAATTGTCATTATTCTCAGTCAATCACCTCACTT
CTTTTTTAATATTATACACTTACTATTTTTAAAAAATTATTTTTCATTTT
TTCTCCCTCTACACAAATTTCTCACAGATATACTTTATTATTTAATCACA
TAAATAATTTCCTTAAGGGCCTGTTTGGTTTAGACTTTGAAAATAGTTAC
CTGAAAAGTTTTTCAAAAATAGTTTTGAGAATTGCGGAAACTCTAGAAAC
CCTTTTTGAGGTCTAAACCAAACACGCCCTAGATTAGACATAATTAAAGA
AGCTAATGTTTCTGTGCTATTAGTTTCTATGTTATATACATTTGTCATTT
GATGTAATAACTCATTTCCTAATTCTGATTTTTTTAATCTCCAGTGACAT
CCACATACAGAGAAACATATCATGCCCCGTTTCACCAATTGGGAGCCCTC
TTCTATATCCAAGGTCACCACAACACTTCAACAACGGTAGAATGTCTCCT
TCACCTATATCTAGCCCTCGCACCACCTCGGGTTCATCCACACCTCTAAC
AGGCGATATCCCATTTCATCAACTTAGTCAATCAGCTTACTTGCATGAAG
GCTGCTTTGGAAGCTTGCCAAAGCCCAACAACAATCCCTTTACCAACGGC
TTGCCCTATTGCGATCCAAACCCTAATAATATCTTTCGAGGATCTCATGC

TTTTCCATCTTATGAAAATGACAGTTTTGGGAAACGATTTGGTAGGACTC
CTCACGGGGAGCTTTATGATGGACAGTCGATCTTGGCCGATCGTGTTTGT
CACCAACTTTTGGGAGATCATGTAAAGTTGAATCCGTCCCTGGATTTGAA
TCCATGCCTGAATGGCTCCTAG
>EUC17152-RA [gene]
ATGATTGATTTGGAATTGCGTCACCTTGAGGAAAGGGTGTACCTCATGTC
CTTGGAGTGGCAGATTTTGGAGAAGGGGCTTACCACAACTGGTTTGATTC
AGAAAATTGCGGACACAGTTGTTGAGAAAATGGGTGGTCCAGTGATTGAT
GCACAAGAAATGATGGAAAGGTGGAGTGCCAGGAGTTATGAATTGAGGAA
TGATTTGAACACTATAATCCTTCCCCTTGGCTGTCTTGATGTTGGACTTT
CACGTCACAGGGCTTTGCTTTTTAAGGTTTGTTAGATATACTTTGCACCG
TTACTCATTGATTATGCAAATATATTGGTAATTCTTTATTCTCCTTAACT
GTTTTTTTAATATGTATATAATTTTTAGTCGGCATAAAATTTTGCCACTA
GATCATGTTTTAAAAAAAATAAGCTAATCTGAAGTACCTCCCTTATTTGCA
CAAAACAAATGCTATTGTTAATGTTCCATTTTTCATAACTTCATTATTGA
TCCATGTTAAGTATATGAAATGTTTCTTTGAAATCTTTTCCTTTAGTTTC
TCTAGGATGAAAAGAAACTCATAAACGTTTGCTTCCTTGTTGTTCTCAAC
CTCAAATAATTGTGGGTGTATTCTTGTACCTTGTTTCAAGTTTTAAATGC
ACAGGGAAGGATTTGATGATCTTATACTTTGGGTACTAGGTACTTGCTGA
TAAGATTGATCTCCCATGTATGCTGGTGAAAGGGAGTTACTATACTGGGA
CTGACGACGGAGCTGTGAACTTGATTAAAGTTGATGATGGAAGGTAACAA
CTTAGAGCTTCACATTAGGCTAATTAAATTGCTTGAATTGCTATTTTTTC
TAAGCCATCCAATTGATCAGTTTCTTTCCCGACTATTTTAGTCTTAGTCT
AACTTCAATTACTTTTCAACTTCATTTAGTAAATAGCTGTATATATCATC
TTTGATTAGCATTCACCTGAGAAAAAGGTGCATTAGGGTTGTGAATGTTC
ATCTCTACTAATTTTCTGTTGGTTTCATTGGGGTTGAAAGCACATTATG
CACGTGGTGGAAATCATGCTTCCTCATGATGCTTTGAGAACTTTTTAGAA
ACTGCTATGGATTGATTATATGGTTGTTAAAGTTTTTGGCCAACGTCATG
CACTTCAATCTAAATTTCATATGTTGTTTGCTTGTTCATCGAGGATTGAA
GGCTAGCCGCTAGTTACCGTTCATACTACTAACTGAAATCTAGATGCTGT
TTACCTTGTTGTGCATTCCATGCTTGCAACTTCTGTTTGGCCAATTTTTT
TTTAATGTTAATTGGTTCTCTTCGAGGCATTAATTCGTTCTCTTTCTTCT
CTTTTTTCTTTTGATTGAAGATGAAGCATGTAATATTTGACTTTTGAACT
TGGACTCTCCATACTTTGTACTTCTTCTGCTAGTTATGCTTTTTGACACC
TTTTCTGTTTACTTCTTTGCTGGATATTTCCATTGTATGTGAACAAATTG
AATAAATTTTAATGAGATAGGCTATGTGCTTTTAATTTGGCCTAAATGAA
AATTCCATTAAAGTTCCCTCATTCACTAGAATGGACCGTTATCGTCGTCT
TAAAAATTTTCAGGAAAACTTTCTAATGATTTGACTGATTATTGGATTGA
GCAGTGAATATATTATTGATTTAATGGGTGCTCCGGGCACACTAATTCCA
GCCGAGGTTCCCAGTGGTCACCTTCAAAATTTGGGGTTAGATATGAGAAG
CCATGCATCCATTGCTGACAAAACCAAAGATTCATGTCCAATAGCTTTTA
AAGGAATTGAATCAGGGATGGTTTCATCTGCTCTTAGTGGCAGTGCACAA
TTCAGCCCTTCAAATTCAGATGAAACATCTCTCGTAGGCATGCAGCCAAA
AAGGGATGACAAAAACACTGGGGGGAAAAATGAAACTGAGATGTTTAAGG

TTGGAAATTTTCTCCCTTTTGAAGGCTCATCCAGTTTTGGCAAGAATGTG

TTGGCTGCAAATGAGTTGAAAGTAAAAGATGTTTCCAGATATGTCATTAG

TGCTGCAAAAAACCCAAATTTTGCTCAAAAACTTCACGCTGTTTTATTAG

AGAGCGGTGCATCTCCTCCTCTGGATTTGTTGTTTTCAGAAATAAATCCC

AATCATCCGGAAGAACAGAAAGCACTTGAGATGGCTCATTGGTTAAATGT

AGATGCCATGGTTGACAGGCCTAGACTTTTTCAAGAAATCCCTGTGGCAA

ACAATGATATGTCTCTTATTCCCTTCACTGGAGGGCATCAGGTCTTCAAT

AATGTCCAGTATAACATCCCACAAAATGTTTCTGCCGAGGAAATGGCAGC

AAAACAACAGGATTTAGAGTCTGGTGTGCCTTCTGATACAAGTGAGAGAT

TTGTGCTTGTAAATAGTGGACCTAGTGAAATGATCCAGGCAGATGGTGCA

GCTGTTCGTAATGATTCTGTTCAGCCATCTGAAATGGTTGCAAGAGCTTC

TGCGTTCTCTCCAACAGGACTGCCTGAAAATGCCATTGCTGCTGATGATA

AAAAATTCTTGAACAATAATGGGGGAAAGCTTCTCAGCAACATTGAAAAA

GACAAAGGACCTTCTGTAAATACTACAGGAACAGACAATTCTATTTTCTC

CACTCATGAGAGAATTTACCCTGTGCTGGGTGAGGTTGCTGAGTGGGAAA

TTCCGTGGGAGGATCTTCAGATTGGAGAACGGATTGGAATTGGTAAGATC

CTCTACATGGCTTTTACTATGGATTCATGCCAATGATAATAACGTAATTA

AATATAAAGGTTATATCTATGCATGAGAAGGTTTATTTGAAACTAATTCT

TGTCTCACTTATATATCAGGAAATTCTTTAATGCAGAGAGTATGATCTCT

GGAGTATTGAATTGTTCTGTTTTGATAATTTTAATATCTTACACACTTCA

CTTCTTACCTCGCAGGTTCCTATGGTGAGGTTTATCATGCAGAATGGAAT

GGGACTGTAAGTTATATATGGATATCATCTACCTTTTTTTCATTTGTTGC

TTGATACTTGTTTCCAGTCTTTTGACGAATGATCCAATTTATTGTCTTAA

AAGAACTTTCTTAGATGATGTGTTGCTTCTACCAAATGATTGAGCCATGA

TGATGATTTGATAAGTTTTTGTTGTGAAATTGACTTGTTTCCAGTAGTTT

GCTCTTTCGTGATATTCATCCATTGCTACCATGCTTTTAGACATGGCAAG

TGGTTCATATATTTGGATTGCTTTATAACCTATTAGCGCATCACTTTTCG

GCTTGTGATAAAAACCATCTTTCTCCTGCTATATCTGGTGTGGTTATGCT

AGTTACTATTGAACTTCTCCTCTGGTCGGGCAAAGGTGTATAGTAATTTG

AGTGCTGCAGGACTAGTTACCTTCTCTGATTGGGAGGTGGAAGAAAGAAC

GAAATTATTTTGGGTACCGGGGAATGAACAGGAGATCCACAGATTTATGG

CCTAACACAAACAAATTAATGCTTAAATTTCCATTGCATGCACTGCTGCA

CCTTTTAGATCTTGTTGCCCTTTGTTATAGATGTTAAGATATAGAATGTG

AGATATTCTGTAGTATTCCTTATATCATCATTTCATTGTGATAGAATGGT

TTTTCATTACAGGAAGTTGCTGTTAAAAAGTTTATGAATCAAGATATCTC

AGGCGATGCACTGATACAATTTAAATGCGAAGTAAGTATCAAATATCTCA

TGTTGTGGACTACCATCTATGGTTTCCATTTTTAGTCTTCATAAAATGAT

CCATTCATTTCCTGTCATTTGTATAATGAAGGTCGAAATCATGTTGAGGT

TGAGACATCCTAATGTTGTTCTTTTCATGGGAGCAGTTACTCGCCCCCCA

AATCTCTCTATATTGACTGAGTTCTTACCAAGGTACATTGNAAAAAAAAA

AACAAAAAAACAAATACACTGTCTTTTTCTTTACTTTCATATTCATTTGC

TTGCAGGGGGAGTTTATTTAAGCTGCTGCATCGTCCAAATATTCAAATTG

AGGAAAAGAGGCGATTGAGAATGGCTCTTGATGTGGTATGAATTCAAATT

TTGTTATTGGGTTCTCTTTCTTTGTGGTAAAAAATAAATATTGCTCATTG

GCTTTCAGGCAAAGGGGATGAATTACTTGCATACAAGTCATCCTGTAATA
GTCCATCGAGATCTGAAGACTCCAAATCTTCTTGTCGATAAGAACTGGGT
CGTTAAGGTCTTGTTATTCAAATTTCACCTATTCTATTCTCTTCACTTTT
ATCTTACAAACATCTGAATAATTTATTTGCGACTTTTTAAAAGTTGTATG
CATTGCAGGTTTGTGATTTTGGGATGTCACGCATGCAGCATCATACTTTC
CTGTCCTCAAATTCTGCTGCTGGAACAGTATAAAATATGAGACCACTTTC
CTTTTTGCAATCTTTTTCACTTTAAGCATCGAATTGGTAACAAAAATGGA
CAAATTTTGCACTGCAGGCTGAATGGATGGCACCAGAAGTTCTAAGGAAT
GAACCCTCCAATGAAAAGTAAGTGTCGAATTCTTTTCTTATGCCAAAACT
CAAAACCAGAGGCAGAGCCACCCCTTTGTCAGGAGGGGCANCCCCCCCCC
CCAAAAAAAAAAAAAAAAGGTTTTAAAAATTAACGTTGCTACCCTTTGTT
TCATTTTTTCACTAATGCCCTTGATAGTTTGTAATTTGTATTAAATTTCT
CAATGCATATCTTACAAATTTAGACTTTTAGACCAATTATTTCATCAATG
TCACATAAAATCACGAAAATATACATGTTAAAGAGTCCTAAATGTACTTT
TCCCATTTCGTTATTGAACAAATATTAAATTTTTCCCGAATTCTTGATTA
CGTTTTTTGAGGTCTAGCTTTGTTAAATGTATGGGGATTTATGATTCTAA
AATTTTAAGAAAGATTAAATTAGCCAATTACAGTTTAATACTATTTTTTC
CCCTTTGCTAGTATTGTTTAATTTTTTTTCCCCCCAAAAATTAAAAATTA
TTCTCTAAGTAATAATTTTTTTTTATATATTTTTTGTGGTCCCACTGTAA
TAAATCCCATTTCAAGAGAGTGAAACACTATTTGATTGCTGATTCTAACA
TATGGGTTTTGTTGGGTTGGATTTGCAGATCTGATGTTTACAGCTTTGGG
GTGATATTATGGGAGCTTGCAACATTGCAAGTACCCTGGATTGGGATGAA
CTCAATGCAGGTTGTTGGAGCCGTTGGTTTTCAGGACAGACACCTCCATA
TTCCACTCGACATTGATCCAACCGTAGCACAGATTATAACCGACTGTTGG
AATTCGTATGTTGTTAAACGTTCTTTAATGTTAATTGATGATTTTTGGGG
TCCCCTGCTGATTGCCTTTGTGTTCTCTTATGACAGTAATCCACAGGCTC
GGCCCTCTTTTGGACAGATAATAACTCGTCTGAGGATTCTTCAGCGTTTG
AAAAGAGAATCTCAAACAAACCAACAAGAACAACAACAACAACAACAACG
TTGTTGA
>EUC20951-RA [gene]
ATGAATATGAAGCATAAGAGGCTGGAGCGGAAGCTGGATCGGCGGAACGC
GATAAAGAACATCGACTACGACGCTTCGAACTCGTCGACTTCGACTTCGA
CTTCGACTTCGTTCGACGGTCAACCGACTCACCGGACGCGTTCCCTTGAC
ATTTACGCCCACACGGATCGCTCCAGCTTCCGAGTCGACGGAATTGATGG
GTTCGATCAGATTTGCAAATCTCTAGGGCTTTCCGGACCTGAGGACTTCT
CAATTCCTACTGCTGCTTGGGAGGCGCGAAAGGCTCGGTCTCCTTCTGAG
CTTTTTCCGAGCTCTAGGTTTTGCGATTATTACAATTCGGTGAAGGAAAC
GACTGAGGATGGTTTGTCGAATGGTATTCCAGCTAAAGTTAGGACTAGTG
ATGAGTACAAAAAGTGTGAAAACGAGTGTTCTAGGTTAGAGAGCGGGTTG
AATTCAGCTAGGGTTAGGGTGAGTGACGGAGTGAGTCTTGAAGATGATGA
GGCCGTTTACTTAAATTTTCCTACAAATACGGCGGGCGGAAGGTGTGCTA
GGGATGGTGGATGTGGGATTAAGGGTGTCCGACCTCCAGTGCTGGCTCCT
CCCCCGCCCTTGTCACGGGCAGTTGTGGATAATAGGAGATCAACTTGGGA
TTTATTCCGAGATTTCGGTCCTCGAGATGATGAAGATATTGGTTTGCCTT

CAGGTGTGGGGCTAGCAAACTCTTTCAGTGAAACAGATCAGGCAATTGAA
GAAAATAAGGAGGTTGATGGACGGCTAGTTTCTAAACAGGATTTGAATGG
GGAGAGGCTGATAGCCTCTATTGCCTTTACAGAATCTTGTTCAAATTCAT
CGAATGATGATGACGATGATGATTCCTTCAGCATGGTTTTGGAGCCTGAA
TATTCTGTTTCACCTGATAGAATATTCAGGCCTAGTATCAAGTCTTGGCA
GAAGGGTGACTTTTTGGGAAGTGGGTCATTTGGAACAGTTTATGAAGGAT
TCACTGAGTATGTTTTCTTGAGCATTTTCTTTTCAATTATTTCCCTGTTA
TTATCCAAATTATAGATGCCTATGTTATGTCTTTGCAAAATACAATGGTA
AATTACTTAGTGGGGTCTATATGGAATATTTTGTATTACGTTTTTGTTTC
TTGTTATTGGGATGTGTTTGATAGAGTTTGCTTTGATTCACTTGGGAGGC
GCAAAATCCATGATTAGTTTGATAGGTATGTGGATATCATTGTCAAAAGC
TATACTATTGAAAAAGTAGATAAGACAACTTCGGAAAGGTTAGTGGTCAT
TTTGCCGTGGCAGTAGACTGAAGAAGTGAAGATGTCGTTGAAGTCCAGGA
AAATCCATCTAAGATTCTTTTTGGAAATCCACCAAAGATTCTTTTTTTTC
CCCCCTTTTTATAGCTCATAGATAGTTGAGTTATAATATGGAACCTAATT
GAAGAGGAGGGTGTAAAACAAGCAGACCTTAAATAATTTGACCTAAATTG
TTCTCAAAGGATCATTAGAACGAAACTCAATGTATGAGGGTAAAAACGCA
ATGCTCATCTGAATTGCATATAGAGAAGTAGTGCAAGCATTTAGTGTTAA
TGTTATACATTTTAGAATAGAAAGAGGGAATAATGTTTTTGAAGCACAAT
GTGCCAACTCTACTGGATGTGTCATCCATGTGATTTTCATAAATGGTCAT
GAGGGGAGAGTTTAAACTTTCTAAGAATAGGGGGGTGTTCTTAAAGTGTA
GGAGTTATGCATCTTCATCATCTAGCAAATTATTGAATTCGAAATAAGTT
TATGTGGTATGTGAACCATGCCTAAGCATTTCAACTCTTTGAAAGTTACT
AGTAAGTTTGGGTGTCAAGTTGAGTGAAAAAGACAATTTACAATTAAGGA
GCTAAAATAAGGAAACAAGGAAATTCTGCATGTTTTCTCCTCAAATCCTA
TATAATTTACAAATGGTATAAAAAGAAATGATTGCTGCCCTAATCTGATC
AAATCCTTTGGCTTTAGACACTCACACCTCATGGTCCTTGAGCCTTGTTA
TCACTCCATTTGAATTGTTGGATCATTACTTATCTCACACCACCACCCCA
ACCTCCAAAGAGAAGAAGAAAAAACTTCTTCAATTCTTTGACGACATATA
TTTTCTTGACATTTGACAACTTACGCGTAAAGTTTAATGGAAAGTGATGC
TTCTTTATCCTTTGCGTAGTTGCTATTTAATTTTCATTGCAAGGGTGCAA
CCATGCAAATGAGTCAGTTGAACTACTGACCCCCATCCCCCACACTAAGA
AGGAAATGAAAGACACTTTCAGAATAGTCTACATGGTAAAGGCCATCCGT
GCATGTTACATTCTCTATCCTCCTCCAACTGCCTACCTTCTTTTTTATTT
TATGTAGACTAATAGATAAAATTACATTACTTTTAAATTTCACTTCCAAG
TAGTAACTAACACATCTTCCTATCTATTTTCTTTGTTGCTTTTAATCAAT
AAAATCCCGAAGTACTTAAATGTATGATGAGGATTTGACTATGACTAATC
ACTAATCACTAGTCACTAACTGAAAGCAGTATCTATATGTGCTGTATGTG
AAATAAACAAAATGGTAAGAGGAAAACTTGCCAGACTTTCCTTCACATAT
GTGAATGATTTCTTGGAATTTGAGGGTTTGATTAGTCAGTTTGATCCTCC
TAGACCTACACTTAACGGGGGGAAAAACTTGGTGATGTTTAGGAAAGATT
AGTCAATGAACAATCTGGCCAGCTAGGTACCTCGCACTGGATACAAAGGT
TCTTCTTTTTATCATTTGGCTGATGGTTTAATTAATAGACAAATCTATAA
ATTATCACATCTTTATGTCACCTTGAAACTAAATTCTATGTGATGTGGAA

ATTCCTTACTTTAGCTTCTACAACAAGGAGATGTAGTTGTCTTATTAGCT
GAAGAAGCTAATTGGAAGGTGTAACATGGACAACATCTCAGTAATTGTTG
GATGATCTCCATGTCACCATCAAATGCATGTACTTAATGGCGCAGTGGAG
CAAATGTGGTCCATATTTGAAACTATTTAAGGTTTCCTTATAACCAGGAC
TTAAAAAGGGAAAAGCTTTAATTGGAGGGCTTTTCATTGAAACTCCTTCC
ACCTAGCTATTAATTGAAACTCCTTCCACCTAACGGATAATGGGATATGG
AGGATTTGAAATTTTTTTGTGCTCCCTACTATTTGGTCTATACAACCATC
AACTTTATCTTTAATGTTAATACACACTGATAAACTCTATATAGATTTAG
TACTTTTTCTAGTACCCAAGTGATTTTTATGATGCTTTTCCACCCAACTT
CTACATGGTCTTGGATTGCATTTATGTGTTGTTTGATTTTGACCGTATTC
AGTAATTTAGGTGTATAGAGGATAATAAATGTCATGATACTTCTATTTTT
GTCACTCTTATTCCATGATTCCATCCTTCTTTCTCTCCCCTTCTTGCCCA
GTCCTTTAGCAATTTAATCCATCTGCCTCAATATCATCCCCCTTTTTTGG
CTGCAGTGATGGGTTCTTTTTTGCTGTAAAGGAGGTTTCCTTGCTTGATC
AAGGAAGCCAGGGTAAGCAAAGCATTTCTCAGCTTGAACAGGTAATTTCT
TTGTTAATTTTGTGTATACGGGATTTAGATGTCTCTATGCACGTGAAGAT
CTTTTTCTTATATCTTGCTAATTGGTTCAGGAAATTTCTCTTCTAAGTCA
ATTTAAACATCAAAACATCGTGCGATATCTTGGCACGGAGAAGGTATTAT
TCTTGGCTCTCAAGTGCTGCTTTTATTTCCTCTCCCTTTTTAGCCGTTCA
ATCTGATACTACAATTACTATGCTGCTGATTTGCTTAATGTCAGTATATG
TCTTAATGTCAGTATGTCTTTTTTGGAAAGATTCTGCCTTTCCCTGGTTG
CTAATTTGGTAATGCATGGAAGGAGATGATCTCTACCAAAAAAATAAAAA
AGCAATTTGGGGGAAGGGCCTCACTTTATCAAGTATACAAAATTATGAGA
TGCTTATTACACTATGCTATGAAGTATAAAGTTAGTCAAACATATGATTG
TTTCATAGTCCCGCAGCATCCTAATTTTTGGAATCCTGTTGCTTTCTTTT
ATTTGTATAAAAGAAAACTGACTATATCTTCAAAAGATCATACCTTATG
TTGGTTAATGAAGTGAACCTTCGTTATATAAAATAACTCTAAATTCAAAC
TGAAGCAAGGTTTTGAGATGTTTTCTTCTTGATGACCTTTCCGTTTAGT
TTCATTGATCTACCAGTGTCTTAGTCAGGGTTCATGTTTCCTTTTTTTCG
TTCTGAACTTTTCAGGATGAGGCCAAGCTCTATATTTTCCTTGAGCTTGT
AAACAAGGGTTCCCTGGCACATCTCTATCAAAAGTATCACTTGAAGGATT
CCCAGGTCTCCGGATACACAAGGCAAATTTTGAATGGATTGAACTATCTT
CACAGGCAAAATGTTGTTCACAGGTGATAAGAATATTTTTGGTTATTTTT
TGATGGTGGTGATTGTGGTTGGGTTCTTCTCCATTTCCCTATCTTCTTTC
TTCTTTTTCTTGCTTATGGAATGGTGGACATATTGATTGGGTGTGTTAAG
GATGGGGATGGCAATCCAACTCAGGTTCGTGGTGTCTCTCCCCTCGTCCT
GTCCCGAGATTGATAGGGTTCCACAATTTTTTAGCCGGGGCATCGGTATG
AAAAAAAGTAACCCACATCGTATTTGGGTTGGTTCAGATGAACTTATAAC
TGCCTTGCTAACCTATCTAGAATTGTAGTATTTTACTTAAAACTTGATAT
ATATATATTAGTGTTATCATGGGCGTTAGCAGGGAGCGCCCTGAGATGGA
GCGCAAGGAAATTGCCCCTGGGCGATTGGGGGGGTGGAATGAAAAACCTT
GTGGGCGTCGCCCAGGGCGTAATCGTATGCCTAGGGTTTTACAAAGAAAA
TAGAGGAAAATGGGAGAAGGCGGTGGAAATCGCACATGATGCACGCGCGC
AGAGAGAGAGTAAACTGGAGAGAAATACGTACTTACTCGTCGGAGAAGTC

GACGATGGTGGCCGATTGCCGGTGGTGGCTACTCTAAACCTACTTGTTGG
AGAAGTCAATGATGGTGGCCGAGGTTGTTGTTGGTGGCTGCTCTAAACCT
ACTCGTCGGAGAAGTCGACGATGGTGGCCGAGTTTGTCAGTGGTGGCTGT
GCTGATCGTCATTCATCCTAGAAGCTGCGGGCTGCATGCTAGGTTGATCG
TCAATCATCCTGATCGTTCATCCTAGACTGACCAATTAGGTCTGTATATA
TGATTAATAACTGACTAAATTATACATTTGGCCCCTTAGTTTTTTAAGAT
TAAATTATACATTTGGCCCCTTAATTTTTTTAAGACTAAATTATACATTT
AACCCTTTAGTTTTTTAATATTTTATCTTAATATAATATATCTTTTTTAA
TTATTTTTCGGGTATTTTTTAATTATATATAATATTTTATAAAATATTAA
AAATTCATGTATCACGGGGCTTATGCCTCGCCCCGGACATTCAAAATGCC
CCGCTCAACCCCCCGCTTTTTACAACACTGATCTATATTGAGAGAGAGAG
AGAGAGATGGCAACCGTTTTGTTTCGGTGTAGGTTTGACAAAAAACGAAA
CTGAAATCGCTTTGTAAAATACAAAACTGAACCGACGCCGATCCATAAAC
CAATGTTTTTTTAGGGGTAGGTTTTTAAATACGAACTGCTTGATTTCTTA
AGAAACCGAAATGAAACTGGAAACCCATTGAAAAAATACAGAGAGACCTG
CAAACTCGATTCTAAATGTCGGGATTGGGGCGGGGTGGGTTGATAAGAAA
TTCAATAAGGGGCAGCTTGGTTGCATAATTTAGAGATCCATTTCGGATGT
CGGGACGGGGCGAGCAAAATTTAGAATGCTCGGGTCTAGTTCAGGTATTG
CATAAATTGCCCCGAACCCGCTCAATTACCTTCCCTAGGTAAGTACTACA
AGCCACTGTTCAGCTTTTCTGCATCAGCAATCTCCTTCAAAAAGTAAATT
AATAAAATAAAATTATAAGAAATGTGAAGAAATAGGAGTTCCTTTAATTT
GTCTTCATCGGAGACGAATCATGTATTGTGGTGAATATGAATAAATCGGT
AGGCTAGTTAGTTATGTTGAATAACGGAGTTCTTTAGGTCGAACAAGAAT
TGTTATTGGTAGTGAATATACTTGCCATTAGGAGACTAGATTACTTGTTT
AAATTGATTTTACGTTTTTATATTGTTTTATGAAATCCTGATAAAGAGAT
GCCTAATTTGGTTTTACGTTTTTATATTGTTTCGTATAGGGACTTCTATT
TGTTTGCCCAAATTAGGCATCTTTTTATCAGGATTTCATACTTGATTGTG
GAGCAGGGTTTTCATTTGTGAGCTGTGTGCTTCTGAGACCGGAGACCCTA
ATTTGGTTGTCTTCTTTCTCCTTCTCTATCCTGTGTTATTTGGCCTTTTG
CTGAGGCTCCACTAGCTATCGGTATGATCATAAGGTCTTTGTACTTGTTT
GTGACTATATAGACTACATGGAGTGGAGTAGGGCTGAGCACCAAGGGGGT
GGATTGGGTTCAGCATCCACCCGCCAGCCGACTGGAAGATGGTCGGGTTT
AGTGCATCCACCTGCAGTAAACTGACTGTAGAAGACAACAAAAAGGAATT
GACCAAAAACGTAATTCCTATATTCAATGCCTTAAATAAGGAGAAGCAAA
TAATTATAAGGACAGTGCCCGGACCACACTGCGTCAGGTGCCACATCACC
ACACCAACGCCACTGCCGCAGCTGGCAGTGCCAGCAACTTCACCAGCCAC
CCCATTGACACCCTGCGTGCCGGCGCTGACATTGTTGGCAATGCTGGCGA
TCTCGTTGGCCCCAAAAGTTTGCTGAAACCCCCACTGGCAGCGCTGGGGA
CTTTGCTGGCGATTGCACCGACGCATGGCGCCGGTGTTGGAAATTCACTG
GAGCTTGCCAGTGTCTACACCAGCACCGTGCGCCGGTGCTGCTGCCTGCT
GGATCTATGGGCAACCTTGTCGGCGCTGTGCCGAAGCTTCCCCGGCGCCT
TTGCCAGTTGTTTCTTCGCATCACTCTACTTACCGGTAACTTTTTGGTGA
AATCTTATTTTCCATATTTATATATATTTATAATTTAATATATATATATA
TATATATTTAATATATAAAATATGCAAAGTATATATCTATAGTATATATG

TTAAATATAGAAATGTATAAATATATTATGTAAATATCCTTTTTTATAAA
TACGACCTTAAATATACCTCTATCATATTTATTATTTGCACTATACACCC
TCAATCTTAAATAACTACTCCATGCATCCCCTTTATTTTGGGATAAATAT
TCGTTCAACGACTTTATTTTGATAATATCATTAATTACATATTTTAATAT
TATTCTTTAACGGATTACCTTTCCTGCAACCGGAAAATCCAATCGGACGG
CCGACGCTGTTGCAAAATGGTCTACATATCTAGGATTCGTGTGAGAGAGA
GATCACTGCACATGCTCCTATATGTTGTATGAAGTCCAATCCGTACTCAA
GGCATTAATGGTCTTTGTCATCACAACGAATTCAAGGAGCCGTATGAGAG
AAAGATCACCGCAGATGCTCCCCTAAGCTGTACGAAGTCTAATCCATATG
CAAGATGCTAACATGAGTGAGCCCCTTAGAGAGCAGGGCTATGTTAAAGA
CTCATCCATCAGGATTCTAAGGAGATTGTGGATTGGAGTATGAACCCGGT
GCCTACGGGGATGTGTCGGCCACGTGCCCTGGCTGTTGGTTTGGTTGATT
GGACCTCGGACTAGGCTAATTTTGAAAGCCTTCTTAATCATGTTATTAAT
GATAAATAATGATATAATCAATTGGATGTTACAAAATCACATTATAAATA
TATTATTGAAAAGGCATTTGAGGGAGAGGGCCTAAGAAGCAGAATAAAGC
GATTACCACATTCTTGCAATCAAGCAAAAACATGCCTAAACAAGCCTGAG
GTCATGTAGTCAGTCCAATGACTGTGTAACTTAGCAATCTCTCTCCTTTT
GCCTAGTTGACCGTTGTTTCATCTCTCTTTCCCAAACTCTCTAGGTCCAT
TTTGTGTAACGCCCTCCAATTACTAAAATACTCCTTAACCCTTTTCTGAC
CCTCAACACTGACCATTTAAGAATGAGCAAGAGATGACCTGGGCTAGGGA
TTGAGACGAATCTCTTAGGATTCAAGACACCCATATTCAGATGCGGTGGG
GCAAACAATAGGAGAGAGTAGAGGGTTTGAGAGGGTAATACCTAATCTAC
ATATATATGCAAGGGATGAGGAGCTATCAATTTGCAAACTTCTGTTGGTT
GGGTTTACCAGACACTAATGGGCTCAATACCAAGCCCTAGGAAGTACCAA
AACTTTTGGTAGGTCTATGTATCTGTGCTGGATACGTTGTGGACACGCCC
ATATACGTATGACACGCCTAAATCCGTGTCTCAATATTTTGTAATTAATT
TATTGTTTGGATACTTCTTGGATATGTGAGGACGCAAATCAATCTACCAT
CTCCCGTCTCTTGATTTTTTATTCAATTGATGGCCTCTTGTGGAGGTAGG
TGAATCAAGGCAGCAATTAATGGAGGGAATTGATATTTTTGATGTTCAGT
TTATGACGTATTAATAATGTTTTTGTTTGGAGCAATTTGAAGTTTAGTTT
CTATTTTTTTTTTTTTTTTAAACTCTAGTTCAGTAGTTTGTTGCTTATGA
CCATATATTTAGACATGCGCATATATAACTTTAAATTTTGCCGTATCCGT
CTTCTATATTTTTAGAATTTGGCATATGACCGTGTCAATGTCAGTGTCCT
GCCATTACCGCTTCCTGTGTCTGTGTCCGTGCTTCTCAACCAAGCCCTGA
CGGGATATTCTTTTAATCCGCCAATTTTGGTTTTGGGAGCAAAGGGTTGG
TCGGGTTTTACATGTTCTGGTTGGGTTTGCTTAGTCGCCCTATATAGTCT
ATTGAAGGGTGAAAGGGGAGCAAGTTGATGTTGACAATTTTTTTTAGTAT
ATATTGGGAGTGAGTGGCCTGTTTCTTTTAAAAAAGGATTTTATTTATTA
TGCTGTGTACAACTGACCCTTCGTGAAGAGGGAGGAAGTTGGTGTTGACA
AATTTTTTTTTTCTAATATATATTGGGAGCGAGTTGCCTTTTTCTTTTAA
AATGATTTTATTTATTATGCTGCGTCCAACTGACCGTCCGTGGATTGGGT
TGCAATTGTTATGTTGCATGAAGGATCACTTAAACGTTATGTTCTGATTT
TAAATTGCATGTTCTAAATTCTTAAATTTTTTTTGTCTTCAAGGGATATA
AAATGTGCTAACATATTGGTGGATGTAAGTGGATCAGTGAAACTTGCAGA

TTTTGGATTGGCAAAGGTTATTTTCTTCATCAAAGCAATAGTCGTTTTAA
TTATTTTTAGTACACTATTTTAAAATAAATTTATTTTGTATGCAAACTAT
ATTAATTCAATCTAATTTATTTTTCCAGACAACCACATTCAATGATGTAA
AATCGTGCAAAGGGACTCCGTTCTGGATGGCCCCAGAGGTTTACCTATAT
TCCTTCTGCAATTTTATAATTATTAAAAAATAGCTCTGCCATTTATTTAT
AAGATTTGCTATATTGCATGCCATGTAATACCTTTCTATGTGTAGATCGG
GAAACGTATTATATTTTTCATTGTTATCTATTTGTGTGACACAAATAAAT
TTAGGTATAATTGGCGATTAGTTTCAAATAGCGGGTGTAATTTGTTGTTT
GTACTACTTAAAATATAAGTTTAGATTTAGTCCCTCATATTGGTTTTGAT
TCCGTATTACTCTCGTATGTGCTCATGTATTGCATGTAGAGGGTAGAGAG
AGCTTGTTGAATAAATCCTTATTACAGTGAATATTAATTACATATAACTA
AAATTAGTTATAAGTTAATAATTACAAATACATTTAACTATATTACATAT
ATCTGTGTAATATTAGTTACATATACTTGTGTAATATTAGTTATACGTAA
TTTTTGTAATTACAGATTACATGTAACTATATTACATGTATTTGTGTAAT
ATTTGTTTCATCTAACAAATATTTTACACACACAAGTTAGATACGTGTAA
CTGCGAGAGCGCAAACGCGTGTATTGGGCCTTCCGCCTTTTTTTAATGCG
TGGGACTTAAACACAAGTTGCAATTGGGTAGGGGACCTTTTTAAAATTAA
GGGTAAAATAAGGGACCGGCCACCGAAACACTCATCAATTTATCATGTAA
AGGACTTGTTTCTAATGAGCTGTTTTAGCCAATTGGTCAGTTGCGCAGAT
ACAAATTTATTCATGTATTGGTCATGGAAGAATGTTTCTTCAAATATTAC
ATGTATTGTTGTTGTTCTTGCTGCTGCTATCTATTCAATTTCCAACTATG
TTAATGAAAGATTGTGACCGAATTTAAAGAAATACTAAAATAATAGAATG
TTAAGGCTAATTAAATGTTTAAATGTGGTTCTCGGCTGAATATACTTCTA
GAACATAGTACAATTAGACGTTGTTGAGGTTCTTGGCAACTGTTTCATTT
TCTTCTATTTTCTATTTTCATGTTTCAGATTCAGAATTATATATTTATCA
TTTGTGGTTCTTATATTTTACAGCTTCATTTTTGTCTAACATTCATATTG
AAATTAAAAGCTAAAAAGTTGATGTGAATTATCAGGTTGTTAAAGGAAGG
AACCGTGGCTATGGGCTTATGGCTGACATATGGAGCCTTGGTTGCACTGT
GTTAGAGATGTTAACTGGTCAAATTCCATATTCTCACTTGGAAGGGGTAT
GCACTTACACAAATTATATATGCTCACCTTTCTTAATAGTTGCATTACCG
ATGACCCCATTTCTGTTGGATGCATTTTTTTTCCTTGTGCTTTCATACTC
GTTAAAGTTTGCAACAAGTTATTTTTTTTTTAACTAATCATGATTGCATT
TGAAAAAATTCGGTGATAATAAGTTCATTTAATTTTCAATGTGATTTGAA
TTCGTTTTGTATCTCGCGACTCTTGTTGTTAATTTGATGATAGATTTTGC
AATCCAAATTTGATTCCATTCTAAAATAATGTGGAGGAAAAAGAGAGGAA
AAATAACTAGGGAACTGTTTGGTAACATTTTTTTTATAACAAGAAAAAGT
TAATTGAACAAAAAAGTACTCTAAAAGCAAAATCAAAAAGTGTGTTTCAT
ACGTCGATGTTATCTTTATAAAAATGCTCTTATCCAAAAGTGTTGGGGGT
TATGGAATGGGTTGGTGGTCAGTTTATAAGTGTGAGGGAAAGTCTCACTT
CATGAGCTAACTTTTGGGGTTGAGAGTGGCCCAAGACTACCCCTACATTA
ATATCAGAGTCCAGGTTATAACATCTCTGGCCCAATACCTAGAAAGAGAG
ATTGATCGATGATTAGCCGATCCAAATGCTCGATGTGTGAAAAGGAGATT
GTTAGATTTATCCCACATCTAATGTGGAAGGGGTTAGTGGTTAAGTTATA
AATGTGAGGGAAAGACTCATCTCATGAGCTGTAGCCCTTGAGGTTATGAA

AAATTGGTAGAGGTCCTAGACATCAATATATTAAAATATAAAAATTGAAG
AAATATAAAACAACTATTTTTTTGTATTTAGAAAAACAGGCAGTTTTACT
TTCTTGATTTTTAAAAGTACTTTTTTGAAGTGTAAAACAAAAGCTACCAT
TATTTTAAGTACATTTTGCTTAGCAGTTGAAAGAAAAGCATTAGCAAAGG
GTGCCTAAAAAAATAAATAGATTTTCTGTTTGCACATGTATAACTAGCAA
AGTTTCTAAGTGGATTTTGATGTGTGTTCTATTGTACTAAGTAAGGGTGT
ATTTTGGTTACCATAGCCGATGCAAGCAATGTTTAGAATTGGCAGAGGCG
AACCTCCTCCAATACCTGATTCCTTATCAAATGAGGCCCAAGATTTCATC
CTCAAATGCTTGCAAGTTGACCCAGATCATCGCCCTACTGCTGCTCAACT
ATTGGATCATCCATTTTTAAAGAAGTCATCCTCAGCTTCCCTGAGCCCTG
CATCTCCCAATTACTATGGTGGGAGACAATAG
>EUC17437-RA [gene]
ATGGACGGTTCTGCTCAGCCATCAGACACCGTAATGTCGGAGGCAGCGGC
GGCGCCTCCCCAATCTGACTATCCGCATCCGCATACTCATCCGGCGGCGG
GGATGGAGAACATTCCTGCGACTCTGAGCCACGGTGGCCGCTTCATCCAG
TACAACATCTTCGGCAACATATTTGAAGTCACCGCCAAGTATAAACCTCC
CATCATGCCCATCGGCAAAGGCGCCTACGGCATCGTCTGGTAAATTCACG
GAAAACAAATTTGCATTTTTTTGTGAATTTTTCTTGGCTATCTGTAATCA
GAGCACTGTTGTTATTTTAATCGAATTTATCTGTAATCAGATTTTGTTTT
TATTTTAATCGAATTCTATTCTGGATGTTTTTATTTTCTTTCTTCTTATT
TAGTTCGGCTTTGAATTCGGAGACTAATGAGCATGTGGCTTTAAAGAAGA
TTGCCAATGCTTTCGACAACAAAATTGATGCGAAGAGAACTTTGCGTGAA
ATCAAACTTCTTCGCCACATGGATCATGAAAATGTCAGTCACTTTGTTTT
TGATTCTTTTTTATTTTTTTTCCTACTATATATTTTCAATGACTTCATTT
GAGAAAATGTGAACTTTGGTTTAACTTGATGTTGTGAAATTGTTTTAACG
ATTTGCGATGAACTACATAAAGTAATTCTCTACGTGGTTGTTAATTGCCT
ATTCGAACTTTGATTTCATACCATTAATGGTTGACTAAGCATTTGGTAAT
CCTTGATGCCAACTATTTTGAGCATGGAACTACCTGTACCTTTAAGCATT
TGTTACTAGATTTGGTACCTTTAAGATCACAAATGAAGAAGTGTGAAGAT
AATGTTGAATTTGTTAATAACTAAGGATCAATTTTTTTACTGGTGCAAAC
TAAGGGTATTGTTGACATAGAAGTTAGAAATACCTGTCGTTATTATAAGC
TGGATAGTAAGGTAGACTTTATGAGAAGAATTTCGCCTTCCCTAATTTCG
AAACAAGGAATCAGATCTCATTAAAGGTTCAAAGAGAAAGGAATGTAAAT
TCGAAGTGAAGAACACAGATCTTGTGGAGGGGAAAGTTTGTATGGTGGTT
GACATCAATGGTTTTATGTTAGTGTCTTTGTGACTTCGTGTGTGTGCACT
GTGCGTGCATGTGTATGTATATGGAGAGAGAGAGTGAGAGAGGGAGTTTA
TTTTAGTAGATTTTCATATAATGGATCCTTGCACTGTCTGGATAAATGAA
GATGAGGGAAAATATTTCATTAAGAGAAAACAAAAGAAGGTGACAGAATA
TAATTAACTTTTTTTCCTATTTGGGAATTCCAGTAATAGAAAAGATGAGA
ACTTATAGGGCCTGTTTGGTTTGGTTTGAAAAACAATTTTCTGTTTTCAA
AATGAAAAAATTCAACTACTACTCTTTTAACAACACAATTCAATAAACGA
TTTTTGGAAACAATTTTCATAATACTTAGTCTATCACATCTCCTACTTTT
TATAAATGTAATTATAAGTTTTTCTATATCTTCATTTTTAATTTCTCTAC
ATTTTCCCTCTACGACTCTACGACACATTCACCCAATAAATGTCACTATT

ATCTATTTTTTAAACGTCATATTTCAAGCAATGACATCATTTTATTGATT
TTGCAATTTGTGCTATTGATTTTTTATTTTTATTTTTTTTGGGTTCTGTA
AATTTTGCTGTGTGATTTCGTTGTTTTTACAATTTTTGCTGTGTGGACGA
GAGAGAATAAACTATGAGTGGGAAATGGAGAGGAGGGACATTAAAAAATG
GGGGTGGGACTCTTAAATAAAAACTATTAAATTGTGTTTGACTAAAGATT
CATTGCCAAAATAAAAAACCTAAAATATAAAAAATTGAAATGTCACCAAC
AGATGTACTCGGTTTTTATTTGTTTATTTTCCTTTTTGAAAAACAGAACT
CCTAAAGTGTCTGATCCTAACACAGCTAAGGATACCGAGTTGGAATTTTG
GGGGGTGGGGGGTGGGTTTCTTATACATTGATAAAATCACTTCAAATACA
TACTATTATCAAAGCAAACTAACAGAGGAATTAATTTGCTATGGGCTACG
CAACTAAGCATTCTCAGCGGTGAAAAAGCTTATTGTCTCTCCCCCCCTTG
CCTGCACAACCACACACAGTAGCACTTGTGAGCATTTCTAGTCCTACTTG
TTTTCTTCTTGTCCTTGGCGATCATTGAGATAATAACCATCTTTTATGAG
GATTCTCAGTAATATTTCTTTCTCATTTCTTTCAATTACCAATGGTTCAA
AATTTGTATTCATATCTTTCAATAAACAAAGCTTCAAAATTTGTATCTTT
CATTTTTGTTATGGATGAAATTTCAGGAAACTAGATTTGCAATGAACGTT
TGAAAGAATAGATCAAGCAAGAGTGATATAATTAATAGGTCCCTTTTTAT
CAGTATTTTCAATGGACTTCCTTAATGAACATAAATCAGGTCTTTTGAGT
TGAATTAGAAGTACGAATTTTCGTCATTAAGGGAGAATGACGTGATTTAT
TTTGTATGCTTTCTCTCTCTCTCTCTCTCTCTTTGCATTACTTTTTTCAAAC
ATTTGCTTATATAGAGGCACAACTGTATGCTCCGTTGTAGTTGAGGCTCA
AGACTAAGGTTTTGTTTGTTGTTGTTTTTGTTTATATTTAATTGTGTGCT
ATTTCTTTGTACCCTCTTTATTTTTATGTTATGAGACTCTTAGACTACTA
TCAGTTCTATAATTTGTCCCCAAAATAATTATGCCTCCAAATCTCTAATT
CCCAATTCTATGCCTCCTATGCTAGATATATGGTGGTGATACCTTTAAAA
AAACAGTTTACTATCTACACTTAGCTGTCTGTACATTGTTGTTGTTTTAC
ATAAACACAATTTCCACACACTTATCTGTATGCTGTTACACTGTATATTA
TGTGTTTGTTCACCAAAAACTGTGACTTAGATTCCCAATCTTATGCCTCC
ATTGCTGAATATGTTGTGGGGTTACTTATGAAGAACAGTATTCTGTGGTA
ATCATGTAGGTAAGCAATTCATGATACTTCAAACTATTAATAGATTTATA
TGCAATGTATTTTCTTTTGGGCTAATATTATAAGGCATTGACTTATTTTT
TGCTAATTCATGAGTTTATCATCATTATTTCTCCTTTCTACATGTGAAAG
CTCGATTGAGGAGGGAGGCATGAGGTTATCGATCTTATAGCATGTCCATC
TTTTTTGTTGAATGAGGTCACTTTCCTTCATTGCAGGTTGTTGCAATTAG
AGACATAATTCCACCACCTCAGAGGGATTCATTTAACGATGTTTATATTG
CGTACGAGCTTATGGACACTGATCTCCATCAAATCATTCGTTCAAATCAA
GGATTATCAGAGGAGCATTGCCAGGTTCTACTTTCCACATTTAAACATGG
TTAAATTGTATTTTACCCCGTATACCATGATTAAGTATCACTTGTTGAAT
TATACACGTGTACTATAACTAAAGCTGAATACTGGTATTGTCATAGATTT
AATGATTTCTTTCATACATACACATGCCACAAAACACATGATTTTCGTAA
ACGGAGTATTTAGTGGACAAACTTATCCCTCTTATAAGTTTCACTAGTAT
GCTATAGTTGAAAGCAAAAAATTATGGTTTATGTGAAGGGGGAGAGGAGG
CATAGTGAAAGGTGGGAGAGTGGAAAAGGAAGGAGGGGCACGGTGTTGCA
GAAATAGTGGGAAAGGGATATGTGGAGGGCAGATGGGTGTTTTTGGGGGT

GAGGATGGGTGTTCTTGTTGATGGTAGGGAAAGGGGGTGTTGGCCAGGGA
ATCTGAAATGCTGATAAGGCTAAGTAGAAGGCATGATCTGGTGCTTCTTG
AATCTATAGATCTTTTATACAAATTTTCAAATGTATTTTTCCTTTCTTAT
TTATTATTATTTTTTTTTAATGTATAAGCTATGTGTAATATGTATGTGCA
TGGCAATAAAGTTGCATATGAATTGGTATGCAGAAAAATGCAGAAAATTT
GAAGCCTTGTTCCCCCCTCCCCGTTTTCAAGCTTAAATTTAATTTACAAA
GAAAGGTTAATCTACTTGAATCTATTATATAATTTTCAAAGAATATTTTG
TTCATTTTTTTTTTTTGATGTGTAACCCTGTGTAGAGTATGTTTGTGCAT
GACAAGAGCGTTGGTGTGGAAAATATGAAGACTAGTTTGTTTGGTTGTTT
AAGCTAAATTTAATTTAAAAAGAAAGGCTAATCAAGTCATTTTGCCTTTT
TCTTTAACGAAATAGTAGTATTCACCTGAGTCAATAGTATTGGAGAGTTA
TGCCCATAATTTGAACGTACAAAGGGGGCTAAGTTATAGAGGGCCTTAGT
TAATGTGGTCCAATGCAATCAACACAATATGCTATAATTGTTTTGATTTT
TTAAGCTAGTGGGAAAAATATGAAGCCATGTTATTTTGGTTGTTTAAGTT
AAATTTAATTTACAAAGAAGGGCTAATCTAGTGATTTTGCCTTTTACTGT
AACGAAATAGTAGTATTCACCTGAGTCAATAGTGTGAGGGGGGTATNGGT
AAAATGCAATCAAAACTATATACTATAATTGTTTCCGAAGTACATTTTTA
ATGTGAAAGCTCAATTTCGATGTACTGATTTCCTGACAACAGTTGAGAAT
ATGTTGTTTCATAGAAAAGATGAAGTGATTTAAGTAGTTTGTCCCTGTTA
ACAAAGGATGCTAGGTTGTTAAATGGCTACTAATCCAGCCATTATTTGGA
CGAAGTATCTTCCTTCAAATTCAAATCCACAATTTGAATGTTGGTTGTCT
TTGTTTGAAGCAAAAGCCATGTACCTTATGATTATTGTCACATGGAATGG
AGTGTTTATAGCATCATAATTTTAAATTAGTCCACATTAATTTGGCTAAA
TACTGCTAGTGGCGATTTATGTCATGGAGATTTCAATTATTCTGTTGGTC
CTATTGATTGATTAAATATTTATGTCTTGATATATTTGGTGCATATTTTT
GTGCAGTATTTCTTATACCAGATCCTTCGTGGGTTGAAGTACATACATTC
TGCAAATGTTTTGCACAGGGACTTGAAGCCTAGCAATCTCCTTCTGAATG
CAAATTGTGATCTAAAAATATGTGATTTTGGACTAGCTCGTGTCACCTCA
GAAACTGATTTTATGACAGAATATGTTGTTACAAGGTGGTATCGGGCACC
GGAGCTGTTGTTAAATTCTTCTGATTATACGACAGCAATTGATGTATGGT
CAGTGGGTTGCATTTTCATGGAATTAATGGATCGGAAGCCTTTGTTTCCT
GGTAGAGATCATGTGCACCAGCTACGATTGCTTATGGAGGTGCGTAGATC
TTTTTATATTCTCAGAATTCATGGTCATGGACTCATTATGTGAATCTCTT
TTTTGCATTTCATTGTTCACCTTTTGATTTGGTATACCTGTTTTTTGCTT
CTCGAAGTTCGGGATTTGAGAGAATATTTCTAAGCATTGCCTTGAGCTCA
TATTTGGTAAACATGTGCGAACTGGGAAAAATGTCTGTTTTCCTTTTCCT
TTTTTTCCCCCCCTCCCTCTCTCTCTTGAGCTCATATCTGTAAAGCAAGA
TGCATAATTTGTTTTGTCTTAAAATCAGTACTTCGTTGATTATGTACTAC
ACGTTGTTACAAAACCAATCTTTTGCATGGGTAATTGATTTTTCCTTCAA
AATTTCATTCTCAAGTTTGTGTTCTAGTTTGATAAATTACAAATCATCGA
TAGCGATGCTTCTAAAATAATTTTTAAGAGGGTTGATTGCACTTTGTGCC
ACTGTAGTAGAGGTGCGGAGCACTATACCCCCTAAGCCAAAATTTTTGAC
ACTTAACCGCCTTGTACTTTACCGCAAAGAGCAATAGTTAGGGAATCTGT
TAAGATTACAAAAAGAATAAAATGCCCCTGCCAAAAAGTTTTAATTGCAA

ATTTACCATGAGAGTGGATAGTCTTTCTTGGATTGCATTACACTTTGTAC
CGTAATAATAGAAATTAAATTAAACTTATGTCCTAATATATAAATTTTGC
GTAAACAATAATGTCTTAATTGATCATAAAATTTGAATTAAAAATAATAT
GCATTATAAAGAAATTAAATAAAGAAAATAACAAAAAATAATAAATACTT
CCATCCAATCATCTATTATTGTATGATTAAATACAAATAAATAATTTAGT
GTACCTATACATTTTTTTATAAGTAACTTAATTATTAAATTTAAAAAAAT
ATAAATCTATTAGAAAAATATAAAATTACAATAACCAACAAAACCTTCTT
TAGATTTTTTTATCTTTCATTTTTTTTTTTTTTTTTCATTTTGGGAATTT
GAGCCGTCATTTCATTTCACTCTAAAAATTCTTTGTGCACCGAACAACGC
CACCTATTCCTATAAACACCCATCCTGTCTCTCGATCTCCTAATCTCCTT
CAGCTTCCCCAAATTCGTAGACTCATGATGATGATTCCTGGTAAAGCTCT
GAACAATTCAATGGGAATCTTCAACTTCCGGTAGAATCAGTGAATCCCAC
GGACTAAGAAGTTGAAGCCATTAAACAATCCCAAATCCACCAAATCAGAA
AACCCAGATCCGAATCAGCGATCCCAAATCCACCATCTCCTTATCTCCTT
CAACTTCCCCAAATTCGTAGACTCTTGATGATGATTCCTGGTAAAGCTCT
GAACAATTCAATGGATTCGGAATCTTCAACTTCCAGCAGAATCAGTGAAT
CCGATGGACTAAGAAGTTGAACCCATTAAACTATCCCAAATCCACCAAAT
CTGGAAACCCAAATTTAAAAACCTGAAAACTCAAAGCCAGATCTACTGAC
GTGAAGGGATTGGCTGGCCGATGGTGGTGGAAAAAACTAAATTGAAACTT
TGCTGCGATTTGAAAGGTAAGGGGTGGTAGATTTTGCTTTGTTGGTGGGT
TGATCGTGTGGTGGCGACGATGAATTTAGGGGTTTTGACATGGTGGCAGT
GAGTGGACAAAGACGGGGGAGGTAGGGGCATGTTGGTGGTGGTGATGATG
GAGAATGGTAAGGGCTTTTAGCAAAGGGCAATTGAATATATACACTTTGG
TACCCTATGCTAATTATTTATTCACTTTATAACTACAAAGGGCAAAATGG
TCATCGTATTAATATTTTGGTTGAGAATCACTTGCTCGCGCACATAACTG
TTGTGTGGTTTGCAAATGGTGCTAATTTTTACAGATTCTGTCATTATTGC
ACTTTATGGTATAGTACAACACGGTAAAGTGTAAAGATTTTAGTTCAGGG
GGGTAAAGTGCTCTGCGCCTATGCTACATGGGGTAAAGTGCAATTAACCC
TTTTTAAAACTCAAATTTTGCATCTGTCTCGAAGAATTTTCCTAGATACA
TTTTCCATCTTAAAAAATTTCATTAAGGGAGATGAGGTATCTTCATTTCC
GTTACATTGTGCTGAATTATATAATGAATTTGTTTGAAGCACGGGAGCTT
AGGATTATATTTAGTTCACTTGTTACATCCTATGCAGTGGAAATGTCAAA
GTACTTGAGCTAAAAAACATGGGAATGTGAGTCAAATATGATCAGTAATT
TGTTAATCAGTTTCATGTGAACATAAATAATAATAGTCAGATTCGCGTGA
CATTTACCTTGTATGCGACACCCGCCAGTTAATCCTCCATTCCTGATTTT
CCACATTTAGTGACCATTGAGAGACGTACCCTGCATCAGGTTAACCTTAT
GTTCCATCACATCTAATTTTCAAGTATATTACGTGATCACATCTAATTTT
CAAGTATATTACGTGATATGGATACATGATGATTTATACTTCTGTATGAT
TGGAAAACTTCAAACCTTGCAGTTGATTGGCACCCCATCAGAGGCTGAGT
TGGGGTTTCTGAATGAAAATGCAAAGCGATACATCCGGCAGCTTCCTCTC
TACCGTCGGCAATCATTCAATGAAAAATTTCCACATGTACACCCTGCTGC
GATTGATCTCATTGAGAGAATGTTGACTTTTGATCCGAGACAGAGAATTA
CAGGTGAGAATCTCCTAAGTCTGGAAACACTTATTTTTTTTTATTGTTAT
TGTAGCTATTGTATACATTAAATTTGCAATTTTTTTATCATTTCACTGTC

AGTTTGCCCCCAATTAGGAATTTGTGACCACAATTGTTCTATTTAACCAG
CCCTAGTATCTAATTATTATGAAGTTAACCTTGATTGCGAACCTTAAATT
TTAAATTTATATAATTAACCCTAATTTATTTGATAATTGGGGGCTCTTGA
TAGGGCCTCATATTTTATAACATTCTGCCCTGATTTGCCCTTATTATTTT
AAGCTTGTATTTTAGGGCCCTCCCTTTCTTTCTGATTTGCCCTTCCAGGA
GTTTTATAATGATTCCAAATGCAAATGACTACTTGAATATAAATCAAAAC
CCACCAAAATATTGAATTCAACTTGTCCGGTCAAGATCTCTCTGCAGACA
CCCATTGTGCATTGGTTTTACTGTGATTTAAAAGACTGGTAGATTCTACT
GCACCTGCGAATACTCCACTGGGTTTACTCTGTATTTTGGGGATGAAACT
AGAACTCAGCATAAAAGTGTCTGTGAGAGATAATAAAGGTGAAAATGTAG
AATTGTCCACTAGTTTTATGGTTGGTGAAATTTGACAAGATAGTAGAATG
CCACGGGTATAATTGCGACAGCACTTTACCCTCAAAAATAGAATAATGGA
ATTCGATAAGCTAGTGGAAATTGACTGAGCACACGTGAACCCCTCTAAAA
ACACCAAACATAGATGTCTTATTGTGATAGCTGTAGAATAGAAATTTAGT
GACATTAGGGTTAGAGAATGATTTTAGGAGTACAAGGGCAAAAATTTCTG
TTTAAATCTTTGATATCCCACGAGTGGGATTAGAACAAATGGAGTTGTTT
GTTGGGAGTTTTTTACGTTAATATGTAAAGGGGTTAAAATATGGATTACC
TGATGTAAGTAGTCTATTTTATGGATTATCCCTGATAGTTTTGAACCCCA
AATCTTCCCCAATTTGATTTTATTAACCCTAAAGTTCCTGGACACAATTA
ATGTTTTTGTGATTCTCACAATTTGAACGTATTTCTACTTATTCCATAGT
TAACCTTAAATGCAATCCTAAATTTTGTTTTATATAATTAACCCTAATTT
GAAGTAGTATTATTTAATTTTAAGAATGTTTTGGAGCAAATCTTACTTTG
AACCTTTTAAAGAACATCGATGAAAATACACTTCTTAAAAAAGTTGTACA
AAAACCACACATCCAATATTTTATACCAATAATACCCCGTCCTCTATCTC
TTGGTCAGATTTCTTATCTCATATACCCATACCCTCCATTTCCCCCAATT
CAATGTCCCTTTTTCAAATTGCACTCTGTGATCCTTCCACCATTTTCCGC
TGCACCCCCACCCACCCCTGGCGTCATACGATCGGCACTGTCTGGCCTCA
AGATGGACCTCCTTTGCTGCTGACTGCATCAACTGATGGGAGTTGTGAAA
ATCTGGCTGTGTCCATTGTATTTGTCCTAGCCGTCGCTCATCCACCACCA
CTGCTCCTTTGTTGCTCTCTCTCTCTGTCTCTCTCTCTCTCTCTCTCT
CTCTCTCTCCAGGTTGGAAAATTATTTTCCAGATCTATCACTCTGAGATT
TGATTTTTCTTTTTTGAAGTTTACTTCAGATTAATAGTTGTTTCAGAAGT
TTGCTATTTCTTTGTTCCAGTTCTGCAAAATATGTGATTGCAATTTCAGA
ATTTTGTTGTGCTTTGATTCTGAAATTTGCTGTGTTTTCTTCTGAATTTG
TTGTTTTTTGGTGCGGTGGCAGTTTGCGGTGGTTGTCTGTTGTGGTGGGC
TGCGGTGATTGAAATTTGCTGTGTTTTATTTGATTTGTTTTCTGCATATT
GATTTGCTACTGGTTTTGATTTTATTTTGATTCTTTTTGTTATAATGTTT
GCTACACATTTTATGTTTGACCATGCCGACTCGTTTTCCGAACGATCGCT
GCTCATTGGACGGGAACCCCATTCGCCTTTGCCGAAGACCGGGGGAAGCT
TTGGAGAGATGGGGAGACCCAGCAGCTGGTCAGTGGCAGTGGTTAGCGTT
AGTACTTAGTAGTCTCCAATATGGTTAAAAAATTTGCCGTAGATTTTTAC
ATTGGCAACTTTAGAGATCAGAAAATTTGTTGTCAATTGTAGTGGTTGGT
AGTGGTGCTCTTTGGTGTGGTCGACGAAGATTTGATTTGTTGTTGTCGAT
TTTGGTGGTTGGTAGTGGTGCTCTTTGATGTGGTTGACGAAGATTTGAGG

CGGTTCTAAAAGTTTGTTGCAAATTAATATGAACAAATTTTCAGAACATG
TTTGGGGTGTGTTGGGGGTGTATTCTAGTAAAATTAATTCATTTTTTAAT
TAGTTGTGTTCTCGTAAAAGAAAAATAATAGGTCGTGTACTTGTGTTAGT
GTAATTAACAATTGAGCTTTTGGGGCCCCTACTGAATCACCCTAATTTTA
TTTTGTTACTAACCCTAAATGTGCATAGATGATTGGTTGTTGGGAGGTTG
CTCTTGGCAGGGAGACTGAGGCAGGGAGGTGAGGAGTGAGGGCTATAATA
GGTTAGAGAAAGAGGCAAAATAGGGGGGTGGGGGGAGCATGGCTGGGGGA
CAGGCATGTAAATTGGTCAAGGGGGAGCTATGCAGTGGTGGTATAGAACA
CATGGAGGCGGAGGTTTGTAGAGGTTGGGGCATGGGGTTGGTGGAACTGT
GGGCCTGTTCCGGTGGTGTGACTGCAATATTAAACCTTGGCTTCGTTTAA
CGGGTTGAGAGGGAGAGAGACATAGACCTACCTGCGTAATGGTGGTGTGG
TGACTGCAGGGGGGGGTGATGGATTCGTGAGAGGCTAGAGAGAGAGAGAG
AGAGAGGGTTGATGCTGAATGAGGCGAGAGAAGGGGGTGGGCTTAACATT
GGGGTCGTCTTTGGGTTTTGGCAGTTGGATTGATGTGTCTGTTTAAATTT
TAAACTTTAAAATGTCATGATATACATATTTATGTTTTCAATTACATTTC
CTGGATGATAAAGTTCATTGAAGTTGTTGCATAGGATGGATATTACTCAT
AATACTGTAAGTTGATTTGTTTTCTGCTACTAATTGGTTAACATTTAATT
TCTTTATACATTAAACATTATACTCATTTGATATCGTCCATTTTTCTAGG
GCATCACTGTTATTCAAAAATTAGGGTTAATATAGTCCAGACATGAAAAT
TTTGATCAATTTTAGAAACAGATGGGAATAGTGAGATTGCCTGATAAATT
GGTTCATGTCTCAAGAACGATTTAGACTTCTTATAATAGTGTGGTTGATA
TGCTTCAAACCTGAAATAATCCTAGGGGCACAAAAACTTATACTTCAAAA
TTCATGTATCACTTTCCAAAACCGTTATCCTGGATCTGGAAAATCCATTT
TGGACACACTATACATTTTTCCAAATCAACATATCTTGGGCTGCAAATTC
GAAAAGGACTGGAAAAATGATCCCTATGTGATCTTGGGCTTTATTATGAA
TTCTAAATTGTTAATTTTGAACTTCCATTATGGAAGTTAAGATTGCACAC
TAACACCTTAAAATCTCGTCAGGAAATCATTTTGGGTCACGATTTTATTT
CACTTGTACTATCATATTTTGGACTAAAAATATAGTAATTGTTCTGTTGG
TCAATTAATTTGTTTCAGTTGAAGATGCACTGGCACATCCCTACCTAACG
TCCCTCCACGACATAAGTGATGAGCCAGTTTGCACGACTCCATTTAGCTT
CGACTTTGAGCAACATGCCCTTACTGAGGAACAAATGAGGGAATTGATAT
ACAGAGAGGCTCTTGCATTCAATCCCGAGTACCAGTAA
>EUC07090-RA [gene]
ATGTCAACAGAGTTTCAGTTTGACAAGTTATCCATGGATGATAGAAACGT
TGATAATAACGAGGAACACCTCGTGGATGAAAAGGCCTTGCGCAATTTAG
ACATGGCAAAAGAATGTAAAAACAGTGATATTGTGCAGCATGATTGTAGA
AAGTATACACAAGATAGATTTAAGGGGTCCGACATGAAACTTAAGGAAAC
AGACTGTTCAAGCTCTTCATTTTATCATACTGTAGCTCAGGTTGATCCAG
AAATTGATGATGTTGGTGAATGTGAAATTCCGTGGGAAGATTTGGTCATT
GGTGAGAGGATTGGACTAGGTAAATATTGCTTTTTAACCTAGCTTCTACC
CTTCTGCTTTTGTGTCCTTCCATTTCATACTTGATTTTTTCTTATTGCAT
GTCCATATACTAGGGCTTGTGCTAGTATGCTTACGAGAACTGTTCATTTG
CCAGTGTGGAACAATGTCCATTTAGCAAATTTGTGAGGGAATTTTTTTTT
TCCCCTTATAAATAAGAATTATATTTCGCGCACCCACTAATATGCCCTCC

ATCACAATCTCAACCTCCCACCTGTCACAACAACAACTAAGTCTTTGTCC

CACTTAAGTAGGGTTGACTACATGAATCTGTTTATGCCATAGTGATCGAT

CAAGGGGATCATCCCCTCAGTTAGGTTTAATGCCCCCAAACCTCCCACCC

ATGAGAAGGAAAAAAAAAAAAAAAAGGAAAAGCAAAGACATACCTGGTTCT

GAATTTAATCTAATTTTTGTCATTAGTTGTAATTCTCAGTTTTTTTCATA

TTCCTTGGCTATATTAGCTTTTATTGTTTGAGAGGATTATTATCTGTTAA

CAGCAGCTTTCTGAATGTATGTATTCTATTCTAATGTAGGTTCGTATGGA

GAGGTCTATCGTGCTGATTGGAATGGCACTGTAAGTTTTCCTGTTTTTTG

TTGTTGTTGTTGTTGTTGTTGTTGTTGTTGTTGTTATTATTGTTATTATT

ATTATATATTCATCATCTCTGGTCAAAGTAAAGAAAAAGGACAAGTTGAT

TTCCCATCCTCCCCCCGATGGGGTGGGGGGTGGGGTGGGTTTCTGCTTAA

TGGTGGTTGGAAGACTCCAATCTCCTTTGTTGTAAACATAGTGATATCCA

TTCATCTGACAGTGAACCTTTTATTGGTCTATCTGGATAAGAATAAAACC

TATTGGAATTTTAAATTCTAAAGTAATGATTTCCGGTTTATTTTTGTATG

AACTAAAAAATGATTACCAATTTTAGTCAATGATAGTCAGTTTCTCTTGA

GTCACCAAACCCTCCTACACCCGTAAAAGGGACAAAAAGAAAATACTTGA

AGTTCCATTCGGATTCAGTATATATATATATATTATATTTGCAAAGTTCA

TTCCAATGCAGTATTTACCTACTTTTTTTTTTCTTGTCTTCATCTTGTCA

TTCTTGAATTTAAGTATTTTATCCATTCTATCTTTTGTTCTCCTTTTGCA

TCATAGAGTTTTATGGAACGTTGTGTTCTTGCATTACTTTTCCCATACTT

TTTTTTGGAAAGAATTACACTTTTTACTCTAGAAATTTTGTGGGTTTCTG

CTTTTCCCTTGAATTTTCAATTGGTTCATTTCTAGCCTTCAGATTTTGCC

TAGAGAGACACCCTTTGATGGTTACAATGTTCCATTGACAAAAAAATATA

ATATAGATAAAAATGACTGTTTAAAAAAATTGTCAAAATAAAGCCACTCC

CTCAAAAATTCTTTTGTTCTTCTCCCTCCGATTGCTCCAATAAATTGCCA

AAAGGATCATACCTCTAAGTTCTGGAAAATAACTTGGGGGGAATCCTCAT

CTCGAACAATATCTGAAAAATCATGGCATACACCATGATATGCTGACCAA

AGAGCACCATTGAGCAATGGTCCCAAGCAGCTGGACAAATTAAGAGCAAG

TGACAAACTGATTCTTCCTCCTTGCGGCATATGACACGTTGGCAACTTGA

ATACCTCTTCTTGTTAAGGTTTCCACCACACACCAGGAAAAAGAAAAAGA

AAAAAAAAGCAGCTACCATTAGGAATTTTGATGCCACAAGGAAAAGAAGA

CCATATTGGCATTCTTAATTTGTACTTGATCTGGATGCAGGAGGTCGCTG

TGAAGAAATTCCTTGATCAGGATTTTTCTGGTGCTGCCTTGGCTGAATTC

AAGAGAGAAGTAAGTGACTAGCTATTGAGTGACTCTTTTTTCTTTTTATT

TTCTTCTCCCTTTTCAGCTTCATTTTATCTGCGAAGAATTGAGACATGTG

GGATAATTTTATCAGTGCTGGTTTCTATCATGCAAAAACATGCTAACTTC

TTCTCTGACAAGTACCTGTGGCAAGTGACACAAAATCATGCCAAACTATT

TGATTCAATTATGAAATAGAGTAAAACTTAATAAATTAAATTGTGGTATG

GTAGAGTTTGTGAGTGGGGTGTCATGTGATGTTTAAGGTATGGCAATGGT

ACTGGTGATAGTGAACCCTGGGGGCAATGAATGACTGTTAATGCAAGCAT

TTTCTCTGCCCTCTTTACATCAACCTATCTTTCATCAAATGCGTATGAAT

GAAAATAGCACCAGAAAGGGCTCTGTTTTCTTCTCTTCTTTTCTTTTCTT

TCTATGAAAGCGCCAACACATGAGAAGAATTGTTAGATTTATTCGTTAAT

GTGCAACCTCATCTGGAGTTGTGAATGTCTTTCTATTCTTCCAGAACCAA

ACAGACCTGAGACAACAATTTATTTGTGCTAGGAGTAACTTCTTTTACC
AGGAAGGGCTGTGCTTTTTCTCTTTTTCTGTGAAAGCCCCAACACTTGCA
GAACTTATGCGTGCATGTGCAACCACGTCTGGAGTCGTTAATGTCTTTCT
TTTCTGTGTTTAGAACCAACAGATCTGATAGAACAATTTATTTGTGCTA
GAAATAAATTCTTTTGGGTTAAGTTGATAAATTTTTGCCCATATTTCTTA
TGCTAGGTACGAATAATGCGTAGATTACGCCATCCAAATGTTGTTCTTTT
CATGGGTGCTGTAACTCGCCCTCCAAATCTTTCAATCATCTCTGAGTTTC
TGCCGAGGTATATTACTATTTTTCTTTTTCCCTTTATCATTACTTGTTTT
GCTTGGAAAATGAATCACTGCATTAGTATGTTTACTTGAGTCGTTGCCGG
TTCAACAGCTCGTGGGTATTTTTTTAGTGAATTCATTGCAATTGAACATG
ATAAGTGTCCTGCCTCCAAACCATTGTTATTGAAATTCCCTTAGTCTCTC
CTCTCAGGTCAAGGCTATGCGAGGCCCACATGCAATGCCATGCCTAGACT
AGGGTTGGCTCCTTAACTAAGGTGCACAGCTTGTGGATTTGGGCACTAGC
CCAAGGCTAATATACGTGCATGGTTTATCTACCTTTAGAACTTCTGATTG
TATGTGGGTCTGTTAAACTTAGGCAGATAGTTGATATCGTATTTAATCCG
AGAGGAATAATTTGCATAACTAAGCAAAATGGTACTGGCTCAATTTTCAA
GTTTCCATTTATTTTATCATCTGTATTCAAATGCTTATTCGCTTTTGTTT
GATTTTTATTTTTTGGTTAGTTGATTTGGTCAATTGAGGCCAATGGGCAA
ATATAATTGTATTGGCTGGTTTAATCAATGTTTGCGTATAAGAATCTCAT
GCAAATACAATACTTCATTAGTTACTTTTAGAAAAGGAAAAACACTGTAT
GATATGTGTGCGCACGTGTGTGAATATTAGTTAAAAGTGTCTTTATCTAC
AAAACACTACTGATGAAGACTGTGCCTTTCATCGTGCCTCGTGTCTATAC
CCTAGGACTTATTGGTGCCTTGTTGTGATCTGTGTCTTTCATTATGCCTT
GTACTTCTTAGTTCCTCTACAATTTTGAATTTTAGGGTTTGCTTATTTCG
TAGATACCTTGTTATAGGAGTATAAGTTTCAATATGTCATGAACTGATGG
TAGTTATCCTCCATTAAATGGCATCTGTACATTCTTCAAAATTGGATACC
AAGTGCCATGTAAAAGAGGCCACTAAATTCTCTTGGGATGCCTGAACATT
GTATCAAGAGTCAAAATTTTAATAGACAAAAAGAATTCAGCACTTAAATT
TTCAGCACTTAAAATTTTAATTTTGTCAAACAAACTTATGATATTTTCAA
TTCTTAAATTTTCGACTCTTAAAAATTAAGTGGTTATCAAACATGCTCTT
ATATCATTTAGAATCCAGAATATATCATTTTCACTTTTCATGTCCCGATA
TAACTCTGGCTAGCATGTGGGCTTGACTCCTATCCTATATGTTGCCTTTT
TTTTTTTTTNTTTGCGTATTCCAGATCAGAATGAGTCAGTCACCCTACTA
TATTTGGTATTTGTTTTTTCAGAGGAAGCTTATTTCGGATAATTCACCGT
CCTAACTGTCAAATGGATGAAAAGCGTAGAATTAAAATGGCTCTTGATGT
GGTATGAGACCATTGATCCTACATTGTTCTTCTAACCCGAGATTTACTGT
TTATTATGTATATTATTTTGAAGCTCCATTTTGCAGGCTAAGGGCATGAA
TTGCTTACATACTAGCACACCGACAATTGTTCATCGTGATTTGAAGTCAC
CTAATCTATTGGTGGATAATAATTGGAATGTCAAGGTATGCAATATTTTC
AGTTCTGGCCCTGTGTTACGTAACTTTTAATTTTAAGTCTAATCTGATGG
TCAAAGTAGTCTCTTGAACCTGGGACTACTAACTAATTCTAAATTAGCTG
ATAACTGAAAATAAAAAATTTCCAAAAAATAACAGATACAAAGAAGAAAA
AGGAAAACGAAAGATATTGTTCAGAATGAGTTACATGATTATAGAGTGCT
TCCATGGTTGGGAGTGGTTTGGGTGACAATCCTTTATTAACGTCAGATTT

TGCTATGCAGGTATGTGATTTTGGGTTGTCAAGGTTGAAGCATAATACAT
TTTTGTCATCAAAATCAACCGCTGGAACGGTGAGAATCCCAAATATCCAC
TTGTAAATTTATTTCTATTCACTACAGATAAATTTCAATTATATAGTGCC
AAAACAGGATTTTGTACAATTTTGAACACAATTTTAAGAGGAAAAGAGGG
GTGTTCAAGCTCTTTCTCATGATCACTTACCTTTTCTGACATTGGTGTTG
GTCATATTCTTTTTGATGTCTTAGCCTGAGTGGATGGCACCAGAAGTTCT
CCGCAATGAACCTTCGAATGAAAAGTGAGTTGGTTACTTAATCTTCTTTT
TTAATTGTTGTTTAGTGATAATGAAGATTTTCTATTGCCTATTTGGTGGC
TGGGTTGGGGGTGGTGGGACTGCTCATGATGAGGTAAATATTGTGTTCTG
GGTACAGAATGATATAGATTTGCCGATATGACAATAACCTCAAAAGATGG
CATACCTAATAAATCAACAAACCTATACACACAAATTAGTGTATAACCCT
TGCCTGAAGGCACTCATTATAATTTACTTTATTTTTTTACTAAATAATCG
TAATATGTTATGGAGATACATGGAAATGGACTTTTATCTAATTAATTTTA
AAAACTAATTGTAAATTAATTCTAGTTTGAAAAGTATCCATATTTTTAGA
AACAAAATTCAATTTCTTCACCCTATTTTTTTAATTCCTTAAATTAATCA
ATTATAATTGCAACTCTAATCTTATCAAATAACACTAATTAGTACTTGTT
GAATTCCACATCGCTTGTGTTACATGATCCTGAGCAATATATAAAGTCTA
AGGAAATCCTTCTCTTACAAGACGTCTTTAAGAGTGAGTTAGGCCCACTG
AACTCCTACATGGTATCAGAGTCTAGATTCTTATGTGATTTTGGGTGTAG
TGAATCCCACATCAATGTTGGGTCTCCCAATATCTATTCGTAGTAGTTTG
TCAAAGTTCACCATGTGGGGGGTGTTGAACCTACATTGATCTTGGGCTCT
TCGCTTTGTCAAATTCCACCCTACGCGTGAGGGGGTGTGTTGAATCCCAC
ATTGCTTGTGTTACCTGATCTTGAGCAATATATAAAGTGTGGGGCAATCC
TCCTCTTACAAGGCGTCTTTTAAGAGTGAGTTAGACCCATTGAGTTTCTG
CATGGTATCACAGCCAGGCCTTTTGTCTGATGCTGGGCGTGTTGAATCCT
ACATTAATGTTGGGCCCCCCAATATCCGTTCACAGTGATTTGTCAAAGGC
CACCTTGCGTCTGAGGGTTGAATCCCATTATATTTACACATACATAACTC
TTTTAATAATATGATTTTTATACCTATTTTACAAATACAATTTTAGTAGG
GAGATATTTTAATTAATGAGATATATAGTAATATATAGATAGAAGAGAGC
ATTTTGAAATTCATACAATTAAAATATAAAAAATGAGATTGAAGTGAAAT
GGATGAGAAATTGAAACAATAGCAAATTACCTCTTGAAAGGATTTTAGAA
ATAACATGTTTTGAATGGATTTACAAATCCCTTTTATCTCCTATTTTATA
AATGAATAGATTTCATAAATTCCTCTTCAGAGTACCCAAACAAGAAATTT
CTTAAACAAAATTTGTGAAATCCCTTACCTAAATGAAATCTTTCCTCAAA
TTACCTTACCCAAACACAACCTAAGTATTAGTATCTTCCATCCCATTTAC
ATGTGATAAGAAAATGTGTTATATCAATCTGCATATTTCCTCCTATTCTG
TACTTTTGTTCTTGAAAAATTATTTTATGTTTGTTGAGTCTGCAGGTGTG
ATGTATATAGCTTTGGTGTAATTTTGTGGGAACTTGCGACTCTGAGATTG
CCATGGACTGGGATGAACCCAATGCAGGTTGTGGGTGCAGTGGGCTTTCA
AAACCGTCGGCTTGAAATTTCCAAGGAAGTTGATCCCTTGGTAGGAAGAA
TTATTTGGGAATGTTGGCAGACGTAAGTTACAGCAATTCAACAGTTATCT
CATAATAAATAAAATAAAATAAAGGTTAAATTGCAAAAGCTAAAATTTGT
GCACGGCAGCACATTGCCACCTGAAGTTTCAATTTTTTGCACATTGCTCG
TCTAATTGGTCCAATTACATGGTTCTGGACACTCCGGTGGCACTCGTTTC

TGTTGCCAGAAAACTTGGAACAAAAGTAGAATCCTGGGAACAATTATATT
GTGTTTTAAAAATCTAGGAACTGAATAAACACATTTTTTTGTTCTCATAA
TTCTGTTCTATCCCCAATTTTCTAGGAACCGAGTCAGAAATCTGGGAACA
TAAGCATTAGAAACACGCTACTTTTGTTGCTGAAGTGGGGAGAAAAATGC
AACCACTCTTTTAACTTTTTCTAATGTACAAGTCACATGATTTTACCCCG
GGAAGAAAATTGACCATATATGGTCAATAGGGGCATTTAGACACGAAAAC
AAATTTCGGGAAAGAATGTGCAAAAATTGAAACTACAGGGAACAATATTC
GACCTTACATAAATTTCAGGTGTGCAAAGTGAAATTTATCCCAAAAAAAG
AAAATGAAGATCGATTCCTACAACTGATTCTGTTGTCTGTTATTTCCAGA
GATCCAAACTTGCGTCCTTCGTTTGCAGACCTCACGGTGGCTTTGAAGTC
CTTACAACGGCTTGTCATCCCATTGCATATTGACCAACAAAGCTCACCTC
TACCCCAAGAAATCTCAGTAAATTTGACTCCTTGA
>EUC03978-RA [gene]
ATGAGTTGCAGTGAGAAGAACAGGGCAGGGGAAGATTTGGAGTATGAGAA
ATCTGTGAAAGGGGTTTTGTCGAATGGATCAATCTCAACCGTCCTTCAAC
CCACGATCGACGAGAGAGTATTGGTTGACCCGAAATTGTTGTTTATCGGA
TCGAAAATCGGAGAGGGTGCTCATGGGAAAGTTTATGAAGGAAGGTGAGC
ATGAGTTTTGGCATAAATTCATGATTTTTTTTTTCTGCTTTTGTTGTTGC
TTAAATTGCTGAAATTCATGATGTTGTATGTTTTGGGTCAATTGCAATGT
CGTTCAATTGTTTAAGGGTGCGTTTGGGTGAAGTATATGAAAGAATGAAT
TTAATTATAAAAAAAAAAAAAAAAGAGGTGTTCTCGGTGAACAATCCACAA
AATAAAAAATTTTTGTCCATACATTTCCCGCGCTACAGTAATTTTTTTGGA
AGTCTAAAGTTTTTGTACCAAATGAAAATCATTGCAATTTTTACATGTAA
AAATTGCAGTAACTTTAATGTCTTGCAGTGGTTTTGGACTTTTTTGGATT
TTTGACAGAATTATTGTACTTTTGAAAAATTATTGTATCGAGTGGGTTGG
GTGAATAGTGGGTTGCAAAATTTGTGCAAAATAAAGATTTGTAAAATACC
ACACTTGAATGCACTCTTTTAAGTTTCTCTAACTTTATGTAGAATCTTGG
TATAGCTTGAAACCCCAATTTTTTAATACGGAAATGTGTTCTATTCTAGT
TGTGTTTAGCAACTTGTAGAGGTTAAAAACTTGGGCCCGTTTGGTTGTTT
CAAGCGTCCTATTTTATGAATTATATTATTTTTGATGAATTAATTTGTAT
ATTTTTTCATTTACCAATGATTTAATTAAATTTACATATTGTTTGTTTGA
ACAAGTGAAAATCCTAAGTTAGTGTTTGGCACACATAATCCAAACTTTGA
AATTGGAAATAAGATCATGATTTTTATATGCGGTGATTTCTAATTCCAAT
TCCACAGCTTCAAAATTCCGTAGTACCAAATGCNTTAAAATAAACTTTAG
TTAAATTGCACTTTCCTTCCCCCTAACTTCTTTCAATCCTTATTTTAATC
AATTCAACTTTTAAAAGTTTTTGAAATCATAATTTCATCCCTAATATTTT
TAAGTTTTGATTTTTACTCATCAACTTGAATCTCAAGTTTAAATTTATTG
TTTCTAAATTTGCCAATTTGACCCCTAAAGGTTTGATTTGAATTTTAATT
AATCCAATTTCCAACCCGAATTTTTCCATGCTCTAAAATGAAATTATTAA
TGACGTACTTGCTAAGTCGGGGTCTTACATGCACTCTCATCTATTATCTG
TGAGTCATATTCCTCTTCAATGAAGTGTACCTCCTCAAATCCAGAATTAT
TGCTTTGGGATTCGGTATTAAGAGCCTCTTCGAAAGATTTAGTCCCTGAA
TCCTGAGAATGATTCTCTTCATCTTCCACTTTGCTGCTTTCAGTTTCACC
TATTTTCTTTCCTTCATCTTTCTTGAAATTTTCGATCTCTATTTCCATCT

CAGTTTGCGAAACTTGGTGATCATTTTTCACTTTATTTTTGTTTCTTTCA
ATTCCATCCTCTCTCTCTTTCGATTTTTGTTCTAAATCTCTGGTGTTGAC
AACAACCGTAGCATTTTCTCTCTCTGGGATGCTGGGGCTTCTCCCTCATT
AGATTTAAGTTTAGAATTAATCGGTATGATTAATACGAATTTAAGGACTC
ATTAGTTCAAGGATTAATTTGATAGATTAATCTCATCCGTCATTTTCCCC
AACATAAATACCATCACATACTCCGTGTGCGCAGCGAGTCAAACTCCACT
ACCGTCCACATCTACCTTTATTCTCAACCGTCAGATTACCCATAACCTAA
AACACAACTTATCTGCTGAAGAAATAGACGGCCAGGATCTCAGGTTCGAA
AGTCCACGTGTCCCGTTACGTTGGAGACGAGATATTAAAATCAAGACAAA
GAGTCAAAGGTGCGTTTGGACTTCAGCAGTAGCTTTCCGACTCCGGCGAG
TAGAGAGAGAGAGCCCACGGTGGAGAGAGAAAGCACACGGGGGAGAGAGA
ACCAACGCAGTGATGGAATAGAGGTAGGTATAGATAGAGAGCGGTCAACG
ATAGATAAGTATTAAGAAATTCTGATGGATGATTCGTTTGTAATTTCTAT
TCAAATTCCCCCAATTTCGTGAAAGAGGGATGCGATTGGGAGTTTGACTT
TGATTATAGAGTCGATTAAAATCTTCATCTTTCGAGATTCAAGCTCGATT
TTTCATAGAACCTCCCAATTCTAGCTGAAGTTATTTCATCCTTTCGGTAG
CCCTAAAACGAGAACTGAATCTTGTATACTTTTACTGCGTTGGAAACTTC
AATTTTGCTGAATTGGGGGTGATGAAATGGGGGAAAATGTGACGGCTCGG
CATACCGTATAAATTCGGTACAGCCACTTGGTCGAGGATTCCGTGTGAAC
TTCGTTACGAGGTTGCTCTACTGTAAGGGATTGGCAGGGTCTAGGGTTGT
TGAAATCACTGAAGATCATACTAGGGATCTATGGTTGTACGATAGTTTCG
TTGTGCCCAATCTCTCTTTATCTTCAATTTGGTTATCCGCAGGAATAACG
AGGCTTCCAGCTCAAGTTTGAATTTCGAGAAGTTTGAAAAGTAAGTCTAT
TCTCTTCGATTCTGAAACAATCGGTGTGAAATTAGTTGAATTGTACTGCA
AGTAGTTTAATTTCTTCTGAGATGGGTACTAAAGTTGTTGCGAGGAGTGC
GCATCTGTTTGGGAATTCTGATAGATAGTTGAGCTTTATTTGATCGAAAA
AAATGGTTCTGTTATAGATAATTAGGGTTTTTCTTTTGTGTGTGTGAAAT
TAGATGAGAGAGGTTTAAGGGTTTTTAGGGTTGGGAAAAATGAGCAAGTG
GGGATAATCTGGGGAAGGGGGCTTGCAGCATGAGTTGCAGTGAGAAGAAC
AGGGCAGGGGAAGATTTGGAGTATGAGAAATCTGTGAAAGGGGTTTTGTC
GAATGGATCAATCTCAACCGTCCTTCAACCCACGATCGACGAGAGAGTAT
TGGTTGACCCGAAATTGTTGTTTATCGGATCGAAAATCGGAGAGGGTGCT
CATGGGAAAGTTTATGAAGGAAGGTGAGCATGAGTTTTGGCATAAATTCA
TGATTTTTTTTTCTGCTTTTGTTGTTGCTTAAATTGCTGAAATTCATGA
TGTTGTATGTTTTGGGTCAATTGCAATGTCGTTCAATTGTTTAAGGGTGC
GTTTGGGTGAAGTATATGAAAGAATGAATTTAATTATAAAAAAAAAAAAA
AAGAGGTGTTCTCGGTGAACAATCCACAAAATAAAAATTTTTGTCCATAC
ATTTCCCGCGCTACAGTAATTTTTTTGGAAGTCTAAAGTTTTTGTACCAA
ATGAAAATCATTGCAATTTTTACATGTAAAAATTGCAGTAACTTTAATGT
CTTGCAGTGGTTTTGGACTTTTTTGGATTTTTGACAGAATTATTGTACTT
TTGAAAAATTATTGTATCGAGTGGGTTGGGTGAATAGTGGGTTGCAAAAT
TTGTGCAAAATAAAGATTTGTAAAATACCACACTTGAATGCACTCTTTTA
AGTTTCTCTAACTTTATGTAGAATCTTGGTATAGCTTGAAACCCCAATTT
TTTAATACGGAAATGTGTTCTATTCTAGTTGTGTTTAGCAACTTGTAGAG

GTTAAAAACTTGGGCCCGTTTGGTTGTTTCAAGCGTCCTATTTTATGAAT
TATATTATTTTTGATGAATTAATTTGTATATTTTTTCATTTACCAATGAT
TTAATTAAATTTACATATTGTTTGTTTGAACAAGTGAAAATCCTAAGTTA
GTGTTTGGCACACATAATCCAAACTTTGAAATTGGAAATAAGATCATGAT
TTTTATATGCGGTGATTTCTAATTCCAATTCCACAGCTTCAAAATTCCGT
AGTACCAAATGCTACCTAAGATTTCTATTCAAAACCTAGCTAGTAGGTCT
AATATGTAGTTTTGCTTTTTTAAGGTATGGAGATCAAATAGTTGCCATAA
AAGTACTTAACTGTGGAAGCACTTCAGAAGAAAGAGCTTCACTCGAGAGC
CGTTTTGCCCGCGAAGTTACTATGATGTCTAGGGTAAAGCATGACAACCT
CGTAAAGGTGAATCCTTTTTTAACGTGTAATATTCCAAGTTAAATAATTT
TGTCTTTCTCGTTTTCATCTTTTTTTTTTTTCCTTTGGCAATGCAGTTTA
TTGGAGCTTGTAAAGATCCTTTAATGGTGATAGTTACGGAGCTATTACCC
GGAATGTCGCTACGGAAGCATTTAGCAAATATTCGGCCAAAACAATTAGA
CCTTCATGTTGCTTTGAGTTTTGCTCTTGACATCGCCCAGGCAATGGAAT
GCCTGCACGCCAATGGAATTATACACAGAGACCTAAAACCCGGTACCCAC
CACCTCTCTCTCTCTCTCTCTCTCTCTCTCTCTCTCTCTCTCTACCTTTT
ATGTCATATTAGTGTTGAGTCGAGTCGTATCTCATATTGGATGGATTCTA
TGTATGCTAGCTATAAATATATGGTAGTTTCGGAGTTTCTAAGACATTGA
GATGTTGTTCGTTTGATTTTTTGAAAATTGTGGGAAGTTGTTTGGTTTGG
TTTTGAAAAAGAGAAGTATTTTGTAATATTGTATTAAAAATTAGAAAAGT
TAAGTGATGCGATTGATTCAAATATAATGTTAAAAAAAAATGAAAAAATAA
AGTGATACAATTGATTGAAGGATTTATATAATGTTAAAAAGGGGGGATAA
TGATGATGTTTGGTGGGAGAACGTGTGGTAGAGAAAATTTGTAGAGAAAT
AAAAAATAAAATGATGATACTTAAATCTGGGAATCATAGTAATTAGTGAA
ATGGCCTCGTGGTCCTTGATCCACGGGTCAATCTTCATGTGGGTTTAGCC
CATTGGCCACTTACAAGGCCCAAGACCAAAGATATAAAGAAAAAAATAGA
AATTAAATTTATAAAATGATAAAATTAAAAGTAGGTGATGTGATAGATTA
AGAATTGTGAAAATTATTTTGTAAAAGTGTTTTTGAGTGGTGTCGTGGAA
TAGTAGTATTTTGAGGTTTTGAAACCTGAAAAAAATAGTATCTGAGCTGC
AAACCAAACATGACCTGAATTTTTTGCTATGAAAAGTATTTTTTATTTAT
ATTTTTTGAAATTGTGAATATGATTGCAGACAATTTGTTGCTTACTGCCA
ATCAGAAGTCGGTGAAACTTGCTGATTTCGGTCTTGCAAGGGAAGAAACA
GTGACGGAGATGATGACTGCTGAAACAGGAACTTACCGTTGGATGGCGCC
CGAGGTTTGTGCCTTCAATCAATTATTATTACTTATATATATATATATAT
ATATATATATATATAGACACCTAGGAATAGAATAATCTGAGGTTTGTTGA
GGGTAGGTATTTTTTATTTTTAGAAATCCTTACCCAAAATTGAAAGTACT
TTTGTTGGATGATTGAATGATTATATTTCAAATCTATCTGACTTAGAAAG
AAGGATTCAAGCAATCTAGGTTGCATTCGAATAGTGTTTCTGTTTCTATT
CCCAATAAACTTGAAACATAAACAGAAAAATTGAAGCAAAAAAATATGTT
TATGTTTCTGTCATAGATTTTTAGAACAAATTTCAGACAACAGAGCAAAA
CCGTAGGTGGTGTTTATTTAATATTTTTTGCTCCTACATTTCTGTTACTG
TTTTCTGAAAACTTGGAACAGCAACTGAAATATGGGGGAAAAATCCGTTT
CTATTCCGTGTGTCCATTTTTTGCAAACTCCAAGTTTTGTCTTGAAACAA
AATTCTGGGAGCAGAAACACAAAATGACGCCACCATAGTTTCTAGTTTCT

TTTTCTCTATTTCTGTTCCAGGTTTTAAGAGAAATAGAGAAACACGAATT
GTATTAAAACTTCAAATTTCACAACCTCATTAAAACTATTATGCAGTTGT
ATAGTACAGTGACATTAAGACAAGGAGAAAAAAAGCATTACAACAACAAG
GTCGATGTTTATAGCTTCGGCATTGTCCTATGGGAAATACTGACGAACCG
TATGCCGTTTGAAGGGATGTCCAATTTGCAGGCAGCTTATGCTGCTGCTT
TTAAGGTACCACAAAAACAAAGGTGGCGTTTGGTTAGTGTTTCTGTTTTT
CTGTCTTTACTTTCAATAACCTGGGATGGGATGAGATAGAATGGAATGAG
AATTTTGTTTCTGTTTTGGAGTATTCTGGAAATAGAAACATGTTAGGTAA
CAACCATTGTCCTGAAATTGTTTCTCTTAATTTATGAGAACAAAAACAAG
AAACAAGTTTGGTTGTCACATTTATTCTTTTTTTTTTTTTTTTTTCTCTT
TTNNTTTTTTTTTTTTTTTTTTTTTTTCTCTTTAAATGTATGGTTGGCCGA
GGGGGTGGTGGGAATAGTTTTGTGGGAGTAAATAAGAATCAACATTTTTT
TTGTTTCTTAGATTCTAAGGAATTCAAGGAATAGTCCCAAATTACTCCCA
AAATTTTGGGAACAAAAACGTGTTTCCCAAACGCGTTTGTGTACATTAAT
CCCAAAATTTTGGGAGCGGAAACAGGAAGTAGGGAACAGAAACAGTAACC
AAATAGGGCCAAACTGTCTGACAGCATTTGGTTTCATGAAAACCAGAGCT
TTAGCAAATCACGTTTTTGTTCCATGATTTCCTTTTTTTTTTTCATTGCA
GCAAGAGAGGCCGAGTCTTCCAGAAGATATTCACAACGATCTGGCATTCA
TCATACAATCGTGTTGGGTTGAGGATCCCAATATGCGGCCAAGTTTCAGC
CAGATTATCCGAATGCTAAACGCATTTCAATTCACACTTCCTCTGCCTTC
TCCTCCTCCTTCTCCGCCACCGCCCATAAAAGAATCTTCTAATAATAATG
AGGCGTCGGCGGCTACTAGTAATGGGACTTTGACCGTGTTGTCTTCACGA
GCTAGAGGAAAATTTGCTTTTATCCGCCAACTTTTCGCGGCAAAGAAGAC
AAAGCAGTGATACGATATATATATATATATATATATTTTTTTTAATTTTTTT
TTGAAGCCTCTTTATTTTTATTTTTGAAAAACTATTATTTAAAAGAGAAA
AAAGGAAGATGTTGTATAGTAGGTACTATGTGATATATGGAACTCACTCA
AGAAATAACAATGGAGAAAAAAAAAGAGGCTAAAATTGTATAGTTAATTT
TCTTGAATCTTGGTATAATTAGGGTTAGGAATGTTCTTGTGTTTGAATTC
GTTGGTTTTGGGTTTAATAGCAGTTTCTGAAAGTATTTACGTTTTTGGGT
TGAATTTGTTGGTTTTGTTTCTACTTCCAATAAACTGGAATGAGGATTTT
ATTCCCGTTCTAGAGTATTCTGGGAATAGAAACATGTTTGGTAATGAACG
TTGTCCTGAAATTGTTTCTTTTAATTTATGAAAATAGCAACAAAAAACAC
ATTTGGTTGCAACTTTTGTTTTTTCCCCCCTTAGATTCATGGTGACCAAC
AAGGTGGTAGTGGGAATAGTTTTATGGGAGTGAATGAGAATCAATATGTT
TTTTTTGTTTCTGGAAATCAGAAAATACTTCCAAAATTTAAGGAACAGAA
ATATATTTATTAATAGTGTTTCCGTACATTAATCTAAAATTTTGGGGGGC
GGAAACAAAAATCACGGCATAGAAACAATAACCAAACATGGCCTTGAATA
CTTTTATGTAATTAATTTTATGTGTTGAATTTATTTAGTATTTATTTTTT
AAATATTTTTAATTTCATAAAAAAAAATTGTGTATTATTTTGTCCTTGAAC
TAGTTTTTACAATTTTATTTATCTAAAATATTCTCTTTTTGCAATGTATG
TATATAACTATTATTACAAGATTTTATCTTGAATCCTATTTTTATAGAAT
TTTGAATTTTTTTAACTAATTTTAATTAGGTGTCAATTTCTTATTTGATA
GTATATTAAAAAAAAAAAAAAAAAAATCCAGTTTCTTACGTGGCGTTGTCT
CTGTTTTTTTACTTCAATAAGCTGAGGATGAGATGAGATGAAAAATTATA

TTCTAATTCTGTAGTATTTCGAAAATAAAAACATATTTACTAACGGTCAT
TGTTCTGAAATTATTTATTTAATTTATGAGAACAAAAATAAAAAACACT
TTTTGTTGTAACATTTTTTTTCCCTTTTTAAATGTATGGTAGTAGTCAGA
GAGATAGTAGTGAAAATAATTTTGTGGAAGTGAATAAAAAAATAATATTTT
TTTGTTTTTCATATTCTTAAAGAATTCAGAAAATGACTCTAAATTACTTT
AAAAATTTTGGGAAAAGAAACGTATTTACCAAACATATTTTTGTATATTA
ATTTTAAAATTTTGAGAACAAGGATTAACAAGAAAAGAAGATATGTTTAT
TTACCAATGGGCTAACATTTGACAATACAATTTACATTTGCATCCTACTG
GACAATGCAATTTATATTTGAATCCTATTACTATGTAAATTGCATTGTGA
TTTGAAATAGAACCACACATTCAAATGTAGGATTCATTTAGTGAACGGTT
TAAGAAGGATGGTTACCGGCAAATCCTATTAAGAGTCAAAATTACCAAAT
AAATCTTCTTTTAAGCGAGTTATCAAATAGTCATTTGCGATTATTCAGTT
AGATTTGCCCTTACAATCACTTTGATTTATAACATCCTAAAAAATAGGAA
TTCAAAATCATATTATTAGAATTACAATAACTATTGTTTTACACTTTTTT
TTTTTTTTCATTATTTAATTTTATATTCAAATTCGTCTGAATAAGATTTT
TAAAATAATATCACATTCACTAATTTTTGAATTAGCTTTTAGATTCATAT
TAAATTCAAAATAGTACTTTTAAATTTTTAATTTTTTAATATTGTATTGC
ATTCAATATAGTTGTATATTACTTTGAAGCCAAAGCCAATGATATCACGT
TTACCATTTTTTCATCAAAAATTTATATTCATATTAAATTAAACATGCTA
TTTTTAAACCTTCATAGTTTTCACATCGTATTGAATTCAACACGATATTT
TTTTCATGATTTAATTATATATTAATATTCATTGAATAAGATTTTCAAAA
TGATATTACATTCACCATATTTTTCATTAGTTTCAAATTTATGTTAAATT
TAACATGGTATTTCTAGAATTTTCATAGTTTTTCGATTTCGTGGTGAATT
CAACATGGTTCTTCAAATGTATATTTCTTTTGAAACCAATGATACTACTT
TCACTATTTTTTTAATTAAATTTTTACATTCATGTTAAATTCAACATATT
ATTTTCGAACATTTATAATTTTCTATATCATGTTGAATTCAATATGTTAT
TTCAACAAAATTTTATAAATGTACTATATTGCTATTGTAAAAAAATATTAA
ATGTAATTAAATAAAAAATAGATTGAAAAATTTAACACTGTGTTGAATTC
AATATAAAATTTATTATCGTGTTGAATTTAATAAATATTAAATTATAAAA
CAAAGTATATACAAATGAATATTGTTTTTAAATTAATGATCCATCGATCA
TAAATTTGTAATCAAATCAAGCAAAAGATAAATAAAAAAAAAAAAAATATC
GTTATTTTAAGAATATAAATTTTATATTCTTATTAAAAATCATTAAAATT
TTATATTTTAATAATTTATTAAGTTAAATTTCATGATTTACCAAAATGAA
ATAGTGGTGCTTATCTATTACCAATATTATATTATTTTTCAAAATTAAAA
TTATAAAAATGATTTTTTGGAATCCTAATTAATGTTGCTTTAGATATTTA
CAAGAATTAA
>EUC24464-RA [gene]
ATGGTGTGTATGTGTGTTGTTTTACTAAAAAAAAATGAAGGCCATGGCCC
ATTATAATTTTTTTCTCAAAAAAATGGGACTATCTCTAAATATTCCTTAA
AAATAAAAGAAAAATAAAGGCCCAGATATAAGAGGCTTAATGGGCGGCCC
AGCAACTGTTCTGACTTGTCCGAACAATGTCAAACCTTAAAAAACATTTA
AACCCAATTTCACTAAAAAAAACCATTCATTATAAACACAGACACGTCAT
CACTTGTACGAGGATGAAACTGAAAAAAACCAACGGAAGAAACCAGTGAT
CATGGGCTGAAGGAAAGCGCTCTAATGAATCGAGTTTTAGCTCCAATTGT

AACTGTAAGTGATGACCGATCCAATTACTCTTTCTTCTTCCAATGTCTGA
ATCGTTTTTCTCTTTTCGATTTCTTCAGTCATATGTTTCTGAGATTCCTA
TGTTCGGTGGTTCGATCAACATTAGGGCTTCAATTTTGTCCGAAAATTGT
TGATCTTGAATGATTCTTCTCTTTGATTTGTGTTAAGACATTGATTTGTT
TGCGGAAAAAATGGCTGGTTTGGAGGAATTGAAAAAGAAGCTTGTCCCATT
GTTTGATGCCGAAAAGGGATTTCCATCTGGTCCAACGTTGGACCCTTCCG
AATCTTACATGGTAAGAACTAGCTTTCATATGCTGAATATATATATATAT
ATAGAGAGANTTGCGGAAAAAATGGCTGGTTTGGAGGAATTGAAAAAGAAG
CTTGTCCCATTGTTTGATGCCGAAAAGGGATTTCCATCTGGTCCAACGTT
GGACCCTTCGGAATCCTACATGGTAAGAACTAGCTTTCATATGCTGATAT
ATATATATATATAGAGAGAGAGAGAGAGAGAGTGATGTATATATCATATGTT
TGTTGTTGTGTAAGAAGGTATCAGATGGAGGGACTGTGAATTTACTGAGT
AGATCTTATGGAGTGTACAACATTAATGAGCTTGGATTACAAAAGTGCCC
CTCATGGCCTGTGGATGATGCAGATGAGAGTGAAAAAACATATAGGTGTG
CCTCCAGTGAGATGAGGATATTTGGCGCCATTGGTAGCGGCGCAAGCAGT
GTTGTTCAGAGAGCTATTCATATTCCTACTCATAGGATTATTGCCTTGAA
GAAGATTAATATTTTCGAAAAGGTTTGCTTCTTTTTTCTTTTCAAGAGAA
AATAGCATATGAACTAGAAGTGTTCTTAGTTCTTACGACCAAATACTAAG
CTATAACAATTCCTAAAATTGACTGATAGAAAAATAATACAATTTGCGAT
TTGAAGGACACAACAAAATGGGCTCTTATGATCCTTTTGGGTGAAGTATT
TTAAGAATGTATTTGATTTCAAGAAGTGATTCCTAAATTTTTCTTATTTG
CTAAGAAAATGTTCCAAAACCCTTCTCGAAATCAAATACATTAACCAAAC
TCACCCTTATATTAGTTTGTCATTTCCTTAAAAACTGTGGAATCCCACAT
TGATGTTGGGCCCCCAGATATCCATTCGCAGTGGTTTTGTCAAATCCACC
ATGCGCGTGAGGGAGCGTGTTGAATCCCACATGGCTTGTATTCCCCGATC
CTAAGCTATATATAAGTCTAGAGGCAATTCTCATCTTACAAGGCGTCTTT
TGAGAGTGAGTTAGGCCCATTGGGTTTCTACAAAAACATTGAAATTAAGA
AACTCAATATTTATAAATTTTGAAACTTATACCTACGAACTAATACTTTT
GCCCGAAACACCTCCAACCAAATATAACACACACACACACACACACATATAC
ATATATGTTTTATTCAATATCGCCTGAATTTTTTGTAAGCAACATGAATA
TGATTTGTGCTGATCACATATCCATATGCATCAGGAAAAAAGGCAACAGC
TCCTTACTGAGATAAGAACATTGTGTGAAGCACCTTGTTATCAAGGTCTA
GTGGAATTTTACGGGGCTTTTTATACTCCTGATTCAGGCCAAATTAGCAT
AGCTTTGGAGTACATGAACGGAGGGTCTCTAGCTGATATAATTCGAGTGC
GAAAATGCATACCGGAGCCAGTGCTTTCACATATGGTTCATAAGCTACTG
CATGTAAATATATTTCTCTTCAGCTTCACTAGGGTTCTTCTCTTTGCCGA
TTTTTCTTATTATTCAGTCCTCCTTTAAATTAGTTCTTACAGCCAATTCC
TTGATCTTTTTTTCTTTTTTATTTGGTTTAGGGTTTAAGTTACTTGCATG
GCGTTAGACATTTAGTTCACAGAGATATAAAGCCGGCAAATTTGCTTGTA
AATCTCAAGGGCGAGCCGAAAATAACAGATTTCGGCATAAGTGCAGGCTT
GGAGAATTCAATGGCAATGGTTAGTAATTGATTTGTGTATTTGTTGATGA
TATACTTTATATTTTGTTCTTCATTATTAAAAATTCAAAACATCAAAAAA
GCGGGGGCGCGGGGGGCGTAGTTGCTTTTTACTCCCTTGTAATTTTAGCT
TTACCCTTGACTGAAAGTAAAAAATAATATTTTGTTAAAAAAGTATAAAT

TAACTAATTTTTTTATCTATTTTTTTCAATTTAAATTTAATGTTTAAAGA

TAAAGAAGGGATAAATTAGCATCCTTGCTCTCTCTTTCTTTACTTTTAGC

GTTTTTTTTTTTTAACAAGTATAAAAACAAATAAAAACTAAAATAAAGGT

TAATTTTGTTTATATTATGTAAATTTTTTATGGGGAATTTAATTTCACCA

ATATTTTATTATTTTCCGTAAAGTTAATTAATTTAGTATAAGGGGGTAAA

GTGTAGTTTCTTAACTATTTTTTTTTGTTAACTTATGGCCGGGTTGTGAT

TGCTAGTGTGCTACTTTTGTTGGAACGGTTACATACATGTCGCCTGAGCG

AATTCGAAATGAGAATTATTCATATCCGGCTGATATTTGGAGCCTTGGCC

TTGCGCTTTTTGAGTGTGGCACCGGAGAATTTCCATATACTGCTACTGAA

GGACCTGTTAATCTTATGTTGCAGGTTGGATTTTCCATTTTATCTTAATT

TTAGCTTCTTTTTTTTATTTGTTCTCTTTCAAAATCACATACAAGATGCT

TAATTTTACACACACTGAGTTTTCATGTTTTGATCATGGCAGATTCTTGA

TGATCCATCTCCCTCACCATCAAAGCAACACTTTTCAGCCGAATTTTGCT

CATTTGTGGATGCTTGTCTGCAGAAAGATGCAGATGCAAGGCCAACAGCA

GAGCAGGTGAGTGGCTTTAGAAAATAATCCTTTTGGGAAAATTACATTTT

TGGTCCCTAAAACGTAGGGTGGGTGACTGGGTGTCTATGTGGATCCTAAG

GTTTACCAAATTGCACATTTGATCCCTCAAGTTCCACATTTGTTTCAATC

CTGCAAAATTGAAAACGTTGTTAATGATTCTGGACGGAAAATGGGTTTTG

CTCAGCACGTGGCCTTCTAATGAGTATATTTTCATCATTTCATCACTCCA

CTAAACTCAGGACATTTTCCCTTGATCTTTCTCTAACAACAATTCAAACT

TCTTTCATCTGTTAAAAGCAAACAACACCTTGAACAATAGTCCATTTAGA

ATTTGAAAAAAAAAAAATAAGTTTCTTTTTTTTCTCCGTTTAGGCACTATT

TGGTAGTGGTTTTATTTAATGTACTTTTTGTGATGAATACAAGAAAATAG

CTTCTATATATAACCAAAAGTGTGTTTGATTTTTGTAGAAAGGATATCTT

AGTCTTATCCTGGTAAAATCTCTTAATGCAAAAGAATATCATAGATGGTT

GACAAAGTTTTGGCCGAGAGGTCCATCAATCTCTTAGAATATAAATATCG

AAGAGAAAAAAAAAAAATATTATTTTCAAAAAAAAAAAAAAAACTACTTAAA

ATCTTGTTTTTATAATCTTTGTTTTCAAATATACTACTTTTTTACAAAAC

AAATGATGCTTTTAAAAAAAATTGTAACCAAACAGAGCCTTAACTTTTCTT

CGTGTTCATTTACCTCAATTTTTTTCTTTTTTGGAAGCAAACGTGATTGA

TATTAATTTTGTTGTCTTTAATTCAAGAAACTGGTTGAACTTGATATTTT

ATATGATTAATATTTTCTTCTTTTACCACAGCTTCTTTCTCACCCATTTA

TTACAAAGTACAAGGATGCCGGAGTTGATTTAGCTACATTTGTTTGCGGT

GTTTTTGATCCAACACAGAGGATGAAAGAATTGGCAGATGTGAGTAACCT

TTTCATCTCTGAAAATATATATTGTAAAAAAAATAAAATTAAAGAAAAAGT

ACTTCAAATTATGTTTTTTACGTTTTTTATTTTTTACTTTTTTAAAAATA

TTTTTAGATGCAAAACAAACGCTATTTACTTGCTTTGTTGCTTGACCAGA

TGCTTACAATACACTATTACTTGCTTTTTGATGGGCCCGATGACCTTTGG

CAACACACGAAGAACTTATACAATGAACGCTCAATTTTCAGGTATCGAAA

CCTACATGTTTGATAGCACAATCCAGCACTTAAAACTGAATAGATTAAGT

ACTTATCAAAGTGTATTTGATAACAAAATGAAGTAATCACTTAAAATACA

AGTATCACTTAAAAATTATTCAAAATTTTAATTGATGTGTGACTCACTTA

TTTTTTAGCGCTTCTCAATTAAAACCATCAAATATTTATATACCACAACT

ACCATTTAACATCAATACCATAAAGCCTTGTAAAAAAAAATATTAATTGAG

AAAATTGTAAAAAAAAAAAAAAAAAAAAGATTGTTGTAAAAAATATTTTAG
GGACAAAATTTGTACAAAGATAAAAAATTTAAGTTAAAAGGATTAAAAAA
GTAAATATTTAACATATTCTGCACCAGTTATTTGCCATAGACTCCAAATT
TTGCAATCCATATTCACCTAACAAACCTTAAATTCAAATATTTTGAGAAA
CTCAATAATTCTATCAAAAATTCAAACTCACTGGAAAACATGAAAGTCAC
TGTAATTTTTTACATGGAAAATTTGCAGTAACTTTCATGTAGTACAATAT
CTTTAGACTTCCAAAAAAATTATTACATCGTATGAACTGCATGCACAAAA
AAATGAAGTGTTTAGAATGTTTACTAAGAAACCCTAAATTCAACACTTAC
AATTTTAGCACTTACAAGATTTCAACACTTAATTTTCAGTCTCATCAAAC
AGATCCCAAGTAACATAAAGGAGCGGTATTAAGCACTGACATAAATAATT
TGTGTTTGTGTTTTTTTAGCTTCTCTGGCAAAGAATCGATTGGCTCGAAC
GATATATTCACTCGTTTGTCAAGCATTAGAAGTACATTAGCCGGTGAATG
GCCGCCTGAAAAGCTAGTGCATGTTGTGGAAAAACTTCAATGTCGTGCCT
ATGGCCAGAACGGCATTGCGATTCGTGTTTCTGGTTCGTTCATCGTTGGG
AATCAGTTTCTGATATGTGGAGAGGGTATCCAAGTGGATGGATTGCCAAA
TTTTAGGGATCTTTCTGTAAATATCGCTAGCAGGCGAATGGGAACATTTC
AGGAGCAGTTTGTTGTAGAACAGGGGAATGTTATTGGTTGCTATTTCATA
GCCAAACAAGAGCTTTATGTTGCCCAAGTGGAAATGTGA
>EUC24477-RA [gene]
ATGGTCATAGTGACTGAACTTCTTCTTGGTGGGACATTGCGCAAGTACTT
GCTGAATATGAGGCCTAGGTGCTTGGATATGCGGGTTGCCATCGGCTTTG
CACTTGACATTGCCCGTGCCATGGAATGCTTACACTCTCACGGGATCATT
CATCGTGATCTAAAACCTGGTACTAAGTCGTTTGCAATCTTCTTCGGTTG
TTCTAGTTTATTAATGTTAACTGCATTCATTGACTGTCTTTCTTAAAATG
GACACAAACACTTGCCAATTTTTGGGGTTCTTTGAATGCATAGCAGTGTG
GTTTTTCTCAGCAAGAACTTGTATAGTGTCTTTTGGACAGTATTTATGAA
TTGAAGCTTACGCATTATTCATGATTTGTTGGATGCAATAAATATTGTGA
TATGCACTCTATGAACCCCCTGGATCTCCTAAATTTTGATGTTGGACATG
TTCTTTGTGTTGCATAATGCATACCACATTTTCTAAAGATCAAACTACTT
TGTCGGTTCGTTAAAGTAGACGCCTTTTGACAGTAATCTTACCAATTTTT
GCTCAAACTCATCACGAGCTCAAAAAAAATGCTATGTTCGAGTAGACAAA
CTCACACGCACAATATTATAACACACAAAGTATTTCTTAACTATCTTTCT
TCATTTTGATGTTTTTGTGTAGAGAACTTGCTGTTGACAGCTGACCACAA
AACTGTAAAACTTGCGGATTTTGGCTTGGCAAGAGAAGAATCATTGACAG
AGATGATGACTGCTGAAACAGGAACCTATCGCTGGATGGCTCCAGAGGTG
TATATTATATACATATGTGTTATGTTTGCTTTCCATTTTAAAACTTGTGT
TAGGAGGTTCCAATTTAAAACCATATGGTACCAAGTGGAGTTACTTCTTA
GGCACATATAATGGTTTAGGCACTCAATATAGGACAACAATCTAACACCC
GCCCTTAAGTGTGATCACAGAGACGAATTCCCATAAACGTAGCTCTGACA
CCATGGTAAGAGGATCGTATTTAAAACTATATGGTGTCAAGTGAAACGAC
TCCTTTGACTTGTATAATGTCGATGTGGGACAGGAATCTAACAGCTTGTG
TTGAATCTCCCACAATGATGTTGGCCCTCCGATATCCGTTCACAGTGGTT
TGTCAAACACCCTGCCTGCGCGTGAGGGGCGTGTTGAATCCCACATCGCT
TGTGTTACGTGATCCTGAGCTATATATAAGTCTAGGGGTAATCCTCCTCT

TTCAAGGCGTCTTTTGAGAGTGAGTGAGGCCCATTGGGTTTCTATAGCTT
GTGTTGTTACTCAAATGTTATTTTTGTGTTTGGTTTCAGCTTTACAGCAC
AGTGACATTGAAGCATGGAGAGAAGAAGCACTATAACCACAAGGTGGATG
CCTACAGCTTTGCAATTGTGTTATGGGAGCTCATACATAACAAATTGCCA
TTTGAAGGCATGTCCAATCTCCAGGCTGCATACGCTGCCGCCTTCAAAGT
AAAAAATTCTTTCTCCTATTGAATTTTCGTACATTGATATAGTATACAAC
TAGTGATTCTAATAGCTCGTGGCTGTATTTTTTCCAGAACGCGAGGCCCA
GCGCTGAAGATCTACCGCAGGATCTTGCTTTAATTGTGACATCGTGCTGG
AACGGGGACCCTAATTCTAGGCCAAACTTTGGGGAAATAATAAAGATGCT
GCTGCATTACCTATCAGCAATCCATCCGCCGGAACCCATTATTCCTCCAA
GGATATTCAAATCGGAGAATGCAGTTTTGCCACCGGAATCGCCAGGTACA
AGCTCCTTGATGGCGATAAGGGACGAGTCCCCCAAGACGCCTGTAGAAAA
CGAACCAGGAAGTTTCTTCTTCTGCTTTAACCACTGTTACTAA
>EUC01494-RA [gene]
ATGGCACTAGTCCGAGACCACCGCCGCCACCTTAATCTCAGCCTACCTTT
GCCGGAACACTCCGAGCGCCGCCCTCGCTTCTCCTTCCCCCTCCTCCCTC
CCCCTTCCCTCTCCACCGCCGTTCCCTCCTCCAACAACACCATTACAGCC
GCGGACCTCGAAAAACTCCACGTCCTCGGCCACGGCAACGGAGGGACCGT
CTACAAAGTCCGGCACAGGAAGACCTCCGCCATCTACGCCCTTAAAGTCG
TCCACGGCGACAGCGACCCCGTCGTCCGCCGCCAGATCTTCCGCGAAATG
GAGATCCTCCGCCGGACGGATTCCCCCTGGGTAGTCCAGTGCCACGGCAT
CTTGGAGAAACCCGGCGGCGACATCGCAATCCTGATGGAGTACATGGACG
CCGGGACTCTCGACTCTTCCCTCAAAATCAACGGGACCTTCAGCGAGACA
TGGCTCGCCGGCATAGCGAAACAGGTGCTCAACGGCCTGAGCTACCTCCA
CTCCCTTAAAATCGTCCACAGGGACATCAAACCTTCGAATCTGCTGGTGA
ACCAGAAGGGCGATGTGAAGATTGCCGATTTCGGAGTGGCTAGATTCATG
TACCGTTCGCTGGATCCCTGCAACTCATACGTCGGCACCTGTGCTTATAT
GAGCCCGGAGAGATTCGATCCCGACACTCACGGCAGGAATTACAACGGCT
ATGCAGCTGACATATGGAGCCTGGGATTGACGCTGCTGGAGCTGTACATG
GGCCATTTTCCTTTGCTAACGCCTGGTCAGAGACCCGATTGGGCAACGCT
AATGTGCGCCATATGCTTCGGAGAACCGCCGTGCTTGCCGGATAATAGCG
CGTCGGAGGAGTTTCGGAGTTTCGTGGACTGTTGTCTTCAGAAGGATTCA
AGCAAGAGATGGACGGCCTCTGAGCTGCTGTCGCATCCATTTCTGTTGTT
GCGTCAATCTGATAACTTAACGACAAAATCTGATGACGGCTTATCGGCCG
GAAGTATCTCGTTGGCCGGCGACCTAGAGATCTCCGACGATAATCAATAG
>EUC05347-RA [gene]
ATGGGTATTTTTATGCATTTAGTTTATGCAAATGGCTGAGTTGGAGAGAG
AGGGGTTGTGAAAGTCTAGGCGTAATTGAGGGAAGAGAGAGAAATTGATG
GCGCAAAAGAATTAGATTGATTGAAGTTTCCATTCTAATTATCCGTAATT
TACGCAATTTACGCAAAGGTAACAATGTCAATTCCACACTTTTTTTTTTT
TTCCACAAACAAGGCTCAATATTTGGTTGATTCTGGTAACCCTTTAATAG
GATAAAACAATAAAGATCTATATTTGATTTAAATTGTCTTTTTTTTTTAA
ATTAAAATTTTATTAAAAAATATAATATAAAAAACTGAAAATTAAACACG
ATTATTTTTAAATTTTACTTTACCCAAAAAACAAATAATTGTTTTTTTGA

AAAAAAAAACTATTGTACTTGAGTTTAAAACCAATTACTAATTAATTTGG
CACAATATGATAATATTTTTTTAAACTAATTTTCAAAATAAGAAAAATTA
TTTAAAAAAAATATATATATATAGATTTTTTGTTGAAAGTCCACAAAATT
AAAATTTTAACTTATACAATCTACTCGCTACAGTAAAATTACAATAACTT
TTATAACGTACTTTGATTTTCAGATTTTTGACAATAAATTTGGAATTATG
AAAAATTACTGTATTATGTGTATTGCATGAACTAAAATTTTATTTTTTGT
AGACTGTTCACTTAAATTTTAATTTTTTTTTATTCAAAAGTAAAAAACTC
GAAAAATGCCTCTCCGATAGTGTAGAGATAATATTTTAATAATCGGATAA
GACAATTAATCTTAGCAACAAAACTAAAATGATTTTTAGATTTAGGGTAT
TCATTAATGAAAATAAAATAATAGGGACCAAAGGGAAACTAAATAAAAAG
TTTAGGCCACATTGTGTAATTATTCCTTTTACACAAACTGATAGAGATTC
AAGAACTTGTGGCTGATTAACAGAAAACGGTCCTCTCCACTCCTTACCGA
TCCCGCGCTATTTTAAGCAAACCCCTCTCTCTTTCCTTGCTATTTTAGGC
AAACTCTCTCTATTTCACTCTCTCTCAACCTCACAAACAAGAACCCCAAA
TTCAATCACAAACTCTCGTTGACCGCTTCAATTCTTGCTCAAAAATGCAG
CATTTGGAGCTGAAAATCAGAATAATCCACCATTCAGACAAACAGGTAAC
CTCCCGCAGCTCTTCATCTATCTCATGTAATCTATCTCCTTTTCTTATGG
TGTTCTTTGAAAAAACTTTTTCAAGTATGTTTTTGAAGTGAACCCATTTC
TCATCTTTCAATGATTCAGCAATTTTAATGTCACGAACATATATAACAAT
GTTGGTTGTTTTCAAATTTGTGAAAATGTGATTCGTTTCATGATTTGATT
GATCATCCTCGTTTTCTTTTTCGTAAATGTTGAAGTTTGTTAAAGGCCAG
TGTTTGATGGCTACATGCGCGGAAAGTGTTTGATAAAATGCTTCTGAGAG
AGAGAATTACGGCGAAATATTTTCCAGTTGAATTTGACTTTTCTTTTTGG
GCACAATCTCTTTATTACAATCATTTCTATTTGAATTCAACCCTTTTTTT
TTCTTTTCCCCGCTGTTTCTCTCAACCTCTACATTGCTATCAATATAAAA
TCCTAATATTCAAACATATGCCTGTGTTGTTCATGTTCAGCTCCACATAC
TTGTAAGACCATGCCCGGTGAAAGACCTCGTACTTCACGCCCGCGTTTCA
ACAACGGTACATCCAACAACCTCAACCGAGCGGACGAATCGTATCGCGAC
ATCTCAGTTCAGACCGGCGAAGAATTCTCTGCCGATTTCCTCCGAGAGCG
CGTCTCGCACAGAGCGGCATCAGCAGACATGGACGAGCAGATGAAGAACG
GGGTTGGTTTCATCGTTGGTCAGAATCATGAGCTTGTTTACGAAGATCTC
AGCCGTATTCTCGGTATCCGAAGGCGAGATTCCGATAACGCCCCCGAAGT
TTCCGAATTCTATCAGGTTCAAAAAAATTATGATTTTGCCCCTGAAATCC
GAAACCACAGAGATTACAGGTTCAATAATGATATCCAACCGAGAAAAAAA
TCTTTCGAGATCGTGTTTAGTCCGACTGGTCCGCCCCTTCATTCGTCGGA
TTCTCCGAGTTCGCAACAGCCACATTACGGTTTGGGCGTTTCAGACGGTT
CTTCATCCGGAAAGGTCAAGTGTTTATGCAGTTTTGGAGGCAAGATAATA
CCAAGGCCGTCCGACGGGAAGCTTCGATACATAGGTGGAGAGACGAGGAT
CATGTCAATCAGGAGAAACGCGACGTTTTCTGAACTCATGAAGAAGACTT
GCGCGATTTGCAACCAACTCCATACGATCAGGTTCTGTACATATGCATAT
CCATGCTAACTGTTAGCGTGCTAAAGAATTTGGTAAATTCGTAGAAATTT
AAAAATGACAAGATTTCAAAATTTTGAATTTTGAAATTCCTCCACTTTGT
AAATTCGTTGTTTGGGGTATATTTTTTAAAAACTGAAGTTTTATTGATGT
AATTTCACTTCGAGAAGATTTTTTTTTTTTTTAAAAACTTATTTTGATT

TTGTCAATTTTGCCCCTTTTAGTGTAATTCTCTGAAATAATGAAATAAAT
GGTGTAATCAATAAAACAATAATTAAAATCATGAAAAATCGAAATTATTG
ACTATTAGCACCACTATTTTGAGCATATCATTAAATTTAAATTTTATCTA
TGATCACAGGTACCAGCTCCCCGGCGAAGATCTAGATGCGCTTATCACTG
TATCGTCAGACAACGATCTTCACCTCATGATTGAAGAGTTTCGTGACTTG
GACAAATTTTCACAGAGGCTAAGAATATTTCTCATCTCTCTGAACGAATC
TGATGGCGGCTCATCTTCTTCTTTCGAAGGCAAAAATTTGCAGCCAAACG
AGGGCGATTATCACTACATGAACGCTCCTAATGGTATGAGCTCAAGCAGG
GAGAGCCTGCCAAGTCCAAGCCAATTGAAAATTCACGAATTGGATAGTCC
CGATAATCCAAATCTACAAATGTACGACGATCACCCGTTTGGAGGTAATT
TTATGTACACCAACAATCAACGCGAATACAAAAATCCATATTTTGACACC
ACGGGATATTACTATAGTAACCCTGTCAATAATGTCCCCGCGATGAATTA
TCAAAATCAGAACAATTTTTTTGCGGAACTGGAATTGGGTCATGATTTGT
ATCCTCAAAATCATGTCCAGGGTAGCGAGTTTCCTCCTCTACCTCTTTAT
GGCGATTTTGCTAATGACAGGCCCGTGCATAACTGTTTCACGTATTTTTC
GACGAAATCGACATTTTCCGAGAAGAAAAACGATCATTTTTCTGGATCGG
ACATTCCAATTGAGGATTGTCATGAACAGTCAAGTGATGCACAGTTGCAG
CTTGACACAACAATGTCGGGTTTGTCGCAAGATCTGGTAATGCAAAAGCT
AGAGACGGGTGATGAGAAATATCAGAAGGGGGGGGAAAGTGTAAATATGC
TCAAGTTCACTATGAACAACGACCAAAGCATGGAATACATCGACTGGGGG
AAAACTACGATAAATTGGATGGGGAAGAAAGAATCTAACGATCGCGAAGG
AGATGAAGATATTAAGGCCAATGCGAATATTCGATCTCGTGAAAATTCGA
GATCCGGTGTTCGAATCGTTGAGCAAGAACGTGAAGCTCGAAAATCTTCT
TTAAACAATGCGGCGGATCAATCTCCTGGAATGGTTGGGTTTGAACCATT
CTTCACGAATCAGGGTAATGCGATCCCGTTTGCTGAATACGAATCGTGTT
TGAGCTTGGATTGGCATGAAAACGACCGTCACATGATTGATTTGTCTCTT
CCTTCTTCGATCACTGCTATCTTGGGTAGAAAAATTTCTAACAAGGTTGA
AACCGAGGTAAAACCGACCTCGAAAGGCTCGAATTTTGTCGAAATTCGAC
AATCGGGTGATCGAAAACACGTCAGCTTGCAAAAGTCGACATTTAATGTT
GAAGATGTGACTCATATCGTGCCCCATGATGTCGCATCGTCCTCGAAAAT
CGTTCCTTACGTACACGATGGAACCAGTGACGGAAGTACATCGCCTAAAG
GATCGGAAACGGAATCAGAAACCGGAAGCAGTATATCCAAAGAATTTAAC
GTGGAGGTAATTTTTTTATTAGATTGTACTTCAAATAATTTACTTGCAA
TTTTCATCCTCGTTTTTTTAAATTGGTCATTTTTCAGTCAATCTTTTTTA
GGGGGAAAATTATTAGTCTGTTCTTGAAATAATTCACTCATAATTTTTTA
TCCTTGATTTTTCTTCTTTATTTTTTTAAGTCTTTCGAATAAGTTTTTAC
TCCTCAGTTGATATTAACTAGATTGATTGTATTTAGATGTTAAATTTGTA
CTTGATATCATAGTTAAATTCAACTAAAATACTATGAAGGGTTAAAAAAT
AAGTTTGGTAAGGACTAAAAACTTACAATTTTTTATCCTTGCTATTTTTC
CGTTTATTTTTCAGGGAAAAATTATTAGATTGTCTTGAAGTAATTCATCT
ATAATTTTTTATCCTCATTTCCTTCTTCTTTACTATTTAGTCCTTCGAAT
AAGGTTTTAATCCTTTAGTTAATATTAACTAAATTGATACTATTTAGATG
TTGAATTTATATCTGGTATCATAGTGAAATTCGACTAAAATATCGGTAAG
GGCTAAAAAATAAATTTGGTAAAGACTAAAAACTTACAATTTTTATCCTT

GCCATTTTTTTGGTTATTTTCTAGTCCTATTTTTTTTAGGGTGAAATTAT
TAGATTGTCCTTGAAAAAATTCGCGTGTAATTTTTTATCCTTATTTTTTC
TTTTTTATTATTTACTCCTTCGAAAACGTTTTTACTCCTTCAGTCAACAT
CAATTAGATTGAAGCTATTCAGTTGAATTTGTATTCGATTATATAGTTAA
TTTCAACTGAAAAAAATTAAAAAACCCAAAATAAATATTTTTATTTTTCC
TCGAAATGGTTCGAGTAATACTTATGCTTTTTTGTAGATTGCTGAAGTCG
ATGGAAACGGTAAGGAAGAGGCTGCCACTCCCGAAGTTTGTATAGCTGAA
ATGGAAGCAGATTTTAATAACTTGCAGGTTTCCTTTTATCTAAAGATTCT
AAACTATATATGATCCAATGCTCTTCTTCTTCTTGCTTGCTTCTTTTAAC
TGAATGGTTAAGAAATTACATATGCCAGATCATTAAAAACGTGGATCTTG
AAGAACAACAGGAGTTGGGATCTGGCACGTATGGAACTGTTTATCACGGG
AAGTGGCGAGGAACGGATGTCGCTATCAAGAGAATAAGAAAGAGTTGTTT
TGCAGGGGGGCCGGCGGAGAAGGAGCGTTTGGTAGGTTCCAAAATTTTTA
GAATGCGTTTACGCTGATTTGAAAAAAAAGAGGTGATTTCTAAGGCGGCG
TTCTCAATTTTTAAACACATTATTAATAAATATTCTCTTTTTTCTAAATC
AAACCAAACAACTGTTCCCAAATTTCAAAAACTGTTTTCGAAAATGAAAC
TGAGCAGGCTCTTAACATTCGTACTCAATTTTTTTTCAAGAAAATTTTGT
AAAGTCAAATTCGGTCTTCAAATACCTCTGCCAAACACAGTCTTAGTTAT
CTTTCACAATGCTTGAAACGTCTAGCAAAAGTACTTCAATTTTTTCATCA
AATTCAACTTATTTTAACAGATCAAAGATTTCTGGAGAGAGGCGAATATC
TTGTCGAAACTTCACCACCCAAACGTTTTAGCCTTTTATGGGGTAGTTCC
TGATGGACCTGGCGGAACATTGGCTACTGTAACCGAATACATGGTTAATG
GCTCATTACGCCATGTCCTCTTAAGCAAAGAACAGTAAGTTTATCGGTCC
TTTTTGTGCAATCCTAAGGGTCTGTTTGGTTGTCTTCAATTCGCAAATTG
AATTATTTTTTGAATTACTCAATTCATGAATTGTTATAATTTAGTGTTTG
GTTGCAAGAATTAAAAACATTTAAACTAACACTTTATTTTTCTGCAGTAC
ACTTGACAGGAGAAAGAAGCTTTTGATTGCGTTAGACGCTGCTTTTGGCA
TGGAATACTTGCATATGAAAAACATCGTACATTTTGATTTGAAATGCGAC
AACTTGCTCGTAAACCTACGAGATCCACAGAGACCAATATGCAAGGTAAT
TATCAAGCTTTAAACGCAACAATAATTATCAGTTTCTACTTAAATTTGTG
CTTCTTAGTTTCTCGAAGAAAGAAAATGAATAGTTTTTTTTTTTTTTTTT
TCCAGGTTGGTGATTTAGGGTTGTCGAGGATAAAGCATAACACGCTCATT
TCGGGTGGCGTAAGAGGAACTCTTCCATATATGGCACCGGAGTTGTTGAC
TGGTAACAGTGGTCGGGTTTCTGAGAAGGTAAAGGAAATATGGCTATTCG
TTATTTTGCACCTTTTTTGAAAAAATTAAGTACTGTTTGGTAACATTTTT
TTTTAAATACTTTTATATTAGAAAAAGTCAATTGAAAAAAAAAAAAAAAA
CTTACATAAAAGTAAAGATTTAAAATCGAAATTGTGTTTGATAACGTAGA
GATGATGATATCTACTGTTATTCATATAAAAACCTCTTGATTCTTAGTAC
TTTTTTAAAGTGAAAAATAAATATTGCTTCTATTTTAAACCATTTTTTAA
GCGTTACCAAACAGTGCCTTAAAAACTCGAAAGACTGAAAGTATGCTTGA
AATTCAACAGGTTGATGTATTCTCCTTCGGCATTGCACTGTGGGAGATCT
TGACCGGCGAGGAGCCATATGCAAATATGCATTGTGGTGCCATTATAGGT
TAAAAAATTATCTATTTCTTCATCTTTCCCTTTTTTTTTTTTTTTAAATTA
AAAATTTTTATTTTATTTTTCGTCTTTTTTTTTCTTCATGAAGGTGGGAT

TTTGAGTAACAAACTTAGGCCGACTATACCTGAAAATTGCAATCCAGATT
GGAGAAAATTGATGGAAGAATGCTGGTCAGGTAACCCTACAGAAAGACCT
TCATTCACAGAGATAACAAACCGGCTCAGGGCTATGACAACGTCGCTTCA
ACCTAAACGACATAATTTCTCAAAGAGATGA
>EUC01374-RA [gene]
ATGAAGACGATGAAGCCACTGAAGGAGCTGAAGCTCTCTGTTCCAGCTCA
AGAAACACCAATCACCAGCTTTTTGTAAGTCGCTTCACTCCCGTTTTGAT
TCGCTTCCTCTAAGTGATTCGAAGCATGCAATTCCCTAATTCATGTTTTT
TATGCCTCCCTGATATTGAATTTGCCTTCAATTTAGTTTCTTCAATCCCT
AATTTCTAGTTTGTAATCTCATTGATTTCTTATCGAATATTCGATTGCAG
AACTGCCAGTGGAACATTTCATGACGGTGATTTGCTCTTGAACCAGAAAG
GTCTGCGATTGATTTCTGAAGAAAAAGAACCTTGTGTAAGTTTGTGTTTG
AATTGTCATTTGACATGGACGCCTTCAAGATAATAAAAATAAACAATCAC
ATCGTGATATTTCATGTAATTTTTTTGTTTTTTTTTGGTCTCAATTTGA
TCATCAGAATCATCGGATGCCTTGAGTGGACAACTTTAAACCATTTTAGT
TTGACTCAGATTCCTATGAAATTTCTAGAGGAATGTTAAGAGACTCAGGA
TTTAAGCTAATGATAATTTTGATGTGCTGAATTTTGATCAAAACATTCTA
TTGAAAGAATTATGACCCATGAGTAGGAGAGGGAAAACTCCATGGACAGA
TTCCATCTTGATGCATCAAGAAATGAGATGGCTATGCATAGCCGCAGTGT
CCCTAAAGAAGAGGATGCTGAAAATTAGAACTCCTACAAATTATGCAACT
TTATCTTAGCAGTATGAAACTAGCTTATCATTATGTACCTTTTTGGAGCC
TTTGACTTGGATAATCAACCGAAGAGAAGGGCTGGGGGTGGGGGGGGNCT
CCCATTTATTCATTGGATTACCATGTTAATTAATCATTGTTGCTCGTAAC
TTTCTTTCTAATGGTCCAATGTTAGGGTAATATAACCATTCACTTTCTTT
GGCCGGAAGGATGTTTGACTAGCCATTTTCCTTTACCCACAACTTCATTA
ATTGTCATATCCAGTGAAGAAACACTCCTAAACAAGACATGACATCAATA
ATTCTTTGTCAAGAAAGTCGTAAAATCACAGATTCTAATGTGCAGCATAG
GTTTTTCATCTAATTCTTTGTCATTTTTTAGCTTGCAACTCATTTCTACT
AATTGAGGTTGTGGAGTTTGCATACCTTTAATTTATTCAGGTCTTTCTGC
TACCAAACATTAACTCCATACTTCCTCTTTTTGTGGGTCTGTGACACTAG
TAAATTTCATAGTAATCACTAGGCAGCAAGATGCAAACGAGAGAAGCATG
ACACGACACACTGACACTTTGTTGGGAATTTTAGGACAATGATCATACTA
CGTGTTCATTGAGCTGCTTTAGATGGCTTACCAAATGGTTTCAGTGATAG
GAACTGTAGGACACATATACTCCCTTGTTACTACAAAAGTTATTAAGTTA
CTGATTATGTTGGAACTTAATATATGTATGTGATCAGGTCAATCACGAGG
CTACACGTGTGGAACATGGAACCTCTTCTTCTCCCACCAAGAATAGTATG
TATTTAGAAGCTTAGTTGCAGGATCCTTATAAAATGGCCACGTTCCCATG
TCAGAGTCTTGATTCCACGCTTCTTATTGAGAATCTCGCATTCCACGCTT
CTTGCTGAGAATCACGCACCTATCATGTTAGAATCTCACTCCCACCACGC
TCCTATCACAACTAAACCTCAAAATATTTGTTTTTTCAAATATATTAGCT
GAAGATTTAATTTAATATAGAAAAACATGTAATAGGACTTTTCAAAAGAA
TATTATATTGTAAAAATATGTTTTATCAAAAATAAAAATAAAAATAAAAT
AAAATTGGAACACTAGTAAACACATCGTATATTTATTTAAAAAGAGTTGC
ACGCTTCTTAAGTGCTCTAAACATGTGAACCACTTGGACTTCGATGATTC

CGTGCTCCCGCCCACCCTACGTGCAATTAACTTTAGAATTATGAGACAGC
TTTATCCTTCCATGATCTCATTTGTCAAGGCCTGTTTCTTGGTGCCTATA
ATTGATTAACACATAAGGGTTTATTTTGGTTATCCTTTACCCCTTTTTAT
ATCTAAAAGTTGAGCAATGCAAACTCCAGACAAGATATATCCGCTACGTA
GACTTTCACATCTGTGTAATTTCTTAATTATTCAAATTTAAATGTTTGTG
ACAATATAACAGTAGTGCATACTTATAATAGCTTTGCATCGACCGTATAG
TATAAAATGCTGGAGTTTGCAACATATCTATGTTCTTAAGCATCTGAAAA
TAAAATATGATACTAATGAGACTCTTTTTCTTGTCCCCTTGCACAGCCTT
CTGAAACTAAAGAAATTGATCTTCAATTCTCATTGGAAGATCTTGAGACT
ATCAAAGTCATCGGGAAGGGAAGTGGTGGCGTAGTTCAACTTGTTCGCCA
TAAATGGGTTGGAACACTATTTGCCTTAAAGGTTGGTGAGAATCAACTAA
ATTTTAACTTCCTTTTAGTTTATTGAACTGCTAGAGAAGTTCGAATTCTT
GCTTATGGTAACAAATAAAGATATCTTAAAAAACTGTTAAAAATCAGAAA
ATCATTTTGCCTATCATGATGTTTGTTGAATCCCACATCGCTTGTGTTAT
CTGATCTTGAGCTATATATAAGTTTACGGGTAATCTCCTCTTTCAAGGCG
TCTTTTGAGAGTGAGTTAGACCCATTGGATGTCTACATGGTATTAGAGCC
AGACATCTTGTTTGATGTTTGGCGTGTTGAGTCCCACATCAATGTTCGGC
CCCCGATATCCGTTCGCTGTGGTTTGTCAAAATCAACTTTGCATGTGAGG
GGGCGTGTCGAATCCCACATTGATGTCGGTTTGTCAAAGTCCACCTTGCG
CGTGAGGGTGTGTGTTGAATTCCACATCGCTTGTTGTACCCAATCCTGAG
CTATATAAAAATCTAGGGGTAATCCTCCTCTTTCAAGGTGTCTTTTGAGA
GTGAGTTAGGCCCATTGAATTTCTACAATGGCCAAAGTAATTTTGGCTAA
TTAACCAATGCTCCCCATATTGTGAGTAGATTGCCAAAACTGTAGACATT
TTTATGTAGTCCATTATGAATACAGTAATGCGAAACTGTAACCTTACAAA
TTCTCTCTTTGAGTATTCAGGTTTGATATGATTTGTAAGGGACCTTGATT
GAGCATGACACTTGGCTTAATATCTGCTTTGAATTCTGTTATGAGAATTA
CTGTGTTAATTTGAGTGTAAACTCATGCGGAAGTATTAAAATGGAAGCGT
ACCTTTGGGATTCGGAAGACCCATTTACAATCCGTTTTTTGTTTCTTCTC
TTGTTGGCAAAAAAAACATTGATAGAGTATTTCCAATAATATCCACACAC
TCACTAACCAAGGTTACTCAATTTTTTTGTAATTTGATGGAATTAGAAAC
CTTAAAGATATTAAAAAAAACATTGATAGAGTATTTCCAATAATATCCAC
ACATTCACTAATCAAGGTTACTCAAATTTTTTGTAATTTGATGGAATTAG
AAACCTTAAAGATATTAATGCCTGTTTATAAAGAGAATTCCAAAGGATGT
GTCCTTTCCGATAGATATCTACCGAATAGATGTAAACTTTTAGACCAAGA
GCCGCACCCATTCACATGGTAGCTATTCAAGGAACTCGGGTCCAATGTGG
GGCGCTCCACGGGTCGACCCGACCCAGTCCATATTCCAACATAGAGTAAT
ACTGAATTTTCTCCAGATGAAACTATTTCAACTGCCTTACTAGGAAGTTC
AATCTATATTTGTCACAGGTAATCCAGATGAATATACAAGAGGATATTCG
AAAACAGATTGTGCAGGAGCTGAAAATAAATCAAGCATCACAATGCTCTC
ATGTTGTAGTTTGCTACCACTCTTTCTATCACAATGGAGCTATCTCTTTG
GTGCTTGAATACATGGATCGTGGATCGTTGGTAGATGTAATCAGACAACT
CAACACAATTCTTGAACCATACCTTGCCGTTGTTTGCAAACAGGTGTGTG
GTTCTTTTATCTAGAATCAAAGATGAGACCTGTCGCACTCTTTTTAATTT
TTTTTTTAACAATTTCAAAAATATATTTCCATCTTATCATACTCCATGCT

TTTTATTTCAGGTCTTACAGGGTCTTGTGTACTTACACCATGAGAGACAT
GTAATTCACAGGGACATAAAGCCTTCCAACCTGCTTGTAAATCACAAAGG
TGAGGTAAAAATCACGGATTTTGGTGTAAGTGCAATGCTGGCGAATTCTA
TGGGACAGCGAGATACATTTGTTGGAACTTACAATTACATGGCGGTAAGT
GACATTATCCTTCTTCAGTATTCAAAGACAATATCAGTTATGGCCAAGTG
ATATTTTTTCCACAAAGATCATTTTTTATAGTAAGCACCATTTGGTAATT
CTTTTTTAAGTACTTTTTATAATAAAAAAGATTAATTGAATAAAAAAGTA
GTTTTACTTAAAGACAAAAGTAAAAAATCAAAAACGTCTTTGATAATGTA
AAGATAATGTCTTTGTGTTATCCATATAAAATATTTCTTAATCTAAAAGA
ATTTTTTAGATGGTTGAAAGTATTTGGGTGGAAAAGTCTTAGTCCATTGA
TCTCTTATAATATAATTTTTTTTCAAAAAAAGTATCTTTACCTATGAAGC
ACGAGTATGGGTTTGGGGGCGCTAGTTTGGAGATTAGGAAATTTTTAAAA
TTCTAGGATACGAGTGTGGCAGGGTTATGTTCTTCTGTTCATGTGTGTAT
ACACACACATGCACATAAATACAATAACAATATAAACAACAGTGAAATAC
ATAATAAAGAGTTATTTAAAATTTCAATATTATTAAACATTAGTTTTTAA
CCAAATATTCTACCCGAAACAAAATTGTGATAAATATTCTACTTCCTTTC
ATTGTTAATTAAAAAATCTCAACCCACAAAAATTCCCCATTGATTTCTAT
TTTAATGAATTTCCGGTGCTTGACAAGCACCCTGGAGAATCCCTGGTGCA
CCAACTCAAAACAATGTTACTTTTTTTTCCCCGGTGTCAAAAAAAAAAAT
AAAAAAATAAAATAAAAATAAAACCACTTAAAAAGCCGTTACTAAATGGT
GCCTTACTTTCCTGAGAATGTATTGGATTATTCTTTGTACTTGGTGTGCA
ACATGGCTTGTACCAAAGCTAATTCAATGTATTTTTAACTCTTCTTTTAA
GCCCGAAAGAATTAGTGGCAGCAGCTATGACTATAAGAGTGATATCTGGA
GTTTGGGCATGGTGATACTTGAGTGTGCTATTGGACGTTTTCCTTACATA
CAATCTGAAGACCAGCAACGCTGGCCAAGCTTTTATGAGCTGTTAGAGGC
AATTGTGGACAGTCCACCGCCTTCTGCTCCTCCTGATCAATTTTCCCCGG
AGTTCTGTTTGTTCATCTCAGCCTGGTAATAATAAATCTACCTTCATCTT
GCATATTGCAAGCAAACGGCTATAGATTTCACTAAACACTTTAGGTCCGT
TTTGTTTCCGTTTTGGATACAATTTTTTTGGTTTTCGAAAACCAAAGATT
TAACTATTTTTTACTTTTACGAAATCACTTAAAAATTATTTTAAGGTAAT
ATTTCAAAATTCTTAATCAATCTTATTATCTTTTTTTTGAATATAAAATA
CTTCTATTTTTTTAAAAATTACTTTTCATTTTTTTTCTCTACTCATTTTT
CTCTGGCACTATATTTTCAATGGATCACATCAATAATCCCATTTCTCATT
TCTAAAATTTAACAATATGATAGCTAGTGCTTTACAATTTCAATCGTGCA
TTTGAACACCATGCACAAACTTGAATGCGCTTTTACGGTGATTTAAATCT
TACACAAACTAACAGTAGTTTAAAACGTGGGTCTGTTTGGTTTACAATCG
AAAACGGTTCTTGTTAATCCAATAAAAAAACATTGTTTCTGGATATAGAC
TATAGTTACCAGGATCTGTTTGGACACGTTTTGGAAACAGTTTAAGAGTC
GTTGAAAATAAAAACAGTTTGGCATATGCAGCCAAATAGACTAATAGTAG
AAAAGAAACCTTGAAAAATCAAAGATATATAGGAAAAGAAAGATGTAAAG
TGCCGCTGCTGCAATGATTTGAAAGAATATCGTCCTAAATCATTTTCAAA
TCGTTCTTCGTACACTGCATCGTCATTTGTGTTGGATTCATGATTTTTTT
TTGCAGTTTGATAATATCTTGTCATGTATTTCCAATTGATCGTTGTTGTT
ATTTGTATTGATCTATGCATATGTAGCATACAGAAGGAACCTACTGATAG

ATCGTCATCTTTGGACCTTTTGGTTAGTTTGTCTTCTCTCTCTCTCTCTC
TCTCNNNNNNNNNNNNNNNNNNNNNNNNNNNNNNNNNNNNNNNNNNNNNN
NNNNNNNNNNNNNNNNNNNNNNNNNNNNNNNNNNNNNNNNNNNNNNNNNN
NNNNNNNNNNNNNNNNNNNNNNNNNNNNNNNNNNNNNNNNNNNNNNNNNN
NNNNNNNNNNNNNNNNNNNNNNNNNNNNNNNNNNNNNNNNNNNNNNNNNN
NNNNNNNNNNNNNNNNNNNNNNNNNNNNNNNNNNNNNNNNNNNNNNNNNN
NNNNNNNNNNNNNNNNNNNNNNNNNNNNNNNNNNNNNNNNNNNNNNNNNN
NNNNNNNNNNNNNNNNNNNNNNNNNNNNNNNNNNNNNNNNNNNNNNNNNN
NNNNNNNNNNNNNNNNNNNNNNNNNNNNNNNNNNNNNNNNNNNNNNNNNN
NNNNNNNNNNNNNNNNNNNNNNNNNNNNNNNNNNNNNNNNNNNNNNNNNN
NNNNNNNNNNNNNNNNNNNNNNNNNNNNNNNNNNNNNNNNNNNNNNNNNN
NNNNNNNNNNNNNNNNNNNNNNNNNNNNNNNNNNNNNNNNNNNNNNNNNN
NNNNNNNNNNNNNNNNNNNNNNNNNNNNNNNNNNNNNNNNNNNNNNNNNN
NNNNNNNNNNNNNNNNNNNNNNNNNNNNNNNNNNNNNNNNNNNNNNNNNN
NNNNNNNNNNNNNNNNNNNNNNNNNNNNNNNNNNNNNNNNNNNNNNNNNN
NNNNNNNNNNNNNNNNNNNNNNNNNNNNNNNNNNNNNNNNNNNNNNNNNN
NNNNNNNNNNNNNNNNNNNNNNNNNNNNNNNNNNNNNNNNNNNNNNNNNN
NNNNNNNNNNNNNNNNNNNNNNNNNNNNNNNNNNNNNNNNNNNNNNNNNN
NNNNNNNNNNNNNNNNNNNNNNNNNNNNNNNNNNNNNNNNNNNNNNNNNN
NNNNNNNNNNNNNNNNNNNNNNNNNNNNNNNNNNNNNNNNNNNNNNNNNN
NNNNNNNNNNNNNNNNNNNNNNNNNNNNNNNNNNNNNNNNNNNNNNNNNN
NNNNNNNNNNNNNNNNNNNNNNNNNNNNNNNNNNNNNNNNNNNNNNNNNN
NNNNNNNNNNNNNNNNNNNNNNNNNNNNNNNNNNNNNNNNNNNNNNNNNN
NNNNNNNNNNNNNNNNNNNNNNNNNNNNNNNNNNNNNNNNNNNNNNNNNN
NNNNNNNNNNNNNNNNNNNNNNNNNNNNNNNNNNNNNNNNNNNNNNNNNN
NNNNNNNNNNNNNNNNNNNNNNNNNNNNNNNNNNNNNNNNNNNNNNNNNN
NNNNNNNNNNNNNNNNNNNNNNNNNNNNNNNNNNNNNNNNNNNNNNNNNN
NNNNNNNNNNNNNNNNNNNNNNNNNNNNNNNNNNNNNNNNNNNNNNNNNN
NNNNNNNNNNNNNNNNNNNNNNNNNNNNNNNNNNNNNNNNNNNNNNNNNN
NNNNNNNNNNNNNNNNNNNNNNNNNNNNNNNNNNNNNNNNNNNNNNNNNN
NNNNNNNNNNNNNNNNNNNNNNNNNNNNNNNNNNNNNNNNNNNNNNNNNN
NNNNNNNNNNNNNNNNNNNNNNNNNNNNNNNNNNNNNNNNNNNNNNNNNN
NNNNNNNNNNNNNNNNNNNNNNNNNNNNNNNNNNNNNNNNNNNNNNNNNN
NNNNNNNNNNNNNNNNNNNNNNNNNNNNNNNNNNNNNNNNNNNNNNNNNN
NNNNNNNNNNNNNNNNNNNNNNNNNNNNNNNNNNNNNNNNNNNNNNNNNN
NNNNNNNNNNNNNNNNNNNNNNNNNNNNNNNNNNNNNNNNNNNNNNNNNN
NNNNNNNNNNNNNNNNNNNNNNNNNNNNNNNNNNNNNNNNNNNNNNNNNN
NNNNNNNNNNNNNNNNNNNNNNNNNNNNNNNNNNNNNNNNNNNNNNNNNN
NNNNNNNNNNNNNNNNNNNNNNNNNNNNNNNNNNNNNNNNNNNNNNNNNN
NNNNNNNNNNNNNNNNNNNNNNNNNNNNNNNNNNNNNNNNNNNNNNNNNN
NNNNNNNNNNNNNNNNNNNNNNNNNNNNNNNNNNNNNNNNNNNNNNNNNN
NNNNNNNNNNNNNNNNNNNNNNNNNNNNNNNNNNNNNNNNNNNNNNNNNN
NNNNNNNNNNNNNNNNNNNNNNNNNNNNNNNNNNNNNNNNNNNNNNNNNN
NNNNNNNNNNNNNNNNNNNNNNNNNNNNNNNNNNNNNNNNNNNNNNNNNN

NNNNNNNNNNNNNNNNNNNNNNNNNNNNNNNNNNNNNNNNNNNNNNNNNN
NNNNNNNNNNNNNNNNNNNNNNNNNNNNNNNNNNNNNNNNNNNNNNNNNN
NNNNNNNNNNNNNNNNNNNNNNNNNNNNNNNNNNNNNNNNNNNNNNNNNN
NNNNNNNNNNNNNNNNNNNNNNNNNNNNNNNNNNNNNNNNNNNNNNNNNN
NNNNNNNNNNNNNNNNNNNNNNNNNNNNNNNNNNNNNNNNNNNNNNNNNN
NNNNNNNNNNNNNNNNNNNNNNNNNNNNNNNNNNNNNNNNNNNNNNNNNN
NNNNNNNNNNNNNNNNNNNNNNNNNNNNNNNNNNNNNNNNNNNNNNNNNN
NNNNNNNNNNNNNNNNNNNNNNNNNNNNNNNNNNNNNNNNNNNNNNNNNN
NNNNNNNNNNNNNNNNNNNNNNNNNNNNNNNNNNNNNNNNNNNNNNNNNN
NNNNNNNNNNNNNNNNNNNNNNNNNNNNNNNNNNNNNNNNNNNNNNNNNN
NNNNNNNNNNNNNNNNNNNNNNNNNNNNNNNNNNNNNNNNNNNNNNNNNN
NNNNNNNNNNNNNNNNNNNNNNNNNNNNNNNNNNNNNNNNNNNNNNNNNN
NNNNNNNNNNNNNNNNNNNNNNNNNNNNNNNNNNNNNNNNNNNNNNNNNN
NNNNNNNNNNNNNNNNNNNNNNNNNNNNNNNNNNNNNNNNNNNNNNNNNN
NNNNNNNNNNNNNNNNNNNNNNNNNNNNNNNNNNNNNNNNNNNNNNNNNN
NNNNNNNNNNNNNNNNNNNNNNNNNNNNNNNNNNNNNNNNNNNNNNNNNN
NNNNNNNNNNNNNNNNNNNNNNNNNNNNNNNNNNNNNNNNNNNNNNNNNN
NNNNNNNNNNNNNNNNNNNNNNNNNNNNNNNNNNNNNNNNNNNNNNNNNN
NNNNNNNNNNNNNNNNNNNNNNNNNNNNNNNNNNNNNNNNNNNNNNNNNN
NNNNNNNNNNNNNNNNNNNNNNNNNNNNNNNNNNNNNNNNNNNNNNNNNN
NNNNNNNNNNNNNNNNNNNNNNNNNNNNNNNNNNNNNNNNNNNNNNNNNN
NNNNNNNNNNNNNNNNNNNNNNNNNNNNNNNNNNNNNNNNNNNNNNNNNN
NNNNNNNNNNNNNNNNNNNNNNNNNNNNNNNNNNNNNNNNNNNNNNNNNN
NNNNNNNNNNNNNNNNNNNNNNNNNNNNNNNNNNNNNNNNNNNNNNNNNN
NNNNNNNNNNNNNNNNNNNNNNNNNNNNNNNNNNNNNNNNNNNNNNNNNN
NNNNNNNNNNNNNNNNNNNNNNNNNNNNNNNNNNNNNNNNNNNNNNNNNN
NNNNNNNNNNNNNNNNNNNNNNNNNNNNNNNNNNNNNNNNNNNNNNNNNN
NNNNNNNNNNNNNNNNNNNNNNNNNNNNNNNNNNNNNNNNNNNNNNNNNN
NNNNNNNNNNNNNNNNNNNNNNNNNNNNNNNNNNNNNNNNNNNNNNNNNN
NNNNNNNNNNNNNNNNNNNNNNNNNNNNNNNNNNNNNNNNNNNNNNNNNN
NNNNNNNNNNNNNNNNNNNNNNNNNNNNNNNNNNNNNNNNNNNNNNNNNN
NNNNNNNNNNNNNNNNNNNNNNNNNNNNNNNNNNNNNNNNNNNNNNNNNN
NNNNNNNNNNNNNNNNNNNNNNNNNNNNNNNNNNNNNNNNNNNNNNNNNN
NNNNNNNNNNNNNNNNNNNNNNNNNNNNNNNNNNNNNNNNNNNNNNNNNN
NNNNNNNNNNNNNNNNNNNNNNNNNNNNNNNNNNNNNNNNNNNNNNNNNN
NNNNNNNNNNNNNNNNNNNNNNNNNNNNNNNNNNNNNNNNNNNNNNNNNN
NNNNNNNNNNNNNNNNNNNNNNNNNNNNNNNNNNNGGAAAAGAAAGATGT
GAAGTGCCGCTGCTGCAATGATTTGAAAGAATATCGTCCTAAATCATTTT
CAAATCGTTCTTCGTACACTGCATCGTCATTTGTGTTGGATTCATGATTT
TTTTTTGCAGTTTGATAATATCTTGTCATGTATTTCCAATTGATCGTTGT
TGTTATTTGTATTGATCTATGCATATGTAGCATACAGAAGGAACCTACTG
ATAGATCGTCATCTTTGGACCTTTTGGTTAGTTTGTCTTCTCTCTCTCTC
TCTCTCTCTCTCTCTCTCCCAAAAAAATGAAGAGAAAATCCATAAAAAAA
AAAAAAAAAGGAATATGAGAACCAAAAAACAAATAAACTGTTTATGTTCT

GTTAGTTTTTTAGAACAAAAGATTGGGAACAAATGAAATTATTCCCTGAT
TTCTGATTATGCTCCAAGTTTTCTGGAAATAGAAAAATAAAAACACTAAA
CAATATTCCACCTAGTGTTTGTTCCTGAAAAGCTTGGAGTATAAACGGAA
ATCTGGGAACAATTCTGTTATGTTCTCCAAAATATGTGCTAAAAAACTGA
GAGCTAAACAGAGAATTTTTTTTGGGAACAAAAACACTTAACAAACGGGA
CCTAAGGCCTGTTTGGTTACTGTTTCCGTTCCCTGATTTCTGTTCCCGCT
CCCAAAATTTTGGGATTAATGTACAGAAATGCGTTTGGTAAACATATTTC
TGTTCCCAAAATTTAGGAAGTAATTTGGGACTATTTCTTAAGTTCCTTAA
GAATAGGAGAAACAAAAAAATGTTAATTCCTATTCACTCCCACAATACTC
CAACCCACCAGCCACCACCACGCTGTCGGCCACTATACATTTAAAGAGGA
CAGAAAAGAACAAATGTTGCAACCAAACACAGGAATAAATTAAAAGAAAC
AATTTCAGGACAATGGCTGTTACCAAACATGTTGCTATTCCCAGAATACT
CCAGAACGACAATAGAATTCCCATCACAGTTTATTAGAAGCAGAAACGCT
AACCAAACGCCACCTAAATTTCTTGATATGTCTTAGACATTGCTACTCTA
CTATATATTCTATTACTAATCTTTAAAATTTGATTTTCAGAATCACCCAT
TCATCAAGAAGTTCGAAGACAAGGACATCGATCTGGGAATTTTGGTAAGT
ACCTTGGATCCTCCTACAAGTTTTCCAAGGTGATCATTTGAACCCCATCA
CATGGACACACAACTCCCCCCACATACTTTAATGTCTTTAGGTGGAGCAA
GTATTGGAGTTTGATGTCCAAGTAGCAGAAGAAATGGCATCTAAAGCCTT
TCTATTTCATGCTGCTATAGTTTTTCTTTGTTAAACATGTTTGACATTGA
ATTCCTTACTATTATAGAAATGACTTGGTGTTTTTATCGAGATAATTTTT
TGTCCATACAGTTTACGTGCGACACTAATTTTTTTAAAGCATAAAGTTAC
TGCATTGTATGAAAATAACCGCATATTTTTAATGTGAAAAATTACATAGA
TTTTCATGTAGTGCAGTGATTTTAGATTTCTGGTAGAATTATGAGACTTT
TCGAAAAGTTTTAGTAGCGAATAATTTGGGTGAATTGTTGCTTGCAAAAT
TTGGAGTGATGACAAAATTTCTCCCTCGACTAAACGTTACCGTCCAAACA
CTTTATTGTTAAATTTATGCAACTTGAAATCACAACCCCTAATCCGACTA
ATTAGATTTCATGCTCTCTCTTCTTTATTCTTTGTTTGTGACTCTCTCTC
CTCCCTTCTTGTCAATTTTCCCAACAAGGAAGATTTATTTTTTATAAATT
TTAGAGAATCTTGTTGAGAGGTCCGAAAATTTAAAAAATCACTTTTTAAA
GATCATGTCATGTGTTAAAAATTACTGTTACAGGACCCTGCAGAGTGATG
GTTTTATAGAATATGATCTTTTACCAAATTTTCCTAAATTTTATTGTACA
TGGGTACATGTTTAATTATAAGTATTAATCATATAAAGATAATATAACAA
GGGTAAATACTAGTCATTTTATATTCTAAAATTATTTGCGATAATCATC
ATAAACACCTATTTTTAATTGCAAAATCACTTCTATTTTTGAATCACGGT
AAAAAAGTAAGGCCAAACACTTACAAATTGTTTTTGATTTTGAAAAGGAA
TCAATTTTATAACTTTTTTCATGAGAATTTTCATCAAAATGAGAAGTAAT
GCCAAACTAAGCCTAAATGTAGAATGGAATTGCATCATCGGAATGTCCTC
AACAACTCTTGTGGGGTTCCAATTGTTGATCTTTAAATCACAGCTCTAGA
AGAAATCGATTTACATCTGGTTAATTTGACACAAGTTTGATGTGTAGTTA
CCGGAAGGGCCTCTACAATGTCAACATCCCAGAGGACCTTATCTCGAGTC
TTAG
>EUC00181-RA [gene]
ATGTCGGTGGAATCGAGCTCTGCTTCAGCTGACCATGGCCACATCAAAGG

AGTACCCACTCACGGCGGTCGTTACGTTCAGTACAATGTCTACGGCAACC
TCTTCGAGGTCTCCCGAAAGTATGTGCCTCCGATTCGGCCCGTCGGTCGT
GGTGCGTACGGAATCGTTTGGTATGAACCGAGAAATCTCTCTCTCTCTCT
CTCGATTCAGTTTCTGCTGATCGAATTACTGTTCGATTTGTGTATATCTT
GTACTTCTCGTTTCCGTGATCTCATAATCGGACTGTCGTTAGTTGTATTG
AACTTGACTAGGATTTGAAGAACTTCAGATTCAGATTTGTTCTTGAAGAA
TGATTTCTTTGTTGTTCAAATGGCTTTCTTTTTTGATTGACTATGTGATT
ATTGAACGTAGAATAGAAACGCGCAGATTAATTTGGTTTTCCTGATCAAT
TGCTTGCAAAATCCTTAAAAATTTCAGAATTCCCTCATCAAAATAAAAAC
CTCTTCCAAATTCTTCACCCACATACACACACACACATGGAAGCTTAAAA
GTGATGATGAATTGGCTTGCTTCTCTGGTACTGGAAGTATGGAAGTTTAG
ATTATTGAATATCAAGATGAAGCAGCCTGAGTGAATGGGGTTGAAGTTTT
TTTAACAAGGTCCGATTCGAGGAAAAGTTTTCTTGCATTATCTTCGTACG
ATGTTTTGAGGGATTCGATATGTTTATTTTCTTTCTTGCATATCCATAAT
GTGGATGGATGATGTTAATTGTTGTTCCTTTGCTTTAAGTGCTGCTATGA
ACTCGGAAACGCGAGAAGAAGTTGCAATCAAGAAGATCGGTAATGCGTTT
GACAACAGGATAGACGCCAAAAGGACTCTACGGGAGATTAAGCTTCTTCG
CCACATGGATCATGAAAATGTATGTTTCTTATATATAACTTCGAATTTCA
GATTTCGTTTCTGTAATTTTATCTCGTTGACGCTTTTGCGTTGCTATTTC
TGTCGTTCGTTTTTGTCATATCTAACAGACTTTTAGAGTATGTTTGGATA
AGGAATTTAATGAATTCGTAGAATTAGAATTGCTGGGATTTTGAAATTTT
CAATTTTAAAATCTCAACATTTGCAAATTTGTTGTTTGGATATATTTATT
CAATTTGAAATTTTATTGATGTAATTTCACGTTAACAGAGAGGAATACTT
CATTCGTATGGATTTACAAAAAAAATAAAAATAAAATAAAGTTCATATTC
GGGGATGTCATTTTATCCACCTTTAATCCCATTTCGAAATTCCTAGAAAT
TTGTAAAATTTCCTTCTTAAAGTAGCCAAACAAGGAATTTTAACTTGTGA
ATTTCAGAATTCAAAATTCTTTATCAAAAATCAAACGCTCTGCCAAAATC
GTTCACCCAAACACACCCTTATGATTGTTTCTTGCAAGTAATATTATCTA
CTGCTAAGCTGCTCTAAAGTTTTTTTTTTTCCTTTTCATGGGCTTGTTT
TCTGTTTCGCCTTTAATCTATTATGATTGCTGCAGGTAATTGCTATCAAA
GACATAATACGCCCTCCGCAGAAGGAAAACTTCAATGATGTCTACATAGT
TTATGAGCTAATGGATACTGATCTTCATCAGATTATCCGATCCAACCAGC
AGCTGGCTGATGATCATTGTCGGGTCAGTCTCTACTTCCCTTTGAACTAT
ACTACATACTAATATTAAAACTTGAACTCTAGTTTTCATGTATTAATCCT
CTATTTGATTATTGAAACCATTCCCTGATCGTAAAATTAGCAAGCAAGGT
ACTTAAAAGTACAATAGAAAGAGTTGGGGCGCGTTTGGGCCAAGGAATTA
GATGAATTCATGAAATTTATAAATGATCGGATTTAAGACTTGTGAAATTT
CAAAATTCCATTATTTGTAAATTTGTTCGAATAGCTTTTGAAAATTGAGA
GTTTAATAATGTAATTCTGTAGCTTCTTTGGTTGCAGTGGTTAAAATGAG
GATTTTTTCACAAAATGAATTTTTGGAATTTTGAATTTCAAAGTTAATTG
TTTGAACAAATGGGCTGTAAGTGGCATCCTCATCTCCATCACGCTTATTT
TCCGAACATGCGTCTTGTTTACCGCCCAACATTTTGAATTATTATAAAAA
TCATTGGTCTTATTACAGTATTGTAGAAATTATCTTACAACTTCAATGAT
ATATTGGTATCAAACGATCACATATCACCCGACTTCATTTCTCTACTTTA

ACCATCGTGCCTTCACTTTATGTGTAATATCATTCCAATTTCACCATTGC
CCTAAATAGTAGAACCTAAATATCTAAATTACATTCTATCAAACATATGG
AGAGCTTTGTTTAAGTACCCTTGAAGCACAAAGCGTCAATAATCGAATTG
TAACATCCATTTTGTAATTCTCATGAATTTGTTCCTGAATTTTAATTTTG
AAACTTCTGAAATCACCTTTTTCAATCAAATCCTCCCTCGAAGTACCTTA
TCCAAGCAGAGACTTCATGGTAGAAAATGGAAAACTATTTCAACTATGAG
AACTCTATTTTATTGAAGTATATATTTCTTGAGCTCGGTTTTCTTTGCTA
ATTATGCAGTACTTCCTTTACCAAATTCTAAGAGGACTCAAATATGTTCA
TTCCGCAAATGTCTTGCATCGTGACCTAAAACCTAGCAATTTGCTACTTA
ATGCGAATTGTGATCTGAAAATTGGAGACTTTGGACTTGCGAGAACAACA
TCCGAAACCGATTTCATGACTGAGTACGTTGTTACTCGCTGGTACCGGGC
CCCGGAATTGCTCCTTAATTGCTCAGAATACACTGCAGCTATTGATATTT
GGTCAGTGGGTTGTATTCTCGGCGAAATCATGACCCGACAACCCCTCTTC
CCCGGCAAAGACTATGTTCATCAGCTAAGGCTTATCACTGAGGTTTGATT
TTGGCCCTTCTAAATTTTGATTCTTTTATCTCCATGAATGAAAAATGACA
ATTTTTTTGGTGATATTTTATATGGATTTTTATTATGTAGTCTTTCATCT
AATGTTTTTAATATTTTTGTCCTGTTTTTAGAGCAACTTAATTTTTTTGG
TTTAGTTGATTTTAACAATGATATCAAATATAAATTCAATATCTAGTTGA
TAAAAACTATCAATTGAATGGATTTACTCTAATTAATATTAACTGAAGCA
CCGAAAGCTTATTTGAAGGACTAAAAAAAAACGGTGATGAAAAATGATAA
GTTAATTATTTTAAGGATGATATAATAACGTTCCCTATTTTATATGCGAA
TGACTTGTTCCGTTTTTGACTTTTTGTTCTTTTTTTGGGGGCGGTCTTAA
AGCTCATAGGTTCGCCCGATGATGCAAGTCTTGGGTTTCTCCGTAGCAAT
AATGCCCGAAGATACGTTAGGCAGCTTCCTCAGTACCCAAGGCAACGGCT
CGTTACTAGATTTCCTAATTCGTCTCCTAGTGTTGTTGATCTTCTTGAAA
GAATGCTAGTCTTTGATCCAACCAAGCGTATTACAGGTATAATGCAGTTT
ACTTCGGTTGGTTGAGTTTGTTTTGTGTTTCTGTTCCCAGAATGTTTTGA
TCTTAGACAGAAAGACGGAAACAAAGGTGGCATTTGTTTAGAAAAAAGAG
ATTCGGGAACAATTTTGTTTTGTTTTGTTCCTAATTTTTTCAAGTAACCT
GGGAGTAGCACAAAAATAATTTTTTTTTCTTCCCATATTTCTTTTTCAGA
AGAAAAAAAGAATCTGAGTACGACAGCACTAAACAAATGGGGCCTTAGTG
TTTCTGTTCTCATTTTTTTCCCTATAAAATTTGGATCAGAAACAGATATT
TGGAAGCAATTTCTCGTCATTTTTTCCTAAAAATTTGAGAACATAACAAG
AAACATATGTTTTCCCCATATTTATATTTCGGTTCCCGGTTTTCTGTGAC
AAATTTGTGAGAAAAGTACAAAAACAGCTGTTTTTTAGTTCCAACTTTAA
ATAATTCCGAGTTCTGCAGAGAGTGACAACGATCCAACTTTCGTATTTAT
TTATTTATTTTTGTTTTTTGCAGTTGATGAGGCTCTCTGCCACCCGTACT
TGGCACCACTCCACGATATCAACGAGGAGCCGGTTTGCCCGAGGCCTTTC
AGCTTCGACTTCGAGCATCCTTCTTGCACCGAAGAGAACATCAAGGAGCT
CATCTGGAAGGAATCCGTTAAATTCAATCCCGATCCAAGTCATTGA
>EUC07900-RA [gene]
ATGCAGCATGATCAAAGGAAAAAGGTAATTTCTCTCGGTTCAAACATAAT
GCATAGATATATCTGCTTTATCCATTTGAATTTGTGTTAGATATTTATCC
ATCTTATCTCATTATAGTTCTCATCACTGATGTAATACAAGCTTACTCTC

TCTCTCTCTCTCTTTCATGTGAGTTTGGGAAGGTAAAGCTCTGCTGTACT
TTCGGAATTATTGTATTCCTTAATCCTGGGTAGGTAGAGAATTTGGCAAG
TTTCATGTCTAATCTTGTCTTGTGCTTAGTTTCCTTAAATTATGGGTCCC
ATTCCCATATGACTACTTTTCTCTCATTCATGAATATGAAATCTGCGTGG
CTTCCTCCCGTGAATGATGGTTGTTAAGCTTTGAACTGAAATATATAACT
CTAAACCCACGGGACCCTTCCCCGATATCCATTCACAGTGGTTTGTTAAA
AGTTCACCTTGAGAGTGATGGGGTGTGTTGAATCCCACATCGCTTGTGTT
ACCTGATCCTGAGCTATATATAAAGCCTGGGGCAATCCTCATCTTACAAA
ACATCTTTTGAGAGTGAGTTAGGCATGTTGAATTTCTACATTGGTGGATC
TTTTTTATGTTTGAATCTTGCTCTACTGTCAATTTGCATGTTTAATCTAA
GTTTTTGTGATTTCTTTTAGTAAGCTAAATAGTTTCAAATCGTATTGCGA
TTTTTCGTAGATTTTGCAGGCCAACTTAAAGCATCTCCGACCATGCTCTT
TATTTTTAGGGTTTCTTAGTCCTCAATTTTTTCCCCTTATTTATAGAACA
ACTATTTTTATTGTCAATATTTTAGTTAACATTAACTATGATATCAAATG
CAAATTTCACTTCTAGTTAACGTTACCTCGACGGATTCAAATTGGTTGAT
GTTAACTGAAGGGCTAAAAACTTATTCAAGTGACTAAAAAATAAAGAGAA
AAAAACTGAGGAAAAAAATTGTAATTGACTTTTCAAAGGAAAATCTACTA
ATTTCAATTTTATTTCAAAACAGCTGGAAAATTATTTTGTCGAACATCAT
TTGGAGTGAGTATTCAAGTCAAGCTCTTTATATTAAAAAAAAAAAAATTAA
AAAAAAAAATAGAGAGAAATTTAATAGTTTTAGACTTATTTAAAAGTAAC
TAGTCTCATTTCACTCAATAGATTTAGCTAGCAAAATGAGTGAGATGAGA
AATAGCTAGCTGAGTAGCTAGTCCATTTTAGAATAAATTTTGTTCAATTT
TTTATTTATTTATTATTATTTTTTTAAATGGCTAAATATTCAAATCTAGT
TGGTCCTTTACATGAATTATATGATGCATTTGTTAAGATGGAGATTCAGG
ATTCCTTATCTATAGCAACAAGCTCTTGAAGAGGGGAAAAAAACTTTATT
TTGCTATATATGTATCGATAACTAATAACCGTTTCGATAACTAATAACTG
TTTCACAAATCAACTCTCTATTTATATGTTAAAATTCACTTTTTTTTTTC
ACTCTCCGATTAATCTGACAATACAGGCATCTGTGGATGTGGACTTCTTC
ACTGAATATGGTGAGGGGAGTCGGTACAAGATAGAGGAAGTAATTGGCAA
AGGAAGCTATGGTGTTGTTTGCTCAGCTTATGATACTCATCTCGGAGAAA
AGGTTGCAATAAAAAAGATAAACGATATTTTTGAGCATGTGTCTGATGCC
ACACGCATCCTTCGTGAGATCAAGCTTCTTAGGTTGCTTCGACATCCTGA
TATTGTAGAAATCAAGCATATCTTACTACCTCCTTCGAGAAGGGAATTTA
AGGATATATATGTAGTTTTTGAGTTGATGGAATCCGACTTGCACCAAGTT
ATTAAAGCCAATGATGACTTGACTCCAGAGCATTATCAGTTCTTTCTGTA
TCAGCTTCTTCGTGGCTTAAAGTACATACACACAGGTTTTTTCCTTTCTA
CAATCTTTTAAGCTGTGTTTAGATGAGGTATTTGAAGAACGAATTTGATC
TTGAAAAAGGGATTTGTAAAATTTTATCAAGAAAATGGTTATTTTATTGC
AGCCTTTTCCCTCTATTTGAATTTGCCATAACCCGATTTTTTTTTTTAAT
CGAATTTTCTCTTCATATACCTCACCCAAACGCACTCTTAATGCTTCAAT
GTGATTCTTTGTATGCATTTCACATGGAAATCTTACATTACTTTGACATT
CAATCTATGCAGCCAACGTATTTCATCGAGATCTAAAACCGAAAAACATC
CTTGCTAATGCTGATTGCAAACTCAAGATATGTGACTTCGGCCTTGCAAG
AGTAGCCTTCAATGATACGCCTACTGCTATATTTTGGACTGTACGTTTCT

TCATATTACCATGGCACAGTGTTACTTTTTAGTCTTTACCCATGAAACTT
CTAGTTGATTATATACTTGAAGAACAACCTTTTATTATTTCCATTCATGT
TTTGTAGGATTATGTTGCAACGAGGTGGTATAGAGCTCCAGAATTGTGTG
GATCCTTTTTCTCTAAGGTGGTCTTTTGTTCAGTGTTTTTTTGTTTCCTT
TCCAAGAAAACTTAGAACAGAAACAAAAAAGCATTAAAAAAAAACTGTGT
ACTTTCTGCTCTGAATTGTTCTAATATTTTGTGAAAATAACGGAAATATA
AAATTATCAATTATTGTTCACGTTAAATTTATGGATTGAATCAAATTAAC
ATTTTCTTTTATTTGTTTTCAGTACACGCCTGCAATAGATATATGGAGCA
TTGGCTGCATATTTGCAGAACTATTAACAGGAAAACCTCTTTTCCCTGGA
AAAAATGTAGTTCACCAGTTGGACATAATGACTGATCTCTTGGGAACACC
ATCTGCTGAAGCCATTGCTAGGGTTTGTTCCTCAATTTTTATTTTATTTA
TTTATTTTTATCATGTTTTCAAAAAAAAAATAAAAGGTGATCTTTAATAGT
TATCATTATGTTCTAAAATCTTTGTTCCCACGCATACAGATACGAAATGA
AAAGGCTCGACGTTACATAAGCAGCATGAGGAAGAAAAGGCCAGTTCCTT
TCTCCCATAAGTTCCCAAATGCAGATCCCCTTGCACTTCGTTTATTAGAA
AGGATGCTAGCATTTGATCCCAAGGATCGACCAACTGCTGAAGAGGTACA
GATATTTACGATTTTTATATACTAGGTGGTGTTTTTTTTTTTTTTTTCGC
GTCTGTTCCCAAAAACTCGGAACTGAAACAAAAATATAGGTATTTTTTTA
TTCTGTTCTGTTTGCTGATTTTTAGAACAAATTTATGGGAATTAATTGTT
CCCGGATTTCTGTTCTGCTCCTGGACTGTAAAAATAGATTTTTAGAAAAA
AAATTATGAACGGTGCAATACTATATTGCTGTTTCTGTTCCCATTTTTTT
TTTGTTCCCACATTTTCTGTCCTAAAAATTTGGAACCAGAACAGGACTTG
AGAACAAAAAGATATATTTCTCTTCCAATTCAGATTTTGTAACGGAAACT
GAAATCCGAGAACAAATTTCTTGGAAAAAAACAGAAACGGTTTTTTCTTT
TCTTTTGTCTTTTTGTTTCTTCTTCTAAAAGAGCTGTGTTCTGGTACAAA
CTGCTTTTAGGCACTTGCAGATCCATATTTTAGGAACTTGGCAAAGGTCG
AGAGAGAGCCTTCGGCTCAACCCGTCACAAAAATGGAATTTGAATTCGAA
AGACGGAGAATTACGAAGGAAGATGTGAGGGAGTTGATATACCGTGAGAC
TCTTGAGTATCATCCCAAGATGCTCAAAGAGTTCTTGGATGGAGCTGAAC
CAACAGGCTTTATGTATCCGAGGTATTTTTCTATTTTTTTCCAACCGATT
TTCATTTTTTAAAAATCATTTTTTGGGTCTTTTTTTGGTTAATTATTTGT
TTTTTTTTTTTTTAGTGCGGTTGATAAGTTTAAGAAACAATTTGCATACC
TTGAGGAACGGTATGGGAACGGTGGAGCTGCTCCTCCTCCCGAGAGACAA
CATGCTTCTTCATTGCCCAGGTAAGCCTTTATTTAAGAAGTATTTCTATA
AGTGGTGTTTGTTTAGTGTTTCTTTTCCCATATTTCTGTTTTTGTTTCCT
CATATCTTGGAACAAAAACTGAAATATGGAAACAAGTGCTCATGGTAATT
CGAACTGGTTCTTTAGATGGCATTTTTAATGTTTCTGTTGACAATTATTT
TGGTTTTAGTTCCCACAAAACATGGAATAGTAACAGAAATCTGGAAACAA
TTCTATTTCATTCTCAGAAATTTGTTCTAAAAATCTGGGAACTGAAACGG
AAATAGAATTGTGTTCCCATATTTTTGTTTGGGAACAGAAACACTAATGT
GTTGTTTGTTTAGTGTTTGTTCCCATATTTACGCTTAAAACTAGCAATTA
AAGCAGAAATATGTGCTTTGACACTTTGTACGCTTGAAGTTTGAACAAAA
TCTCACTTTGGTCCTCATGGTTTCAATTTGAACATATGGCACCTTAAAGT
TCATTAATCATCTCTTTACAGACCAAGACATTGTACCCATTACAATTTTG

GACGGAAAACATTCACCTACCACGCACATGAATATTTTTAAGAGGTTTCA
TGGGCAATTCTAGCCTCATATATATATATATATAGAGAGAGAGAGAGAGA
GAGAGAGAGATTTTGGTAGAAAATATGTAACGGGGAAGGGAAGAGGTTGA
AAATACCCATAAAGTCCCCTTACAAATAGTTGGATGCTCAGAAATTGATT
GTTTGTCGTCTAAAATTGTGATGGAATTATTGATTTGGAGTAGAGCGGGA
TGATTATGGAACTTCAAGATGTTATATGTTTAAATTGCAACTATGGGGAG
CAAAGTGAAATACTATTCAAAGTTTAGGGGTGCAAAATGTTAATACCCTG
CAGAAATATGAGGACAGAAAAATCTGTCCCCATTCTGTTCCGAAAATGGA
CCAAATTTCAAGGAATAGAAAAAAATTGTTCCCAAATTTCTATTTTTGTT
CCAAAAATTTCCCAGAAACTGAAACAAAAATCTGGGAACAAAAACATTAA
ACAAATGTCACCTAAGGTGCTGCTGCCCGCTTGTTTAATGTTTACGATCC
CATAAAATATAGAACAGAAAAGAAATACGAGAAAAATCTGTTGCTGTTCT
GTTCCCAATATTTCAGAACAAATTTCTGGGAACAGAAAAGATTTTTTCCT
CAATTTCTCTTTCTATTCTAAGTTTTTTGGGTACAGAAACATAAATTTCG
AGAACAGAAACACTAAAAACAAACCCCACCTTAGATTTATATAATTACTA
ATCCAAATCAAAATATATGATATGTTGGTTAATATTGTTNAAAATATTTT
TATAGATGCAGACCGTGTGTATTATATTCGGATAATTCAAATTCAGCACA
AAATTCATCGGACATCTCGAATGAACTCTCCAAATGTTCAATTAAAGAAG
CTGAGAAGCCACATCATACTAACAGGACTTCTGCTATCCCTATAACAAGG
CTTCCGCTCCATGTTCCTCAAACCATACAAGGTACTTTAGCTGGCGTTCC
TATCCGCTTTCCTGTTTCTACTCCGAATAAGGGGATGAGAATTCTGTTTC
TATTTTGAAGTATTCTGGGAACATTATCGCATTTGGTAACAATAATTATT
CTTAAAATGTTTTTATAATTCTCAGAACAAAAACAAAAAAATGCGTTTGC
TTTGCAACTAAGCTTAGATCATTCATATATATGTGTGTGAGTTGTGTGTG
CGTGAGACAGAGAAAAGAGATGAGAGAAGATGAAGATGTACGATGGTGGT
GGTGGTTCGGCGTTCGGGTGTCTGGCATGGGGGTGTCCAGTGTGGGTGGT
GATGGCCAACATGGTGGCCGGCCTAGGTGGTGACTAGCATGGTGGTGGTT
CTCAATATTTACCCTTATGAAATAGATTGGGAATAGTTTCATAGGAATGA
ATAGGAATCAAGATGTTTGTGTTTCTCAATTTCTTAAGGAATTCCGGAAT
TACTCTTAAAATTTTGAGAATATTTGGGAACAAAAACATGTTTAACAAAC
ACGTTTATGTACATTAATCCCAAAATTTTGAGAGCAAGAACAGAAATCAG
GGAGCAAAAACACTAAACAAACAGGGCCTATATATTTTTATTTCTGTTTT
TCTGTTTCTGTGCCAACGTTTTTCAAAACCAAAAAAAAAAAAAAAAAGAAA
GAATAGAAACATAAGCCAAACAGCACCGTGGACTCCGTTTGTTTAGTGTT
TCTGTTCCCTGATTTTTATTTTTGTTCCCAGTTAACTGACAAAAGAAACT
AAAAAGGGGGAAAAAAAAAAAGTTGTGAAGATACAAAACAAATTTAAAATT
ATAAAGTAAAAGAAAATCACTTGATAAATTCAATTTTTAAAACTTAAAAT
TCTTTTAGCACTTAATTTTCGGTTTTATCAAAAGGGTAAGTTGTCAATTT
TTCTGGAAACAAGTCAGAAATTTGTTCCCATATTTCCTGAGAACAGAAAC
ACAAACAGACAGAAACACCAATTTGTTTAGGTAGTGTTTGGCAACACAGA
ATTGAAGGCTTAGAATTGGAATCGGAAATGAACACATTCCAATTCTCTGA
AACCAAACACTGCCTTATTGTTTTTGTTCTCATACGGGAACAGAAAGAGT
AATAATATGTGAGAAAAAAAAAATTATTTCTGTTCCGTTCCTTAATTTTTA
GCTTAAATTCTCAGAACACCAGTTTTTTCACAAATTTCAATTTCTATTCC

AATTTTTTCAAGAACAGAAACCGAAATATGGGAACTAAACACAAACTGAT
TTTTTTTTTGCTTTCACAGCAGGAGGCGCGGCAAGGCCGGGAAAAGTCGT
CGGTTCCGTACTTCATTACAACAACTGTGATGCCATTGTCTCAGCTGCGG
CGCCGGCGGCAGAGGCAGCACACCAGCAAAGAATTGTCAGGAATCCGGCA
GTCCCCTCGCAATACACCACCGTCTCTACCTCCTATCCCAAACGAAGCTC
CGTATGTAAAAACGATAGGGGAGAAGACGGAACTCAGATGCCGCCCAAAC
CTGATCAGTACATTCCGAGGAAAGTTGCTGCCGCTCAAGGTGGCGGTTCC
GGAAGCCACTGGTATTGA
>EUC14834-RA [gene]
ATGAGACCGCTTCAGCCTCCGCCAGCCGCCTCTGGCTCCGCCGCAGCCAA
TCGCAACCGTCCACGGAGGCGTCCTGACCTAACCTTGCCTCTCCCGCAAC
GCGAGACTTCCTTGGCCGTACCGCTCCCTCTGCCGCCGTCCTCAGCTCCA
TCGTCTTCGAATCCGTCGCAGTTTCCGTCCGCCTCGACGCCGATTAATTT
TTCCGCTCTGGAGCGGATCTGTCGAATCGGGAGCGGTAGCGGCGGCACGG
TCTATAAGGTACGCCACCGTCCTACCGGGAAGCTGTACGCGCTCAAGGTG
ATCTACGGTCACCACGACGACTCCGTCCGCCGCCAGATCTGCCGCGAGAT
CGAGATCCTACGCGACGTCGATAATCCCTCCGTCGTCAAGTGTCACGATA
TGTTCGACCACAACGGTGAAATTCAGGTCCTCCTCGAGTACATGGACGGC
GGATCTCTTGAAGGCACTCACATCCACCACGAACCCTCCCTCGCCGATCT
CGCTCGCCAGGTCCTCTCCGGCCTCGCCTACCTCCACCGCCGGAGAATCG
TCCACAGAGACATCAAACCCTCCAATCTACTAATCAACACTCGCAAGCAA
GTGAAAATCGCCGATTTCGGGGTTTCAAGAATCCTAGCTCAAACAATGGA
TAACTGTAACTCCTCCGTCGGTACAATCGCTTACATGAGCCCGGAGCGGA
TCGATACGGATCAGAATCACGGAAAATACGATGGCTACGCCGGCGATATC
TGGAGCTTTGGGGTAAGCATACTTGAATTTTATCTTGGCCGGTATCCATT
TGCGGTGGGTAGACAAGGCGACTGGGCTAGTCTCATGTGCGCAATCTGCA
TGTCCCAGCCGCCGGAAGCGCCACCCACAGCGTCGCCGGAGTTTCGAGAC
TTCATAGCTTGCTGTTTGCAAACAAACCCAGCAAAACGGTCGACCGCCCC
AAAATTGTTAGCCCACCGTTTCATTGCGCAGTACGCCGGCTCTACTGGCC
ATAGCGGCAATCATCAGATGCACCAAGTACTCCCTCCACCTCGACATTTC
TCTTCTGCTTGA
>EUC17901-RA [gene]
ATGGCGATGGAAGACAACGAGAGCTGTGGGAGCAGAGCGGTGGAATCGTC
ACCATCAACGAAGCAGAACCGACAGGAGAGACAGAGGCGCGAGGTTTTTA
ACGAAGTGCTTAGCAGGCTACAAGACTTGAATCACGAAGAAGCTAGGCTT
ACTGGTTTTGAAGACCAGCTCTGGCTTCATTTCAATCGCCTACCTCCTAG
GTAAATTTATCTCCCCTTCCCGTTTCTCGATTTTGAGTTTTTTTTTTTTA
CCGGTTTTTGTTTTCATTTTTTTTTTTTTTGTTTGTGTTACGCTGTGTTT
GGTTGACGGGAAACTGCATGAAAGGACGGAAAATTGGGAGTCTCACTTGA
CGTTGAAACGGTGATTTCTTTTGTTCGTAGGATGAGCTTCGTATTCAGGG
ATCAGTTTCTTGAGCGGTCATTTTTACATACGCGCATCGATCAAATTTTC
GCACTGGAATTGATCTACGTGAATATTGAGATGATCTCAGTATCAACCTG
TTCATCTTTTGTCTTTGATTTCCACGTCAAGCTTGAATTTGATTATTTAC
CGTTATGCTTTCTCTCAGTGGTATGATGTGACTTGAAGACTTTGATTAAT

CTTCATGCTGTCACGCAGATATGCACTGGATGTAAATGTGGAAAGAGCGG
AAGATGTTCTTACGCATAAGAGACTGCTATCTTTGGCAGAAGACCCTGCC
AATAGACCTGCCTTTGATATTCGCCTTGTGCAGGTAAGAAACGCTTTCAG
TCTCTTATAATTTGTAAGTAGCAACAGGACTTAGTTTCATTTAAGAGCAG
AGTAATTTGGTGAAGATGGTGTACCATGTTCTAATGTTCGCAGACAGGAG
ACAAGCTTTACTCTTTTAGGGTCCCTGTTTGTTGGGCACCACAATAATTG
AATGTTGACCTTTAACACTCGTTATTTGTTTTCTAGACATTATTATCAAA
CAAAACTTCTGGTGTCCAGTTGGTCAATGATCCTTTATTCGGCAACTGTT
GGCATATTTTCAAAGAATATCTTCACGTGTATGACAACTGCCTAAAACTG
TAGTACCCTAGTCTTAGATGCAAGAAAAGTAATGGAAGTAATATTTCTTA
GTTACTTGAGGAATTCATGTTATCTTCTGGGCTAGGTATATAATTGTATG
AAAAAAAGGATATCACTGTTCATTCGAGAAGCTCCAAAAATGCTTTCTCA
TCCATTTCTGCTTCGCATTTCTTTTTGCCTTATGTGTGAGGTAGTCTTAT
GTAGGTTCCTTCTACCTCTGGAAATGCTAGTGATTCTATCAATTTGAATT
CTCCTGTTGAAGAAGACAATCACAGCTTTCGAAAAGAGGGGTACATTTTT
TTAGAACTTATCACATTGCCGATTCATTGAAATATGTTGATTATCTGCTT
TGTATTTGAGATCCTGTAATATTCCCTGTTTGTTTCCTTGCAACGTTTTT
AATATAACCATGCTCTCTCTCTCTCTCTCTCTCTCTCCCTCTCTCTTTGT
GCAGAATCCATCCTCCACCTACCTTTGGTTCGTCAGCCAATCTTGAGGCC
CTTGCACATCGAGCTAATGACTCTCATGATGATGATGATGGAGACAGATC
AATTTCACAGATTCAAAGGTAAAGAACTTTTAAACATACTTGCAAAGTAT
AATCGTTTTATTGCGTTCATCATTTGAAGAAATTTCTAAGCCTTATATTT
TGTTTCTCATTGAAGTTCTTGAAATCAACTTCTTTTCCTCCTGATGTTTT
CCGTCCAAGTAAATTAATGTAATTTGGATAGTAATGTCTCTCTATCTCCA
TATTCGGGTGGACAGCTGAATGCAGAAAAAATGCATGCCTCCAACAAAGCA
ACAAAACAAATTTTGGTTATCCAAGCAATCACTTAACTACCTCATTATTC
GAGTATTTTTTCCATGTTTCTTGTGTGTGACTTGTAACTGTTGGGCTGAT
GAGCCACCAGGTTTTCACTTTACTTGCGTGATTCGTAGCTTCTCTTAAAC
TCTTTAATATTTTTTTTCTTCGGTTTTACCTTGTTGTCTGGGACCTGGGT
TACGATGATAAGAAAGTGAAGGTTCATTATATCCCTTTATGGCATAGATG
TAAAGTTCTGCTGCCCATTTGTTCTTCTCAGTGTCAAATTGCTATGCTTG
AATTAATGTGTTTGCAGAACGCTATTATCTTGTTTCACCATTAGGCACTG
CATCGGACGTCCTTGTACATAAAATAGGATTTTTCGGTGGTCAATGCTAC
GATAATCCACTTAGTTTACGAAGATTTGGAAATAATGCAGGCCTATGCAC
GAGATCACATTTTCAACAGTTGACAAGCCAAAACTGCTCAGCCAGGTTGG
TTTCTGCTGCTTCTATTACAATTTTGGTCACTTGGATTGGCAACATAAGT
AAGTCTTTCAGGTGGGATATTCATTCTCTTCCCTGTCCTGGGCCTTGCAG
AAGGCTTCCTATATGGGATCTCTATGACGGACCACATCTTTAATGGTTCT
ATGCATAACAATTTTAAAGTGAAGTAATACGGAACATTTTCAGTGGCTAT
AAAAAAATGTGCAATAATATATTTTTAGGAAATCTTTTGTGAAGGAATAT
ATTCCTTGGTGTGTGCTAATGCTTCTTATCCAATGCTGACTCTCCAACTT
TTTTCAACTGTCCAGCTGACTTCGTTACTCTCTGAGATTGGACTTAACAT
TCAAGAAGCGCATGCTTTTTCCACCCATGATGGTTTTTCCTTGGATGTTT
TTGTAGTTGATGGGTGGCCTCATGAGGTATGATCTTTTACTTCTCAAACG

TTCATCAGTTATTCCAAAAGAATTTGTTTCATTAATCAGTGGCCTTAATT
ACTAGCACAATTCATCCTTCCAATGTGTAAAAAATGTTTTATCTTGTTCC
TTTTTTTTGTTTGGCTACACTTCACTTGTTCTGAAGTAACTATCACCGTG
CTTCTATCGTTCTATCACAAATTTTGTTTGACAGGCATTCATGCTCAATT
CATTTTGTAGGCTTAGTTGGCGAAAATAAGTACCATAAAATTTGGATGTC
ACGTTCTCTTCCTTGTGATTAAGGTTGTCTCCTTCAAGCTTCACAATCAA
GATAGTTTTTCAGGATTGTTCACCCCCCCGAAAGCCATATCTTTCATGAT
ATCATTCCAAAGAAAATGTGAGAGTTCATGAATCATTCCAAGGGATGAAA
GTTGATCTTAAATTGTTGATAATTGGTTAATTTCAAATGGTATATGTCTA
ATGCATGAGAACGTAAATATTAATAATTGTGGACAAGCATTTATCCTTCT
GTACGATATTTAATCTCAAAAGAGCGATATTATTGGACATGTTTTTGTAT
GCCATGTGTAATTTAGACCTCACAAGTAAATTCTTCATTCGTAGAGTTGT
TTTTGTTGTAAGCGCTTGGAAGCTTTTTAAGTTTTGATTTGCATACTTGG
ATCTAAAACAATATTAGGTAATTGAGTTCTAAATTCATGGATAGTAGCAT
GCGTCATTTTTCCTTCCCTTTGAGATAGTCGTAGTTCAGTTTAGTTAATT
ACAAGGTTATTTAGGGTCCTTTTGGTTTTCAACCTCAAATTTTTTTTCCA
GTGACTCTAAAATTGTTTTAAGAAACTGTTTAAACAAGTTAAGTCTCATA
TAAAAAGCTTAAATGAAATTTTTTTTTGGCAGGAAATCATTTCCTGATTA
GCGACTCTTTTCAGGTTGCAAGACAGTCCCTTATTATTTATTTCTCTGTA
GAGTTTGTATGTCGTTAACTCTGATGATCTGCTTTGTAGTGCCTCATAAA
ATTTTGGTTTCAATAAATGTTTAGGTGAGACAGTAAATCCTTATATTCTC
TTCTCCTTTTCAAATTACTGAATGCTAATTGGCGTGCTTATATGGAACTT
CTAGGAAACCATGCAGCTCAGAAATGCACTGGAAATGGAAATCTTAAAGA
CTAAGGTAGGTAGTTATTCTCAGCAAATGTTTTGCAAAAACTGATGAGAT
TTTCTATATGTGGACTCTGAATTTACATGCGTAGCAATGTTTCTTCTGAC
AGAATTTGAAGCTGGTGGGTGTTTTGGTTTTTAATGAACCTTCCGGATTT
GTAATTGTTCTCATTGGTTGCTGCAGCATTGTTTGCTGAATCGCTTTTCA
AAGAACCCTAGAAGTTCCTTTTAAATGTATTTCTACATCCTTTTTAGTTT
GTGTGAGGATGTGTTTGTGGGTGGGGGGCTGTGGTTTTGATGGTACTAGT
AAATTGTTATGTCGAAACTACTAGTCCTAACTAGATTATTGGATTAGGTT
TGTCCTGATGCATCTAAGGATGTGATTCTCTGATTAGTTATGCAGCCATG
TTCTTGAGTTAGTGTTTCTGTAGTAAGTAGTAAATTAAACTCTGTTTGCT
AGCTCTACCAAGATCTGGACTTCTTGAATGAAGTTTACATAACTAATATG
ACTAGGTTTGCTCATGTCGAGTTTGTACTTGGTTCTAAGGCTTCAATCTC
CCTTTCTAATGCGAAAGAAATGTTTTTAAATGATGTAATACATGGCTAAA
TCTGTCGTCCATTTTTTTAAGAGATGTTAGTAGATTGCAACAATTTCTGAA
AATTCTATCACACTTGGACAACAGGTCTTTAGTATTGACATCTAGAAAGT
ATTATGTGTTAAACGACCATCATTTCAGCCCCTCCATAAGTGTAAATAAT
GAGAATAAATGTGCAATCAATCAATAGTAATTGTTGACTATAACTTAGGA
TTTTAATTCCATAATTTACTTTTTATACTTTTATAAATAGAGATTTACTT
GAAAGAAGAATATACAAGAAATTCTCCTTCTAAAATGGTATCTAAACGAT
ATTTTCTCCTTTTTTTGCCGCCACTGCTGCACCTACAACCCTCTCTTCTT
CTTCGCGATCGATCTTCACGGCACTTGCGGAACTCTTCTCGTTCACGAAC
TAATCATTTTCCTATAGATTGTTGGAGTCGAGATCCTTTCTCTTCTTTCT

CTATACAATTTCTACTTATTTCCATTAGTTCTCAACTTTTCTAAAAATGA

CTAAAACATCCTCAAGTCATTCTAACACCTGCAGAAACTGCCCCACAACT

TTTTGGAACCTACAGAAGCAGCCCTACAGCTTTCTGGAACCCGGAAAAAC

TGTCCCACAACTTTCTGAAACTTGCAGAAACGACCCCATTACTTCCAAGA

ATTTACAGAAATAGCACCAAACCTTTCAAGTAGTTACAAAAATGGCCCCA

ATTGCAGAATGGTACCTTACTTTTTGGAATTTATGGAACAACCCCACATC

CTTTGCCAATTGCTATTTGAAACCCAAAATTTTCATTTTTGTTTTCTCTC

TATTTCCACTGCCCCTTCTTCATTATGATTACATATTTCACTAGTAGATA

CGCCGCCTACAATACCACCTCTCCTAAAAGCTGCCCCTATCCCTCAAGTG

TGCTTGGCCCTGCTTCTGCACCCTCATCTGTGTTTCTGATATGTTTTGGG

ATTGGCCACTCTACCCAAAAGTGCGCTCTTGGCTCTCTTCATTTGCGAAA

TCTTTTGTTCGTTGAACTCCTCTCTCTTACCCTAGCTGGTATCTTGATAC

TGGTGCTTCTACCCACATGATGGGCAACCCGACTCTTCTCAAGCAGCACA

CATCCTATAATGACCGTGACTTCGTTCAACTTGGTAATGGTGATCAACTC

CCAATCATCCCATGACACTACCACTTGCCTCATCATCATTTCTTCTTAAT

ACTGTTTATGTTGTTCCTTCTATGCATAAAACTTACTCTCTATTGCTCAA

TTTTGCACTGAAAATCACATTATTTGTGCTTTTGATGCTCATTGCGTCTA

TATCTTTGGTCTTGTCAATGGTTCTCTCCTTTATCAAGGCCTATGCGAGG

ATGGACTCTACAAGTGACCTACCCTTTCGTCTTGCCTACACGCTTTATCT

GCCACCCACAATTCATGTTCCCTATGGCACAATCGGCTAGGTCATCCTTC

AGCTCAAGTTATGTCTTACTTAGGTATAATAAATTGTTATCTTCTATCTT

CAAATTCAAATCCGTTTGTTCTGGATGTGTCTTGGGTGATTCCACTTGCC

TCTCTTTTCTCTTGTCTAATACAATAAAAACCTCATTCTCGTTTACTTTA

GTATGCTCAGATGTATGACAATCTCCATTGCTTTCGGTTTCTAGTTATAA

ATATTGCATGTTATTTAATGATAATTACACTCGCTTCACTTAGCTTTACT

TTATGAAACAAAATTATGAAGTCCTCAATCTTTTCTATAAATTTCTTTGC

TTATATACAAACTAATTTTTTCACTCACGTCCAACACTTTCAAAGTGACG

GTGGTGGGAAATTTGATAGTGCCCCCTTCAAATCCCTTTGCTCTTCCCGC

GATATTCATCATCGTCTCTCTTGTCCTCACACTCCTGAACAAAATGATCT

TGTTGAATGTAGATACCGCCACATTGCTGACGTAGCCCTCACCCTCCTTC

TCACAAGTCATAATCCTCTCTGTCACTGACCTGAAACGGTCTCCACTGCT

GTTCATTTCATTTGATTAACCGTCTCCCCTCCTCTAAACTTCAGTGGTCC

TTGCCATTCTCTTTTCTTTATGGTTATACTCCTTCATACTCTTAGTGCAT

GTGTTTGGTTGTGCATGTTATCTGTATTAAGATGCTTGTTTTACTAATAA

ACGTTTGCCAAATACTGTTGAATGTGTGTTTTTAGGCTACAACTTGCAGC

ACAAGGGCTACTGTTGTCTGGATCGTAGAACTGGCAGAGTTTATATATCC

CGGCATGCTCGTTTCAATGAACAATACTTCTTGTTCACATCTAACACCAA

CAATACTCCACTAGTCCCATCATCGTGGGCATTTGCTCCTCTATTGTCTC

CAGCTCCTGTGATATCTTTGCAGTCTATCCCTCAATATAACTTTTCGGCT

CCTTCAAGTTCCCCCAGCTCCTATTCAACAACCTTTTCCATATCATTATC

AACACCAACATATTCGGCCCTCAGAGACTCATTCTTCTACTACTGCTGCC

CCGAGTCTTCTCCTCCATTCCTTCGCAACATTCTATGGTTACACGTCATC

AAGATGTTACCCATTGCCCTCTTGTCTGAATAGATGGTACAGTCCGATAT

TCTCTCCCAAGGGCTTACCTCCCTCAGCGGCCATCTCCCATTAATGAGTT

GATTTGGTACGCCCAAGCTGTTCGTATCCCTAAATGGCGTCAGGCTATGA
CCAGTAAGTTTAATGCATTATTGAAGAACGACATTGGGTGGTTGTTCCTC
CTTCCCCACATCAACATTTTGTAGGTTGTAAGTGGGTCCTCAAACTCTTG
CAAAGCAGATTGTTCCATTGAGAGGTATGGAGCTAGCCAAGGGATTTCAT
TAACAGGTGGGTGTTGACTTTGATGAGACATTCAGTCCTTGAATCAAACC
TGCTACTGTTCGTATTATACTCGGTCTTCTCGTATCCTTTGGGTGGACAA
TGCGTCGGCTTGATGTGAAGAATGCATTTTTTCACGGGATTTTGGATGAA
TTCATTATTTAATGTCTAGTAAATTATTCATCTAGTACTCATGCAGCCGA
CAAGCTACAGTATCTTTCGGTTGACTTCTCAATCCTTGTAGTTCATTTGA
TCTGATATCTTATTGTATTCATCATTTTTCTTTAAAATCTTTTTTCCCTT
AAAATTTAACACAATCACACATTATTCAAGTCTATCTTATTTCAGCTGTG
GCTTGAGTCCACCCATATAGCGACGAACAATTGAAATCAAGTAGAGATCT
TTGACCTCTTTTTTGATTACATAGGAAAAAATTGTTGAATAATTGATCAT
TTTATAGATTGTGTCTCCATATTTTTTGCTGATATGCAATTACGCATGAT
GGCCTGAATAGAAAGCAGGATGTTATTTTTATTATTTCCTTTAATGTTGT
TGTTTTACATTGGACTGATGCATTATATCCCTTTATGTACTTTCTAACCA
TTTGCAGGGGCAATTTTTTACAAAACAACATTCAGTTTCTAGGGTGGGTG
ACCACTCCAAAGCAGTTTCTGAGTCTTTTCATAACTTGGTGAAAATCCCA
ACTGATGGATCTGATGTCTGGGAAATTGATGCCAGCATGCTGAAATACGA
GAACAGATTTGTGTCTGGGACATTTTTTGAACTGTATGCTCTACAACTCA
TTTTCCGTTTTATTGTGTTTGACTTTCCAATTTGTATACACTTTGTCTTT
TCAGGTATAAAGGAACATATTGTAGCCAGGACGTTGCTATAAAAAAAAAC
CTCATGAATGACAGGCTAAATGCAGATATGTTGAAGGAGTTTTCCCAGGA
AGTCTTTATCATGAGGTTTGTTATAGATGTTCTGATCCTAGGTGAACTTA
CTCTTTTCCTACATTTTATCTTTGAGTAGTGCATTGTCACTTTGAAATTG
AATTCTTTGCGCTCCTCTATTTGGGGAGGTTTTTCCTTGGGCAACTGATC
CTACTCGTGGATCTTTTCTGAAGATAGCATGACGGAACTTTTTATGGCTT
ACTTGTCGTTCTTTGGTCAAATATATAGTACAGTAAGAAGTTGTGGTTTT
TTAGCATTTTGTTGATCAGATATGAAGTTTTACGCTATGTCAGCTTGGAA
AGACACTTGATTAATCTTGAGATTTATGTTACAAAAAAATAGGGAAGTAT
GTTTGTTCAGATTGGGCTATTGTGATTTGAAAAAAAGAAAAATATATAGG
AATAAGAATACAATATATAAGAAGATTCAATCAAATGAATTTCTTCTCCA
CCTATTGTGTATATGAAGATGAACTTAATATTTGCTCTGAAGTCATATTT
ATCTTTGACATTTATTCTACATATTCATGTTTAATACGAAGATTCTACAT
ACTATGATGAAGTTTAGCATTTTTCTAGCCGATTTCTCTTGAAGTGCCTT
GCTGCTATAAAATTTGATGTTTTTCTTTTTCTGGAAGTCATACACAAAAT
TGGAACAGCAACGTCATATTGATTGTCAAGTGTGGCGTCCTTTAGTCATT
TTTTTCTATGATTGTCATCCTCATTATGCTTTTTCGTGATACCTAACCCT
AACTTAACCATATTTGATTTACCTTTCTTTGACTTTTTTTCCCCATTTTC
TTGGCAGGAAAATTCGGCACAAGAATGTTGTTCAATTCATCGGTGCATGT
ACCCAACCTCCAAATCTATGTATTGTCACTGGTAACTTGGTTGAAATTTT
AAAACATATCATCTCATATGTAACGATGTCTTGTATTTTCTAGAATGCTT
GGTTGATACCTTATCTGAATGATACTATTAACTACTTCATTGAATGCCAC
ATCTATGACCATTGCTATGAAAGCTCACAAGTGCTTCAAATTTATAGCAT

CTAGAATTCTGTACTCTTGCTCCATATAAAGAAAGATAATTTCTTATTGA
TGCTCGTGTTTCTGTTTTTTCAGAGTACATGTCCAGAGGAAATGTTTACG
ATCTTCTGCACAAGAAAAAGGGTTTCTTTGAGCTTCCAACTGTACTCAAG
GTAGCTATTGACACTTCAAAGGGGATGAACTATTTGCACCAAAATAATAT
AATCCACAGGGATCTGAAAACTGCTAATCTTCTGATGGATGAAAACGAAG
TAAGAAGCTCTTGCAAACTACAATGCTTCTGATTGTCATCAATTATCCTT
TTTCCCATTTATCCTTATGTGCATGACCGCATGGGTATCACCACCTCGAA
TTCTGTCACTTATTTACTTTATCTCTGTGTGTAAAAATTCTAGTAGTTTC
GTGTTAATCATGTGTCACGTGCTATTTAGATGTTGCTTGCTGCAATTTCA
CGCAATCTTTCCTTTCCCCCCCTTTCCTACATGTATTCATTATAGGGTTG
TGTTAGGTAGTTAACAATCACTATAAACCTTTGTCTAAACTCTCATGACA
TGAATCCGAGGATTAGACGAAGGGAGTTGGTGGACGTGATGAGTAGATGA
AGGATTATTGTTTTATATTTATAGGAAACTAAGTAGGTGGGGGAGAGATC
TATAGAGACTAAGAGTACAAGGTATAAATTATGGTATATGTGGAAGGAAA
ACCATATGAATGGGATGGAGATTATGATAAATAGACGTTTCAAAGATACC
ACGATAGATGCAAGAAGAATATGAGATATAATTATAGTCGTACTATTAAA
GGTAATTATAGTTGGAAAAGGATTTTGGAGAGGAATAAAATATTTTGTAA
TTGTGTAATAGATTCTATACAATAAGGCTCTATGCTCCCTAATATATTGT
TATAACGGTGAGCTAAATTAAGAAGAATATCACCCTAATAAATGGTCTAA
TTAACGGAATAAAGAAAGTTATTAAATTGGTCAACATGACCTAAATACTA
AATAAGAAATAACATTAATGGAATATTAAATATTCCAAGAATCCCCCTCA
AGTTAGGGTGAAGATGTTTTGGAGTTCTAGCTTGACGAAAGTTGACCGGA
GACCTTCCCCCTTTCCGATAACCATCAAAACGGATGATACGACGGAAAAA
AATGGAAGAATTGTTGTTGTCAAAAATAACGACAGAGATACAACGAAGGT
AACCGAACACTTGGTTACCTTGACGACAAAGATAGACGATCGTTATGTTG
AGCACGATTGATGGTTTTTGCTGCGTTGGAAGACCAACGATGACAACTGA
AACGTCGGAAGACCAATGGCGACAACTAGAAATCTGGGCAACAAACGGAG
ACAACTGGAACATCGAGCGACCAACGACAACAATTGGAACTTCTGGTGAC
CAACGGTGACAACTGGAACGCTGGGTGACCAATAGCGACAACTGAAACGC
CAGGGGACCAACGGGACAATTGGAACGCCGGACAACCATCGTGAGAGAGA
TGGCCAAAGAGAGGGGCCAATGTGAGGTTAAGGAGAAGGCAGTGCTGCCG
CGGGACAAAAGGTGCAACAACGCTTGTGGTTGGAAGGTGATTCAGCAACA
TATAAAAGCGGTGGCCTGAAGGTGGTTTGGTGTCAGGCGGTTGGCAGTGG
GTGGCGAGGCACAGGCAGGACAAAAGGTGCAACAACGCCTGTGGTTGGAA
GGTGATTCAGCAACATATAAAAGCAGTGGCCTGAAGGTGGTTTGGTGTCA
GGCGGTTGGCAGTGGGTGGCGAGGCACAGGGGGGCTAGGGCTTTCCTCCT
CTCCTTCTCTTCTCATTATTTTTCTCTTTTTGGGCTCTGATACCAAAAAG
AATTTTGGAGAGGAAGAGAATATTTTGCTATTATGCAATACATTCTATAT
AACAAGGCTCTACGCTCCTTATCATGTAAACCACAACGGTGGACTAAATT
AGGAAGGATATCGCCCTAATAAATGGGCAAATTAATGGCATAAAGAAATT
CCAAATTGGTCAACACAATTTAAATAGAAAATATGACCTAAATATTAAAT
AAAAAATAATGTATTAATGAAAATATAAATAATAAATATTCCAGCAGTTG
GAGAATTGTAATTTGTATCTAACCAAAAGTTGGATTGCAAGATAATGTTC
GACAAGAGTTTGAGTACATATGGATGGAGTTTTTCAAGGTCTAATGGGAA

TTCTTAAAAAATGGTTCTGATTTGTTTAGTTCTTGAATATTATTAAATGGT
TTTCCGAGTTGTATGTGACTACCAGATTTTCAGACGCATCACAGTTTGGT
TTTTTATTGGATGTCAGTTGCATATATTCTCCTTAATTTTTTGTGCTTTA
AGATACTCAAGGCTATCCTAAAATCCCTTGTTTTTTTATCCTGAAGGTT
GTAAAGGTTGCTGATTTTGGAATTGCCAGGGTGCAGGATCATACTGGTGT
GATGACTGCCGAAACTGGAACATACCGTTGGATGGCTCCTGAGGTGTGCA
TATTTTCATAACTTAAAAGACAATGCTGACATTTTTACGCAAATGTAGGC
ACCGTTCGGTAATATTTTCTTTAAGTACTTTTTGCAATAAAAAAAGTTAA
ATGAGAACTTTTACTTTTACTTAAGAACAAAAAAATGTGTTTGTTAACGT
AAAGCTCATATCTTTGTATTATCCATATAAACGATCTCTTAATTCATGAT
ATTTTTTTTTTTAAATGCAAAATAAACGCTACTACATTTTGGTTAAAAGC
AAAAAAGTACTTAAAAAAGCTTTATCAAATGGTGCCTTAATTTTTAATCA
TTCCAAGCCAAAGTGCTATTCGGATGCTATTTGATGGACCAAGCAATATG
CTCAATATCAATTCATGTCAAGAATGAAAAACAAATAGCATCAGCATTCT
TAGTTCTGTGTCTGATCATCTTATAAAACCTAATTTTTTCTGAGATCTTC
ATTAATATGTTTTTTATGAATCAAAGGTTATTCAACACAAGCCATATGAT
GACAAGGCTGATGTTTTCAGCTTTGGGATAGTGTTATGGGAGCTTCTAAC
AGGAGAAGTAAGCCATGCTACATTCTGCAGAGAGAAAAAAACACAACATT
TTATCTTCTTAAATCACTCTTATTTTATGCATTCACTGATTGGATGTGCA
GATTCCTTATTCCTACTTATCACCATTACAAGCTGCAATTGGCGTGGTAC
AACAGGTAATAATTTTACGTTGATCCGAAGACAAACTTGAATGTTCACAC
CATTTTTTTTTCTGGAAAAAGAAAATAATTCGAGTTATATTTTTTTATTT
TTAAATCAGGGTTTAAGACCTACAATTCCTAAGCAAACTCCTCCAAAGCT
TGCAGAACTAATAAAAAAATGCTGGCATCAAGACCCAACTCTAAGACCCA
ATTTCTCTGAAATACTCGAGATCTTGCAAAAGATAGCCAGAGAGGTATTG
TGTTATCTATAAAAAGAGATTGGTTCCGTTGAGACCGACCATTCTTTTAC
TTTACATTTGCATTTTTCTCTTCTTTCCAAATAGGTGGGAGGCGATGGAG
GAGATGACCGCATCGATGTTAAACCAATTGTGGATTTTCCCCTGCACATA
GATAGGAGAGGCCATCACTGAAATTTAGTAACACTGATGGAATGATTTGG
CCTAATCTTCACAAAAATTCTACAGAAACTTGCGTTTCTTCTTTGAATGA
ACAAATCCAGTTTATTATAGCGACTTCTTTTTTGTTTTTCTATTTTTTTT
TTTTTGGTATGTGTGTAAATCTCATATGATCTTCCCAAATCACAAGCGTG
TGCAGAAAATGTCCAAATGAAATGAAATGAAATGTTTTTTTTTTTTTTTT
TCTGTCATTTTTCTGTTTTGTGGAATAGAGTTGGTGTTGCAATAATTGAT
GATTTTGCTCTCAGGTAATATGGCGATGGCGGTTTCAAATGCCTATTCGT
GAATTATGGAGTTGTAAAGTAGAAATTGTCGGAGTTAAGACAGGTAGATT
TTGTGCCTTCCCATGGCGCCAAACCTCTTTCCCACCACCGCCATCATGCG
GCATTGAACCACCTATCCACTGCAGTTGCTGCACCCTTCGTCCCGCGGCA
GCATTGCCTTTACCTTCATCTCATGTTGGCCCCTCTCTCTCTCTGTATCT
CTCACGATGATTTTCTGGCGTTTTAATTGTCGTCGTTGGTCCCCTAGTAT
TCTAGTTGTCGTCGTTGGTCGCTTGATGTTCCTATTGTCGTTGTTGGTTG
CCCGAAGTTTCAGGTCGCTAGTCACTCGGTGTTCCAATTGTTGTTGAATT
CGCTCATGTTTCATTTGTTTTGTTGATTGCCCGACGTTTTAGTTCTTGCC
GTATATCGCTCGGCATTCCAGTTGTTGTTGTTGGTTGCGTGGCGTTCCAA

TTGTCGCCTTGTGTAGCCCGACGTTCCAATTGTCATTCTTGTTCGCCCAA
CATTCCAATTGTCTCTATTGTTTGCTCAATGTTCTAGTTGTCGTTGTTAG
TCATTCCAGTTATCGTCGTTGAACACCCGACATTTATTTTCATCCTATAT
CTGTTGTTTTTTCTGGCAATGACCATTTTTCCGTTTTTATTCTTGTTAAG
TTCGGATTTCAAAACATTTTTGTCCCAATTTGAGGAAGAGTGTTGGAATA
TTTAATATTTTTATTAATACATCATTTATTATTTTTTGATTTAGGTCATG
TTGATTATTTTTATAATTTTTTTTATTCTATTAATCAGCTCATTTGTTAG
GGTAATATTTTTCTTCATTTAGCTCCATGACTATATAATAGAGTGTATGC
TTTATTATATAAAATTGTTACACGTTAACAAAAAAATTTCTTCCTATCTA
AAACCCTTTTGAAACTACATTTGGGTGTGTGCCAAACAGGCAATAGTTAA
>EUC14352-RA [gene]
ATGAAGTCCGGCGGTGGATCTTCACTGCCTGATTCTGAAATTGGAAATGG
AAATGAAAATCGAAATGGGTTTGTGTTTCTCGTGAATAATGAGGTTGGCT
TATCCGTTCGTACTGATCCTCCTCCTGGCTCGGAGGTCGACTACGTGGAG
AAAGACCCCCGAGGGCGATACGTTCGGGTAAATCTTCTGATTCCATACAT
GTTTTTTGCATTGAATTTTATGTAATTCTCCGATTCATAATCTGAGTGTT
AGTTTTCTGTAACTCTTTGAAGCTGCATTGCTCTTGATTATGCTGCATTT
ACCTATGGCTATGATTAATCCATCAAACATACCCGATGATCAAGCAAATT
TAGTTTTGTTTGACTTAAGTGCAAGCAATAATTTTGAACAAGTGTTTGCT
TTGCTTAGTATAAATTTAAGTTGATATGATGATTTTTTGCCCCATATATG
TGTGAAGATGTGGGCGTAATCTTAACCCTTCTATTGGAGAGAAATTTTCA
ACGCTTGGAGACATTCTATAGACCATAATTATGTTGAAGGATTTATTATA
TAGATTGGATAATTACCAAAAATTGTATTAAAGATCACTTATTGCTCTTT
GCTCATTTTTCTTTTCCATGGTCCTCCACAGTACAGTGAGATATTGGGGA
AGGGTGCATTCAAGACTGTGTATGCTTCCAAAACTTCATCTTATTATTAT
TATTTTTTTTATTTGTTGGATCGATCTACACTTGCAGGGAAAAAAAAAAT
CCTTATTACAAATATAAATTTGTTGACTCTATGGATGGTATTTTTGTTTT
TCGCTTTTGTTCAATTGTTGTTATATGTATAGCTTCAAGGCATTTGATCA
ACTTGATGGAATAGAAGTTGCTTGGAACCGAGTTAAGATTGATGATGTGT
TACAATCACCCGAAAATTTGGAGAAACTTTATTCAGAAGTGCATCTTCTT
AGATCGCTCAAACATGAAAACATCATCAAAATGTATGATTCTTGGATCGA
CGAAAAGAAGAAGACCATTAACATGATCACTGAGTTGTTCACTTCTGGAA
ACCTGAGGCAGTGAGTTCTCTCTCCTCGTGCTTTTGCCTTTATAATTTTT
TGGGGTTTTGACACTTTTTATTCCCGAAGTTTGACTGAAATCTCACTTTG
ATACCCACAGTTTTAATATATAATACCTTAAAGTTTCTTAATCATTCCAC
TACAGTCCAAGTCATTATTGCCATCACAATTATGGACAAAAAAGTCACTT
GCCATGCAACTAACTATTTTTAGGAAGTTTTATGGACAATTCCAGTCTCA
TCATTCCCTCCGATTCTCCTTCCCACCAAAGTACTCTAATATATTGATAT
AGTATATACACACACATAGAGAGAGAGAGTTTTTTAAAGAAAATTTATAA
CGGGGGAAGGATGTCCCCTTAAAAAATAATTGGGTGCACGACAGTTGATT
GTTTGTTGTCCAAAATTGTAATGGATTTACTAATTTGTACTGAAATAGGA
TGATTATGGACTTCAAGGTGTTATGTGTTCAAATTGCAACTATGAAGACC
AAAGTGAAATCATGGTCAAACTTTAGAGGTGCAAAGTGTCAAAGACCCTA
TTTTTTCCCCATTTTTAAGCTGTGAAACTAATTGTATGCTTTTTCTTTTT

AGATACCGTAAAAGGCATAAGAGTGCTGATATGAAAGCTATCAAAAACTG
GGCGAGACAGATTCTCCAGGGGTTGGTCTATCTTCATAGTCAGAACCCGC
CCGTTATTCACAGGGACTTGAAATGCGACAATGTGTTTGTTAATGGAAAT
CATGGAGAAGTTAAAATTGGAGACCTTGGTTTGGCAATTGTTATGCAACA
GCCAACTGCTAAGAGTGTTATTGGTAACATTAATAAAAGTCATTGATTTT
ATTTTTGACAATAAGGCACCGTTTGGTAATTATTTTTTTAATATTTTCGA
GGAAACCTGATTTATTTTTTGTGGCAATTTTCCAATTAGGAACCCCCTGAA
TTCATGGCTCCGGAGCTTTATGAAGAGGAATACAATGAACTTGTTGATAT
ATATTCTTTTGGTATGTGCATGCTGGAAATGGTTACTGCTGAGTATCCCT
ATAATGAGTGCAAAAATCCTGCTCAAATTTACAAGAAAGTTACCTCTGTG
AGTTGGGTTAATTGCCTTTTTCTCCCGTGTGCTTTGATCTTTTTTCATTT
TGTCCCCTCTACTTTAAAAACATGCACTTTTATCCCCTTCCACTTGCAAT
AAATTAGAAATGATTCACCCAATTTCAGGGCATTAAGCCTGCTTCTCTTA
GCAAGGTGAATGATCCTGAAGTGAAGGAATTCATTGAGAAATGCATAGTT
CCGGCCTCCCAAAGGCCGCCTGCTAAGGACCTCCTTAAAGATCAATTTCT
TCAGCTACAAAATCCAATCAACAATCCAATGCAGTTAGCGAATCGCATTC
CTAAATCGCTAAGTTTATTGGATTGCGGGCCTCTTTCAATGGAAATAGAT
TCTGATTACAACCAGTCGGTTTGTACGGATTCCAACTGTGGAAGTCCACA
GTCTCTGGTCTTGGAATTTCAAAGGATGCATCAAAACAATGAATTCAGAT
TGAAAGGCAAGAAACACGATGATAATTCAGTATCACTCACCCTGCGAATT
TCATATCCATGTGGTAAGTTTGAAAATTACACATTGACCCCTAATTATTC
GGTTGATGAGCCTCTCAATTAAAAAGATGAGAAATGATTGTTATAATTTG
ATTTTATTGGTCTTTGATTGGATTCAATTCCAGGACGGGTGAAGAATATA
CACTTTCTGTTCTATCTTGACACGGACACTGCAGTCTCAGTGGCGGGTGA
GATGGTCGAACAATTGGAGTTGGAAGATCACGACGTGGCCTTCATTGCTG
AATTCATTGATTTCTTGATTATCAAAATTCTACCCGATTGGAAACCTTCA
TCCGATCACCATCATGATCATGATAGCCCCCCAACCAACTTAACCACCTC
TACTGAAAATCCTCCTCAAGAAGGCCTATTGGATGGAAATAATTTTGAGT
GCACATCTCCTTTATCGGTCTTCTACTCCCCTTCCAGTTTGCTAAACTTT
GATGATAAAGAATCACAGGCATCAGCTGGTTCTGAAGACGTTTCGATGAA
GAACGAGAAAACATCAGACTGTGTTGAGTACTTCATCGGGGGGAATATGT
CTGAGCTGGAGTTTGGAGATTTGTACGAAGAAGAAGACGAGTACAGCAAG
ATACATGAGGAGGAGGAGGGGGAGGAGTGGTCTTGAGTGCATTGCCTTGAA
TGGATTCTCACGGTTGGCGTCACTTGATTTTGGCAATTTTTCTAAAGTGA
CAAGCCTGTCGAGCAGCTGTTCTTCACCTGATCAAAATCAAAACCATCAG
GACGTTGAACTGAAGATGGAGCTTGATAAGGTGGAAGCGCAGTATCAGCA
TTTGTTTCAGGAGTTAACGAGAATGAAGGAGGAGGAAACCAAGGCTGCAA
AGAAGAGGTGGATGGAGAGGAAGAAGATATCATCAGTTCTTTGA
>EUC15557-RA [gene]
ATGGGTTCGTCGTTAATGGGATCGGAGACCGGAGACGACGACATAGCCGA
GACATCACCGGATGGACGATACATTCGTTACCACGATGTTATCGACCGGT
GTTCTTTCAAGGTCGTTTACAAGGGTTTCGATCAGGACGACGGGAAAGAG
ATTTCTTGGTGTCAAATTTGCATCGACGACTGCGTTATACAGTCGACGGA
GCATCGCCGGGCCTTGTTGTCGGAGGCCAATCTTGTGAAATCTCTGCGGC

ACGGGAACTTGGTCAAGTGTTACCAGTACTGGCTTGACGGAAAGAACAAG
ACGATCAACATGGTTACAGAGTTGTTCAGTTCCGGCAATCTCCGGCACTT
CCGGAAAAAGCATAAGAGCGTCGGCCTGAAGGTGATCAAGAAATGGGGGA
GGCAGATTCTAAGAGGATTACACTATCTCCACACCCAGAATCCGCCGATC
ATCCACTGGGATTTGAAGTGCGACAACATCTTCATCAACGGGAACCACGC
CGAGGTCAAAATCGGCGATCTCGGGCTAGCCACCGTCCTTCACCGGGGAA
CAGAACGGAGCATTATCGGAACGCCGGAATTCATGGCACCGGAGCTCTAC
AACGATGAATACAACGAACTCCTTGACATCTATTCCTTCGGCATGTGTAT
GCTTGAACTGATCACCTGCGAATGCCCTTATAGTGAATGCGAAAATCCGG
CAGAGATATGCGAGAAGGTGACCGCTGGAGTAAAACCTGTTGCGCTGGGA
AAAGTGAAGGATCCGAAATCCAAGGAGTTCATAGAGAAATGTATTATTCC
GTCGTCTCAGAGACCGTCGGCGTTGGAGCTTTTGGAGGACCCGTATCTGT
CTATTGAGAATTTCACCGATTCACCGCAGATTCCGAATGCGATTCCGGCG
AACTTACCGAAATTCGATTCTCAAATCTCCATCGATTTGAGCTCGGAGGA
AGGTAACGATCTCTCCGTCGGAAGCTCTACGGTCGATTCAGTTTCTTCTC
TGGAGATGTGGAGATCGAACGAGACGGTGAAATTGCGGCTGAGTGGCACA
AAGGTCGACCAGAAATCCATCACGTTCAACATGAAGATTGGGAGCTTGTC
GGCTTCGACAACCGAAGTTTGCGAATTCGCGTTTGACGTCTGCGCCGACG
ACGCGCTCACGGTGGCCGTCGAGATGGTGAGGCAGGAGATGCTGTCGTTC
AAGGACGTAGCTCTGGCGGTGGAGCTCATTGACTGCATGCTTCTGGAGCT
TGTACCCAGCTGGAAACCGTCCTATGGGTACTACGTGGGACCAAAGAGCT
GA
>EUC26609-RA [gene]
ATGATGATAGAAGATAGCGAGAGCTGTGGGAGCAGAGCGAGCGAATCTTC
GCCGGCGAATACTCGACAACAGAGAAAAAGGCAGGAGGTCTACAATGAGA
TCCTTCGTAGGCTTAGAGAATCTAATAACCAGGAGGCTAAGGAGCCAGGA
TTCGACGATGAGCTTTGGGCTCACTTTAACAGACTTCCTTCTCGGTAACT
TTACCTTTCAGACCTACATTTGCCAATCTTTCCGTACAACACGTACTTTT
TTCTCCTTGTTTGTTTCTGATATTTATATATGATTATGTGTGTGTGCAGT
ATTTAGATACATGTATGTATCTGTTTGAGTCGGTTTATTGATTTAGATAC
ATGTATGTATCTGTTTGAGTCGGTTTTATTCTTTGATCTTGAGTTTTGCA
TCTTCATTCTTTCAGCGATTAGGTTTTGGATTCCTGGTTTAAAAATTTAT
TTATATATTGTTTACTCTTCTTTTGACGTATAAGCGTAATGATTTCTCCA
AATGTTTGATTTGTGCTCTTCCTGGTTATCATTTGCAAGTTAGCTTGCAT
TGTGGTGTGCTTCAGATTGAAGCATAGTCGGGGAATGAAACTATTTTTTT
TTTTGACGTTTGTATACCGCTACATGTATATTCACTTTTAATCTTTAGGG
CTGATCTCTCTCTCTCTCTCTCTCTCATCACAACACGCATGCAGAAGCAC
ACCAACCCACCCGCTGAAAATGGTTTCATTGAAAGTCCTTAGATCATTTG
GTTTATAAGTTCGTACGTGTTTCAATGGTTTTGAAACATTGGTTTATTTG
CACTGAAGGTATGCATTGGATGTGAATGTGGAAAGGGCAGAAGATGTTCT
CACGCACAAGCGTTTGCTGAATCTTGCTCATTATACTGCTAATAAAACTG
TATTTGACGTTCGCCTGGTGCAGGTGGGTTGATTTGACTAGACTTTAAAC
TCGAGCATCGTGATAAGACATAAATTTAGCATCTCTTGCATCTTGATGCT
GTTTGTGATTTAATATTTGTCTTTTCACACACATATATCAGTATATGCAT

AGGTATTATTAGCCATGGCATATCGGTATGGACTTGTTATTGGCAAGATA
TCTTTATTAGGCACTTTAATGTGTTCAACTCTTTAATTCATTCGCGTGGT
GGTCCGATCACCTTAAGCCAAAATAAGTTGAAATTTGAAGTAGTGTCTTG
TTCTTCTTCATTATTAAAGTCAGCAGCACACAATGAGCCCATCAATCTGT
TAGAATTTGTCAAAACTTGTCAAAAGTAGACTACTTCATCTTGAGATTCC
AAATGATATTATGTCATGGTCATATCTCAACCAAGTCAAAGTGACTCACT
ACGCAATTTCACTAATGGAGCTCTATCATTATGGACCCTGCGAACTTAGA
GATTACTCAATTAATATTTTGAAACATGGTTTTATGAGTGTTTTCTTCAA
ATGTGCATGAGTATTTGTTTGGTATGAGTGGGTGGGGAATAAATTGGTTA
AGCTTTCTTGCTTGATTAAACTTTTATTTTGTATCAATATCTTTCATAGG
TAGCTCCTATATCTGATGGGAATTCACCTGATTCAGTTCATTCAAGGTCT
CCAAGGAAGGAGGTCGTTCAAAGGTTGTTTTAACTGTTTAGCAAATCATT
TATCACTAGGTTAAATGTCTATTACATGCAAAAATTGTATAAGAATATAA
TTCAAAAGCCCAGTTTTTGTTGTAAATTTTAAATTTAGTTATTGTACTAA
GGTCAACAACTCCCTGATAACGTGTCAGCATTCATCCACCACCTGCCTTC
GGCTCATCTCCAAATCTTGAAGCCCTTGCACTTGAAGCAAGCAAAGCTGA
ACTTCAAGATGGCGACAGTGCTGCAAATACCCATGCAAAGTTTTCTCGGT
ATTGGATTTTATTTTATTCTTATTTATTTTAACTTATGTGTGATGTACGT
AGCGCCTTACCATCATTTGGTTATACCGGATTTAATAGAACGCGGATTTC
TTGCATTGCTCTCGAAATTGTATTAGAATTCTATAGTGCTAAAGTTTCTC
ATACAAAAATCTTGTTTACTTCCTTGCACGTGCCTACTTTGTTATCGTCA
GTGCATGGAAACCTCTTTCGTTTTTCTTCTTTTGACCTAGCCAAGGTGGG
GGTTGGGGAGTTGTTCCGGAAGAGCCTACCTGGCCCCAAGTTTCGGGTCT
GGTCTTGTGACAATTAGCATCTAGTGCTTCTCTCTGGATAACACCAACTA
TATTATGGAAATCTTTCCGGACAGTACTTTTTGGTCCTGTCCCCCCCCCA
CCTCTCTCTCTGTTTGTGTGGGCGTGTGAGTGCTAATGTGATGAGGCAAT
CGACAATATGTTAGAAGCAGTTCATGAATCGTGATATATGTCCATGAATT
TGTATAAGATGTCAGTTTGATTGCCAGATCTTAGGGTATCCTTATCCTCA
CCATCATCACATAAATAGTAGTCATGTTATTTTGAAAATTCACTTATGCT
GTTGGTTTGATTGTCTAACATATTTCAAAACAGCGTGTTTTCACCTTCAT
CAACTTATGTATTGCATTGTTTTGGAACTGTTACAGTTTTATTCTCATAG
CTTACCGCTTACTTTTGTATGATTATATGCAGGCCCATGCATGAAATCAC
ATTTTCAGCAGATGACAAACCTAAGCTCCTCAGTCAGGTAGTTGTTCTGC
CTGTATATTTATATTCTAGTTGGCAATTTCACCACTTAGAGAAAATGTTT
TGTGCTCATACTAACTGAGTATGATTTTCTTGAAGCAGCATCCTTTCTCC
ATTTAGCACTTTGTTAAGAAATCTGGTGTATGAAATTTTATTAAGAAACT
TACTGATGTGCACTGTTCAGTTGACTTCCTTACTGGCTGAGGTTGGNCCC
GGGGAGATGAATCAAAATCGGAATTGGTTTTTTACGGGAATTGTGTTATC
CTCGTGTCCAAACAGCGGCTATATGTCCACATCTCCTACCCAAAACAGTT
GGAAATGCCACGCGCTTTTCTCATCACACCTCAGCAAAAGCGCTTGACAA
CACGTGACATGCACGTGCATACCGACATTTTCACCCATATCACATGAACT
TTTCTAGTACTAATGTACTATGTACCAAAAATGTCACAAGTAGTTTGAAA
ATCTTAAATATGTCTTTTTGGACTATCCTACAATATAACACCTATATTGG
ATATAGCAGGCAGGTTACAATGTTAGCCCAAATAAATTGTAAATTATTTC

ATTAAAATTACAAGTTCATTTAAGACCTTTTAATTTTTTCAGTCAAATCT
TATAAATTTACGGATTGTACCGTACAGCTGCTGTAGGTTAGATTTCTCCT
TTTTTTTTTTTTTTTTTAAATGATATTTTTCGTTGTCTATGGAAGTAGGA
GGGGCGTATCTAAAAAATTATCGACAAGCTTTAAAAAAAATATTTTTGAT
GTTTATTGGATAAATCGATAAATCAGAGGACTAATGTAAAATTTTAAAAA
TTTTAATTGGTTTTTAAAAATAATAGAGGGTTAAAAGTGAAATTTTTAAA
AAAATTAAGAGAGGACTTGAGTCTCACGTAGGCCTTCTATAGGTCCGTCA
TTGGATTACAGTATCACAATGATATTCTCCACGAACAAACTTAACATACT
GAAAAGAGAAATTGTTATTTTGTTCAGTAACATATTATAAATGGCAATGA
TTGACTTCTCTTTGTTTTATATGCAAATCAAATTGTTGATATATTTACTG
AGACTGTTGATATATTCTTCCATAGGTCTTGTGCAGCTCCGCCATTGGAT
TACAGTATAAGAATGTTATTCTCCACGAACAAACTTCACATACGAGAAAA
AAAAAATAAAAAAAAATCTCATTTTGTTCAATAACATATTATAATTTGCA
CTGATCAACTTCTCTTTGTTTGATCTGCATACTAGATTGTTGATATATTC
TTCCATAGGTCTTATGTAGCTCTGCCATTGGATTACAGTATCAGAATGAT
GTTCTCTACAAACAACTTAACATACTAAAGAAAAAAAATATGTTATTTTG
TTCAGTAACATATTATAACCAGCACTGATCGACTTCTCTTAACAAACCAG
ACTGTCAATATATTTACCAAAATAATTTTCGAATTCAAACTCAAGTTGGA
CTCTGTTTTCATGCCCTGCGTTTGAGAGGGAGATATTGCAATAATATGGA
ACCCTAAAGCACTATATTTTCTCATTTTAGGCCCATTTCAGAGTAATTCT
CTATGGTCATTGTAATACATAAAAGGCTCTGAATTAACTTAATAAACAAA
AATTCAATTCTTTATCTTTCTTTCTACTTAAAGACCATATGCCGAACATC
ATTCGGATTTTGAGTGAGATTTTTACCTTGTTAGTACTGATTGATTTGAA
CAGAGTACTTTGTAATATCAAATTTATAAATTATTCCGGAGACAATATCT
AAATATTAAAATTAAAAAAAAAAAATACCCATGGAAATAGTTACTTCAAAT
TTATTGCTATATATATATATTTTTTAAATTTTAAAATATTTTTATCAATACC
ATATCAAATAAAAGAAATTTTCAATTTTGCTTTAACCACCACATCAACCG
TTTTTTTCAAATAACATCTTTAATTTTATTATTTTTTTAAATATTTTAAA
AAATATACTGAAACATTACAGAATCCTATTTTTAAATGTCACCCACGATT
TTCGATTTTTCAGGGGTGACTCTGAATCCAACGAATAATAAAAATCTTTG
TTGATCACGTTATTTTTATCATCCGTTAGATACAGTTCGATAACAAGTTG
ATCAGAAAAACAGTCGACCGGATGATCTGAATCCAGATATATCGATATTT
ATGCCTAGTCATTGCCACGTAGGACAAAAGCCCTTTATCCATTGAAAAAT
GACACACAACGGATNTTGTGATTTAATATTTGTCTTTTCACACACATATA
TCAGTATATGCATAGGTATTATTAGCCATGGCATATCGGTATGGACTTGT
TATTGGCAAGATATCTTTATTAGGCACTTTAATGTGTTCAACTCTTTAAT
TCATTCGCGTGGTGGTCCGATCACCTTAAGCCAAAATAAGTTGAAATTTG
AAGTAGTGTCTTGTTCTTCTTCATTATTAAAGTCAGCAGCACACAATGAG
CCCATCAATCTGTTAGAATTTGTCAAAACTTGTCAAAAGTAGACTACTTC
ATCTTGAGATTCCAAATGATATTATGTCATGGTCATATCTCAACCAAGTC
AAAGTGACTCACTACGCAATTTCACTAATGGAGCTCTATCATTATGGACC
CTGCGAACTTAGAGATTACTCAATTAATATTTTGAAACATGGTTTTATGA
GTGTTTTCTTCAAATGTGCATGAGTATTTGTTTGGTATGAGTGGGTGGGG
AATAAATTGGTTAAGCTTTCTTGCTTGATTAAACTTTTATTTTGTATCAA

TATCTTTCATAGGTAGCTCCTATATCTGATGGGAATTCACCTGATTCAGT
TCATTCAAGGTCTCCAAGGAAGGAGGTCGTTCAAAGGTTGTTTTAACTGT
TTAGCAAATCATTTATCACTAGGTTAAATGTCTATTACATGCAAAAATTG
TATAAGAATATAATTCAAAAGCCCAGTTTTTGTTGTAAATTTTAAATTTA
GTTATTGTACTAAGGTCAACAACTCCCTGATAACGTGTCAGCATTCATCC
ACCACCTGCCTTCGGCTCATCTCCAAATCTTGAAGCCCTTGCACTTGAAG
CAAGCAAAGCTGAACTTCAAGATGGCAACAGTGCTGCAAATACCCATGCA
AAGTTTCCTCGGTATTGGATTTTATTTTATTCTTATTTATTTTGACTTAT
GTGTGATGTACGTAGCGCCTTACCATCATTTGGTTATACCGGATTTAATA
GAACGCGGGTTTCTTGCATTGCTCTCGGAATTGTATTAGGATTCCGTAGT
GCTAAAGTTTCTCATAGAAAAATCTTGTTTACTTCCTTGCACGTGCCTAC
TTTGTTATCGTCAGTGAATGGAAACCTTTTTCTTTTTTCTTCTTTTGACC
TAGCCAAGGTGGGGGTTGGGGAGTTGTTCCGGAAGAGCCTACCTGGCCTC
AAGTTTCGGGTCTGGTCTTGTGACAATTATTGCATCTAGTGCTTCTCTCT
GGATAACACCAACTATATTATGGAAATCTTTCTGGATATATGCCCATGAA
TTTGTATAAGATGTCAGTTTGATTGCCAGATCTTAGGGTATCCTTATCCT
CACCATCATCACATAAATAGTAGTCATGTTATTTTGAAAATTCACTTATG
CTGTTGGTTTGATTGTCTAACATATTTCAAAATAGCCGTGTTTTCACCTT
CATCAACTTATGTATTGCATTGTTTTGGAACTGTTACAGTTTTATTCTCA
TAGCTTCGCAGGTTTATTTACCGCTTACTTTTGTATGATTATATGCAGGC
CCATGCATGAAATCACATTTTCAGCAGATGACAAACCTAAGCTCCTCAGT
CAGGTAGTTGTTCTACCTGTATATTTATATTCTAGTTAACAATTTCACCA
CTTAGAGAAAATGTTTTGTGCTTATACTAACTGAGTAGGATTTTCTCGAA
GCAGCATCCTTTCTCCATTTAGCACTTTGTTAAGAAATCTGGTGTTTGAA
ATTTTATTAAGAAACTTACTGATGTTCACTGTTCAGTTGACTTCCTTACT
GGCTGAAGTTGGGCTGAACATCCAGGAAGCGCATGCTTTTTCTACAATTG
ATGGCTACTCCTTGGATGTTTTTGTTGTTGATGGTTGGCCGCACGAGGTA
TCTTATTATTATTATTATTATTTTTTTTCAGTTAATTATGCAACTTGAAT
ATTTCTGCTTATGAAAATCTTCTTGACGTGTGCTGCTTTTTCCATACACT
AATTAAGATCTGCTGAGAAGCTGCATCAGTCACGTTTTTCTGCATTGCGT
TTCATTAATGTTGCTAAATCTTTTAGTCATGCCCATGGCTTCTCCATCAG
TAGTATTTTGACTTAGTGTCCTTCCTTGTTTAGGAAAAAATCATTCGTAT
GTTTTTGGTTGTATATGTTCTTCAATTGTGAGTCCCTTCTGAAACTGCAT
TATTGGTTATGCATGTCTGATCTTTGGTATGTTTAACTGCATTATCAAAC
TGAAAGTTAAAGTTAAAGTTATAATTTTGAGGAGTAATTTATTGTTTTCT
TAAATTTTGGTAGAGATTAACAAGAAAGATGCTGATTTATTCCTGACAAT
TTTCAGGAAACCGTGCTGCTCCGAACCACATTAGAGAAGGAAATTTTGAA
GACTGAGGTACACCCCTTTCTATTCTTTTTCCCATCAAGAATCACTATTC
ATCTAAGTATTGTTAACAAATTTTTGTTCAATTTTTCATTTGCATCGAAC
CTGTTTAGCATTTAGGAGTCTTTTTGTGTTATATCGGTGTTTTTTAATTT
CATTTTTCGGAAAAGGATTTTCCACCACTGTAGAAAATCCTACTGGTATA
TGGTATTGATAAACCAACCAAGCCATTAATTTTTTTTATTTAATTTTCTT
TTCTTTTTGTTTTGTTTTTTCCTTTGCTTCAGGCTCATTGGGTAAATGAA
AGTGCTAGACCCTTGACTATGCTTAACTTTTGCTTAACCACCATGATTTC

AGAATAGTTATTTTTCTGTTGAATCCCACATTGCTTGTGTTACCTGATCT
TGACCTATATATAAAACCTAGGGCAATCCTCATATTACAAGGTGCCTTTT
GAGAGTGAGTTAGGCCAATTGGGTTCTTACATGGTATTAGAGCCAGAACT
CTTGGCTCATGTTGGATGTGTTAAATCCCACATCGATGTTAGGTCTCCCG
ATATCCGTTCGTAGTGGTTTTCCATAAAGTCCACCTTGCGGGTAGGGGGA
TGTGTTGACTCCCACATCGATGTTGGGACCCTCCTCCGATTTCCGTTTGC
AGTAGTTTGTCAAAAGTCCACCCTACGTGTGAGGGGGCGTGTTGATTCCC
ACATCGCTTGTGTTACCTAATCCTGAGCCATATATAAAGCATAGGGCGGT
CCTCATATTACAAGGCGTCTTTTGAGAGTGAGTTAGTCCCGTTGGGTTTC
TACATTTACAACTCTCATTGTTACCAAATTGCAAGCAAATCTTGAGTTTC
GGTCCTTTCAACTTCTTATGGTTATAAAGGTTGGATGTGATGTAGATGTT
CAGGATTTTATGCACCTTCGCCATGACTGTCTTTCGTATTCATTTTTATT
TGGGTTTTGTGGTGGTCATTCGTGCTAATCGGTAGGAGCATACCTTGGTT
TTTGATTGAAAATATCTGAGCTGCTATGCAGATGCACTCTTGGCCAAATC
AACATTCATTTTCTTCTCTGGGCGAGCAAGAGCAACCTGGGAGGATCAAA
CAAGAACCTGATTTTTTGACAATTCCAAATGATGGTACTGATGTTTGGGA
AATTGATCCTCGATTTCTGAAATTTGAGAACAAAGTTGCTTCTGGATCTT
ATGGTGATCTGTAAGTTTGCATATTTAAGACTATTTCCTGTTTGTGTATT
GAGTGCTCCATGTGGTATTGTGGTTCAAGCTAAGTGCAGGTTTTGTGTAT
CTAATTTATTATACGCTAGAACTTGGAGAGAAAAAATAATACTCTCTCCT
TGGCTGATCCAGATACAAGGGTACATACTGTAGTCAAGAGGTAGCTATCA
AAGTTCTCAAGGCGGAGCGTTTAGATTTAGACATGCAGAGGGAGTTTGCC
CAAGAAGTATTCATCATGCGGTTTGTCAAAAACTTTTCTATGCAACAGTT
AATTGTTCTATTTGTAGGGTGGTCTTGGACCTCTCTCAATTCCAAAACCT
AGCTCATGAGGTGAGACTTTCTCTCATACTTATAATCTGATCACAAGCCA
CTTTTACATCTGATATGGGATAAACTCAACAATCTTTCCCGCGCATATCG
GGCATTTGGTCAGCTAACCCTCGACTAGTCTCTCCCTTTGAGGCTGTTGG
GCCAGGGTTGTTGTAATCTAGGCTCTGATACCAGTGTTGGGTGGTCTTGG
GTCTCTCAATTCCAAAAACTAGCTCAAGAGGTGAGGTTTTCCCTCACACT
TATAACCTGACCACGAGTCCATTCCACATTCGATGTGGGACAAGTCCAAC
AATTATTATAAACCATGTTGGTATTGTTCTAATTGGTAGGTGGTACAAGG
TAATTTTTGTTTAGTAGTATACTAGATTTTGCTTGTTAATGCTCAATTTC
AGTTTTTTTTTTTTATAGACAATATTGGTTTTGACTTCTTTTTCTTTTGC
GACTTTTATAGGAAAGTTCGACACAAGAATGTTGTACAATTCATTGGTGC
ATGTACAAAACCCCCAAGCCTTTGCATCGTGACAGGTAAAAGTCCAATAA
ATTGTTACCGAGTGTCCATGGTGAGTTGCAATTTTTACTTTCATTTAAAA
AAAAAATTCCCTCATTTTATCCTTTTTGTGGGCATTTTTTTCTCAGAATT
TATGTCTGGTGGGAGTGTTTACGACTTTCTACACAAACGGAAGGGAATTT
TTAAGCTTCCATATTTACTCAAAGTAGCAATTGATATATCAAGAGGAATG
GACTACCTGCACCAGAACAACATAATCCACAGGGACTTGAAGGCTGCCAA
TCTTCTAATGGATGAAAATGAAGTAAGATGCCCTCTCTATCCATAAAGAC
ACTCATTTTAATGCCTTGTTCTATTGTTGATCTCTATCTTGTTGACCATG
ATTCTGTGAATCCCTATTTAATTTTGACTCAGTTGGGTCCTTTTAACCAT
CTTGATGATGTAGAGAACACCCAGGCCGAAAAATCATAGGGAAAACTGAT

AGATTGTCCTTGCAACTAGTCGATCATATTATTTAATCTTATTTTTTTG
TTTTGTTTTGTTTTGTTTTTTATTCTGTTTATTTTTCAATCATTTGAATA
GTTTTTAGTCCTTCAGTTAATGTTAACTAGAATGAATCTATTGAGTTAAG
GTTACTAGATATGAAATTTGTATTTGATGTGGTGATTGGTACTGGTGGTC
ATGGGAAGTTCGGTGACCGGTGGGGTAGGCCCGGTGATCAGTGACTAGAG
GTCCCGTGACCCGGCAGCCAAAGTCCAGAGTTGGTGGGTGTCATATTTAA
CATTAACTATGATACCCATCATAGTTAATGTTAACTAAGGGCTCAAAAAT
AAGTTGAAAATAAATTGTTAAAGACTAAAAAATAAATTGCTCTATAAATA
GGATAAAAGTACTTAAAACCTAAAAGTTGAGGGCCATATACTAAAAAATC
CCATGACGTTTTTCTCTGAGTAAAAAAAAAGCAAAAAATAAAACAAAATA
AAACAGTAGCTTTTGTTCTACATAAAATTGTTATTTGGTCAGAAACAAAA
AAGAGTATTCATCAGGTCGTCTTTTTTATTCTTACATGGTGCTTTGTTCT
TAGGTTGTTAAGGTGGCTGATTTCGGAGTTGCCAGAGTGAAAGCCCAGAC
CGGAGTTATGACAGCAGAGACTGGGACATATAGATGGATGGCGCCCGAGG
TGAATCCTAATTCATTATATTTTTTAAGAAGGTTTTATTGATTGTCTAGT
ATGCCCCCTTGAGTCAATGATTGTGGAGTTGCAAATATAGTAACTCCCCC
ACCTTAAAAAATAAAATAAAAAATAGAGGTTTTTGCACTTTGCACCCCTG
AAGTTTTGGAGAAATCTCACTTTAGCCCCTATACTTTCAATTTAAACATG
ACCTTGAAGTATCAAAATGTGTCAAGTAAATCTAAATCGGTAATTCCGTT
ATTTTTTATAACGGAAATCAGCCACATGTCAGACATGTGGTTTTTCTGTC
ATAAAAAATGGCGGAAGGACTGACTTGACATGATTTTGATACTTCAGGGT
TTAATGTGTTCAAAATTAAAATACATGAGTGAAGTGAGATTTCTCTAAAA
CTTCAGGGGTACAAAGTGCAAAAAGCTCTAAATTATATATACATATATAC
ACACAACTGAATATGTTTTATCTAGGTGTATATATACATATATATACACA
ACTGAATTATATATTAACAACTGATTATGTTAATTTCTTATGATGTTTTT
TGGCGTTGAAGGTTATAGAACACAAGCCCTATGATCACAAGGCTGATGTC
TTTAGTTTTGGGGTTGTACTATGGGAATTGCTAACTGGAAAGGTACACCG
GAAATTAGCAAGTTTCGCACTTTATAGATTTTCAGCTAAATTGCACTTTG
CACCCCTGATGTTTGGTTACAATCCCAACGTTATTTTTCGCAATGTACCC
CATGATTGGTCCAATCAACACATTTCAGCTATCTTAATTGCCAGAGCACC
AACACGTGCATCATAATTGAGGAAAAAATTCAAAGAAAAAAAAAATCCCAA
CCCTTGTGATACAAGTGTTGCTGCTCTAACAATTAAGATGGCCGAAACAT
ATTACAGGCAAAATTTAAACTTTAGGAGGTACACTGCGAAAATTTGAACA
TCAGAGGATATATTGAGATTGCACTCAAACTTTAGGGTTGCAAAGTGCGA
CTTATCTTTGGACTTTAATTTTGTGTTTGGCTCATTTAGTTGATATTCTC
TCATATTTGGTCCTTTTTATGCAGCTTCCATACCAGTACTTAACCCCATT
ACAAGCAGCCGTTGGAGTAGTCCAAAAGGTAAAACTACACATGCCGGTTA
TTAACATATTTTACAACCTTGAGTTTAATCCTTTCTTACAAAAGTACATC
AAAAAGGAAACTAACCGAAGTTTTATTAGATATAATCATGTAAGGGTCTG
CTTGGTTGTCCTTCAATTAGCAAATTATATTATTTTTCAATTAGTCAGTT
TATCGGATATTGGTTGTGTTGAATCCCACATCGATGTTGGGCCACCTGAT
ATACGTTCATGGTGGTTTGTCAAAATGTCCACCCTATGCATGACGAGGCG
TGTTGAATCCCACATCGATGTTGGGCCTTTCTCTCAATATCTGTTTGTAG
TGGTTTGTTAAAAAGTCCACCCTGCTGTGACGGGATGTGTTGAATCCCAC

GTCGATTGTGTTGCCTGAGTTATTTATAAAACCTAGGGCAATCTCATCTT

ACAAGGCGTCTTTTGAGAGTGAGTTAGGCCCATTGGGTTTCTCCAAAATA

GGATTATTTAGATAACCAAACGACCCTTATGTTTTTGTCCTCGTTAGTTT

ATGAGGGTGAGCTTTTGGTAGAAATTAGGCACTTGAGCATAGAACATGGT

TTAATCTTTTGAACTGCTTGTGTGCTTTATTTTACTCGGCGTTTCATGTG

AGTATTTACGTTTTTGTCCTTGGCTGATGGGTGAACTTTTGGTATGAGGG

CACTTACAACGTGGTTGAAACTTTTAAAACTCTCAGCCTTCATTTGGATG

AATTGATTTGAAATCAAGGGACTTAGGTAGTGTTTGGTTGCAAGGAATTG

AGGGATCAAAATTGGAATTAGAAATGAACGCATGCAAAATTATGTATGTT

TTTGCTTTATGGATTCTAAATTCTAAGCTATATATATATATATATATATA

TATATAATTTTTGACGGTGAATCCATTCAATTTCAAATTCATCAATCCAA

ACATAACCTTAATTGATTGCGGTTTCTTTTCGTCGGTGGTGCTTTAGGGT

CTACGGCCTACCATCCCCAAGAACACTCCTCCAACGCTTGCTGAGCTACT

TGAGAGGTGCTGGGAGAAAGATCCGACTTTAAGACCCAATTTTTGTGAGA

TCATAGAAATCCTGCAGCGTTTAGTGAAGGAGGTATGTGACTTTGGTGTA

CCAAATTATCTTGAATTATATTGTTTAAGTGTTTCTGTTCCCAAAAATCT

TGGAATAGAAACAGGAACCTAGGGATACTTCTATTATGTTGCCAGGAATT

TCTCTGAAAATATCGGAACAAAGCAGGAACAGATTTATTTTAAGAGATTT

CTATTTTTCTTCTCATTTTTCTGGCAACAAACAGAAATATGGGAACAGAA

ACACTAAACTAAACAAACAGGGACTTAGTGTTTCTGTTTTTTGCTTTTAC

TTGAAACAGAAACACAAAAATGGGAACAAATAATCTGTTCATATTTTGTT

CCAAAATTTTCAGAACAAATTTATGAAAAGAAGAACAGAAACAAATTCTT

TTTTTAATTCCCGTATCTCTGTTTCTTTCCAAGTTTTACGAGAACATGTA

AAAAATGAGTGTCCGCATGTTTTCTGGAAACAAACAGAAATATGGGAACA

GAAACACTAAACAAACAGGGTCTTAGCGTTCAACATGGCCTCAGTGTTTC

TGTTTTTTGCTTTTTTCCTGCCCCAGAAAACTTGAAACTGAAACACAAAA

TGGGAACAAATAATCTGTTCATATTTTGTTCCGAAATTTTCAGAAAAAAA

ATATGAAAAGTAACAAAACACCCTTTTTTTTTTTTTTTTTTTTTTTTATTC

CCATATCTCTGTTTCTTTCCAAGTTTTACAAGAACAAAAAAAAATAAAAAA

ACCAGAGAAACCGAAACACTTGTACCCTTGAAGTTTGAGTGCGATCTCAA

CGTGCCTCATGATGTTTCAATTTTCGCAACGTACCCCCTTAAGGTTTGAA

ATTTGCCCTAATTGGTCCAATCGACACATTTCGGCCCTCTTAATTGTCGA

AGTAACAATACTTGTATGACACATTGACTTTTTCCCCTAATTGTTCTCTC

AATTACGATTCTAGTGTTATTGCTTTGGCAATTAAGGTGGTGTTTGGTTA

GTGTTTCTGTTTTTTTTGTTTCTATTTCCAATAAACTGAGATGAGAATTC

TATTTTCGTTCTAGAGTATTCCGAGAATAGAAATATGTTTGGTAACGACC

GTTGTCCGAATTTTTTTTCTATTAATTTATGAAAACAGAAATAAGAAACA

CGTTTGGTTGCAACAATAGTTTTGTGGGAGTGAATAGAAATCAACGTTTT

TTTGCTTCCCAGTTAAGAAATTCAGAAAGTAATTCCAAATTAGTTCCAAA

AATTTAGGAACATAAACGTGTTTATCAAACGCTTATCTGTACGTTAATTC

AAAAAATTTAGGAGTGAACATGAATCAGGGAACATAAACAGTAACTAAAC

TAAACAAGACCTAAATTCGGACTGACTAGAGGCTAAATTGTGAGGGATAT

ATTGCAAAAATTAAAATCTGAGAGAACTAATTGAGATTGCACTCAAACAT

TAGGGGTACAAAGTGTGATTTCGCCTAATAATTTTGTTCTAAATGGTTCC

ATATTGAAAATCTCATGCACAATTGTTAAAATAGTGTGGATATTTTCATT
TTTTGGATTTCATATAGTTAGAAATTTTTTAACATTTGTATTAATTAATT
TGTGGAATTCGCGATATTTATAATATAAAATAGACAATTTATTAATTAAT
TATATTTTAAATTATAGTTTGAATAATATTAAAGCATAGCTTTAGATACA
GAGAATTGATAGTTCTGTTATATGACAGGTCGGAGATGAAGCAGAGGAGC
GGCGTAAGGATAAATCATCCGGTGGTTTCTTCTCCGTTCTTAAACGAGCA
CATCATTGA
>EUC16962-RA [gene]
ATGCCGAGCATGGAGTCCGATCAGGACTCCGATCGCGATTCGGAGCCGTT
CGTAGAGACCGATCCGACCGGCAGATACGGCCGTTACGGCGAGCTCCTCG
GTTCCGGCGCCGTTAAAAAGGTGTATCGGGCGTTCGATCAAGAAGAAGGA
ATCGAGGTGGCCTGGAACCAAGTCAAATTGCGAAACTTCTGCGAAGATCA
ATCCATGGTCGACCGGCTGTTCTCGGAGGTACGGCTGCTGCGAAAGCTGA
AGAACACGAACATCATAGCTCTGTACAGTGTGTGGAGAGACGAAGATCGT
AACACGTTGAACTTCATCACCGAAGTCTGCACCTCCGGCAACCTGAGGGA
TTACCGGAAAAAGCACAAACAGGTTTCGATCAAAGCTTTGAAGAAGTGGT
CGAAGCAGATCCTCGAGGGTTTGGACTATTTGCATACGCATGATCCTTGC
ATCATCCACAGAGATCTCAATTGTAGCAACGTCTTCATCAATGGCAATGT
TGGTCAGGTACTAATAATAATTATTTGGGTGTTTATGTGACTAAGTTTTT
TTATTTTATTTTTAATTTAAAAAAAAAATTCATACGTAATTTCAAGTTGA
CACAACACCCAATAGATGCGCTACACTCTCGTAATTACATGTATCTATTT
GTGTATAAAATATTTGTAAGATGTAACGAATATTACAAAAATACATACAC
CATAGTTAGATGTAACTTGTAACTAATATTATACAGTTACATGTAACTAA
TATTACATAAGCGAGCACGTGCGGGAATAATATGCAAGTAAAAATTAATT
TAAGAGATTAAATTAAAATTTATGTTTTAAATAGAACAAATAACATATTA
CACACGCTATTTAGAGCTAATCACTAATTATCTCTTGGCTCTATTCATGC
AAAAACTATCAAAATGCTAAATTGTTTATAATATACTCAAAATCAACCAA
AATGAATGAAAATGGATAAAAATTAATGCTAAAGAAATGTTTCATAATAC
AAAATTTAATTTTTCTGGGTTCTTTCATCTTGGATTGAAAAACGAATTAT
GGAAATAAAGCAATTTTGATAGCATTAGTGTCTCCCAAGATCTGAACTAG
AACTAAAATGATTAGTAGCTAATTTAGTGATTTAAATTCTTACAGCCAAA
CACTAAAACAGAAAAATTCATAAAATTAACTAATTGAAAAATATTATAAT
TTGTGAATTAAAGTATAATCAAACGGGCCCTAAGATTTTTTAATGAATAG
GTAAAGATTGGCGATTTTGGATTAGCAGCAGTAGTCGGAAAGAACCACTC
AGCCCATTCAGTTCTAGGAACCCCAGAATTCATGGCGCCGGAGCTATACG
ATGAAAATTATACTGAGCTAATCGACATATACTCGTTCGGTATGTGTTTG
CTCGAGATGGTAACTCGAGAATTACCCTATAGTGAATGCGATAATGTCGC
TAAAATCTACAAAAAGGTCACCTCAGGTGTGAGACCTCGAGCCATGGACA
AGGTCAAAGATCCAGAGGTCAAAGTGTTTATAGAGAAGTGCCTCGCTCAA
CCAAGGGCGAGACCATCAGCCTCTGATCTGCTCAAAGACTCGTTTTTTTA
TGGTATCGACGATGACGACGAAAATATATATGATTTTTTATAA
>EUC06431-RA [gene]
ATGGCGGTTTTTTTGGTGATTTGTTAGGGTTTCTATGTTATCTGCTGTTA
ATTTGTTCATGTTTTCATTTTTTTTTCAGTTGAAGATGCCAAGCGCGCA

GTCCGATCAGGCTGATCGCGATGCGGAACCGTTTGTGGAGGTCGATCCGA
CGGGCCGATACGGCCGGTACAGCGTGTTGCTCGGTTCCGGCGCCGTGAAG
AAAGTGTATCGAGCGTTTGATCAGGAGGAAGGGATCGAGGTGGCTTGGAA
CCAGGTGAAATTGCGGAAATTCAGCGAAGATCAAGCGATGAAGGATCGGC
TGTTCTCGGAGGTCCGATTGCTGAGGGATCTGAGGAACAAAAACATCATT
GCGCTGTACAGCGTGTGGACGGACGAAGAGCGCGGCATGTTGAATTTCAT
CACCGAAGTTTGTACCTCCGGGAACTTGAGAGAGTACAGGAAGAAGCACA
AACAAGTATCGATGAAGGCATTGAAGAAGTGGTCAAAGCAGATTCTCGAG
GGTTTGGACTATCTCCACACGCATGAACCGTGTGTCATTCACAGAGATCT
CAATTGCAGCAACGTGTTCATCAATGGCAATGTTGGTCAGGTAAAATATT
CATTATTTCCTTCTCTCTCTCTCTCTCTCTCTGTAGTTTAATGTTAAAAA
ATATGAAGAAATTTATTAATTGCATTAAAAATTGACCATTATTTGGAAAT
AGAAATTTGTTTAGTTATCATGTGTTGTTTGTACAGCAGGAACACTTTTT
TAGGAGACTATTAGTAAATGCGATCTCTTTGGAAATTGTGTTTGTCTCTC
ACATTCAAATCCATATGTGCCTCATTTTTCTGTCCTTTTGTTGTTTTGGT
ATTTGAATATTTAAATTGTTAGGCTAACTTGAGTCCCACCTCAATTTTTT
TTTTTTTTCCAATTTAAATTGAAAATGCAATATGTATAATTTTCTTTGAA
AACAATTACGAATCGCTTTTTAAATCAAGTTTCTCCATCAAATACCTCAG
CCAAACACAGGCTGAAAGTTCAACTCAAATTTAATTCGAGTTGAACTTTT
ATCTTTGATCAAATTTTAATGTATTAGCTTAAGTAAATTATTTAAGCTCA
ATTTTTAGAATATTGAATCAAATTAAAATTTCATAATGCCAATAGGAGTT
GGAGAAGTTAAAACTCCAAGAGCTTCTCCATATTTTAACAAATATATGGG
TAATGTTATTGGAGATGCTCTTAAATTAGCTTAAATTTCTTCTTATGCTC
TGTTGAAATAGGTAAAAATTGGTGATTTGGGTTTGGCAGCAATCGTAGGA
AAGAATCACTCAGCACATTCTGTACTTGGGACGCCAGAATTCATGGCACC
AGAGCTATACGAGGAGGATTACAACGAGCTGATCGACATCTATTCCTTTG
GGATGTGTTTGCTCGAGATGGTGACACTTGAACTTCCTTACAGCGAGTGC
GACAGCATTGCCAAGATATACAAAAAGGTTACATCTGGGGTGAGGCCTCA
GTCCATGAACAAGGTTAAGGACCCCAAGGTGAAAGAATTCATAGAGAAGT
GCCTCGCTCAGCCAAGGGCAAGGCCTTCTGCTTCTGATCTCCTGAAAGAT
CCCTTTTTTGAGGGGATAGACGACGAAGATGATGACGAAGAAAACAACAA
CAATGGTGATGTTTCATAA
>EUC24974-RA [gene]
ATGCAGGACTTTATCGGTTCGGTTCGTCGATCTCTAGTCTTCAAGCCATC
AGGAGACTTAGACGATGGTGTTGGTGGTGGATTTGGAGGTTTTGTTGAGA
AGATCGGCTCCAGCATTCGGAAATCAAGAATTGGTCTCTTCCAGAAGCCA
CAATTTCAGGCTTTGCCTCCGATTGCAAAGCCCGATAGAGTGAAAGCCAA
AAAGCATGAATCTTCTCCGATCCGGTGGAGGAAAGGTGAATTGATCGGTT
GTGGTGCATTTGGTAGGGTTTATATGGGGATGAATCTCGACTCTGGAGAG
CTACTTGCTGTCAAGGAGGTCGATTTTCAACTTTTTTTTTTTGGTTGTTT
GGTTTTGTTTTTGGTTTTGATTTATGTTATTTGAGTATTTCCTTAAAGCT
TTAATTTTCCATTTGTTGACTTGTTTAGGTATCAATTGCAGCGAATAGTG
CATCAAATGAGAAAGCACAGGTAGGGGATCCAACTGAGCTTAAAGGCTTA
AATTCATGGTCTTCTTTTGTACCGTTTCAAATATTTCAAATAATTGATGT

ATCCTAATTTAGTCTCCTTGAGTGCTTGATCGATATTAATCATTTTCATT
CAAACTACTAATCCCAATTATACGCTTCCCCAAGTTCTTTTTCTTTTTCC
TTAATATCAAGGGATGTTTTTTTCCCTTCATTACTTCATATTTTGGGGGA
AATTTTCTGAATTGTTTGTTTTGTCTGTGATGACTTATAGGCTCACATTA
GAGAGCTTGAGGAAGAAGTCAATCTGCTAAAGAATCTCTCACATCCCAAC
ATTGTTGTAAGCATCATATTATCTTCTCTTGTGGAGGAGGTGTTTCATTT
TTTTTTAACCATTTTCCATATTGTTTCTTGTAGAGATACTTGGGAACCGC
CAGAGAAAATGATTCACTGAATATATTGCTGGAATTTGTTCCTGGTGGAT
CAATCTCTTCACTTCTTGGGAAGTTTGGATCCTTCCCAGAGTCTGTAAGT
TTTGTTTAGTTGAACTGTTGAAGTAGAAATACATAATTCACAAACTTTTT
TGAAAATAAAAAATGAAAAATCCTACATCATTACCTTAAAAACTTTATCT
CTGTGTACAAGCATTAATCTTTTGATGGAAATTTAGACATGGCACATCAC
TTTGAAATGGTATGATTTAGTTGTTTACAATATTGGAATTTGTTTTCGAA
CCTATTCGGCACGTGTTCTACCAGGATTGAAAATGGGTGAAATGCTGCTG
TCTAAAATTGAATGCATGCTTTCTGAACTATATAGGGCTGGTGTTTATAG
TTTTGAGATTCATCTGAATCTGTTTGCTATCATCAGGTAATAAGAATGTA
CACAAAACAACTGTTATTGGGACTAGAGTACCTTCACAAGAATGGTATTA
TGCACAGGGACATAAAGGTATCTTCCTTTTTTTCATGAACATGTTTTTCT
ATTTTTATTTGCACTTAGTGCAGGCATAAATTTCATCTTCTTGTTCTGTC
TAATAAATGCCATGCTTTTAGGGGGCAAATATTCTTGTTGATAATAAAGG
ACGTATTAAACTTGCAGACTTTGGTGCATCCAAGAAAGTTGTTGAACTGG
TATTGACTCTTATTTTGATTTGTTTTTTTACATATGCATTTATGCTCATT
GGAATGTATTCTACGAATTGATTTTAGCTTTGCTGGTTCATTCTTTTATT
GTCCTTAGACTTTCTTGCTGGAACTCTGTTATAACAGAAGTTTGTATGAT
GTTACAGGCTACCATAAATGGCGCCAAGTCAATGAAGGGTACTCCATATT
GGATGGCTCCTGAAGTCATTCTCCAGACGGGTCACAGCTTGTTAGTGCAA
TCATCCTACTCTTCATGCTTGTAGTTAATCAATTTTGATTTCTACGTGGA
AACTGATAGTTATAATTCTCCTCTCTGTGCTGATATCAGTGTTTGATCAA
TCCTATTCAAGAAGTCTTTGAAATTCTTCGAGGGCATGCAGTAGAAAGTA
GGACTATAATCCACACTAAGTTTAACGGACCAAAGCTTGAACATTTAAGC
GGATGTCCTCTGATGGTAGTTTTGTCATTATCTTTTGTGTCAAAAAATTCC
AATGATGTGGTGAGTGATATGGCGGTGCTATTAGATGTAACAAAAAATATA
GCAAAAAATAGACAGGACATCAAAGTTTTATTTTGGCCATTATGTTCTGT
TACTTTAATTTAGACCTGCTGTAGAATGGGAAAGCTGACTCATGGAATGC
ATTTGTATCTTCAGAATTGCACTATAGAGAGCCCGTTTCTAGAAAAACTA
ACTTTAACCATTATGACAGTGTTCGTCCATCTGTATTGATTCTTACAATA
TAGATTAGCACTTCTTACCAAGTACTAGTGTCATGTTGCTTTATTAATTC
GTATTGGGATTAGTTTGCAATTTGCCTATTTTTAGGTTCTCCATGCTTGG
CCTTTCTGCTGCTTTCTCAATGGCATCATGCATGCTGTCTGCCTTTCACT
TTGGCAATCGTAGATATACGGTATTCTTGCGTTTCTTATTGTGACCACTA
GGCTCACTTATCTTTTGCTTAAAAATTATCAGCTCTGCTGATATATGGAG
CGTTGGATGTACTATCATCGAAATGGCTACTGGAAAGCCTCCTTGGAGTC
AGCAGTATCAGGAGGTTCAAACTCATTCCTTATACTTGTTATTTTGTTTC
CTTTGCTTCTATATTCACCAAGAATTTGTTCTGCTTTCAGGTTGCTGCCC

TCTTCCATATAGGGACAACTAAATCTCATCCACCCATTCCCGATCATCTC
TCATTACAGGCAAAGGATTTTTTGTTAAAATGTTTACAGAAGTATGTCAT
TTCTTCTTTAATTCTTCTGATGCCTTCATATATTTAACACCTTTTCAATT
CTAGTTAGAAGATGCTAAGCACTTTCTCTTCTCTCCACTAACCATAATTT
TGCGGTAGGGAACCAAACTTAAGGCCCACGGCTTCAGATTTGCTGCAGGT
AATTCTACATGATTATGTAACAAAAAATTAGATTCTGGATATGCAATGAT
AATCTGCTTTGGGTTATCTGGTGCATGATTTGCTTGTGCCAACAAAGCCC
AGAAATTAAGCAAATGAAGTATTTAACTGATAGGGATTACCCTTTTTTTT
CCTGATAATCTGCAGCATCCATTTGTCACTGGGTACTACAGAGAGGCTCA
CCATGTTTTTCGATCTTCAGTAACGGTTGGTGTTCGCCTGATGTTTTCAT
ATGGTTATTTCTATTACATGAATCTGATATTATTCATCTGCAATCAGCAG
GAAAGCTTTGGAAACCAGAATGCAGTACTAGGATCTGACCTTACAAAATC
GTAAGTGTTTTCTTTGGTGTTCCCCTTCTTTTGTTTTTTGCAGATTCATA
TTCTACAAATCACTTTTTATGCTACAGCATGAATCCCGAGATCAGGACAA
CTTGTTCTGGTTTGAAGGATGTTGGTGATATGAGCAGTGTGAGATGCTCC
ACTATATATCCTGAAAAATTCTCAGGAATAGGCTCAATGTGGACATCGAC
CAACAATGATGATGACATGTGTTGTATAGATGATAAAGACGATCTCATGA
TTGGTTCGTCAATGAAGTTTACTTCAACTTTGCTGTCTCATGATTTAAAT
AAGGTAATAAATCATCTTAAAAGTGTCCTGTTATGAAGAATAATATGTTT
TGATGCTTTTGTTTGTTTCATCCAGAGCTTCAATCCTATGAGCGAGCCTA
ACGATGACGGGCAATGCAAGTTTGATGGAAGTCCAGAGATTGAGAGGAGT
GAAGCAAATTTATATGCCAGTCAGGACAAGGATTTCACGTTTATGAACGG
GCCATTAGTAGCCGAGGATGAGGATGAATTAATCGACTCCAAAATCAAAG
CTTTCCTTGATGAGAAGGTTACATTTTTCTTTCCCCTAATTCTTCATTTT
TTTTTATTTTTTTTTTACCTGTTGAATCTCACATGGCTTGTGGTACCTAA
TCCTGAGCAATATATAAAACGTAGGGCAATCCTCATCTTTTAAGGCTTCT
TTTGAGAGTGAGTTAGCCCCATTGGGTTTCTACATGGTATTAGAGTCTGG
CTCTTGTCTGATGTTGGGCGTGTTGAATCCCACATCAATGATGGGTCCCT
CGATATCCGTTTGCAGTGGTTTGTCAAAGTCTATCCCACGCGTGAGGGGG
CGTGTTGAATCCCACATTGATGTTGGGCCTCCCGATATTCGTTCGCAGTA
ATTTGTCAAAGCCCACTCTACACATATGAGGGGGCGTGGTGAATCCCACA
TTGCTTGTGTTACCTAATCTTGAGTTATATGTAAAGCTTAGGGCAATCTT
CCTCTTTCAAGGCGTCTTTTGAGATTGAGTTAGGCCCATTGTGTTTCTAC
ATTTTCGATGAACAAATCTTGAGATAATAATAGAGTTTGCACTTAAAATT
TGTAATTTACTGCTCTAGGCTTTAGATCTGAAGAAGCTGCAAACGCCTCT
ATATGAAGAATTCTACAATTCGCTAAATGCTATCAGCTCTCCAAGTCATA
TCGGAAATGGGGAGAAGGAAAATATTTCGAATAATATGAATTTGCTGCCA
CCCAAAAGCAGGTCGCCCAGTCGGGTGCTTAGTAGAAGACTCTCTGCTGC
TGTTGATTTTGCCTATAATGCCAGTAGTAGCCCGGGAGGTCGTTCCAAGC
GTAATATATCAAATCTTCAAAGTTCTTCCACCAGTTTAGCTCCTCAAGAA
GTTTCTTCACCTCAGATTAATGAAGGGAAAGGTGTTCTTGGGTCTCAGCA
GGAAGCAATTAGTCCAAGGTTCGTAGATATATAAATTGACGGGTGATTTG
CTTTTGTTTTGAATTAATTGTTTCTCTTTCTTTTGGTAAGTTGAGATTGG
GGATTTTGTTTTCCACTGACAGTATGAGCTTCTCCGAGAGACAAAGGAAA

TGGAAGGAAGAGCTTGATGAGGAGCTCGCGAGAAAGCGAGGCAAGTTTTT
AAACGATTCATATTTTCTAATGTCAAATCCATATTATGCTACTTTGTGAT
TCGTTGGGCCCCGTTTATTTAGTGTTTCTATTCCCAGAAAACTGTTGGAT
TTATCTTATATCGGATATGGATGGGACTGGTGGTCAGGTTATAAGTGTCA
GGAAAAACCTCACCTCTTAAGCTAGTTTTTGAGGTTGAGAGAGGCGCAAG
ACCACCTTACAAAACTAGAAATAATCTGTTTCTTTTCTGCTCCCAAATTT
TTAGAACAAATTTCTAGGAAATTTGGTCCAAGGTTTCTGGTAACAGAAGC
AAAAACTGGGAACACATGCTATGCATGTGCAAAGCAGTTATAAGGGAGAG
GTGAGTCTGTAGTTACCAAAGGTCACCTCCCTTAATATTTTAGTAATAGA
TTTCCGGAAAGGCTTATTTTTGATCGAGGAAATCGGAACAAATTTAGCAG
AAGAAACATAATTTATTTGTTTCGTTTTTCCCAGTTTATTTCTAGAGAAA
TTGTATTTGCATGCATTAGGCTAAATTGCACTTTACACCCCTGAAGTTTG
AGTATGATCTCAATATGCCTCTGATATTTCAATTTTTGCAATGTGCTCCT
TAAAGTTTTAATTTGCCTCAACTCTGCTTGACATATTTCGGTCATCTTAA
TTTCCCGAGCAACAACACGTGCATCAAACTTGTTTTTGTCTTAAATTTTT
CTTCAAGTATGATGCACATATTGTTGCTTTGGCAAATAAGATGGCCAAAA
CGTGTCGATTGGACCAATTAAAGGCAAAAACAAATTCCAGGCAGTACATT
GTGAAAATTAAAACATCAGGGGCACATTAAGATCTCACTCAAAAATCACG
GCAAAGTGCAAGTGTGATTTAGCCTAGCCTATTATGTTTTTAGTCTATAA
TCTTCACGGCCCCTTTTTTGAGGAACAAAAACAGATATAAAAGAAAAAAA
AAAAAAATCAATTTAGCTCCCCGACTTTTATAATACATTTTTGGGAACAA
AACAGAATTGTTCGCAGTTTTTTTTTTTGTTCCAAGTTTTTTGGGAACAC
ACAGGAAATAAAAACTTGGTTCAATGTCACTCCATTTTGAATATCTGAAA
ATCCTTTTACTGAATTGCATATTACAGAAATGCTTCGCCAGGCGGGTAAA
GTAAAGACATTGTCTCCAAAGGATCGGATTATGAACCGACAGATAGATCG
ATTAAGGTTTGCATTTCCAGGACAATGAATGATCACCCAAAAAAAAGAAA
AGTTGGTTTTCGTGCTGCTTTCCATTAGCAATTACAACTGCAAAAAACTG
CATTTTACAGAGAAGATCCAACCGGTTTTTGTGAGGGAAGAAGAACGACG
TCATCTCCCAAAGATCAAACCCTAAATTGGCCAAAGTGA
>EUC09794-RA [gene]
ATGGATTCAAAAACCAATGGAAACGCTCCGAAAACTCAGAGTTTGGGCGA
TCCAGAAGCATTTCGGGAGTTGAAATTGAATGGTGCAGGAAGTGTTAGTG
AGAAAAATATTTCTCAGAGGAGTTCTACTAGCATTAGTAGTCGAGATATG
ATTTTTCGAGCCGATAAAATCGATCTGAAAAGCTTGGATGTTCAATTGGA
GAAGCATTTGAGCAGGGTTTGGTCAAGAAATACGGAGCCCCAAAGGCCTA
AAGAGCTGTGGGAGATTGATCCATCTAAGCTGGATATAAGATATCTTATT
GCTAAGGGTACATATGGTACCGTGTATCGCGCTACCTATGATAACCAAGA
TGTTGCAGGTATAAAAAATTGCTTTCTTTTTAGGGACAATGGGTAAGAGT
TCCTATATTTAAGTTCTAATTGGTCAAAAGTTCTACATGTGGTAAAAACT
TTTGATTTAGTTCCATTTCTTTTAAAAACGCGGAAGCTTTACTCATGTGA
TAATCACGCGTTCGTTACATGTAACTATTTTCTGTGTAATATCACTATAT
TAGTTATAAGTAATTAATTTTTTTTTTTTTTATTTTTTTTATTTTTTTTT
CAGTTACATATTCCAATAATATGTAACTTTTTAGTTACATGTCTCTGATA
TGTAACTAAAAAGTTTGATATTTTTCAGTTACATATTTCAATAATATGTA

ATTTTTTAGTTACATGTCTCTAATATGTAACTAAAAAGTTTGATTTTTTT
TTAATTACATTACAAAATATTACACATATAAATGTAATTATAACATGTAA
TTAAAGTACATGTAACCAAAATACCTAAATATATGTGAAAAATAACAATT
ATCAAATTGTAACTAACATTAGTTATATGTAACAGAATTCACTATAAAAA
CCCAATGTACTCACTAAGGACTCTCTACAATTCCTACAATATTCTTACAA
TTTCTGCAATGCGAAACTAAAATACAAGTGAAATTTAACTTAAAGAATTA
AATCAAAACCCAATAACACAAAATGACTTTTCACCAATTAGACTTTAAAA
ATGAGACTTTTCATCGATTTTTCCAAAAAATTATTATTTTTGAATTTTTG
AGGATATTCTGATGATTTTGAAAAATTCACGACTTTTATGGCCAATTCCC
GTTACGGATAGATCAATACTTCTGATATCGTTACACCGCGACCGTTATCG
TTGCATAACCCGATGCGGCGATTTAAAACCTTGATAAATTTTAGAAAGTA
TGGTATGTAGTTATTTTGTAGATAGAATATTAAACTTTAGAAATATACTT
TTGTTGGCTATTTTGGTTGGAAGAAGTTTTTAGTTTATGAATCTTGATTT
GGTTTTACTAATTGGGGAACAAAAATAAGTTCACATTATTTTTTGGTTAA
TATATTAGTTAATGTTCTTTTGGTTATTATTTAGCTTGTGTATTTGGCTA
ATGTGAGTTATTTAAGTTTTTTGTGTTGTGATGAATTTGTAGTGTTTATT
GTTTGGATTTAGATTTTAAATTATGTTAAAAAAAAATAAAAAAAAATAAAAC
CAAACCAATTTCAGTACTTTTTGAGACACTAAAAAGTATCCTGAGTAAGT
TATTATGGCGTAATGTAATGTCGTAAGATTAAAGTTGAGACGGTAAATAA
TGCCCTTATAGACACAATTTGTGATGCATTCATATGTCCTAGTAGAGGTT
TTTCACAACTCTTTGTATTGTCCTAAAATATTTAATTTGAAAAATATAAA
ATGATATAGCGACAATATATAGTGCCTCGAAAAGTTGGTTCATACAACAC
CTATTATTGCCTTCACAAGTTCACACTAAAAGGGCATTATTTGGTGTCCT
AATAACCTATTGGGGCGGTGTTTTATAGGGCACGTGCACAAATGTCCCAA
ACAGACCAAGTGTCACAATAAAATCTCTATCGCAATACTTTTTCTATCCC
GATAGGTCAATTTTGTTGTAGTGATTCCTCAAGAGAATTCTGGGGATATG
ATAGCGAAGACAATGACAATTGAAGAAATGAGGTTGTGTGCAGCCTCAGT
TAGCTTTGAAAGGTAGTGTAGAGGCAAAACACAAGAAGAAGAGATTGCAG
TGATAGAAAGAATTCTCCGACCAAGTGAAAGTTGAAAAGGGAAGTTTACG
GACATCACCTTTCCTCCAAATTTGGTGTTTAGACAGGTTGCCGCCTTACC
AACGGTTGGGCCCCTTTCAAGCATCAAACGTTTGGGCTTTTGGAGGATAC
ATGTTCGAACGCACCGCCTTGCCATTTGTTTCCTAATTCCCTTTATTTTC
TTACTTTGCTAGGGTTTTGTTTATGTGTGCTTATAAATAAGTAACACCTA
GGATTTAGGTGTATGAGTGTGAATATTCTATAACCTCATGTAAGGTTGTT
CTCGGTAAACCTATCTTCTATAAATAAAATAAAATTATTAATTCTTATAG
CATGGATTAGACTTACGTCGAATCATGTTAGTTCATTTTGTTGTTCCTGG
ATGTTTATTATTCTTCATCTTTCGCTTTAATTGTTGTGCTGTTAGGAATT
AGGTTTTGACCCTACTAACTCATCCACCTCCTCTTCACCCCCAACCACCA
AAAAAAAAAAAAAGTAAAAAAGAAACCTTTATACATATCTCAGGCACCAT
TTGAAAATTCAATAATATCTATTTGTTTAATGCAATATTAGATGATATAA
AACTGGGAATTCAAATAATGTGCTAAACGGAACGCAAGTTTTGTTGAATT
TTGACTTTAATGTCACTAGGAGGACATTTTTGCTTTGTTTGGTGCCCCCC
CATATTTTGAAGATTACTTGAAGCACATAACCATCCTTGATGCTATAAGA
TGTGCTTTGTTGACAAATAAAAATATGAAATTTCTCGTGGTCAATATTGT

TCACCGCATATATGTACGTAGATAAACATAACACTACATGTCTATAAGCA
AACATTAAACTCCAAAAACTAATATACATGTATATATACACACANNNNNN
NNNNNNNNNNNNNNNNNNNATACTGCGACTTTAGTAATCTTTCCAAAGCG
TGACATGAATTGTGAATATAATAGTACTAACAGGGAGATAGGTACAGTAC
AAACGAAAACTAAATTCCGACATACCTATCAAACACGGTTAAGACTATCA
TGATAGCTTAATATTGCAGGTTATCTAAGATTTATATTTGTACCAAACTA
TTCTAAGATTATGATATTGATACATATAGTTTGAGATTTCTGTGAAGTAT
ATGGCTAGAGACAGTGACCTCTGTAATTCGTTCACTTTACATTATCAATA
AAAGTGATGGCATCATCAGTTCCTCCAACAATACAGCCAAATGATTTATC
GAAACGCAGTGAACTCAGGTGACACCTAATCAAAACTGAAACTGGTTGGT
TAGTCCCAATTCTGTCTTTTTTGTATGCAACAAAACAAATAAAATGGTTT
ACACTAGCTTCTGATGAAACGAACACTGTATCCCCATAGATTAGTAACAT
TGTGTATACAACATAGAGGCGAGATAGGTCAAATATGCTAGAATATATAA
TTATTATAAACAGAGAACATGATGGATATAACATGAATGTTAATCATAGT
TGACACCTTAATTTGATGTGAATTATAACTGTGAATGAAGATAAGATATA
ATAAAATACAAAAAAAGATGAATTAAGGATCCAATTCTACTGATATATAT
CAAGATCTAATAATAACATATATTAAATAGCAACAATCAACCAGCATTAT
GACCTTGACAAATTGGGGTTGACCAAATGTGGAAATAATTAGTGGCTAGA
AGCAAATGACTCTTTTCATCTTTCCTATTTTCTATTATAAAAAATGGATT
AGACATATTAGTGTATTGTATATAACGAAAATAAACTATACAACGAAACC
ATTATAAATATAAAAAAAAGAAATATAAATTCAAATATGATCATAAGTCG
TAATTTATTGAGAAACGATTGGTCGAGCCTCTCCACAATTAGGCCATAAC
TTATATTTAGATAGATACCATCTCAGACTTAGTTAAATGGAGTAGGAATT
ACAACTCTATGGTATAAAAAATTACCACGTCAATTCACTCGAGATTACCT
ATCTCATTATTAGATCAACATCTTTTCTTGAATTTATAAATAAAATATAT
ATACATGAGTCTTATAGCTCACAACTACATAATGATTGGATATGTAAACC
TCAAAATCAACCGTAACTACAAACCACAAAACATATGAAAATTTGATGCC
TGGTCAGAGAAAGTGATGATATATAATCGAAAAATAAAAAGCCCCTGTCA
CACCTATGTATTCTTGGTTGAGAAAGGAAAAAAAAATAGTGTACCGGCCA
CACCCAAGAAAGCCGGAGAGTGATGAATGTGGTTAAGGGGAGTTTGAAGC
GCTTAGACGCTCTCCAGTATGCTTCTATGAAACGTCTACATTTGTTAACA
GAAACAACATTCGTACGGTTCTTGCCCCATAAATTTTAGCCCTGCATAAT
CACCACATTTTAAAAGATCTCTCCTCACGACCAAAATCATGGTAACAAAA
TGCATGAACCAATTATCGTCATGAGAATTCTCATAATGGCCAAAATAGTT
TTCGGGAAAATTTGAACTATTTTAAAAAAAACAATAACAGAATATCCACA
TTGTCACTCTACGTCAACAATATACATTTTCATAAAACGGCACTGTGTAA
TAATATGCGGGCACGCCCGTGACATACGCAAACCTATACTCAGATGTTAT
GGAGTACGCACTATTATAACACGATCAATCACACTCTGTTCAATTAACAG
ACTAAAGATGAGAACGACACGGTTGAATAATATCTAGATGCCGTGCGCTC
ACGTATAGATCTTATAGAACAAAATGTTGTCTCATTGTGGGATTTCAGAA
CGTATCGTCCCTTGCTATAGTGCACTAAGATAATTAGAGACATCCTCGTA
GAGGATATAAGTTTATTTGTCATCTGATGTAAAAAGTCGAGAACCTCAAC
CATTGATTAGACTAAGAACTTTTATTTATTCCTATACAGCTCTTAGCTCT
CAGTTGTAGGATTTTCTTGATAAAATGCCCACCCGCCGTGTAAGAATTTT

TGGCAGGTTGTGTCGTAACGGAAATCGGGCAAAATGTCACCCGTGTTAAA
CATTGAGGGTCAAAAGAGATTAAATATCAAAAAGGTTGAGAGAAAGAAAA
AGTAAAAGGAGTGTCGTTTTTTTATCCGGCGAATCCCCAACCCTTTCTCT
TTCTCTGCTCAGTATCTGTGTGCTCGTGTTCTCTCTCTCTCCTCTTCTCC
CGGCGTTCTCGGTCATCGGGTTCCCAGTTAGCTACATAGTACGCATATCT
CCCAGCCTTCTCTCTCTGCCCTCTCCTCGTCTTAAGTCATGAGCGTAAGG
GTTCTTTGGGTCCCTTTGCACTCAAAAGCATCAGCGATCGGTTTCTGCGT
TCTTACTGGTGCTTAGGGTTCGGCAACCCCTTTTTTGGATCGGCGTGCCA
AAGCTTTTCGTTTTGTTGTATTTTCTCAATTCTTTGTTTAATGCATTGAT
TCTTCTTCTTTTTTTTTTTTTTTTTTTAATTTTTCAAATTTCGTCCGACA
TTTGACTTCCGTTGTCGTTTTTCCGTCTCATCTTGCTAATTGAATATTTT
TTTGTAGTTTGTGAGCTTGAAATTGTTGATTGTGGAAAATTAGGTTCGGA
GAAGCTCAAAAATTTTCCTGATTTCATTCTAGGTTGCTTTAATACATTTT
CTATAATTGTTGATTTGATTCAGCGGTCAATAATAGAGGGAATGTGGTAC
TCCGGTTGGGCTTGCCTTATTTCTTCCTCTAATAATAATTAATAATTTGA
ATCCTGTTGACATGATGAATCTTATTTTGGCATCCTCGCTCTAAAATTGT
CTCGACTTGAAGTTGTCTCTGTTTGTTCCTGATTCGGCAAGCAGTCTAAT
CTTATTTTAAATCTAGCAGTAGCCCACCCATATTATCCATTGTTCTAATT
CGATATTTACAGAGTTTTTGTTATTCCTTTGAATTCCGAAGTTCCACATT
GATTCTCCAAAAAAAATGATTCAAAAAACCAATGGAAACGCTCCGAAAACA
GAGTTTGGGCCATCCAGAAGCATTTCGGAGTTGAAATGGATGGTGCAGGA
ATGTTAGTGAGAAAAATATGTTCTCAGAGGAGTCTAACTAGCATTAGTAG
TCGAGACATATTTTTCGAGCCGATAAAATCGATCTGAAAACTAGGATGTT
CAATTGAGAAGCATTTGAGCAGGTTTGGTCAAGAAATACGGAGCCCCCAA
AGCCTAAAGAGCTGTGGAGATTGATCCATCTAAGCTGGATATAAGAATCT
TATTGCTAAGGTACATATGGTACCGTGTATCGTCGCGCACCTATGATAAC
CAAGATGTTGCAGGTATAAAAAATTGCTTCTTTTTATGATCCTAATATTT
TTTATTCCTATGTGTTGATTTTTTTTTTTTTTTTTGGTTTTGTCTATTTGG
ATTTGTTCGTGTTAATTAAGTGTTGAATTGTTTATATCTATTTGTCTACT
AATATGTTCTTAAATTGTTTTGTCTGACTGTAAGGGCCCGTTTGGTTGTC
CTTCAATTTATAAATTTTATTATTTTTCTAATTATTTAATTTATAAATTG
TTATGGTTTAATATTCAGTTCAGTGTTTAAGAATCGGCTGTTACGTAGCC
GTTGCCGTTACAATTATAGGCCGATAAACCGTTTGTAGACCGATTCAATG
TTTTTGTAATATGACAATTGATAATGTGATGTACAAATACAATTTACTGA
TTTATTATATTTGTGTGATATTTTTTTGATTAAATGTTGTGTGCATCATC
TTATTCTTTTAAGATTGTTCAGTACAACGTTTTTTCGTTTTTTTCTCTTC
TTCATATCTTTAATGTGTTGTATATGCAGTTAAATGCTATATATATTTGA
TAAAATGTTCTTAGCAAGGGTTAAATAAGTAAAACAATTACTATTATTGA
GTTTTTGAGGATATTTTGGTGATTTTAAAAAATTCTCGACCGTTATAGCC
AATTCCTGTTACGGACATGCCGATACTCCGGATACTATTACACTGCATGA
GGGCCCGTTTTGGATCCCACTAAATGTTGGGCCCTCCGATATCCGTTCGC
AGTGGTTTGTCAAAGTCCACCATGCGCGTAAGGGGACGTGTTGAATCCCA
CATCGCTTGTGTTATTTGATCCTGAGCTATATATATAAAGCCTAGGACAA
TCTTCATCTTAGAAGACGTCTTTTGAGAGTGAGTGAGGCCCATTGGATTT

CTACGTGTATATATATATTTCAAACTTAAAGAATGTTTTTGTGTCAATTC
CCAACGAATATTCTCAACTAGAAATCTACGTAACTTTGAAGTACTTAGCA
GAAGCTCAGAGAAGTTGCAGAGTAAGGGTATGGAGTTTTCAGTTCTTAAG
ATGTCGTTTGTTTAGTGTGTCTGTTTTTTGTTCTGTTCCAGTAAAACATG
GAACATAAAAAGGAAAGCATGAACCAAAAAAAAATCTGTTTCTTTCATGT
TTCTAGATTTTTAGAACGAATTTTTGAGATCAGAACAGACAGATTTGTTT
TGAACTATGTAGTTGCCTTTAAATTTGAACTAATTCCACCATACTCCCAA
ATGTGGGATTAATTCTCCAGTTGTTTTCTTTTGCACTTTGTCTCTGGCTT
AAAGTTAGACTTGTTTGAAATTGAATCAACAGTGAAGCTGTTGGACTGGG
GGGAGGACGGCATGGCTACAAATGCCGAAACCGCAGCTTTACGTGCATCG
TTTCGGCAAGAAGTCGCTGTTTGGCACAAGCTTGACCACCCCAATGTTAC
AAAAGTACAATTTTTTATTTCTCAACTTCTTCTGGATTAAGGTAGTGTTT
GGTTGCATAAAATTGAAGCCTCGGAATCAGATTTGAAAATGAAACATGTA
AAAACGATAAAAATATTTACATGCATCCATTTCCAATTCCAATTCCAGGG
CTTGCTGCGCTACCAAACAACATAATTTTAATAATCAATTCCTTTGGAAG
ACATCAATAGGTTCGGAAACTGTTTTTAGAACTTCATTTCAACTTCTTCG
TCTTCCCATTTTTTTTTTCAGTTTGTTGGTGCTTCCATGGGAACATCAAA
TCTTAAGATACCCTCTAAGAACCCTTCAACCGATGGTTATATTGATCTCC
CATCAAGGGCGTGTTGTGTGGTTGTTGAATACCTACCGGGTGGAACATTG
AAGAACTTCTTGTATAAGAACCGGAAGAAGAAACTTGCGTTTAGAATTGT
GATTCAACTTGCTCTTGATCTTGCTCGAGGGTGAGTCATATTTTTGAAGT
ATATGTGAAATTATTTTAATTTATTTTTTAGTCGTTGGGTAAATATCAAC
TAAATTTAATTTATTATTGTTAATATCAACCTGATGTTGAATTTGTATTT
GGTTTTTACAGGTTGAGCTATCTACATTCGAAGAAGATTGTACATCGCGA
TGTGAAAGCTGAAAACATGTTGCTTGATATTAATAGAACTCTGAAAATTG
CGGATTTTGGAGTTGCTCGTGTTGAGGCTCAAAATCCAAAGGACATGACT
GGTGAAACTGGAACTCTTGGCTACATGGCTCCAGAGGTCTCTCTCTCTCT
CTCATTGTCTATTCTGTTCTGTTCCCAGAACATTGTTCTAAAAATATGGG
AACAGAAACAGATATCCCGATGTTCCCATATTTCTACTTCTTCTCTCAGT
TTTCAGGAAAAAGAAAACAAAAATAAAATTAGAAACACTATTTAAGATTG
GAATGAGAAAAAAAACGAGAATGAAAAGAAGTTTCTGTTCCGTTGCGAGA
TTTCAGATTGAATTCCTTGGAATAGAACGGAAAGAGATTCTTTTTGTTCT
TGTTTTTTCATCCAAGTTTTATGGAAACAGAAACAAAATCCGAGCACAAA
AATACTAAACAAACGCCATCTTCTGTGTTTCAGTCTCTCTTCCTGTGTTT
CTGTTTGCTAAAAACAAATTTCCAGGATCGGAACAAAGAGCACAAATCGG
TCTCTTCTTGTTGAATGTCGATACAGTTTGTGTGTATGTGTATTAGTTTC
CAAGTAAAATGACTTGAAAACATGATGTTTTTACGCTCTCTCTCGCGCTC
TCCTCTGTAGGTTCTGGATGGGAAGCCTTACAACAGAAAATGCGATGTCT
ACAGCTTCGGCATATGCCTATGGGAAATTTACTGCTGTGATCTTCCATAC
CCAAATCTTAGCTTCGCCGATGTCTCATTTGCTGTTGTTCGACAGGTCAA
TTAAATTAATGATTTTTTTTTTGGTAAATTGAAATATTCAACCTAGAAAA
AAACCCATAAATAATGTTAGTTTTTTCAATAATTGCAAATATTTTTGTTA
CAGAATTTGAGGCCTGAGATCCCGAGATGTTGCCCTAATTCTTTTGNTAA
TGTAGTGGTCGGCGCTCCTAGAATGGCTGTCTTCTAGAGACTGCCAACCA

GAGGTGGTGGTTTGAACTCGTGGTCAACGCATTTGGAAAGGGTCACCATA
TGAACCCTGCCGACCAGGAATAGCACGACAGGGCAGTGATTGTACGATAC
GGTGATGCCAACCAGGGGGCAGTGAAGTTAAACTCTTGTATGGCGCATTT
GGAGAGGGTCATCATATAAACCCTGCCGGCCAGGAATAGCATAGCAGGGC
GGTCACCGCATGATACAGTGTTACTGGGTAGGGGCGGTGAGGCGGAACTC
TTGGTCGGCGCTTTTGGGAGGCACCTGCCGTCAAGGGATAGCTCGGCAGG
GTAGTGTGCGCGTGATAATGACGATACGGCCACGTTGCACGGGGGAGATG
AACGCGTGGCTGGGGCTTAGGTCTAACAAGGTGACATCCAGAAATACCCT
CATCAATTGCCCCTAGTTCAGGGCGTCGAAAGGAGGGAGGTACTCAGGAC
TACCAGACTTCATACCGGATGACGTTGTCCGCATGTAAATCACTCAAAGC
TTGTTCCTTTTGGATGGAGAATGACAAGCAGACAACTAGGGGGTGCCACG
TGTCTCTTCTTCAATGATGTGACATGACAATCGAGGCATCATTTCTGTTT
CCCACCATCTTTTCCTATAAATAGGAGATGATAGAGCCTCATTCGTACAC
TTGACGTTTCTTGAGAGAATTTCCTCTACACCCTTGCTTAGTATTTTTCT
AACCGATAGCAAAAATGGCCCTTCAATTTCTCACCTTTTCTAATTCTTCC
TCTTTTCTCTCCGGCAATAGCTCAGTGTCCAGCTCATTGGAATGACCCTC
CTTTTGCCCCTTATCAAGATGCTAGTGTAGCCCTCGCTCTACAAAGATCT
TTCTTTCCGTCCTTGGCGGATCGGCTACACGCATCTGGGGAGGCCTTTCA
GTCATTGGCCAAACTGGTGACCGTGGTTGGCCGTCCTGACGATCACAGTA
GAGCAAGCTCTTCGCGAGACCCTCGTATCGTTCAGAAAGAAGGCACCGTG
ACAGAGACGTTAGTTCTAGAAGCGGTTCTCTTGAGATCGCTGATAGTCAG
CGGTGTAGGGGCCAAAATTATTTACTTTCCCATCCCCGTTTGGAGCCCTC
TCTGGGAGCTTCAGTCCAATGTTACTAAGAAAGAGTTGAGGAGGATCCAC
AGTACATACACCTTCTCGAGCTCGGTGGTGATGAGAATCCCTATTGCCGA
GTAGAGAGAACACCAGCCGCCCAAAGGTTTTCTTACTGTTTACATTCATC
AGCTCGAGGTCGGGCTTCGCTTTCTCGTTCACCCGTGGATCCGTGTAGTC
TGTAAGGAGTATGGTTTGTGTCTGTCACTTGTGAACAACATGAGGATGGT
GATAGTGTCCCACATCATTTTCAAGGCGGAAGGAATAGAACTGAGCTTAG
AGCTCTTCCGTGCTCTATTCTTCACCAAGTCCCTACCTCAAGGTGTTGGG
TAGTGCTACGTGTCCTCCAGATTGACATTTTTGACCGACCTGGATTCCAA
GATTCATGGATGGAGACCAAAGTACTTTTACCTCAGGGGGAACGATTGAG
AGTTCGGTCCTGACGAAAAGGATTGCTCGAACCCCATTTTGTGGGACTGG
GAGAGAGTCAGCAAGGAAACCTCAATGGTAGAGAAAGGTTATCAGATTGA
TGAATTTATCTTACTTGAGTTTTTGTTTCATTTGCACAGAACAAGAAGG
CCACGAAGAAGAAGAAGAAGAAGAAGAAGAAGAAGGAAAAGCTCCGACCA
AAAATCGCAGAAAAGAAAGAGAGGGTGTTGCGAACTGATTTCCATCAAGT
GAACGAGCTCCTCAAGTCGATGTTGTTTGACATCTTCTTTTACTGGGCAC
CGTCCTTACCCCCTACCGAGCTCCCATCCAAACCATCCAACAGTAAGTTG
AATATTTAATTCATTTGGTTGTCACTTTTTCGACACTTAATTCTAATTTG
AATTTGTGACTGACTTTTTGTGTTTATTGTTGGCAGAAATAGACTATGCT
GCTATCTTTAGGAAGGTTGTTGCTTAGAGAAAGGCTGGGGGTGCGGGGAG
CTCTTTCGGCGCCTAGGAGCAGGTCTCCCTCGCCCACCAGGGTGACGACC
AAAGACTTGTCAATCCATCCCCAGCATGCAGGATCGGGACCATGGGTACA
GACTACCGTAGGAGGTCCAGCTATCACCCTTGACACTGCGCACCGGGATG

TCGCTTCTTGTAGACGAATGCATGAATCAGACATATTGCACCAAACGCTT
TTTCATTGATAGAGAAATTCTTTACACTTGTAGAATTTTGAAACTTTTAC
AATAGTGGTTCTTCCGTACAAATGTACGGCCTATCCCAAGTTGAGGCTAG
CTTTCCGGCATTTTACTCATTGGTGGCCAGAGTGACCTTCCCGAAGACGA
GGTTACCGACGTCAAAGGAGCGTAGGCAAACTCGACTATCGTAATATCTA
GCTGCCCTTTGCTAGTAGGCAACTTGCCGCACACGGGCTTGTTACCTTTT
CTTCTCGAGGAGATCAAGATTCAAGGCCAAAACCGTCTCGTTTTTAGCTT
GATAAAAGACCACAATGGTCCTTAGTAAAGGCATACCGATCTTTACGAAG
ATGATGGACTCAGAGCCAAACGCCAATCTGAAGAGCGTCTCCCCGGTAGG
GGGTTCTTGAAGTTGTGTGCTACGCCTAGAGAATGGGTACAAGTCCTCCA
CCCATCTGTCTTTTGATTTGTCAAGTCTCATCTTTAGGTCGTGTAGAATC
GTCCAGTTGATGGCCTCCACTTGCCCATTTGCATGGGGGTGACCTCGAGA
AGCAAAGTGCGTCCAAATCCCAAGTCTAGAGTAGAGCTCAGTGAATAGAT
TGTTATCGAACTGCTAGCCATTATCGCACACAATGGCTTTAGGGATGCCG
AAATGACAAATCATATTCTTCCACACGAAGTCCCTGATTTTCTCTTCAGT
NTTTTTTTTTTCAGTTTGTTGGTGCTTCCATGGGAACATCAAATCTTAAG
ATACCCTCTAAGAACCCTTCAACCGATGGTTATATTGATCTCCCATCAAG
GGCGTGTTGTGTGGTTGTTGAATACCTACCGGGTGGAACATTGAAGAACT
TCTTGTATAAGAACCGGAAGAAGAAACTTGCATTTAGAATTGTGATTCAA
CTTGCTCTTGATCTCGCTCGAGGGTGAGCCATATTTTTGAAATATATGTG
AAATTATTTTAATTTATTTTTTTGTTATTGGATAAATATCAACTAAATTT
AATTTATTATTGTTAATATCAACCTGATGTTGAATTTGTATTTGGTTTTT
ACAGGTTGAGCTATCTACATTCGAAGAAGATTGTACATCGTGATGTGAAA
GCTGAAAACATGTTGCTTGATATTAATAGAACTCTGAAAATTGCGGATTT
TGGAGTGGCTCGCGTCGAGGCTCAAAATCCAAAGGACATGACTGGCGAAA
CTGGAACTCTTGGCTACATGGCTCCAGAGGTCTCTTTCTCTCTCTCATTG
TCTATTCTGTTCTGTTCCCAGAACTTTGTTCTAAAAATATGGGAACAGAA
ACAGATATCCCAATGTTCCCATATTTCTACTTCTGCTCTCTTTTTTCAGG
AAATAGAAAACAAAAATAAAATTAGAAACACTATTTAAGATTGGAAGGAA
AAAAAAAACGAGAATGAAAAGTTTCTGTTCCGTTGCAAGATTTTGGATTG
AATTCCTTGGAATATAACGGAAATAGATTCTTTTTGTTCTTGTTTTTTCA
TCCAAGTTTTATGGAAACAGAAACAAAATCCGAGAACAAAAATACTAAAC
AAACGCCATCTTCTGTGTTTCAGTGTCTCTCTTCCTGTGTTTCTGTTTGC
TAAAAACAAATTTACAGGATCAGAACAAAGAACACAAATCGGTCTCTTCT
TATTGAATGTTGATCCAGTTTGTGTGTATATGTATTAGTTTCCAAGTAAA
ATGGCTTGAAAACATGATGTTTTTACGCTCTCTCTCGCGCTCTCCTCTGT
AGGTTCTGGATGGGAAGCCTTACAACAGAAAATGCGATGTCTACAGCTTC
GGCATATGCCTATGGGAAATTTACTGCTGTGATCTTCCATACCCAAATCT
TAGCTTTGCCGATGTCTCATTTGCTGTTGTTCGACAGGTCAATTAAATTA
ATGATTTTTTTTTTGGTAAATTGAAATATTCAACCTAGAAAAAAACCCAT
AAATAATGTTAGTTTTTTCAATAATTGCAAATATTTTTGTTACAGAATTT
GAGGCCTGAAATCCCGAGATGTTGCCCTAATTCTTTTGCGAGCGTAATGA
GGAAATGCTGGGATGTGAATCCAGAAAAACGGCCGGAGATGGAAGAGGTG
GTTGGGTTGTTGGAAGCCATTGATACCAGCAAGGGAGGGGGGATGATACC

TGATGATCAGGCTGCTCACGGCTGTTTCTGCTTTGCTCCCTCCAGGGGTC
CTTAA
>EUC15935-RA [gene]
ATGGCGGGCACCGAAACAGACGCGGGTCGCTACAGGGTGCTGGTAAATCG
GTTTCGGAGTTTGGAAGTGAGTCAGGCAAAGCTCAAGGAGCAGCTCGGTG
TGCTGGCTCAAGAGACAGGCGTTGAAGATTTTTGGGAAGAAGTGGTGCCG
GAGGCCGGTGAGGGGAAGTCGTCGTACCCCGGTTGGAGTCGTATTCCTGG
TGTGTTCTTGTCGGGGATTCCTTACAGGCAGGTGCTGGATTGCATGGGTC
ATGCAGTTCATATTAGAAGAGCCGACTCTGGGGTGATCATTTACTGGTAA
CTATTCTTTTAGCCATTTTAGACCGAGCTAAATTACCTCGTTATTTTATT
GGTTGGTTATGGTTATGTCTGGTTAGTTATTTCAGTTGCTTTTTGTTGAA
TCCATGTTGGACCTGTGGTTATTTCAGTTATTTTCGTTCGTAGTGGTTTG
TCAAAGTTCACCCTGCTCGTGAGGGATTTGTTGAAACCTACGTCATTTGT
ATTACCTAATCCTATAAAGCCAAGGGCTATCCTCCTATTACATGGGGTCT
TTTGAGAGTGAGTTGGGCCCATTCGATCTCTACATAGTATCAGAGTCAAT
CATTTATCTGAGATTGGGCATGTTGAATCTCACGTCAATGATGGGCCCTC
GATATCCGTTTATAAAACTTTGTCAAAGTCCACCCTGCACGTGAAAGGAC
GTGTTGAATCCCAATGTTGGGCCCCTCGATATCCGTTAGTAGTTTGTCAA
AGTCAACCTTGGCATGTTGAATGCCACATCGCTTGTGTTACCTGATCCTA
AGCTATATATAAAGTTGAGGGGCAATTCTCCTCTTACAAGGCATCCTTTG
AGAGTAAGTTAGGCCCATTGGATTTCTAAACTTTTGGTGGAACTTTATCA
TGCAATTTTGTTGAATCCTTGCTTGTGTTACCTGATCCTGAGATATATAT
ATAAAGTCTAGGGCAATCCTCATCTTACAAGATGCCTTTTGAGAGTGAGT
TAGGTCCATTGGGTTTCTACATGGTATCAGAGTCGACTCTTATTTGATGT
TGGGTGTATCGAATCCCACATCAATGTTGGGCCGTTCCCCTGATCTTTGT
TAGCAATGGTTTGTCAAAAATCCACCTCCACCCTACGCATGAGGGAGCGT
GTTGAATCCCACATCGCTTGTGTTACCTGATTCTGAGCTATATATAAAGT
TTATGGCAATCTTCATCTTACAAGACGTCTTTTGAGAGTGAAGTAGGCCA
TTGGATTCTACAAATTTAGTTTGAAAATGTTTCCTGTTTTATTGAAAACA
TGTTTGAAATTGGTTGATGCGGGTAAAGTTGCCATGCTTGTGATTGGATT
CAAACCAAGCAGACCCTAAACAATTGTTTTGACTAGGTGGTATAACAGAG
NTATATTAAGAATAGCTGAATAGGGTTTATGTGAAATACTTCACGGGGAT
AAAGGGTTTCAATAAAGTTTTTTATCCTATTAATAGAAAAATTATTTTTT
TTATCTTTATCAACTTATTTTTCAGTCTTTCATAGTGTTTTAGCTAACGA
TTATTACGACATCACAGATAAATTCAATACTAGTTAACATCAACTAATTC
AATCTAATTTATCCTAAATAAAGGACTACTGATTTGGAATGCTAAATTTG
GCGATTTGATTTTTTTTTTTAACTCGAATACTTTAATTTAAAAGTTAGGC
CGGTAACTATTGAATCCCACACTGCTTGTGTTACCCGATCCTGAGCTACA
TATAAAACCTACGGCAATACTCATCTCACAAGGCGTCTTTTGAGAGTGAG
TGAGTTAGGCCCATTGAACTTCTACGCTAACTAAAAAATAGGTTCTGATA
CTTATGCTTTGTTACTAATGCAACTAATGACTTGCCTCTCTTTAAAAAAA
AGAAAAAAAATTACTGTAGCGAGCAGCTTGGATGAATAGTGGTCAGCGAC
GATTGGGGCTCATGGCAAAAAACACTTCTCTTTTTTCTTTGTTTTTTTTT
TTGTAGGAATCCTTCTGCGGAAAAGCTGTATGGGTACAAAAAATCTGAAG

TACTTGGACGAACAGTTGAAGAGTTGCTCATTGACGACAACTACTACAGT
TCCACACAGGAAATCATGGAAAGGTTGACAGATGGTCAATCTTGGTCTGG
TCAGTTTCCTTTGAAGAAGAGGTCTGGCAAGGTATTCATGGCGCTTGTAA
CAAAAACCCCATTGTACGAAGACGGTGTGCTTGTTGGTGTTATCACAGTT
TCAAGCGACGCAGCGGTATTTAATAAAATAAACTCAGAAACACTAAGCAA
AACTCAGGATCAGGATCATGCTAACGTCAACCAATCAGGATTTCAGAGGA
TGAACCTCAAAAACATTCAGTGGCCTCCACGAGCGCAAATGGCATCCTCA
ATTTCTAATCTGGTGCTTATTCATTCTGTCTACACATATTCACTTTTTTC
CATTTTTATTCCTCGTTTAACTTTTTGCGCCAAATTTTTACCACAGGCTT
CAAAAATGCTTTCACAAAAACGTGAAGATTATAGATGGAATGAATATACT
AATACTGCAACGGAAAGAAAGGAGATTGTAGTCGAGGGCCTGGAGGCTCA
AGACGTAGGAGCCACAAAGTCATTGCTAAGTTTACTATTGTTGTAACAGC
AGATACTTAATAAGGAAAATTTACTGTTTCAGGCAACGAAGGCGACGAAC
GGCAAAGATGGTGGGGAAAGCTATAGGAAGAATGAGTCTACATTTGAGCT
TGTTCAACCTTCTAAAATGGTATATATCTTCTCCCTTGTTTTAGTTATTT
GGTTTAATTTATGTATTAGGAGTTCATAGGATATGACAATTATAGAAATC
CCAAAGCACTGGCAAACAGGAATCTAAGAGGGTGTGTGGTTTCCAATCCT
AGTACATACTCTTTGTTTTCAAAAACCAAACAAAGGCTTTTTGCACTTTG
CACCCCTGAAGTTTAAGAGAAATTTTACTTTAACCCCTGCCCTTTCAATT
TGAAAACATTAGTCCCTGAAGTATCAAAATCATGTCAAGTCAATTCGTCC
ATCTTTTTTTTATGACGAAATTCATCCATGTGAGTGATTCCGTGTCTGAC
ATGTGGTTGATTTCCGTCATAAATAATGACGAAGCGGCCAATTTTGACTG
ATGTGATACAATTTGAAAATTTCAAAGTTTAATGTGTTCAAATTGAAAGT
ACAGAGGCTAAAATGGGATTTCTCCAAATTTTAGGGGTGCAAAGTGAAAA
AACCTCAAACAGGGAAAAAAATAGAAAAAAGAAACTTGGTAGTTTTACAA
CGTAACTCATAACCTATTTTACATAACAATTTTCACAATCCTCAATCAAT
CACGTTACTTCTTTTTTAATATCATATGCTTATGATTTTGAAGATTTCCT
TCTCACATACATATATTTTTGATTGATCACATCCATACTGTATTTCCTCA
CTTTTTCAAAATTTTCAATCAACATATTGATATCACATCACTTATCATTT
TAATTTTTAATACCTTTTTAATAAAGTTTATGTTTTTAAAAAATTATTAA
AATCGAACCAAACAAGTTCTTATAATTTTCAAAAACTGTTTTCTAAAATT
GTATTGGAAATTAGTGGAAGTTATTTTCGAGTTGTAAACTAAACGGGTTG
TAAAAATTTAGGAAGCAAATGAGACCACACATGTTTGTGACTTTGGAGTC
GAAAAATTTATTAGGGCAGTTTGGTAATGTAACTGTTAAATGCTTTGGTA
GATCATGATAAAGTCTAGTAAAAAGGGCTGCCCAGAACGAAGGCTCCCCG
CCATTGCGGGGTCTAGAGAAACTTCAATATTATAATTAGGAAGGAAACTT
CAATTTTAACCCCCCCAAAACAACAATAATACCAAAATTACAAGCAAAAG
GTAGATTTATGTCAACCTTAAGGTGGATAATGCATCTCTTGAATTACTTA
TTGGTTTGCCTGTAGGCTGCTAAGGTATTACCTGAGCTGTGTAATGGGAA
CCTCCAAAAGGACGAGGCTGGAAGCATTTTACAAAACGGCTCTTCAATTA
TTGAGCAAATAACAAATGATTCATATTTTCCAAGACGTCTACAAGCAACG
ACTTCAGACTATTGCGTCGTTGATGCTGATTATAGTAAAAACAACTTCAG
TAGAGGAATTTCGCCTGCTGTGAAAGGAATATGTCCACTTATGAGTGCCA
AAATAATTTCCGCCGATATTGGACCAGGCAATGCTAAGATCTTCCCCAGA

TGTCCTAAAGTGCCTCGACCTCTAAATCAGTTTCCAAGATCTGGCATCCA
GTTATACGCAAACGAGTTTGAGGCAGATGTAAATGATTCAAAACCCACGG
AGATGGAGAATGCATTACTATGGCTGCCGGATCCTCAAAAGCAACCCTGC
TCAGGGGAGAACAGTGGTGGCAGCCATGGCATTGCACCAAGCAAGGGTGA
AAATGAGTCCAACATGATAATAGATTGTGAAATTCACTGGGAAAATTTAC
AGATCCGTGAAGAGATAGGGCAAGGTGAACATATTGGAAAACTATAATCA
TTCGTATCCTTCTTAGTAGTCATATGCAGAATCCCACACCATATGGTTTT
GAGTTGTAACCTCCTAACATGGTATTAGACTAGATTTGGGAAATCTCGTT
TAGTTGTCCCCGATTGCTTGAATGTTTCTCTTATTTCTTCTGTTGTGTGA
TCGCATCACGTGCAAAGCGGGGTTGCACGTGTGGGTGGGTATTAGAATCC
CACATCGAATCTGTGAGGTTTCTCAAAGTAGTCTATGTATAAAAGGAGTT
ACTCCACTTGACACCATATGGATATAAGTTAGAACCTCCTAACAATATGT
TTTTTTGGTGCTTATGGGAAGTACTCCCTTTGTCCCTATATTCTTAATTT
CCTTAATTGTATATTAAAAAATATTTGTCTTATTTCCTTTTTAAGAAAGT
AAAAGTAAGTTATTTTCCAATTCTACCCTTTTGAACATAGTGCGATGACT
TCAATTTGCATTACAAATGGTGAAAGAAAATACACTTGGAAGTAGAAATT
TTGCAGTTTTATTCATTTTCTGCAAAAGATTTTGGAGGTAAAACCGTGCA
TAGTCAAAGGAGAGAATAATTTAGGGATGGAGGGAGTACTATAAACTGGA
TTGGGGGTAAAACCGTCAAAGAGAAGCTTGCGGGAAGTTTTTGTACAGAC
ACAAACCTTTTTTTTTTATTAAACCTTGTTGTTTAACCTTTACTTTTTTT
GTATTTGTTTCCAAATTTTCAGGTTCATATGCAGTTGTGTATCGTGGAAT
TTGGAATGGATCGGTATATTCTTACAGTTCTTTAGCTCCTTGGATTCAAT
GATGAAGCTGTGTGTATCTCATTTGAAAGGCCTTTTTTTTTTCTTTTAAA
TTCTTCAGGAAGTGGCTGTGAAGGTTTATTTTGGGAATCAATACAGTGAA
GTGGCCTTACTTGACTACAGAAAAGAGGTATTTTATGAAGTTGTTCCTTG
GAAAAAAATGCACCTGAAAATGGATTCTCACCCTGATTATAATATATAGA
TAGATATAATGAGGAGACTGAGACATCCAAATGTGTTGCTGTTTATGGGA
GCGGTGTTTTCACAAGATAAGCTCGCCATAGTTACGGAGTTCTTGCCCAG
GTAAGTAAGGCACTATTTTTGGTAATGTTTTCTTCAAGTATTTTTTTTTG
TAATTAAGAAAGTTAATTGCACAAAAAAGTATGAGTCATATTTTGACTCA
ACAACGAAAAACAAAATTAAAAGTGTGTTTGATAACACGGCAATGATATC
TTAGTATTATCCATATGAAAATTTGAGTTAATCAGTCCTAATCCGTCAAT
CTCTTAAAATATAAATATGAAAGAAAAAAAGAAAAAAACCTATTCTTTGG
CTTTAGAAACGTTTTGGCCCCGTTTGGTAACCCATCCCATCCCAGAAATT
CCCATTCCATTCCCAGATTTTTGGGAATTCTGTTTGGGAATAGCGTTTGG
TAATTTTTGTTTTCGTTTCCATTTCATTCCCATTTGAGAATTGTGCCCAC
TTTTTTCTTAAGAAATGAGAAACAAAAAAAAATTGTTTCTCATTATTCCC
ATTCCATTCCCAAAACACATGATTANCAAAACCAAAAAAAAATTATCGTT
ACCAAACGTGATTTTACTCCCAAAAAGAAAACAAAATTCTCATCCCATCC
CTTCCCATTCCCATCCCACTGCTAGAATGAAAACAGAAACGGTTGCCAAA
AGCCACCTTACTTTCTCACTTTGAAATGTTTTTCTTTAAAGCACAAAACA
AACACTACTTTTAATTTCAAGTAGTTTAGGCCCCGTTTGGTTATTGTTTT
TGGTCCTTGATTCCTATTCCCCCGCTCCCAAATTTTTGGGATGAATGTAC
ATAAACTCCTTTGGTAAACACGTTTCTGTTCCCAAAATTTTGGAGTAATT

TGAAACTATTTCCTGAATTTCTTAAGAATCTGAGAAAAAAAAAAATGTTGA
TTTCTATTCACTCATATAAAATTGTTCTCACTACCATCCTGCCAGCCACC
ACCACCATCCCCACAAGCTACCATCCCGCCGGCCACCACCACCATCCCCG
CAAGCTACCATACCGCCGGCCACCATACATTTTGTAATTTATGTTCTCAT
AAATTAAAAGAAACAATTTCAGAATACTCCAGAATGAAAACATAATTCCC
ATCCCATCCCAGTTTATTGGAAGTAAAAACAAAAACACTAACCAAACAGA
AACACTAACCAAACGCTATGTTATTTTTTGCTTGCAAAAGACTACTTACG
AACTTCATCAAATGGTGCCTCTATAATGATACTTATGGAAAATAACATTG
ATAGAGCAACCTGTTCAATTTCTCTCTCTCTTTTTTTTTTTTTTTTTTGG
GTTTTCTCTGGCATAAGTAAATGTTGTATTATATAAACAGGGGAAGTCTT
TTTAAAGCACTTCACAAGGGCAATCAGAAATTAGACATCAAAAGGCGTTT
GAGGATGGCTCTTGATATTGTTCGTATCTTTTTCATTTTCGCCTGAATTT
ATAATTTCTTTTGGTTAATTCTCTCTTGTCAAGAGGTTAGGAGGTTCCAA
CTTAAAACTATATAGTGTCAAGTAGAGTAACTTCTTTCATATATTGACTA
CTTTGGGAAGCCTCGTAAATTTGATGTGGGATGGTTTTAAGTTGGAACCT
CCTAACATGATATCAGAACTAGATTTGAGAAGTTTCTTTTGTTTGCCCCT
GATTGTTTGCATGTTTTTCTTCTCTCTTCTGTTTGTTTGATCGCATCACG
TGTAAGACAGGATTGTACGTCAGGGTCAGTGTTAGAATCCCACATCAAAT
TTGTGAGACTTTTCAAAATAGTCTATATATGAAAGGAGTAACTTCACTTG
ACACCATATCGTTTTAAGTTGGAACCTCCTAATTCCTAACACTCTCGCAT
GCCCATTGTCCATTTTTTTGTGGCTTGTTCTTCTTTTGCAGGCAAGAGGT
ATGAATTACTTGCATTGCATAAATCCACCAATTGTGCACAGAGACCTTAA
ATCTTCGAATCTTCTGGTTGACAGAAACTGGAGTGTGAAGGTAATTTTTG
CTCGAAAATTATTATATTATCCTCGAAAAGCCACTTAGCATTTTTATTCC
TTTTTTTCATTTTCTTTAGGTTTAATTGCCTTCTCCTCCCTATACTTTGA
TCTTGTTTCATTATGTCCCCTCTACGGAAAAAAAAATGTAACCTTTTTTTT
TTTTGTTTCTTTATTTTAACCAAATATGCTAAAAAAATGGGTGAATTAATT
TTTCCATTGAAATGGATAAAACTATCCTTTATTTGAACTTGAATTTTATT
TTGTCTTTATTAAAAAAGTAAAGAAAAATAGAAGTATAGAGAGAGAGCTA
GAGAATGTTAGTTTCTTTTCCTTCTTTTAATCTTTAAATATTAAATTTAA
ATTTAAAAAATAAAAGACAATTAAGTCATTTTATTTTTTTAGGTGAAGTG
TGAGTTTATCCCTTTCGTCAAAAGTAGAAGAGTGTGAAACTTTAGGTTAT
TAAAGTAGAGGGCTTAAAATGAAACTTAACCAAAGAACAAGGGGGTAAAA
GGCAATTAAAACTTTTCTTTGTTTCTAAGTATTTTGAATAAATTTTTACC
CTTTCAGTTAATACCAACTACTAAACTATTGATAAATGAGTGAAATATAA
ATTGTCAGGTCGGAGACTTTGGCTTATCGAGGTTAAAGATTGCAACGTTC
TTGACAGCGAAATCTGGGAGAGGAACTGTAAAGATTTTCACCTTTTCTAT
TCTTGTTAAGTTGGGCCGATGGGGTCCTTTTGGTTAAGAATTTGGATGTG
CTTTGAGGAAAAAAAAAAAAACATCTAATTTTTTTTTTACAAAATAATTCT
CACAATTATCAATCCGTCATGCTTCTTTACTCAAACCAACAAGTTCTTGA
AATTTTAAAAAAATTATTTTTGAAAATTGTTTTAAACTTTTCGACAAACT
GATTTCATGTTCTAAACCAAAGGAGACCCTAATTGTTGGTCAAATGAATA
CTCATTGTTTGTTATAGTACATGTCGTTTTATTCTTAAATACAGCCCCAA
TGGATGGCTCCTGAAGTCCTACGAAGTGAACCTTCAAATGAGAAGTAATC

TCCTGAAACTAAGCTTTGGAAGTATATACTTTTGCATTTTTTTTCTTCAT
CTTTCTCTTTTCTCATGGAGTTGGGCTTCCAAAACTCAGACCATTTGGAG
CCGATTTCTTTTAAGCTCTACAATTCGATATAAATTAATAATGTTTTTCT
TTTCCACAACTCATGACGGGGAATGGGTTATATTTCTTATAATTGTGTAT
TTTATAGTTCAATTCTTTTTATTTTGCTCGACAGGTCAGATGTCTTTAGT
TTTGGAGTTATTCTGTGGGAACTAATGACAGAGTCAATCCCATGGAGCAA
CCTCAACCACTTGCAGGTATCACATTCTTCTTTCTTCGTGAAAACCAAAT
CCACTCTTTTTAAGGGAGTGTTTGGGTGAAGGATATGATTTGATTTTGAT
GAAGTATTTCCGAAATTCATAAGTTAAAACAAATTCTTTATTTTTTGGTA
CTTCAGAAAGGAATTTGTGAAATTCATTGGAACTTTGAAATGGGATTTAA
GGGGGTAAAATGACATCTGCAAATACCCTCCTAAGGAGGTGAAAATCAAG
AACATTCTAGTTATAACAAAATGTACCCAAACAACAAATTTGCAAGTGAG
TTAATAGTTCTAAATTCAATGAATAATTCAGGAAAATTACATTTTTGTTC
CCTAATTTATGGGTTGGGTCTTTGTGGACCCTGAGGTTTGTCAAATCGTA
CATTTGGACCCTCAAGTTTCATATTTGTTTCAATCTCATAAAATTAAAAA
CGTTAACGATTTTAGATGAAAAATTAGATTTGCTCAGTACATGGCCTTCT
AATAGGGATATTTTTATTATTTGATCACTTCTCAAAGCCTAAGTATTTTC
CTAACTTTTTCTAACACCAATTCCTCTGAAGAATGAGAGAGAGAATGTTG
TCTTTAATTTAATGGAGTGATGAAATGACAAACACATCCCTATTTGAAGG
TCATGAGCTAAACACAACCAATTTTTCGTCCAAAATTATTAACAACCTTT
TCAGGAATTGAAACAAATGTGGAATTAGGAGATTAAATGAGCAATTTGGT
AAATTTCAAGGCTCACATAGACACTCACTCACTTTAGGGACCAAATGTAA
TTTTCCTGAATTGTTAAATTCCATATCAAAAGGCATTCTTCGTTTATCAA
ATGCACTCTTAATTTATCAAATTCCATATGGGCGCTTTTGAGTAAGATAT
TTGAAGAAAATATTCAATTTTGAGAAGGGATCTGTAAATTTTTGCTAACA
AAATTTCTTAAGAAAAGTTCACAAAATCCCTTCTCGATATGAAATATTTT
TCATAGGTACCTCGCCAAAAAATGGATCATAAGGTTTTTGTTTGAGTCTGT
ATTTCATTTCAAGAAGGAATCTGTAAATTTTTCTTAATTAAATTTCTTAA
GAAAAATATACAAATTTCTTCACAAAAATAAAATTCTCTTCAAATACCTC
ACCCAAACACTCCCTTAAAAAAATTTCTCATGGGAACTTTACAATTTTCT
TTTTTTTTCTTCTTCAAATAATTCACCTAAATTGACTCTAAATAGTCAGT
GAAACTTTCTTTGTTTCATCTCATTGATCTTCTATACCTCTGATTTCAAA
GGTTGTTGGAGTTGTTGGCTTCATGGATCGTAGATTGGACATACCAGAAG
GCCTTGATCCCCGAATTGCTTCCATCATCACCGACTGTTGGCAAAGGTGA
CTATGTGTTTATAGTTTTTGTTCCCAGTTTCTATTTCCGTTCTCAAAAAA
AATCGAACAAAAAATAGGAATTTGGGAACAGTTATCTTCTGTTCTAAAAAG
TTTGTTTTAAAAAAACTGGGAGCACAACAATTTTATTTTATTTTTTTTGA
TTTTTTATATTATAGGATGTCAATAAAAATCAATATGTTCAAATTTTCTT
AGCCATTTATGAAATATATATATATAATTTTTTTTTTTGAATCAAGAGAT
CGTTTATAAGAATAACACTAGAATATTATGTTAACTCAATCAAACATATT
TTTTATTTTTGTTGTTGCTTTTATTTAATTTTTTTCAATTTTAAATATTC
GAAGAAAATGTTATCAAATGGTGTTTTATTTTGTTCCCAGTTGTTTCTAT
TCCCAAAAAATTGTTCTAAAAGAATCGGGTGCAATTTTATTTTTGTTTTT
TTCAATTTTTATGTTCTCAATTATTTGGGAACAACAATATAAATTTGGGA

ACAAAAACACAAAAACAAGCAGCACCATAGTATACCAATTTATTTATTTT
TCCGTGTGCCTGTGTGAGACAATTTTATGGTTAACTAATCGAGAATTTAT
TTTTGGTAAGTAACAGCAATCCAGAAGATCGTCCTTCATTTCAAGACATT
ATACAAGAAATGGGCAACCTTATCTACCGTTACGGGACCATTGGATCAAA
GTAGGCGCCTTCATTTTACGCAATTTTTCTCGTTAAACCTTTCTACTCTT
CTACTACTTCCTTTTGATCCCTTAGGTCGTTGGTTTCATTAATTGGAGCT
TTTGAATTGAAATTTCAAACACATAAAAATCTCAAAATTTTACGTGCGTT
CATGCATAATTTGTTGTGTTTGACAATAGTACTTTAGGCTTTTCATCATT
TTTTCTTCAATTTCATTTTTATATAATTTGTTTTCGGTGTAATATTTTCC
TCTAAGGTGGCGTTTTTCCTTTTTTCTTTTTTTTTTTATTTTCTTTTTCA
AAAAAATTTAGGATGATTAATGACATTTTGCCTCTATAGTTTAATCAAGT
TGTACTTTATACTCGTGTATTTTTGTCAGCAATTATTATCTTGTGGTTTA
GACTGAAGTACAATGATTACATTTTCTATTGAAATTAACGGTTAAATCTC
AAAAAGTTAAAATCAACAAATCATTAAATATTGTTGACATTTATACTCCA
GGGGTCGGGTGGGGTGAGGTGGAATAATTTTTATTTTTATTTTTTTGGGA
TCAGGTATGAGAGTAAGTTTATCATTTTTCAAAATTTCATTAATTATTTA
ACCTTTGCACTTAAGTCTGAACCGTAGGAAAACGAGTGCTGACGAAAATA
TCACACAGATGTAAAATTCCGTCAAACTACAAAGGAAAAAATGTGATTAA
AAATTTGATAGGGCAAATAAAAATCTTGCTCCCTCAACTTTTGAGGGATA
TTTTAAGTGACCCTCCAATTGAAGATCTTAGCAATTATGGTCTTCCATAT
TTTTATTAAAAAAAATAAAGGACTTTCTGTTAATTATTAGCTTTAATTTG
GTCGGTGTTGGCATCATGTGAAAGAGCACATGTTTGTCAAATGGATAAGT
AAGCCATTTACATTTGGGAGTACAAAACAAATTGTAGAAAATTGGGAAAT
GTTTTTTCATTTTTGTTTCTATTTTTCTATACATGTTCCTAATTTTGTAG
GAATAGAAATGTTCCTAATTTTGTAGGAATAGAAATACGCCAAGTACAAC
ATTAGAACAGAAGATATTTTAATGGTCCAGATTTATATTTAAAAGATGAA
TGGTTTAATAATTTCAAATTTTTTGCACCACTCAATGGGCTTAGATGAAG
AGTTAAAATATTTGAAGAAAAAATCTCGCTTAAAAATTTTACACCTCTAT
TTCTAATACAGTTAAATTTTATTATACTATGCACCTGTGTTATTCGATCT
TGAACTATATATATGAACAATCTCTCTCTCGAGCCATCTTTTGAGAGTGA
GATATGCCTATTAATTTTTATTATTTATTATTTTTTTAACTTTATGAATT
TAAAAATCGATATAATATCTTTAAACATGTGAAATTATTTATTAATAGAT
TATGACTATTTTTTTTTAAATATTAGTTTTTCAAATATTTAGTTTAATTA
TATGTTTTATTCTTTAAATTTTTTATTTTATTATTTATTATATTTAAAAA
ATTAAAAATCAATATAGTCCATACCGTATAAATTGCAGGAAGTGGCATTG
CAGATGA
>EUC20701-RA [gene]
ATGTACAAGTCGCGATTGGGTGAACCCGTCGAGTTGGGTTACATAGAAAT
GGACCCTTCTTCTCGATATGGACGAGTGAGTTCCTGACCTAAATTTTCAT
CTCTATTTGTTTGAATTTTGAAATGGGTTTTGTTTGTTTTCGATTGTTTC
ATCATCCATGATATGAAAAAGGGCTAAATTTTTCAAAGAATTTCATCAAA
ATTGTTGTTTGCAGTTCAGAGAAATACTGGGTAAGGGTGCGATGAAGACG
GTGTACAGAGCATTTGATGAGGTGTTGGGCATGGAGGTAGCTTGGAACCA
GGTGAAGCTCAACGATGTTTTTCAATCGCCAGATGAACTGCAGCGTCTCT

ACTCGGAGGTTCATCTCCTCAACAACCTTGACCATGAATCCATCATGAAA
TTCCACACCTCTTGGATCGACGTTGATCGGAGAACTTTCAATTTTATAAC
CGAGATGTTCACCTCCGGCACCCTCAGAGAGTAATAACCAACTTTATCTT
ATTACAAATCCTAATATGTGATTTTCATGAGTTCTACCATGATAGAAATT
TAGGGTTTTTTCTACCTATAATTCTTAAAAACAGTGATTCAGTTTTATAT
GTTGAATTGTGTTATCAAACGAGATGTTAGATTGAGATTTTGATGATTGT
AGGTATAGGCAGAGATACAAGCGAGTGGATATGAGGGCGGTTAAGAATTG
GAGTCGACAAATCCTGAGAGGACTTGCTTATCTGCACGGACACAGTCCGC
CTGTCATACATCGAGACCTCAAGTGCGATAACATCTTTGTAAATGGGCAT
CTTGCTCAAGTTAAGATTGGTGATCTAGGGTTAGCTGCCATACTTAAAGA
CTCAAACCACGCTCACAGCGTTATAGGCATGCAAATCTTCCCGTTGTCTT
CGCTCTTTTCATCATATATCGCTTCAATGTAATAAATTCTGATTTTTGTT
TTTGTTTTTTTAGGAACGCCTGAATTCATGGCACCTGAACTGTATGAGGA
GGACTACGACGAGCGGGTGGATGTGTACTCCTTTGGCATGTGTGTTTTGG
AGATGCTCACTTCTGAATACCCTTACAACGAGTGCTCTAACCCGGCTCAA
ATTTACAAGAAAGTCACTTCTGTAAGCATAAACTAGCTTCTGAACTCTAA
CGGCTCGTTTGGTTGTCTAAATTATCTTATTTTTGATGAATTAGTTTGTA
TATTACAGATGATTTAATCAAATTTATGTATTGTTTAAATAGTATATGAA
CTGGAAGTAAAATGGTTAACTAGCTAATTTAAGCGTTTTTAATTCTTACA
ACCAAACACTAAAGTATAACAATTGATGTGTTGACTAATTTATAAACAGT
ACAATTTTCGAATTGAAGGACAACCAAACAGGCCGTGGCGGAGCTGTTTG
GTGCCCGGGGGGAGTCAAACCCCTCCTGGACTTTTGAAATTTTTCACTAT
TATCCCTGTATTTTTAGAAAAAGTCAATTAAAATCTCTTACATAAGCCCC
CCTGAATTATAAAAATATGTAATAAACTCCTCAAAGAAAAATTTAGCTCC
CCTCTGTAAATAAGCCCTGGTACAGCAGGTTGAAAATCTGATGAATCTTA
TTGACTGAACTCAAATAGGGAAAGCGGCCGGAAGCATTTCACAGGATTCA
TGATGGTGAAGCCAGACTGTTTGTGGGTAAATGCTTGGAGAGTGCTTCAA
AGAGGCCATCTGCAGAGGAGCTCTTGATGGACCCTTTTCTTGCTGCTGAT
GATGAAGATGAACATGAAGCCATCCCAGATGATCGCAATTCATATGTTTT
AAATGGAGGAAGAACCAAGGAAGTCCCACAAATATCGATTTTGGGTGATT
CAGGCTCTCCTAAAAGATCAGACATGAGAATCACAGGCACTATAGATCCC
CAAGATGACACCATATTTCTCAAAGTTCAGATCTCCGATGAGGATGGTAC
TATATTGTGCATGGATATATATATGCAGTTTTTAAGTTTCTGTTTCTTGG
GGTGGTGATAAGTGTTATTTTTTTTTTTCAGGGGAAGGTAGAAACATATA
CTTCCCATTTGACATATCAAGTGACACAGCGCTTGATGTAGCCATTGAAA
TGGTGAAGGAATTAGAGATCAGAGATTGGGATTCCTTGGAGATTGCTGAG
ATGATTGATGAACAGATTTCTACTTTAGTTCCTACTTGGAAGTGCAACCA
TTGTCAGCAGCATAGTTTCGGCTACCACTACCAAGAAGAAGATGAATATG
AAGAAGATAATGATGATGACGACGATAGAACCCACCACCCCTTTTACTCT
CTCTCCTCCCATTCTTCCTCCCGAGCGTCTCTCCCGCCTGCCCTTTTCAC
TTCCTGCCATGATTGGCTTCAAGGTATATATATGTTTCCCCCTTTTTTTC
CAGGGTTTTTGACACTTTGTACCCATGATCACTTTGGTCCTATAATTTCA
ATTTGAACACATGGCATCCAAATCAAAGCTCCATTACAATTTCAGAAGGA
AAACCGTCATGTGTCTTGCACCAAACTATTTTTAAAAGGACATTAAGGGC

ATTTCCATTCTCTTCCTTTGTCCTCTATAAAATTTCTATAAAAAAACGTA
TATNACGAGGTTCTGTCCAAAAGAGATGAAGATGGACAGGGATTGTTACA
GGCATTGGAATGATGGGAGAGATAAACTGACTAGGATACAATCGTTTGTG
GATGTTCGTAGGCAGCTGCTGCACCGGACACTTGTGGAGGAGATAAACAA
GAGGAGGATGTTCAAGACCGTTGGGGCGGTTGAGAACATCGGTTTTCAAG
ACCCTTGTCATGGTTTTCACCGCAAGACAAGGATTTGA
>EUC09614-RA [gene]
ATGATCACGGAGCTCTTCTGCTCCGGGAGTTTAAGACAGTACCGGAAAAA
ACACAGGAACGTCGATCTCCGAGCGATCAAGAACTGGGGAAGACAGATCT
TAAAAGGGTTAAACTATCTCCACACCCATGATCCACCGATCATCCACCGC
GATCTGAAGTGCGACAACATTTTTGTGAACGGAAATCAAGCCGAAATCAA
GATCGGCGATCTGGGTTTCGCCACCATCATGAATCAACCCACTTCCCGGA
CCTTGATCGGAACGCCGGAATTCATGGCGCCGGAGATGTACGATGAGGAA
TACGACGAACTCGTCGACGTCTACGCCTTCGGTATGTGCATGTTAGAATT
GATCACCTGCGAGTACCCCTACAGCGAGTGCAGAAACCCTGCTCAAATCT
ACAAGAAGGTGACAACCGGCGTAAAACCGGCGGCTCTGCAAAAACTGAAA
GATCCCCATGTGAAGCTCTTCATCGAGAAATGTCTGGTTCAAGCTTCTCT
CCGGCCATCAGCCATGGAGCTTCTCAAGGACCCATTCTTATCAATGGAGG
AAACCGTGTTTCAGTTTCCGACATTACCCAAATCTTTAAACCCTCCAAAA
ATCGAATCCCATCATTCTGCCATGGAAGCTTGGAGGGTTAAGGACGTGAT
TCAGTTCGGGCTAAAAGGGTTTAAGGTTGATCAGAACTCCATCTCTTTAA
ACCTTAAAATCATCGATTTATCCACCGGTAAGCTCGAGAATTCGACGTTT
GAGTTCTATCTGAGTTCCGACGATGCGCCCTCCATTGCTGTGGAGCTTGT
TAGAGAGAGAATCTTGTCGGTGAATGATTTGCCGGTTGTTTGTGAGATGA
TCGACGGGATGATTCTTGAGTTTGTGCCGGATTGGGAACCTTCATATGGT
TATTACAAGGATTCTGGGTTGAACGAAGCCATGTCTGAAAATTACAGTTC
GGAGATTTCTTTTACAGATTCATCATGCAACGCCATGTCTAAGGAATTTG
GGTATGAGCATTTCTTTATGTAG
>EUC18639-RA [gene]
ATGGAATGCAGGGATATGGCAACTCCAGTTGAGCCTCCGAATGGGGTCAA
ATCACCGGGAAAGCATTACTTCTCCATGTGGCAAACCTTGTTCGAAATTG
ATACCAAATATGTACCCATTAAACCAATTGGGCGGGGGGCCTATGGTATT
GTCTGTTCCTCTGTCAACAGAGAAACGAACGAGAAGGCTGCAATCAAGAA
GATAAATAATGCCTTTGAAAATCGTATCGATGCTCTGAGGACGTTGCGTG
AACTGAAGCTTCTGCGCCATCTGAGGCATGAAAACGTGATTGCTCTAAAA
GATGTGATGATGCCCATCCACAGAAGAAGCTTCAAGGATGTCTACTTGGT
TTATGAACTTATGGATACGGATCTGCATCAGATTATCAAGTCGTCTCAAG
CACTCACCAACGACCATTGCCAATATTTTCTCTTCCAGGTAGAGTTTTTG
ACTGGCTAAAAGGTTGATTGTTTGAATGTAAAAGATTTATTGTTTGTGAA
AGCGTTGATTATGCATATTGGAACCATACGGCTATGCATGTTTACGAACC
GAATTTTAGATAGTTTTGACTTTAGTGAGTGGCCAACTCTATCCTGGTAG
CACTTGCATACAGTTTTTAGACTTTCATGATTGCCGAGACTAGATTTACC
TAAATGTTCTACCTATGATCAGAGCAGACATGAATTAGTGACATATTGCT
TTGCAAATTGGAATCTGTACGCTCATTCACAACATTTGATGTTGCTTATG

TGAAACTACCATTTCCTCCTTGGCTCAGAATTAAAATGCCAATGTCACTG
TTTCATCTTGGAATTATTGTGTTACTGTGCGTATGTTGACATGTGGTGGT
GCAAATTTTGATGGGGATTTGTTTGTGTGCACTTCTACCTTCAATAATCT
ACGTTTGAGTGCCTTCAGACAGAGTTATCATGTCCAGGACTCATGGCGTC
TAGTAGTTGCTGCTCTCTTCATCATAGTCCTTGCCATTCTTTCAAAATCT
GAATTACCCAAGTTTCGTTTGTTTCTATGCTGCTTGTGCTTGATTAGTTT
TGTTATGAAATGTAAACTGTAAATTGAATCTCCATTTCTAGTTGCTTCGA
GGCCTCAAGTATCTTCACTCAGCAAACATCCTTCACCGTGACTTGAAGCC
TGGGAACCTACTCATCAACGCAAACTGTGACCTAAAAATATGCGACTTTG
GACTTGCACGGACAAGCAACGGTAAGGGCCAGTTCATGACCGAGTACGTT
GTCACCCGCTGGTACCGAGCCCCAGAGCTTCTCCTCTGTTGCGACAACTA
TGGCACCTCCATTGACGTATGGTCTGTTGGTTGCATCTTCGCCGAACTTC
TCGGCCGAAAACCAATTTTCCCCGGAACGGAATGTCTCAACCAGCTTAAA
CTGATCATCAACATACTGGGCAGCCAGAGAGAAGAAGACCTTGAGTTCAT
TGACAACCCAAAGGCCAGAAAATACATTAAATCACTCCCTTATTCCCTCG
GAACTCCTTTTTCCCGTCTCTACCCCCATGCGCATCCACAGGCAATTGAT
ATTCTTCAGAAGATGCTCGTCTTTGACCCTTCAAAGAGAATCAGCGTGAC
AGAAGCACTCCAACACCCTTACATGTCTCCGCTGTACGATCCGAGTTCCA
ATCCTCCGGCACAGGTCCCGATCGACCTCGACATAGATGAGGATTTGAGT
GAAGAGATGATAAGGGAGATGATGTGGCAGGAAATACTTCACTACCATCC
TGAAGTTGCTGCCAGCCAATCCTGA
>EUC24948-RA [gene]
TCGCCTCAATCGAACGAAGAGTTTTGCCTATTCATATCCACCGATAAGGC
AAGCCACTTCTTCTTCTCTCTCTCTCTCTCTCTCTCTACATGTGGTGATA
ACGATGTTGATAAGATAAGGCTCGCATTTCTAGGTTTTTCTTCTGCATTC
ATTTCGTCGTGTTCTTGGATTGTAGATTCGCTACTATTTATGACCTCCAC
TCTCAATTTTGTTTTCTTTCAGTTAGTAATTTATTAGTTTTTTGTGTCTC
GTCAGTTCGATCTTTGTTTTTATATGTCTATGTCATTCATATTTTTTTCT
CTGTAATCATGCACGACAACCACTCAGTTGACAATTCAAAATTGGCGGTT
CCAGTAATTTGTTATATATATATATATATATATGAGCGTATGGTTGAACAAG
TACTTTGATGAAGTCGTAACTGTCATCATTTGCTCTGAGTAATTCAAGGT
GTATATATATATATACTTCTCTGAAAAGTTTTTGACGGAAAAACAGCTCA
ACGATTTGCAATCGGAGAAAAGGTCCTTTGGAGGAAAAAAAGCCCTTAAG
TCCTAGTGAGTTTAGTTTTGAATCCCATTCTAATTTGTTGTTCAAAATCT
TAGATCAATTTTTTGTGCAGGTAAACAGTGTGTAGATATTTCTTCCAAAT
TGATGTACTTACTGCTGTCAATATTGATGTTTATTTAATTGTTGATATTT
ACTGTTTATTGTTGAGGAATGGAGTGAGCCGAGTTTCGGAGAAGGACATG
AAGATCTTAGTTGTGTTGGTCTCTAGTTGTCTATGCATATAACTTGCGTT
TCTTCTAGTCTTTTGAATATCAATTAGGTGACCTTCTTGACTATAGGGCT
CATGGGAAAATATTCTTCAAGTCTGTTTAGTTTAGGAAGATTAATTGGAA
TTTAGCCCTATATGTTGTTCTATTGTATTTTATGCAAACATGTTATCTTT
TCACCTGCTGGAGTAATCTTTGAGTTGGATGTTGTGTGAATTTAGGATTT
TCCCACTGGAAGGAAATATATCTATATAACATTTGTAGGCCTCTTGTGCC
TTATGGAAACAAATGATTTGAGAAGGTATTTATGGTGGATTTTTTTTGGA

ACTGTTGAATCCCATATCACTTGTGTAACTGTGTTACCCGACCCTGAGGT
ATATATAAGTAGTCTAGGGGTAATCCTCCTCTTCCAAGGTATGTTTTGCG
AGTGAGTTAGACTCATTAGATTTCTACATGTGTTGAATCCCACATCAATG
TTGGGCTCCCGATATCTGTTTGTGGTAGTTTGTCAAAGTTCACCCTACAC
GTGAGGGAACATGTTGAATCCCACATTGATCTTGGGCCCCCGATATCGGT
TCGTAGTGGTTTGTCAAAGTTCACCCTGGGGGCGTGTTGAATCCCACATC
ACTTTTATTACCTGATTCTAACCTATATATAAGTCTAGCCATCTAGGGGT
ATTCCTCATCTTCCAAGGCGTCTTTTGAGAGTGAGTTAGGCCCATGAGAG
TTCTACAGGAACGTTGGTCTAAGATGTGGTAGCTGTTTGATGTAGATAAG
AAATAAATCAATCAGACACGTATTTCTACCAAAAAATAACTAGTTGTGGG
TCCACCGAGTTAAGAGTTGGTCGACCAACAACTAATACGTTGGTCCATCA
ATGCGTTTGTTGGTTGACCGCCAGCATAAATTGGTCGACCAATGAGGTGG
ATTGGTTGAGCACCACAGAAGGCTAGGCAATTTGGTCAATTATTGGATTT
TGAACCATGTTTAAACCATTTTAAAATATTTTCAAGGCACTACGGTGGCG
TTTGGTTAACATTTCCGTTCTTCTGTTTCTACGCCAAAAAAATTGGGATG
AGAATTATGTTTCCATTCTCAAAGATACACGTTTTTTTACACCATTAGTT
CTCATATTTCTTTTTAATTTTACTTTAAATGTAAATAATAGACTGACATA
TTTCTCTTAGGCAATAAATTTTGCAAATTACATTCAAATAAAAATACGCC
TATGAGATTAATTACAACTAAATAAAGTAATTATAGCAAAGTACTTTATT
GTATTTCATTGGCCTAAATTGATGAATGGAGGGAAAAAAATAAAAAGAAA
TATCACTTGTCTATATTTGAGGCTAACAAGGATTGAAGGGAGGACCTCGT
GAAGAATTTTAAAAATACCGACCCTGTCCTTTTAAGTGAGAATAAATTGA
GATTTTTTTTGTTGGAAGGAATGGATACAATATTTTTTTTGTTTCTCAAA
ATCCCAAGAATTTTGGGATTACTCTCAAAATTTTGGCAATATTTGGGAAC
GAAAAATATTAACCAAATAGGTTTTGTTCTTAAATTTCAAAATTTTGGGA
AGGGGGACAAGTGTTTGGGAGCAGAAACAATAAAAAAAGGGGCCCATGGT
TCCAAAAAACTTCGATTATTTCAAAAACATTTCAAATTTTTTAAGCCCAA
CAAATAACCTAAAAAATATTTTTTGCAATATTTACTCACTTATGGTAAAA
TTACCAAAACACCCTTTTTGAGATAAAATACATTTAAAGTAGGAATTAAG
GGTAAATTGATTTTAACTCATTCTAGCAACACATAAGTGTGTGTGTGCAC
ACCCTAGACAGTTCAAGAAAAGTCGATACTGAAAAGTGTTGTTACAATTG
CCTAGAAAAGAGAAGATTACACGTTGAATTTTTGTTTGCTTTAACTCTTC
TTCTCATCTTCGCTTGTACTCTTCATTAAATTTTTCCTTCATGTGAATCA
TGCATATGTTGGCTTTGACTTCTCTTCTAAATTTAGAACACTTAAAAGAC
TTAAATGGGATTAGTACAAGGACATATATATTAGCACACGTAGAATGAAA
TTGAAACCTCTAGGCTACGGACAAGCGCAGTGGTAAGTTCATGATTTGTG
TTTATTTATGACAATATAAGCTTACTCAACTGTGCACAAGAATGTTGGAA
ATAGTTTATCCAATCAATTGTCAAATTTTTTTTACGATGTTTAGCGGACT
CCATTTCATGATTCTTCTTAATCAGAATTTAAAATGTTTGAGAGTTATGT
TTTTCTGAAAATATTGAAAGATTGCTGCTTGTGACTTTGTTTTAGCCACT
CTACGTCCAATAATTAAGCTCTTCTTTATATTAATGGACAAACCAGTTAT
AGTAAGTCCCACAGGAAAACACATGGATTCCAGGAAAGAAACACCCAGTA
TTGGCTAGCTGTTGTTAGAAGCTGCCAAATACTTTACAAATGGGTGGAGT
AATGTATTTTCCAGAAGGGATTTCAATAGCAGCATCCAAATGTCTATACG

AACTTAATTTATATGTTGGGGGGTAGAAACGTAGAACTTGAACCAATGGG
TCTTGGAAGATAAATAATCGTGGAATTCTTTTTCTGGATGAGTGAAATGT
TTCCTTCGCCCCTGTTATCGGGGAACAGCTTAAATAATGATATGATGTAA
TCTGGTCCTTTGACTAATGCATATTAGATGTAAAACAAGTTCATGGGACT
TGATTCTATCATATTCCATGTCTTGTTAAAAATACTATGCTATGTCATAA
TTATTTTGTGCATAAATTTTAGTGTGAAACTACTTATACCTGACAACCAA
ACGGTCAAGGTTGTCCATTCTCAAGGGCAGCTAAATTCTCGTGCTAATTA
TGCCTGGCTATAAAAGTAGTTTCCCTTCTACAAGCTTTTTTGTTATTTTA
GAATAAGTAATTTGTTTTTGTAACCATTTAAGAAATAATAATGGTGGTTT
CTTCTCCCTTTACTTGTGGCTTTCTGTAGTTCTTTAACCTCATGATTGGT
GCTAATAATGTTTTATTTTAATTCAGAATATGCTTGACAAAGATTTCTTC
ACCGAGTATGGTGAAGCATGTCAGTATGAAATCCTTGAGGTTGTTGGCAA
AGGAAGTTATGGCGTAGTTGCAGCTGCGGTTGATACTCACACTGGAGAGA
AGGTAGCTATCAAGAAGATGAAAGATATATTTGAGCATGTTTCAGATGCC
ACTCGCATTCTTAGAGAAATCAAGCTTCTCCGGCTACTCCGACACCCAGA
TATTGTAGAGATAAAGCATATAATGCTTCCTCCTTCTCCCAGAGAATTTA
AAGATATATATGTTGTTTTTGAGTTGATGGAATCTGACCTTCACCAAGTA
ATCAAAGCAAACGATGATCTTACTCCTGAACATCATCAGTTTTTCCTGTA
CCAGCTTCTTCGAGGTTTAAAGTATATGCATACAGGTTGGCGTTTTATTG
TTATTTTTGCTTTAATTAATTTCTTTGGCCTTTCATAAATAGAATGTTGA
TTATTAGTTTTTTTCCTACTGCAGCGCATGTGTTCCATCGAGATCTAAAA
CCGAAAAATATCCTTGCTAATGCTGATTGCAAGTTGAAGATTTGTGATTT
TGGGCTAGCACGCGTATCATTTAATGAGTCCCCATCGGCTATTTTTTGGA
CTGTATGTGTCTTTTTCCTGTAATAATTTTTTATCACTTAAGGTTATTTT
AAGAACCTTTGACCTTAAGAAAAATTTCGTATCTTCTCATTGGTAATGCA
TTGGCTGTTTTATTTATTAAAACCCCATTTAAAACTTTCCTGACGGTTTT
CTACTTTTAAAGGATTATGTGGCAACTCGGTGGTACCGTGCTCCTGAACT
TTGTGGTTCTTTTTGCTCCAAAGTAAGTAGAACCAACCATGCATGCAATA
CATTCATATCTGTGTGTTTCCTGATGAATTGAAGTTGTATATATTATCCA
TCATGACAATGATATTTTCTAGGAAGCTATCGAAAGACAAATCGATACTG
AACTGCATTATCTGTTACTGCCCATCAAATCATGATCAGATGGCAATAGT
CATATAGTGTTCCCATGTGATATGAGTTTTAGACCCCATTCCCCTCTATG
TAAATGAAGTCCTTGCATATATCCAGCCTAGTGTGCCCTATAAAGTCACA
GCCATTTATTAACTAATTCTTGCTTGTTCAATCTCCGCTAGTTGGATGAG
GATGATTGATCTTTCTGTCAGATCTCTTCTTCTTTCTCATACTGGAGTTA
TCTTATCAGTTAACTGTAGCTGCTGTTAATCTTGGGCTTAAATAATATTT
AGAAATAATTATGGACAATGTCTGAAGATATACTTCTAGGTAGATAATAT
GAGGGATTCATTTGATAGATTGATTGTGTAATTGTGATAATATTTAACAA
TGTAATGCAGTGGAATAAAGAAATCACAATCCAAACAACATATAATAGTA
AATCATAGGAGGATAGAGATAGAGACAATCAATCACATAACACACAAGGA
TTTCTAGAGTAGTTCAGGAAAATCAATTTGCCCACATCTACTCCTTTGAG
CTCCTTCGGAGAGTTCTAATCCACTAACGGCTCGTTTAGTTGGACGGAAT
GGAATGAAGGTGAAATGGAATAGAATAGAAGAAGTAATGAAATGAGAGAA
GGAATGAAATGGAATTGTCAATGGAATCACTATTCAATTCACTTGTTTGG

TTGGTATAGAATGAGAGAAGGAATGGAATAGTGAAATATAATAAAATTTC
TATTATGCCCTTATAATAAAATTTTATTATATTTTACTATTCTTTAATTT
CTGTTTCTGTTCTCTAATTTTTGGATTGGGCTGGATCAACGATGGGCTTT
TTTGCAATTTTAGTTTTGATTTTGTAATTTTAGTTTTGATTCCATTGGGC
TGAACTTGATTCCAGCCCATTTCAAATGGAATTCCATTCCCTTAGTTTTC
ATTCTAATAGAATCACAAAAGTGATTTCATTGGAGAACCAAACAACGGAA
TAGGAATCGAAAGCAGAATGCTTTCCATTCCTCTCCCGATTCTGTCCAAC
CAAACATGCCGTAAGTATTGGATCTTTTTCCTTGGTTAAGACCAGACCAT
TACAATCTCTCTCTCACAGGACAAGTTCATTTATGTCCTAGCAAAAGCTT
ACAACTCTCACTAGACAAGGTCGTTTATGCCTAGTTACTCTCACACAAGG
TAAGGTATGTTTATATCTTGCTACAACTCTCTCATAAGACAAACTATGTT
TTTGTCTTGCTACATTCTCTTGTCAGACAAGTTACATTTTTGTCTAGTTA
CAATCTTTCACAAGGTATGGTTGTTTATACCTTGCTACTCTCTCACAATA
CAAGCTATGTTTGTGTCTTTCTACATTCTCTCACTAGACAAGGTATGTTT
ATGTCTAGCTAATTCTCTCACAAGACTAAGTTATGTTTATGTCTTGCGAC
ACTCATACCAAGGTTTGCACCCCCTGGATTTTCTAATTTCTTTCCCTAGG
CAGAGATCATGCCTTACCAAGGTTGATCACTTTGGATTTCTCAAGTTCAA
CAGTAATCAATATAACTCTCAAAGAAGCAAATACAGTTACAAAAATAAGT
AGACAAAACTCAAACTAGCTTGTATCTACCCTAGAGATGATTCAATATCC
TGAATAATCTCTTCCCCGTATAGATCTAGTCGATGTTTATTAATGATTTT
TCCCTTTAGTTCTTTATTCTTCAGTTCAATGGTTGAACGTGTCATCTTTT
GAATAGCATTCCATCAAGATGTAGATATAATATGCAAACGCTTGACAGTC
TAAGTACACTGGACAAGTGACTTTAGACTTTGTGATATGTCCCAAAGACT
AACGGAGCTTCTAAACACTATCTTAAGATGTATATCAAAGAATTAAGTCA
TCTAGTGTTTTTGTGCTGTCATGCATTGTACACTTTATTTCATGAATGTT
CTTGAAGTGCTAAGTGTAAACTAGACTAATGATATTCTCATTAAGGTTTA
ATTCTAGTTTTAAGATTCAACCATTTATATAGGCGTTGTTATTTCTTGAT
CACACAAGAGGATATACCATGAATACATGGTGACAATGATCATGATACAG
ATAAGCAAGTAAGAGCAAGTAGGAAGTCATAACATCATGTTCATGATACT
TAGCCTTTTTCAACTAAGATCAATATGTGCAACTTTACGTCCATCAATGG
CTTGGGACATAGTGCACGCTTAATCACATATAAGATGTTATTAATAAGTA
CATGTTATGTAAAGAATCAATTTATCAACTCAGCTTAAGAATAAGTACAT
GTGATGTAAAGAATCACATATAGGATGTATGATTCATTCCAATGTGAAGT
TAAGACACTTTTCAAGAAAACAATTAACTATTCGAAAGTTTAGTGAAAAT
GTCCAATTTAGCTCACCTCTCATTGGAATGGTATGCGGGAGATTTAAGAT
CTCGATGAGTAATTTAATCTCGCCGGCGGTCCGAATTCTTCCTATGTCCA
ATTACCAATTCCAATATATCTTTTTGATTTATCTTAGAAACCTCCTTTTG
GTTTTCACAAAACGTTGAATTTTATAAGTAATGAATGTAACTCTCAGAAA
GCACAATTTGAAAATCCATTTTGTTTTTATTACAAAATCAATTTTATATA
CTTTATCATCTAGATATATAGATATGGTCAATATAAACAACTTTGATAGA
ACAATTAGTTAGTAAGGACTTAAGAAGGTTCTAACCCCCTCTAGATTAGT
GGTTAGTTTGTTAGAGATAGTTTGTTAGTAGAATTTGTTAGAATGTTTGT
TAGGTTGTTGGTTAGTTAGAGTTTGTTAGCTGTGAGCTTAAGGTTGAGAT
CATGTTTCAGCTATAAATAGCATTGAGGAGGGATAGGAAAGGTACGGAAA

ATCTGAGATCAAAAGGGAGAGACTAGGCTCTCGAATTCCTAGCTTCCATT
TCTGTTTTTCTGAATTCTTCATNGGTATTAAAGGCCATTCGGGTCGGGTC
TAGATCCACTGGGTCATGGAGCAAACAGTCGGGTTCACCTTATTTGTGCC
CTAAGTATTCGACGAACTTCGCAACGACAACGATTCGACTCACGAATTCA
TTCCGATCACTGTCGACGACGACCACTGGTCACGCCGGACCAGATCAGAG
GAAGACGCCACTGGAGGAGATGTCGGCGACACAAAAATCATTGGCCTATC
GGCAAATCACTGACTCTGAACTGCTGAGCCATCACGCGAGAGAATTGTGT
TTTCAATTTTTTGTTGATCGTGCATCAGGAGAATCCAGCATCAAAGAAGG
TGAACTAGATTTAATGTTCCACAGGCATATGATCTATGATGATTTTAGAG
GAGTTGGTGAAGCTCTTGAGGAGACAGTTTGCGTGAACAAGACTTACGTG
GCGTCAATAGCAGGAATGCGATTTATGTGGAAGAATATCGTTCGGTCCAC
TAGAAAAATCAAGTATAGGGACAAAGGCTTTAGTCGTATATTGGTTCATA
TCATGGGTTTAGGACTAAGAACTGTGCTAATTGCTATATCGCAATGTGTT
CTACAAAATTGGCAGCATGAGTTTGGGAAGTGGACACTTGAAGAGTTGAA
GCCTCGTCGTGTCTTCATGCTCAAAGATGGACCGAGGAAGAGAAAGATGG
ATTTGTTTCCTAAGTGGAGCTCTAAGTTAATGTCTAAGGGGAAGATCCTA
AAAGAAGCATCATATAATGAACTACTGGTTTTTTGTCAAGAATTTCAAAA
CCTTGTGAATGCACATAACAATACATCCGGTTTTCAGAAGCAACAACTAA
TGATTCTCAGCAAAGAAGTAAAAGACCCAAAGAGAAGATTCAGAAGATTC
AGATTGAGGACCTTGAGTGTGTTACACAAATAACGCTAACAACAAACACT
CAATTATCTACTTTTCACCTTGAGGACAAGGTGAGAGTTCGAGGGGACGG
TATTGTTAGAACAATTAGTTAGTAAAGACTTAAGAAGGTTCTAACCCCCT
CTAGATTAGTGGTTAGTTTGTTAGAGATAATTTGTTAATAGAATTTGTTA
GAATGTTTGTTAGGTTGTTGGTTAGTTAGAGTTTGTTAGTTGTGAGCTTA
AGGTTGAGATCATGTTTCAGCTATAAATAGCATTGAGGAGGGATAGGAAA
GGTACGGAAAATCTGAGATCAAAAGGGAGAGACTAGGCTCTCGAATTCCT
AGCTTCCATTTCTGTTTTTCTGAATTCTTCATTCAATTGATTGAGTGAAT
TCAATACAATTTCTTTTCATCTATCAATTTCTTGTTCTACTAGTTCATTT
CATCAAACAAATATTGGAAGATATCTATCAAACTTCTAGATGTGTAAGTG
TGCATACAAGTAGATTAATCATCTCACCAAGCATGATATGCATAAAATGT
CTAGACAAATAAGATGGAGCATGTAAAGCATGTTCTAGATTTTTTCAATA
AAGCACATAATAGACTTGTTCCAAAATTTAAACAATTAAGTGTGAGGTAA
TGTATAATGGAACAAACTCACTGGATGTAATGATAGTGATGTGGGCGTGT
TGCTTCTTCTGGGATGACCAATTTTCTCCTTTTAACCTCCCTCACAACCT
TAGAGCATCATAAAGTTTTGCAAGAAAGCTCTACTCTAGGTTTGACACTC
ATAAGATCACACTTGTTTTTGTAGTGTTTTCTCACATTATATCACTCATT
TTAGAACTAAGAGGAGTCTCTAAAGGATGGTAAATATATATCAATAAAGC
TCAGACCTTCATCCTTGTGAAAGGCGTGCAACAATGTGGGATTTATATAG
GGTTGGTCAAGGTAGTGAATTGCACCAAATCTAGGATTAAAAGGCTATTA
ATTTGTTATAATCAATTAATTGAATGTTATGAGCATGAAATATGATCACT
AACGGTCAATTTGGTTAATTTGGGCAAGAACTTGTTGGGAGACAAAGTGC
ACCAAGAAAGGATGAAATTTAGGGTTCAAAAAATGGAAACATTTGAACAT
CCAAGTTGATTCGACATTCTTCACATCAAGTTGGCGCATTGTCCGCTCAG
AGGATACGACAAATAATGAGCACAATACAAACAGTAGTAGGTTTGATTAC

AGGACTAAATGTCTCATCAAAATCAACACCCGCTTGTTGATGGAAACCAT
TGGCAACTAAGTGAACTTTGTATCACTTAATGGAACCATTTGCTTTGCGC
GTGAGTTTGGAGACTCACTTACAACCCACAACATGTTGATGTGGGGGAGG
GACAGGAACCCAAGTGTTGTTCTTCAATAAGGTATTAAATTCACTGGTCA
TGGCATGACACCATTAAGGGACATGAACAGTTTGAGTATGGCAGGTAGGC
TCGTCAGTGGGAGATGAAAGCTGAGCAAATCAAGCCTGTAGAAGAGGATA
TTGGACTGTACTATCTATCCGAAGAAAAGGGCGACGAGTGCCATCCTGAC
GACGTGTTACCATTGAATGTTGCGAAGAAAAGTGGAGAAGTAGGAGTCTC
ATGCGTGGCAGTAGCATAATGATTAATTTGTGAGGGTCAAATTTGTCGAT
GCTGTTAATGATATGGAAATGGTTGATGAATTGGAGGTGGAGCACTATTT
TGTGGAACCAAGAGCTGGTGGTAGTGGAGGAATAAAGGCCCATGACGATG
GGTTTGGTGGCTTCTGGTGTTAGATGCAAACACAAAGTGTTGTTGATTGA
AATGAACATACCGAGAAATATAAATTTTGCCAGTTGTGCAGTCCAGACAA
CGGTAGCCCTTGTGCTGCAAGCTGTAGCCTAAAAACTCACATTCAACAGT
TCTTAGTAAACGTTTATTAGTTAGGTAAGCACCTAAATGCGGATAACATG
CATAACCAAACACACGTAGTTTAAAGTAGGAAGGAGTATGGCCATAAAGA
CGGGAGAATAGTGATGACCACTGAAGCTTGGAGAAGGGGAGACGATTGAT
TAGGTGAATAGCAGTGGAGATTGCTTCGGGGTAGTGATAGATGGGAACAT
GACTTGTTAAAAGGAAAGCGCGGGCCATGTCAGCAATGTGGTGTTGTTTC
TGTTCGGTAAGGCTATTGTGTTTGGGAGTGTGAGGACAAGAGAGCCAATG
ATGAATACCTAGGGAAGAACAAAGCGATCTAAAGAGGCTGCTACCATATT
CCCCACTACCATCACTTTGAAATTGTTGTACATGTGTGGCAAATTGAGTT
TAGAAATAGGCTAAAATGTTTTTGAGATGCTGTAGAACCTCAGATTTTTG
TTTCATAAAGTATAGCGAAGTAAAACGAGTGTGGTCGTCAGTGAACAACA
CATAATACTTATAGCCAGCGACAGAAAGCAAAAGGAGATTTTTATTTCAT
GTGAGCTAGAAAATGGGAGATGTGTGGATTTACCCAAAGCAGACTCTGAG
CAGAAAGAGGACTTAAATTTAAAAGGAACTGATGACAATTTATTAAAACC
CAAATAAGACATGACTTTAGCAAATGGATGACCTAGCGGATTGTGCCAAA
GCGTGGACGAGTTGATCGAGGTAGATAAAGCGTTTAGGCGGTCAGAGATG
ATAGGTACCTTGTAGAGACCATCCTTACATAGGCCCTGATAGAGGAGAGA
ACCTAAGGTAAGATCAAAGATATAGAAGTGGTGAGTATCAAAAGTACAAA
GAACATGATTATCAGTGCAAAATTGAGCAATGGTGAGTAAATTTTTACAC
ATTGAAGGAACGACATAGACATTATTAAGAAGAAAGGAGGAAGAATCAAG
TGGTAGTGTTATGTTACCCGTATAAGTTATTGGTGGGTGATCACCATTGC
CAAGTTGAACAGAGTCAGAACCACTGTATGGTGTGTACTGCTGTAGGAGA
GTGGGGTTGCCGTCATGTAGCTAGATGCACCAGTGTTAGGATACCAATTG
GAATCATTGAGTGACCACTGTGAAGTTTGTTTGCTCTGATGCGCTTGAGT
TGGAGCAATCAGTAGGTCTACAAAGGTAGAGTTCTGCTATGGAGGAACAT
ATGGTTGTGCAAATGAAAAGAGCCGGTAGCAATTTTGGGCGAAATGACTA
ATCCCAAAGCACAATTGACATTGAGACATAGATGTGGGTGTAGGAGCATG
GCCAAGAATACCCGAGGGATTGGTGTTGTTGGTGGATTGGCGACCAGTGT
TTTTGCGACCTTTGTGGTGAGAATTTTTTCGTGGAGCGGCACCGGAATTA
GTCTGAAGAGCGAGGGTGAGTATGGCTGGACTAGGACAGATATGCGATAA
GTAGCAGCTAGGGTTTCCTGGTTTGCAGGTTTCTTTTGTTTTTTTTTTTT

TTGGCAGATTGGTGATGGATCCTCAAGTTTATGAGGAATCTTAAGAGATT
TGAGCAATGAGCAGTGGAGGTAGGATCAAAGTGAAGTTCCGAATACCAAT
TGATGATGAAGGCGGCCAATTGGAAAAAAAAAAAAACAATAAAAAAAAGA
AGAAATCTTGCTTAATAGCTTAATAATCAGTGATCAAGCTTTTCTCTTCC
AGTGCATTAAAGTCCATTATATACGCTTAATCTAAATATGAAAATAACAA
ATAATCCCATCAATGGATTATCTCAATTAATGGGAAGGAAATAATTTAAA
CGAGAATAACAGCTACTAAAATAAGGCTGTGATTAACTAATTTGGAAATA
AACACATTAGTGATTTTTCCGCCCCACGTTGTACATAAGTTCGAGGCAGT
GGCCTTGGTTGATTCCCACCAAGTTTCTGAAACTTTGTACCTTTGCAGTA
CACCCCTGCCATTGATATTTGGAGCATAGGATGTATATTTGCAGAAATGC
TTACAGGGAAGCCATTGTTTCCTGGAAAGAATGTGGTGCACCAATTGGAT
CTCATAACCGACTTGCTTGGTTCTCCCGCGGCAGAATCTGTTGCAAGGGT
TTGTCCTTCTTCCTTTACTTGTAGAACTTGGTTATACCTTTTGGGCCAAG
TTCGAGTTTTAAGGATGTAGATGTTGTGCTCATATTCTCTCAAAATTTTG
ATTCTTACTTTTCAGATTCGGAATGAAAAGGCAAGGAAATATTTAAGTAG
CATGAAGAAGAAAGCACCTGTTCCTTTATCTCAAAAATTCCCTAATGTTG
ATCCTTTGGCTCTTCGTTTACTTGAGCGTTTGCTTTCATTTGATCCCAAA
TTTCGTATATCTGCTGCAGAGGTTAGATCCAGTAGATTAACTGTCCAATG
TTGCACTCTGAATTTGGAAAGTTATTCAAACGTTTTACTTGGGGATATTT
GTATTTCCTGATACTGGGGAGTGTATTCTTTTCAGGCATTAGCAGATCCT
TATTTTCGTAGTGTTTCAAATGTGGAACAAGAACCGTCCAGACAACCAAT
ATCGAAATTTGAGTTTGAGTTTGAAAGAAGGAAGTTGACAGAAGATGATG
TTAGAGAGCTAATTTATGGGGAGGTATGACAATTGGACTGATCTTTCTTG
TTTTACTTAATTTATTTTACAATCTACTTAAAGATTATCATCTTTCAAAG
CAACAGATCTTGGAGTATCACCCGCAGATGCTTCAGGAGTACCTGCGTGG
TGTAGATAAGACTAGCTTCATGTATCCAAGGTTTGGCATTTTGAAAGTAC
TCTTTCATTCATTCATATTCCATTTCGATGTGGAATGTGTTAGGAGCTGT
TTTCTTACTATATGCTCTTACGAAAGTAATCTTTCTGTTCTTTTGTTTAT
TGTTGGCTTTATCACGTTGCTCATATAAAAAGGGGCTCTCCTCTCCCAAC
AATAGTAAATGAATAAATAAAAAGAGATAAACCCATAATAAGCAACCCAG
GTATTCTTTTTTCTTTTAAAAACAAATTTCATGTTCGTAATTTCTCCTAC
AACCTATATAATATTTTAAACTTCAACTCCTAGTGATTGTCAAAAGGCTA
TGCAACTGTTCTCTTGGTTCAGTTGCTTCTTTGGGAGCATTATTGCAACA
GGTTAGTCTAAATTGTAAATTGGCAATACATAAAGAATGCTCCCAAGATA
CAAGTACCTCCTAACATCAGTAATCGGTGCATTGATACCACTTAGTAAAA
ATAGCAAATATCTATGTATGGATCTTCATTTTAGTTGCTCAAGGTAAGAG
CAAGCAATTAGCACCTGATACCTGTGATCGGCATCCAAATCCTTCAAAAT
GATGTGCACGAACTTTGACTTATAATCTTATATGCAAGGGTGCTTTTTGT
GTTGTAGAAACCTTTTTCCAAAGACAGTACTATACAACAGTTAATGTCAC
TTGTTCAGTTTACATTTGGTTATGACTTGAAAGTTCATGGTCTATCACCA
CATGATATTCATGTCCCTAGAGTCCTCTTATTATGAGATGGTCCTATAAG
AGGACATAGATGCAACACCAAGCGGATGGCAAAGTCCAAAAAATTAGCAAA
TTGGCATGGATGGGGTAGGATGCAATTCATACAACAAATAAAAAAGCAGG
ACATAGATGCAACACTATTCAGACAGCGAAGTCCAAAAAATTAGCAAATGG

GCACAGATAGAATAGGATGCAAGTCATACAACAAAATAAATAAGCACATA
TTTTTATGCGTATACCATATAAATTAAAGGCTTAGTTGTTGTTGTTGTTG
TGATATCATATAAATTAAATTCCACTGTCTTGATTCAAAGTTGAGTCGAC
CGTAATATAAATGATAGCAGCAAGAGGTCATACAAGATATTAGAATATAC
TATATAACACATTTATATTGGGGGAAATTAAGTTAACCAAGAAATTAATG
TCTGATACCTGTGATTGGCATCTAAATCCTTCAAGATGTTGTGCATAAAC
TTTGACTAAGAATCTTATATGCTATGTTGTTTTTTGTGTTGTAGAAAGCC
TTACTATTAATGTCACTAGTTCAGTTTGCATTTGGTTATGACTAGAAAGT
TCATGGTTTATTGCCACAGTGATATTCACATCCCTGGTGTCCTTTTATTC
TAAGATAGACTTATAAGCAGGACATGGGTGCAATACCACAGAACGGCAAA
ATCCAAATAGTAGAAAATGCACAAGGATAGAGGATGCAAGTCATACAACA
AATAAATAAGCACATATTAGGACAAAAACTTGAACCTTTTGTGCATTAAA
TTTTGATTTGACATAACTAGGCTATGGTGAGTGATCATCGATTAGTTTAT
AATGTTTGATTCCCAGTCTATGAACTTCCTTTTGACCTTGTTGTAATTTT
CTCGGTGTCATGTTACTTACTGGTTCAATATTACTCCTATGAACTTGGGA
ATAGACCAAAGACTATTTTATTTGTCATAGATTATTCATATGTGTCTTCA
GGTGTATTTCTTTCTTCTCTTGTTTGCTTGAATAAAATCATACTAACTCG
GCCTGTTCTACATCTTATCTAGTGGAGTTGATCAATTTAAACAACAATTT
GCCCGTCTCGAGGAGCATTATGGTAAAGGAGAAAAAAGCACTCCACCTCG
AAGACAATACACATCTTTACCAAGGTAAAATTCTTTGAAACAAGTGTTAA
TGGTTTTTTTGATGATGATAGATTACTTTTCTGTTGAAGTCAATAGTCAT
AATGATTGGCATCCTTCTGCTAGGGAACGGGTCCGTGCACTTGAAGATGA
TGGTGTGGATGAAAGCAGTGATCTTGAAAGGCGCGATGATGTTGCTTTTA
TGCGTATGTCCCTTCAAAGCCCCACAACATCACGGGGGAGCAAGGAAGTG
GAAATCAATGACCCAAGTGCAGTAGCTGCACAAGATGGTCTGGGTGTATC
AAAATATAGTACGCGTGCTATGTCGAGAAGTCCTACCGTTAGCGCTTCCA
CGTGCGTAGGCATGCAAAGAAGGCATTTGCAGGTAAGTCACTAGCATGCG
AATCTCGGAAGCACAGATATATCTCTTGGATTTTTTGGCCTAGTAATCAA
TGCTTTAATAACCAGATTGGACCCGCGGTGACAATTGTGGCCAGCGGGAG
ATTTTTAGTAGACCAAAATAATAAGAAAGAATAAGAAGTAAAATAAAAAT
AAAAATCAAATGTTATAGGGGCAAATAAGTATTTAATAATTATTTATAAG
GGCAAATCTTGTCTATTTAAGTTTTGTTTCTCAATTTTGTGGGGGGTAAT
TCTACATGCACATTTTTTCCTAAATGTACACCCCTATTTAAATAAAATAA
AAAAATCTAAATGATTTTAAAGTTATTATTTTATCCTCATTTAAAATAAC
TTTAGCTTTTTTTTTTTTTTACCCTTTTAGCTTTTTAAAAATTCACCAAC
AACCTTAACCCAAAAAAACTCACCAACAACCTTAACCCAAAAAAACTCAC
AAACCTTCCCCCAATTTACCGGTCACCCCCGCCCTTGTCCTTGCCGTCCT
TAGCCCCTCCTCCTCCTCCTCCTCTTCTTCTTCTTTGTCTCCCTCTCTAT
ATGTATATATACATATATAATATACATGTATACATGCTTGGATCTGAGAG
AGACATCAAATGAGAGTGAGAGAAGAATAATAGGAGCAGGAGGGGCTGAG
TAAGGCAGAGACGGGGGTTGGGGAGTTGGAAGATGAGAGAGACAGAGAAA
CAAAGAGAGAGAGAGGAGAGAAAAATCCAGTGCCGGTGGTGGTACTTGCC
GTAGAAGAAGGGGGAGGAAGAAGGAAGAGGGAGGAAAGTATAAAAAACAA
AAAGCTAAAAGGGATTTATGTATTGGTAATTTAAAAAATAGAACAGTAAT

TGAATAAAATCATTATTTTAAGGGGTGTACATTTACTTTTCATTTTACTC
AATAATATTTTGTTTTGTCTTATTGACGAATTGATATAATAGTAGTAGTA
TGCATCACCTGCCTGCCTTGTTTTGTATTATAATTTATGTTGGGCGTGTT
CAATGCACATGTCGTATCTAACTATGTTTCTGATATAAAATGCATGCGAC
AGCATATTAAACAAGTGTTTAATTTCTGTTCTAATATGCTCTATTTTTGA
TTTAATGTTTTTCCTGTTAGGGACTGTTTCCCTCTTTAATCATATGAATA
TGTTCTCTCTTCTTCTACATGCTAAGTTCTTTTGGGTGTCAGGTGATTTG
GTTTTGGTGTGTCAATGATTTTATAATTCTGTTTTCAAGTTTAAGTTGTT
CTAAATGTGACTTTCGCTCAATAGCTGCTGTGTATAAGTAGGAGCCGAAG
ATGAGCTGAAATTGGGTAGTCCAATAACTACTGCTCGATCTTCTCATCCA
TTTTCCTTATTTGCTGCAGGGCGCAATCTCGGAGGTTTTCTAA
>EUC10801-RA [gene]
ATGGCGAGTCTTTTCAGTAGTGATCATGGACTGGAATCTTCTCGTTACTT
GCTCCGATTTCTTTACCAGTTGGCTTATCAGTCGGTATTTCCGCCTTTTC
CGGTATGATTACATGGTTTTATCGTCGGAATTTTGCTTTTCGTTATCTAA
ATTGAGAGTATTTAATGGTTATGACTTCAGTAAATTCCTTCAGTGCGAGG
AACCGATCTTAAATTTTACAGATGGTGAACATGATTTTTTTTGGGTGGGG
ATTAGATCATTGATATACGGAACCACTATTGTCACTTCTATGGACAATCA
ACCAGCCATCTTCAATATATTCTAGTTTAATCCATTCATTATTCATCAGA
GAAGTAATTTGATCTTACACTTAACAATAATCTCAACACTTCCATTGCAT
GTTAGTCTGATGTGATGCACAAACTTGATTGTTTGAGTTCAATTTCTTCA
ATTTATAGTTTTATTTGTTCAATTTTACCGCCTACTGCGCTCTGTTTCTC
CTATTCTCCTGATTTCATTACCGTTTTGAGCAACGGCTACTCCAACTGTC
ATTTTGGCTAAGGTAAAAAGCATTTGTGCCTAAATGGCACAGATAATGGT
CGAGCTGTTAGAACACATTTTGGCATCAAAATTGCCGAAATTTGTGATGG
CACAGAGGATGGTCTAGCTGTTAGAGTTGCTCTTACAAACTTACGGTAAT
ACTTTTCAATATCGACCTTCTTTGTCCCTTCATTTCCTTGCTTCTACTTG
CTTTCCCCCATCTTTCTTTTCTTCTCTTTTCTTGCTTGGACAATTCCCCA
TATAATGTATAAATGAATAGCTGATTGCTGGTACTCACCTAACAATAAAG
GAGATAAAATATACTGGACAAGAAGAAAGTGAAGAGGGCCATGTTTTACT
ATTTGAGGGACATGATGAATGCACTTAAGAGACATTTACAACCCTTCTTA
GCTAGTGCCTAGTGGAGATGAAAGGTGTGTTTCTTGATCTTAGAATGGTG
GGGTTGGAAGTTGTTAGTGATAGGTCTTGTCCCTACCTCTTATCCTTTTA
TTTTTTTTGATAATGTCAGGACACTTAAACCTTAGTCTTTGTTTGGATTG
AAAGAAACGGGGAAAAGGAAAGAGAAAATTAAATGTTTATCTCTTCATTT
CTCTTGTTTGGTAATTTTAAAGGAAAAAATGAAGAAACGAAGAAAAAGTT
CATCAATTAACTTTCTCCACTAATTGGGAAAATTTGAAGAGAAATCGAAT
GAAATTATTAATAATTTCTCACATACTTTTTTTTGTGTGCGTGCACGGGC
GTTAATAGGGCATAATAGTATTTTTTTCTTGAAAAATTATAATTCCTTTG
TCATTATCTCTTAATGAGATTTACCAAACAAAGGTTTAAAAAAAAGGTCA
ATTTTTTTTTTATTCCCTTCTTTTCTTTTTTCTTTAATGAAATGATTTCT
GTTGATTGGACTTGCTTGTTATACTGAGTATCTCCAATGATTAACTGTGA
ATCCCGAATACAAATTTGAAATGAAAGCTTGTGAATTTAGGTTTTATTTT
GACTTTTGAATGAGATGATTTGTAGAAATAAAGACACAAGAATTGTCCTA

TGTTTTATATAAAATTCAGGATTAGATAACACAAGTATTTAGTATGTCCC
CTCACACGTGGGGTGGACTTTGTCAAACTATTGGGAACAGATATTGGGGG
GCCTAACATCGATGTGAGATTCAACACACCTCCTCACGTGCAGGGCGGAC
TTTGCCAAACTACTACGAACAGATATTAGAAGTCCCAACATTGAGGTGAA
ATTTAACACACCCGACATTAGACAAAAGGTCAAGCTCCGATACCATGTAG
AAAACCCAATGGACCTCACTCACTCTCAAAAAATGCCTTAGAAGAGAAGG
ATTGCCCCAACCTTTATACACGCTCCCTCATGCGCAGGGTGGACATTGCT
AAACTACTGCGAACAGATATCGGAGGGCCCAACATCGATGTGGGATTCAA
CACGCCCCCTCAGTCACATAAGGTTGAACTTTGCTAAACTACTGCGACGT
TTATTGAGGGGCCCGATATCGATGTGGGATTCAACATGATTTATTACTCG
TTCCATTGACATCTATTTGGCCCTATATTTGCTGGGTTAGAACTTGTTTG
GAAGGGCTTAACGAAAATGTGTCCTGGAGAAGCAGTTTCATGCTGCTTTG
TCCTTAAATAGACAGCATCACAGCGCGGCTCCAAACACAACCTTAATCTC
GAACAATATCCACCCAAGAAATGCTTCCCAGTTCCTATATGGCCGAACCA
GGTATATTCTATGTATATATTATACAATTTGAGTGATGCATTATCCAAAA
TTAAGATTGATGGGATTTCCCTAATGGGTTTTGAATTATTCTTCTAATCT
AAATGCTGAACATAAAATAAATTAAAATAATAATAATAATAATAATTGAA
TTGACAGGTTTTTTTTTGCTTGTGTGGCTCCTTATGATTGGGTTTGCGT
AAACTTTTGGTTATTTCCTATAATTATTCAGTGCTTATTGTGGAAGCATC
ACTTGTTTATATATGCATCTCTAGTCAAAAGAACTATGAGGAGGGTAACA
CTAATATATAGTTTAATTTGATTTATGAATTTAATATTGTGAATTGTCAA
CTAAATTCATCAAATCACTTTTGCATCTTTTTTCAGTATTTTTTCTTTAA
TGATCTGGAAAGGCCAGCTACTGCAACCATTCAACTTCAAATAGCTATTC
ATCTTATTGTTGTGGTGCTTTCTGGTATTTGATTTGTAACTCACTAGTTT
TTTTTTTTTTTTTTAATTGATTCTAAAACCCTCTGTTTTTGGATTTTGGG
AAACTTGTTAGTGAGAAAATATAAAAAATTTGTGCTTCCAAAGTTGCAGG
ATGTTGGGTTTATCCCACATTGCATGTGAAAGAGTCTGGTGATCATATTA
TAATTGTGAGAGAAATGTTCACCTCATAAGCTGGTTTTTGGAGTTGAGAG
AGACCCAAGACCACTCTACACTGATATTAGAGCTTAGATTACAACAACTC
CGGTCCAATAGCCATAAAAGAGAGACTGATCGAGGGTTAGCCGACCAAAG
GCTCAATGTGTGAGGGAAAGATTGTTAAGTTTATCTCACATTGGATGTGG
AAGTAGCTAGTGGTTAAGTTATAAGTGTGACAGAAAGACTTACATCACGA
GTTAACGTTGAGAGAGCCCAAAGACCACCCAATACAAGCTAAACACACTC
TTAATATATTCCAATACAACTTTATGGGGGCAAATGCATGTGAGGTTTGA
TGAATAATAGGTAAGCTTTTATGGGCAAAATAGCTTGTGGACTTGTAAAT
AAACAATCCCACACCTTAAAAAAAAAAAATCCGCACTTTGCAAGTAATCAC
ATTAACACACGTTGGGGTGTCCTTGTCGGTGGTTTCTTTTTTCTTGCACA
TATAGTATAGCATTGGCATGTCCATGTGCAAGCATTAATGATCCATGTAA
GCTTTATTTTTATATGTTTCTTTTTTTCCTTTTGTATGGGAAGCATCATA
GTGGAATAGGTACTGTTATTTTTTCTGTTTTTTCATTCTTGTTTTTGTTC
CCTATTTTTGGGTTCAGAACAAAATGTTTTTGTTCCATGATTTTTGGGAA
CAAAAAAAAGGAAAAGTAGGTGCAAGAACACTATTGTAGAATAGAATTCT
CTGCTTGGCTTTCAATTTGTCAGTTTCCAGAGGAATATATAATTTTTCGA
TAAGAATTTGCAGAAAAATAAGTAAAAGACCAATTTGACTAGAGTTAGGA

ATTGGCAAAGCTAAGTGTATTTTTTTTTTTCGTTTCACAAGAGCTAATTA
ATTCTGTGATCAGAAATGAAGGCTGCTTCTAATAAAGTTAAACAAATTGA
CGCCTACCACCCCTAAACTTCTTTACTTTTTCACTTTGACAAGTAAAATG
AGAAGGAGAAGAAAAAACTATTTCTTTTTGAACTTCCATATGCGCCAAAA
ATTAACCTTTTAATACAAGATTTGGAGATTTGAGTAATAAAGGATGAGGA
GAATTTCAGACCAGTCTGTCGGTTAGCTGATTTCTCTGTTTAAGGCAGCA
GATCACAGAAGCCTCTCCCTCTCCCTCTCTNCTCTCGCTCTCTCTCTCTC
TCTCTCTCTCTCTCTCTCTCTCTCACACACACACACACACACACACACCC
TCCCTTCTTTTTTACACTCCTGTGCTGATTTGAATTTCATCAGTTGATTT
CAGCTCGTGGGGTTTCAAAATTTCATCTTTTTTTGGATTTGGGTTTGCAA
TTTGGAAAGAATCAACTTGATAAAGTGAGCTTGATCCGAAAGCAGAATCC
AAAGCGAAAGAACTGGGGTTTAGTAGTTCTGCTTGACCCGTAACTTGACC
CTGAAAATGCCCCAGGAAACTGACCAGGAACCAGATGATTCCGATGTTGC
TGAATTCGTTGAGGTCGATCCTTCTGGTCGTTATGGTCGGGTCAGTTCAT
TGAAACTCTCTCTCTCTCTCTCTCTCTCTCTCTCTCTCTCTCTCTCCGTA
TACACACACACACACACACATACATATGGTATTATCATTCATGTAGTAAA
GATTCTTGTTTTGGGTTGCATAAATGCTTGGATTAATTTCTGCTGTGTCT
TTTGTTGATGTATTTCTACAGTATAAGGAGGTTCTAGGCAAGGGTGCTTT
CAAAAAAGTGTATCCTTTTTAACAATATTCCTTGGTATTTCTGTGCTTTT
TAGTATTATAGCAGTATTTTGAAAATCCTTGATGAACATTGGGAATATTA
GATACAGAGCATTCGATGAATTAGAAGGAATCGAGGTTGCTTGGAATCAA
GTTAAGGTTGTGGATCTTTTAAAGCATCCAGAAGACTTGGAGCGCCTGTA
TTCAGAAGTTCATTTGCTTAAAACCCTAAAACACAAGAACATCATCAAAT
TTTACAATTCGTGGGTCGATTCGAAAAAGGAGAGCATCAATTTCATCACC
GAGATTTTCACCTCCGGAACACTAAGACAGTATGTTGGCTTAATTTAGGT
GCAGTTTGTTTAGGTAGTGTTTGGTTGCACCGAATTGGAGCCTTGGAATT
GGAATTAGAAATAAATGCATATAAAAATCTAAATATATAGTTATATACAT
GCCTTCATTTCAAATTCTGATTCCATGGATTTAATTAGGTGTTCCCAAAC
GCTACCTTAGTGTTTTTGTTCCAAGGAAACTTGGAACATAAAAAAAAAAA
GAAGTAAATTTGTGTGGTGTTCCCACAAAACGCCACCATAGCGCTTTCTT
TGAATTTCTATCGTTTTGTGGATTCATATCAATACATTTATGTAGGTATC
GAAAGAAACATAAGCATGTCGATTTGAGGGCATTGAAGAAATGGTCCAGG
CAGATTCTAGAGGGACTCTTTTACCTTCACAGCCATGATCCACCCGTGAT
TCATCGGGATCTGAAGTGTGATAACATCTTTGTTAATGGAAACCAAGGCG
AGGTTAAAATTGGGGATTTAGGACTCGCTGCAATTCTTCGCCAGGCTCGT
TCTGCTCACAGTGTCATAGGTTAGGAATCGATTCTTCTCTCTCCTCTCTC
TTTCTCTATCTCTCCCTTGATGATTTCATTTCGTTTTGAGATTAGGTACG
CCGGAGTTCATGGCACCGGAGCTCTACGAGGAGGAATACAATGAACTTGT
TGATATCTACGCCTTTGGTATGTGTTTGCTCGAGTTGGTGACTTTCGAGT
ATCCATATGTGGAGTGTGCAAATGCTGCTCAAATCTATAAGAAAGTGACA
TCAGTAAGCGTTCTTTTTTAATCTCCTTGTTGAGAAGTTGGTACAATTAT
CTGATATCTGCAAACATTTGGGGCACGTGTGGTTTTTTTTTTTTTGTGAAA
ACTTGTTTGGTTTGGTCAAAAAACAAAATATTCATTAATAATGTGTTAAA
AATAAAAAGAAAAGGTGATCAGATTGATAGATTTTTTTTTTTGAAAATAT

TAGGAAAGATGTTATGCTGTTATTGATACGAAAATGCGTGTTTATGAGAG
AGAAGGTAGAGACATAAATGTGAGGTTCAAACAAAAAAAAAATGTGAGGA
AGAAACTATATATATGTGTGTGTGTGTATATATATGTATGTATGTATTAA
AAGAGTCTATGTTATCAATTGAGAATTGTGATTTTTTTTTGGGAGGGGG
ATTATGTTGTAAAAAGAAATGTCGTTTTGTTGAATTTCACGTCACTTGTG
TTACCTGATCCTGAGGTATGTATAAGTCTTGGGATAATCTTCCTCTTTCA
AGCCATCTTGTGAAAGTGAGTTACACCCATTGGATTTCTACATGGTATTA
GAGTCAGGCTTTTCGTCTGATGTTGGGCGTGTTGAATCCCACATCAATGT
TGGGCTCCCGATATCCGTTCGCAGTGATTTTTCAAAGTCCACCCTGCGCG
TGAGGGGGCATGTTGAATCCCATATTGATGTTGGGTCCCCCGATATCCGT
TCGCAACGGTTTGTCAAAGTCCACCCTGTGCATGAGGGGCATGTTGACTC
CCACATCGCTTGTGTTACCTAATCCTAAGCTATACATAAGTCTAGAGGTA
ACTCTCCTCTTTCAAGGCGTCTTTTGAGAGTGAGTTACCCGTTGGATTTC
TACAAGTTTTGAGTTTTTATTCGTTTTCGAAAACAAGGAAGTGGTTATCT
GAGAACAAAGCCTAACACACCCTTGACACTTGTTCTTCTATTTTTCGACA
GGGAATCAAGCCAGCTTCATTAGCTAAAGTCAAGGATCCCGCGGTTAGGG
CATTCATAGAAAAGTGTATTGTGGAAGTGTCTGAGCGGTTGCCCGCCAAG
GACCTATTGATGCATCCCTTTCTTCAGTCCAATGATGATAATGGAAGCAT
AGGTCGATCTTTGCGACCCAATCCCAATCACACAGGTGAAAAAAAAATAT
GTGTCAATCAGTAAGGCAGTGTTTGGTAGCACAGAATTCAAGCCTCAAAT
TTTGAATTCGAAACAAGCAAATGAATTCCATGAAAGCAAACGCTAACTAA
CTAAAATTCGTTGGATTTCCAGTTATATCTAGTCATTCTGATTTTTACAA
TTCAAATTTATACAGACAATCTGATTGAAACTCACAAGGATTCTTTGCTT
GATGGAAGCCGAGATTTCTCAGTCGAGAGTCAAAGGAAAGACCTCAACAC
GATATTCCTTAAACTACGAATAGCCGATTCTTCAGGTTCTTTTGCCTCTG
ATTTCATCATCGGTTTATTCGTTCTTATTCAAGTCGTAACAACAACAATA
AATAATTTATTTATTTAAAAAAAGTTAAAAGCGAAGGCACAACGCTTTTT
TTTAAGTACTTCTTACAATACAAAAAGTTAATTAATAAAAAAAAACTTTC
TTATTATATAATCATCTATTTTTGTTTTTAAAGTTTTTCATTCTGTATAT
AGAGAATTTATTTTTTGATCCATATCAACTTATTTTTTAGACTTTACTGG
TATTTTAATTAACCTTAATATTAAATACAAATTTAACAATAATTAACATT
AATTATATAAATTCAATCTAGTTGATGGTAACTAAAGAGCTAAAAACTAA
AAAGAAAAAAAAAACGTGGACAAAATATTGCGAGTGACTTATTGAAGGATA
ATATAATAGTTTTCCCTTAAATCATACTAGTACTTTAAGGGTGTTTTGGT
GGCATTTTCCTTATTTTACCCTTAATTTTAAAAAAGTCTTCTATGTAACT
TGTGTTTTAGTCCCGCACATTAAAAAAAGACGAAAGGCGCAATGTATGTG
CCTGCACCCCTCGTAGCCATACTAAATAGAAACATCATTTTGGTGTAAAC
AAATTTGTTACATGCATTTAATTTCTGCGTAAATATTTGTTAGATGTAAT
AATATTAAACGATTTCATCCAAAAATGTGATATATTCATTGTTTTCTTAG
TTTTTTATGAATAATCGTATTTACATGTAACTACTATTAACGATTTGGTT
TTAAATCTGAGAGATACAAATGGTGTAGTTTTTTCAACATAAAAGTAGTT
ACGCGTAAATAATATTACACAAATACATATAATATAGTTACATGTAATTT
GTAATTATTAACTTGTAACTAATAATATTTACTCTAATATGGAATCACTC
GACAACCTCTCTTTACGCTCCACATACAATGCGTGAGCACACGCGGGACT

AATATGGAACAAAAAACCAATTTAAGGGACTAATACTAAACTTATGTTTT
AAATAGGACAAATAACAGGTAACCCCTCTATTTAGGACTATTTAGGACTA
ATTGCGGATTACCTCTTACTTTAAAGTGAAATAGTGTGCTATCTTAATCA
AAAAGAATTTTGTAGACTGTTGAAAAAGTTTAGGTATAGAGGGCCTAGTT
ATCAATGGCTTAAAATATAAATATGAAAAATAACTTTGTTTTGCCTTTCA
TTAAATGTGCTTTTACTCTCTTAATTTCTAAAAATAACTTTTTAAAACAC
AAAAATAAGGCTCCTTTATTTTAAAACACAACAAAATTTACTTAAAACGG
TGCCTAATTTCTAAAATTCAAATTTTGGCTTATATCTTTAGGTCAAATTC
GGAACATACACTTCCCTTTCGACATTGAGGTCGATACCCTGACAGCCGTT
GCTAGCGAAATGGTTGAAGAACTCGACCTAACCGATCAAGACGTTTACGC
CGTTGCTTCAATGATCGAATTGGAAATTCAGTCATGCATTCCGAATTGGG
TTCCCAGAGAATTTTCCGGCGATGAAGTCAGCAATGCAGATGGTGGTGGT
TCTGCCGCTATCTCCGCCTTCGAATCTCAGCCCGATGCCTCTCCCCACTC
TCCCTCTTCTAATGCTTCACCCCGTTCCCCCGGTCTGGTTCTAGAACGAC
TGCCCTCCGGTCGGAAATATTGGTGCGACTCGCCCAAGTCTAGACCGGGA
CCCTCAAACTTATCACCCTCTAATTTATCACAGTCCAATTCGATGAACCC
TAGAGAAAACAATGAATCGCCAGATTCGAATGAGGAGCTTGAGCTTGAGA
TTGTGGAGAAGCTTGAGAATCTGTTGGTTGAACAGAGGAGGGAGATGGAA
GAACTCAAGATGAAGCATGAACTAGCTTTATCGGATTTCCTGAGGGAGAT
TCCTCACGAAATTCGTATAGCTGTATGCAAAATGTGCGATCCGAAGGTTT
CTGACCATGAAAAAACCCATTTCGAGACGAGATTTTCGTTGAAAACATGA
>EUC20904-RA [gene]
ATGGATGAAGAGGCTAATTCTTGGTTAAGGAGGGCGAAGTTCTCTCATAC
GGTTTGTCACCGTTTCGACGTAGCGAGATTATCCTCCGTTCCTCTATCGA
TCCAGCAAGATCGATTTTTGGATTTGGGTTCGAAATCTAGGACTTTTGGC
CTCGATTTGGGGTCAAAATCGGGCGAAAAAATACTTGAATTTCGTCGAAA
TTCAACTGTAAACAAGCAAAGGGCGGTTTCTCCTCTCCCAGAGACAAAAA
TTCCTACTACTTTTAAGGAAGCCCGATCCGATCGAAAACGATTCTCGACT
CCTCTTCCCCGGAGGAAACAACCAGATAAGGGAATTTCCAAGGGTAAATC
GATCAATTCACATTCGGATTTGAACATAAATACGAGTCCGCTCAAACATT
TTTCTTCATTGAAAATCGGTGAAAAAGGGAAGGGGGGGAATAGGGATTCG
TCGTGGGCTAAGTACTTTGATTATGGCGGAGGGGGAAGGGTCAGGGCGTT
GGATGCGGCGGACGAGCACACGGTGAATCTCTCTAAGCTTTTTCTAGGGC
TTAGATTCGCCCATGGAGCCCACAGTCAGCTATATCACGGGATATATAAC
GATGAGGCGGTGGCCGTGAAGATAATTAGGGTTCCTGATGATGACGAAAA
TAACGGATTAAGCTATCGGTTGGAGAATCAATTCAATAGGGAGGTCACAC
TCTTGTCTCGTCTCCACCACCCGAATGTGATCAAGGTAATTCATCTCTTT
CCTCTTTATATATATATATGTGTGTGTGGTATATATATCTATGTATTTAT
ATTTGCTTGTGATTGATTGATTATATCATTGATGTTTGGTTAGTGTTTCT
GTTTCTGTTCCCAAAAAGATTGAATAGAAACCTAGTTTTGTTCTCATTCA
ATGTTTTTTTTTCCGGTTATAGTTTTTGTTTTTGGGATGAAAAACGAGAA
AAAAGAGAAACAGAAACTCTAGACAAACGCGACCTCAACTTACATTAGCA
CCCCTTAATGCTTGTGATAACGTGTGATATCGGTTTTACGCTAGCCAAAC
GCTGCCTTAGCTTACATAAGCTCTCCTTAATGCTTATGATTGATCATATC

ATTGTTCCCTAATTTATGGGAGTGTAGTTGGTATTTCTGTTTCTGTTCCC
AAAAAATTTAGAAGAAAAATAGGGGGGGGAAATGGAAAGGAAAAATTATG
TTTTTAGATCTCATTTAAGAAAAAAAAAGAGCAAGTTTCTAGGAAGGGAA
ATAGAACAGAAACAAATATTTTTTTTTGTTTTTAAGTTTTTCTATTTCTG
TTCCAATTTGTTTTGCAAAGAAGACGACGAAAAAAAAAACTGAAACAGAA
ACACTGATCAAACATCAACGACATAATCAATCAATCGCAAGCAAATATAA
ATCAAAACAGAAACAGAAAAAGAAATATGAAACAAGACCGATTATTTTCA
TGTTCCCGTGTTTCTGTTTTCGTCCCAAGTTTTCTAGGAACAAAAATTGA
AACACTGTTTAGTTGTTGTTAGTGTCTCTTGTTTCTTTCTTTTTTTTCCT
GCTCTGTTCTTTGGGAATTAGGAACGTAAACAAAACTATGATAAATTTTT
TAAGATCAGAATAGAAATGTTCTTATATTTTTGTACACGTTCTCGATTTT
CTGAGAGCAGAAACACTAAACAAATGAGGCTATGTCACCCTTCAGTAACT
GATTTTTGTATAAATGCTCTTCATAGGGTTTTCTCATCCATTTGTGATTT
ATAGGAAGAACAGAACATCATATTTTATGTTCCCATATTTCTGTTTCTGT
TCCAAGTTCCCCCCCCAACAAAAAAAAAGAACACTAAACTTTCCTGTTTG
GTAATGCAGTTTGTGGCGGCGTGTCGAAAACCGCCGGTTTTCTGTATAAT
CACTGAATATCTATCCGAGGGTTCTTTGAGGGCATATCTACACAAGCTCG
AGCATAAACTTCTACCATTAGAGAAGCTCATAGCGATGGCGTTAGACATT
GCTCGGGCAATGGAATATATTCATTCGCAAGGCGTTATTCATCGTGATCT
TAAGCCCGAAAACATACTCATAAATCAAGCTTTCCGATTGAACATTGCTG
ATTTCGGAATAGCTTGTGAGGAGGCTTATTGCGATCTCTTGGCCGATGAT
CCCGGAACTTACCGGTGGATGGCGCCGGAGATGATCAAGCGGAAATCGTA
CGGCCGGAAAGTTGATGTTTACGGATTTGGGCTTATTTTATGGGAAATGG
TTGCCGGAAATATCCCATACGAGGAAATGACGCCGATTCAAGCCGCTTTT
GCCGTCGTGAATAAGGTATATTTCCGTCGATTTTTTTTTTCTCCGTATTT
TACAAATTCTCAAAATGTTTCTAAAACAAAATATTTTTTAGCATTCCTAC
TAGCATTTTAGTTGATTTTAACTATGATATTACATGATCCAAAAAAAACGG
GTTGAGTGGGCGATATGTTTTTTTGAGTTGATTACATTTTGCTTCCCGCC
CTGTAGTTCGAATGAATTGCACTTTACACCCACGAGGTATTTTCGTCATC
ATTTATCCCCTTATAGTTTAGACTTATATGCAATTGTTAAATATCTAATA
AAATTATGTAAAATGACAAATTTACCCTTATGTCTGACCCGACTCGATCT
GAAATGACTCGACCCTGGGAGAATAAAGAAANTTCGGGGGACTTATGTAA
AATGATTAATTTGCATGATTCTCCATTATTCATACAACAAAATTCATATT
TTTTTTTTTTTCAGAATTTGAGGCCGGCTATTCCGGTGGGTTGCCCGCCC
GCGATGAGCGCTTTGATCCAGCAATGTTGGGCCTTGAACCCGGAAAAAAG
GCCCGAATTCTGGCAGGTAGTGAAGGTTCTAGAACAATTCGAATCCTCCG
TGGCCCGCGATGGAACCCTTAATCTTGTGCCGAATTCAACATGTCAAGAT
CACAAGAAAGGGCTTCTTCATTGGATTCATAAGCTCGGGCCCGTTCATCC
CGATGCTTCTGGGCCCGTTCCCAAGCCTAAATTCGCCTGA
>EUC24332-RA [gene]
ATGAAGAAAGGAAACCTAGCTCCTAATCTCAAGCTTTCGCTGCCTCCTCC
CGATGAAGTCTCTAAGTTCCTGTAATTCCGCCTCTGACACTCTATGAATT
GCTTTAATTATTGGCATATACTGTTCCTCGGTGTCTTTTTCTTAATGTGT
GTGCTTTGATTGTTTGTAGGACTAAATCGGGAACGTTTATGCATGGTGAT

CTGCTGGTGAACAGAGATGGTGTTCGAATTGCGTCTCAGGCGGAAGTGGA
AATTGTAAGATATTTAAGAATTTTCAATCTTAAGAAGCAATAAGTTGTGT
TGAACTGAATTGATCGGCATTTTCTTGCATTTGATCCCAAGATTGATCAA
AGGCAGTTGGGCACAATCAAAATTTCTGCAGTTTTCCTTTAATCGAAGTG
TTTGTGCTAGATATTTTGGATCTGTAATGTCCTAACTAATTTATGTTTAA
ATTCGTCTTATGTGAAACCTGAATTTCATCCATTCCGTTGTTTTTCATTG
CGATTTGGTGATTTTTTGGTAGATATTGCATGTTATATGAGAGGTTTAGC
TGCCTAAAGAGCTACACGGACTATTTGATAAAAGAAAAGAACTGGCAGGG
GAAATGCAAGGCCCTGCATCTGCTGATTATTTCTTATTTCTTCAGCAAAT
ATGGTTGTTTGCTAATTATGATATATTTAGGTGTGACAATACTAAGACGT
TGAAAGGGCTTCAGTAGCAATCTGTTTGTGAAGGCGGCCTTGTAATATTA
TGAAAATACGCCCATTGGAAACATTTCTTATTTATGCCTTCTCTTTTCAT
TTTTTATTTTATTTTATTTCCTTATTTTAGAAGGATTGTGATATTTCAAT
TAGGTTACAGTATTAAGTTAGATTACTTTTGGAATGTTTTTCTTAGGCAC
TTTGGTAACATTTTTTTAAGTACCTTTTAAAATAATTAAAAAAGAAGAAG
ATAATTTGACAAAAAAAAAAAAAAAAAAACAAATACTTTTGCTTAAAGGCA
AAAGTCAAAAGTCAAAACGTGTTTGATAATATCTTAGTATTATCCCTATA
AATGATCGCTTAATCCCAAAAAAAGAAAAAATCACAGATGATTGAAAAAA
GTGGGTGAGGAGGTCCTATTCCATCGATTTACAATAAATAAATTGCTATT
ATTTGCTTTTAAAAAAAATACTTAAAATTATATTTTTAGTTTTTTTAAAG
TATTTTTTTGTAATTGCAAAACAAACACGACTTTTAAAATTAAGTATTTT
TTCGCTTAAAAGCGAAAAAAATACTTTAAACGTTATTAAACAGTGCCTTA
CTTTTCTTAGGATTTATGCTAATGCCTTCCTTGTTAGATGATATTAAATT
TTTATTCTTGACATTATGTATATTCAAATTTGTGCTAAGTTGTTCCTAAT
ATGTTTTCACACCTGGATATGCTGATGGGAAAAATATGTTAGTTGTATAA
AAGGTTGTTGGTGTTGGGAGGTTCCAATCAAAAGGATATAGGGAAAGAGG
AAAAGTATTTTCATATGTCAGAATAGGCACTATTCTTCTAACCATATAGT
CACAATAGTCGGCTAAAATTGGAAGAATATTACCGGAAATAAATGGGCCA
TTAAAGCCCTACAATAACATTATCGGAAATAAATGGGCCATTAAAGTCTT
ACAATAACAATGCCAAGAATACAATGATTTAAGTAGTAACTAAATTAATG
CCTATTCCAACACTCCTCCTCCAGCTAAGGCGAAGATATTGTTGAGCCCT
AACTTGCCAATGGCCGACTGAAGAAGAGATAATTCTGTAGACTTCATGAA
CATATCAATCCCCTGTTTGGAAGATGGGACGAAGTTGTGATAATTCTCGA
TCGAACTTTCTCACGAATAAAGTGGATACCCATCTCGATGTGTTTCGTTT
TTTCATGAAGTACTGAATCAGATGATAACATTATGGCGAACTTGTTATTG
CAAAACAACGTAGATGAGTTTTTCATGGGAAGTCTCAACTCTGTTAGAAG
AGAACGGAGCCAAAGAGGTTCATATGCTCCCTCAGCCATGACGCGATACT
CAATTTCAACAGAAGATCGAGAGACAACAGATTGCTTCTTATTCTTCCAC
GAGATTAGGTAACTTCCATGAAAGCTGCACGGACCAGAAGTCGAACGCCT
GTCATCCTATGACCTTGTATAGTCAGCATTTGTAAAGCTGGAGTAACCCT
CCTATGCCCCTGTCACATAAAAAAGACCAAGACCAGGACATGTCTTAAGG
TACCGAAGGATATAGTAGGCTGCATTTAGGTGTGGTGTGCATGGTGAGTG
CATAAACTGACTTACAACACTAACAACATACTTAAGGTTTGGCCTTGTAT
TTGTTTGATAGATGAGACGACCCACAAGCCGCTGATATACTGACGGCATA

GTAATAATTCTCCTGAGTCTGCTGTCAACTTAAGATATGGATCCATAGGA
GTAGAAGCAGGTCTACATCCAAGTGTGCCAGTATCTTGAAGAAGGTCTAC
ATCCAAGCATGCCAGTATCTTGAAGAAGGTCATGGCGTATTTTCGCTTGG
ATAGGGAGATACCAAGTCGAGACTGAGCAACCTGGGACTAATTAAGTCCA
TAGAGGGATTTTCGAAGACGAATACTCCCCCCGAGTCCATTATCCCGATG
CAGGCTCCATGTAGATAGTCTTAGTCAGATTGCCATTGAGCAATGCTGAC
ATCGAGCTCATGCAGAAGCCAGGAATACGAAGCAACCAGCGAAACAAGGA
CACAAACAGTGGTCATCTTCGCAACAGGCGCAAAGGTGGCACTGAAGTCT
TTGTCCAGCATTTGTGTGAATCCCTTGGTGACAAGGCGTGCTTTGTATCG
GTCAATGGAGCTATCTACTAGATGTTTGACACTGAACATCCACCTACATG
CAACCGTCGTTCGCCAGGAAGCAAGGTAACCAATTCCAACGTCTGATTCT
GCTGTAGAGCATCCATTTTCGCTTTCATAGCGGCTATCCACTTTGGATTT
TGGAGGGCCTCATGGACTGATCAAGGAATAAGGACTGAATCAATCTGTCC
AAGTAAAGATTTGTATTGCTTCGACAGACCCTGAAAAGACTTGTACTTGG
AAATCGGATGATTAGTACAGCAAGAAAGAAGGTAGTGGGAGAGTGGACGT
TGTTCATGAGTAGGGTATTGTGGGGGAGAAAGGTTAAAAACTACAAGAGA
TAGAGATATACCTAATTCCAAAGAAGATGTTGGCAAAGGATTGGGATCAT
GTGGGCGACGAGAGTAAGCCTGTAACGGTGCGGATGGGGAGTTAGGTGGT
AAGTGTGGTGTGGGTGGTGGAAAGACATCAAGGATAGGAATAGGGCGCAT
AAGTAAGATATCATCCTCTGTAGAGTTTGCCACGATAGGTGGGGTAGAGA
TATTGCATGAAAAAAAAGAAGAACATTTTCGAGAAACGTATTATCTAAAG
AGTGATAACTATGACAAGTTATAGGACTGTAGCACCTATATCCTTTGGAC
ATGGAGGAATATTCTAAAAAGAGACACCAGATAGCTTTATGATCTTACTA
GGTTCGAGTATGACTCCGATTCTGGACAAAGCTCCTGCACCCAAAGACAG
GTAAAATAGGGAAAAGGGTGTTGTCTAGCTGAGGTATATGGAGAGGGTCC
TTGCATGTAAGGACATGACTAGGGATGCTATTAATGAGATAAGCGGCGGT
AAGAACAATTGTGTGCCAAAGAGACTTGGGAACATTCATACCCCATAAGA
GACATCGAACCACAGACATGATGTGACGGTTCTTATGTTTCACCACACCA
TTCTGTTCGGGGTATAACGACAGGAAAGTTGCTAAAGAATACCTTGCTTA
TTAAGCTTAGTCTGAAATTTGTCAGATAGGTATTCCTTTCTGTTATCTGA
CCTCATATTGCACAAGACATTTTGTATTGGGTTTCTATCATTTAGAGAAA
GTGAATAAAATGAGTAAAACTTTTGATTTTTCTTCATTAGAAAAACCTTA
GTGCATAGACTCTATCTATGAATGTGACATAATAGTGATGTTTATTTAAT
GCTGTAATAGGCGAGGGTCCCCAAATATCAGACTGAATGAGATTGAAGGC
ATTAGGTGCATGCGAGAGATGTCTGAAGTGTGAGTATGTTTAGATAATTC
ACAAATCATACATTGAAATTTAAAATCTTTGCAAGTTTTATTAACAGAAG
GGAATAACAAACATAAATGACAGGAATGCCACAAGTCCAAAATTTTAAAC
TAATAGATGAGGGGTCAAAAACAGGTAAGACAAATGCTTGCATATTAGAA
GGAGATACTTCAAGAGGTGGAGGGTTCCCGAAGTATAAGAGTCCATCCTG
CTCATATCCCTGCCAAAAATCTTCTTTGAAGTCAGGTCCTGCACGAAGTA
ATGGTCGGATAAGAAAACGACGACATAGTTAAGGTTCTTAGTAAGACGAC
TAATAGATAGAATATTATAGACAAATTTAGGGACATAGTAAACAAAGTGA
AGGGTGATATCTGGGAACAAACAGGCATCTTCCTAGCAACGAATGAGGAT
AGAGGTGCCATCAACAATAAAAACAGATTGATCGACAGATAAAACAGGGG

AGTTAATGGTAGATAGTTCACCAGTCATGTGATTATTGGCCCCGGAATTG
AGAATCCAAGTAGGGTGACCAGACTTATCATGAAGCGCAGTAGGGGTACC
CGATGCAGGAGAAGCCGTGGGGCCGGTAGACAATGCCGAGTTGTTTAATT
GGGCCTGAAAGTTTGGCAATTTGGGCCTGAACTTGGGTAACATCTGGTTG
ACTTGGTGGTGGATCTAATGGGACATCAGTTGCAACACTAATACGTGAAG
TTCTTTTCCTTTCCTACTTCTTTGGTTCCGAGGTTTCAACTCAAGATGGA
GGTCATAGCAGTGCCAGTGATCAATGTGGTAACTAGGCTTGCGGCAATGC
TGACAGACTGGACATCATCCTGTTGAGCGTGGATTGGAGGGGGCTGCAAA
GGCCATCTGCTCGGGGACTGAAGGAGTGACTTCAGGTGGAAGGAGAAGTC
GTTGTCTCGCATCTCCATCAACAATAGTGAATGCTGCAATTAGGTCAGGT
ATCGAAGTTGTTTTTTAAAATCTGAGTGAGAAGATTCTAATACTCAAGTT
TTAAACCCATCTTGAAGTGGTATGTGTGACGGCGATCCTGGAGCTTGGAC
TCAATCTCAACAACATTTTCGGGAAATTCGGTCAGAGGTTCATATTGTGC
CAGTTCCTCCCATTGAGTCAAAAGGTACCCGAAGTAGTCTGCAACCGACA
ACCCCAGGGATGCTTGGATGCATGACAGATCTCACAATGGAGCTTGAAGA
TCTTCGACTCTTTGTTAGAGTAGGACCATAGGCCTTTTACCGTCTCATGA
TACGTGAATATATTGTAGATCCGTTCCTCCATAGAATTGAATATCCATCC
TTGGATGATATGGTTGTCCGCATTCCACTGGGTGAAGTTGGAATCGATGA
TGTCTGGCCTTGGCTCAGTGCCCAAGAGCCATCCGGTCTTGTGTTTTGCA
CCATGGTAGAGACGTTAAGGCTAGCCCCTCTCATGAGTGAGTCAACTTTG
TTCACTTAGCATATGTTCTTTCAGAACCCCGTTCAAGCAAAGGTGTGGAA
TACTGCTAGAGGCGAACGAAGGCTTTCTTTTAGTGTGGAGAAGGTTAGGT
TGTTGGAAGGCTTAAGCGAAGGTTGCTATTAGAACGATTTTTTCTAAGAC
TAGGGTTATGCCCTTTGGTAAGCATTCCTTTAGCAAGTAATTACTTGGGT
ACGAAACTACGCTACCCTATTTAAAGCATCGAGAAAGAAAAATAGAATGT
TAAAACTCGTGCAATGTTATAAAATGGACTAGAATCCTAGTTGAGGGACT
TCGGAATGACTGGGTGTAAAGGGCACGTAGACAGTGAATATGGTTGAAAG
TGAAAGTAGCCAAAAAGTGGTGGAATGCTCTTGAAACCTTGAGTGTACAG
TTGGGGGCCGGGAGGCTAGCCTACCAAAGAAAGAGCTGCTTTGTAGACTT
TTCGCTTTTGCTTCGTTATGAGATAGCAGGTAGCTAACAAGTGGATCCAG
GCCATATGTACTGAAATGAAGTGGTGTGGGGGCACGCTTTTTGGGATAAA
AGGGAACAAGATCGTGCATGGAAATTCATCCCATTTAGTAACACAATTGT
TACACGTTGGACCGGGGCATTAGTATCACGAGTCGGATCTGGGCGGCCTG
TTGAAGGCAGGGCAAGAATGGTCTCTGATTTAACAACTTTTTAGGAAGCC
ATGTGCTCAGAATATACAAGATTTGGCAGCAAACAATTTGACGGAGACGA
GCAAACTAATTGTGTTCTGGAAAAAAAGGAGGACAATGTAATGATGGTAA
GCAAATACACGGTTACCTAACCATACATTAAATAAAAAAAACTACCTTTT
GTTTAGTACATTCAGAACACCAATGCGTGCTAAACAAGAGCGACAGGCAG
CAGCCACGGGACGACGGTGCTGGAACGGAAGCAGCTACCGCACGACAGTG
CTGAAATGACGGACAGGCCGCTAGGGCCAGCAGTGGACAGAACAGCCGCA
ACATGCGGTAGCAGACACGACAGCTGCAGGGGGCAGTGGTGAACAGTAGC
AGTTAGGGTTGCTTGTGGTGGTGGGCAGCCAGCAGTAGGTCTAGGGTTGG
AAAAAATATGTAGCTCTAGATACTAAAAAGGATATAGGGAAAGACGAAAA
GTATTTTTGTACGTAAGAATAGGCACTATTCTCCTAATTATATAGCCACA

GTAATTGGCTAAAATAGGAAGGATATTACGGGAAATAAATGGGCCAGTAA
ATCTCTACAATAACAATACTGAAAATGCAGTGATTTAAATAGTAACTAAA
TTAATTCGTATTCCAACACAATCTAAAATCACATGACATTGAGTGGAGTA
GTTCCTTTCATATATAAATTATTTGGGAAGCCTCACAAATTCGATAAAT
TCGATATAGGATTCTAACATCGACTCTCATGTAATACTTAGTCTTGCACT
TGATGTGATCAGACATACATCGTAATAGAGAAGATGGAGATGAGAAGACA
AAAGAGAGGAGACAAACAATTTGGGACAAACAAACAAGACAAGACTTTTC
AAACCCGCTTTGGCTATCATGTTAGGAGGTTTCAACCTAAAACTATATGG
TATAACGTGGAAGTAGTTCTTTTTATATATAGACTATTTTGGAAAGTCTC
AAAATTCAATGTGAGATTCTAATAGTTACTTGGGATAACTAATGAAAAAC
TCTATGTGGGATGGAGCAAGCTTCCTTGTACTTGTATAGAGGTTTGCCAC
TTTGCCTTTCCCTTTCTTCCCGTCTTTTGGCCTTTTGGGCACACCTGTTG
GTTACAATAATTTGGATCACATGCTGAAGGATAATGCGCGCGCACATAGA
CACACACACACACATATATATTTGCAAATGTAAGAATTTTTGCTTGTCTA
GTGTAGCATGATATGGGTACAATATATGTATGCTATCACTGATCTCTTTA
ATTGTTTCATTTTTTTTTGCAATTTCTTTCCCAGCCATCCTTAATACACC
CATCAGACAATCAGTTGAGCTTAGCTGACTTTGATGCTGTTAAAGTCATT
GGAAAGGGAAATGGTGGAATTGTGCGATTGGTGCAACATAAATGGACGGA
GCAGTTTTTTGCTTTGAAGGTACCATTAAACTCAACCCAGTTCTCTGATA
TCCCAAATTAATTCATGAAAAGGATTAATAAAAAATTATACTTTTCATTT
CATGTACTTTTTATTCAATACCCTATCCTGGTTTACTTATATTAAGAAAA
ACCTATCTGGGTCCCCTAGTCCTACATGCCCGTGATGTGAAGTACTGCGG
AGAGTATTAATTTTGGTTGGCAAGATAGACACAGAAATTATGGTTTTGTA
GTGTTACTAGTTTTGCATGCTTGAGAGAGGCTCTCTGTTAGTCCTGCCTT
TGGAGTTAAGTTGTTTGAAGAGCATAAGACAAATAATAGTAAACCCATTT
CTTATGCACAAATAATAGTTTGATTTCATTATCAGAATTGGATATGCTCG
TAAGACGGTTTGACAAATTCTCCTTTGGCAACAACATACTCTTTCCTTCA
CGGATTTGCTTATTCTGATGTCTCCATCTTACTTCTTTTCACTAGGAAGG
ATTCATTGTGATATAATCTTTGGCATTGCAGGTTATTCAAATGAATATTG
AGGAGTCTGCTCGCAAGCAGATTGCGCAAGAACTAAAAATTAATCAATCC
TCACAATGTCCAAATGTTGTTGTCTGTTACCAGTCTTTCTATGATAACGG
TGCCATTTCTATAATATTGGAGTACATGGATGGGGGATCTCTTGCAGATT
TCTTGAAAAAGGTCAAAACAATACCTGAACCTTATCTTGCAGCCATCTCC
AAGCAGGTGAGAAATGTAGACAAGGGATTATTTATTTCCAATGACTATT
GCTTATTTATGTTTTCCACCTTTTTTCAGGTGCTCAAGGGCTTGTGGTAT
CTCCACCATGAGAAACATATTATTCACAGGGACTTAAAACCTTCTAATTT
GTTAATAAACCATAGAGGTGAAGTCAAAATCACCGATTTCGGTGTTAGTA
CAATCATGGAAAACACCTCTGGACAGGCCAATTCTTTTGTTGGCACATAC
AACTATATGTCTGTGAGTTATTTATCATTTGCTGCAAGTTTGTCTAGCAA
GTTAAGGCACATATATTGTTCTGTTCGTTGTTTCCTCTCACATATATTGT
TCTGTTCGTTGTTTCCTCTCACCAAATTCTTACATTTGATTCTTCATTGC
AGCCTGAGAGAATTGTTGGCACTAAATATGGCTACAGAAGTGACATATGG
AGCCTGGGGTTAGTTTTGCTCCAGTGCGCAACGGGACATTTCCCATATTC
CCCGCCAGAAGGGGAGGAGGGATGGGTTAATGTCTATGAACTTATGGAAA

CAATAGTTGGCCAACCACAGCCTTGTGCGCCCTCCAATAATTTTTCTCCT
GAGTTCTGCTCTTTTATTAATGCATGGTAAAGCTGTCACATTTCGTGCTT
ATGAAACTTGGGTGACAATAAGTTAGGCACCGTTTGGTGATACATCTTAA
CCACTTCTTTAATTAAAAAAATAAATAAAAATAAATCATTTTTACACAAA
AAATAAATAAAATAAAAGTACATCCATTTAAAATTACAAATGAAAAAGAA
ATATGTATAAGAAGTAAAAGACAACTACTTCTAAAAATTTTGCATTTTTA
CGTAGAAGCTAAAATTGCCTAAGATAACCTTCACCAAAGATTGCCTTGGG
TTCCCATAAATCCAAATCCCTACACCCCCCAAATCTATTATTAATATTTT
CTCAGCTGTTTCTAGAATCAACAACAGTCAGCTTTTCTTCTTCAATGCCA
ACTTGTGATGATGCTATATTGGTATCCCTCCACTTGCCTATCCCAATTTC
CCTTTATGCTACCTTCTTTGGGAGTTCCATGTTTCAAGCTAAATAATCTA
CCAAGCAGTCTAGCTTTTATGCTTTCCTACCATTTTCTCAAGCTAAATAA
TCTACCAAGCAGTCTGGCTTTTATGCTTTCCTACCATTTTCTCATCTAAA
AACAATGTTACAAATTATCAGCTTGTTTCTTAACCGGCACAATCCAGGTG
ATTTCGTATTTCTCATTTATCATGTTGAATTCATTCTTTTGGACTCCACA
CCATAGTAAATACTCTGATGAAAGAAACAGTATAATGAATACCTTCAATT
TTCATTTTCATTTTATTATCTTATAGAAAATGTCATCTCTATTTAGTGTG
CAGAAAGACCCAAAGGCCAGACAGTCTGCAAATGAGCTATTGGTATGAAT
ACAATATTTTTCTTCTATTTTTCAAAGATTATGATGTGCCAAGGTATAAT
TCGACTCCATTGCCTTTTGTCTTTATGAAATCCAGGCGCATCCTTTCATT
AGCATGTTTGACGATATGGACATTGATCTAGCATCTTACTTCACTAATGT
GGGATCTCCACTTGCAACTCTATAA
>EUC01391-RA [gene]
ATGGCTGACGTCAACGCAGCGGCGGGACAGTACCCCGAATTTCCGGCGGT
TCCGGCACACGGAGGTCAGTACGTTCAGTACAACATTTTCGGAAACCTGT
TCGAGATCACCAACAAATACCGCCCTCCGATCATGCCCATCGGCCGCGGT
GCTTATGGAATCGTCTGGTAAATAATTAATTTTTTCTTATTTGTTGTCTT
TGGATCCCTTTTAGCAGCTCATTTAGCGATTTTTCCTCGATTTTCATTGG
TTTGACTTTTATATCGATGCGTATAGATTTACTGATTTTATTTCTTTTTT
GGAATTTTGAAGCTCGGTTTTGAATTCGGAGACGAATGAGATGGTCGCGA
TTAAGAAGATAGCCAATGCTTTTGATAATTATATGGACGCCAAGCGAACG
CTTCGCGAGATCAAACTTCTTCGACATTTGGATCACGAAAATGTTACTAT
TTCTTCTTTTAAAGTTTTTGCTCTCTTGTTCATTACATTGAAAATGATTC
TTGTATCTTATCTGAATCCATACGCATATGTGCTCATAATCTGTTCCTAT
TTTCGTTTTCTCCCTGTGTAATCTTTGTGCTAAGTGCCAACTCCAGTTCA
TATGTCAAATAACTGCTAATGCTGGAATTTAATATGACTTTCAAAATAAA
TCCTTAAATATCGAGCTTGTGCCATGCCAACTTTTGAGGAAGACATATTT
GTTTTTCCATTTTAGCATGTATAACATTCTTCATATCTTGCCAATACAAG
GAAAAAACAATTTTTGAATAGATTGAATGTCAAAATACATGGCGTTGACA
TGGAAGAAGTAGCTTTTAAACCCTTATAATTTCCACTGCTTGATGATTTT
TACTTGATTGTGGATGCTTTTCCAAGGTTCGGTTCTTTTTGTATTTGTTT
TTACATTTTTCTATTTTAAGAAATCGAAATTTGAGCCTCTGATTCATTAG
CCATACTAATCATCAAATTGTGTCATAACTAAAGAAGCTTTTGGGTTATT
GAAACATGAACTTGCTCTTTGTTTCTGAGGAATCCTTCAAATGTTGTTCA

CTTAATCTTATCATATGCCAGTTATATGTTATTCTGTTTGCAGCATATGT
CGACCTTGATCTAGTGACCCTATCATGTTATTTTTTTCTTGAATGACCTC
TTTTGTGTTTAAGAGCTAAAATTACTTTTGAATAAATTCTGAGCAAACCT
ATTGTTTTACTGGATGCCATGTCTCTTTCCAGCCAGCGTCATTAATTAGG
CAAATGAAATGATACAGGTTATAGCTTTACGAGATGTGATTCCCCCACCT
CTACGGCGAGAATTTTCAGATGTCTACATTGCCACTGAACTCATGGACAC
TGATCTCCATCAAATCATTCGGTCTAATCAGGGTCTATCGGAGGAGCACT
GCCAGGTAACCTTTTGATCTCAAGTTCTGACTCTTGGTGGCATTTGTTTA
GGTAAAAAGCATATATATATATATATATAGTAATCAAACGCCACCAATAT
AACCCATAAACATTTCAAATTTACAAGCTGAAGCTGATTCTCTTTATTCT
GCTATTTGCGTACAGTACTTCTTGTATCAGCTCCTTCGAGGATTAAAATA
CATCCATTCAGCAAACGTTATTCATCGTGATTTGAAGCCCAGCAACCTTT
TGCTCAACGCAAATTGTGATCTTAAAATCTGTGATTTTGGTCTTGCCCGG
CCAAACACAGAGAACGAAGGCATGACTGAGTACGTAGTAACCAGATGGTA
TAGAGCACCTGAGCTACTGCTCAACTCTTCAGATTACACAGCTGCAATTG
ATGTTTGGTCGGTTGGATGCATCTTCATGGAGCTCATGAACAGAAAACCT
CTGTTTCCTGGTAAAGACCACGTTCATCAGATGCGCCTGCTGACTGAGGT
AGCCATTAACATTACCAAGTGCATTGTCTAAGAGCTCGTTTGGTTGTTTA
AATTGTCTTATTTTTATGAATATTTTTATTTTTAATGATTTGATTTTTGT
ATTTCTCTATGTATGATTTAATCAAATTTACTTGTTTGTTTGAACAAAGA
AACATACAATTGTGTTGGTTAAGTTAAAAGTTTAAGATAGTTATTTAATT
TAATTGTTTTTCATTCTTACAACCAAAACTAGACTATGACAATTCTTCAA
TGACTAATAAAGTAGTACAATTTGCGAATTGAAGGACAGCCAAACCATCC
TAATTCTTGTTTGGTCTAAATATGATTCTTATGTTTCCTATCAAGTAAGA
AAATAATACTAGTTTACAAGGCAGCACGTTAAATGATATGAATTGGACTT
CATATATATCTAGGTTTCGTTATCAAAAATATGACGGCGTTTGTCTTTCA
GTTTCTGTTTCTGTTTGTTCCCAAAAAACTGGGAATGGAAACAGTAAACA
AATGCCACGAAGTTGTCCTGAAAGAGCAGGCTCTTCTAGATGTTTAGAAA
GTTCTGTAAGGACCCTATATTTTCAAACTTTTATTCCTTTTTCATTTTTA
GGTCGCAATATTAGGGGGAAATTATGTGAATTTTTGTGTACTTTTTTTGT
CCTTCAAATTTTGATTTTAACCTAACCAAAAGCCTATTTTATTGATTGCA
TTTCAACGATCTATATCTTTTTTCACTCTCTGTTGCGTCAGATTCAGTTG
ATCAAATTTTGAAATTGGCCCAATTGTCACATAGTTAGTAGTGAAGGAAG
TAATCTAATATTTTCTGTTTATGTTCCCAAAAACCTGTGAATAAAAACAG
AAATATTGGAACAATTTTTCCATCTCATCTATTTGAATGCTGACGCGAAG
ACATTATTTTTTTTTAATTTCAGCTTCTTGGAACACCCACCGACGCTGAT
ATCTGGTTCATGCAAAATGAGGAGGCAAGAAGGTATATCAGACAGCTACC
ACGTCATCCACGGCAGGAGTTGGCAAGAGTCTTCCCACACGTTCATCCTC
TGGCAATGGATCTTGTTGATAAAATGTTGACATTTGATCCCACTAGAAGA
ATTACAGGTAATTCTGTTATCCCATTAAGTCTAATTTGATTATTTTTTCT
TGCAAATATGATATGAATTCTATATAGAAAATGTTATTTTTAGAACAGAA
TAGAAGTGTTTCCAGATGTTTGTTTCTGTTCTAAAATTTTCAGGAAAAAA
GCAGAAGTCTAAGGACAGAATTTTTTTTGGAAAAAAAGCAGTTTCTGAAAA
TTTTTGGAAGTGTTTTTGAAAATTAAGAAATATTTCTTGGTCCCCTTGGT

TTTATTTCATTCAAAATCATAATTTTATTTGTGGATGCAGTTGAAGAAGC
ATTGGCTCACCCTTATCTAGCGAGATTACACGACATAGCCGATGAACCAG
TCTGCTCTAAGCCATTCTCTTTTGAATTCGAGAAACAAGGCTTGGAAGAA
GAACAAATAAAGGATATGATTTACCAGGAGTCCATAGCCCTCAATCCAGA
GTATGCATAA
>EUC09325-RA [gene]
ATGCATTGGTGGCAGAGCGCCTTCTCCTCACCCTCACCCTCACCCTCTCC
GTCCTCCTCTCCTCCCTCCAAATCCGACAATAACGTCGTCAACAGGTTCA
ACATCTTCTCTACCCGCGGCCGTCGCTTGAGGTCTCATCATCGCCGTCTC
ACCCGGGCCAAGAAACTCCGGCACCTCTCAGAGAATGAAGCTGAACGTCG
CCTTCCCCCTATTGACCCTTCCGAGCTTTGGAGATTGCCTAGTACTCTCG
AGCATTCTGTTCGATCATCATCCCAAACGACCGCAGCTGCTCCGCAGCCC
CTCCCTTTGCCAGAATTGGGTCAGTTGCTCCGTCGCGACGCTAACTTGGT
GTCATGTTCGAACCCTGGCGATCGTCATCTGCGATCGCCTAAAGACGTTA
CAGGAGGTGGTGGAGACGAGAGAGAAAAATGCAATGTAGTGAACGGCGAT
GGAGTTCCGTCTGGGAGGTAATTTCCAAAACTCGTCTGATTTGCGTTCAC
CTTTCGCGTGAACCTTATCAAAATTAACCGATTATCATGCACAGCATAAA
TAAATCAATGGGTGGAATGCCTTGACGAATTCTCATTGATCAGCGCTTGC
AATTTGCTTCTCCAATTCATTTAACTTTCCTGATTTCATTTTGAATCCGC
AATGTTCTTCATCGCCCTCATTTTTTCTCTCACCATAACCCAAATCATAA
AATGCTGAACGTGGTGGTCCTTGATTCCTGCATGAATGATTGGTGGGGTT
ACATAAATAACCATGAAGTCTGCCAATAAATGACTTAAGCAACTTATTTA
GTTCCTTTCAGAAATTCAAATATTCGGCAACAACTATATCTGGTGTTCAT
TTGTGTATGCGTTTATGTTTATCATTTCTTAGATTATTATTTAGTTGTTG
TTGCTTCTATTCTGAAAAATATTCTCGCTTTCTTCACCCAAACAATCTTC
CTATTTGCAGTCGAATAGCTAGCCAAGATGCTCAGAGCAGTACAGAGCAA
CCAAAATCTCGACACCGGAGAAAGTCCCCTCAGCCGGTGAATGGTGGAGC
AAGAAATAAAGGCAACTACAGGATCAGCATTCCAACCAGTGCTCCGACCA
GCCCATTTTCAAGCCCTAGCCCACAAAGAAATACCCCAGATTTTTACACA
TCATATTACATGACGCCCCCAATTTTTCAAGTTTGGTCTGCACCCGAGTT
GCCCCCTTCAGACATGACCGTAGGCCAAGGATTTCCTTATCTAATGTCTC
CTGAGAAAACTGCATTTAGTGTAGATAGCTCACCCCTTCATAGTCCAAGG
TTAAGTCCTCACCCAACCACCAGGAGTCCTTGTGGACCTCCATCACCCCC
TACAGCTAGACGTGAAAGTAATAATCAGGTTAACATACACCCCTTACCCC
GTCCTCCGGTAGCTACAATACCTTCACCTCCAGCTCTTGTTCCCCAAACT
ACAACTAAAAAGGGGATTAGGCCATTGAAACGTCGATGGAAAAAAGGAAA
GCTTATTGGACGTGGGACATTTGGAAGTGTTTATATTGCCTCCAATCGGT
ACTTCAAGTTTTCCAACTTTTCGATGAATTAATTTCTTTAATTTTACATT
TACCTGTAATTTAATCAAATTTACATATAGCTTGTTTGAACAAAGAAACA
TACAATTATTATGATTTAATAAAAAAATTAATCTATATTTTACTCCAGTT
ACACAAGAAAATAACGTATAGGCTGGAATCAAAATATTTAACGCTAATTT
AAGTGTTTTTAATATTTACAACCAAACCCTAAACTATAATAATTCATAAA
TCAACTACTTTTAAAATCTTGCAAATTGGAGGACAACCAAACATGCTCTT
AGTCAATTTGATTGTTTCTAGTTGCACTTAGATATTATATTCTTTGTCCA

ACAGGGAAACTGGAGCTTTGTGTGCAATGAAACAAGTAGAGATATTACCA
GATGACCCCAAGTCTGCCGAGTGTATAAAGCAATTAGAGCAGGTTGTATT
CCAATAATCTCTCTCCTTTTTTCTCCTGTTGTTCATATTTCTATCTCGAT
TAATCTATTTTAAACTCCAACACAGTACCCTTCTCTCGATTCTCATGTGT
AAAATGCTGAACAAGCTCATTACAATTTTAGACAATGTCCTAAAGATGTG
CTTGTATTTTTCCCCCCTTGCTTTTGCAGGAAATTAAAGTTCTCAGCAAG
CTAAAGCATCCAAACATAGTCCAATATTATGGTAGTGAAACGGTGAGTAA
TGGCTATGGTGCATATTTGAATTTGTCTGCTTAGGTTCCTTAATTTTATG
AGATATTAACGTATGCATGTGTATAGGTTCGTAACCGGTTTTACATATAC
CTAGAGTATGTTCATCCCGGTTCAATTACTAAGTATATACATGACCATTT
TGGAGCAATTACTGAACCTGTTGTTCGCAATTTCACTCGACATATTCTTT
CGGGGCTGGCTTACTTGCATAGCATGAAGACAATTCACAGGTAGCTATTG
CATTAAGTTTTATACATTTTATCAAATTGAATGTTAAGTTTACGTGGTAG
AAACTAGTTTTGAGTTATATTATTGTATTGAAGCAGATAAAATCTCTTCA
TGCATGACTGCTGCTGTTATTTTTTAAACAGGCCCGTCTCTACAATTAAT
TGGTAGTATCAATTGTGGCAATTTTTATGAAGGTTACTTTTCACAGGGAC
ATTAAAGGGGCTAATTTGCTTGTTGATGCATATGGGGTTGTCAAGCTTGC
CGACTTCGGAATGGCTAAACATGTGAGTTTTATACAATCATTTCGTAAGT
ATAATAAAATCACTTGTGTGTCAGTTACAGATACTAAAGGATATGGGGAG
AAGAAAAATAAAATATATTGATTGTTTTGTACATTCAGAATGAGTCTTAT
TCCCCTAATTATATAGTCGTAGGAGCAGGCTAAAATAGGAAGAATATTAT
TGAAATAAATGAGCTAATAAAACCCTACAATAAAGATACCAAAAATAGAA
ATTAACACGTAAATATAATATTTCAACACTCCCCCTCAAGTTGGGGCAAA
AATGTTGATGAGCCCTAACTTGCTAATAGCTGACTGAAGAAAAGATGGTC
TTGTAAACTTGGTGAACATATCAGCAGCCTGTTCTGAACATGGGATGAAG
GCTCGTGTGATGAATCCCAATCGAACTTTCTCACGAATGAAGTGGATATC
TACTTCAACGTGTTTCATCCTTTCATTAAGGACTGAGTCTGAGGATAATA
TTATGGCAAATTTGTTTTCACAGAACAATTTAGATGAGTTTCTCACAGGA
AGACCCAACTCGGTCAAAAAAAGAACGATGCCAAAGGAGTTTACACATTT
CTTGAGCCATAGCACAATATTCAGTTTCAGCAGAAGATTGGGAGACGATA
GATTACTTCTTACTCTTCCACAAGATTAGGTGTTTTCCATTAAATGTACA
CAGGCCAGAGGTCGAATGTTTATCGTCCTTCGATCCTGCATAGTCAATAT
CTGTAAAGCAGAAGAGTCCCTCCTATGCCTTTGTCATATAAAAGAGCCCA
AGACCAACGCATGTTTTGAGGTACCGAAGGATATGGTAGACAACATCCAG
GTGTGATATACGTGGTGAGTGCATAAAGTAGCTCACAACATTAACAACAT
ACGTAAGATCTAGCATTGTATTAGTCAAATGGATAAGACGATCCACAAGT
CGCTGATATATTGACGGATCTGATAATAATTTTCCTAAGTCTGTTGTTAA
CTTAATATTTGGATCCATGGGAGTAGAGGCGGGTCTACATCTAAGCATCC
CAGTGTCCTGAAGAAAATAAAGGACATATTTTCGTTGGGATAGAGAGATA
CCTTTCTGAGACTGAGCAACCTCAATACTGAGAAAGTAGCTGAGAGAACC
CAAATCCTTGATGTAAAAAAACTGTCCGAGATCCTTGTTTACCTGCTGAA
TACCAGGTGTGTCATCTCCTGTGATAATAATGTCATTCACATAGACTAAG
ATAATGATGCACTGTTCATGATGTTGGCGACGGATGAAGCAAGTATGACT
TGAGTGATACTGGGTAAACCCCATAGTGAGGATGGTTTCCATGAATTGGT

TGAACCATGCACAAGGAGATTGTTTAAGTCCATAGAGGAATTTTCGGAGT
TGACAGACTTTGCCTGAATATTCCCCCTGAGTCCGAAATCCTGGTGGAGG
CTCCATATAGATAGTCTCAGTCAGGTCACCATTAAGGAAGGCATTCTTGA
CATCTATTGGTGCGGAGGCCAAGAATGGGATGCAGCTAACAAAACAAGGA
GCCAGACAGTAGTCAACTTAGCAACCGGGGCAAAGGTGGCACCGAAGTCT
TTGCCTGGGATTTGCGTGTGGCCCTTAGCAACAAGTCGTGCTTTGTACCG
ATCAATCGATCCATCAACCAGATATTTGACACTAAAGACCCACTTATAAC
CAATTGGTCGTTCACTAGTCAACAAGTCATCTAAGTCCTATGTCTGATTC
TGCTGTAGGGCATCAATCTCTGCTTACATAGCAGTAACCCACTTTGGATT
TTGGAGGGCCTCACGAACGAAGCGAGAAATGACAACCGAATCAAGTTGTC
TAAGAAAGGATTTATATTTAGCAGAGAGATCTTGAAAAGACAAATATTTG
GAAATCGGATGATTAGTAAAACATGAAAGAAGATAGCGGGATGGTGGACG
ACGTTCCCAAGTCGAGTAACGGCGGGGAACAGGGTCAGAGATAATGGGAG
ATGGAGATATGCCTGAGCCCGGAGAAGATATCGGCAGGGGATATGAGACA
CGTGGATGACGGGAGTAGACTCGTAAGGGGTCAGATGGTGGGTGTGATGT
GGGCGGGGAAAATATATCTAAGACAGGGATAGGACACAGAAGTAGGGTAT
GATCCTCGGTAGTGGCTGCCACAATAGGTGGAGAAGATACGGTACCGAAA
AAAGTAGGGTACAGTCTCGAGGAACATAACATCGAGATAGTGGTAAGTAT
GGTGGGTAATTAGATCGTAGCACTTATATCCTTTTGACATAGAAGAATAG
CCGAGGAAAACACACCGGATAGATTTATCATCTAATTGAGTGCGAGTAGG
GCTCCGAGATTCTGGACAAAACACGTACAACCAAAGACACGGGGTAAGAT
AGGGAAAAGGGTACTAGTGTGTTGCAATATATGAAGGGGAGTTTTGTCTG
TAAGGACATGGCTCAGTGTGAGGCTAATGAGATAGGCAGCAGTAAAAATT
GTTGTGCCAAAAAGATTTGGGAACATTCATTCCTTGCAAAAGACAACGGA
CTACAAACATGAGTTGACGATTCTTACGTTCCGCCACACCATTTTGTTCA
GGGGGTGTAGGGACAGGTAAGTTGCTGAATGATACCTTATTAAGTTCAGT
CCGAAAATCATCGGATAAGTATTTCCGGCCATTATCTGATCTGATATTGC
ACACTACGGTTTTATATTGGGTTTTTATCATTTGAAGGAAGAGAGTAAAG
TGAGTGTAAATCTCATCTTTGTGTTTTATCAAATAAACCCAAGTGCACCG
ACTATAATCATCTATGAAGGTGACATAGTAGCGGTGTTTGAAAATGGCTG
TAACAGGAGAGGGTCCCCAAATATCAGAATGGATAAGATCAAATGGTTGG
AAGGCACGGTGCATGTGAGGAATATAAGAGGTACGCGTGTGTTTAGACAA
TTCACAAACCACACATTGAAATTTAAAGTTCTTGCAAGCTTTATTAATAG
ACGAAAACAACCAAGACAAATACTGAAAATTTGTATGTCCCAGACAAGCA
TGCCACAAGTTTAAATTTTTGAAAGAACACATAGAAAAATAAAAAACAGA
TAATACAAAAGTTTGCATACTAAAAGATGGAACGGGTCGAGATGGAGGGC
TGTCGAAGTAGTAGAGGCCATCGTGTTCATATCCCCTACCAAAAATCTTC
CTCGTAGTCAGGTCCTGCAAGAAGCAACGATCTGGTAAGAAAACAACGAT
GCAGTTAAGATTTTTAGCAAGAGTTCTTTACATAATAAATATTTTTTTTA
AAAAAAATTCCTGACAACACGCGAGTTTGAACTTATTTACCAGGCTCGAA
TATCCTGGTGTTCGTAACATCTGGACCGTTTCAAATTTTTTATTTTTTTG
GTCCCGACGTTCTGAACGCCAGGAGGAAAAAAAAAAAAAAATCTTGGCTT
AGTCTCAGCGTTCACAACGCCGGGACTAAGTCCCTGTTTTTTTGTCTTTT
TTTTTNTTTTTTTTTTTTTTTTTTTTTTTTTTGTGCCTATAGACTACAGAG

AGAGCAAAGAGAGTCACAACAATGAAATGAAATGGGTTGAAAACCTAAGT
ATTTTTTTACATTTTATACCCCTAACGTTTAACTATTATTTTATTTTAAA
CAATTTTTTTTTTTGTTTTTTTTTACTTTTTACTCTTTTTTTTTTTTTT
TTAAANTTTTTCTCTTTTTTTTTTTTTTTTATAAAAAAAAACTTTTTTTATT
TGTTAATTTTAATTTCTTTGATTGTTGTAATTTAATTTTAAAAGTGTGGT
CAAACGGTCGATCAAATAAGTCAAACGTTTAACTGATTAAAACAATGAGG
TAAAAAGTTCTGAAAACTAAAAATAATTTTCATAAATATTATATTTTTTT
AAAAAAAAAATTACTGAGAAGATGTCATTTTTAACTTATTTACGAAACTA
GAGTTACCCGGGATTGTGATCCCCAGGTATTAAAACTCAGAGTTCACAAC
CCCGGGTACAAAAGATTTTTTATTTTTTTAAATTTTTTTTAAGGTCAGGG
ACAAACCTGGGGTACTGTACCTTGGGTCTACATAAAAAAACCTCAAAGACA
AGTGAATTAATTTTTATTTTCGGCAATTTTGAAATAATTATCGGTGGTCT
ATCGGTATTTTAAATAAATTCAATCTTTTTGTGCATAAATTACGTTGAAA
ATACTTCATAATGTTTTTTACAACACTAAAAACGGACAAGTGAATGATTT
AAAAAAAATATTTTTTGTAAAAATATTATTTTATTTTAAAGTAAAAATAT
TTTGGAATAGTAAAAATAGTTCTATTTTTTTTATTTACTTCCATTTCATTT
TCGTGACTCTCTCTCTCTCCTCTCTCTGTTTGGGAAAAAAAAAAATATTGG
AGCATGATTGTTGGGGCACAACACAGATCTAAAATATTTTTACTTTAAAA
TAAAATAATATTTTTATAAAAAAAATATTTTTGATGTAGAAATATTGTTGA
AGTCGATTTCCACTTAAAAAAATCTAGTATAATAAAAAATCATACTAGAT
TTTTTAAAGCAAAAAATATATTTATCTTTTTTCAATTGAAGTAAATTTTT
ATTTTTATTTTTTATTTTCAATTGAATTCAAGTAAAAATTCAATTTGGTC
GACAAGATGCCCAATTCGTTTGACAGGTTAAATCAAGATATGCAATTTAA
ACCCACTATTTTCAACAGTTGACTGATTTGTCAAGTCGGTCGACTAACAT
GATTAGTTTTCAGAACTTTTTACCTCACTATTTTAACCAGTTAAACGTTT
GACTTATTCGATCGATCTTTTGACCTCACTTTTAAAATTAAATTACAACA
ATCAAAGAAATTAAAATTAACAAACAAAAAAAGTTTTTTTTAATAAAAAA
ACGAAATTAAAAAAAAAAAAAAAAGAGTAAAAAGTAAAAAACAAAAAAAAA
TTGTTTAAAATAAAATAATGGTCAAACTTTAGGGGTATAAAATGTAAAAA
AATACTTGGGTTTTCAACTCATTTCATTTCATTGTCATGACTCTCTCTCT
CTCTACTCTCTTTCTCCTCTCTTTATAGTCTGTAGGCACAACAAAAAAAA
AAAAANNNNNNNNNNNNNNNNNNNNAAGCCAAGAATTTTTTTTTTTTTG
TCTCGGCGTTCAGAACGCCGGGACCAAAAAAATAAAAAATTTGAAGCGGT
CCCGGTGTTCGCAACACCGGGATACTCGAACTTGGTAAATAAGTTCAAAC
TCGTGTGTTGTCAGGAATTTTTTTTTAAAAAAATATTTATTATGTAAAGA
ACTCTTTTTAGCAAGACGACTAACCAATAGAAGATTATTGACAAATTTAG
GAATATAGTAAACAAAGTGAAGAGTGATATCAGGGGACAAGCAGACATCT
CCCTGACAACAAATAAGGATAGAGGTACCATTGGGAATGCGAAACAGGGG
AGGTGAAGGTAGAAAACTCACCAGTCATGTGATTGTTAGGCCCGGAATCC
AAAATCCAAGTAGGGTGACTAAACTTACCATGAAGCGCAGTAAGAGTACC
TGAGGCAGAAGCAGCCGTGGGGTCGGAAGACGATGCCAAAGTGGTTAACT
GAGCCTGAAGCTGGGCAAGTTGGGCCTGGACGTGGAAAATATCCAGGGGT
GCTAGTAGAGGATCTTCTAGACCAGGGACAATATCAGCAATAACACCAGT
ACGTGGTCCTCCTCTTCCTTTCCTCCTTCCTAGGTTTCGGGGTTTCAATT

CAGGATGAAGATTGTAATAGTGATCAATGTTGTGACCAGACTTTCGACAG
TGCTGACAGTATGGTCGCGTGCCTAATGGGAGAAGACCAGAGGGAGCCAG
AAAGGTCATCTGCGGACCAGAGAGAGCCGGAAAGGCTACCTGGTCAAGGA
CTGGAGGAGTGACTTCAAGTGGGAAAAGGAGCTGACGTTTCGAATTGTTG
TTAACAATAGAGAATGCATTGTATAGATCTGGGATCAGTGATGTGTTCAA
AATCTGGGTGCGAAGATTCTCGTACTCCAGAATTAAACCCCATCAAGAAA
TGGTATGTGTGTAGACAATCCTGATGCTTGGCTTCGATCTTAGCAGCATT
CTCAGGGAATTTACTTAGAGGTTCATACTGCGCAAGTTCCTCCCACCGAG
ATTGAAGGTAGCCAAATTAATCCGCAACTGAAAGTTCTAAGGATGCCTGG
GAGGCATGAGAAATCTCACGATAAAGCTCGAAAATCCGTGATTTGTTGCG
AGAATGGGTATATATCTTCGTAAGTGCAAACCATAATCCGCTGAGAGTTT
CGTAGAACATGAAGATATTATAAATCCATTCCTCCATAGAATTGAAGATC
CACCCGAGGATGGTACAATTATCAGAGTCCCACTGAGCAAACTTGGGATT
GTTGATATCTGACTTTGGTTCAGTGTCGTTGAGCCATCCAGTCTTACGTT
TTCCACCAAGGTAGAGATGGAGAGAGTGAGACTAGGCAAGAAAATTCGTT
CCGTTGAATAAGACGATGGTAACACGTTGGATCGTGGCCTTAGGGGCACT
AGTAGAGTCTGAACGGGCGGGTAGCTGTGGAGCACAAACTGTCTGAGATA
GGACGTCCCCTTTTTTTTCGGATGCTATTAATATAGACGAGATGAGATCG
TTGTAATGAAATATATGGACATATGCTGCGGAAGACTAACAAGGCTCCAA
AAAAAGAAGGTGCAATATGAGAAAGGTAACCTTAACATAGTTACCAGATC
ATTAAAGAGATGGTCATGTATTTAAGACATCTGGAACAACAACAGATGGA
GAACAACCGCGACGTGCAGTTGAGATTTACACGAAAAACAATCTGCAATG
TTGCAGCAGTAGATAGAAGCACATCAATGTGCGATGCGCGAGATGGAGCG
CGAAGGACTAGTTCACGATATCTTGGTTCGCGACTTTGTGGTTCGCAATG
TGACAACAGTGGCGGCTAGGGTTTGGGTTCACGGTTTGCGAATGGGCAGT
GGTTTGTAGATGGTTCGTGGCGGCTAGGGTTTCGCAGGGGTGGGTGTGGT
TCGCAATGGGCAGTGGTTTCGCAAAGGTGGTTGTGGTTCGCGAACAGGCA
ATGTTTCGCGATGGAAGGGGTGGGTGCAGCTAGGGTTTGCAGGGGTTGGA
TGCGATGACGGCAGCGGCATGGGGTTCACAATTGGGGGTTAGTGGCGGCT
AGGGTTTGCAGGGGTGGGATGCGATGGCGGCGACTAGGGTTTGAGGTGGC
GACGACGGCTGGGGGTTCACAAATGGGGGTCGATGGCGGCTAGGGTTTCA
CAAAGGGGGTCAGCGGCTAGGGTTTTGGGACTAGTGGCAGCAGTGGTGGC
GATTTGGTGGTGGTGGTTGATGGTTTCAACAGCTAGTGATGGCTGCCGAT
AGTGTGTGGGTGGGAAAAATTGGCTCTAATGCCATAAAGGATATAGAGAG
AAGAAAAAGAAAATATATTGATTGTTTTGTACATTCAGAATGAGTCTTAT
TCTCTTAATTATATAGCCGTATGAGCAAGCTAAATTAGGAAGAATACTAA
AGAAATAAATGGGCTAATAAAACCCTACAATAAAAATTCCAAAAAGAGAA
ATTAACGCGTAAATATAATATTTCAACATATACCGATCTGATTTCAATCT
GAATTGAACATGCCTCAAAACGTAATCTGATTTGTAATATCCAGTTGTAA
TTTTATATTAAATTAAGCAAGCCTATGTCTATCCTTTTTTCTCAATTAAA
TTAGTTCATGTGGTATAACTTTTTTGTAACGGACTGAGGTTATTTTCACC
AAATATTATATATTAGTATGAATTTATTTATGGTATTATGGTATATGTTA
GGAACCATAAAAAAAACTGGTGAATGCTCCGCAAAATTAAAATTCATGTCC
ATGTAGTCTACTCTATCTCATGGTAACTATGCGAGAAATCTAAAAAAATT

GACAGAAATCTAAAATCGCCGCATTATGATTACGTGAAAATCACTTTGAT
ATTTAAAATGATTTTAATTCAATGCAGTGATTTAGAATTTCGGACAAAGT
TATTAGATTTCTCATGAGGTTACTGTATTGCATGGATTGGATGAATAGTG
GGCCGCAAAAATTGGGGGCCATGGCAAAAAACATGATTTTTTTCTTAGAA
TTTCTATGAGATTTGTATTTTAATTGAATTTGAAAATCTCTTTCATTTTT
GGTTATTAAGAATTTGGTTCTTTTGCACAGCTGACTGGACAAGGAACTAA
TCTTTCTATGAAGGGAAGTCCTTACTGGATGGCGCCAGAGGTATTTCTGT
GATTCAATATTATTGAGATTTCTGAAAGCAGAGATCTGTCTATGCTTGAC
ACCATTGTCTTTCTCTTGCAGCTCTTGCAATCTGAGCTCCAGACAGATGG
TAGGGCTGATCATGCTTTAGCTGTTGATATTTGGAGCTTGGGTTGTACTA
TTATTGAAATGCTTAATGGAAAACCTCCTTGGAGCGAGTATGAAGGAGTA
AGTTCACTCTGCATTGTTCTTATCTATGCTCAGTTATTCACTATTCAATA
ATATATTATTATTATTATTTTGAGAGCGGGGAGGGGCGCTTTTGTCCTGT
TTGTTTTTATTATCTATTACCTAAGTAGCGTTTGGTGTCATGGACTTGGT
GCCTCGGAATTGGAAGTGGGAAAATGAACACATGAAGATTTTTACATGGG
ATTATTTGTAATTCCAAATCTGAGGCTCCAAATCCATGAAACCAAACTCT
ACCTAAGGAAGTTTAGAAAATCCAAGAAGATTACTCTGCCCTTTTTGAGT
TTAATTTTTTGTAATAATGCAGCCTGCAGCCATGTTCAAGGTCCTGAAAG
AGACACCGCCAATACCCGAATCATTGTCGCCAGAAGGCAAAGATTTCTTG
CTTTGCTGCTTTCGTAGAGACCCTGCAGCAAGGCCAACGGCTACCATGTT
GTTGGAACATCCATTTTTGAGAAACTCGCAGCTGGATGTCCGATTTTGTG
CTCAATTAGTTGACGACATGAAATTGACTGTAAATTTTGACGCCTCACTC
CATTTTTTTTAAATGAAATAAAATTCTTTCGATTTACGCATAAAAATTAT
CTTATTTTCAATAGGATAAAACGCAAACTCCAAGAGAATCTTCACCTTCT
GGTTTTAAATATGACTATCCCCAAATATGAATGGCACCAAAGGGAGATCA
CCGAACAAGTAAGAATTGTTTTCCTCATTTACCCTTCTCCCATTATACAT
ACATACATATATACACACACACATTACAAGTGTCTAAATTCTCAAATACT
GTTGTGATTTCTTTTGCATGACTGCTGCCTTTTAGCTTGACTGGTGTTTT
TTGTTCTCCGGTGAAGCTGGCCGACAACCTCCTCATGAGACTTCTGACTT
AATGGTGCGTTCTTTTGGATCTTATGCAAATGCATCCATTTAG
>EUC21330-RA [gene]
ATGGCAGTGGAATCGAGCTCTGCTTCAGCTGAACATGGACATATGAGAGG
CGTCCCGACTCACGGTGGCCGCTACATGCAGTACAATGTGTACGGCAACC
TCTTCGAACTTTCCAGAAAGTACATCCCTCCGATTCGGCCAATTGGTCGT
GGCGCGTATGGCATGGTTTGGTGAGAACTCTCTCTCTCTCTCTCTCCCCC
TCTGGTTCTTCAGTTCTTCTAAAAGCGATTCATCTTACGCTAGTTCAAGC
TTCAAATTTACATCGTGTATGCATCTATTTAACTATATCAGTAGAGATCA
ATTGGGATGTCCGTTGCAATAAGAGCTCAAGACTGTTGTTAATTGGACTT
GAATCCATTTGCGTTTCTGCGGAAATTTCAATTTCAGTTAGTGTGTGATA
TTTGTTTTACTACTTGTTAGGTGTGATTTCGGATAAGTGTTTTACAAGAA
AATGTGTAGTCTCTTTGAATTGGCTTTCAGTTTGTTCAATTAGATGGCCT
GCGTTTTAATTCAAAGCACTGTTTACAAAAAATATGCAAATAATTTACTA
TCATGGATTATGAAGCAATTGATTATTAATAACATAATTTATTGGGTAAA
AAATCTTGTGAGAGTGGTAAAGGTTTCTTGTGTAGGCAGTGAAAGCAACT

TAAATTGGCAGTGCATGGTAAAGAAGAGACTTATTCAATTGATCTTCCTA
TCAGACACAGACACACACACACACAGTCTATGCAAGTTTTTGTTTTTAT
TTTAATTCTAATATTTATTTCAATATCAAGAGGGTGAACCAAGTCTTTGT
TAAATACTAACTTCCCATTTTCTTGTATGCCTCATCATAATGTCCATGTA
TATTACGCCATTGATTATGGGACCTTCATCTTTACTTCCAGTATCATCAT
GCTACAAAACCCTGGTTTTCTAAAGTAGAAACGGATATATGGGTTGGGTG
ATACGTGGACTAAATATTTTGACTTATGGTGGGTTTGATTGAGGTATTTG
AAGAAAGAATTTGATTTGGAGGGATTTATAAATTTTGTTATGGTCTGTTT
TGATTTGAAAAAGGCACTTGATTCAAAAAGAGATTTATAAAAATTTTGTT
AAGAAAAATGTACAAATTCCTTTCCTAATCAAAAGCTTTTCTTCGAATAC
CGTATCCAAAAGTACCCTTAAGGAATTTCATTTAAAATTTTTTATAAATC
CCTTTGTCAAATCAATTTTTTTTTACCAAATACCTCACCCAAACGTGCTC
TTATTTCGTTGAACACGTGATTCATTTTAAACAATATCCATGAAGTGTCA
ATCATGTCTAGCAATAAACCTGTTTGCTATGGTCAACCATTTAACCGTCA
AGAATTGAGAATCAGGGGTTTCATTGTTTTTGATTACTCTTGAACGACTG
ACATTCGGTTTGATATTTAAAGCAGTCTTAATTTCGGACTGTTTTCTCTT
TGAATGATGTGCTTTTTGAAGGGATTCGGTGAAGGTTATTCATGTTTATT
TAGTTTGCTTAATTCCTTTGTTGTATGCATTATATAAGGAAATATGTGTT
TCTTGTGTTTGATTCAGTACCGCTGTCAACTCAGAGACACGAGAAGAAGT
TGCTATCAAGAAGATTGGCAATGCATTTGACAATATAATTGATGCCAAAA
GGACACTACGGGAAATCAAGATTCTTTGCCACATGGATCACGAAAATGTA
AATTGCATCATTTTCAGCTCTCAAGATGTTTAAAATCTACTCAACTGTTG
TATCTTCCTGGGTTTTGCATTACTATTTCTGTTCTCCATTTTCAGGTCAC
TACTCATGTAAGATTAATTTATGCGTGCCAGTGCGAGAGTACTCAGCCAC
ATTAGAACGAAAGGAAAACAGCTAATGTGGCTGAAAGCACAGTTCATGTA
TTTACCTTCAGAAAGAAATGACTGTCGCTCTTGCTTTCATGTCCGCATAC
ATGAACTGTGGACATGAAAGCACCTTAATTTGCTTTCATGTCCACATACA
TGAACTGTGGACATGAACTTATGCAGAGAATGAGCTCTTGGTGGTTCTTT
ATTTCGACAGCAAGCACCTTAATTTGTTGTAATTTTCCATTCTTTGGTGC
AATTGTAAACTTACTGCTCATTTTTTTTTTCTTCTCGTGCGATAGATTGT
TGCGATCAAAGACATCATACGGCCTCCACAAAAGGAAAACTTTAATGATG
TATACATCATTTCTGAGCTAATGGACACTGATCTTCATCAGATAATTCGA
TCCAACCAACCATTGACTGATGATCACTGTCGGGTAAGTTTCTACTTTCT
TGTAGCATAGATCAGACATTTGATACAGTTTTAATCTTCACATGTTTATG
TTTAAACTGCTATTCCCAGGTGATACAAGGATATATATATATATTCACTT
GTAAAGGCTCTGTGCTTTTCAATTAATTAGTTTTAATCGTTAAACATATT
AATTAATTGTAATTGCAAAATAGCGATTAAAGGCTCTGTGCTTTTCAATT
AATTAGTTTTAATCATTAAAAATATTAATTAATTGTAATTGCACAATAGC
GATTAAATTAATTAAATTTTGTTAGTTATGGTCGGAAGATTTAACTATGC
AAATTAAAGTGAAATTTCAGGGAGCAATATGAGAAACTTGAAATACTAAG
GTTCAAAGTAAAAATTGTTCTACACACAAAAAATTAACTCAAATCATGTT
CAAGGTCCGGAGAAATAGGGGTAGAAAGTGTTTGGGCGGGACTTTTTCCC
CAAAAAATTCGTTAAACAATTTCATTGGCATGGTGTGCACTAATTCATAT
AACCAACCTTACTTAGGTGGGAAAAGGCTTAGTTGTTGTGGTGTATATAG

ATACACACAACACACAGATTAGCATAACAATTCCATTTATAAGGCTTGCA
ACTAAAACTATAAAAAAAAATTGTATTGCACTGCTCTCTATTCATATAAT
TATGCTACATATTTGATTTCTTGAATTTACTTTGAGGATTTTTTTATGA
GCATCTCCAACAATATTATTATATTTTTGTTATAATAGGGAATGAAACAG
AAAAAATAGGGAATGATAAAGTAGTTTACTCCAACAATGTTGAGTTTATC
CCATATCGGATGTGGAAGAGGCCAATGGTCAGATTACAAGTGTGAAGAAA
AGTCTCACTTCATGAGTTAGTTTTTGAGGTTGAGAGAGGTCCAACACCAG
TCCACTCTTCACTGGTATCAGAGCTCAGGTTACAACAACTTTGGCCTAAT
AGCCATGAAGGGAGAGGTTGGTGGAAGATTAGCCAACCAAAGGCCCAATG
TGTGAAAGAGAGATTGTTGGGTTTATCCCTCATCGAATGTGAAAAGGACT
AGTGGTCAGATTACAAGTGTGAGGGAAAATCTTACCTCATGAGCTACTTT
TTGGGGTGAGAGAGGCCTAAGACCACCCTTCACAATACTCCCCTATATTA
ACTACAATTATTATTTTTAATTAGCGGTTACTTTTAACATATTAATTTTA
AATAATATGAAAGAAGTGAAAGGTTGACATAAGAGGAGAAAGAGTTTATG
TAACCAAAATGTGTCTCTACGTGAGAACCAAAGGTAATCTCCTTAAATTA
GGCATGTACATATAGAATCTATTGGAGCTTAATTATTTGCTTAAAACCTC
CCCATTTTAATTTAAAAAACAAAAATTGGTGCTAACTTGTAAACGGAATT
AAATGGTCATTTCTGCAGTATTTCCTCTACCAACTACTTCGAGGACTCAA
ATATGTTCATTCAGCAAAAGTGTTGCACCGTGATTTAAAACCCAGCAATC
TCTTCCTCAATGCCAATTGTGACCTAAAGATCGGAGACTTTGGGCTGGCA
AGAACAACCTCTGAAACAGATTTCATGACTGAATACGTTGTTACTCGCTG
GTATAGGGCTCCGGAATTACTCCTTAATTGTTCTGAGTATACTGCTGCCA
TTGATATTTGGTCAGTTGGTTGCATACTTGGTGAACTCTTCACCAGACGA
ACCCTTTTCCCTGGCAAAGATTATGTTCAACAGCTGACGCTTATCACTGA
GGTATGCATGTCTTTTCTCAGCTTTGATGATGAGATTTTCTTTATCAATT
TATTAATTTTGAAAGTTCAATTTGCTCTTACATTATCATGTTTGCTAGTT
CAAATGAGTACTCCTTCGATGTTTATTTACTTGCACATCGTTATGTGAGC
AGAACAATAAAAGATTTTAAAACTAGTGTTTGTTTTACACTATAACAAAC
ACTTTTAAAAGTAAAAACATAATTTTAAATCATTTACATCCAATAAAAAG
TGAACCAAAGCTTCTATGACACCAGTTCCGAATATATCTGGCTAGATCCT
CGTAATTAGGCAAAAAACCAGTGTTTCGTAGGTCTTTTTACGCTGTTTTG
TCACTTTTTTATTGGGATTTGATGGAGTTGAGAGTCTGAATAATGAGAT
TCTTCATACAAAAGAAAGAAAGAAGGAATGCACGTCCTTCCTCGTCCACT
TGGTAGGAAGAGTAATCTGAGTCTACTTCTTATATCAGTAGATTTCGTTC
CACCGATTCTTTAGTCTTGTTACGATTCACCGAAGAAGTCTAGGCTTTAG
TGCCTCTTCTCCGGTCTCACTCGCGTCACACTTAGATTCTTCTTCTTTAT
CAGAGGAGGTAAGATCACTTTGTTGGTTCTTCCTCTTCCACGGATTTAAT
TACAACAATTGTGAATCGTATGGCGGTTAGACCAATGAAAAACACTCTCA
GGTTTTTTTAATAAAAAAATAATTGTTATTTGTTCCTTCGGTTTTCATAT
TTTGAGAGACCAGTAATTCTCTATACCGTAAATAAAACTCCTTTGAATGA
AGACATATAATATGGGTATAACTACAAAGACATTATCTTTGCACGATTAA
ATATATTTTTGATTTTTTTAAAAGTATTTTTTTTGATAGATTTATTTATT
TTTTATTATAAAAAGTAATTTAAAAACACTATGAAACGATGGCTTAATTT
TTCAATTTTGCGTCGCTATAACTTCTAAAGCACACTCACTCCTCTTCTAT

GAGGGTTGGGTTTTGTTTTCTTTAACCTTTTCTAATCCCGACACGTGTTA
CACACTTTCATTTTTGCAATTTTATTTCTTTTGCGTATTTAAAATTTAAC
TTGATGCCTAAAAGTCTAAAAATGTTGATTTTTGTCGCATATAATAAGAG
TTTAAATTCGAGACACCCCTTATCATTTTGACCAATACTAAAGACACTCT
ATATAATTTTGATAATACTTGAGCCACTTCTTTATCTTTTTTATTTCTAC
TCTCTTTCTTGTTTTCTATTTTTTATTTCATTTGTTTTATTTTTCTTTCA
TTTTTGTTTTTAAACATATTTCTTTATTTTTTTTATATGGTATCTATAGA
CCAAAAAAAAGGAATTCAAAAACAAAAAATTTAAATTAAAAATAAAAAAA
TTAAAAAAAAAAAAGAAAAAAAATACCAAAACATGAAAATGTATCTTTTA
TGTTTTAAATTTAAAGGATAAAACTGTGTACATCTTACCCTCTTCAAGAT
CCCACAACGGCGGTAGTTTGGGTTGCTATTAATCTTGTTAGTATTACTAT
TTTACACTGAGCATGACAATATTTATTGTTTTTTGTCAACAAAAAGCTAA
TAGGTTCGCCGGACGATGCTAGTCTTGGCTTTCTTCGAAGTGAAAATGCC
CGGAAATATGTCCGACTGCTTCCCCAGTACCCACGGCAACAATTTTCTTC
TAGATTTCCCAATGCCTCTCCTGGAGCTGTTGACTTGCTAGAAAAAATGT
TGCTCTTTGATCCCACCCAGCGAATCACAGGTAGACAAACACACTTACTT
CAAATCTTTTATGCGGTGTTTCTTTTAGTGTTTCTGTTCTCAGATTTTTG
TTTCTAA
>EUC23670-RA [gene]
ATGGCAACTCCAGTTGAGCCTCCGAATGGGGTCAAATCACCGGGAAAGCA
TTACTTCTCCATGTGGCAAACCTTGTTCGAAATTGATACCAAATATGTAC
CCATTAAACCAATTGGGCGGGGGGCCTATGGTATTGTCTGTTCCTCTGTC
AACAGAGAAACGAACGAGAAGGCTGCAATCAAGAAGATAAATAATGCCTT
TGAAAATCGTATCGATGCTCTGAGGACGTTGCGTGAACTGAAGCTTCTGC
GCCATCTGAGGCATGAAAACGTGATTGCTCTAAAAGATGTGATGATGCCC
ATCCACAGAAGAAGCTTCAAGGATGTCTACTTGGTTTATGAACTTATGGA
TACGGATCTGCATCAGATTATCAAGTCGTCTCAAGCACTCACCAACGACC
ATTGCCAATATTTTCTCTTCCAGGTAGAGTTTTTGACTGGCTAAAAGGTT
GATTGTTTGAATGTAAAAGATTTATTGTTTGTGAAAGCGTTGATTATGCA
TATTGGAACCATACGGCTATGCATGTTTACGAACCGAATTTTAGATAGTT
TTGACTTTAGTGAGTGGCCAACTCTATCCTGGTAGCACTTGCATACAGTT
TTTAGACTTTCATGATTGCCGAGACTAGATTTACCTAAATGTTCTACCTA
TGATCAGAGCAGACATGAATTAGTGACATATTGCTTTGCAAATTGGAATC
TGTACGCTCATTCACAACATTTGATGTTGCTTATGTGAAACTACCATTTC
CTCCTTGGCTCAGAATTAAAATGCCAATGTCACTGTTTCATCTTGGAATT
ATTGTGTTACTGTGCGTATGTTGACATGTGGTGGTGCAAATTTTGATGGG
GATTTGTTTGTGTGCACTTCTACCTTCAATAATCTACGTTTGAGTGCCTT
CAGACAGAGTTATCATGTCCAGGACTCATGGCGTCTAGTAGTTGCTGCTC
TCTTCATCATAGTCCTTGCCATTCTTTCAAAATCTGAATTACCCAAGTTT
CGTTTGTTTCTATGCTGCTTGTGCTTGATTAGTTTTGTTATGAAATGTAA
ACTGTAAATTGAATCTCCATTTCTAGTTGCTTCGAGGCCTCAAGTATCTT
CACTCAGCAAACATCCTTCACCGTGACTTGAAGCCTGGGAACCTACTCAT
CAACGCAAACTGTGACCTAAAAATATGCGACTTTGGACTTGCACGGACAA
GCAACGGTAAGGGCCAGTTCATGACCGAGTACGTTGTCACCCGCTGGTAC

CGAGCCCCAGAGCTTCTCCTCTGTTGCGACAACTATGGCACCTCCATTGA
CGTATGGTCTGTTGGTTGCATCTTCGCCGAACTTCTCGGCCGAAAACCAA
TTTTCCCCGGAACGGAATGTCTCAACCAGCTTAAACTGATCATCAACATA
CTGGGCAGCCAGAGAGAAGAAGACCTTGAGTTCATTGACAACCCAAAGGC
CAGAAAATACATTAAATCACTCCCTTATTCCCTCGGAACTCCTTTTTCCC
GTCTCTACCCCCATGCGCATCCACAGGCAATTGATATTCTTCAGAAGATG
CTCGTCTTTGACCCTTCAAAGAGAATCAGCGTGACAGAAGCACTCCAACA
CCCTTACATGTCTCCGCTGTACGATCCGAGTTCCAATCCTCCGGCACAGG
TCCCGATCGACCTCGACATAGATGAGGATTTGAGTGAAGAGATGATAAGG
GAGATGATGTGGCAGGAAATACTTCACTACCATCCTGAAGTTGCTGCCAG
CCAATCCTGA
>EUC06660-RA [gene]
ATGGTGATGGAATATACTGAGAGTTGCAGTAGTAGAGCTTCGGATTCACT
GCCGAAGCAAAGTCGGTTGCGAATACAGAAGGTTGAGGTTTATCGCGAGG
TTCTTCGTAGACTCAAGGAGTTGGACATTGAAGAGGCGAGTCAGCCTGGT
TTTGACGATGAGCTTTGGGCTCATTTCGATCGACTTCCGATTAGGTACTC
TGTTCTCCACTCATGCCCTAGCTATAGTCTTTTGATTCCTCTGTTAGTTG
TCCACGAAAAGCGATCGAAGCAAGCGGAGGAAGGGGGAAATAAAAAGGAA
GGGAAATCCTCAAGTTATGTAATATTTATCATACTTTCTGTTTCTCTCAA
TTTGATTGATATATTTTTTTCTTTGTTTTGCATCTCAGCCGAACAATCT
AATTAGTATAACTATTAATGATCAGAATTGTCCAGTGAAAACTGTTAAGT
TGAATTTCTCCCAGGATCAGAATCATGACCAGATCACCAGTATGCTCGTT
GGGAGATGGGAATTGATGATCTAATGATGTGGCTGGCTCTGGGAGTGTCT
TTCTTTGTTAATTTTGTCCATGGTGATTCTTTTTGTTAGGAGCTTGGCTC
CCATGTATACTTCTTGTTCTTTTTCTTTGTTCTTATTGTTTTGCTATGAA
TATCACTTTTCAAAAATGTGTTGCTTATCTGCTTGATCAAAGTCCTCGAA
GATATCTATATAGGAAATCAGCCCCCAGTTGAACAAATAAGACATGCATG
CTCGTCTAGGTTAAAAATAGAGTACTATATATATATATGACTTTGAAGGA
TATCATAGTAGCTATTTAACCAAAGAAGTAAAATCTAATTTGTTGTTCTG
GTTGCCCACTGCTGGCTTTTGGATCCGTCATTCACACTATCTTTCTCATG
CGGGATTTTAATGGTTGATTTTGCTCATTGTATTCATTGTGCTCATTGTA
GGTATGCACTTGATGTGAATATTGAAAGGGCACAGGATGTTCTTATGCAC
AAGAGATTGTTGCATATGGCACATGATCCTACAATGAGGCCTGCATTTGA
AGTCCGATTAGTGCAGGTAGATCTCATAAGAGGTTGCATTGACTACATGA
AATTGTTTCCCAATGGAAAAAATTTGGGAGTTTCCATGTGGATCCTAAAA
CTTCTCATGAAGGCAAAGAGTGGATGTAATCTCATGAAAACAATGTATTC
TCAGCTCCCTAGGAATGGGGCAGCATTGTAGTGTTTTTCCTAAATTGAAG
GTTTTCCCGAAGGAAAAACAATAACAAGTACTCATCGTATTTCTATTCCT
AAAATTTTAAGGAGAAAATGAAGAGGACGAAGAATTGAAAATGGAAACAT
GGTCAAATATGCCCTTAATTTTACAGTGATTTGGCTTATAATTGTTGATT
TTCACTTAAAGCACTGCCAATGATATATATATTTTTTGTATCATTAATTTAA
TTCAATATGATATACTTTCTACAATTGGTATACTAGAGCCGAAGTTTTTT
CTCTCTCGCGACCTTTCTGCTCTCTAACCAGGCAGTAAAGGAACAATTTT
CCGGGTACACGTATTTATAGGTGTATGACATTGATGTCTTTGGGTCTATG

TTTCAAGTCTTTTGACTTTGCTGTTTCATGTTGTTCTTCTCTTATGCAGG
TTTATCCCCAAAGTGATCCGGATTGTGGCGAATCTGTTCATTCAAAATTT
ACTATAGAAAATGATGATCACTACATTGAGTATCCTGGCAGCCATAGGCA
TGACTCTCTCAACACTGAACTGCTTATCTCGTTTCCTGTTGTAAAAATCA
TCATGATGCTTTGTGAACTCTATTTACAGTATTTTCATGTTTCTCAATTG
TTCAGCAAACATCTGCCACCTGCATTTGGTCTGTCACCTGGTTTTGAGCT
TGTACTTGAAGCCAATACACGTCAGCAAAATTGCAGCAGTTCGGGGGGTG
GCAATCTGCAGTTATTGCGGTAATTAATTCTAAAAGGTTTGCGCCAATTT
GAGCTTATCTATTTCTCCACTGTGTTTTAGCCACTTGGTTTCCTGCTTAT
GCAATTCTTTAAGTACCATGATTTCACTCTTCAACATTGGAAATTGGAAG
GTAAGGTCGAATGGATGGATGGATGGATAGTTGAAGGACATAATTACTTG
AGCTGCTGTTATGAGTATCATGCCACCACCCCCTCCCCCCTTTTTCGATA
ATAAAGAAGTCAAATGATGCATGCTAAGTCTACAACTCTACAATAACAAA
TAAGAACCATACTCCTATTGTAGTCTCAGAATAATATTTTTCTTCACTTC
CCTTTTATTTGGTTACCTCTAACAGTGGAGAGTGATTGCCTTGACGTAGG
AAAGAAAAATTATAGTCAGCTTCACCAGAAGTGTGACTCAAGTTGGGTTG
TAGGGAGCCCCATCTTATGAAATTTCCAAATATAGGAGGGGAATCGGCTC
TATATCTTTGTTGGGGTAATAATAAGTTCAAAATAACACGTCTACTGTCC
AAACATAAAAGCCCATATGGGCGAGAATTTGTATAAACAAGTACTAGGGC
TGGAAGGAACATCTGACCATTGTGTAAACTATATGCACGAGACACATAAC
TTTTGAGCAGGGGAAGTTTTATTGCATATACCATTTTCATTGCTTAGGGA
TATCAAATACTGATACAATCAAAGTTATTATTCCAAAGCTTCATGTTGAT
GTTTGATTAAATCACATTTCTTCCATCCAACGGAAATCACCATTTTATTG
TTATCAATTAGGCTATTACATATCTCTTGAAATCTGAATATTTCTTCACA
ATTAAAAGACGAGTTCTATTACAATTTCTTACCAAAATGCGAAGAAATAA
AATATGTATGACTTTCATTTCTATGCTTAAACTTTTCGTCGAAACAGTCA
TTATCTGTTTTAAGAGTTTGGGAGTTCGATAAAACAAATTTCTGATGATT
GGTATTTAATTGTTTGTTGGAAGATTCTTTCAAATCTTCATGAGATGACA
GAGTAGTTCTTTCACAATTTTTGTAGGCCTTTGCATGAGATTACAATTTC
AGCAAATGACAAGCCAAAGCTTCTCAGTCAGGTGGCTATATGTTACTGTT
TTTTCTTTCATTGCATTTGATATACTTCTGCAAGTCTTTAGATTATAGTT
GATTTCTTGCTGATAGCTTCATGCCAAATCACAAATATTTTGCAAGATTT
ATTTGATCCTGTGTTCGATGGCAGTTGTTATTCCTTGCTCATTTCTACAT
GATGATATTTTTGAGACATTTAATCCCAAAAAATGATTCGTGGATTGAGA
AAAGAGGAAATAAACCCACTTAGTGATACAATATTATTGATTATCCGGAC
AAGCCGTGCCATAACTCACGACTCACGCAATGTTCTCTTTAAATCTGGTG
GGGGTATTTATCATAAGTGGCGCTTTTCGTGTAGTCTCCTGTTTAGCATA
TCCAGATGAAACTATAAGACTTAGATGTATATTAGAAAAACCTGGCACTA
CCTATGATTGTTCTCCTCTGCAAAAGTAAATGATAAATTTATTCTGCTTC
TGAACTTAATTGTTTATTACAGTTGACTTCCTTACTTTCTGAAATTGGGC
TGAACATTCAAGAAGCCCATGCCTTTTCCACCACAGATGGTTACTCCTTG
GATGTGTTCCTTGTGGATGGCAGAGCATTCGAGGTTCTCTCTTCTTCCTC
AAATGTTTCTAATACAATATGAATTATTTCTTTGTCAGTGTGATAAGTCT
ATATTTTTTGATGATTTAAAGATGTTTATGAATGGTATATCCTGCATTAC

CTTTTTCTCTCTCTTATGGAGATCTGAAGTTTAATTTAGTTTTGTTGGAG
TCCACTATCAATTCTGAAGAAAGACATTAAAGATTAAAAACCACGAGTTG
GATTTAATTCATTGCAAAATAGTTGACCTTTCAATCACTGGATTTTTTTA
GGAAACCGAGCAGCTTAGGAATGTACTGGTAAAGGAAATTAAAAAAATTG
AGGTAACCAGTCAACACGTCACTTGTTGGTTTTAATGAATAGGGTACTAT
GCATCTATTATATCTTATATTGGATGAATTGCAGAAGCAATCTTGGTTGA
ATCCTTACGTCATGGTTTCTGCTGCGGATTTAAGGAAAACTGGGGTCAAT
CTTTTCCAGAGCCATGTAAACATACCAACTGATGGGACTGATGTGTGGGA
AATAGATGTCAAGCTTTTGAAAACTGAGAGAAAAATTGCCGTTACATCAT
ATGGGGATTTGTGAGTTCTACCATATTTAGAAACAGCTGCTTGATTCTCA
TCGCTGTCTTATTTATCTGTCTCTGACATCAGGTATAAAGGTACTTATTG
TAGTCAGGATGTGGCTATCAAAGTTCTCAAACCTGAATATCTAAATGAAG
ATGTGCAGAGGGATTTTGCCCAAGAAGTTTATATATTGAGGTTCGTCACA
TAATTTGCATAATGCTCTCTCAAGTTGTAGTTTGTCTTTGGGGTCTCTCC
CAACTTCAAAAGTTAGCTCATGAAGCGAGACTTTCTCTCCACACTTATAA
CCTGACCACTAGCCCATTTCGTCTATGATGTGGGATAAACCCAACAATTT
TTCTTTCACACATCGAGCCTTTGGTTGGCTAACTCTCGACCAATCTCTCC
CTTTGTGGCTATTGGGCCAAAGTTATTGTAACCTGGACTATGGTATCAGT
GTAGGGTGGTCTTGGACCTCTCCCAACGCTAAAAGTGAACTCATGTGGTG
AGGCTTTCCCTCACACTTATAACTTGACCACTTATCCCTTCCACATCCGA
TGTGGGATAAACCCAACAGATTTTACGTTCATGGCTAAGTTCTTAAAGAA
GCGTTCCATGAATTTCTGTCTGCAAATGCCTTTCTTTTCGTGCATATGTA
GATGAATTGTACTCAGTGTGGTCAATCACAGCTAGAGCCTTTATATGAAA
ATTAACTTTTGTCTATTTTTCTAATGACCCTTAGAACCTTTATAATAAAT
GAGAAAGTAAAGCTGGTTGATTATACAGAAGTTCATACTTCGTAGGGGTC
CGCTATTGGTTTACATGTCTCTGTGCTTTCAAGATCTCTTTTCTGATACT
TGCAGGAAAGTACGTCACAAGAATGTTGTGCAATTTATCGGAGCCTGTAC
ACATCCTCCAAGCCTATGTATTGTGACAGGTAAATTCAGAAATATTACTT
GGATGCAAACGATTGCAACTTTCCTCTTTACCTTGTTTTTTATTTCACGT
GACTTCTAATTTTGAAATATCATTTTGTCTACCCTTTAGAATACATGTGT
GGCGGAAGTGTCTACGATTTGTTGCATAAACAAAAGGGCATACTCAAGCT
CCCAGCTACACTCAAAGTAGCAATTGATGTTTCCAAGGGAATGAACTACT
TGCATCAAAATAATATAATTCACAGAGACCTTAAGGCTGCCAACCTTTTG
ATGAATGAAAATGAAGTAAGCAATTTGTCTGCATGCTTTACTTGTTGGTT
CTTGGCTTTTTGATGTTCTTGAATTGTAGTTTTATTGCATTGTTTGTCAT
CGACACTTCTTACTGTTAGATTCACACATTGAATTTGTGATGCTTCCTAA
AGTAGTCTATATTTGAAAGGAGTTACTCCACTTAATGTCATATGGTTTTA
GGTTGGAACCCCCTAACATGGTATTAGAACTAGGTTTGCGAAATTTCGTT
TGTTTGTCTCTGATTGTTTAAATGTTTGTCTTCTCTCTTCTCTTTGTTTG
ATCGTATCACGTGCAAGGCGGGGTTGCACATGAGGGTGGGTGTTAGGATC
ACACATTGAATTTGTGATACTTCCTAAAACAATCTATATATAAAAGGAGT
TATTCCATTAATGCTATATGGTTTTAGGTTTGAACCTCCTAACACTACCC
TTACTGCACCATCTGAATACTCGTATGCACTTAATGCTTTTAAAATATCA
TTTGTTTCTTCATATGCATAAATTTTCTCTCCCTCTGTCTGTCCAAGTAT

ACACAAGTACATGCATATACCATGTTCATAAGTTCATATATATGGGCTGC
TTATGATATTTGACAAACATGACATTCGGCGGGCATTCTAAAATATCCAG
AGCTCCTCTGACAGGCTGTTAAGGTTGCGGACTTTGGCATCGCCAGAGTG
AAGCCTCAGACTGGCGTGATGACTGCAGAAACAGGCACATACCGTTGGAT
GGCTCCGGAGGTCGGTAATTTGTGGATGCACTATTGCATTTGGGATTTTC
TTTTATAACCCTTTGAGTTTCAATAAATTGTGAGACGGCCACCTTCCTTT
TTTTTTTTTTTTAATAGATTTATTGCAGGCTACCGCTTGATTCAAACAG
TCAAATAGACATCATGAACTACGCCTAGTGTTGCATTTGCGTAGTGAAAG
GCCACATATGGCATACATCATCGCTTATGCTTATTTTGTTTTTTCTATTG
TTGAAAGGTAATTGAACACAGGCCATATGATCACAAGGTTGATGTTTTCA
GCTTTGGAATCACTCTTTGGGAGCTTTTAACAGGGAAGGTACAGTCATTT
TGCAAGTTGCAATTTGCAACTTTTTTTTTGTCCTTTTCACTTTTAGTAGT
GAGGGCAGGTTTTCAAACAAAAATACTGACATTTTAGTTCATTGTTGACT
ATGTTAGGATATTCACTAGATTTTTTTCATCGACTGATGTTTCAGCTTCC
GTATGAGCACTTAACCCCATTACAGGCAGCACTTGGAGTGGTCCATAAGG
TAACTTCTCCATGTTAAATTTGACTATTTACATAGACCCCATTATGTGAT
CTTCTCCGTAAGATAACTCTTGTATAGACGTTAAAATTCACTATTTACGT
AGAATCCGTTATATGATCTTCTCCGCAAGGTAATTTTTGCTTAGACGTTA
CAATTCACTATTTACGTAGCCTCCGTTATAAGATCTTCTCCATAAGGTAA
CTTGAGCATAGATGTTAAAATTCAGTATTTACACAGACCCCGTTATATGA
TCTTCTCCATAAGGTAAATTTTGCATAGATGTTAAAATTCACTATTTACG
TAGACCTCTCTGTAAGATCTTCTCCGCAAGGTAACTTTTGCATAGACGTT
AAAATTCACTATTTACGTAGACCCCGTTATATGATCTTCTCCATAAAGTA
ATTAGACGTTAAAATTCACTATTAATGTAGACCACGCTATATGATCTTCT
CCGCAAGGTAACTTTTGCATAGACATTAAAATTCGCTATTTATAAGATCT
TCTCCATAAGATAACTTTTGCGTAGACGTTAAAATTCACTATTTACGTAC
AACCCGTTTTACGTCCCTCTTTTTTTAACCTTTTTTCTTTCCCCCTTGCT
AAGTTGAGGCAAAACCCTACTGATTTTCTTCACTTAAATTTTAGGGTCTA
AGGCCTACAATACCAAGGCAAACTCATCCCAAGCTTGTGGAATTGCTGGA
GAGATGTTGGCAGAGAGACCCAACATTGAGACCGGAGTTTTCTGAAATTA
TTGAAATTTTGGAAAACATAGCTAAGGCCATGAAGGTATGAACATGTATG
TGTAATTGTGTGTGGCATTTCGAGAAGAAACTTGAAATGAGCAGGATATT
TCATATACAATAAACAGGTTGTTGAAGGTGAGAGCAATAGTAAAAAGAGA
AGGAATTACCTAGAATTTTGTTGA
